# Amino acid influx via LAT1 regulates iron demand and sensitivity to PPMX-T003 of aggressive natural killer cell leukemia

**DOI:** 10.1038/s41375-024-02296-6

**Published:** 2024-06-24

**Authors:** Ryo Yanagiya, Yuji Miyatake, Natsumi Watanabe, Takanobu Shimizu, Akane Kanamori, Masaya Ueno, Sachiko Okabe, Joaquim Carreras, Shunya Nakayama, Ami Hasegawa, Kazuaki Kameda, Takeshi Kamakura, So Nakagawa, Takuji Yamauchi, Takahiro Maeda, Keisuke Ishii, Tadashi Matsuura, Hiroshi Handa, Atsushi Hirao, Kenichi Ishizawa, Makoto Onizuka, Tetsuo Mashima, Naoya Nakamura, Kiyoshi Ando, Ai Kotani

**Affiliations:** 1 Department of Innovative Medical Science, Tokai University School of Medicine, Isehara, Japan; 2 Department of Hematology and Oncology, Tokai University School of Medicine, Isehara, Japan; 3 Department of Neurology, Hematology, Diabetology, Endocrinology, and Metabolism (3rd Department of Internal Medicine), Faculty of Medicine, Yamagata University, Yamagata, Japan; 4 Department of Regulation of Infectious Cancers, Division of Cellular and Molecular Biology, Research Institute for Microbial Diseases, Osaka University, Suita, Japan; 5 Division of Molecular Genetics, Cancer and Stem Cell Research Program, Cancer Research Institute, Kanazawa University, Kakuma-machi, Kanazawa, Japan; 6 WPI Nano Life Science Institute (WPI Nano LSI), Kanazawa University, Kakuma-machi, Kanazawa, Japan; 7 Division of Molecular Biotherapy, Cancer Chemotherapy Center, Japanese Foundation for Cancer Research, Tokyo, Japan; 8 Department of Pathology, Tokai University School of Medicine, Isehara, Japan; 9 Laboratory of Veterinary Physiology, College of Bioresource Science, Nihon University, Kanagawa, Japan; 10 Department of Molecular Life Science, Tokai University School of Medicine, Isehara, Japan; 11 Department of Medicine and Biosystemic Science, Kyushu University Graduate School of Medical Sciences, Fukuoka, Japan; 12 Division of Precision Medicine, Kyushu University Hospital, Fukuoka, Japan; 13 Perseus Proteomics, Inc, Tokyo, Japan; 14 Department of Hematology, Gunma University Graduate School of Medicine, Maebashi, Japan; 15 Faculty of Health Sciences, Tohoku Fukushi University, Sendai, Japan; 16 Department of Hematology, Hiroshima University, Hiroshima, Japan; 17 Department of Hematological Malignancy, Institute of Medical Science, Tokai University, Isehara, Japan

**Keywords:** Non-hodgkin lymphoma, Apoptosis

## Abstract

Aggressive natural killer cell leukemia (ANKL) is a rare hematological malignancy with a fulminant clinical course. Our previous study revealed that ANKL cells proliferate predominantly in the liver sinusoids and strongly depend on transferrin supplementation. In addition, we demonstrated that liver-resident ANKL cells are sensitive to PPMX-T003, an anti-human transferrin receptor 1 inhibitory antibody, whereas spleen-resident ANKL cells are resistant to transferrin receptor 1 inhibition. However, the microenvironmental factors that regulate the iron dependency of ANKL cells remain unclear. In this study, we first revealed that the anti-neoplastic effect of PPMX-T003 was characterized by DNA double-strand breaks in a DNA replication-dependent manner, similar to conventional cytotoxic agents. We also found that the influx of extracellular amino acids via LAT1 stimulated sensitivity to PPMX-T003. Taken together, we discovered that the amount of extracellular amino acid influx through LAT1 was the key environmental factor determining the iron dependency of ANKL cells via adjustment of their mTOR/Myc activity, which provides a good explanation for the different sensitivity to PPMX-T003 between liver- and spleen-resident ANKL cells, as the liver sinusoid contains abundant amino acids absorbed from the gut.

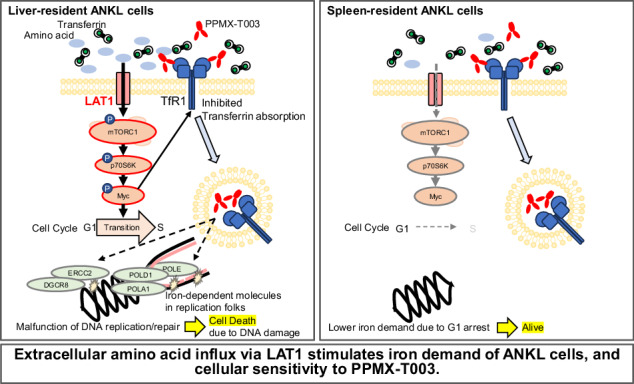

## Introduction

Aggressive natural killer cell leukemia (ANKL) is a rare hematological malignancy associated with Epstein–Barr virus reactivation in natural killer (NK) cells [1]. ANKL has a fulminant clinical course with a median overall survival of less than two months despite performing intensive chemotherapies [2–4]. As the standard of care for ANKL has not been established due to its rare incidence, the treatment of ANKL remains challenging. Although several genetic investigations have suggested that overactivation of Myc and JAK/STAT pathways accompanied by malfunction of *TP53* represent common genomic alterations of ANKL [2, 5, 6], the molecular pathogenesis contributing to the fulminant clinical appearance of ANKL remains poorly understood, as most of the reported gene abnormalities are common in extranodal NK/T cell lymphoma, another mature NK cell neoplasm presenting a relatively indolent clinical course [7]. To overcome this problem, we previously established ANKL-patient-derived xenograft mouse models (ANKL-PDXs) and discovered that ANKL cells predominantly proliferate in the liver sinusoid, depending on the transferrin-transferrin receptor 1 (TfR1) interaction [8]. Transferrin, the most common iron carrier protein, is specifically expressed by hepatocytes, and its receptor TfR1 is upregulated in ANKL cells under the regulation of Myc. ANKL cell proliferation requires massive supplementation of transferrin-binding iron from the liver microenvironment [8]. Although we further uncovered the anti-neoplastic efficacy of TfR1 blockade by a human anti-TfR1 inhibitory antibody, PPMX-T003 [9], in liver-resident ANKL, spleen- and bone marrow-resident ANKL cells with lower proliferative activity were resistant to PPMX-T003 [8]. This led us to hypothesize that the iron dependence of ANKL is determined by microenvironmental factors.

In this study, we revealed that PPMX-T003 causes DNA damage to liver-resident ANKL cells via malfunction of DNA replication and repair in the S-phase cell cycle, which is regulated by LAT1-mediated uptake of extracellular amino acids abundant in the liver sinusoid via activation of the mTOR/Myc axis.

## Materials/subjects and methods

### Study approval

The experiments using patient-derived materials were approved by the Institutional Review Board of Tokai University (H18-144). The experiments in this study were approved by the Animal Care and Use Committee (221046) and Genetically Modified Experiment Safety Committee of Tokai University (22-009-27R2). Written informed consent was obtained from all patients.

### In vivo CRISPR screening

A total of 482 genes encoding iron-dependent molecules were selected based on a previously published study [10], and the sgRNA library, including sgRNAs targeting those genes and 1004 control (non-target) sgRNAs, was constructed following a previously reported methodology [11], except for plasmid usage of Ubi-RFP-sgRNA. The Ubi-RFP-sgRNA plasmid and sgRNA library oligonucleotide pool were kindly provided by Prof. Hirao (Supplementary Table S1). Briefly, the Ubi-RFP-sgRNA plasmid was constructed from FG12 (Addgene, #14884) by cloning the RFP gene derived from pRSI12-U6-sh-HTS4-UbiC-TagRFP-2A-Puro (Addgene, #28289) into the GFP gene site and the sgRNA expression cassette used in a previous work [11] into the siRNA expression cassette. The RNA oligonucleotide pool was ligated into the Ubi-RFP-sgRNA plasmid digested with *BsmBI* using the Gibson Assembly Master Mix (New England BioLabs, #E2611S). Transformation of the 10-beta Electrocompetent Escherichia coli (New England BioLabs, #C3020) with sgRNA library-inserted plasmids was then performed using Gene Pulser II (Bio-Rad; 1000 V, 200 Ω, and 25 µF). More than 100 colonies per sgRNA were harvested, and sgRNA library-containing plasmids were purified using NucleoBond Xtra Midi (Macherey-Nagel, #740410). The constructed sgRNA library plasmids were then transduced with the lentiCas9-Venus plasmid (Addgene, #70267) into ANKL1 cells, and Venus^+^ and RFP^+^ cells were sorted 48 h after each transduction procedure using flow cytometry. Finally, 2 × 10^5^ (i.e., 40 cells per sgRNA) of sorted Venus^+^RFP^+^ cells were intravenously injected into a Nod/Shi-Scid, IL-2RγKO mouse (NOG mouse; In-Vivo Science) to establish PDX, and genomic DNA was extracted from the remaining sorted cells (as Input) using DNeasy Blood&Tissue Kit (Qiagen, #69504). Fourteen days after in vivo cultivation, ANKL1 cells were harvested from the liver, and genomic DNA was extracted (output). Integrated sgRNAs in genomic DNAs were initially amplified using Tks Gflex DNA polymerase (TaKaRa, #R060A) with a pair of primers, 5′-GTCTAGAGAGGGCCTATTTCCCATGATTCC-3′ and 5′-CACCGACTCGGTGCCACTTTT-3′, followed by additional amplification to add unique i7-index adaptor sequence per sample using a pair of primers, 5′-CAAGCAGAAGACGGCATACGAGATCXXXXXXTTTCTTGGGTAGTTTGCAGTTTT-3′ and 5′-AATGATACGGCGACCACCGAGATCTACACCACCGACTCGGTGCCACTTTT-3′ (“X” indicates i7-index adaptor sequences of Illumina TruSight Tumor 15, named R701 to R706). Amplified, i7-index-added sgRNAs were then mixed and sequenced by DNBSEQ-G400 (MGI Tech), using index primer of 5’′-TTTCAAGTTACGGTAAGCATATGATAGTCCATTTTAAAACATAATTTTAAAACTGCAAACTACCCAAGAAA-3′, and sequence primer of 5′-CGGTGCCACTTTTTCAAGTTGATAACGGACTAGCCTTATTTTAACTTGCTATTTCTAGCTCTAAAAC-3′. The reference FASTA-formatted file of the sgRNA library was created using Biostrings version 2.66.0 [12]. The obtained FASTQ-formatted files were then mapped, and sgRNA reads were counted using Rsubread version 2.12.3 [13] and GenomicAlignments version 1.34.1 [14]. In total, 479 of the 482 targeting sgRNAs were detected in the input samples. Positively- and negatively selected sgRNAs were statistically analyzed using the “test” command of MAGeCK version 0.5.9.5 [15], with default settings of alpha cut-off value. Gene Set Enrichment Analysis (GSEA) was performed using “pathway” command of MAGeCK with GMT-formatted file of HALLMARK of human Molecular Signatures Database (MSigDB) v2023.1 (https://www.gsea-msigdb.org/gsea/msigdb/index.jsp; data obtained on 7th Oct. 2023), and gene sets with false-discovery ratio lower than 0.25 were extracted as significant. This procedure was independently performed three times, and three NOG mice (Mouse #1, Mouse #2, and Mouse #3) were analyzed.

### Connectivity scoring analysis

NK92 cells were pre-incubated for 18 h and then equally separated into two dishes containing fresh medium. Then, 10 µg/mL of PPMX-T003 was added to one of them, and both samples were cultured for an additional 6 h. After harvesting the cultured cells, total RNA was extracted using Sepasol-RNA I Super G (Nacalai tesque, #09379-55) following the manufacturer’s protocol. A cDNA library was prepared using the TruSeq Stranded mRNA Sample Prep Kit (Illumina, #20020594). Sequencing was performed using a NovaSeq6000 (Illumina) with the NovaSeq6000 S4 Reagent Kit v1.5 (Illumina, #20028312). The FASTQ-formatted files obtained were trimmed using Trimmomatic version 0.38 [16], followed by mapping using HISAT2 version 2.2.0. Mapped reads were counted using StringTie version 2.1.3b [17] and EdgeR version 3.17 to obtain differentially expressed gene signatures between non-exposed and exposed NK92 cells to PPMX-T003. To identify agents that induce gene expression changes similar to PPMX-T003, connectivity scoring analysis [18] was performed using the Link database between Chemotherapeutic Agents and Gene Expression (JFCR_LinCAGE; available at http://molpro.jfcr.or.jp/db/cs/index.html; accessed on 23rd Oct. 2023) [19], following the procedure described in a previously published article [20].

### Single-cell RNA sequence

In the six ANKL1-PDXs, 10 mg/kg PPMX-T003 was intravenously injected into three of them twice a week for 2 weeks (a total of four times). After sacrificing the treated and non-treated ANKL1-PDX, human CD45-positive ANKL cells were harvested from the spleen using FACS Aria III. A single-cell cDNA library was constructed using a BD Rhapsody WTA Amplification Kit (Becton, Dickinson, #633801) and a BD Hu Single-Cell Sample Multiplexing Kit (Becton, Dickinson, #633781). Sequencing was performed using NextSeq550 (Illumina), and 247,753,594 aligned reads of 28,021 putative cells were detected by data processing using the BD Rhapsody Sequence Analysis Pipeline version 1.11 provided in SevenBridge (Becton, Dickinson, performed on 29th Oct. 2022). Clustering and detection of featured genes were performed using Seurat version 4.3.0.1 [21]. GSEA of feature genes was performed using clusterProfiler version 4.6.2 [22].

### Western blotting and quantitative PCR

Detailed materials and methods performed as the previous study [23] are described in supplemental materials.

### Statistical analyses

Statistical analyses were performed using GraphPad Prism 10.1.0 (GraphPad software). Two-sided Welch’s *t*-test was used to analyze the differences between two independent groups described by continuous variables. A two-sided paired *t*-test was used to analyze the differences between the two groups with some dependencies. In the analyses of differentially expressed genes or GSEA, the Benjamini-Hochberg method was used to adjust the *p*-value. All experiments were independently replicated more than two times. Sample sizes for all experiments were determined empirically from previous experimental experience with similar assays. Specific sample sizes of each study can be found in the figures, their accompanying legends, or within the methods section. All analyzed samples were included in the analyses. All results of statistical analyses were described in figures, as follows; “*” when *p* < 0.05, “**” when *p* < 0.01, and “***” when *p* < 0.001.

## Results

### ANKL cells in the spleen and bone marrow were resistant to treatment with an anti-TfR1 antibody, PPMX-T003

**Fig. 1:**
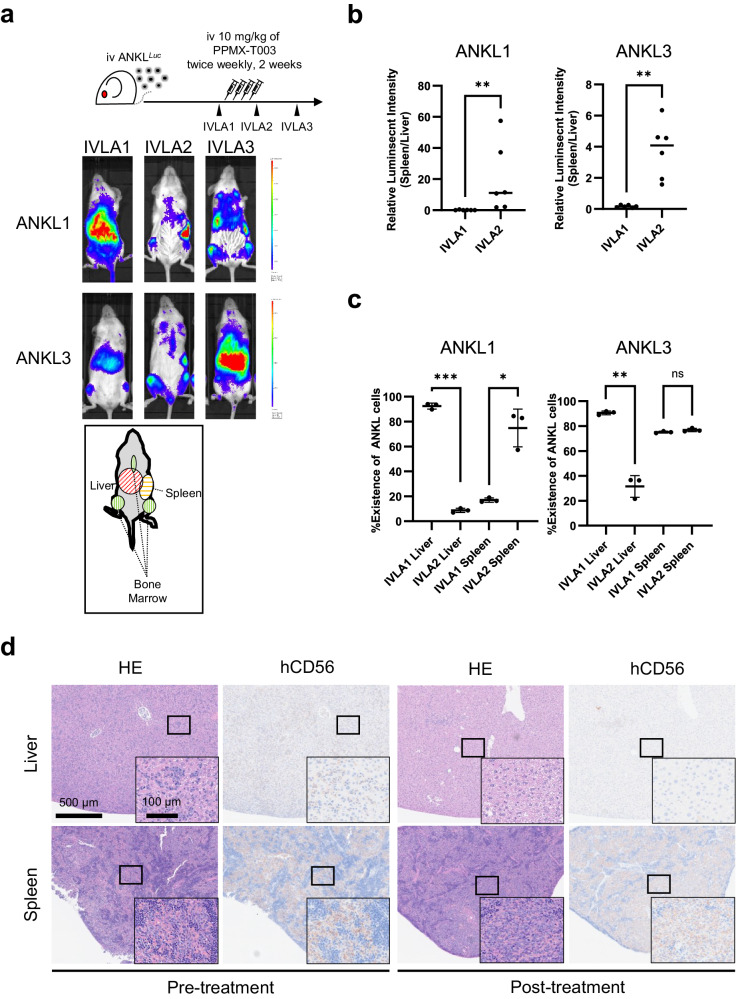
ANKL cells in the spleen and bone marrow were resistant to treatment with an anti-TfR1 antibody, PPMX-T003. **a** In vivo luciferase assay (IVLA) of pre- (IVLA1), early after- (IVLA2), and late after- (IVLA3) treatment of two ANKL-PDXs (ANKL1-PDX and ANKL3-PDX) with PPMX-T003 as previously reported [8]. **b** Relative luminescent intensities of the spleen compared with the liver of six ANKL1-PDXs and six ANKL3-PDXs at the time of IVLA1 and IVLA2 in (**a**). Setting of the regions of interest was shown in Supplementary Fig. S1. **c** Proportion of human CD45-positive cells (i.e., ANKL cells) of liver- and spleen-derived cell suspension of ANKL1- and ANKL3-PDXs at IVLA1 and IVLA2. Hepatocytes were excluded by density gradient centrifugation, and proportions were measured using flow cytometer (see Supplementary Fig. 1b). Three mice per stage per lineage were analyzed. **d** Hematoxylin and eosin (HE) staining and immunohistochemistry with an anti-human CD56 antibody of the liver and spleen derived from pre- and post-treated ANKL1-PDX. Squares in pictures of low-powered fields indicate the place of high-powered fields.

In vivo luciferase assay of two established ANKL-PDXs (ANKL1-PDX and ANKL3-PDX; Fig. 1a) demonstrated that luciferin luminescence derived from ANKL cells was predominantly detected in the spleen and bone marrow after the injection of PPMX-T003 (IVLA2) and eventually re-grew in the liver long time after treatment (IVLA3). Statistically, in both ANKL-PDXs, the relative luminescent intensities of the spleen versus the liver remarkably increased after the treatment of PPMX-T003 (Fig. 1b and Supplementary Fig. S1a). By the flow cytometric analysis, the liver-resident ANKL cells were decreased in both strains, whereas the spleen-resident ANKL cells were increased in the ANKL1- or unchanged in the ANKL3-PDX with the treatment of PPMX-T003 (Fig. 1c and Supplementary Fig. S1b). The immunohistochemistry (Fig. 1d) revealed that the spleen-resident ANKL cells were still largely detected after treatment, whereas liver-resident ANKL cells were remarkably decreased. Altogether, the sensitivity to PPMX-T003 is different between the liver- and the spleen-resident ANKL cells, raising the possibility that cellular iron requirement depends on the microenvironment in which they are localized.

### PPMX-T003 caused DNA double-strand breaks to S-phase ANKL cells, similar to conventional cytotoxic agents

**Fig. 2:**
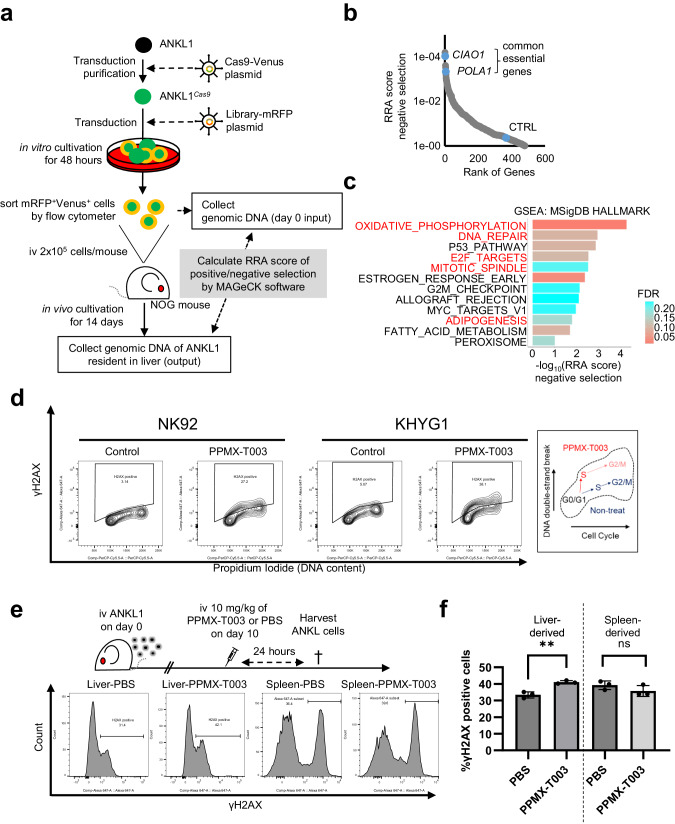
PPMX-T003 caused DNA double-strand breaks to S-phase ANKL cells, similar to conventional cytotoxic agents. **a** A schema of in vivo CRISPR screening targeting iron-require molecules. The procedure was independently performed three times, and three mice (Mouse #1, Mouse #2, and Mouse #3) were analyzed. **b** Negative selection-Robust Ranking Algorithm (RRA) scores of all genes of Mouse #1. The blue dots indicate the RRA value of sgRNAs targeting *CIAO1*and *POLA1*, and control (non-target) sgRNA (CTRL). **c** All annotated gene sets targeted by negatively selected sgRNAs in Mouse #1 using GSEA. Color value and red characters indicate the false-discovery ratio (FDR) and five commonly annotated gene sets in all the three mice (Mouse #1, #2, and #3; see Supplementary Fig. S2c, d). **d** Flow cytometric analyses of NK92 and KHYG1 exposed to PPMX-T003. Cells were cultured for 24 h with or without 10 µg/mL of PPMX-T003 before analyses. Relationships of cell cycle (DNA content) and DNA damage were plotted. **e**, **f** Flow cytometric analyses of liver- and spleen-derived ANKL1 cells of PBS- or PPMX-T003-treated ANKL1-PDXs. Total of three mice per treatment group were analyzed.

To investigate the molecular mechanisms of iron dependency in ANKL cells in the liver sinusoid, we performed in vivo CRISPR screening, targeting genes encoding molecules that require iron for their enzymatic activities. We designed sgRNA library targeting 482 genes of iron-dependent molecules (10 sgRNAs per gene) and 1004 non-target control sgRNAs to assess the contribution of these molecules to in vivo cellular proliferation or survival of ANKL cells in the liver (Supplementary Table S1). Cas9- and library sgRNA-transduced ANKL1 cells were purified, intravenously inoculated into NOG mice, and re-harvested from the liver 14 days after in vivo cultivation, followed by genomic DNA extraction and deep sequencing of the integrated sgRNAs to detect positively or negatively selected sgRNAs during cultivation (Fig. 2a). Deep-sequence analysis showed that sgRNAs targeting common essential genes such as *CIAO1* and *POLA1* (annotated by Depmap portal [https://depmap.org/portal/]; accessed on 7th Feb, 2024) were negatively selected (“dropped-out”), whereas control sgRNAs (CTRL) were not (Supplementary Tables S2–4; Fig. 2b; Supplementary Fig. S2a–b). Those results assured the reliability of the screening. A total of 2884, 2494, and 2861 sgRNAs were dropped-out in Mouse #1, Mouse #2, and Mouse #3, respectively (Supplementary Tables S2–4), which signified their contribution to cellular proliferation or survival of ANKL cells. GSEAs of the genes targeted by negatively selected sgRNAs using HALLMARK of MSigDB in the three mice indicated that five gene sets (oxidative phosphorylation, DNA repair, E2F-targets, mitotic spindle, and adipogenesis) were commonly dropped-out with false-discovery ratios lower than 0.25 (Fig. 2d; Supplementary Fig. S2c–e; Supplementary Tables S5–7). In particular, the gene sets of oxidative phosphorylation and DNA repair showed a lower false-discovery ratio in all three mice. Gene sets of DNA repair and E2F-targets included various genes crucial for DNA repair, such as *ERCC2* (contributing to nucleotide excision repair) and *DGCR8* (contributing to the repair of ultraviolet radiation-induced DNA damage; Supplementary Fig. S2f). Furthermore, sgRNAs targeting to several DNA polymerase- and ribonuclease-coding genes (*POLA1*, *POLE*, and *RRM2*), which are required for DNA replication, were also significantly dropped out in all three mice (Supplementary Fig. S2f). These results suggested that the survival of liver-resident ANKL cells depended on extracellular iron supplementation mainly through two cellular functions, oxidative phosphorylation and DNA replication/repair system.

We then compared the differentially expressed gene profiles of PPMX-T003-treated NK92 cells (an ANKL-derived cell line) with those of tumor cells treated with various functionally well-known anti-neoplastic agents deposited in JFCR LinCAGE to assess their similarities in predicting the molecular mechanism of the PPMX-T003-induced anti-neoplastic effect (connectivity scoring analysis; Supplementary Fig. S2g). Among the assessed agents, conventional cytotoxic agents that induce DNA damage, such as anthracyclines and DNA intercalators, had higher connectivity scores, indicating that the transcriptomic alterations induced by PPMX-T003 resembled those induced by these agents (Supplementary Fig. S2h; Supplementary Table S8). The γH2AX expression, a well-validated cytological marker of DNA double-strand breaks, as well as the total cellular DNA content of ANKL-derived cell lines (NK92 and KHYG1) exposed to PPMX-T003 in vitro, indicated that PPMX-T003 caused DNA damage throughout the cell cycle progression (Fig. 2d; Supplementary Fig. S2i). Furthermore, ANKL1 cells derived from liver of PPMX-T003-treated ANKL1-PDXs also increased γH2AX expression, whereas those derived from spleen did not (Fig. 2e, f). These findings suggest that replication-dependent DNA damage is responsible for the antineoplastic effect induced by PPMX-T003, and it depends on somewhat liver-specific microenvironmental factors. Previous GSEA also indicates that the liver-resident ANKL cells show more enrichment in gene expression related to DNA repair than the spleen-resident cells, which supports the higher sensitivity of liver-resident ANKL cells to PPMX-T003 [8].

### PPMX-T003-sensitive ANKL cells were characterized by higher activity of the mTORC1/Myc/TfR1 axis

**Fig. 3:**
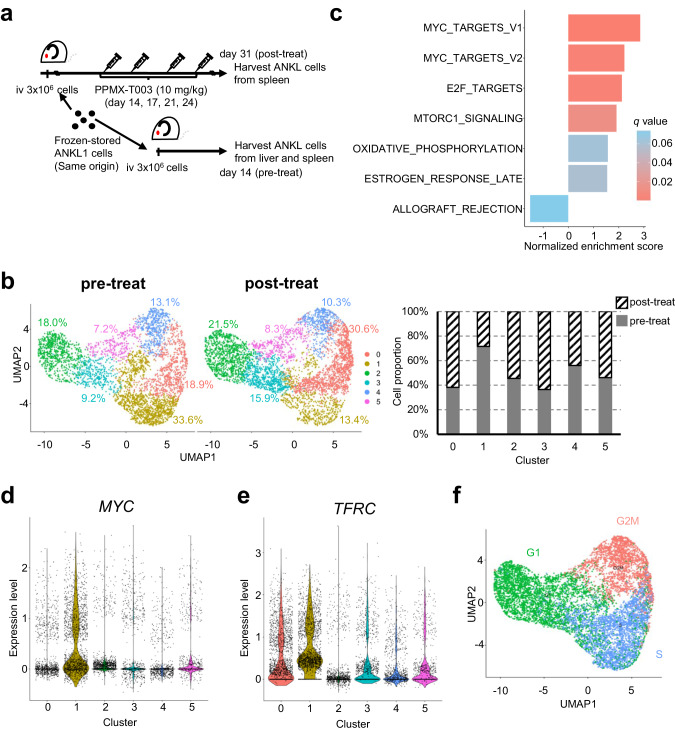
PPMX-T003-sensitive ANKL cells were characterized by higher activity of the mTORC1/Myc/TfR1 axis. **a** A schema of sample preparation for single-cell whole-transcriptome analysis. **b** Unsupervised clustering (UMAP) of analyzed ANKL1 cells. The bar graph indicates the cell populations of each cluster. **c** GSEA of feature genes in cluster 1. All annotated gene sets in HALLMARK of MSigDB with adjusted *p*-values lower than 0.25 were described. *MYC* (**d**) and *TFRC* (**e**) expression profiles of all the analyzed spleen-derived cells per cluster. Color consistency represents the amount of expression in each cell type. **f** Results of cell cycle scoring analysis of all the analyzed spleen-derived cells (left) and its schema describing cell cycle transition (right).

Next, we characterized the subpopulation of spleen-resident ANKL cells sensitive to PPMX-T003. Spleen-resident ANKL1 cells harvested from pre- and post-PPMX-T003-treated ANKL1-PDXs were subjected to single-cell whole-transcriptome analysis (Fig. 3a). Unsupervised clustering analysis separated the cells into six clusters, suggesting that the population in cluster 1 decreased after treatment (Fig. 3b, Supplementary Fig. S3a). GSEA with HALLMARK from MSigDB indicated that the activities of mTORC1 and Myc were significantly upregulated in cells belonging to cluster 1 (Fig. 3c, Supplementary Table S9). Furthermore, the expression levels of *MYC* and *TFRC*, which is the Myc target gene that encodes TfR1, were specifically upregulated in cluster 1 (Fig. 3d, e). Cell cycle scoring analysis annotated cluster 1 as a DNA replicative cell cluster, which was strongly related to the anti-neoplastic mechanism of PPMX-T003 as shown previously (Fig. 3f). These results suggest that the PPMX-T003-sensitive subpopulation of ANKL cells is characterized by a higher activity of the mTOR/Myc/TfR1 axis. The results agree with those that liver-resident ANKL cells (sensitive to PPMX-T003) show higher activity along the mTOR/Myc/TfR1 axis [8]. Furthermore, we investigated the antineoplastic effect of PPMX-T003 against mTORC1-suppressed ANKL-derived cell lines by sublethal dosages of rapamycin, and revealed that PPMX-T003-derived antineoplastic effect was decreased in mTORC1-suppressed cells via apoptosis and cell viability assays (Supplementary Fig. S3b–c). It also supports the results described above.

### LAT1-mediated amino acid influx positively regulates ANKL cell proliferation

**Fig. 4:**
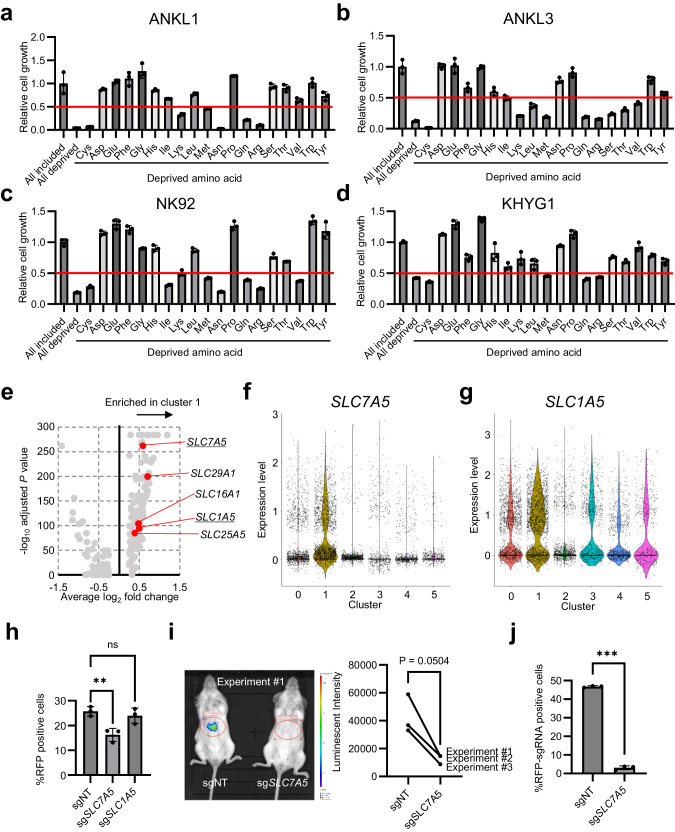
LAT1-mediated amino acid influx positively regulates ANKL cell proliferation. **a**–**d** In vitro cell proliferation assay of liver-derived ANKL cells and ANKL-derived cell lines cultured in single amino acid-deprived RPMI 1640. Each bar indicates the relative proliferation value to all amino acid-included conditions. The red line indicates a relative proliferation value of 0.5, a cut-off of these assays. Total of three samples per treatment group were analyzed. **e** Volcano plot of feature genes of cluster 1 of single-cell whole-transcriptome analysis described in Fig. 3, compared with other clusters. Red dots and underbars indicate SLC family genes and genes encoding amino acid transporter, respectively. **f**–**g** Violin plots of *SLC7A5* (**h**) and *SLC1A5* (**i**) of single-cell whole-transcriptome analysis described in Fig. 3. **h** In vitro competitive proliferation assay of transduced Cas9-overexpressed NK92 cells with sgRNA-RFP plasmids by lentiviral vector. Proportions of RFP-positive cells (i.e., target gene-knocked-out cells) were measured using flow cytometry two and eight days after lentiviral transduction. %RFP-positive cells is determined by dividing the number of RFP-positive cells of day 8 by those of day 2. Total of three samples per treatment group were analyzed. **i** In vivo luciferase assay of ANKL3-PDXs established with Cas9-overexpressed ANKL3 cells transduced with sgNT- or sg*SLC7A5*-RFP plasmids. Assays were performed seven days after ANKL3 cell inoculation. Luminescent intensities of liver regions were measured. Experiments were independently performed three times, and statistical value was calculated using paired *t*-test. **j** Proportions of RFP-positive cells (i.e., target gene-knocked out cells) of liver-derived ANKL3 cells at the timing of in vivo luciferase assay in (**h**), measured using flow cytometry.

To identify the extracellular factors regulating the mTORC1 activity of ANKL cells, a re-analysis of the bulk RNA-seq data of liver-derived ANKL1 and ANKL3 cells and peripheral NK cells of healthy volunteers (deposited in the Gene Expression Omnibus; GSE189722) was performed, focusing on the metabolic status. GSEA with the Kyoto Encyclopedia of Genes and Genomes and MSigDB-HALLMARK indicated that ANKL cells were characterized by higher activities of amino acid metabolism, but relatively lower glucose and fatty acid metabolism (Supplementary Fig. S4a–c). In particular, the ANKL cells metabolized sulfur-containing amino acids (Supplementary Fig. S4c). Pathview [24] of sulfur-containing amino acid metabolism showed that the transcription of genes associated with the methionine cycle and polyamine synthesis was specifically upregulated (Supplementary Fig. S4d), which is known as an mTOR stimulator [25]. Additionally, mRNA expression analyses of solute carrier (SLC) family molecules showed that various amino acid transporters were highly expressed in ANKL cells compared with primary NK cells (Supplementary Fig. S4e). The investigation of the dependency of ANKL cells on extracellular amino acids by in vitro culture of those cells in the single amino acid-deprived RPMI-1640 medium revealed that all the assessed ANKL cells (ANKL-PDX-derived cells and cell lines) showed impaired proliferation (<50% compared with all amino acid-included medium) in the single sulfur-amino acid depletion (cysteine and methionine; Fig. 4a–d). These results suggest that ANKL cell proliferation highly depends on the influx of sulfur-containing amino acids. Furthermore, single-cell whole-transcriptome analysis data explained in Fig. 3 indicated that *SLC7A5* and *SLC1A5*, which encode LAT1 and ASCT2, respectively, were upregulated in PPMX-T003 sensitive cluster (cluster 1; Fig. 4e). LAT1 is a cancer-specific large neutral amino acid transporter that can transport methionine [26, 27], whereas ASCT2 is annotated as a cysteine transporter [28]. Violin plots indicated that *SLC7A5* was more specifically expressed in cluster 1 than *SLC1A5* (Fig. 4f–g). *SLC7A5* was annotated as a mTORC1-related gene in HALLMARK of MSigDB (Supplementary Table S9). In vitro growth competition assay using the CRISPR-Cas9 system in NK92 cells indicated that the sg*SLC7A5*-RFP transduced cells significantly decreased the survival (RFP positive) ratio compared with non-target control sgRNA (sgNT)-RFP-transduced cells, whereas the sg*SLC1A5*-RFP transduced cells did not (Fig. 4h, Supplementary Fig. S5a). Furthermore, we established ANKL-PDXs with sg*SLC7A5*-RFP- or sgNT-RFP-transduced ANKL3 cells to assess the contribution of LAT1 to the proliferation of liver-resident ANKL cells (Supplementary Fig. S5b). In vivo luciferase assay and flow cytometric analysis seven days after inoculation indicated that LAT1-knocked-out cells decreased proliferative ability (Fig. 4i–j). These findings show that amino acid influx via LAT1 is crucial for regulating cell proliferation through the cell cycle in liver sinusoid ANKL cells.

### Amino acid influx via LAT1 is essential to the therapeutic efficacy of PPMX-T003 through positive regulation of mTOR/Myc activity

**Fig. 5:**
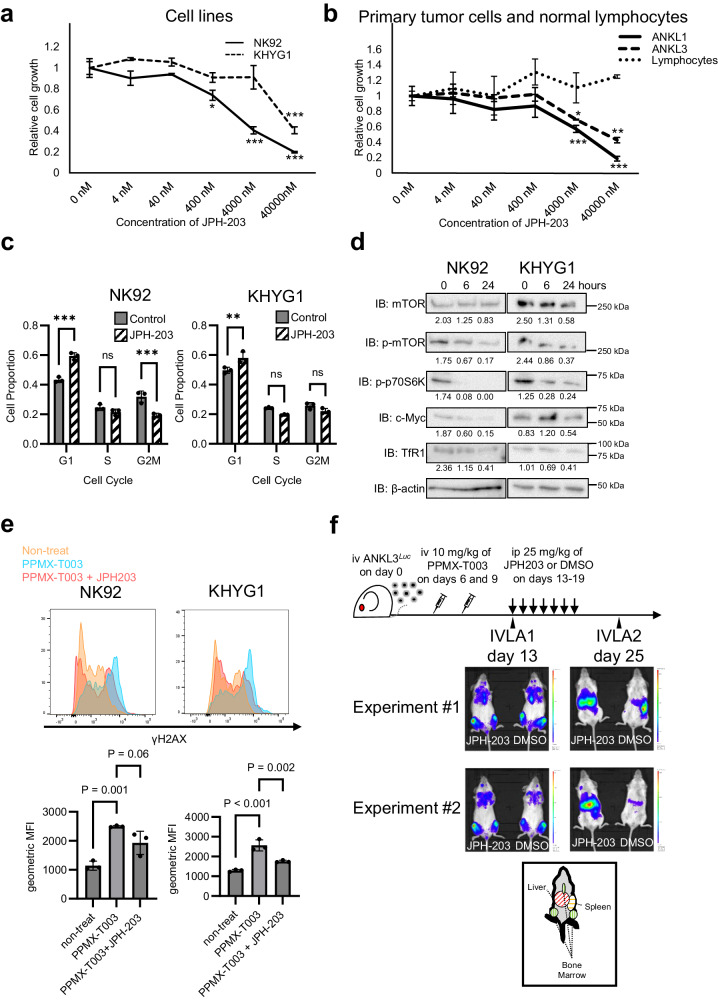
Amino acid influx via LAT1 is essential to the therapeutic efficacy of PPMX-T003 through positive regulation of mTOR/Myc activity. Proliferation assay of ANKL-derived cell lines (**a**), liver-derived ANKL-PDX cells (**b**), and normal lymphocytes derived from peripheral blood of healthy volunteer (**b**) treated with various concentrations of JPH-203 for 48 h (**a**) or 24 h (**b**). Total of three samples per treatment group were analyzed. **c** Cell cycle assays of JPH-203-treated NK92 and KHYG1 cells. After cultivation with 40 µM JPH-203 for 24 h, cell cycle proportions were measured using flow cytometry. Total of three samples per treatment group were analyzed. **d** Western blot analysis of JPH-203-treated NK92 and KHYG1 cells. After cultivation with 40 µM of JPH-203 for 0, 6, or 24 h, the relative luminescent intensities of each band compared with those of β-actin were calculated and described under the band. **e** Cellular γH2AX expression value of NK92 and KHYG1 cells cultured with or without 40 µM of JPH-203 and/or 10 µg/mL of PPMX-T003 for 24 h, measured using flow cytometry. Total of three samples per treatment group were analyzed. **f** In vivo luciferase assay for ANKL3-PDXs treated with PPMX-T003 and DMSO or JPH-203 sequentially.

Finally, we investigated the relationship between LAT1 and mTORC/Myc activity, and cellular sensitivity to PPMX-T003. The inhibition of LAT1 using JPH-203 [29] caused the dose-dependent inhibition of cell proliferation, accompanied by G1 arrest (Fig. 5a–c). This growth suppression effect was observed specifically in neoplastic cells (Fig. 5b). Western blotting analyses of NK92 and KHYG1 cells treated with JPH-203 revealed the downregulation of phosphorylated mTOR and p70S6K, one of the major targets of mTORC1 contributing to cell proliferation, as well as Myc and TfR1 in a single day (Fig. 5d, Supplementary Fig. S6), suggesting that LAT1 positively regulates mTOR/Myc activity and TfR1 expression. Quantitative PCR analyses of JPH-203-treated ANKL-derived cell lines also showed downregulation of *TFRC* expression (Supplementary Fig. S7a, b). The intracellular ferrous ion (Fe^2+^) concentration was significantly decreased by LAT1 inhibition without any alteration of iron supplementation from culture medium (Supplementary Fig. S7c), indicating that the cellular iron demand (usage) was decreased. In addition, LAT1-inhibition of ANKL-derived cell lines decreased γH2AX expression in response to PPMX-T003 exposure in vitro (Fig. 5f) and the anti-neoplastic effect of PPMX-T003 toward the liver-resident ANKL cells in vivo (Fig. 5g). These findings suggest that the regulation of mTOR/Myc activity via LAT1-mediated amino acid influx is a determinant of iron dependency for survival and the resultant sensitivity to PPMX-T003 in ANKL. The single-cell transcriptome of liver-resident ANKL, presenting a favorable response to PPMX-T003, showed a wider distribution of *SLC7A5*-expressing cells in active cell cycles than spleen-resident ANKL (Figs. 3a and 4f, Supplementary Fig. 7d–f), supporting those findings. We finally assessed the antineoplastic potential of JPH-203 in vivo. Although knockout of LAT1 in ANKL cells decrease their growth in the liver of ANKL-PDX (Fig. 4h–j), no obvious therapeutic effect of JPH-203 in ANKL-PDX was observed (Supplementary Fig. S7g).

In conclusion, the influx of amino acids, which is abundant in the liver sinusoid, through LAT1 promotes the cell cycle of ANKL cells via mTOR/Myc activation and stimulates replication-associated DNA damage caused by PPMX-T003.

## Discussion

We previously reported that PPMX-T003 is a promising therapeutic agent for ANKLs, especially in the liver [8]. Based on our findings, a phase Ib/II clinical trial of PPMX-T003 against ANKL was initiated in April 2023 (jRCT2061230008). To enhance the evidence for the high efficacy of PPMX-T003, a detailed mechanistic analysis of the anti-neoplastic efficacy of PPMX-T003 was investigated in this study. PPMX-T003 induced DNA double-strand breaks in ANKL cells, specifically in the S-phase cell cycle. Furthermore, LAT1-mediated amino acid influx is crucial for the G1-S cell cycle transition of ANKL cells via mTOR/Myc activation, suggesting that abundant extracellular amino acids are key determinants of sensitivity to PPMX-T003 of ANKL cells.

Since accumulated studies have reported that the anti-neoplastic efficacy of iron chelators, such as deferoxamine and deferasirox, is caused by G1 arrest followed by p21-triggered apoptosis [30–39], the cell death induced by PPMX-T003-mediated specific TfR1 blockage in ANKL is unique. Further investigation of the mechanisms, including iron sensing, iron metabolism, and iron cellular distribution, is needed to clarify the differences in cell cycle regulation between iron chelation and specific TfR1 inhibition.

Although the results obtained by in vitro experiments suggest that the survival of ANKL cells highly depended on extracellular cysteine rather than methionine, those by in vivo CRISPR/Cas9 gene knockout analyses suggest that a methionine transporter LAT1 was more crucial for their in vivo growth than a cysteine transporter ASCT2. These inconsist results might be caused by activity of other cysteine uptake pathways, as several molecules coded in SLC family genes (e.g., *SLC7A11*, *SLC7A9*, and *SLC3A1*; according to SLC TABLES; https://www.bioparadigms.org/slc/; accessed on 7th February). Further investigations were needed to evaluate cysteine dynamics in liver sinusoid of ANKL-PDXs. LAT1 is a cancer-specific neutral amino acid transporter widely investigated as a therapeutic target molecule [26, 29, 40]. In ANKL, we found that LAT1 plays a crucial role in excess cell cycle progression in the liver sinusoids via mTOR/Myc activation and increases cellular sensitivity to PPMX-T003 by enhancing cellular iron requirements. Accordingly, in the PPMX-T003 treatment of ANKL, LAT1 activity should be maintained, rather than inhibited, to obtain a favorable therapeutic effect. Single-cell transcriptome analysis suggested that liver-resident ANKL cells express *SLC7A5* (LAT1 coding gene) regardless of their cell cycle, whereas spleen-resident ANKL cells specifically express it only in their S-phase cell cycle, suggesting that LAT1 expression is regulated by some microenvironmental factors. To improve the therapeutic potential of PPMX-T003, the detailed molecular mechanism of the environmental regulation of LAT1 expression should be elucidated, followed by drug discovery to regulate tumor LAT1 expression. Clinically, LAT1 can potentially be a surrogate marker of the therapeutic response to PPMX-T003, which will be investigated in an ongoing clinical trial. JPH-203 monotherapy was ineffective to ANKL in ANKL-PDXs (Supplementary Fig. S5g), in spite of the significant growth incompetence of LAT1-knocked out ANKL cells in liver sinusoid. We speculated that the discrepancy between knockout of LAT1 and its pharmacological inhibition was caused by insufficiency of the systemic permissive dose of JPH203 to exert enough inhibition of LAT1 in the liver sinusoid, where LAT1 might be highly upregulated by environmental factors as discussed in the manuscript. The fact that inhibition of LAT1 is a therapeutic effect in ANKL as well as its expression confers the sensitivity of ANKL to PPMX-T003 suggest that TfR1 and LAT1 should not be co-targeted in ANKL. The potential of LAT1 as a novel therapeutic target of ANKL should further investigated focusing on these concerns.

In conclusion, PPMX-T003 induces DNA double-strand breaks in ANKL cells, depending on their DNA replication in the S-phase of the cell cycle, and its pharmacological efficacy is determined by LAT1-mediated amino acid influx via mTOR/Myc activation.

### Supplementary information

Supplemental Information

## Data Availability

High-throughput data have been deposited in the Gene Expression Omnibus (GSE246541).
