# Supplementary material for: Amino acid influx via LAT1 regulates iron demand and sensitivity to PPMX-T003 of aggressive natural killer cell leukemia
Source: Leukemia. 2024 Jun 24;38(8):1731–41. doi: 10.1038/s41375-024-02296-6 (PMC11286515; doi:10.1038/s41375-024-02296-6)
Supplement: Supplementary file 1 — Supplemental Information [file 41375_2024_2296_MOESM1_ESM.pdf]

Supplemental Information

Supplemental Methods

Supplemental References

Supplemental Figures

Supplemental Tables

Supplemental Methods

Cell culture

HEK293T was purchased from Riken cell bank (#RCB2202) and cultured at 37°C with 5% CO_2 in D-MEM (FUJIFILM Wako, #043-30085) with 10% fetal bovine serum (FBS; Gibco, #26140079) and Penicillin-Streptomycin Solution (FUJIFILM Wako, #168-23191). Normal human lymphocyte was obtained from peripheral blood mononuclear cells of a healthy volunteer and cultured at 37°C with 5% CO_2 in RPMI1640 (FUJIFILM Wako, #189-02025) with 10% FBS. NK92 was purchased from the American Type Culture Collection, and KHYG1 was provided from the Japanese Collection of Research Bioresources. ANKL1 and ANKL3 are primary ANKL cells harvested from two lineages of previously established ANKL-PDXs, ANKL1-PDX and ANKL3-PDX, as described in our previous study. ANKL1 was cultured at 37°C in a CO_2-free incubator in Leibovitz's L-15 Medium (Gibco, #11415064) with 5% FBS, non-essential amino acids (FUJIFILM Wako, #139-15651), 100 units/mL human recombinant interleukin (IL)-2 (Peprotech, #200-02), and 0.1 mM 2-mercaptoethanol (FUJIFILM Wako, #131-14572). The other cells were cultured at 37°C with 5% CO_2 in Artemis Medium-1 (Nihon Techno Service; no available catalog number) with 2% heat-inactivated human serum (KOHJIN BIO, #12181201).

Antibodies and reagents

The following antibodies, purchased from BioLegend, were used for flow cytometry analysis:

biotin-conjugated anti-human CD45 (#34004), FITC-conjugated anti-human CD45 (#368508), APC-conjugated anti-human CD45 (#304012), DyLight 649 goat anti-mouse IgG (#405312), and Alexa Fluor 488 goat anti-mouse IgG (#405319). All antibodies were used at ×500 dilution. DNA was stained using propidium iodide (PI; Sigma-Aldrich, #P4170) for cell cycle analysis and dead cell detection. The antibody used for CD56 immunohistochemistry was purchased from Leica Biosystems (#CD56-504-L-CE). Antibodies used for western blotting were purchased and diluted as follows: mouse anti-beta actin (Sigma-Aldrich, #A5441; ×1,000 dilution), rabbit anti-CD71 (Proteintech, #10084-2-AP; ×1,000 dilution), rabbit anti-Myc (Cell Signaling Technology, #9402; ×500 dilution), rabbit anti-phospho-p70 S6 kinase (Cell Signaling Technology, #9205S; ×500 dilution), mouse anti-L-type amino acid transporter 1 (LAT1; Santa Cruz Biotechnology, #sc-37432; ×500 dilution), HRP-conjugated goat anti-mouse IgG (Proteintech, #SA0001-1; ×2,000 dilution), and HRP-conjugated goat anti-rabbit IgG (Proteintech, #SA00001-2; ×2,000 dilution). PPMX-T003, an anti-human transferrin receptor 1 inhibitory antibody, was kindly provided by Perseus Proteomics, Inc. JPH-203, a specific inhibitor of LAT1 was purchased from TargetMol (#TQ0081) and was diluted in dimethyl sulfoxide. Rapamycin, a mammalian target of rapamycin inhibitor, was purchased from AdipoGen (#AG-CN2-0025-C100) and was diluted in dimethyl sulfoxide.

Animal breeding and reproduction of ANKL-PDXs

Six-week-old female NOD/Shi-scid, IL-2RγKO (NOG) mice were purchased from In-Vivo Science and bred in a specific pathogen-free area of the animal facility of Tokai University, where the temperature, humidity, and illuminance are adequately controlled. Seven to eleven-week-old mice were used in all the experiments. All mice received humane care, and the humane endpoint was decided for mice whose body weight continuously decreased by more than 15% of their maximum body weight or showed remarkable debilitation. The ANKL-PDXs used in this study were established as described in our previously published article in detail(1). ANKL-PDXs were reproduced (passaged) by intravenous injection of ANKL cells harvested from ANKL-PDXs into another NOG mouse.

Harvesting primary ANKL cells from ANKL-PDXs

The liver and spleen were extracted from ANKL-PDXs seven to 11 days after inoculation with ANKL cells. Both organs were homogenized and filtered with a 70-µm cell strainer (Falcon, #352350), followed by enzymatic digestion in HBSS (+) (FUJIFILM Wako, #084-08965) containing 1 mg/mL collagenase A (Roche, #11088793001) and 100 U DNase I (Roche, #10104159001) for 15 min at 37°C. Hepatocytes were removed using density gradient centrifugation using 35% Percoll (Sigma-Aldrich, #P1644) diluted in HBSS (-) (FUJIFILM Wako, #085-09355). Finally, the ANKL cells were sorted using automated Magnetic-Activated Cell Sorting (autoMACS; Myltenyi Biotech) using a biotin-conjugated anti-human CD45 antibody

and anti-biotin microbeads (Miltenyi Biotech, #130-090-485). Harvested ANKL cells were stored in a deep freezer with a CELLBANKER (ZENOGEN PHARMA #11910). The original method for harvesting ANKL cells from PDX was described in our previous study(1).

Flow cytometric analysis and cell sorting

Cells were washed several times with 2% FBS-containing phosphate-buffered saline, followed by incubation in an adequate concentration of the antibody solution for 30–60 min. The stained cells were washed several times with 2% FBS-containing phosphate-buffered saline. For intracellular staining, cells were fixed and permeabilized with chilled 70% ethanol after staining the surface molecules and then blocked with 2% bovine serum albumin for 15 min, followed by intracellular antigen staining. Surface or intracellular molecular expression was analyzed using FACS Lyric or LSR Fortessa (Becton, Dickinson). Cell sorting was performed using a FACS Melody or Aria III (Becton, Dickinson). Data processing and analysis were performed using FlowJo version 10 (Becton, Dickinson).

Western blotting

Cells were directly lysed in 1x Laemmli sample buffer (60 mM Tris-HCl pH 6.8, 2% sodium dodecyl sulfate, 10% glycerol, 5% 2-mercaptoethanol, and 0.02% bromophenol blue), followed by boiling at 96°C in a heat block for 10 min. Samples were applied in 10–15% acrylamide gel

electrophoresis. Proteins in acrylamide were then transferred to 0.2-μm poly-vinylidene di-fluoride membrane (FUJIFILM, #033-22453) using HorizeBLOT 4M-R (ATTO, #2322470). Membranes were blocked with 5% skim milk for 2 h. Primary antibodies were diluted with 5% skim milk and applied to the membranes at room temperature for 2 h. After washing the membrane with Tris-buffered saline containing 0.05% Tween 20, the secondary antibody was diluted with 5% skim milk and incubated at room temperature for 45 min. After washing the membrane, an adequate volume of Immobilon Western Chemiluminescent HRP substrate (Merck Millipore, #WBKLS0100) was applied, and luminescence was detected using FUSION SOLO.7S.EDGE (Vilber Bio Imaging).

Quantitative PCR

Total cellular RNA was extracted using Sepasol-RNA I Super G (Nacalai Tesque, #09379-55) followed by reverse transcription using ReverTra Ace qPCR Master Mix with gDNA Remover (TOYOBO, #FSQ-301). PCR was performed using THUNDERBIRD SYBR qPCR Mix (TOYOBO, #QPS-201) and StepOnePlus Real-Time PCR System (Applied Biosystems). *ACTB* expression value was selected as an internal control, and the $2^{-\Delta\Delta CT}$ method was used for data analysis. Primer sequences used for PCR were as follows; *ACTB*-Forward: 5′-CTCTTCCAGCCTTCCTTCCT-3′, *ACTB*-Reverse: 5′-AGCACTGTGTTGGCGTACAG-3′, *MYC*-Forward: 5′-CACCAGCAGCGACTCTGA-3′, *MYC*-

Reverse: 5′-GATCCAGACTCTGACCTTTTGC-3′, *TFRC*-Forward: 5′-ACTTCTTCCGTGCTACTTCCAG-3′, *TFRC*-Reverse: 5′-ACTCCACTCTCATGACACGATC-3′, *TXNIP*-Forward: 5′-CAGCAGTGCAAACAGACTTCGG-3′.

Transduction using lentiviral vector

Second-generation lentiviral packaging vectors psPAX2 (Addgene, #12260) and pMD2.G (Addgene, #12259) were cotransfected into HEK293T cells with plasmids containing the proposed gene (encoding sgRNA, Cas9, or luciferase) using PEI MAX (Polysciences, #24765-1). The culture supernatant was collected 48 h after transfection and centrifuged at 10,000 × *g* for 4 h to concentrate the lentiviruses. Target cells were then treated with concentrated lentiviral supernatant on culture plates coated with RetroNectin (Takara Bio, #T100A) and centrifuged at 1,000 × *g* for 90 min for infection.

sgRNA designation and related plasmid construction

sgRNAs targeting specific genes were designed by an online-available tool, CHOPCHOP (http://chopchop.cbu.uib.no/). The non-target sgRNA sequence was obtained from BRDN0001149198 (#80248; Addgene). Oligonucleotides were obtained from Eurofin Genomics and ligated into the FG12 (Addgene, #14884)-based sgRNA and mRFP expression plasmid, which was kindly provided by Prof. Hirao, after enzymatic digestion using *BsmBI* (New England

BioLabs, #R0580S) according to the manufacturer's protocol. Sequences of sgRNAs were as follows: non-target sgRNA (sgNT): 5′-GTATTACTGATATTGGTGGG-3′, sg*SLC7A5*: 5′-TGGCGGGTAGCGGCTCACCA-3′, sg*SLC1A5*: 5′-CAGCGCCACACCAAAGACGA-3′.

CRISPR-Cas9 gene knockout procedure

Cells were sequentially transduced with lentiCas9-Venus (Addgene, #70267) and the sgRNA plasmid as described above, followed by purification using flow cytometry for 48 h or more after transduction.

In vivo imaging assay

The Lampyridae luciferase gene was cloned into CSII-CMV-MCS (Riken, #RDB04377) and digested with *AgeI* (New England Biolabs, #R3552S) and *BamHI* (New England Biolabs, #R3136S). ANKL1 and ANKL3 cells were then transduced with the constructed vectors ($ANKL1^{Luc}$ and $ANKL3^{Luc}$, respectively), and ANKL-PDXs were established using them. On the day of the analysis, 150 mg/kg luciferin (Promega, #P1043) was intraperitoneally injected, and luminescence was detected using an IVIS Lumina II (Caliper Life Science) under 2.5% isoflurane inhalation. Luminescence was measured using the Living Image software (version 4.3.1; PerkinElmer).

Histological analysis

The livers and spleens were obtained from sacrificed ANKL-PDX mice, fixed in 4% formaldehyde, and embedded in paraffin. The sections were stained with hematoxylin and eosin. Immunohistochemistry was performed using Bond Polymer Refine Detection (Leica, #DS9800), as previously reported(2). Images of the tissue slides were scanned and converted into a virtual slide image format using CellSense software (Olympus) and observed using NDP.view2 (Hamamatsu Photonics).

Transcriptomic analysis with publicly-available RNA-seq data

Transcriptomic data of primary ANKL and normal NK cells used in our previous study were obtained from the Gene Expression Omnibus (GSE189722)[1]. FASTQ files were mapped to GRCh38.95, available in Ensembl (https://www.ensembl.org; accessed on 10th Sep. 2021), using HISAT2 version 2.2.0. The mapped reads were counted using StringTie version 2.1.3b. Differentially expressed genes were detected using DESeq2 version 1.38.3, and gene set enrichment analysis was performed using clusterProfiler version 4.6.2, with a GMT-formatted file of HALLMARK of human MSigDB v2023.1, as described in the Methods section of the manuscript.

DNA damage detection assay

The cells were cultured for adequate periods with or without reagents, harvested, fixed, and

permeabilized with chilled 70% ethanol. DNA damage was detected via the detection of γH2AX using DNA Damage Detection Kit (Dojindo, #343-09421), following the manufacturer's protocol. DyLight 649 goat anti-mouse IgG antibody was used as the secondary antibody in several analyses instead of the secondary antibody in the kit. Fluorescence was detected using flow cytometry.

Single-amino acid deprivation assay

Amino acid-deprived RPMI-1640 medium was purchased from the Research Institute for the Functional Peptides. Purified amino acids were purchased from FUJIFILM Wako, as follows; L(+)-Arginine (#019-04611), L-Asparagine Monohydrate (#019-04812), L-Aspartic Acid (#013-04832), L-Glutamic Acid (#070-00502), Glycine (#073-00732), L-Histidine (#086-00681), L-Isoleucine (#123-00861), L-Leucine (#126-00851), L-Methionine (#135-01601), L(-)-Phenylalanine (163-01301), L(-)-Proline (#163-04601), L-Serine (#191-00401), L-Threonine (#206-01321), L-Tryptophan (#206-03381), L-Valine (#220-00081), L-Cysteine (#039-20652), and L-Tyrosine (#204-03561). L-Glutamine was purchased from Gibco (#2530081). Adequate volumes of each amino acid were independently added to the amino acid-deprived RPMI-1640 medium at the same concentration as the original RPMI-1640, and a single-amino acid-deprived RPMI-1640 medium was prepared. NK92, KHYG1, ANKL1, and ANKL3 cells were cultured at 37°C with 5% CO_2 in single-amino acid-deprived RPMI1640 with 10% FBS and 100 units/mL

recombinant human IL-2 for 24 h (ANKL1) or 48 h (other cells). Cultured cells were harvested and counted using the MTT Cell Count Kit (Nacalai Tesque, #23506-80; for NK92 and KHYG1) or trypan blue staining (FUJIFILM Wako, #207-17081; for ANKL1 and ANKL3), following the manufacturer's protocols.

In vitro growth competition assay of single gene-knocked-out cells

Cas9-Venus overexpressed cells were transduced with the sgRNA-RFP expression plasmid and cultured without further purification. The proportion of RFP^+ cells in $Venus^+$ cells was measured using flow cytometry five and eight days after transduction, and the relative existence ratio of knocked-out cells in eight days compared with five days after transduction was calculated. The lentivirus titer was adjusted as the RFP^+ cell ratio reached 40–60% five days after transduction.

In vivo growth competition assay of single gene-knocked out ANKL cells in the liver

Cas9-Venus overexpressed ANKL1 and ANKL3 cells were transduced with sgRNA-RFP expression plasmid and purified RFP^+ cells 48 h after transduction using flow cytometry with more than 90% efficiency. The sorted cells were intravenously inoculated into NOG mice and harvested from the liver seven days after injection. The collected cells were analyzed using flow cytometry to calculate the RFP+ proportion in Venus+ cells.

Cell growth and viability assay

First, 3×10^4 cells (cell lines) or 1×10^5 cells (primary cells) were cultured at 37°C with 5% CO_2 in 800 μL adequate culture medium with antibodies or reagents for 48 h (cell lines) or 24 h (primary cells). Then, cells were transferred into a 96-well plate, and an MTT assay or trypan blue staining was performed to assess cell growth or viability following the manufacturer's protocol, as described above.

Live cell staining of intracellular ferrous ions and mitochondrial reactive oxygen species

Intracellular ferrous ions and mitochondrial reactive oxygen species were stained with FerroOrange and mtSOX deep red (Dojindo, #F374 and #MT14, respectively) following the manufacturer's protocol. FerroOrange and mtSOX deep red fluorescence was detected using light microscopy (IX83; Olympus) and flow cytometry, respectively.

Cell cycle assay

Harvested cells were fixed and permeabilized, and then RNAs were digested using 20 μg/mL RNase (NIPPON GENE, #313-01461). Cellular DNA was stained with 50 μg/mL PI, and fluorescence was measured using flow cytometry to determine the cell cycle. To detect DNA-synthesizing cells of liver-resident ANKL cells *in vivo* for better separation of the G0/1 and S-phase cell cycles, 50 mg/kg EdU was intraperitoneally inoculated into ANKL-PDX two hours before sacrifice. EdU was labeled using the Click-iT Plus EdU Flow Cytometry Assay Kit

(Thermo Fisher Scientific, #C10634) and detected using flow cytometry.

Supplemental References

1. Kameda K, Yanagiya R, Miyatake Y, Carreras J, Higuchi H, Murayama H, et al. The hepatic niche leads to aggressive natural killer cell leukemia proliferation through the transferrin-transferrin receptor 1 axis. Blood. 2023 Jul 27;142(4):352–64.
2. Carreras J, Kikuti YY, Beà S, Miyaoka M, Hiraiwa S, Ikoma H, et al. Clinicopathological characteristics and genomic profile of primary sinonasal tract diffuse large B cell lymphoma (DLBCL) reveals gain at 1q31 and RGS1 encoding protein; high RGS1 immunohistochemical expression associates with poor overall survival in DLBCL not otherwise specified (NOS). Histopathology. 2017 Mar;70(4):595–621.

Supplemental Figures

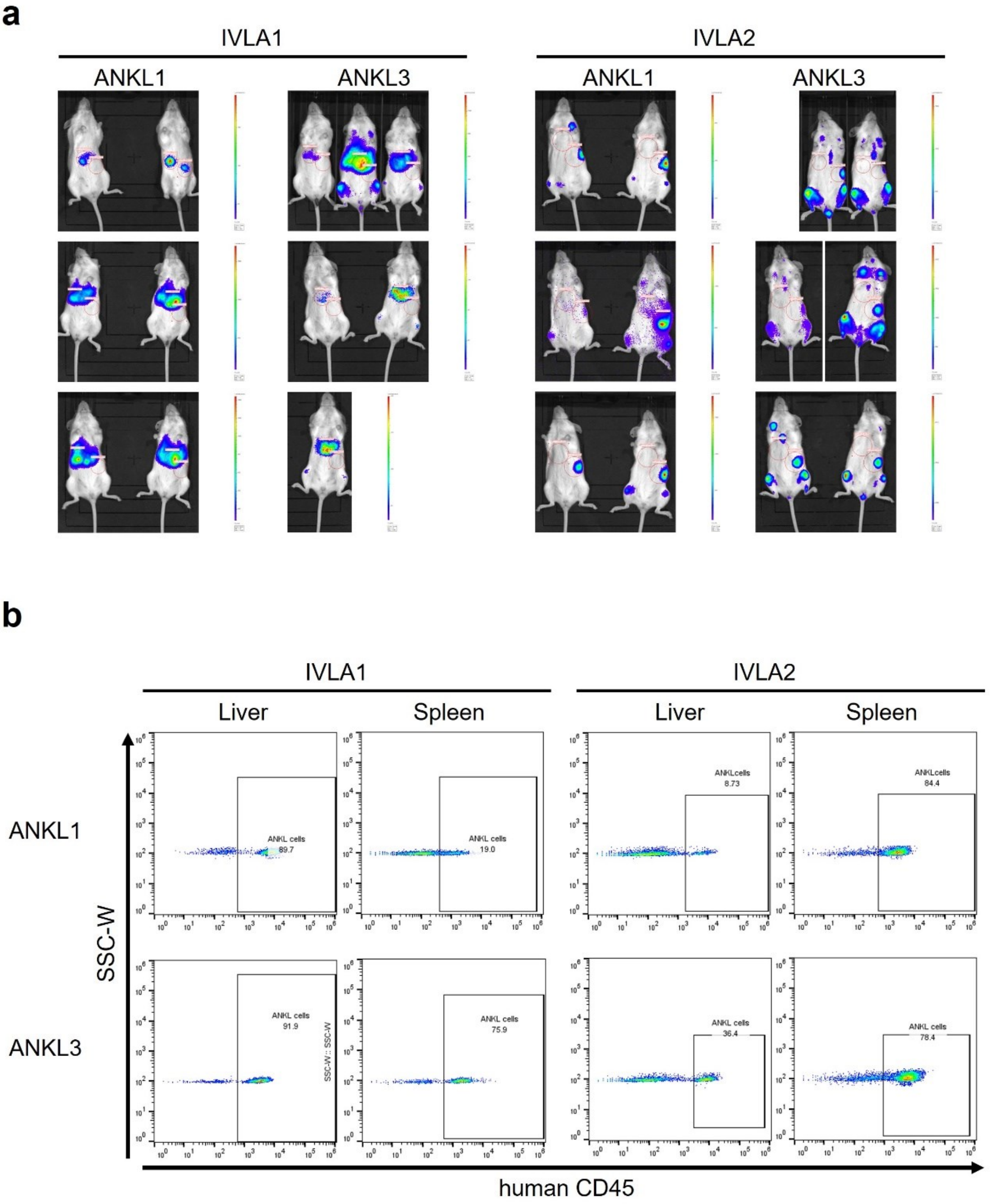

Supplemental Figure S1. ANKL cell existence in the liver and spleen of ANKL-PDXs before and after treatment with PPMX-T003.

a. Raw IVIS images of ANKL-PDXs at IVLA1 and IVLA2 stages shown in **Figure 1b**. Six mice per stage per lineage were analyzed.

b. Gating images of flow cytometric analyses shown in **Figure 1c**.

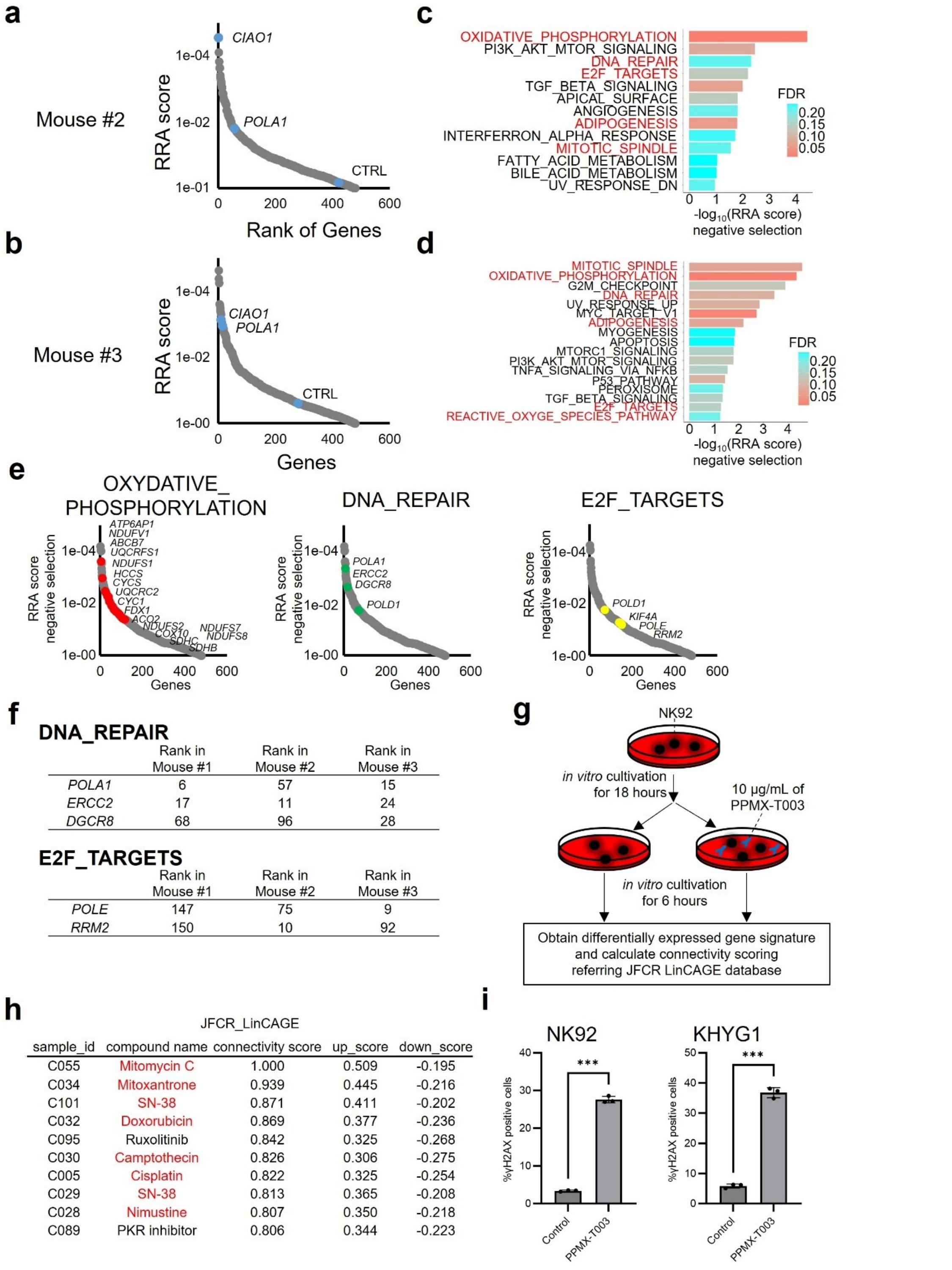

Supplemental Figure S2. ANKL cells require iron for DNA replication/repair and oxidative phosphorylation.

a–b. Negative selection RRA scores of all Mouse #2 (**a**) and #3 (**b**) genes. Blue dots indicate the RRA values of sgRNAs targeting *CIAO1* and *POLA1*, and non-target sgRNA (CTRL).

c–d. All dropped-out gene sets in Mouse #2 (**c**) and #3 (**d**) using GSEA. Color values indicate FDR and red characters indicate five commonly dropped-out gene sets, as described in **Figure 3c**.

e. Negative selection RRA scores of all genes of Mouse #1. Red, green, and yellow dots indicate genes annotated in oxidative phosphorylation, DNA repair, and E2F targets, respectively, in MSigDB-HALLMARK.

f. Commonly dropped out sgRNAs targeting genes classified as DNA repair and E2F targets of HALLMARK gene sets in all 3 mice and their rank of negative selection RRA scores.

g. A schema of sample preparation for connectivity scoring analysis.

h. Ranking of connectivity scoring analysis referring to JFCR LinCAGE. Cytotoxic agents causing DNA damage were described with red character.

i. Measurement of γH2AX-positive cell proportion shown in **Figure 2g**. Total of three samples per treatment group were analyzed.

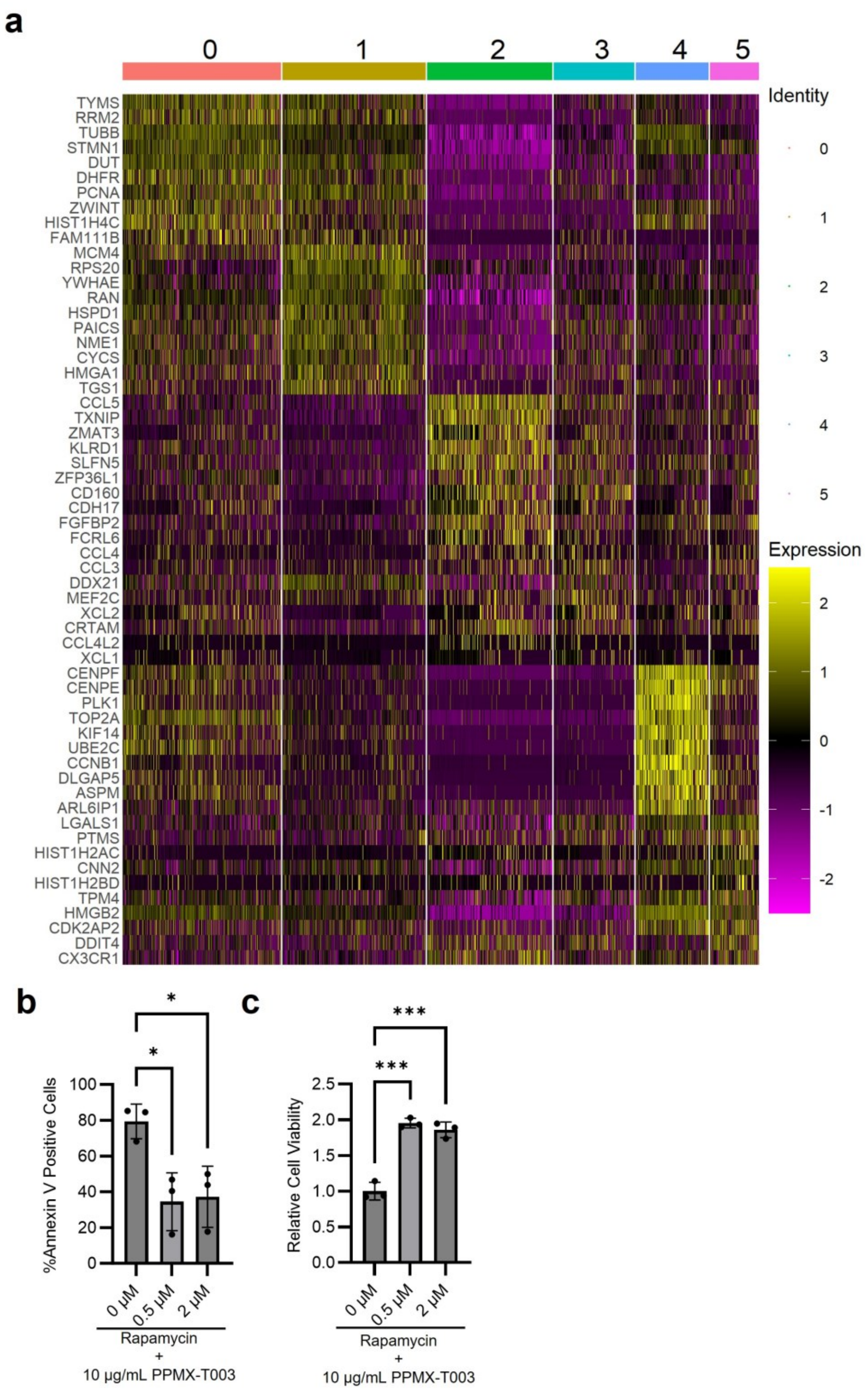

Supplemental Figure S3. Single cell RNA-sequencing of ANKL1 cells treated with PPMX-T003 indicated the association of mTORC1 activity and cellular sensitivity to PPMX-T003.

a. Heatmap of top 10 feature genes in each single cell transcriptome analysis cluster.

b, c. Apoptosis assay (**b**) and MTT cell viability assay (**c**) of NK92 treated with various concentrations of rapamycin and 10 μg/mL of PPMX-T003 *in vitro* for 48 h. Total of three samples per treatment group were analyzed.

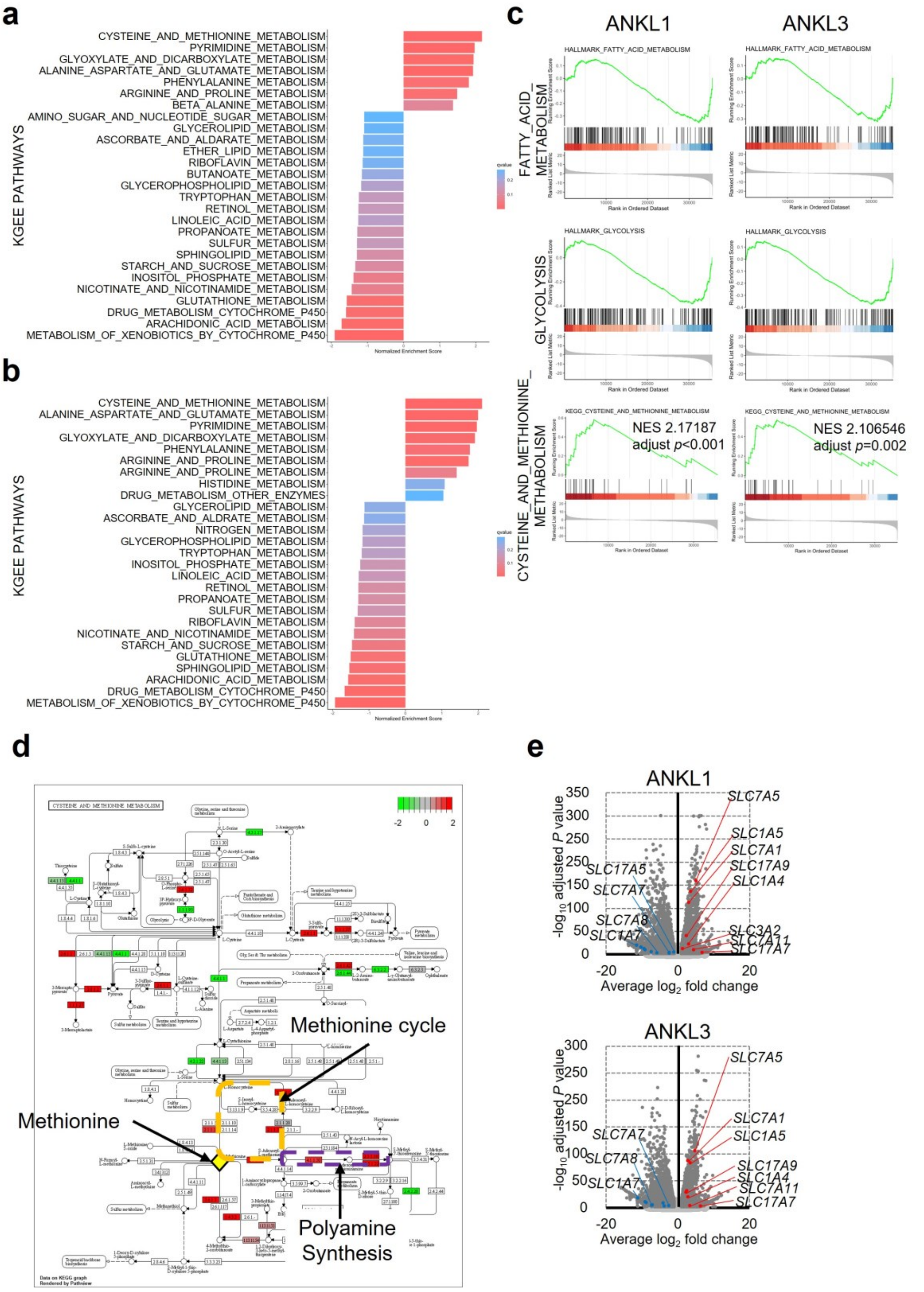

Supplemental Figure S4. ANKL cells require LAT1-mediated amino acid influx for proliferation.

a–b. GSEA of liver-derived ANKL1 (**a**) or ANKL3 (**b**) cells compared with normal NK cells using

the KEGG pathway database. Pathways associated with metabolism with an adjusted *p* value of less than 0.25 were extracted.

c. GSEA plots of “FATTY_ACID_METABOLISM”, “GLYCOLYSIS”, and “CYSTEINE_AND_METHIONINE_METABOLISM” comparing liver-derived ANKL and normal NK cells.

d. Pathview of “KEGG_CYSTEIN_AND METHIONINE_METABOLISM” in **Supplemental Figure S3a** (liver-derived ANKL1 cells versus normal NK cells). The yellow rhombus, orange dashed line, and purple dashed line indicate the methionine, methionine cycle, and polyamine synthesis pathway, respectively.

e. Volcano plots of the bulk transcriptome of liver-derived ANKL and normal NK cells. Red (increase) and blue (decrease) dots indicate SLC family genes and annotated dots indicate amino acid transporters.

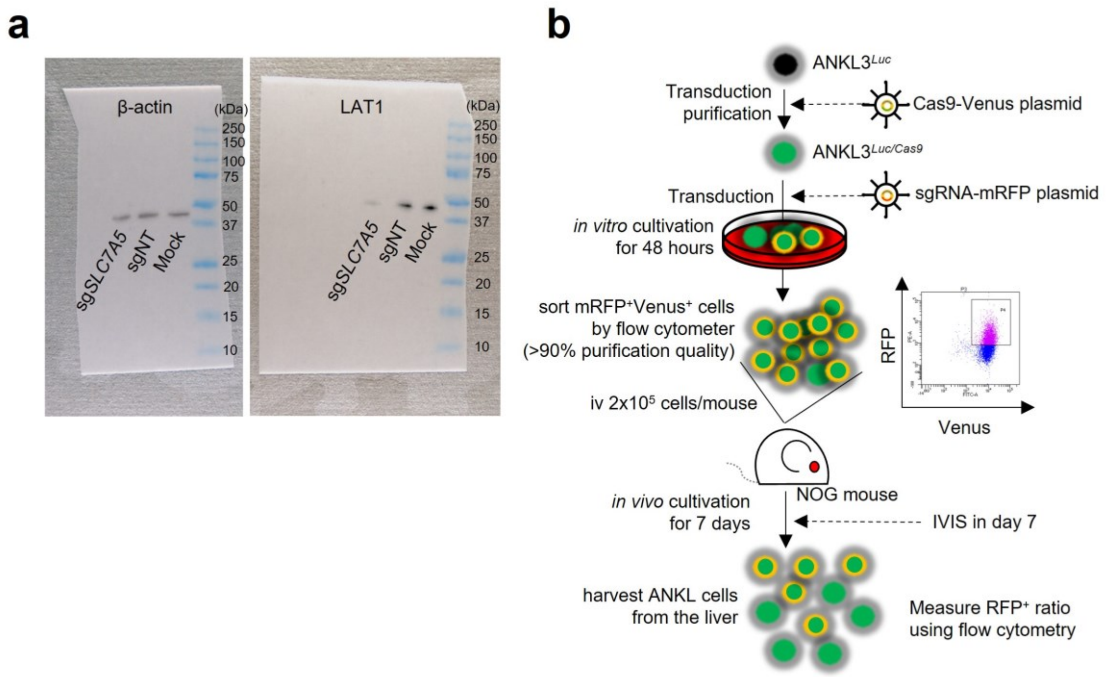

Supplemental Figure S5. CRISPR-Cas9 mediated *SLC7A5* knockout analyses.

a. Western blotting of Cas9-overexpressed NK92 cells transduced with sgNT-, sg*SLC7A5*-, and empty (mock)-RFP plasmids to validate the knockout efficiency of LAT1 due to sg*SLC7A5*.

b. Scheme of *in vivo* competitive proliferation assay of ANKL3 cells transduced with Cas9 and sgRNAs.

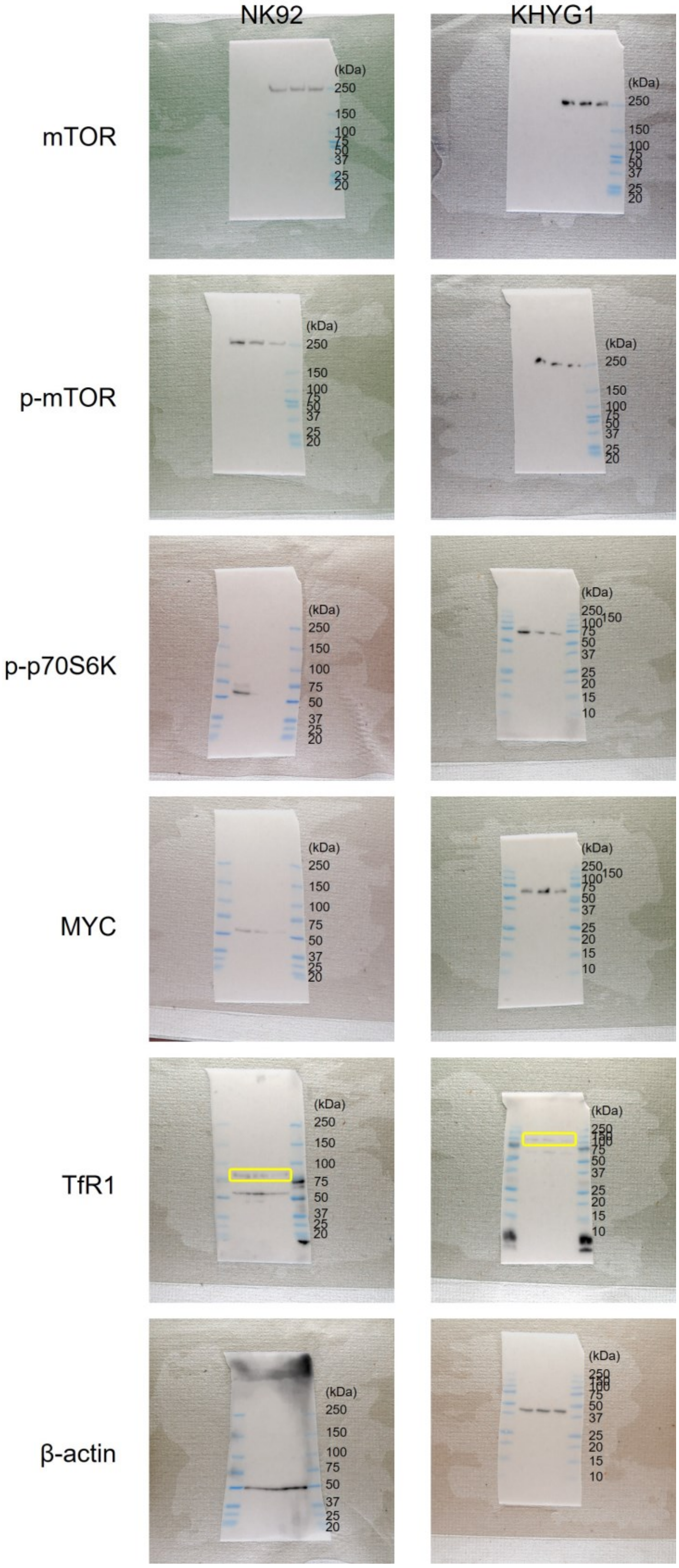

Supplemental Figure S6. Non-cropped pictures of western blotting shown in Figure 6d.

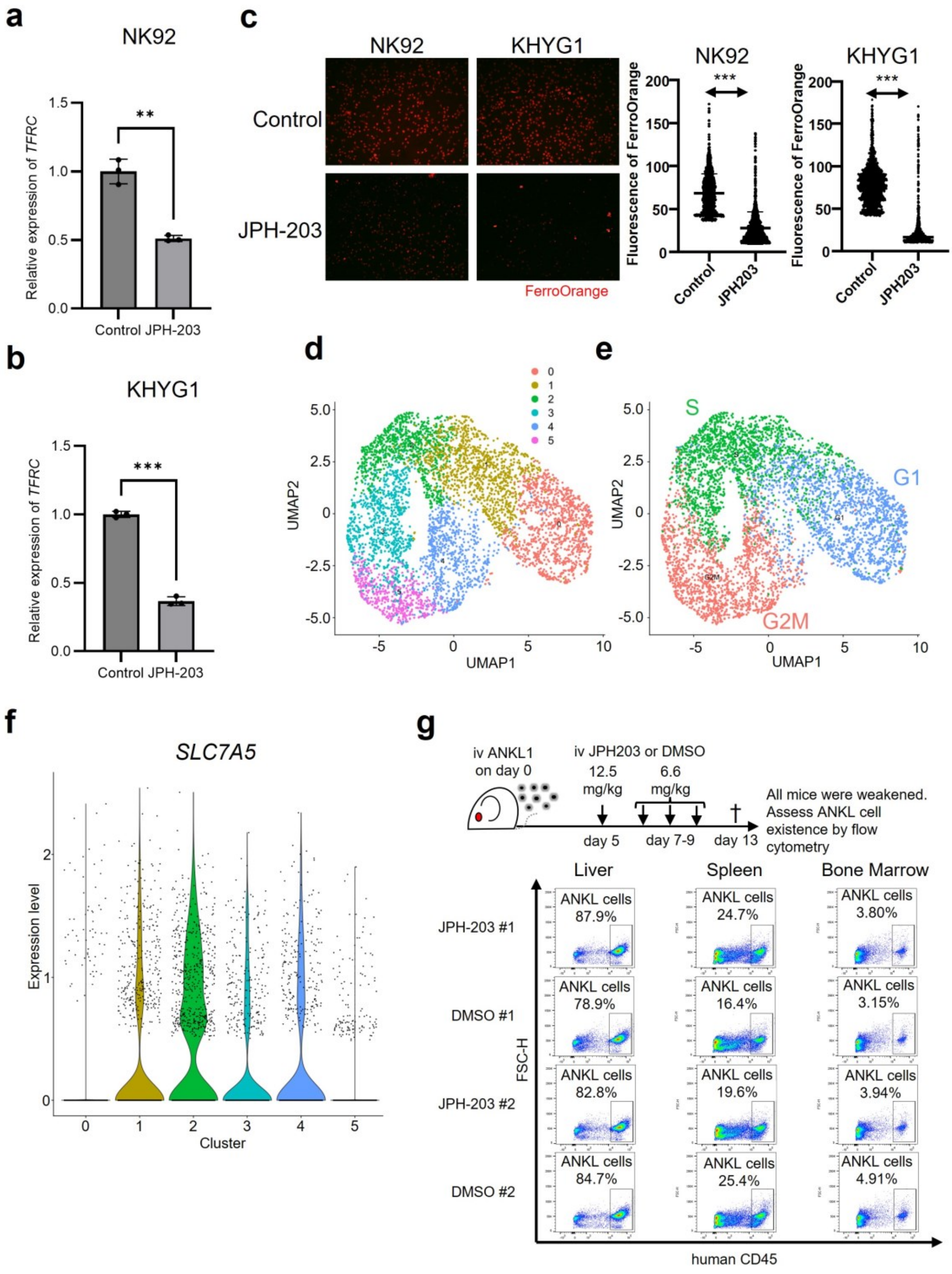

Supplemental Figure S7. JPH203 reduces mTOR/MYC activity and cellular content of ferrous ions.

a–b. Quantitative PCR of *TFRC* transcription of NK92 (**a**) and KHYG1 (**b**) treated with 40 μM of JPH-203 for 6 h.

c. Intracellular ferrous ion (Fe^{2+}) staining of control or JPH-203-treated NK92 and KHYG1 cells, using FerroOrange with or without the existence of 40 μM JPH-203.

d. Unsupervised clustering of pretreated liver-derived ANKL1 cells described in **Figure 3a**.

e. Cell cycle scoring analysis of liver-derived ANKL1 cells.

f. Violin plot of *SLC7A5* expression of liver-derived ANKL1 cells per cluster.

g. Flow cytometric analysis of ANKL1-PDXs treated with JPH-203 or dimethyl sulfoxide (DMSO). Total of two mice per group were analyzed, and all mice were weakened and sacrificed in day 13.

Supplemental Table S1. sgRNA library targeting genes encode iron-requirement molecules

#	sgRNA ID	Symbol	Chromosome	sgRNA location	Genomic strand targeted	sgRNA sequence
1	sgABAT_1	ABAT	chr16	8862079	-	GGAACGCGCCCATGAAGGAG
2	sgABAT_2	ABAT	chr16	8868765	+	GTGGACGAGGTCCAGACCGG
3	sgABAT_3	ABAT	chr16	8851624	-	TGAGTTTCAGCAGGGCGGGG
4	sgABAT_4	ABAT	chr16	8860079	+	GCAGAGGGGCTTCTCCCAGG
5	sgABAT_5	ABAT	chr16	8862054	-	GCTGTAGTCGGGGCAGCCAG
6	sgABAT_6	ABAT	chr16	8862788	+	TGTGAAAGAGAACCAACAGG
7	sgABAT_7	ABAT	chr16	8829604	-	GCGCTGGGCGAGCAACATGG
8	sgABAT_8	ABAT	chr16	8829631	-	GTAGCTGTGCTGGAAGCTGC
9	sgABAT_9	ABAT	chr16	8829653	-	GCTTACCAGGCACCAGCAGG
10	sgABAT_10	ABAT	chr16	8839920	-	GGGACTTCCGTCTTCATCAG
11	sgABCB6_1	ABCB6	chr2	220075503	-	CGGGGACTGAAGCTGAGCGG
12	sgABCB6_2	ABCB6	chr2	220083173	+	GCACGTAGGGAGAGATGCGA
13	sgABCB6_3	ABCB6	chr2	220083135	+	GGGCAGCGCCGCCTGAAGTG
14	sgABCB6_4	ABCB6	chr2	220083045	+	GGCCAGACTCTCCAGCACGG
15	sgABCB6_5	ABCB6	chr2	220082874	-	GGAACAGCCCACAGTGGTGG
16	sgABCB6_6	ABCB6	chr2	220082440	+	GGACTGGGGACGAAGTCCAG
17	sgABCB6_7	ABCB6	chr2	220081482	+	ACAGGTAGCCACTCAGGAGG
18	sgABCB6_8	ABCB6	chr2	220078317	+	GTAGGTGCCAAACCAATTGA
19	sgABCB6_9	ABCB6	chr2	220074959	-	GGATGGCTGCATCGTGGAGA
20	sgABCB6_10	ABCB6	chr2	220083207	+	AGACAGCGAATCAGCACCAG
21	sgABCB7_1	ABCB7	chrX	74375988	-	TTAGCGGCTCAGGTCCGCAG
22	sgABCB7_2	ABCB7	chrX	74288866	-	TGAGCCTGGAAAGCCTTCGG
23	sgABCB7_3	ABCB7	chrX	74334591	-	ACAGTTCTTAGATGCTGCAA
24	sgABCB7_4	ABCB7	chrX	74332749	-	TGGCATGGTCATGCAGGAGG
25	sgABCB7_5	ABCB7	chrX	74332723	-	CCACACAGACCCAAAAGAAG
26	sgABCB7_6	ABCB7	chrX	74293708	-	TCACAGTTGCAGTCACACGG
27	sgABCB7_7	ABCB7	chrX	74290276	+	ACAGTTCCCAGAAAGTTCAG
28	sgABCB7_8	ABCB7	chrX	74284929	-	TGGATATGACACCCAAGTAG
29	sgABCB7_9	ABCB7	chrX	74376048	+	CTTTTCGAAAGCAGCCGCCG
30	sgABCB7_10	ABCB7	chrX	74376015	+	TAAAGGCCGGATCAGAATCG
31	sgABCE1_1	ABCE1	chr4	146042361	+	GGAAGACTTAAACCTGATGA
32	sgABCE1_2	ABCE1	chr4	146044414	-	CAGCAGGTTTGCCCAAGCAA
33	sgABCE1_3	ABCE1	chr4	146029177	-	GACAATTGATAAGGCGCCAA
34	sgABCE1_4	ABCE1	chr4	146031261	-	AAAATCTCCTGCCAGTCAGG
35	sgABCE1_5	ABCE1	chr4	146031371	+	CCAGATTCCTAAGGCTGCAA
36	sgABCE1_6	ABCE1	chr4	146031360	-	GGAATCTGGTCTACATATTG
37	sgABCE1_7	ABCE1	chr4	146031514	+	TATATTGTGGCAGGGGACAG
38	sgABCE1_8	ABCE1	chr4	146029246	-	CCTGTGAAGTTTGAAGGCAT
39	sgABCE1_9	ABCE1	chr4	146031381	-	AAACCGACCTTTGCAGCCTT
40	sgABCE1_10	ABCE1	chr4	146044400	+	GTAGCTTTAGCCCTTTGCTT
41	sgABCG2_1	ABCG2	chr4	89034518	+	GAGTTGATGACAGAAGGAGG
42	sgABCG2_2	ABCG2	chr4	89053743	-	GCCATCCTGGGACCCACAGG
43	sgABCG2_3	ABCG2	chr4	89053034	-	GCTGCAAGGAAAGATCCAAG
44	sgABCG2_4	ABCG2	chr4	89022423	+	AACACTGCTGAAACACTGGT
45	sgABCG2_5	ABCG2	chr4	89053728	+	GAAGATTTGCCTCCACCTGT
46	sgABCG2_6	ABCG2	chr4	89052227	-	AGAGTTAGGTCTGGATAAAG
47	sgABCG2_7	ABCG2	chr4	89042840	+	AGGCTCATCCAAGAACAAGA
48	sgABCG2_8	ABCG2	chr4	89039262	+	AGCTGATTCAAAGTATCCCA
49	sgABCG2_9	ABCG2	chr4	89036139	-	TAATGGAGATTCCACTGCTG
50	sgABCG2_10	ABCG2	chr4	89036125	+	TCTGTTTAATGCCACAGCAG
51	sgACO1_1	ACO1	chr9	32418367	-	GGTGGATGATTCCTGAGCCA
52	sgACO1_2	ACO1	chr9	32420859	-	CCACTACCCCAACCTGGCGG
53	sgACO1_3	ACO1	chr9	32429408	+	GGTGTCTAGGTTTGACGTGG
54	sgACO1_4	ACO1	chr9	32418410	+	AGAGTGGTATTTGATCAGGA
55	sgACO1_5	ACO1	chr9	32419132	-	AGTGGATGTTACCAGAGGGT
56	sgACO1_6	ACO1	chr9	32423396	+	TCAAGACCCAGACTTCACCC
57	sgACO1_7	ACO1	chr9	32425918	-	GACCACAGAACCATGAGCAA
58	sgACO1_8	ACO1	chr9	32429442	+	ATGACCTGCATTGGCAACAG
59	sgACO1_9	ACO1	chr9	32434677	+	GCTCGCTACTTAACTAACAG

60	sgACO1_10	ACO1	chr9	32436284	-	TGACGGCGTCATTACCTCGG
61	sgACO2_1	ACO2	chr22	41919830	+	GCAGGAAGGACATCAAGAAG
62	sgACO2_2	ACO2	chr22	41904025	+	GGGGCGAGAAAGACCTGCGC
63	sgACO2_3	ACO2	chr22	41907916	+	GCAGGTGCCAAATATGGCGT
64	sgACO2_4	ACO2	chr22	41920918	+	GGATGGCAAGAAGTTCAGGC
65	sgACO2_5	ACO2	chr22	41923318	+	CGGAGACGAGAACTACGGCG
66	sgACO2_6	ACO2	chr22	41923944	-	GTAGTCAGCCGGGTCAGCGA
67	sgACO2_7	ACO2	chr22	41903954	+	CAGCAGCGGGCTGTCCAAGG
68	sgACO2_8	ACO2	chr22	41911843	+	GGCATCCTCACGGTGAAAGG
69	sgACO2_9	ACO2	chr22	41916258	+	ATGGCCTCTGGACATCCGAG
70	sgACO2_10	ACO2	chr22	41918972	-	ATAGCCGTCCCGCTCAATGG
71	sgACP5_1	ACP5	chr19	11687213	-	GCTCAAGAAACAGCTGGCGG
72	sgACP5_2	ACP5	chr19	11688061	+	AGGGGTGGCACCATCAGCCA
73	sgACP5_3	ACP5	chr19	11688029	-	GTAGCCGTGGGTGACTGGGG
74	sgACP5_4	ACP5	chr19	11687562	+	GGGCAGAGACATTGCCAAGG
75	sgACP5_5	ACP5	chr19	11687375	+	GCAGGCGGTAGAAAGGGCTG
76	sgACP5_6	ACP5	chr19	11687120	+	GTAGCTGCTTGACCAGGCAG
77	sgACP5_7	ACP5	chr19	11688046	+	TACAAAGCGCAGGGCAGGGG
78	sgACP5_8	ACP5	chr19	11687604	+	CGTACCAGGGCACTTTGCGA
79	sgACP5_9	ACP5	chr19	11687192	-	GGCCAGGGAGGACTACGTGC
80	sgACP5_10	ACP5	chr19	11687979	-	CCGGGAAATGGCCAATGCCA
81	sgADHFE1_1	ADHFE1	chr8	67356882	-	GCACAGGAGGGAGCTTGGAG
82	sgADHFE1_2	ADHFE1	chr8	67361147	-	GGCATGTGGAGGGTGTGCAG
83	sgADHFE1_3	ADHFE1	chr8	67361110	+	GAGAGCCATCAAACCCACAC
84	sgADHFE1_4	ADHFE1	chr8	67361184	-	AAGCACATCAAAGCCACTGT
85	sgADHFE1_5	ADHFE1	chr8	67366371	+	GAAATGCTGGTGTTCATCTG
86	sgADHFE1_6	ADHFE1	chr8	67369310	-	TGAGCACCACAGAAAGGCCA
87	sgADHFE1_7	ADHFE1	chr8	67369336	-	CGTGAAAGTGAACACCGCTG
88	sgADHFE1_8	ADHFE1	chr8	67356920	-	GGGGATGCCATTCTTCACTA
89	sgADHFE1_9	ADHFE1	chr8	67357496	+	GCCTATGTTGCTGTCGGTGG
90	sgADHFE1_10	ADHFE1	chr8	67357563	-	TAGGAAATCAGAATGAGGGC
91	sgADI1_1	ADI1	chr2	3504600	+	GAAGCGGTGATAGATCCCCG
92	sgADI1_2	ADI1	chr2	3523168	+	GCTGCTCCAGGCCCACTGGG
93	sgADI1_3	ADI1	chr2	3504666	-	TGAGGGACAAGGAGGACCAG
94	sgADI1_4	ADI1	chr2	3504619	-	GACATGGTGACGCTCCCCGC
95	sgADI1_5	ADI1	chr2	3517682	-	GAAGAGAGAGGAACTACTCC
96	sgADI1_6	ADI1	chr2	3504644	-	GATCCGGATCTTCATGGAGA
97	sgADI1_7	ADI1	chr2	3517694	-	TAGAAAAGATCCGAAGAGAG
98	sgADI1_8	ADI1	chr2	3504684	-	GCAGTGGGTACTTCGATGTG
99	sgADI1_9	ADI1	chr2	3523228	-	CTGGTATATGGACGACGCCC
100	sgADI1_10	ADI1	chr2	3504706	-	GAGATCCGCTACATCCTGGA
101	sgADO_1	ADO	chr10	64565219	+	GACAAGCTAGACGCGGGCGG
102	sgADO_2	ADO	chr10	64565029	-	GCTGCAGTGTGGCCTTGCGC
103	sgADO_3	ADO	chr10	64565161	-	GCATGCCGTGCATGCCCGGG
104	sgADO_4	ADO	chr10	64565332	+	TTCGCGGGCCGAGTACACCG
105	sgADO_5	ADO	chr10	64565365	-	GGTGCGGTGTGAGGATGCAG
106	sgADO_6	ADO	chr10	64565497	+	GGAGCCGGTCAGGCCCAAGG
107	sgADO_7	ADO	chr10	64564864	-	GGAAGGTGAGGCAAGCCTGG
108	sgADO_8	ADO	chr10	64565077	+	CTACATGCACATCTACGAGA
109	sgADO_9	ADO	chr10	64565113	-	TCTTGAGCAGGAACACGCCC
110	sgADO_10	ADO	chr10	64565145	-	CGGGTGGTCGTGCAGCGGGA
111	sgAFM_1	AFM	chr4	74350026	+	GGAAAAGCTGGTGAAAGACA
112	sgAFM_2	AFM	chr4	74351720	-	GGATCCAGGGTAGGGAAAGG
113	sgAFM_3	AFM	chr4	74357655	+	GAAAAGAAAATACCAGAGCG
114	sgAFM_4	AFM	chr4	74361129	-	GCGTAACGGTAACAACCTGG
115	sgAFM_5	AFM	chr4	74365862	-	AAATCTTGAGAGAAAGGTGG
116	sgAFM_6	AFM	chr4	74352741	+	TGTTGCTGTTCATTTTGAGG
117	sgAFM_7	AFM	chr4	74365823	-	GCTTTCAAACTCTCAAAGCA
118	sgAFM_8	AFM	chr4	74347551	+	TAACCCTGCCCACACAACCT
119	sgAFM_9	AFM	chr4	74352730	-	ACAGCAACAGTTAGAAGTGT
120	sgAFM_10	AFM	chr4	74363428	+	ACATTTCCAGAATTTGGGGA

121	sgAFP_1	AFP	chr4	74315789	+	GCATTGGCAAAGCGAAGCTG
122	sgAFP_2	AFP	chr4	74319503	+	GCAAAAGCCACAAATAACAG
123	sgAFP_3	AFP	chr4	74318154	+	GAAATGACTCCAGTAAACCC
124	sgAFP_4	AFP	chr4	74310789	+	GTACATGAGCACTGTTGCAG
125	sgAFP_5	AFP	chr4	74316373	-	GATGGCCATCAGCTCCGACG
126	sgAFP_6	AFP	chr4	74303967	+	ACTGCAATTGAGAAACCCAC
127	sgAFP_7	AFP	chr4	74303985	-	GAAGACTGTTCATCTCCAGT
128	sgAFP_8	AFP	chr4	74308059	-	GCAGCCCAAAGAAGAATTGT
129	sgAFP_9	AFP	chr4	74310816	+	GTGCTGGATTGTCTGCAGGA
130	sgAFP_10	AFP	chr4	74313245	+	AAACTGACCACGCTGGAACG
131	sgAGMO_1	AGMO	chr7	15599808	+	GCATCATCCAGGCGACCTGG
132	sgAGMO_2	AGMO	chr7	15601431	+	GGGAAACTGAAACATCCTGC
133	sgAGMO_3	AGMO	chr7	15470651	+	GACAGACTGTCTCAGTGCTG
134	sgAGMO_4	AGMO	chr7	15458185	-	CCAATTTTGGATCCATACAG
135	sgAGMO_5	AGMO	chr7	15601449	-	GAAGAACCCAGAAGCCCAGC
136	sgAGMO_6	AGMO	chr7	15584456	+	CAAGTCCATGGAGAATCCCA
137	sgAGMO_7	AGMO	chr7	15584391	+	GCTTACCATGAGCCATACGA
138	sgAGMO_8	AGMO	chr7	15433771	+	GAATCAGTTCCAAAGGACCA
139	sgAGMO_9	AGMO	chr7	15427033	-	TCTCAGTGAAGAAATTCCAG
140	sgAGMO_10	AGMO	chr7	15405805	+	TAATGAAGCAAACCCTCAGA
141	sgAIFM3_1	AIFM3	chr22	21327594	+	AGTGGAGCTCAAGATCGAGG
142	sgAIFM3_2	AIFM3	chr22	21327757	-	GCAGCCTCCACGCAATCCTG
143	sgAIFM3_3	AIFM3	chr22	21331176	+	GAAGTTCTACATGCAGACGG
144	sgAIFM3_4	AIFM3	chr22	21333576	+	GAACATGTTGGCGCAGGAGG
145	sgAIFM3_5	AIFM3	chr22	21327645	+	GGAGCTGTCGGCCAGTGGGA
146	sgAIFM3_6	AIFM3	chr22	21327699	+	GGCCCGCCACTTCCACACGG
147	sgAIFM3_7	AIFM3	chr22	21328540	+	GGTGAAGATTGAGAAGGAGA
148	sgAIFM3_8	AIFM3	chr22	21328825	+	GCAGCGAAGGACCAAGGTGA
149	sgAIFM3_9	AIFM3	chr22	21329078	-	GCTTGGGACGGTCGTAGGGA
150	sgAIFM3_10	AIFM3	chr22	21330494	+	GGGATTGCAGGTGGTCACAG
151	sgALB_1	ALB	chr4	74272360	-	CTGCTGAAGATACTGAGCAA
152	sgALB_2	ALB	chr4	74280853	-	GCATTCATGAGGATCTGCAG
153	sgALB_3	ALB	chr4	74285332	+	TAAGGAGACCTGCTTTGCCG
154	sgALB_4	ALB	chr4	74270100	-	ATCTCGACGAAACACACCCC
155	sgALB_5	ALB	chr4	74276034	+	TGAACTTCGGGATGAAGGGA
156	sgALB_6	ALB	chr4	74283300	+	GTAGAGGTCTCAAGAAACCT
157	sgALB_7	ALB	chr4	74283890	-	GCATGGTCGCCTGTTCACCA
158	sgALB_8	ALB	chr4	74270031	+	ACGCCTTTGGCACAATGAAG
159	sgALB_9	ALB	chr4	74274344	+	GCAACTCTTCGTGAAACCTA
160	sgALB_10	ALB	chr4	74274363	-	GCAGTCAGCCATTTCACCAT
161	sgALKBH1_1	ALKBH1	chr14	78174332	-	CGAGATGGGGAAGATGGCAG
162	sgALKBH1_2	ALKBH1	chr14	78174206	-	AGGGGTCATCGACTTCTCGG
163	sgALKBH1_3	ALKBH1	chr14	78170765	-	GAGCAGAATGCATATAGAGC
164	sgALKBH1_4	ALKBH1	chr14	78146262	-	GTTTACTGGAGAAACTGCGT
165	sgALKBH1_5	ALKBH1	chr14	78174261	+	GAAGCGGAAAAGTTTCCGAA
166	sgALKBH1_6	ALKBH1	chr14	78174224	-	CGGGACCGCAGACCTGGAAG
167	sgALKBH1_7	ALKBH1	chr14	78174170	+	GGGCACCAGGACCCTTGCCA
168	sgALKBH1_8	ALKBH1	chr14	78161196	+	AGTGCCACTGGTAACCTGGG
169	sgALKBH1_9	ALKBH1	chr14	78142091	-	AGGATTTCCGAGCTGAAGCA
170	sgALKBH1_10	ALKBH1	chr14	78141993	+	ACTCACCTGAATGACAGCAA
171	sgALKBH2_1	ALKBH2	chr12	109530446	-	AGAGAGGCCCCAGGGAATGG
172	sgALKBH2_2	ALKBH2	chr12	109530475	-	ACAAAGAAAGCACAAGGAAG
173	sgALKBH2_3	ALKBH2	chr12	109530570	-	GGACAGATTCCTGGTGAAAG
174	sgALKBH2_4	ALKBH2	chr12	109530540	-	GGGCCTTTTGAGGAAGCAGG
175	sgALKBH2_5	ALKBH2	chr12	109530436	+	TGAGTGGCCTCCATTCCCTG
176	sgALKBH2_6	ALKBH2	chr12	109530363	-	AGTCCTGTTTGGCAAAGCTG
177	sgALKBH2_7	ALKBH2	chr12	109530462	-	AAGGAAGAGGCCCAGGAGAG
178	sgALKBH2_8	ALKBH2	chr12	109530421	-	ACTCAGCAGGCCCTAGCTGG
179	sgALKBH2_9	ALKBH2	chr12	109530500	-	GAAGAGCCAGCTGTGTTGGG
180	sgALKBH2_10	ALKBH2	chr12	109530378	+	GGACTGTGTAACTGCAGTCC
181	sgALKBH3_1	ALKBH3	chr11	43904641	-	GTGGAGATGGCTCTTAGCAG

182	sgALKBH3_2	ALKBH3	chr11	43911340	+	AAGATGTTCCCTGGAAACAG
183	sgALKBH3_3	ALKBH3	chr11	43911356	+	ACAGAGGACTGGCATCAGAG
184	sgALKBH3_4	ALKBH3	chr11	43923184	+	ATGATGAACCCTCACTAGGG
185	sgALKBH3_5	ALKBH3	chr11	43940614	+	GGAAAGAGTGAAGATACCCT
186	sgALKBH3_6	ALKBH3	chr11	43904252	-	AATGGCCTGGCTTTTAACAG
187	sgALKBH3_7	ALKBH3	chr11	43908172	+	GAAATCAGCCTGTCACCCAC
188	sgALKBH3_8	ALKBH3	chr11	43923119	-	AGAGTAAGGAGTTGAAGGTG
189	sgALKBH3_9	ALKBH3	chr11	43923195	-	ATGGGGCACCTCCCTAGTGA
190	sgALKBH3_10	ALKBH3	chr11	43923270	-	AAGAGAATACTCACTGGTGG
191	sgALKBH4_1	ALKBH4	chr7	102100144	+	GATCAGCATCACTCCTGGGA
192	sgALKBH4_2	ALKBH4	chr7	102100125	-	CGAGGACTTTGTGACCCGGG
193	sgALKBH4_3	ALKBH4	chr7	102100060	-	AGCTCTCCCAGTCTGGACGG
194	sgALKBH4_4	ALKBH4	chr7	102105203	-	GGACCTGTCTGATCTGCGAG
195	sgALKBH4_5	ALKBH4	chr7	102100084	-	GGCTCATGGACCGTGACCCC
196	sgALKBH4_6	ALKBH4	chr7	102105229	+	CCTTGCAACCGCATTCCCGA
197	sgALKBH4_7	ALKBH4	chr7	102100177	-	AGGAGTCTGACTTTGAGGGC
198	sgALKBH4_8	ALKBH4	chr7	102100071	+	GGGAGAGCTTCCAGGGGTCA
199	sgALKBH4_9	ALKBH4	chr7	102100218	+	TGTCGGAGCAGTAAATGAAA
200	sgALKBH4_10	ALKBH4	chr7	102100110	-	CCGGGAGGAAGAAGCCGAGT
201	sgALKBH5_1	ALKBH5	chr17	18110255	-	CATCAGGGTCTGCCTTGCGG
202	sgALKBH5_2	ALKBH5	chr17	18087611	+	GTCCCGGGACAACTATAAGG
203	sgALKBH5_3	ALKBH5	chr17	18087691	-	GTAAGGTTCGGCGGCAGCGG
204	sgALKBH5_4	ALKBH5	chr17	18087798	+	GAGGCGCGCAAGGTGAAGAG
205	sgALKBH5_5	ALKBH5	chr17	18087895	-	GTACAGGCCCTTCTCAGCGC
206	sgALKBH5_6	ALKBH5	chr17	18087908	+	CCTGTACAACGAGCACACGG
207	sgALKBH5_7	ALKBH5	chr17	18088088	+	GGAGCACCGCGTCATCCCCG
208	sgALKBH5_8	ALKBH5	chr17	18088201	-	AAAGAAGGACACGGACACGA
209	sgALKBH5_9	ALKBH5	chr17	18087863	+	GATCGAGGCCCGCATTGACG
210	sgALKBH5_10	ALKBH5	chr17	18087884	+	GGTGGTGTCCCGCGCTGAGA
211	sgALKBH6_1	ALKBH6	chr19	36501924	-	GCTTCCTCATCCCCGAGGGA
212	sgALKBH6_2	ALKBH6	chr19	36501536	-	ACTGTCAGCACCATCAGCCT
213	sgALKBH6_3	ALKBH6	chr19	36501499	-	TGGACTTCTACGAGCCGCGG
214	sgALKBH6_4	ALKBH6	chr19	36505127	-	GTGGGGCGGAAATGGCTGGG
215	sgALKBH6_5	ALKBH6	chr19	36505083	-	GAAATTGGAGGGGACGCTGG
216	sgALKBH6_6	ALKBH6	chr19	36504269	-	GGACGCCAGAGTCCCAGCCC
217	sgALKBH6_7	ALKBH6	chr19	36504248	-	GGAACCGTTCAGAGTGGAGC
218	sgALKBH6_8	ALKBH6	chr19	36503966	+	GGGACATAGTAGATTACAGG
219	sgALKBH6_9	ALKBH6	chr19	36502308	-	GGGAGAAAGTTACAGAACTG
220	sgALKBH6_10	ALKBH6	chr19	36501846	+	GGAGGCCTCCAAAGAGGCTG
221	sgALKBH7_1	ALKBH7	chr19	6374230	+	GAGAGACAGAGAAGTCGCGC
222	sgALKBH7_2	ALKBH7	chr19	6372900	-	GCAGGCGGCTCAGCACGGAA
223	sgALKBH7_3	ALKBH7	chr19	6373005	-	GATCGTATTCGTAGCGGCGG
224	sgALKBH7_4	ALKBH7	chr19	6374296	-	GGTCTGGCCGGGGCCAAAGG
225	sgALKBH7_5	ALKBH7	chr19	6374326	-	CAGGTCCAGCACGTGCACGG
226	sgALKBH7_6	ALKBH7	chr19	6374354	-	CGTGGGGCTTGATGTAGCCG
227	sgALKBH7_7	ALKBH7	chr19	6374492	-	CAGGAGAGACAGGCCGGCGA
228	sgALKBH7_8	ALKBH7	chr19	6374519	-	GTGCACCAGCCGCATAACGC
229	sgALKBH7_9	ALKBH7	chr19	6374529	+	GCTGGTGCACACCCAGGAGC
230	sgALKBH7_10	ALKBH7	chr19	6374589	-	GGAGGTACCTAAGGATGTAG
231	sgALKBH8_1	ALKBH8	chr11	107393133	-	GCAGCACAAGACATACCCCT
232	sgALKBH8_2	ALKBH8	chr11	107422521	+	AAGAAATACTCACCTTGCCC
233	sgALKBH8_3	ALKBH8	chr11	107403059	+	ATCACCAGCAAACTCCGACG
234	sgALKBH8_4	ALKBH8	chr11	107396268	-	GCATCTGAGAGTCTTAAAAG
235	sgALKBH8_5	ALKBH8	chr11	107424650	+	TCTACTACCATGAGTCCTGG
236	sgALKBH8_6	ALKBH8	chr11	107422536	-	AATCAGTATGAACCTGGGCA
237	sgALKBH8_7	ALKBH8	chr11	107403107	-	ATGGATTTTAAGCACCCAGA
238	sgALKBH8_8	ALKBH8	chr11	107393068	-	ATAGTGGCTGATATTGGATG
239	sgALKBH8_9	ALKBH8	chr11	107381668	-	TTGGCAGTACCAGTCCGCAG
240	sgALKBH8_10	ALKBH8	chr11	107427710	-	AGCCTGGTTGTTGCCAATGG
241	sgALOX12_1	ALOX12	chr17	6899465	-	GGAGAAGAGCCAGGCCCCGG
242	sgALOX12_2	ALOX12	chr17	6900319	+	GACATCCTGAGCCTGCCCGA

243	sgALOX12_3	ALOX12	chr17	6902307	-	GCAGTTCCAGGAGCTCAGGA
244	sgALOX12_4	ALOX12	chr17	6909218	-	TCAGCTGAGCTGCCGCCCGA
245	sgALOX12_5	ALOX12	chr17	6909837	+	AAAGGGATGACATAGTGAAG
246	sgALOX12_6	ALOX12	chr17	6909877	+	CTGGTGTCGGGAGATCACGG
247	sgALOX12_7	ALOX12	chr17	6899499	-	CGACCAGCCAAAGCTGCACG
248	sgALOX12_8	ALOX12	chr17	6901859	+	GTTCCAGAAGCATCGAGAGA
249	sgALOX12_9	ALOX12	chr17	6902042	-	GGGTAACCCTTCCTTCCAGG
250	sgALOX12_10	ALOX12	chr17	6902688	-	GCGTCTCAACAGCATGGGGT
251	sgALOX12B_1	ALOX12B	chr17	7990739	-	GGCCACCTACAAAGTCAGGG
252	sgALOX12B_2	ALOX12B	chr17	7990616	-	GAGAGACTTTGCAACTGGGG
253	sgALOX12B_3	ALOX12B	chr17	7989389	+	GAAGTGGTAGATACGGCCGT
254	sgALOX12B_4	ALOX12B	chr17	7983207	+	GAGGTACTGGTACCCAAAGA
255	sgALOX12B_5	ALOX12B	chr17	7982746	+	GTCCAAAGTGCAGCAGGCAG
256	sgALOX12B_6	ALOX12B	chr17	7980331	+	GCAGGTTCCTCAGCAAGGCC
257	sgALOX12B_7	ALOX12B	chr17	7979563	+	GCTCCACAAAGTCATTGGGG
258	sgALOX12B_8	ALOX12B	chr17	7989484	+	GGATGATGATGAGCTCACCC
259	sgALOX12B_9	ALOX12B	chr17	7989343	-	CGAGACCCTGGCACTCCGGG
260	sgALOX12B_10	ALOX12B	chr17	7984228	-	GTTACCGCCCTCCGGTGCGG
261	sgALOX15_1	ALOX15	chr17	4542371	-	GAAACACCGGGAAGAAGAGC
262	sgALOX15_2	ALOX15	chr17	4539214	+	GTAGGCAAGAAAAGGGGAGG
263	sgALOX15_3	ALOX15	chr17	4544820	-	GAAGCGACTGTGGCCCGCAC
264	sgALOX15_4	ALOX15	chr17	4542404	+	GAGGGTCCTCGCCCACAGTG
265	sgALOX15_5	ALOX15	chr17	4542384	-	AGGGCCTGTTCCAGAAACAC
266	sgALOX15_6	ALOX15	chr17	4539230	+	GAGGTGGTGGGGATCCTGTG
267	sgALOX15_7	ALOX15	chr17	4536509	+	CCGGTCGGCCAAGTCATCAG
268	sgALOX15_8	ALOX15	chr17	4535506	+	GGTGCAGGTGAAGATACACA
269	sgALOX15_9	ALOX15	chr17	4535473	+	TACCTGGCCCAGGTGCACAG
270	sgALOX15_10	ALOX15	chr17	4544907	+	GCGAGGCCCCAGTGGACACG
271	sgALOX15B_1	ALOX15B	chr17	7943245	-	GCTGTTGCTGGAGCACAGGG
272	sgALOX15B_2	ALOX15B	chr17	7942826	-	GCGGTGTCAGCTGGAACCAG
273	sgALOX15B_3	ALOX15B	chr17	7942895	+	GGGGACCCTGGTGCTGCAGG
274	sgALOX15B_4	ALOX15B	chr17	7946167	+	AAAGGATCTTCAACTTCCGG
275	sgALOX15B_5	ALOX15B	chr17	7948189	-	GAGACCATTCAGGAACTGGG
276	sgALOX15B_6	ALOX15B	chr17	7948279	-	GGTCCCAGGACCCAACACTG
277	sgALOX15B_7	ALOX15B	chr17	7948970	-	GCAGTGGGGCAGCTGACGCA
278	sgALOX15B_8	ALOX15B	chr17	7950933	-	GGAGCAGGTGAATATCACCA
279	sgALOX15B_9	ALOX15B	chr17	7951126	+	AAAGGCCTGGCAACATGCGA
280	sgALOX15B_10	ALOX15B	chr17	7945689	+	GGAAGGCTTACAACCCAGGT
281	sgALOX5_1	ALOX5	chr10	45878056	-	GGAACTCGATGTAGTCCCCG
282	sgALOX5_2	ALOX5	chr10	45938508	+	GGCACGTGCAGATGGTGCAG
283	sgALOX5_3	ALOX5	chr10	45938556	-	GGCCTCGGGAAAGCACAGGG
284	sgALOX5_4	ALOX5	chr10	45939207	+	GCTGTCGGAGTACCTGACCG
285	sgALOX5_5	ALOX5	chr10	45939227	-	GGAGGCGGTGAAGATCACCA
286	sgALOX5_6	ALOX5	chr10	45939260	-	TACCTGGCCGAAGTTGACCG
287	sgALOX5_7	ALOX5	chr10	45878097	+	GCGATGTCGAGGTTGTCCTG
288	sgALOX5_8	ALOX5	chr10	45920550	+	GCGGCAGCTCAGCTTGGAGC
289	sgALOX5_9	ALOX5	chr10	45924131	-	CCAGGAACTGGAGTGTGCAG
290	sgALOX5_10	ALOX5	chr10	45924202	-	GCAGCCTACCTGGATGGCAA
291	sgALOXE3_1	ALOXE3	chr17	8013807	-	GGACTACTGGATCCTGGCGG
292	sgALOXE3_2	ALOXE3	chr17	8013272	-	GCAGGTGAACACCATCGCGA
293	sgALOXE3_3	ALOXE3	chr17	8013258	+	GAGCAGCGTGGCCCTCGCGA
294	sgALOXE3_4	ALOXE3	chr17	8020115	-	GATTGAAGGCTACTGCACCG
295	sgALOXE3_5	ALOXE3	chr17	8014751	+	TGGGCAAGCTAGAGATGCAG
296	sgALOXE3_6	ALOXE3	chr17	8013454	-	GCTGTGCGAGGCCTTCGCCA
297	sgALOXE3_7	ALOXE3	chr17	8013440	+	GCGCAGCGTGGCCATGGCGA
298	sgALOXE3_8	ALOXE3	chr17	8012632	+	GATGAGGCCTTGCCTCCCGA
299	sgALOXE3_9	ALOXE3	chr17	8012541	+	TGGGGATAGCCAGGACGCCG
300	sgALOXE3_10	ALOXE3	chr17	8006692	+	GCTGATGTTCACTTCAGGGA
301	sgAMBP_1	AMBP	chr9	116823193	+	AGGGCTGCCTACCATCACCA
302	sgAMBP_2	AMBP	chr9	116840459	+	GCAGCAAGAGCAGGGCCCCG
303	sgAMBP_3	AMBP	chr9	116838973	+	CCAGGGGCAGGTGGAACCGA

304	sgAMBP_4	AMBP	chr9	116838924	-	GAGCACGCTGGTGCTGGGAG
305	sgAMBP_5	AMBP	chr9	116836317	+	GTAGAGCTTGGCAGTAATGG
306	sgAMBP_6	AMBP	chr9	116825008	-	GGCTGTGCTACCCCAAGAAG
307	sgAMBP_7	AMBP	chr9	116823772	-	TGTGAGACTTTCCAGTACGG
308	sgAMBP_8	AMBP	chr9	116823241	-	GAACAAGTTCTACTCAGAGA
309	sgAMBP_9	AMBP	chr9	116823222	-	AAGGAGTGCAGAGAGTACTG
310	sgAMBP_10	AMBP	chr9	116840444	-	GCTGCTGAGCGCCTGCCTGG
311	sgAMDHD1_1	AMDHD1	chr12	96337197	-	GCTGCGCGTTCTCCAGCAGG
312	sgAMDHD1_2	AMDHD1	chr12	96350481	+	ATGGAAATTCACCAGGCCGG
313	sgAMDHD1_3	AMDHD1	chr12	96350501	+	AGGAGGGATCCACTTTACCG
314	sgAMDHD1_4	AMDHD1	chr12	96350561	-	GGAGCCGTTGCTGCAAGGAG
315	sgAMDHD1_5	AMDHD1	chr12	96350591	+	GAGGGCTGGCACCACGCTGG
316	sgAMDHD1_6	AMDHD1	chr12	96354245	+	CAGAAATGGGGAAATACACG
317	sgAMDHD1_7	AMDHD1	chr12	96337219	+	GTGGTGCTGGTGTGCGCCCG
318	sgAMDHD1_8	AMDHD1	chr12	96337287	+	GGAAGGCGCCAGCCTGGTGG
319	sgAMDHD1_9	AMDHD1	chr12	96350525	+	GCGCACGCGCCAAGCCACAG
320	sgAMDHD1_10	AMDHD1	chr12	96350665	+	GCGTGATTGAGCGCGCCCGG
321	sgAOX1_1	AOX1	chr2	201468050	+	GGATTGCCAGAATTTGAGGA
322	sgAOX1_2	AOX1	chr2	201469558	-	GGGGTTCTACATACCCACAG
323	sgAOX1_3	AOX1	chr2	201460064	+	GATACAACCCCATCACCAAG
324	sgAOX1_4	AOX1	chr2	201462134	-	GATGGGAATCAGACAGGCAT
325	sgAOX1_5	AOX1	chr2	201477477	-	GGAATTCTTGGCACAGATGG
326	sgAOX1_6	AOX1	chr2	201501746	-	GTTTGCGGCAAATGCAGTGA
327	sgAOX1_7	AOX1	chr2	201515855	+	GAAGAAGAAAGGACTGGCCA
328	sgAOX1_8	AOX1	chr2	201515903	-	GACCAGCAGCACGTGAGCCA
329	sgAOX1_9	AOX1	chr2	201521598	+	TCTGTGCTGGTCACTCACGG
330	sgAOX1_10	AOX1	chr2	201533383	+	GAACTGAATTATTCTCCCCA
331	sgASPH_1	ASPH	chr8	62596719	-	ATGGAGGACACAAGAATGGG
332	sgASPH_2	ASPH	chr8	62596735	-	TAAACAGAGACAAAGCATGG
333	sgASPH_3	ASPH	chr8	62596683	-	CAGGAACTTCATTCTTCACG
334	sgASPH_4	ASPH	chr8	62593571	-	CTAGGAATCTATGATGCTGA
335	sgASPH_5	ASPH	chr8	62593551	-	TGGTGATGGAGATTTTGATG
336	sgASPH_6	ASPH	chr8	62596702	-	GGGAGGAAAGGCGGACTCTC
337	sgASPH_7	ASPH	chr8	62566151	-	TGAGCCACACACTGAGCCCG
338	sgASPH_8	ASPH	chr8	62546244	-	GAACAGCAGGAAGTACCACC
339	sgASPH_9	ASPH	chr8	62563611	-	GAAATGGTACACGCAGAACA
340	sgASPH_10	ASPH	chr8	62563659	+	GCTTCATCTTCGATATTCTG
341	sgASPHD2_1	ASPHD2	chr22	26829791	-	GATAACAGTACCACACGAAG
342	sgASPHD2_2	ASPHD2	chr22	26829659	+	GATGTCGCTCGAGTGGCTGG
343	sgASPHD2_3	ASPHD2	chr22	26829724	-	CCGCACGGACTGGATGCCGG
344	sgASPHD2_4	ASPHD2	chr22	26829757	-	GGCCACAGTGATGACAGCGG
345	sgASPHD2_5	ASPHD2	chr22	26829921	+	GTGCGCTGCACCCACAACGA
346	sgASPHD2_6	ASPHD2	chr22	26829953	+	GAAGCTGTACCACAACCTGC
347	sgASPHD2_7	ASPHD2	chr22	26830108	-	GTCCCGGGAGAAATAGGGCG
348	sgASPHD2_8	ASPHD2	chr22	26830178	-	AGAGGGTCTCAAACTCACAC
349	sgASPHD2_9	ASPHD2	chr22	26838498	-	GGAAAGAGTCATCAAAGAGA
350	sgASPHD2_10	ASPHD2	chr22	26829838	-	GAGGGAGTTGACGGAGACGT
351	sgBACH1_1	BACH1	chr21	30698546	-	GCATTCTTGCTGGTCTGCAG
352	sgBACH1_2	BACH1	chr21	30698634	+	GGATCTAGAAACTGATGAAG
353	sgBACH1_3	BACH1	chr21	30699303	+	GAGTAGTGTGGAGCGAGAAG
354	sgBACH1_4	BACH1	chr21	30698692	-	TACCTGCGGAGTTTACACTG
355	sgBACH1_5	BACH1	chr21	30698804	-	TTTGGGGCATAAAGAAGGCA
356	sgBACH1_6	BACH1	chr21	30699384	+	AATGCAGTTATCACCTGCTG
357	sgBACH1_7	BACH1	chr21	30699454	-	CTAATCCTGATACCTAACCA
358	sgBACH1_8	BACH1	chr21	30699652	+	GACTCTGAGACGGACACCGA
359	sgBACH1_9	BACH1	chr21	30693682	-	ACAGCACATCTTTCTTCCGC
360	sgBACH1_10	BACH1	chr21	30693703	+	CGATGTCACCATCTTTGTGG
361	sgBBOX1_1	BBOX1	chr11	27141327	+	GGACTTTACAGACATTGGAG
362	sgBBOX1_2	BBOX1	chr11	27077028	+	GCAGATCCTCTGGTATGATG
363	sgBBOX1_3	BBOX1	chr11	27077143	+	GAAGCTCTTGATGTGAACAT
364	sgBBOX1_4	BBOX1	chr11	27114778	+	ATGATGAACACGCATACAAG

365	sgBBOX1_5	BBOX1	chr11	27141213	+	CATAAAGCAAACAGTCACAG
366	sgBBOX1_6	BBOX1	chr11	27077121	+	AAAAGCACGGAAACTTCTAG
367	sgBBOX1_7	BBOX1	chr11	27141295	-	GACAAAATCTGGAATGCCTG
368	sgBBOX1_8	BBOX1	chr11	27141317	-	TGTAAAGTCCACAAAGGTAG
369	sgBBOX1_9	BBOX1	chr11	27114722	-	GGAGCTCTGAGCCCCAGTAT
370	sgBBOX1_10	BBOX1	chr11	27137089	-	TTACCCCAGGTGGATGATGG
371	sgBCMO1_1	BCMO1	chr16	81295767	-	GTTGTCGGTGAAATCGGGGA
372	sgBCMO1_2	BCMO1	chr16	81295867	+	GACTCTGGAAACCCTGGAGA
373	sgBCMO1_3	BCMO1	chr16	81301572	-	GGGGAGAGCAGGGAGCGGGA
374	sgBCMO1_4	BCMO1	chr16	81303831	-	GACGTGATGGAAGACCACCA
375	sgBCMO1_5	BCMO1	chr16	81303987	-	GGGCACGGCAAACCTCCTGA
376	sgBCMO1_6	BCMO1	chr16	81314491	+	AGTGGCATCTACAACAGCCA
377	sgBCMO1_7	BCMO1	chr16	81319218	+	GTGGAGTCCAATCCCAACCA
378	sgBCMO1_8	BCMO1	chr16	81303811	+	CAAGTTTTACACAGACGCCA
379	sgBCMO1_9	BCMO1	chr16	81303848	+	GTCAACGCCTACGAAGAGGA
380	sgBCMO1_10	BCMO1	chr16	81303919	-	GGTTGGCCAGGTAGAAGAGC
381	sgBCO2_1	BCO2	chr11	112072877	+	ACAGAATCTCAGGAAGGCTG
382	sgBCO2_2	BCO2	chr11	112085515	+	GAGGACCTAGAAAAGGAAGG
383	sgBCO2_3	BCO2	chr11	112086997	+	GGAACCAATGAAGAAGATGG
384	sgBCO2_4	BCO2	chr11	112071444	+	GGGAACCCCAGTGTAATACG
385	sgBCO2_5	BCO2	chr11	112084558	+	AGTGCTGTGAAACAGGCTGA
386	sgBCO2_6	BCO2	chr11	112064230	+	TCACCAGTTCAGAATGGCAA
387	sgBCO2_7	BCO2	chr11	112070471	+	GGAGACAATCCATGGAGTCC
388	sgBCO2_8	BCO2	chr11	112084476	-	AAGGCAAAACAAACCTTCGA
389	sgBCO2_9	BCO2	chr11	112070441	+	TCGGGTTCCTCCAGAGAAGG
390	sgBCO2_10	BCO2	chr11	112071457	+	TAATACGCGGTTTCATGTGG
391	sgBOLA1_1	BOLA1	chr1	149871760	+	GAGCTTCGCAACGAGAGCGG
392	sgBOLA1_2	BOLA1	chr1	149871801	+	CAGTGAGACTCACTTCCGCG
393	sgBOLA1_3	BOLA1	chr1	149871819	-	GAGAGCTCACCACAGCCACG
394	sgBOLA1_4	BOLA1	chr1	149871903	+	GGGAGGTCCGGTCCATGCGC
395	sgBOLA1_5	BOLA1	chr1	149871947	-	AGAGTTCTCTCTCCACTGGG
396	sgBOLA1_6	BOLA1	chr1	149871994	+	GGTGGGAACAAGAAAACTCT
397	sgBOLA1_7	BOLA1	chr1	149871660	-	TGCCCTGGCACAAACAAACG
398	sgBOLA1_8	BOLA1	chr1	149871717	+	CATTCGCACGAAGTTGGAGG
399	sgBOLA1_9	BOLA1	chr1	149871614	-	GACCAGCCGCCCACTCAGCA
400	sgBOLA1_10	BOLA1	chr1	149871687	+	ATCCGGGGCCATCGGTCCGG
401	sgBOLA2_1	BOLA2	chr16	29465727	+	CAAGCAGCCGGGCTTTCCAG
402	sgBOLA2_2	BOLA2	chr16	29465688	-	GACGTACGCCTTAGTAAGGG
403	sgBOLA2_3	BOLA2	chr16	29465532	+	CCCGCTGCAGCTTCTCGCGG
404	sgBOLA2_4	BOLA2	chr16	29465334	+	TACCTGTGTCTCTGAAGCAG
405	sgBOLA2_5	BOLA2	chr16	29465746	-	GCGCGAAAAGCCTGGACCGC
406	sgBOLA2_6	BOLA2	chr16	29465664	-	AGTGAGTTTTCCAGCGGAAG
407	sgBOLA2_7	BOLA2	chr16	29465604	+	GCAGGGACACAACCCCAGCT
408	sgBOLA2_8	BOLA2	chr16	29465637	-	TGTAAGGCAGCAAGGTAGCG
409	sgBOLA2_9	BOLA2	chr16	29465587	+	GCTGGAACGGCAGCCCAGCA
410	sgBOLA2_10	BOLA2	chr16	29465364	-	GTGGTGTCGGCCAAGTTCGA
411	sgBOLA3_1	BOLA3	chr2	74372397	+	TCTGAGTGGCAAACATCCGA
412	sgBOLA3_2	BOLA3	chr2	74374950	-	TCTCCTCCGCGGGATCCGCG
413	sgBOLA3_3	BOLA3	chr2	74372416	-	GGCAGCTTCCACTTCACCAT
414	sgBOLA3_4	BOLA3	chr2	74369401	-	GCAGCACCAGATGGTTAATC
415	sgBOLA3_5	BOLA3	chr2	74362712	-	CCCAAACGCTGACCACGCCC
416	sgBOLA3_6	BOLA3	chr2	74362697	+	GCATCTATGCAGCCAGGGCG
417	sgBOLA3_7	BOLA3	chr2	74374986	-	GGGCATGGCTGCATGGAGCC
418	sgBOLA3_8	BOLA3	chr2	74362662	+	ATGTCAGTGAAGTTCATCCA
419	sgBOLA3_9	BOLA3	chr2	74362750	-	TCAAAGAGATGCATGGATTG
420	sgBOLA3_10	BOLA3	chr2	74362682	-	GATGCTGCTGCTTAAGACCT
421	sgBRIP1_1	BRIP1	chr17	59886095	+	GCACCTAGAACAGTGGCCAG
422	sgBRIP1_2	BRIP1	chr17	59885954	-	AGATTACTAGAGAGCTCCGG
423	sgBRIP1_3	BRIP1	chr17	59870961	+	CAAAATGGGAAAAGTAGCAG
424	sgBRIP1_4	BRIP1	chr17	59853793	-	GTGTGCCAGACTGTGAGCCA
425	sgBRIP1_5	BRIP1	chr17	59821864	-	GTCATTGTAGAACCACAGGG

426	sgBRIP1_6	BRIP1	chr17	59763211	+	GAGATAATGCTACTTGGTAG
427	sgBRIP1_7	BRIP1	chr17	59885938	-	CCGGAGGACGGCATATTCAG
428	sgBRIP1_8	BRIP1	chr17	59885872	+	AGTTACCGACTACCTCAGGA
429	sgBRIP1_9	BRIP1	chr17	59876606	-	AGATGAAGCTCATAACATCG
430	sgBRIP1_10	BRIP1	chr17	59858324	-	CGATTCAACAGACTTACTCC
431	sgC16orf52_1	C16orf52	chr16	22019696	-	GGTGTTGATCCAGTCCGGGT
432	sgC16orf52_2	C16orf52	chr16	22019666	-	GGCGATGGCGAAGATATCGG
433	sgC16orf52_3	C16orf52	chr16	22019681	-	CGGGTTGGCGATGCTGGCGA
434	sgC16orf52_4	C16orf52	chr16	22019639	-	AAAGAGACATCCTGAGATGA
435	sgC16orf52_5	C16orf52	chr16	22086831	+	CGACAGTGTCAAACAATCCA
436	sgC16orf52_6	C16orf52	chr16	22086889	-	CAGTGTGGTGACCCACTCCG
437	sgC16orf52_7	C16orf52	chr16	22019626	+	GATAAACTGACCATCATCTC
438	sgC16orf52_8	C16orf52	chr16	22019706	+	GATCAACACCGGGGAGTCTG
439	sgC16orf52_9	C16orf52	chr16	22086971	-	TTGTAGCTTCTCTTCGCCAG
440	sgC16orf52_10	C16orf52	chr16	22086800	+	CTCCCCAGGAGCACTCACTG
441	sgCACNA1C_1	CACNA1C	chr12	2622013	+	GGGGAGATTTCCAGAAGCTG
442	sgCACNA1C_2	CACNA1C	chr12	2760871	+	GAAGCTGCTGAGCCGTGGGG
443	sgCACNA1C_3	CACNA1C	chr12	2775888	-	GTGGCGGCTGAATCCGCCGG
444	sgCACNA1C_4	CACNA1C	chr12	2783800	-	GGAGCACTCACCACCTGCAG
445	sgCACNA1C_5	CACNA1C	chr12	2791788	+	GAAGATGAACCATGACACGG
446	sgCACNA1C_6	CACNA1C	chr12	2224561	+	CCACAGTCAGCTCCACGCAG
447	sgCACNA1C_7	CACNA1C	chr12	2224678	-	GCTGATGCAGGCCCTCCGGA
448	sgCACNA1C_8	CACNA1C	chr12	2558237	-	GTAGGTTCCAGCCGTTGCGG
449	sgCACNA1C_9	CACNA1C	chr12	2566770	+	GATGGGGCAAACGCTCTCGG
450	sgCACNA1C_10	CACNA1C	chr12	2566804	+	GATTTGATGTGAAGGCGCTG
451	sgCAT_1	CAT	chr11	34472622	-	AGTGGAGAACCGAACTGCGA
452	sgCAT_2	CAT	chr11	34475425	+	GCTGGTTAATGCAAATGGGG
453	sgCAT_3	CAT	chr11	34482833	-	GATAATTGGGTCCCAGGCGA
454	sgCAT_4	CAT	chr11	34460583	+	CCAGCGACCAGATGCAGCAC
455	sgCAT_5	CAT	chr11	34460596	+	GCAGCACTGGAAGGAGCAGC
456	sgCAT_6	CAT	chr11	34470745	+	GATGTCCTGACCACTGGAGC
457	sgCAT_7	CAT	chr11	34470820	-	ACCACATCCTGAACAAGAAG
458	sgCAT_8	CAT	chr11	34473644	+	GACACAGTTCGGGACCCTCG
459	sgCAT_9	CAT	chr11	34473662	-	AATTTCACTGCAAACCCACG
460	sgCAT_10	CAT	chr11	34474666	+	AAATCCTCAGACACATCTGA
461	sgCBS_1	CBS	chr21	44483110	-	GGAGCAGACAACCTACGAGG
462	sgCBS_2	CBS	chr21	44492234	+	CCGAGTGTGGCCCTGAGCGG
463	sgCBS_3	CBS	chr21	44492161	+	GCATCGGGCCGGATCCACAG
464	sgCBS_4	CBS	chr21	44492103	+	GTGGTGATGTGGGGACTCGG
465	sgCBS_5	CBS	chr21	44485341	+	GGTGGTGTCGTAGTGAGCCA
466	sgCBS_6	CBS	chr21	44485323	+	CTGCAGGATCTCATCAGCGG
467	sgCBS_7	CBS	chr21	44484084	-	AGGGAAGCTGGACATGCTGG
468	sgCBS_8	CBS	chr21	44480636	-	TGGCAGTGCTGGCAGCACGG
469	sgCBS_9	CBS	chr21	44480599	-	GCCGCGCAGGAGCTGCAGGA
470	sgCBS_10	CBS	chr21	44480573	+	GCAGAATGACCACGCAGCGC
471	sgCD163_1	CD163	chr12	7654039	-	GAGGAACAGACAAGGAGCTG
472	sgCD163_2	CD163	chr12	7647967	-	CTGGAGCTAAGACTTAGAGG
473	sgCD163_3	CD163	chr12	7653984	-	AGTGGAAGTGAAAGTCCAGG
474	sgCD163_4	CD163	chr12	7653853	+	AATGCGTCCAGAACCTGCAC
475	sgCD163_5	CD163	chr12	7651751	-	GAAATGAGGCTGACGCGTGG
476	sgCD163_6	CD163	chr12	7651516	-	GGAACTGCAAACATCAAGGA
477	sgCD163_7	CD163	chr12	7647911	-	GATTCAGAGACTGTTAGGGA
478	sgCD163_8	CD163	chr12	7647892	-	AAGGTGTGTGACAGAGGCTG
479	sgCD163_9	CD163	chr12	7647792	-	AAATCCAGGCAACAAACACA
480	sgCD163_10	CD163	chr12	7647729	-	GGGACTGCAAGAACTGGCAA
481	sgCDK5RAP1_1	CDK5RAP1	chr20	31982821	-	GCGGACCAGTAACCTCCAAG
482	sgCDK5RAP1_2	CDK5RAP1	chr20	31967269	+	CATCAGGAAAATCCTTGGGG
483	sgCDK5RAP1_3	CDK5RAP1	chr20	31961926	-	GTGTGTTGGAGGCCATGCGG
484	sgCDK5RAP1_4	CDK5RAP1	chr20	31954680	+	CAACTGGGTACAGCCCACAG
485	sgCDK5RAP1_5	CDK5RAP1	chr20	31982806	+	GAAATGCATGTACCTCTTGG
486	sgCDK5RAP1_6	CDK5RAP1	chr20	31979988	-	TTAAAGCCTTGAAGACAAGG

487	sgCDK5RAP1_7	CDK5RAP1	chr20	31975228	+	TCAGCAACAGCCAGCAGCCG
488	sgCDK5RAP1_8	CDK5RAP1	chr20	31967440	-	TAGTTTTCGGGACAATTCGG
489	sgCDK5RAP1_9	CDK5RAP1	chr20	31967400	-	GTGCCTACCAATCTCAGTCG
490	sgCDK5RAP1_10	CDK5RAP1	chr20	31954696	-	AAAGCCAATCAGACCTCTGT
491	sgCDKAL1_1	CDKAL1	chr6	20739762	+	GGAGAACAAGAAAATCGTAC
492	sgCDKAL1_2	CDKAL1	chr6	20758840	+	TCGTGTGGTAGAAGTTGTGG
493	sgCDKAL1_3	CDKAL1	chr6	20955666	+	ATGGTTGACCAGTGAAGACA
494	sgCDKAL1_4	CDKAL1	chr6	21000565	+	GGCTGACTTCAAAAGAGTAG
495	sgCDKAL1_5	CDKAL1	chr6	21065438	+	GGAACAAGTTCCAGCACAAG
496	sgCDKAL1_6	CDKAL1	chr6	21065451	-	AAAGATCTTACCACTTGTGC
497	sgCDKAL1_7	CDKAL1	chr6	20546679	+	ATGTTGTCCCGAAGGTACGA
498	sgCDKAL1_8	CDKAL1	chr6	20846328	+	TGCAAAACTAAACACGCCAG
499	sgCDKAL1_9	CDKAL1	chr6	21000484	-	GCAGAAAAGCGTAGACTCTG
500	sgCDKAL1_10	CDKAL1	chr6	21201460	+	CATCAGCAAACCGCTAGCAA
501	sgCH25H_1	CH25H	chr10	90966867	+	CAGGATATCCAGGACCACGA
502	sgCH25H_2	CH25H	chr10	90966809	+	CGGATGGCGAGAAGTCAGGG
503	sgCH25H_3	CH25H	chr10	90966995	+	ACAGCTGCCCGGAGCTGCAA
504	sgCH25H_4	CH25H	chr10	90966959	-	GGACCACCTGAGGAGCTGGG
505	sgCH25H_5	CH25H	chr10	90966918	+	GGAGAAGATGACCGGGAAGA
506	sgCH25H_6	CH25H	chr10	90966749	+	GGAACACAAACATCACATGC
507	sgCH25H_7	CH25H	chr10	90966650	+	GTAGCAGGCAGAACAGGATG
508	sgCH25H_8	CH25H	chr10	90966569	+	GGTGCACCTTGTGGAAGGTG
509	sgCH25H_9	CH25H	chr10	90966545	-	GAACTCGTCCTCGTTCGCGC
510	sgCH25H_10	CH25H	chr10	90966432	+	GACCACGTGGAAGGTCAGGG
511	sgCIAPIN1_1	CIAPIN1	chr16	57468082	+	GAACAGACTGTACTTCCTCA
512	sgCIAPIN1_2	CIAPIN1	chr16	57466461	-	GGACTCAGTGAAACCTGCTG
513	sgCIAPIN1_3	CIAPIN1	chr16	57474699	+	TGATGTTTTCCACAGACACG
514	sgCIAPIN1_4	CIAPIN1	chr16	57466445	+	GCAGCAGCAGGGTCCACAGC
515	sgCIAPIN1_5	CIAPIN1	chr16	57465113	-	AGCCAGATCCAGCTTCCCTG
516	sgCIAPIN1_6	CIAPIN1	chr16	57465065	-	AGAGGAAGGCCTGTAAGAAC
517	sgCIAPIN1_7	CIAPIN1	chr16	57473096	-	GAGCCAGTAGAGACAGCTGT
518	sgCIAPIN1_8	CIAPIN1	chr16	57468119	-	GTCTTGAATTCTAGCTGCAG
519	sgCIAPIN1_9	CIAPIN1	chr16	57468000	+	GAAGAACCCACTTCAAAGTT
520	sgCIAPIN1_10	CIAPIN1	chr16	57470570	-	TGGTCTTGTGGAAGTGAAAG
521	sgCISD1_1	CISD1	chr10	60036888	+	GCAGCAGTTACCATTGCTGC
522	sgCISD1_2	CISD1	chr10	60029043	+	ACTTCCAGTTCCAGCGTACG
523	sgCISD1_3	CISD1	chr10	60029056	-	CCCCACTTCACCTCGTACGC
524	sgCISD1_4	CISD1	chr10	60037051	+	CTGTGTACTGCCGTTGTTGG
525	sgCISD1_5	CISD1	chr10	60047357	+	GAAGAGACTGGAGACAATGT
526	sgCISD1_6	CISD1	chr10	60047317	+	CTAGTTCCCATTCTGTGATG
527	sgCISD1_7	CISD1	chr10	60047327	-	GTGTGAGCCCCATCACAGAA
528	sgCISD1_8	CISD1	chr10	60036901	-	TGCAGCTGTCCCAGCAGCAA
529	sgCISD1_9	CISD1	chr10	60036986	-	GGTTGTCTTTCTGGATGTGA
530	sgCISD1_10	CISD1	chr10	60037013	+	AGTACATGCTTTTGACATGG
531	sgCISD2_1	CISD2	chr4	103806399	-	AGTGCGAGTACACCAAGGAA
532	sgCISD2_2	CISD2	chr4	103790229	+	GCCGCTGGCCAGGATGGTGC
533	sgCISD2_3	CISD2	chr4	103790253	+	GAGCGTGGCCCGTATCGTGA
534	sgCISD2_4	CISD2	chr4	103790265	-	GGAGCTGCACCTTCACGATA
535	sgCISD2_5	CISD2	chr4	103790286	-	GGAGCCGCTTCAGATATGCA
536	sgCISD2_6	CISD2	chr4	103806449	+	CCCGAAGAAGAAACAACAGA
537	sgCISD2_7	CISD2	chr4	103806431	-	GGAGGAATGGACGAACTGCA
538	sgCISD2_8	CISD2	chr4	103806553	+	AAGCAGCTTATTGTAGGTGT
539	sgCISD2_9	CISD2	chr4	103808492	+	TTTTAGTTTCCTGCCTGCGA
540	sgCISD2_10	CISD2	chr4	103808504	-	TTATGTGAACCATCGCAGGC
541	sgCISD3_1	CISD3	chr17	36887025	-	CAGGAGGAGATGTCCCGCCG
542	sgCISD3_2	CISD3	chr17	36887643	+	TGGCAGGGAAAACCTACAGG
543	sgCISD3_3	CISD3	chr17	36887008	+	TGCAGGACCTGAACCCGCGG
544	sgCISD3_4	CISD3	chr17	36887658	-	GCCACACACACACCACCTGT
545	sgCISD3_5	CISD3	chr17	36887590	+	AACCCCAGCCAGGTCCGTGG
546	sgCISD3_6	CISD3	chr17	36887580	+	GGTTCCCTAGAACCCCAGCC
547	sgCISD3_7	CISD3	chr17	36887626	-	CCACCAGCTCCACCTTGATG

548	sgCISD3_8	CISD3	chr17	36887671	+	GTGTGGCCGCAGCAAGAAGC
549	sgCISD3_9	CISD3	chr17	36887559	+	TTCCCTGGCCACAGGCCCAG
550	sgCISD3_10	CISD3	chr17	36887601	-	CTTCAGGGCCACCACGGACC
551	sgCOPA_1	COPA	chr1	160283905	-	GTCCTTCAGAATCAAAGGCA
552	sgCOPA_2	COPA	chr1	160278904	+	GGAGTCAGCATCTTTAGGGA
553	sgCOPA_3	COPA	chr1	160268941	+	GGATCAATGGTGAGTACCCG
554	sgCOPA_4	COPA	chr1	160265859	-	GGAGACATTTGACCCAGAGA
555	sgCOPA_5	COPA	chr1	160263219	-	GCAGCTGGTGGGGCTGAAGA
556	sgCOPA_6	COPA	chr1	160261954	-	GAAGAATGGTGTACCAGCTG
557	sgCOPA_7	COPA	chr1	160261831	+	CAAGAAGTGGCACACTGAGA
558	sgCOPA_8	COPA	chr1	160303425	+	TCGGATGGTCTGATCATCGG
559	sgCOPA_9	COPA	chr1	160295353	-	ATTTGGAACTACAGATGCAG
560	sgCOPA_10	COPA	chr1	160283885	-	TGGGAGGTTGATACCTGCCG
561	sgCOQ7_1	COQ7	chr16	19087047	+	GGGGACCGCCTTGCTCGGGA
562	sgCOQ7_2	COQ7	chr16	19087131	+	GATCAGGACGCTGATGGAGG
563	sgCOQ7_3	COQ7	chr16	19083393	+	GTCCTGGGTCGGACCAGCGT
564	sgCOQ7_4	COQ7	chr16	19085335	+	GAACGTGCTGGGGTTTGCAC
565	sgCOQ7_5	COQ7	chr16	19087059	+	GCTCGGGAAGGAAGGTGCCA
566	sgCOQ7_6	COQ7	chr16	19083371	-	CCATCTGCCCGGCATAGATG
567	sgCOQ7_7	COQ7	chr16	19087071	+	AGGTGCCATGGCCTGCACCG
568	sgCOQ7_8	COQ7	chr16	19083320	+	TGTGGATCGAATAATCCGGG
569	sgCOQ7_9	COQ7	chr16	19085323	+	GATGCCCTTGTGGAACGTGC
570	sgCOQ7_10	COQ7	chr16	19088652	+	GAGCTTGAGCACCATGACAT
571	sgCOX15_1	COX15	chr10	101489383	+	GCTCAGCAGCCTTTGAGGGA
572	sgCOX15_2	COX15	chr10	101486844	-	GGAGTACTCACACCGAATGT
573	sgCOX15_3	COX15	chr10	101483795	+	AGGCGGTACTGACTGACCCG
574	sgCOX15_4	COX15	chr10	101478183	-	GGGAGAATCCTGGATCCCGG
575	sgCOX15_5	COX15	chr10	101478109	+	CCGGTGATCAAACTGCACCA
576	sgCOX15_6	COX15	chr10	101489442	+	GAGATGGTGCTGTATTGCCC
577	sgCOX15_7	COX15	chr10	101486774	-	AGAAAGGGCTGGCTCAGCCG
578	sgCOX15_8	COX15	chr10	101483766	+	GAACCAGGGCTGATCCCAGG
579	sgCOX15_9	COX15	chr10	101483752	+	GCTGGCACAATAAAGAACCA
580	sgCOX15_10	COX15	chr10	101483702	+	ACAACTCTTACCTTGTGCGG
581	sgCOX5A_1	COX5A	chr15	75230316	+	TTGCGGCCACAGCGCAGCGG
582	sgCOX5A_2	COX5A	chr15	75230244	+	CAGGGGCCTTACCCACGGCG
583	sgCOX5A_3	COX5A	chr15	75230284	+	GTGCAGGAGGCCTCGAGGGT
584	sgCOX5A_4	COX5A	chr15	75219188	+	GGGCTCTGGAACCATATCAT
585	sgCOX5A_5	COX5A	chr15	75219118	-	TAGTACAGTTCGTATCCTAG
586	sgCOX5A_6	COX5A	chr15	75230299	+	AGGGTCGGCCCGGGTGGTTG
587	sgCOX5A_7	COX5A	chr15	75221509	-	ATGAGGAGTTTGATGCTCGC
588	sgCOX5A_8	COX5A	chr15	75219100	+	CACTGACCTTAACAACCTCT
589	sgCOX5A_9	COX5A	chr15	75221452	+	ATTACCTTTACGCAATTCCC
590	sgCOX5A_10	COX5A	chr15	75221472	-	CAAGCCAGATATAGATGCCT
591	sgCYB561_1	CYB561	chr17	61514795	+	GTACAGCCCGAGCCACGCGC
592	sgCYB561_2	CYB561	chr17	61512516	+	GCACCAAAGAAGATGTGCTG
593	sgCYB561_3	CYB561	chr17	61512484	+	GGGCGGTGCCCACGGAAAGG
594	sgCYB561_4	CYB561	chr17	61513455	-	GCACCACCAAGGTCCTGCAC
595	sgCYB561_5	CYB561	chr17	61513054	+	CAAAGTACAGGACAAAGACA
596	sgCYB561_6	CYB561	chr17	61512553	+	GCAGGGAGAATGAAGCTCCG
597	sgCYB561_7	CYB561	chr17	61514858	+	CACGTAGTAAGGCAGTGCTG
598	sgCYB561_8	CYB561	chr17	61514827	-	GCTGCTGGGCCTGACCTTGG
599	sgCYB561_9	CYB561	chr17	61514779	-	GTACCGAGGCGGCATTGCCT
600	sgCYB561_10	CYB561	chr17	61514901	-	GTTTGCCTCAGCATGGAGGG
601	sgCYB561A3_1	CYB561A3	chr11	61120584	+	GGAGGAAGACAGCAAAGCCC
602	sgCYB561A3_2	CYB561A3	chr11	61124119	+	AGAGGATGCACATAGAGCCC
603	sgCYB561A3_3	CYB561A3	chr11	61121415	-	GGGTGGGGCCCAAACTGCCC
604	sgCYB561A3_4	CYB561A3	chr11	61121259	+	GCAGGCGAAGAGGAAGACAG
605	sgCYB561A3_5	CYB561A3	chr11	61118560	-	GCGGTCTTTGCCAACAGCAC
606	sgCYB561A3_6	CYB561A3	chr11	61124101	+	GCATCCAGTAGATAGTGAAG
607	sgCYB561A3_7	CYB561A3	chr11	61124088	-	TGGATGCAGTACTGGCGTGG
608	sgCYB561A3_8	CYB561A3	chr11	61120501	+	GATGGACAGAGAGAGGATGG

609	sgCYB561A3_9	CYB561A3	chr11	61120483	+	GCCCGAAATGACGGATGCGA
610	sgCYB561A3_10	CYB561A3	chr11	61118549	-	CAACAGCACCGGGATGCTGG
611	sgCYB561D1_1	CYB561D1	chr1	110036771	-	GGAACCAGACCTACCTCCAG
612	sgCYB561D1_2	CYB561D1	chr1	110036755	+	CACGGCCATGCAGCCCCTGG
613	sgCYB561D1_3	CYB561D1	chr1	110038386	-	GGATGGCTTCAGCCATGCAG
614	sgCYB561D1_4	CYB561D1	chr1	110036802	+	GGGAGCCGAGACTGACCCGC
615	sgCYB561D1_5	CYB561D1	chr1	110036877	-	GGACAGCGCTGTCAGAAAGA
616	sgCYB561D1_6	CYB561D1	chr1	110036782	+	TCTGGTTCCCGCTCCAGCTG
617	sgCYB561D1_7	CYB561D1	chr1	110036813	+	CTGACCCGCTGGCTGCGGAG
618	sgCYB561D1_8	CYB561D1	chr1	110038407	-	AGTGTTCAGGTGAGAAGAGT
619	sgCYB561D1_9	CYB561D1	chr1	110038430	-	TCGGGAGCAGAAGAAGAACA
620	sgCYB561D1_10	CYB561D1	chr1	110038457	+	GGATCCGGCTCCACTGGGCA
621	sgCYB561D2_1	CYB561D2	chr3	50389460	-	ACAGCCAAAGACATAAGCAC
622	sgCYB561D2_2	CYB561D2	chr3	50388959	-	GGCAAGCACAGCCACAAAGA
623	sgCYB561D2_3	CYB561D2	chr3	50388936	-	TAAAGCCCAGGGCCACAAGG
624	sgCYB561D2_4	CYB561D2	chr3	50388947	-	CACAAAGATGGTAAAGCCCA
625	sgCYB561D2_5	CYB561D2	chr3	50388903	-	CAGAAGCAGTACGCAGAGCT
626	sgCYB561D2_6	CYB561D2	chr3	50389444	-	GCACCGGGTGCCAGGAGAAC
627	sgCYB561D2_7	CYB561D2	chr3	50388858	+	GCTGACGATGGCCCCTTTCTG
628	sgCYB561D2_8	CYB561D2	chr3	50388873	-	GTGACTCGGTCTCCGCAGAA
629	sgCYB561D2_9	CYB561D2	chr3	50388985	-	GAATTCTACTTACTGGAGCC
630	sgCYB561D2_10	CYB561D2	chr3	50390764	-	CCAGCAGCTGCAGCACCCAG
631	sgCYB5A_1	CYB5A	chr18	71959092	-	GATGGCAGAGCAGTCGGACG
632	sgCYB5A_2	CYB5A	chr18	71930705	-	TCCTTTGAACAGCATCCTGG
633	sgCYB5A_3	CYB5A	chr18	71930625	-	CGGGCACTCTACAGATGCCA
634	sgCYB5A_4	CYB5A	chr18	71930605	+	GAATGTTTTGGACATTTCCC
635	sgCYB5A_5	CYB5A	chr18	71959033	-	ACAACCACAGCAAGAGCACC
636	sgCYB5A_6	CYB5A	chr18	71959014	-	CTGGCTGATCCTGCACCACA
637	sgCYB5A_7	CYB5A	chr18	71928152	-	ACCAAAGTTAAACAAGCCTC
638	sgCYB5A_8	CYB5A	chr18	71920863	-	GATCCCTGCCATCTCTGCAG
639	sgCYB5A_9	CYB5A	chr18	71920837	+	TAGGCGATACATCAAGGCGA
640	sgCYB5A_10	CYB5A	chr18	71920827	-	GTATCGCCTATACATGGCAG
641	sgCYB5B_1	CYB5B	chr16	69458608	+	GCGGAAGCTAGCGGCAGCGA
642	sgCYB5B_2	CYB5B	chr16	69458690	+	GCAACTCCTTGAAGGAACTG
643	sgCYB5B_3	CYB5B	chr16	69481049	+	GTAGCACCCTGGAGGAGAAG
644	sgCYB5B_4	CYB5B	chr16	69482018	+	AGTGACCTTAAACCTGAAAG
645	sgCYB5B_5	CYB5B	chr16	69458654	-	CAACCGGTAATAGGTGACTG
646	sgCYB5B_6	CYB5B	chr16	69458724	-	TGACATCGTAGACTCGCCCA
647	sgCYB5B_7	CYB5B	chr16	69458739	+	TGTCACCCGCTTCCTCAACG
648	sgCYB5B_8	CYB5B	chr16	69458754	-	TCCCAGGCCCCACCTCGTTG
649	sgCYB5B_9	CYB5B	chr16	69458704	+	GAACTGTGGCTTGTGATCCA
650	sgCYB5B_10	CYB5B	chr16	69458589	+	GTCCGGTTCAATGGCGACTG
651	sgCYB5D1_1	CYB5D1	chr17	7761497	+	TCAGCGTCGCTATTTCACGC
652	sgCYB5D1_2	CYB5D1	chr17	7762049	+	GCAGGGGTCGTATTATGAGG
653	sgCYB5D1_3	CYB5D1	chr17	7762121	+	GTCGCAGGAGCACACACTGG
654	sgCYB5D1_4	CYB5D1	chr17	7761442	+	GAGCAAGAGCCATGCCGCGC
655	sgCYB5D1_5	CYB5D1	chr17	7761554	-	GTCCCAGGTAAGATACCCAG
656	sgCYB5D1_6	CYB5D1	chr17	7761773	-	TGGTCTTTGGATCAAACCAG
657	sgCYB5D1_7	CYB5D1	chr17	7761956	+	CCTGAGGTACTGCACCCCGC
658	sgCYB5D1_8	CYB5D1	chr17	7761983	-	GAGGCGGAACGTGCACAAAG
659	sgCYB5D1_9	CYB5D1	chr17	7761591	+	TCATTGGCACAGGAATACAA
660	sgCYB5D1_10	CYB5D1	chr17	7761725	+	CCTGCTGCTGAAACCCATCG
661	sgCYB5D2_1	CYB5D2	chr17	4058010	-	TACCTGGGTCAGTGCCGGGG
662	sgCYB5D2_2	CYB5D2	chr17	4058027	+	GTAGAAGCTGCGATCACCAG
663	sgCYB5D2_3	CYB5D2	chr17	4058105	+	GCGGAGTGGAGCTCAGCCAG
664	sgCYB5D2_4	CYB5D2	chr17	4057975	+	GACAGGACGGTTCTACGGAG
665	sgCYB5D2_5	CYB5D2	chr17	4057962	+	TCACAGGGAGGGTGACAGGA
666	sgCYB5D2_6	CYB5D2	chr17	4057993	+	AGAGGATGGGCTGCCCACCC
667	sgCYB5D2_7	CYB5D2	chr17	4058086	+	GACATTCCCGCCGTGCAACG
668	sgCYB5D2_8	CYB5D2	chr17	4058133	+	GGCTCTGGTGCTCCCAGAAG
669	sgCYB5D2_9	CYB5D2	chr17	4060344	-	GAGCATGTGATGGCTAGCGG

670	sgCYB5D2_10	CYB5D2	chr17	4060229	-	TGGTTCTCACACACACGCAG
671	sgCYB5R4_1	CYB5R4	chr6	84569517	-	GGGGCCGGGAAAGACTGGGA
672	sgCYB5R4_2	CYB5R4	chr6	84665042	+	GCACCTATTTCTGAATGGAA
673	sgCYB5R4_3	CYB5R4	chr6	84644419	-	GAGGTAAACATGTTGCCCAA
674	sgCYB5R4_4	CYB5R4	chr6	84569543	+	GCAGCAGCGTGTCGCCTCCG
675	sgCYB5R4_5	CYB5R4	chr6	84603264	+	TATATGGAGTATCATCCTGG
676	sgCYB5R4_6	CYB5R4	chr6	84618755	+	ATGCTGAAAGAATGCCTGGT
677	sgCYB5R4_7	CYB5R4	chr6	84624127	+	CTATACAGACTATCGTGAGG
678	sgCYB5R4_8	CYB5R4	chr6	84627763	+	AAGAAGGTCCTAGTTATCCA
679	sgCYB5R4_9	CYB5R4	chr6	84649783	+	GTTTCTGTAAGCAGTCCTGA
680	sgCYB5R4_10	CYB5R4	chr6	84649860	+	TGGAACAGGCTTCACACCAA
681	sgCYBA_1	CYBA	chr16	88714440	+	GGTGGAAGGATACATGGAGT
682	sgCYBA_2	CYBA	chr16	88713204	+	GGGCCCGAACAGCTTCACCA
683	sgCYBA_3	CYBA	chr16	88712572	+	GGTGGCCAGCAGGAAGCCGG
684	sgCYBA_4	CYBA	chr16	88712554	+	GCAGGCGGTCCCAAGGATGG
685	sgCYBA_5	CYBA	chr16	88717379	-	GTGGGCCAACGAACAGGCGC
686	sgCYBA_6	CYBA	chr16	88714458	+	GTAGGCACCAAAGTACCACT
687	sgCYBA_7	CYBA	chr16	88713519	-	GAGGAAGAAGGGCTCCACCA
688	sgCYBA_8	CYBA	chr16	88713224	-	ACAGAAGTACATGACCGCCG
689	sgCYBA_9	CYBA	chr16	88714476	-	CAGCTGGGCGCTTCACCCAG
690	sgCYBA_10	CYBA	chr16	88713499	+	GGAGACTCACCAGCGCTCCA
691	sgCYBB_1	CYBB	chrX	37663174	+	GAAGGGGTTCAAAATGGAAG
692	sgCYBB_2	CYBB	chrX	37655295	+	CCTCCACCAAAACCATCCGG
693	sgCYBB_3	CYBB	chrX	37658313	-	GAGGGTTTCCAGCAAACTGA
694	sgCYBB_4	CYBB	chrX	37664295	+	AGATGTGTTCAGCTATGAGG
695	sgCYBB_5	CYBB	chrX	37664354	-	TGACTTGAGAATGGATGCGA
696	sgCYBB_6	CYBB	chrX	37639334	+	GGGAACTGGGCTGTGAATGA
697	sgCYBB_7	CYBB	chrX	37660568	+	TTGGCGATCTCAACAGAAGG
698	sgCYBB_8	CYBB	chrX	37660580	+	ACAGAAGGTGGTCATCACCA
699	sgCYBB_9	CYBB	chrX	37663156	+	CGAGCTACAGATGAAGAAGA
700	sgCYBB_10	CYBB	chrX	37663319	+	GAGGGGCTGTTCAATGCTTG
701	sgCYBRD1_1	CYBRD1	chr2	172409909	-	GTATGGGCATGAGAAATGCT
702	sgCYBRD1_2	CYBRD1	chr2	172409889	-	CGGAGAGAAAGCGGAGCCCA
703	sgCYBRD1_3	CYBRD1	chr2	172409960	+	AGTGATTGCAACAGCACTTA
704	sgCYBRD1_4	CYBRD1	chr2	172411126	+	GGATAGTCACCAGACCGCAA
705	sgCYBRD1_5	CYBRD1	chr2	172411062	-	TTTACGAAAACACCTTCTGG
706	sgCYBRD1_6	CYBRD1	chr2	172411044	-	GGCGGGAATGTACTGTATGC
707	sgCYBRD1_7	CYBRD1	chr2	172411103	-	AAATGAGGGCCCCGAACACC
708	sgCYBRD1_8	CYBRD1	chr2	172411139	+	ACCGCAATGGAAACGTCCTA
709	sgCYBRD1_9	CYBRD1	chr2	172379138	-	GTAGTGGAGGACCCAGACGA
710	sgCYBRD1_10	CYBRD1	chr2	172398098	+	TCATCGTCTACAGACTGCCG
711	sgCYC1_1	CYC1	chr8	145150751	+	TCGAAGTCTGGCCTTTCCCG
712	sgCYC1_2	CYC1	chr8	145150882	-	GGTGAGACCACGGATAGCTG
713	sgCYC1_3	CYC1	chr8	145151105	+	GGTGGGCGTGTGCTACACGG
714	sgCYC1_4	CYC1	chr8	145150909	-	TGTGGTCCAAGGAAGAGAGG
715	sgCYC1_5	CYC1	chr8	145151068	-	GAAGTCCATGCTGTGGCAGG
716	sgCYC1_6	CYC1	chr8	145151251	+	AGACGGCCCCAATGAAGATG
717	sgCYC1_7	CYC1	chr8	145151473	+	GGATCTGGTCCTAGGCATGG
718	sgCYC1_8	CYC1	chr8	145151526	+	CTACTGCGAGCCACCCACCG
719	sgCYC1_9	CYC1	chr8	145151543	-	TTCCCGCAGTGACACCCCGG
720	sgCYC1_10	CYC1	chr8	145151612	-	GACATCTGTGTAGATGGGAG
721	sgCYCS_1	CYCS	chr7	25163454	-	TTTAGGCATCATCTGGGGAG
722	sgCYCS_2	CYCS	chr7	25163459	-	TCTTGTTTAGGCATCATCTG
723	sgCYCS_3	CYCS	chr7	25163572	-	ACAGCCGCCAATAAGAACAA
724	sgCYCS_4	CYCS	chr7	25163618	+	TCTTCCGCCCAAAGAGACCA
725	sgCYCS_5	CYCS	chr7	25163586	+	GGCTGTGTAAGAGTATCCAG
726	sgCYCS_6	CYCS	chr7	25163658	+	GTGCTTGCCTCCCTTTTCAA
727	sgCYCS_7	CYCS	chr7	25163605	-	CGGAAGACAGGTCAGGCCCC
728	sgCYCS_8	CYCS	chr7	25163668	-	TGCCACACCGTTGAAAAGGG
729	sgCYCS_9	CYCS	chr7	25163663	+	TGCCTCCCTTTTCAACGGTG
730	sgCYCS_10	CYCS	chr7	25163638	-	AAGACTGGGCCAAATCTCCA

731	sgCYGB_1	CYGB	chr17	74533589	-	GCGAGATGGAGATCGAGCGC
732	sgCYGB_2	CYGB	chr17	74527601	+	GCACAGAGGACACCTTGTCG
733	sgCYGB_3	CYGB	chr17	74533603	-	GGAGAAAGTGCCAGGCGAGA
734	sgCYGB_4	CYGB	chr17	74533561	-	GAGCGAGGAGCTGTCCGAGG
735	sgCYGB_5	CYGB	chr17	74527734	+	GCTGAAGTACTGCTTGGCCG
736	sgCYGB_6	CYGB	chr17	74527718	-	CAGCCAGTTCAAGCACATGG
737	sgCYGB_7	CYGB	chr17	74527690	+	GGGCTCCGCTCCATCTCCAG
738	sgCYGB_8	CYGB	chr17	74527562	-	CCACGCCCTCAAGCACAAGG
739	sgCYGB_9	CYGB	chr17	74527129	+	GAGGCCACGCAGCTTGGCCC
740	sgCYGB_10	CYGB	chr17	74533549	-	GTCCGAGGCGGAGAGGAAGG
741	sgCYP11A1_1	CYP11A1	chr15	74637483	+	CGAGACACTGCATCCAACAG
742	sgCYP11A1_2	CYP11A1	chr15	74636292	-	GGAGCGCCAGGGGATGCTGG
743	sgCYP11A1_3	CYP11A1	chr15	74636274	-	GGAGGAAGTAGTGAACCCCG
744	sgCYP11A1_4	CYP11A1	chr15	74635392	+	GGAGTCTGTAGAGGATGCCA
745	sgCYP11A1_5	CYP11A1	chr15	74632022	-	GCAGGATATGCTGCGGGCAG
746	sgCYP11A1_6	CYP11A1	chr15	74631029	-	AAAATTTTGACCCAACCCGA
747	sgCYP11A1_7	CYP11A1	chr15	74630954	-	GTGTGCGGCAGTGTCTGGGA
748	sgCYP11A1_8	CYP11A1	chr15	74637445	-	GCACAGGCGCATCAAGAAGG
749	sgCYP11A1_9	CYP11A1	chr15	74637425	-	CGGGCTCCGGAAATTACTCG
750	sgCYP11A1_10	CYP11A1	chr15	74636173	-	GTCTGTTCAGGACCAAGACC
751	sgCYP11B1_1	CYP11B1	chr8	143958560	+	GAGGAACCTCTGCACAGCGT
752	sgCYP11B1_2	CYP11B1	chr8	143957740	-	ACAGTACACCAGCATCGTGG
753	sgCYP11B1_3	CYP11B1	chr8	143961142	-	ACTGGGCACGAGAGCCGCCC
754	sgCYP11B1_4	CYP11B1	chr8	143961086	-	CCCGGCGTCCAGGCAACAGG
755	sgCYP11B1_5	CYP11B1	chr8	143960498	+	GGGCTCCAGGCTCATCCTGT
756	sgCYP11B1_6	CYP11B1	chr8	143957228	+	GCAGGGCCTGCTGCACGTTG
757	sgCYP11B1_7	CYP11B1	chr8	143961115	+	CAAAGGGCAGCACTGTCCTG
758	sgCYP11B1_8	CYP11B1	chr8	143960996	+	AATGGGCCCTAGTTCCTGGA
759	sgCYP11B1_9	CYP11B1	chr8	143960554	-	TGTGATGCTGCCGGAGGACG
760	sgCYP11B1_10	CYP11B1	chr8	143957698	-	ACTGTCGCCAGATGCCATCA
761	sgCYP11B2_1	CYP11B2	chr8	143994801	+	GCAGGATCTGCTGCACGTCG
762	sgCYP11B2_2	CYP11B2	chr8	143994090	-	GCAATGCCGCCTTGTTCCCG
763	sgCYP11B2_3	CYP11B2	chr8	143993988	-	GCATGCGCCAGTGCCTCGGG
764	sgCYP11B2_4	CYP11B2	chr8	143999194	-	GGCTGTCCCTGCAAAGGGCA
765	sgCYP11B2_5	CYP11B2	chr8	143999143	+	AAACGGCAGCACCGTCCTAG
766	sgCYP11B2_6	CYP11B2	chr8	143999032	-	ACCAGACCTTCCAGGAGCTG
767	sgCYP11B2_7	CYP11B2	chr8	143995763	-	ACACTACACAGGCATCGTGG
768	sgCYP11B2_8	CYP11B2	chr8	143995745	-	GGCGGAGCTCCTGTTGAAGG
769	sgCYP11B2_9	CYP11B2	chr8	143995721	-	ACTGTCACTAGAAGCCATCA
770	sgCYP11B2_10	CYP11B2	chr8	143994727	-	CCACCGAGCTGCCCTTGCTG
771	sgCYP17A1_1	CYP17A1	chr10	104595087	+	GTGTGCGCCAGAGTCAGCGA
772	sgCYP17A1_2	CYP17A1	chr10	104597065	+	GGGCCAAAACAAATAAGCTA
773	sgCYP17A1_3	CYP17A1	chr10	104596971	+	GCATATGGCCGTGTCTGGGG
774	sgCYP17A1_4	CYP17A1	chr10	104596848	-	GGTGCTTATTAAGAAGGGCA
775	sgCYP17A1_5	CYP17A1	chr10	104595062	-	GCTGCATCGAAGGCTGGCGA
776	sgCYP17A1_6	CYP17A1	chr10	104592777	+	GGTCCATTTAACCACAGAGG
777	sgCYP17A1_7	CYP17A1	chr10	104592318	+	GAGGCGAAGCACCTCTCGGA
778	sgCYP17A1_8	CYP17A1	chr10	104592261	+	GGCACACCTGGAGTCAACGT
779	sgCYP17A1_9	CYP17A1	chr10	104591298	-	GGCGCTGCATCACAATGAGA
780	sgCYP17A1_10	CYP17A1	chr10	104594726	-	GATATGCTGGCCACCCACAA
781	sgCYP19A1_1	CYP19A1	chr15	51535017	+	GAGGAGCAGGACTGGCATGG
782	sgCYP19A1_2	CYP19A1	chr15	51514690	+	TGACCATACGAACAAGGCCG
783	sgCYP19A1_3	CYP19A1	chr15	51507337	+	GAAGAACAAAGAGACAGACA
784	sgCYP19A1_4	CYP19A1	chr15	51529157	+	GAATCTGCCGTGGGAGATGA
785	sgCYP19A1_5	CYP19A1	chr15	51529147	+	CCATCCACAGGAATCTGCCG
786	sgCYP19A1_6	CYP19A1	chr15	51529078	-	GCGAGTCTGGATCTCTGGAG
787	sgCYP19A1_7	CYP19A1	chr15	51514645	-	ACATCTGGACAGGTTGGAGG
788	sgCYP19A1_8	CYP19A1	chr15	51510808	+	GCCATGCATCAAAATAACCT
789	sgCYP19A1_9	CYP19A1	chr15	51507375	+	TCAGCATTTCCAATATGCAC
790	sgCYP19A1_10	CYP19A1	chr15	51507300	-	AAAGCACCCTAATGTTGAAG
791	sgCYP1A1_1	CYP1A1	chr15	75015316	-	AAGGCCTGAAGAATCCACCA

792	sgCYP1A1_2	CYP1A1	chr15	75015189	-	AATTGGCTCCACACCCGTGG
793	sgCYP1A1_3	CYP1A1	chr15	75014686	+	GAAGGCATTCAGGGAAGGGT
794	sgCYP1A1_4	CYP1A1	chr15	75013354	-	GAAAGGCTTTTACATCCCCA
795	sgCYP1A1_5	CYP1A1	chr15	75015256	+	CAGGTGCGGGTTCTTTCCCA
796	sgCYP1A1_6	CYP1A1	chr15	75015216	+	GCAGCACGTCCCCATACTGC
797	sgCYP1A1_7	CYP1A1	chr15	75015173	+	AGGCCGCTCAGCACCACCAC
798	sgCYP1A1_8	CYP1A1	chr15	75014805	+	GGTGGTTGTGGTCATAGCGC
799	sgCYP1A1_9	CYP1A1	chr15	75014616	-	GCACTACAAAACCTTTGAGA
800	sgCYP1A1_10	CYP1A1	chr15	75013764	-	GGTACAGAGAAAGATCCAAG
801	sgCYP1B1_1	CYP1B1	chr2	38302342	-	GCCACTGATCGGAAACGCGG
802	sgCYP1B1_2	CYP1B1	chr2	38302145	+	GCCGCCGGACACCACACGGA
803	sgCYP1B1_3	CYP1B1	chr2	38301953	-	ACGGCGCCTTCCTCGACCCG
804	sgCYP1B1_4	CYP1B1	chr2	38301890	+	GTAGCGGCAGCCGAAACACA
805	sgCYP1B1_5	CYP1B1	chr2	38301856	+	TGAGCAGCTCACGGAACTCG
806	sgCYP1B1_6	CYP1B1	chr2	38301503	+	GAGCAGCCACTGCAGCGCGG
807	sgCYP1B1_7	CYP1B1	chr2	38302467	+	GTGGTCTGCTGGATGGACAG
808	sgCYP1B1_8	CYP1B1	chr2	38302403	-	AGCGGCTGCTGAGGCAACGG
809	sgCYP1B1_9	CYP1B1	chr2	38302297	+	GCGCCAGGCGAGCGAACGAG
810	sgCYP1B1_10	CYP1B1	chr2	38301920	+	GACGTTGGCCACGGCCACGA
811	sgCYP20A1_1	CYP20A1	chr2	204111567	-	GAGGCGCCTGCCAAACCAGA
812	sgCYP20A1_2	CYP20A1	chr2	204103787	-	GAAGATCGCGAAGTCCAACA
813	sgCYP20A1_3	CYP20A1	chr2	204110566	+	AAGGCTTCCAGACAAGCTGC
814	sgCYP20A1_4	CYP20A1	chr2	204111538	+	GCATGAGAGATATGGGCCTG
815	sgCYP20A1_5	CYP20A1	chr2	204111586	-	CAGTGCCCAAACTAACCACG
816	sgCYP20A1_6	CYP20A1	chr2	204143300	-	AAACAGACTCCAGTTGCATG
817	sgCYP20A1_7	CYP20A1	chr2	204156989	-	ACCAAGGGCATAAAGGACGA
818	sgCYP20A1_8	CYP20A1	chr2	204157005	-	GATCCTGAAGTACCACACCA
819	sgCYP20A1_9	CYP20A1	chr2	204116722	+	TTAAGGTATCAATCTGGTGG
820	sgCYP20A1_10	CYP20A1	chr2	204116747	+	GTGTGAGTGAAAACCACATG
821	sgCYP21A2_1	CYP21A2	chr6	32006293	-	AAGAAGCCCGGGGCAAGAGG
822	sgCYP21A2_2	CYP21A2	chr6	32007567	+	AGAGAAGAGGGATCACATCG
823	sgCYP21A2_3	CYP21A2	chr6	32008182	+	GATTCAGCAGCGACTGCAGG
824	sgCYP21A2_4	CYP21A2	chr6	32006902	-	GGGAGTAGTCTCCCAAGGAC
825	sgCYP21A2_5	CYP21A2	chr6	32007581	+	ACATCGTGGAGATGCAGCTG
826	sgCYP21A2_6	CYP21A2	chr6	32008311	-	CTAAGGGCACAACGGGCCGC
827	sgCYP21A2_7	CYP21A2	chr6	32006887	+	GAACTACCCGGACCTGTCCT
828	sgCYP21A2_8	CYP21A2	chr6	32006922	-	CTTGTGGGCTTTCCAGAGCA
829	sgCYP21A2_9	CYP21A2	chr6	32007772	+	CCCTCTGCAGGAGAGCCTCG
830	sgCYP21A2_10	CYP21A2	chr6	32007790	-	CCCTCCACTGGCCTGCCACG
831	sgCYP24A1_1	CYP24A1	chr20	52789499	+	GGTTTGATCTCCAGCCGCTG
832	sgCYP24A1_2	CYP24A1	chr20	52788191	-	GGGAAGGGGAAGACTGGCAG
833	sgCYP24A1_3	CYP24A1	chr20	52786181	-	GATGAGCTCTGTGATGAAAG
834	sgCYP24A1_4	CYP24A1	chr20	52782319	-	GCTTCTCCAGAAGAATGCAG
835	sgCYP24A1_5	CYP24A1	chr20	52781039	-	GCACAAGAGCCTCAACACCA
836	sgCYP24A1_6	CYP24A1	chr20	52779264	-	AGAGCTCCAGCTGGCTGCGG
837	sgCYP24A1_7	CYP24A1	chr20	52790073	+	GCAGGAAGGCGGCAAGCGAG
838	sgCYP24A1_8	CYP24A1	chr20	52790062	+	GCGCAGCTGCTGCAGGAAGG
839	sgCYP24A1_9	CYP24A1	chr20	52789985	+	GGGCAGACTGGCACCTCTCG
840	sgCYP24A1_10	CYP24A1	chr20	52789863	-	CAAGAAACAGCACGACACCC
841	sgCYP26A1_1	CYP26A1	chr10	94834072	+	AGTTCCTGCAGATGAAGCGC
842	sgCYP26A1_2	CYP26A1	chr10	94834703	+	GGGCTGCGAACCCCAACTGG
843	sgCYP26A1_3	CYP26A1	chr10	94834171	+	GCATCTTGCTCGGAGAGCAC
844	sgCYP26A1_4	CYP26A1	chr10	94834580	+	GCCGGTGATCACCGAGGAAG
845	sgCYP26A1_5	CYP26A1	chr10	94834611	+	CTGGAGCAGTGGCTGAGCTG
846	sgCYP26A1_6	CYP26A1	chr10	94834640	+	CGGCCTCCTGGTCTACCCCG
847	sgCYP26A1_7	CYP26A1	chr10	94834690	-	GCAGCCCAGTAGGATGCGCA
848	sgCYP26A1_8	CYP26A1	chr10	94834898	+	GCGCTCAGGGCATGAAGGCG
849	sgCYP26A1_9	CYP26A1	chr10	94834138	-	CGCGCCCATCACCCGTACGG
850	sgCYP26A1_10	CYP26A1	chr10	94834231	-	GAGGCAGCCAGATCCCAGAA
851	sgCYP26B1_1	CYP26B1	chr2	72362378	+	GAAGCCCAGCAGCACCCGGA
852	sgCYP26B1_2	CYP26B1	chr2	72361916	-	GAGCAGCAAGGAGCACGGGA

853	sgCYP26B1_3	CYP26B1	chr2	72374901	+	GGACACCAGGCACGCGGCGA
854	sgCYP26B1_4	CYP26B1	chr2	72374829	+	GCAGCTCTTGTCGCGAGTGG
855	sgCYP26B1_5	CYP26B1	chr2	72371251	+	GCGCCGGTCACGCGTATCAG
856	sgCYP26B1_6	CYP26B1	chr2	72362275	+	GCCGGTAGCCACTGAAGGGC
857	sgCYP26B1_7	CYP26B1	chr2	72362012	-	GCAGATCCTGCAGAAGGGGC
858	sgCYP26B1_8	CYP26B1	chr2	72361980	+	GCACTGCAGCTTCTCCCGGA
859	sgCYP26B1_9	CYP26B1	chr2	72360331	+	GCAGCTTCTCCAGCACAGTG
860	sgCYP26B1_10	CYP26B1	chr2	72371195	+	GCCACTCGGTGCTCACGAGG
861	sgCYP26C1_1	CYP26C1	chr10	94825781	+	GCTGCTACTGCAGCATCCGG
862	sgCYP26C1_2	CYP26C1	chr10	94822616	-	GATGCGCGCGGCCATGCGGA
863	sgCYP26C1_3	CYP26C1	chr10	94822746	-	GGGGCGGCCGTACCTTGCGT
864	sgCYP26C1_4	CYP26C1	chr10	94821066	+	GGCACTGCTCTCCTGTGCGC
865	sgCYP26C1_5	CYP26C1	chr10	94821116	-	GCATCCAGCGGAGGGTCCAG
866	sgCYP26C1_6	CYP26C1	chr10	94821128	-	GGTCCCGGCTCAGCATCCAG
867	sgCYP26C1_7	CYP26C1	chr10	94821181	-	GCCGAAGAAGGGCCACCCCA
868	sgCYP26C1_8	CYP26C1	chr10	94821808	+	AGTGTTCAAGACGCACCTGC
869	sgCYP26C1_9	CYP26C1	chr10	94821895	-	GCCACTGGCTGCGCACCAGG
870	sgCYP26C1_10	CYP26C1	chr10	94822514	+	GCTACGTGCCGCGCCTGCAG
871	sgCYP27A1_1	CYP27A1	chr2	219674386	+	GGAGCAAGTGATGCGGCAAG
872	sgCYP27A1_2	CYP27A1	chr2	219677312	+	CCTGCAGCGATCCATCCCCG
873	sgCYP27A1_3	CYP27A1	chr2	219677663	+	TGAGAAGCTCGAAGATATGG
874	sgCYP27A1_4	CYP27A1	chr2	219678782	+	AAAGGACCCTGAGATCCAGG
875	sgCYP27A1_5	CYP27A1	chr2	219678803	+	GGCCTTGCACGAGGAAGTGG
876	sgCYP27A1_6	CYP27A1	chr2	219679329	+	AAAGCTTCCAGCCCCACCGC
877	sgCYP27A1_7	CYP27A1	chr2	219679345	-	GGCTGTTTCTCAGCCAGCGG
878	sgCYP27A1_8	CYP27A1	chr2	219679459	+	GATGCAGCTACTCCTCGCAA
879	sgCYP27A1_9	CYP27A1	chr2	219674413	+	GTACCCAGTACGGAACGACA
880	sgCYP27A1_10	CYP27A1	chr2	219676984	+	CCAGCGGTTGCTGAAGCCAG
881	sgCYP27B1_1	CYP27B1	chr12	58160780	+	GACGCGATGGAACACTCTGG
882	sgCYP27B1_2	CYP27B1	chr12	58158661	-	GAGGCAGCCATGAGGAACGG
883	sgCYP27B1_3	CYP27B1	chr12	58158193	+	GGGCAGCTGGGACAGAACAG
884	sgCYP27B1_4	CYP27B1	chr12	58159797	+	GCAGTCCGCAAGCCCGCTGG
885	sgCYP27B1_5	CYP27B1	chr12	58159236	+	GGAGCGGGGCCAGGAGACTG
886	sgCYP27B1_6	CYP27B1	chr12	58158966	+	GCGCGAGCCGAGCAGAACCG
887	sgCYP27B1_7	CYP27B1	chr12	58158896	+	CAAACACCGAGCCCACAGCG
888	sgCYP27B1_8	CYP27B1	chr12	58158845	+	GCACAAGGTGGCGCAGCCAG
889	sgCYP27B1_9	CYP27B1	chr12	58158686	-	GAGGCACGTGGAGCGGCGAG
890	sgCYP27B1_10	CYP27B1	chr12	58158647	-	GAACGGAGGACAGCCCGAGA
891	sgCYP27C1_1	CYP27C1	chr2	127958830	-	GCTGACTGTGGAATACATCG
892	sgCYP27C1_2	CYP27C1	chr2	127958774	+	GATGGCGCCTGCATACATGG
893	sgCYP27C1_3	CYP27C1	chr2	127958756	+	GGGGCGAAGCCATCTGGGGA
894	sgCYP27C1_4	CYP27C1	chr2	127958721	+	GACCTGCAGAATTCCCGCCA
895	sgCYP27C1_5	CYP27C1	chr2	127953045	+	GAGGTACACAGTCCAAGACA
896	sgCYP27C1_6	CYP27C1	chr2	127951415	-	AGTGCTGCCAGGGAACGGCC
897	sgCYP27C1_7	CYP27C1	chr2	127950721	+	GCTGCGAACCCCATGACCAA
898	sgCYP27C1_8	CYP27C1	chr2	127950663	+	GCCCTACCTGGATCACGACG
899	sgCYP27C1_9	CYP27C1	chr2	127958876	+	CAAACGACTCTCATAAAGGA
900	sgCYP27C1_10	CYP27C1	chr2	127957002	+	GCAGCGTCAGAGCCTGGCTA
901	sgCYP2A13_1	CYP2A13	chr19	41599633	-	GCAGCAGGAAACCGTAGCGC
902	sgCYP2A13_2	CYP2A13	chr19	41600331	-	GGGGAGACAGCACCTTAGGG
903	sgCYP2A13_3	CYP2A13	chr19	41600214	+	GGCCAAGATGCCCTACACAG
904	sgCYP2A13_4	CYP2A13	chr19	41600279	-	GACCCTGTGGGCCAAACCCA
905	sgCYP2A13_5	CYP2A13	chr19	41596003	-	AAAACCCCTTAGGGTGGCGA
906	sgCYP2A13_6	CYP2A13	chr19	41596088	-	ACTCACCGTGCGTGCCCCGG
907	sgCYP2A13_7	CYP2A13	chr19	41597714	+	GGAGGACTTCATCGCCAAGA
908	sgCYP2A13_8	CYP2A13	chr19	41599597	-	TCTCAGTGCCCGCAAAGAAG
909	sgCYP2A13_9	CYP2A13	chr19	41599620	-	GTAGCGCAGGGTGGTGCTCA
910	sgCYP2A13_10	CYP2A13	chr19	41600943	+	AGCACTTCCTGGATAAGAAG
911	sgCYP2A6_1	CYP2A6	chr19	41351238	+	GACTCTGCGGGCCAAACTCA
912	sgCYP2A6_2	CYP2A6	chr19	41356302	+	GGCCACCAGAAGCATCCCTG
913	sgCYP2A6_3	CYP2A6	chr19	41356243	-	TGGCAGCAGAGGAAGAGCAA

914	sgCYP2A6_4	CYP2A6	chr19	41351898	+	ATAGCGCAGGGTGGTGCTGA
915	sgCYP2A6_5	CYP2A6	chr19	41350579	-	AGCACTTCCTGAATGAGAAG
916	sgCYP2A6_6	CYP2A6	chr19	41354653	-	CCAGGCGTGGTATTCAGCAA
917	sgCYP2A6_7	CYP2A6	chr19	41356280	-	GCTGGTCTGCCTGACTGTAA
918	sgCYP2A6_8	CYP2A6	chr19	41354514	+	CTCACCGCCAGTGCCCCGGA
919	sgCYP2A6_9	CYP2A6	chr19	41351935	-	ACCACGTTGAACCTCTTCAT
920	sgCYP2A6_10	CYP2A6	chr19	41350594	+	GTGCTGGGGATTGAAGTCCT
921	sgCYP2A7_1	CYP2A7	chr19	41382492	+	GCTGGGGATTGAAGTCCTGA
922	sgCYP2A7_2	CYP2A7	chr19	41386472	-	GCTTTGCCATCGCCACCCTG
923	sgCYP2A7_3	CYP2A7	chr19	41386405	-	GGAGTCGGGCTTCCTCATCG
924	sgCYP2A7_4	CYP2A7	chr19	41384684	+	AAGGAGTCGATGAAGTCCTG
925	sgCYP2A7_5	CYP2A7	chr19	41387984	+	TCCAATGAAGGGCAGTGGGG
926	sgCYP2A7_6	CYP2A7	chr19	41386420	-	GGAGCGCATCCAGGAGGAGT
927	sgCYP2A7_7	CYP2A7	chr19	41382476	+	TTGTCATCCAGGAAATGCTG
928	sgCYP2A7_8	CYP2A7	chr19	41383134	+	AACCCTGCGGGCCAAACTCA
929	sgCYP2A7_9	CYP2A7	chr19	41387956	+	TGTGCTCTGTGTTCAGCTGG
930	sgCYP2A7_10	CYP2A7	chr19	41386457	+	CACCCCGAAGTCCCTCAGGG
931	sgCYP2B6_1	CYP2B6	chr19	41510293	+	GGAGGAGCGGATTCAGGAGG
932	sgCYP2B6_2	CYP2B6	chr19	41512870	-	GAATCGTTTTCCAAAGACGA
933	sgCYP2B6_3	CYP2B6	chr19	41515909	-	GCTGAATTCACTGTGTGCGT
934	sgCYP2B6_4	CYP2B6	chr19	41518240	-	GAAGCTCTGGAGGGCGATGT
935	sgCYP2B6_5	CYP2B6	chr19	41497246	-	GGAGTAGCAAGAGTCCTGTG
936	sgCYP2B6_6	CYP2B6	chr19	41497279	-	GGCGGTCATGGGTGTTAGGG
937	sgCYP2B6_7	CYP2B6	chr19	41509992	+	GGCCCTTGTGGACAAGGCTG
938	sgCYP2B6_8	CYP2B6	chr19	41510041	+	ATGGTCGACCCATTCTTCCG
939	sgCYP2B6_9	CYP2B6	chr19	41510319	+	AGTGTCTGATAGAGGAGCTT
940	sgCYP2B6_10	CYP2B6	chr19	41512816	-	GAGGAAGGTGGGGTCCATGA
941	sgCYP2C18_1	CYP2C18	chr10	96447922	-	GAGTCATGAGGCAGAAACGC
942	sgCYP2C18_2	CYP2C18	chr10	96454807	-	ATGGAGAGCTCAGAATCCTG
943	sgCYP2C18_3	CYP2C18	chr10	96480266	+	GCTCCTGCTGAAGTACCCAG
944	sgCYP2C18_4	CYP2C18	chr10	96447579	-	ATATCCATGCAACACCACAA
945	sgCYP2C18_5	CYP2C18	chr10	96447613	+	GGCCCTGATTGATCATGGAG
946	sgCYP2C18_6	CYP2C18	chr10	96447955	+	GATGGGGAAGAGGAGCATCG
947	sgCYP2C18_7	CYP2C18	chr10	96480204	+	GTAACTGATATGTTTGGGGC
948	sgCYP2C18_8	CYP2C18	chr10	96484152	-	GACTCCTGTCCTGCATACAG
949	sgCYP2C18_9	CYP2C18	chr10	96484224	-	GGGGCAGGTTGGTGGGGAGG
950	sgCYP2C18_10	CYP2C18	chr10	96493076	-	ATTGTGCAGCACAGAAGTCA
951	sgCYP2C19_1	CYP2C19	chr10	96534871	+	GGTGCTGCATGGATATGAAG
952	sgCYP2C19_2	CYP2C19	chr10	96580304	+	GCAGCTGACTTACTTGGAGC
953	sgCYP2C19_3	CYP2C19	chr10	96602643	-	GGCCCCTGTCCTGCATGCAG
954	sgCYP2C19_4	CYP2C19	chr10	96609696	-	GTCATGTAGCACAGAAGTGA
955	sgCYP2C19_5	CYP2C19	chr10	96541697	+	GATGGACATCAACAACCCTC
956	sgCYP2C19_6	CYP2C19	chr10	96541715	-	AGCAATCAATAAAGTCCCGA
957	sgCYP2C19_7	CYP2C19	chr10	96602605	+	GAAGAGATTGAACGTGTCGT
958	sgCYP2C19_8	CYP2C19	chr10	96609752	-	CCTTCATCCAGAAAGTGACG
959	sgCYP2C19_9	CYP2C19	chr10	96534847	+	GTATTTTGGCCTGGAACGCA
960	sgCYP2C19_10	CYP2C19	chr10	96522556	-	ACTGGGAGAGGAGTAGGGCC
961	sgCYP2C8_1	CYP2C8	chr10	96818231	+	GGGAAACAATCAATGAGTAG
962	sgCYP2C8_2	CYP2C8	chr10	96827055	+	AGGTTGTGAGGGAGAAACGC
963	sgCYP2C8_3	CYP2C8	chr10	96827044	+	AAAATTCCGCAAGGTTGTGA
964	sgCYP2C8_4	CYP2C8	chr10	96826986	-	GGAAGCTCACTGCCTTGTGG
965	sgCYP2C8_5	CYP2C8	chr10	96802773	-	GGAGCCCCTGCATGCAGGAT
966	sgCYP2C8_6	CYP2C8	chr10	96802687	+	ACTGCATGGGGCACACCGGT
967	sgCYP2C8_7	CYP2C8	chr10	96802675	+	GTATCAGTGGTCACTGCATG
968	sgCYP2C8_8	CYP2C8	chr10	96818148	-	AAAAGAACACCAAGCATCAC
969	sgCYP2C8_9	CYP2C8	chr10	96818130	-	ACTGGATGTTAACAATCCTC
970	sgCYP2C8_10	CYP2C8	chr10	96805557	+	GTGGTCCTACCTGTGACCTC
971	sgCYP2C9_1	CYP2C9	chr10	96745812	-	GTCATGTAGCACAGAAGTCA
972	sgCYP2C9_2	CYP2C9	chr10	96731912	+	GCAGTTGACTTGTTTGGAGC
973	sgCYP2C9_3	CYP2C9	chr10	96740989	-	GGCTCCTGTCTTGCATGCAG
974	sgCYP2C9_4	CYP2C9	chr10	96708984	+	AATGGACATGAACAACCCTC

975	sgCYP2C9_5	CYP2C9	chr10	96698538	-	CAATCACTGGGAGAGGAGTG
976	sgCYP2C9_6	CYP2C9	chr10	96701667	-	ATATCCATGCAGCACCACTA
977	sgCYP2C9_7	CYP2C9	chr10	96701731	+	TGGAAGAGGCATTTTCCCAC
978	sgCYP2C9_8	CYP2C9	chr10	96745868	-	CCTTCATCCAGAAAGTGATG
979	sgCYP2C9_9	CYP2C9	chr10	96707668	+	TCAAGATTTTGAGCAGCCCC
980	sgCYP2C9_10	CYP2C9	chr10	96740951	+	GAAGAGATTGAACGTGTGAT
981	sgCYP2D6_1	CYP2D6	chr22	42525770	+	GGGTGATGGGCACAGGCGGG
982	sgCYP2D6_2	CYP2D6	chr22	42524310	+	CCAGCGCTGGGATATGCAGG
983	sgCYP2D6_3	CYP2D6	chr22	42526700	+	GTGCAGCCCAGCGTTGGCGC
984	sgCYP2D6_4	CYP2D6	chr22	42525756	+	GAAACCCAGGATCTGGGTGA
985	sgCYP2D6_5	CYP2D6	chr22	42523925	+	GGTCAGCCACCACTATGCAC
986	sgCYP2D6_6	CYP2D6	chr22	42526748	+	AGATGGCCACTATCACGGCC
987	sgCYP2D6_7	CYP2D6	chr22	42525743	+	GGGAACGCGGCCCGAAACCC
988	sgCYP2D6_8	CYP2D6	chr22	42523881	+	CCAGGCCAGCGTGGTCGAGG
989	sgCYP2D6_9	CYP2D6	chr22	42525811	-	CGCGAGGCGCTGGTGACCCA
990	sgCYP2D6_10	CYP2D6	chr22	42526778	-	AGGTATGGGGCTAGAAGCAC
991	sgCYP2E1_1	CYP2E1	chr10	135342041	+	GGTGATGCACGGCTACAAGG
992	sgCYP2E1_2	CYP2E1	chr10	135342116	-	TGTGCGCATGGAACGCGGGG
993	sgCYP2E1_3	CYP2E1	chr10	135345110	+	CTACCTGGAAGGACATCCGG
994	sgCYP2E1_4	CYP2E1	chr10	135345129	-	GGGTGGTCAGGGAAAACCGC
995	sgCYP2E1_5	CYP2E1	chr10	135345176	+	GCAATGAGAGCCGGATCCAG
996	sgCYP2E1_6	CYP2E1	chr10	135345217	+	GAAGCACTCAGGAAGACCCA
997	sgCYP2E1_7	CYP2E1	chr10	135350687	-	GGGCAGGTTGGAGGGCACGA
998	sgCYP2E1_8	CYP2E1	chr10	135340911	-	GCAGGGCCACGGTGACTCCG
999	sgCYP2E1_9	CYP2E1	chr10	135341000	-	GGGAAGCGGGAAAGGGCCTG
1000	sgCYP2E1_10	CYP2E1	chr10	135341993	-	TGAACACCGGCCCGAAGCGC
1001	sgCYP2F1_1	CYP2F1	chr19	41622187	-	GAGAGGGGTCTGGGTCCCGG
1002	sgCYP2F1_2	CYP2F1	chr19	41622482	-	AAAAGGCAGGGTAGTCACCG
1003	sgCYP2F1_3	CYP2F1	chr19	41626343	+	TGAGGAGCGAATCCTAGAGG
1004	sgCYP2F1_4	CYP2F1	chr19	41626361	+	GGAGGGCAGCTTCCTGCTGG
1005	sgCYP2F1_5	CYP2F1	chr19	41630634	+	GGAGGAGATCGACCTCGTGG
1006	sgCYP2F1_6	CYP2F1	chr19	41622422	-	TCACAGCTTGGTACCCGCTG
1007	sgCYP2F1_7	CYP2F1	chr19	41622441	+	AAGGAGGCCCTGGTGGACCA
1008	sgCYP2F1_8	CYP2F1	chr19	41622462	+	GGAGAGGAGTTTAGTGGCCG
1009	sgCYP2F1_9	CYP2F1	chr19	41626307	+	GATTCTACGGAATTTCGGGA
1010	sgCYP2F1_10	CYP2F1	chr19	41626380	+	GCGGAGCTGCGGAAAACTGA
1011	sgCYP2J2_1	CYP2J2	chr1	60370651	+	GTAGGGCATGGACTCCCGGG
1012	sgCYP2J2_2	CYP2J2	chr1	60392364	+	GAGGATGGACCACTGCCCAG
1013	sgCYP2J2_3	CYP2J2	chr1	60392286	-	GCGCCCAAAGAACTACCCGC
1014	sgCYP2J2_4	CYP2J2	chr1	60392345	+	GTGCCCAGTAGGAGAGTCCG
1015	sgCYP2J2_5	CYP2J2	chr1	60392250	+	AGAAGTTGCCAAGGAAGGGC
1016	sgCYP2J2_6	CYP2J2	chr1	60377873	-	AGAGGAACGCATTCAGGAGG
1017	sgCYP2J2_7	CYP2J2	chr1	60373498	-	CAACTTCCACAACTCTGCGA
1018	sgCYP2J2_8	CYP2J2	chr1	60377361	+	AAAGCGTTCTCCGAAGGTGA
1019	sgCYP2J2_9	CYP2J2	chr1	60370695	-	GATTGACAGAGTGATTGGCC
1020	sgCYP2J2_10	CYP2J2	chr1	60366694	+	GGTCCGGATTGAATGTGTCA
1021	sgCYP2R1_1	CYP2R1	chr11	14913718	-	GAGAGCTGAAGAGGGCGCGG
1022	sgCYP2R1_2	CYP2R1	chr11	14913643	-	GCTGAAGCAGAGGCGGCCGA
1023	sgCYP2R1_3	CYP2R1	chr11	14913529	-	GCAGAGCCAGGTGTACGGAG
1024	sgCYP2R1_4	CYP2R1	chr11	14900704	-	GAGCGATTTCTGGACAGCAG
1025	sgCYP2R1_5	CYP2R1	chr11	14913603	+	CCGATAAATGGCAGCCCCGG
1026	sgCYP2R1_6	CYP2R1	chr11	14913560	+	GACATGGGGAAGCTCGGATG
1027	sgCYP2R1_7	CYP2R1	chr11	14901968	+	CAGGATGCCAATCCATGGAA
1028	sgCYP2R1_8	CYP2R1	chr11	14913732	+	GCTCTCCAAAGCTTCCACAT
1029	sgCYP2R1_9	CYP2R1	chr11	14913653	-	TCCGCCAGCTGCTGAAGCAG
1030	sgCYP2R1_10	CYP2R1	chr11	14901989	+	GGCATTATACAAGAAGACTG
1031	sgCYP2S1_1	CYP2S1	chr19	41700494	-	AGGACCACCACAGGCCGCCA
1032	sgCYP2S1_2	CYP2S1	chr19	41700593	+	GAAGGGACTTTTGATGGCCA
1033	sgCYP2S1_3	CYP2S1	chr19	41703775	+	CGAGGAGCTGATCCAGGCGG
1034	sgCYP2S1_4	CYP2S1	chr19	41704384	-	GCCTGGGCCAGCAGCAGGGA
1035	sgCYP2S1_5	CYP2S1	chr19	41704406	-	GGAGCAGACTACGTTGGAGG

1036	sgCYP2S1_6	CYP2S1	chr19	41704440	-	TATCCTCATAGGAGAAGCGG
1037	sgCYP2S1_7	CYP2S1	chr19	41707230	-	CAGGAGGGTATAGCCGACCG
1038	sgCYP2S1_8	CYP2S1	chr19	41707249	-	GAGGGTATTTCATCAGGAGC
1039	sgCYP2S1_9	CYP2S1	chr19	41709428	-	GAACCGCGTCGGTGTAAGGG
1040	sgCYP2S1_10	CYP2S1	chr19	41709514	-	GGTGTACCCTCGGAAGCGGG
1041	sgCYP2U1_1	CYP2U1	chr4	108853108	-	GGTGAGCCAGGAGCACCTGC
1042	sgCYP2U1_2	CYP2U1	chr4	108853134	-	GAAGATGCTGCCGTACACGC
1043	sgCYP2U1_3	CYP2U1	chr4	108853253	-	ATGGAGATGAGCGGCACCCG
1044	sgCYP2U1_4	CYP2U1	chr4	108866318	-	GGAGCAAATGATGTTAGAGA
1045	sgCYP2U1_5	CYP2U1	chr4	108866458	-	AAGGGAAGGTAATAAAGCCA
1046	sgCYP2U1_6	CYP2U1	chr4	108870576	+	AGCCATTTGGGAGAAACCGG
1047	sgCYP2U1_7	CYP2U1	chr4	108852936	+	TGGGCTGGAGCTGGCTGCGG
1048	sgCYP2U1_8	CYP2U1	chr4	108852996	-	GAAGTTGCCCACCAGAGGCC
1049	sgCYP2U1_9	CYP2U1	chr4	108853162	+	TATCGGCCACTACCTGGTGG
1050	sgCYP2U1_10	CYP2U1	chr4	108853189	-	GCACGCTGTGGAAGTCGCTG
1051	sgCYP2W1_1	CYP2W1	chr7	1024178	-	GATGAGCTGGAAGATGGCGA
1052	sgCYP2W1_2	CYP2W1	chr7	1024853	-	GCGGCCGAAGAGGAGCGCGA
1053	sgCYP2W1_3	CYP2W1	chr7	1024104	+	GGTGCTGACGGGGTTCGAGG
1054	sgCYP2W1_4	CYP2W1	chr7	1024119	+	CGAGGCGGTCAAAGAGGCGC
1055	sgCYP2W1_5	CYP2W1	chr7	1024714	+	GGGCAGCTGGATGGCTACAG
1056	sgCYP2W1_6	CYP2W1	chr7	1024893	-	CCAGCAGGGACACAAACACG
1057	sgCYP2W1_7	CYP2W1	chr7	1026316	-	GGACCTCCTCGATCTTGCGC
1058	sgCYP2W1_8	CYP2W1	chr7	1026337	-	GGAGGGTCCTCAGAATGGCA
1059	sgCYP2W1_9	CYP2W1	chr7	1026763	+	GTTTGCTGAGGCCAACGCGG
1060	sgCYP2W1_10	CYP2W1	chr7	1026796	+	GGACATGGTCATGGCCGGGA
1061	sgCYP39A1_1	CYP39A1	chr6	46607301	+	TCAGTTGCCCAGTAAACTGA
1062	sgCYP39A1_2	CYP39A1	chr6	46620211	+	GAATCCAGCCCTTGATGCAC
1063	sgCYP39A1_3	CYP39A1	chr6	46563735	-	TAGAAAAGTGGTGAAGCCTG
1064	sgCYP39A1_4	CYP39A1	chr6	46620256	+	GAAGGAGTAAGAACAGAGCA
1065	sgCYP39A1_5	CYP39A1	chr6	46620189	+	TCAAATCCAACTCCAATCCA
1066	sgCYP39A1_6	CYP39A1	chr6	46598789	-	GACATTATTGCAAGCTACGC
1067	sgCYP39A1_7	CYP39A1	chr6	46598774	-	TACGCTGGATATTGTAGAGA
1068	sgCYP39A1_8	CYP39A1	chr6	46555832	+	AACATCAACAAGTCACCAGA
1069	sgCYP39A1_9	CYP39A1	chr6	46555772	+	GGTTTGAACAATTCAGGCTC
1070	sgCYP39A1_10	CYP39A1	chr6	46554897	-	TTTTCTTCCCACAGGAACGT
1071	sgCYP3A4_1	CYP3A4	chr7	99359653	+	GGGCCTTGTACCTTTCAGGG
1072	sgCYP3A4_2	CYP3A4	chr7	99381680	-	TCTCATCCCAGACTTGGCCA
1073	sgCYP3A4_3	CYP3A4	chr7	99370287	-	TGATGGTCAACAGCCTGTGC
1074	sgCYP3A4_4	CYP3A4	chr7	99375685	-	GGGCTTTTGTATGTTTGACA
1075	sgCYP3A4_5	CYP3A4	chr7	99361618	-	TCTGTCCGATCTGGAGCTCG
1076	sgCYP3A4_6	CYP3A4	chr7	99367748	+	CTTGAGTTTTCCACTGGTGA
1077	sgCYP3A4_7	CYP3A4	chr7	99358588	-	CAGATTCAGCAAGAAGAACA
1078	sgCYP3A4_8	CYP3A4	chr7	99367846	-	ACTCTAGCCTTTTGGTCCAG
1079	sgCYP3A4_9	CYP3A4	chr7	99367836	+	ATAAATCCCACTGGACCAAA
1080	sgCYP3A4_10	CYP3A4	chr7	99364851	+	TCAAGAATTGGGATGAGGAA
1081	sgCYP3A43_1	CYP3A43	chr7	99441788	-	GGATCCATGATGACCAGCAT
1082	sgCYP3A43_2	CYP3A43	chr7	99441772	+	TGAGGGGCAACAGCCCATGC
1083	sgCYP3A43_3	CYP3A43	chr7	99445817	+	ATATGTTGGTGAGAAGCCTG
1084	sgCYP3A43_4	CYP3A43	chr7	99434132	-	AGTTCCCAGAAAAGGCAGAG
1085	sgCYP3A43_5	CYP3A43	chr7	99447183	-	AGTGATTACATCCATGGTGT
1086	sgCYP3A43_6	CYP3A43	chr7	99457525	-	ATACATAATGAAGGGGAGAG
1087	sgCYP3A43_7	CYP3A43	chr7	99459359	+	AATGGAGTGTTCATTCCCAA
1088	sgCYP3A43_8	CYP3A43	chr7	99425774	+	GGTTCTTGTGGCTACCAGCC
1089	sgCYP3A43_9	CYP3A43	chr7	99434122	-	AAAGGCAGAGGGGTTGGCCC
1090	sgCYP3A43_10	CYP3A43	chr7	99445123	-	GCACTTTTCAGAAATCCCAT
1091	sgCYP3A5_1	CYP3A5	chr7	99273754	+	ACATTTCCCAACAAAGGCAG
1092	sgCYP3A5_2	CYP3A5	chr7	99270251	+	GATCACGTCGGGATCTGTGA
1093	sgCYP3A5_3	CYP3A5	chr7	99270239	+	TAGCACTGTTCTGATCACGT
1094	sgCYP3A5_4	CYP3A5	chr7	99273726	+	CAAGCAACTCACCTGACGAT
1095	sgCYP3A5_5	CYP3A5	chr7	99270294	-	TCCCCACACAGAACGTATGA
1096	sgCYP3A5_6	CYP3A5	chr7	99273786	-	TTAAGAGACTGGGAATTCCA

1097	sgCYP3A5_7	CYP3A5	chr7	99270277	-	TGAAGGTCAACTCCCTGTGC
1098	sgCYP3A5_8	CYP3A5	chr7	99270262	+	GATCTGTGATGGCCAGCACA
1099	sgCYP3A5_9	CYP3A5	chr7	99264262	-	TATTGGTGAGAAACTTGAGG
1100	sgCYP3A5_10	CYP3A5	chr7	99258200	+	GAAGGAAAGAACACTGCTGG
1101	sgCYP3A7_1	CYP3A7	chr7	99306647	+	GGGCCTCCTACCTTTCAGGG
1102	sgCYP3A7_2	CYP3A7	chr7	99311094	-	AAAGACTCTGAGACCCACAA
1103	sgCYP3A7_3	CYP3A7	chr7	99317994	+	GGATCTGTGATAGCCAGCAT
1104	sgCYP3A7_4	CYP3A7	chr7	99332653	+	GGAGTATCAGGCTGACAGCC
1105	sgCYP3A7_5	CYP3A7	chr7	99332692	-	TCTCATCCCAAACTTGGCCG
1106	sgCYP3A7_6	CYP3A7	chr7	99319953	-	GGGCTATTGGACGTTTGACA
1107	sgCYP3A7_7	CYP3A7	chr7	99313386	+	GAGAGAACGAATGGATCTAA
1108	sgCYP3A7_8	CYP3A7	chr7	99308384	-	TGTCCAGCAGAAAGTGCAGA
1109	sgCYP3A7_9	CYP3A7	chr7	99318010	-	TGACTGTCAACAGCCTATGC
1110	sgCYP3A7_10	CYP3A7	chr7	99315240	+	ATAAATCCCACTGGCCCGAA
1111	sgCYP46A1_1	CYP46A1	chr14	100192563	-	GCTGCCCGATGCAGGAGCGG
1112	sgCYP46A1_2	CYP46A1	chr14	100150801	-	GAAGGTGCAGCAGAGGCCGA
1113	sgCYP46A1_3	CYP46A1	chr14	100150819	-	GCTGCGAGCGCGGTGCACGA
1114	sgCYP46A1_4	CYP46A1	chr14	100166387	+	ATGAGCGCTGGCACAAGCAG
1115	sgCYP46A1_5	CYP46A1	chr14	100173032	+	GGTGGAGATTCTAGAAGCCA
1116	sgCYP46A1_6	CYP46A1	chr14	100182478	+	AGAGGGAGCCCAGGACGACG
1117	sgCYP46A1_7	CYP46A1	chr14	100191729	+	TCAGCACCTATGTCATGGGG
1118	sgCYP46A1_8	CYP46A1	chr14	100150837	+	GCCGCTACGAGCACATCCCC
1119	sgCYP46A1_9	CYP46A1	chr14	100165811	+	GATGTCAACCAAGTACAACA
1120	sgCYP46A1_10	CYP46A1	chr14	100166403	+	GCAGCGGAGAGTCATAGACC
1121	sgCYP4A11_1	CYP4A11	chr1	47407051	+	GGATTCCAGAGACATCACCC
1122	sgCYP4A11_2	CYP4A11	chr1	47398657	-	AAAAGTGTGGCCCAACCCAG
1123	sgCYP4A11_3	CYP4A11	chr1	47403770	-	ACAACGGATTCAGAAATGGG
1124	sgCYP4A11_4	CYP4A11	chr1	47398478	+	GAACCCGGTGCAAAACGGAA
1125	sgCYP4A11_5	CYP4A11	chr1	47403730	-	TGTCCTCATTGGCTATGGGG
1126	sgCYP4A11_6	CYP4A11	chr1	47406970	+	TGAGCAGCCACTGCCTGTGC
1127	sgCYP4A11_7	CYP4A11	chr1	47402374	+	AGGGCTTCAGGATGTCATAG
1128	sgCYP4A11_8	CYP4A11	chr1	47400751	+	GTGAAAGGCATTCCTCACAC
1129	sgCYP4A11_9	CYP4A11	chr1	47399873	-	GGAGGAGATCCACAGCCTCC
1130	sgCYP4A11_10	CYP4A11	chr1	47407072	-	GCTGAGCCCCAGCAGACTCC
1131	sgCYP4A22_1	CYP4A22	chr1	47603193	-	GGATCCCGGAGACACCACCC
1132	sgCYP4A22_2	CYP4A22	chr1	47611761	-	GAACCCGGTGCAAAACGGGA
1133	sgCYP4A22_3	CYP4A22	chr1	47607854	+	GACATCCTGAAGCCATACGT
1134	sgCYP4A22_4	CYP4A22	chr1	47603176	+	AGCCCCAGCAGACGCCTGGG
1135	sgCYP4A22_5	CYP4A22	chr1	47606512	+	TGTCCTTATTGGATATGGGG
1136	sgCYP4A22_6	CYP4A22	chr1	47607281	-	TTTGCACACATACCAATCCG
1137	sgCYP4A22_7	CYP4A22	chr1	47609434	+	GAATTCTCAGTCCTACATCC
1138	sgCYP4A22_8	CYP4A22	chr1	47611581	+	AAAAGTGTGGCCCAACCTAG
1139	sgCYP4A22_9	CYP4A22	chr1	47611594	-	GGACCACATACCTCTAGGTT
1140	sgCYP4A22_10	CYP4A22	chr1	47603264	+	AGCTCTACCTGCATAGGCAG
1141	sgCYP4B1_1	CYP4B1	chr1	47279929	+	GGAAGAAGATCCAGAACCGG
1142	sgCYP4B1_2	CYP4B1	chr1	47264849	+	GCTGCTGCGGAGGCAGACGT
1143	sgCYP4B1_3	CYP4B1	chr1	47280831	-	GAGAAACCAGGAGATACCAC
1144	sgCYP4B1_4	CYP4B1	chr1	47282774	-	GGGGCACAGGTGGGTAGAGG
1145	sgCYP4B1_5	CYP4B1	chr1	47264755	-	GGAGAGGAAGCTGGGCACCA
1146	sgCYP4B1_6	CYP4B1	chr1	47276524	-	GGTGGGCATACGGGAACTGG
1147	sgCYP4B1_7	CYP4B1	chr1	47276597	+	GCCAAAGCTGTGTACAGCCG
1148	sgCYP4B1_8	CYP4B1	chr1	47276824	-	GAAGAAGTCATACACATCAG
1149	sgCYP4B1_9	CYP4B1	chr1	47279156	+	CAAGTGGGAAGAGAAAGCTC
1150	sgCYP4B1_10	CYP4B1	chr1	47279957	-	GGAGAATGTCCAGGAAGTCC
1151	sgCYP4F11_1	CYP4F11	chr19	16045062	+	GAGGAAAACACTGGAGGCGG
1152	sgCYP4F11_2	CYP4F11	chr19	16040354	-	GCTGGTGACCACATATCCCC
1153	sgCYP4F11_3	CYP4F11	chr19	16040299	+	GGGTGGCATAAAATGAGGAG
1154	sgCYP4F11_4	CYP4F11	chr19	16040282	+	TAGGCCGGATAATGTCAGGG
1155	sgCYP4F11_5	CYP4F11	chr19	16038128	-	GATGGGCTCCTGCTGAGTGG
1156	sgCYP4F11_6	CYP4F11	chr19	16045084	+	GCAGTTGTCATAGAAGGTGT
1157	sgCYP4F11_7	CYP4F11	chr19	16040333	+	CCAGCCACAACTTAAAGCCC

1158	sgCYP4F11_8	CYP4F11	chr19	16045101	+	TGTAGGTCCAGGCCAGGACG
1159	sgCYP4F11_9	CYP4F11	chr19	16038227	+	CTCAATTACTCACCCAGCCA
1160	sgCYP4F11_10	CYP4F11	chr19	16038084	+	AGGCAGGCGTCAACATCCGA
1161	sgCYP4F12_1	CYP4F12	chr19	15784360	+	CTGGCTGGGCCTCAGACCGG
1162	sgCYP4F12_2	CYP4F12	chr19	15784477	-	GTGGGAAACACTGGAGCCGG
1163	sgCYP4F12_3	CYP4F12	chr19	15789067	+	TCAGATCACTCCTACAGAGG
1164	sgCYP4F12_4	CYP4F12	chr19	15789110	+	ATGTCGGCCACCTATTCCCA
1165	sgCYP4F12_5	CYP4F12	chr19	15795908	+	GGGTCCTGTACAACCTTGCG
1166	sgCYP4F12_6	CYP4F12	chr19	15807262	+	ACCCAGAGAACAGCAAGGGG
1167	sgCYP4F12_7	CYP4F12	chr19	15784374	+	GACCGGTGGCAACGTCCCCA
1168	sgCYP4F12_8	CYP4F12	chr19	15789094	+	GAAGAACTCGACCCAGATGT
1169	sgCYP4F12_9	CYP4F12	chr19	15789181	-	TAGACCGGATGGTGTCAGGG
1170	sgCYP4F12_10	CYP4F12	chr19	15806984	-	GGACCCCTATAATATCGATG
1171	sgCYP4F2_1	CYP4F2	chr19	16008259	+	GGGGTTGTGGGAAACACCGA
1172	sgCYP4F2_2	CYP4F2	chr19	16006348	+	GGGTGGCACAAACTGAGGAG
1173	sgCYP4F2_3	CYP4F2	chr19	16008370	+	GGGATGCTGCCACTGGCCAG
1174	sgCYP4F2_4	CYP4F2	chr19	16008287	+	GCAGTTGTCATAGAAGGCGT
1175	sgCYP4F2_5	CYP4F2	chr19	16006445	-	GCAGGTCAACCCCACAGAGG
1176	sgCYP4F2_6	CYP4F2	chr19	16000412	+	ACAGGAAGTCAATATGCAGG
1177	sgCYP4F2_7	CYP4F2	chr19	16008337	+	AGGAGGCCCCGACCAGCAGG
1178	sgCYP4F2_8	CYP4F2	chr19	16008226	-	GTTTTGGGGACACCAGGGCA
1179	sgCYP4F2_9	CYP4F2	chr19	16006331	+	CAGACCGGATGATGTCGGGG
1180	sgCYP4F2_10	CYP4F2	chr19	15996762	-	GGAGGTGCAAGAACTTCTGA
1181	sgCYP4F22_1	CYP4F22	chr19	15640613	-	GGGTGCACCAGAACCAACAG
1182	sgCYP4F22_2	CYP4F22	chr19	15658983	-	CGAGAGACAAGAGTGACAGG
1183	sgCYP4F22_3	CYP4F22	chr19	15661536	-	GAGAAGGGCACATAGGCCAG
1184	sgCYP4F22_4	CYP4F22	chr19	15636224	-	GAGCAGGAAGAGGAGAAGGG
1185	sgCYP4F22_5	CYP4F22	chr19	15640516	+	GCAGTACCTTCCAAATGAGG
1186	sgCYP4F22_6	CYP4F22	chr19	15648219	-	ACTGGGCACTCACCTAGCCA
1187	sgCYP4F22_7	CYP4F22	chr19	15651315	-	GGTGCAAGCGATACTGGCGC
1188	sgCYP4F22_8	CYP4F22	chr19	15651419	+	AAGTCATCCAGGAACGGCGG
1189	sgCYP4F22_9	CYP4F22	chr19	15654960	+	GGTCACGACACAACATCCAG
1190	sgCYP4F22_10	CYP4F22	chr19	15658994	+	TGTCTCTCGCCAATGCACGG
1191	sgCYP4F3_1	CYP4F3	chr19	15760083	-	CAAGAACTTACTCCTGGCAA
1192	sgCYP4F3_2	CYP4F3	chr19	15760795	-	ACAGGAAGTCTATGTACAGG
1193	sgCYP4F3_3	CYP4F3	chr19	15769147	-	GTGCAGCAGCGAGAGACGGC
1194	sgCYP4F3_4	CYP4F3	chr19	15752258	-	GGGATGCTGCCATTGGCCAA
1195	sgCYP4F3_5	CYP4F3	chr19	15752231	+	ACAGCTGAGCCTGTCCTCGC
1196	sgCYP4F3_6	CYP4F3	chr19	15752341	-	GCAGTTGTCATAGAAGGTAT
1197	sgCYP4F3_7	CYP4F3	chr19	15759992	-	CAGACGGGCACTACCCTCTG
1198	sgCYP4F3_8	CYP4F3	chr19	15763697	+	CCAGGAGCGCTGTCGGCAGG
1199	sgCYP4F3_9	CYP4F3	chr19	15752243	-	GCCAAAGGCCCAGCGAGGAC
1200	sgCYP4F3_10	CYP4F3	chr19	15769345	-	TCAGGGTCCGGCCACACGGC
1201	sgCYP4V2_1	CYP4V2	chr4	187113115	+	GATGCGGCCCATCCCCACGG
1202	sgCYP4V2_2	CYP4V2	chr4	187115664	+	GCAGATCATTGAGTACACAG
1203	sgCYP4V2_3	CYP4V2	chr4	187113054	-	GCTCAGGACCAGACTGGCGC
1204	sgCYP4V2_4	CYP4V2	chr4	187115704	-	CCGACCCAGAGCTTCAGCAG
1205	sgCYP4V2_5	CYP4V2	chr4	187130311	+	GAGATACTTCCCCAACCCCG
1206	sgCYP4V2_6	CYP4V2	chr4	187130329	-	GCTCAGGCTGGAACTCCTCG
1207	sgCYP4V2_7	CYP4V2	chr4	187113151	-	GCAGCGCGTGGCCCACCAGT
1208	sgCYP4V2_8	CYP4V2	chr4	187117217	-	AGAAGTCCTAGGCCAAGCCA
1209	sgCYP4V2_9	CYP4V2	chr4	187120148	-	CAGAGATCAAGCCAAAGCCA
1210	sgCYP4V2_10	CYP4V2	chr4	187113010	+	GAAGCTGCTGCTGTGGGGCG
1211	sgCYP4X1_1	CYP4X1	chr1	47495723	-	GGGCCCAATCCAGAAAGGGA
1212	sgCYP4X1_2	CYP4X1	chr1	47497143	-	ACATACATACCAAGAAGTGG
1213	sgCYP4X1_3	CYP4X1	chr1	47504345	+	GGAAAGAAAGAAATCCCTCC
1214	sgCYP4X1_4	CYP4X1	chr1	47505142	+	GCATCAAGAGAGATGCCGGG
1215	sgCYP4X1_5	CYP4X1	chr1	47489575	+	CCATTAAGCTGTACCTGCGG
1216	sgCYP4X1_6	CYP4X1	chr1	47489641	-	GTGCCCAAGGAACCAGTGGG
1217	sgCYP4X1_7	CYP4X1	chr1	47497127	-	GTGGAGGTGAGAATTTCTGC
1218	sgCYP4X1_8	CYP4X1	chr1	47498995	+	GAAAGCATACATTGAGGTGA

1219	sgCYP4X1_9	CYP4X1	chr1	47501822	+	CGAGTGTTGAATCAGTACAC
1220	sgCYP4X1_10	CYP4X1	chr1	47515144	-	AGAATGGTAAGTAGGCATAG
1221	sgCYP4Z1_1	CYP4Z1	chr1	47533291	-	GGGGTGCAGGAAACAGGTGC
1222	sgCYP4Z1_2	CYP4Z1	chr1	47533311	-	GCCATAGAACCAGTGGGCAG
1223	sgCYP4Z1_3	CYP4Z1	chr1	47560289	+	ATACTACTCAGAAAAGGCGC
1224	sgCYP4Z1_4	CYP4Z1	chr1	47534314	+	TGAGGTGTATCATAAGCTGA
1225	sgCYP4Z1_5	CYP4Z1	chr1	47534376	-	GACACTGAAGAACATCGTAA
1226	sgCYP4Z1_6	CYP4Z1	chr1	47564851	-	AAGGATCCAGGAGATAGCAC
1227	sgCYP4Z1_7	CYP4Z1	chr1	47571829	-	GCATTCCTTGATGCACATCG
1228	sgCYP4Z1_8	CYP4Z1	chr1	47564870	-	GGTACTTTGCCAAGCAGTAA
1229	sgCYP4Z1_9	CYP4Z1	chr1	47564912	-	GGAGTTCCCTGATTTCATCT
1230	sgCYP4Z1_10	CYP4Z1	chr1	47571901	-	GCGTCCATCTGGAAAGGTGA
1231	sgCYP51A1_1	CYP51A1	chr7	91755561	+	CCATACCTGAAACTAGGCAA
1232	sgCYP51A1_2	CYP51A1	chr7	91752436	-	GCCTCCTTTAACTTATGACC
1233	sgCYP51A1_3	CYP51A1	chr7	91747836	-	GAGAATGGCCAGAACTCCTC
1234	sgCYP51A1_4	CYP51A1	chr7	91752534	-	TCAACTACTAGTGCTTGGAT
1235	sgCYP51A1_5	CYP51A1	chr7	91747825	+	GATACTCACCTGAGGAGTTC
1236	sgCYP51A1_6	CYP51A1	chr7	91763669	-	GACGGAGTGAATGGCGGCGG
1237	sgCYP51A1_7	CYP51A1	chr7	91763476	+	AATGTACGTACCACCCCTGC
1238	sgCYP51A1_8	CYP51A1	chr7	91761158	+	ATTGGGGAGAAAATGTATGG
1239	sgCYP51A1_9	CYP51A1	chr7	91763639	-	GCTGCTGCTGGGCTTGCTGC
1240	sgCYP51A1_10	CYP51A1	chr7	91761140	+	GCATGCCCAAGGAATGGAAT
1241	sgCYP7A1_1	CYP7A1	chr8	59409722	-	GCACAGAAGCATTGACCCGA
1242	sgCYP7A1_2	CYP7A1	chr8	59409230	-	GAAGGCCAAGACACACCTCG
1243	sgCYP7A1_3	CYP7A1	chr8	59409212	+	GCGATGCCCAGAGGACCACG
1244	sgCYP7A1_4	CYP7A1	chr8	59405065	-	GTATAATCAAGGAATCGCTG
1245	sgCYP7A1_5	CYP7A1	chr8	59410964	+	CAAATTGCAGAGCACAGCCC
1246	sgCYP7A1_6	CYP7A1	chr8	59409586	+	TTTGAGTTAGAGGAGACTGG
1247	sgCYP7A1_7	CYP7A1	chr8	59409400	+	GGGAGGCCTGCTACCAGGGC
1248	sgCYP7A1_8	CYP7A1	chr8	59409368	-	CAGGACTGCGCACAATGCCC
1249	sgCYP7A1_9	CYP7A1	chr8	59409324	-	GGCACGAGAACCTCCAAAAG
1250	sgCYP7A1_10	CYP7A1	chr8	59409275	+	TGTCATTGAGAAACATGCGC
1251	sgCYP7B1_1	CYP7B1	chr8	65711027	+	TGCGCCGGACAAGCAAGCAG
1252	sgCYP7B1_2	CYP7B1	chr8	65711012	+	GAGGCGCTTACCTGGTGCGC
1253	sgCYP7B1_3	CYP7B1	chr8	65528321	-	AGTTAGCCAAGATGCAAGGA
1254	sgCYP7B1_4	CYP7B1	chr8	65527697	-	TCTGCGGCACCCAGAAGCTA
1255	sgCYP7B1_5	CYP7B1	chr8	65517261	+	GGGTCACCATGTAGGACTGG
1256	sgCYP7B1_6	CYP7B1	chr8	65711126	-	GATGGCAGGAGAAGTGTCCG
1257	sgCYP7B1_7	CYP7B1	chr8	65711100	+	CTCCAGCGAAAAGCGGCCCG
1258	sgCYP7B1_8	CYP7B1	chr8	65711043	+	GCAGAGGGCCAGGAGCAGCA
1259	sgCYP7B1_9	CYP7B1	chr8	65517350	+	CTCCTCAACAAAACGAATGG
1260	sgCYP7B1_10	CYP7B1	chr8	65517325	-	GACTCTCAGTTCAGAGACCG
1261	sgCYP8B1_1	CYP8B1	chr3	42917171	+	GGCATGGCCAAGCCAGGGCA
1262	sgCYP8B1_2	CYP8B1	chr3	42916741	-	GAGCTTGTTCGGCTACACGA
1263	sgCYP8B1_3	CYP8B1	chr3	42916591	+	GCATCTTGTGAAAGAGACGC
1264	sgCYP8B1_4	CYP8B1	chr3	42916567	-	CGTGAGCCACAGCCAGGAGA
1265	sgCYP8B1_5	CYP8B1	chr3	42916484	+	GAACTTGTCCTGCATAGCTG
1266	sgCYP8B1_6	CYP8B1	chr3	42916422	+	AAGAGGGCCCAGAAAGAGGT
1267	sgCYP8B1_7	CYP8B1	chr3	42916265	-	GCGTGGTGGAGGAGACGCTG
1268	sgCYP8B1_8	CYP8B1	chr3	42916085	+	GAAGCGATCGTACTTGAAGA
1269	sgCYP8B1_9	CYP8B1	chr3	42915967	+	TGAGTGCAAAGAACCTCCCA
1270	sgCYP8B1_10	CYP8B1	chr3	42917233	+	GGAGCATCCCTGGCAGGCAC
1271	sgDDX11_1	DDX11	chr12	31236778	-	AAAGTCACGGAGCCAAGAGA
1272	sgDDX11_2	DDX11	chr12	31242349	+	AATGAAGACGTGAAAAGCCT
1273	sgDDX11_3	DDX11	chr12	31244705	-	GTCCTGCAGCCGGATGCCCG
1274	sgDDX11_4	DDX11	chr12	31253994	-	GATCCCGCTGCAGATGACGA
1275	sgDDX11_5	DDX11	chr12	31249833	-	TCAGTGGAGAAGCTGGTCGC
1276	sgDDX11_6	DDX11	chr12	31256617	-	AGGTAGCTTTGACCTCCACA
1277	sgDDX11_7	DDX11	chr12	31236974	+	GGACCTGGTGGACCGACTAA
1278	sgDDX11_8	DDX11	chr12	31249863	-	TGAGAGCTGCCAGGAAGCCT
1279	sgDDX11_9	DDX11	chr12	31256544	-	CTGGTCCAGGAGCACTACGC

1280	sgDDX11_10	DDX11	chr12	31256602	+	CTGGATCCGAGCCCGTGTGG
1281	sgDGCR8_1	DGCR8	chr22	20073604	-	GCACCACTGGACGTTTGCAG
1282	sgDGCR8_2	DGCR8	chr22	20073725	-	GAGGAGGCGGCCCTTAGAGA
1283	sgDGCR8_3	DGCR8	chr22	20077511	-	CAAGGGATGCTACTCAGAGG
1284	sgDGCR8_4	DGCR8	chr22	20074117	+	CTAGATGAAGAAGGAGCAGG
1285	sgDGCR8_5	DGCR8	chr22	20074200	-	GCTGCCAACATACCTCGTAG
1286	sgDGCR8_6	DGCR8	chr22	20077202	-	GCAGCGGCTCTGTAGGTGGG
1287	sgDGCR8_7	DGCR8	chr22	20077268	+	CCTACACAGAGAGTCTCGGG
1288	sgDGCR8_8	DGCR8	chr22	20077649	-	ATAGAGTCAGGCTCATCCAG
1289	sgDGCR8_9	DGCR8	chr22	20077738	+	GCAGGTGAAGGCCAAAGTCG
1290	sgDGCR8_10	DGCR8	chr22	20079024	+	TCAGGACTTGGGCTGAGCGG
1291	sgDOHH_1	DOHH	chr19	3492442	-	GAGTGGCTGCAGCAGCACGG
1292	sgDOHH_2	DOHH	chr19	3496794	-	GATGGTGACGGAGCAGGAGG
1293	sgDOHH_3	DOHH	chr19	3496784	-	GAGCAGGAGGTGGATGCCAT
1294	sgDOHH_4	DOHH	chr19	3496765	+	GTCCACCAGCGTCTGCCCGA
1295	sgDOHH_5	DOHH	chr19	3496675	+	GGCCTGGCTGATCCATGCAA
1296	sgDOHH_6	DOHH	chr19	3496578	+	GCAGCACGTCCACCAGCATG
1297	sgDOHH_7	DOHH	chr19	3492400	+	GCAGGGTCCACGGAGAGGTA
1298	sgDOHH_8	DOHH	chr19	3492376	+	ACGTCACGCTCCTCAGCCGG
1299	sgDOHH_9	DOHH	chr19	3492316	+	GCGCGGTATCGCTCGAAGAG
1300	sgDOHH_10	DOHH	chr19	3496749	-	GGACCCCAAGCAGCCCCTGC
1301	sgDPH1_1	DPH1	chr17	1943831	-	GCAGCAGCCTCACAAAGGAA
1302	sgDPH1_2	DPH1	chr17	1936920	+	CTGGAGGATCCAACAAGCCC
1303	sgDPH1_3	DPH1	chr17	1939318	+	TGTGGATGACTTCACAGCGA
1304	sgDPH1_4	DPH1	chr17	1940193	-	GCAGCCCAGGATCTCTCCAG
1305	sgDPH1_5	DPH1	chr17	1943067	-	TGACAGACTCCAGATGGAAG
1306	sgDPH1_6	DPH1	chr17	1943595	-	GGACTTAGCTGAGCGGGCAG
1307	sgDPH1_7	DPH1	chr17	1943867	-	GAAGTAGGCTAAGCTTGCTG
1308	sgDPH1_8	DPH1	chr17	1944782	-	GGAAATGTCCCTCAGAGCCA
1309	sgDPH1_9	DPH1	chr17	1936809	-	GATTGGCCACGCGGCCCCGA
1310	sgDPH1_10	DPH1	chr17	1936904	+	AGATCCCCAAGACCATCTGG
1311	sgDPH2_1	DPH2	chr1	44435856	+	GATGTTTAGCAGCCCTGCCG
1312	sgDPH2_2	DPH2	chr1	44437843	-	CAGGTGGTGGGAGAGCCACG
1313	sgDPH2_3	DPH2	chr1	44435880	+	GGCGCTGCAGCGAGAGACCG
1314	sgDPH2_4	DPH2	chr1	44436288	+	CCAGCTATTGGGAGATGCTG
1315	sgDPH2_5	DPH2	chr1	44436359	+	TGGGTGACACAGCCTACGGC
1316	sgDPH2_6	DPH2	chr1	44436854	-	AAAACCCCTTACCCAGGGCA
1317	sgDPH2_7	DPH2	chr1	44437950	+	GAAGCTTGGCTCTGACCCCA
1318	sgDPH2_8	DPH2	chr1	44436742	-	AGAACGTTGACGAAGCACGA
1319	sgDPH2_9	DPH2	chr1	44435908	-	GGAAGAGGAGTAAGCAGTCC
1320	sgDPH2_10	DPH2	chr1	44435931	+	CCTGGACGGAGTGTACGAGC
1321	sgDPYD_1	DPYD	chr1	98386452	+	CGAGTCCTTACTGAGCACAG
1322	sgDPYD_2	DPYD	chr1	98187132	+	TCCACCTACACAAAGATCAG
1323	sgDPYD_3	DPYD	chr1	98187091	-	GAGGGACCCATTAATATTGG
1324	sgDPYD_4	DPYD	chr1	98348903	+	GTGTTCGAGGATTTAAAGCC
1325	sgDPYD_5	DPYD	chr1	98293672	-	GAGCTCTCCGAGAAGCAATG
1326	sgDPYD_6	DPYD	chr1	98386442	-	TAAGGACTCGGCGGACATCG
1327	sgDPYD_7	DPYD	chr1	98293703	-	GACATCAAGCACACGACTCT
1328	sgDPYD_8	DPYD	chr1	98205993	+	TGGACAGCTCTTCTGACACG
1329	sgDPYD_9	DPYD	chr1	98187167	-	ACTTGGTCTGACTTGTGGAA
1330	sgDPYD_10	DPYD	chr1	98348859	+	GTCTAATTTCTTGGCCGAAG
1331	sgDUOX1_1	DUOX1	chr15	45434284	-	GAAGCAACAGAGGGTCCCGA
1332	sgDUOX1_2	DUOX1	chr15	45444667	-	GAGGCGATGGAAGTCCACGG
1333	sgDUOX1_3	DUOX1	chr15	45427462	+	ACGGAGTCCCAGCAATCCCC
1334	sgDUOX1_4	DUOX1	chr15	45427738	+	GCGCTGCGGAGCTTCTCCAG
1335	sgDUOX1_5	DUOX1	chr15	45433173	-	ACAGAGGTCCAGGGTCCCGG
1336	sgDUOX1_6	DUOX1	chr15	45436268	-	GCAGGTACACAAGCACGGGC
1337	sgDUOX1_7	DUOX1	chr15	45436286	+	GCAGCCCGGGCAGATCCGTG
1338	sgDUOX1_8	DUOX1	chr15	45437172	+	GCATCCAGGAGTGGGAGCTG
1339	sgDUOX1_9	DUOX1	chr15	45444565	-	GGAGAACATGAAAGAGATGC
1340	sgDUOX1_10	DUOX1	chr15	45444680	-	CGGTGGAGGCAATGAGGCGA

1341	sgDUOX2_1	DUOX2	chr15	45392315	-	GCTTCGTGGAGAACTACCGG
1342	sgDUOX2_2	DUOX2	chr15	45405524	+	GGAGCCTGATACTTGCCCGA
1343	sgDUOX2_3	DUOX2	chr15	45401740	-	GGAGTTGGAGGACAACATAG
1344	sgDUOX2_4	DUOX2	chr15	45401115	+	CTGTACGGGAGAATTTGCCA
1345	sgDUOX2_5	DUOX2	chr15	45400305	+	AGGACAATGGCACTGAACAG
1346	sgDUOX2_6	DUOX2	chr15	45399101	+	ACAGTCAGGGGTGCACACTG
1347	sgDUOX2_7	DUOX2	chr15	45399089	+	AAGAAGTCAAGCACAGTCAG
1348	sgDUOX2_8	DUOX2	chr15	45399043	+	GCAGAGAGCAATGATGGTGA
1349	sgDUOX2_9	DUOX2	chr15	45399018	+	CTAAGAGCTCACCTAAGGGA
1350	sgDUOX2_10	DUOX2	chr15	45397886	-	AGGCTGTGACAAAGCAGCAG
1351	sgEGLN1_1	EGLN1	chr1	231557618	-	GCCATGGCCAATGACAGCGG
1352	sgEGLN1_2	EGLN1	chr1	231557346	-	CCGGGAGCCCAGGAAGGCAG
1353	sgEGLN1_3	EGLN1	chr1	231557007	-	CAAGCACGGCATCTGTGTGG
1354	sgEGLN1_4	EGLN1	chr1	231556880	-	GACTCGTCCAAGGACATCCG
1355	sgEGLN1_5	EGLN1	chr1	231556974	+	GCTGTCCGGTCTCCTTGCCG
1356	sgEGLN1_6	EGLN1	chr1	231556753	-	GCAGCTACAAAATCAATGGC
1357	sgEGLN1_7	EGLN1	chr1	231509822	+	GCCCGGATAACAAGCAACCA
1358	sgEGLN1_8	EGLN1	chr1	231509728	-	TAATAAAGACTGGGATGCCA
1359	sgEGLN1_9	EGLN1	chr1	231557475	+	GCTTCTTCCAGTCCTGACGC
1360	sgEGLN1_10	EGLN1	chr1	231557048	+	TCGAGCGCCAGCTTCAGCGC
1361	sgEGLN2_1	EGLN2	chr19	41306691	+	ACTGCCAGCCCTCTTCGGGA
1362	sgEGLN2_2	EGLN2	chr19	41306748	-	GCAGCGCCTTCACTCTGCAG
1363	sgEGLN2_3	EGLN2	chr19	41306834	+	GAAATGGGCCGAGGATGGTG
1364	sgEGLN2_4	EGLN2	chr19	41306885	+	GGCCAGGCAAGAGAACCAGG
1365	sgEGLN2_5	EGLN2	chr19	41307102	+	GAGGTGGAGGCCCTCAAACG
1366	sgEGLN2_6	EGLN2	chr19	41307171	-	TCCCCACGGATGCTGCGCGG
1367	sgEGLN2_7	EGLN2	chr19	41312508	+	GTTGACAATCCCCACGGCGA
1368	sgEGLN2_8	EGLN2	chr19	41306564	+	TGGCCGGGCCAGGATGGGAG
1369	sgEGLN2_9	EGLN2	chr19	41306722	+	GGCAGGATGGTGGTGAGCTG
1370	sgEGLN2_10	EGLN2	chr19	41306897	+	GAACCAGGAGGCAGAGCGGG
1371	sgEGLN3_1	EGLN3	chr14	34419847	-	GGACAACTTCCTGGGCGAGG
1372	sgEGLN3_2	EGLN3	chr14	34419799	-	CAAGCAGCTGCACTGCACCG
1373	sgEGLN3_3	EGLN3	chr14	34419668	+	GAGGGACAGGAGGAAGCTGA
1374	sgEGLN3_4	EGLN3	chr14	34419611	-	GCAAATACTACGTCAAGGAG
1375	sgEGLN3_5	EGLN3	chr14	34400341	+	GATGCAGCGACCATCACCGT
1376	sgEGLN3_6	EGLN3	chr14	34419913	-	GGACCTGGAGAAAATTGCCC
1377	sgEGLN3_7	EGLN3	chr14	34419883	-	CGTGCCCTGTCTGCACGAGG
1378	sgEGLN3_8	EGLN3	chr14	34419650	+	GAGGACCAGCCTGTCGATGA
1379	sgEGLN3_9	EGLN3	chr14	34400415	-	GATTTCTCCATAGGCAATGG
1380	sgEGLN3_10	EGLN3	chr14	34400354	-	GTGGACAACCCCAACGGTGA
1381	sgEIF2AK1_1	EIF2AK1	chr7	6066457	+	GCACTGGACACCTTTTACGG
1382	sgEIF2AK1_2	EIF2AK1	chr7	6098675	-	GGTCCGCAAGCGCGAAGAGG
1383	sgEIF2AK1_3	EIF2AK1	chr7	6098623	+	GCGGGAAAGTCGATGGCCGG
1384	sgEIF2AK1_4	EIF2AK1	chr7	6094259	+	GTTTGCAACTGCAAAAGGGA
1385	sgEIF2AK1_5	EIF2AK1	chr7	6078222	-	GGATAGTCGAGAGAAACAAG
1386	sgEIF2AK1_6	EIF2AK1	chr7	6098662	-	GAAGAGGAGGGCGACGGGGC
1387	sgEIF2AK1_7	EIF2AK1	chr7	6094237	+	GCAAAGAAACCAGCAAGAGT
1388	sgEIF2AK1_8	EIF2AK1	chr7	6080654	-	GGAGCTCCAGGAAAATGGCT
1389	sgEIF2AK1_9	EIF2AK1	chr7	6084268	+	CCAGCACCTTCACTTCCCGT
1390	sgEIF2AK1_10	EIF2AK1	chr7	6082579	+	TCGGAGAGCACTTCCAGAGA
1391	sgELP3_1	ELP3	chr8	27967894	-	GGCACGGATAGCTCTCATGG
1392	sgELP3_2	ELP3	chr8	27987142	+	GCAGAGTGTTTATGAAGATG
1393	sgELP3_3	ELP3	chr8	27954793	+	TGATTGAAGCCCACGAGCAG
1394	sgELP3_4	ELP3	chr8	27957391	-	GCAATGATATCCACCAGGCG
1395	sgELP3_5	ELP3	chr8	27957460	+	AAACCCATCAGAACTGCTAG
1396	sgELP3_6	ELP3	chr8	27965419	+	TGTACTGGCAGATACTGCCC
1397	sgELP3_7	ELP3	chr8	27965471	-	ATAGCCAGTGTAAGACTGGG
1398	sgELP3_8	ELP3	chr8	27995380	-	CGGTACACTCGAGTCCATGG
1399	sgELP3_9	ELP3	chr8	27950724	+	GCGCAGAAATGAGGCAGAAG
1400	sgELP3_10	ELP3	chr8	27957421	-	AAGACCTTGCGATACTGAGG
1401	sgEML6_1	EML6	chr2	55176023	-	GGAGCTGCTCGGCTAACGGG

1402	sgEML6_2	EML6	chr2	55181168	-	GAAGCACCGCAGCATGGAGA
1403	sgEML6_3	EML6	chr2	55186364	+	GATGGACTCATAGTGACCGG
1404	sgEML6_4	EML6	chr2	55194183	+	GAAGCAGGTAACTGAAGCCG
1405	sgEML6_5	EML6	chr2	54952238	+	GAGTGGGTGTACGGGTACCG
1406	sgEML6_6	EML6	chr2	55044006	-	TACCCTGTCAGAATGGCCGG
1407	sgEML6_7	EML6	chr2	55054741	+	AGGGATATTTGGCAAAACAG
1408	sgEML6_8	EML6	chr2	55080865	+	AATTTGGCCCAAATACACTG
1409	sgEML6_9	EML6	chr2	55086741	+	GTGGGTGTTGAGCACAGGAG
1410	sgEML6_10	EML6	chr2	55093971	+	CAAACGAGAAAAGGCTCCTG
1411	sgEPX_1	EPX	chr17	56270402	-	GGTCTCCACTGCCCCAGGGG
1412	sgEPX_2	EPX	chr17	56270427	+	CCTGCGAGACTGCATAGCAG
1413	sgEPX_3	EPX	chr17	56270809	-	GGCCCGAACAACTGTCCTGG
1414	sgEPX_4	EPX	chr17	56270863	+	AAGAGAAGTTACAACCCCAG
1415	sgEPX_5	EPX	chr17	56272516	-	GTACCTTGATGGGAAAGCAG
1416	sgEPX_6	EPX	chr17	56274460	-	GTTGCGGAGCCGCAGCGAGA
1417	sgEPX_7	EPX	chr17	56274475	+	GCAACCGGACCAACTACCTG
1418	sgEPX_8	EPX	chr17	56276485	+	TGAGACGCCTGAATCCCCGG
1419	sgEPX_9	EPX	chr17	56276502	-	AGTTTGTCTCCATTCCACCG
1420	sgEPX_10	EPX	chr17	56277090	-	GACATGCGAGTTGGGTGCGG
1421	sgERCC2_1	ERCC2	chr19	45873402	-	GCGGGAGCTCAAACGCACGC
1422	sgERCC2_2	ERCC2	chr19	45867692	+	GGCCTCGTCGAAGACCACGA
1423	sgERCC2_3	ERCC2	chr19	45867549	-	TGAGCGTCAACCTCACCCGC
1424	sgERCC2_4	ERCC2	chr19	45867530	+	GGCACCGGTCAAGGGTCCGG
1425	sgERCC2_5	ERCC2	chr19	45867316	+	GCAGCCCCTCCACCAGACGC
1426	sgERCC2_6	ERCC2	chr19	45867262	+	GCAGCACGGGGTTGGCCAGG
1427	sgERCC2_7	ERCC2	chr19	45867110	+	GCAGCCGCCTCAGGAAGCCC
1428	sgERCC2_8	ERCC2	chr19	45864863	+	GAGTATGCAGCAGGGACCGG
1429	sgERCC2_9	ERCC2	chr19	45864803	+	GGGTGGCAAAGTTAGCAAGG
1430	sgERCC2_10	ERCC2	chr19	45872241	-	TTATCGGCAGGCATATCCGC
1431	sgETFDH_1	ETFDH	chr4	159601738	+	GATAAGGACAAGAGATGGGA
1432	sgETFDH_2	ETFDH	chr4	159627433	+	GGAGTACTGGGTGTATATGG
1433	sgETFDH_3	ETFDH	chr4	159627825	+	ATTGAGTATCCAAAACCCGA
1434	sgETFDH_4	ETFDH	chr4	159603379	+	GATGTTGTAATAGTTGGTGC
1435	sgETFDH_5	ETFDH	chr4	159611545	+	GATGTAGGGATACAAAAGGA
1436	sgETFDH_6	ETFDH	chr4	159620204	-	AGGTTGGCCGAATGCTAGGA
1437	sgETFDH_7	ETFDH	chr4	159620250	+	GGAGCCAGAGCTCTCAATGA
1438	sgETFDH_8	ETFDH	chr4	159627791	+	TGAACGGCTCAAGCCAGCCA
1439	sgETFDH_9	ETFDH	chr4	159627981	-	GCAGGACAGAATCGCTGCTC
1440	sgETFDH_10	ETFDH	chr4	159618774	+	TTGGACAGACATACCTATGG
1441	sgETHE1_1	ETHE1	chr19	44012195	-	AGGGTTCACAGTGTCCACCG
1442	sgETHE1_2	ETHE1	chr19	44030754	-	GGGTGACAGAGAGTCCCGGG
1443	sgETHE1_3	ETHE1	chr19	44015665	+	GACGAAGGTGACACAGCCTG
1444	sgETHE1_4	ETHE1	chr19	44030767	+	GTCACCCAGCAGGTACGTGA
1445	sgETHE1_5	ETHE1	chr19	44030424	+	TGACAGACTGGCAGCCAGGG
1446	sgETHE1_6	ETHE1	chr19	44015650	+	GCTGTGGTCATTCAGGACGA
1447	sgETHE1_7	ETHE1	chr19	44012178	+	AGTCCTCTCCTCCTCCACGG
1448	sgETHE1_8	ETHE1	chr19	44012149	+	TCACAGCTGAGGGTGAGCCG
1449	sgETHE1_9	ETHE1	chr19	44012138	+	TGACAAACTCCTCACAGCTG
1450	sgETHE1_10	ETHE1	chr19	44030716	-	CAGTCCTGGAAACAGCGCCT
1451	sgFA2H_1	FA2H	chr16	74753050	-	CCACCCTGCAGAGTACACGG
1452	sgFA2H_2	FA2H	chr16	74752951	+	TCATGTGGAACAGGAAGCGG
1453	sgFA2H_3	FA2H	chr16	74808522	-	GCTTCGTGCGGCACCACCCG
1454	sgFA2H_4	FA2H	chr16	74773935	-	GGAACCACGGTTCAAAGTGG
1455	sgFA2H_5	FA2H	chr16	74750432	+	GAAGACGCCGATCACCAGGG
1456	sgFA2H_6	FA2H	chr16	74750311	+	TGTGCGGCGAGCCAAAGTGC
1457	sgFA2H_7	FA2H	chr16	74808439	+	TTGGCCGAGTGCCTGTGCGG
1458	sgFA2H_8	FA2H	chr16	74761171	+	GTCTGAGTGGAAGAGGCGGA
1459	sgFA2H_9	FA2H	chr16	74760182	+	CAGCTGAGATACAGCACCAG
1460	sgFA2H_10	FA2H	chr16	74760159	-	CTACTACCGAACCTTTGCCC
1461	sgFADS1_1	FADS1	chr11	61584129	-	GCTCAGGGTGCGAGGAGCGG
1462	sgFADS1_2	FADS1	chr11	61584180	+	CGGGGTAGGTCCCTGAGCCG

1463	sgFADS1_3	FADS1	chr11	61580050	+	GGTACAGCAGGAAGAAGACA
1464	sgFADS1_4	FADS1	chr11	61579987	+	AGGACGTCCCAAAGACCCAA
1465	sgFADS1_5	FADS1	chr11	61579954	+	TGAGCAGCACCGCACAGAGG
1466	sgFADS1_6	FADS1	chr11	61578322	+	CATGTGGTTCCACCAACTGG
1467	sgFADS1_7	FADS1	chr11	61572205	+	GTAGAGAGGCAGCAAGGCTG
1468	sgFADS1_8	FADS1	chr11	61570333	+	GCACACAAGGACTGCACCAG
1469	sgFADS1_9	FADS1	chr11	61584357	+	AGCACCGCAGGGCAGACCGG
1470	sgFADS1_10	FADS1	chr11	61584342	-	GTGCTGAAAACCCGGCGCGC
1471	sgFADS2_1	FADS2	chr11	61595850	+	GTCGGCAGGCAGCATGGGGA
1472	sgFADS2_2	FADS2	chr11	61595868	+	GAAGGGAGGGAACCAGGGCG
1473	sgFADS2_3	FADS2	chr11	61605307	-	GCCAGTTCACCAATCAGCAG
1474	sgFADS2_4	FADS2	chr11	61607877	-	GGAGGAGGAGGAAGAACACG
1475	sgFADS2_5	FADS2	chr11	61615629	+	AGGGTGCCTCTGCCAACTGG
1476	sgFADS2_6	FADS2	chr11	61624934	-	TACATGGGGATGAGCAGCGG
1477	sgFADS2_7	FADS2	chr11	61630769	-	GTGTGACCCACACAAACCAG
1478	sgFADS2_8	FADS2	chr11	61632670	-	GCACATAGAGACTTCACCAG
1479	sgFADS2_9	FADS2	chr11	61595948	+	ATAACCTGCGCACCGACAGG
1480	sgFADS2_10	FADS2	chr11	61605329	-	GTCCTGGCTGGGCTCCTCCG
1481	sgFADS3_1	FADS3	chr11	61644400	+	GAAGCGGGCATAGAAGCTGG
1482	sgFADS3_2	FADS3	chr11	61647547	+	GCCAGCTCTCCAATCAACAG
1483	sgFADS3_3	FADS3	chr11	61646822	+	GCACCCAGCCAGGACCCAGG
1484	sgFADS3_4	FADS3	chr11	61646787	+	AGAGATGGCCAGGATGAAGG
1485	sgFADS3_5	FADS3	chr11	61646772	+	AACTGGGGTCACCTGAGAGA
1486	sgFADS3_6	FADS3	chr11	61646250	-	GAAGTCCTGGTGGAACCACG
1487	sgFADS3_7	FADS3	chr11	61644384	+	GGAGGTAGGATAAGAAGAAG
1488	sgFADS3_8	FADS3	chr11	61643893	+	GTGTGATCCACACGAACCAG
1489	sgFADS3_9	FADS3	chr11	61643875	-	ACAGATGAACCACATCCCCA
1490	sgFADS3_10	FADS3	chr11	61643411	-	GAGACACAACTACAGCCGGG
1491	sgFBXL5_1	FBXL5	chr4	15656829	-	GGTGGGGCTCTACTGCGACA
1492	sgFBXL5_2	FBXL5	chr4	15629645	-	TTTTACAGAAGAGTCTGCGG
1493	sgFBXL5_3	FBXL5	chr4	15627153	+	GTCCTTACTAAAACAACCAG
1494	sgFBXL5_4	FBXL5	chr4	15627078	+	TGAGTGACCACAATACGCAA
1495	sgFBXL5_5	FBXL5	chr4	15656863	-	TCTTCACCGCCCCACACTGG
1496	sgFBXL5_6	FBXL5	chr4	15656847	-	CTGGCGGATGAAGCAGCTGG
1497	sgFBXL5_7	FBXL5	chr4	15642413	-	TCACATGAAAGAGGAAGAGG
1498	sgFBXL5_8	FBXL5	chr4	15638275	-	GAAGTGTCAGAACACTCCAC
1499	sgFBXL5_9	FBXL5	chr4	15638181	-	GCAGTCAAGTAAGCATGAAA
1500	sgFBXL5_10	FBXL5	chr4	15627314	+	AAGAAACAGGCTTAGTCCAG
1501	sgFDX1_1	FDX1	chr11	110327660	-	GTGACAGGTTGAACAAGCCA
1502	sgFDX1_2	FDX1	chr11	110300878	+	GCCGCTTCTGCTGTCCTCGG
1503	sgFDX1_3	FDX1	chr11	110300937	-	GCAGGCCGCTGGATCCAGCG
1504	sgFDX1_4	FDX1	chr11	110300997	+	GTCGCTGAGCGTGTCGGCGC
1505	sgFDX1_5	FDX1	chr11	110306577	+	GTCCACTTTATAAACCGTGA
1506	sgFDX1_6	FDX1	chr11	110300947	+	AGCGGCCTGCTGAGGAACCG
1507	sgFDX1_7	FDX1	chr11	110300925	-	ATCCAGCGCGGGACCCAGCG
1508	sgFDX1_8	FDX1	chr11	110301024	-	GCACGGCGCCCTACCTGCTC
1509	sgFDX1_9	FDX1	chr11	110306630	+	TGATTCTCTGCTAGATGTTG
1510	sgFDX1_10	FDX1	chr11	110327640	+	AGGTGCATGTGAGGGAACCC
1511	sgFDX1L_1	FDX1L	chr19	10421539	+	GAGGAGGCAGGAGATCCAGG
1512	sgFDX1L_2	FDX1L	chr19	10426405	-	GCTGGAGAGGAGGACGCGGG
1513	sgFDX1L_3	FDX1L	chr19	10421582	+	GCAGGTGGAGCAGGCCAGGG
1514	sgFDX1L_4	FDX1L	chr19	10426614	-	GTTCTACTGCAGGCTGCCAG
1515	sgFDX1L_5	FDX1L	chr19	10426591	-	CACCTGGTGGAACAGACCTG
1516	sgFDX1L_6	FDX1L	chr19	10426653	-	ATGGCCGCCTCCATGGCCCG
1517	sgFDX1L_7	FDX1L	chr19	10426604	-	AGGCTGCCAGGGGCACCTGG
1518	sgFDX1L_8	FDX1L	chr19	10426059	+	TACCTTCCAGGTCCACCCCG
1519	sgFDX1L_9	FDX1L	chr19	10421560	+	GGTCTTCACTCACATACACA
1520	sgFDX1L_10	FDX1L	chr19	10426636	-	CCGGGGAGGCGTGAGTGCCA
1521	sgFECH_1	FECH	chr18	55247400	-	GCTGGAGGGTCTGTCAGCCA
1522	sgFECH_2	FECH	chr18	55247338	-	GAAACAGCCCAGCATGCCCA
1523	sgFECH_3	FECH	chr18	55233681	+	GTGGTGGAGCAGCTGTACTG

1524	sgFECH_4	FECH	chr18	55253790	+	ATCGCGGAGCAGGACGCCCG
1525	sgFECH_5	FECH	chr18	55230151	-	GACGGAAGCCCACGATGAAG
1526	sgFECH_6	FECH	chr18	55222138	+	ACAGTGGCGCTTACCTCCTG
1527	sgFECH_7	FECH	chr18	55222089	-	GCAACCCCTACCGACTGGTG
1528	sgFECH_8	FECH	chr18	55221497	-	GTACTCTCAAGTTTTAGCCA
1529	sgFECH_9	FECH	chr18	55240506	+	GGTCCAAGAAGAGTCTCAGA
1530	sgFECH_10	FECH	chr18	55238715	-	AGATTCAAGAGCAGTACCGC
1531	sgFLVCR1_1	FLVCR1	chr1	213031797	+	GGCGCGGCCAGACGATGAGG
1532	sgFLVCR1_2	FLVCR1	chr1	213031781	+	GGGGAGCCTGGGATATGGCG
1533	sgFLVCR1_3	FLVCR1	chr1	213031976	-	AGGCCCGCCCAGAACTCCCG
1534	sgFLVCR1_4	FLVCR1	chr1	213032110	-	AGGAGCACCACGAAGCGCCG
1535	sgFLVCR1_5	FLVCR1	chr1	213032246	-	GTAGGCCAGCATGTACACCA
1536	sgFLVCR1_6	FLVCR1	chr1	213032415	-	GGGCCACCGAGCACAAGCAC
1537	sgFLVCR1_7	FLVCR1	chr1	213061852	-	CAAATTCAAAACCCAAAGGG
1538	sgFLVCR1_8	FLVCR1	chr1	213031853	+	AAGGATACCTCCCGTTGCCG
1539	sgFLVCR1_9	FLVCR1	chr1	213032086	-	GAGAGCGCCGTAAGGGGCAG
1540	sgFLVCR1_10	FLVCR1	chr1	213032513	-	CTGATTGCCCAGCACGGCGG
1541	sgFLVCR2_1	FLVCR2	chr14	76088544	-	CAATGATGACAAGGATGAGG
1542	sgFLVCR2_2	FLVCR2	chr14	76107323	-	ACAGCAAACTCAAATCCCAG
1543	sgFLVCR2_3	FLVCR2	chr14	76088451	-	TGTTGGGTACCAAAACAGGA
1544	sgFLVCR2_4	FLVCR2	chr14	76107378	-	AGATATGTTGAGGAGGCCGG
1545	sgFLVCR2_5	FLVCR2	chr14	76107602	-	CAAGAGTAAGGAACACACAC
1546	sgFLVCR2_6	FLVCR2	chr14	76091001	-	GAGAGGTCAAGGCATAGCTC
1547	sgFLVCR2_7	FLVCR2	chr14	76101259	+	GAAGTGAATGCTGGAAGAAT
1548	sgFLVCR2_8	FLVCR2	chr14	76105773	-	CAGTGATGAACACTACCCAC
1549	sgFLVCR2_9	FLVCR2	chr14	76107568	+	ACAACTATGGAACCAAGCCT
1550	sgFLVCR2_10	FLVCR2	chr14	76090983	-	TCAGGGATTGGGCCCTGCTG
1551	sgFRAS1_1	FRAS1	chr4	79308550	+	GAAGTTCTCCACATTAGCAG
1552	sgFRAS1_2	FRAS1	chr4	78979218	+	GTATTGCCTCATCATTCCGA
1553	sgFRAS1_3	FRAS1	chr4	79166426	-	GGAGTCCTCAAAACACACTC
1554	sgFRAS1_4	FRAS1	chr4	79173565	-	GCACACACTACATGGAGAAG
1555	sgFRAS1_5	FRAS1	chr4	79173630	-	TGGTGTCCACATGACAGCGG
1556	sgFRAS1_6	FRAS1	chr4	79204000	-	GGCACTCACACACCTTGCAA
1557	sgFRAS1_7	FRAS1	chr4	79204058	-	AGTGTGCCCACTGGACATGG
1558	sgFRAS1_8	FRAS1	chr4	79205645	+	GGATGGTGTGTGCACAGCTG
1559	sgFRAS1_9	FRAS1	chr4	79284686	-	GGAGATCAGCTGCACAGTGC
1560	sgFRAS1_10	FRAS1	chr4	79284767	-	AAGGGCAGTCAGGAACACAG
1561	sgFRRS1_1	FRRS1	chr1	100214227	-	AAAGTAACACAGTCATGCCA
1562	sgFRRS1_2	FRRS1	chr1	100194051	-	GCAGATGGTGCAGCTAATGA
1563	sgFRRS1_3	FRRS1	chr1	100181155	-	ATGCCGTTTATATACAGGGG
1564	sgFRRS1_4	FRRS1	chr1	100212896	+	AATGTGAAGGAGCCAATAGG
1565	sgFRRS1_5	FRRS1	chr1	100206368	+	ACGGGAGGTAACGTTGGCAA
1566	sgFRRS1_6	FRRS1	chr1	100178029	+	AAGTCATCACTATACAGCCG
1567	sgFRRS1_7	FRRS1	chr1	100177992	+	GGCCTGAAGACTGCCAGAAG
1568	sgFRRS1_8	FRRS1	chr1	100203755	-	GAACTGTGACCCAGAGAAGG
1569	sgFRRS1_9	FRRS1	chr1	100203726	+	TGTGAAGGACAAGAAGACAC
1570	sgFRRS1_10	FRRS1	chr1	100203648	+	CTGATCATGAGACAATGCAA
1571	sgFRRS1L_1	FRRS1L	chr9	111929560	-	GAGCGAGGATGCGCAGACCG
1572	sgFRRS1L_2	FRRS1L	chr9	111929358	-	GCTGCTCCTGCTGCTACTGA
1573	sgFRRS1L_3	FRRS1L	chr9	111911974	+	CAGGAGGAGCAGTAGGGAAG
1574	sgFRRS1L_4	FRRS1L	chr9	111909371	-	GAGCTGAGTGCAGACACAGA
1575	sgFRRS1L_5	FRRS1L	chr9	111929413	-	ATCTGTGCGCACGGATGGCG
1576	sgFRRS1L_6	FRRS1L	chr9	111903751	-	AACCCTGCCAGAGATGAAGA
1577	sgFRRS1L_7	FRRS1L	chr9	111911958	+	TTGGCAAATGGATCCACAGG
1578	sgFRRS1L_8	FRRS1L	chr9	111909418	+	GTAGCTGAGGAAATAGTCAC
1579	sgFRRS1L_9	FRRS1L	chr9	111903828	+	GCCATTGTCATCATGGACGC
1580	sgFRRS1L_10	FRRS1L	chr9	111903785	-	TAATGTAGGCCAGTGGGCAA
1581	sgFTH1_1	FTH1	chr11	61734781	+	CACCATGGACAGGTAAACGT
1582	sgFTH1_2	FTH1	chr11	61732834	+	TGTTCACCTTGATATCCTGA
1583	sgFTH1_3	FTH1	chr11	61734771	+	CGCCCGCGCTCACCATGGAC
1584	sgFTH1_4	FTH1	chr11	61732545	-	GGAGAGCGGGCTGAATGCAA

1585	sgFTH1_5	FTH1	chr11	61732252	-	AGCGCCCGAATCTGGCTTGG
1586	sgFTH1_6	FTH1	chr11	61732225	-	TCTCTTTGACAAGCACACCC
1587	sgFTH1_7	FTH1	chr11	61732276	-	CGTGACCAACTTGCGCAAGA
1588	sgFTH1_8	FTH1	chr11	61734877	+	CGAGGTGGACGCGGTCGTCA
1589	sgFTH1_9	FTH1	chr11	61732872	-	ATGAAGCTGCAGAACCAACG
1590	sgFTH1_10	FTH1	chr11	61732963	-	CTACTTTGACCGCGATGATG
1591	sgFTHL17_1	FTHL17	chrX	31089906	+	GTAGCGGAAGAAGTTCTCCA
1592	sgFTHL17_2	FTHL17	chrX	31089584	+	CCGGGGAACAAATCTTGCGC
1593	sgFTHL17_3	FTHL17	chrX	31089770	-	GGAGAGCGGGCTCGTGGCCA
1594	sgFTHL17_4	FTHL17	chrX	31089707	+	CCAGCTGGTACAGATCCAGC
1595	sgFTHL17_5	FTHL17	chrX	31089515	+	GGGCTCAAGTCTCTTTGACG
1596	sgFTHL17_6	FTHL17	chrX	31089992	-	CATCAACAGCCACATCACGC
1597	sgFTHL17_7	FTHL17	chrX	31089890	+	CCGACAGGCGCAGGAAGTAG
1598	sgFTHL17_8	FTHL17	chrX	31089656	+	GGTAGTGGCTCTCCAGGAAG
1599	sgFTHL17_9	FTHL17	chrX	31089626	-	AGTCAAGACCATCAAAGAGC
1600	sgFTHL17_10	FTHL17	chrX	31089954	+	GGCCATAGACAGGTACAGGT
1601	sgFTL_1	FTL	chr19	49468856	-	GGGACTCACCAGAGAGAGGT
1602	sgFTL_2	FTL	chr19	49469916	-	GCCACCCAGCCTGTGGAGGT
1603	sgFTL_3	FTL	chr19	49469087	+	TTGGCCGAGGAGAAGCGCGA
1604	sgFTL_4	FTL	chr19	49469570	+	AGACGCCATGAAAGCTGCCA
1605	sgFTL_5	FTL	chr19	49469639	-	GGTCCGTGCGGGCAGAACCC
1606	sgFTL_6	FTL	chr19	49469044	-	CTTCCAGAGCCACATCATCG
1607	sgFTL_7	FTL	chr19	49469074	+	CTTCTTCCGCGAATTGGCCG
1608	sgFTL_8	FTL	chr19	49468785	+	GAATTATTCCACCGACGTGG
1609	sgFTL_9	FTL	chr19	49469152	-	TGATGTCCTGGAAGAGAGCG
1610	sgFTL_10	FTL	chr19	49468799	-	GTTGACGGCTGCCTCCACGT
1611	sgFTMT_1	FTMT	chr5	121187855	-	GAAGTTCTGGCGCACCCGAG
1612	sgFTMT_2	FTMT	chr5	121187877	-	TGGCAGCCTCGGAGTCGGGG
1613	sgFTMT_3	FTMT	chr5	121187701	-	GGGCGCAGAGACGCCAGCGA
1614	sgFTMT_4	FTMT	chr5	121187816	-	GGTAGGGTCCCGGGAGGAGG
1615	sgFTMT_5	FTMT	chr5	121188046	+	ATGAGGCTGCAGAACCAGCG
1616	sgFTMT_6	FTMT	chr5	121188117	+	GGAAAGCGGGCTGCATGCCA
1617	sgFTMT_7	FTMT	chr5	121187732	+	GCTTCGCGCTCCCGCTGCGT
1618	sgFTMT_8	FTMT	chr5	121187948	-	CCGGGAGAAGTAATAGGCCA
1619	sgFTMT_9	FTMT	chr5	121188096	+	GAAGCCGGAACAGGACGACT
1620	sgFTMT_10	FTMT	chr5	121188137	-	CAAGAGTAGAGCACACTCCA
1621	sgFTO_1	FTO	chr16	53860053	-	GAGGAAGGTCTCACAAGCAG
1622	sgFTO_2	FTO	chr16	53907748	-	CGGTGGGTGGAACTAAACCG
1623	sgFTO_3	FTO	chr16	53860282	+	GAAAATGCCATACCTGAAAG
1624	sgFTO_4	FTO	chr16	53860357	+	TCTGGTGGACAGGTCAGCGG
1625	sgFTO_5	FTO	chr16	53922790	+	ACAGAAAGTGCACTGACTGG
1626	sgFTO_6	FTO	chr16	53967920	+	TAAAAGAGAGGGGCTCCCCG
1627	sgFTO_7	FTO	chr16	53738099	+	GAAGCGCACCCCGACTGCCG
1628	sgFTO_8	FTO	chr16	53738120	+	GGAACGAGAGCGCGAAGCTA
1629	sgFTO_9	FTO	chr16	53860164	+	GTATGTCTGCAGATTTCCCC
1630	sgFTO_10	FTO	chr16	53860185	-	GGATGAACCCATCCCAACCC
1631	sgFXN_1	FXN	chr9	71650815	-	TGTCGGTGCGCAGGCCACGG
1632	sgFXN_2	FXN	chr9	71668080	+	AAGACTAGCAGAGGAAACGC
1633	sgFXN_3	FXN	chr9	71650857	-	CGCGGATACTTACTGCGCGG
1634	sgFXN_4	FXN	chr9	71661312	-	AAATCTGGTTGAGGCCACGT
1635	sgFXN_5	FXN	chr9	71661393	-	GTGTTTTATCTTACCCTGGG
1636	sgFXN_6	FXN	chr9	71650755	+	CCAGGCCCAGACCCTCACCC
1637	sgFXN_7	FXN	chr9	71679860	+	GTCTTAACTGTCAAACTGGG
1638	sgFXN_8	FXN	chr9	71650719	-	CCAGGAGGCCGGCTACTGCG
1639	sgFXN_9	FXN	chr9	71650734	-	CTGGGCTGGGTGACGCCAGG
1640	sgFXN_10	FXN	chr9	71661295	+	TTTCAGAGTTCGAACCAACG
1641	sgGALT_1	GALT	chr9	34649475	-	GTGGCAGAGCGCAGGAGCGG
1642	sgGALT_2	GALT	chr9	34647223	-	CGGATGGCCCCAGGACACAG
1643	sgGALT_3	GALT	chr9	34647847	-	GCGTTACATCCGACCAGGGG
1644	sgGALT_4	GALT	chr9	34648415	-	CGGCTGTACTCCATTAGCAG
1645	sgGALT_5	GALT	chr9	34648845	-	GTAGCCGCCGCACATGCCGA

1646	sgGALT_6	GALT	chr9	34648877	-	CTGACCATCACGCTCAGCAG
1647	sgGALT_7	GALT	chr9	34649405	+	AGGGGCTCCCACAGGATCAG
1648	sgGALT_8	GALT	chr9	34647234	+	GGCCATCCGAGCCAACGGAG
1649	sgGALT_9	GALT	chr9	34648134	-	GGGGTTAGAACAGCCCATCA
1650	sgGALT_10	GALT	chr9	34649004	-	CAAGAGCTTCTTCATGATGG
1651	sgGLRX2_1	GLRX2	chr1	193066763	-	GAACTGGACCTGCTTGAATA
1652	sgGLRX2_2	GLRX2	chr1	193066734	+	TGTAAAGAGCATCTTGGAAC
1653	sgGLRX2_3	GLRX2	chr1	193066825	+	TGCCATTGTACAGTAAGAAC
1654	sgGLRX2_4	GLRX2	chr1	193066752	+	ACTGGTTTCCATATTCAAGC
1655	sgGLRX2_5	GLRX2	chr1	193065836	-	GAGGTGCAACTGACACTCAT
1656	sgGLRX2_6	GLRX2	chr1	193065822	-	ACTCATAGGCTTCACAAAGA
1657	sgGLRX2_7	GLRX2	chr1	193074649	-	GGACGCGCTCAGCTGCGAGG
1658	sgGLRX2_8	GLRX2	chr1	193074431	-	AGGCTGGCTTGACAGGGCGG
1659	sgGLRX2_9	GLRX2	chr1	193074712	+	CGTGTAAACATCCTTTAGAG
1660	sgGLRX2_10	GLRX2	chr1	193074455	-	GGTTTGGAGCAGGAGCGGCT
1661	sgGLRX3_1	GLRX3	chr10	131934712	+	GTAGCGGCCGTGGAGGAGGT
1662	sgGLRX3_2	GLRX3	chr10	131959188	-	GAGTCAATTTCTTCAAGCGA
1663	sgGLRX3_3	GLRX3	chr10	131973332	+	GGGAGGATTGGATATTGTGA
1664	sgGLRX3_4	GLRX3	chr10	131959149	-	GATGTTCATTAGCGCTGGGT
1665	sgGLRX3_5	GLRX3	chr10	131964839	+	TCAGATGAAGAGGTTCGACA
1666	sgGLRX3_6	GLRX3	chr10	131943466	+	GATTTACAGGTCCCTCCTTG
1667	sgGLRX3_7	GLRX3	chr10	131943480	-	CCAGAAATGGACCACAAGGA
1668	sgGLRX3_8	GLRX3	chr10	131959240	+	ACTCCTCAAGAACCACGCTG
1669	sgGLRX3_9	GLRX3	chr10	131959255	-	GGCAGCTTCTTACCACAGCG
1670	sgGLRX3_10	GLRX3	chr10	131964766	+	TACAGGTTTCAGCAAGCAGA
1671	sgGLRX5_1	GLRX5	chr14	96001567	+	GCTGGTGAAGAAGGACAAGG
1672	sgGLRX5_2	GLRX5	chr14	96001624	+	GTGCGGCTTCAGCAACGCCG
1673	sgGLRX5_3	GLRX5	chr14	96001549	+	GGCGGAGCAGTTGGACGCGC
1674	sgGLRX5_4	GLRX5	chr14	96001644	-	CAGCCGCAGGATCTGCACCA
1675	sgGLRX5_5	GLRX5	chr14	96001700	+	GACGACCCGGAGCTCCGACA
1676	sgGLRX5_6	GLRX5	chr14	96001413	+	GCTTGCGTGCGGAGATGAGC
1677	sgGLRX5_7	GLRX5	chr14	96001591	+	GGTCTTCCTCAAGGGGACGC
1678	sgGLRX5_8	GLRX5	chr14	96001607	+	ACGCCGGAGCAGCCCCAGTG
1679	sgGLRX5_9	GLRX5	chr14	96001678	+	TTACGCGGCCTACAACGTGC
1680	sgGLRX5_10	GLRX5	chr14	96001447	-	GGAGCAGAGCCGCCGCAGCT
1681	sgGRXCR1_1	GRXCR1	chr4	42964962	-	AGGTTGTCCGGACCACACGA
1682	sgGRXCR1_2	GRXCR1	chr4	42965117	-	ATCAATGAACACAACAGGGA
1683	sgGRXCR1_3	GRXCR1	chr4	42895440	+	GATGGACTAGGTGATTCCGA
1684	sgGRXCR1_4	GRXCR1	chr4	42895608	+	GTCAAATACAAAGTGAGTGC
1685	sgGRXCR1_5	GRXCR1	chr4	42965094	-	AAGGAGCTTCAGAAACTCGT
1686	sgGRXCR1_6	GRXCR1	chr4	42895338	+	CGGATCGCGTCCTCTCACAG
1687	sgGRXCR1_7	GRXCR1	chr4	42895303	+	AGCCAGAAAGTGACAGGCCA
1688	sgGRXCR1_8	GRXCR1	chr4	42895368	+	CTGAAGGAAGTGTATGAAGA
1689	sgGRXCR1_9	GRXCR1	chr4	42895398	-	TCAGAATCCAGAGAGCCTGA
1690	sgGRXCR1_10	GRXCR1	chr4	42895493	+	TGATGAGAATGAGAATGACC
1691	sgGSTP1_1	GSTP1	chr11	67352646	+	GGACATGGTGAATGACGGCG
1692	sgGSTP1_2	GSTP1	chr11	67352676	-	GGGAGATGTATTTGCAGCGG
1693	sgGSTP1_3	GSTP1	chr11	67351600	+	TGCAGTGCCGCCCTACACCG
1694	sgGSTP1_4	GSTP1	chr11	67351951	-	GATCTGCCAGCAGCATGCGC
1695	sgGSTP1_5	GSTP1	chr11	67351969	+	TCAGGGCCAGAGCTGGAAGG
1696	sgGSTP1_6	GSTP1	chr11	67351984	+	GAAGGAGGAGGTGGTGACCG
1697	sgGSTP1_7	GSTP1	chr11	67352613	+	CTATGGGAAGGACCAGCAGG
1698	sgGSTP1_8	GSTP1	chr11	67353652	+	GGGAGGCAAGACCTTCATTG
1699	sgGSTP1_9	GSTP1	chr11	67351620	-	TCGAACTGGGAAATAGACCA
1700	sgGSTP1_10	GSTP1	chr11	67352217	-	GCGGCCCAGGTGACGCAGGA
1701	sgGUCY1A2_1	GUCY1A2	chr11	106888706	-	GGAGACCAGCCCGGAGGAGG
1702	sgGUCY1A2_2	GUCY1A2	chr11	106810646	+	GCAAACCCCACAATATGGTG
1703	sgGUCY1A2_3	GUCY1A2	chr11	106810596	-	GAAGATCTATCGGCTGGATG
1704	sgGUCY1A2_4	GUCY1A2	chr11	106681006	-	AGATGGGTTGAAGAAAAGGA
1705	sgGUCY1A2_5	GUCY1A2	chr11	106680888	-	GTAGCCCAGCAATTATGGCA
1706	sgGUCY1A2_6	GUCY1A2	chr11	106647233	+	CAATGGGTTTAGCATGGCAG

1707	sgGUCY1A2_7	GUCY1A2	chr11	106647181	-	GAAGAGGTGCTGACACCTGA
1708	sgGUCY1A2_8	GUCY1A2	chr11	106579250	+	GGGCTGACATTGATGCGCCG
1709	sgGUCY1A2_9	GUCY1A2	chr11	106810454	+	GGAACTTGGGAGGTTCCCTG
1710	sgGUCY1A2_10	GUCY1A2	chr11	106810444	+	GAGGTCCGCAGGAACTTGGG
1711	sgGUCY1A3_1	GUCY1A3	chr4	156631952	-	GATGCCGGGAAGAATCAGGG
1712	sgGUCY1A3_2	GUCY1A3	chr4	156634471	+	TGAGCAAGCCCACCAAGCCC
1713	sgGUCY1A3_3	GUCY1A3	chr4	156631686	+	GATTTCAGGAGTTCCAGTGG
1714	sgGUCY1A3_4	GUCY1A3	chr4	156631773	-	GGGTGCCTCCAACCACCCCA
1715	sgGUCY1A3_5	GUCY1A3	chr4	156632121	-	GCGAGGACTGGGGTTTGCTG
1716	sgGUCY1A3_6	GUCY1A3	chr4	156632131	-	GGAATCACCAGCGAGGACTG
1717	sgGUCY1A3_7	GUCY1A3	chr4	156634412	+	GAACAAGCCCGAGCTCAAGA
1718	sgGUCY1A3_8	GUCY1A3	chr4	156634542	+	GTGAGGTTGCTCAGCAGCTG
1719	sgGUCY1A3_9	GUCY1A3	chr4	156634608	-	AACGATGTCTGAGAAGAGCA
1720	sgGUCY1A3_10	GUCY1A3	chr4	156629425	+	GCAGAGCAAGCAGTTGCAGC
1721	sgGUCY1B3_1	GUCY1B3	chr4	156680974	+	GATCCGCAATTACGGCCCCG
1722	sgGUCY1B3_2	GUCY1B3	chr4	156710923	-	GCATCAGTGCACCTAAAGGA
1723	sgGUCY1B3_3	GUCY1B3	chr4	156711022	+	ACAGTGGCACAACAAATCCA
1724	sgGUCY1B3_4	GUCY1B3	chr4	156716516	-	CCAGCGAGAAGACAGACAGA
1725	sgGUCY1B3_5	GUCY1B3	chr4	156696124	+	GAGGCACAGTTAGATGAAGA
1726	sgGUCY1B3_6	GUCY1B3	chr4	156717525	+	CAAGGAAGGATTGTTGGATG
1727	sgGUCY1B3_7	GUCY1B3	chr4	156721137	+	ATACAAACTCACCCAAGAAC
1728	sgGUCY1B3_8	GUCY1B3	chr4	156723517	+	CGTCTGTTGCCAATGAGCTG
1729	sgGUCY1B3_9	GUCY1B3	chr4	156725750	+	AATAGGGATACACACTGGAG
1730	sgGUCY1B3_10	GUCY1B3	chr4	156680941	+	CGGATTTGTGAATCACGCCC
1731	sgHAAO_1	HAAO	chr2	43019655	-	GGAGCGCCGCCTGGGAGTGA
1732	sgHAAO_2	HAAO	chr2	43015698	-	CGGAGGCCCCAACACCAGGA
1733	sgHAAO_3	HAAO	chr2	43015680	-	GAAGGACTATCACATCGAAG
1734	sgHAAO_4	HAAO	chr2	43010522	-	GGGTGCCCCACTCACCACAG
1735	sgHAAO_5	HAAO	chr2	43010506	-	ACAGAGGTTTGCCAACACCG
1736	sgHAAO_6	HAAO	chr2	43019643	-	GGGAGTGAGGGCCTGGGTGA
1737	sgHAAO_7	HAAO	chr2	43019633	-	GCCTGGGTGAAGGAGAACCG
1738	sgHAAO_8	HAAO	chr2	42996959	+	CGTGTGCTCAGAGGGAATGG
1739	sgHAAO_9	HAAO	chr2	42995040	-	TATGGGCAAGGCAGCAGCGA
1740	sgHAAO_10	HAAO	chr2	43010992	-	ACAGGTATTTTACCAGCTGG
1741	sgHAMP_1	HAMP	chr19	35773529	+	CTCCTCGCCAGCCTGACCAG
1742	sgHAMP_2	HAMP	chr19	35775744	-	TGTTGCGCTCACCATCCAGC
1743	sgHAMP_3	HAMP	chr19	35773543	-	GGAAAACAGAGCCACTGGTC
1744	sgHAMP_4	HAMP	chr19	35775726	+	GAGCTGGAGCCAGGGCCAGC
1745	sgHAMP_5	HAMP	chr19	35775710	+	CTGCAACCCCAGGACAGAGC
1746	sgHAMP_6	HAMP	chr19	35773497	-	GAGGCAAGCGGCCCAGATCT
1747	sgHAMP_7	HAMP	chr19	35775884	-	GCCGCAGCAGAAAATGCAGA
1748	sgHAMP_8	HAMP	chr19	35775905	+	GCTGTCATCGATCAAAGTGT
1749	sgHAMP_9	HAMP	chr19	35775842	+	CCATGTTCCAGAGGCGAAGG
1750	sgHAMP_10	HAMP	chr19	35773519	-	TGGCGAGGAGGAGGAGGAGC
1751	sgHBA1_1	HBA1	chr16	226759	-	GTGCGCGCCGACCTTACCCC
1752	sgHBA1_2	HBA1	chr16	226775	+	GCACGCTGGCGAGTATGGTG
1753	sgHBA1_3	HBA1	chr16	226942	-	GTAGGTCTTGGTGGTGGGGA
1754	sgHBA1_4	HBA1	chr16	227000	+	TAAGGGCCACGGCAAGAAGG
1755	sgHBA1_5	HBA1	chr16	227024	+	CGACGCGCTGACCAACGCCG
1756	sgHBA1_6	HBA1	chr16	227093	+	GCACGCGCACAAGCTTCGGG
1757	sgHBA1_7	HBA1	chr16	226739	+	GACCAACGTCAAGGCCGCCT
1758	sgHBA1_8	HBA1	chr16	226749	+	AAGGCCGCCTGGGGTAAGGT
1759	sgHBA1_9	HBA1	chr16	227068	-	GCTCAGGGCGGACAGCGCGT
1760	sgHBA1_10	HBA1	chr16	227080	-	CGCGTGCAGGTCGCTCAGGG
1761	sgHBA2_1	HBA2	chr16	222955	-	GTGCGCGCCGACCTTACCCC
1762	sgHBA2_2	HBA2	chr16	222971	+	GCACGCTGGCGAGTATGGTG
1763	sgHBA2_3	HBA2	chr16	223138	-	GTAGGTCTTGGTGGTGGGGA
1764	sgHBA2_4	HBA2	chr16	223196	+	TAAGGGCCACGGCAAGAAGG
1765	sgHBA2_5	HBA2	chr16	223220	+	CGACGCGCTGACCAACGCCG
1766	sgHBA2_6	HBA2	chr16	223289	+	GCACGCGCACAAGCTTCGGG
1767	sgHBA2_7	HBA2	chr16	222935	+	GACCAACGTCAAGGCCGCCT

1768	sgHBA2_8	HBA2	chr16	222945	+	AAGGCCGCCTGGGGTAAGGT
1769	sgHBA2_9	HBA2	chr16	223264	-	GCTCAGGGCGGACAGCGCGT
1770	sgHBA2_10	HBA2	chr16	223276	-	CGCGTGCAGGTCGCTCAGGG
1771	sgHBB_1	HBB	chr11	5248207	-	AGTCTGCCGTTACTGCCCTG
1772	sgHBB_2	HBB	chr11	5248181	-	AAGGTGAACGTGGATGAAGT
1773	sgHBB_3	HBB	chr11	5247840	+	ACAGTGCAGCTCACTCAGTG
1774	sgHBB_4	HBB	chr11	5248191	-	CCTGTGGGGCAAGGTGAACG
1775	sgHBB_5	HBB	chr11	5247857	+	GTGTGGCAAAGGTGCCCTTG
1776	sgHBB_6	HBB	chr11	5248022	-	TCCCACCCTTAGGCTGCTGG
1777	sgHBB_7	HBB	chr11	5246869	-	GGCTGCCTATCAGAAAGTGG
1778	sgHBB_8	HBB	chr11	5246941	-	ACAGCTCCTGGGCAACGTGC
1779	sgHBB_9	HBB	chr11	5246890	-	AGAATTCACCCCACCAGTGC
1780	sgHBB_10	HBB	chr11	5246879	+	ATAGGCAGCCTGCACTGGTG
1781	sgHBD_1	HBD	chr11	5255593	-	AAAGTGAACGTGGATGCAGT
1782	sgHBD_2	HBD	chr11	5255619	-	AGACTGCTGTCAATGCCCTG
1783	sgHBD_3	HBD	chr11	5255436	-	TCCTACCCTCAGATTACTGG
1784	sgHBD_4	HBD	chr11	5255603	-	CCTGTGGGGCAAAGTGAACG
1785	sgHBD_5	HBD	chr11	5255337	-	GGTGAAGGCTCATGGCAAGA
1786	sgHBD_6	HBD	chr11	5255425	-	GATTACTGGTGGTCTACCCT
1787	sgHBD_7	HBD	chr11	5254307	-	GCAGCTCTTGGGCAATGTGC
1788	sgHBD_8	HBD	chr11	5254277	-	GCTGGCCCGCAACTTTGGCA
1789	sgHBD_9	HBD	chr11	5254256	-	GGAATTCACCCCACAAATGC
1790	sgHBD_10	HBD	chr11	5254238	-	GCAGGCTGCCTATCAGAAGG
1791	sgHBE1_1	HBE1	chr11	5291054	-	GAGCAAGATGAATGTGGAAG
1792	sgHBE1_2	HBE1	chr11	5290881	+	GGGTCCAGGGGTAAACAACG
1793	sgHBE1_3	HBE1	chr11	5290723	+	CAGCTCACTCAGCTTAGCAA
1794	sgHBE1_4	HBE1	chr11	5291031	-	CTGGAGGTGAAGCCTTGGGC
1795	sgHBE1_5	HBE1	chr11	5291096	-	GCATTTTACTGCTGAGGAGA
1796	sgHBE1_6	HBE1	chr11	5291076	-	AGGCTGCCGTCACTAGCCTG
1797	sgHBE1_7	HBE1	chr11	5290788	+	AAGTCAGCACCTTCTTGCCA
1798	sgHBE1_8	HBE1	chr11	5290816	+	GACCTTGGGGTTGCCCAGGA
1799	sgHBE1_9	HBE1	chr11	5290800	-	GGTCAAGGCCCATGGCAAGA
1800	sgHBE1_10	HBE1	chr11	5291016	+	AACCAATGCTTACCTGCCCA
1801	sgHBG1_1	HBG1	chr11	5270667	+	GAGATCATCCAGGTGCTTTG
1802	sgHBG1_2	HBG1	chr11	5269621	-	GCAGAAGATGGTGACTGCAG
1803	sgHBG1_3	HBG1	chr11	5270679	+	GTGCTTTGTGGCATCTCCCA
1804	sgHBG1_4	HBG1	chr11	5270945	-	CTGGAGGAGAAACCCTGGGA
1805	sgHBG1_5	HBG1	chr11	5270736	+	GGGGTTGCCCATGATGGCAG
1806	sgHBG1_6	HBG1	chr11	5270961	-	GTGAATGTGGAAGATGCTGG
1807	sgHBG1_7	HBG1	chr11	5270775	+	AAAGCTGTCAAAGAACCTCT
1808	sgHBG1_8	HBG1	chr11	5271016	-	CATGGGTCATTTCACAGAGG
1809	sgHBG1_9	HBG1	chr11	5270930	+	CACCAGAGCCTACCTTCCCA
1810	sgHBG1_10	HBG1	chr11	5270990	-	AGGCTACTATCACAAGCCTG
1811	sgHBG2_1	HBG2	chr11	5274539	-	GCAGAAGATGGTGACTGGAG
1812	sgHBG2_2	HBG2	chr11	5275603	+	GTGCTTTATGGCATCTCCCA
1813	sgHBG2_3	HBG2	chr11	5275869	-	CTGGAGGAGAAACCCTGGGA
1814	sgHBG2_4	HBG2	chr11	5275660	+	GGGGTTGCCCATGATGGCAG
1815	sgHBG2_5	HBG2	chr11	5275885	-	GTGAATGTGGAAGATGCTGG
1816	sgHBG2_6	HBG2	chr11	5275699	+	AAAGCTGTCAAAGAACCTCT
1817	sgHBG2_7	HBG2	chr11	5275590	-	AAAGCACCTGGATGATCTCA
1818	sgHBG2_8	HBG2	chr11	5275940	-	CATGGGTCATTTCACAGAGG
1819	sgHBG2_9	HBG2	chr11	5275854	+	CACCAGAGCCTACCTTCCCA
1820	sgHBG2_10	HBG2	chr11	5275914	-	AGGCTACTATCACAAGCCTG
1821	sgHBM_1	HBM	chr16	216011	+	GGAGCGCGCCCAAATCGCGC
1822	sgHBM_2	HBM	chr16	216328	+	GCGACGCAGCTGCTGAGCCA
1823	sgHBM_3	HBM	chr16	216293	-	CAGGTGCGGGAAGTAGACCT
1824	sgHBM_4	HBM	chr16	216053	+	CCACGAGGCGCAATTCGGGG
1825	sgHBM_5	HBM	chr16	216351	+	GCAGCGCATGCTGGCGGCTG
1826	sgHBM_6	HBM	chr16	216464	-	GAAAGGCCTCACCGGAAAGT
1827	sgHBM_7	HBM	chr16	216038	+	GGACCTGATTGCGGGCCACG
1828	sgHBM_8	HBM	chr16	216421	-	AGCGCGTGCAGGTCCGCCAG

1829	sgHBM_9	HBM	chr16	215998	-	GCGCTCCTGGGCGCTGAGCA
1830	sgHBM_10	HBM	chr16	216067	+	TCGGGGCGGAGCTGCTGCTC
1831	sgHBQ1_1	HBQ1	chr16	230531	+	AAGAAGCTGGGCAGCAACGT
1832	sgHBQ1_2	HBQ1	chr16	230685	-	GGAGAAGTAGGTCTTCGTGG
1833	sgHBQ1_3	HBQ1	chr16	230827	-	GCAGCTGGCACGCGTGCAGG
1834	sgHBQ1_4	HBQ1	chr16	230706	-	GCCGGGGCTCAGGTCCAGGT
1835	sgHBQ1_5	HBQ1	chr16	230740	+	AGCCCACGGCCAGAAGGTGG
1836	sgHBQ1_6	HBQ1	chr16	230862	-	GCAGCCGCTCACCTGGAAGC
1837	sgHBQ1_7	HBQ1	chr16	230548	+	CGTCGGCGTCTACACGACAG
1838	sgHBQ1_8	HBQ1	chr16	230670	-	CGTGGCGGGGAAAGCCAGGA
1839	sgHBQ1_9	HBQ1	chr16	230817	-	CGCGTGCAGGTGGCTCAGCG
1840	sgHBQ1_10	HBQ1	chr16	230559	+	ACACGACAGAGGCCCTGGAA
1841	sgHBZ_1	HBZ	chr16	202910	+	TGTCTCTGACCAAGACTGAG
1842	sgHBZ_2	HBZ	chr16	202922	-	AATGATGGTCCTCTCAGTCT
1843	sgHBZ_3	HBZ	chr16	204344	-	CGATAGGAACTTGTCCCAGG
1844	sgHBZ_4	HBZ	chr16	202950	+	GTGGGCCAAGATCTCCACGC
1845	sgHBZ_5	HBZ	chr16	204009	+	AAGAGCATCGACGACATCGG
1846	sgHBZ_6	HBZ	chr16	202937	-	GGCCCACATGGACACAATGA
1847	sgHBZ_7	HBZ	chr16	204038	-	GCAGCTCGCTCAGCTTGGAC
1848	sgHBZ_8	HBZ	chr16	204071	-	AGTTGACCGGGTCCACGCGC
1849	sgHBZ_9	HBZ	chr16	202982	+	TCGGCACCGAGACTCTGGAG
1850	sgHBZ_10	HBZ	chr16	203906	-	AGTAGGTCTTGGTCTGCGGG
1851	sgHEBP1_1	HEBP1	chr12	13142247	-	TCTACGGGAAGCAATGCCCA
1852	sgHEBP1_2	HEBP1	chr12	13140107	-	GTTAAGATTGAGGAACGGGA
1853	sgHEBP1_3	HEBP1	chr12	13142315	-	GAAGAAAGGGCCTGTGAAGG
1854	sgHEBP1_4	HEBP1	chr12	13140258	-	TGTGTTTTCAGGAATTGGGA
1855	sgHEBP1_5	HEBP1	chr12	13153004	-	TGTTCGGAAGCGTAGAGACG
1856	sgHEBP1_6	HEBP1	chr12	13152981	-	CCTTGGCAGGTCCTAAGCAA
1857	sgHEBP1_7	HEBP1	chr12	13140201	+	GCAGAGAGCCATCTTCATTG
1858	sgHEBP1_8	HEBP1	chr12	13142277	-	AGAAGTGACAGATAAGCCTG
1859	sgHEBP1_9	HEBP1	chr12	13140214	+	TTCATTGGGGAACACAGCAA
1860	sgHEBP1_10	HEBP1	chr12	13140149	+	GGGTCGCTTTGAAATTGGTT
1861	sgHEBP2_1	HEBP2	chr6	138726317	+	CAAGTGGGTCAGCACGTCCG
1862	sgHEBP2_2	HEBP2	chr6	138725634	-	TGGCTGGAGCGGCTCGGCCA
1863	sgHEBP2_3	HEBP2	chr6	138725705	+	TGGAAGGCCCCGGAGGACGC
1864	sgHEBP2_4	HEBP2	chr6	138727189	-	AGAGGGAATATACAGGGAAA
1865	sgHEBP2_5	HEBP2	chr6	138727227	-	TCTGACTCTAAAGGCCTGGG
1866	sgHEBP2_6	HEBP2	chr6	138726301	+	GACACTATGGACCAGCCAAG
1867	sgHEBP2_7	HEBP2	chr6	138725685	+	AAGCTGTGGAGACGCCGGGC
1868	sgHEBP2_8	HEBP2	chr6	138727134	+	GTGACAAGCTACGTGGAGCC
1869	sgHEBP2_9	HEBP2	chr6	138727210	+	AACAGCAATTTGATCCACCC
1870	sgHEBP2_10	HEBP2	chr6	138726288	+	AGTTATGAGATCCGACACTA
1871	sgHERC2_1	HERC2	chr15	28508195	-	GATGTGGTCAAAGTCCGCTG
1872	sgHERC2_2	HERC2	chr15	28501376	-	GAACAGCCTGAAGCAGACGG
1873	sgHERC2_3	HERC2	chr15	28491921	-	CAGCTGGCGGCACTTCGCGG
1874	sgHERC2_4	HERC2	chr15	28413659	+	GGGGCCGCCGAGGAGAACGA
1875	sgHERC2_5	HERC2	chr15	28377368	+	AGGCGATGTCAACCACACGG
1876	sgHERC2_6	HERC2	chr15	28369211	+	AGAGTGTCGAACCCAACAGA
1877	sgHERC2_7	HERC2	chr15	28366495	+	GATGCGGTTCAGCTCCACGA
1878	sgHERC2_8	HERC2	chr15	28361899	-	TGATCGTGACACCCAACGGG
1879	sgHERC2_9	HERC2	chr15	28544550	-	GGAGAGCTCCCTCCTAGAAA
1880	sgHERC2_10	HERC2	chr15	28525336	+	GAGTCGCAGGGCTGAGAGGG
1881	sgHGD_1	HGD	chr3	120365173	-	GATGTCTTTGAGGAGACCAG
1882	sgHGD_2	HGD	chr3	120363218	-	GAGGATCGCCAAGTACCAGG
1883	sgHGD_3	HGD	chr3	120352004	-	GCACCTGAGAGGATTGCCGA
1884	sgHGD_4	HGD	chr3	120393808	+	AGATTGTAGGGGCAGACCTG
1885	sgHGD_5	HGD	chr3	120393797	+	GCTCAGCATAGAGATTGTAG
1886	sgHGD_6	HGD	chr3	120389338	+	AAGGGCTTGTGAGAAACTGA
1887	sgHGD_7	HGD	chr3	120369625	+	TCTCCATGGAGGTATTGCAG
1888	sgHGD_8	HGD	chr3	120363248	+	ATGGGTATCAAGAAATCACG
1889	sgHGD_9	HGD	chr3	120363229	+	GCGATCCTCATACCAGGCAA

1890	sgHGD_10	HGD	chr3	120357368	+	CAGCAATGGCCACTCCAGGG
1891	sgHIF1AN_1	HIF1AN	chr10	102305777	-	GGATGTAAAGAACATCACCA
1892	sgHIF1AN_2	HIF1AN	chr10	102295845	-	GGGTCGCTCTGACTCAGACG
1893	sgHIF1AN_3	HIF1AN	chr10	102296387	+	CAGGATATACAGCAGCGAGG
1894	sgHIF1AN_4	HIF1AN	chr10	102300403	+	GCAAACGCTCAATGACACTG
1895	sgHIF1AN_5	HIF1AN	chr10	102296319	+	ATTTCCAGAACTTTAAGCCG
1896	sgHIF1AN_6	HIF1AN	chr10	102300479	+	CAACAGGGAAAGCGTGGCTG
1897	sgHIF1AN_7	HIF1AN	chr10	102304790	-	GGCACTCGAACTGATCCGGA
1898	sgHIF1AN_8	HIF1AN	chr10	102306971	+	GAAGATGCTTGGAGAGGCCT
1899	sgHIF1AN_9	HIF1AN	chr10	102295747	-	CCGGGGCTCTCCAGAGCCAG
1900	sgHIF1AN_10	HIF1AN	chr10	102304811	-	GATGAACAGGGTATGGGTAG
1901	sgHMOX1_1	HMOX1	chr22	35779106	-	AGGGCCTCTGACAAATCCTG
1902	sgHMOX1_2	HMOX1	chr22	35782875	+	TGTGAAGCGGCTCCACGAGG
1903	sgHMOX1_3	HMOX1	chr22	35782977	+	AAAGATTGCCCAGAAAGCCC
1904	sgHMOX1_4	HMOX1	chr22	35782998	+	GGACCTGCCCAGCTCTGGCG
1905	sgHMOX1_5	HMOX1	chr22	35783039	-	GGCACTGGCAATGTTGGGGA
1906	sgHMOX1_6	HMOX1	chr22	35779177	+	GAGGAACTTTCAGAAGGGCC
1907	sgHMOX1_7	HMOX1	chr22	35782817	-	CCAGCGGGGCCCGTACCAGA
1908	sgHMOX1_8	HMOX1	chr22	35782831	-	GGGATGACCTCCTGCCAGCG
1909	sgHMOX1_9	HMOX1	chr22	35782928	-	CAGGTAGCGGGTGTAGGCGT
1910	sgHMOX1_10	HMOX1	chr22	35782941	-	CAGACAGGTCACCCAGGTAG
1911	sgHMOX2_1	HMOX2	chr16	4557724	-	GTATGTGAAGTAAAGTGCCG
1912	sgHMOX2_2	HMOX2	chr16	4557737	+	CACATACTCAGCCCTCGAGG
1913	sgHMOX2_3	HMOX2	chr16	4557790	-	GGGGAAGTACAAAGGGGCAA
1914	sgHMOX2_4	HMOX2	chr16	4557827	+	GGAGGCGCTGACCAAGGACA
1915	sgHMOX2_5	HMOX2	chr16	4557875	+	GGAGCAGGTGCAGTGCCCCA
1916	sgHMOX2_6	HMOX2	chr16	4557938	+	GAACGAGCCGGAGCTACTGG
1917	sgHMOX2_7	HMOX2	chr16	4555533	+	CGGAAGTGGAAACCTCAGAG
1918	sgHMOX2_8	HMOX2	chr16	4557948	-	GCATGGGCCACCAGTAGCTC
1919	sgHMOX2_9	HMOX2	chr16	4558029	+	GCACTGAAACTCCCCAGCAC
1920	sgHMOX2_10	HMOX2	chr16	4558141	+	TGAACATGAAGACCAAAGAG
1921	sgHPD_1	HPD	chr12	122287672	-	GGACACCACACACACCCTGG
1922	sgHPD_2	HPD	chr12	122294256	-	GAAGGACATTGCGTTCGAGG
1923	sgHPD_3	HPD	chr12	122285077	+	CATCCACGGACCAGAAGCGG
1924	sgHPD_4	HPD	chr12	122284821	-	GGAATATGTGGACTATAACG
1925	sgHPD_5	HPD	chr12	122281618	-	GGAGAACATTGATGCCCTGG
1926	sgHPD_6	HPD	chr12	122295263	-	GGAGACCGGTTCCCGGGAGG
1927	sgHPD_7	HPD	chr12	122295236	-	CCATGTAATCAAACAAGGGA
1928	sgHPD_8	HPD	chr12	122294491	-	GCGCTCAACCCCTGGAACAA
1929	sgHPD_9	HPD	chr12	122294480	+	GCACCTCACCTTTGTTCCAG
1930	sgHPD_10	HPD	chr12	122294274	-	GAAACACGGTGACGGAGTGA
1931	sgHPDL_1	HPDL	chr1	45792834	-	GATGTGGCACAAACGAAGGG
1932	sgHPDL_2	HPDL	chr1	45793159	+	GGGTGCCGCCACTTACGCCG
1933	sgHPDL_3	HPDL	chr1	45793499	-	GACTCAGCCAGAACAAGAGT
1934	sgHPDL_4	HPDL	chr1	45793531	+	GACGACACGACAGGACCAGG
1935	sgHPDL_5	HPDL	chr1	45793603	+	GTATACGCCTAACATTGTGG
1936	sgHPDL_6	HPDL	chr1	45793860	-	GTTGCCCTGACCAAAGCCAG
1937	sgHPDL_7	HPDL	chr1	45792947	+	CGGCAGCTAGCCCTGCGCAG
1938	sgHPDL_8	HPDL	chr1	45793050	-	GAAGCACAGGTTTGTGGCGC
1939	sgHPDL_9	HPDL	chr1	45793344	-	GTGGAACCAGCGCAAAAGTG
1940	sgHPDL_10	HPDL	chr1	45793363	-	GGCAAAAGCCCAGGCAGTCG
1941	sgHPX_1	HPX	chr11	6458709	-	GGTGCCTCCCAGGTACCCGC
1942	sgHPX_2	HPX	chr11	6458690	+	AAGTAGTCTCGGACATCCCG
1943	sgHPX_3	HPX	chr11	6459624	-	GCAGCTGTGGAATGTCACCG
1944	sgHPX_4	HPX	chr11	6458779	+	GTAGCGGCCCAGCCATCTCA
1945	sgHPX_5	HPX	chr11	6462149	+	GCTCCACAACCCCAGTGCAA
1946	sgHPX_6	HPX	chr11	6461713	+	GGTTCCATTGTCATCCAGGG
1947	sgHPX_7	HPX	chr11	6461485	-	GGAAGAGTCACAAATGGGAC
1948	sgHPX_8	HPX	chr11	6461442	-	GAAGAATTTCCCCAGCCCTG
1949	sgHPX_9	HPX	chr11	6458856	+	CCAAGTCCCAGAACCACTCG
1950	sgHPX_10	HPX	chr11	6458804	+	GAGGAGCAGTTCCCAACAGC

1951	sgHR_1	HR	chr8	21986390	-	GCAGCAAAGAGGGACTGCGC
1952	sgHR_2	HR	chr8	21984910	-	GAAGTGCCAGGAGGGCCTGG
1953	sgHR_3	HR	chr8	21983097	-	GCCACTCTCAGCAAGTGCGG
1954	sgHR_4	HR	chr8	21986659	-	GAGTACGCCCAGCTTCCTGA
1955	sgHR_5	HR	chr8	21986544	+	GCAGGCTCTCCCAGGCACAG
1956	sgHR_6	HR	chr8	21986455	-	GGACATGCTCCCACTTGTGG
1957	sgHR_7	HR	chr8	21986328	-	CCAGCGTGCCCACCTCGCTG
1958	sgHR_8	HR	chr8	21986116	-	GCACCCCTGGGTATACTCCG
1959	sgHR_9	HR	chr8	21985037	-	GGAACCTTGGGTACCAGCTG
1960	sgHR_10	HR	chr8	21984967	+	AGGATGAACAGCAGCCCCGC
1961	sgHRG_1	HRG	chr3	186383932	+	ATCAATAAAAGGCGACGGGA
1962	sgHRG_2	HRG	chr3	186383954	+	GCTACCTTTTCCAATTGCTG
1963	sgHRG_3	HRG	chr3	186386814	-	GATGGACGTCTGGAATCAGG
1964	sgHRG_4	HRG	chr3	186389541	-	GATTCGGTCCACTCTGAAAG
1965	sgHRG_5	HRG	chr3	186389500	+	AGCCCTTGAGAAGTACAAAG
1966	sgHRG_6	HRG	chr3	186390563	+	CCATCTGTTCTAGAGAGGAG
1967	sgHRG_7	HRG	chr3	186390584	+	GGAAGGAACTGGTTACTTCG
1968	sgHRG_8	HRG	chr3	186383885	-	CTCAACAGCACTGCAGTCAG
1969	sgHRG_9	HRG	chr3	186392904	+	GTTCTATGATGTAGAAGCCT
1970	sgHRG_10	HRG	chr3	186383982	+	TGATGCCCACTTGGACAGAG
1971	sgHSPBAP1_1	HSPBAP1	chr3	122496570	-	GGGATGAAAAGCATGAGCAC
1972	sgHSPBAP1_2	HSPBAP1	chr3	122459852	-	AGCCTGGTTAAACCCCACTG
1973	sgHSPBAP1_3	HSPBAP1	chr3	122474114	-	CATGCGGTTACACTGAGCCC
1974	sgHSPBAP1_4	HSPBAP1	chr3	122459942	-	GGAAGAGGATCACCTAGCCC
1975	sgHSPBAP1_5	HSPBAP1	chr3	122512492	+	GATCACAGGAGTGGTCGCCT
1976	sgHSPBAP1_6	HSPBAP1	chr3	122487601	+	CATAAGCCCAGAACTTGGAA
1977	sgHSPBAP1_7	HSPBAP1	chr3	122478151	-	ATGGACAGGAAAGTACATTG
1978	sgHSPBAP1_8	HSPBAP1	chr3	122512478	-	GTGATCGTTGCGGCTGGGGC
1979	sgHSPBAP1_9	HSPBAP1	chr3	122496625	-	GAATGCTAAATACCTTTCGC
1980	sgHSPBAP1_10	HSPBAP1	chr3	122478165	-	GTTTCCTGGAAGAAATGGAC
1981	sgIDO1_1	IDO1	chr8	39775468	+	GCTTCGAGAAAGAGTTGAGA
1982	sgIDO1_2	IDO1	chr8	39775666	+	AGTTCTGGGATGCATCACCA
1983	sgIDO1_3	IDO1	chr8	39775686	-	TTTGCCCCACACATATGCCA
1984	sgIDO1_4	IDO1	chr8	39782784	+	AGGGTTCTGGGAAGACCCAA
1985	sgIDO1_5	IDO1	chr8	39782796	+	AGACCCAAAGGAGTTTGCAG
1986	sgIDO1_6	IDO1	chr8	39771435	+	TACAAGAATGGCACACGCTA
1987	sgIDO1_7	IDO1	chr8	39782866	+	GGCATCCAGCAGACTGCTGG
1988	sgIDO1_8	IDO1	chr8	39781008	+	GGCAATGCAAATGCAAGAAC
1989	sgIDO1_9	IDO1	chr8	39782758	-	ACCAGACCGTCTGATAGCTG
1990	sgIDO1_10	IDO1	chr8	39776402	+	GGTTTATGCAGACTGTGTCT
1991	sgINPPL1_1	INPPL1	chr11	71939521	-	GAGGCATCCCGGTCATCCGG
1992	sgINPPL1_2	INPPL1	chr11	71936087	-	CAGGTCGCGGTGGTACCAGG
1993	sgINPPL1_3	INPPL1	chr11	71936168	+	GAGACAGCGAGAGCGTGGCG
1994	sgINPPL1_4	INPPL1	chr11	71936195	-	TCACAGGACGCAGAGCGCGA
1995	sgINPPL1_5	INPPL1	chr11	71939466	-	GCAGGGCGCACACAAGGCCC
1996	sgINPPL1_6	INPPL1	chr11	71939757	+	ACCCCTGCCCCACAGATGGGG
1997	sgINPPL1_7	INPPL1	chr11	71939851	-	GGAGTCTCAGGAGCTGGCAG
1998	sgINPPL1_8	INPPL1	chr11	71940192	+	GACCTGGAAGCTGTGAGGGG
1999	sgINPPL1_9	INPPL1	chr11	71941266	+	GAGACAGCGGGACTCCCAGG
2000	sgINPPL1_10	INPPL1	chr11	71941410	-	GCTGGGACTTAATGAGCTGG
2001	sgIREB2_1	IREB2	chr15	78758623	+	AATGCACCAAATCCTGGAGG
2002	sgIREB2_2	IREB2	chr15	78764122	-	TGAGCCATTCCAGTTCCAGG
2003	sgIREB2_3	IREB2	chr15	78778144	-	GTAATACTCCACTTGAACTG
2004	sgIREB2_4	IREB2	chr15	78780615	-	GCATAAGCTACCACTAAGGG
2005	sgIREB2_5	IREB2	chr15	78782759	+	CACAGATGGGGAATAAACGG
2006	sgIREB2_6	IREB2	chr15	78786260	-	CGTAAGAGTTGAATTCACGA
2007	sgIREB2_7	IREB2	chr15	78755299	+	GCTGCTGTACGAAATTGTGA
2008	sgIREB2_8	IREB2	chr15	78757590	+	TAGTGGAATACCAGCAATGG
2009	sgIREB2_9	IREB2	chr15	78757630	+	GAGGCAGTGAAAACTCTTGG
2010	sgIREB2_10	IREB2	chr15	78758707	+	AGAGGCCAGACTACCTGCCG
2011	sgIRG1_1	IRG1	chr13	77526656	-	GGTGTCCCACTTTCAAGCCA

2012	sgIRG1_2	IRG1	chr13	77529497	-	AAGGACAGCCCCAGAAGGGT
2013	sgIRG1_3	IRG1	chr13	77526646	-	TTTCAAGCCATGGATTGCTG
2014	sgIRG1_4	IRG1	chr13	77527757	-	TGGCTGACCCCAAACAGTGC
2015	sgIRG1_5	IRG1	chr13	77526636	+	AGCTTTGCCACAGCAATCCA
2016	sgIRG1_6	IRG1	chr13	77526748	-	TATGTGAAACACTTCCGTAG
2017	sgIRG1_7	IRG1	chr13	77529461	+	CCATGGATTTTGATGACACG
2018	sgIRG1_8	IRG1	chr13	77529530	+	CTTTAGCAGAAGCCCTGCCA
2019	sgIRG1_9	IRG1	chr13	77529573	-	TGAAAGCCAGCAGCAGGTCA
2020	sgIRG1_10	IRG1	chr13	77529638	+	AGGCCAATGACATGCCAAAG
2021	sgISCA1_1	ISCA1	chr9	88897281	+	GCGAGCACTCACCAGGGTGA
2022	sgISCA1_2	ISCA1	chr9	88889090	+	CTGGTTTTACTTACATGCTC
2023	sgISCA1_3	ISCA1	chr9	88897341	+	CCGGACAGTTGCCCGGACTA
2024	sgISCA1_4	ISCA1	chr9	88897329	-	CTGTCCGGGCTGTGAGCAAG
2025	sgISCA1_5	ISCA1	chr9	88897294	+	AGGGTGAGGGCTGCCCGGGT
2026	sgISCA1_6	ISCA1	chr9	88897355	-	GATGTCGGCTTCCTTAGTCC
2027	sgISCA1_7	ISCA1	chr9	88880986	-	CCAAACATCAAAGGGACTTG
2028	sgISCA1_8	ISCA1	chr9	88886999	-	AAAGTTGGTGTCCGAACCAG
2029	sgISCA1_9	ISCA1	chr9	88886980	+	AGAAAGGCCATTACAGCCCC
2030	sgISCA1_10	ISCA1	chr9	88897310	-	GAGGAAGCTGCAGCCCACCC
2031	sgISCA2_1	ISCA2	chr14	74960978	+	AGGCTTTTGGAAATCACCGA
2032	sgISCA2_2	ISCA2	chr14	74960521	+	GAGCGGTCACTCCCTGGCCG
2033	sgISCA2_3	ISCA2	chr14	74960834	-	GGACGCAACTGTCTGTGAGG
2034	sgISCA2_4	ISCA2	chr14	74960818	-	GAGGCGGATCTGCCCTTCGC
2035	sgISCA2_5	ISCA2	chr14	74961007	+	ATTCCTCAGGCTGCAAGTGG
2036	sgISCA2_6	ISCA2	chr14	74960472	+	AGGAAGATGGCTGCCGCCTG
2037	sgISCA2_7	ISCA2	chr14	74960504	+	AACGGCCGCGACGCAGAGAG
2038	sgISCA2_8	ISCA2	chr14	74960535	-	TGGTACCTGCCCCTCGGCCA
2039	sgISCA2_9	ISCA2	chr14	74960796	+	TCGTCCTCCAGCCCCGAGGC
2040	sgISCA2_10	ISCA2	chr14	74961023	+	GTGGAGGGAGGTGGATGCTC
2041	sgISCU_1	ISCU	chr12	108961023	-	GAGCAGTGCAGTTTCACGGG
2042	sgISCU_2	ISCU	chr12	108958111	+	AAATGTTGGAACTGGACTGG
2043	sgISCU_3	ISCU	chr12	108959170	+	CCAGCTCATTAGCCACTGAA
2044	sgISCU_4	ISCU	chr12	108959185	-	CTTTCCTTTCACCCATTCAG
2045	sgISCU_5	ISCU	chr12	108961006	-	GGGAGGAAGGCAGAGCTCCT
2046	sgISCU_6	ISCU	chr12	108959105	+	GGATGAAAAGGGGAAGATTG
2047	sgISCU_7	ISCU	chr12	108962617	+	AGATGCAATCAAGGCCGCCC
2048	sgISCU_8	ISCU	chr12	108962634	-	CAATTTGTAATCAGCCAGGG
2049	sgISCU_9	ISCU	chr12	108956386	+	GCAAGCCGGCAAGATGGCGG
2050	sgISCU_10	ISCU	chr12	108956491	+	GGCCCGACTCTATCACAAGA
2051	sgJAK2_1	JAK2	chr9	5054759	+	GAAGTAAAAGAACCTGGAAG
2052	sgJAK2_2	JAK2	chr9	5081779	-	AAAGGCACCAGAAAACCCCA
2053	sgJAK2_3	JAK2	chr9	5064975	-	GAGGTGCTACTTCTTTACAG
2054	sgJAK2_4	JAK2	chr9	5069973	+	CAACCTCACCAACATTACAG
2055	sgJAK2_5	JAK2	chr9	5089712	+	TCTACAGGACAACACTGGGG
2056	sgJAK2_6	JAK2	chr9	5123100	+	GAAGAGTAAAAGTCCACCAG
2057	sgJAK2_7	JAK2	chr9	5072489	+	AAGAATGAAAGCCTTGGCCA
2058	sgJAK2_8	JAK2	chr9	5080258	-	GGATTTTCAATGCATTCAGG
2059	sgJAK2_9	JAK2	chr9	5080321	+	ACTTTGTGGGAAATCTGCAG
2060	sgJAK2_10	JAK2	chr9	5089772	+	GCACCTAAGAGACTTTGAAA
2061	sgJHDM1D_1	JHDM1D	chr7	139796450	-	ATACAAAGCACAGACTGCAG
2062	sgJHDM1D_2	JHDM1D	chr7	139876722	-	AAAGATGGCCGGAGCGGCGG
2063	sgJHDM1D_3	JHDM1D	chr7	139876611	+	GGCACACACAGTACACGGGC
2064	sgJHDM1D_4	JHDM1D	chr7	139826607	-	AAGGATGTCTGAATTGGTGG
2065	sgJHDM1D_5	JHDM1D	chr7	139796364	-	GTATCGCTATGATAAACCAG
2066	sgJHDM1D_6	JHDM1D	chr7	139791656	+	GGGGTGAAGGGGCACCGAGA
2067	sgJHDM1D_7	JHDM1D	chr7	139824478	-	GGTGTTCTTTGGAGATAAGG
2068	sgJHDM1D_8	JHDM1D	chr7	139798712	-	GAGTTCAGGAGTAGAGGGAG
2069	sgJHDM1D_9	JHDM1D	chr7	139791789	-	AGGAATTATGTGGACAGCAG
2070	sgJHDM1D_10	JHDM1D	chr7	139826468	-	GATTTCCACATTGACTTCGG
2071	sgJMJD1C_1	JMJD1C	chr10	64975053	-	AGATGAAGAGAAGATGAAGG
2072	sgJMJD1C_2	JMJD1C	chr10	64968961	+	GGGGTGGGCTGATGTAGCCA

2073	sgJMJD1C_3	JMJD1C	chr10	64967968	+	GGTTGGTGTTTAATCAAAGG
2074	sgJMJD1C_4	JMJD1C	chr10	64966866	+	AGTGCTATCCAGAGGAAGGG
2075	sgJMJD1C_5	JMJD1C	chr10	64966509	-	GTGAAAGCAAGTCAGAGCAA
2076	sgJMJD1C_6	JMJD1C	chr10	64960378	-	CAAGAAGCTGAAGCAAACTG
2077	sgJMJD1C_7	JMJD1C	chr10	64952730	+	AGATCTGCTAACCAGTGCAG
2078	sgJMJD1C_8	JMJD1C	chr10	64974858	-	TAAAAGAAGGAAACCTGAGG
2079	sgJMJD1C_9	JMJD1C	chr10	64973987	+	GGTTTAACAACTTCAGGTGA
2080	sgJMJD1C_10	JMJD1C	chr10	64973756	+	GTTTCTGACCCAATAAGTGC
2081	sgJMJD4_1	JMJD4	chr1	227923024	+	GAAACGGTGAGGACCAGCCT
2082	sgJMJD4_2	JMJD4	chr1	227922834	+	GCCCCGCACAAAGTCGGCGT
2083	sgJMJD4_3	JMJD4	chr1	227922793	+	AAGGCGCTGGAAAACACGCA
2084	sgJMJD4_4	JMJD4	chr1	227922407	-	CTGGAAAGAGTACATACAGG
2085	sgJMJD4_5	JMJD4	chr1	227922388	-	GCGGGCTACTCCTCTCCCAG
2086	sgJMJD4_6	JMJD4	chr1	227922375	+	GTAGAGACAGCCCCTGGGAG
2087	sgJMJD4_7	JMJD4	chr1	227921331	+	GACATTGACAGACCAGCTGA
2088	sgJMJD4_8	JMJD4	chr1	227921211	-	GCGACACACACCTGCACCCA
2089	sgJMJD4_9	JMJD4	chr1	227921143	-	GAGATGGTGTTTGTGCCCAG
2090	sgJMJD4_10	JMJD4	chr1	227923069	-	GCGCTGGCGGGGCAGAAACG
2091	sgJMJD6_1	JMJD6	chr17	74722440	-	GAGCTTCTCGCTGAGCCCGG
2092	sgJMJD6_2	JMJD6	chr17	74721859	-	TGAAAGACCTTACAAGCCCG
2093	sgJMJD6_3	JMJD6	chr17	74721750	-	AAGTGTGGTGAGGATAACGA
2094	sgJMJD6_4	JMJD6	chr17	74721563	-	AGTATGCTGGGGAGAAGCGC
2095	sgJMJD6_5	JMJD6	chr17	74719985	-	AAAGTGACCCGAGACGAAGG
2096	sgJMJD6_6	JMJD6	chr17	74717935	-	CAGCAGCACCAACTTCCCTG
2097	sgJMJD6_7	JMJD6	chr17	74714920	-	GAGTCTGGATCCGAGGGCGA
2098	sgJMJD6_8	JMJD6	chr17	74714892	-	TGCACCGCAGGAAGAAGAGG
2099	sgJMJD6_9	JMJD6	chr17	74714876	-	GAGGAGGACGTGCAGCATGG
2100	sgJMJD6_10	JMJD6	chr17	74722527	-	GAGCAAGAAGCGCATCCGCG
2101	sgJMJD7_1	JMJD7	chr15	42120378	-	GAGCCACTCACCCCTTGCAG
2102	sgJMJD7_2	JMJD7	chr15	42127080	-	GCTCCCACCTGAAATAGGGG
2103	sgJMJD7_3	JMJD7	chr15	42128722	+	GGTGGTGGATGAAGAGGCCA
2104	sgJMJD7_4	JMJD7	chr15	42126980	-	GTAGAAGTGGAGCGGAGTTG
2105	sgJMJD7_5	JMJD7	chr15	42127064	-	GGGGAGGGACCACTTCTGGA
2106	sgJMJD7_6	JMJD7	chr15	42127267	-	GCACGAAGCTCAGGGGCAGG
2107	sgJMJD7_7	JMJD7	chr15	42128704	+	TGAAGAGGGCACCTTTAAGG
2108	sgJMJD7_8	JMJD7	chr15	42120365	+	GAATTCCCGGCCGCTGCAAG
2109	sgJMJD7_9	JMJD7	chr15	42126993	-	AGACCCAGTCCCGGTAGAAG
2110	sgJMJD7_10	JMJD7	chr15	42127177	+	GGGCTCCACAGAGGTGAGTG
2111	sgJMJD8_1	JMJD8	chr16	734109	-	GGAGGAGGAGCGCTGCACGG
2112	sgJMJD8_2	JMJD8	chr16	734291	+	AGCGCGAGCAACCGCGACGC
2113	sgJMJD8_3	JMJD8	chr16	734070	+	GCACGAACTCCGCGTAGGTG
2114	sgJMJD8_4	JMJD8	chr16	733882	+	CAGGATGACGGGCCTGACGA
2115	sgJMJD8_5	JMJD8	chr16	733563	+	GCAGCTGCTCCACATACTCC
2116	sgJMJD8_6	JMJD8	chr16	733404	-	GGGACAACAACTTCACCGAG
2117	sgJMJD8_7	JMJD8	chr16	733377	+	GGAGTAGTGCCGAAAGAGAG
2118	sgJMJD8_8	JMJD8	chr16	734344	-	AAGGTTCCCGGCGTGCAGCG
2119	sgJMJD8_9	JMJD8	chr16	733863	-	GCAGGGACTCACGGACAACT
2120	sgJMJD8_10	JMJD8	chr16	733748	+	GCAACCTGTCGCGGGAGCAC
2121	sgKDM2A_1	KDM2A	chr11	67012738	-	GGCCTCACAGGTGTTAACCG
2122	sgKDM2A_2	KDM2A	chr11	67012769	-	GGCTCCTGACACAATCGGGG
2123	sgKDM2A_3	KDM2A	chr11	67017614	-	GGCTCATCGCAGCTCCGCAG
2124	sgKDM2A_4	KDM2A	chr11	67017645	-	GGAAGTGGGTGAATGAGGCG
2125	sgKDM2A_5	KDM2A	chr11	67017660	-	GATGAGCTGCAGCATGGAAG
2126	sgKDM2A_6	KDM2A	chr11	67017698	-	GACCGAGTCACCATACCCCG
2127	sgKDM2A_7	KDM2A	chr11	67017759	+	GCGATGAGCGCTTCAAACGG
2128	sgKDM2A_8	KDM2A	chr11	67017796	+	GCAGGCCACAGAGCGCACCA
2129	sgKDM2A_9	KDM2A	chr11	67017824	+	GAAAAGGAGAACAATCCCAG
2130	sgKDM2A_10	KDM2A	chr11	67017978	-	GGAATGGCGAAGGTTGGCAG
2131	sgKDM2B_1	KDM2B	chr12	121947456	-	GAAGGGCCTGAAAGCTCTGG
2132	sgKDM2B_2	KDM2B	chr12	121883161	-	GGCGGGGAAGGAAGACACGG
2133	sgKDM2B_3	KDM2B	chr12	122016716	-	GGACTTCGTGCACGCCATGG

2134	sgKDM2B_4	KDM2B	chr12	121958860	+	GAACACCAAAGAGTCTACAG
2135	sgKDM2B_5	KDM2B	chr12	121947786	-	GGATTCCTGGCTGGAGATGG
2136	sgKDM2B_6	KDM2B	chr12	121947750	-	TCAGCAGCCTCAGGAGGAGG
2137	sgKDM2B_7	KDM2B	chr12	121947728	-	GAGAAGGACGAGGAGGGCGA
2138	sgKDM2B_8	KDM2B	chr12	121947695	-	GCACCCAAACCGCCCACCGA
2139	sgKDM2B_9	KDM2B	chr12	121891082	+	GTTGGCGGCCAACTTCACCG
2140	sgKDM2B_10	KDM2B	chr12	121883183	+	GCCACACACAAGGCACACGG
2141	sgKDM3A_1	KDM3A	chr2	86705092	-	CATACTCCAAACCCACACCG
2142	sgKDM3A_2	KDM3A	chr2	86709796	+	GGAGTGCTGGAAACAAGGGC
2143	sgKDM3A_3	KDM3A	chr2	86683668	+	TCTTCAAGTCAACTGTGAGG
2144	sgKDM3A_4	KDM3A	chr2	86693826	-	GGTGCATTTGAAACATCCGA
2145	sgKDM3A_5	KDM3A	chr2	86707440	+	TGGCTGGCCGACCTAACCAG
2146	sgKDM3A_6	KDM3A	chr2	86709112	-	GTGCTGGATTTGCTTAAAGG
2147	sgKDM3A_7	KDM3A	chr2	86709195	-	AAGAATTCAAGAGATTCCGG
2148	sgKDM3A_8	KDM3A	chr2	86669231	+	TCCGCAGCCGACGGCAGCGA
2149	sgKDM3A_9	KDM3A	chr2	86669297	-	CGAATGGTCCCGGAGAGCCA
2150	sgKDM3A_10	KDM3A	chr2	86676929	+	GGTGTGTGTGGAATTTGATG
2151	sgKDM3B_1	KDM3B	chr5	137721855	+	GTAGACAGTAATGGGAGCGA
2152	sgKDM3B_2	KDM3B	chr5	137722303	-	GAAGTAACTCACCTCTCCCG
2153	sgKDM3B_3	KDM3B	chr5	137713493	+	CTACAAGAGATATTCAGCCG
2154	sgKDM3B_4	KDM3B	chr5	137715298	+	GGTCAAAATATATCAGCCAG
2155	sgKDM3B_5	KDM3B	chr5	137721766	+	TAGAGGGGAAAGATGGCCGG
2156	sgKDM3B_6	KDM3B	chr5	137721873	+	GATGGAGGTGAGGCAAGCCG
2157	sgKDM3B_7	KDM3B	chr5	137721910	+	GGAATGCCAGTGGAGAGCCA
2158	sgKDM3B_8	KDM3B	chr5	137722219	-	GATCCTCACTGTGTTTGGGG
2159	sgKDM3B_9	KDM3B	chr5	137727119	-	GGGAAGGCTGGAGTGAGGGT
2160	sgKDM3B_10	KDM3B	chr5	137727826	+	GGGAATTCCAGAGCACCTGA
2161	sgKDM4A_1	KDM4A	chr1	44160384	+	GTATCTTCTGTAAGAAGCGG
2162	sgKDM4A_2	KDM4A	chr1	44121260	+	AGGTTGTTCCTCCAAAAGAG
2163	sgKDM4A_3	KDM4A	chr1	44131409	-	GAGACACAGTACCTTTGGCG
2164	sgKDM4A_4	KDM4A	chr1	44132690	-	ACTCCGCACAGTTAAAACCA
2165	sgKDM4A_5	KDM4A	chr1	44133637	+	AGAGGAGGACATGGAAGGGG
2166	sgKDM4A_6	KDM4A	chr1	44134908	-	GGTGGGCCTCACAGGGGCGA
2167	sgKDM4A_7	KDM4A	chr1	44137269	-	AGGATTCACAGAAAGATCCA
2168	sgKDM4A_8	KDM4A	chr1	44154610	+	GGAAGAGGCTGAGGAGACAG
2169	sgKDM4A_9	KDM4A	chr1	44128607	+	GACTTGGTGGAAAAGGAGAG
2170	sgKDM4A_10	KDM4A	chr1	44133596	+	AGTGAACTGCCTCCAAGAGC
2171	sgKDM4B_1	KDM4B	chr19	5039882	+	GTATGATGACATCGACGACG
2172	sgKDM4B_2	KDM4B	chr19	5041141	+	TCACCAGGTACTGTACCCCG
2173	sgKDM4B_3	KDM4B	chr19	5041208	-	AGATCGGGGAGACAAAGGTG
2174	sgKDM4B_4	KDM4B	chr19	5082418	-	GAAGCCGGCGTGGTAGCCGT
2175	sgKDM4B_5	KDM4B	chr19	5082478	-	GTAGTCAATCCACCGCAGGG
2176	sgKDM4B_6	KDM4B	chr19	5110805	-	GAGGAGCTTGGCCTTCAGCG
2177	sgKDM4B_7	KDM4B	chr19	5119787	+	GCCGGAGGTTGACCCCGAGG
2178	sgKDM4B_8	KDM4B	chr19	5131491	+	GAGGACGGTGCAGCCAGCAG
2179	sgKDM4B_9	KDM4B	chr19	5131936	+	GAAGAGCCGGCGCCATCCCC
2180	sgKDM4B_10	KDM4B	chr19	5133994	+	CGAGAGGAAGTTCAACGCAG
2181	sgKDM4C_1	KDM4C	chr9	6849632	+	TGCATGGCACACCGAAGACA
2182	sgKDM4C_2	KDM4C	chr9	6981097	+	AAGTAAGAAAAGCATCCCGA
2183	sgKDM4C_3	KDM4C	chr9	6986452	-	ATAGCCACTAGACAATGGAA
2184	sgKDM4C_4	KDM4C	chr9	6986546	+	GAGTAATGGTGTGTTAACAG
2185	sgKDM4C_5	KDM4C	chr9	7011705	+	TGAGGTTCTGTCCATTGAGG
2186	sgKDM4C_6	KDM4C	chr9	6793039	+	GACCTTCAGACCCTCCATGG
2187	sgKDM4C_7	KDM4C	chr9	6793056	-	GAACTCCCGGAACTCCTCCA
2188	sgKDM4C_8	KDM4C	chr9	6805597	+	AGGTGATTCCTCCTAAGGAG
2189	sgKDM4C_9	KDM4C	chr9	6849549	+	GATGTGGTTGAAGAAGAGTG
2190	sgKDM4C_10	KDM4C	chr9	6981070	+	TAAAAGCATGGCTGCAGAGG
2191	sgKDM4D_1	KDM4D	chr11	94731631	+	GTTACCCAGGAGAGCAGCGC
2192	sgKDM4D_2	KDM4D	chr11	94731730	-	GTGAAGAGCACACAAGGGTG
2193	sgKDM4D_3	KDM4D	chr11	94731792	-	GCTGTGGACAGCAGCAGCCC
2194	sgKDM4D_4	KDM4D	chr11	94730639	+	ATTGCTTACATGGAATCCCA

2195	sgKDM4D_5	KDM4D	chr11	94731243	+	GGGGTTGTGGGGCCTTCCTG
2196	sgKDM4D_6	KDM4D	chr11	94731388	+	CCATGGTTTCAACTGCGCAG
2197	sgKDM4D_7	KDM4D	chr11	94731531	+	GCTATGACCTGTGGAAACGT
2198	sgKDM4D_8	KDM4D	chr11	94731671	-	GAATGCCGGGCCCAGTGGGA
2199	sgKDM4D_9	KDM4D	chr11	94730775	-	AAACACCCCTGCCCGCCCAG
2200	sgKDM4D_10	KDM4D	chr11	94731020	+	GACCTGCTGGAAAAGGAATG
2201	sgKDM4E_1	KDM4E	chr11	94759419	+	GGGGCTGTGAGGCCTTCCTG
2202	sgKDM4E_2	KDM4E	chr11	94759683	-	GGGTTGCACAATGCGCACAA
2203	sgKDM4E_3	KDM4E	chr11	94759743	+	TTGTGGAACACACAGAGCCC
2204	sgKDM4E_4	KDM4E	chr11	94759964	+	CAGACCCAGTCACTTACCCT
2205	sgKDM4E_5	KDM4E	chr11	94759983	-	AACCCTGGCTGACATCCCCA
2206	sgKDM4E_6	KDM4E	chr11	94760198	-	TCATTGGCAAGCGGGAGCTG
2207	sgKDM4E_7	KDM4E	chr11	94759884	-	TGGGTTGTAACAGTGCCCTG
2208	sgKDM4E_8	KDM4E	chr11	94758856	-	TGGGTGGAATTACCTTGGCA
2209	sgKDM4E_9	KDM4E	chr11	94760094	+	GTTGTTGTACTCGAGAACTG
2210	sgKDM4E_10	KDM4E	chr11	94760172	+	GGAGTAGAGCTCAAGGCCAC
2211	sgKDM5A_1	KDM5A	chr12	418974	-	GGAAACCAGAGATACAGCCA
2212	sgKDM5A_2	KDM5A	chr12	498193	+	AAGACGGGGCACTCTGGCGG
2213	sgKDM5A_3	KDM5A	chr12	463244	-	GTGTCCTAAATGTGTCGCCG
2214	sgKDM5A_4	KDM5A	chr12	459789	-	AAAGATTCTGCCAGAAGAAG
2215	sgKDM5A_5	KDM5A	chr12	442718	+	GCTGGGATTCAAATAACTCG
2216	sgKDM5A_6	KDM5A	chr12	430235	-	GACTCGGACCAAACTGACAG
2217	sgKDM5A_7	KDM5A	chr12	419007	-	GGAGCCTCTGAGTGATCTGG
2218	sgKDM5A_8	KDM5A	chr12	416691	-	CCAGCGTATGGTGGAACAGG
2219	sgKDM5A_9	KDM5A	chr12	416216	-	GCAGTCTGCTTTTAACCGGG
2220	sgKDM5A_10	KDM5A	chr12	406288	-	GATTCCCATCAAATCCGAGG
2221	sgKDM5B_1	KDM5B	chr1	202699063	-	AGGGCCTCTCCAGTGAGCGG
2222	sgKDM5B_2	KDM5B	chr1	202777408	-	GCGGCCACCACACTGCACCC
2223	sgKDM5B_3	KDM5B	chr1	202777278	+	GATCTTGTGGATGAAAGCGA
2224	sgKDM5B_4	KDM5B	chr1	202742307	-	GCAGTGGGCTCACATATCAG
2225	sgKDM5B_5	KDM5B	chr1	202710607	-	GAGACGTCTCATAGACCTAG
2226	sgKDM5B_6	KDM5B	chr1	202710580	-	GCTGGCCCCGTATTCAGCAG
2227	sgKDM5B_7	KDM5B	chr1	202702918	-	GCTGTCGCCTCTCCAAGATG
2228	sgKDM5B_8	KDM5B	chr1	202702681	+	GAAGTGCATCTCCCTCAGGA
2229	sgKDM5B_9	KDM5B	chr1	202700039	-	AGTGAGACCCAGCAGTGAGA
2230	sgKDM5B_10	KDM5B	chr1	202699083	-	GAAGAGACGCCTGGAAAGAG
2231	sgKDM5C_1	KDM5C	chrX	53230806	-	GAAGCTAGACCTGAACCTGG
2232	sgKDM5C_2	KDM5C	chrX	53247085	+	GGGCTACCCGAGCCCACCGA
2233	sgKDM5C_3	KDM5C	chrX	53231087	+	GAAGCCGCTGTGGTAAGCAC
2234	sgKDM5C_4	KDM5C	chrX	53227776	-	AGTCTGAAGCCCGTGAGCGG
2235	sgKDM5C_5	KDM5C	chrX	53227721	-	GAACTGCCTGAGTGAGGCAG
2236	sgKDM5C_6	KDM5C	chrX	53226208	-	GGGTGTTCTGGAACAGGTGG
2237	sgKDM5C_7	KDM5C	chrX	53226062	-	GGCAGGTGGAACAGGCGCGA
2238	sgKDM5C_8	KDM5C	chrX	53224200	-	ACAGCACCAAGCGCAGCCGG
2239	sgKDM5C_9	KDM5C	chrX	53224182	-	GGTGGATGGAGAAGGAGCTG
2240	sgKDM5C_10	KDM5C	chrX	53223905	-	GCAGATCGTGGCCTTCAAGG
2241	sgKDM5D_1	KDM5D	chrY	21877290	-	GAAGAACTGCCTGAGTGAGG
2242	sgKDM5D_2	KDM5D	chrY	21905050	-	CCAAAGGCTAAATGAACTGG
2243	sgKDM5D_3	KDM5D	chrY	21897624	-	AAAACAGCCAGAGCCTACAG
2244	sgKDM5D_4	KDM5D	chrY	21897341	-	GTGAAGGATGAGCAAAGTGG
2245	sgKDM5D_5	KDM5D	chrY	21894504	+	CTGGGGATTTCAGGAAGGGG
2246	sgKDM5D_6	KDM5D	chrY	21878296	+	GGGAGAAGACACAATAGCGC
2247	sgKDM5D_7	KDM5D	chrY	21877522	-	AGTGCGAGTGGCCTTGGAGG
2248	sgKDM5D_8	KDM5D	chrY	21871680	-	GCAGGATGTCCTGGAACAGG
2249	sgKDM5D_9	KDM5D	chrY	21871604	+	AACAGGGACCGCAATAGCCC
2250	sgKDM5D_10	KDM5D	chrY	21871465	-	GCTCTCTGGTCATCATGCAG
2251	sgKDM6A_1	KDM6A	chrX	44928888	-	GAGATGCTGGGAAGGCCCAG
2252	sgKDM6A_2	KDM6A	chrX	44732857	+	TGAGGAAAAGAAAATGGCGG
2253	sgKDM6A_3	KDM6A	chrX	44732927	+	GAGGAGAGGGAGGCGCTCGG
2254	sgKDM6A_4	KDM6A	chrX	44913099	+	CCTGGGAGATAAAGCCACCA
2255	sgKDM6A_5	KDM6A	chrX	44918501	+	TCAGCAGCAAAATCAGCCCA

2256	sgKDM6A_6	KDM6A	chrX	44922669	+	GAGACCAACAGGAGTTGCAC
2257	sgKDM6A_7	KDM6A	chrX	44923013	+	CCAGCAGCGCAGAGGAGCCG
2258	sgKDM6A_8	KDM6A	chrX	44732844	-	TTCCTCATCACCGAAAGCGG
2259	sgKDM6A_9	KDM6A	chrX	44732870	+	ATGGCGGCGGGAAAAGCGAG
2260	sgKDM6A_10	KDM6A	chrX	44732884	+	AGCGAGCGGCGAGAGCGAGG
2261	sgKDM6B_1	KDM6B	chr17	7750215	-	GTAGCCAGGCCAGGCAGGGG
2262	sgKDM6B_2	KDM6B	chr17	7750652	-	GGCGTAAGGCACGCAGGCGG
2263	sgKDM6B_3	KDM6B	chr17	7749265	-	GAGCATAATATGGTTTGCTG
2264	sgKDM6B_4	KDM6B	chr17	7749569	+	GAGGAAGCTTCGCTGAGCTG
2265	sgKDM6B_5	KDM6B	chr17	7749986	+	ACTCTCAGGCCCCTCAGGGG
2266	sgKDM6B_6	KDM6B	chr17	7750015	+	TCAGCCCTGGAGGCAAGCGA
2267	sgKDM6B_7	KDM6B	chr17	7750891	+	GCGCTGGAGGTCTCTCACCA
2268	sgKDM6B_8	KDM6B	chr17	7750967	-	GGATAGGTGGGGAGTGGCAG
2269	sgKDM6B_9	KDM6B	chr17	7751169	-	GCCCCAAGAAGCCCTCGCGA
2270	sgKDM6B_10	KDM6B	chr17	7751191	-	ACAGAAAAGCGGGAGGCCGG
2271	sgKDM8_1	KDM8	chr16	27225051	+	GGCCGTGCATGCAGAAGTGG
2272	sgKDM8_2	KDM8	chr16	27221509	-	GTGCGGCAGGAGCGCCCTGA
2273	sgKDM8_3	KDM8	chr16	27221558	+	GGAGAAAGTGGAGAGGAGCG
2274	sgKDM8_4	KDM8	chr16	27221630	+	CGAGTGTCTGCAGAGCAGCG
2275	sgKDM8_5	KDM8	chr16	27221858	-	GGATGGCAGCGACTTTAAGA
2276	sgKDM8_6	KDM8	chr16	27224983	-	ACTGCTCCCTGAAATGCTGG
2277	sgKDM8_7	KDM8	chr16	27225019	+	GCCCGTGATCCTGAAAGGCG
2278	sgKDM8_8	KDM8	chr16	27226221	+	TGGCTGCCGAACTGTCCCAG
2279	sgKDM8_9	KDM8	chr16	27226272	+	ATGGTCCCAGACCCTCATGA
2280	sgKDM8_10	KDM8	chr16	27230378	-	AGTGGGGAGATGGTTCCCTG
2281	sgKIAA1456_1	KIAA1456	chr8	12870221	+	GAACAGCCAGGTACATACCG
2282	sgKIAA1456_2	KIAA1456	chr8	12870281	+	GAATAGAGGATGTGAAGCCA
2283	sgKIAA1456_3	KIAA1456	chr8	12863785	+	GCGACCTGCAGAGCAAAGCC
2284	sgKIAA1456_4	KIAA1456	chr8	12863806	-	GAACTGGCGGACACGAGGCC
2285	sgKIAA1456_5	KIAA1456	chr8	12863819	-	TCTGCTCTTGCAGGAACTGG
2286	sgKIAA1456_6	KIAA1456	chr8	12870237	+	ACCGTGGGCTGTGACTACTG
2287	sgKIAA1456_7	KIAA1456	chr8	12870267	+	GTAGAGATTGCCCGGAATAG
2288	sgKIAA1456_8	KIAA1456	chr8	12870345	+	GATGCCATCATCTCCATAGG
2289	sgKIAA1456_9	KIAA1456	chr8	12863728	-	ATGCTGCTTCTCCAGCTGGG
2290	sgKIAA1456_10	KIAA1456	chr8	12863714	+	GGATCATGAAGCCGCCCAGC
2291	sgKLKB1_1	KLKB1	chr4	187173328	-	ACACTTACCCATCAAAGCAG
2292	sgKLKB1_2	KLKB1	chr4	187173266	+	GCTCAGAGGCACCTGTGTGG
2293	sgKLKB1_3	KLKB1	chr4	187175751	-	AGATGCGCCAAACATCCTGC
2294	sgKLKB1_4	KLKB1	chr4	187178471	-	AGCCAGCACAGACCATCCGT
2295	sgKLKB1_5	KLKB1	chr4	187153301	+	GAAAACGCCTTCTTCAGAGG
2296	sgKLKB1_6	KLKB1	chr4	187171487	-	GTTGGGGTGATAGGTGCAGA
2297	sgKLKB1_7	KLKB1	chr4	187171505	-	GAATGTAAAGAAGAGGCAGT
2298	sgKLKB1_8	KLKB1	chr4	187172420	-	GGTGTTTTCTTGAGGAGTAG
2299	sgKLKB1_9	KLKB1	chr4	187173288	+	GGTCACTCATAGGACACCAG
2300	sgKLKB1_10	KLKB1	chr4	187177200	+	CCAACTGTTGGGTAACCGGA
2301	sgLCN2_1	LCN2	chr9	130911875	-	GGCTGGGATCAGGTCTGAGG
2302	sgLCN2_2	LCN2	chr9	130911898	-	AGAGGGACCTTGCTCAGAGG
2303	sgLCN2_3	LCN2	chr9	130913962	+	CCAGCCCGGCGAGTTCACGC
2304	sgLCN2_4	LCN2	chr9	130912641	-	CCCTCACCTAAACAGGACGG
2305	sgLCN2_5	LCN2	chr9	130911846	+	GGCTCTGCATGCCCAGGCCC
2306	sgLCN2_6	LCN2	chr9	130912503	+	TCAGTCCCTTGCAGTTCCAG
2307	sgLCN2_7	LCN2	chr9	130912522	-	CTACCACATACCACTTCCCC
2308	sgLCN2_8	LCN2	chr9	130914210	-	TGGTGCTCACCACTCGGACG
2309	sgLCN2_9	LCN2	chr9	130914508	-	GGCCCAGAGATTTGGAGAAG
2310	sgLCN2_10	LCN2	chr9	130911812	-	GCCCAGCCACAGGAGACCTA
2311	sgLEPRE1_1	LEPRE1	chr1	43213001	-	GGAGTGAAGGCTGTCACCAG
2312	sgLEPRE1_2	LEPRE1	chr1	43232391	-	AGTGTGCCGCCGACTTCCCG
2313	sgLEPRE1_3	LEPRE1	chr1	43224922	-	GAAGGGCCCTATGACTACGA
2314	sgLEPRE1_4	LEPRE1	chr1	43215881	+	GATGAGAGTAGGAAAAGTAG
2315	sgLEPRE1_5	LEPRE1	chr1	43213897	+	GGGCTCTTTGACACACACGA
2316	sgLEPRE1_6	LEPRE1	chr1	43213033	+	AGTGCCTGAAGAGAATCCCA

2317	sgLEPRE1_7	LEPRE1	chr1	43232606	-	GAAGCTGCTGACCACACTGC
2318	sgLEPRE1_8	LEPRE1	chr1	43232316	+	GAAGCTCAGGTCGCGCAGGG
2319	sgLEPRE1_9	LEPRE1	chr1	43232195	-	GAGCCCCTACAACTACCTGC
2320	sgLEPRE1_10	LEPRE1	chr1	43224992	-	AGCTGTGCCCCACCTAGAGG
2321	sgLEPREL1_1	LEPREL1	chr3	189700837	-	ATAGATCGAGACCTAAGAGA
2322	sgLEPREL1_2	LEPREL1	chr3	189711951	-	GAATGCCGGACCCTATGTGA
2323	sgLEPREL1_3	LEPREL1	chr3	189681805	-	GGGGTGAAGGCAGTCACCAA
2324	sgLEPREL1_4	LEPREL1	chr3	189706750	+	TGGGAGAGAGGCGGCCAGGG
2325	sgLEPREL1_5	LEPREL1	chr3	189706713	+	TGTAGGTAATCATAGTGCAG
2326	sgLEPREL1_6	LEPREL1	chr3	189692447	+	GTGATGTTCTCATAGAGTAG
2327	sgLEPREL1_7	LEPREL1	chr3	189692355	-	GTGCCGGGAGCTCCACAGCG
2328	sgLEPREL1_8	LEPREL1	chr3	189691774	-	GGAATCATGCTTGTTGGTGA
2329	sgLEPREL1_9	LEPREL1	chr3	189681788	-	CAAGGGAAAGAGGTGTGCTG
2330	sgLEPREL1_10	LEPREL1	chr3	189706767	-	AGGGAACTTGCCACCCGCCC
2331	sgLIAS_1	LIAS	chr4	39460732	+	GAGGAAATGTCTCTACGCTG
2332	sgLIAS_2	LIAS	chr4	39460754	+	GGGATGCAGCCCGCACCCTG
2333	sgLIAS_3	LIAS	chr4	39463834	+	GCTAAAGACAGAGATTCCCA
2334	sgLIAS_4	LIAS	chr4	39466732	-	GGCTCACTGGCATCCAGTGG
2335	sgLIAS_5	LIAS	chr4	39469245	+	AAACAGTCCCGGAATTACAG
2336	sgLIAS_6	LIAS	chr4	39465218	-	GGCTGGCCCTACCATGATCG
2337	sgLIAS_7	LIAS	chr4	39471655	-	GACTGATCAAAATTGGCCCG
2338	sgLIAS_8	LIAS	chr4	39462481	-	GAAGGTCTGGTCCATTCTGT
2339	sgLIAS_9	LIAS	chr4	39462554	+	GGAAACCTAAAACGCCAGAA
2340	sgLIAS_10	LIAS	chr4	39466746	+	TGAGCCCTACAATACTGCAA
2341	sgLPO_1	LPO	chr17	56320352	-	GGAGGGCTGGGAGATGGAGA
2342	sgLPO_2	LPO	chr17	56329841	-	GAAGCAGGGCACACGGGCAG
2343	sgLPO_3	LPO	chr17	56332208	-	GAGAAAGAGGGTGTGGGATG
2344	sgLPO_4	LPO	chr17	56332257	+	AAAGAGACTCAACCCTCAGT
2345	sgLPO_5	LPO	chr17	56332325	-	GGGACTCCCTACCTGCACGA
2346	sgLPO_6	LPO	chr17	56342201	-	GTGGCCAAAGCGGAAGGCGA
2347	sgLPO_7	LPO	chr17	56342247	-	GCTGATAATTCTCATCCAGG
2348	sgLPO_8	LPO	chr17	56326481	-	GCACGGGTCGCATCTCACCA
2349	sgLPO_9	LPO	chr17	56327840	+	GGTCCCTGCTCTTCATGCAG
2350	sgLPO_10	LPO	chr17	56327937	-	GGATACAGTACTCATCACAC
2351	sgLTF_1	LTF	chr3	46486841	-	GAAGTCCTGCCACACCGCCG
2352	sgLTF_2	LTF	chr3	46497426	-	GTGGCTGTGGTGAAGAAGGG
2353	sgLTF_3	LTF	chr3	46490461	-	GCGGGTCGTGTGGTGTGCGG
2354	sgLTF_4	LTF	chr3	46490342	+	GGAGCTCCCTACCAGCACCA
2355	sgLTF_5	LTF	chr3	46484942	-	GAGATACTACGGCTACACTG
2356	sgLTF_6	LTF	chr3	46497857	-	GATGCTGTGACCCTTGATGG
2357	sgLTF_7	LTF	chr3	46497804	-	CAAACTGCGACCTGTAGCGG
2358	sgLTF_8	LTF	chr3	46497445	-	ACGAACTCACTATTATGCCG
2359	sgLTF_9	LTF	chr3	46492020	-	ACGAAGTGTGAATGGCAAGG
2360	sgLTF_10	LTF	chr3	46491987	-	GAATCTTCTCCGCCAGGCAC
2361	sgMAP2_1	MAP2	chr2	210517903	+	TGAACGGAAAGATGAAGCAA
2362	sgMAP2_2	MAP2	chr2	210517936	+	GACCTCAGCACCGCTAACAG
2363	sgMAP2_3	MAP2	chr2	210517991	+	GATCAAGGCGGAGCAGGGGA
2364	sgMAP2_4	MAP2	chr2	210574693	-	GAGTGATGGCAGTAGACCCA
2365	sgMAP2_5	MAP2	chr2	210594651	+	AATGCTCATCATGTACCTGG
2366	sgMAP2_6	MAP2	chr2	210517979	+	CCTGAGATTAAGGATCAAGG
2367	sgMAP2_7	MAP2	chr2	210518029	+	CAATGGATTCCCATACAGGG
2368	sgMAP2_8	MAP2	chr2	210518135	+	TCAGCTGACAGAGAAACAGC
2369	sgMAP2_9	MAP2	chr2	210570367	-	CCTGACACACCTCGCCGAGG
2370	sgMAP2_10	MAP2	chr2	210574765	-	GAGGGGTCCTGGGATAGCTA
2371	sgMB_1	MB	chr22	36007080	-	GAAGTCAGAGGACGAGATGA
2372	sgMB_2	MB	chr22	36006933	-	GATCCCCGTGAAGTACCTGG
2373	sgMB_3	MB	chr22	36013231	-	GAGGCTGACATCCCAGGCCA
2374	sgMB_4	MB	chr22	36007027	+	ACCCAGGGCGGTGAGCACGG
2375	sgMB_5	MB	chr22	36013216	+	AGGACTTCCTGCCCATGGCC
2376	sgMB_6	MB	chr22	36007015	+	CTTAAGGATGCCACCCAGGG
2377	sgMB_7	MB	chr22	36006961	+	GGTGGCATGCGACTGTGCCA

2378	sgMB_8	MB	chr22	36013291	-	TGCGCCATGGGGCTCAGCGA
2379	sgMB_9	MB	chr22	36006999	-	TAAGAAGAAGGGGCATCATG
2380	sgMB_10	MB	chr22	36013262	-	GCAGTTGGTGCTGAACGTCT
2381	sgMETAP1_1	METAP1	chr4	99955431	+	ATTAATACTGACCCATGGGC
2382	sgMETAP1_2	METAP1	chr4	99955446	-	GTATATCGATAACCTGCCCA
2383	sgMETAP1_3	METAP1	chr4	99964472	+	GTGAATGAAGTCATTTGCCA
2384	sgMETAP1_4	METAP1	chr4	99916903	+	GCATGGCGGCCGTGGAGACG
2385	sgMETAP1_5	METAP1	chr4	99916975	-	GATGCCCAGCTTGATGCAAG
2386	sgMETAP1_6	METAP1	chr4	99962203	+	GAAGTTTTGGATGTTGCTGC
2387	sgMETAP1_7	METAP1	chr4	99962218	+	GCTGCCGGCATGATTAAACC
2388	sgMETAP1_8	METAP1	chr4	99964488	+	GCCATGGAATACCAGACAGA
2389	sgMETAP1_9	METAP1	chr4	99916997	-	GCGAGCAGAAGTACGAGCCC
2390	sgMETAP1_10	METAP1	chr4	99916934	+	GACAGACGGCTGCAGCAGTG
2391	sgMETAP1D_1	METAP1D	chr2	172928564	-	AACTCTTCCCAGCCAAGAGG
2392	sgMETAP1D_2	METAP1D	chr2	172930407	-	GGAAAACCTCCATAGCCTAG
2393	sgMETAP1D_3	METAP1D	chr2	172930444	-	ACAGAGCACGTTGTTTACAG
2394	sgMETAP1D_4	METAP1D	chr2	172943944	+	AAACGACAGTGATCTACCCA
2395	sgMETAP1D_5	METAP1D	chr2	172944851	+	TACAGAGCCAATCATCACGG
2396	sgMETAP1D_6	METAP1D	chr2	172864895	+	GTCCACCTGCTCGTCCGCAG
2397	sgMETAP1D_7	METAP1D	chr2	172926351	-	TGAGCTGAAGAAACTGCAGC
2398	sgMETAP1D_8	METAP1D	chr2	172928465	+	GACAGGCATTGTACCAGACT
2399	sgMETAP1D_9	METAP1D	chr2	172935745	+	AATTGCAGCTTGCAGAGCAG
2400	sgMETAP1D_10	METAP1D	chr2	172944893	+	CCTGGAGGATGCATGGACTG
2401	sgMFI2_1	MFI2	chr3	196753555	-	GGAGCACGGCCTGAAGCCGG
2402	sgMFI2_2	MFI2	chr3	196754759	-	TCGGTGGCATGGAGGTGCGG
2403	sgMFI2_3	MFI2	chr3	196754710	-	CAAGTGCGGCAACATGAGCG
2404	sgMFI2_4	MFI2	chr3	196754657	+	GGTGCCCCGGACGCAGAGGA
2405	sgMFI2_5	MFI2	chr3	196749840	-	GAGAGATACTACGACTACAG
2406	sgMFI2_6	MFI2	chr3	196748277	-	ACGGTACTGGAGAACACGGA
2407	sgMFI2_7	MFI2	chr3	196754729	+	GTGCTGCTCTGGGTCCGAGG
2408	sgMFI2_8	MFI2	chr3	196754633	+	GAGCTGGACGCAGTGGTCGG
2409	sgMFI2_9	MFI2	chr3	196753582	-	TGGAGGAGCCATCTATGAGG
2410	sgMFI2_10	MFI2	chr3	196751313	+	GCTCCTCCTGACCACAGCCA
2411	sgMINA_1	MINA	chr3	97664644	-	GGGAAATGAGGAGGAAACAG
2412	sgMINA_2	MINA	chr3	97686356	-	GATGAAGTTAGAAGCAGCTG
2413	sgMINA_3	MINA	chr3	97680473	-	AATGTGTACATAACTCCCGC
2414	sgMINA_4	MINA	chr3	97677957	+	GTGCCAGGGGCACAGTGGGG
2415	sgMINA_5	MINA	chr3	97668790	-	GACACTTGCAGACCGGCTGG
2416	sgMINA_6	MINA	chr3	97686392	-	GAGTGGGAAGGAAGAGGGGC
2417	sgMINA_7	MINA	chr3	97677942	-	GGCACGAGAGTACAGCGTGG
2418	sgMINA_8	MINA	chr3	97677926	-	GTGGAGGCCGAGGAAAGGAT
2419	sgMINA_9	MINA	chr3	97673314	-	GATTTGTTGTACTTTCCCAG
2420	sgMINA_10	MINA	chr3	97669668	-	TGATACTGCAAAGGAAGACG
2421	sgMIOX_1	MIOX	chr22	50927518	-	GGAGTCGCAGAAAACCACGG
2422	sgMIOX_2	MIOX	chr22	50926419	-	GGAAGGCATGGAAGGAGTTG
2423	sgMIOX_3	MIOX	chr22	50927667	+	GGGATGTATCAGCCCCACTG
2424	sgMIOX_4	MIOX	chr22	50927684	-	GGACCCTGTCGAGCCCACAG
2425	sgMIOX_5	MIOX	chr22	50925320	+	TCTCAGGATGAAGGTGACGG
2426	sgMIOX_6	MIOX	chr22	50926347	+	AATGACAGTCATGGAGGCCG
2427	sgMIOX_7	MIOX	chr22	50926456	+	CGGAAGGCCCACCCAGACAA
2428	sgMIOX_8	MIOX	chr22	50926714	-	CGTGCAGGAGCCCGACGAGG
2429	sgMIOX_9	MIOX	chr22	50925837	+	CCGACCTGATGTGGACCCAG
2430	sgMIOX_10	MIOX	chr22	50926100	-	GTGAAGACACGGTCCAGGAG
2431	sgMOCS1_1	MOCS1	chr6	39880638	-	GGAGGAATCCAGCACAGCCA
2432	sgMOCS1_2	MOCS1	chr6	39893569	-	TCAGTACTGCATGCCCGAGG
2433	sgMOCS1_3	MOCS1	chr6	39893463	-	GACAAGATCCGGCTCACAGG
2434	sgMOCS1_4	MOCS1	chr6	39883800	+	ACATCCGCTCACCTTTCCTG
2435	sgMOCS1_5	MOCS1	chr6	39881520	-	GCTGGGCTACAACCCTGTGA
2436	sgMOCS1_6	MOCS1	chr6	39880668	-	GCAGTGGCCAGAGCTGGAGA
2437	sgMOCS1_7	MOCS1	chr6	39877667	-	GAAACTCTGAGGTATCCCTG
2438	sgMOCS1_8	MOCS1	chr6	39893434	-	GCTTATCCGGCCGGACGTGG

2439	sgMOCS1_9	MOCS1	chr6	39880684	-	TAGACACTGTCCGGCAGCAG
2440	sgMOCS1_10	MOCS1	chr6	39880656	-	GCTGGAGAAGGTGCCAGAGG
2441	sgMOXD1_1	MOXD1	chr6	132649220	+	AGGGATAATCCAACATGAGG
2442	sgMOXD1_2	MOXD1	chr6	132722468	+	GAGTCCAGGAGGGTCCGGTG
2443	sgMOXD1_3	MOXD1	chr6	132722394	+	CTGCAGTGCGCACCTGGAGG
2444	sgMOXD1_4	MOXD1	chr6	132722338	+	GACGATGTCGGCGGACGCCA
2445	sgMOXD1_5	MOXD1	chr6	132643907	-	GCGTCATTTTCGAAAAGGGA
2446	sgMOXD1_6	MOXD1	chr6	132722328	-	CGACATCGTCGTGGGCGGGG
2447	sgMOXD1_7	MOXD1	chr6	132649200	+	GCGGATCTAATGGAGTGCCA
2448	sgMOXD1_8	MOXD1	chr6	132649154	-	GATAATCCCACTTATGAGGA
2449	sgMOXD1_9	MOXD1	chr6	132722457	+	ACTTGCCCTCCGAGTCCAGG
2450	sgMOXD1_10	MOXD1	chr6	132722302	+	CTGGAGGTAGGGCCGCCCGT
2451	sgMPO_1	MPO	chr17	56356738	+	GCAGCCAGCGCACAAAGGCA
2452	sgMPO_2	MPO	chr17	56356666	-	CAAGCGCAACGGCTTCCCGG
2453	sgMPO_3	MPO	chr17	56357971	+	GCAGCACCTTCAGAGGGCTG
2454	sgMPO_4	MPO	chr17	56357773	-	GGTGCTGAGCTCCATGGAGG
2455	sgMPO_5	MPO	chr17	56357758	-	GGAGGAGGCCAAGCAGCTGG
2456	sgMPO_6	MPO	chr17	56357735	-	ACAAGGCCTACAAGGAGCGG
2457	sgMPO_7	MPO	chr17	56357290	-	GGTGGCAGCCACCAGGACGG
2458	sgMPO_8	MPO	chr17	56356886	+	GTTGTTGCACATCCCGGTGA
2459	sgMPO_9	MPO	chr17	56356772	-	GCAGACGCAGCCCCACGCTG
2460	sgMPO_10	MPO	chr17	56355312	-	GCAACATGTCCAACCAGCTG
2461	sgMPPED1_1	MPPED1	chr22	43821111	+	CAGCCGGCTCATCATCGAGG
2462	sgMPPED1_2	MPPED1	chr22	43821192	-	GCACATGCGGTGGCTGGAAG
2463	sgMPPED1_3	MPPED1	chr22	43894197	+	GTAGACATCCTGATAACCCA
2464	sgMPPED1_4	MPPED1	chr22	43898536	+	GGGTCCCCAAGAAGATGCAG
2465	sgMPPED1_5	MPPED1	chr22	43821012	+	TGCCAGCGTCCTGAAGGCGG
2466	sgMPPED1_6	MPPED1	chr22	43821051	-	ATGCCATGCCCAGGCCGCAG
2467	sgMPPED1_7	MPPED1	chr22	43821155	-	GTAGAAGGTGAAGGCCTGGG
2468	sgMPPED1_8	MPPED1	chr22	43821166	+	ACCTTCTACAACATCAACCA
2469	sgMPPED1_9	MPPED1	chr22	43821202	-	ACTTACATCTGCACATGCGG
2470	sgMPPED1_10	MPPED1	chr22	43831076	+	CTGGGGACTTCACTGAGCTG
2471	sgMRE11A_1	MRE11A	chr11	94180387	-	GGAGGGTCTCAAAGAGGAAG
2472	sgMRE11A_2	MRE11A	chr11	94203781	-	GTTTGCTGCGTATTAAAGGG
2473	sgMRE11A_3	MRE11A	chr11	94180459	-	GCAGCAACCAACAAAGGAAG
2474	sgMRE11A_4	MRE11A	chr11	94212842	-	CAATCATGACGATCCCACAG
2475	sgMRE11A_5	MRE11A	chr11	94180435	-	CGAGGAAGAGGTCGAAGAGG
2476	sgMRE11A_6	MRE11A	chr11	94180408	-	GGGCAGAATTCAGCATCGAG
2477	sgMRE11A_7	MRE11A	chr11	94197392	-	TTTCAGGTGGACTATAGTGG
2478	sgMRE11A_8	MRE11A	chr11	94194104	-	GTACTTTCAAACCGCAGAGA
2479	sgMRE11A_9	MRE11A	chr11	94192576	-	CGAAGACAAAATCGATGAGG
2480	sgMRE11A_10	MRE11A	chr11	94224057	-	ATGGAGAAAGATGCAGTCAG
2481	sgMSMO1_1	MSMO1	chr4	166259068	+	ATTGGGAAAGAATGCCAAGA
2482	sgMSMO1_2	MSMO1	chr4	166261409	-	CCAAGAATTAGAGTCTCCAA
2483	sgMSMO1_3	MSMO1	chr4	166261366	+	GTCTTAGGCTCCATTTGGAA
2484	sgMSMO1_4	MSMO1	chr4	166262975	-	TTCCAATGAAGTTCATGTGG
2485	sgMSMO1_5	MSMO1	chr4	166258936	+	CGTAGGATAAGCCAGAGACA
2486	sgMSMO1_6	MSMO1	chr4	166254658	+	AAGTTCCAGATTGCAACATG
2487	sgMSMO1_7	MSMO1	chr4	166262942	-	GCCGAGAACCAGCATAGAAA
2488	sgMSMO1_8	MSMO1	chr4	166258948	+	CAGAGACATGGGAAAACCAA
2489	sgMSMO1_9	MSMO1	chr4	166254566	-	ATCTACATATTCCACAGCCA
2490	sgMSMO1_10	MSMO1	chr4	166254552	+	TAGTTCAGCATCCTTGGCTG
2491	sgMUTYH_1	MUTYH	chr1	45800156	-	GAGGAAGCCACGAGCAGCCG
2492	sgMUTYH_2	MUTYH	chr1	45799210	-	GGCCAGGCAGCCGGAAGAGG
2493	sgMUTYH_3	MUTYH	chr1	45799175	+	TAGATGGTATGAGGAGACAG
2494	sgMUTYH_4	MUTYH	chr1	45798487	-	GAGGTGAATCAACTCTGGGC
2495	sgMUTYH_5	MUTYH	chr1	45797704	-	GTCGGGCAGTCCTGACGTGG
2496	sgMUTYH_6	MUTYH	chr1	45797094	-	GCACCTCCGGCACCTTGGGG
2497	sgMUTYH_7	MUTYH	chr1	45800115	-	AGGCAGCCAGCCAGGAAGGG
2498	sgMUTYH_8	MUTYH	chr1	45799142	-	CTGAAGTCACAGCCTTCCGA
2499	sgMUTYH_9	MUTYH	chr1	45798781	+	GGTATAGTAGTTGATCACAG

2500	sgMUTYH_10	MUTYH	chr1	45798459	-	GCTACTATTCTCGTGGCCGG
2501	sgNARF_1	NARF	chr17	80430563	-	GGGCTCACCAGGACAGGCAG
2502	sgNARF_2	NARF	chr17	80438984	-	AACAAGGGGCCACAATGACG
2503	sgNARF_3	NARF	chr17	80439043	-	CCGGGAGCCATGCAAAGCAG
2504	sgNARF_4	NARF	chr17	80442686	+	GAGGTTTGGAGACTTGAAGG
2505	sgNARF_5	NARF	chr17	80442779	+	GGAGCTGTTCAACGAGGATG
2506	sgNARF_6	NARF	chr17	80443490	+	GTTCCCATTCCACTTTGTGG
2507	sgNARF_7	NARF	chr17	80426723	-	TGGATGCATCAGTTACACTG
2508	sgNARF_8	NARF	chr17	80436747	-	AAAGAGCCCATGACCTGCTG
2509	sgNARF_9	NARF	chr17	80430516	+	CTATCGCCAGCACAGTGAGG
2510	sgNARF_10	NARF	chr17	80430548	-	GGCAGAGGTCAGCATGGGCA
2511	sgNARFL_1	NARFL	chr16	782314	+	GGGCTGGTTCCAGGTCGGGG
2512	sgNARFL_2	NARFL	chr16	781661	-	GAGCCCACCAGCCATCGGGG
2513	sgNARFL_3	NARFL	chr16	780984	-	CAGGAACAAAGACTTCCAGG
2514	sgNARFL_4	NARFL	chr16	787234	+	GATAAGCACGGTCTCTGCGG
2515	sgNARFL_5	NARFL	chr16	787200	+	GAACCTTCTTCAGCTCCTCG
2516	sgNARFL_6	NARFL	chr16	784729	+	GAGCACACCTGGGCAGGCAG
2517	sgNARFL_7	NARFL	chr16	782292	+	CATACGTACAGGCTGTCCAG
2518	sgNARFL_8	NARFL	chr16	781683	-	GTGCAGCGGTGCCTCTGCAG
2519	sgNARFL_9	NARFL	chr16	780939	-	GGTGCTGCTGCACTTCGCAA
2520	sgNARFL_10	NARFL	chr16	787299	-	CCGGAGGCTGGAGAAGGCCA
2521	sgNDUFS1_1	NDUFS1	chr2	207011765	-	GATTTGGGAACAACAGGCAG
2522	sgNDUFS1_2	NDUFS1	chr2	207007515	-	GATGTGGCAGCAATTGCAGG
2523	sgNDUFS1_3	NDUFS1	chr2	207006687	+	ACAGTGGTGCCTCAAAACGT
2524	sgNDUFS1_4	NDUFS1	chr2	206997786	+	GAACTGCCTAAAACCACCAT
2525	sgNDUFS1_5	NDUFS1	chr2	206994878	+	GGAGAAACAGCACCTTGGGA
2526	sgNDUFS1_6	NDUFS1	chr2	207007531	-	GAGTTTTCAAGGCAAAGATG
2527	sgNDUFS1_7	NDUFS1	chr2	206997711	-	CAAAAGATTCGGATGACTAG
2528	sgNDUFS1_8	NDUFS1	chr2	207012312	-	GCTGTGGAAGACAAGAACAT
2529	sgNDUFS1_9	NDUFS1	chr2	207009688	-	ATTGTGGTTAGCACAAGAAC
2530	sgNDUFS1_10	NDUFS1	chr2	207009675	-	CAAGAACTGGAGAAGTGATG
2531	sgNDUFS2_1	NDUFS2	chr1	161172168	+	GGAGTAAGATGGCGGCGCTG
2532	sgNDUFS2_2	NDUFS2	chr1	161183230	-	GCAGTATATGTGGCTCCTGG
2533	sgNDUFS2_3	NDUFS2	chr1	161173292	+	CAAGCAAAGAAACAGCCCAC
2534	sgNDUFS2_4	NDUFS2	chr1	161173324	-	GGTCACTCACCATTCCAAGG
2535	sgNDUFS2_5	NDUFS2	chr1	161176288	+	GATGGAATTGAGTGGGGAGA
2536	sgNDUFS2_6	NDUFS2	chr1	161179307	-	GTGTGGTCACAGCCATGATG
2537	sgNDUFS2_7	NDUFS2	chr1	161180151	+	GCAGAAGAAGCACTTAACTA
2538	sgNDUFS2_8	NDUFS2	chr1	161180432	-	GGTCGTAAACATCATAGGGC
2539	sgNDUFS2_9	NDUFS2	chr1	161182138	+	GAGGTACCTGTGCCGGGTGG
2540	sgNDUFS2_10	NDUFS2	chr1	161183480	-	TGATCTTGCATCGATAAGGG
2541	sgNDUFS7_1	NDUFS7	chr19	1388866	+	GCTCCCAAACCCAGCAGCCG
2542	sgNDUFS7_2	NDUFS7	chr19	1391143	+	GCTACGTGGTCTCCATGGGG
2543	sgNDUFS7_3	NDUFS7	chr19	1388880	+	CAGCCGGGGCGAGTATGTGG
2544	sgNDUFS7_4	NDUFS7	chr19	1391158	-	GCCCTGCACTCACCTCCCCA
2545	sgNDUFS7_5	NDUFS7	chr19	1387820	-	CAAGGATCCGGAAGCCGCGC
2546	sgNDUFS7_6	NDUFS7	chr19	1390859	+	GGCCTCCGCAGAGTTCTCTG
2547	sgNDUFS7_7	NDUFS7	chr19	1390886	-	GCAGGCCAGGCCGAAGGTCA
2548	sgNDUFS7_8	NDUFS7	chr19	1390944	+	CGACATGGACCGCTTTGGCG
2549	sgNDUFS7_9	NDUFS7	chr19	1393276	-	GCAGCCCCTCACCACCGAGT
2550	sgNDUFS7_10	NDUFS7	chr19	1388512	+	ATCTCCCGGCAGCTCCAGCG
2551	sgNDUFS8_1	NDUFS8	chr11	67799635	-	GGCCCGCAGCAGCATAGGCG
2552	sgNDUFS8_2	NDUFS8	chr11	67799769	+	GAGCCTCCACAGCAGTGCAG
2553	sgNDUFS8_3	NDUFS8	chr11	67800657	-	CATGCTCCCCACGGAAGCGA
2554	sgNDUFS8_4	NDUFS8	chr11	67800397	+	GAACATGCAGGATCCCGAGA
2555	sgNDUFS8_5	NDUFS8	chr11	67800456	-	GAAGAGCTCAGTCCACAGCA
2556	sgNDUFS8_6	NDUFS8	chr11	67800678	+	GCTGCGCCGGTACCCATCCG
2557	sgNDUFS8_7	NDUFS8	chr11	67803742	+	CAAGAGCTGATGGCAGCCGC
2558	sgNDUFS8_8	NDUFS8	chr11	67803800	+	CTACTGCGGCTTCTGCCAGG
2559	sgNDUFS8_9	NDUFS8	chr11	67799656	-	TGCACGTGCAGCCTGGGCCA
2560	sgNDUFS8_10	NDUFS8	chr11	67799758	-	TGGAGGCTCCGGCCACCAGG

2561	sgNDUFV1_1	NDUFV1	chr11	67375928	+	TGTACGGCCGCCATGACTGG
2562	sgNDUFV1_2	NDUFV1	chr11	67377051	-	GATGTAGATATAGGCAGCGC
2563	sgNDUFV1_3	NDUFV1	chr11	67378533	-	CAAACCAGGTACCTCCACGG
2564	sgNDUFV1_4	NDUFV1	chr11	67378917	-	GTGGGGTAGACGAGCCGCCA
2565	sgNDUFV1_5	NDUFV1	chr11	67379387	-	GAACTCAATGAGGCGGGCGA
2566	sgNDUFV1_6	NDUFV1	chr11	67379595	+	GGACTGGATGAACAAGGTGA
2567	sgNDUFV1_7	NDUFV1	chr11	67376059	+	GACAAAGGAGATCCTGCTGA
2568	sgNDUFV1_8	NDUFV1	chr11	67376933	+	CTGGTGGTGAACGCAGACGA
2569	sgNDUFV1_9	NDUFV1	chr11	67377007	+	GCTGCTGGAAGGCTGCCTGG
2570	sgNDUFV1_10	NDUFV1	chr11	67378965	+	GCTGATGGACTTCGATGCGC
2571	sgNDUFV2_1	NDUFV2	chr18	9126878	+	CTGGCAAAATCCCAAAACCA
2572	sgNDUFV2_2	NDUFV2	chr18	9126898	-	AATAAAGCATACCTTGGCCC
2573	sgNDUFV2_3	NDUFV2	chr18	9102735	+	GCCCGCCATGTTCTTCTCCG
2574	sgNDUFV2_4	NDUFV2	chr18	9126825	+	CCAGGAGGATTTGACAGCTA
2575	sgNDUFV2_5	NDUFV2	chr18	9102774	+	GGCTGGCCTCACCGCCCACT
2576	sgNDUFV2_6	NDUFV2	chr18	9134251	-	CCTGCTTGTACACCAAATCC
2577	sgNDUFV2_7	NDUFV2	chr18	9117866	+	GTTATGCAAAATGGAGCTGG
2578	sgNDUFV2_8	NDUFV2	chr18	9119572	-	TACCTTGTTCATAGCAGAGA
2579	sgNDUFV2_9	NDUFV2	chr18	9134209	-	AAAGAGGTAAGACCTCCAGC
2580	sgNDUFV2_10	NDUFV2	chr18	9134224	+	TCTTTGACTGAACCACCCAA
2581	sgNENF_1	NENF	chr1	212606414	+	AGTGCGGCTTTTCACCGAGG
2582	sgNENF_2	NENF	chr1	212606435	+	GGAGCTGGCCCGCTATGGCG
2583	sgNENF_3	NENF	chr1	212615959	-	ACACCACTTACCCTTTCCGG
2584	sgNENF_4	NENF	chr1	212615945	+	GTTTGATGTCACCTCCGGAA
2585	sgNENF_5	NENF	chr1	212617716	+	ACGGGGAAGGACTCCACTAG
2586	sgNENF_6	NENF	chr1	212617756	-	GGTGAGGTCTGCAGGATCCA
2587	sgNENF_7	NENF	chr1	212617778	-	TAATCTGGCTCACAGTGTCA
2588	sgNENF_8	NENF	chr1	212606322	-	ACCAGGGCCAGCGCTGCCAG
2589	sgNENF_9	NENF	chr1	212617703	-	TCCCCGTCAAGGCATTGTAG
2590	sgNENF_10	NENF	chr1	212617674	+	ACTACAGAGTTTTATGGACG
2591	sgNFS1_1	NFS1	chr20	34260720	-	GGAGGAAGTGGACTACACAG
2592	sgNFS1_2	NFS1	chr20	34263064	-	GTGGAGGCCCTGCAGAGTGG
2593	sgNFS1_3	NFS1	chr20	34263011	-	AGTGCCCACACCCTTAGTGG
2594	sgNFS1_4	NFS1	chr20	34262972	-	TGAGGTGGCACAGCAAGAGA
2595	sgNFS1_5	NFS1	chr20	34262274	-	ACGTTGCCTTATCCTCAGGG
2596	sgNFS1_6	NFS1	chr20	34260738	-	TGGCCGCTTCACTACAGAGG
2597	sgNFS1_7	NFS1	chr20	34286476	+	TCTGCGGGAACCGCAGACTG
2598	sgNFS1_8	NFS1	chr20	34285697	+	AGCATGGCATCAAGCACCCG
2599	sgNFS1_9	NFS1	chr20	34262507	-	GAATCTCAAAGTTGTCAGAG
2600	sgNFS1_10	NFS1	chr20	34287174	-	TTGGAGGCGGGCGGCAGTGG
2601	sgNFU1_1	NFU1	chr2	69627516	-	GCTGCAGTTTTATATTCCGG
2602	sgNFU1_2	NFU1	chr2	69642331	+	GGTGTTTCCTCAGTAACCAG
2603	sgNFU1_3	NFU1	chr2	69627632	-	GGAGGGGATGTAATCTACAA
2604	sgNFU1_4	NFU1	chr2	69642319	-	GAAACACCTTCAGGAGAAGC
2605	sgNFU1_5	NFU1	chr2	69627498	-	GGAGGTAGAAGGCGTAGAAC
2606	sgNFU1_6	NFU1	chr2	69627648	-	AACTGTGCAGGAAGATGGAG
2607	sgNFU1_7	NFU1	chr2	69627596	-	ATTGTACAGCTGAAACTCCA
2608	sgNFU1_8	NFU1	chr2	69642350	-	CTTTGCATCTGGCTTACCCC
2609	sgNFU1_9	NFU1	chr2	69627660	-	ACCTCACAGGCCAACTGTGC
2610	sgNFU1_10	NFU1	chr2	69650702	+	ACAGTAATATACCTAGCCAG
2611	sgNGB_1	NGB	chr14	77735567	+	GTGGTCCAGGAACTCAGGCG
2612	sgNGB_2	NGB	chr14	77734874	-	GGAAGACCTGTCCTCACTGG
2613	sgNGB_3	NGB	chr14	77735588	+	GGAGAGACAGTCCTCTGGGC
2614	sgNGB_4	NGB	chr14	77734842	-	CCAGCCTGGGCAGGAAGCAC
2615	sgNGB_5	NGB	chr14	77734820	+	TGAGCTTCACACCCACTGCC
2616	sgNGB_6	NGB	chr14	77735618	+	GCGGCAGTTGTACTGGAAGA
2617	sgNGB_7	NGB	chr14	77735602	-	CCGCCAGTTCTCCAGCCCAG
2618	sgNGB_8	NGB	chr14	77737201	+	AGGACGGTGCCGTGCTCCAG
2619	sgNGB_9	NGB	chr14	77735639	+	GGGCAGCAGGTCAGGCTCCA
2620	sgNGB_10	NGB	chr14	77734860	+	GGCAAGGTACTCCTCCAGTG
2621	sgNIF3L1_1	NIF3L1	chr2	201756900	+	GGAGGAGGTGCTGCAAAAGA

2622	sgNIF3L1_2	NIF3L1	chr2	201758004	+	GCTCCCAACTACCCTACAGA
2623	sgNIF3L1_3	NIF3L1	chr2	201760092	+	GGAAATTCTGTCACTGGAGA
2624	sgNIF3L1_4	NIF3L1	chr2	201764106	+	AGAGTCTCAAGTCAAAGTCG
2625	sgNIF3L1_5	NIF3L1	chr2	201756795	-	CCCAACTCTCAGCAAACGAG
2626	sgNIF3L1_6	NIF3L1	chr2	201756879	+	TGACCTGACTGAGGAAGTGA
2627	sgNIF3L1_7	NIF3L1	chr2	201757981	-	GGAAGGATGTATGGGCCTGG
2628	sgNIF3L1_8	NIF3L1	chr2	201760029	+	TCAGAAGGCTTTGATGCAGG
2629	sgNIF3L1_9	NIF3L1	chr2	201756813	+	GGACAATGTTGGATTACTGG
2630	sgNIF3L1_10	NIF3L1	chr2	201756945	-	TGGGTCGGAAGATAGGCGGA
2631	sgNOS1_1	NOS1	chr12	117715846	+	GGAGCGGCAGGACATCGAAG
2632	sgNOS1_2	NOS1	chr12	117768815	+	GAGACGAACAGAAATGACAT
2633	sgNOS1_3	NOS1	chr12	117768417	+	GAGGCCCCATCCACTGCCAG
2634	sgNOS1_4	NOS1	chr12	117768279	-	AGAGTCAGCCTCCAAGGCAG
2635	sgNOS1_5	NOS1	chr12	117768222	-	GTGCTGAGCCTTCTCACCAG
2636	sgNOS1_6	NOS1	chr12	117768200	-	GGAGCAGAGGGGTCAAGGGA
2637	sgNOS1_7	NOS1	chr12	117768157	-	GAAAGATATGGGAATCCAGG
2638	sgNOS1_8	NOS1	chr12	117728141	-	CAAGAACTGGGAGACTGAGG
2639	sgNOS1_9	NOS1	chr12	117723087	+	GATGTAGTTGAACATCCCGT
2640	sgNOS1_10	NOS1	chr12	117710303	+	GCATGTTGGACACGGCGGGG
2641	sgNOS2_1	NOS2	chr17	26125713	+	AGGGAAAACTCACCTGGAGG
2642	sgNOS2_2	NOS2	chr17	26115892	+	GATCCTCACATGCCGTGGGG
2643	sgNOS2_3	NOS2	chr17	26109987	+	GGATCCTCCCAATGCAGCGT
2644	sgNOS2_4	NOS2	chr17	26107887	-	CAAGTACGGCCGCTTCGATG
2645	sgNOS2_5	NOS2	chr17	26107829	+	GGTGGGATTTCGAAGAGCTC
2646	sgNOS2_6	NOS2	chr17	26106033	-	GTGGTACGCCCTGCCTGCAG
2647	sgNOS2_7	NOS2	chr17	26105910	-	CCAGCGCTACAACATCCTGG
2648	sgNOS2_8	NOS2	chr17	26101341	+	GTGATGCTCCCAGACATGGG
2649	sgNOS2_9	NOS2	chr17	26101318	+	GCATCTCCTGGTGAAACACG
2650	sgNOS2_10	NOS2	chr17	26099381	-	GACAGGAAAATCAGAGGCGC
2651	sgNOS3_1	NOS3	chr7	150693584	+	GCAGCTGCTGAGTCAGGCCC
2652	sgNOS3_2	NOS3	chr7	150693856	+	GCTCCCAGGCCCACGAACAG
2653	sgNOS3_3	NOS3	chr7	150693878	+	GCTTCAAGAGGTGGAAGCCG
2654	sgNOS3_4	NOS3	chr7	150693979	+	GCTGCGTGGGCCGGATCCAG
2655	sgNOS3_5	NOS3	chr7	150695505	+	ATCAAGTATGCCACCAACCG
2656	sgNOS3_6	NOS3	chr7	150695747	+	AGCCAACGTGGAGATCACCG
2657	sgNOS3_7	NOS3	chr7	150696320	-	GCATGTTGGACACTGCCGGG
2658	sgNOS3_8	NOS3	chr7	150696431	+	TCACCGCTACAACATCCTGG
2659	sgNOS3_9	NOS3	chr7	150697633	+	GTGGAAAGACAAGGCAGCAG
2660	sgNOS3_10	NOS3	chr7	150698378	+	GGAGAATGAGCAGAAGGCCA
2661	sgNOX1_1	NOX1	chrX	100117712	+	AGATAGGCTGGAGAGAATGG
2662	sgNOX1_2	NOX1	chrX	100117461	-	CAGCTACTGAGTTCATCCGG
2663	sgNOX1_3	NOX1	chrX	100106242	+	GCTGGGAGCGGTAAAACCGG
2664	sgNOX1_4	NOX1	chrX	100117385	+	TGCCGTGAATCCCTAAGCCA
2665	sgNOX1_5	NOX1	chrX	100106224	-	GCAGAAGGTTGTGATTACCA
2666	sgNOX1_6	NOX1	chrX	100105329	-	GAATTGCAGATGAACAAGCG
2667	sgNOX1_7	NOX1	chrX	100105312	-	GCGTGGCTTCAGCATGGAAG
2668	sgNOX1_8	NOX1	chrX	100105223	+	GAAATCTTCCTCTGGAGCAG
2669	sgNOX1_9	NOX1	chrX	100104868	-	GGATGTTTTCCAGTATGAAG
2670	sgNOX1_10	NOX1	chrX	100104849	-	GTGGCTGTGCTGGTTGGAGC
2671	sgNOX4_1	NOX4	chr11	89185019	+	GAGGTTAAGAACAGATGCTG
2672	sgNOX4_2	NOX4	chr11	89165995	-	GACAGGGGTCTGCATGGTGG
2673	sgNOX4_3	NOX4	chr11	89155092	+	GCATGTAGAAGACAAAGAAG
2674	sgNOX4_4	NOX4	chr11	89073267	-	GAGATATCCAGTCCTTCCGT
2675	sgNOX4_5	NOX4	chr11	89073252	+	TAAATCTGCAAACCAACGGA
2676	sgNOX4_6	NOX4	chr11	89223629	-	ACTACCTCCACCAGATGTTG
2677	sgNOX4_7	NOX4	chr11	89184949	+	TTACCTTCTGTGATCCTCGG
2678	sgNOX4_8	NOX4	chr11	89133443	+	ATGACTCATGACCGAAATGA
2679	sgNOX4_9	NOX4	chr11	89069054	-	TGTTTATAGGACGTCCTCGG
2680	sgNOX4_10	NOX4	chr11	89184966	-	ACACTCTTGGCTTACCTCCG
2681	sgNOX5_1	NOX5	chr15	69341367	+	GGAGATGGACCAGGCCGAGG
2682	sgNOX5_2	NOX5	chr15	69325448	-	GTGGTTGTGCCAGTAGGCGC

2683	sgNOX5_3	NOX5	chr15	69325506	-	GCCCGAAGAGCAGCACGTGG
2684	sgNOX5_4	NOX5	chr15	69327807	+	GCTGTCCCTCGTGCACACCG
2685	sgNOX5_5	NOX5	chr15	69329397	-	TGAGCAGAAGCCACACGAGG
2686	sgNOX5_6	NOX5	chr15	69318876	+	GTGCCGAGGAGGATGCCAGG
2687	sgNOX5_7	NOX5	chr15	69320643	-	GGGGCTGCCATGGATGAGCA
2688	sgNOX5_8	NOX5	chr15	69324024	+	GAAGCTGGACCAGCTGACGC
2689	sgNOX5_9	NOX5	chr15	69325561	+	GTCATGGTGGCCAAGGGCTG
2690	sgNOX5_10	NOX5	chr15	69327827	-	AAAGTTCACAGTGTGAGCCA
2691	sgNPAS2_1	NPAS2	chr2	101604633	+	GACTCAGCCCCAGCTCGGGG
2692	sgNPAS2_2	NPAS2	chr2	101541619	+	ACAAGTCTGAGAAGAAGCGT
2693	sgNPAS2_3	NPAS2	chr2	101541715	-	GATGACCTTTTCCAACACGG
2694	sgNPAS2_4	NPAS2	chr2	101580532	-	GTTGTCAAAACCATTACAGG
2695	sgNPAS2_5	NPAS2	chr2	101580607	-	TGGTGTTGCCAGACGAACGG
2696	sgNPAS2_6	NPAS2	chr2	101587449	+	CAGTTACGCAGATGTCCGGG
2697	sgNPAS2_7	NPAS2	chr2	101591289	-	AGTGTGTTAAAGTGCTGCCG
2698	sgNPAS2_8	NPAS2	chr2	101591349	-	GAACTTCTTGAGGACGCCGA
2699	sgNPAS2_9	NPAS2	chr2	101598724	+	TCAAAGACCAGCTAGAGCAG
2700	sgNPAS2_10	NPAS2	chr2	101598832	-	GAAGGGGATCACCTGGACGT
2701	sgNR1D1_1	NR1D1	chr17	38250183	-	GCTGGGCCTCTTCACCGCGG
2702	sgNR1D1_2	NR1D1	chr17	38253394	+	GCTCCCATTATAGAAGGAGG
2703	sgNR1D1_3	NR1D1	chr17	38252159	+	GGGTGAGGGGACCGGAGCAG
2704	sgNR1D1_4	NR1D1	chr17	38252138	+	AAACTGGGAGAAGCCCACCA
2705	sgNR1D1_5	NR1D1	chr17	38252071	-	GGAGGATGTGATATCCCAGG
2706	sgNR1D1_6	NR1D1	chr17	38251747	-	GTATGCAGCCCCAGAAGGCA
2707	sgNR1D1_7	NR1D1	chr17	38251229	+	GAGAAAGGTCACGGAAGCCC
2708	sgNR1D1_8	NR1D1	chr17	38253622	+	GGAGCCACTGGAGCCAATGT
2709	sgNR1D1_9	NR1D1	chr17	38253601	+	AGGGCTGGTGCGGCTTGGGG
2710	sgNR1D1_10	NR1D1	chr17	38253418	+	GGATGACGACGAGGAAGATG
2711	sgNR1D2_1	NR1D2	chr3	23997587	+	TATGGAGTTCATGCTTGCGA
2712	sgNR1D2_2	NR1D2	chr3	23996118	-	AGATGGAACAGAAGAGGAGG
2713	sgNR1D2_3	NR1D2	chr3	24003903	+	TCAATGGACAGTTCAAAGGG
2714	sgNR1D2_4	NR1D2	chr3	24006551	+	TCCAGCAGTGAAAGAAGTGG
2715	sgNR1D2_5	NR1D2	chr3	23996079	-	AGAACCCTCACTGTGACAAG
2716	sgNR1D2_6	NR1D2	chr3	24003542	+	ATGATGAACAGCCAGTTCAG
2717	sgNR1D2_7	NR1D2	chr3	24006594	-	TGAGAGAGATCTCTGAACCC
2718	sgNR1D2_8	NR1D2	chr3	24009390	+	TTTACACTCAATGGGAGCAG
2719	sgNR1D2_9	NR1D2	chr3	23996244	+	CAAGCAAATCGAGTGCACCT
2720	sgNR1D2_10	NR1D2	chr3	23997569	+	GTGGCGTCAGGATTCCACTA
2721	sgNTHL1_1	NTHL1	chr16	2097817	+	GCGGTCATGCCGGACTCCTG
2722	sgNTHL1_2	NTHL1	chr16	2094625	+	GCTCACCCTCCAGAAACCGA
2723	sgNTHL1_3	NTHL1	chr16	2093675	-	ATCCTGCAGCAGCACTACGG
2724	sgNTHL1_4	NTHL1	chr16	2090171	-	GAAGGCAACCAAGTCCCCAG
2725	sgNTHL1_5	NTHL1	chr16	2097798	+	CAGCATCCTCGCGCTCAAGG
2726	sgNTHL1_6	NTHL1	chr16	2097715	+	GCAGCCTCTCTTCTCCGGAG
2727	sgNTHL1_7	NTHL1	chr16	2094738	-	AACCAAAGACCAGGTGACGG
2728	sgNTHL1_8	NTHL1	chr16	2094666	-	GCAGACAGATGATGCCACGC
2729	sgNTHL1_9	NTHL1	chr16	2093601	-	CAAGATGGCACACCTGGCTA
2730	sgNTHL1_10	NTHL1	chr16	2090202	-	ACAGAATCGCCAACAGGCTG
2731	sgNUBP1_1	NUBP1	chr16	10855271	+	GAAGCTGCCCATCATCGGGG
2732	sgNUBP1_2	NUBP1	chr16	10855287	+	GGGGTGGTGGAGAACATGAG
2733	sgNUBP1_3	NUBP1	chr16	10855625	+	GATATTCCCTCCCACAACCG
2734	sgNUBP1_4	NUBP1	chr16	10855688	-	GATCCAGGGGCACTCTGCCG
2735	sgNUBP1_5	NUBP1	chr16	10837807	+	TCTCTGCGCAGACTGTCCAG
2736	sgNUBP1_6	NUBP1	chr16	10846514	+	AATGGGATTGGAAGGAGAGC
2737	sgNUBP1_7	NUBP1	chr16	10850545	+	AGTACGTGGAAGACAACCTG
2738	sgNUBP1_8	NUBP1	chr16	10855667	-	GGAGAGGGACCTCCAAGTCC
2739	sgNUBP1_9	NUBP1	chr16	10855701	-	CCACCTATGAGCGGATCCAG
2740	sgNUBP1_10	NUBP1	chr16	10837708	+	CAAAGGCGACGGAATGGAGG
2741	sgNUBP2_1	NUBP2	chr16	1836824	+	GCTGTGCACCAGTGCGACCG
2742	sgNUBP2_2	NUBP2	chr16	1836910	+	GGAGAAGCCGGACGAGGCCG
2743	sgNUBP2_3	NUBP2	chr16	1837715	+	GGAGCTGGACTACCTGGTGG

2744	sgNUBP2_4	NUBP2	chr16	1837986	+	GAAGACGGGCTTGCGGGTGA
2745	sgNUBP2_5	NUBP2	chr16	1836754	+	GCAGGTGGGAATCCTGGATG
2746	sgNUBP2_6	NUBP2	chr16	1836844	-	GGAAGACGGGTGCCCAGCCG
2747	sgNUBP2_7	NUBP2	chr16	1837747	-	GTGCTCATCGGAGGTCCCCG
2748	sgNUBP2_8	NUBP2	chr16	1837774	-	GGGACGCAGGGCTTCTATGG
2749	sgNUBP2_9	NUBP2	chr16	1836556	-	CAGGATGATGTGCCTGACGC
2750	sgNUBP2_10	NUBP2	chr16	1837685	+	AAAGCAGTTTGTGTCCGACG
2751	sgNUBPL_1	NUBPL	chr14	32031310	+	AGAACACAAATCATGTCCCG
2752	sgNUBPL_2	NUBPL	chr14	32031376	+	GTTGTGGCTTCTGGAAAGGG
2753	sgNUBPL_3	NUBPL	chr14	32257016	+	GTAGACATGCCACCAGGAAC
2754	sgNUBPL_4	NUBPL	chr14	32295867	+	GCATTGATGGATGCACACAA
2755	sgNUBPL_5	NUBPL	chr14	32030682	+	GTGTCGCTCCGGGCTGGTGG
2756	sgNUBPL_6	NUBPL	chr14	32030732	-	GGCGCCCACAAACCATCGCT
2757	sgNUBPL_7	NUBPL	chr14	32142735	+	GAGAGGCCTTATGGTAATGT
2758	sgNUBPL_8	NUBPL	chr14	32257028	-	TGCACATCTCCAGTTCCTGG
2759	sgNUBPL_9	NUBPL	chr14	32315682	-	TCATGTTTTGGACAAGGCCA
2760	sgNUBPL_10	NUBPL	chr14	32315761	+	AGGAAACTAGCACAGACCCT
2761	sgOGFOD1_1	OGFOD1	chr16	56485524	+	GATGAATGGGAAGCGGCCAG
2762	sgOGFOD1_2	OGFOD1	chr16	56503957	+	CATGTGGAATGGAGCAGCCG
2763	sgOGFOD1_3	OGFOD1	chr16	56504396	+	CAGGTTTTATGAGAAAGCTG
2764	sgOGFOD1_4	OGFOD1	chr16	56504609	+	TGAGCCAGAGAATAATCAGA
2765	sgOGFOD1_5	OGFOD1	chr16	56487201	-	GGGATCACACAGTGAAGAAA
2766	sgOGFOD1_6	OGFOD1	chr16	56500071	+	GCTGTGCCATGATGATGAGC
2767	sgOGFOD1_7	OGFOD1	chr16	56508813	-	GGGCAAATTCAGCCTTGCTA
2768	sgOGFOD1_8	OGFOD1	chr16	56487239	-	GAAGCCCTTCTAAGAAGTCT
2769	sgOGFOD1_9	OGFOD1	chr16	56500132	-	CCCATGCTCCTGTCCCAGGG
2770	sgOGFOD1_10	OGFOD1	chr16	56500829	-	AAAACCAGTTTGTTCCACGA
2771	sgOGFOD2_1	OGFOD2	chr12	123463070	+	AATCGGACATGCCTAAGGGG
2772	sgOGFOD2_2	OGFOD2	chr12	123463302	+	GCTGCTGCACGAGCTCGGGC
2773	sgOGFOD2_3	OGFOD2	chr12	123461194	+	ACCCCAGCTTGAGCAGGAGG
2774	sgOGFOD2_4	OGFOD2	chr12	123461449	-	GGACACGCTGTACTCAGTCA
2775	sgOGFOD2_5	OGFOD2	chr12	123463375	+	GCCCTGCTGTACCCTGACTG
2776	sgOGFOD2_6	OGFOD2	chr12	123463419	-	ATTTGACCACAAAGGCCCGG
2777	sgOGFOD2_7	OGFOD2	chr12	123461268	+	CGAGTTCCTACCACCCGGCA
2778	sgOGFOD2_8	OGFOD2	chr12	123461281	-	AGACCTCAGGCCGTGCCGGG
2779	sgOGFOD2_9	OGFOD2	chr12	123461293	+	TGAGGTCTACGACTCACTGC
2780	sgOGFOD2_10	OGFOD2	chr12	123461435	-	CAGTCACGGCCAGGAACTCG
2781	sgOGFOD3_1	OGFOD3	chr17	80356087	-	GGAGGGTGCCAACAAGACGG
2782	sgOGFOD3_2	OGFOD3	chr17	80376347	-	GTCCGATGGCTCCTCAGCGG
2783	sgOGFOD3_3	OGFOD3	chr17	80376324	-	GCCGCAACCAAGGCGCCCGA
2784	sgOGFOD3_4	OGFOD3	chr17	80373463	+	ACAGCCTCTGCACCTCCCGC
2785	sgOGFOD3_5	OGFOD3	chr17	80373439	-	GAGGCCGTGGCTAAGGACCG
2786	sgOGFOD3_6	OGFOD3	chr17	80361932	-	GGTCCAGCTCACCATTGCTG
2787	sgOGFOD3_7	OGFOD3	chr17	80356141	-	GTACCTCTCCAACTACCTGG
2788	sgOGFOD3_8	OGFOD3	chr17	80356108	-	AGGGCGGTTCATGTTCATGG
2789	sgOGFOD3_9	OGFOD3	chr17	80373482	-	CCAAGAAGGACCGAGCCCCG
2790	sgOGFOD3_10	OGFOD3	chr17	80373452	-	AGAGGCTGTGGCAGAGGCCG
2791	sgOSGEP_1	OSGEP	chr14	20922739	+	GGCGTGACGTAAGTCCGCCG
2792	sgOSGEP_2	OSGEP	chr14	20920269	+	ACAACAGCCACAGAAACCAG
2793	sgOSGEP_3	OSGEP	chr14	20922756	-	AGGTGCTGGCGAACCCGCGG
2794	sgOSGEP_4	OSGEP	chr14	20920285	+	CCAGTGGGGCACCCATGCCA
2795	sgOSGEP_5	OSGEP	chr14	20917368	-	GGAAACCATCGATATTGCAG
2796	sgOSGEP_6	OSGEP	chr14	20916111	-	AGAGATCACAGAGCGAGCCA
2797	sgOSGEP_7	OSGEP	chr14	20915583	-	GTGATTCTGGGGTTACACAG
2798	sgOSGEP_8	OSGEP	chr14	20920491	-	CGACTGCATTGCATACACCA
2799	sgOSGEP_9	OSGEP	chr14	20920215	+	CAGTGGTTCACACCCACCAA
2800	sgOSGEP_10	OSGEP	chr14	20920198	-	CTGTATAGGCCACATTGAGA
2801	sgP4HA1_1	P4HA1	chr10	74831888	-	ACAGCGACAAAAGATCCAGA
2802	sgP4HA1_2	P4HA1	chr10	74810824	-	GAAATGCTGTGCCGTGGGGA
2803	sgP4HA1_3	P4HA1	chr10	74806686	-	TCAAAGACCTAGCAAAACCA
2804	sgP4HA1_4	P4HA1	chr10	74774018	-	CAGATGCTTTCAAAGAGCTG

2805	sgP4HA1_5	P4HA1	chr10	74828605	+	TGGAAGATTACCCTTTGAGA
2806	sgP4HA1_6	P4HA1	chr10	74806775	-	TCTGGCTCCAGCTAAACAGG
2807	sgP4HA1_7	P4HA1	chr10	74790040	-	ACTAGATGTTTCCACAGCAG
2808	sgP4HA1_8	P4HA1	chr10	74813225	-	GCCCTAAGGCAACTGGATGA
2809	sgP4HA1_9	P4HA1	chr10	74813151	-	GGTATATCAGCAGGGAGACC
2810	sgP4HA1_10	P4HA1	chr10	74810810	+	CCATTTTGATACCCTCCCCA
2811	sgP4HA2_1	P4HA2	chr5	131549623	-	GACCCAGGCACAATTTCCAG
2812	sgP4HA2_2	P4HA2	chr5	131545974	+	TACCAAGGGAGAGCAGGCGG
2813	sgP4HA2_3	P4HA2	chr5	131544913	-	GCTGAGCTAGCAACCCCAGA
2814	sgP4HA2_4	P4HA2	chr5	131530626	+	GCAGCCCACAAGCACAGGGC
2815	sgP4HA2_5	P4HA2	chr5	131553473	-	GAAAGAGTACATCCTTGTGG
2816	sgP4HA2_6	P4HA2	chr5	131546172	-	TGTGGATGACTGCTTTGGGA
2817	sgP4HA2_7	P4HA2	chr5	131546088	-	AAAGCAGCTTGATGCCGGGG
2818	sgP4HA2_8	P4HA2	chr5	131546028	+	ACTGGAAGACAGCATAGCTG
2819	sgP4HA2_9	P4HA2	chr5	131544845	-	CGAGAGCCTCTGTCGTGGGG
2820	sgP4HA2_10	P4HA2	chr5	131543520	+	GTGGGGCCCTGTTGCCATGG
2821	sgP4HA3_1	P4HA3	chr11	74015317	-	GAGGCCAGTGAGAACATCCG
2822	sgP4HA3_2	P4HA3	chr11	74013462	+	GGGGCTGTACAGGTCAGTGA
2823	sgP4HA3_3	P4HA3	chr11	74009307	-	AGAGTGGAAGACAGAGGATG
2824	sgP4HA3_4	P4HA3	chr11	74000133	+	GAAACTCCCGAGAGAGGCTG
2825	sgP4HA3_5	P4HA3	chr11	73997347	+	TGACAGCCTCAGCTACCACG
2826	sgP4HA3_6	P4HA3	chr11	73996949	-	GCTGCTCCAGCCCATCCGGA
2827	sgP4HA3_7	P4HA3	chr11	73996916	+	GAGCAATGTAGGGCTCCAGG
2828	sgP4HA3_8	P4HA3	chr11	73988122	+	CTGTGAGGGCAGCAATGCGG
2829	sgP4HA3_9	P4HA3	chr11	73988064	-	GTGGTGAACTATGGCATCGG
2830	sgP4HA3_10	P4HA3	chr11	74022488	-	GCTGAGGCGGTACCTGCGCG
2831	sgP4HTM_1	P4HTM	chr3	49027773	+	CCACTGCCAGGCTCAGGCGG
2832	sgP4HTM_2	P4HTM	chr3	49027848	-	AGGCGCGCGAGCAGATGCCG
2833	sgP4HTM_3	P4HTM	chr3	49028338	-	GCGGACTTACCGAAGAGCAG
2834	sgP4HTM_4	P4HTM	chr3	49041667	-	GGATGGCACGCATGATGTGG
2835	sgP4HTM_5	P4HTM	chr3	49042348	+	GCAGGTTGTTCGATATGGTG
2836	sgP4HTM_6	P4HTM	chr3	49043516	+	TGGACCTCCGTGACACACGG
2837	sgP4HTM_7	P4HTM	chr3	49027697	+	CAGCGGCGGTGACAGGCCAG
2838	sgP4HTM_8	P4HTM	chr3	49027739	+	AGGAGGCCTCGAGGCCGCAG
2839	sgP4HTM_9	P4HTM	chr3	49027834	-	ATGCCGCGGGGCTTGCACAG
2840	sgP4HTM_10	P4HTM	chr3	49039004	-	GCAGCCGGAAGAGGTCCAGC
2841	sgPAH_1	PAH	chr12	103310882	+	TGGGTTTTCCAGGACCGCAG
2842	sgPAH_2	PAH	chr12	103271263	-	TCAGATTCTCAGCTATGGAG
2843	sgPAH_3	PAH	chr12	103310863	+	AGAGTTTCCTGCCCAAGCCT
2844	sgPAH_4	PAH	chr12	103288654	+	GAGAAGGTCTAGATTCAATG
2845	sgPAH_5	PAH	chr12	103246690	+	GCAGGCCAGCCACAGGTCGG
2846	sgPAH_6	PAH	chr12	103246664	+	GCCACCCAAGAAATCCCGAG
2847	sgPAH_7	PAH	chr12	103237505	+	GCTGTCTTCTCCAGCTCCAG
2848	sgPAH_8	PAH	chr12	103234236	-	GCTACGACCCATACACCCAA
2849	sgPAH_9	PAH	chr12	103310899	-	GGGAGCCAGCATGTCCACTG
2850	sgPAH_10	PAH	chr12	103310851	-	GAAACTCTCTGACTTTGGAC
2851	sgPAPL_1	PAPL	chr19	39589240	-	GGTGTATGTAGAGCTTCCGC
2852	sgPAPL_2	PAPL	chr19	39590919	+	CATGCGGCTCATTGAACCCG
2853	sgPAPL_3	PAPL	chr19	39590939	-	GTACGGCAGGCTGGCAGCCA
2854	sgPAPL_4	PAPL	chr19	39591417	-	GAGAAAGAAATAGACCTCGG
2855	sgPAPL_5	PAPL	chr19	39591448	-	ACTGCCTCTGTACCAAGTGG
2856	sgPAPL_6	PAPL	chr19	39591659	-	GCAGTCGTCCAGATCTGCGT
2857	sgPAPL_7	PAPL	chr19	39597612	-	GGGCCTCGGGAAGACAGCAA
2858	sgPAPL_8	PAPL	chr19	39589142	-	AATTGCACTTCAGAGCGGGT
2859	sgPAPL_9	PAPL	chr19	39589606	+	GCTGTGGCAGTGCGCAGGGC
2860	sgPAPL_10	PAPL	chr19	39589634	-	GGGCCCTGAAGCGGAACCGA
2861	sgPER1_1	PER1	chr17	8053530	+	GACGTACCTGCAGCCACTGG
2862	sgPER1_2	PER1	chr17	8052805	+	GGGCAGGCGAGATGGAGCAG
2863	sgPER1_3	PER1	chr17	8049748	+	GGAGGTGGTATAGGAGGAGG
2864	sgPER1_4	PER1	chr17	8046902	+	CATTGGGGTCACCAAAGGGG
2865	sgPER1_5	PER1	chr17	8045189	-	GGTTTTCTGAGGACCAGCGG

2866	sgPER1_6	PER1	chr17	8052833	+	TAGAAGACTCCCACATCCTG
2867	sgPER1_7	PER1	chr17	8051353	+	GGATGCAGGAACAGGAGCAC
2868	sgPER1_8	PER1	chr17	8050658	+	GCCGACTCCACAGAGTCCCG
2869	sgPER1_9	PER1	chr17	8050605	-	GGGTCCTCCAGTGATAGCAA
2870	sgPER1_10	PER1	chr17	8049431	+	CCAGACAGCGCTGCTGACGG
2871	sgPER3_1	PER3	chr1	7887729	+	AAGTGGGAGGCACAAAGCGA
2872	sgPER3_2	PER3	chr1	7844927	+	GAGACCTCGAGATGCCCCGC
2873	sgPER3_3	PER3	chr1	7845020	+	GCCCCGAGTTCCATCTGCAG
2874	sgPER3_4	PER3	chr1	7870051	+	GCACCGTGTGGAGGAGACGA
2875	sgPER3_5	PER3	chr1	7870600	+	GGGACACGCACAGAACCGAA
2876	sgPER3_6	PER3	chr1	7880574	+	GGACGGTCCATAGACACAGG
2877	sgPER3_7	PER3	chr1	7887334	-	GGTGTGCGGAGAGGAGGCCG
2878	sgPER3_8	PER3	chr1	7887407	-	GGAGGGGAAAAGCTGGGACG
2879	sgPER3_9	PER3	chr1	7890117	-	CCTGCTGGGAGGCAATCCCA
2880	sgPER3_10	PER3	chr1	7890143	-	ACAGTGGCAGTAGGATGGGA
2881	sgPGRMC1_1	PGRMC1	chrX	118370361	-	GCTCTCCAGATCGCTTGGGT
2882	sgPGRMC1_2	PGRMC1	chrX	118370437	-	TGTAGAGCAGGAAGATGCAG
2883	sgPGRMC1_3	PGRMC1	chrX	118370525	-	AAGTCGCGCCGCTTGAGGCG
2884	sgPGRMC1_4	PGRMC1	chrX	118370587	-	TGATGGCCATGAGTATGCGC
2885	sgPGRMC1_5	PGRMC1	chrX	118370612	+	AAGGTGTTCGATGTGACCAA
2886	sgPGRMC1_6	PGRMC1	chrX	118370326	+	CATGGCTGCCGAGGATGTGG
2887	sgPGRMC1_7	PGRMC1	chrX	118374292	+	GCTGGAAGAGATGCATCCAG
2888	sgPGRMC1_8	PGRMC1	chrX	118370553	-	GTCGAAGCGCCGCAGCTCGG
2889	sgPGRMC1_9	PGRMC1	chrX	118374311	-	GCAAAATGTGGCAAGGCCCC
2890	sgPGRMC1_10	PGRMC1	chrX	118374369	-	GGGCAGCAGTGAGGTCAGAA
2891	sgPGRMC2_1	PGRMC2	chr4	129208958	-	GGGGAGGAGGAGGAAGGCGC
2892	sgPGRMC2_2	PGRMC2	chr4	129208932	-	GCAGTGATGGCGGCTGGTGA
2893	sgPGRMC2_3	PGRMC2	chr4	129208878	-	AGCGAGAGCAGCAACGACGG
2894	sgPGRMC2_4	PGRMC2	chr4	129208652	+	CATGCGAGGCAGAGAGGTGG
2895	sgPGRMC2_5	PGRMC2	chr4	129208942	-	GCGCTGGCGGGCAGTGATGG
2896	sgPGRMC2_6	PGRMC2	chr4	129208768	-	AATGCTGCTGAACGTGGCGC
2897	sgPGRMC2_7	PGRMC2	chr4	129208611	-	GAGCAGCTGCGCCAGTACGA
2898	sgPGRMC2_8	PGRMC2	chr4	129208722	-	TACCGGCTGTGGGTGCGCTG
2899	sgPGRMC2_9	PGRMC2	chr4	129208576	+	TGACCGCGAGCAGGATGCGC
2900	sgPGRMC2_10	PGRMC2	chr4	129208551	-	AAAGTCTTCGACGTGACCAA
2901	sgPHF2_1	PHF2	chr9	96339043	+	GCGGCGCGGCAACATGGCGA
2902	sgPHF2_2	PHF2	chr9	96438952	-	GGTGCCGGCAGAGATGGAGG
2903	sgPHF2_3	PHF2	chr9	96392255	+	TGTTGGGGTGGAAGAGGAGG
2904	sgPHF2_4	PHF2	chr9	96398706	+	GACCTGGCACAAACACGGCC
2905	sgPHF2_5	PHF2	chr9	96398769	-	GCAGCTCCTTGATGAAGAGC
2906	sgPHF2_6	PHF2	chr9	96408039	-	GACGTCACTGACATAGAACG
2907	sgPHF2_7	PHF2	chr9	96411473	-	GGTGTCAGAGAACTCGAGGT
2908	sgPHF2_8	PHF2	chr9	96418240	-	GAAGGCCAGGCAGTCCACAG
2909	sgPHF2_9	PHF2	chr9	96418258	-	GTGGAGGAAATGTCCCGCGA
2910	sgPHF2_10	PHF2	chr9	96425206	+	GAGTGTGCCCAACAAAGATG
2911	sgPHF8_1	PHF8	chrX	54040813	+	GAGTACATGGTACCAGACAG
2912	sgPHF8_2	PHF8	chrX	54037612	-	GATGTTCTTTGGGGACCAGG
2913	sgPHF8_3	PHF8	chrX	54029059	+	AGAAGTTCCCTCCAAAGGCA
2914	sgPHF8_4	PHF8	chrX	54028676	-	GAGCCTATGAGATTGAGAAG
2915	sgPHF8_5	PHF8	chrX	54014289	-	GCTGATGGCAGAACAGGTGA
2916	sgPHF8_6	PHF8	chrX	54012364	-	GGAGGACTATACAACAGATG
2917	sgPHF8_7	PHF8	chrX	54011416	-	CTGGAGAACCGAGAGCGAGG
2918	sgPHF8_8	PHF8	chrX	53970623	+	GGGTCCCCTCACGCACAGGA
2919	sgPHF8_9	PHF8	chrX	54069155	-	GGGCCGAGCGAAGATGGCCT
2920	sgPHF8_10	PHF8	chrX	54069135	+	GAGGCAATACACCGGCACCG
2921	sgPHYH_1	PHYH	chr10	13336430	-	CAGATACTGCACTCTCCCCG
2922	sgPHYH_2	PHYH	chr10	13325764	-	GGTGCACCTGGTGATGGAGA
2923	sgPHYH_3	PHYH	chr10	13325704	-	ATCTGGTCAGAATAAAACCC
2924	sgPHYH_4	PHYH	chr10	13323035	-	CAGTCAAGAAAACATCGAGA
2925	sgPHYH_5	PHYH	chr10	13336484	-	AAGTGAGAAGATGATCACGA
2926	sgPHYH_6	PHYH	chr10	13325785	-	CTACGAGGAAAACAAGGCCC

2927	sgPHYH_7	PHYH	chr10	13330430	-	GAGCACATCAGCCGGAACAA
2928	sgPHYH_8	PHYH	chr10	13330397	-	GTGCTCCCAGGCACACACAA
2929	sgPHYH_9	PHYH	chr10	13330350	+	GCAGACCTACCTCCCACTTG
2930	sgPHYH_10	PHYH	chr10	13323069	+	AATGTAGTGGCAATCGGCAC
2931	sgPHYHD1_1	PHYHD1	chr9	131684604	-	CGAGGGGCTCAGGCAGGCCA
2932	sgPHYHD1_2	PHYHD1	chr9	131702700	+	GCTGGGCGTGTGGATCGCAG
2933	sgPHYHD1_3	PHYHD1	chr9	131702980	-	CCTACCTCTCTGCACTGGGG
2934	sgPHYHD1_4	PHYHD1	chr9	131684614	+	GAGCCCCTCGCAGCTCCAGA
2935	sgPHYHD1_5	PHYHD1	chr9	131689371	+	GTGGCCATGCAACAAAGGAT
2936	sgPHYHD1_6	PHYHD1	chr9	131696067	+	ACAGACTATTTCTTGAGCAG
2937	sgPHYHD1_7	PHYHD1	chr9	131702669	-	GGGCTCCGTGTACAGGAAGG
2938	sgPHYHD1_8	PHYHD1	chr9	131702876	+	AGGTGGTGTGTCAAGAAGGA
2939	sgPHYHD1_9	PHYHD1	chr9	131703731	+	GGTCCTCATCCATGGAGAAG
2940	sgPHYHD1_10	PHYHD1	chr9	131703832	-	CCAGTTCTCCGGGCTCCAGG
2941	sgPIR_1	PIR	chrX	15509287	-	GAGAAGCATTGGCAGACCCG
2942	sgPIR_2	PIR	chrX	15509298	-	GCGAGGGTCCGGAGAAGCAT
2943	sgPIR_3	PIR	chrX	15477793	-	CACACTGGTAAAATGAACCC
2944	sgPIR_4	PIR	chrX	15477845	-	GGTATCCTACCTCCTGGAAG
2945	sgPIR_5	PIR	chrX	15474168	-	GTGTTTGCAGTGGATGACTG
2946	sgPIR_6	PIR	chrX	15509351	+	TGAGAGAGTAACTTTCTTGG
2947	sgPIR_7	PIR	chrX	15509314	-	GTCGGAAGGGGTTGGAGCGA
2948	sgPIR_8	PIR	chrX	15497871	+	GGATGATCAGGAAATCCTCC
2949	sgPIR_9	PIR	chrX	15497850	+	ACTGTTTCAAAACCTCGATG
2950	sgPIR_10	PIR	chrX	15444090	+	TGGTGTGCGAGTGTAAACCT
2951	sgPLOD1_1	PLOD1	chr1	11994872	+	GCTGCTGCTGGCCGAAGCGA
2952	sgPLOD1_2	PLOD1	chr1	12010522	+	GCTGGAGACCAAGTATCCGG
2953	sgPLOD1_3	PLOD1	chr1	11994890	+	GAAGGGCGACGCCAAGCCGG
2954	sgPLOD1_4	PLOD1	chr1	12008034	+	CAACCTTTTAGTCCTCACGG
2955	sgPLOD1_5	PLOD1	chr1	12008052	+	GGTGGCCACTAAGGAGACCG
2956	sgPLOD1_6	PLOD1	chr1	12008082	-	GAGCTGAGCGCTTGAAGCGA
2957	sgPLOD1_7	PLOD1	chr1	12009844	+	GGAGGACTGGAATGTGGAGA
2958	sgPLOD1_8	PLOD1	chr1	12009916	+	GGAGAAGCACGCAGACAAGG
2959	sgPLOD1_9	PLOD1	chr1	12014886	+	GGAGCAGATCAATATCACCC
2960	sgPLOD1_10	PLOD1	chr1	12014913	-	TCTGGAAGATACGGCAGCGG
2961	sgPLOD2_1	PLOD2	chr3	145878707	+	GACAGGGATTCCAGGGGTGG
2962	sgPLOD2_2	PLOD2	chr3	145839007	-	CAAGGAGAAGAATGGAGAGG
2963	sgPLOD2_3	PLOD2	chr3	145806422	+	AACAATATGTCCAGAAACCG
2964	sgPLOD2_4	PLOD2	chr3	145828168	-	CCAAAAGGCAAACCACAAAG
2965	sgPLOD2_5	PLOD2	chr3	145828074	-	AAACGCTATCTGAATTCAGG
2966	sgPLOD2_6	PLOD2	chr3	145799611	-	TAACTCGTCATGGAAAGCTG
2967	sgPLOD2_7	PLOD2	chr3	145828152	-	AAAGTGGTCTTTGCAGCAGA
2968	sgPLOD2_8	PLOD2	chr3	145789194	-	TAGGATAGCCGTATATCTGG
2969	sgPLOD2_9	PLOD2	chr3	145878733	+	AGCGCCAGGAGCAGCAGCTG
2970	sgPLOD2_10	PLOD2	chr3	145878670	-	AAGCCCTCGAGCATCCCCAC
2971	sgPLOD3_1	PLOD3	chr7	100859756	-	GTGGAGGACAGAAGGTCCGG
2972	sgPLOD3_2	PLOD3	chr7	100855908	+	AACACGGCCAGAAACACCCG
2973	sgPLOD3_3	PLOD3	chr7	100860512	+	GGCAGCAGCAGCAGGAACCG
2974	sgPLOD3_4	PLOD3	chr7	100860047	-	GAAGCTGCTGGTGATCACTG
2975	sgPLOD3_5	PLOD3	chr7	100860028	-	GTGGCCACAGCTGAAACCGA
2976	sgPLOD3_6	PLOD3	chr7	100859990	+	TGAAGAACTCCGCAGAGCGC
2977	sgPLOD3_7	PLOD3	chr7	100859775	-	GATGTGGCTCGAACAGTTGG
2978	sgPLOD3_8	PLOD3	chr7	100856144	-	GCTTCTGCAACCAGGACCGG
2979	sgPLOD3_9	PLOD3	chr7	100855858	+	GTAGCAGCCGCTGCAGGAAG
2980	sgPLOD3_10	PLOD3	chr7	100855597	+	GAGAAGTGGTCCTGGAGCTG
2981	sgPOLA1_1	POLA1	chrX	24735558	+	AGAGGAAGTGAAACAAGAGG
2982	sgPOLA1_2	POLA1	chrX	24745991	-	AAAGCCATCACGACAAGCGG
2983	sgPOLA1_3	POLA1	chrX	24750550	-	CAGAAGTGTGACTGAAAGGG
2984	sgPOLA1_4	POLA1	chrX	24763638	+	AGAGGCACAGAAAGTTACAG
2985	sgPOLA1_5	POLA1	chrX	24767088	+	GGTGACATACAAAGGAAGGG
2986	sgPOLA1_6	POLA1	chrX	24906218	+	GAAAGCTACACTTCAACCAG
2987	sgPOLA1_7	POLA1	chrX	24735049	-	AGAGGAACAGGAGTTAATGG

2988	sgPOLA1_8	POLA1	chrX	24735423	+	CGAGAGTACAGAAGAAGAGC
2989	sgPOLA1_9	POLA1	chrX	24735486	+	GCCCATGGAAGTTGAAGAGG
2990	sgPOLA1_10	POLA1	chrX	24757507	+	GGTGAAAGAAATGCTACCTG
2991	sgPOLD1_1	POLD1	chr19	50902104	+	GCAGGATGGATGGCAAGCGG
2992	sgPOLD1_2	POLD1	chr19	50905492	-	GGTGATGCGCAGGAACGGGG
2993	sgPOLD1_3	POLD1	chr19	50919733	-	GGATGGGCTCGAAGATGCGC
2994	sgPOLD1_4	POLD1	chr19	50902174	-	GTGCATCATCATCATCCCAG
2995	sgPOLD1_5	POLD1	chr19	50902213	+	GGAGGACCTGGCACTGATGG
2996	sgPOLD1_6	POLD1	chr19	50902252	+	CAGGCTGCAGGAGCAGGAGG
2997	sgPOLD1_7	POLD1	chr19	50905075	-	GGAGCACAGGCACGGAGCCG
2998	sgPOLD1_8	POLD1	chr19	50905502	-	GCAGCGCCACGGTGATGCGC
2999	sgPOLD1_9	POLD1	chr19	50905911	+	GTGGTCAGTCACCCACCGGA
3000	sgPOLD1_10	POLD1	chr19	50906437	+	CAAGGTGCAGAGCTACGAGA
3001	sgPOLE_1	POLE	chr12	133241964	-	GAAGCGCTGCAAGAACATGG
3002	sgPOLE_2	POLE	chr12	133226002	-	GCAGCGTCTGGAGTCGGCAG
3003	sgPOLE_3	POLE	chr12	133225538	-	CCAGCGAGTCGCTAAAGCGG
3004	sgPOLE_4	POLE	chr12	133214608	+	GATGTACTCCACGTAAGCGA
3005	sgPOLE_5	POLE	chr12	133210946	-	GCAAGACTCCCAGAAAGCAG
3006	sgPOLE_6	POLE	chr12	133208942	+	GCAGCAAGTGGGAACCGGGG
3007	sgPOLE_7	POLE	chr12	133263885	-	TCCATGTCTCTGAGGAGCGG
3008	sgPOLE_8	POLE	chr12	133257234	-	TAAGCGCTTAGGCAGTGCAG
3009	sgPOLE_9	POLE	chr12	133256180	-	GCTGTCCTTCCACACTGTGG
3010	sgPOLE_10	POLE	chr12	133254169	+	GGATCTTCAGGTCAATGGAG
3011	sgPPAT_1	PPAT	chr4	57267553	-	AAGGTCTGAAGGAAACCCAG
3012	sgPPAT_2	PPAT	chr4	57273850	-	GCTGGTATTGTGACTAGTGA
3013	sgPPAT_3	PPAT	chr4	57267018	-	GCTAGCGATTGAAGCACCTG
3014	sgPPAT_4	PPAT	chr4	57269244	-	GGAAATCGTCCCTTATGCAT
3015	sgPPAT_5	PPAT	chr4	57268318	-	ATCAGAAACAGAAGGATGGG
3016	sgPPAT_6	PPAT	chr4	57267498	-	AGACCAGACAGTATGTTCGA
3017	sgPPAT_7	PPAT	chr4	57273868	-	CACAGGGGTCAGGAGAGTGC
3018	sgPPAT_8	PPAT	chr4	57266957	-	CCTGCTGCTCTTGCTTACGC
3019	sgPPAT_9	PPAT	chr4	57301531	-	GTGATCACTCTGGGACTCGT
3020	sgPPAT_10	PPAT	chr4	57301518	-	GACTCGTGGGGCTGCAGCAC
3021	sgPPP1CA_1	PPP1CA	chr11	67168214	+	GGAGCAGGAAGAAGTTCTCG
3022	sgPPP1CA_2	PPP1CA	chr11	67167111	-	GACGCTACAACATCAAACTG
3023	sgPPP1CA_3	PPP1CA	chr11	67167081	+	GCAGTTGAAGCAGTCAGTGA
3024	sgPPP1CA_4	PPP1CA	chr11	67166496	-	GGCTGGGGCGAGAACGACCG
3025	sgPPP1CA_5	PPP1CA	chr11	67166476	+	CAAAGGTAAAAGAGACGCCA
3026	sgPPP1CA_6	PPP1CA	chr11	67166431	+	GGTCCAAGTCGTGCTTGTGG
3027	sgPPP1CA_7	PPP1CA	chr11	67167034	-	AAGATCTTCTGCTGCCACGG
3028	sgPPP1CA_8	PPP1CA	chr11	67166545	+	GCAGGTCACACAGCAGGCCC
3029	sgPPP1CA_9	PPP1CA	chr11	67166511	-	GACAAGGACGTGCAGGGCTG
3030	sgPPP1CA_10	PPP1CA	chr11	67168312	+	AAGAGGTAGTTGCTCTCGGG
3031	sgPPP3CA_1	PPP3CA	chr4	102267905	-	GTTGTCGACGACCGACAGGG
3032	sgPPP3CA_2	PPP3CA	chr4	102030134	+	CAACATAGTCCCCTAAGAAG
3033	sgPPP3CA_3	PPP3CA	chr4	101953462	-	CCAACTGGCATGCTCCCCAG
3034	sgPPP3CA_4	PPP3CA	chr4	102019592	+	TCATCAGGGCAGCCAGGGGA
3035	sgPPP3CA_5	PPP3CA	chr4	101953426	-	GGGAAGCAAACCCTGCAAAG
3036	sgPPP3CA_6	PPP3CA	chr4	102019567	-	CAGTTCCTGTGTGTGCATGG
3037	sgPPP3CA_7	PPP3CA	chr4	102015022	-	GACCTATGTGTGATATCCTG
3038	sgPPP3CA_8	PPP3CA	chr4	101982255	-	GAACTAGGGTCAGAAGAAGA
3039	sgPPP3CA_9	PPP3CA	chr4	102117175	-	AAAGGCGCATCTTATGAAGG
3040	sgPPP3CA_10	PPP3CA	chr4	102030182	-	GATGAAGCTCTTTGAAGTCG
3041	sgPPP3CB_1	PPP3CB	chr10	75204502	-	GTGGAGTGTTAGCTGGAGGA
3042	sgPPP3CB_2	PPP3CB	chr10	75204485	-	GGACGGCAGACCCTGCAAAG
3043	sgPPP3CB_3	PPP3CB	chr10	75239239	+	TCAGATGTCAAGCGATGTGT
3044	sgPPP3CB_4	PPP3CB	chr10	75239190	+	GTTCTTCAGAACATCAACCC
3045	sgPPP3CB_5	PPP3CB	chr10	75239103	+	GGTTTTCTCTCTCCGAAGGA
3046	sgPPP3CB_6	PPP3CB	chr10	75231269	-	CAGTTTCTTTGTGTTCATGG
3047	sgPPP3CB_7	PPP3CB	chr10	75230927	+	CACATTGGTCCAAATGCAGG
3048	sgPPP3CB_8	PPP3CB	chr10	75230912	+	GACCATAACAAGTCACACAT

3049	sgPPP3CB_9	PPP3CB	chr10	75206275	-	AATTCGAGCAATTGGCAAGA
3050	sgPPP3CB_10	PPP3CB	chr10	75255564	+	TTTGACGACGCGGTCAGCCC
3051	sgPPP3CC_1	PPP3CC	chr8	22298912	+	CCGAGGGGACCATGTCCGGG
3052	sgPPP3CC_2	PPP3CC	chr8	22370906	+	ATACCCACAACACTGTCCGA
3053	sgPPP3CC_3	PPP3CC	chr8	22298924	-	GTGGAAGCGCCTCCCGGACA
3054	sgPPP3CC_4	PPP3CC	chr8	22368681	-	CATGTACACAGAGAAACTGC
3055	sgPPP3CC_5	PPP3CC	chr8	22370827	-	CACACAGGTCCAAAGGCGGG
3056	sgPPP3CC_6	PPP3CC	chr8	22370853	+	GCTTTGGTCTGATCCCTCAG
3057	sgPPP3CC_7	PPP3CC	chr8	22380192	-	GTGATGGAAAGCCTGTGGCT
3058	sgPPP3CC_8	PPP3CC	chr8	22389786	-	GACCCGTGCCATCTTCCCAA
3059	sgPPP3CC_9	PPP3CC	chr8	22370842	-	GACCAAAGCAGGTCACACAC
3060	sgPPP3CC_10	PPP3CC	chr8	22298951	-	TTTGATGACGCGGTCGGTGG
3061	sgPTGES2_1	PTGES2	chr9	130885398	-	GCAGGGAGGAGATGAAGTGG
3062	sgPTGES2_2	PTGES2	chr9	130889766	-	GGCTGTACCACACGGCGCGG
3063	sgPTGES2_3	PTGES2	chr9	130887546	-	CAGAAAGGTGCCCATCCTGG
3064	sgPTGES2_4	PTGES2	chr9	130886791	+	GAGGGCGCTGATGATGACAG
3065	sgPTGES2_5	PTGES2	chr9	130886104	+	TAGGTGATGATCTCTTCCAG
3066	sgPTGES2_6	PTGES2	chr9	130885999	-	GAGGCCCAGCAAGTGTATGG
3067	sgPTGES2_7	PTGES2	chr9	130885280	-	GGGCAAGTTCGGAGCCGTGG
3068	sgPTGES2_8	PTGES2	chr9	130887684	+	GGTACAGGGTCAGCTGCAGG
3069	sgPTGES2_9	PTGES2	chr9	130887592	-	TGGAGGTGAACCCTGTGCGC
3070	sgPTGES2_10	PTGES2	chr9	130886066	-	TGTGAACGAGCAGGGCAAGG
3071	sgPTGIS_1	PTGIS	chr20	48130897	+	GAGCAGGAGCCAGAAGGCAG
3072	sgPTGIS_2	PTGIS	chr20	48184569	+	AGGACACTCACCGCGTGCGG
3073	sgPTGIS_3	PTGIS	chr20	48166688	+	GGGATGCTGCCCAGGTCCAG
3074	sgPTGIS_4	PTGIS	chr20	48166668	+	CCAAGGCATACCCCAACCAG
3075	sgPTGIS_5	PTGIS	chr20	48164502	-	GGACCCACACTCCTACGACG
3076	sgPTGIS_6	PTGIS	chr20	48160849	+	GGAAGCTGTAGGAGAAGTCG
3077	sgPTGIS_7	PTGIS	chr20	48140660	-	GAGTTACCTGCTGCACCTGG
3078	sgPTGIS_8	PTGIS	chr20	48129619	-	AGAAATCTACACAGACCCAG
3079	sgPTGIS_9	PTGIS	chr20	48184608	+	GCAGCAACAGTGCGGCCAGG
3080	sgPTGIS_10	PTGIS	chr20	48156146	+	GGTCGAGCTGGCGAAAGGTG
3081	sgPTGS1_1	PTGS1	chr9	125143687	-	TCCTCCTGAGCAGGAAGCGG
3082	sgPTGS1_2	PTGS1	chr9	125143771	-	CAGAAGTTTTGAAGAACTGG
3083	sgPTGS1_3	PTGS1	chr9	125143795	+	GATGGGTCCTGGCTTCACCA
3084	sgPTGS1_4	PTGS1	chr9	125145889	-	GAAGCAGCCCAAACACCTCC
3085	sgPTGS1_5	PTGS1	chr9	125145911	-	GTGGCATACAGCATGAGCCC
3086	sgPTGS1_6	PTGS1	chr9	125141159	-	GGGGCAATCTTTAGGCACAG
3087	sgPTGS1_7	PTGS1	chr9	125143734	-	AAAGAAGGCAAACATGAGGT
3088	sgPTGS1_8	PTGS1	chr9	125145815	-	ACAGGCGCCTCTTCTACCGA
3089	sgPTGS1_9	PTGS1	chr9	125145971	+	AAGGCTGAGCACCCCACCTG
3090	sgPTGS1_10	PTGS1	chr9	125145985	-	GCTGCTCATCGCCCCAGGTG
3091	sgPTGS2_1	PTGS2	chr1	186646814	-	TCAAGACAGATCATAAGCGA
3092	sgPTGS2_2	PTGS2	chr1	186648275	+	GTAGTGCACTGTGTTTGGAG
3093	sgPTGS2_3	PTGS2	chr1	186647438	+	GGGCTCTAGTATAATAGGAG
3094	sgPTGS2_4	PTGS2	chr1	186646887	-	AAGTTCATCCCTGATCCCCA
3095	sgPTGS2_5	PTGS2	chr1	186645764	+	GATGCTCAGGGACTTGAGGA
3096	sgPTGS2_6	PTGS2	chr1	186647413	+	GGGCAATCATCAGGCACAGG
3097	sgPTGS2_7	PTGS2	chr1	186646792	-	GCCAGCTTTCACCAACGGGC
3098	sgPTGS2_8	PTGS2	chr1	186645752	-	TGAGCATCTACGGTTTGCTG
3099	sgPTGS2_9	PTGS2	chr1	186645693	-	TGTATGCCACAATCTGGCTG
3100	sgPTGS2_10	PTGS2	chr1	186645643	-	AAACAGGAGCATCCTGAATG
3101	sgPXDN_1	PXDN	chr2	1680752	+	GAAGTACACGGTGTTCCCCG
3102	sgPXDN_2	PXDN	chr2	1652965	+	GGATCATGACAGAGAAGCAG
3103	sgPXDN_3	PXDN	chr2	1652333	+	GCCAAACCTGAAGGCCGCGG
3104	sgPXDN_4	PXDN	chr2	1748211	-	GCCATGGCCAAGCGCTCCAG
3105	sgPXDN_5	PXDN	chr2	1748176	+	GCACGAGCGCCAACAGGCAG
3106	sgPXDN_6	PXDN	chr2	1748072	+	CAGATGCATGCAGCGCACGG
3107	sgPXDN_7	PXDN	chr2	1684092	-	ACTGCGACTGTGAAATCCTG
3108	sgPXDN_8	PXDN	chr2	1680809	-	GGTTGGGCACAGAAAGGCCC
3109	sgPXDN_9	PXDN	chr2	1670223	-	AATCCAGCCACAGAATACAG

3110	sgPXDN_10	PXDN	chr2	1670196	-	GGTTGGGGAGAGCGTCACGC
3111	sgPXDNL_1	PXDNL	chr8	52284469	-	GAACTGCAGCGAGATCCCGA
3112	sgPXDNL_2	PXDNL	chr8	52721799	+	GGACGGTGCTCTTAAAGCAA
3113	sgPXDNL_3	PXDNL	chr8	52721778	+	CCAGCATCAAGTGCATGCAG
3114	sgPXDNL_4	PXDNL	chr8	52361654	+	GGGGTTACTGTAAATTGTGG
3115	sgPXDNL_5	PXDNL	chr8	52361570	+	GTCCAGACAATAACAGGAGG
3116	sgPXDNL_6	PXDNL	chr8	52321827	+	ACTGTGGCGACCAGCCGGGG
3117	sgPXDNL_7	PXDNL	chr8	52321598	+	GAAGAGCATGCAGGGCGCGT
3118	sgPXDNL_8	PXDNL	chr8	52321379	+	GGGCAATAAGGGCTTTCCGG
3119	sgPXDNL_9	PXDNL	chr8	52321259	+	GAACCACAGGGTGTGCATGG
3120	sgPXDNL_10	PXDNL	chr8	52321243	-	GTTCCGGGAACACAACAGGA
3121	sgREV3L_1	REV3L	chr6	111709087	-	GGATCTGTGGACTACAGCGA
3122	sgREV3L_2	REV3L	chr6	111694329	-	AGAATCGTCGCCACAACCAG
3123	sgREV3L_3	REV3L	chr6	111804024	+	CAGGTATCCAGCCCCTGCAG
3124	sgREV3L_4	REV3L	chr6	111697830	+	GGTAATCTTAGCAGAAGATG
3125	sgREV3L_5	REV3L	chr6	111696163	+	GTGGGAGACCAGCAGCGAGG
3126	sgREV3L_6	REV3L	chr6	111695079	-	AACCTGAAAGAGTAAAACCG
3127	sgREV3L_7	REV3L	chr6	111685076	-	CAGCATACAAAACTTACAGG
3128	sgREV3L_8	REV3L	chr6	111672947	-	GAGATTCAGATGCATTCCTG
3129	sgREV3L_9	REV3L	chr6	111654420	+	GAACAGAGTTGCTATAGAAG
3130	sgREV3L_10	REV3L	chr6	111631190	-	CCAGCTTGTAAGGCGCCCAG
3131	sgRFESD_1	RFESD	chr5	94990024	+	AATTCCTTTTCAGACTCAGG
3132	sgRFESD_2	RFESD	chr5	94989886	+	GTGGTCATTTTCTACCACAA
3133	sgRFESD_3	RFESD	chr5	94991763	+	GACCGTGTATAGTTTGCCCC
3134	sgRFESD_4	RFESD	chr5	94991847	+	ATCCATCAGCAAAACCCAAG
3135	sgRFESD_5	RFESD	chr5	94988838	-	CTAGACATGCCAACAGCTTG
3136	sgRFESD_6	RFESD	chr5	94988826	+	AATTCTCTTCCCCAAGCTGT
3137	sgRFESD_7	RFESD	chr5	94988030	-	TTCTATGGACTCACTTGGAG
3138	sgRFESD_8	RFESD	chr5	94989782	+	CTGCACAAGATCCTGAAAAG
3139	sgRFESD_9	RFESD	chr5	94991858	+	AAACCCAAGTGGTGCTCCAA
3140	sgRFESD_10	RFESD	chr5	94989903	+	CAAGGGAGAATATCATGCTA
3141	sgRORA_1	RORA	chr15	60806848	+	GTAATCGACAGTGTTGGCAG
3142	sgRORA_2	RORA	chr15	60803576	+	CAGGTAGAAGCTGCTGACGG
3143	sgRORA_3	RORA	chr15	60797752	-	AAGAGCTCCAGCAGATAACG
3144	sgRORA_4	RORA	chr15	60806817	-	GCCGTAGGGATGTCTCGAGA
3145	sgRORA_5	RORA	chr15	60803417	+	GCCATTACCTAATTCTGCCA
3146	sgRORA_6	RORA	chr15	60795806	-	AGAAGCTATACAGTATGTGG
3147	sgRORA_7	RORA	chr15	60795784	-	GAGTTTGCCAAACGCATTGA
3148	sgRORA_8	RORA	chr15	60795013	+	TGTTCTGAGAGTCAAAGGCA
3149	sgRORA_9	RORA	chr15	60794963	+	GGATTTGAAGACGTCGGGGC
3150	sgRORA_10	RORA	chr15	60803617	-	CATTGACGGGCACACCCCTG
3151	sgRORB_1	RORB	chr9	77257342	+	GGAGGATGTCCAAGAAGCAA
3152	sgRORB_2	RORB	chr9	77249590	-	ACAGTTTCTCTGCCTTGGGC
3153	sgRORB_3	RORB	chr9	77249636	-	GCAGTCGGCAGTGTTGGCAA
3154	sgRORB_4	RORB	chr9	77257361	+	AAGGGACAGCCTGTATGCTG
3155	sgRORB_5	RORB	chr9	77257415	+	GCGGCAGCAGCAGAGTGGGG
3156	sgRORB_6	RORB	chr9	77275567	+	GGAGACATGTCAATACACCA
3157	sgRORB_7	RORB	chr9	77286796	+	GGATGATGAGACCTTGGCAA
3158	sgRORB_8	RORB	chr9	77257433	+	GGAGGCAGAAGCCCTTGCCA
3159	sgRORB_9	RORB	chr9	77257447	-	GCTGCTGTACACCCTGGCAA
3160	sgRORB_10	RORB	chr9	77257488	+	AACCTGAACAACGAGACCAG
3161	sgRORC_1	RORC	chr1	151787870	-	GCCGCATGTCCAAGAAGCAG
3162	sgRORC_2	RORC	chr1	151785490	+	GGAGTGGGAGAAGTCAAAGA
3163	sgRORC_3	RORC	chr1	151787803	-	ACAGCAGCAACAGGAACCAG
3164	sgRORC_4	RORC	chr1	151787782	-	GGTCAAGACCCCTCCAGCAG
3165	sgRORC_5	RORC	chr1	151787419	-	CAGTTTCCGCAGCACACCGG
3166	sgRORC_6	RORC	chr1	151786054	-	GTGTGCCCACCACCTCACCG
3167	sgRORC_7	RORC	chr1	151789718	+	TGTCCCCACAGATTTTGCAA
3168	sgRORC_8	RORC	chr1	151789158	-	CCTGCAGAAATGCCTGGCGC
3169	sgRORC_9	RORC	chr1	151787749	-	AGATACCCTCACCTACACCT
3170	sgRORC_10	RORC	chr1	151787701	+	GCAGGTCAGGCGAGGAGCCC

3171	sgRPE_1	RPE	chr2	210880779	+	CGAGGCTACTGAGAACCCAG
3172	sgRPE_2	RPE	chr2	210882227	+	TTGGATATAGAGGTCGATGG
3173	sgRPE_3	RPE	chr2	210880689	+	CTTTCTAGACATGCACATGA
3174	sgRPE_4	RPE	chr2	210882262	+	CACTGTCCATAAATGTGCAG
3175	sgRPE_5	RPE	chr2	210880732	+	GTAAAGCCAATGGCTGTAGC
3176	sgRPE_6	RPE	chr2	210867457	-	CCCCAGAGTCTAGCATCCGG
3177	sgRPE_7	RPE	chr2	210874453	-	ACACCCACTTACCAAAGAAA
3178	sgRPE_8	RPE	chr2	210867493	-	GTTACCCGTCCATTACGTCC
3179	sgRPE_9	RPE	chr2	210867481	+	TTATCTGCACCTGGACGTAA
3180	sgRPE_10	RPE	chr2	210880960	+	GCACAGTCAGGGCCCATTGC
3181	sgRPE65_1	RPE65	chr1	68910476	+	GCAGGGATCTGGGAAAGCAC
3182	sgRPE65_2	RPE65	chr1	68914372	-	AGGGTTGAGCATCCTGCTGG
3183	sgRPE65_3	RPE65	chr1	68912490	+	AGAGTCCTGGCCCACATCGA
3184	sgRPE65_4	RPE65	chr1	68912442	+	GGGCTTGCCCATCAAACAGG
3185	sgRPE65_5	RPE65	chr1	68906526	+	GTAAACTCACCTGCTTGCAG
3186	sgRPE65_6	RPE65	chr1	68897203	+	GTCACTGCACAGAATTGCAG
3187	sgRPE65_7	RPE65	chr1	68912511	+	GGAGACTGCCGGTGAGCCAG
3188	sgRPE65_8	RPE65	chr1	68912395	-	GACATGTCACATACCACAGA
3189	sgRPE65_9	RPE65	chr1	68905260	+	GCTTGAATCGGTCACTGCAG
3190	sgRPE65_10	RPE65	chr1	68896750	-	GATGCCTTGGAAGAAGATGA
3191	sgRRM2_1	RRM2	chr2	10263612	+	GCAGATGTATAAGAAGGCAG
3192	sgRRM2_2	RRM2	chr2	10263571	-	GGGAAGATGACAAAGCGGCG
3193	sgRRM2_3	RRM2	chr2	10263938	+	GCTTTCTTTGCAGCAAGCGA
3194	sgRRM2_4	RRM2	chr2	10263138	+	TGGCCAGCAAGACCGCGAGG
3195	sgRRM2_5	RRM2	chr2	10263889	+	CTGGGAATCCCTGAAACCCG
3196	sgRRM2_6	RRM2	chr2	10263157	+	GAGGATCTTCCAGGAGCCCA
3197	sgRRM2_7	RRM2	chr2	10263870	+	TCTCCAAGGACATTCAGCAC
3198	sgRRM2_8	RRM2	chr2	10263633	+	GGCTTCCTTTTGGACCGCCG
3199	sgRRM2_9	RRM2	chr2	10263650	-	TCCTCCGATTACCTCCTCGG
3200	sgRRM2_10	RRM2	chr2	10267262	-	GAATATCGACGCAAAAGAAC
3201	sgRRM2B_1	RRM2B	chr8	103236302	-	AAGCAGATTGGGCCTTGCGA
3202	sgRRM2B_2	RRM2B	chr8	103251057	-	GGCCGGGCTGGATCAGGATG
3203	sgRRM2B_3	RRM2B	chr8	103226284	-	TGATGCTGTCAAAATTGAGC
3204	sgRRM2B_4	RRM2B	chr8	103237213	-	TCAGGAGGTGCAGGTTCCAG
3205	sgRRM2B_5	RRM2B	chr8	103231164	-	TCTCCCAGGGGAAAGAGTGG
3206	sgRRM2B_6	RRM2B	chr8	103251084	-	GATGGGCGACCCGGAAAGGC
3207	sgRRM2B_7	RRM2B	chr8	103237228	-	GGTGGAGCGCTTTAGTCAGG
3208	sgRRM2B_8	RRM2B	chr8	103251074	-	CCGGAAAGGCCGGAAGCGGC
3209	sgRRM2B_9	RRM2B	chr8	103237115	-	ACATCAGAGATCCCAAGAAA
3210	sgRRM2B_10	RRM2B	chr8	103238219	+	GCTTTAAGCTTGTTCCAGTG
3211	sgRSAD1_1	RSAD1	chr17	48557049	-	GCATGGCAGCCTCCTCCAGG
3212	sgRSAD1_2	RSAD1	chr17	48559643	-	ACAGCTGGTAGAGGGAGAGG
3213	sgRSAD1_3	RSAD1	chr17	48559673	+	GGGCACCGCACTCTTCGCCC
3214	sgRSAD1_4	RSAD1	chr17	48559769	-	GGTGGAAGCCAGCCTCCCGA
3215	sgRSAD1_5	RSAD1	chr17	48560768	+	GGAGCCTGACAACTGGATGA
3216	sgRSAD1_6	RSAD1	chr17	48560790	+	GAGGTGATGCTGTTTGGCCA
3217	sgRSAD1_7	RSAD1	chr17	48557397	+	GGCAGAGTTCGGGGCAGCAG
3218	sgRSAD1_8	RSAD1	chr17	48559475	-	AGTGCGTCCGTCCCAACAGC
3219	sgRSAD1_9	RSAD1	chr17	48559572	+	GGGCTGCCGGCACAGCAGGT
3220	sgRSAD1_10	RSAD1	chr17	48559631	-	GGGAGAGGTGGTCATCACAG
3221	sgRSAD2_1	RSAD2	chr2	7018022	-	GCCCTCAGCCAGCAGAACAG
3222	sgRSAD2_2	RSAD2	chr2	7018063	+	CAAGAGGAGAAAGCAGCAGC
3223	sgRSAD2_3	RSAD2	chr2	7018096	+	GCCAGATGAGACCAAAGAGG
3224	sgRSAD2_4	RSAD2	chr2	7018130	-	GTTGGGGTGGTGGGCAGAGG
3225	sgRSAD2_5	RSAD2	chr2	7023628	+	GAAGCCTGATCCGGGAGAGG
3226	sgRSAD2_6	RSAD2	chr2	7018143	-	ATAGTTGACGCTGGTTGGGG
3227	sgRSAD2_7	RSAD2	chr2	7023507	+	GAGAAGATCAACTTTTCAGG
3228	sgRSAD2_8	RSAD2	chr2	7027159	+	TGGAAAACCTTCAAAAGCTG
3229	sgRSAD2_9	RSAD2	chr2	7023606	+	AGCGTGAGCATCGTGAGCAA
3230	sgRSAD2_10	RSAD2	chr2	7027091	+	CTCCTGTGACAGCTTTGACG
3231	sgRTEL1_1	RTEL1	chr20	62293929	+	GCAGCTGTGCATCCATCCTG

3232	sgRTEL1_2	RTEL1	chr20	62305370	+	GGTGCTGGAGGAGCAGACCA
3233	sgRTEL1_3	RTEL1	chr20	62309659	+	GAGCTGCCTGGAGACGACAG
3234	sgRTEL1_4	RTEL1	chr20	62309678	+	GCGGTGTCACCAAGCCAGGG
3235	sgRTEL1_5	RTEL1	chr20	62317154	+	ATCCTGATGCTGGTCACCGG
3236	sgRTEL1_6	RTEL1	chr20	62317204	+	ACCACTGCAGCCAGAAAGCG
3237	sgRTEL1_7	RTEL1	chr20	62319028	-	GGACCAGCTCGTGCATGCTG
3238	sgRTEL1_8	RTEL1	chr20	62319093	-	CAGAGCAAAGGAGGACACCG
3239	sgRTEL1_9	RTEL1	chr20	62319667	-	GGAGCCCATAGGGCACCACG
3240	sgRTEL1_10	RTEL1	chr20	62320893	-	GGCCCGTGACAATCACACCA
3241	sgSC5D_1	SC5D	chr11	121175095	+	TTACTGTCCAGGCATTGCCA
3242	sgSC5D_2	SC5D	chr11	121174130	-	CATGTGGCTGGATACACGTA
3243	sgSC5D_3	SC5D	chr11	121174142	-	TCATCTTCTGGCCATGTGGC
3244	sgSC5D_4	SC5D	chr11	121177148	+	TTCATCTACTGGATTCACAG
3245	sgSC5D_5	SC5D	chr11	121175139	+	CTGTTCTTGCTGGAGATAAG
3246	sgSC5D_6	SC5D	chr11	121177165	+	CAGAGGCCTTCATCATAGAC
3247	sgSC5D_7	SC5D	chr11	121177781	-	GGAATCTTCCAAATATGGTG
3248	sgSC5D_8	SC5D	chr11	121177802	-	GCATGACTTGCAAATGGAGT
3249	sgSC5D_9	SC5D	chr11	121178110	+	GGGACCGCTCAGTTATGTGA
3250	sgSC5D_10	SC5D	chr11	121178123	+	TATGTGAAGGAGATGACAGA
3251	sgSCD5_1	SCD5	chr4	83601966	+	GGGAGAAGAAGAAGCCCCGG
3252	sgSCD5_2	SCD5	chr4	83719605	-	GGGCTCGAAAGCTCTGAGGG
3253	sgSCD5_3	SCD5	chr4	83626458	+	ACAGCCAGAAATATCCTCAG
3254	sgSCD5_4	SCD5	chr4	83626498	+	ACCTGTGGCTCCACAAGCGA
3255	sgSCD5_5	SCD5	chr4	83602017	+	AGTACTTGTGGTGGGCTCGG
3256	sgSCD5_6	SCD5	chr4	83719652	+	GAAAGGGATCTTCCCCGCGT
3257	sgSCD5_7	SCD5	chr4	83719626	-	GCCAAGGAAGAAATCCGTGC
3258	sgSCD5_8	SCD5	chr4	83719481	+	GGCTTTGGGGATGAGCACCA
3259	sgSCD5_9	SCD5	chr4	83626544	+	GGCCAGGAGGAAGCAGAAGT
3260	sgSCD5_10	SCD5	chr4	83626527	-	GCCGCTCTGGGTGTGACAGC
3261	sgSDHB_1	SDHB	chr1	17380475	+	GCAACCGGCGCCTCAAGGAG
3262	sgSDHB_2	SDHB	chr1	17380508	-	GTCAGGAGCCAAGATGGCGG
3263	sgSDHB_3	SDHB	chr1	17354290	-	AAGAAGGATGAATCTCAGGA
3264	sgSDHB_4	SDHB	chr1	17350510	-	CCAGCTGCCCCAGCTACTGG
3265	sgSDHB_5	SDHB	chr1	17380442	+	CCTGCAGGCAGGCTCCGCCA
3266	sgSDHB_6	SDHB	chr1	17355198	-	TGTGCAATGAACATCAATGG
3267	sgSDHB_7	SDHB	chr1	17355173	-	ACACTCTAGCTTGCACCCGA
3268	sgSDHB_8	SDHB	chr1	17349186	-	CTCCAGAGATGACTTCACAG
3269	sgSDHB_9	SDHB	chr1	17349165	-	GGAGCGCCTGGCCAAGCTGC
3270	sgSDHB_10	SDHB	chr1	17380459	-	TTGCCGGCCACAACCCTTGG
3271	sgSDHC_1	SDHC	chr1	161284194	+	AGATGGCTGCGCTGTTGCTG
3272	sgSDHC_2	SDHC	chr1	161332116	+	CAGATGTGGGACCTAGGAAA
3273	sgSDHC_3	SDHC	chr1	161293444	-	TACTTTCTGATACAGAGCTG
3274	sgSDHC_4	SDHC	chr1	161298201	+	AACCACGGCCAAAGAAGAGA
3275	sgSDHC_5	SDHC	chr1	161298262	-	ATAGTAATGTGGGGAGACAG
3276	sgSDHC_6	SDHC	chr1	161310378	+	TTACAGTTGGTCTCTTCCCA
3277	sgSDHC_7	SDHC	chr1	161326567	-	CAAGTGCAAACTTAGCTGTG
3278	sgSDHC_8	SDHC	chr1	161326484	+	ATGTCGGCCCTGTTACTCCC
3279	sgSDHC_9	SDHC	chr1	161332190	+	TGTGTTGTCCTCTATGGGGC
3280	sgSDHC_10	SDHC	chr1	161332201	-	CATGGCTGCCAGCCCCATAG
3281	sgSDHD_1	SDHD	chr11	111957662	+	TGCGGTGCCCTAGGAGGCCG
3282	sgSDHD_2	SDHD	chr11	111958582	+	TCTGTTGCTTCGAACTCCAG
3283	sgSDHD_3	SDHD	chr11	111959718	-	GCCAGTGACCATGAAGAGTG
3284	sgSDHD_4	SDHD	chr11	111965573	+	TGCAGAAAGCTGCCAAGGCA
3285	sgSDHD_5	SDHD	chr11	111959683	-	GAATAGTCCATCGCAGAGCA
3286	sgSDHD_6	SDHD	chr11	111959705	-	AAGAGTGAGGGCTGCAGCCA
3287	sgSDHD_7	SDHD	chr11	111957627	+	ACGAGATGGCGGTTCTCTGG
3288	sgSDHD_8	SDHD	chr11	111958601	-	ATATGAGCAGGTCTGACCAC
3289	sgSDHD_9	SDHD	chr11	111965588	-	AAGTGCCAAAAGCCCTGCCT
3290	sgSDHD_10	SDHD	chr11	111958613	-	AGAAATGCTGAGATATGAGC
3291	sgSLC11A1_1	SLC11A1	chr2	219249895	-	GCAGAGCAAGCCCAACACGG
3292	sgSLC11A1_2	SLC11A1	chr2	219257774	-	GAAGACAGCCACGAGCACGG

3293	sgSLC11A1_3	SLC11A1	chr2	219248997	-	GAGGAAGCCAGGCCCCGTGA
3294	sgSLC11A1_4	SLC11A1	chr2	219249944	+	GACAGGCAAGGACTTGGGCG
3295	sgSLC11A1_5	SLC11A1	chr2	219254663	-	GGAGACGGACAGGGCGATGG
3296	sgSLC11A1_6	SLC11A1	chr2	219255988	+	TGGCCGTGGACATTTACCAG
3297	sgSLC11A1_7	SLC11A1	chr2	219257702	+	AGGGCTTCCTGAGGCTGCGG
3298	sgSLC11A1_8	SLC11A1	chr2	219251437	-	TGAGAGCAGATTGAATGCAA
3299	sgSLC11A1_9	SLC11A1	chr2	219251915	-	GAAGAAGAAGGTGTCCACGA
3300	sgSLC11A1_10	SLC11A1	chr2	219252635	-	GATGTTGTGGGGCATGATGA
3301	sgSLC11A2_1	SLC11A2	chr12	51398646	+	GAGCAGCAGCCCCACAAGGG
3302	sgSLC11A2_2	SLC11A2	chr12	51402336	+	GAGAGGGATTACTATAGGCA
3303	sgSLC11A2_3	SLC11A2	chr12	51402264	-	GAAGATCTCCATTCCTGAGG
3304	sgSLC11A2_4	SLC11A2	chr12	51385429	+	GAGGATGGGTATGAGAGCAA
3305	sgSLC11A2_5	SLC11A2	chr12	51384625	-	GCATGTGGCATTATATGTGG
3306	sgSLC11A2_6	SLC11A2	chr12	51398624	+	CCAGTCTAGCTGCAAGCCGC
3307	sgSLC11A2_7	SLC11A2	chr12	51390682	+	GTGCGACAGCCTGAACAGGA
3308	sgSLC11A2_8	SLC11A2	chr12	51389417	-	TTTTGGGAAAACCAACGAGC
3309	sgSLC11A2_9	SLC11A2	chr12	51386069	+	AGAGCGAGTCAGAACCACTC
3310	sgSLC11A2_10	SLC11A2	chr12	51402320	+	CCAGGGGACTGTGAAAGAGA
3311	sgSLC25A28_1	SLC25A28	chr10	101380078	-	GGTGGATGGAGTTGGAGGGG
3312	sgSLC25A28_2	SLC25A28	chr10	101373600	-	CTGGAGGATTATAAGAACGG
3313	sgSLC25A28_3	SLC25A28	chr10	101373520	+	GCAGGCAAAATAAAGGGCGT
3314	sgSLC25A28_4	SLC25A28	chr10	101372302	-	GCAGCCATGAACCCTGCGGA
3315	sgSLC25A28_5	SLC25A28	chr10	101379852	-	GCACATGGTGGCAGGCGCCG
3316	sgSLC25A28_6	SLC25A28	chr10	101373551	-	AACGTCACAGCAACAGGCGC
3317	sgSLC25A28_7	SLC25A28	chr10	101373477	-	GAGTGATGTAATCCACCCTG
3318	sgSLC25A28_8	SLC25A28	chr10	101372288	+	AGGAATCATTACCTTCCGCA
3319	sgSLC25A28_9	SLC25A28	chr10	101380067	-	TTGGAGGGGCGGGGTGCTGG
3320	sgSLC25A28_10	SLC25A28	chr10	101373589	-	TAAGAACGGAGGGCCTATGG
3321	sgSLC25A37_1	SLC25A37	chr8	23386570	+	GGGGACAGCCGAGATGGCGG
3322	sgSLC25A37_2	SLC25A37	chr8	23386692	+	GCACTCGGTCATGTACCCGG
3323	sgSLC25A37_3	SLC25A37	chr8	23386510	+	TGGCGGATGGAGCTGCGCAG
3324	sgSLC25A37_4	SLC25A37	chr8	23423828	+	GGAAACAGCCACCTAGCCAA
3325	sgSLC25A37_5	SLC25A37	chr8	23386551	+	GGTGGCGCGGAGGATGGATG
3326	sgSLC25A37_6	SLC25A37	chr8	23386656	+	CCACATGACAGCAGGAGCGA
3327	sgSLC25A37_7	SLC25A37	chr8	23423684	+	AAGAAAATCATGCGGACCGA
3328	sgSLC25A37_8	SLC25A37	chr8	23423759	-	GCAAAATACATGGCATGGGC
3329	sgSLC25A37_9	SLC25A37	chr8	23425870	+	GCGGTAATGAATCCAGCAGA
3330	sgSLC25A37_10	SLC25A37	chr8	23386646	-	TGTCATGTGGGTGGACACGG
3331	sgSLC40A1_1	SLC40A1	chr2	190445177	-	GCCTAGTGTCATGACCAGGG
3332	sgSLC40A1_2	SLC40A1	chr2	190444563	+	GTAGAGAAGGAATTTTGCAG
3333	sgSLC40A1_3	SLC40A1	chr2	190430210	-	GCTGTGGCTTTATTTCGGGA
3334	sgSLC40A1_4	SLC40A1	chr2	190428501	+	GGAGAAACGGACAAGTCCAG
3335	sgSLC40A1_5	SLC40A1	chr2	190445155	-	GGAGATCACAACCGCCAGAG
3336	sgSLC40A1_6	SLC40A1	chr2	190444550	+	GAGAATGACCAAGGTAGAGA
3337	sgSLC40A1_7	SLC40A1	chr2	190430116	-	GAAAGCTGGTCTTAAAGAAG
3338	sgSLC40A1_8	SLC40A1	chr2	190428934	-	AGATACTGAGCCAAAACCCC
3339	sgSLC40A1_9	SLC40A1	chr2	190428836	+	GAAGGGCTCAGCCATCTGGG
3340	sgSLC40A1_10	SLC40A1	chr2	190428727	+	TGGTGATGCAGTCAAAGCCC
3341	sgSLC48A1_1	SLC48A1	chr12	48172931	-	GAAGGTGCAGAAGGCAGCGA
3342	sgSLC48A1_2	SLC48A1	chr12	48167176	+	CGGTGGTCTACCGACAGCCG
3343	sgSLC48A1_3	SLC48A1	chr12	48172949	-	GGTGATGGCCAGCACGAGGA
3344	sgSLC48A1_4	SLC48A1	chr12	48167095	-	GCCGAGCTGCAGCCTGGACG
3345	sgSLC48A1_5	SLC48A1	chr12	48167125	-	GGAGCTGATGCCGGAGTAGG
3346	sgSLC48A1_6	SLC48A1	chr12	48167203	-	TGCGAGCCCTCCCATGGCCG
3347	sgSLC48A1_7	SLC48A1	chr12	48167159	+	CATCTTCCTCGTCTGGACGG
3348	sgSLC48A1_8	SLC48A1	chr12	48172812	+	GGTGCTGGCACTGTGGGTCC
3349	sgSLC48A1_9	SLC48A1	chr12	48172884	+	GCTGCGCGGCTTCTTCTTCG
3350	sgSLC48A1_10	SLC48A1	chr12	48172914	-	CGATGGAGACGGCCGAGAAG
3351	sgSRC_1	SRC	chr20	36024627	+	AAGATCCGCAAGCTGGACAG
3352	sgSRC_2	SRC	chr20	36024680	+	CAACAGCCTGCAGCAGCTGG
3353	sgSRC_3	SRC	chr20	36028676	+	GTCACGGAGTACATGAGCAA

3354	sgSRC_4	SRC	chr20	36030029	+	GGGAGACAGGCAAGTACCTG
3355	sgSRC_5	SRC	chr20	36030058	-	GCAGCCATGTCCACCAGCTG
3356	sgSRC_6	SRC	chr20	36030882	-	TGGCTGCACGAAGGTCCCGG
3357	sgSRC_7	SRC	chr20	36030918	+	GAACCTGGTGTGCAAAGTGG
3358	sgSRC_8	SRC	chr20	36031248	+	CTGAGCTCACCACAAAGGGA
3359	sgSRC_9	SRC	chr20	36012617	+	GCCGAGAACGTGCACGGCGC
3360	sgSRC_10	SRC	chr20	36012731	+	GCCGAGCCCAAGCTGTTCGG
3361	sgSTC2_1	STC2	chr5	172755102	-	GACGCCACCAACCCACCCGA
3362	sgSTC2_2	STC2	chr5	172755143	+	GGTGGCCAACACCAAAGCCA
3363	sgSTC2_3	STC2	chr5	172750374	+	GAACCTGTGCCGCAGAGCGT
3364	sgSTC2_4	STC2	chr5	172750320	-	AAATGGTGTCCCAGTTGCAG
3365	sgSTC2_5	STC2	chr5	172752890	-	ATGACTTTTCTGCACAACGC
3366	sgSTC2_6	STC2	chr5	172750268	-	GGCTGCCCAGGAGAACACCC
3367	sgSTC2_7	STC2	chr5	172750226	+	GCAGCAGCAAGTCCTTGAAA
3368	sgSTC2_8	STC2	chr5	172755130	-	GGCCACCTTTGACCCGGCGC
3369	sgSTC2_9	STC2	chr5	172755087	+	CTGTCTTGGGGACCCTCGGG
3370	sgSTC2_10	STC2	chr5	172750247	+	GGATCATCTCCACTATCACC
3371	sgSTEAP1_1	STEAP1	chr7	89790567	+	ATAGTCTGTCTTACCCAATG
3372	sgSTEAP1_2	STEAP1	chr7	89790109	+	TCCTTTGTAGCATAAGGACA
3373	sgSTEAP1_3	STEAP1	chr7	89791290	+	GTCTCTGGGAATTGTGGGAT
3374	sgSTEAP1_4	STEAP1	chr7	89793813	-	GTGTATTGTGCCCAGTAGAA
3375	sgSTEAP1_5	STEAP1	chr7	89793962	-	AGTATCTTCTTCCTCAAGCA
3376	sgSTEAP1_6	STEAP1	chr7	89790158	-	TGGTGCAAATGCAAAAGCAC
3377	sgSTEAP1_7	STEAP1	chr7	89790208	-	GTGTGTGCTGAAGTTCTGAA
3378	sgSTEAP1_8	STEAP1	chr7	89791308	+	ATTGGCAATACTGGCTCTGT
3379	sgSTEAP1_9	STEAP1	chr7	89790186	-	GCAGTCAAATTCATCAGCAT
3380	sgSTEAP1_10	STEAP1	chr7	89790373	+	CATCAACAAAGTCTTGCCAA
3381	sgSTEAP2_1	STEAP2	chr7	89859269	-	CCAGGAGGGAAAGTAAGCCA
3382	sgSTEAP2_2	STEAP2	chr7	89854680	+	GAGAACATTATACCTCCCTG
3383	sgSTEAP2_3	STEAP2	chr7	89859284	-	GGATAGAAGTGACTGCCAGG
3384	sgSTEAP2_4	STEAP2	chr7	89854695	-	ATGTCTCAGGTCCCACAGGG
3385	sgSTEAP2_5	STEAP2	chr7	89856751	+	GCCTCTGCTTACCGATGAGA
3386	sgSTEAP2_6	STEAP2	chr7	89854465	+	TGGTATCAAAGATGCAAGGA
3387	sgSTEAP2_7	STEAP2	chr7	89856341	-	TGGGAATGAAATTCAACTGG
3388	sgSTEAP2_8	STEAP2	chr7	89856669	-	AACCAGGTTTCCAACCAAGG
3389	sgSTEAP2_9	STEAP2	chr7	89854490	+	ACTGTAGGTGTGATTGGAAG
3390	sgSTEAP2_10	STEAP2	chr7	89854543	+	TATTAGATGCGGCTATCATG
3391	sgSTEAP3_1	STEAP3	chr2	120003306	+	GGAGGCAGTGAGCTCCCCGG
3392	sgSTEAP3_2	STEAP3	chr2	120003387	+	GCTGGCGGGCAAGATCCTGG
3393	sgSTEAP3_3	STEAP3	chr2	120003532	+	CTGCAGGCTGGCCCAAGGGA
3394	sgSTEAP3_4	STEAP3	chr2	120005421	-	GAAGCAGACGAAGAGCCCCA
3395	sgSTEAP3_5	STEAP3	chr2	120005703	-	GCTGTAGAGGGCGTGCAGGG
3396	sgSTEAP3_6	STEAP3	chr2	120005716	-	GCGGCAAGCAGAAGCTGTAG
3397	sgSTEAP3_7	STEAP3	chr2	120005749	-	GGTTGACCAGGTCGTAGCGG
3398	sgSTEAP3_8	STEAP3	chr2	120012342	-	CAGCAGGGACAACGTGCCGA
3399	sgSTEAP3_9	STEAP3	chr2	120003096	+	ACTGATCAGCCTCCACCTGG
3400	sgSTEAP3_10	STEAP3	chr2	120003108	-	TATCGCTGTCCACCAGGTGG
3401	sgSTEAP4_1	STEAP4	chr7	87913406	-	AAGACCACCCTACTGCCCAG
3402	sgSTEAP4_2	STEAP4	chr7	87913463	-	GGATTGAAAATGCTCCAGTG
3403	sgSTEAP4_3	STEAP4	chr7	87910232	-	GAGAGAGTTCCGATTTGTCC
3404	sgSTEAP4_4	STEAP4	chr7	87913167	-	ATTTAACACCATCTCAGCCT
3405	sgSTEAP4_5	STEAP4	chr7	87910338	+	TGAATCACTGAGCCAGGCTG
3406	sgSTEAP4_6	STEAP4	chr7	87913156	+	CTGGAGAGCCCAGGCTGAGA
3407	sgSTEAP4_7	STEAP4	chr7	87913146	-	GGCTCTCCAGTCAGGAGCAC
3408	sgSTEAP4_8	STEAP4	chr7	87910255	+	TTGACTGCATTGCTAACAGA
3409	sgSTEAP4_9	STEAP4	chr7	87913135	-	CAGGAGCACTGGATGCAAGT
3410	sgSTEAP4_10	STEAP4	chr7	87913387	+	GCTCAAGACTTCTGCACCAC
3411	sgSUOX_1	SUOX	chr12	56396410	-	GCTGGAGTTATCACCAGAGA
3412	sgSUOX_2	SUOX	chr12	56396008	+	GCTGCTGCTGCACAGAGCTG
3413	sgSUOX_3	SUOX	chr12	56396391	-	AAGGTGAGGCTGGGGCGCTG
3414	sgSUOX_4	SUOX	chr12	56396326	+	GACTCAAGTCAATCCCCTCA

3415	sgSUOX_5	SUOX	chr12	56396344	-	GGCCTGAATGCAGATCCTTG
3416	sgSUOX_6	SUOX	chr12	56396371	-	GGGCTGAAATGAATCATTTG
3417	sgSUOX_7	SUOX	chr12	56396495	-	CCCTACTTACCCTACACCGA
3418	sgSUOX_8	SUOX	chr12	56396459	+	ATTAGGTCTCGGTGCAGTGT
3419	sgSUOX_9	SUOX	chr12	56396034	+	TCAGGCTCCAACAGGCCTGC
3420	sgSUOX_10	SUOX	chr12	56396448	+	ATGGGGACCCTATTAGGTCT
3421	sgTBXAS1_1	TBXAS1	chr7	139655311	-	GGCCTGCCAGGAGTCCACCG
3422	sgTBXAS1_2	TBXAS1	chr7	139706914	-	GATAGGGCAGGCCTTCCTCG
3423	sgTBXAS1_3	TBXAS1	chr7	139636072	-	AGGACTGAAAGCAGACATCA
3424	sgTBXAS1_4	TBXAS1	chr7	139653214	+	AAAACGCTATGCGGAATCTG
3425	sgTBXAS1_5	TBXAS1	chr7	139653234	+	GGGACGCATTTGACATCCAG
3426	sgTBXAS1_6	TBXAS1	chr7	139655293	-	CGGGGTGCCAAAGGCGACGC
3427	sgTBXAS1_7	TBXAS1	chr7	139661815	-	GTTCGGCTTGCACCCAGTAG
3428	sgTBXAS1_8	TBXAS1	chr7	139661941	-	GGTGGCAAAAGAAAGTGTGT
3429	sgTBXAS1_9	TBXAS1	chr7	139706940	+	TGGTGATTGCAGAGACGCTG
3430	sgTBXAS1_10	TBXAS1	chr7	139715581	+	GCTGTGCTAGAGATGGCCGT
3431	sgTDO2_1	TDO2	chr4	156825225	+	GGTGTGAATAGAGCCAGCAA
3432	sgTDO2_2	TDO2	chr4	156828902	-	GAACAGAATCCAACTCCCAG
3433	sgTDO2_3	TDO2	chr4	156831342	+	GACACTTCTGGAATTAGTGG
3434	sgTDO2_4	TDO2	chr4	156831315	+	ACTGCTACTTAAATCTGAGC
3435	sgTDO2_5	TDO2	chr4	156832649	+	GCATGGCTGGAAAGAACTCC
3436	sgTDO2_6	TDO2	chr4	156832717	+	TATCACCAGAGGCCTGGAAG
3437	sgTDO2_7	TDO2	chr4	156839333	+	GGCAGCAAAGCTGGCACCGG
3438	sgTDO2_8	TDO2	chr4	156839352	-	GTGATAGCCTGAGGAACCAC
3439	sgTDO2_9	TDO2	chr4	156824897	+	AGTGCCTTTTCACCATGAGT
3440	sgTDO2_10	TDO2	chr4	156831207	-	ATAGTCGGAATTGCAAACTC
3441	sgTET1_1	TET1	chr10	70332786	+	GAGACATTGAATGATACCAG
3442	sgTET1_2	TET1	chr10	70406221	+	GAGATATCCTGAATCAGCAG
3443	sgTET1_3	TET1	chr10	70332213	+	TCAGTCAAGACTTTAAGCCC
3444	sgTET1_4	TET1	chr10	70332747	-	CCACAGCGTGTCCCTTCCAG
3445	sgTET1_5	TET1	chr10	70333180	-	GGCCTGTCCTAGGAAAGAGG
3446	sgTET1_6	TET1	chr10	70333704	+	GGTCCTAGCAAATCAGACAG
3447	sgTET1_7	TET1	chr10	70360745	+	AGGAAAACAAGAGGCCCCAG
3448	sgTET1_8	TET1	chr10	70404586	+	CAGCATGACAGGCATCGAGG
3449	sgTET1_9	TET1	chr10	70446142	-	GAGGAATAACACCCAAAGAG
3450	sgTET1_10	TET1	chr10	70446261	-	GCGGGGTGCCAGGACCTCGA
3451	sgTET2_1	TET2	chr4	106156327	-	GGAACCTGTGGAAGAGGAGG
3452	sgTET2_2	TET2	chr4	106156339	-	GAAGGAAGCTGAGGAACCTG
3453	sgTET2_3	TET2	chr4	106156897	+	ATGACCTCCAAACAATACAC
3454	sgTET2_4	TET2	chr4	106157189	-	CAAGTGCTGTTTCAACACTG
3455	sgTET2_5	TET2	chr4	106157296	-	GGGAGATGTGAACTCTGGGA
3456	sgTET2_6	TET2	chr4	106155148	-	GGAGGTGATGGTATCAGGAA
3457	sgTET2_7	TET2	chr4	106155166	-	GGTTCTGTCTGGCAAATGGG
3458	sgTET2_8	TET2	chr4	106155217	-	GGATGAGCTCTCTCAGGCAG
3459	sgTET2_9	TET2	chr4	106155403	-	TGAAGGAGCCCAGAGAGAGA
3460	sgTET2_10	TET2	chr4	106155478	+	GTAAGCCAAGAAAGAAATCC
3461	sgTET3_1	TET3	chr2	74273735	+	GAGGCTGGGAACAACAGCAG
3462	sgTET3_2	TET3	chr2	74274247	-	CAGGCCATGAGGGAGCCCGG
3463	sgTET3_3	TET3	chr2	74326516	+	GGAGAACCAGAATGCAAAGG
3464	sgTET3_4	TET3	chr2	74274011	-	GGCACCTCTGAGCTGAGGAG
3465	sgTET3_5	TET3	chr2	74274114	-	GAGGGCCTCAATGGCAATGG
3466	sgTET3_6	TET3	chr2	74274153	-	GGAATGAGAAGGCTGCGGGA
3467	sgTET3_7	TET3	chr2	74274196	-	GAGGTGACAAGGCCTCAGGA
3468	sgTET3_8	TET3	chr2	74274215	-	GGAGATCTGAAAGGGGCAGG
3469	sgTET3_9	TET3	chr2	74274257	-	GGAGGAACCACAGGCCATGA
3470	sgTET3_10	TET3	chr2	74274371	-	GAGCTCCGGGGCGTTGCTGG
3471	sgTF_1	TF	chr3	133475832	+	GGAGCTTCTCAACCAGGCCC
3472	sgTF_2	TF	chr3	133465278	+	CCGGAAGATGAGGCTCGCCG
3473	sgTF_3	TF	chr3	133467363	-	GCAACACTGGGACCATCGGA
3474	sgTF_4	TF	chr3	133474287	-	AGGGTGGAGCAGCCACACCC
3475	sgTF_5	TF	chr3	133476763	+	GCCATCCGGAATCTACGGGA

3476	sgTF_6	TF	chr3	133485120	+	AGGGTATTTTGCTATAGCAG
3477	sgTF_7	TF	chr3	133485202	+	ACGGCAGTTGGCAGAACCGC
3478	sgTF_8	TF	chr3	133486900	-	GGAGTCTTTCTTAGACCCAG
3479	sgTF_9	TF	chr3	133472501	+	CAATAACCTGAAGCCTGTGG
3480	sgTF_10	TF	chr3	133474266	-	GGACACAGTTGACACAGCTG
3481	sgTH_1	TH	chr11	2188175	+	GAAGCGCTCCAGCAAAGCAA
3482	sgTH_2	TH	chr11	2186978	-	GCAGCTGTACTGGTTCACGG
3483	sgTH_3	TH	chr11	2186948	-	GCTGTGTAAGCAGAACGGGG
3484	sgTH_4	TH	chr11	2192938	-	GCTGGACGCCAAGCAGGCAG
3485	sgTH_5	TH	chr11	2191078	+	CCTGCGCCCAATGAACCGCG
3486	sgTH_6	TH	chr11	2190893	+	ACAGCTCGGGACAGCGCCGA
3487	sgTH_7	TH	chr11	2189808	+	GGCGCACGAAGTACTCCAGG
3488	sgTH_8	TH	chr11	2189761	+	GCGCACACCACTGAGCAGGG
3489	sgTH_9	TH	chr11	2188240	-	GGAGGTCTACACCACGCTGA
3490	sgTH_10	TH	chr11	2187934	+	GCTGGCCAGGAAGTCCCGGG
3491	sgTHAP4_1	THAP4	chr2	242545775	-	GCAGCCCTTCCAGTACCTGG
3492	sgTHAP4_2	THAP4	chr2	242545837	+	GTGCCCAGCATCCAGGACAG
3493	sgTHAP4_3	THAP4	chr2	242545785	+	GAAGGGCTGCAGTGTGGGGT
3494	sgTHAP4_4	THAP4	chr2	242542412	-	CAAGCCCGACACCAACAAGG
3495	sgTHAP4_5	THAP4	chr2	242541396	-	GGCGTGGTGGAAGTGGAGGA
3496	sgTHAP4_6	THAP4	chr2	242541332	+	GGCGAAGGAGATCCTGGCGA
3497	sgTHAP4_7	THAP4	chr2	242541317	+	TACGTGGGGCTCCTTGGCGA
3498	sgTHAP4_8	THAP4	chr2	242545865	-	CCCCAAGATGAACCCAGTGG
3499	sgTHAP4_9	THAP4	chr2	242545801	+	GGGTAGGTCCCGGCTCCAGG
3500	sgTHAP4_10	THAP4	chr2	242545754	-	GGAGGTTCACATCTCCCACG
3501	sgTMEM62_1	TMEM62	chr15	43441305	+	GGGCACTTTGGAACTTGAGG
3502	sgTMEM62_2	TMEM62	chr15	43452955	-	ACGGAGAATAAATGATGCCA
3503	sgTMEM62_3	TMEM62	chr15	43426047	-	GAGCATGGCCACCAGCGCTG
3504	sgTMEM62_4	TMEM62	chr15	43438747	-	GATGAACGAATAGTTGCCAA
3505	sgTMEM62_5	TMEM62	chr15	43438770	+	GTAGATGCCACTGTAAATCC
3506	sgTMEM62_6	TMEM62	chr15	43440964	+	GGAGCTCTTATTACTGGCCA
3507	sgTMEM62_7	TMEM62	chr15	43441052	-	ATTGACCGGATTCCTGGTGA
3508	sgTMEM62_8	TMEM62	chr15	43441328	+	GAGACTGGAAGGATAATAGG
3509	sgTMEM62_9	TMEM62	chr15	43444055	-	GGATTGGTGATAAGAACCAC
3510	sgTMEM62_10	TMEM62	chr15	43446893	-	AACTGTGACAGAAGTAATGG
3511	sgTMLHE_1	TMLHE	chrX	154743761	-	AGCTTCTTAGAAACCAACGA
3512	sgTMLHE_2	TMLHE	chrX	154743776	+	AAGCTCTGGCAATCTACCGA
3513	sgTMLHE_3	TMLHE	chrX	154754253	+	GACGTAATCAAAGCGCATCA
3514	sgTMLHE_4	TMLHE	chrX	154741387	+	GGTGTACGCAGTGTCACCTC
3515	sgTMLHE_5	TMLHE	chrX	154736695	-	GGTACTTCAAAAGGCACCTG
3516	sgTMLHE_6	TMLHE	chrX	154774846	+	AAGTTGGGCTGTGGAAGGGC
3517	sgTMLHE_7	TMLHE	chrX	154741438	-	ACAGAGAAACCATTTATGGG
3518	sgTMLHE_8	TMLHE	chrX	154736757	-	CTTAAACATGAAGGAACTGG
3519	sgTMLHE_9	TMLHE	chrX	154736659	+	GCTTCAATGGCACTTTACTG
3520	sgTMLHE_10	TMLHE	chrX	154774907	+	TGTGTAGGTGGGACAATCTG
3521	sgTMPPE_1	TMPPE	chr3	33135180	-	GCTCAGGCCTGCCCTGGCAG
3522	sgTMPPE_2	TMPPE	chr3	33134904	+	GAGGAGCGACAGCCGTCCGC
3523	sgTMPPE_3	TMPPE	chr3	33134744	-	GCCACACGGGCCCAACGTGG
3524	sgTMPPE_4	TMPPE	chr3	33134691	-	GGACTGGATCTGCTTGGCTG
3525	sgTMPPE_5	TMPPE	chr3	33134622	-	GGATCTTGACAAGGCCCTGG
3526	sgTMPPE_6	TMPPE	chr3	33134338	-	TCACAGAGCTCATCCTGCAG
3527	sgTMPPE_7	TMPPE	chr3	33135170	+	GTCACTCCCACTGCCAGGGC
3528	sgTMPPE_8	TMPPE	chr3	33135022	-	ACTTGGGCCCCACAGTGGGC
3529	sgTMPPE_9	TMPPE	chr3	33134413	+	GACATACACGAATGTAGCCT
3530	sgTMPPE_10	TMPPE	chr3	33135108	-	CCCGGCTGTGAAAACTGTGG
3531	sgTPH1_1	TPH1	chr11	18050858	-	GAAGAGGAGATTAAGACCTG
3532	sgTPH1_2	TPH1	chr11	18050797	+	GAGATACTCTCTGCAAGCAT
3533	sgTPH1_3	TPH1	chr11	18048135	+	GTAACCAGCCACAGGACGGA
3534	sgTPH1_4	TPH1	chr11	18050892	-	TAGTGGAGACCCCATTCCAA
3535	sgTPH1_5	TPH1	chr11	18047196	+	AACTAGGTTCAGCCAAAAGC
3536	sgTPH1_6	TPH1	chr11	18047169	-	ATTCTCCCAAGAAATTGGCT

3537	sgTPH1_7	TPH1	chr11	18054905	-	CAGGTATGGAAACTGTTCCT
3538	sgTPH1_8	TPH1	chr11	18048024	+	TAGAAGTACTTACGGCTCTG
3539	sgTPH1_9	TPH1	chr11	18047145	-	TTCTCTTGGCGCTTCAGAGG
3540	sgTPH1_10	TPH1	chr11	18044437	+	GGGATCAAAGGGCTTTACTT
3541	sgTPH2_1	TPH2	chr12	72388324	+	AGATGTTCAGAAACTAGCCA
3542	sgTPH2_2	TPH2	chr12	72332795	+	GTAAATACTGGGCACGGAGA
3543	sgTPH2_3	TPH2	chr12	72338391	-	CTAACTCAGAGATCTTCCGA
3544	sgTPH2_4	TPH2	chr12	72335388	+	AATGACGACAAAGGCAACAA
3545	sgTPH2_5	TPH2	chr12	72338363	+	CAGAGCTAGAGGATGTGCCC
3546	sgTPH2_6	TPH2	chr12	72366296	+	TAGTGGTCAGCCCATTCCCA
3547	sgTPH2_7	TPH2	chr12	72366423	-	CCACAGTATTTAGTCAGCAG
3548	sgTPH2_8	TPH2	chr12	72372732	+	AAAGGTCTGGCTTCACGGTG
3549	sgTPH2_9	TPH2	chr12	72372779	-	AGTCCTGCCAGAAAGTCTCG
3550	sgTPH2_10	TPH2	chr12	72416211	+	CAAGCAAGAAGGGCAACTGC
3551	sgTPO_1	TPO	chr2	1500530	-	GAAAGGACTGACCTTTGCAG
3552	sgTPO_2	TPO	chr2	1520672	-	ATGGAGATCCAAGTCACCCG
3553	sgTPO_3	TPO	chr2	1426851	+	GGAGGAAAGCAAGCGCCTGG
3554	sgTPO_4	TPO	chr2	1488456	-	GGACACAGTGGGGTTGGCGG
3555	sgTPO_5	TPO	chr2	1488468	-	GGAGAACACGTTGGACACAG
3556	sgTPO_6	TPO	chr2	1488495	-	GGCATGGCCGAAGCGGAAGG
3557	sgTPO_7	TPO	chr2	1488527	-	GCGTCCAGCCTCCTCACCAG
3558	sgTPO_8	TPO	chr2	1497568	+	GTGCAGGTTACAATGAGTGG
3559	sgTPO_9	TPO	chr2	1507765	-	GGTGTTTCTGCACCTCGCAG
3560	sgTPO_10	TPO	chr2	1507830	+	GACGATGGGAGAACCTGCGT
3561	sgTYW5_1	TYW5	chr2	200820147	+	CGAGAAACGCCCTCCAGCCG
3562	sgTYW5_2	TYW5	chr2	200813138	-	ATGTACAAGCAAATGGACAG
3563	sgTYW5_3	TYW5	chr2	200813180	-	ACAGAGAAAACCTCTTGTGT
3564	sgTYW5_4	TYW5	chr2	200801136	+	TATAAATACTGGGCATCTCG
3565	sgTYW5_5	TYW5	chr2	200820118	-	CATGCAGCACCTCTACCCAC
3566	sgTYW5_6	TYW5	chr2	200813167	+	TCAATCCCTTCCAACACAAG
3567	sgTYW5_7	TYW5	chr2	200800717	+	GTGGATATTTAGCCAAGTCT
3568	sgTYW5_8	TYW5	chr2	200813116	-	GATTACCTAAGCCAAGTTGG
3569	sgTYW5_9	TYW5	chr2	200803671	-	GTTCACCAGGATTACAACTA
3570	sgTYW5_10	TYW5	chr2	200820106	+	AGGGCCTCACCTGTGGGTAG
3571	sgUQCRFS1_1	UQCRFS1	chr19	29704005	+	GCGGGATGCTACCGACAACA
3572	sgUQCRFS1_2	UQCRFS1	chr19	29703807	+	CTCACCATTGAGGCCCACGG
3573	sgUQCRFS1_3	UQCRFS1	chr19	29698958	-	GTCTTCAAGAGAAAGCAGCG
3574	sgUQCRFS1_4	UQCRFS1	chr19	29699030	-	TTATTCCCACACAGACATCA
3575	sgUQCRFS1_5	UQCRFS1	chr19	29698740	+	GGTTCTATGACGCACAAACA
3576	sgUQCRFS1_6	UQCRFS1	chr19	29698495	+	GTGGGGACTTCAAGGTTGAG
3577	sgUQCRFS1_7	UQCRFS1	chr19	29698724	-	AACCCAGAAGGAAATTGAGC
3578	sgUQCRFS1_8	UQCRFS1	chr19	29698948	-	GAAAGCAGCGAGGCTAGGAA
3579	sgUQCRFS1_9	UQCRFS1	chr19	29703897	+	CAACACAGGCTGCTCCGGGG
3580	sgUQCRFS1_10	UQCRFS1	chr19	29703966	+	GCGGGACGTGGCCGACAGGA
3581	sgUTY_1	UTY	chrY	15591475	-	GAGGCAAAGAAAATGGCGGA
3582	sgUTY_2	UTY	chrY	15448132	+	GTGATACTGGGCTGACCCAG
3583	sgUTY_3	UTY	chrY	15591516	+	GGTAGTGAGCGACACTGCGC
3584	sgUTY_4	UTY	chrY	15591495	+	ATCACCGAAGGCAACAGCGG
3585	sgUTY_5	UTY	chrY	15591416	-	CCTGACAGTCGAGGAAAGGG
3586	sgUTY_6	UTY	chrY	15591175	+	GCCTCACGAACCCGAAGAGA
3587	sgUTY_7	UTY	chrY	15526631	+	AGTAGAAGTAGACCAAACCA
3588	sgUTY_8	UTY	chrY	15478229	-	AGTAGGAGACAAAGCCACAA
3589	sgUTY_9	UTY	chrY	15469827	-	GGTTCTGATAACTGGAATGG
3590	sgUTY_10	UTY	chrY	15467257	-	GAGACACAAAGAAGTTGCTC
3591	sgXDH_1	XDH	chr2	31624104	-	GAATAGGAAGCACCAAGACG
3592	sgXDH_2	XDH	chr2	31605869	-	GAAGCAAGTCAAGTCTGTGG
3593	sgXDH_3	XDH	chr2	31560649	+	GAAGCAGCCAGGAAGAGGGG
3594	sgXDH_4	XDH	chr2	31621546	-	GAGAGAATTGCCAAAAGCCA
3595	sgXDH_5	XDH	chr2	31620536	-	GGGCTTCCGGACCTTTGCCA
3596	sgXDH_6	XDH	chr2	31609382	-	GAAGCAGCTGCGATTTGAAG
3597	sgXDH_7	XDH	chr2	31605932	-	GCTTCCTGCCCAAAAGACAG

3598	sgXDH_8	XDH	chr2	31604545	+	GTTGAGGTCGGAGATGGGGC
3599	sgXDH_9	XDH	chr2	31602783	+	GCAGGGTCTTTCTGTAGCCA
3600	sgXDH_10	XDH	chr2	31602735	-	AGAGATCCCCTACAGCAGGG
3601	sgSLC46A1_1	SLC46A1	NA	NA	NA	GACTCCAAACTAATCGGCTA
3602	sgSLC46A1_2	SLC46A1	NA	NA	NA	GATGCTGGCAAGCCTCCTCG
3603	sgSLC46A1_3	SLC46A1	NA	NA	NA	GAAGGCCACCGAAGTCGCCG
3604	sgSLC46A1_4	SLC46A1	NA	NA	NA	GGCCACCGAAGTCGCCGAGG
3605	sgSLC46A1_5	SLC46A1	NA	NA	NA	GCCCTCCTCGGCGACTTCGG
3606	sgSLC46A1_6	SLC46A1	NA	NA	NA	GGAGCTTGGAGCGACAGTGT
3607	sgSLC46A1_7	SLC46A1	NA	NA	NA	GCCGCCCACGTTCATGTAGA
3608	sgSLC46A1_8	SLC46A1	NA	NA	NA	GGCGGGTGCCATTGTAGCCG
3609	sgSLC46A1_9	SLC46A1	NA	NA	NA	GGCACAGCACGGCAGCCGCA
3610	sgSLC46A1_10	SLC46A1	NA	NA	NA	GGGCGGGCGCGGGGCTTTTC
3611	sgIDO2_1	IDO2	NA	NA	NA	GGAGACTTCGACAAATGGAA
3612	sgIDO2_2	IDO2	NA	NA	NA	GGGCTGCAAGATAGCATTCG
3613	sgIDO2_3	IDO2	NA	NA	NA	ATCCAAGGCCTATAATGATC
3614	sgIDO2_4	IDO2	NA	NA	NA	TCAGGAACTGGCAGCTCAGC
3615	sgIDO2_5	IDO2	NA	NA	NA	CCAGTTCCTGAAGGGTCACC
3616	sgIDO2_6	IDO2	NA	NA	NA	CTGAGCTGCCAGTTCCTGAA
3617	sgIDO2_7	IDO2	NA	NA	NA	GCTGAGCTGCCAGTTCCTGA
3618	sgIDO2_8	IDO2	NA	NA	NA	TCACCAGCTTCAAGCTCATG
3619	sgIDO2_9	IDO2	NA	NA	NA	AATCAATTGAGGAAGTTTGT
3620	sgIDO2_10	IDO2	NA	NA	NA	TTGTTGGCAATTTCCATCCA
3621	sgCYP4F8_1	CYP4F8	NA	NA	NA	GCGGCGGCCGTTGTGATAGA
3622	sgCYP4F8_2	CYP4F8	NA	NA	NA	GCCTTTGGCTGCCGTACTAG
3623	sgCYP4F8_3	CYP4F8	NA	NA	NA	GCAGGCGTCACAAGCGACGG
3624	sgCYP4F8_4	CYP4F8	NA	NA	NA	GGTTATTCCGTTTCACTACA
3625	sgCYP4F8_5	CYP4F8	NA	NA	NA	GTGCAGCCGCGGGCGAATGT
3626	sgCYP4F8_6	CYP4F8	NA	NA	NA	GTTTTCTGGGTCGAAGCGGA
3627	sgCYP4F8_7	CYP4F8	NA	NA	NA	GGGCGTTTTCTGGGTCGAAG
3628	sgCYP4F8_8	CYP4F8	NA	NA	NA	GACCTCTTCTGGGCGTTTTC
3629	sgCYP4F8_9	CYP4F8	NA	NA	NA	ACCTATGCCTTCTATCACAA
3630	sgCYP4F8_10	CYP4F8	NA	NA	NA	TCCGGTGTTTCCCGCAGCCC
3631	sgABL1_1	ABL1	chr9	133738383	+	GAAGAAATACAGCCTGACGG
3632	sgABL1_2	ABL1	chr9	133730343	+	GCTGAGTATCTGCTGAGCAG
3633	sgABL1_3	ABL1	chr9	133738194	+	GGTTCATCATCATTCAACGG
3634	sgABL1_4	ABL1	chr9	133747512	+	GCAGGAGGACACCATGGAGG
3635	sgABL1_5	ABL1	chr9	133748298	-	GTAGTCCAGGAGGTTCCCGT
3636	sgABL1_6	ABL1	chr9	133748320	+	GAGGGAGTGCAACCGGCAGG
3637	sgABL1_7	ABL1	chr9	133753872	+	GGTGTATGAGCTGCTAGAGA
3638	sgABL1_8	ABL1	chr9	133755950	+	CAGAGCTGCCCACCAAGACG
3639	sgABL1_9	ABL1	chr9	133729489	-	GCGGCTTCACTCAGACCCTG
3640	sgABL1_10	ABL1	chr9	133738163	-	GGTGTTGAAGCGGCTCTCGG
3641	sgAPBA1_1	APBA1	chr9	72131727	-	GGAGTACACGGAGCAGGCAG
3642	sgAPBA1_2	APBA1	chr9	72131580	+	AGGGCTCGTCCTCACCGCGG
3643	sgAPBA1_3	APBA1	chr9	72132096	-	GGAGGGGTCTGCGGAGGTGG
3644	sgAPBA1_4	APBA1	chr9	72132081	-	GGTGGAGGTGACCGACGAGG
3645	sgAPBA1_5	APBA1	chr9	72131661	+	CCAGCGAGTGGAAGTGCAGG
3646	sgAPBA1_6	APBA1	chr9	72131460	-	CGAGCAGGAGCGCGACGAGG
3647	sgAPBA1_7	APBA1	chr9	72131325	-	GGACAGCTACGAGCAGGAGG
3648	sgAPBA1_8	APBA1	chr9	72131064	-	GGCCATCAAGGACATCAAGG
3649	sgAPBA1_9	APBA1	chr9	72130945	+	CTGGTCGTCACAGTCCCTGG
3650	sgAPBA1_10	APBA1	chr9	72064617	+	CATGTTGGCAATGATCACGG
3651	sgAPBB1_1	APBB1	chr11	6424582	-	GGACTGAAGGAACCTGAGGA
3652	sgAPBB1_2	APBB1	chr11	6424416	-	AGAGCCGTTGCCCCAAGAGG
3653	sgAPBB1_3	APBB1	chr11	6423916	-	AGGCTGGGTAGAGATGACCG
3654	sgAPBB1_4	APBB1	chr11	6417339	+	CAAGATGGTGAGGGTAGCAG
3655	sgAPBB1_5	APBB1	chr11	6417070	+	GAACATGTGGCAGCAGAAGG
3656	sgAPBB1_6	APBB1	chr11	6425027	+	GAAGGCGTTGGGGTTCCAGA
3657	sgAPBB1_7	APBB1	chr11	6423415	-	GGATCTGCTACTGCAGCTGG
3658	sgAPBB1_8	APBB1	chr11	6422848	+	GTGCCTCACAGCGAAACACG

3659	sgAPBB1_9	APBB1	chr11	6422810	+	GCAGATCTCATGCAGGCTGG
3660	sgAPBB1_10	APBB1	chr11	6422614	+	AGAGTCCATTTACCAAGCAG
3661	sgAPBB2_1	APBB2	chr4	40823876	+	GGGATAACTCACCATGCAGG
3662	sgAPBB2_2	APBB2	chr4	40829206	-	ACGGAAGAATGCCAAAGCGC
3663	sgAPBB2_3	APBB2	chr4	40825696	-	GCTGTCAGTGAACATGAACG
3664	sgAPBB2_4	APBB2	chr4	40823944	+	AAACGTGGCACTCAAAGCGC
3665	sgAPBB2_5	APBB2	chr4	40825723	-	TATGACCTCATCCAACAAGG
3666	sgAPBB2_6	APBB2	chr4	40824007	-	GTCCTTCATGGGTGTTGGGA
3667	sgAPBB2_7	APBB2	chr4	40823929	+	TAGGCTCGCACCAGAAAACG
3668	sgAPBB2_8	APBB2	chr4	40829156	+	AGGGACATCGAGGTTCACAT
3669	sgAPBB2_9	APBB2	chr4	40829136	+	GCCACTGACTTACCTTGCAA
3670	sgAPBB2_10	APBB2	chr4	40829146	+	TACCTTGCAAAGGGACATCG
3671	sgAPBB3_1	APBB3	chr5	139943809	-	GCTGGGCAAGGATTACATGC
3672	sgAPBB3_2	APBB3	chr5	139943468	-	GGACCACAGTCTGGAGGTGG
3673	sgAPBB3_3	APBB3	chr5	139943353	+	GCATCTCCTAGTTCCCAGGT
3674	sgAPBB3_4	APBB3	chr5	139943181	-	TGGAGAGTTCACTGGACCGG
3675	sgAPBB3_5	APBB3	chr5	139941939	+	TCACCCCAGGCACCATCTGG
3676	sgAPBB3_6	APBB3	chr5	139941016	-	GATCTTGTCAGAGCGAGTAG
3677	sgAPBB3_7	APBB3	chr5	139940959	+	GCAGGTCTTCAGGAGAGATG
3678	sgAPBB3_8	APBB3	chr5	139940606	-	ACTGCCAGTCACCAAGGCCA
3679	sgAPBB3_9	APBB3	chr5	139940071	-	GGCAGAGGCCAGTACAGAGG
3680	sgAPBB3_10	APBB3	chr5	139939933	-	CTGGTGCCAGCCCCATGCAG
3681	sgAPLP1_1	APLP1	chr19	36365680	+	GTGCGGAGCAGAAGGAACAG
3682	sgAPLP1_2	APLP1	chr19	36359558	-	GCGACTTAGACCGCGAGCAG
3683	sgAPLP1_3	APLP1	chr19	36360640	-	GCTGTGGGTCTGGTTCCCAG
3684	sgAPLP1_4	APLP1	chr19	36361847	-	GCACCAGCGCTCCATGGGGA
3685	sgAPLP1_5	APLP1	chr19	36361902	-	GGAAGGGCACAACCTGGTGG
3686	sgAPLP1_6	APLP1	chr19	36362211	+	AATGTGAGAGTTCAACCCGG
3687	sgAPLP1_7	APLP1	chr19	36362230	+	GAGGCATCAGGAGGCACAGG
3688	sgAPLP1_8	APLP1	chr19	36363438	+	GGGGAAATCAGTGAGCACGA
3689	sgAPLP1_9	APLP1	chr19	36363467	+	GAGGGCCAAGATGGACCTGG
3690	sgAPLP1_10	APLP1	chr19	36365482	-	GATAAGGGCGATGACGCGGG
3691	sgAPP_1	APP	chr21	27462247	+	CGGCCGGCTCACCTAAGCAG
3692	sgAPP_2	APP	chr21	27284055	-	CCAGCCAACACAGAAAACGA
3693	sgAPP_3	APP	chr21	27425603	-	GCAAATTCTTACACCAGGAG
3694	sgAPP_4	APP	chr21	27394333	-	CAAAGTAGTAGAAGTAGCAG
3695	sgAPP_5	APP	chr21	27347369	+	GCAGCGAGACCTACCCGAGG
3696	sgAPP_6	APP	chr21	27326970	+	TGCGCTCATAAATCACACGG
3697	sgAPP_7	APP	chr21	27326931	-	GCTCTACAACGTGCCTGCAG
3698	sgAPP_8	APP	chr21	27284152	-	GACCGAAACGAAAACCACCG
3699	sgAPP_9	APP	chr21	27284135	+	CACGGGAAGGAGCTCCACGG
3700	sgAPP_10	APP	chr21	27284082	+	TCAGCCCCAAAAGAATGCCA
3701	sgASAH2_1	ASAH2	chr10	52008335	+	GAATGTCTCCAAGTTAGAGA
3702	sgASAH2_2	ASAH2	chr10	52008246	-	GGGACCATTGAAAACCACAA
3703	sgASAH2_3	ASAH2	chr10	52008284	+	GCTGAGAAGGGCCACTGTGA
3704	sgASAH2_4	ASAH2	chr10	52005146	+	GTTGGGCAGCTGTGGAGCCC
3705	sgASAH2_5	ASAH2	chr10	52005128	+	GCTGGGTGGCTGTGGAGCGT
3706	sgASAH2_6	ASAH2	chr10	52005114	+	GGTGGCTGTGGAATGCTGGG
3707	sgASAH2_7	ASAH2	chr10	52002988	-	TGTCAGCATCGACATAGGCA
3708	sgASAH2_8	ASAH2	chr10	52008304	+	TGGCACTCATCATTACAAGG
3709	sgASAH2_9	ASAH2	chr10	52005157	+	GTGGAGCCCTGGGTGGCTGG
3710	sgASAH2_10	ASAH2	chr10	52005096	+	AGTGGCTGTGGAGCTCTGGG
3711	sgATOX1_1	ATOX1	chr5	151131309	-	TCTGTGGACATGACCTGTGG
3712	sgATOX1_2	ATOX1	chr5	151131325	-	TCAGAAGCACGAGTTCTCTG
3713	sgATOX1_3	ATOX1	chr5	151131267	-	CGGGTCCTCAATAAGCTTGG
3714	sgATOX1_4	ATOX1	chr5	151131287	-	GCTGTGCTGAAGCTGTCTCT
3715	sgATOX1_5	ATOX1	chr5	151125879	+	GCCCCTGCTACTCAAGGCCA
3716	sgATOX1_6	ATOX1	chr5	151125873	+	GACCAGGCCCCTGCTACTCA
3717	sgATOX1_7	ATOX1	chr5	151125978	-	CATTGACCTGCCCAACAAGA
3718	sgATOX1_8	ATOX1	chr5	151125951	-	CATTGAATCTGAGCACAGCA
3719	sgATOX1_9	ATOX1	chr5	151125899	-	GGAAAGACTGTTTCCTACCT

3720	sgATOX1_10	ATOX1	chr5	151131293	+	AGCTTCAGCACAGCCTCCAC
3721	sgATP6AP1_1	ATP6AP1	chrX	153657474	-	CCTGGGACCCAGCTCCAGGG
3722	sgATP6AP1_2	ATP6AP1	chrX	153660185	+	GAGGATTTCACAGCATATGG
3723	sgATP6AP1_3	ATP6AP1	chrX	153660767	-	GAATGAGCAGCAGAGCAGGG
3724	sgATP6AP1_4	ATP6AP1	chrX	153662563	+	GATGTAGCCGTGGTGGCCGG
3725	sgATP6AP1_5	ATP6AP1	chrX	153662733	-	GTTCCTGCACCCCAAAGGTG
3726	sgATP6AP1_6	ATP6AP1	chrX	153663638	-	GGGCAGACACTGGGTAGAGG
3727	sgATP6AP1_7	ATP6AP1	chrX	153663706	-	GGAAGCATTGAAGTAGGCGA
3728	sgATP6AP1_8	ATP6AP1	chrX	153657038	+	TATGATGGCGGCCATGGCGA
3729	sgATP6AP1_9	ATP6AP1	chrX	153660632	-	CAGGAAGCACCAGTGAGGAG
3730	sgATP6AP1_10	ATP6AP1	chrX	153660655	-	GCTGACTGCATACCAGTCGA
3731	sgATP6V0A2_1	ATP6V0A2	chr12	124220117	+	GGAGCGGAGGGAGATCCAGG
3732	sgATP6V0A2_2	ATP6V0A2	chr12	124206938	-	GGCTGGCCTCTCCTTCAGGA
3733	sgATP6V0A2_3	ATP6V0A2	chr12	124209200	+	GGAGCAGTTGCAGAAGCTCG
3734	sgATP6V0A2_4	ATP6V0A2	chr12	124210811	+	AGAGGCTGGGAGCAAAACTG
3735	sgATP6V0A2_5	ATP6V0A2	chr12	124212334	+	GTGTCTGGCCTAATTAACCA
3736	sgATP6V0A2_6	ATP6V0A2	chr12	124220084	-	GATAGGGGTACACGTGGCAG
3737	sgATP6V0A2_7	ATP6V0A2	chr12	124220104	+	CAAACACAGCCGAGGAGCGG
3738	sgATP6V0A2_8	ATP6V0A2	chr12	124221602	+	CCAGGTACTGCACAAAACCG
3739	sgATP6V0A2_9	ATP6V0A2	chr12	124221655	-	ACGGCTGTAGACAGACTCGG
3740	sgATP6V0A2_10	ATP6V0A2	chr12	124221740	+	CAACAAGTGCCTCATTGCTG
3741	sgATP6V0D1_1	ATP6V0D1	chr16	67487511	-	GCTCAAGGAGAAGATGGTGG
3742	sgATP6V0D1_2	ATP6V0D1	chr16	67478528	+	CTTGGGCACGAGCTCAGCGA
3743	sgATP6V0D1_3	ATP6V0D1	chr16	67473017	+	TGGTGATGATGAAGGCGCGG
3744	sgATP6V0D1_4	ATP6V0D1	chr16	67478497	-	AGGCAGCTTCGAGCAGATGG
3745	sgATP6V0D1_5	ATP6V0D1	chr16	67478446	-	GCTCTACAATGCCATTCTGG
3746	sgATP6V0D1_6	ATP6V0D1	chr16	67473214	-	AAGTTCTGCACCCTACTGGG
3747	sgATP6V0D1_7	ATP6V0D1	chr16	67472944	+	GGGTAGAGCCGCCCACAGTG
3748	sgATP6V0D1_8	ATP6V0D1	chr16	67472754	-	GAGTACAAGCTGCTCTTCGA
3749	sgATP6V0D1_9	ATP6V0D1	chr16	67514969	+	AAGCTCCGGGAAGAACGACA
3750	sgATP6V0D1_10	ATP6V0D1	chr16	67514959	-	CCCGGAGCTTTACTTTAACG
3751	sgATP6V1A_1	ATP6V1A	chr3	113503085	+	GGAGATCCTGTACTTCGCAC
3752	sgATP6V1A_2	ATP6V1A	chr3	113503660	-	GAGGTATCATAATTCCCAGG
3753	sgATP6V1A_3	ATP6V1A	chr3	113507701	+	AGTCCTCCGGGACTTCCCAG
3754	sgATP6V1A_4	ATP6V1A	chr3	113503115	-	GGACCAAGCTCTACAGAGAG
3755	sgATP6V1A_5	ATP6V1A	chr3	113503543	+	GTTGGTAGTCATATCACTGG
3756	sgATP6V1A_6	ATP6V1A	chr3	113513976	-	GCCTGCTCGTTCATAAAACG
3757	sgATP6V1A_7	ATP6V1A	chr3	113499898	+	GGTTACAGCCTGTGACATGG
3758	sgATP6V1A_8	ATP6V1A	chr3	113503175	-	TGACTGCTGATATCCGACAA
3759	sgATP6V1A_9	ATP6V1A	chr3	113503199	+	CAAAGCATCTACATCCCCAG
3760	sgATP6V1A_10	ATP6V1A	chr3	113505128	-	TACAGGCCATACTTGCACCA
3761	sgATP6V1G1_1	ATP6V1G1	chr9	117354823	+	TGAAAACAGGAAAGAACCGG
3762	sgATP6V1G1_2	ATP6V1G1	chr9	117354851	+	GCAGGCCAAAGAAGAAGCTC
3763	sgATP6V1G1_3	ATP6V1G1	chr9	117354896	+	GCAGAGGGAGAAAGAATTCA
3764	sgATP6V1G1_4	ATP6V1G1	chr9	117350117	+	CCATGGCTAGTCAGTCTCAG
3765	sgATP6V1G1_5	ATP6V1G1	chr9	117350160	+	CGAGAAGCGGGCAGCCGAGA
3766	sgATP6V1G1_6	ATP6V1G1	chr9	117350189	-	AAACTCACTTTTGCGGGCCT
3767	sgATP6V1G1_7	ATP6V1G1	chr9	117359918	-	TCTGCCGGAAGTATGTCTGG
3768	sgATP6V1G1_8	ATP6V1G1	chr9	117359879	+	TGAAGTGGAGAAGGAGACCC
3769	sgATP6V1G1_9	ATP6V1G1	chr9	117359844	+	CTCCAGGCATTGGGATCCCG
3770	sgATP6V1G1_10	ATP6V1G1	chr9	117359864	-	CTTCAGTGCTGCAACTGCCA
3771	sgBLVRB_1	BLVRB	chr19	40971542	+	GGTGAGCCCGGTCTGGCCAG
3772	sgBLVRB_2	BLVRB	chr19	40964366	+	GAACATCTCCCACTACCACG
3773	sgBLVRB_3	BLVRB	chr19	40964335	-	GATGTGGACAAGACCGTGGC
3774	sgBLVRB_4	BLVRB	chr19	40964077	-	TCATGGTGTGGACAAGGTCG
3775	sgBLVRB_5	BLVRB	chr19	40971521	+	TTGCACCGCCTGCGCCAGGG
3776	sgBLVRB_6	BLVRB	chr19	40957319	+	GCACCTTGTGCATCCGGATG
3777	sgBLVRB_7	BLVRB	chr19	40964403	-	CCTCCAGGCTGCCATCAGAG
3778	sgBLVRB_8	BLVRB	chr19	40964101	-	GAACATTGTGGCAGCCATGA
3779	sgBLVRB_9	BLVRB	chr19	40957273	-	GCTGTGATGCCGCCACACAT
3780	sgBLVRB_10	BLVRB	chr19	40971569	+	GAAGATCGCGATCTTCTTGA

3781	sgBOLA2_1	BOLA2	chr16	29465727	+	CAAGCAGCCGGGCTTTCCAG
3782	sgBOLA2_2	BOLA2	chr16	29465688	-	GACGTACGCCTTAGTAAGGG
3783	sgBOLA2_3	BOLA2	chr16	29465532	+	CCCGCTGCAGCTTCTCGCGG
3784	sgBOLA2_4	BOLA2	chr16	29465334	+	TACCTGTGTCTCTGAAGCAG
3785	sgBOLA2_5	BOLA2	chr16	29465746	-	GCGCGAAAAGCCTGGACCGC
3786	sgBOLA2_6	BOLA2	chr16	29465664	-	AGTGAGTTTTCCAGCGGAAG
3787	sgBOLA2_7	BOLA2	chr16	29465604	+	GCAGGGACACAACCCCAGCT
3788	sgBOLA2_8	BOLA2	chr16	29465637	-	TGTAAGGCAGCAAGGTAGCG
3789	sgBOLA2_9	BOLA2	chr16	29465587	+	GCTGGAACGGCAGCCCAGCA
3790	sgBOLA2_10	BOLA2	chr16	29465364	-	GTGGTGTCGGCCAAGTTCGA
3791	sgBOLA2B_1	BOLA2B	chr16	30205069	+	CAAGCAGCCGGGCTTTCCAG
3792	sgBOLA2B_2	BOLA2B	chr16	30205030	-	GACGTACGCCTTAGTAAGGG
3793	sgBOLA2B_3	BOLA2B	chr16	30204874	+	CCCGCTGCAGCTTCTCGCGG
3794	sgBOLA2B_4	BOLA2B	chr16	30204676	+	TACCTGTGTCTCTGAAGCAG
3795	sgBOLA2B_5	BOLA2B	chr16	30205088	-	GCGCGAAAAGCCTGGACCGC
3796	sgBOLA2B_6	BOLA2B	chr16	30205006	-	AGTGAGTTTTCCAGCGGAAG
3797	sgBOLA2B_7	BOLA2B	chr16	30204946	+	GCAGGGACACAACCCCAGCT
3798	sgBOLA2B_8	BOLA2B	chr16	30204979	-	TGTAAGGCAGCAAGGTAGCG
3799	sgBOLA2B_9	BOLA2B	chr16	30204929	+	GCTGGAACGGCAGCCCAGCA
3800	sgBOLA2B_10	BOLA2B	chr16	30204706	-	GTGGTGTCGGCCAAGTTCGA
3801	sgCA6_1	CA6	chr1	9017282	+	GGTGCGTCCTCGGAGATCAG
3802	sgCA6_2	CA6	chr1	9017299	+	CAGCGGCTCTGAGCACACCG
3803	sgCA6_3	CA6	chr1	9019054	-	GTTTCTGCTTACCTCAACGA
3804	sgCA6_4	CA6	chr1	9027794	-	GAGGCGTGGTGAGTGAGCCA
3805	sgCA6_5	CA6	chr1	9027833	-	AATCTGCCAGCACAAACCAG
3806	sgCA6_6	CA6	chr1	9030994	+	CCAGCCCCTGAACCACAGAG
3807	sgCA6_7	CA6	chr1	9019007	+	TGATATAGCCCAAGATGCGC
3808	sgCA6_8	CA6	chr1	9027743	-	GCAGCATGTCCTGAACGTCA
3809	sgCA6_9	CA6	chr1	9027773	-	GGTAGGTGTAGTAGTGCTGG
3810	sgCA6_10	CA6	chr1	9027813	-	TGGACGTTCTCAGTGCAGGG
3811	sgCA9_1	CA9	chr9	35673981	-	GGGATCAACAGAGGGAGCCA
3812	sgCA9_2	CA9	chr9	35674070	+	GAGGTTGCCCCGGATGCAGG
3813	sgCA9_3	CA9	chr9	35674142	+	GGAGGATCTGCCCAGTGAAG
3814	sgCA9_4	CA9	chr9	35674160	+	AGAGGATTCACCCAGAGAGG
3815	sgCA9_5	CA9	chr9	35674178	+	GGAGGATCCACCCGGAGAGG
3816	sgCA9_6	CA9	chr9	35676188	-	TCACCTCGGCAGGGAAACGG
3817	sgCA9_7	CA9	chr9	35679230	-	GAAGTAGCGGCTGAAGTCAG
3818	sgCA9_8	CA9	chr9	35679954	-	GCTGTCCACTCCAGCAGGGA
3819	sgCA9_9	CA9	chr9	35679980	-	GTACCTGGCTCAGCAGCCCG
3820	sgCA9_10	CA9	chr9	35674188	-	GGTAGATCCTCCTCTCCGGG
3821	sgCCDC115_1	CCDC115	chr2	131099635	-	GCAGCTGCTTGGGGACCTGG
3822	sgCCDC115_2	CCDC115	chr2	131099596	-	AACGGTGTTGAACGCCCGGG
3823	sgCCDC115_3	CCDC115	chr2	131099291	-	GGGACTCCAGAAGTTCAAGG
3824	sgCCDC115_4	CCDC115	chr2	131099614	-	GGAGCTGGAGGGGAAACGAA
3825	sgCCDC115_5	CCDC115	chr2	131099499	-	GGGCTGGCTCTCGCTCGCCA
3826	sgCCDC115_6	CCDC115	chr2	131099414	+	CTGGCGTGGAGGCAGACCTG
3827	sgCCDC115_7	CCDC115	chr2	131098545	+	GGGGTCCTGAGGGGCCTCAG
3828	sgCCDC115_8	CCDC115	chr2	131099678	+	TCGCAGGTCAAGCGCCGCCA
3829	sgCCDC115_9	CCDC115	chr2	131099256	-	TCCACGCCCCAGAGGAGGTG
3830	sgCCDC115_10	CCDC115	chr2	131098605	-	TTCTTTCCTCAGGTCTGCGG
3831	sgCDO1_1	CDO1	chr5	115146966	+	GTAGCATCTTCAGAAAGCAG
3832	sgCDO1_2	CDO1	chr5	115152041	+	GATGCGGATCAGATCAGCCA
3833	sgCDO1_3	CDO1	chr5	115152001	-	CGATGAGGTCAATGTAGAGG
3834	sgCDO1_4	CDO1	chr5	115151942	+	GGCGTACATTGCCCACTCGG
3835	sgCDO1_5	CDO1	chr5	115142122	+	GCATGGCATGTATCAAAAGG
3836	sgCDO1_6	CDO1	chr5	115152068	-	AGACCGAAGTGCTGAAGCCA
3837	sgCDO1_7	CDO1	chr5	115142138	+	AAGGTGGACTGTACAAGTGA
3838	sgCDO1_8	CDO1	chr5	115152025	+	CAAAGAGCTGGTGCAGGATG
3839	sgCDO1_9	CDO1	chr5	115151957	-	ACGAGAGCGACCCCACCGAG
3840	sgCDO1_10	CDO1	chr5	115148880	-	GTTGGGGTGAAGGACATGGC
3841	sgCIAO1_1	CIAO1	chr2	96933092	+	AAGGCCACCAGCGCACCGTG

3842	sgCIAO1_2	CIAO1	chr2	96933132	-	AGGCCAGGTAATTACCGCAG
3843	sgCIAO1_3	CIAO1	chr2	96933152	-	GGTAGCATCAAAGCTGGCAG
3844	sgCIAO1_4	CIAO1	chr2	96934227	+	GAAGCTGTACCGGGAGGAAG
3845	sgCIAO1_5	CIAO1	chr2	96934271	-	GGATTCATGGCCCTCAAGGG
3846	sgCIAO1_6	CIAO1	chr2	96932173	+	ACCCTGCTGGCCTCGTGCGG
3847	sgCIAO1_7	CIAO1	chr2	96932205	+	AATCCGCATCTGGGGCACGG
3848	sgCIAO1_8	CIAO1	chr2	96933359	+	ACAGTGTGTAACCACTCTCG
3849	sgCIAO1_9	CIAO1	chr2	96933641	+	GGTTTGGCACCCAAGTCAGG
3850	sgCIAO1_10	CIAO1	chr2	96935015	+	GCAGCGGCTCTGACCCCAGT
3851	sgCLSTN1_1	CLSTN1	chr1	9795026	-	GAAGTGGAGCCTGAAGGGGA
3852	sgCLSTN1_2	CLSTN1	chr1	9811666	-	GGAGAAGTCCTACAAAGCCA
3853	sgCLSTN1_3	CLSTN1	chr1	9804662	-	GAGCTGCTGCCATCCCCGAG
3854	sgCLSTN1_4	CLSTN1	chr1	9801170	-	GATAGAAACTCAGCTCGTGG
3855	sgCLSTN1_5	CLSTN1	chr1	9795114	+	AAGCTGCTCGGGCAAAATGG
3856	sgCLSTN1_6	CLSTN1	chr1	9794141	-	GGATACCTGTGAGGTCACGG
3857	sgCLSTN1_7	CLSTN1	chr1	9794114	-	AGAGGAGCTGAACCACGAGC
3858	sgCLSTN1_8	CLSTN1	chr1	9794099	-	CGAGCAGGAGAGCCTGGAGG
3859	sgCLSTN1_9	CLSTN1	chr1	9793578	-	CACCATGGCCAGCTACGAGG
3860	sgCLSTN1_10	CLSTN1	chr1	9791852	+	CTGACAGGTCAACAAAGGAG
3861	sgCLSTN2_1	CLSTN2	chr3	140167458	-	GCAGCTCTGTGACGATCTGG
3862	sgCLSTN2_2	CLSTN2	chr3	140167476	+	GCAGACTAATTACATTGGGA
3863	sgCLSTN2_3	CLSTN2	chr3	140178369	-	GAGGTCAATGATGCCAGAGG
3864	sgCLSTN2_4	CLSTN2	chr3	140178473	+	CAGGGTGCCAAAGTCCCCGA
3865	sgCLSTN2_5	CLSTN2	chr3	140185495	-	TCCGCAAGAGAAAGACGAGG
3866	sgCLSTN2_6	CLSTN2	chr3	140277558	-	GTGCCCCGGAGGGTGATCCG
3867	sgCLSTN2_7	CLSTN2	chr3	140277568	-	GAAGTGGTCTGTGCCCCGGA
3868	sgCLSTN2_8	CLSTN2	chr3	140277584	-	GGGCAGCAGGTCTCCAGAAG
3869	sgCLSTN2_9	CLSTN2	chr3	140282966	+	TATCACAGTCAACCCCATGG
3870	sgCLSTN2_10	CLSTN2	chr3	139894790	+	TAGTCAATAAGCACAAGCCA
3871	sgCLSTN3_1	CLSTN3	chr12	7288414	+	ACAGAGAAGCTGCAGTACAG
3872	sgCLSTN3_2	CLSTN3	chr12	7289434	-	CAACAGATCCACCTCCCCAG
3873	sgCLSTN3_3	CLSTN3	chr12	7289547	+	GCTGTGCAGGTGCCCCTGGG
3874	sgCLSTN3_4	CLSTN3	chr12	7290585	-	GGAAGGCAATCCTACAGCCG
3875	sgCLSTN3_5	CLSTN3	chr12	7290616	-	GGGCGGGCACTCTCAAGCAG
3876	sgCLSTN3_6	CLSTN3	chr12	7293886	+	ACAGTCACACTCTATACCGA
3877	sgCLSTN3_7	CLSTN3	chr12	7295484	-	GGTAGCCATGGAAGTAGTGG
3878	sgCLSTN3_8	CLSTN3	chr12	7295776	+	ACAGTCCCTGCTCACCCTGG
3879	sgCLSTN3_9	CLSTN3	chr12	7301597	-	GTAGCCCTCCACTTCAGGGA
3880	sgCLSTN3_10	CLSTN3	chr12	7303612	-	GTGTCCAGCCATCTCAGGCG
3881	sgCNNM2_1	CNNM2	chr10	104679554	+	GGAGCTCCGCACCAAGACGG
3882	sgCNNM2_2	CNNM2	chr10	104678432	+	GGTGGGCGAGAATGAGGAGA
3883	sgCNNM2_3	CNNM2	chr10	104678593	+	CCTTCACCGAGCACGAGCGG
3884	sgCNNM2_4	CNNM2	chr10	104678679	-	GTGCGGATGCCGCATCGCTG
3885	sgCNNM2_5	CNNM2	chr10	104679283	-	GAAGATGACGATACCGATGG
3886	sgCNNM2_6	CNNM2	chr10	104679742	-	ACAGTCATCGGGATCCACGA
3887	sgCNNM2_7	CNNM2	chr10	104678456	+	GATCATCGGGCTGCGACTGG
3888	sgCNNM2_8	CNNM2	chr10	104678536	-	GTACACCCGCAGCTTGACCC
3889	sgCNNM2_9	CNNM2	chr10	104678560	+	AGAACATCAATAACGAGACG
3890	sgCNNM2_10	CNNM2	chr10	104678722	-	GCGGCGGTTGAGAATGATGT
3891	sgCOQ6_1	COQ6	chr14	74420249	-	GAGAAGCGTTGCAGAGCCAG
3892	sgCOQ6_2	COQ6	chr14	74424839	+	GACCTCCCCAGACCGAGTGA
3893	sgCOQ6_3	COQ6	chr14	74424925	-	GGTAATATGAACCCAAGGGC
3894	sgCOQ6_4	COQ6	chr14	74425905	-	GAGCAGAGCAATAGGCCCAG
3895	sgCOQ6_5	COQ6	chr14	74426131	-	GGACCAAACCAAGGAACTCA
3896	sgCOQ6_6	COQ6	chr14	74426171	+	AGAGCTAGTTAGCATGGATG
3897	sgCOQ6_7	COQ6	chr14	74428026	-	GCAGCATGTCCCAACCCAAG
3898	sgCOQ6_8	COQ6	chr14	74428046	+	TGAGTACGTCAGGCCTCGGG
3899	sgCOQ6_9	COQ6	chr14	74428545	-	CCTGAGCAACACAAGCGGGG
3900	sgCOQ6_10	COQ6	chr14	74420230	+	AGGGTCAGCTCCATTTCCCC
3901	sgCOX10_1	COX10	chr17	13977715	+	TCAGGAATGTCAATAAGCAG
3902	sgCOX10_2	COX10	chr17	13980281	+	AGACAAAAGAGGAAAAGCGG

3903	sgCOX10_3	COX10	chr17	13972930	-	GGAGAGAGTGTGCGGAGATG
3904	sgCOX10_4	COX10	chr17	13972958	-	CGGGTACAGTACCTGTCAGG
3905	sgCOX10_5	COX10	chr17	14063226	+	TAGGACAAAGAACAGACCGC
3906	sgCOX10_6	COX10	chr17	14063245	-	CTGATCTGTCCACGAACCAG
3907	sgCOX10_7	COX10	chr17	13980178	-	GATAGGCTGGGCGGTGAGAG
3908	sgCOX10_8	COX10	chr17	13980270	+	TGTAGGGAAAGAGACAAAAG
3909	sgCOX10_9	COX10	chr17	14005546	-	ACTGACCTGATTGATGGAGT
3910	sgCOX10_10	COX10	chr17	13980190	-	TTTCTGGACAAAGATAGGCT
3911	sgCP_1	CP	chr3	148928055	-	GAAGAACAAAGTCCTGGGGA
3912	sgCP_2	CP	chr3	148904438	-	GTGTGGTACTTATTCAGCGC
3913	sgCP_3	CP	chr3	148895719	-	ACAAGGCCTCACAATGCACG
3914	sgCP_4	CP	chr3	148928109	-	GCAGATGACAAAGTATATCC
3915	sgCP_5	CP	chr3	148919990	-	GAACAAGGTACCACAAGAAT
3916	sgCP_6	CP	chr3	148903093	-	ATACTATATCGCAGCAGTGG
3917	sgCP_7	CP	chr3	148897430	-	GTGAAACTCTCACTTACGTA
3918	sgCP_8	CP	chr3	148939463	-	GGGATTATGCCTCTGACCAT
3919	sgCP_9	CP	chr3	148925138	+	AAGATGTCCTACCTTTCAGA
3920	sgCP_10	CP	chr3	148924043	+	AGTGTCTAACATGCTTCCCA
3921	sgCYB5R1_1	CYB5R1	chr1	202935921	+	GGAGAGTGACCTGAGGCCGG
3922	sgCYB5R1_2	CYB5R1	chr1	202935659	+	CAGGCAGCCCCAGAGTGTGG
3923	sgCYB5R1_3	CYB5R1	chr1	202936002	+	CCAGGGAGGCCAGCAGGACG
3924	sgCYB5R1_4	CYB5R1	chr1	202935081	+	CAGGCTGCCATCAATTCGGG
3925	sgCYB5R1_5	CYB5R1	chr1	202934594	-	GTGCACCCCAAATTTCCTGA
3926	sgCYB5R1_6	CYB5R1	chr1	202933748	-	AAACTGGGAATGATTGCCGG
3927	sgCYB5R1_7	CYB5R1	chr1	202932251	-	GGAGGACTTAGAGGAACTGC
3928	sgCYB5R1_8	CYB5R1	chr1	202935680	+	GGGCGGTGGGCAGGGCAAAG
3929	sgCYB5R1_9	CYB5R1	chr1	202935669	-	CCACCGCCCACCACACTCTG
3930	sgCYB5R1_10	CYB5R1	chr1	202932269	-	AAAGGATATCATCTTGCGGG
3931	sgCYB5R2_1	CYB5R2	chr11	7693734	-	AAATCAGCCACAACACCCGG
3932	sgCYB5R2_2	CYB5R2	chr11	7693682	+	CTACAGGAAGCCCTAAGACA
3933	sgCYB5R2_3	CYB5R2	chr11	7687775	-	GCTTCTTTTCTAGACAGAGG
3934	sgCYB5R2_4	CYB5R2	chr11	7693715	+	GCAGTCCAAAGCGGAACCTC
3935	sgCYB5R2_5	CYB5R2	chr11	7689718	-	TCACCTGGGAATGATTGCTG
3936	sgCYB5R2_6	CYB5R2	chr11	7687669	+	ATGGATGTCGTACCAATGGG
3937	sgCYB5R2_7	CYB5R2	chr11	7690883	+	GTCATCATCACTGGAGACAG
3938	sgCYB5R2_8	CYB5R2	chr11	7689013	+	GTGGCGAATGAGCTGCAACA
3939	sgCYB5R2_9	CYB5R2	chr11	7689732	-	AAAACACTGGCCGATCACCT
3940	sgCYB5R2_10	CYB5R2	chr11	7688979	+	ACATCCTGGTCCTGTCACTG
3941	sgCYB5R3_1	CYB5R3	chr22	43032723	-	GCTGCGGCTCATCGACCGGG
3942	sgCYB5R3_2	CYB5R3	chr22	43027372	+	GGCGTGACTCACCGACAGGG
3943	sgCYB5R3_3	CYB5R3	chr22	43027417	+	GCAGGGCAAAGCGGAAGCGC
3944	sgCYB5R3_4	CYB5R3	chr22	43027389	+	GGGAGGCCCAGGATGTGCTG
3945	sgCYB5R3_5	CYB5R3	chr22	43024146	+	GACCCGAATCACCTTTGCCC
3946	sgCYB5R3_6	CYB5R3	chr22	43027400	+	GATGTGCTGGGGTGACGGCA
3947	sgCYB5R3_7	CYB5R3	chr22	43023365	+	GCGCGGATCACCTGCAGCAT
3948	sgCYB5R3_8	CYB5R3	chr22	43032771	+	TGATGGCTGGCGTGGAGCGC
3949	sgCYB5R3_9	CYB5R3	chr22	43032739	-	CGGACATCAAGTACCCGCTG
3950	sgCYB5R3_10	CYB5R3	chr22	43024167	+	GGTAGACCAGCAGCCCACTG
3951	sgCYB5RL_1	CYB5RL	chr1	54656519	+	AATGATGCAGAAGGCCACGA
3952	sgCYB5RL_2	CYB5RL	chr1	54661271	-	GATGATGGCTGAGAGGGAAG
3953	sgCYB5RL_3	CYB5RL	chr1	54661256	-	GGAAGAGGACGACGACACTG
3954	sgCYB5RL_4	CYB5RL	chr1	54644866	-	GAAAACCTTCCTCCAAGAGC
3955	sgCYB5RL_5	CYB5RL	chr1	54644824	+	GGCTGAGTACAAAGAAGGTA
3956	sgCYB5RL_6	CYB5RL	chr1	54661200	+	GCCGCAGCACTGGGAAGGCA
3957	sgCYB5RL_7	CYB5RL	chr1	54661127	-	GGAGGCAGCCCAAGCCAGCA
3958	sgCYB5RL_8	CYB5RL	chr1	54656536	-	GCTGAACCCAGAGACCTTCG
3959	sgCYB5RL_9	CYB5RL	chr1	54656447	-	GGAACAGCCAGCTTGGCCTG
3960	sgCYB5RL_10	CYB5RL	chr1	54644960	+	GCTCTGCAGGATAGGCACCA
3961	sgCYP1A2_1	CYP1A2	chr15	75042132	-	TACCAGGCAGAAGATGGCAG
3962	sgCYP1A2_2	CYP1A2	chr15	75042289	-	GCAGGACGTCCCCGTAGCGC
3963	sgCYP1A2_3	CYP1A2	chr15	75042525	-	GGAAGCTGGGTCAGAGGCGA

3964	sgCYP1A2_4	CYP1A2	chr15	75044130	-	GTACATGAGGCTCCAGGAGA
3965	sgCYP1A2_5	CYP1A2	chr15	75044173	+	GAGGAAGATCCAGAAGGAGC
3966	sgCYP1A2_6	CYP1A2	chr15	75045572	+	GCTGTGTCTTCGTAAACCAG
3967	sgCYP1A2_7	CYP1A2	chr15	75042195	+	TGAAAAGTCCACCAGAGCCA
3968	sgCYP1A2_8	CYP1A2	chr15	75042249	-	CAGGTGCGGGTTCTTCCCCA
3969	sgCYP1A2_9	CYP1A2	chr15	75042299	+	GACGTCCTGCAGATCCGCAT
3970	sgCYP1A2_10	CYP1A2	chr15	75042327	-	GCTCAGCACCAGCACGGGCG
3971	sgDDX5_1	DDX5	chr17	62500927	-	GATTTGGAGGAAGTAGGGCA
3972	sgDDX5_2	DDX5	chr17	62500893	-	AAAGAAGTTTGGAAACCCTG
3973	sgDDX5_3	DDX5	chr17	62499589	-	AAGTCTACTTGTATCTACGG
3974	sgDDX5_4	DDX5	chr17	62502209	-	TATTCGAGTGACCGAGACCG
3975	sgDDX5_5	DDX5	chr17	62499944	+	GGAATGGCTGATGATTGATG
3976	sgDDX5_6	DDX5	chr17	62498633	-	GGAAGAGATCATGAGTGAGA
3977	sgDDX5_7	DDX5	chr17	62498120	+	GTACTAACCTAGCCCTCTGG
3978	sgDDX5_8	DDX5	chr17	62500430	-	ATTTGTTTTGCAGCAAGAGG
3979	sgDDX5_9	DDX5	chr17	62500117	-	GGAGTGGCACAGACTGGATC
3980	sgDDX5_10	DDX5	chr17	62498311	+	CTTGTCACCATGGATACCCA
3981	sgDEGS1_1	DEGS1	chr1	224377417	-	AATGCAACTGCCAAACGCAT
3982	sgDEGS1_2	DEGS1	chr1	224377719	-	GGTTTGGGGTTGATGAACAG
3983	sgDEGS1_3	DEGS1	chr1	224371053	+	CTCGCGGGAAGACTTCGAGT
3984	sgDEGS1_4	DEGS1	chr1	224377748	+	TCTGGAAGTTATCAATACCG
3985	sgDEGS1_5	DEGS1	chr1	224377768	-	GTCAAAAGTGACCTGTGCCA
3986	sgDEGS1_6	DEGS1	chr1	224377442	-	GAATAGCCAGAGTCATTGAG
3987	sgDEGS1_7	DEGS1	chr1	224377495	+	GCAAAGCAATGTGGAATCGC
3988	sgDEGS1_8	DEGS1	chr1	224377632	-	AACCAGCCCTCAAAATCGGT
3989	sgDEGS1_9	DEGS1	chr1	224377836	+	GCAGCATCTTTACTTGGCCT
3990	sgDEGS1_10	DEGS1	chr1	224377856	-	CAGAAATTGGGTGCAAACCC
3991	sgDEGS2_1	DEGS2	chr14	100625891	-	GAGCCGCAGCGACTTCGAGT
3992	sgDEGS2_2	DEGS2	chr14	100625862	-	CCGACCAGCCGCACACGCAG
3993	sgDEGS2_3	DEGS2	chr14	100615769	+	CGTAGGGCACACCCACGGGC
3994	sgDEGS2_4	DEGS2	chr14	100615641	+	CAGCCAGAGCAGCTTGCGGG
3995	sgDEGS2_5	DEGS2	chr14	100615988	+	GCACCGCCCACTTGAGGCGC
3996	sgDEGS2_6	DEGS2	chr14	100615746	+	GTGGTACTTCTTGAAGGAGG
3997	sgDEGS2_7	DEGS2	chr14	100615577	+	TGCGGGTCACGGCCTTGGGG
3998	sgDEGS2_8	DEGS2	chr14	100615547	-	CAACACGCTGGTGCAGCTGG
3999	sgDEGS2_9	DEGS2	chr14	100615461	+	GTGCAGGCCCAGGCCCAGGA
4000	sgDEGS2_10	DEGS2	chr14	100615364	-	CAACTGGATCACCTTCAATG
4001	sgDLG3_1	DLG3	chrX	69711986	+	ACAGCTGCCTGCCAAGCCAG
4002	sgDLG3_2	DLG3	chrX	69712112	+	GTGTGATCCCCAGTAAGAAG
4003	sgDLG3_3	DLG3	chrX	69719815	+	GGACAACAAGTTCATCGAGG
4004	sgDLG3_4	DLG3	chrX	69719737	+	GCCTCGACGTGATAATGAGG
4005	sgDLG3_5	DLG3	chrX	69719863	+	GACCAGCATCCAGTCAGTGC
4006	sgDLG3_6	DLG3	chrX	69720391	-	GGGCTTGATGAAAATGGCAA
4007	sgDLG3_7	DLG3	chrX	69720741	+	TTGGCAGGGAAATGAACCGA
4008	sgDLG3_8	DLG3	chrX	69699047	+	ATGAACAGCAGCATGAGCTC
4009	sgDLG3_9	DLG3	chrX	69719776	-	GTTCTCGGGACACCACAAAG
4010	sgDLG3_10	DLG3	chrX	69719878	+	AGTGCGGGCAGTTGCAGAGA
4011	sgDNAJC24_1	DNAJC24	chr11	31436470	+	AAAGAGAGTATGACCTGCAG
4012	sgDNAJC24_2	DNAJC24	chr11	31436381	+	AGATGTACCAGCAGGAACAG
4013	sgDNAJC24_3	DNAJC24	chr11	31436475	+	GAGTATGACCTGCAGCGGTG
4014	sgDNAJC24_4	DNAJC24	chr11	31392283	+	TCTGGTTCCATGGATGATGG
4015	sgDNAJC24_5	DNAJC24	chr11	31436373	+	CAAAGTACAGATGTACCAGC
4016	sgDNAJC24_6	DNAJC24	chr11	31392322	+	AAAGGATTGGTACAGCATCC
4017	sgDNAJC24_7	DNAJC24	chr11	31436391	-	CATTCCTCCACTGTTCCTGC
4018	sgDNAJC24_8	DNAJC24	chr11	31436486	-	AGCACCTACCACACCGCTGC
4019	sgDNAJC24_9	DNAJC24	chr11	31451832	+	TATCTGAGTTGCAGATGTGG
4020	sgDNAJC24_10	DNAJC24	chr11	31451861	+	CAGTGTTTCCAAGGATGAAG
4021	sgDPH3_1	DPH3	chr3	16306288	+	GAAGTTATCTCCACATGGGC
4022	sgDPH3_2	DPH3	chr3	16306363	+	CTCGTCATGAAACACTGCCA
4023	sgDPH3_3	DPH3	chr3	16306283	+	ATGGAGAAGTTATCTCCACA
4024	sgDPH3_4	DPH3	chr3	16306264	+	CCCTGAAGTTACCTTGGTGA

4025	sgDPH3_5	DPH3	chr3	16302287	+	TCTTTGTTGGCTGAAGGGGC
4026	sgDPH3_6	DPH3	chr3	16305712	-	TTTGGAGAATGGGGAAGACG
4027	sgDPH3_7	DPH3	chr3	16305721	-	GTAGGAAGATTTGGAGAATG
4028	sgDPH3_8	DPH3	chr3	16306335	-	CGAGGACTTCCAATATGACG
4029	sgDPH3_9	DPH3	chr3	16306278	-	AGATAACTTCTCCATCACCA
4030	sgDPH3_10	DPH3	chr3	16306353	-	TCATGACGAGGTGGAAATCG
4031	sgENAH_1	ENAH	chr1	225702536	+	GAAGCCTGTGCAGGCCCTGG
4032	sgENAH_2	ENAH	chr1	225702485	+	GATGGGAGTGGAGGAGGTGG
4033	sgENAH_3	ENAH	chr1	225742747	+	GTACTTCAACCCTTTAGGAA
4034	sgENAH_4	ENAH	chr1	225702434	+	GGTACTTGATTAGGGAGAGG
4035	sgENAH_5	ENAH	chr1	225700693	-	GATACCTCTTTCCCAAGTGG
4036	sgENAH_6	ENAH	chr1	225755042	-	AAGAAGTGGGTGCCAGCTGG
4037	sgENAH_7	ENAH	chr1	225707249	-	GACAACTACAAGAACAGCAA
4038	sgENAH_8	ENAH	chr1	225707180	-	GAGAAAGGTTGGAGAGAGAG
4039	sgENAH_9	ENAH	chr1	225707110	-	GGAGAGAGAGAGACAAGAAC
4040	sgENAH_10	ENAH	chr1	225706902	-	GAGCGCAGAATATCAAGTGC
4041	sgEVL_1	EVL	chr14	100551027	+	ACAGAGTATCTGCCAAGCCC
4042	sgEVL_2	EVL	chr14	100551047	-	GTAGACCATCACGGAAGCCC
4043	sgEVL_3	EVL	chr14	100594870	-	GCAGATGAAGGATGTCCTGG
4044	sgEVL_4	EVL	chr14	100594951	-	GGGGTAGCCCCAGTGGGTGG
4045	sgEVL_5	EVL	chr14	100595948	+	GATGCCAACCGGGCAAGCAG
4046	sgEVL_6	EVL	chr14	100595992	+	GGAGGAAATGAACAAACTGC
4047	sgEVL_7	EVL	chr14	100608093	+	GAGAGAGCTCCACAAGGTGA
4048	sgEVL_8	EVL	chr14	100608109	+	GTGAAGGAGGAGATCATCGA
4049	sgEVL_9	EVL	chr14	100594902	-	GAGGCCCACTACATGAGACG
4050	sgEVL_10	EVL	chr14	100604082	-	GGGGCTCTTTGCCACAGACG
4051	sgEXO5_1	EXO5	chr1	40980495	+	ACTGCAAACAGCATATGGGA
4052	sgEXO5_2	EXO5	chr1	40980920	-	GTGGTGGATTAGGCTAGCAG
4053	sgEXO5_3	EXO5	chr1	40980368	-	GGGTTTGTCATCCTTCCCAA
4054	sgEXO5_4	EXO5	chr1	40980762	+	TGAGCTGCACTATACAGCCA
4055	sgEXO5_5	EXO5	chr1	40980797	+	TGGCGGAACTCAAGACACGC
4056	sgEXO5_6	EXO5	chr1	40981118	-	AATCTCAGTACCCAGCACAG
4057	sgEXO5_7	EXO5	chr1	40981149	+	AGAGAAGGAGGTGAGAGCCA
4058	sgEXO5_8	EXO5	chr1	40981209	+	GCCCCAGGGAGTTGACGTGG
4059	sgEXO5_9	EXO5	chr1	40980715	+	CCAGTGTTTGGGGAAGGGGA
4060	sgEXO5_10	EXO5	chr1	40980947	+	TGTGTCTAGAAAAGCCACTG
4061	sgFABP7_1	FABP7	chr6	123101461	+	GGGAAATGTGACCAAACCAA
4062	sgFABP7_2	FABP7	chr6	123101586	-	ACAGTTTCTATCATCTGCAG
4063	sgFABP7_3	FABP7	chr6	123100927	+	GGAAAGGGCAAGGATGGTGG
4064	sgFABP7_4	FABP7	chr6	123102268	+	GTTCACATACAGAAATGGGA
4065	sgFABP7_5	FABP7	chr6	123101437	+	CGTGGGCTTTGCCACTAGGC
4066	sgFABP7_6	FABP7	chr6	123101542	+	CACGGAGATTAGTTTCCAGC
4067	sgFABP7_7	FABP7	chr6	123101451	-	CACATTTCCCACCTGCCTAG
4068	sgFABP7_8	FABP7	chr6	123101524	+	TCTCAGCACATTCAAGAACA
4069	sgFABP7_9	FABP7	chr6	123101560	-	CATCAAACTCTTCTCCCAGC
4070	sgFABP7_10	FABP7	chr6	123102252	-	GAACAAGTTTGTCTCCATCC
4071	sgFDXR_1	FDXR	chr17	72861843	-	GACCCCACCTGAGCACCTGG
4072	sgFDXR_2	FDXR	chr17	72862255	-	CGGGCTTCCTGAGAACCAGG
4073	sgFDXR_3	FDXR	chr17	72861038	-	CCAGAGAACGGACATCACGA
4074	sgFDXR_4	FDXR	chr17	72861008	+	GACTCTGCCTCAGTACACCC
4075	sgFDXR_5	FDXR	chr17	72860408	-	GAACGGCCACAGAGAAGCCA
4076	sgFDXR_6	FDXR	chr17	72860319	+	GGTGAGGGCAGCACCTGCTG
4077	sgFDXR_7	FDXR	chr17	72860120	+	TGAGCACCAGCCCACAAGGG
4078	sgFDXR_8	FDXR	chr17	72859229	+	GTAGCCAGGCCTGGGGCCAG
4079	sgFDXR_9	FDXR	chr17	72862914	-	TGTGGCGCCTGATCACCCCG
4080	sgFDXR_10	FDXR	chr17	72862277	+	GTACCAGCCCACGAAGGCCC
4081	sgFOLH1_1	FOLH1	chr11	49168368	+	CTGTCTCTTCACTTCTCCCC
4082	sgFOLH1_2	FOLH1	chr11	49214361	+	GGAGAGAAAGCACTGAAAGG
4083	sgFOLH1_3	FOLH1	chr11	49204779	-	TCAGAATATGCTTATAGGCG
4084	sgFOLH1_4	FOLH1	chr11	49221884	+	GTGCTAGCTCAACAGAATCC
4085	sgFOLH1_5	FOLH1	chr11	49207339	+	AGCAAAGTAGTCAGCAGGGT

4086	sgFOLH1_6	FOLH1	chr11	49207303	+	AAGATTCCAACCATCTGGAT
4087	sgFOLH1_7	FOLH1	chr11	49229838	+	CCCTACCGAAGAGGAAGCCG
4088	sgFOLH1_8	FOLH1	chr11	49229928	+	CAGCCGAGTCGGTTTCGTGA
4089	sgFOLH1_9	FOLH1	chr11	49229917	+	CGCGGTGGCCACAGCCGAGT
4090	sgFOLH1_10	FOLH1	chr11	49229858	-	GCGGGTGGCTTCTTTCTCCT
4091	sgHBA1_1	HBA1	chr16	226759	-	GTGCGCGCCGACCTTACCCC
4092	sgHBA1_2	HBA1	chr16	226775	+	GCACGCTGGCGAGTATGGTG
4093	sgHBA1_3	HBA1	chr16	226942	-	GTAGGTCTTGGTGGTGGGGA
4094	sgHBA1_4	HBA1	chr16	227000	+	TAAGGGCCACGGCAAGAAGG
4095	sgHBA1_5	HBA1	chr16	227024	+	CGACGCGCTGACCAACGCCG
4096	sgHBA1_6	HBA1	chr16	227093	+	GCACGCGCACAAGCTTCGGG
4097	sgHBA1_7	HBA1	chr16	226739	+	GACCAACGTCAAGGCCGCCT
4098	sgHBA1_8	HBA1	chr16	226749	+	AAGGCCGCCTGGGGTAAGGT
4099	sgHBA1_9	HBA1	chr16	227068	-	GCTCAGGGCGGACAGCGCGT
4100	sgHBA1_10	HBA1	chr16	227080	-	CGCGTGCAGGTCGCTCAGGG
4101	sgHBA2_1	HBA2	chr16	222955	-	GTGCGCGCCGACCTTACCCC
4102	sgHBA2_2	HBA2	chr16	222971	+	GCACGCTGGCGAGTATGGTG
4103	sgHBA2_3	HBA2	chr16	223138	-	GTAGGTCTTGGTGGTGGGGA
4104	sgHBA2_4	HBA2	chr16	223196	+	TAAGGGCCACGGCAAGAAGG
4105	sgHBA2_5	HBA2	chr16	223220	+	CGACGCGCTGACCAACGCCG
4106	sgHBA2_6	HBA2	chr16	223289	+	GCACGCGCACAAGCTTCGGG
4107	sgHBA2_7	HBA2	chr16	222935	+	GACCAACGTCAAGGCCGCCT
4108	sgHBA2_8	HBA2	chr16	222945	+	AAGGCCGCCTGGGGTAAGGT
4109	sgHBA2_9	HBA2	chr16	223264	-	GCTCAGGGCGGACAGCGCGT
4110	sgHBA2_10	HBA2	chr16	223276	-	CGCGTGCAGGTCGCTCAGGG
4111	sgHCCS_1	HCCS	chrX	11130243	-	GCATCGGGCATCCTGAAGGT
4112	sgHCCS_2	HCCS	chrX	11130182	-	AGCAGATGGAGACAAACCCA
4113	sgHCCS_3	HCCS	chrX	11135393	-	GCTGGTGTTTGATTTGGTGG
4114	sgHCCS_4	HCCS	chrX	11136618	+	TAGGTGGAAGTGGAAGGATG
4115	sgHCCS_5	HCCS	chrX	11130196	-	ACTGCAACAGCAGGAGCAGA
4116	sgHCCS_6	HCCS	chrX	11133022	-	ACTCATAGGCGCGTTCCTGG
4117	sgHCCS_7	HCCS	chrX	11133049	+	GTGTCCCATTAGGGGCACTG
4118	sgHCCS_8	HCCS	chrX	11136678	+	TCACAATCAGAATAACGAGC
4119	sgHCCS_9	HCCS	chrX	11136701	+	CTTGGAAGGAGATTTTGAAG
4120	sgHCCS_10	HCCS	chrX	11135411	-	GCAAATGGCTGATCTGGAGC
4121	sgHDAC1_1	HDAC1	chr1	32790091	+	GAGGACTGTCCAGTATTCGA
4122	sgHDAC1_2	HDAC1	chr1	32792601	+	GCACCATGCAAAGAAGTCCG
4123	sgHDAC1_3	HDAC1	chr1	32793127	+	CTGATCCTAGGTATCACCAG
4124	sgHDAC1_4	HDAC1	chr1	32796199	+	GATGTTCCAGCCTAGTGCGG
4125	sgHDAC1_5	HDAC1	chr1	32796413	+	ATGCTGATGCTGGGAGGCGG
4126	sgHDAC1_6	HDAC1	chr1	32796444	-	GCACCGGGCAACGTTACGAA
4127	sgHDAC1_7	HDAC1	chr1	32757764	+	GAGCAAGATGGCGCAGACGC
4128	sgHDAC1_8	HDAC1	chr1	32768280	-	GCAGCAAATTATGAGTCATG
4129	sgHDAC1_9	HDAC1	chr1	32792621	-	GTAACAGAAGCCAGATGCCT
4130	sgHDAC1_10	HDAC1	chr1	32793170	+	TATTCACCATGGTGACGGCG
4131	sgHDAC2_1	HDAC2	chr6	114265485	-	GATGCTGTTCATGAAGACAG
4132	sgHDAC2_2	HDAC2	chr6	114262896	-	GATAAATCCAAGGACAACAG
4133	sgHDAC2_3	HDAC2	chr6	114292052	+	GCCTCCTTGACTGTACGCCA
4134	sgHDAC2_4	HDAC2	chr6	114264607	-	GATTCTGAGGATGAAGGAGA
4135	sgHDAC2_5	HDAC2	chr6	114277264	-	GATATGGCTGTTAATTGGGC
4136	sgHDAC2_6	HDAC2	chr6	114267252	-	CCATTACTGATGCTTGGAGG
4137	sgHDAC2_7	HDAC2	chr6	114277815	+	TCAAAGAGTCCATCAAACAC
4138	sgHDAC2_8	HDAC2	chr6	114274454	-	GGGGAATACTTTCCTGGCAC
4139	sgHDAC2_9	HDAC2	chr6	114281105	+	ACAGCAAGTTATGGGTCATG
4140	sgHDAC2_10	HDAC2	chr6	114277827	-	GAAGATTGTCCAGTGTTTGA
4141	sgHEPH_1	HEPH	chrX	65417538	-	AGGGCTTGGCAACCAAGCCA
4142	sgHEPH_2	HEPH	chrX	65409645	+	GGACAGATGCTGACTACCCG
4143	sgHEPH_3	HEPH	chrX	65414959	+	GGTGGGTGACACCATTCAGG
4144	sgHEPH_4	HEPH	chrX	65417606	-	ACAAGCAGGATCCTGAGCAG
4145	sgHEPH_5	HEPH	chrX	65417692	-	CCAGCCCTGCACACCAGCAG
4146	sgHEPH_6	HEPH	chrX	65418707	+	ATTGGGCCTGCAGAAAGGGG

4147	sgHEPH_7	HEPH	chrX	65420422	+	CATGTGCAAGGGTGACACAG
4148	sgHEPH_8	HEPH	chrX	65427098	+	GTATTCAGGGAATACACTGA
4149	sgHEPH_9	HEPH	chrX	65428021	-	GAGCATGCACAGAGTAGGGG
4150	sgHEPH_10	HEPH	chrX	65479950	+	GAACTACCGGGCAGATGTGG
4151	sgHEPHL1_1	HEPHL1	chr11	93778954	-	GGGCCCAGGAATCCAAGCCA
4152	sgHEPHL1_2	HEPHL1	chr11	93800781	+	GGTAACACCTTCATCAGCAG
4153	sgHEPHL1_3	HEPHL1	chr11	93803531	+	GTTCTGCAGCTGGTATGCTG
4154	sgHEPHL1_4	HEPHL1	chr11	93822096	+	GGAGTTCGAAAAGCAGCACG
4155	sgHEPHL1_5	HEPHL1	chr11	93796809	-	GATGTGCGAATGGTACACCC
4156	sgHEPHL1_6	HEPHL1	chr11	93803702	-	AAAGGGTGCTTACCTGCCAG
4157	sgHEPHL1_7	HEPHL1	chr11	93806213	+	CAAGGGGACAACAGAATAGG
4158	sgHEPHL1_8	HEPHL1	chr11	93806472	+	AGGCCCAGTCATCAAGGCAG
4159	sgHEPHL1_9	HEPHL1	chr11	93808373	+	GTGAAACCTTCACATACAAG
4160	sgHEPHL1_10	HEPHL1	chr11	93819302	-	CATGTGGGGAAACAGGGCCA
4161	sgHFE_1	HFE	chr6	26093353	-	GACTAGGGTGCCAGACGGTG
4162	sgHFE_2	HFE	chr6	26087721	-	CAAGCGCCCCTGCAGGACCG
4163	sgHFE_3	HFE	chr6	26087690	-	AGCATCAGGAGGAGAAGCGC
4164	sgHFE_4	HFE	chr6	26087701	+	CCTGATGCTTTTGCAGACCG
4165	sgHFE_5	HFE	chr6	26093367	+	CTAGTCATTGGAGTCATCAG
4166	sgHFE_6	HFE	chr6	26087668	+	AATGGGCCCGCGAGCCAGGC
4167	sgHFE_7	HFE	chr6	26087678	-	AGAAGCGCCGGCCTGGCTCG
4168	sgHFE_8	HFE	chr6	26093340	+	GTTTTAGAGCCCTCACCGTC
4169	sgHFE_9	HFE	chr6	26094403	+	TGTCTCCACAGGAGGAGCCA
4170	sgHFE_10	HFE	chr6	26094423	-	AGCTAAGACGTAGTGCCCCA
4171	sgHIF1A_1	HIF1A	chr14	62200891	+	AGGATGCTTGCCAAAAGAGG
4172	sgHIF1A_2	HIF1A	chr14	62205044	+	CAGACACCTAGTCCTTCCGA
4173	sgHIF1A_3	HIF1A	chr14	62207543	-	TAATGGTGACAACTGATCGA
4174	sgHIF1A_4	HIF1A	chr14	62187200	-	GAACTCACATTATGTGGAAG
4175	sgHIF1A_5	HIF1A	chr14	62193480	+	AAGTGTACCCTAACTAGCCG
4176	sgHIF1A_6	HIF1A	chr14	62194225	-	GGTTTCTTATACCCACACTG
4177	sgHIF1A_7	HIF1A	chr14	62204919	-	TGGCTTTGGCGTTTCAGCGG
4178	sgHIF1A_8	HIF1A	chr14	62204939	-	GGGTCAGCACTACTTCGAAG
4179	sgHIF1A_9	HIF1A	chr14	62187237	-	GATGGTAAGCCTCATCACAG
4180	sgHIF1A_10	HIF1A	chr14	62194211	+	ACAGTAACCAACCTCAGTGT
4181	sgHSCB_1	HSCB	chr22	29138094	+	GGAGAGCCGGGGCTTTGCTC
4182	sgHSCB_2	HSCB	chr22	29138203	-	GGCCGCCGCAGTTCCAACAG
4183	sgHSCB_3	HSCB	chr22	29139906	-	GCAGTTGCTGGTACCTGTGC
4184	sgHSCB_4	HSCB	chr22	29138070	+	AGGCCGCCGGCCAGATGTGG
4185	sgHSCB_5	HSCB	chr22	29139939	-	TCTGGCTGAAGAAATCTGGG
4186	sgHSCB_6	HSCB	chr22	29138187	+	GAAGCAATTATCCCCGCTGT
4187	sgHSCB_7	HSCB	chr22	29139890	+	ATACAGCGAAGCTCCAGCAC
4188	sgHSCB_8	HSCB	chr22	29141883	+	GGACAGATTATGAAATGGAC
4189	sgHSCB_9	HSCB	chr22	29138291	-	AGGCTGAAGTAGTCTCGAGT
4190	sgHSCB_10	HSCB	chr22	29139876	-	CTGTATCAACTCTGAAGGAA
4191	sgHSPA6_1	HSPA6	chr1	161494715	+	GAAGCACTGGCCCTTCCGGG
4192	sgHSPA6_2	HSPA6	chr1	161496319	+	GTGGCACTCAAGCCCGCCAG
4193	sgHSPA6_3	HSPA6	chr1	161494945	-	CAACACGTTGAGCCCCGCGA
4194	sgHSPA6_4	HSPA6	chr1	161494964	+	GCGGATCATCAATGAGCCCA
4195	sgHSPA6_5	HSPA6	chr1	161496023	+	GCAGTACAAGGCTGAGGATG
4196	sgHSPA6_6	HSPA6	chr1	161494729	-	GCCCTCGCTCACCACCCGGA
4197	sgHSPA6_7	HSPA6	chr1	161494983	-	TAGGCGATGGCAGCTGCCGT
4198	sgHSPA6_8	HSPA6	chr1	161496157	+	GCAAAATGCAAGACAAGTGT
4199	sgHSPA6_9	HSPA6	chr1	161494891	-	GTCATTGAAATAGGCGGGCA
4200	sgHSPA6_10	HSPA6	chr1	161495841	+	GGCATCCCTCCTGCCCCACG
4201	sgHTT_1	HTT	chr4	3225755	+	GGAGCAAGAGATTCAAGCAA
4202	sgHTT_2	HTT	chr4	3237399	-	CCAGCGATTCTGCATCCAGG
4203	sgHTT_3	HTT	chr4	3076691	-	TGAGGAAGCTGAGGAGGCGG
4204	sgHTT_4	HTT	chr4	3107095	-	GGGCAGCACGCAAACTCCGA
4205	sgHTT_5	HTT	chr4	3129066	+	ACATCATCACAGAACAGCCA
4206	sgHTT_6	HTT	chr4	3131731	+	TCTTCCTGATGAAGCCTCGG
4207	sgHTT_7	HTT	chr4	3133414	-	CAGCTGCTCCCACACAGCTG

4208	sgHTT_8	HTT	chr4	3134339	-	GCAGTGGCTCCTCGAACCTG
4209	sgHTT_9	HTT	chr4	3134355	-	GGTCCCACAGAGAATGGCAG
4210	sgHTT_10	HTT	chr4	3134392	+	CAGCAGGTCCCGCTTCCACG
4211	sgIBA57_1	IBA57	chr1	228362483	+	GATCCGGCGGAAGGTCACGG
4212	sgIBA57_2	IBA57	chr1	228362390	+	CAGGCTCCAGGAACACTCGG
4213	sgIBA57_3	IBA57	chr1	228362563	+	GGGCTGCATCGCTGCAGGAG
4214	sgIBA57_4	IBA57	chr1	228362613	+	GACCCGCGAACAGCACGCAT
4215	sgIBA57_5	IBA57	chr1	228353723	-	GGTCAGCAGCCCTAGCAGGA
4216	sgIBA57_6	IBA57	chr1	228353748	+	ACTGCCGCTTCCGAGTCCTG
4217	sgIBA57_7	IBA57	chr1	228362429	+	GGAGTGTGACAGCTCGGTGC
4218	sgIBA57_8	IBA57	chr1	228362599	-	CGGGTCGCGGATGAGGATGG
4219	sgIBA57_9	IBA57	chr1	228353660	-	GTCCAGCCGGAAGCAGGCCC
4220	sgIBA57_10	IBA57	chr1	228353711	-	TAGCAGGAAGGGCGCCGCGT
4221	sgKAT2B_1	KAT2B	chr3	20161104	-	GAAATTGTCCCAGCCACAGG
4222	sgKAT2B_2	KAT2B	chr3	20167436	+	GCAAGGTTGGAAGAGCGCAG
4223	sgKAT2B_3	KAT2B	chr3	20189777	+	GAGCATCCTCCAGCAGGTGA
4224	sgKAT2B_4	KAT2B	chr3	20082131	+	GGCGACGGCAGTGGCTGCAG
4225	sgKAT2B_5	KAT2B	chr3	20082265	-	GCGAGCGCGTACCTTGCAGG
4226	sgKAT2B_6	KAT2B	chr3	20167405	+	TCTGTCAGCACACTCGGCCA
4227	sgKAT2B_7	KAT2B	chr3	20167537	-	GGTGGGAGAAAACGTTCTGT
4228	sgKAT2B_8	KAT2B	chr3	20189457	+	GAGACAGGCTGGAAACCGAG
4229	sgKAT2B_9	KAT2B	chr3	20082209	+	GCAACTACGCTCCGCTCCGC
4230	sgKAT2B_10	KAT2B	chr3	20113944	-	TTAGGAACTCACCTAGGGCA
4231	sgKAT5_1	KAT5	chr11	65479866	+	GCTGCCGCCTACCCGTGCTG
4232	sgKAT5_2	KAT5	chr11	65479876	-	GGTTCCGCCGCAGCACGGGT
4233	sgKAT5_3	KAT5	chr11	65481060	+	GCAGAAACGGAAGGTGGAGG
4234	sgKAT5_4	KAT5	chr11	65479729	+	GGGAGGGAAGATGGCGGAGG
4235	sgKAT5_5	KAT5	chr11	65480508	+	TGGCTCTCCAGAGAGAGAGG
4236	sgKAT5_6	KAT5	chr11	65481100	-	GGGGCTGTCTCGCTGGGCAC
4237	sgKAT5_7	KAT5	chr11	65481298	+	GAATTGTTTGGGCACTGATG
4238	sgKAT5_8	KAT5	chr11	65482154	-	GGCAGAACTCGCACAGGTAG
4239	sgKAT5_9	KAT5	chr11	65486155	+	GACCATCCTGGAGATCCTGA
4240	sgKAT5_10	KAT5	chr11	65480497	-	GGAGAGCCAGGACGGGACCC
4241	sgKIF4A_1	KIF4A	chrX	69510323	+	GAAGGGAATTCCCGTAAGAG
4242	sgKIF4A_2	KIF4A	chrX	69595069	-	GCTCCTGGAGACGTTTGCGG
4243	sgKIF4A_3	KIF4A	chrX	69606527	+	TGTGCTCAGACGTAAAACGG
4244	sgKIF4A_4	KIF4A	chrX	69607059	+	AGGATGCTCTCCAGAAACAA
4245	sgKIF4A_5	KIF4A	chrX	69607097	+	CGGAAAGAGACTCAGAGCCG
4246	sgKIF4A_6	KIF4A	chrX	69615601	+	AAAGATCCTGGCTCAAGATG
4247	sgKIF4A_7	KIF4A	chrX	69622466	+	AAGACAGACCAAAACAACGC
4248	sgKIF4A_8	KIF4A	chrX	69521841	-	CGACTGGGAGTTCATAGCCG
4249	sgKIF4A_9	KIF4A	chrX	69595996	+	ATGCTGAGAAGTTTAGACAG
4250	sgKIF4A_10	KIF4A	chrX	69626904	+	GTCCAGGAAGAACATCCAAG
4251	sgKIF4B_1	KIF4B	chr5	154393524	+	GCCCGGGGAGACTCAGGTGG
4252	sgKIF4B_2	KIF4B	chr5	154393710	+	AATGGGAGGTGCATACACTG
4253	sgKIF4B_3	KIF4B	chr5	154393434	+	GAAGGGAATTCCTGTAAGAG
4254	sgKIF4B_4	KIF4B	chr5	154393571	-	GGGGTCAAACACAAAATCGT
4255	sgKIF4B_5	KIF4B	chr5	154395213	-	GCTCCTGGAGAAGTTTGTGG
4256	sgKIF4B_6	KIF4B	chr5	154395601	+	CGGAAAGAGACTCAGAGCCA
4257	sgKIF4B_7	KIF4B	chr5	154395732	+	AAAGATCCTGGCTCAGGATG
4258	sgKIF4B_8	KIF4B	chr5	154396598	+	GTGATGGCGACAGTGATGAG
4259	sgKIF4B_9	KIF4B	chr5	154396676	+	AAGGGTGTTCCTGCAAGGGC
4260	sgKIF4B_10	KIF4B	chr5	154396929	+	AGAGATGTGTGACATGGAGC
4261	sgLDHD_1	LDHD	chr16	75149534	-	GCTCTGCAGGGACTTCGTAG
4262	sgLDHD_2	LDHD	chr16	75149516	-	AGAGGCTCTGAAGGCCGTGG
4263	sgLDHD_3	LDHD	chr16	75149469	+	GTGCTGCTCTCGGACCACCG
4264	sgLDHD_4	LDHD	chr16	75149208	+	GGCCACACCACAGCATCAGG
4265	sgLDHD_5	LDHD	chr16	75148507	+	GGTGCCGTAGCGGACCGCGT
4266	sgLDHD_6	LDHD	chr16	75148482	-	GGACAACGTGCTCAACCTGG
4267	sgLDHD_7	LDHD	chr16	75147928	+	ACTGGGGAACGCACACGTGG
4268	sgLDHD_8	LDHD	chr16	75146793	+	GATGGGCACACACACATCCG

4269	sgLDHD_9	LDHD	chr16	75146762	-	GGAGATCGTGGTGCAGACCA
4270	sgLDHD_10	LDHD	chr16	75146543	-	GGTCAACCCTGATGACGCCG
4271	sgLRP1_1	LRP1	chr12	57522783	+	GCTCTCAGCTCTGGTCGCGG
4272	sgLRP1_2	LRP1	chr12	57569847	+	GTGGAGGACAAGATCTACCG
4273	sgLRP1_3	LRP1	chr12	57572301	+	GACCGGCCACAATGTCACCG
4274	sgLRP1_4	LRP1	chr12	57578681	+	GACAGGTGAGAACCGCGAGG
4275	sgLRP1_5	LRP1	chr12	57590897	+	GAGGGCTATGCACCCCGCGG
4276	sgLRP1_6	LRP1	chr12	57605558	-	AAGACGTGCTCCTCACACCG
4277	sgLRP1_7	LRP1	chr12	57522758	-	CAGCAGCAGGAGCAACGGCG
4278	sgLRP1_8	LRP1	chr12	57535273	+	GATGAGGGGCCCCACTGCCG
4279	sgLRP1_9	LRP1	chr12	57537528	-	GCTGCTGTTGCAGTAGCAGG
4280	sgLRP1_10	LRP1	chr12	57537560	+	GCAGATGGCAAGACCTGCAA
4281	sgLRP2_1	LRP2	chr2	170135898	-	GCATCCTAGAGGAATTGCCG
4282	sgLRP2_2	LRP2	chr2	170048474	+	GGAATGCACCTCCTGTCCGG
4283	sgLRP2_3	LRP2	chr2	170177289	-	GACGCGGATGAAATTGGCTG
4284	sgLRP2_4	LRP2	chr2	170148864	-	CCAGACTATCCGACCTGCGG
4285	sgLRP2_5	LRP2	chr2	170148848	+	AGTGAACTGGTAACCACCGC
4286	sgLRP2_6	LRP2	chr2	170147399	-	GATGGGATTTTAGATTGCCC
4287	sgLRP2_7	LRP2	chr2	170145558	+	GTGGTTGATGATATAACCTG
4288	sgLRP2_8	LRP2	chr2	170136928	-	GGAGTCTCAGAATCGTGGAG
4289	sgLRP2_9	LRP2	chr2	170136902	+	ATAGTGGAAAGCCACACCCA
4290	sgLRP2_10	LRP2	chr2	170131731	-	AGGAAGACTGTAGTTCATGG
4291	sgLYRM4_1	LYRM4	chr6	5216870	-	AATAAAGCCAAGAGAGACCT
4292	sgLYRM4_2	LYRM4	chr6	5216956	-	TTAGAACATATGCTGTCAGG
4293	sgLYRM4_3	LYRM4	chr6	5216850	+	CCTGTCGACGAATTACTCCA
4294	sgLYRM4_4	LYRM4	chr6	5260937	+	TAACACTTGTGCGCGACTGG
4295	sgLYRM4_5	LYRM4	chr6	5216860	+	AATTACTCCAAGGTCTCTCT
4296	sgLYRM4_6	LYRM4	chr6	5260871	+	GCTGGGTCACCTGTAATTGT
4297	sgLYRM4_7	LYRM4	chr6	5144455	+	GTTCCCCGACTTCCTCCTGG
4298	sgLYRM4_8	LYRM4	chr6	5144470	-	AGCAAGGCACAGCCGCCAGG
4299	sgLYRM4_9	LYRM4	chr6	5144440	-	GGAACAGCAGCCGGAGCCTG
4300	sgLYRM4_10	LYRM4	chr6	5109666	-	TTGAGAATCGAGACATGCCC
4301	sgLYRM7_1	LYRM7	chr5	130506701	+	GGTGAGGAGAGCCATGGGAC
4302	sgLYRM7_2	LYRM7	chr5	130515795	+	AGCTCTTTAAAACACTGCAC
4303	sgLYRM7_3	LYRM7	chr5	130515839	+	AATGATGCCAGAGCATTAGA
4304	sgLYRM7_4	LYRM7	chr5	130506710	+	AGCCATGGGACGGGCAGTCA
4305	sgLYRM7_5	LYRM7	chr5	130506715	-	CACCTTGACTGCCCGTCCCA
4306	sgLYRM7_6	LYRM7	chr5	130535234	-	CTACAAGAAGGTCTTTCCTA
4307	sgLYRM7_7	LYRM7	chr5	130535265	-	TGAGTTGGTGCATCACAATA
4308	sgLYRM7_8	LYRM7	chr5	130535215	+	TTCTTTCAGAACTGGTCCCT
4309	sgLYZ_1	LYZ	chr12	69743998	+	TGTAATGATGGCAAAACCCC
4310	sgLYZ_2	LYZ	chr12	69742221	-	CCTGGACCGTAACAGAAAGG
4311	sgLYZ_3	LYZ	chr12	69742239	-	ACCTTTCAAAGACCTTGCCC
4312	sgLYZ_4	LYZ	chr12	69743893	+	TGTTTGGCCAAATGGGAGAG
4313	sgLYZ_5	LYZ	chr12	69743926	+	CGAGCTACAAACTACAATGC
4314	sgLYZ_6	LYZ	chr12	69744017	-	ACAGGCATTAACTGCTCCTG
4315	sgLYZ_7	LYZ	chr12	69746044	+	AGGGTTGTCCGTGATCCACA
4316	sgLYZ_8	LYZ	chr12	69742266	+	GGCCAGAACTCTGAAAAGAT
4317	sgLYZ_9	LYZ	chr12	69742285	+	TTGGGAATGGATGGCTACAG
4318	sgLYZ_10	LYZ	chr12	69743947	+	GGAGACAGAAGCACTGATTA
4319	sgMARK3_1	MARK3	chr14	103941420	-	ACTGCCACTTGATTTCCGGG
4320	sgMARK3_2	MARK3	chr14	103958173	-	GAAGCGGATTCGATCTGGGG
4321	sgMARK3_3	MARK3	chr14	103958268	-	GACAGGCTGGGAGAGGCAGG
4322	sgMARK3_4	MARK3	chr14	103871410	+	CAGCACACGTCACATGGAGA
4323	sgMARK3_5	MARK3	chr14	103871532	+	ACTGTTGAAAACAATCGGCA
4324	sgMARK3_6	MARK3	chr14	103932380	-	GCACCAGGAAACGTTTGAGA
4325	sgMARK3_7	MARK3	chr14	103958235	-	GTTGCGGTTCGCCGTTCCCG
4326	sgMARK3_8	MARK3	chr14	103871500	+	GCAGATGAACAACCTCACAT
4327	sgMARK3_9	MARK3	chr14	103918299	+	ATCATGGAATATGCAAGTGG
4328	sgMARK3_10	MARK3	chr14	103933507	+	ATTGGGGAGAAAATCTTCAG
4329	sgMCOLN1_1	MCOLN1	chr19	7591383	-	GAAGAGGTGTCGGAAGGCGA

4330	sgMCOLN1_2	MCOLN1	chr19	7590019	+	GCAAGTGGTCAAGATCCTGG
4331	sgMCOLN1_3	MCOLN1	chr19	7593550	-	GAAGGAGTGAGCGGGCGCAG
4332	sgMCOLN1_4	MCOLN1	chr19	7593835	+	GAAGATCGGCATCGAGGCCA
4333	sgMCOLN1_5	MCOLN1	chr19	7594019	-	GCAGCGTCGAGGTGCCCAGG
4334	sgMCOLN1_6	MCOLN1	chr19	7587630	+	CCCCAGCATGACAGCCCCGG
4335	sgMCOLN1_7	MCOLN1	chr19	7589875	-	CCGCCTGGGTCCCATACCCG
4336	sgMCOLN1_8	MCOLN1	chr19	7589938	-	AGTATTTGAGACGACGGCGA
4337	sgMCOLN1_9	MCOLN1	chr19	7592449	-	AGAGGGTGAGATCGTCGCTG
4338	sgMCOLN1_10	MCOLN1	chr19	7593531	-	GAGGAGGAAGGACAGGGAGC
4339	sgMDM4_1	MDM4	chr1	204494666	-	AGATGTTGAACACTGAGCAG
4340	sgMDM4_2	MDM4	chr1	204494689	+	AGTGCTTGCAGGATCTCTCC
4341	sgMDM4_3	MDM4	chr1	204518655	+	TGTGAGAAAAGACCACGAGA
4342	sgMDM4_4	MDM4	chr1	204518729	+	CAGAAGACTAAAGAAGGCTG
4343	sgMDM4_5	MDM4	chr1	204518547	+	CAAGAGACCATCTCAAGCAT
4344	sgMDM4_6	MDM4	chr1	204518445	-	GCATCTTTAGGTCTAACGAC
4345	sgMDM4_7	MDM4	chr1	204518492	+	TTTTGATCCCTGCAACTCAG
4346	sgMDM4_8	MDM4	chr1	204518502	-	AAGAATTCCACTGAGTTGCA
4347	sgMDM4_9	MDM4	chr1	204518557	-	CTGTTCTCCCATGCTTGAGA
4348	sgMDM4_10	MDM4	chr1	204518670	+	CGAGACGGGAACATTATTCA
4349	sgMETAP2_1	METAP2	chr12	95867984	-	GCCATTCAGGTGGCTCCCGG
4350	sgMETAP2_2	METAP2	chr12	95905738	+	GCCGATTGTGAAAGGAGGGG
4351	sgMETAP2_3	METAP2	chr12	95905753	+	AGGGGAGGCAACAAGAATGG
4352	sgMETAP2_4	METAP2	chr12	95867994	-	GATCCAGGTCGCCATTCAGG
4353	sgMETAP2_5	METAP2	chr12	95888772	+	GAATGGATTAAATGCAGGCC
4354	sgMETAP2_6	METAP2	chr12	95867958	+	GGCGGGTGTGGAGGAGGTAG
4355	sgMETAP2_7	METAP2	chr12	95868073	+	CGAAAGAAGAAGAAGAGCAA
4356	sgMETAP2_8	METAP2	chr12	95869902	+	ACAGTTGGAAAGATCAGCAT
4357	sgMETAP2_9	METAP2	chr12	95869866	+	TAAAGAATCAGGAGCCTCAG
4358	sgMETAP2_10	METAP2	chr12	95879679	-	CAGGTCACATATTGGAACTG
4359	sgMIPEP_1	MIPEP	chr13	24463449	-	GGTGCTAGAATGCTGTGCGT
4360	sgMIPEP_2	MIPEP	chr13	24334314	+	TAGCACCATACCCCACGAGG
4361	sgMIPEP_3	MIPEP	chr13	24330716	-	AGGGAGATGCTGGCCCACGG
4362	sgMIPEP_4	MIPEP	chr13	24330699	+	TGGGCTCCCTGCCTCCACCG
4363	sgMIPEP_5	MIPEP	chr13	24463333	-	CAGCACCAGCTGGTCTCCCG
4364	sgMIPEP_6	MIPEP	chr13	24460502	+	GAGCTCATCGAAGATCAGCA
4365	sgMIPEP_7	MIPEP	chr13	24455878	-	GCCAGCATTCAGAGAAGCTG
4366	sgMIPEP_8	MIPEP	chr13	24444322	-	CATACAGCGTAAAAGAGCAG
4367	sgMIPEP_9	MIPEP	chr13	24443456	+	GAGAGCCCTGTGAGAAAACG
4368	sgMIPEP_10	MIPEP	chr13	24334329	-	GCTGCGATTCAGCCACCTCG
4369	sgMMS19_1	MMS19	chr10	99221281	+	AAAGCACTTGGCAGCAGCGG
4370	sgMMS19_2	MMS19	chr10	99236614	+	GAGGTCATGGACGATGCGGA
4371	sgMMS19_3	MMS19	chr10	99230524	+	AGACCCACCTCAGCAAATCG
4372	sgMMS19_4	MMS19	chr10	99229444	-	GTATGGACAGAAGGAACTGA
4373	sgMMS19_5	MMS19	chr10	99229414	+	TAGAAGCCCAAAGGCTGGGG
4374	sgMMS19_6	MMS19	chr10	99222363	+	AGTGCCAATGACAGACACCA
4375	sgMMS19_7	MMS19	chr10	99220720	-	GCTAGCTGTGGACAAAGTGG
4376	sgMMS19_8	MMS19	chr10	99220677	+	GTGAAGGCCTGACTACGACA
4377	sgMMS19_9	MMS19	chr10	99219202	+	GAAGAGGCTGAAGGCAGCTG
4378	sgMMS19_10	MMS19	chr10	99218968	+	TGAGGTACTTACCACAGGGG
4379	sgMYLIP_1	MYLIP	chr6	16129577	+	GGACGCGGTGCTGATGGAGG
4380	sgMYLIP_2	MYLIP	chr6	16141901	-	GCTCTGGGGAACACAAGAGG
4381	sgMYLIP_3	MYLIP	chr6	16144026	+	TAAAATGATCAGCACCAGGG
4382	sgMYLIP_4	MYLIP	chr6	16129599	+	GAGGTGGAGGCGAAAGCCAA
4383	sgMYLIP_5	MYLIP	chr6	16130882	+	GGATCTCCCAGCAGATGGAT
4384	sgMYLIP_6	MYLIP	chr6	16142032	-	ACAGCCTCACCTGTTCAAGG
4385	sgMYLIP_7	MYLIP	chr6	16143354	+	CATTCTGTGCGGGATAGCGA
4386	sgMYLIP_8	MYLIP	chr6	16145263	+	GGTGTATGACCATGCCAGGA
4387	sgMYLIP_9	MYLIP	chr6	16145372	+	ATGAACTGCAGCAGCTGCGA
4388	sgMYLIP_10	MYLIP	chr6	16145425	+	GGAGAAGCTACGCAAGCTGA
4389	sgNDFIP1_1	NDFIP1	chr5	141511427	-	GAAATGCTGCTGTAAGGTGG
4390	sgNDFIP1_2	NDFIP1	chr5	141511394	+	GGAGAACCTGAACAGGCTGC

4391	sgNDFIP1_3	NDFIP1	chr5	141517377	-	AGAGAGACCAAATCCTGAAA
4392	sgNDFIP1_4	NDFIP1	chr5	141511439	+	AGCATTTCTGCAGAGAGCGC
4393	sgNDFIP1_5	NDFIP1	chr5	141520156	+	ATGGTCAGTACTGGCTCTGG
4394	sgNDFIP1_6	NDFIP1	chr5	141511873	-	AGGGATAGTAGCTTCAGCCT
4395	sgNDFIP1_7	NDFIP1	chr5	141517344	+	TGACCACTTCAGCTGCAGGA
4396	sgNDFIP1_8	NDFIP1	chr5	141488527	+	TGCCGGCTGCGCCATGGCGT
4397	sgNDFIP1_9	NDFIP1	chr5	141488545	+	GTTGGCGTTGGCGGCGCTGG
4398	sgNDFIP1_10	NDFIP1	chr5	141511841	+	ACTGCCCAGTTATGATGAAG
4399	sgNDOR1_1	NDOR1	chr9	140109927	-	AGGAGGAGCAGAGGCCCCGG
4400	sgNDOR1_2	NDOR1	chr9	140100242	-	CCGAAGAGCACCAGAAGCTG
4401	sgNDOR1_3	NDOR1	chr9	140100343	+	GGCCCTGGACTCCTACCCGG
4402	sgNDOR1_4	NDOR1	chr9	140100705	-	GCACAAACAAATATCACCAG
4403	sgNDOR1_5	NDOR1	chr9	140107021	-	GGAGTCCCCGAGGCCCAGGA
4404	sgNDOR1_6	NDOR1	chr9	140108309	+	GCGATGACCAGCATGAGCTG
4405	sgNDOR1_7	NDOR1	chr9	140108700	+	GCACGGGCTCTGAGGGGCAG
4406	sgNDOR1_8	NDOR1	chr9	140108751	-	GAAGGGCTTCGACTCTGACG
4407	sgNDOR1_9	NDOR1	chr9	140109449	+	GCCCCGCAGGACCATCCTGG
4408	sgNDOR1_10	NDOR1	chr9	140100357	-	GCGAGCCCTCACCACCGGGT
4409	sgNEK6_1	NEK6	chr9	127064287	+	ACAACCTCTGCCACACCCTG
4410	sgNEK6_2	NEK6	chr9	127074850	+	AGGCCGAGGACAGTTCAGCG
4411	sgNEK6_3	NEK6	chr9	127064300	-	GATGCACAGGCCCCAGGGTG
4412	sgNEK6_4	NEK6	chr9	127074798	-	GCAGCGAAAAGACAGCGTGT
4413	sgNEK6_5	NEK6	chr9	127074907	+	AGTGGCTCTGAAGAAGGTGC
4414	sgNEK6_6	NEK6	chr9	127083806	+	GCTGGAGTTGGCTGACGCAG
4415	sgNEK6_7	NEK6	chr9	127088662	+	TGTGCAGCTGTGCAGCGCCG
4416	sgNEK6_8	NEK6	chr9	127110075	-	GAGTAGTGCTCCCCGGGGAG
4417	sgNEK6_9	NEK6	chr9	127074836	+	ATCGAAAAGAAGATAGGCCG
4418	sgNEK6_10	NEK6	chr9	127076225	+	GGCGAGGCAGGACTGTGTCA
4419	sgNIPA1_1	NIPA1	chr15	23052653	+	GGAGTGGATAATCAGCACGA
4420	sgNIPA1_2	NIPA1	chr15	23060867	-	AAACTTCCTGGCTTACACGG
4421	sgNIPA1_3	NIPA1	chr15	23060821	+	GGTACTCCAAGGGCGCCCAG
4422	sgNIPA1_4	NIPA1	chr15	23052616	-	GACAACTCAGGCTGAGCTGG
4423	sgNIPA1_5	NIPA1	chr15	23052681	-	GGGTGCCTGCTAAGCTGTGC
4424	sgNIPA1_6	NIPA1	chr15	23060811	+	TCACCCGAACGGTACTCCAA
4425	sgNIPA1_7	NIPA1	chr15	23052701	-	TCAACATCTTGGGCAAGTTG
4426	sgNIPA1_8	NIPA1	chr15	23052597	-	GAGGAAAAGCTGACCAATCC
4427	sgNIPA1_9	NIPA1	chr15	23052712	-	GAAGGAAAAGCTCAACATCT
4428	sgNIPA1_10	NIPA1	chr15	23060890	-	ACAGTGGCTGTTGGCCAGAT
4429	sgNIPAL1_1	NIPAL1	chr4	48035075	-	AGAACACTCAAAGCACCCAG
4430	sgNIPAL1_2	NIPAL1	chr4	48018881	-	TCTCGGCAGGGCTCTCCGGG
4431	sgNIPAL1_3	NIPAL1	chr4	48027118	-	GATCTCACACCAAGCCTGGG
4432	sgNIPAL1_4	NIPAL1	chr4	48032146	+	GACATTCTTACCTGAAGGAA
4433	sgNIPAL1_5	NIPAL1	chr4	48027101	+	GGTCTGTCCAAACTCCTCCC
4434	sgNIPAL1_6	NIPAL1	chr4	48036991	+	TATCCATGCCCCACAAGAAG
4435	sgNIPAL1_7	NIPAL1	chr4	48037004	-	GATGTGACTTCCTCTTCTTG
4436	sgNIPAL1_8	NIPAL1	chr4	48035061	-	ACCCAGAGGGGTGACCAAGG
4437	sgNIPAL1_9	NIPAL1	chr4	48018893	-	AGACACAAACCTTCTCGGCA
4438	sgNIPAL1_10	NIPAL1	chr4	48027305	+	AAAGAAGGGCCTCTTGCAAC
4439	sgNOX3_1	NOX3	chr6	155776203	-	GTTCTACTGGTATGAAGAGG
4440	sgNOX3_2	NOX3	chr6	155774602	-	GCTGCAGAGGACCGTGGAGG
4441	sgNOX3_3	NOX3	chr6	155764395	+	GCAGGAACTCACTGTGGGGA
4442	sgNOX3_4	NOX3	chr6	155750151	+	GTTCCAGGACTCCAGAGGGG
4443	sgNOX3_5	NOX3	chr6	155743876	+	AGATTTCAGAAGAGCAGCGA
4444	sgNOX3_6	NOX3	chr6	155776910	-	GGGGTGCTGGATTTTGAATG
4445	sgNOX3_7	NOX3	chr6	155764493	-	CCACTGGAGCCAGTCCGAGG
4446	sgNOX3_8	NOX3	chr6	155761143	+	GTACCAGAACAACTCATAGG
4447	sgNOX3_9	NOX3	chr6	155761094	-	CAGCCTGGCCATCCATGGGA
4448	sgNOX3_10	NOX3	chr6	155750116	-	GTGGCTTTAAAATGGCGCCA
4449	sgPARN_1	PARN	chr16	14693799	-	GGAGGAGCTGAATGATGCTG
4450	sgPARN_2	PARN	chr16	14576522	+	AGGGTGTAGGGTATGCACTG
4451	sgPARN_3	PARN	chr16	14540827	+	GGTCTGCTCAAGCTCAGTGT

4452	sgPARN_4	PARN	chr16	14704532	-	ATGTCCTGTCACGATTCCTG
4453	sgPARN_5	PARN	chr16	14647932	+	GAAAAGCTGGTAAAGGTCGC
4454	sgPARN_6	PARN	chr16	14576568	-	GACTGAAGATAGCTGGAAGG
4455	sgPARN_7	PARN	chr16	14540870	-	GAGGAAGCTGGCCTGGAGGA
4456	sgPARN_8	PARN	chr16	14711481	+	GCTTGCTAGAAAGTCAATGC
4457	sgPARN_9	PARN	chr16	14698005	-	GCAGAAACATGCCAAAGAAC
4458	sgPARN_10	PARN	chr16	14687147	+	AGGTCACTTACCGCAGGCAG
4459	sgPDE4D_1	PDE4D	chr5	58286645	+	GCATGATAACAGTCAAGGGC
4460	sgPDE4D_2	PDE4D	chr5	58271579	-	GTGGACGGACCGGATAATGG
4461	sgPDE4D_3	PDE4D	chr5	58287646	+	GAAATCATCATACCTTGGCA
4462	sgPDE4D_4	PDE4D	chr5	58271563	-	ATGGAGGAGTTCTTCCGCCA
4463	sgPDE4D_5	PDE4D	chr5	58271542	-	GGAGACCGAGAGAGGGAACG
4464	sgPDE4D_6	PDE4D	chr5	58287753	-	AGACCAATGTCTCAGATCAG
4465	sgPDE4D_7	PDE4D	chr5	58273104	+	TAAAGCCCACAGCCAAATGA
4466	sgPDE4D_8	PDE4D	chr5	58285647	+	TGTGATAGGCCACATCAGCA
4467	sgPDE4D_9	PDE4D	chr5	58273120	+	ATGATGGTTCTCTAAGACTG
4468	sgPDE4D_10	PDE4D	chr5	58272217	-	ACTAAGAAAGTGACAAGCTC
4469	sgPRC1_1	PRC1	chr15	91527970	-	GGAGCTAATTGGGATTCCAG
4470	sgPRC1_2	PRC1	chr15	91524158	-	TGAAGAAGAAAGAGAAGCTG
4471	sgPRC1_3	PRC1	chr15	91519964	-	AATCGATTTACAAACCGAGG
4472	sgPRC1_4	PRC1	chr15	91527982	+	TTAGCTCCCATATTTCCCGA
4473	sgPRC1_5	PRC1	chr15	91527946	-	CCAGCGGTTACAAAGAACTG
4474	sgPRC1_6	PRC1	chr15	91524803	-	CACAAGCTTTGAAAGAGATG
4475	sgPRC1_7	PRC1	chr15	91523464	+	GTACTAACCAGCACAGAAAG
4476	sgPRC1_8	PRC1	chr15	91519908	-	GCTCCAGAAAATGCTGCCCA
4477	sgPRC1_9	PRC1	chr15	91517403	-	CGAACACCTAGCAAGCGGCG
4478	sgPRC1_10	PRC1	chr15	91513645	+	AAGGAGGAAGTCGAGACACG
4479	sgRAB6B_1	RAB6B	chr3	133614303	-	GCTGCCAGCACCATGTCCGC
4480	sgRAB6B_2	RAB6B	chr3	133560156	-	CTACATCCGGGACTCCACGG
4481	sgRAB6B_3	RAB6B	chr3	133557087	-	TAGGCAGATAACCATCGAGG
4482	sgRAB6B_4	RAB6B	chr3	133560139	+	GTACACCACCACAGCCACCG
4483	sgRAB6B_5	RAB6B	chr3	133557019	+	GTTGTAGCCAGTCTTCGCAC
4484	sgRAB6B_6	RAB6B	chr3	133558363	-	GGTGGGCAACAAGACGGACC
4485	sgRAB6B_7	RAB6B	chr3	133558407	-	GACGACGTCAGGACAGAGAG
4486	sgRAB6B_8	RAB6B	chr3	133553424	-	GTCCAGGAGAAAAGCAAAGA
4487	sgRAB6B_9	RAB6B	chr3	133560220	-	GACAGGTGCGACTGCAGCTC
4488	sgRAB6B_10	RAB6B	chr3	133558381	-	TGATGTTATCATCATGCTGG
4489	sgRASD1_1	RASD1	chr17	17399294	+	GGATGGAGTAGAACTTGCGG
4490	sgRASD1_2	RASD1	chr17	17399273	+	CGAGCTGGTAGACCTCGCCG
4491	sgRASD1_3	RASD1	chr17	17399358	+	GGTGAGGAAGCGCGACACGA
4492	sgRASD1_4	RASD1	chr17	17399246	+	GGTGGTTGCCGGACGTGTCG
4493	sgRASD1_5	RASD1	chr17	17399217	+	GATGGAGAGGCGCCGCATGG
4494	sgRASD1_6	RASD1	chr17	17399387	+	TGCCCACCTTGGACGAGCCG
4495	sgRASD1_7	RASD1	chr17	17399337	+	GTAGGCGTCCTCGAAGCGGC
4496	sgRASD1_8	RASD1	chr17	17399204	+	GCTCACCTGTGAGGATGGAG
4497	sgRASD1_9	RASD1	chr17	17399463	+	GCACATCTTCTTGATCATCG
4498	sgRASD1_10	RASD1	chr17	17399453	-	GAAGATGTGCCCGAGCGACT
4499	sgRSBN1_1	RSBN1	chr1	114354999	-	GAAGAACGGCCGACAAGTGG
4500	sgRSBN1_2	RSBN1	chr1	114354971	-	GGAGAGACTCCAATGCCCAG
4501	sgRSBN1_3	RSBN1	chr1	114354851	-	GGTGCGCGTAGTACGGGCGG
4502	sgRSBN1_4	RSBN1	chr1	114354818	-	GGAGCCGGACAAAGAGGGGA
4503	sgRSBN1_5	RSBN1	chr1	114354577	+	GGTGCCAGCGAAGGTGGCGG
4504	sgRSBN1_6	RSBN1	chr1	114354881	-	TGTGGGTGAAATGGCTGCGC
4505	sgRSBN1_7	RSBN1	chr1	114354800	-	GAAGGAGAAACCTCATGCTG
4506	sgRSBN1_8	RSBN1	chr1	114354770	+	GCTGCCGTTTAACTCCCCGC
4507	sgRSBN1_9	RSBN1	chr1	114354757	-	CGGCAGCGCCGATCTAGCAG
4508	sgRSBN1_10	RSBN1	chr1	114354513	+	CGAGAAGGTGAAGAGGGCCG
4509	sgRSBN1L_1	RSBN1L	chr7	77325895	-	TTGGCGGACAGAGAACCCGG
4510	sgRSBN1L_2	RSBN1L	chr7	77326073	-	GCTCCGAGAAGAGGACGGGG
4511	sgRSBN1L_3	RSBN1L	chr7	77326136	-	CGGCTGAGACAAGGAAGCGG
4512	sgRSBN1L_4	RSBN1L	chr7	77326351	+	GAGGTGAAGCCGCTGCCCCG

4513	sgRSBN1L_5	RSBN1L	chr7	77325926	+	CTGAGGAGAAGAAGGCACCG
4514	sgRSBN1L_6	RSBN1L	chr7	77325945	+	GCGGAGAGTGAACGGAGAAG
4515	sgRSBN1L_7	RSBN1L	chr7	77326056	-	GGGAGGGAGCAGCAGACAGA
4516	sgRSBN1L_8	RSBN1L	chr7	77326241	-	GCGCGACTTGGCGTTAGCCG
4517	sgRSBN1L_9	RSBN1L	chr7	77326290	+	GAGGAGGCACGGTCTCGGTG
4518	sgRSBN1L_10	RSBN1L	chr7	77379088	+	ATAGAGCACCAGCCTAATGG
4519	sgSCARA5_1	SCARA5	chr8	27779098	+	GGAGATGTTCCGCAGTGCGA
4520	sgSCARA5_2	SCARA5	chr8	27824043	-	CAGGTCCATGTCACAAACGG
4521	sgSCARA5_3	SCARA5	chr8	27779511	-	GCAGGCGGTGCAGACCGAGC
4522	sgSCARA5_4	SCARA5	chr8	27779436	-	GGAGCTCTACCAGCTGCAGG
4523	sgSCARA5_5	SCARA5	chr8	27779175	-	GCTGCAGCTGAAGCAGGAGC
4524	sgSCARA5_6	SCARA5	chr8	27764729	-	GAGGTACCCCAGGATTGCCC
4525	sgSCARA5_7	SCARA5	chr8	27762304	-	AGAGAAAGGAGACAGAGCTG
4526	sgSCARA5_8	SCARA5	chr8	27737236	+	GACCTGAGCCATTCACCAGG
4527	sgSCARA5_9	SCARA5	chr8	27737152	-	GGACAAGAAGGACGGAGACG
4528	sgSCARA5_10	SCARA5	chr8	27737107	+	GGTACACCTCCTCCACACCG
4529	sgSCD_1	SCD	chr10	102112187	-	GCCGAGCTTTGTAAGAGCGG
4530	sgSCD_2	SCD	chr10	102116488	-	GAAACCAGGATATTCTCCCG
4531	sgSCD_3	SCD	chr10	102116448	-	AAGGACGATATCCGAAGAGG
4532	sgSCD_4	SCD	chr10	102114247	+	GATCCTCATAATTCCCGACG
4533	sgSCD_5	SCD	chr10	102116344	-	GTTTCACCCCAGAAATACCA
4534	sgSCD_6	SCD	chr10	102112214	-	CAATGATCAGAAAGAGCCGT
4535	sgSCD_7	SCD	chr10	102116423	-	AGCACTGTTCACCAGCCAGG
4536	sgSCD_8	SCD	chr10	102114311	+	ACCCAGCTGTCAAAGAGAAG
4537	sgSCD_9	SCD	chr10	102107867	-	TCCATTCTGCAGGACCCTGG
4538	sgSCD_10	SCD	chr10	102107273	-	TCACATCGTCCTGCAGCAAG
4539	sgSDHAF1_1	SDHAF1	chr19	36486385	-	TACGAAGGCGCCCATGGCGG
4540	sgSDHAF1_2	SDHAF1	chr19	36486438	+	GTGGGTTGCCAGCCTGACGA
4541	sgSDHAF1_3	SDHAF1	chr19	36486324	+	ATCGAGTACCTGTACCGCCG
4542	sgSDHAF1_4	SDHAF1	chr19	36486219	+	TACCGCGATCTGCTGCGCGC
4543	sgSDHAF1_5	SDHAF1	chr19	36486313	-	GTACTCGATGCGCAGCACGT
4544	sgSDHAF1_6	SDHAF1	chr19	36486354	+	CAGCTGCAGCTGCTACGCTC
4545	sgSDHAF1_7	SDHAF1	chr19	36486509	-	CCTGTCACCGTCCGTCGGGG
4546	sgSDHAF1_8	SDHAF1	chr19	36486163	+	GCTTAGCCGCGGTCATGAGC
4547	sgSDHAF1_9	SDHAF1	chr19	36486185	+	GCACAGCCGGCTGCAGAGGC
4548	sgSDHAF1_10	SDHAF1	chr19	36486449	-	GACTGTCGCCGTCGTCAGGC
4549	sgSET_1	SET	chr9	131454951	+	GCTTGGGGAGGAAGATGAAG
4550	sgSET_2	SET	chr9	131454258	+	ACATTTGTCAACCATCCACA
4551	sgSET_3	SET	chr9	131454935	+	GCCTAGTGTCTGCACTGCTT
4552	sgSET_4	SET	chr9	131446265	+	GCAGGCTTGCCGAAGAAGGG
4553	sgSET_5	SET	chr9	131451852	+	GAGCAGCACCATGTCGGCGC
4554	sgSET_6	SET	chr9	131451895	+	GAGCTCAACTCCAACCACGA
4555	sgSET_7	SET	chr9	131452299	-	CGTCCGGCCGCACCATGTGG
4556	sgSET_8	SET	chr9	131451923	-	GCTCTCACCTGAGGTCTCGT
4557	sgSET_9	SET	chr9	131447467	-	AGGTAGGTTTCAAGCTGGGC
4558	sgSET_10	SET	chr9	131447483	-	TGTGGCTCACCCAGCAAGGT
4559	sgSETD7_1	SETD7	chr4	140477258	-	CGAGATGGTGGAGGAGGCGG
4560	sgSETD7_2	SETD7	chr4	140477270	-	GGATAGCGACGACGAGATGG
4561	sgSETD7_3	SETD7	chr4	140468176	-	GACGATGACGGATTACCGCA
4562	sgSETD7_4	SETD7	chr4	140468103	-	TTCACGGAGAAAAGAACGGA
4563	sgSETD7_5	SETD7	chr4	140454479	-	GATGATGCCTTGCAGGGCCA
4564	sgSETD7_6	SETD7	chr4	140454458	-	GGAGTTTACACTTACGAAGA
4565	sgSETD7_7	SETD7	chr4	140444522	+	AGGATCTGGAAGAAGAGCAT
4566	sgSETD7_8	SETD7	chr4	140441516	-	GAATCTCTTATTTCCAGTGC
4567	sgSETD7_9	SETD7	chr4	140441433	-	AGTTCGAATTACACACCAAG
4568	sgSETD7_10	SETD7	chr4	140439044	+	GTAGATGCAGTTTGGAGTGA
4569	sgSGK1_1	SGK1	chr6	134495182	+	AGGTTGGGAGATCTTCAAGA
4570	sgSGK1_2	SGK1	chr6	134493851	+	GCAGCATAGAAACGAGCCCG
4571	sgSGK1_3	SGK1	chr6	134495711	-	CCACAGCTTTCATGAAGCAG
4572	sgSGK1_4	SGK1	chr6	134495153	+	GGGTTGGCATTCATAAGCTC
4573	sgSGK1_5	SGK1	chr6	134492199	+	TCTGCAGGAGGCCCTCCAGG

4574	sgSGK1_6	SGK1	chr6	134492178	-	GGACAGGACAAAGCGGCTCG
4575	sgSGK1_7	SGK1	chr6	134494659	+	GAGGATTGGACGACGGGCCA
4576	sgSGK1_8	SGK1	chr6	134494220	+	AGTGAAGGCCCACCAGGAAA
4577	sgSGK1_9	SGK1	chr6	134494205	+	CAGTCTGGAAAGAGAAGTGA
4578	sgSGK1_10	SGK1	chr6	134494168	-	GTCCTAGACTACATTAATGG
4579	sgSLC22A17_1	SLC22A17	chr14	23817141	+	GTAGTTGAGGAGGGAAGCAA
4580	sgSLC22A17_2	SLC22A17	chr14	23821323	-	GAGCAGCCTCCCAATGCCAG
4581	sgSLC22A17_3	SLC22A17	chr14	23821286	+	GCTGGCTGCTAGGGCAGCGC
4582	sgSLC22A17_4	SLC22A17	chr14	23821233	+	GGGGCGAAGCCGCTGCACGA
4583	sgSLC22A17_5	SLC22A17	chr14	23821214	+	GCAATGGTTGAAGTCCGGCG
4584	sgSLC22A17_6	SLC22A17	chr14	23818607	+	TCAGCAGCACAATCCCGCGA
4585	sgSLC22A17_7	SLC22A17	chr14	23818517	+	GAAAGCCCAAGAGGAATCGG
4586	sgSLC22A17_8	SLC22A17	chr14	23817457	-	AGTGAAGCGGCAGATTGAGG
4587	sgSLC22A17_9	SLC22A17	chr14	23816774	+	AGAGAAGAAGGATGCCCCGG
4588	sgSLC22A17_10	SLC22A17	chr14	23816339	+	AAGGAGGGTGCTGAGGATGG
4589	sgSLC39A1_1	SLC39A1	chr1	153935072	+	GAGCACCAGCAGCAGCACCA
4590	sgSLC39A1_2	SLC39A1	chr1	153935048	+	GGGCACCAGGCTGCAGAGGA
4591	sgSLC39A1_3	SLC39A1	chr1	153935162	+	CAGGAGCTCTGGCTCTCCCC
4592	sgSLC39A1_4	SLC39A1	chr1	153935131	+	GCTCTGAAGCTACCGCCTCG
4593	sgSLC39A1_5	SLC39A1	chr1	153935101	-	TGTGGGGCTGGAGGTGAAGT
4594	sgSLC39A1_6	SLC39A1	chr1	153935027	+	TGGCCGGCGCAGCACACAGA
4595	sgSLC39A1_7	SLC39A1	chr1	153935007	-	GGAGCTAACCATGAAGGCTC
4596	sgSLC39A1_8	SLC39A1	chr1	153934747	+	CAGCAGGTCCAGGAGACAAG
4597	sgSLC39A1_9	SLC39A1	chr1	153935151	+	GGGCGCCACACCAGGAGCTC
4598	sgSLC39A1_10	SLC39A1	chr1	153934800	+	CTAGGCTCAGGGCTTTCTGG
4599	sgSLC39A10_1	SLC39A10	chr2	196545312	-	CAAGATGATGATGCAAACGA
4600	sgSLC39A10_2	SLC39A10	chr2	196545408	+	AACAGAGACCAATAAAACCC
4601	sgSLC39A10_3	SLC39A10	chr2	196581571	+	TGAGGAAGCATAATCACCAG
4602	sgSLC39A10_4	SLC39A10	chr2	196581626	-	GATCGGATCCAGAATGACAG
4603	sgSLC39A10_5	SLC39A10	chr2	196581696	+	GGCATCCACAACTTCAGTGA
4604	sgSLC39A10_6	SLC39A10	chr2	196548503	-	GATATAACAATGCAGGGCAA
4605	sgSLC39A10_7	SLC39A10	chr2	196581477	+	ATAGACCATTCTCACAGTGA
4606	sgSLC39A10_8	SLC39A10	chr2	196581531	+	GCTGCTGCACATAACCACCA
4607	sgSLC39A10_9	SLC39A10	chr2	196581551	-	GCACAGTTTTGTTCTCGCCG
4608	sgSLC39A10_10	SLC39A10	chr2	196544838	-	GGTCATGTTCTTCATGGCAA
4609	sgSLC39A11_1	SLC39A11	chr17	70645369	-	GGGATCCAGAATTTCCCCGA
4610	sgSLC39A11_2	SLC39A11	chr17	70845883	-	GCAGCCACTGGCCTTCCAGA
4611	sgSLC39A11_3	SLC39A11	chr17	70845835	-	GGGAATCTGGCACAGCCCGG
4612	sgSLC39A11_4	SLC39A11	chr17	70645327	+	GAGAAGCCTGCCCCTCGCAA
4613	sgSLC39A11_5	SLC39A11	chr17	70645050	+	GCACCAAAGACCCCGGCCAG
4614	sgSLC39A11_6	SLC39A11	chr17	71084855	+	AGAAGGTCCCCAGCAAGGCC
4615	sgSLC39A11_7	SLC39A11	chr17	71027795	+	GACGTGGCCATCTCAACTGC
4616	sgSLC39A11_8	SLC39A11	chr17	71027691	+	TCACCAAGTGAGGCATCAGG
4617	sgSLC39A11_9	SLC39A11	chr17	70845869	+	GGACAGCAGGACCCTCTGGA
4618	sgSLC39A11_10	SLC39A11	chr17	70645081	-	GTATGGGCAGCTGAGCGGCA
4619	sgSLC39A12_1	SLC39A12	chr10	18242296	+	GCCCAGGATAGCAGAAGCCG
4620	sgSLC39A12_2	SLC39A12	chr10	18242280	+	GACAGACAAACCCTCAGCCC
4621	sgSLC39A12_3	SLC39A12	chr10	18242414	+	AGAAAACTGGGTGCCCACGG
4622	sgSLC39A12_4	SLC39A12	chr10	18254519	-	GTCCCAGACAAACACCCTGG
4623	sgSLC39A12_5	SLC39A12	chr10	18276406	+	AGAATACGGCTACAGCACGG
4624	sgSLC39A12_6	SLC39A12	chr10	18280116	+	GGGCATTTCCATGAAAGCAA
4625	sgSLC39A12_7	SLC39A12	chr10	18242245	-	CGGCTGAGTAGAAGAAACAA
4626	sgSLC39A12_8	SLC39A12	chr10	18250557	+	AGATCAGCTTAGAGAAGAAG
4627	sgSLC39A12_9	SLC39A12	chr10	18270351	-	GTAAGTGGCAGGAGCAGCTG
4628	sgSLC39A12_10	SLC39A12	chr10	18276441	+	TCACACTGGGCTCCATGCTG
4629	sgSLC39A13_1	SLC39A13	chr11	47433496	+	GCAGCTGCTCAGCTTCGCCC
4630	sgSLC39A13_2	SLC39A13	chr11	47431688	-	GCAGTGAGGAAGAGGAGCCT
4631	sgSLC39A13_3	SLC39A13	chr11	47431717	+	GGAGCTCTTGGAAAGGGCTG
4632	sgSLC39A13_4	SLC39A13	chr11	47433982	+	GATGTTCCTGGACAGCAAGG
4633	sgSLC39A13_5	SLC39A13	chr11	47434975	+	GCTGCCGCCGCGCTCAATGG
4634	sgSLC39A13_6	SLC39A13	chr11	47436384	+	GCTGCAACTCTCAACAGCGC

4635	sgSLC39A13_7	SLC39A13	chr11	47436644	-	GATGTAGAGAAAGCCGCCAG
4636	sgSLC39A13_8	SLC39A13	chr11	47431661	-	GCCATGCCACAGCCAGGGCA
4637	sgSLC39A13_9	SLC39A13	chr11	47431903	+	GCTTGTCATTCCCCTAGAGA
4638	sgSLC39A13_10	SLC39A13	chr11	47433544	+	GCATCTGCTGCCCGAAGCCT
4639	sgSLC39A14_1	SLC39A14	chr8	22265966	+	GGAGAATGAGCAGACGGAGG
4640	sgSLC39A14_2	SLC39A14	chr8	22262427	-	GGCCCACTCCCACATCCAGG
4641	sgSLC39A14_3	SLC39A14	chr8	22273658	+	TGGATGATCACTCTGAGCGA
4642	sgSLC39A14_4	SLC39A14	chr8	22273768	-	CATGTGGGAACTCCTCACAG
4643	sgSLC39A14_5	SLC39A14	chr8	22262348	-	TATTAGATCCTGCAGGAAGG
4644	sgSLC39A14_6	SLC39A14	chr8	22265914	-	GGAATCCAGCTGCTGGAGGA
4645	sgSLC39A14_7	SLC39A14	chr8	22273323	+	CTCCAAGAAGGACCAGGAGG
4646	sgSLC39A14_8	SLC39A14	chr8	22273413	+	GGACGGCAAGGCGCCCATGG
4647	sgSLC39A14_9	SLC39A14	chr8	22273679	+	GGCCTCCATAATTTCATCGA
4648	sgSLC39A14_10	SLC39A14	chr8	22262276	-	GGTTCTCCATAAGCCAAGCA
4649	sgSLC39A2_1	SLC39A2	chr14	21467971	+	GCATATGACTGCTGAAGCCC
4650	sgSLC39A2_2	SLC39A2	chr14	21467929	-	CACCAGCAGAAATACAGCCC
4651	sgSLC39A2_3	SLC39A2	chr14	21467942	+	GCTGGTGTTTTCCTGGGAGC
4652	sgSLC39A2_4	SLC39A2	chr14	21467699	+	TTCCAGATTGATGCAGCCAG
4653	sgSLC39A2_5	SLC39A2	chr14	21468010	+	GATTCAGAAGTTCATGGTGC
4654	sgSLC39A2_6	SLC39A2	chr14	21467651	+	TTGGCTCTCACTCTGGGCTG
4655	sgSLC39A2_7	SLC39A2	chr14	21467641	-	TGAGAGCCAACAGGGCAAAC
4656	sgSLC39A2_8	SLC39A2	chr14	21467956	-	TATGCATGAACCCTGCTCCC
4657	sgSLC39A2_9	SLC39A2	chr14	21467991	-	CTGTGATTCAATTTCCTCCA
4658	sgSLC39A2_10	SLC39A2	chr14	21468284	+	GCAAGTGAGAGAAATTCTTC
4659	sgSLC39A3_1	SLC39A3	chr19	2737207	+	AGAACACGCCCACCATGCAC
4660	sgSLC39A3_2	SLC39A3	chr19	2737235	+	GGCCACTAGCAATTTCACCA
4661	sgSLC39A3_3	SLC39A3	chr19	2737219	-	GGCCAAAATCCTGTGCATGG
4662	sgSLC39A3_4	SLC39A3	chr19	2737150	-	CATCGAGACAGATTTTGAGA
4663	sgSLC39A3_5	SLC39A3	chr19	2737105	+	CAAAGGTGTTGCAGAGAGAG
4664	sgSLC39A3_6	SLC39A3	chr19	2737064	+	CAGCAGAGCGTTGAAGCACG
4665	sgSLC39A3_7	SLC39A3	chr19	2737088	+	CAGAAACACCCCTCCTCCAA
4666	sgSLC39A3_8	SLC39A3	chr19	2737048	-	GCTGCCCGCTGTGAGGGAAA
4667	sgSLC39A3_9	SLC39A3	chr19	2737165	+	CGATGATCTTCACGGGGAGC
4668	sgSLC39A3_10	SLC39A3	chr19	2733438	-	CATCAGCACCGACTACCCGC
4669	sgSLC39A4_1	SLC39A4	chr8	145641353	+	GCTGAGGTACAGGACGGCGG
4670	sgSLC39A4_2	SLC39A4	chr8	145640767	-	TCAGCTGCTGGAGGAGGCGG
4671	sgSLC39A4_3	SLC39A4	chr8	145641295	+	GGTCTGCATGAGAGGCCCAG
4672	sgSLC39A4_4	SLC39A4	chr8	145641235	+	GCAGCAGCCAGCTCAGGCCC
4673	sgSLC39A4_5	SLC39A4	chr8	145640663	+	GAAGTACTGAGGGCTCGGCA
4674	sgSLC39A4_6	SLC39A4	chr8	145640214	-	ACAGGCTGGGGTGACCCCGG
4675	sgSLC39A4_7	SLC39A4	chr8	145639781	+	GCAGATGAGCAGCGTGGCCA
4676	sgSLC39A4_8	SLC39A4	chr8	145639759	+	GGAGGCCAAAGACCGCGCAG
4677	sgSLC39A4_9	SLC39A4	chr8	145639452	-	GCTGCATACACACAGCGAAG
4678	sgSLC39A4_10	SLC39A4	chr8	145639190	+	CCAGCTGCAGGGACACACCG
4679	sgSLC39A5_1	SLC39A5	chr12	56626555	+	AAGGCCCCTCACCTACCCCG
4680	sgSLC39A5_2	SLC39A5	chr12	56626589	-	GTGAAGGAGGTCCAGGCCCG
4681	sgSLC39A5_3	SLC39A5	chr12	56628652	-	GAGGGGTCAGAGGAGCAGCG
4682	sgSLC39A5_4	SLC39A5	chr12	56628669	-	CAGCAGAGCAAACTGACGAG
4683	sgSLC39A5_5	SLC39A5	chr12	56628959	-	GCTGAGCAACAGGACTGCCA
4684	sgSLC39A5_6	SLC39A5	chr12	56630181	+	GATGCTGCAGGCGAAAACGA
4685	sgSLC39A5_7	SLC39A5	chr12	56630497	+	GGTCTACACAACCTCACTGA
4686	sgSLC39A5_8	SLC39A5	chr12	56631071	-	GCAGTGACCCCAAACACCCA
4687	sgSLC39A5_9	SLC39A5	chr12	56628981	-	GCAGGGATAGGGGAGAAGGG
4688	sgSLC39A5_10	SLC39A5	chr12	56629061	-	ACAAAGAGTGCCCACCGCCA
4689	sgSLC39A6_1	SLC39A6	chr18	33694074	-	GGCCTGCACAATTTCAGCGA
4690	sgSLC39A6_2	SLC39A6	chr18	33702148	+	GAAGACAGATGACTGAAAAG
4691	sgSLC39A6_3	SLC39A6	chr18	33694213	+	TATGATGGTAGTCATGATGG
4692	sgSLC39A6_4	SLC39A6	chr18	33694177	+	GAGGATGGTGGTTTTGGTGG
4693	sgSLC39A6_5	SLC39A6	chr18	33694144	+	GCTCCTCCCGAGAGTAGCGC
4694	sgSLC39A6_6	SLC39A6	chr18	33702188	-	TAGCCATGAAGAACCAGCAA
4695	sgSLC39A6_7	SLC39A6	chr18	33702175	-	CCAGCAATGGAAATGAAAAG

4696	sgSLC39A6_8	SLC39A6	chr18	33702111	-	GTGCCTATTTTGATTCCACG
4697	sgSLC39A6_9	SLC39A6	chr18	33694375	+	CAGGCTGCTGAGAATCAAAG
4698	sgSLC39A6_10	SLC39A6	chr18	33694162	+	GCTGGCTGTGACTGTGAGGA
4699	sgSLC39A7_1	SLC39A7	chr6	33170387	+	AGAGGGGTTCAGAAGAGGCG
4700	sgSLC39A7_2	SLC39A7	chr6	33169049	-	GCAGTCCCACGGCCACCCAG
4701	sgSLC39A7_3	SLC39A7	chr6	33169581	-	GGGGAGAGTTCGACTCCACG
4702	sgSLC39A7_4	SLC39A7	chr6	33169600	-	TGAAGTAGAGAGCGATGCCG
4703	sgSLC39A7_5	SLC39A7	chr6	33169881	+	GGACATGGACACTCCCACAG
4704	sgSLC39A7_6	SLC39A7	chr6	33170111	+	TTTGTGAGACATGTGAAAGG
4705	sgSLC39A7_7	SLC39A7	chr6	33170691	-	TCAGGTACCCCGACACACGC
4706	sgSLC39A7_8	SLC39A7	chr6	33169018	+	GCGTGATGGCCAGAGGCCTG
4707	sgSLC39A7_9	SLC39A7	chr6	33169075	-	CAGAAGCCCCAAGGTCGCCC
4708	sgSLC39A7_10	SLC39A7	chr6	33169187	-	GGGCATGGCTGTGGCCATGG
4709	sgSLC39A8_1	SLC39A8	chr4	103189017	+	AGAGCTCCTTACCTAACTCG
4710	sgSLC39A8_2	SLC39A8	chr4	103265729	-	GCCAGGGCTAGCCTTCAGCG
4711	sgSLC39A8_3	SLC39A8	chr4	103228691	+	GTGGAGTCAAAATCAATCCG
4712	sgSLC39A8_4	SLC39A8	chr4	103189124	-	GCCCTCCACAATTTCATCGA
4713	sgSLC39A8_5	SLC39A8	chr4	103265751	-	GCCTCGGAGGAGTGGCGGAG
4714	sgSLC39A8_6	SLC39A8	chr4	103265715	+	GCTCAGCACATCCTCGCTGA
4715	sgSLC39A8_7	SLC39A8	chr4	103236907	+	GATGACAGAGAATTTGGAGC
4716	sgSLC39A8_8	SLC39A8	chr4	103265780	-	GGCCGGGCTCCTGTTGCTGG
4717	sgSLC39A8_9	SLC39A8	chr4	103265654	+	CCATCTGCTCCAGCAAGTGC
4718	sgSLC39A8_10	SLC39A8	chr4	103189075	+	GAGTCCCTGAAGGAGAGACA
4719	sgSLC39A9_1	SLC39A9	chr14	69908915	-	GAAAACGAAGCCCAGAACGA
4720	sgSLC39A9_2	SLC39A9	chr14	69922455	-	GAAACCAGTCCAAAAGCAGC
4721	sgSLC39A9_3	SLC39A9	chr14	69922477	-	TAAGCCAGCATGCATCAAGA
4722	sgSLC39A9_4	SLC39A9	chr14	69890849	-	CCAGAGCAGTTCCACAGAGA
4723	sgSLC39A9_5	SLC39A9	chr14	69922517	+	GCACTTGCTGGTCTTTGCAT
4724	sgSLC39A9_6	SLC39A9	chr14	69866113	+	GCTGTCTCTGGCTATGTTGG
4725	sgSLC39A9_7	SLC39A9	chr14	69866163	-	CTCTGAGAAATTAACAGCCA
4726	sgSLC39A9_8	SLC39A9	chr14	69908966	-	ACCGTCAGTAGAATGCACAT
4727	sgSLC39A9_9	SLC39A9	chr14	69922558	-	CAGTCCTAAGTATGTCACCA
4728	sgSLC39A9_10	SLC39A9	chr14	69866143	+	CGTGGCCGGAATCATTCCCT
4729	sgSOD2_1	SOD2	chr6	160113723	-	GAACAACCTGAACGTCACCG
4730	sgSOD2_2	SOD2	chr6	160113753	-	GCACCACAGCAAGCACCACG
4731	sgSOD2_3	SOD2	chr6	160113702	-	GGAGAAGTACCAGGAGGCGT
4732	sgSOD2_4	SOD2	chr6	160105961	+	GCCAACCCCAACCTGAGCCT
4733	sgSOD2_5	SOD2	chr6	160106003	-	CAAGTTTAAGGAGAAGCTGA
4734	sgSOD2_6	SOD2	chr6	160105944	-	GGCTTGGTTTCAATAAGGAA
4735	sgSOD2_7	SOD2	chr6	160105882	+	TAACCTGTTGTTCCTTGCAG
4736	sgSOD2_8	SOD2	chr6	160113816	+	AGTCGTAGGGCAGGTCGGGG
4737	sgSOD2_9	SOD2	chr6	160113735	+	GGTTGTTCACGTAGGCCGCG
4738	sgSOD2_10	SOD2	chr6	160106035	-	GAAGCCATCAAACGTGACTT
4739	sgSP1_1	SP1	chr12	53800474	+	AAGCCGGTCGGAGGACCCGG
4740	sgSP1_2	SP1	chr12	53775957	+	GAGAACAGCAACAACTCCCA
4741	sgSP1_3	SP1	chr12	53776143	+	AAGAATCGCACAGTCTCTGG
4742	sgSP1_4	SP1	chr12	53777080	+	TCATCCGGACACCAACAGTG
4743	sgSP1_5	SP1	chr12	53775565	+	GGCAGTAGCAGCAGCACTGG
4744	sgSP1_6	SP1	chr12	53776374	+	ATCACAAATCGAGGAAGTGG
4745	sgSP1_7	SP1	chr12	53776719	+	ACTACTACCACCAGCAACAT
4746	sgSP1_8	SP1	chr12	53776783	+	CTCAAGGCCAGACACCCCAG
4747	sgSP1_9	SP1	chr12	53776842	+	CAGCAAAACCAGACATCTGG
4748	sgSP1_10	SP1	chr12	53776967	-	GTGCTGCTTGGAGGGCCTGG
4749	sgSTEAP1B_1	STEAP1B	chr7	22533347	-	TTCAGAACTTCAGCACGCAC
4750	sgSTEAP1B_2	STEAP1B	chr7	22459413	+	GCAGGATCTGTCACAAGGTC
4751	sgSTEAP1B_3	STEAP1B	chr7	22478304	-	CTGGCCAAGACACAACTGGT
4752	sgSTEAP1B_4	STEAP1B	chr7	22478196	-	TCAGCAGCCAGCCCTTCAAA
4753	sgSTEAP1B_5	STEAP1B	chr7	22478186	+	AACATTTCCCTTTGAAGGGC
4754	sgSTEAP1B_6	STEAP1B	chr7	22478284	-	AGGCACACCAAATTTCTGGC
4755	sgSTEAP1B_7	STEAP1B	chr7	22478235	+	CTCCTAGTCTTGGAAGGTGG
4756	sgSTEAP1B_8	STEAP1B	chr7	22478271	-	TTCTGGCAGGAGCAGCAGTG

4757	sgSTEAP1B_9	STEAP1B	chr7	22532249	-	ACTGGCAATACTGGCTCTGT
4758	sgSTEAP1B_10	STEAP1B	chr7	22533397	+	TGCTGCAAATGCAAAAGCAC
4759	sgTMEM199_1	TMEM199	chr17	26684849	-	GACGGAAAGAAACCAAGCGT
4760	sgTMEM199_2	TMEM199	chr17	26684805	+	CTGGGGAAGAAGCACAAGGG
4761	sgTMEM199_3	TMEM199	chr17	26684764	+	AGCGGCTACCCCGAAAGCTG
4762	sgTMEM199_4	TMEM199	chr17	26684867	-	GGTGCAGATCCCGGATGAGA
4763	sgTMEM199_5	TMEM199	chr17	26686007	-	TATCCCTTACATACCCGTGG
4764	sgTMEM199_6	TMEM199	chr17	26686361	+	GGAGAAGATTAAGATACAGC
4765	sgTMEM199_7	TMEM199	chr17	26684820	+	AAGGGCGGTGATAGCTCCAG
4766	sgTMEM199_8	TMEM199	chr17	26685955	-	TGCCTTCTAGGAGCTCATGG
4767	sgTMEM199_9	TMEM199	chr17	26687546	+	TCCCAGGATACAAGACATGG
4768	sgTMEM199_10	TMEM199	chr17	26684727	+	GTGCGTGCTTTGGGCCCCGG
4769	sgTSHZ1_1	TSHZ1	chr18	72997663	-	GAGACGCTGTCGGGACACTG
4770	sgTSHZ1_2	TSHZ1	chr18	72997928	-	GGAGCTGCTGCTGGTACTGG
4771	sgTSHZ1_3	TSHZ1	chr18	72998198	+	ACTCCGAGAAGACCAAGAGG
4772	sgTSHZ1_4	TSHZ1	chr18	72998393	-	GGAGGGGACCAGTTTGGTGA
4773	sgTSHZ1_5	TSHZ1	chr18	72998814	+	AAAGCAGCCCGACTCTCCCG
4774	sgTSHZ1_6	TSHZ1	chr18	72998984	+	TGGACGACAGCCCCAAGGGA
4775	sgTSHZ1_7	TSHZ1	chr18	72999064	+	AATGGTGCGCCCTCATGGGG
4776	sgTSHZ1_8	TSHZ1	chr18	72999253	-	GACACGTTGCTCTTGTGCGG
4777	sgTSHZ1_9	TSHZ1	chr18	72999282	+	GGAGCTGGTGGAGAAGGTCA
4778	sgTSHZ1_10	TSHZ1	chr18	72999533	+	CCAACGGCTGTAACAACCTG
4779	sgTSHZ2_1	TSHZ2	chr20	51870051	+	GGAACAGCTGAAAGAAGAGG
4780	sgTSHZ2_2	TSHZ2	chr20	51870069	+	GGAGGAAATAAAAGAAGAGG
4781	sgTSHZ2_3	TSHZ2	chr20	51870109	+	TCAGTAGCTCAACTGCAGGG
4782	sgTSHZ2_4	TSHZ2	chr20	51870126	+	GGGTGGCAATGACACAGGGA
4783	sgTSHZ2_5	TSHZ2	chr20	51870554	-	GAACAGGCTGGGTTTCGAGA
4784	sgTSHZ2_6	TSHZ2	chr20	51870609	+	GTGCGGGACTGTGTTCACAG
4785	sgTSHZ2_7	TSHZ2	chr20	51871254	+	GGTATTAGACCCGTTAGCAG
4786	sgTSHZ2_8	TSHZ2	chr20	51871516	+	GAAGATGGCTCAAAGGGTGG
4787	sgTSHZ2_9	TSHZ2	chr20	51871703	+	CTAATCTCACCAACAAGCTG
4788	sgTSHZ2_10	TSHZ2	chr20	51871756	-	TGTGTGGGCATAGAAACCAG
4789	sgTSHZ3_1	TSHZ3	chr19	31840117	-	GGAGAAGCATCATGCCGAGG
4790	sgTSHZ3_2	TSHZ3	chr19	31840099	-	GGAGGAAGCAGCAGGCGCCC
4791	sgTSHZ3_3	TSHZ3	chr19	31840077	+	GGCTCGTACCTGCTGCGCGC
4792	sgTSHZ3_4	TSHZ3	chr19	31768092	+	GGAGATGCTGGAAGGAGTGG
4793	sgTSHZ3_5	TSHZ3	chr19	31770513	+	GGAGTTCTGGTAGCTGGGGC
4794	sgTSHZ3_6	TSHZ3	chr19	31770398	-	GAACGAAGAGGAGACCAAGG
4795	sgTSHZ3_7	TSHZ3	chr19	31770344	-	GGATAGCCTGGAGCAGATGA
4796	sgTSHZ3_8	TSHZ3	chr19	31770003	+	GTGCACCGTCAACTCCACCA
4797	sgTSHZ3_9	TSHZ3	chr19	31769993	-	GACGGTGCACATGAACGAGA
4798	sgTSHZ3_10	TSHZ3	chr19	31769940	-	ATAACAACAACCCCAAGCGC
4799	sgTYW1_1	TYW1	chr7	66548414	+	TAGGCACCACACCAACCCCG
4800	sgTYW1_2	TYW1	chr7	66483109	+	ATTGCATCATAGAGACACCG
4801	sgTYW1_3	TYW1	chr7	66463161	+	CCAGATTGTCATCAAGACGC
4802	sgTYW1_4	TYW1	chr7	66520853	+	GAAGCTTTGCAGGTGGACAA
4803	sgTYW1_5	TYW1	chr7	66548431	+	CCGTGGGCACTGAGTGGCGG
4804	sgTYW1_6	TYW1	chr7	66660243	-	GGATCAGATCCACCAACTCG
4805	sgTYW1_7	TYW1	chr7	66483082	+	GGAGGAAGGATCTCATGAGC
4806	sgTYW1_8	TYW1	chr7	66532272	+	TCCATGCTCCGAGGGAGAGG
4807	sgTYW1_9	TYW1	chr7	66582476	-	ACAGCTGAGTAACCGGCTCG
4808	sgTYW1_10	TYW1	chr7	66660200	-	ATGCCAGGGCACGTGGGCCA
4809	sgUBE2D1_1	UBE2D1	chr10	60121125	-	CCAGCTGAACAGTGAGCAGG
4810	sgUBE2D1_2	UBE2D1	chr10	60123369	+	CCTGATAGCGCATATCAAGG
4811	sgUBE2D1_3	UBE2D1	chr10	60121273	-	ACTTACAGGCCCCATAATAG
4812	sgUBE2D1_4	UBE2D1	chr10	60121262	-	CCATAATAGTGGCTTGCCAG
4813	sgUBE2D1_5	UBE2D1	chr10	60124615	+	CCAGCTCTGACTGTATCAAA
4814	sgUBE2D1_6	UBE2D1	chr10	60121149	-	GGCTTACAGTCATCTCCCAC
4815	sgUBE2D1_7	UBE2D1	chr10	60127732	-	GTGCAATATCTGGTACTAAG
4816	sgUBE2D1_8	UBE2D1	chr10	60094963	-	CTGAATCCTCTTCAGCGCCA
4817	sgUBE2D1_9	UBE2D1	chr10	60121243	+	TGTTTGTTTCAGTGTTCCAC

4818	sgUBE2D1_10	UBE2D1	chr10	60124589	+	TCGATATTCTGAGGTCACAA
4819	sgUQCRC1_1	UQCRC1	chr3	48646673	+	GAACTGGAGCGCCTGAGCGA
4820	sgUQCRC1_2	UQCRC1	chr3	48646604	+	GGGCTGAGAGGACTGCTCGG
4821	sgUQCRC1_3	UQCRC1	chr3	48638524	+	AAGGTAGAGCATCATCACGG
4822	sgUQCRC1_4	UQCRC1	chr3	48646972	+	GCCACCACCTCACCGAGCGG
4823	sgUQCRC1_5	UQCRC1	chr3	48641805	-	GGAAGACTCACAGATTGAGA
4824	sgUQCRC1_6	UQCRC1	chr3	48640999	-	ATGGTGCTGGCAGCAGCTGG
4825	sgUQCRC1_7	UQCRC1	chr3	48638797	+	GAAGCGGCATGGAGTAAGAG
4826	sgUQCRC1_8	UQCRC1	chr3	48637959	-	ACGGAGAGTGAGGTGGCCCG
4827	sgUQCRC1_9	UQCRC1	chr3	48646687	-	GAGTACGGCAACCTTCGCTC
4828	sgUQCRC1_10	UQCRC1	chr3	48646615	+	ACTGCTCGGAGGCCACACGC
4829	sgUQCRC2_1	UQCRC2	chr16	21969871	+	TCATCTTTCAAGATAACCCG
4830	sgUQCRC2_2	UQCRC2	chr16	21974161	-	GCTTTGTCAATCTTTAGCTG
4831	sgUQCRC2_3	UQCRC2	chr16	21982920	+	GCAAAGGCCAACTACCGTGG
4832	sgUQCRC2_4	UQCRC2	chr16	21973796	+	GGAAAACATGGCTTATACTG
4833	sgUQCRC2_5	UQCRC2	chr16	21976805	+	TGGAAAAGTGACATCAGAGG
4834	sgUQCRC2_6	UQCRC2	chr16	21982881	+	CAGTTTCTCAACATGAGGGG
4835	sgUQCRC2_7	UQCRC2	chr16	21964767	-	TGAGCTCACCGAGAAAGAGC
4836	sgUQCRC2_8	UQCRC2	chr16	21968628	-	GGACTAACCTCAAGGTCCTG
4837	sgUQCRC2_9	UQCRC2	chr16	21968859	-	TGTAAGACGCAGCAAATGGG
4838	sgUQCRC2_10	UQCRC2	chr16	21973826	-	GTACTTACACATCACCCCGC
4839	sgVWF_1	VWF	chr12	6172167	-	CGTGCGCCTCAGCTACGGGG
4840	sgVWF_2	VWF	chr12	6140645	-	GAAACGGGTCACCATCCTGG
4841	sgVWF_3	VWF	chr12	6105215	-	GGGCTGCATGAAATCCATCG
4842	sgVWF_4	VWF	chr12	6232341	-	GATTCCTGCCAGATTTGCCG
4843	sgVWF_5	VWF	chr12	6230446	+	GAAAAGGCTGCATCGGGCCG
4844	sgVWF_6	VWF	chr12	6219644	-	GTGGCCAGGATCGATGGCAG
4845	sgVWF_7	VWF	chr12	6184661	+	GTGGCAGCGGGCAAACACCG
4846	sgVWF_8	VWF	chr12	6181512	+	CGAGAGAGGGAGGTGCCGGG
4847	sgVWF_9	VWF	chr12	6181499	+	GGTGTTGCAGTCTCGAGAGA
4848	sgVWF_10	VWF	chr12	6180491	-	GCATTTGCCGAAACAGCCAG
4849	CTRL00001	NA	NA	NA	NA	GGTCCATGGGTGGAGTTACG
4850	CTRL00002	NA	NA	NA	NA	GGACGCTAAACCAACGGTGC
4851	CTRL00003	NA	NA	NA	NA	GCCGTGCCGTAGCTATCCGG
4852	CTRL00004	NA	NA	NA	NA	GTCCGTTCGACAATTTCACA
4853	CTRL00005	NA	NA	NA	NA	GTACAGCTAAGTTAAACTCG
4854	CTRL00006	NA	NA	NA	NA	GCTGCTTGCGATACCAATAG
4855	CTRL00007	NA	NA	NA	NA	GTGACACAGGGAACGAGGCG
4856	CTRL00008	NA	NA	NA	NA	GGACACGGCATTTCCGCTGT
4857	CTRL00009	NA	NA	NA	NA	GCTGGTATACCGTCTTCATC
4858	CTRL00010	NA	NA	NA	NA	GGGACAAGTTGGACAGTTGC
4859	CTRL00011	NA	NA	NA	NA	GTCGGGCCGCTATATAGGCG
4860	CTRL00012	NA	NA	NA	NA	GATTTGATGTGGTGCTCACA
4861	CTRL00013	NA	NA	NA	NA	GAGCAGCGCGACAACCCATT
4862	CTRL00014	NA	NA	NA	NA	GAGTCTGACGTCGACTATCT
4863	CTRL00015	NA	NA	NA	NA	GTGCATCAACAAGTACGCGT
4864	CTRL00016	NA	NA	NA	NA	GTAACGCAGTACTTAGGGCC
4865	CTRL00017	NA	NA	NA	NA	GTAGCCGAATTCGTTACGTA
4866	CTRL00018	NA	NA	NA	NA	GGTGATCCTAGTCGACTGGC
4867	CTRL00019	NA	NA	NA	NA	GGGTGCGACAACAATTGATC
4868	CTRL00020	NA	NA	NA	NA	GCCGCACAGGGAACGCATAT
4869	CTRL00021	NA	NA	NA	NA	GTCCCGAGTCGCGCTCTCGA
4870	CTRL00022	NA	NA	NA	NA	GCTATTGTCGGCTGGATGGA
4871	CTRL00023	NA	NA	NA	NA	GGCTGGTGGCTTACTTACCT
4872	CTRL00024	NA	NA	NA	NA	GATCGGCAGGTTACCTCTGA
4873	CTRL00025	NA	NA	NA	NA	GCTAGATCTACGTAGCGTGC
4874	CTRL00026	NA	NA	NA	NA	GCGGCTAATTGTAGGTCCAT
4875	CTRL00027	NA	NA	NA	NA	GCTACTCCGCATGCCACTAC
4876	CTRL00028	NA	NA	NA	NA	GTGTCGCGAGCACCGCGATC
4877	CTRL00029	NA	NA	NA	NA	GTCGAAGTACTCGTTAAATG
4878	CTRL00030	NA	NA	NA	NA	GCGCCCTCACCGTCGTGATT

4879	CTRL00031	NA	NA	NA	NA	GACATTGTTAGTAACGACTC
4880	CTRL00032	NA	NA	NA	NA	GCCAATCGGATTGTAGAAAT
4881	CTRL00033	NA	NA	NA	NA	GCTCCGACCGTTGATGCAGC
4882	CTRL00034	NA	NA	NA	NA	GTTTGTTAACTAGATACGGG
4883	CTRL00035	NA	NA	NA	NA	GAAGGCGGAGAATCCGGGCA
4884	CTRL00036	NA	NA	NA	NA	GTATTTACTCGGTGTCGTCA
4885	CTRL00037	NA	NA	NA	NA	GATGCGAAACCTAACGCCGC
4886	CTRL00038	NA	NA	NA	NA	GTCGGAGGTAGCCGCCTGTT
4887	CTRL00039	NA	NA	NA	NA	GTTTACGCCTTCACTTGACC
4888	CTRL00040	NA	NA	NA	NA	GGTCGATTCAAGCGGTCGTT
4889	CTRL00041	NA	NA	NA	NA	GTCAATCGGCGTTCGACACA
4890	CTRL00042	NA	NA	NA	NA	GTGGGATCTCATTCGGAGGA
4891	CTRL00043	NA	NA	NA	NA	GCCGGGACCAAAGGCACTGC
4892	CTRL00044	NA	NA	NA	NA	GAGATGTACACTTTGCCAAC
4893	CTRL00045	NA	NA	NA	NA	GCACGTGTAGTCGATCTGAT
4894	CTRL00046	NA	NA	NA	NA	GGTGAGCTCCGACCCGTGGA
4895	CTRL00047	NA	NA	NA	NA	GTGACGCCGGTCCATGCGAT
4896	CTRL00048	NA	NA	NA	NA	GCAACGCGGTGGCGTAACGT
4897	CTRL00049	NA	NA	NA	NA	GGAATAAGGTTCTCGCGAGA
4898	CTRL00050	NA	NA	NA	NA	GTGCGAGCCCAACAGTAAGT
4899	CTRL00051	NA	NA	NA	NA	GAGTAACTCTCTGACCCGCC
4900	CTRL00052	NA	NA	NA	NA	GCATAACGCTAGTGTAACGA
4901	CTRL00053	NA	NA	NA	NA	GCTGGATATTACTACGTCGG
4902	CTRL00054	NA	NA	NA	NA	GGCACGCTCGGGAACCTCTG
4903	CTRL00055	NA	NA	NA	NA	GCTCGTTCGCAGTTGATCGC
4904	CTRL00056	NA	NA	NA	NA	GTATACAACATCGACGTGGG
4905	CTRL00057	NA	NA	NA	NA	GCGCGACCCTCCACTGGCTC
4906	CTRL00058	NA	NA	NA	NA	GTAATAGCTTGCGGTGAGGA
4907	CTRL00059	NA	NA	NA	NA	GGTCCGCACTTTATCAACAG
4908	CTRL00060	NA	NA	NA	NA	GCAGATTCGACGTGCCCGTG
4909	CTRL00061	NA	NA	NA	NA	GTGGTAGCCACCTGGTGCGC
4910	CTRL00062	NA	NA	NA	NA	GCCATCTAGGCCTGTGTTGC
4911	CTRL00063	NA	NA	NA	NA	GCGCCGAGCGTAACAATGTT
4912	CTRL00064	NA	NA	NA	NA	GATTTGTATGAGCTCAACTC
4913	CTRL00065	NA	NA	NA	NA	GACGGTGCCACACTTACCAG
4914	CTRL00066	NA	NA	NA	NA	GGCTATAATACGGTATCGTA
4915	CTRL00067	NA	NA	NA	NA	GTAATCGAGTCGCCTCTGCA
4916	CTRL00068	NA	NA	NA	NA	GTTTCTCGCACCACCTCAAG
4917	CTRL00069	NA	NA	NA	NA	GTGTTCGTACCCGCCACATG
4918	CTRL00070	NA	NA	NA	NA	GTTTCCCAGTGGGTTTACGA
4919	CTRL00071	NA	NA	NA	NA	GAATTGAGACTAGAACGTAG
4920	CTRL00072	NA	NA	NA	NA	GACTACGAGATATACGATCG
4921	CTRL00073	NA	NA	NA	NA	GTCGGCCAGCCAAGACGGTT
4922	CTRL00074	NA	NA	NA	NA	GCTGCCAAGACTTCACGGAA
4923	CTRL00075	NA	NA	NA	NA	GTAACGTCTACTAACGGATC
4924	CTRL00076	NA	NA	NA	NA	GCGCTAGGTCCCTGGTAAGG
4925	CTRL00077	NA	NA	NA	NA	GCGTGCCCGAACCACGGTTC
4926	CTRL00078	NA	NA	NA	NA	GCTTACCTGTTATCGACTCT
4927	CTRL00079	NA	NA	NA	NA	GTACTATTTACGCCCGGTAT
4928	CTRL00080	NA	NA	NA	NA	GCGCATCAATATGCCCGCAC
4929	CTRL00081	NA	NA	NA	NA	GTAGCATCGCGCTCGTACGC
4930	CTRL00082	NA	NA	NA	NA	GAGCCGCTTTCATGATCGAG
4931	CTRL00083	NA	NA	NA	NA	GCGGAATTACTCTAAACTTC
4932	CTRL00084	NA	NA	NA	NA	GGTTGCAATCGCCTAAGACG
4933	CTRL00085	NA	NA	NA	NA	GATCGTGGTTAACTCCGACC
4934	CTRL00086	NA	NA	NA	NA	GTGGTAGAATCCTCAATCGA
4935	CTRL00087	NA	NA	NA	NA	GAGGTCCCGCCTCCGCCCAA
4936	CTRL00088	NA	NA	NA	NA	GCGCCATGTCGCAATACCAG
4937	CTRL00089	NA	NA	NA	NA	GGGACCGAAGACTATTGATC
4938	CTRL00090	NA	NA	NA	NA	GCCGAAATATCCCGCGAATA
4939	CTRL00091	NA	NA	NA	NA	GTAAGCCGAAATACGCGGTT

4940	CTRL00092	NA	NA	NA	NA	GGCATTACCCGCCGTCCGGC
4941	CTRL00093	NA	NA	NA	NA	GGTTCCACCAGGTAGTCGAC
4942	CTRL00094	NA	NA	NA	NA	GCGGACATGACTTCTATCCA
4943	CTRL00095	NA	NA	NA	NA	GTCGTCTCACCGTGCGGCGA
4944	CTRL00096	NA	NA	NA	NA	GGCTAACGCACGCCGAGGTG
4945	CTRL00097	NA	NA	NA	NA	GATTGGCCGGCGACAGCGCT
4946	CTRL00098	NA	NA	NA	NA	GCGGTCTCTCTACTGCGACG
4947	CTRL00099	NA	NA	NA	NA	GTCCACCACCAAGCTTGTAG
4948	CTRL00100	NA	NA	NA	NA	GAACAATGGCTTCGTCGACT
4949	CTRL00101	NA	NA	NA	NA	GACCTTATGTTCGCCGCCTC
4950	CTRL00102	NA	NA	NA	NA	GAGACACCGTAACTCGAATT
4951	CTRL00103	NA	NA	NA	NA	GCTCGTAGGTCAATCGCGGA
4952	CTRL00104	NA	NA	NA	NA	GGACGGTTCCGATAACCTTA
4953	CTRL00105	NA	NA	NA	NA	GGGATCTCTCAATATGGCGC
4954	CTRL00106	NA	NA	NA	NA	GAGCGCCGTTGTTGAGAAGA
4955	CTRL00107	NA	NA	NA	NA	GCCGCCGGCCCTAACTGACC
4956	CTRL00108	NA	NA	NA	NA	GTGACTAGACCCTTACGCGG
4957	CTRL00109	NA	NA	NA	NA	GATCGGAGAGCCTGAAGTTG
4958	CTRL00110	NA	NA	NA	NA	GAGAACTCAGCGGTCTCAAG
4959	CTRL00111	NA	NA	NA	NA	GATGTCCGTTGTAGTCCTCG
4960	CTRL00112	NA	NA	NA	NA	GCCGGTCGCAATTGTTGCTA
4961	CTRL00113	NA	NA	NA	NA	GTGCCTATTCAGTAGGTCGT
4962	CTRL00114	NA	NA	NA	NA	GGTGCGGTAAGCGTGGCTAG
4963	CTRL00115	NA	NA	NA	NA	GCCATGGAGCCCAATCAATC
4964	CTRL00116	NA	NA	NA	NA	GGTGTAGTTCGGATTGATGA
4965	CTRL00117	NA	NA	NA	NA	GGGAATTGGAGACGATGCCG
4966	CTRL00118	NA	NA	NA	NA	GAGTGCCTCGTAGTTGTGGG
4967	CTRL00119	NA	NA	NA	NA	GCCTCGTTTCCACGTAATTC
4968	CTRL00120	NA	NA	NA	NA	GATTAGGTCGTTCTGTAACA
4969	CTRL00121	NA	NA	NA	NA	GCCGGTAGTGACCACGAACA
4970	CTRL00122	NA	NA	NA	NA	GTCTTCGCGAAAGCGTATTC
4971	CTRL00123	NA	NA	NA	NA	GGCCACACACGCGGCTTTAC
4972	CTRL00124	NA	NA	NA	NA	GGGCCTCATTAGTCGTCTGG
4973	CTRL00125	NA	NA	NA	NA	GAAGCCGCGAATGGCTAACT
4974	CTRL00126	NA	NA	NA	NA	GATATGCTAGACGTGACTCA
4975	CTRL00127	NA	NA	NA	NA	GTTAGCAATCGTACGGGTCA
4976	CTRL00128	NA	NA	NA	NA	GTCTCTTCGGATTCTTGGCA
4977	CTRL00129	NA	NA	NA	NA	GCGGGTCTGGAAAGGCGTAT
4978	CTRL00130	NA	NA	NA	NA	GCTCCGTAGTTCGGGACGCT
4979	CTRL00131	NA	NA	NA	NA	GACTCCGGCAAGCCAGCTCT
4980	CTRL00132	NA	NA	NA	NA	GCCCGCTCTAGCCTATAAGA
4981	CTRL00133	NA	NA	NA	NA	GGAAGATTCCCACTCATCCG
4982	CTRL00134	NA	NA	NA	NA	GTACGGATGCTGAACTCTAG
4983	CTRL00135	NA	NA	NA	NA	GAACCTACGGGCTACGATAC
4984	CTRL00136	NA	NA	NA	NA	GCGTGTGAAGGGCGATATAC
4985	CTRL00137	NA	NA	NA	NA	GCTAGGCGCGGGAGTCCGCT
4986	CTRL00138	NA	NA	NA	NA	GGACCCAGCTGCAGTAACGA
4987	CTRL00139	NA	NA	NA	NA	GATTGGTTACCTTGCGTCTC
4988	CTRL00140	NA	NA	NA	NA	GGGTTCTGCCGCTTATAATA
4989	CTRL00141	NA	NA	NA	NA	GGCAATTCAGTGCTATATCG
4990	CTRL00142	NA	NA	NA	NA	GGCGTCGGATCGATGGTTCT
4991	CTRL00143	NA	NA	NA	NA	GTTCGATCCGGCGACTCGAA
4992	CTRL00144	NA	NA	NA	NA	GTCGGTACATGTATAGACGA
4993	CTRL00145	NA	NA	NA	NA	GCGCCACGACGTCCGGCATG
4994	CTRL00146	NA	NA	NA	NA	GGCCTGCCCGCTCGGCATAT
4995	CTRL00147	NA	NA	NA	NA	GTATGCTCTATGGGATAAAC
4996	CTRL00148	NA	NA	NA	NA	GGGTCCTGCCCGTATCACCG
4997	CTRL00149	NA	NA	NA	NA	GAAGGCCAAGGAGGATGATC
4998	CTRL00150	NA	NA	NA	NA	GACGAGACCTTGCGATATAG
4999	CTRL00151	NA	NA	NA	NA	GACCACGCCCGTAATGTGCC
5000	CTRL00152	NA	NA	NA	NA	GCGAATCCCATAACGCCACA

5001	CTRL00153	NA	NA	NA	NA	GATGACGCATCGAGGAGAAG
5002	CTRL00154	NA	NA	NA	NA	GTACTGGCGTTCAACCTAAC
5003	CTRL00155	NA	NA	NA	NA	GGAAGTCAAGTCCGCACCGC
5004	CTRL00156	NA	NA	NA	NA	GCTCGCCTTAACTTGATGGT
5005	CTRL00157	NA	NA	NA	NA	GAGTCGCCACTCTCAGGTAG
5006	CTRL00158	NA	NA	NA	NA	GCGTTTCACACGATCGGAGA
5007	CTRL00159	NA	NA	NA	NA	GGGCCGCGCAGGATAAACAA
5008	CTRL00160	NA	NA	NA	NA	GCAACTCGAGCTTGCGCGGA
5009	CTRL00161	NA	NA	NA	NA	GAGGTTATGTCCCGCACCCG
5010	CTRL00162	NA	NA	NA	NA	GTACGCTGCGACACCCGGTG
5011	CTRL00163	NA	NA	NA	NA	GCTCGGCCTATGCCTATGCC
5012	CTRL00164	NA	NA	NA	NA	GCACCGAATTACGCCAACTC
5013	CTRL00165	NA	NA	NA	NA	GAGCAGACGAGTCGAGACAT
5014	CTRL00166	NA	NA	NA	NA	GTCCCAGGCATTACCATCGG
5015	CTRL00167	NA	NA	NA	NA	GAGTCTATCGTCGAGTGAGA
5016	CTRL00168	NA	NA	NA	NA	GGGTGTGGTCACGCCTTGCA
5017	CTRL00169	NA	NA	NA	NA	GCGATACACTACCGTCTCCG
5018	CTRL00170	NA	NA	NA	NA	GGTCAAACGGTATGGAGGCG
5019	CTRL00171	NA	NA	NA	NA	GGGTTGGCACGTCCAACGAC
5020	CTRL00172	NA	NA	NA	NA	GATTGTCGTTTACGATCCCA
5021	CTRL00173	NA	NA	NA	NA	GTGCTCGGATCAAATCATCA
5022	CTRL00174	NA	NA	NA	NA	GTTCGAAACCCAAAGGCGTG
5023	CTRL00175	NA	NA	NA	NA	GACCATACGAGTAACTGCAA
5024	CTRL00176	NA	NA	NA	NA	GCTCGCGCGCGCTAAAGACT
5025	CTRL00177	NA	NA	NA	NA	GAGTGGATGCGTAATACAAC
5026	CTRL00178	NA	NA	NA	NA	GCATACCCACTTATCACCGC
5027	CTRL00179	NA	NA	NA	NA	GGAGGTCGCTACAAACGGTA
5028	CTRL00180	NA	NA	NA	NA	GGGCGCCTAGCGTTTGGACG
5029	CTRL00181	NA	NA	NA	NA	GGCTGACGGTATGATCAACT
5030	CTRL00182	NA	NA	NA	NA	GGAGCAATCCGAATCGCTTC
5031	CTRL00183	NA	NA	NA	NA	GATGTACGTGCCCACCGGCA
5032	CTRL00184	NA	NA	NA	NA	GCATTCCTTGCTTCGTGCAG
5033	CTRL00185	NA	NA	NA	NA	GGGCGGTCCAAACCTGAACG
5034	CTRL00186	NA	NA	NA	NA	GCCCTACGTTGGAAACCAGT
5035	CTRL00187	NA	NA	NA	NA	GCTTAAACCCGTATACTATG
5036	CTRL00188	NA	NA	NA	NA	GAGTCCGGTTGCCCGTCGTA
5037	CTRL00189	NA	NA	NA	NA	GTACAGAACGGGTCGGTAGC
5038	CTRL00190	NA	NA	NA	NA	GATTGTGGTCTCATCGGGCG
5039	CTRL00191	NA	NA	NA	NA	GAATAATACGGGCCGTTGGA
5040	CTRL00192	NA	NA	NA	NA	GTTGATGTCGAACCCGGAGA
5041	CTRL00193	NA	NA	NA	NA	GTCAGCCCGGGTATCTCGTA
5042	CTRL00194	NA	NA	NA	NA	GTCGGTTTGAGCGTACAAAT
5043	CTRL00195	NA	NA	NA	NA	GGAGGGCTTATTCGCGCCAC
5044	CTRL00196	NA	NA	NA	NA	GGGCCTTAATAGTTCAACGC
5045	CTRL00197	NA	NA	NA	NA	GGATTCTTAGACAGTTGGGC
5046	CTRL00198	NA	NA	NA	NA	GTCGTGGCATAGAGCGACCC
5047	CTRL00199	NA	NA	NA	NA	GTTTACGATTGTCGTTCATC
5048	CTRL00200	NA	NA	NA	NA	GCACCATTGTTGAACGCTCG
5049	CTRL00201	NA	NA	NA	NA	GTCTAACCGAACAATACGAC
5050	CTRL00202	NA	NA	NA	NA	GAGACCGTTAGCCTGAGATA
5051	CTRL00203	NA	NA	NA	NA	GACGTACGATACACGGTTAG
5052	CTRL00204	NA	NA	NA	NA	GTCTCGACGAAACGTGCTGT
5053	CTRL00205	NA	NA	NA	NA	GACGTATCACCATAGAAGCG
5054	CTRL00206	NA	NA	NA	NA	GACTAGATGGGCGTTCTATC
5055	CTRL00207	NA	NA	NA	NA	GCGGGATCTATCTCATCCAA
5056	CTRL00208	NA	NA	NA	NA	GCTTGCCGGGTCTATTAATG
5057	CTRL00209	NA	NA	NA	NA	GACGTTGAGGTCCACTAGCT
5058	CTRL00210	NA	NA	NA	NA	GCTTCGAGGCGTATATGATT
5059	CTRL00211	NA	NA	NA	NA	GCTTTCGGCGCCGGTGGCCA
5060	CTRL00212	NA	NA	NA	NA	GCCACGTTGGTCACTCGGCG
5061	CTRL00213	NA	NA	NA	NA	GCGCCACGGCTGCACTATCC

5062	CTRL00214	NA	NA	NA	NA	GTATTGGTATCTCAGCAGGC
5063	CTRL00215	NA	NA	NA	NA	GGTTTCCGCGGGCCCAGAGG
5064	CTRL00216	NA	NA	NA	NA	GTGTGATCCGACACCTTTCC
5065	CTRL00217	NA	NA	NA	NA	GAACGGTGAGGCGTTAACAA
5066	CTRL00218	NA	NA	NA	NA	GCGAGCCAGCATCCCTTCGC
5067	CTRL00219	NA	NA	NA	NA	GATTGACGCGCTCTATACGA
5068	CTRL00220	NA	NA	NA	NA	GTAGAAGTCCTCCGTCAAAC
5069	CTRL00221	NA	NA	NA	NA	GTTCGGCTCGAGATGAGTAC
5070	CTRL00222	NA	NA	NA	NA	GTTATATCGGGATACTTGAC
5071	CTRL00223	NA	NA	NA	NA	GCGCTCACCATTCGTTGCTA
5072	CTRL00224	NA	NA	NA	NA	GGGATTCCGCCGGTGTTTGT
5073	CTRL00225	NA	NA	NA	NA	GGTCTTTCACGTCCCGCGTA
5074	CTRL00226	NA	NA	NA	NA	GAGGGTCGTGGGTGTTGCGT
5075	CTRL00227	NA	NA	NA	NA	GGCATGGCAGTATCCCATAG
5076	CTRL00228	NA	NA	NA	NA	GCGGTAGGGCGGTATAACCT
5077	CTRL00229	NA	NA	NA	NA	GTTTGCCTTACGCTGGAAAT
5078	CTRL00230	NA	NA	NA	NA	GAAGGAGGCGGCGGTAGTGG
5079	CTRL00231	NA	NA	NA	NA	GGTTCTAAGAACAACTCGAC
5080	CTRL00232	NA	NA	NA	NA	GAGGCACTTGCCGACCTTAC
5081	CTRL00233	NA	NA	NA	NA	GCGCCGCGTGTATCCTATGC
5082	CTRL00234	NA	NA	NA	NA	GACAGACCTCGTAGGGAGCC
5083	CTRL00235	NA	NA	NA	NA	GCGGACGGGTGCAATTAGGT
5084	CTRL00236	NA	NA	NA	NA	GAGCGAGGTCTTCGATGAAT
5085	CTRL00237	NA	NA	NA	NA	GTAGCCGTCACCTTCTGACA
5086	CTRL00238	NA	NA	NA	NA	GATGTCACGCGATGACTCGT
5087	CTRL00239	NA	NA	NA	NA	GCCGCTATCTCTATTACGAC
5088	CTRL00240	NA	NA	NA	NA	GATGTAACAACGCACAGCGA
5089	CTRL00241	NA	NA	NA	NA	GAGGCTTTATCTCTAGACGC
5090	CTRL00242	NA	NA	NA	NA	GTTCTCTCGCCAACCGGTAT
5091	CTRL00243	NA	NA	NA	NA	GGGTCAAGTCAGAGGCGTCG
5092	CTRL00244	NA	NA	NA	NA	GACACCCACTGAGCGCCGCG
5093	CTRL00245	NA	NA	NA	NA	GGACATTCGCGGCATGGCAC
5094	CTRL00246	NA	NA	NA	NA	GCATAGCAACGTTGTGACCG
5095	CTRL00247	NA	NA	NA	NA	GGACGCGTAACGGTCTTATT
5096	CTRL00248	NA	NA	NA	NA	GAGCAATGCGCCGCAACCAT
5097	CTRL00249	NA	NA	NA	NA	GCGTTCGTTCAACCACTATC
5098	CTRL00250	NA	NA	NA	NA	GTACAGGTGAGTAACCGACG
5099	CTRL00251	NA	NA	NA	NA	GCTTAAAGAGATACCGTATC
5100	CTRL00252	NA	NA	NA	NA	GCCGACCTCGGCTGTTCGCC
5101	CTRL00253	NA	NA	NA	NA	GCGGGTTCTATAGACCTCTT
5102	CTRL00254	NA	NA	NA	NA	GCACGGTTTCGCTAATGCTA
5103	CTRL00255	NA	NA	NA	NA	GATTGCGTTGGTTTACTAGG
5104	CTRL00256	NA	NA	NA	NA	GCGAATCGACGGTTGATAAT
5105	CTRL00257	NA	NA	NA	NA	GGTTAGGTAGTATTTATCCG
5106	CTRL00258	NA	NA	NA	NA	GAGCGGTTATCGGCAAACAC
5107	CTRL00259	NA	NA	NA	NA	GGTACTGCACTATTCCCGTG
5108	CTRL00260	NA	NA	NA	NA	GCCGTACTACACGGCTTTCA
5109	CTRL00261	NA	NA	NA	NA	GCTAGGCGACGAAAGCATGA
5110	CTRL00262	NA	NA	NA	NA	GGCGATACCTCCCTATGAGC
5111	CTRL00263	NA	NA	NA	NA	GAGATTGGGCGTTTACTCGG
5112	CTRL00264	NA	NA	NA	NA	GACATAGCAAACTGACGAGC
5113	CTRL00265	NA	NA	NA	NA	GCGAGTAGGTCCAGCTGGAT
5114	CTRL00266	NA	NA	NA	NA	GTTCTTTAGGCAGGCGTGGT
5115	CTRL00267	NA	NA	NA	NA	GAACGATCGACCTTTGCGTA
5116	CTRL00268	NA	NA	NA	NA	GCGACCTTATGAATACGCGA
5117	CTRL00269	NA	NA	NA	NA	GGTGTTGCTAGTACTTAGGC
5118	CTRL00270	NA	NA	NA	NA	GAATGAGTCACTGCCGCGTG
5119	CTRL00271	NA	NA	NA	NA	GCTATTTCGGAATCGTCAGG
5120	CTRL00272	NA	NA	NA	NA	GATTCTTCCGCCGCTTCGTA
5121	CTRL00273	NA	NA	NA	NA	GGTTCCGGGACCGTAACGCT
5122	CTRL00274	NA	NA	NA	NA	GGCCACCTATGACAAACTCA

5123	CTRL00275	NA	NA	NA	NA	GCAGGACGATAGTAACGTCC
5124	CTRL00276	NA	NA	NA	NA	GCTGACTCGCGCGTCGTGCT
5125	CTRL00277	NA	NA	NA	NA	GGGCACCATGCGGAAGATCG
5126	CTRL00278	NA	NA	NA	NA	GGCCGGATGCGAAGCTCGTT
5127	CTRL00279	NA	NA	NA	NA	GGCATTAACGTACTGGCCGA
5128	CTRL00280	NA	NA	NA	NA	GCGTCGGATCATTTCATACC
5129	CTRL00281	NA	NA	NA	NA	GCCGGTTCACTCATAAGTGA
5130	CTRL00282	NA	NA	NA	NA	GAACACACCAAAGCGCCGCT
5131	CTRL00283	NA	NA	NA	NA	GAGTTCCTACCGGCCCGACA
5132	CTRL00284	NA	NA	NA	NA	GTCGAAGCGAGCAAGGTCAC
5133	CTRL00285	NA	NA	NA	NA	GACTGGCCGAAGCTTTAGCG
5134	CTRL00286	NA	NA	NA	NA	GGGTGTACCAAATAGTGATC
5135	CTRL00287	NA	NA	NA	NA	GGATACGAATGACTTCGGAC
5136	CTRL00288	NA	NA	NA	NA	GCATTTGTTCCAAATACGTC
5137	CTRL00289	NA	NA	NA	NA	GGTACGAAGAACCCTATCTC
5138	CTRL00290	NA	NA	NA	NA	GCCTCGTCTGAGGTTAATGG
5139	CTRL00291	NA	NA	NA	NA	GCAGCCAGCGGTTGAACGCT
5140	CTRL00292	NA	NA	NA	NA	GACGGCACGCCGCACTTGCA
5141	CTRL00293	NA	NA	NA	NA	GGCCGTTTCGATTGTGCAAG
5142	CTRL00294	NA	NA	NA	NA	GGTATGCCAACCGAACCGTA
5143	CTRL00295	NA	NA	NA	NA	GCGACAATGAATGTCGACAT
5144	CTRL00296	NA	NA	NA	NA	GTGTAATCCAAAGACGTCAG
5145	CTRL00297	NA	NA	NA	NA	GAAGATTTACGACACTTCGT
5146	CTRL00298	NA	NA	NA	NA	GGATCATGTCTTACTATCGG
5147	CTRL00299	NA	NA	NA	NA	GGTAGGGTGTTGTCGGGTAT
5148	CTRL00300	NA	NA	NA	NA	GAATCATACGTGTCGAAATG
5149	CTRL00301	NA	NA	NA	NA	GGCCGTAATACAGGTATGTA
5150	CTRL00302	NA	NA	NA	NA	GCGAATTGTCCCATTTCCGT
5151	CTRL00303	NA	NA	NA	NA	GGACGATCTGTCAAGAATAT
5152	CTRL00304	NA	NA	NA	NA	GCCGCGGTTCAGTAACGCTA
5153	CTRL00305	NA	NA	NA	NA	GTATAATTCGTGAGCGGGTG
5154	CTRL00306	NA	NA	NA	NA	GATTCTGTAGAACAGACCGT
5155	CTRL00307	NA	NA	NA	NA	GCAGCGTTGATTGAACGAAA
5156	CTRL00308	NA	NA	NA	NA	GCAGACTTCTATACTTGTCG
5157	CTRL00309	NA	NA	NA	NA	GGTGCGCTGATTCATACGTG
5158	CTRL00310	NA	NA	NA	NA	GACTACCAGAGCGACCCGTC
5159	CTRL00311	NA	NA	NA	NA	GGAATCCATCGGGCGAATAC
5160	CTRL00312	NA	NA	NA	NA	GGCCTAGACGAGTCGCGTTG
5161	CTRL00313	NA	NA	NA	NA	GATATGGTAGCGGCGATCAA
5162	CTRL00314	NA	NA	NA	NA	GACACGCCCGCGGTAGGTGC
5163	CTRL00315	NA	NA	NA	NA	GAATAGTGGTTCGCGATCGC
5164	CTRL00316	NA	NA	NA	NA	GTTACGGGATAACCCGCCCG
5165	CTRL00317	NA	NA	NA	NA	GCTAACACGCCTACTAATGC
5166	CTRL00318	NA	NA	NA	NA	GGATTCCGCCCTAGGTGGTA
5167	CTRL00319	NA	NA	NA	NA	GACGTTGCGCCTGCCCGCAT
5168	CTRL00320	NA	NA	NA	NA	GAGAACGTGATAAGACTCGG
5169	CTRL00321	NA	NA	NA	NA	GTATCAAACACGTCCGCGCA
5170	CTRL00322	NA	NA	NA	NA	GAAGCGCATTTAGCCGAAGA
5171	CTRL00323	NA	NA	NA	NA	GATGAGGTCCCTATAGAGCG
5172	CTRL00324	NA	NA	NA	NA	GCCACAGGAGCATATTCCGA
5173	CTRL00325	NA	NA	NA	NA	GTACCGTACCTCTTGCGGTG
5174	CTRL00326	NA	NA	NA	NA	GCCGGTCCGCCGGCGAACTT
5175	CTRL00327	NA	NA	NA	NA	GAAACCGCGTCTAAATCCGA
5176	CTRL00328	NA	NA	NA	NA	GACCTGTCCGTGACGATAGG
5177	CTRL00329	NA	NA	NA	NA	GAGTGGCTCCTTTGTCGCGT
5178	CTRL00330	NA	NA	NA	NA	GGTTAGTATATTCCGGTGCT
5179	CTRL00331	NA	NA	NA	NA	GCGCAACCGTGGATAGGGTT
5180	CTRL00332	NA	NA	NA	NA	GAGTACGTGTCCATATCAAT
5181	CTRL00333	NA	NA	NA	NA	GTTAGAGCTATCGCTCGCGG
5182	CTRL00334	NA	NA	NA	NA	GCGTATTTGGTGAATGTGGA
5183	CTRL00335	NA	NA	NA	NA	GGGATCGCTCCCTTTATCGA

5184	CTRL00336	NA	NA	NA	NA	GAATACTACGGGCACGGACA
5185	CTRL00337	NA	NA	NA	NA	GATTCCGCACCGTGGATCCA
5186	CTRL00338	NA	NA	NA	NA	GCCTCAGGTCTTTCTATCCG
5187	CTRL00339	NA	NA	NA	NA	GGACCAAACATGGGCTTGCG
5188	CTRL00340	NA	NA	NA	NA	GGCACCGAGTGTTCGACGCA
5189	CTRL00341	NA	NA	NA	NA	GCTAAGGGACGGGCGCATGG
5190	CTRL00342	NA	NA	NA	NA	GTCAGCTAAATCACTACGAG
5191	CTRL00343	NA	NA	NA	NA	GGCGGCCGCTAGTATGGCAT
5192	CTRL00344	NA	NA	NA	NA	GCTCGAGCGACGTGGTAGGT
5193	CTRL00345	NA	NA	NA	NA	GCACGTATGTAACACTACGA
5194	CTRL00346	NA	NA	NA	NA	GGTTTCAGAGGACGCAAAGT
5195	CTRL00347	NA	NA	NA	NA	GGGCTTCTGGCGATCTAGCA
5196	CTRL00348	NA	NA	NA	NA	GGATAGCGGGCCTGATATCT
5197	CTRL00349	NA	NA	NA	NA	GCCCATAATGTTGAACGCAC
5198	CTRL00350	NA	NA	NA	NA	GTCCAAGCTTCGTGTGGTAA
5199	CTRL00351	NA	NA	NA	NA	GTAGACCTCTTCGCCTGCAA
5200	CTRL00352	NA	NA	NA	NA	GGATCCATACGACGTAGCGC
5201	CTRL00353	NA	NA	NA	NA	GCTGCGTAGTACAATAGCGT
5202	CTRL00354	NA	NA	NA	NA	GGCTAACACTAGCGAGTGAG
5203	CTRL00355	NA	NA	NA	NA	GCGACGCGCGTTGTTCTAGC
5204	CTRL00356	NA	NA	NA	NA	GCTACTCTGGCTCACGTTAG
5205	CTRL00357	NA	NA	NA	NA	GACAACAACGAGAGCTCGTG
5206	CTRL00358	NA	NA	NA	NA	GGCGCCTGAAGTGTCTACGT
5207	CTRL00359	NA	NA	NA	NA	GTTTCGAGGCTATGGGAGTT
5208	CTRL00360	NA	NA	NA	NA	GATTGCCCTAGGTACTCGTG
5209	CTRL00361	NA	NA	NA	NA	GGGCGCCAGTACACGACTTC
5210	CTRL00362	NA	NA	NA	NA	GACTTGTGTGTAATTCGCCC
5211	CTRL00363	NA	NA	NA	NA	GCTAGCACGCCGCCGGTTTC
5212	CTRL00364	NA	NA	NA	NA	GGCTAGACCACGCGCGATTG
5213	CTRL00365	NA	NA	NA	NA	GATATCTTTGCAGCTAGCTC
5214	CTRL00366	NA	NA	NA	NA	GTAGGCCCGAAAGGCTTAAT
5215	CTRL00367	NA	NA	NA	NA	GTTAAATCGCGCGGCGCTCC
5216	CTRL00368	NA	NA	NA	NA	GTCAGGCGACGCTCGACATA
5217	CTRL00369	NA	NA	NA	NA	GGCTGTGGACCACATCGTGA
5218	CTRL00370	NA	NA	NA	NA	GCTCGGAAGCGTGAAGGGCT
5219	CTRL00371	NA	NA	NA	NA	GCCCACGTGCTTCCTGCCGG
5220	CTRL00372	NA	NA	NA	NA	GATTACCCAGTAGCAAACGC
5221	CTRL00373	NA	NA	NA	NA	GATCTTCGCCGAGCTCGCTT
5222	CTRL00374	NA	NA	NA	NA	GTTGCCACATCCCACCAACG
5223	CTRL00375	NA	NA	NA	NA	GCGACCACTCTACGATATCA
5224	CTRL00376	NA	NA	NA	NA	GATCCATGTGAAACCGCTAC
5225	CTRL00377	NA	NA	NA	NA	GTATAGCGTAACCCGACCGG
5226	CTRL00378	NA	NA	NA	NA	GCGTATCAGGCGGTCGTAGG
5227	CTRL00379	NA	NA	NA	NA	GCGCGAGCACTACCAGAAAC
5228	CTRL00380	NA	NA	NA	NA	GACGTGGGAGCATCGGCAGT
5229	CTRL00381	NA	NA	NA	NA	GTCTTATGGTGACGATCAAA
5230	CTRL00382	NA	NA	NA	NA	GACACTGTAAACCAACGACA
5231	CTRL00383	NA	NA	NA	NA	GCGACTGAACTGGCTTCGTG
5232	CTRL00384	NA	NA	NA	NA	GCAATGACTCTGCGGATTCT
5233	CTRL00385	NA	NA	NA	NA	GATAGTTAAGGCCGACGTAC
5234	CTRL00386	NA	NA	NA	NA	GTCAGAGGCACGGATCCGTA
5235	CTRL00387	NA	NA	NA	NA	GTCGTCGTTGCCAGTTGGTG
5236	CTRL00388	NA	NA	NA	NA	GCCACTTATAGCCACTCAAT
5237	CTRL00389	NA	NA	NA	NA	GCTTTCGACTGGTTAGGTGT
5238	CTRL00390	NA	NA	NA	NA	GGGTAGGAACTGAGAAGCGT
5239	CTRL00391	NA	NA	NA	NA	GACCTGTCAGGTAGAACGAT
5240	CTRL00392	NA	NA	NA	NA	GTGTATAGTTTCGAATAGCG
5241	CTRL00393	NA	NA	NA	NA	GGTAGAAAGAACGATTATGG
5242	CTRL00394	NA	NA	NA	NA	GCTTAGCCGGCGCGTTTATC
5243	CTRL00395	NA	NA	NA	NA	GAACTATAGCGGCTTAGAAT
5244	CTRL00396	NA	NA	NA	NA	GACGTGCGTCTTCATCGAAA

5245	CTRL00397	NA	NA	NA	NA	GCGTACCAACACGTGATGTT
5246	CTRL00398	NA	NA	NA	NA	GATAGCGTGTCCACAGATGA
5247	CTRL00399	NA	NA	NA	NA	GACACAACTACGTCAGTCCG
5248	CTRL00400	NA	NA	NA	NA	GCTAGTTCAGCGACTATCGC
5249	CTRL00401	NA	NA	NA	NA	GATCCCTTAACGAATTCTAG
5250	CTRL00402	NA	NA	NA	NA	GCCCAGCTGTCAAAGTCGCC
5251	CTRL00403	NA	NA	NA	NA	GCGAGCTCACGGGCGAAGGC
5252	CTRL00404	NA	NA	NA	NA	GAAATCACTCTACGAGGCTA
5253	CTRL00405	NA	NA	NA	NA	GGACTGACGTATACGCTTGC
5254	CTRL00406	NA	NA	NA	NA	GTGATACGTCTGTACTTAGG
5255	CTRL00407	NA	NA	NA	NA	GCCGAGCACGCGTAAAGCTA
5256	CTRL00408	NA	NA	NA	NA	GCGACGCACTTTACGCAAAC
5257	CTRL00409	NA	NA	NA	NA	GCACCGAATACAGCGGCTGT
5258	CTRL00410	NA	NA	NA	NA	GCACTAATCTTGGTACACCT
5259	CTRL00411	NA	NA	NA	NA	GGTATCTGGGCAGTGGTACG
5260	CTRL00412	NA	NA	NA	NA	GTCTGATTTCAGCCGGTGGT
5261	CTRL00413	NA	NA	NA	NA	GGATATTCCCGACGAACCCT
5262	CTRL00414	NA	NA	NA	NA	GCTAGAATCACTGTGATCGT
5263	CTRL00415	NA	NA	NA	NA	GTTTCGACCGTCCCGTCGCC
5264	CTRL00416	NA	NA	NA	NA	GAACCGCAATCCTGGTGTAC
5265	CTRL00417	NA	NA	NA	NA	GCCATCCTCGCACAATACGT
5266	CTRL00418	NA	NA	NA	NA	GTGTACTGCCGATACACTCC
5267	CTRL00419	NA	NA	NA	NA	GGTGGTATCTGGTCGTCGAC
5268	CTRL00420	NA	NA	NA	NA	GCGACCTGTTTGCTAGGCGT
5269	CTRL00421	NA	NA	NA	NA	GTGATGTCCATCTCAACGAA
5270	CTRL00422	NA	NA	NA	NA	GCGCAGCGGCTGCCCAGGAT
5271	CTRL00423	NA	NA	NA	NA	GCAGTAGAAACGAGGCGAAC
5272	CTRL00424	NA	NA	NA	NA	GTGGTTATTCGGCAGATCCG
5273	CTRL00425	NA	NA	NA	NA	GCCGGCGCCAAGCTAGTACA
5274	CTRL00426	NA	NA	NA	NA	GTCGGACACTCGGAACCGCC
5275	CTRL00427	NA	NA	NA	NA	GAGATAAGCAGCGATACACG
5276	CTRL00428	NA	NA	NA	NA	GTCGGGTTCCCATCCCTTCA
5277	CTRL00429	NA	NA	NA	NA	GGTTCCCGACGCGAGTGATA
5278	CTRL00430	NA	NA	NA	NA	GTAAATCTGCGGGATATTGC
5279	CTRL00431	NA	NA	NA	NA	GCGAGGGTGGCCACGTATTT
5280	CTRL00432	NA	NA	NA	NA	GCGAACACACCGCTACGGCG
5281	CTRL00433	NA	NA	NA	NA	GATCTCGGCTTGTCGCAGCT
5282	CTRL00434	NA	NA	NA	NA	GCGTTATGGCTCCGACCGGC
5283	CTRL00435	NA	NA	NA	NA	GATATCGCCGAGGACGTGCT
5284	CTRL00436	NA	NA	NA	NA	GATGTCTGACGGCACGCGTG
5285	CTRL00437	NA	NA	NA	NA	GAAAGACCACCGTCGGACGA
5286	CTRL00438	NA	NA	NA	NA	GTTAGACGCGCAACACCGCA
5287	CTRL00439	NA	NA	NA	NA	GCAGAACCCGTACTAATATC
5288	CTRL00440	NA	NA	NA	NA	GTCCGCTAGTGCCTCCACAT
5289	CTRL00441	NA	NA	NA	NA	GGTTCTACCGGATTACCGAC
5290	CTRL00442	NA	NA	NA	NA	GCGCATATCCTGTCCGATAC
5291	CTRL00443	NA	NA	NA	NA	GTAGGCTCCCTTCGCCAATA
5292	CTRL00444	NA	NA	NA	NA	GCAGGGACACATTCGTGTTC
5293	CTRL00445	NA	NA	NA	NA	GGAGCCGATTGCTCTTAGCA
5294	CTRL00446	NA	NA	NA	NA	GCTAAACGTTCCTCTCGCAG
5295	CTRL00447	NA	NA	NA	NA	GCTAGGAAATCCCGACGTAA
5296	CTRL00448	NA	NA	NA	NA	GCTATACGCTCTAGGCCAAT
5297	CTRL00449	NA	NA	NA	NA	GCCGGCCCTTTACGAGGGCA
5298	CTRL00450	NA	NA	NA	NA	GTCACACGCTTACGGCTTAC
5299	CTRL00451	NA	NA	NA	NA	GCGTTAGGGAAGGGCGGTGT
5300	CTRL00452	NA	NA	NA	NA	GTGATCTCACCGGCACGTGA
5301	CTRL00453	NA	NA	NA	NA	GGGTCGACCATTCGGGACGA
5302	CTRL00454	NA	NA	NA	NA	GACCGCTAAGAACAGCGCTC
5303	CTRL00455	NA	NA	NA	NA	GAACGAGTCGCATCCGATGT
5304	CTRL00456	NA	NA	NA	NA	GAGGTAGATGTGTCTTGTGA
5305	CTRL00457	NA	NA	NA	NA	GTCAGCCCATTGTGTAACGG

5306	CTRL00458	NA	NA	NA	NA	GCTTGGCCGAAACAGGCGAA
5307	CTRL00459	NA	NA	NA	NA	GTCATCGCGGTTTGCGTACG
5308	CTRL00460	NA	NA	NA	NA	GCCCAAACTGTGCTACCGCA
5309	CTRL00461	NA	NA	NA	NA	GCACGGACTGGTTCCGACAT
5310	CTRL00462	NA	NA	NA	NA	GTACAGGAAGCATCCGTAGG
5311	CTRL00463	NA	NA	NA	NA	GGCCCGGATGTCTGGAGCCA
5312	CTRL00464	NA	NA	NA	NA	GAATCTCGTACCGTACACGA
5313	CTRL00465	NA	NA	NA	NA	GCGAGCTCCATCTCACCAGT
5314	CTRL00466	NA	NA	NA	NA	GCGGATATGACTATGATTGG
5315	CTRL00467	NA	NA	NA	NA	GGATGGACTCCTGCTACGCC
5316	CTRL00468	NA	NA	NA	NA	GCATCTACGGCCGATGTTCT
5317	CTRL00469	NA	NA	NA	NA	GTATGTCGTCGCTTGCCCTG
5318	CTRL00470	NA	NA	NA	NA	GCGACGCGCTGCGACCCTTC
5319	CTRL00471	NA	NA	NA	NA	GATCAAGACAACGGTCGACA
5320	CTRL00472	NA	NA	NA	NA	GGTATTGTAGGAAGCACCGC
5321	CTRL00473	NA	NA	NA	NA	GCGCTACGTAGTGGGTACAT
5322	CTRL00474	NA	NA	NA	NA	GCGCAAGGAGTCCGGACCGG
5323	CTRL00475	NA	NA	NA	NA	GCGGACCTCCCGTTTGGAGA
5324	CTRL00476	NA	NA	NA	NA	GTCTACGCTGACCCGGGTAT
5325	CTRL00477	NA	NA	NA	NA	GCGAGTGCTTACGTTAAGCT
5326	CTRL00478	NA	NA	NA	NA	GTTGCTGGCTTACTCTAAGT
5327	CTRL00479	NA	NA	NA	NA	GTTACGGAGTTTCACCTCAG
5328	CTRL00480	NA	NA	NA	NA	GAAGCTGCTGTTGCCCGACC
5329	CTRL00481	NA	NA	NA	NA	GTAACGCGCTTGGGTCAAGC
5330	CTRL00482	NA	NA	NA	NA	GACCTGATGGCCGAGATTAA
5331	CTRL00483	NA	NA	NA	NA	GGTAACCTGCTGCCTAGCGT
5332	CTRL00484	NA	NA	NA	NA	GTGAATACCCGTGACGGACA
5333	CTRL00485	NA	NA	NA	NA	GAGTGAACGACGCCTACGAG
5334	CTRL00486	NA	NA	NA	NA	GGAGGATTGGACGTTCCCTA
5335	CTRL00487	NA	NA	NA	NA	GAGGTTAGAGCTTCGTGGTC
5336	CTRL00488	NA	NA	NA	NA	GCTCAATATATCGTGACCGA
5337	CTRL00489	NA	NA	NA	NA	GAGACCTTCGTTTCCCGCGT
5338	CTRL00490	NA	NA	NA	NA	GCATGCGACCATTAGGACCC
5339	CTRL00491	NA	NA	NA	NA	GTGGCCTCGTCAAATCGGAT
5340	CTRL00492	NA	NA	NA	NA	GGATACTTCTTCGAACGTTT
5341	CTRL00493	NA	NA	NA	NA	GGCAATCGGCACGGCAAGGT
5342	CTRL00494	NA	NA	NA	NA	GGCAATACTAGGCCTTGCTG
5343	CTRL00495	NA	NA	NA	NA	GTTCGCAGCAAAGATTACCC
5344	CTRL00496	NA	NA	NA	NA	GTAGTTTATTTCCGTATCGC
5345	CTRL00497	NA	NA	NA	NA	GGTAGGAATCTAGAACTCGG
5346	CTRL00498	NA	NA	NA	NA	GACGCGTCATAGCGGATAAA
5347	CTRL00499	NA	NA	NA	NA	GGCCTAACCCACATAAGCGG
5348	CTRL00500	NA	NA	NA	NA	GTCCACGACGACCTAGAATC
5349	CTRL00501	NA	NA	NA	NA	GCAGCGGTCGGTCAACTTAT
5350	CTRL00502	NA	NA	NA	NA	GCGAATTGGCGTGGCATCGT
5351	CTRL00503	NA	NA	NA	NA	GGGAGTGGTAAGCTGGTGCG
5352	CTRL00504	NA	NA	NA	NA	GGTTCTATGTTACGCAGGGC
5353	CTRL00505	NA	NA	NA	NA	GCATGAACTCCGGCCATTAA
5354	CTRL00506	NA	NA	NA	NA	GTAAAGACCCACCATCAGAG
5355	CTRL00507	NA	NA	NA	NA	GTGTGTCGACATGATACGGG
5356	CTRL00508	NA	NA	NA	NA	GCATCTCTACCGTTCACCGC
5357	CTRL00509	NA	NA	NA	NA	GCGAATTCACTCCCTTAGCG
5358	CTRL00510	NA	NA	NA	NA	GTGTTAACTCGGGTGTGGAC
5359	CTRL00511	NA	NA	NA	NA	GAGTAGTTCGTCGTCTTCGT
5360	CTRL00512	NA	NA	NA	NA	GGTCCGATGCCCAAAGCATT
5361	CTRL00513	NA	NA	NA	NA	GCGGCAAACAACCTGCGGCT
5362	CTRL00514	NA	NA	NA	NA	GACGTCGGTCACTGAGATGC
5363	CTRL00515	NA	NA	NA	NA	GAGAAAGCCCGCTCTCGTAT
5364	CTRL00516	NA	NA	NA	NA	GACTCGCCGACCCGAAGGCA
5365	CTRL00517	NA	NA	NA	NA	GATGGATCCCTATAGCTACG
5366	CTRL00518	NA	NA	NA	NA	GATAATGTACGTATGTTGCG

5367	CTRL00519	NA	NA	NA	NA	GCCTAGCCTCGCTATGAACG
5368	CTRL00520	NA	NA	NA	NA	GGTTGGTGCTAGTGGTCGAA
5369	CTRL00521	NA	NA	NA	NA	GCAATCACATGCAACCGATA
5370	CTRL00522	NA	NA	NA	NA	GGTGCGCCCGATGGTGCATA
5371	CTRL00523	NA	NA	NA	NA	GCCCTGATCGCATACGTTGA
5372	CTRL00524	NA	NA	NA	NA	GTATTAGTATGTCCTCAGCG
5373	CTRL00525	NA	NA	NA	NA	GTTCTCCGCCCAAATCGCAT
5374	CTRL00526	NA	NA	NA	NA	GTCCCTAGCTGAAGGACCGG
5375	CTRL00527	NA	NA	NA	NA	GATGTCGACACGTTGGAATA
5376	CTRL00528	NA	NA	NA	NA	GACCATACTAGCGAGAAGCG
5377	CTRL00529	NA	NA	NA	NA	GGCGTTATAAGCATTTCGGG
5378	CTRL00530	NA	NA	NA	NA	GTATTACGCCGGACCGCCGA
5379	CTRL00531	NA	NA	NA	NA	GAATCGGGCATGGAGTCACG
5380	CTRL00532	NA	NA	NA	NA	GTCGCCGGTATGCATCTGCT
5381	CTRL00533	NA	NA	NA	NA	GATTAGTAGCCGAACCCTTT
5382	CTRL00534	NA	NA	NA	NA	GCAATATCGCCTGTATTACC
5383	CTRL00535	NA	NA	NA	NA	GGTTGCGCCCATGGCCTTTA
5384	CTRL00536	NA	NA	NA	NA	GCCCAGCGCATCCCGGTGGA
5385	CTRL00537	NA	NA	NA	NA	GTACTGGCGGTTGAAGCCGC
5386	CTRL00538	NA	NA	NA	NA	GAAAGCCTAATCTGCACAGC
5387	CTRL00539	NA	NA	NA	NA	GTAATGCCAGTGAACTACGC
5388	CTRL00540	NA	NA	NA	NA	GTTCGTCTTCGATTGGGATA
5389	CTRL00541	NA	NA	NA	NA	GTTCGTATCTCCTGACAAGA
5390	CTRL00542	NA	NA	NA	NA	GTCAGAATTTGCCACCATAA
5391	CTRL00543	NA	NA	NA	NA	GTACTGCCGGACGTAGCGTT
5392	CTRL00544	NA	NA	NA	NA	GCGGCCTTTCGCGCGTCCTT
5393	CTRL00545	NA	NA	NA	NA	GGGCTGTTCTCACTCGTAGC
5394	CTRL00546	NA	NA	NA	NA	GCTTCGGTCACTCTATCGAT
5395	CTRL00547	NA	NA	NA	NA	GCGATGGGACTTCCCTACAG
5396	CTRL00548	NA	NA	NA	NA	GCGCCGTTTGAATTGCGACG
5397	CTRL00549	NA	NA	NA	NA	GCGGCGGTGAGTCGTTTATG
5398	CTRL00550	NA	NA	NA	NA	GCATACCTCGTGAGCTGAAA
5399	CTRL00551	NA	NA	NA	NA	GCGAAGAGTGTATGCCGCAA
5400	CTRL00552	NA	NA	NA	NA	GCCCGTTCGCGCGTATGACC
5401	CTRL00553	NA	NA	NA	NA	GCCGATAACGATTCCGCCTG
5402	CTRL00554	NA	NA	NA	NA	GCTTCCACGCGCGCGGGTTA
5403	CTRL00555	NA	NA	NA	NA	GGCGCAGCCGGTTCCTTCTA
5404	CTRL00556	NA	NA	NA	NA	GAGGCGGCCATCGGCTCAAT
5405	CTRL00557	NA	NA	NA	NA	GCTCGTACGGGTTAACCTTC
5406	CTRL00558	NA	NA	NA	NA	GTTCCGGGTCGTCGTTCTTC
5407	CTRL00559	NA	NA	NA	NA	GTCGACAGAACGAACATAGG
5408	CTRL00560	NA	NA	NA	NA	GTCTCGTGCAACCTGCGCGT
5409	CTRL00561	NA	NA	NA	NA	GTACTAGCATGCCAGCCCAC
5410	CTRL00562	NA	NA	NA	NA	GTTCCACGTAGACACGCAAG
5411	CTRL00563	NA	NA	NA	NA	GCTCAACATACGCCCACACT
5412	CTRL00564	NA	NA	NA	NA	GCCCTTCACACGCAGAACTC
5413	CTRL00565	NA	NA	NA	NA	GTAAGCAGATCATGAGCGGT
5414	CTRL00566	NA	NA	NA	NA	GATGCGATCCTCTACTCACG
5415	CTRL00567	NA	NA	NA	NA	GTGACCTACTAATTTCGGAC
5416	CTRL00568	NA	NA	NA	NA	GTTTCTATAACGTCGGGTGA
5417	CTRL00569	NA	NA	NA	NA	GAGCGGATTGGCCTGCGTCC
5418	CTRL00570	NA	NA	NA	NA	GTATCAGTCCGCCGCTGTTA
5419	CTRL00571	NA	NA	NA	NA	GCTTAGCGAAGAGTTCAGGA
5420	CTRL00572	NA	NA	NA	NA	GGCCTGCGACCATCGCCAAC
5421	CTRL00573	NA	NA	NA	NA	GCGGAGTTGCTCGGCAAGTG
5422	CTRL00574	NA	NA	NA	NA	GCCCGTATACTGTTTAGGGC
5423	CTRL00575	NA	NA	NA	NA	GGAGACACGGTTGGTTCGGG
5424	CTRL00576	NA	NA	NA	NA	GATGATAATCGCGGTCGAGA
5425	CTRL00577	NA	NA	NA	NA	GGGCCTAGGATCCGCTATCG
5426	CTRL00578	NA	NA	NA	NA	GTTGCATTGACGGAGCGTAA
5427	CTRL00579	NA	NA	NA	NA	GACATCTGTCTACGTAACCC

5428	CTRL00580	NA	NA	NA	NA	GGCAAACGGACGCTAAACAG
5429	CTRL00581	NA	NA	NA	NA	GTTGCGTTTACCGTAGACGT
5430	CTRL00582	NA	NA	NA	NA	GTCGTGATGGACTTCGTAGG
5431	CTRL00583	NA	NA	NA	NA	GTCCTAGTAGATACCTCGTC
5432	CTRL00584	NA	NA	NA	NA	GCGCCAGTAGGCACATCGAC
5433	CTRL00585	NA	NA	NA	NA	GTAAGACGTTCAATGCGCTA
5434	CTRL00586	NA	NA	NA	NA	GGACGGCTATTAGATAGTTT
5435	CTRL00587	NA	NA	NA	NA	GATCGACTCACACACGACAC
5436	CTRL00588	NA	NA	NA	NA	GCCGAACATACACGACGCCT
5437	CTRL00589	NA	NA	NA	NA	GGATGACGTCGGATGTTAAT
5438	CTRL00590	NA	NA	NA	NA	GTACCTACCGCCAGCGTTCC
5439	CTRL00591	NA	NA	NA	NA	GAGGGTACACTGCCGCTCGA
5440	CTRL00592	NA	NA	NA	NA	GGTATCGTCAGATTTCCGCG
5441	CTRL00593	NA	NA	NA	NA	GTAATAAATTGCCTGCCGTT
5442	CTRL00594	NA	NA	NA	NA	GGTCGATAGGGTCACGAGAA
5443	CTRL00595	NA	NA	NA	NA	GGAGACACGAATAGCCGCCT
5444	CTRL00596	NA	NA	NA	NA	GGCTAAAGGGCAAGGCGCGC
5445	CTRL00597	NA	NA	NA	NA	GAATTAGTCGCGGTTAGGTG
5446	CTRL00598	NA	NA	NA	NA	GGATCGATCCACACTAAGGA
5447	CTRL00599	NA	NA	NA	NA	GCGATGGCGCTGCTTGCGAA
5448	CTRL00600	NA	NA	NA	NA	GCAAGCTGTCGTTACACATA
5449	CTRL00601	NA	NA	NA	NA	GATCCCGAGGGCGGCGTGTC
5450	CTRL00602	NA	NA	NA	NA	GGTAATTGCGGAATCGTCCG
5451	CTRL00603	NA	NA	NA	NA	GGCAACGCTTCATACGAAAT
5452	CTRL00604	NA	NA	NA	NA	GCCAGCAACTTTCGCCATGC
5453	CTRL00605	NA	NA	NA	NA	GCTGAAGACGGTCTCTGATT
5454	CTRL00606	NA	NA	NA	NA	GGACGGTCAATGTGTTATCG
5455	CTRL00607	NA	NA	NA	NA	GCCGGTCCGTGCAACATACT
5456	CTRL00608	NA	NA	NA	NA	GGTATGAAACACGACCGCGG
5457	CTRL00609	NA	NA	NA	NA	GGAACCGCTTTACGCATACG
5458	CTRL00610	NA	NA	NA	NA	GTGTACTTCGGACTGGTCCG
5459	CTRL00611	NA	NA	NA	NA	GAATATCTACCCAACGACTA
5460	CTRL00612	NA	NA	NA	NA	GCCGCAGGCCTCGCACAATA
5461	CTRL00613	NA	NA	NA	NA	GAGGACGAACCCGTCATCAC
5462	CTRL00614	NA	NA	NA	NA	GTTGGCCTCGTCAATAACGT
5463	CTRL00615	NA	NA	NA	NA	GCGCGCTGAACCAGCCCTTA
5464	CTRL00616	NA	NA	NA	NA	GGTTAAAGAGATTGTACGCC
5465	CTRL00617	NA	NA	NA	NA	GGGACTACTCCCGGAGATCG
5466	CTRL00618	NA	NA	NA	NA	GACTTTAACTAGGGTACGCG
5467	CTRL00619	NA	NA	NA	NA	GTAGCCCATAGCCGGAGAGG
5468	CTRL00620	NA	NA	NA	NA	GTTAGTCGATAACCAAACGG
5469	CTRL00621	NA	NA	NA	NA	GGTGGAGCACCCGGTACTCA
5470	CTRL00622	NA	NA	NA	NA	GCGTACCCGCTCATGTATGC
5471	CTRL00623	NA	NA	NA	NA	GTTATGAGCGGGCTTCTAAC
5472	CTRL00624	NA	NA	NA	NA	GGGCTAACGGATTTACCCGC
5473	CTRL00625	NA	NA	NA	NA	GACTGACACGAGTAGACCGC
5474	CTRL00626	NA	NA	NA	NA	GACGCCCAGACTGTTAGCGC
5475	CTRL00627	NA	NA	NA	NA	GCGCTCACCGTTGTAAGTTC
5476	CTRL00628	NA	NA	NA	NA	GTTGGTCACACAGCGAATAT
5477	CTRL00629	NA	NA	NA	NA	GCACGTGATGTCTCACTTCG
5478	CTRL00630	NA	NA	NA	NA	GCAGATCGTTCATTTCAGCG
5479	CTRL00631	NA	NA	NA	NA	GTTAACGCGTGCTACTGGTT
5480	CTRL00632	NA	NA	NA	NA	GTCGTCCAGAAGCTATCGAG
5481	CTRL00633	NA	NA	NA	NA	GCGAACACTTAACGGATACG
5482	CTRL00634	NA	NA	NA	NA	GGACGCAAGTTTGGTGCAAA
5483	CTRL00635	NA	NA	NA	NA	GAGACGGCTCATGCACTTAC
5484	CTRL00636	NA	NA	NA	NA	GAATGGGAGCGCGCAGTACC
5485	CTRL00637	NA	NA	NA	NA	GGGTTGCACGTAGGATATTC
5486	CTRL00638	NA	NA	NA	NA	GGACGGGTAACGAGTCGGCT
5487	CTRL00639	NA	NA	NA	NA	GCCGTAATCCGCTCCCTAAA
5488	CTRL00640	NA	NA	NA	NA	GCGTATTGTATCCGCCACCG

5489	CTRL00641	NA	NA	NA	NA	GCCGTCGAGTTTCTAGCGAT
5490	CTRL00642	NA	NA	NA	NA	GCGATCCGGAGATAGTTATG
5491	CTRL00643	NA	NA	NA	NA	GTCGATCCTCACTTCGCACT
5492	CTRL00644	NA	NA	NA	NA	GCAGCGTGGATTACTTGCCT
5493	CTRL00645	NA	NA	NA	NA	GGCACTATGTTCTGCGCGGC
5494	CTRL00646	NA	NA	NA	NA	GCAAGAGCTCGATACAACCA
5495	CTRL00647	NA	NA	NA	NA	GGCCGCACGACACTTTATAC
5496	CTRL00648	NA	NA	NA	NA	GGGTATTAGCAGTGTGACAG
5497	CTRL00649	NA	NA	NA	NA	GTCGTGAATTCGCCATCACG
5498	CTRL00650	NA	NA	NA	NA	GATTAGACGGCTCTTCCCGT
5499	CTRL00651	NA	NA	NA	NA	GGATCCGTCCTTCGCTATCA
5500	CTRL00652	NA	NA	NA	NA	GCAAGCACTATCCGAGTACG
5501	CTRL00653	NA	NA	NA	NA	GCCACTAGTCGTGTGTCACA
5502	CTRL00654	NA	NA	NA	NA	GATTATTCCCAGAGTACTCC
5503	CTRL00655	NA	NA	NA	NA	GGACTCCAGCCAGGTAGCGC
5504	CTRL00656	NA	NA	NA	NA	GAGAATTGCCGGAGGAGTAT
5505	CTRL00657	NA	NA	NA	NA	GGGTCGCTAGCGTATGGCTC
5506	CTRL00658	NA	NA	NA	NA	GCGGGTCGCAGGAACCGGGA
5507	CTRL00659	NA	NA	NA	NA	GAGATTGCTCATGGTTCCCG
5508	CTRL00660	NA	NA	NA	NA	GTGTACAGATGCCACCGTAA
5509	CTRL00661	NA	NA	NA	NA	GTCCTCTCCGGACGAGATGG
5510	CTRL00662	NA	NA	NA	NA	GGTACATAGCGGCTGTAACG
5511	CTRL00663	NA	NA	NA	NA	GAACCGAAATCTCACGGACA
5512	CTRL00664	NA	NA	NA	NA	GACGGGTATACCGATAAGCA
5513	CTRL00665	NA	NA	NA	NA	GCACTAGTTGCGACACGCTC
5514	CTRL00666	NA	NA	NA	NA	GTACACATGTGATATGTCGA
5515	CTRL00667	NA	NA	NA	NA	GGACCCACCGGACTTGTGGC
5516	CTRL00668	NA	NA	NA	NA	GCTGTTGGCTCGACGAAAGT
5517	CTRL00669	NA	NA	NA	NA	GCCCGTCTCGCATTCGGGAT
5518	CTRL00670	NA	NA	NA	NA	GGCCCGGAGGGTTTACCACA
5519	CTRL00671	NA	NA	NA	NA	GGACGGAGGAGCGCGGTCAA
5520	CTRL00672	NA	NA	NA	NA	GAGCGGTAACAAGGCTTGCG
5521	CTRL00673	NA	NA	NA	NA	GAGTAAAGCCGATTATATCG
5522	CTRL00674	NA	NA	NA	NA	GGTGCACGTCCGGCTTAAAT
5523	CTRL00675	NA	NA	NA	NA	GATCGCGAAATCCAAGCCCT
5524	CTRL00676	NA	NA	NA	NA	GCTACATTCTGACATCGCAT
5525	CTRL00677	NA	NA	NA	NA	GCTCCGTCGGGCTGTATCCG
5526	CTRL00678	NA	NA	NA	NA	GGTTCGCCCGATGGAGGTCT
5527	CTRL00679	NA	NA	NA	NA	GGCGTTATAGCTCGCCGTTA
5528	CTRL00680	NA	NA	NA	NA	GCTCTTATTGTCCTCGAGTT
5529	CTRL00681	NA	NA	NA	NA	GTTGCTACCGGATCGTCGTC
5530	CTRL00682	NA	NA	NA	NA	GGGCTCAAGGGTGTTCGAAC
5531	CTRL00683	NA	NA	NA	NA	GATAGACGGCTATCCGACGG
5532	CTRL00684	NA	NA	NA	NA	GACATCCGTTGTCTTATCTA
5533	CTRL00685	NA	NA	NA	NA	GCGATGACATACTGCTCGTG
5534	CTRL00686	NA	NA	NA	NA	GGGCGTCCTTTAGAGTGAGA
5535	CTRL00687	NA	NA	NA	NA	GTATCTGAGGCTTTAGACGG
5536	CTRL00688	NA	NA	NA	NA	GACCCTGAACAACCTAGGAT
5537	CTRL00689	NA	NA	NA	NA	GGACCGGCACCTTATTATAT
5538	CTRL00690	NA	NA	NA	NA	GATTCCGACTATCTCGACCA
5539	CTRL00691	NA	NA	NA	NA	GTACCTCCACCTACCCTAGT
5540	CTRL00692	NA	NA	NA	NA	GCGGCTTCCGGGCTTTAATG
5541	CTRL00693	NA	NA	NA	NA	GATAGCCAGTGACGGATGGC
5542	CTRL00694	NA	NA	NA	NA	GTCGCTCAATTCTCGGTAGG
5543	CTRL00695	NA	NA	NA	NA	GTACGCGTACGGTTCCGTCG
5544	CTRL00696	NA	NA	NA	NA	GAATGCTGAGTACGGTCTGT
5545	CTRL00697	NA	NA	NA	NA	GCGATCTGCCGTATCATCAT
5546	CTRL00698	NA	NA	NA	NA	GGTCTATGGCCACCCGCTAC
5547	CTRL00699	NA	NA	NA	NA	GAATCGGAATCGTTCCAGGC
5548	CTRL00700	NA	NA	NA	NA	GAGTATCACCTGAGCCGATT
5549	CTRL00701	NA	NA	NA	NA	GACGATCTTCCGCGCGAGAG

5550	CTRL00702	NA	NA	NA	NA	GGCTTTCGCGGTTCACCAGG
5551	CTRL00703	NA	NA	NA	NA	GACATCAACCATCTGCGTGA
5552	CTRL00704	NA	NA	NA	NA	GCCCGGGACCGTGTCGATGA
5553	CTRL00705	NA	NA	NA	NA	GTGTGATCGTGGAATCCTAC
5554	CTRL00706	NA	NA	NA	NA	GTGGTTGTTTGTGGAGCCGA
5555	CTRL00707	NA	NA	NA	NA	GCACGCCGATAGCACCCTGA
5556	CTRL00708	NA	NA	NA	NA	GCTAGACTAACGTTTGTGTC
5557	CTRL00709	NA	NA	NA	NA	GCACGTCGCCTTTGCTGCTG
5558	CTRL00710	NA	NA	NA	NA	GGGACGTAAATTCTACCTGC
5559	CTRL00711	NA	NA	NA	NA	GCGCGACAACACGACCAGGA
5560	CTRL00712	NA	NA	NA	NA	GTCAAGCCGAACTTGTTAGC
5561	CTRL00713	NA	NA	NA	NA	GTACGCAAACGCCCTTTGGG
5562	CTRL00714	NA	NA	NA	NA	GAGTTGTGCCCGCTCGACTT
5563	CTRL00715	NA	NA	NA	NA	GGTTCGCGCAAAGCTAGTAA
5564	CTRL00716	NA	NA	NA	NA	GCGCATCCTACGGGCTCCCT
5565	CTRL00717	NA	NA	NA	NA	GCCAGAGTTCTTGCACCCGA
5566	CTRL00718	NA	NA	NA	NA	GGATAACGGGATGACTGCGC
5567	CTRL00719	NA	NA	NA	NA	GCGCACAATCCCATCTCCAA
5568	CTRL00720	NA	NA	NA	NA	GTATTCTTGACTATTGCACG
5569	CTRL00721	NA	NA	NA	NA	GTACCTACGTGTTGTAAAGG
5570	CTRL00722	NA	NA	NA	NA	GTCTTAGCTGCGTTGCATGG
5571	CTRL00723	NA	NA	NA	NA	GCGATACACTCCTAGCTATG
5572	CTRL00724	NA	NA	NA	NA	GTATTACGCTAGACTTCACG
5573	CTRL00725	NA	NA	NA	NA	GACGCTGTTCGCGCTTCGAA
5574	CTRL00726	NA	NA	NA	NA	GTAAGGACAGAAGTAGATCA
5575	CTRL00727	NA	NA	NA	NA	GCGAACGCGATGTGTCAATC
5576	CTRL00728	NA	NA	NA	NA	GATCTATCTAAGCGGCGGAA
5577	CTRL00729	NA	NA	NA	NA	GGGTACCTCGCGAGGTCTCT
5578	CTRL00730	NA	NA	NA	NA	GACCGACGTGTCTATTCACT
5579	CTRL00731	NA	NA	NA	NA	GCAATCGCATGCAGCGGTTA
5580	CTRL00732	NA	NA	NA	NA	GTTCAAGCATAAAGGTCGCG
5581	CTRL00733	NA	NA	NA	NA	GGGTGGTCCGGAGGTTCACT
5582	CTRL00734	NA	NA	NA	NA	GATCTGAGGATACAGACGGA
5583	CTRL00735	NA	NA	NA	NA	GACCCTATCCAACTTGGCAC
5584	CTRL00736	NA	NA	NA	NA	GGAGTAATCCTGTCGCAAGC
5585	CTRL00737	NA	NA	NA	NA	GAAGATGTGTTGAATTCTCG
5586	CTRL00738	NA	NA	NA	NA	GTCGAATCGTCGATAGGTTC
5587	CTRL00739	NA	NA	NA	NA	GCCGATCAAGGTCTCTCTTC
5588	CTRL00740	NA	NA	NA	NA	GTATGCCGGCACGTTACGTT
5589	CTRL00741	NA	NA	NA	NA	GGTGGATGTGATATGCGACG
5590	CTRL00742	NA	NA	NA	NA	GAATAGCTGACAACTTGACC
5591	CTRL00743	NA	NA	NA	NA	GCTCCCGCATATCGGTTAGC
5592	CTRL00744	NA	NA	NA	NA	GTTTAAACGCTCCACAGGTT
5593	CTRL00745	NA	NA	NA	NA	GGTTCCACTGCGGCCTCGAT
5594	CTRL00746	NA	NA	NA	NA	GTCTTATTGAACGGAGATGG
5595	CTRL00747	NA	NA	NA	NA	GTCCGGTCTTTACCGAAGAT
5596	CTRL00748	NA	NA	NA	NA	GCCTCCGCAAGACGTGGTAT
5597	CTRL00749	NA	NA	NA	NA	GGGCAAACCGCTCCGATGAA
5598	CTRL00750	NA	NA	NA	NA	GCTGGACCAAGCCTGCGATT
5599	CTRL00751	NA	NA	NA	NA	GAGTTAGAAAGTCGACCTAA
5600	CTRL00752	NA	NA	NA	NA	GCGGGATCGTCATCTCACTA
5601	CTRL00753	NA	NA	NA	NA	GGAAACATCTGGTAGCCTCG
5602	CTRL00754	NA	NA	NA	NA	GTCATGTAACCGCTCACGGG
5603	CTRL00755	NA	NA	NA	NA	GCTGACGCTCGTACTGGTCG
5604	CTRL00756	NA	NA	NA	NA	GTGTCACGCAACGCCTATGA
5605	CTRL00757	NA	NA	NA	NA	GTGTAGTTAGAGCTCGCGGA
5606	CTRL00758	NA	NA	NA	NA	GTTCGCCCGGAGCTCAGGTC
5607	CTRL00759	NA	NA	NA	NA	GACAAGCGCGACGTATCCTT
5608	CTRL00760	NA	NA	NA	NA	GGACGCCCTTGTGAACTGAT
5609	CTRL00761	NA	NA	NA	NA	GTAGCTACTGCGTGGAGCTA
5610	CTRL00762	NA	NA	NA	NA	GATTCGCCGCGCCAATCCAA

5611	CTRL00763	NA	NA	NA	NA	GGCTCTCTGACCTGTTCGGA
5612	CTRL00764	NA	NA	NA	NA	GACTTTATGACGAAGGCCCG
5613	CTRL00765	NA	NA	NA	NA	GGTCACCACGCCTTGGCATC
5614	CTRL00766	NA	NA	NA	NA	GCGCCGTACAGTAGCGCACT
5615	CTRL00767	NA	NA	NA	NA	GCCGAAACTAGCGAAGCGCC
5616	CTRL00768	NA	NA	NA	NA	GAGCGTTACGATTCGCTGAC
5617	CTRL00769	NA	NA	NA	NA	GGCTCTTAGTATACCAGCGG
5618	CTRL00770	NA	NA	NA	NA	GGAGCATTGCTGATAGGTCC
5619	CTRL00771	NA	NA	NA	NA	GCTGTGGGACATAGATCGCG
5620	CTRL00772	NA	NA	NA	NA	GCTATTTAGCTTGTTGTCGA
5621	CTRL00773	NA	NA	NA	NA	GGCTAACGTGTGAGCACTGG
5622	CTRL00774	NA	NA	NA	NA	GCCCATGTCACCGCTTGAAG
5623	CTRL00775	NA	NA	NA	NA	GTAAGGCGTCTTCGCGCGAA
5624	CTRL00776	NA	NA	NA	NA	GTACCGTTGACCGACCAGGC
5625	CTRL00777	NA	NA	NA	NA	GTTATTCCAAGTGTAGTCGG
5626	CTRL00778	NA	NA	NA	NA	GACTGGTGAAGTAGGCGCCG
5627	CTRL00779	NA	NA	NA	NA	GTATCAATAGCAAGCGTCGC
5628	CTRL00780	NA	NA	NA	NA	GATTCGAAAGGGACGGGCGC
5629	CTRL00781	NA	NA	NA	NA	GCCTCATCCACGAAAGATGC
5630	CTRL00782	NA	NA	NA	NA	GTGCCATCGAGCTCGTAGGA
5631	CTRL00783	NA	NA	NA	NA	GCGGCGATAGAAGCTATATC
5632	CTRL00784	NA	NA	NA	NA	GTCGCCACCGGTTGGTTGGT
5633	CTRL00785	NA	NA	NA	NA	GTGCCGTAGGGTAAACTGTC
5634	CTRL00786	NA	NA	NA	NA	GTCCGCTGTTGCACGAGAGT
5635	CTRL00787	NA	NA	NA	NA	GCTGGGATATGTCACCCGCT
5636	CTRL00788	NA	NA	NA	NA	GATTGATAAGGCTAGTTGCG
5637	CTRL00789	NA	NA	NA	NA	GGCAAACTTCGCTAGGCGAT
5638	CTRL00790	NA	NA	NA	NA	GGGACCTACGCATAGACGTG
5639	CTRL00791	NA	NA	NA	NA	GTGACGGTTATTGTCTGTTA
5640	CTRL00792	NA	NA	NA	NA	GCCCTTGTGCGTCGGTCGTT
5641	CTRL00793	NA	NA	NA	NA	GACCGATCGGCGAATGCGAT
5642	CTRL00794	NA	NA	NA	NA	GTTACGCCCGAATTTGCAAA
5643	CTRL00795	NA	NA	NA	NA	GCGCCCACGCTCTAAGGATG
5644	CTRL00796	NA	NA	NA	NA	GTAACGGCAGCTGTGCATGG
5645	CTRL00797	NA	NA	NA	NA	GCAGCATCGTCGCTCCGGCA
5646	CTRL00798	NA	NA	NA	NA	GCGCATGACGTGAGAATGAT
5647	CTRL00799	NA	NA	NA	NA	GACTTTAGGAGGTAGTTCGT
5648	CTRL00800	NA	NA	NA	NA	GGAGAAGAATTCGCCGTTGC
5649	CTRL00801	NA	NA	NA	NA	GAGCCAGAACGTTTCTAACG
5650	CTRL00802	NA	NA	NA	NA	GCGGTCCGGACTCATTCATA
5651	CTRL00803	NA	NA	NA	NA	GTCATACGGTTGGTTCCGAA
5652	CTRL00804	NA	NA	NA	NA	GCGCCGCCCAAATAAATCTG
5653	CTRL00805	NA	NA	NA	NA	GTAGTCTCCTTGGTAATCGA
5654	CTRL00806	NA	NA	NA	NA	GTTTACGCTACGACTTCGGC
5655	CTRL00807	NA	NA	NA	NA	GGGCGTCACCTGCTACACGG
5656	CTRL00808	NA	NA	NA	NA	GTACCGCCTTTCTGTCCCAC
5657	CTRL00809	NA	NA	NA	NA	GGAGTAAGATTCGAAGTCGT
5658	CTRL00810	NA	NA	NA	NA	GTGCTCTTTGTACCGACAGG
5659	CTRL00811	NA	NA	NA	NA	GCCTAATGACCGACATTAAG
5660	CTRL00812	NA	NA	NA	NA	GTATTCATATCTATCGCCGG
5661	CTRL00813	NA	NA	NA	NA	GTAGCCAACAGCTCGAATAG
5662	CTRL00814	NA	NA	NA	NA	GTGGGTCGGAATGAAAGTGG
5663	CTRL00815	NA	NA	NA	NA	GATTATAGTCGCCGACTAGA
5664	CTRL00816	NA	NA	NA	NA	GCCATTCGGTTAACCTAAAG
5665	CTRL00817	NA	NA	NA	NA	GAACCACTTCCGCATTGATC
5666	CTRL00818	NA	NA	NA	NA	GGCCATTATACCGTTGTTGT
5667	CTRL00819	NA	NA	NA	NA	GGTCGTTTAACGGACCTATG
5668	CTRL00820	NA	NA	NA	NA	GTAGAGTTAGCGGTCAGATT
5669	CTRL00821	NA	NA	NA	NA	GTTCGTATCGGCTGCGTCCA
5670	CTRL00822	NA	NA	NA	NA	GCGGTGTGCTGAAGACACTT
5671	CTRL00823	NA	NA	NA	NA	GTTCCGGAATGCGCCACGAT

5672	CTRL00824	NA	NA	NA	NA	GCCGATGGCGATTCTGTATT
5673	CTRL00825	NA	NA	NA	NA	GCCGCGGTTGGTCAATAGTT
5674	CTRL00826	NA	NA	NA	NA	GTTAGCCGCCCAAGTCGATC
5675	CTRL00827	NA	NA	NA	NA	GCTGTCACGCAACGGCATGT
5676	CTRL00828	NA	NA	NA	NA	GTTCGACTGCTCGTTCTGTC
5677	CTRL00829	NA	NA	NA	NA	GCCCATTTCGTGTAGCACGC
5678	CTRL00830	NA	NA	NA	NA	GGCATACATCCAAACCGACC
5679	CTRL00831	NA	NA	NA	NA	GCGCCCTACATGCGGCTGTA
5680	CTRL00832	NA	NA	NA	NA	GTACCCAGACTACATTATTG
5681	CTRL00833	NA	NA	NA	NA	GACACCTTGTATCCGCGATG
5682	CTRL00834	NA	NA	NA	NA	GCGACAGGTTTAACCCGCAC
5683	CTRL00835	NA	NA	NA	NA	GGCTATAGACGTCGGTTCGG
5684	CTRL00836	NA	NA	NA	NA	GATCTAACATCAATTCTGGG
5685	CTRL00837	NA	NA	NA	NA	GTCGCCCGCTCCTGGGAATG
5686	CTRL00838	NA	NA	NA	NA	GTTGACGACAAGCAATTCAG
5687	CTRL00839	NA	NA	NA	NA	GACCCGGCCAGCCTCGTAGA
5688	CTRL00840	NA	NA	NA	NA	GCACGGTAGAAGATTGACAC
5689	CTRL00841	NA	NA	NA	NA	GGATCATTCGGACGGTAGGA
5690	CTRL00842	NA	NA	NA	NA	GAACACGGCTCACGTGTCCT
5691	CTRL00843	NA	NA	NA	NA	GAGCAGAGAGTTAACGCCGC
5692	CTRL00844	NA	NA	NA	NA	GGCGTGGGCCGGACGAGTAT
5693	CTRL00845	NA	NA	NA	NA	GCGTCGGACGGATCTCTACA
5694	CTRL00846	NA	NA	NA	NA	GAACTATACATGGACGATGC
5695	CTRL00847	NA	NA	NA	NA	GATTTCCCATATTAACGGCG
5696	CTRL00848	NA	NA	NA	NA	GTACGGCAGATAGTCGGTGG
5697	CTRL00849	NA	NA	NA	NA	GCGCTCGAGACATCTGCAGT
5698	CTRL00850	NA	NA	NA	NA	GTGCCTTATTATGTCCGGGC
5699	CTRL00851	NA	NA	NA	NA	GGTCAGGTTACGGTCAGATC
5700	CTRL00852	NA	NA	NA	NA	GAATTAGCGGATTACTACGG
5701	CTRL00853	NA	NA	NA	NA	GGAGTCGGTGCTTACACGTT
5702	CTRL00854	NA	NA	NA	NA	GCGAGCAACAAACAACACCG
5703	CTRL00855	NA	NA	NA	NA	GCATAGTAATAACCCGAGTT
5704	CTRL00856	NA	NA	NA	NA	GTACGCAGTAGCTCCGGCAC
5705	CTRL00857	NA	NA	NA	NA	GTGTTCGCACTCATCTGGCG
5706	CTRL00858	NA	NA	NA	NA	GTGAACGAGGAGGGTGGCGA
5707	CTRL00859	NA	NA	NA	NA	GGCGAGCAACCAACCGCCCA
5708	CTRL00860	NA	NA	NA	NA	GGAACCACGTTGCGGCCGGG
5709	CTRL00861	NA	NA	NA	NA	GATAGATTATCGGAAGTCTC
5710	CTRL00862	NA	NA	NA	NA	GAGTTGACCGGTCCCTACTG
5711	CTRL00863	NA	NA	NA	NA	GACTGCCTACTTGATGCCGT
5712	CTRL00864	NA	NA	NA	NA	GCTGCCGAACACTGAAGTGG
5713	CTRL00865	NA	NA	NA	NA	GCCTAGTACGATATATTGGC
5714	CTRL00866	NA	NA	NA	NA	GACCGTACCGGGTTACGAAC
5715	CTRL00867	NA	NA	NA	NA	GCTGCCTTATAATGTGACGA
5716	CTRL00868	NA	NA	NA	NA	GGCGTGCGCTGGGTTAGCCT
5717	CTRL00869	NA	NA	NA	NA	GACGTGCCCGGATTCCCACG
5718	CTRL00870	NA	NA	NA	NA	GACCCAACACTACCGGAGGG
5719	CTRL00871	NA	NA	NA	NA	GCCATACAACTTGTCGTTGT
5720	CTRL00872	NA	NA	NA	NA	GCGTGGTCCTGGTGACGCGA
5721	CTRL00873	NA	NA	NA	NA	GCTAGCTGCGCTGGCTACGC
5722	CTRL00874	NA	NA	NA	NA	GACTGACTCGCGCAGCGACC
5723	CTRL00875	NA	NA	NA	NA	GGTATCCTAAGGAGCGCCCT
5724	CTRL00876	NA	NA	NA	NA	GGTTCGGATGTCGTCGTCCC
5725	CTRL00877	NA	NA	NA	NA	GCATGTCGCGAAAGCCCATA
5726	CTRL00878	NA	NA	NA	NA	GCTATTATCGGCGAACTCAA
5727	CTRL00879	NA	NA	NA	NA	GATAGAATGGTCGGTCATGC
5728	CTRL00880	NA	NA	NA	NA	GAGCTAACAGGATACCGGCT
5729	CTRL00881	NA	NA	NA	NA	GGTTAGTCGAAGTCGGAGGT
5730	CTRL00882	NA	NA	NA	NA	GTGGTATTCATGACAACGCG
5731	CTRL00883	NA	NA	NA	NA	GGGTGGCGAGAGTTACCGCT
5732	CTRL00884	NA	NA	NA	NA	GGGCGCCCGCATTTATCGAG

5733	CTRL00885	NA	NA	NA	NA	GACCACTGAGATACACCGCC
5734	CTRL00886	NA	NA	NA	NA	GAAGCAAGTAGGTCGACAGC
5735	CTRL00887	NA	NA	NA	NA	GACGTCGTTAATCCGAGGAG
5736	CTRL00888	NA	NA	NA	NA	GACTCATTGGAACATACATG
5737	CTRL00889	NA	NA	NA	NA	GTACAGACCTAGATATAGGC
5738	CTRL00890	NA	NA	NA	NA	GCGATAAAGAGAACGGGCAG
5739	CTRL00891	NA	NA	NA	NA	GCCATCGAAGGTCTTAGATG
5740	CTRL00892	NA	NA	NA	NA	GAACGGCCACCGTATTTAAT
5741	CTRL00893	NA	NA	NA	NA	GGTTCGGAACTGACGATGCT
5742	CTRL00894	NA	NA	NA	NA	GGCGGTTTGTACCTCGTGTG
5743	CTRL00895	NA	NA	NA	NA	GAGCCCGACTAAAGAGGCCG
5744	CTRL00896	NA	NA	NA	NA	GATGCTGATGATACGGGATC
5745	CTRL00897	NA	NA	NA	NA	GTCCAAGCTGAATGCGAGCA
5746	CTRL00898	NA	NA	NA	NA	GATGCTCGCTCGGAGACGCC
5747	CTRL00899	NA	NA	NA	NA	GTTACTCCCAACCGACGCAA
5748	CTRL00900	NA	NA	NA	NA	GCTTGACTACGGCCCGCACT
5749	CTRL00901	NA	NA	NA	NA	GACAAGGGATTCGACGCGTA
5750	CTRL00902	NA	NA	NA	NA	GGTCTCCGGTACCGGTATCA
5751	CTRL00903	NA	NA	NA	NA	GGTACGTTTGTCATATGCAC
5752	CTRL00904	NA	NA	NA	NA	GGCGATACTGTCGTTATGGC
5753	CTRL00905	NA	NA	NA	NA	GCTAAGCAAACACCTGTCGT
5754	CTRL00906	NA	NA	NA	NA	GCGAGCGATGGGACCGTAAC
5755	CTRL00907	NA	NA	NA	NA	GCGATAACTTCCTCACCACT
5756	CTRL00908	NA	NA	NA	NA	GATAACGCTACAGTGGTGTC
5757	CTRL00909	NA	NA	NA	NA	GTTCCGAAGGGCACACGGTG
5758	CTRL00910	NA	NA	NA	NA	GCTGTTGCAACAACGAACAT
5759	CTRL00911	NA	NA	NA	NA	GGGTGCTGCGGTACGATCCG
5760	CTRL00912	NA	NA	NA	NA	GTTAATGGAGATGAAGCTAC
5761	CTRL00913	NA	NA	NA	NA	GGACCTGTTGCAAACTCCGT
5762	CTRL00914	NA	NA	NA	NA	GGAGCAGACGTTGAGATGAT
5763	CTRL00915	NA	NA	NA	NA	GTGTCGTCAGGCAGTCATCG
5764	CTRL00916	NA	NA	NA	NA	GAGACACGTCCCGTTACAAT
5765	CTRL00917	NA	NA	NA	NA	GACGAGTACAGCGTCGCGGC
5766	CTRL00918	NA	NA	NA	NA	GGTACAAGCCGTACGTGCGC
5767	CTRL00919	NA	NA	NA	NA	GAATCGAAGGAATAAGGAGA
5768	CTRL00920	NA	NA	NA	NA	GACTTCGAACCTACGTCACA
5769	CTRL00921	NA	NA	NA	NA	GCGGCGTATAGATGCCCATA
5770	CTRL00922	NA	NA	NA	NA	GATCGCAGGGTTAGAGTATA
5771	CTRL00923	NA	NA	NA	NA	GGCCTGTCGAAGGTTCTCGC
5772	CTRL00924	NA	NA	NA	NA	GGTGGCAACAAGAAGGGTCG
5773	CTRL00925	NA	NA	NA	NA	GTGACCCGTAGATCTCGCAG
5774	CTRL00926	NA	NA	NA	NA	GACTACGATACGTGTGCGAT
5775	CTRL00927	NA	NA	NA	NA	GTGCGGTAAGGCAGTCCCAT
5776	CTRL00928	NA	NA	NA	NA	GCCAGCATGCTACCTCGAAA
5777	CTRL00929	NA	NA	NA	NA	GCTGACTCCGTGGGATATAC
5778	CTRL00930	NA	NA	NA	NA	GACGTTGCCCGCCTTGCCAA
5779	CTRL00931	NA	NA	NA	NA	GCCTCTTTGCATACAAAGCG
5780	CTRL00932	NA	NA	NA	NA	GCCGCCTACGAGATTCTATG
5781	CTRL00933	NA	NA	NA	NA	GTAGCACATGGCGACTCTTA
5782	CTRL00934	NA	NA	NA	NA	GGTCCCGGTAATATGCGAAG
5783	CTRL00935	NA	NA	NA	NA	GTCCGGAACTTTGGGATACG
5784	CTRL00936	NA	NA	NA	NA	GGCTCAACGGACTGTCACGG
5785	CTRL00937	NA	NA	NA	NA	GTTAAGGAAGGTCGCTGCGA
5786	CTRL00938	NA	NA	NA	NA	GTTAGCCATCAGGTCGGAGC
5787	CTRL00939	NA	NA	NA	NA	GGAATGCAAACGGCTGCGAC
5788	CTRL00940	NA	NA	NA	NA	GAACTTGACGACGTAAGAGA
5789	CTRL00941	NA	NA	NA	NA	GATATCGGACCGACCGATAC
5790	CTRL00942	NA	NA	NA	NA	GAAGGGCGAGTACAGGCGAT
5791	CTRL00943	NA	NA	NA	NA	GGGTTGCAGCCTGATGTATA
5792	CTRL00944	NA	NA	NA	NA	GCTTGTTGACTCGCAGCGAA
5793	CTRL00945	NA	NA	NA	NA	GCTCGGAACCCTCTTCCGAA

5794	CTRL00946	NA	NA	NA	NA	GACCAGTCCGCGCTACGAGT
5795	CTRL00947	NA	NA	NA	NA	GCTGCCGGGACCGCTCTATC
5796	CTRL00948	NA	NA	NA	NA	GCGACTAAGACCGGCTCACG
5797	CTRL00949	NA	NA	NA	NA	GACCGTCTCGCAAACGTATA
5798	CTRL00950	NA	NA	NA	NA	GCATGTCGAAACGGGCGCAC
5799	CTRL00951	NA	NA	NA	NA	GGGCCGCCCAATGAAGCGAT
5800	CTRL00952	NA	NA	NA	NA	GCTGAAGCACTAACGCATAA
5801	CTRL00953	NA	NA	NA	NA	GGCGTTGTGATCGCACCTGC
5802	CTRL00954	NA	NA	NA	NA	GTATATACCGGACAGCCCAT
5803	CTRL00955	NA	NA	NA	NA	GCAGCGCGGCTTAGTTATGG
5804	CTRL00956	NA	NA	NA	NA	GATTGAACGGCCAATTCGAC
5805	CTRL00957	NA	NA	NA	NA	GGGATACATCTGGATCGGCG
5806	CTRL00958	NA	NA	NA	NA	GGGATGGGAACCTGTACCGC
5807	CTRL00959	NA	NA	NA	NA	GGCGCGAAGCGCTTATCGGC
5808	CTRL00960	NA	NA	NA	NA	GTATCCGTGCGCTGGTACAG
5809	CTRL00961	NA	NA	NA	NA	GGAAAGCCTACGCCTGCGCC
5810	CTRL00962	NA	NA	NA	NA	GTGTGCTCGGACTTTCGTAG
5811	CTRL00963	NA	NA	NA	NA	GGTTAACTATGCCACTAGTA
5812	CTRL00964	NA	NA	NA	NA	GGGCCTGCTTACCGTACCGT
5813	CTRL00965	NA	NA	NA	NA	GAACTGGTTCATACACTGAG
5814	CTRL00966	NA	NA	NA	NA	GCGACGGTGACTTATTTCGA
5815	CTRL00967	NA	NA	NA	NA	GTGCCGAACCCTTTCCGTCG
5816	CTRL00968	NA	NA	NA	NA	GGCAACCCAAACTATACGAA
5817	CTRL00969	NA	NA	NA	NA	GACCCGGGCACCAACTGGTT
5818	CTRL00970	NA	NA	NA	NA	GACGGTAAATCCCGGACGGA
5819	CTRL00971	NA	NA	NA	NA	GGCCAATATCGCGAATGTAC
5820	CTRL00972	NA	NA	NA	NA	GCGGCGGCGCGAGTTATAAG
5821	CTRL00973	NA	NA	NA	NA	GCGATCCCATTCCGTAGAAT
5822	CTRL00974	NA	NA	NA	NA	GTCGCCACGGTCAGACTACG
5823	CTRL00975	NA	NA	NA	NA	GTCGTGTAGGACTGGAAGAC
5824	CTRL00976	NA	NA	NA	NA	GACCGACCGTAAGGTTCTTT
5825	CTRL00977	NA	NA	NA	NA	GACAAGTTTCGGCCCTAAGC
5826	CTRL00978	NA	NA	NA	NA	GATCATCCGCTCCTACATCA
5827	CTRL00979	NA	NA	NA	NA	GAACTGCGGTGGAAACACGA
5828	CTRL00980	NA	NA	NA	NA	GACGCCTTGACCGGTCTGCA
5829	CTRL00981	NA	NA	NA	NA	GGGCTTATGCGGACTAAGGT
5830	CTRL00982	NA	NA	NA	NA	GTAAACCCGGTTAGACCTTA
5831	CTRL00983	NA	NA	NA	NA	GTAAAGCGTAGAACCAAGCG
5832	CTRL00984	NA	NA	NA	NA	GTCGGCTATACTCGGACGGT
5833	CTRL00985	NA	NA	NA	NA	GCGTAAAGAGTACGAAGTCA
5834	CTRL00986	NA	NA	NA	NA	GCCGAGTAGGAAATTCGGGT
5835	CTRL00987	NA	NA	NA	NA	GCTTCCCGACGGGAATATAC
5836	CTRL00988	NA	NA	NA	NA	GCTTTGATTATAGCCGAGAT
5837	CTRL00989	NA	NA	NA	NA	GCGGAGCGTCTATACAATTG
5838	CTRL00990	NA	NA	NA	NA	GGAACCACCTTCCGCCGAAC
5839	CTRL00991	NA	NA	NA	NA	GCCCTGTCCGTGGAACCAAC
5840	CTRL00992	NA	NA	NA	NA	GTACGCAAATTCGAGCTACA
5841	CTRL00993	NA	NA	NA	NA	GGGTGCGTAAGTACGGCTTA
5842	CTRL00994	NA	NA	NA	NA	GAAGTTATATACTGTTCCGC
5843	CTRL00995	NA	NA	NA	NA	GCCAGTGGTAAATCCCGATA
5844	CTRL00996	NA	NA	NA	NA	GGTCTTGCTAGTGAGTACGG
5845	CTRL00997	NA	NA	NA	NA	GAGTGATGGTACCAGCCCGT
5846	CTRL00998	NA	NA	NA	NA	GGCTTACCTAAATTGGAGGT
5847	CTRL00999	NA	NA	NA	NA	GATATCCTCTGTCGAGGAGC
5848	CTRL01000	NA	NA	NA	NA	GGACCAAGTCGTCGATCTTA
5849	CTRL01001	NA	NA	NA	NA	GTGTCAACGGAGAATGGTCA
5850	CTRL01002	NA	NA	NA	NA	GACGAGTCGATTATGAGCGG
5851	CTRL01003	NA	NA	NA	NA	GATACGCTCTCGCCCTTCCC
5852	CTRL01004	NA	NA	NA	NA	GCTAGCTAGTCTCGCGGGCA

Supplemental Table S2. Result of MAGeCK test of Mouse #1

id	num	Rank	neg|score	neg|p-value	neg|fdr	neg|rank	neg|goodsgrna	neg|lfc	Rank2	pos|score	pos|p-value	pos|fdr	pos|rank	pos|goodsgrna	pos|lfc
P4HTM	10	1	6.28E-05	0.00050204	0.111905	1	7	-4.3876	383	0.69726	0.86325	0.99915	383	3	-4.3876
CIAO1	10	2	9.12E-05	0.00064122	0.111905	2	9	-4.1746	460	0.97521	0.97844	0.99915	460	1	-4.1746
FDXR	10	3	9.71E-05	0.00070086	0.111905	3	9	-4.184	464	0.97981	0.98136	0.99915	464	1	-4.184
ATP6AP1	10	4	0.00024365	0.0019137	0.22619	4	10	-4.3322	479	0.99907	0.99915	0.99915	479	0	-4.3322
THAP4	10	5	0.00029842	0.0023611	0.22619	5	9	-4.3618	470	0.98675	0.98673	0.99915	470	1	-4.3618
POLA1	10	6	0.00045272	0.0033552	0.267857	6	8	-4.468	406	0.76453	0.89738	0.99915	406	2	-4.468
RTEL1	10	7	0.00063106	0.0047271	0.323469	7	9	-4.011	477	0.99784	0.99789	0.99915	477	0	-4.011
FLVCR2	10	8	0.00086666	0.0063674	0.379437	8	5	-1.7168	414	0.81908	0.91648	0.99915	414	2	-1.7168
NDUFV1	10	9	0.0010406	0.0076896	0.379437	9	9	-4.2922	475	0.99502	0.9953	0.99915	475	0	-4.2922
METAP1	10	10	0.0011458	0.0083259	0.379437	10	4	1.6833	25	0.01622	0.072378	0.99915	25	5	1.6833
PRC1	10	11	0.0012219	0.0087136	0.379437	11	7	-4.454	457	0.96126	0.97176	0.99915	457	1	-4.454
LIAS	10	12	0.0015316	0.010374	0.39747	12	7	-4.4575	163	0.15677	0.38454	0.99915	163	3	-4.4575
SLC39A6	10	13	0.0016963	0.011418	0.39747	13	8	-3.8354	428	0.86656	0.9358	0.99915	428	2	-3.8354
FDX1L	10	14	0.0017884	0.011905	0.39747	14	7	-4.215	300	0.44915	0.70873	0.99915	300	3	-4.215
PPP3CB	10	15	0.0019354	0.01278	0.39747	15	3	0.96839	226	0.28644	0.55836	0.99915	226	3	0.96839
ISCA2	10	16	0.0020079	0.013277	0.39747	16	9	-3.7639	474	0.99447	0.9948	0.99915	474	0	-3.7639
ERCC2	10	17	0.0023099	0.015275	0.403024	17	8	-4.3313	468	0.98443	0.98475	0.99915	468	1	-4.3313
ASPH	10	18	0.002598	0.016826	0.403024	18	4	0.4427	267	0.37665	0.64304	0.99915	267	4	0.4427
KDM4B	10	19	0.0026969	0.017402	0.403024	19	6	-2.6056	452	0.92217	0.9616	0.99915	452	1	-2.6056
GSTP1	10	20	0.0027897	0.017919	0.403024	20	8	-4.02	450	0.91623	0.96019	0.99915	450	1	-4.02
ELP3	10	21	0.0031511	0.019788	0.403024	21	9	-4.3149	384	0.7002	0.86484	0.99915	384	1	-4.3149
CYP4F22	10	22	0.0032498	0.020255	0.403024	22	7	-4.2267	207	0.24502	0.5145	0.99915	207	3	-4.2267
ABCB7	10	23	0.0032687	0.020395	0.403024	23	8	-4.0767	458	0.97028	0.97564	0.99915	458	1	-4.0767
ABCE1	10	24	0.0034407	0.021528	0.403024	24	8	-4.4215	386	0.70601	0.86804	0.99915	386	1	-4.4215
UQCRFS1	10	25	0.0036366	0.022492	0.403024	25	9	-4.3045	461	0.97655	0.97916	0.99915	461	1	-4.3045
GRXCR1	10	26	0.0037151	0.02288	0.403024	26	6	-4.0165	47	0.026554	0.10839	0.99915	47	2	-4.0165
APBB1	10	27	0.0039725	0.024252	0.403024	27	6	-4.1987	140	0.12051	0.32588	0.99915	140	3	-4.1987
PHYHD1	10	28	0.0040628	0.024729	0.403024	28	9	-3.8435	478	0.99825	0.9984	0.99915	478	0	-3.8435
PER1	10	29	0.0041848	0.025256	0.403024	29	7	-4.283	349	0.60388	0.80872	0.99915	349	3	-4.283
AOX1	10	30	0.0043266	0.025902	0.403024	30	6	-1.3	240	0.31217	0.5837	0.99915	240	4	-1.3
NFS1	10	31	0.0047629	0.028388	0.403024	31	7	-4.2053	393	0.719	0.8757	0.99915	393	2	-4.2053
TYW5	10	32	0.0048105	0.028586	0.403024	32	4	0.41785	370	0.67303	0.84913	0.99915	370	3	0.41785
DPH1	10	33	0.0049591	0.029362	0.403024	33	9	-3.786	467	0.98372	0.98417	0.99915	467	1	-3.786
NDUFS1	10	34	0.0051802	0.030505	0.403024	34	6	-4.2664	235	0.29882	0.57078	0.99915	235	2	-4.2664
HPX	10	35	0.005265	0.030893	0.403024	35	9	-2.0423	453	0.92625	0.9624	0.99915	453	1	-2.0423
KDM8	10	36	0.0052723	0.030903	0.403024	36	5	0.44943	272	0.38819	0.6538	0.99915	272	4	0.44943
HCCS	10	37	0.0053244	0.031131	0.403024	37	7	-3.5812	308	0.45981	0.7181	0.99915	308	2	-3.5812
CYCS	10	38	0.005548	0.032404	0.408459	38	7	-3.457	278	0.41131	0.67475	0.99915	278	3	-3.457
UQCRC2	10	39	0.0057656	0.033547	0.412027	39	6	-4.2979	432	0.88397	0.94397	0.99915	432	1	-4.2979
BLVRB	10	40	0.0070095	0.040446	0.484345	40	7	-1.6328	141	0.12244	0.32957	0.99915	141	3	-1.6328
NARFL	10	41	0.0073773	0.042395	0.495296	41	6	-2.0945	222	0.28262	0.55485	0.99915	222	2	-2.0945
CYC1	10	42	0.0082272	0.046958	0.535544	42	7	-3.8213	389	0.71102	0.87088	0.99915	389	3	-3.8213
CYP27B1	10	43	0.0085295	0.048578	0.537946	43	8	-3.7945	442	0.90074	0.95195	0.99915	442	1	-3.7945
ALKBH5	10	44	0.0087151	0.049652	0.537946	44	7	-3.932	254	0.33735	0.60778	0.99915	254	3	-3.932
NDOR1	10	45	0.0091387	0.05175	0.537946	45	8	-4.1936	447	0.91105	0.95716	0.99915	447	2	-4.1936
JHDM1D	10	46	0.0092298	0.052217	0.537946	46	7	-3.9958	365	0.66026	0.84114	0.99915	365	2	-3.9958
CYP11A1	10	47	0.0094962	0.053609	0.537946	47	7	-3.6565	381	0.69357	0.86123	0.99915	381	3	-3.6565
C16orf52	10	48	0.0095536	0.053907	0.537946	48	4	0.64808	290	0.427	0.68907	0.99915	290	3	0.64808
HBM	10	49	0.010177	0.057367	0.560787	49	7	-3.8933	328	0.51765	0.75971	0.99915	328	2	-3.8933
DDX11	10	50	0.010848	0.060717	0.573653	50	6	-2.9322	346	0.59585	0.80408	0.99915	346	2	-2.9322
CA6	10	51	0.011214	0.062128	0.573653	51	6	-2.5795	286	0.42081	0.68339	0.99915	286	3	-2.5795
PXDNL	10	52	0.012073	0.065797	0.573653	52	7	-4.0558	401	0.75036	0.89309	0.99915	401	2	-4.0558
NUBP2	10	53	0.012096	0.065856	0.573653	53	5	-1.3905	187	0.19505	0.44259	0.99915	187	4	-1.3905
FDX1	10	54	0.012246	0.066443	0.573653	54	8	-4.1849	403	0.75541	0.89453	0.99915	403	1	-4.1849
HBE1	10	55	0.012929	0.068998	0.573653	55	4	2.108	96	0.071957	0.22683	0.99915	96	6	2.108
CLSTN3	10	56	0.013071	0.069624	0.573653	56	6	-4.1073	376	0.68028	0.85334	0.99915	376	2	-4.1073
METAP2	10	57	0.013076	0.069654	0.573653	57	8	-3.8542	449	0.91612	0.96015	0.99915	449	2	-3.8542
RSBN1	10	58	0.013371	0.070797	0.573653	58	7	-4.0216	411	0.80451	0.91066	0.99915	411	2	-4.0216
CYP46A1	10	59	0.013727	0.072189	0.573653	59	8	-3.9632	402	0.75116	0.89332	0.99915	402	2	-3.9632
SLC39A14	10	60	0.013948	0.073004	0.573653	60	8	-3.8785	181	0.18541	0.4278	0.99915	181	2	-3.8785
HSCB	10	61	0.013975	0.073054	0.573653	61	7	-2.5792	465	0.98212	0.98289	0.99915	465	1	-2.5792
PPAT	10	62	0.01604	0.081285	0.602933	62	7	-3.9501	424	0.85586	0.93114	0.99915	424	2	-3.9501
BCMO1	10	63	0.016351	0.082449	0.602933	63	5	-1.0819	392	0.71843	0.87543	0.99915	392	3	-1.0819
RSBN1L	10	64	0.016927	0.084496	0.602933	64	7	-1.5148	441	0.9003	0.9517	0.99915	441	1	-1.5148
ACO2	10	65	0.017025	0.084894	0.602933	65	8	-3.9611	455	0.93489	0.96419	0.99915	455	1	-3.9611
TMEM199	10	66	0.017522	0.086574	0.602933	66	5	-1.5596	294	0.43324	0.69454	0.99915	294	4	-1.5596
MIPEP	10	67	0.017573	0.086733	0.602933	67	7	-4.0464	313	0.47376	0.73067	0.99915	313	3	-4.0464
DGCR8	10	68	0.017956	0.088185	0.602933	68	7	-3.8851	335	0.54423	0.77401	0.99915	335	3	-3.8851
METAP1D	10	69	0.018059	0.088503	0.602933	69	5	-0.12692	186	0.19465	0.44193	0.99915	186	2	-0.12692
CYP1B1	10	70	0.018294	0.089477	0.602933	70	7	-2.7597	416	0.82373	0.9181	0.99915	416	2	-2.7597
POLD1	10	71	0.018329	0.089547	0.602933	71	8	-2.4929	472	0.99215	0.99244	0.99915	472	0	-2.4929
SLC39A7	10	72	0.018813	0.091445	0.602933	72	8	-3.461	125	0.10481	0.29942	0.99915	125	2	-3.461
FECH	10	73	0.018979	0.092072	0.602933	73	7	-4.1007	396	0.74029	0.88843	0.99915	396	2	-4.1007
NDUFS2	10	74	0.020028	0.096187	0.602933	74	6	-4.0146	205	0.24322	0.51181	0.99915	205	3	-4.0146
ATP6V1A	10	75	0.020653	0.098514	0.602933	75	8	-3.74	448	0.91159	0.95748	0.99915	448	2	-3.74
EGLN1	10	76	0.020707	0.098723	0.602933	76	6	-4.0314	359	0.64781	0.83366	0.99915	359	2	-4.0314
KAT5	10	77	0.020716	0.098762	0.602933	77	5	-1.057	373	0.67686	0.85135	0.99915	373	3	-1.057
COQ7	10	78	0.020854	0.099349	0.602933	78	6	-3.4912	463	0.97897	0.98078	0.99915	463	1	-3.4912
STEAP1	10	79	0.020938	0.099617	0.602933	79	6	-2.6116	309	0.4607	0.71897	0.99915	309	4	-2.6116
GUCY1A2	10	80	0.021465	0.10162	0.602933	80	6	-1.3189	262	0.36917	0.63611	0.99915	262	4	-1.3189
EXO5	10	81	0.021861	0.10311	0.602933	81	8	-3.7575	405	0.76169	0.89633	0.99915	405	2	-3.7575
ACP5	10	82	0.021914	0.10322	0.602933	82	5	-1.1857	367	0.66443	0.84363	0.99915	367	3	-1.1857
IBA57	10	83	0.022925	0.10687	0.616781	83	7	-3.952	476	0.99783	0.99788	0.99915	476	0	-3.952
DPH3	10	84	0.023534	0.10916	0.618582	84	8	-4.0202	407	0.77831	0.90169	0.99915	407	2	-4.0202
NUBP1	10	85	0.023747	0.11004	0.618582	85	7	-4.1017	337	0.54823	0.77613	0.99915	337	1	-4.1017
CYP2B6	10	86	0.024395	0.11234	0.618582	86	6	-3.9101	431	0.88347	0.94374	0.99915	431	1	-3.9101
COX10	10	87	0.024397	0.11235	0.618582	87	4	0.44312	233	0.29676	0.56862	0.99915	233	3	0.44312
SDHC	10	88	0.024763	0.11366	0.618696	88	4	0.748	216	0.26	0.53379	0.99915	216	3	0.748
CYB5B	10	89	0.025898	0.11767	0.633307	89	8	-3.1428	234	0.29747	0.56927	0.99915	234	2	-3.1428

TSHZ3	10	90	0.026662	0.12025	0.633825	90	5	-0.14356	93	0.062638	0.20468	0.99915	93	2	-0.14356
PHF2	10	91	0.026856	0.12105	0.633825	91	8	-2.4633	445	0.90746	0.95517	0.99915	445	1	-2.4633
SDHB	10	92	0.027036	0.12174	0.633825	92	3	0.53902	84	0.058238	0.19372	0.99915	84	3	0.53902
MYLIP	10	93	0.02908	0.12911	0.663765	93	8	-3.6575	427	0.86641	0.93571	0.99915	427	2	-3.6575
RORC	10	94	0.029751	0.13162	0.663765	94	6	-4.0124	253	0.33485	0.60547	0.99915	253	4	-4.0124
MINA	10	95	0.029909	0.13217	0.663765	95	7	-3.4947	382	0.69725	0.86325	0.99915	382	2	-3.4947
CH25H	10	96	0.030131	0.13303	0.663765	96	8	-2.8252	454	0.93309	0.96383	0.99915	454	1	-2.8252
ALOX5	10	97	0.030563	0.13451	0.664237	97	6	-0.97088	108	0.086216	0.26053	0.99915	108	3	-0.97088
P4HA2	10	98	0.03162	0.13824	0.672151	98	7	-1.3286	219	0.2678	0.54114	0.99915	219	3	-1.3286
SOD2	10	99	0.032805	0.14239	0.672151	99	7	-3.9365	302	0.45283	0.71208	0.99915	302	3	-3.9365
HPD	10	100	0.033499	0.14462	0.672151	100	7	-3.6326	471	0.98973	0.9898	0.99915	471	0	-3.6326
HGD	10	101	0.033518	0.14466	0.672151	101	6	-3.3994	160	0.15443	0.38087	0.99915	160	4	-3.3994
KDM5C	10	102	0.03432	0.14759	0.672151	102	7	-3.2278	419	0.83275	0.92181	0.99915	419	2	-3.2278
KDM4A	10	103	0.034984	0.14984	0.672151	103	3	1.3552	15	0.0077757	0.038717	0.99915	15	4	1.3552
NDUFS7	10	104	0.036066	0.15319	0.672151	104	5	-1.7802	53	0.030948	0.12155	0.99915	53	3	-1.7802
OGFOD1	10	105	0.036203	0.15362	0.672151	105	8	-3.5318	243	0.31425	0.58582	0.99915	243	2	-3.5318
LEPRE1	10	106	0.036218	0.15368	0.672151	106	5	-1.9329	350	0.61421	0.8141	0.99915	350	2	-1.9329
ALKBH1	10	107	0.036351	0.15408	0.672151	107	7	-3.2113	357	0.64064	0.82948	0.99915	357	3	-3.2113
GLRX5	10	108	0.036662	0.15506	0.672151	108	6	-3.3136	251	0.32759	0.59899	0.99915	251	4	-3.3136
RPE65	10	109	0.038169	0.16008	0.672151	109	6	-3.8469	379	0.69249	0.86067	0.99915	379	2	-3.8469
BOLA1	10	110	0.038442	0.16091	0.672151	110	6	-3.4779	324	0.49562	0.74791	0.99915	324	3	-3.4779
SLC40A1	10	111	0.038782	0.16204	0.672151	111	7	-3.2599	469	0.98503	0.98521	0.99915	469	1	-3.2599
GALT	10	112	0.038827	0.16215	0.672151	112	6	-3.7061	55	0.03162	0.12363	0.99915	55	2	-3.7061
MPO	10	113	0.039485	0.16419	0.672151	113	7	-3.5308	320	0.48712	0.74123	0.99915	320	1	-3.5308
HRG	10	114	0.0395	0.1642	0.672151	114	5	-0.52751	198	0.22725	0.48922	0.99915	198	5	-0.52751
APBA1	10	115	0.04001	0.1659	0.672151	115	5	0.12811	49	0.028245	0.11389	0.99915	49	3	0.12811
NDUFS8	10	116	0.040379	0.16695	0.672151	116	8	-3.5482	223	0.28274	0.55502	0.99915	223	2	-3.5482
ALB	10	117	0.04077	0.16819	0.672151	117	3	0.7561	250	0.3272	0.59858	0.99915	250	3	0.7561
OSGEP	10	118	0.040797	0.16829	0.672151	118	7	-1.1927	256	0.33974	0.61002	0.99915	256	1	-1.1927
TET2	10	119	0.041126	0.1693	0.672151	119	7	-3.2598	433	0.88887	0.94639	0.99915	433	1	-3.2598
ABCG2	10	120	0.041456	0.17033	0.672151	120	5	0.79537	16	0.0084305	0.04161	0.99915	16	5	0.79537
FTL	10	121	0.041558	0.17071	0.672151	121	4	0.79954	14	0.0062249	0.032165	0.99915	14	4	0.79954
JMJD6	10	122	0.04168	0.17119	0.672151	122	5	-0.52112	321	0.48855	0.74244	0.99915	321	4	-0.52112
MB	10	123	0.043348	0.17694	0.684773	123	5	-0.48024	420	0.84127	0.92515	0.99915	420	1	-0.48024
SLC25A28	10	124	0.043441	0.17727	0.684773	124	7	-3.8226	413	0.81751	0.91593	0.99915	413	2	-3.8226
PAPL	10	125	0.0447	0.1814	0.693519	125	4	1.7503	18	0.0091387	0.044622	0.99915	18	5	1.7503
SCARA5	10	126	0.045012	0.18243	0.693519	126	5	-0.27608	264	0.37354	0.64026	0.99915	264	3	-0.27608
SLC39A5	10	127	0.045485	0.18401	0.694019	127	8	-3.7595	358	0.64577	0.83245	0.99915	358	2	-3.7595
CYP3A5	10	128	0.046675	0.18785	0.702958	128	5	-0.99027	168	0.16406	0.39568	0.99915	168	4	-0.99027
SLC39A11	10	129	0.048334	0.19348	0.709542	129	4	2.1098	73	0.046675	0.16376	0.99915	73	5	2.1098
FTO	10	130	0.048447	0.19384	0.709542	130	6	-3.2237	385	0.70336	0.86666	0.99915	385	3	-3.2237
CIAPIN1	10	131	0.048495	0.19405	0.709542	131	6	-0.96519	203	0.24127	0.50889	0.99915	203	4	-0.96519
SLC11A2	10	132	0.050187	0.19934	0.723359	132	5	-1.3333	33	0.020014	0.085978	0.99915	33	4	-1.3333
COQ6	10	133	0.050799	0.20101	0.723935	133	6	-3.716	352	0.61833	0.81656	0.99915	352	3	-3.716
ALKBH7	10	134	0.051449	0.20301	0.725675	134	7	-2.6257	372	0.67464	0.84999	0.99915	372	2	-2.6257
HBZ	10	135	0.052882	0.20758	0.730987	135	8	-0.92901	261	0.36898	0.63601	0.99915	261	2	-0.92901
CDK5RAP	10	136	0.053298	0.20877	0.730987	136	5	0.068028	41	0.023164	0.096983	0.99915	41	3	0.068028
COX15	10	137	0.053381	0.20907	0.730987	137	7	-3.1704	440	0.897	0.94987	0.99915	440	2	-3.1704
FTMT	10	138	0.054514	0.21262	0.734926	138	7	-2.1891	439	0.89679	0.94978	0.99915	439	2	-2.1891
KIF4A	10	139	0.054687	0.21327	0.734926	139	6	-3.5635	126	0.10629	0.30223	0.99915	126	4	-3.5635
MARK3	10	140	0.058624	0.22315	0.745756	140	6	-1.2086	123	0.10167	0.29389	0.99915	123	4	-1.2086
ALKBH4	10	141	0.05896	0.22409	0.745756	141	7	-3.603	31	0.019547	0.084248	0.99915	31	2	-3.603
REV3L	10	142	0.059113	0.22442	0.745756	142	6	-2.3461	188	0.1975	0.44641	0.99915	188	2	-2.3461
CYP1A1	10	143	0.059252	0.22467	0.745756	143	5	0.14881	315	0.47488	0.73146	0.99915	315	4	0.14881
SP1	10	144	0.059526	0.22521	0.745756	144	7	-2.5173	423	0.85524	0.93098	0.99915	423	1	-2.5173
UQCRC1	10	145	0.059965	0.22647	0.745756	145	7	-2.943	446	0.91047	0.9569	0.99915	446	1	-2.943
KDM2B	10	146	0.06049	0.22764	0.745756	146	6	-3.59	362	0.65387	0.83715	0.99915	362	2	-3.59
POLE	10	147	0.060945	0.22886	0.745756	147	7	-3.57	257	0.34473	0.6146	0.99915	257	3	-3.57
CYP2J2	10	148	0.063152	0.23426	0.758189	148	6	-1.8595	437	0.89517	0.94906	0.99915	437	2	-1.8595
CACNA1C	10	149	0.064718	0.23808	0.759119	149	7	-2.9417	417	0.82502	0.91853	0.99915	417	1	-2.9417
RRM2	10	150	0.065368	0.23973	0.759119	150	6	-2.023	204	0.24168	0.50944	0.99915	204	2	-2.023
ASPHD2	10	151	0.065907	0.24091	0.759119	151	7	-1.6879	378	0.6847	0.85591	0.99915	378	2	-1.6879
NIPAL1	10	152	0.06642	0.24227	0.759119	152	3	3.3974	9	0.0023477	0.013575	0.722487	9	7	3.3974
CCDC115	10	153	0.066516	0.24247	0.759119	153	7	-1.9982	109	0.087395	0.26302	0.99915	109	2	-1.9982
HBQ1	10	154	0.068049	0.24595	0.765012	154	5	-1.3253	275	0.40474	0.6688	0.99915	275	3	-1.3253
PIR	10	155	0.070134	0.25087	0.775284	155	7	-2.8705	347	0.60194	0.80755	0.99915	347	2	-2.8705
AGMO	10	156	0.072004	0.25537	0.779021	156	7	-3.2535	443	0.9016	0.95242	0.99915	443	1	-3.2535
HEBP1	10	157	0.072314	0.25617	0.779021	157	5	0.77195	76	0.049683	0.17174	0.99915	76	5	0.77195
DUOX2	10	158	0.072843	0.25766	0.779021	158	5	0.17173	69	0.042352	0.15228	0.99915	69	4	0.17173
TF	10	159	0.07324	0.25859	0.779021	159	6	-3.3501	83	0.056728	0.19003	0.99915	83	3	-3.3501
CYP7B1	10	160	0.07459	0.26158	0.780761	160	6	-1.1932	148	0.13376	0.34859	0.99915	148	4	-1.1932
FADS3	10	161	0.075356	0.26335	0.780761	161	6	-3.5912	348	0.60294	0.80817	0.99915	348	3	-3.5912
CYP4A11	10	162	0.075605	0.26406	0.780761	162	6	-3.5037	236	0.30153	0.57333	0.99915	236	3	-3.5037
NENF	10	163	0.078418	0.27083	0.795866	163	6	-1.0035	217	0.26201	0.53572	0.99915	217	4	-1.0035
KDM6B	10	164	0.079378	0.273	0.797372	164	3	2.5402	36	0.021073	0.089746	0.99915	36	6	2.5402
CYP2E1	10	165	0.081598	0.27815	0.807489	165	7	-0.65772	397	0.74251	0.88992	0.99915	397	3	-0.65772
KDM2A	10	166	0.084667	0.28544	0.823652	166	5	-1.6355	422	0.85295	0.93011	0.99915	422	2	-1.6355
CYP2W1	10	167	0.087395	0.29161	0.831185	167	6	-0.54788	32	0.019763	0.085093	0.99915	32	3	-0.54788
SCD	10	168	0.087774	0.29245	0.831185	168	6	-0.4218	142	0.12654	0.33657	0.99915	142	4	-0.4218
COPA	10	169	0.089929	0.29714	0.831185	169	7	-3.2498	258	0.35626	0.62509	0.99915	258	2	-3.2498
CYB561D2	10	170	0.090165	0.29756	0.831185	170	5	0.79078	52	0.03018	0.11953	0.99915	52	5	0.79078
ACO1	10	171	0.090584	0.29844	0.831185	171	5	-0.18915	307	0.45662	0.71546	0.99915	307	3	-0.18915
EGLN2	10	172	0.090798	0.29888	0.831185	172	4	1.2791	133	0.11317	0.31388	0.99915	133	5	1.2791
DDX5	10	173	0.091858	0.30125	0.831185	173	5	-1.0493	366	0.664	0.84342	0.99915	366	3	-1.0493
ALOX15	10	174	0.092175	0.30193	0.831185	174	6	-1.4067	179	0.18159	0.4222	0.99915	179	3	-1.4067
ATP6V0D1	10	175	0.095982	0.31069	0.849824	175	6	-2.1761	388	0.70898	0.86968	0.99915	388	3	-2.1761
PLOD2	10	176	0.096667	0.31225	0.849824	176	5	-1.8425	334	0.54024	0.77201	0.99915	334	3	-1.8425
PARN	10	177	0.097852	0.31504	0.85103	177	5	-0.46283	304	0.45336	0.71253	0.99915	304	3	-0.46283
PLOD3	10	178	0.098392	0.31625	0.85103	178	5	-0.99965	399	0.74837	0.8924	0.99915	399	2	-0.99965
SLC39A13	10	179	0.099953	0.31977	0.855693	179	7	-1.5025	214	0.25395	0.52722	0.99915	214	2	-1.5025
P4HA1	10	180	0.10426	0.32883	0.871152	180	6	-2.5643	314	0.47428	0.73097	0.99915	314	3	-2.5643

PHF8	10	181	0.10443	0.32918	0.871152	181	5	-0.82339	146	0.13238	0.34638	0.99915	146	5	-0.82339
CYP2C19	10	182	0.10798	0.33673	0.881978	182	6	-3.2192	196	0.21326	0.46902	0.99915	196	3	-3.2192
GUCY1B3	10	183	0.10951	0.34013	0.881978	183	6	-0.96259	418	0.82966	0.92045	0.99915	418	1	-0.96259
CYB5R3	10	184	0.11107	0.34356	0.881978	184	3	1.8501	39	0.022524	0.094706	0.99915	39	5	1.8501
SRC	10	185	0.11116	0.3437	0.881978	185	7	-2.0021	391	0.71566	0.87375	0.99915	391	2	-2.0021
DOHH	10	186	0.11119	0.34381	0.881978	186	7	-2.4509	270	0.3814	0.6475	0.99915	270	3	-2.4509
CYP2S1	10	187	0.11219	0.34607	0.881978	187	6	-0.39969	435	0.89124	0.94739	0.99915	435	1	-0.39969
CYP27A1	10	188	0.11226	0.34616	0.881978	188	6	-3.2621	268	0.37712	0.64357	0.99915	268	4	-3.2621
BBOX1	10	189	0.11418	0.34977	0.886458	189	3	0.82378	88	0.05988	0.19784	0.99915	88	3	0.82378
SLC11A1	10	190	0.11728	0.35626	0.887832	190	7	-1.841	165	0.16085	0.39075	0.99915	165	3	-1.841
CYP4Z1	10	191	0.11729	0.35626	0.887832	191	3	1.5045	26	0.016351	0.072855	0.99915	26	4	1.5045
MFI2	10	192	0.11817	0.35802	0.887832	192	7	-1.996	101	0.078067	0.24127	0.99915	101	2	-1.996
AFM	10	193	0.12043	0.36261	0.887832	193	6	-3.0802	128	0.10794	0.30499	0.99915	128	4	-3.0802
FLVCR1	10	194	0.12067	0.36303	0.887832	194	6	-3.1189	277	0.40803	0.67174	0.99915	277	2	-3.1189
SDHAF1	10	195	0.12225	0.36681	0.887832	195	6	-2.2774	333	0.53723	0.77037	0.99915	333	3	-2.2774
CYB5D1	10	196	0.12314	0.36871	0.887832	196	7	-2.2958	192	0.203	0.45457	0.99915	192	3	-2.2958
CLSTN2	10	197	0.12347	0.36939	0.887832	197	4	0.98944	249	0.32486	0.59615	0.99915	249	4	0.98944
NGB	10	198	0.12359	0.36957	0.887832	198	7	-2.156	390	0.71277	0.87196	0.99915	390	2	-2.156
CYP4B1	10	199	0.12498	0.37267	0.887832	199	7	-1.813	412	0.81578	0.91533	0.99915	412	2	-1.813
PGRMC2	10	200	0.12521	0.37308	0.887832	200	7	-2.1931	462	0.97787	0.98002	0.99915	462	1	-2.1931
JMJD7	10	201	0.12808	0.37904	0.887832	201	4	2.5163	74	0.0474	0.16569	0.99915	74	6	2.5163
COX5A	10	202	0.12824	0.37939	0.887832	202	7	-0.61622	436	0.89171	0.9476	0.99915	436	2	-0.61622
IREB2	10	203	0.12925	0.38157	0.887832	203	5	-1.159	156	0.14908	0.37257	0.99915	156	4	-1.159
MPPED1	10	204	0.13062	0.38431	0.887832	204	5	0.076149	105	0.084196	0.25597	0.99915	105	1	0.076149
HIF1A	10	205	0.13068	0.38444	0.887832	205	6	-0.69807	269	0.38087	0.64707	0.99915	269	3	-0.69807
APBB3	10	206	0.13084	0.38483	0.887832	206	7	-1.7887	363	0.65835	0.83986	0.99915	363	3	-1.7887
ABAT	10	207	0.13114	0.38549	0.887832	207	3	2.13	11	0.0043266	0.023347	0.99915	11	6	2.13
CYP24A1	10	208	0.13117	0.38553	0.887832	208	6	-1.9713	164	0.15899	0.38792	0.99915	164	4	-1.9713
IRG1	10	209	0.13266	0.3884	0.890169	209	4	0.79225	340	0.56409	0.78552	0.99915	340	3	0.79225
DUOX1	10	210	0.13367	0.39038	0.890442	210	5	-1.6004	239	0.30896	0.58017	0.99915	239	3	-1.6004
EPX	10	211	0.13477	0.39251	0.891052	211	6	-2.8544	113	0.091858	0.2728	0.99915	113	3	-2.8544
CYP2C18	10	212	0.1383	0.39904	0.901606	212	5	0.1019	400	0.74948	0.8927	0.99915	400	2	0.1019
CYP11B2	10	213	0.14161	0.4059	0.909074	213	6	-1.8397	263	0.37238	0.63902	0.99915	263	3	-1.8397
JMJD8	10	214	0.14328	0.40913	0.909074	214	5	-0.00247	325	0.49655	0.74829	0.99915	325	3	-0.00247
CYP8B1	10	215	0.14338	0.40931	0.909074	215	7	-1.3997	421	0.85161	0.92951	0.99915	421	2	-1.3997
ASAH2	10	216	0.14479	0.4116	0.909074	216	5	0.70563	178	0.17747	0.41602	0.99915	178	5	0.70563
LRP2	10	217	0.14493	0.41184	0.909074	217	6	-0.42099	459	0.97341	0.97733	0.99915	459	1	-0.42099
HAAO	10	218	0.14727	0.41633	0.914777	218	6	-2.1209	318	0.48426	0.73863	0.99915	318	3	-2.1209
NTHL1	10	219	0.14867	0.41913	0.916732	219	5	-1.416	345	0.5949	0.80355	0.99915	345	3	-1.416
RFESD	10	220	0.15006	0.42179	0.918344	220	7	-1.3334	305	0.45441	0.71347	0.99915	305	3	-1.3334
HSPBAP1	10	221	0.15458	0.43073	0.932801	221	6	-0.68508	12	0.0060525	0.03139	0.99915	12	2	-0.68508
DPYD	10	222	0.15915	0.43914	0.932801	222	5	-1.2945	395	0.72586	0.88004	0.99915	395	3	-1.2945
SCD5	10	223	0.15972	0.44019	0.932801	223	5	-1.4968	319	0.48623	0.74043	0.99915	319	3	-1.4968
STEAP3	10	224	0.16085	0.4423	0.932801	224	5	0.07674	322	0.49043	0.74421	0.99915	322	4	0.07674
NR1D1	10	225	0.16102	0.44256	0.932801	225	6	-2.0667	473	0.99313	0.9934	0.99915	473	0	-2.0667
TMEM62	10	226	0.16122	0.44289	0.932801	226	4	0.42492	429	0.87758	0.94099	0.99915	429	2	0.42492
P4HA3	10	227	0.16155	0.44352	0.932801	227	6	-2.4908	444	0.90394	0.95355	0.99915	444	1	-2.4908
GLRX3	10	228	0.16183	0.44401	0.932801	228	7	-0.962	434	0.88954	0.94676	0.99915	434	2	-0.962
ALKBH3	10	229	0.16319	0.44638	0.933323	229	5	0.18482	289	0.42609	0.68825	0.99915	289	3	0.18482
FADS1	10	230	0.1643	0.44837	0.933323	230	7	-1.3761	410	0.79346	0.90676	0.99915	410	2	-1.3761
RSAD1	10	231	0.16529	0.4501	0.933323	231	5	-0.45413	58	0.034537	0.13193	0.99915	58	5	-0.45413
DLG3	10	232	0.16708	0.4531	0.933525	232	7	-0.80915	291	0.4283	0.69013	0.99915	291	3	-0.80915
HR	10	233	0.16877	0.4561	0.933525	233	6	-2.6411	332	0.53366	0.7684	0.99915	332	2	-2.6411
NOS1	10	234	0.16913	0.45678	0.933525	234	6	-2.5615	260	0.36091	0.62912	0.99915	260	3	-2.5615
CYP1A2	10	235	0.17074	0.45978	0.933525	235	7	-2.2022	295	0.43341	0.69475	0.99915	295	2	-2.2022
CISD3	10	236	0.17083	0.45994	0.933525	236	6	-2.5394	220	0.27318	0.54649	0.99915	220	2	-2.5394
LRP1	10	237	0.17298	0.46341	0.933683	237	5	-0.30149	134	0.1133	0.31404	0.99915	134	4	-0.30149
ALOX15B	10	238	0.17335	0.46392	0.933683	238	6	-1.2037	331	0.53306	0.76804	0.99915	331	3	-1.2037
FTHL17	10	239	0.17588	0.46697	0.935744	239	4	1.8225	112	0.089913	0.26857	0.99915	112	6	1.8225
BRIP1	10	240	0.17736	0.46885	0.935744	240	5	0.01109	285	0.41998	0.6828	0.99915	285	4	0.01109
AMBP	10	241	0.17949	0.47178	0.93769	241	6	-2.2082	151	0.13732	0.35397	0.99915	151	4	-2.2082
SGK1	10	242	0.18131	0.47437	0.938932	242	6	-2.0174	361	0.65384	0.83714	0.99915	361	3	-2.0174
CDKAL1	10	243	0.18601	0.48056	0.940707	243	6	-2.3148	228	0.28953	0.56145	0.99915	228	4	-2.3148
HSPA6	10	244	0.18772	0.48311	0.940707	244	4	1.7357	201	0.23203	0.4957	0.99915	201	5	1.7357
CYB561D1	10	245	0.18953	0.48574	0.940707	245	5	-0.73559	98	0.074538	0.23296	0.99915	98	3	-0.73559
ISCA1	10	246	0.19465	0.49253	0.940707	246	5	-0.59568	227	0.28787	0.55975	0.99915	227	3	-0.59568
NR1D2	10	247	0.19476	0.49268	0.940707	247	5	-1.5435	351	0.6174	0.81601	0.99915	351	2	-1.5435
HPDL	10	248	0.19612	0.49471	0.940707	248	5	-1.4347	169	0.16417	0.39589	0.99915	169	4	-1.4347
KDM5D	10	249	0.19698	0.49585	0.940707	249	3	2.1912	80	0.052605	0.17919	0.99915	80	6	2.1912
LYRM4	10	250	0.19892	0.4986	0.940707	250	6	-0.9919	342	0.57061	0.78922	0.99915	342	2	-0.9919
HERC2	10	251	0.19892	0.4986	0.940707	251	4	1.401	35	0.020862	0.089	0.99915	35	5	1.401
CYP2R1	10	252	0.20034	0.50062	0.940707	252	3	2.6213	4	0.00029555	0.0020927	0.250595	4	5	2.6213
NDUFV2	10	253	0.20196	0.5028	0.940707	253	6	-1.9529	276	0.40687	0.67068	0.99915	276	3	-1.9529
RAB6B	10	254	0.20217	0.50308	0.940707	254	6	-1.9109	44	0.024702	0.10233	0.99915	44	4	-1.9109
PLOD1	10	255	0.20367	0.5051	0.940707	255	6	-1.6246	380	0.69275	0.86078	0.99915	380	3	-1.6246
SLC25A37	10	256	0.20458	0.50624	0.940707	256	5	-0.78577	247	0.32258	0.59383	0.99915	247	4	-0.78577
SLC39A2	10	257	0.20492	0.50669	0.940707	257	5	0.29588	212	0.24994	0.52123	0.99915	212	3	0.29588
HEBP2	10	258	0.20858	0.51103	0.945109	258	6	-2.2369	451	0.92005	0.96121	0.99915	451	1	-2.2369
KDM3A	10	259	0.21188	0.51509	0.947156	259	6	-1.3774	119	0.098513	0.28828	0.99915	119	2	-1.3774
LPO	10	260	0.21391	0.51797	0.947156	260	5	0.24053	296	0.43809	0.69923	0.99915	296	3	0.24053
ATP6V0A2	10	261	0.21399	0.51807	0.947156	261	5	0.14589	183	0.18748	0.43102	0.99915	183	3	0.14589
ABCB6	10	262	0.21718	0.52247	0.949775	262	6	-1.4812	299	0.44767	0.70741	0.99915	299	3	-1.4812
JMJD1C	10	263	0.2179	0.52347	0.949775	263	6	-2.0421	293	0.43133	0.69299	0.99915	293	3	-2.0421
CYP4A22	10	264	0.22348	0.53084	0.959063	264	3	0.61108	246	0.32031	0.59155	0.99915	246	4	0.61108
SUOX	10	265	0.22774	0.53613	0.959063	265	5	0.45494	162	0.15671	0.38446	0.99915	162	5	0.45494
CYGB	10	266	0.23054	0.53955	0.959063	266	6	-2.0019	317	0.4774	0.73352	0.99915	317	3	-2.0019
JMJD4	10	267	0.23238	0.54175	0.959063	267	4	1.9263	7	0.0018707	0.010762	0.722487	7	5	1.9263
EIF2AK1	10	268	0.23321	0.54297	0.959063	268	3	1.2168	79	0.051646	0.17689	0.99915	79	5	1.2168
MIOX	10	269	0.23372	0.54362	0.959063	269	5	0.000553	238	0.30764	0.57908	0.99915	238	4	0.000553
PTGES2	10	270	0.24	0.55148	0.959063	270	6	-1.2108	81	0.054947	0.18564	0.99915	81	4	-1.2108
CYP4F11	10	271	0.24021	0.55171	0.959063	271	5	-1.3972	387	0.70792	0.86917	0.99915	387	2	-1.3972

EGLN3	10	272	0.24071	0.55235	0.959063	272	6	-1.2417	110	0.089232	0.26725	0.99915	110	4	-1.2417
SLC39A4	10	273	0.24177	0.55363	0.959063	273	6	-1.2046	338	0.54992	0.77707	0.99915	338	3	-1.2046
NFU1	10	274	0.24625	0.55932	0.959063	274	5	-1.1945	377	0.68402	0.85557	0.99915	377	3	-1.1945
DNAJC24	10	275	0.24726	0.5606	0.959063	275	5	-0.13109	281	0.41441	0.67737	0.99915	281	1	-0.13109
HTT	10	276	0.2486	0.56222	0.959063	276	4	0.40184	415	0.82311	0.9179	0.99915	415	2	0.40184
SETD7	10	277	0.24899	0.56261	0.959063	277	5	0.87716	100	0.076296	0.2369	0.99915	100	5	0.87716
CP	10	278	0.25448	0.56976	0.959063	278	4	0.70626	131	0.11107	0.31049	0.99915	131	4	0.70626
CBS	10	279	0.25661	0.5725	0.959063	279	4	1.9815	92	0.062025	0.20321	0.99915	92	6	1.9815
TDO2	10	280	0.25706	0.57296	0.959063	280	4	0.95878	107	0.085182	0.25823	0.99915	107	4	0.95878
FRAS1	10	281	0.26578	0.58321	0.959063	281	4	0.22433	369	0.66903	0.84647	0.99915	369	3	0.22433
PPP3CA	10	282	0.26688	0.58441	0.959063	282	6	-1.1327	103	0.08064	0.24752	0.99915	103	4	-1.1327
MMS19	10	283	0.27319	0.59188	0.959063	283	5	-0.82698	310	0.46174	0.71993	0.99915	310	3	-0.82698
APP	10	284	0.27366	0.59233	0.959063	284	5	-1.1567	409	0.7922	0.90631	0.99915	409	2	-1.1567
IDO1	10	285	0.27494	0.59381	0.959063	285	6	-1.7936	329	0.51944	0.76057	0.99915	329	1	-1.7936
BOLA3	10	286	0.2774	0.59634	0.959063	286	6	-1.4702	456	0.94695	0.96718	0.99915	456	1	-1.4702
CAT	10	287	0.27888	0.59797	0.959063	287	3	2.5401	48	0.027017	0.11019	0.99915	48	7	2.5401
CYP4F2	10	288	0.28121	0.60046	0.959063	288	6	-0.87679	166	0.1627	0.39361	0.99915	166	4	-0.87679
ISCU	10	289	0.28273	0.60225	0.959063	289	6	-0.84726	466	0.98235	0.98302	0.99915	466	1	-0.84726
XDH	10	290	0.28734	0.6076	0.959063	290	6	-1.152	425	0.86027	0.93302	0.99915	425	2	-1.152
AMDHD1	10	291	0.28976	0.61064	0.959063	291	5	-0.8519	189	0.2002	0.45044	0.99915	189	3	-0.8519
CYP4F8	10	292	0.29296	0.61436	0.959063	292	5	0.49616	199	0.22872	0.49122	0.99915	199	5	0.49616
DEGS2	10	293	0.3018	0.62502	0.959063	293	4	1.7158	177	0.17423	0.41144	0.99915	177	5	1.7158
SLC39A8	10	294	0.30274	0.62609	0.959063	294	5	0.53388	85	0.058433	0.1943	0.99915	85	5	0.53388
EVL	10	295	0.30608	0.63006	0.959063	295	6	-0.9455	138	0.11884	0.32332	0.99915	138	2	-0.9455
CYP51A1	10	296	0.30812	0.63221	0.959063	296	5	-0.10345	94	0.063155	0.2059	0.99915	94	5	-0.10345
HFE	10	297	0.30857	0.63273	0.959063	297	5	-0.57094	22	0.012929	0.059782	0.99915	22	4	-0.57094
PAH	10	298	0.31044	0.6349	0.959063	298	6	-0.8045	194	0.20881	0.46289	0.99915	194	3	-0.8045
CYP7A1	10	299	0.31177	0.63657	0.959063	299	5	0.42255	127	0.10638	0.30249	0.99915	127	5	0.42255
PXDN	10	300	0.31389	0.63911	0.959063	300	3	2.6379	46	0.025373	0.10464	0.99915	46	7	2.6379
SLC39A3	10	301	0.31425	0.63952	0.959063	301	4	1.5303	132	0.11267	0.31309	0.99915	132	5	1.5303
CA9	10	302	0.31548	0.64084	0.959063	302	4	1.1409	210	0.24902	0.51991	0.99915	210	3	1.1409
AFP	10	303	0.31603	0.64144	0.959063	303	6	-0.94194	354	0.62623	0.82152	0.99915	354	2	-0.94194
FABP7	10	304	0.31671	0.64217	0.959063	304	4	0.77086	104	0.082593	0.2521	0.99915	104	4	0.77086
ETHE1	10	305	0.31723	0.64273	0.959063	305	6	-0.60834	438	0.89639	0.94957	0.99915	438	2	-0.60834
PDE4D	10	306	0.31729	0.64277	0.959063	306	5	-1.1996	356	0.63929	0.82899	0.99915	356	3	-1.1996
MSMO1	10	307	0.31794	0.64358	0.959063	307	4	0.4215	161	0.15529	0.3822	0.99915	161	3	0.4215
NOX1	10	308	0.32284	0.64922	0.959063	308	4	0.92329	364	0.65878	0.84016	0.99915	364	3	0.92329
MUTYH	10	309	0.32487	0.6515	0.959063	309	4	1.3941	170	0.16449	0.39638	0.99915	170	5	1.3941
HDAC1	10	310	0.3254	0.65217	0.959063	310	4	0.50873	242	0.31369	0.58535	0.99915	242	4	0.50873
CYP19A1	10	311	0.32733	0.65425	0.959063	311	6	-0.99353	37	0.021465	0.091137	0.99915	37	4	-0.99353
ETFDH	10	312	0.32892	0.65578	0.959063	312	5	0.26066	63	0.038337	0.14176	0.99915	63	4	0.26066
JAK2	10	313	0.33013	0.65733	0.959063	313	5	0.69189	224	0.28499	0.55707	0.99915	224	4	0.69189
CYB5R4	10	314	0.33065	0.65789	0.959063	314	3	1.8855	118	0.097632	0.28618	0.99915	118	6	1.8855
HBB	10	315	0.33125	0.65856	0.959063	315	4	1.0643	237	0.30305	0.57477	0.99915	237	4	1.0643
CYP11B1	10	316	0.33181	0.65922	0.959063	316	6	-0.44195	209	0.2486	0.51944	0.99915	209	3	-0.44195
FBXL5	10	317	0.33254	0.65987	0.959063	317	4	1.7874	34	0.020249	0.086743	0.99915	34	5	1.7874
FOLH1	10	318	0.33369	0.6611	0.959063	318	5	-0.99866	171	0.16636	0.3992	0.99915	171	4	-0.99866
TSHZ1	10	319	0.33375	0.66113	0.959063	319	4	2.6275	10	0.003314	0.018635	0.892619	10	5	2.6275
SET	10	320	0.33454	0.66193	0.959063	320	6	-1.2187	326	0.50989	0.75579	0.99915	326	3	-1.2187
LTF	10	321	0.33462	0.66206	0.959063	321	6	-0.18302	360	0.65044	0.8349	0.99915	360	3	-0.18302
HMOX2	10	322	0.33468	0.66213	0.959063	322	6	-0.63874	273	0.39449	0.65985	0.99915	273	4	-0.63874
SLC48A1	10	323	0.33485	0.66237	0.959063	323	3	1.2548	62	0.037299	0.13887	0.99915	62	4	1.2548
MRE11A	10	324	0.33615	0.66368	0.959063	324	5	0.37569	30	0.019092	0.082439	0.99915	30	4	0.37569
MAP2	10	325	0.33751	0.66529	0.959063	325	5	-1.0217	61	0.035914	0.13514	0.99915	61	4	-1.0217
KDM6A	10	326	0.33855	0.6663	0.959063	326	5	-0.65016	327	0.51244	0.75714	0.99915	327	3	-0.65016
HAMP	10	327	0.33974	0.66769	0.959063	327	5	0.52531	215	0.25438	0.5278	0.99915	215	2	0.52531
AIFM3	10	328	0.34084	0.66885	0.959063	328	6	-0.27983	144	0.12808	0.33927	0.99915	144	2	-0.27983
ALOX12B	10	329	0.34092	0.66889	0.959063	329	5	-0.77099	77	0.051325	0.17601	0.99915	77	4	-0.77099
HMOX1	10	330	0.34331	0.67139	0.959063	330	5	0.23648	64	0.039441	0.14447	0.99915	64	4	0.23648
OGFOD3	10	331	0.34503	0.67321	0.959063	331	4	2.4235	72	0.046136	0.16238	0.99915	72	6	2.4235
PER3	10	332	0.34539	0.67362	0.959063	332	4	0.082606	271	0.38707	0.65263	0.99915	271	2	0.082606
FTH1	10	333	0.34539	0.67362	0.959063	333	6	-0.64799	339	0.55497	0.78015	0.99915	339	2	-0.64799
MDM4	10	334	0.34913	0.67763	0.959063	334	6	-0.26284	344	0.58583	0.79805	0.99915	344	3	-0.26284
KDM5B	10	335	0.35014	0.67861	0.959063	335	5	-0.20559	136	0.11418	0.31541	0.99915	136	4	-0.20559
FXN	10	336	0.35038	0.67883	0.959063	336	6	-0.30931	330	0.5289	0.76558	0.99915	330	3	-0.30931
CYB5D2	10	337	0.3504	0.67885	0.959063	337	3	1.0761	106	0.084625	0.25693	0.99915	106	4	1.0761
HBG2	3	338	0.35043	0.50429	0.940707	338	1	0.12977	280	0.41307	0.48879	0.99915	280	1	0.12977
LDHD	10	339	0.35121	0.67983	0.959063	339	6	-0.23836	57	0.032516	0.12618	0.99915	57	4	-0.23836
FADS2	10	340	0.35246	0.68127	0.959063	340	6	-0.63469	229	0.29159	0.5634	0.99915	229	4	-0.63469
SLC39A12	10	341	0.35384	0.68276	0.959063	341	5	0.5653	288	0.4233	0.68559	0.99915	288	4	0.5653
SDHD	10	342	0.3575	0.68644	0.960361	342	5	-0.11432	149	0.13594	0.35197	0.99915	149	3	-0.11432
ABL1	10	343	0.36018	0.68907	0.960361	343	5	0.76874	244	0.31759	0.58896	0.99915	244	4	0.76874
INPPL1	10	344	0.36072	0.6897	0.960361	344	4	0.68482	312	0.47016	0.72757	0.99915	312	4	0.68482
CYB561A3	10	345	0.37022	0.69935	0.9647	345	4	0.097668	283	0.41525	0.67814	0.99915	283	4	0.097668
ATP6V1G1	10	346	0.37104	0.70015	0.9647	346	5	0.097774	66	0.04001	0.14604	0.99915	66	4	0.097774
CYB561	10	347	0.37354	0.70275	0.9647	347	4	1.4396	174	0.16755	0.40091	0.99915	174	5	1.4396
CNNM2	10	348	0.37384	0.70308	0.9647	348	5	0.5018	375	0.67813	0.85205	0.99915	375	3	0.5018
ALOXE3	10	349	0.37921	0.70794	0.9647	349	4	1.1713	230	0.29169	0.56352	0.99915	230	4	1.1713
NPAS2	10	350	0.3818	0.70979	0.9647	350	4	1.4001	121	0.099501	0.29005	0.99915	121	5	1.4001
EML6	10	351	0.38259	0.71029	0.9647	351	2	2.0969	24	0.015457	0.069525	0.99915	24	7	2.0969
KIF4B	10	352	0.38405	0.71143	0.9647	352	5	-0.09236	374	0.67728	0.85157	0.99915	374	3	-0.09236
KDM4D	10	353	0.38754	0.71397	0.9647	353	5	-0.2252	135	0.11388	0.31507	0.99915	135	3	-0.2252
STC2	10	354	0.39528	0.71975	0.9647	354	5	-0.36607	430	0.88076	0.94229	0.99915	430	2	-0.36607
CLSTN1	10	355	0.39573	0.72007	0.9647	355	4	2.4089	70	0.04376	0.15607	0.99915	70	6	2.4089
ALKBH8	10	356	0.39924	0.72253	0.9647	356	3	1.8529	89	0.061344	0.2016	0.99915	89	5	1.8529
APLP1	10	357	0.40062	0.72362	0.9647	357	4	0.56113	232	0.29423	0.56621	0.99915	232	4	0.56113
KDM4C	10	358	0.40103	0.72396	0.9647	358	5	-0.12833	78	0.051471	0.17635	0.99915	78	3	-0.12833
ALOX12	10	359	0.40369	0.72595	0.9647	359	3	0.76344	245	0.31917	0.5905	0.99915	245	3	0.76344
ADO	10	360	0.40578	0.72746	0.9647	360	2	1.5566	213	0.25262	0.52537	0.99915	213	3	1.5566
DEGS1	10	361	0.40725	0.72851	0.9647	361	5	0.30101	167	0.16281	0.39381	0.99915	167	5	0.30101
CYP27C1	10	362	0.40803	0.72906	0.9647	362	5	0.24067	311	0.46488	0.7229	0.99915	311	3	0.24067

CYP26B1	10	363	0.42048	0.7379	0.970091	363	3	1.6393	185	0.19171	0.43759	0.99915	185	5	1.6393
CYP26A1	10	364	0.42235	0.73921	0.970091	364	5	0.42188	20	0.011214	0.053181	0.99915	20	5	0.42188
IDO2	9	365	0.43314	0.73587	0.970091	365	2	1.7267	175	0.16969	0.40008	0.99915	175	5	1.7267
CTRL	1004	366	0.44066	0.75271	0.97408	366	398	1.3038	1	8.72E-14	4.97E-06	0.002381	1	465	1.3038
NIPA1	10	367	0.44271	0.75402	0.97408	367	4	1.166	180	0.18181	0.42256	0.99915	180	4	1.166
UTY	10	368	0.44576	0.75626	0.97408	368	3	1.5169	231	0.29342	0.5654	0.99915	231	4	1.5169
FA2H	10	369	0.44842	0.75813	0.97408	369	5	-0.42902	147	0.13266	0.34681	0.99915	147	3	-0.42902
NOX3	10	370	0.45125	0.76025	0.97408	370	5	0.22631	184	0.19118	0.43676	0.99915	184	3	0.22631
STEAP4	10	371	0.45343	0.7618	0.97408	371	3	0.99616	252	0.32922	0.60022	0.99915	252	4	0.99616
MOCS1	10	372	0.45598	0.76359	0.97408	372	4	1.0124	255	0.3395	0.60978	0.99915	255	4	1.0124
CYB5R1	10	373	0.45854	0.7655	0.97408	373	5	-0.17095	298	0.44336	0.70372	0.99915	298	3	-0.17095
CYBRD1	10	374	0.45881	0.76565	0.97408	374	5	0.2327	157	0.15031	0.37431	0.99915	157	4	0.2327
NEK6	10	375	0.46087	0.767	0.97408	375	2	2.4541	28	0.018296	0.079556	0.99915	28	7	2.4541
RORB	10	376	0.46285	0.76839	0.97408	376	5	-0.29641	279	0.41197	0.67535	0.99915	279	3	-0.29641
NOX5	10	377	0.46328	0.76869	0.97408	377	5	-0.08588	301	0.45088	0.71043	0.99915	301	3	-0.08588
RSAD2	10	378	0.46688	0.77173	0.975355	378	5	0.3272	143	0.12741	0.33804	0.99915	143	4	0.3272
TET1	10	379	0.4748	0.77712	0.977179	379	5	0.21616	137	0.11574	0.31817	0.99915	137	4	0.21616
KLKB1	10	380	0.47688	0.77847	0.977179	380	5	0.26442	200	0.2293	0.49209	0.99915	200	4	0.26442
CYBB	10	381	0.47937	0.77997	0.977179	381	4	0.76553	303	0.45286	0.71211	0.99915	303	3	0.76553
NOS3	10	382	0.48177	0.78134	0.977179	382	5	0.34906	129	0.10814	0.30538	0.99915	129	4	0.34906
PPP1CA	10	383	0.4912	0.78822	0.979099	383	4	0.57055	282	0.41482	0.67775	0.99915	282	3	0.57055
APBB2	10	384	0.49485	0.79063	0.979099	384	5	-0.01973	45	0.025035	0.10349	0.99915	45	4	-0.01973
HBD	10	385	0.49562	0.79116	0.979099	385	3	1.0431	218	0.26454	0.5381	0.99915	218	3	1.0431
CYP4F12	10	386	0.49922	0.79325	0.979099	386	5	-0.00907	368	0.66709	0.8454	0.99915	368	2	-0.00907
SLC39A10	10	387	0.50297	0.79595	0.979099	387	5	0.29799	241	0.31307	0.58461	0.99915	241	4	0.29799
PHYH	10	388	0.50398	0.79675	0.979099	388	3	3.2253	6	0.0012569	0.0075505	0.602778	6	7	3.2253
ALKBH2	10	389	0.50826	0.79995	0.979099	389	5	0.12127	343	0.57133	0.78967	0.99915	343	3	0.12127
CYP4X1	10	390	0.51944	0.80752	0.979099	390	2	2.5461	42	0.0241	0.10027	0.99915	42	7	2.5461
KDM3B	10	391	0.51957	0.80758	0.979099	391	4	1.1765	206	0.24378	0.51264	0.99915	206	4	1.1765
NUBPL	10	392	0.52111	0.80849	0.979099	392	3	1.345	355	0.63704	0.82771	0.99915	355	3	1.345
TYW1	10	393	0.52171	0.80883	0.979099	393	4	0.56704	248	0.32382	0.59503	0.99915	248	3	0.56704
NIF3L1	10	394	0.52213	0.80908	0.979099	394	4	1.3933	159	0.15308	0.37871	0.99915	159	5	1.3933
TSHZ2	10	395	0.52312	0.80977	0.979099	395	4	1.1869	97	0.073116	0.22981	0.99915	97	5	1.1869
TMLHE	10	396	0.52568	0.81149	0.979099	396	4	1.577	153	0.14026	0.35862	0.99915	153	4	1.577
RPE	10	397	0.53173	0.81559	0.980923	397	5	-0.27892	353	0.62551	0.82104	0.99915	353	2	-0.27892
CYP17A1	10	398	0.54368	0.82328	0.984271	398	3	0.97593	287	0.42113	0.68371	0.99915	287	3	0.97593
KDM5A	10	399	0.54494	0.82399	0.984271	399	5	0.3206	54	0.031442	0.12311	0.99915	54	4	0.3206
CD163	10	400	0.56005	0.83385	0.985942	400	4	1.8128	27	0.018294	0.079556	0.99915	27	5	1.8128
CYP4V2	10	401	0.56244	0.83558	0.985942	401	4	1.2793	122	0.10009	0.29106	0.99915	122	4	1.2793
ENAH	10	402	0.56324	0.8361	0.985942	402	4	1.4435	202	0.23645	0.50213	0.99915	202	4	1.4435
FRRS1L	10	403	0.56382	0.83642	0.985942	403	5	-0.01588	341	0.56899	0.7883	0.99915	341	2	-0.01588
NDFIP1	10	404	0.56426	0.83665	0.985942	404	5	0.80828	82	0.055216	0.1862	0.99915	82	5	0.80828
OGFOD2	10	405	0.56596	0.83774	0.985942	405	3	1.8044	115	0.094187	0.27813	0.99915	115	5	1.8044
LYRM7	8	406	0.57006	0.81709	0.980923	406	2	1.3777	404	0.75971	0.86494	0.99915	404	2	1.3777
TH	10	407	0.57362	0.84282	0.986808	407	3	1.7684	117	0.095149	0.28051	0.99915	117	6	1.7684
DPH2	10	408	0.57575	0.84427	0.986808	408	5	0.32029	266	0.37571	0.6422	0.99915	266	4	0.32029
UBE2D1	10	409	0.57924	0.84647	0.986808	409	5	0.74723	292	0.4304	0.69212	0.99915	292	4	0.74723
LCN2	10	410	0.57998	0.84699	0.986808	410	5	-0.06874	284	0.41588	0.67874	0.99915	284	3	-0.06874
KAT2B	10	411	0.58576	0.85073	0.986808	411	3	0.9673	265	0.37503	0.64164	0.99915	265	3	0.9673
KDM4E	10	412	0.5924	0.85505	0.986808	412	5	0.32052	40	0.023141	0.096883	0.99915	40	4	0.32052
HEPH	10	413	0.59339	0.8558	0.986808	413	4	0.6421	274	0.4021	0.6665	0.99915	274	3	0.6421
CYP2A13	10	414	0.59914	0.85926	0.986808	414	4	0.23844	323	0.49374	0.74693	0.99915	323	3	0.23844
CYP3A7	10	415	0.60152	0.86099	0.986808	415	4	1.7573	86	0.058705	0.19493	0.99915	86	5	1.7573
SLC22A17	10	416	0.60194	0.86122	0.986808	416	4	0.82813	398	0.74437	0.89106	0.99915	398	3	0.82813
CYP26C1	10	417	0.60578	0.86354	0.986808	417	4	0.27033	90	0.061519	0.20201	0.99915	90	3	0.27033
FRRS1	10	418	0.60796	0.86475	0.986808	418	1	2.4795	65	0.039947	0.14588	0.99915	65	6	2.4795
LEPREL1	10	419	0.60889	0.86526	0.986808	419	5	0.35482	408	0.77875	0.90189	0.99915	408	1	0.35482
PGRMC1	10	420	0.61877	0.87088	0.990369	420	5	0.61995	191	0.20254	0.45388	0.99915	191	4	0.61995
CISD1	10	421	0.62319	0.87364	0.990369	421	4	0.96211	225	0.28531	0.55743	0.99915	225	4	0.96211
CISD2	10	422	0.62888	0.87597	0.990369	422	4	0.74822	259	0.36078	0.62901	0.99915	259	4	0.74822
SLC39A9	10	423	0.63055	0.87665	0.990369	423	4	0.9854	316	0.47724	0.73338	0.99915	316	4	0.9854
TPH2	10	424	0.63612	0.87907	0.990762	424	3	1.454	182	0.18568	0.42815	0.99915	182	5	1.454
KIAA1456	10	425	0.65335	0.88566	0.992825	425	3	0.98148	195	0.21301	0.46871	0.99915	195	3	0.98148
ALKBH6	10	426	0.65926	0.88814	0.992825	426	4	0.36927	120	0.099125	0.28941	0.99915	120	4	0.36927
TPH1	10	427	0.67346	0.89403	0.992825	427	3	2.0222	91	0.061521	0.20202	0.99915	91	5	2.0222
CYB5RL	10	428	0.67527	0.89469	0.992825	428	3	1.7429	17	0.0090777	0.044323	0.99915	17	5	1.7429
CYP2F1	10	429	0.67574	0.89489	0.992825	429	4	1.9248	145	0.13007	0.34265	0.99915	145	5	1.9248
BCO2	10	430	0.6846	0.89853	0.992825	430	3	1.9383	99	0.07483	0.23359	0.99915	99	6	1.9383
CYP2C9	10	431	0.68528	0.89877	0.992825	431	4	0.55339	306	0.4565	0.71538	0.99915	306	3	0.55339
CYB5R2	10	432	0.69851	0.90382	0.992825	432	2	2.4425	51	0.0298	0.11837	0.99915	51	6	2.4425
HIF1AN	10	433	0.69961	0.90436	0.992825	433	4	0.58484	371	0.67346	0.84936	0.99915	371	3	0.58484
CYB5A	10	434	0.7037	0.906	0.992825	434	4	1.4472	71	0.0447	0.15862	0.99915	71	5	1.4472
NOS2	10	435	0.70425	0.90623	0.992825	435	4	1.1033	221	0.27723	0.55003	0.99915	221	4	1.1033
SC5D	10	436	0.70748	0.90742	0.992825	436	3	2.3654	60	0.035194	0.1333	0.99915	60	6	2.3654
VWF	10	437	0.71926	0.91217	0.992825	437	3	1.8403	114	0.09291	0.27524	0.99915	114	6	1.8403
CYP2D6	10	438	0.7222	0.91345	0.992825	438	4	0.16861	394	0.72082	0.87695	0.99915	394	3	0.16861
TMPPE	10	439	0.72285	0.91376	0.992825	439	2	1.7496	197	0.21927	0.47779	0.99915	197	5	1.7496
GLRX2	10	440	0.72354	0.91406	0.992825	440	3	3.0766	19	0.010054	0.048658	0.99915	19	7	3.0766
HBG1	3	441	0.72473	0.76236	0.97408	441	1	2.8745	155	0.14599	0.24198	0.99915	155	2	2.8745
NOX4	10	442	0.74437	0.9225	0.99492	442	4	0.60177	426	0.8618	0.93368	0.99915	426	2	0.60177
CDO1	10	443	0.75024	0.92486	0.99492	443	3	2.9904	21	0.011825	0.055537	0.99915	21	7	2.9904
RRM2B	10	444	0.75459	0.92661	0.99492	444	3	0.98085	336	0.54544	0.77465	0.99915	336	3	0.98085
MCOLN1	10	445	0.77651	0.93535	0.99492	445	4	0.75592	139	0.12038	0.32563	0.99915	139	1	0.75592
ADI1	10	446	0.78249	0.93796	0.99492	446	3	1.3847	56	0.032256	0.12555	0.99915	56	5	1.3847
ADHFE1	10	447	0.78285	0.9381	0.99492	447	3	2.0048	152	0.1396	0.35751	0.99915	152	5	2.0048
CYP2A6	10	448	0.78448	0.93868	0.99492	448	2	3.2288	8	0.0021898	0.012611	0.722487	8	7	3.2288
BACH1	10	449	0.78747	0.93975	0.99492	449	3	1.2877	173	0.16723	0.40049	0.99915	173	5	1.2877
CYP2A7	10	450	0.7883	0.94001	0.99492	450	3	2.236	50	0.029506	0.11753	0.99915	50	6	2.236
PTGIS	10	451	0.78832	0.94002	0.99492	451	4	1.0986	68	0.04077	0.14779	0.99915	68	4	1.0986
PTGS2	10	452	0.79346	0.94182	0.99492	452	4	1.7183	193	0.20649	0.45964	0.99915	193	5	1.7183
PTGS1	10	453	0.79415	0.94214	0.99492	453	4	0.57562	150	0.13723	0.35384	0.99915	150	3	0.57562

CYBA	10	454	0.80565	0.94649	0.99492	454	4	2.026	102	0.078568	0.24251	0.99915	102	5	2.026
LYZ	10	455	0.80992	0.94823	0.99492	455	4	0.7889	154	0.14131	0.36033	0.99915	154	4	0.7889
CYP21A2	10	456	0.81323	0.94957	0.99492	456	4	0.65465	208	0.24839	0.51915	0.99915	208	4	0.65465
CYP2U1	10	457	0.81383	0.94977	0.99492	457	4	1.0828	124	0.1019	0.29429	0.99915	124	4	1.0828
NARF	10	458	0.82296	0.95368	0.99492	458	4	0.9423	130	0.10885	0.30649	0.99915	130	4	0.9423
GUCY1A3	10	459	0.82311	0.95378	0.99492	459	3	2.3449	67	0.040471	0.1471	0.99915	67	6	2.3449
CYP3A4	10	460	0.82784	0.95554	0.99492	460	4	0.26831	190	0.20217	0.45337	0.99915	190	4	0.26831
TET3	10	461	0.84998	0.96079	0.99492	461	3	1.8282	176	0.17391	0.41092	0.99915	176	5	1.8282
SLC39A1	10	462	0.86154	0.96344	0.99492	462	2	2.1931	38	0.021612	0.091724	0.99915	38	5	2.1931
CYP3A43	10	463	0.86239	0.96364	0.99492	463	3	2.8381	23	0.01457	0.065996	0.99915	23	6	2.8381
SLC46A1	10	464	0.86412	0.96411	0.99492	464	3	1.0927	211	0.24965	0.52078	0.99915	211	4	1.0927
STEAP2	10	465	0.88839	0.97051	0.99492	465	2	2.8474	29	0.018601	0.080639	0.99915	29	6	2.8474
TPO	10	466	0.89186	0.97131	0.99492	466	3	0.90699	111	0.089866	0.2685	0.99915	111	4	0.90699
RASD1	10	467	0.89993	0.97351	0.99492	467	3	1.5408	172	0.16712	0.40029	0.99915	172	5	1.5408
RORA	10	468	0.90726	0.97545	0.99492	468	1	3.9561	2	4.18E-05	0.00031315	0.075	2	8	3.9561
STEAP1B	10	469	0.91689	0.97812	0.99492	469	3	1.6332	116	0.094254	0.27835	0.99915	116	5	1.6332
HDAC2	10	470	0.91748	0.97823	0.99492	470	3	2.1506	95	0.065945	0.21267	0.99915	95	6	2.1506
HEPHL1	10	471	0.92004	0.97882	0.99492	471	3	0.57206	297	0.44167	0.70215	0.99915	297	2	0.57206
CYP20A1	10	472	0.92529	0.98038	0.99492	472	3	1.9774	59	0.034984	0.1329	0.99915	59	6	1.9774
TBXAS1	10	473	0.93948	0.98456	0.997045	473	3	2.1864	75	0.049341	0.17089	0.99915	75	6	2.1864
MOXD1	10	474	0.96098	0.989	0.999432	474	1	3.7985	5	0.00040772	0.0026991	0.258571	5	7	3.7985
CYP39A1	10	475	0.97253	0.99141	0.999528	475	2	3.2188	13	0.0061277	0.031728	0.99915	13	7	3.2188
CYP4F3	10	476	0.9827	0.9943	0.999528	476	2	2.0994	87	0.059588	0.19717	0.99915	87	6	2.0994
ATOX1	10	477	0.98876	0.99617	0.999528	477	2	1.9283	158	0.15188	0.37674	0.99915	158	5	1.9283
PPP3CC	10	478	0.99855	0.99869	0.999528	478	1	3.727	3	0.00027917	0.0019734	0.250595	3	8	3.727
CYP2C8	10	479	0.99949	0.99953	0.999528	479	0	2.816	43	0.024116	0.10028	0.99915	43	6	2.816

Total dropped sgRNAs
2884

Supplemental Table S3. Result of MAGeCK test of Mouse #2

id	num	Rank	neg|score	neg|p-valu	neg|fdr	neg|rank	neg|goodsgrna	neg|lfc	Rank2	pos|score	pos|p-valu	pos|fdr	pos|rank	pos|good:	pos|lfc
CIAO1	10	1	2.77E-05	0.000214	0.102381	1	8	-1.2355	462	0.94565	0.97711	0.999915	462	2	-1.2355
THAP4	10	2	7.76E-05	0.000601	0.144048	2	8	-1.4486	387	0.61581	0.84209	0.999915	387	2	-1.4486
NDUFV1	10	3	0.00014789	0.001208	0.174762	3	10	-1.3459	479	0.99985	0.99992	0.999915	479	0	-1.3459
CYP4F22	10	4	0.00022557	0.001794	0.174762	4	6	-1.4506	332	0.45808	0.73865	0.999915	332	3	-1.4506
FDXR	10	5	0.00022925	0.001824	0.174762	5	9	-1.2211	471	0.99254	0.99335	0.999915	471	1	-1.2211
IBA57	10	6	0.00038219	0.002709	0.202083	6	9	-1.1805	210	0.1975	0.47426	0.999915	210	1	-1.1805
NDOR1	10	7	0.00043523	0.002997	0.202083	7	7	-1.4713	396	0.64304	0.85957	0.999915	396	3	-1.4713
CYP27B1	10	8	0.0004929	0.003375	0.202083	8	8	-1.3975	361	0.55243	0.80083	0.999915	361	2	-1.3975
ASPHD2	10	9	0.00057766	0.003862	0.205556	9	9	-1.0943	433	0.81841	0.93376	0.999915	433	1	-1.0943
RRM2	10	10	0.00063055	0.00432	0.206905	10	9	-0.97037	478	0.9989	0.99902	0.999915	478	0	-0.97037
ERCC2	10	11	0.00071927	0.004896	0.213203	11	9	-1.0449	470	0.99229	0.99321	0.999915	470	1	-1.0449
OSGEP	10	12	0.00086666	0.005841	0.222527	12	7	-0.98632	472	0.99407	0.99457	0.999915	472	1	-0.98632
ABCB7	10	13	0.0009009	0.006039	0.222527	13	7	-1.3403	329	0.44959	0.73309	0.999915	329	3	-1.3403
KAT5	10	14	0.0012865	0.008445	0.252801	14	9	-1.0453	466	0.96524	0.98499	0.999915	466	1	-1.0453
CYP2E1	10	15	0.0013369	0.008654	0.252801	15	9	-1.1465	367	0.56326	0.80818	0.999915	367	1	-1.1465
HSCB	10	16	0.0013376	0.008654	0.252801	16	8	-1.0996	232	0.23781	0.52236	0.999915	232	2	-1.0996
KDM5B	10	17	0.0013868	0.008972	0.252801	17	3	3.1219	163	0.14417	0.38467	0.999915	163	6	3.1219
ELP3	10	18	0.00182	0.011607	0.308862	18	8	-1.0962	94	0.063155	0.22144	0.999915	94	2	-1.0962
NDUFS7	10	19	0.0020703	0.013267	0.313975	19	6	-1.2993	262	0.3068	0.60146	0.999915	262	4	-1.2993
ATP6V0D1	10	20	0.0021337	0.013654	0.313975	20	6	-1.2749	318	0.42046	0.71302	0.999915	318	4	-1.2749
SP1	10	21	0.0021898	0.014012	0.313975	21	8	-1.155	454	0.92041	0.96834	0.999915	454	2	-1.155
SDHC	10	22	0.0023047	0.014808	0.313975	22	8	-1.2183	142	0.11275	0.32538	0.999915	142	2	-1.2183
COX10	10	23	0.0023497	0.015076	0.313975	23	8	-1.0507	477	0.99859	0.99867	0.999915	477	0	-1.0507
JHDM1D	10	24	0.002598	0.016428	0.316071	24	6	-0.75066	338	0.46552	0.74385	0.999915	338	4	-0.75066
UQCRC1	10	25	0.0026637	0.016816	0.316071	25	8	-1.1997	439	0.82403	0.93623	0.999915	439	2	-1.1997
CYP2B6	10	26	0.0028619	0.017889	0.316071	26	7	-1.2743	459	0.93384	0.97282	0.999915	459	1	-1.2743
NARFL	10	27	0.0029037	0.018068	0.316071	27	8	-1.2095	295	0.35975	0.6573	0.999915	295	1	-1.2095
PPP1CA	10	28	0.0029652	0.018476	0.316071	28	9	-1.1714	464	0.95007	0.97887	0.999915	464	1	-1.1714
FDX1	10	29	0.004379	0.025246	0.405159	29	7	-1.2654	376	0.59044	0.82627	0.999915	376	3	-1.2654
GLRX5	10	30	0.0044088	0.025375	0.405159	30	8	-1.1676	414	0.70945	0.88816	0.999915	414	2	-1.1676
UQCRFS1	10	31	0.0047212	0.026916	0.411235	31	8	-0.90076	447	0.8781	0.95659	0.999915	447	2	-0.90076
SLC46A1	10	32	0.0048793	0.027473	0.411235	32	8	-1.0776	474	0.99652	0.99677	0.999915	474	0	-1.0776
ISCA2	10	33	0.0051377	0.028706	0.416667	33	5	1.3223	237	0.24252	0.52821	0.999915	237	5	1.3223
PRC1	10	34	0.0060109	0.032871	0.463095	34	7	-1.2401	330	0.45471	0.73642	0.999915	330	3	-1.2401
CCDC115	10	35	0.0063203	0.034511	0.472313	35	5	0.7791	187	0.17398	0.43684	0.999915	187	5	0.7791
MB	10	36	0.0068788	0.037265	0.481122	36	8	-1.0277	278	0.33597	0.63261	0.999915	278	2	-1.0277
EVL	10	37	0.0069058	0.037394	0.481122	37	8	-1.2273	359	0.54653	0.7969	0.999915	359	1	-1.2273
MIPEP	10	38	0.0072628	0.039035	0.481122	38	7	-1.0922	406	0.68887	0.88049	0.999915	406	2	-1.0922
CYP46A1	10	39	0.0073092	0.039204	0.481122	39	7	-1.1665	393	0.63612	0.85543	0.999915	393	3	-1.1665
NPAS2	10	40	0.0077757	0.041371	0.481122	40	5	-0.34759	438	0.82393	0.9362	0.999915	438	2	-0.34759
RSBN1	10	41	0.0079336	0.042136	0.481122	41	7	-1.151	271	0.32649	0.62315	0.999915	271	2	-1.151
NDUFS8	10	42	0.0079451	0.042186	0.481122	42	7	-0.88987	458	0.93108	0.97186	0.999915	458	1	-0.88987
CYP11A1	10	43	0.0094962	0.049573	0.548214	43	7	-1.0083	465	0.9606	0.98375	0.999915	465	1	-1.0083
LTF	10	44	0.0096556	0.050358	0.548214	44	6	-0.92552	252	0.27876	0.56968	0.999915	252	4	-0.92552
ATP6AP1	10	45	0.0099949	0.052098	0.55455	45	7	-1.1302	463	0.94648	0.97748	0.999915	463	2	-1.1302
FBXL5	10	46	0.011984	0.060886	0.615519	46	7	-1.0227	60	0.032509	0.13162	0.999915	60	3	-1.0227
CYP1B1	10	47	0.012014	0.061005	0.615519	47	6	-1.1141	213	0.20087	0.47851	0.999915	213	4	-1.1141
SLC39A6	10	48	0.012642	0.063649	0.615519	48	4	1.7824	195	0.18317	0.45203	0.999915	195	5	1.7824
HIF1AN	10	49	0.012929	0.064763	0.615519	49	5	-0.14849	220	0.22039	0.5017	0.999915	220	4	-0.14849
SDHD	10	50	0.013668	0.067974	0.615519	50	8	-1.0137	450	0.89195	0.96001	0.999915	450	1	-1.0137
HTT	10	51	0.013993	0.069107	0.615519	51	7	-0.85755	311	0.39814	0.69592	0.999915	311	3	-0.85755
EGLN1	10	52	0.014491	0.071205	0.615519	52	5	-0.84062	283	0.34223	0.6392	0.999915	283	3	-0.84062
ACP5	10	53	0.014617	0.071781	0.615519	53	8	-0.72403	435	0.82027	0.93462	0.999915	435	1	-0.72403
COQ6	10	54	0.01464	0.071891	0.615519	54	7	-1.0687	339	0.46975	0.74675	0.999915	339	3	-1.0687
ALKBH7	10	55	0.014642	0.071901	0.615519	55	5	-0.23395	46	0.023766	0.10316	0.999915	46	4	-0.23395
HEBP2	10	56	0.01466	0.07196	0.615519	56	4	1.5511	264	0.31562	0.61116	0.999915	264	5	1.5511
POLA1	10	57	0.01602	0.077657	0.629859	57	6	-1.073	358	0.54523	0.79598	0.999915	358	4	-1.073
SLC39A12	10	58	0.016207	0.078363	0.629859	58	7	-1.006	246	0.26058	0.54981	0.999915	246	3	-1.006
APP	10	59	0.01635	0.078949	0.629859	59	8	-0.82529	428	0.7792	0.9169	0.999915	428	2	-0.82529
CYP27A1	10	60	0.016351	0.078949	0.629859	60	4	1.8225	155	0.13179	0.36246	0.999915	155	5	1.8225
LYRM4	10	61	0.01667	0.080212	0.629859	61	7	-1.0152	417	0.72339	0.89367	0.999915	417	1	-1.0152
C16orf52	10	62	0.018043	0.085341	0.659332	62	6	-0.9732	415	0.7167	0.89105	0.999915	415	3	-0.9732
JMJD7	10	63	0.018974	0.08903	0.672314	63	8	-0.91115	468	0.98584	0.99	0.999915	468	1	-0.91115
HMOX2	10	64	0.019763	0.09244	0.672314	64	5	-0.04273	288	0.35616	0.65388	0.999915	288	4	-0.04273
NTHL1	10	65	0.019988	0.093324	0.672314	65	8	-0.76503	449	0.8903	0.95962	0.999915	449	1	-0.76503
SDHB	10	66	0.020097	0.093792	0.672314	66	6	-0.69401	443	0.83712	0.94237	0.999915	443	3	-0.69401
CISD3	10	67	0.020858	0.096824	0.672314	67	6	-0.93598	421	0.74474	0.90249	0.999915	421	3	-0.93598
CYP26A1	10	68	0.021465	0.098842	0.672314	68	6	-0.57903	267	0.31964	0.61547	0.999915	267	4	-0.57903
UQCRC2	10	69	0.022056	0.10069	0.672314	69	5	-0.16183	249	0.27588	0.56673	0.999915	249	4	-0.16183
JMJD6	10	70	0.022278	0.10136	0.672314	70	5	2.1953	43	0.022727	0.099597	0.999915	43	5	2.1953
NR1D1	10	71	0.022699	0.10254	0.672314	71	7	-1.1048	93	0.063101	0.22127	0.999915	93	3	-1.1048
TMEM199	10	72	0.023141	0.10397	0.672314	72	7	-0.68948	331	0.45688	0.73781	0.999915	331	1	-0.68948
GRXCR1	10	73	0.023228	0.10429	0.672314	73	6	-1.0477	365	0.56106	0.80663	0.999915	365	4	-1.0477
HBM	10	74	0.023993	0.10673	0.672314	74	6	-0.77	261	0.30626	0.60086	0.999915	261	3	-0.77
POLE	10	75	0.024589	0.10866	0.672314	75	7	-0.87648	448	0.88839	0.95924	0.999915	448	1	-0.87648
NFS1	10	76	0.024676	0.10892	0.672314	76	7	-0.9598	407	0.69666	0.88328	0.999915	407	1	-0.9598
KDM2A	10	77	0.024801	0.10933	0.672314	77	7	-0.90685	248	0.27369	0.56446	0.999915	248	3	-0.90685
CYP4F2	10	78	0.02486	0.10948	0.672314	78	3	2.1039	204	0.1909	0.4655	0.999915	204	6	2.1039
SLC11A2	10	79	0.026554	0.1146	0.694846	79	6	-0.67451	360	0.54661	0.79692	0.999915	360	2	-0.67451
CYB561D1	10	80	0.027162	0.1168	0.699315	80	6	-0.80461	388	0.61833	0.84376	0.999915	388	3	-0.80461
PTGES2	10	81	0.027917	0.11892	0.703263	81	7	-0.98366	325	0.43548	0.72344	0.999915	325	3	-0.98366
MYLIP	10	82	0.028855	0.12208	0.705423	82	5	-0.62169	52	0.026554	0.11179	0.999915	52	4	-0.62169
KDM4A	10	83	0.029297	0.12345	0.705423	83	7	-1.0283	411	0.70774	0.88757	0.999915	411	2	-1.0283
ALKBH1	10	84	0.029611	0.12454	0.705423	84	6	-1.0688	416	0.71844	0.89164	0.999915	416	2	-1.0688
KDM4B	10	85	0.029934	0.1255	0.705423	85	6	-1.0053	275	0.33134	0.62811	0.999915	275	4	-1.0053
DPH3	10	86	0.030896	0.12857	0.705423	86	6	-0.92681	461	0.93991	0.97494	0.999915	461	2	-0.92681
FADS1	10	87	0.030903	0.1286	0.705423	87	7	-0.85848	154	0.13114	0.36142	0.999915	154	2	-0.85848
RTEL1	10	88	0.03141	0.13037	0.705423	88	7	-1.0346	181	0.16712	0.42513	0.999915	181	3	-1.0346
CYB561D2	10	89	0.031727	0.13129	0.705423	89	6	-0.94338	473	0.99645	0.99671	0.999915	473	0	-0.94338
SCD5	10	90	0.03212	0.13254	0.705423	90	7	-0.71734	440	0.8348	0.94126	0.999915	440	2	-0.71734
CYC1	10	91	0.032715	0.13447	0.707823	91	5	-0.41869	269	0.32382	0.62016	0.999915	269	4	-0.41869
EGLN2	10	92	0.035156	0.14179	0.712676	92	7	-0.87348	167	0.14479	0.38556	0.999915	167	3	-0.87348
GLRX3	10	93	0.036067	0.14439	0.712676	93	7	-0.90645	469	0.98768	0.99073	0.999915	469	1	-0.90645
POLD1	10	94	0.036121	0.14447	0.712676	94	7	-0.96803	303	0.37823	0.67603	0.999915	303	2	-0.96803

CYP4Z1	10	95	0.036662	0.146	0.712676	95	5	-0.32021	180	0.16707	0.42506	0.999915	180	4	-0.32021
DGCR8	10	96	0.03702	0.14705	0.712676	96	7	-0.96063	251	0.27758	0.56843	0.999915	251	1	-0.96063
LEPRE1	10	97	0.038188	0.1506	0.712676	97	7	-0.84596	413	0.70938	0.88815	0.999915	413	3	-0.84596
NR1D2	10	98	0.038442	0.15131	0.712676	98	3	3.8682	116	0.086017	0.27195	0.999915	116	6	3.8682
DDX5	10	99	0.03902	0.15297	0.712676	99	5	-0.42917	205	0.19185	0.46689	0.999915	205	4	-0.42917
SLC25A28	10	100	0.039183	0.1535	0.712676	100	7	-0.81814	345	0.49088	0.76018	0.999915	345	3	-0.81814
NUBP1	10	101	0.039384	0.15404	0.712676	101	7	-0.99448	104	0.07292	0.24435	0.999915	104	3	-0.99448
CISD1	10	102	0.04001	0.15586	0.712676	102	5	-0.23145	286	0.35509	0.6527	0.999915	286	1	-0.23145
LYRM7	8	103	0.040695	0.14331	0.712676	103	6	-0.82708	467	0.97082	0.97622	0.999915	467	1	-0.82708
DPH2	10	104	0.040744	0.15778	0.712676	104	6	-0.94052	378	0.59916	0.83186	0.999915	378	4	-0.94052
ALOX12	10	105	0.040961	0.15848	0.712676	105	6	-0.60535	455	0.92474	0.9696	0.999915	455	1	-0.60535
RPE65	10	106	0.040973	0.1585	0.712676	106	6	-0.95129	403	0.67965	0.87673	0.999915	403	2	-0.95129
INPPL1	10	107	0.041552	0.16019	0.712676	107	7	-0.9379	240	0.24592	0.53229	0.999915	240	3	-0.9379
ADI1	10	108	0.04168	0.16069	0.712676	108	4	1.5031	255	0.29381	0.58659	0.999915	255	5	1.5031
STEAP4	10	109	0.043348	0.16589	0.72803	109	4	2.9122	184	0.16994	0.42996	0.999915	184	6	2.9122
RSBN1L	10	110	0.043791	0.16719	0.72803	110	7	-0.80626	133	0.10794	0.31613	0.999915	133	2	-0.80626
TMEM62	10	111	0.044529	0.16944	0.731167	111	7	-0.95152	397	0.64422	0.86028	0.999915	397	3	-0.95152
STEAP2	10	112	0.045588	0.17255	0.737946	112	3	0.40967	199	0.18733	0.45906	0.999915	199	4	0.40967
MFI2	10	113	0.047018	0.17644	0.741374	113	6	-0.94287	446	0.86281	0.95249	0.999915	446	2	-0.94287
IDO2	9	114	0.048099	0.17561	0.741374	114	4	0.36497	239	0.24403	0.51598	0.999915	239	5	0.36497
CP	10	115	0.049683	0.18446	0.752761	115	5	-0.13696	409	0.70428	0.88631	0.999915	409	3	-0.13696
ETFDH	10	116	0.049762	0.18471	0.752761	116	3	0.92433	215	0.20727	0.48598	0.999915	215	5	0.92433
SLC39A13	10	117	0.049811	0.18482	0.752761	117	5	-0.52687	352	0.51023	0.77297	0.999915	352	3	-0.52687
FTH1	10	118	0.050565	0.18693	0.752761	118	6	-0.59068	404	0.68048	0.87703	0.999915	404	3	-0.59068
FTO	10	119	0.050612	0.18701	0.752761	119	6	-0.67418	203	0.19036	0.46447	0.999915	203	4	-0.67418
CACNA1C	10	120	0.051953	0.1909	0.759366	120	5	-0.76035	341	0.47205	0.74825	0.999915	341	4	-0.76035
LRP2	10	121	0.05231	0.19182	0.759366	121	5	2.1165	119	0.091261	0.28209	0.999915	119	5	2.1165
COQ7	10	122	0.054514	0.19816	0.778005	122	4	-0.19212	185	0.17298	0.43535	0.999915	185	4	-0.19212
PHF2	10	123	0.056594	0.20422	0.795296	123	2	3.8312	20	0.0080677	0.042464	0.999915	20	5	3.8312
MRE11A	10	124	0.057175	0.20602	0.795833	124	5	-0.57058	323	0.43493	0.723	0.999915	323	3	-0.57058
NFU1	10	125	0.05988	0.21317	0.816857	125	5	-0.25567	336	0.46298	0.7419	0.999915	336	3	-0.25567
PHF8	10	126	0.062479	0.22024	0.823792	126	6	-0.89106	453	0.91578	0.96687	0.999915	453	2	-0.89106
RSAD2	10	127	0.063155	0.222	0.823792	127	4	-0.04395	425	0.76734	0.9118	0.999915	425	3	-0.04395
ISCU	10	128	0.063435	0.22283	0.823792	128	7	-0.94941	418	0.73162	0.89682	0.999915	418	2	-0.94941
KDM2B	10	129	0.063629	0.2234	0.823792	129	6	-0.81808	198	0.18541	0.45578	0.999915	198	2	-0.81808
CYB561A3	10	130	0.064071	0.22469	0.823792	130	6	-0.61865	308	0.3893	0.68717	0.999915	308	3	-0.61865
OGFOD3	10	131	0.064968	0.22692	0.823792	131	3	2.12	226	0.23019	0.51338	0.999915	226	5	2.12
UTY	10	132	0.066151	0.23006	0.823792	132	6	-0.74934	451	0.89654	0.96117	0.999915	451	2	-0.74934
SET	10	133	0.066589	0.23127	0.823792	133	6	-0.83953	432	0.81705	0.93314	0.999915	432	3	-0.83953
DDX11	10	134	0.067805	0.23416	0.823792	134	6	-0.82155	333	0.45913	0.73928	0.999915	333	3	-0.82155
HPD	10	135	0.068009	0.23471	0.823792	135	5	-0.6542	423	0.75246	0.90581	0.999915	423	2	-0.6542
COX15	10	136	0.068362	0.23554	0.823792	136	7	-0.76884	139	0.11163	0.32344	0.999915	139	3	-0.76884
SOD2	10	137	0.068383	0.23561	0.823792	137	6	-0.91869	437	0.82281	0.93568	0.999915	437	2	-0.91869
METAP1D	10	138	0.069365	0.23824	0.826932	138	7	-0.82186	354	0.5181	0.77813	0.999915	354	3	-0.82186
VWF	10	139	0.071533	0.24393	0.83289	139	7	-0.58192	444	0.84588	0.94628	0.999915	444	2	-0.58192
CYP2S1	10	140	0.07169	0.24432	0.83289	140	7	-0.6291	159	0.13571	0.36961	0.999915	159	2	-0.6291
EXO5	10	141	0.073268	0.24863	0.83289	141	6	-0.85873	370	0.56824	0.81142	0.999915	370	4	-0.85873
GALT	10	142	0.07343	0.24893	0.83289	142	7	-0.59443	475	0.99677	0.99698	0.999915	475	0	-0.59443
FTMT	10	143	0.074093	0.25072	0.83289	143	5	-0.49172	111	0.07907	0.25737	0.999915	111	4	-0.49172
MCOLN1	10	144	0.074538	0.25189	0.83289	144	3	4.7183	51	0.025824	0.10958	0.999915	51	6	4.7183
HRG	10	145	0.074656	0.25213	0.83289	145	6	-0.81401	79	0.046675	0.17404	0.999915	79	3	-0.81401
TMPPE	10	146	0.077166	0.25875	0.848907	146	7	-0.54517	284	0.34876	0.64618	0.999915	284	2	-0.54517
KIF4A	10	147	0.079378	0.26437	0.85112	147	5	-0.27139	124	0.095315	0.2908	0.999915	124	4	-0.27139
CNNM2	10	148	0.079574	0.26485	0.85112	148	5	-0.52852	368	0.56573	0.80965	0.999915	368	3	-0.52852
COX5A	10	149	0.080002	0.26589	0.85112	149	4	0.36036	313	0.40104	0.69886	0.999915	313	4	0.36036
MMS19	10	150	0.080987	0.26837	0.85112	150	6	-0.64738	337	0.46384	0.74239	0.999915	337	4	-0.64738
PIR	10	151	0.082107	0.27113	0.85112	151	4	0.092504	372	0.57812	0.81779	0.999915	372	4	0.092504
HBE1	10	152	0.08362	0.2749	0.85112	152	4	1.5249	70	0.040779	0.15612	0.999915	70	5	1.5249
PER1	10	153	0.084193	0.27594	0.85112	153	6	-0.58278	382	0.60402	0.83506	0.999915	382	4	-0.58278
ALKBH2	10	154	0.084196	0.27595	0.85112	154	4	2.2474	68	0.039212	0.15173	0.999915	68	5	2.2474
CYCS	10	155	0.086007	0.27962	0.85112	155	6	-0.76838	442	0.83541	0.94152	0.999915	442	3	-0.76838
DOHH	10	156	0.08698	0.2815	0.85112	156	6	-0.78861	457	0.9295	0.97129	0.999915	457	1	-0.78861
KIF4B	10	157	0.087302	0.28211	0.85112	157	6	-0.72591	38	0.019763	0.089944	0.999915	38	3	-0.72591
CYB5B	10	158	0.087774	0.28305	0.85112	158	6	-0.73859	250	0.27655	0.56749	0.999915	250	3	-0.73859
NOX3	10	159	0.088173	0.28383	0.85112	159	5	1.7439	80	0.048568	0.1799	0.999915	80	5	1.7439
ALKBH4	10	160	0.092155	0.2913	0.85112	160	5	-0.09241	176	0.15825	0.41002	0.999915	176	4	-0.09241
P4HTM	10	161	0.092175	0.29133	0.85112	161	5	-0.44787	342	0.48	0.75304	0.999915	342	3	-0.44787
KDM8	10	162	0.093034	0.29314	0.85112	162	6	-0.65376	408	0.70214	0.88531	0.999915	408	3	-0.65376
BRIP1	10	163	0.09474	0.2964	0.85112	163	6	-0.74054	353	0.51341	0.77519	0.999915	353	4	-0.74054
P4HA3	10	164	0.094944	0.29686	0.85112	164	6	-0.81739	436	0.8225	0.93562	0.999915	436	2	-0.81739
EIF2AK1	10	165	0.096125	0.29928	0.85112	165	6	-0.73397	289	0.35626	0.654	0.999915	289	2	-0.73397
CA6	10	166	0.096592	0.30021	0.85112	166	6	-0.74289	441	0.83513	0.94137	0.999915	441	3	-0.74289
ACO2	10	167	0.096937	0.30089	0.85112	167	6	-0.76208	309	0.39594	0.6939	0.999915	309	3	-0.76208
BCMO1	10	168	0.097215	0.3013	0.85112	168	6	-0.80342	82	0.049992	0.18408	0.999915	82	2	-0.80342
EML6	10	169	0.098469	0.30404	0.85112	169	6	-0.76759	460	0.93618	0.97357	0.999915	460	2	-0.76759
SLC39A5	10	170	0.10125	0.30961	0.85112	170	5	-0.6124	424	0.75314	0.90603	0.999915	424	3	-0.6124
STEAP1	10	171	0.10167	0.31035	0.85112	171	5	2.0539	136	0.11107	0.32233	0.999915	136	5	2.0539
APBB1	10	172	0.10186	0.31071	0.85112	172	6	-0.81158	476	0.99691	0.9971	0.999915	476	0	-0.81158
CYP26B1	10	173	0.10386	0.31438	0.85112	173	5	-0.19995	422	0.75098	0.90512	0.999915	422	2	-0.19995
PLOD2	10	174	0.10481	0.3162	0.85112	174	3	5.3962	42	0.021596	0.096178	0.999915	42	7	5.3962
TSHZ2	10	175	0.10638	0.31943	0.85112	175	3	0.33875	216	0.20771	0.48646	0.999915	216	4	0.33875
FECH	10	176	0.10699	0.32049	0.85112	176	4	-0.33532	351	0.50639	0.77029	0.999915	351	4	-0.33532
CYP4F12	10	177	0.10794	0.32216	0.85112	177	5	-0.5062	429	0.78712	0.92019	0.999915	429	3	-0.5062
DEGS1	10	178	0.10951	0.32515	0.85112	178	5	-0.03366	320	0.42398	0.71548	0.999915	320	4	-0.03366
OGFOD2	10	179	0.11263	0.33093	0.85112	179	4	2.7965	110	0.077874	0.25473	0.999915	110	5	2.7965
HBQ1	10	180	0.11526	0.33583	0.85112	180	6	-0.79775	236	0.24052	0.52577	0.999915	236	4	-0.79775
ABL1	10	181	0.11595	0.33704	0.85112	181	6	-0.78263	347	0.49655	0.76367	0.999915	347	3	-0.78263
CISD2	10	182	0.11675	0.33836	0.85112	182	6	-0.77499	228	0.23099	0.51428	0.999915	228	3	-0.77499
TF	10	183	0.11689	0.33859	0.85112	183	6	-0.69601	379	0.59968	0.83215	0.999915	379	3	-0.69601
CYP3A7	10	184	0.11729	0.33932	0.85112	184	3	1.914	77	0.045174	0.16983	0.999915	77	5	1.914
ALKBH5	10	185	0.11783	0.34032	0.85112	185	6	-0.66486	427	0.77012	0.91279	0.999915	427	3	-0.66486
ABCG2	10	186	0.11786	0.34036	0.85112	186	5	-0.38805	380	0.60119	0.8331	0.999915	380	1	-0.38805
ADO	10	187	0.1183	0.34116	0.85112	187	6	-0.68147	398	0.64671	0.86189	0.999915	398	4	-0.68147
HBB	10	188	0.11898	0.34242	0.85112	188	5	-0.15073	349	0.50077	0.76653	0.999915	349	4	-0.15073
PPAT	10	189	0.11938	0.34325	0.85112	189	5	1.7563	212	0.20058	0.47823	0.999915	212	5	1.7563
HBZ	10	190	0.11961	0.34367	0.85112	190	5	1.028	268	0.32231	0.61856	0.999915	268	5	1.028

FABP7	10	191	0.12303	0.35044	0.85112	191	6	-0.60171	381	0.60348	0.83473	0.999915	381	3	-0.60171
CYP7B1	10	192	0.12398	0.35209	0.85112	192	5	2.0334	28	0.012929	0.064564	0.999915	28	5	2.0334
CYB5R1	10	193	0.1246	0.35333	0.85112	193	6	-0.62202	362	0.55411	0.80203	0.999915	362	4	-0.62202
NOS1	10	194	0.12478	0.35368	0.85112	194	6	-0.70317	235	0.23917	0.52401	0.999915	235	2	-0.70317
CIAPIN1	10	195	0.12501	0.35409	0.85112	195	4	0.69261	307	0.38338	0.68095	0.999915	307	5	0.69261
SLC39A9	10	196	0.12584	0.35572	0.85112	196	5	-0.4523	280	0.33974	0.63666	0.999915	280	3	-0.4523
HCCS	10	197	0.12616	0.35636	0.85112	197	5	-0.45066	377	0.59586	0.82968	0.999915	377	3	-0.45066
CYBRD1	10	198	0.12654	0.35704	0.85112	198	2	4.8748	72	0.042389	0.16115	0.999915	72	7	4.8748
JMJD4	10	199	0.1271	0.35807	0.85112	199	5	0.43953	260	0.30305	0.59719	0.999915	260	5	0.43953
CYBA	10	200	0.13114	0.36579	0.85112	200	4	3.6707	7	0.0019661	0.012481	0.854082	7	5	3.6707
HDAC1	10	201	0.13148	0.36628	0.85112	201	5	-0.50175	27	0.011296	0.057675	0.999915	27	4	-0.50175
SRC	10	202	0.13197	0.36713	0.85112	202	6	-0.65435	229	0.23105	0.51437	0.999915	229	4	-0.65435
FA2H	10	203	0.13408	0.37069	0.85112	203	4	-0.26913	328	0.44934	0.73286	0.999915	328	4	-0.26913
MARK3	10	204	0.13534	0.37304	0.85112	204	3	2.3499	222	0.22506	0.50744	0.999915	222	5	2.3499
JMJD1C	10	205	0.13571	0.37367	0.85112	205	2	5.8812	13	0.0051805	0.028487	0.965406	13	7	5.8812
COPA	10	206	0.13635	0.37473	0.85112	206	5	-0.39611	304	0.37899	0.67681	0.999915	304	4	-0.39611
CYP2J2	10	207	0.13732	0.37639	0.85112	207	6	-0.74094	291	0.35755	0.65527	0.999915	291	3	-0.74094
PXDN	10	208	0.13812	0.37761	0.85112	208	6	-0.71431	214	0.20458	0.48305	0.999915	214	1	-0.71431
TH	10	209	0.13875	0.37885	0.85112	209	4	0.036933	97	0.06642	0.23041	0.999915	97	4	0.036933
CYP4F8	10	210	0.13915	0.37957	0.85112	210	4	3.7636	123	0.09474	0.28937	0.999915	123	6	3.7636
APBB2	10	211	0.13952	0.38029	0.85112	211	4	2.1623	175	0.15823	0.40995	0.999915	175	5	2.1623
BOLA1	10	212	0.14037	0.3819	0.85112	212	4	-0.35385	392	0.62908	0.85091	0.999915	392	3	-0.35385
CYP2C18	10	213	0.14046	0.38206	0.85112	213	5	-0.59399	430	0.79221	0.9222	0.999915	430	3	-0.59399
MPPED1	10	214	0.14159	0.3841	0.85112	214	5	0.9362	156	0.13419	0.36667	0.999915	156	5	0.9362
KDM6B	10	215	0.14202	0.38478	0.85112	215	4	-0.12332	412	0.70888	0.88793	0.999915	412	3	-0.12332
KDM5D	10	216	0.14328	0.38695	0.85112	216	3	-0.00202	256	0.29423	0.58705	0.999915	256	3	-0.00202
CBS	10	217	0.14372	0.38762	0.85112	217	4	1.9162	259	0.29747	0.59059	0.999915	259	5	1.9162
MPO	10	218	0.14574	0.39077	0.85112	218	5	-0.10031	355	0.52273	0.78097	0.999915	355	4	-0.10031
LRP1	10	219	0.14609	0.39136	0.85112	219	5	-0.4378	183	0.16931	0.42899	0.999915	183	3	-0.4378
LIAS	10	220	0.14684	0.39269	0.85112	220	5	-0.61934	420	0.74326	0.90183	0.999915	420	3	-0.61934
FTL	10	221	0.15098	0.4	0.863074	221	6	-0.68127	426	0.7674	0.91182	0.999915	426	2	-0.68127
STEAP3	10	222	0.15258	0.4029	0.865417	222	6	-0.69844	321	0.4344	0.72261	0.999915	321	3	-0.69844
CYGB	10	223	0.15379	0.40501	0.866061	223	3	2.4077	194	0.18284	0.45142	0.999915	194	6	2.4077
BOLA3	10	224	0.15528	0.40769	0.867926	224	4	2.9511	171	0.15175	0.39826	0.999915	171	6	2.9511
EGLN3	10	225	0.15677	0.41028	0.869585	225	4	-0.23431	322	0.43445	0.72263	0.999915	322	3	-0.23431
SLC40A1	10	226	0.16161	0.41844	0.880474	226	5	-0.59931	431	0.81653	0.93299	0.999915	431	2	-0.59931
KDM3A	10	227	0.16241	0.41973	0.880474	227	6	-0.67343	272	0.32855	0.62509	0.999915	272	2	-0.67343
UBE2D1	10	228	0.16413	0.4225	0.880474	228	6	-0.71369	221	0.22378	0.50587	0.999915	221	4	-0.71369
FOLH1	10	229	0.16565	0.42506	0.880474	229	6	-0.63483	348	0.49749	0.76426	0.999915	348	2	-0.63483
TET2	10	230	0.16645	0.42632	0.880474	230	5	-0.04736	373	0.57953	0.81877	0.999915	373	4	-0.04736
IDO1	10	231	0.17006	0.43221	0.880474	231	2	5.2183	21	0.0091729	0.047763	0.999915	21	8	5.2183
CYP4B1	10	232	0.17083	0.43379	0.880474	232	3	3.7559	91	0.059526	0.2113	0.999915	91	6	3.7559
AMDHD1	10	233	0.17146	0.43481	0.880474	233	3	0.27305	211	0.19986	0.47731	0.999915	211	4	0.27305
DPH1	10	234	0.17152	0.43495	0.880474	234	5	-0.47786	277	0.33262	0.62927	0.999915	277	4	-0.47786
KDM3B	10	235	0.17241	0.43632	0.880474	235	4	2.1278	149	0.12073	0.34066	0.999915	149	5	2.1278
NDUFV2	10	236	0.17298	0.43724	0.880474	236	5	1.8958	206	0.19272	0.468	0.999915	206	5	1.8958
HAAO	10	237	0.1732	0.43758	0.880474	237	5	-0.46954	300	0.36438	0.66209	0.999915	300	2	-0.46954
RASD1	10	238	0.17736	0.44387	0.880474	238	3	2.0711	160	0.13874	0.37492	0.999915	160	6	2.0711
APBB3	10	239	0.17768	0.44434	0.880474	239	6	-0.5672	327	0.4427	0.7283	0.999915	327	3	-0.5672
CYB5R4	10	240	0.17776	0.44448	0.880474	240	3	1.9401	65	0.036545	0.14369	0.999915	65	5	1.9401
OGFOD1	10	241	0.17828	0.4453	0.880474	241	6	-0.57161	369	0.56736	0.81084	0.999915	369	2	-0.57161
SUOX	10	242	0.17907	0.44675	0.880474	242	4	2.2784	191	0.17956	0.44598	0.999915	191	5	2.2784
CYP1A1	10	243	0.1801	0.44851	0.880474	243	6	-0.52098	402	0.67335	0.87427	0.999915	402	2	-0.52098
CYP39A1	10	244	0.18171	0.45099	0.88174	244	3	3.3691	168	0.14524	0.38635	0.999915	168	6	3.3691
ATP6V1A	10	245	0.18726	0.45992	0.887943	245	6	-0.47058	276	0.33216	0.62892	0.999915	276	4	-0.47058
JMJD8	10	246	0.18748	0.46035	0.887943	246	5	-0.32468	279	0.33867	0.63544	0.999915	279	4	-0.32468
NDUFS1	10	247	0.18802	0.46138	0.887943	247	4	2.1391	186	0.17327	0.43576	0.999915	186	5	2.1391
DEGS2	10	248	0.18861	0.46249	0.887943	248	4	-0.05904	173	0.15512	0.40451	0.999915	173	4	-0.05904
PXDNL	10	249	0.18953	0.46394	0.887943	249	5	2.2109	66	0.036662	0.14398	0.999915	66	5	2.2109
NUBPL	10	250	0.19036	0.46529	0.887943	250	6	-0.65472	452	0.90537	0.96367	0.999915	452	2	-0.65472
TPO	10	251	0.19179	0.46758	0.888766	251	3	1.4736	114	0.084193	0.26812	0.999915	114	5	1.4736
ATP6V0A2	10	252	0.19694	0.47586	0.891862	252	5	-0.36471	294	0.35861	0.65622	0.999915	294	4	-0.36471
HPDL	10	253	0.19714	0.47628	0.891862	253	5	-0.55113	270	0.32623	0.62292	0.999915	270	4	-0.55113
CYB561	10	254	0.19716	0.4763	0.891862	254	5	2.1929	134	0.10842	0.31701	0.999915	134	5	2.1929
ETHE1	10	255	0.1974	0.47665	0.891862	255	3	4.0862	45	0.023164	0.10103	0.999915	45	6	4.0862
KDM5C	10	256	0.20076	0.48206	0.896349	256	6	-0.4937	161	0.14177	0.38076	0.999915	161	3	-0.4937
FADS2	10	257	0.20282	0.48514	0.896349	257	2	3.7599	131	0.10687	0.31415	0.999915	131	6	3.7599
MUTYH	10	258	0.20492	0.48838	0.896349	258	5	-0.46763	122	0.093763	0.28741	0.999915	122	2	-0.46763
TET1	10	259	0.20542	0.48907	0.896349	259	2	4.1659	74	0.043573	0.16496	0.999915	74	7	4.1659
GSTP1	10	260	0.20579	0.48958	0.896349	260	5	-0.57112	290	0.35742	0.65515	0.999915	290	2	-0.57112
AIFM3	10	261	0.20684	0.49107	0.896349	261	5	-0.14659	383	0.60973	0.83862	0.999915	383	4	-0.14659
NGB	10	262	0.20858	0.49349	0.896349	262	6	-0.51561	273	0.33033	0.62695	0.999915	273	4	-0.51561
APBA1	10	263	0.20894	0.49407	0.896349	263	5	-0.25846	92	0.05988	0.2124	0.999915	92	3	-0.25846
TPH2	10	264	0.21221	0.49891	0.896349	264	6	-0.50229	385	0.61241	0.84019	0.999915	385	3	-0.50229
DUOX2	10	265	0.21301	0.50008	0.896349	265	4	0.4354	238	0.24336	0.52924	0.999915	238	4	0.4354
CYB5D2	10	266	0.21326	0.50041	0.896349	266	5	-0.52296	405	0.68152	0.87736	0.999915	405	2	-0.52296
CYP2D6	10	267	0.21388	0.50151	0.896349	267	5	1.9216	162	0.14247	0.38193	0.999915	162	5	1.9216
PGRMC2	10	268	0.21622	0.50513	0.899478	268	6	-0.55686	324	0.43543	0.72341	0.999915	324	3	-0.55686
CYP8B1	10	269	0.22179	0.51386	0.911631	269	5	-0.51397	188	0.1759	0.43996	0.999915	188	2	-0.51397
LDHD	10	270	0.22687	0.52096	0.919073	270	3	1.8954	230	0.23536	0.5195	0.999915	230	5	1.8954
HBG2	3	271	0.22688	0.35625	0.85112	271	2	-0.87497	335	0.46197	0.5636	0.999915	335	1	-0.87497
METAP1	10	272	0.22825	0.52243	0.919073	272	3	2.7624	117	0.089488	0.27899	0.999915	117	5	2.7624
CYP27C1	10	273	0.23095	0.52538	0.919073	273	5	-0.23751	245	0.259	0.54806	0.999915	245	4	-0.23751
SCARA5	10	274	0.23304	0.52765	0.919073	274	4	0.46197	207	0.19322	0.4686	0.999915	207	4	0.46197
PHYH	10	275	0.23509	0.53007	0.919073	275	1	5.1335	50	0.025624	0.10901	0.999915	50	6	5.1335
RSAD1	10	276	0.23573	0.53078	0.919073	276	5	1.683	209	0.19749	0.47424	0.999915	209	5	1.683
CYP24A1	10	277	0.23645	0.53149	0.919073	277	3	3.3221	150	0.12115	0.34144	0.999915	150	6	3.3221
SLC22A17	10	278	0.24252	0.53855	0.922393	278	5	-0.38756	401	0.65731	0.86778	0.999915	401	3	-0.38756
METAP2	10	279	0.24322	0.53932	0.922393	279	4	0.094211	242	0.25248	0.54037	0.999915	242	4	0.094211
KDM4D	10	280	0.24457	0.54096	0.922393	280	3	3.0707	179	0.16528	0.422	0.999915	179	6	3.0707
SLC39A7	10	281	0.24662	0.5432	0.922393	281	5	0.42024	301	0.37337	0.6712	0.999915	301	5	0.42024
CYP2A13	10	282	0.24726	0.54392	0.922393	282	3	4.5573	102	0.071765	0.24168	0.999915	102	6	4.5573
TYW5	10	283	0.24902	0.54583	0.922393	283	3	2.7654	120	0.091974	0.28367	0.999915	120	5	2.7654
SLC39A2	10	284	0.24994	0.54689	0.922393	284	4	3.0382	58	0.030948	0.12639	0.999915	58	6	3.0382
HEBP1	10	285	0.25541	0.55347	0.930217	285	5	0.11636	112	0.079378	0.25803	0.999915	112	4	0.11636
SLC39A11	10	286	0.25773	0.5559	0.931027	286	4	0.080145	399	0.64785	0.8626	0.999915	399	3	0.080145

FDX1L	10	287	0.2631	0.56172	0.931541	287	5	-0.43113	456	0.92691	0.97039	0.999915	456	2	-0.43113
HSPA6	10	288	0.26403	0.56276	0.931541	288	4	3.9619	143	0.11292	0.32573	0.999915	143	6	3.9619
CLSTN2	10	289	0.26511	0.5639	0.931541	289	5	-0.24259	374	0.58295	0.82117	0.999915	374	4	-0.24259
KDM6A	10	290	0.26585	0.56465	0.931541	290	2	4.151	90	0.059283	0.21078	0.999915	90	6	4.151
NIPAL1	10	291	0.26716	0.56593	0.931541	291	3	2.188	73	0.043348	0.16424	0.999915	73	5	2.188
PPP3CB	10	292	0.2779	0.57685	0.937975	292	3	5.9598	22	0.0094201	0.048916	0.999915	22	6	5.9598
ALKBH6	10	293	0.27888	0.57778	0.937975	293	4	-0.23237	287	0.35548	0.65313	0.999915	287	4	-0.23237
NOX4	10	294	0.28599	0.58492	0.937975	294	4	-0.16148	316	0.4021	0.69997	0.999915	316	3	-0.16148
ALOXE3	10	295	0.2885	0.58785	0.937975	295	4	0.20062	293	0.35817	0.65586	0.999915	293	3	0.20062
GUCY1A3	10	296	0.29004	0.5896	0.937975	296	4	-0.1531	281	0.34001	0.63697	0.999915	281	3	-0.1531
ENAH	10	297	0.29042	0.58996	0.937975	297	4	0.22939	192	0.18026	0.44735	0.999915	192	4	0.22939
KDM4E	10	298	0.29057	0.59017	0.937975	298	5	-0.22322	225	0.22825	0.5111	0.999915	225	3	-0.22322
PPP3CA	10	299	0.29274	0.59255	0.937975	299	4	0.49323	319	0.42048	0.71304	0.999915	319	3	0.49323
ALOX5	10	300	0.293	0.59284	0.937975	300	5	1.1628	48	0.024715	0.1061	0.999915	48	5	1.1628
CYB5R3	10	301	0.29423	0.59419	0.937975	301	4	1.5695	8	0.002598	0.01615	0.925661	8	5	1.5695
CYP11B2	10	302	0.29676	0.59674	0.937975	302	3	4.7504	15	0.0060525	0.032712	0.965406	15	6	4.7504
IREB2	10	303	0.29882	0.59917	0.937975	303	4	1.4594	247	0.26283	0.5524	0.999915	247	5	1.4594
MDM4	10	304	0.29952	0.60002	0.937975	304	5	1.0138	296	0.35982	0.65744	0.999915	296	5	1.0138
PDE4D	10	305	0.30141	0.60192	0.937975	305	3	0.28916	141	0.11218	0.32458	0.999915	141	4	0.28916
REV3L	10	306	0.30305	0.60359	0.937975	306	3	4.2436	63	0.033677	0.13505	0.999915	63	7	4.2436
NENF	10	307	0.30497	0.60572	0.937975	307	4	2.0949	170	0.14831	0.39204	0.999915	170	5	2.0949
CYP11B1	10	308	0.30626	0.60706	0.937975	308	4	2.8421	78	0.04596	0.17205	0.999915	78	5	2.8421
KIAA1456	10	309	0.31146	0.61228	0.937975	309	5	1.6908	23	0.0097641	0.050696	0.999915	23	5	1.6908
SLC39A14	10	310	0.31232	0.61352	0.937975	310	4	0.32807	350	0.50592	0.77	0.999915	350	4	0.32807
SETD7	10	311	0.31354	0.61483	0.937975	311	5	-0.47232	400	0.65051	0.86419	0.999915	400	2	-0.47232
FLVCR1	10	312	0.31917	0.62064	0.937975	312	3	2.6492	129	0.098851	0.29802	0.999915	129	5	2.6492
ATP6V1G1	10	313	0.31977	0.62113	0.937975	313	4	1.4287	292	0.35766	0.65539	0.999915	292	5	1.4287
CYP4X1	10	314	0.32284	0.62452	0.937975	314	2	3.837	158	0.13563	0.36943	0.999915	158	6	3.837
MAP2	10	315	0.32517	0.62704	0.937975	315	4	0.7636	59	0.03162	0.12862	0.999915	59	3	0.7636
HAMP	10	316	0.32609	0.62802	0.937975	316	5	2.0095	157	0.13423	0.36675	0.999915	157	5	2.0095
NEK6	10	317	0.32649	0.62836	0.937975	317	3	1.3425	243	0.25498	0.54335	0.999915	243	5	1.3425
KDM4C	10	318	0.32693	0.62881	0.937975	318	5	-0.4265	357	0.54397	0.79522	0.999915	357	1	-0.4265
NIF3L1	10	319	0.32988	0.6314	0.937975	319	5	-0.36475	16	0.0063512	0.034203	0.965406	16	4	-0.36475
HSPBAP1	10	320	0.33134	0.63305	0.937975	320	5	-0.28942	31	0.014642	0.071772	0.999915	31	4	-0.28942
TDO2	10	321	0.33615	0.63742	0.937975	321	5	1.2624	126	0.095701	0.29151	0.999915	126	5	1.2624
CYB5R2	10	322	0.3395	0.64091	0.937975	322	5	-0.0874	394	0.6389	0.8571	0.999915	394	2	-0.0874
ALB	10	323	0.33964	0.64102	0.937975	323	5	-0.24245	340	0.47073	0.74724	0.999915	340	4	-0.24245
FRRS1	10	324	0.33974	0.64113	0.937975	324	4	2.0873	193	0.18138	0.44923	0.999915	193	5	2.0873
CAT	10	325	0.34064	0.64199	0.937975	325	3	2.1812	44	0.022927	0.10025	0.999915	44	5	2.1812
RPE	10	326	0.34212	0.64346	0.937975	326	3	4.1044	87	0.053446	0.19383	0.999915	87	7	4.1044
PHYHD1	10	327	0.34328	0.64471	0.937975	327	4	4.4544	105	0.073897	0.24623	0.999915	105	6	4.4544
CYP4A22	10	328	0.3454	0.64674	0.937975	328	4	2.5452	202	0.18933	0.46276	0.999915	202	6	2.5452
CLSTN3	10	329	0.34697	0.64841	0.937975	329	4	2.3295	130	0.10499	0.31042	0.999915	130	5	2.3295
IRG1	10	330	0.34733	0.64882	0.937975	330	5	-0.41718	208	0.1974	0.47414	0.999915	208	3	-0.41718
FXN	10	331	0.35067	0.65195	0.937975	331	4	1.8431	223	0.2255	0.50805	0.999915	223	5	1.8431
SLC11A1	10	332	0.35481	0.65594	0.937975	332	2	2.5013	138	0.11141	0.32292	0.999915	138	5	2.5013
CYP3A43	10	333	0.35509	0.65618	0.937975	333	2	4.6372	49	0.02515	0.10742	0.999915	49	6	4.6372
ALOX15	10	334	0.35593	0.65708	0.937975	334	5	-0.08048	263	0.30693	0.60157	0.999915	263	3	-0.08048
PER3	10	335	0.35614	0.65726	0.937975	335	4	2.1361	95	0.064789	0.22588	0.999915	95	5	2.1361
TMLHE	10	336	0.35683	0.65795	0.937975	336	4	0.43029	227	0.23054	0.51375	0.999915	227	4	0.43029
ASAH2	10	337	0.36156	0.6622	0.939103	337	5	-0.29169	410	0.70537	0.88666	0.999915	410	3	-0.29169
PAPL	10	338	0.36207	0.66267	0.939103	338	5	-0.16715	391	0.62625	0.84906	0.999915	391	4	-0.16715
P4HA2	10	339	0.36783	0.66859	0.944704	339	4	-0.26582	76	0.045012	0.1693	0.999915	76	4	-0.26582
CYB5RL	10	340	0.37556	0.6758	0.946103	340	4	-0.09191	282	0.34069	0.63759	0.999915	282	3	-0.09191
SLC39A1	10	341	0.37636	0.67652	0.946103	341	5	-0.09478	395	0.64079	0.8581	0.999915	395	4	-0.09478
SGK1	10	342	0.38483	0.68447	0.946103	342	1	4.9638	34	0.016463	0.07882	0.999915	34	7	4.9638
TET3	10	343	0.38595	0.68561	0.946103	343	2	5.8364	17	0.0063722	0.034263	0.965406	17	7	5.8364
PLOD3	10	344	0.38819	0.68789	0.946103	344	3	0.14688	302	0.37423	0.67204	0.999915	302	4	0.14688
APLP1	10	345	0.3883	0.68799	0.946103	345	4	-0.04059	306	0.38087	0.67865	0.999915	306	4	-0.04059
CYP2C8	10	346	0.39042	0.69003	0.946103	346	5	-0.22722	71	0.041955	0.15974	0.999915	71	2	-0.22722
ALKBH3	10	347	0.39174	0.69119	0.946103	347	5	1.2978	219	0.21963	0.50088	0.999915	219	5	1.2978
HGD	10	348	0.39307	0.69251	0.946103	348	5	-0.43844	371	0.57186	0.81395	0.999915	371	4	-0.43844
SCD	10	349	0.3966	0.69609	0.946103	349	5	-0.11782	344	0.48881	0.75885	0.999915	344	4	-0.11782
NOX5	10	350	0.39814	0.69733	0.946103	350	2	5.2971	14	0.0054472	0.029571	0.965406	14	7	5.2971
LPO	10	351	0.39882	0.69796	0.946103	351	5	-0.31038	375	0.5876	0.82431	0.999915	375	3	-0.31038
PLOD1	10	352	0.40078	0.69989	0.946103	352	3	0.50429	315	0.40192	0.69976	0.999915	315	5	0.50429
KAT2B	10	353	0.40103	0.70012	0.946103	353	5	-0.21981	356	0.54024	0.79295	0.999915	356	3	-0.21981
AGMO	10	354	0.40578	0.70443	0.946103	354	3	3.36	36	0.018524	0.085501	0.999915	36	5	3.36
ASPH	10	355	0.40636	0.70494	0.946103	355	5	1.0207	298	0.36329	0.6609	0.999915	298	5	1.0207
JAK2	10	356	0.40903	0.70734	0.946103	356	3	2.3515	172	0.15374	0.40192	0.999915	172	5	2.3515
ADHFE1	10	357	0.41147	0.70965	0.946103	357	4	0.36192	11	0.0043266	0.024322	0.965406	11	4	0.36192
NOX1	10	358	0.41762	0.71513	0.946103	358	2	2.0229	197	0.18427	0.45394	0.999915	197	5	2.0229
MSMO1	10	359	0.41868	0.71618	0.946103	359	3	-0.12189	364	0.55914	0.80534	0.999915	364	4	-0.12189
FRRS1L	10	360	0.42151	0.7187	0.946103	360	5	-0.38345	190	0.17907	0.44521	0.999915	190	4	-0.38345
CYP7A1	10	361	0.42285	0.71992	0.946103	361	5	0.24519	2	0.00086666	0.005811	0.854082	2	4	0.24519
RORC	10	362	0.4233	0.72034	0.946103	362	5	-0.33859	297	0.36322	0.66081	0.999915	297	4	-0.33859
PAH	10	363	0.42503	0.72192	0.946103	363	3	0.20621	266	0.31707	0.61275	0.999915	266	4	0.20621
NUBP2	10	364	0.42508	0.72196	0.946103	364	5	-0.26465	196	0.18362	0.45285	0.999915	196	3	-0.26465
SLC39A3	10	365	0.42553	0.72241	0.946103	365	5	0.063515	127	0.09633	0.29285	0.999915	127	4	0.063515
SLC39A8	10	366	0.42609	0.72291	0.946103	366	2	0.51266	299	0.3639	0.66152	0.999915	299	4	0.51266
DUOX1	10	367	0.43135	0.7279	0.950039	367	5	-0.30208	312	0.39924	0.69709	0.999915	312	4	-0.30208
ACO1	10	368	0.43466	0.7309	0.951365	368	4	-0.24823	363	0.55564	0.80311	0.999915	363	4	-0.24823
P4HA1	10	369	0.44474	0.74025	0.952326	369	3	0.009599	64	0.034984	0.13922	0.999915	64	4	0.009599
HR	10	370	0.44697	0.7422	0.952326	370	4	1.889	148	0.11878	0.33695	0.999915	148	5	1.889
CYP20A1	10	371	0.44797	0.74318	0.952326	371	5	0.40268	254	0.28915	0.58124	0.999915	254	5	0.40268
BCO2	10	372	0.44887	0.74394	0.952326	372	5	-0.38193	99	0.071299	0.24057	0.999915	99	3	-0.38193
SDHAF1	10	373	0.45283	0.74675	0.952326	373	3	0.75663	314	0.40156	0.69946	0.999915	314	4	0.75663
CYB5A	10	374	0.45535	0.74842	0.952326	374	3	0.87976	86	0.053298	0.19339	0.999915	86	5	0.87976
ISCA1	10	375	0.45854	0.75028	0.952326	375	4	2.2878	29	0.013659	0.067785	0.999915	29	6	2.2878
CYP4F11	10	376	0.46171	0.75205	0.952326	376	4	0.098685	32	0.016351	0.078492	0.999915	32	4	0.098685
BLVRB	10	377	0.46805	0.75638	0.952326	377	2	5.1694	40	0.021446	0.095601	0.999915	40	7	5.1694
PTGS2	10	378	0.46958	0.75735	0.952326	378	4	-0.23288	182	0.16755	0.42581	0.999915	182	3	-0.23288
MIOX	10	379	0.4785	0.76231	0.952326	379	2	2.1195	241	0.24776	0.53453	0.999915	241	5	2.1195
PGRMC1	10	380	0.48043	0.76339	0.952326	380	3	0.13898	445	0.84831	0.94743	0.999915	445	3	0.13898
NOS2	10	381	0.48478	0.76586	0.952326	381	4	2.5272	12	0.0045583	0.025534	0.965406	12	5	2.5272
MINA	10	382	0.48499	0.76598	0.952326	382	3	-0.22055	244	0.25794	0.5468	0.999915	244	3	-0.22055

MOXD1	10	383	0.48617	0.76674	0.952326	383	3	0.71373	343	0.48235	0.75445	0.999915	343	4	0.71373
MOCS1	10	384	0.48807	0.76795	0.952326	384	2	4.8218	61	0.032691	0.13217	0.999915	61	6	4.8218
HEPHL1	10	385	0.48881	0.76843	0.952326	385	4	4.3914	108	0.076771	0.25221	0.999915	108	6	4.3914
CA9	10	386	0.48902	0.76857	0.952326	386	4	0.32924	125	0.095349	0.29085	0.999915	125	4	0.32924
NDUFS2	10	387	0.49043	0.76942	0.952326	387	4	-0.04296	389	0.61933	0.84447	0.999915	389	4	-0.04296
LYZ	10	388	0.50015	0.77484	0.956366	388	3	2.0388	174	0.15677	0.4075	0.999915	174	5	2.0388
CYP4A11	10	389	0.50311	0.77667	0.956366	389	4	-0.21413	384	0.61208	0.84	0.999915	384	3	-0.21413
NOS3	10	390	0.51346	0.78337	0.960218	390	3	3.8214	96	0.065172	0.22706	0.999915	96	7	3.8214
CYP2C9	10	391	0.51447	0.78381	0.960218	391	2	2.8352	53	0.027162	0.11406	0.999915	53	5	2.8352
FLVCR2	10	392	0.52079	0.78752	0.9623	392	3	1.9635	265	0.31645	0.61203	0.999915	265	5	1.9635
TBXAS1	10	393	0.53128	0.79384	0.964627	393	4	4.9555	67	0.038169	0.14859	0.999915	67	6	4.9555
CYP26C1	10	394	0.53447	0.79595	0.964627	394	3	2.2464	107	0.076154	0.25096	0.999915	107	5	2.2464
RAB6B	10	395	0.53573	0.79678	0.964627	395	3	0.47989	390	0.62004	0.84501	0.999915	390	4	0.47989
KLKB1	10	396	0.54171	0.80031	0.964627	396	4	2.2097	200	0.18778	0.45996	0.999915	200	6	2.2097
ALOX15B	10	397	0.5431	0.80122	0.964627	397	4	0.061498	326	0.43915	0.72592	0.999915	326	3	0.061498
AMBP	10	398	0.54368	0.80151	0.964627	398	2	0.37224	201	0.18892	0.4621	0.999915	201	4	0.37224
CYP1A2	10	399	0.55243	0.80691	0.968702	399	4	2.1386	113	0.081877	0.26324	0.999915	113	5	2.1386
CYP4V2	10	400	0.56792	0.81652	0.976138	400	3	2.3131	218	0.21783	0.49872	0.999915	218	5	2.3131
FRAS1	10	401	0.57133	0.81871	0.976138	401	3	3.128	84	0.052767	0.19189	0.999915	84	6	3.128
NDFIP1	10	402	0.57304	0.81965	0.976138	402	2	5.2604	41	0.021452	0.095611	0.999915	41	6	5.2604
CH25H	10	403	0.57619	0.82139	0.976138	403	3	2.9378	178	0.16241	0.41722	0.999915	178	6	2.9378
STC2	10	404	0.58148	0.82455	0.976138	404	4	1.9044	152	0.12709	0.35345	0.999915	152	5	1.9044
CYP17A1	10	405	0.5826	0.82534	0.976138	405	3	0.19049	234	0.23835	0.52298	0.999915	234	3	0.19049
LCN2	10	406	0.58759	0.82851	0.977475	406	4	0.60698	109	0.077768	0.25451	0.999915	109	4	0.60698
CLSTN1	10	407	0.60131	0.83666	0.982392	407	4	-0.14859	419	0.73691	0.89899	0.999915	419	3	-0.14859
HEPH	10	408	0.61017	0.84187	0.982392	408	4	0.001946	386	0.61369	0.84082	0.999915	386	3	0.001946
HPX	10	409	0.61043	0.84201	0.982392	409	4	2.2515	189	0.17736	0.44225	0.999915	189	5	2.2515
CYB5D1	10	410	0.62214	0.84872	0.982392	410	3	2.7589	30	0.01439	0.070569	0.999915	30	5	2.7589
DPYD	10	411	0.62496	0.85049	0.982392	411	4	5.9459	26	0.01111	0.056919	0.999915	26	6	5.9459
ALKBH8	10	412	0.62568	0.85093	0.982392	412	3	0.019026	310	0.39704	0.69505	0.999915	310	4	0.019026
RORB	10	413	0.63343	0.85565	0.982392	413	4	-0.24659	146	0.11786	0.33513	0.999915	146	3	-0.24659
GLRX2	10	414	0.64503	0.86225	0.982392	414	4	0.33157	434	0.81912	0.93415	0.999915	434	3	0.33157
FADS3	10	415	0.64778	0.86381	0.982392	415	3	3.1458	118	0.089828	0.27943	0.999915	118	6	3.1458
FTHL17	10	416	0.65008	0.86506	0.982392	416	4	0.020347	153	0.12804	0.35537	0.999915	153	4	0.020347
BACH1	10	417	0.66463	0.87375	0.982392	417	4	3.1512	166	0.14471	0.38549	0.999915	166	6	3.1512
HERC2	10	418	0.6671	0.87533	0.982392	418	4	0.48402	62	0.033303	0.13394	0.999915	62	5	0.48402
CYBB	10	419	0.66955	0.87668	0.982392	419	2	3.9322	128	0.0968	0.29371	0.999915	128	6	3.9322
TSHZ3	10	420	0.67003	0.87694	0.982392	420	3	2.3524	47	0.023919	0.10349	0.999915	47	5	2.3524
PARN	10	421	0.67191	0.87805	0.982392	421	3	1.6916	258	0.29707	0.59006	0.999915	258	5	1.6916
CTRL	1004	422	0.67769	0.88146	0.982392	422	281	2.7713	1	3.32E-13	4.97E-06	0.002381	1	530	2.7713
KDM5A	10	423	0.68521	0.88591	0.982392	423	1	4.5539	88	0.054947	0.19871	0.999915	88	6	4.5539
TPH1	10	424	0.68902	0.88806	0.982392	424	3	1.61	285	0.35249	0.6502	0.999915	285	5	1.61
CYP2R1	10	425	0.69008	0.8886	0.982392	425	2	0.45969	253	0.28869	0.58061	0.999915	253	3	0.45969
ABCE1	10	426	0.69027	0.88866	0.982392	426	3	3.6156	81	0.049389	0.18219	0.999915	81	7	3.6156
NARF	10	427	0.69068	0.8888	0.982392	427	2	4.4971	85	0.052974	0.19255	0.999915	85	6	4.4971
TYW1	10	428	0.69192	0.88948	0.982392	428	3	0.48768	334	0.45917	0.7393	0.999915	334	4	0.48768
GUCY1A2	10	429	0.69532	0.89135	0.982392	429	3	-0.06533	100	0.071624	0.24129	0.999915	100	4	-0.06533
CYP2A7	10	430	0.7002	0.89304	0.982392	430	2	0.3213	10	0.0034217	0.020345	0.965406	10	4	0.3213
CYP2W1	10	431	0.70214	0.89362	0.982392	431	3	0.98044	106	0.076073	0.25079	0.999915	106	5	0.98044
ABCB6	10	432	0.70615	0.89492	0.982392	432	3	1.5387	56	0.0298	0.12256	0.999915	56	5	1.5387
SLC39A4	10	433	0.71097	0.89653	0.982392	433	3	4.3211	57	0.030459	0.12488	0.999915	57	6	4.3211
GUCY1B3	10	434	0.7119	0.89687	0.982392	434	3	3.5398	89	0.057611	0.20625	0.999915	89	7	3.5398
AFM	10	435	0.71848	0.89915	0.982392	435	3	2.2539	19	0.0077757	0.041003	0.999915	19	5	2.2539
CD163	10	436	0.74326	0.90814	0.982392	436	3	2.4495	147	0.11805	0.3354	0.999915	147	5	2.4495
EPX	10	437	0.74395	0.90836	0.982392	437	2	6.6031	3	0.0013528	0.008724	0.854082	3	6	6.6031
CYP2C19	10	438	0.74496	0.90879	0.982392	438	3	3.0298	115	0.08451	0.26881	0.999915	115	5	3.0298
HMOX1	10	439	0.74877	0.91019	0.982392	439	2	4.9011	25	0.010667	0.054891	0.999915	25	6	4.9011
RFESD	10	440	0.75147	0.91133	0.982392	440	1	4.5543	35	0.018059	0.08393	0.999915	35	7	4.5543
HDAC2	10	441	0.75313	0.91196	0.982392	441	3	4.4851	103	0.072752	0.24406	0.999915	103	6	4.4851
HBD	10	442	0.76072	0.91457	0.982392	442	1	3.434	145	0.11665	0.33279	0.999915	145	6	3.434
TSHZ1	10	443	0.76405	0.91573	0.982392	443	2	5.1809	18	0.0074536	0.039512	0.999915	18	8	5.1809
DNAJC24	10	444	0.77458	0.91975	0.982392	444	3	1.2461	305	0.38031	0.67815	0.999915	305	5	1.2461
ALOX12B	10	445	0.78928	0.9252	0.982392	445	3	2.0326	101	0.071654	0.24138	0.999915	101	5	2.0326
CYP3A4	10	446	0.79341	0.92658	0.982392	446	3	2.6784	132	0.10743	0.31514	0.999915	132	5	2.6784
SLC48A1	10	447	0.79844	0.92845	0.982392	447	3	0.1301	366	0.56302	0.80801	0.999915	366	4	0.1301
SLC25A37	10	448	0.79855	0.9285	0.982392	448	2	2.0389	121	0.092175	0.28408	0.999915	121	5	2.0389
HFE	10	449	0.8061	0.93147	0.982392	449	2	4.3926	69	0.04005	0.15416	0.999915	69	6	4.3926
CYP19A1	10	450	0.8121	0.93397	0.982392	450	2	-0.01562	177	0.16122	0.41525	0.999915	177	4	-0.01562
SC5D	10	451	0.81349	0.93458	0.982392	451	3	0.59712	317	0.41131	0.70682	0.999915	317	4	0.59712
STEAP1B	10	452	0.81625	0.93555	0.982392	452	3	-0.31641	169	0.14553	0.38691	0.999915	169	4	-0.31641
NIPA1	10	453	0.82235	0.93789	0.982392	453	3	1.5126	233	0.23811	0.52273	0.999915	233	5	1.5126
CDK5RAP	10	454	0.82264	0.93792	0.982392	454	3	2.7846	164	0.14421	0.38474	0.999915	164	5	2.7846
BBOX1	10	455	0.82466	0.93869	0.982392	455	2	4.5001	75	0.043994	0.16614	0.999915	75	7	4.5001
ATOX1	10	456	0.82467	0.93869	0.982392	456	3	2.6384	165	0.1445	0.38512	0.999915	165	5	2.6384
CYP51A1	10	457	0.82639	0.93932	0.982392	457	3	0.25607	274	0.33125	0.62798	0.999915	274	4	0.25607
CDKAL1	10	458	0.84195	0.94595	0.987172	458	3	4.7441	37	0.018601	0.085769	0.999915	37	6	4.7441
CYP4F3	10	459	0.84998	0.94921	0.988421	459	1	4.4043	4	0.0014137	0.009072	0.854082	4	9	4.4043
AOX1	10	460	0.86438	0.95534	0.99264	460	3	3.6987	144	0.11387	0.32755	0.999915	144	6	3.6987
HBG1	3	461	0.86567	0.86538	0.982392	461	0	4.3306	231	0.23671	0.36463	0.999915	231	2	4.3306
RORA	10	462	0.87808	0.96113	0.995946	462	3	0.43671	346	0.49655	0.76367	0.999915	346	4	0.43671
CYP2F1	10	463	0.89987	0.96614	0.995946	463	2	4.8514	55	0.029639	0.1221	0.999915	55	7	4.8514
SLC39A10	10	464	0.91731	0.97051	0.995946	464	2	5.6173	39	0.020742	0.093235	0.999915	39	6	5.6173
RRM2B	10	465	0.91981	0.97107	0.995946	465	2	3.1981	9	0.0028523	0.017392	0.925661	9	5	3.1981
XDH	10	466	0.92094	0.97149	0.995946	466	2	1.5281	151	0.12673	0.35272	0.999915	151	5	1.5281
CYP2A6	10	467	0.92648	0.97295	0.995946	467	2	3.7777	137	0.11135	0.32279	0.999915	137	6	3.7777
CYP21A2	10	468	0.93522	0.97523	0.995946	468	2	5.2361	24	0.010328	0.053291	0.999915	24	8	5.2361
CYP2U1	10	469	0.93893	0.97647	0.995946	469	2	0.15435	217	0.21436	0.49447	0.999915	217	3	0.15435
PTGIS	10	470	0.94346	0.9779	0.995946	470	2	4.4671	98	0.068547	0.23456	0.999915	98	6	4.4671
CYP3A5	10	471	0.9553	0.98204	0.995946	471	2	-0.28536	224	0.22687	0.50954	0.999915	224	3	-0.28536
ABAT	10	472	0.9604	0.98401	0.995946	472	2	3.365	140	0.11204	0.32421	0.999915	140	6	3.365
CDO1	10	473	0.96238	0.98488	0.995946	473	2	1.8561	257	0.29462	0.58754	0.999915	257	5	1.8561
PTGS1	10	474	0.96385	0.98555	0.995946	474	2	3.3521	33	0.016384	0.078591	0.999915	33	5	3.3521
AFP	10	475	0.98754	0.99205	0.996498	475	1	6.449	6	0.001935	0.012273	0.854082	6	8	6.449
HIF1A	10	476	0.99204	0.9939	0.996498	476	1	3.9057	83	0.052736	0.1918	0.999915	83	6	3.9057
LEPREL1	10	477	0.99263	0.99415	0.996498	477	1	4.1223	54	0.029422	0.12131	0.999915	54	7	4.1223
DLG3	10	478	0.99315	0.99442	0.996498	478	1	6.2827	5	0.0016201	0.010384	0.854082	5	8	6.2827

PPP3CC	10	479	0.99762	0.99766	0.997659	479	0	4.0378	135	0.10932	0.31889	0.999915	135	6	4.0378

Total dropped-out sgRNAs
2494

Supplemental Table S4. Result of MAGeCK test of Mouse #3

id	num	Rank	neg|score	neg|p-valu	neg|fdr	neg|rank	neg|goodsgrna	neg|lfc	Rank2	pos|score	pos|p-valu	pos|fdr	pos|rank	pos|goods	pos|lfc
PRC1	10	1	2.35E-05	0.000194	0.072222	1	10	-2.7355	479	0.99998	0.99999	0.999985	479	0	-2.7355
ATP6V1A	10	2	4.11E-05	0.000313	0.072222	2	9	-2.8259	415	0.74948	0.92046	0.999716	415	1	-2.8259
UQCRC1	10	3	5.66E-05	0.000452	0.072222	3	9	-2.8626	477	0.99692	0.99723	0.999716	477	1	-2.8626
CYB5B	10	4	0.00024353	0.001914	0.192857	4	6	-1.4105	368	0.55218	0.81864	0.999716	368	3	-1.4105
OGFOD1	10	5	0.00025918	0.002033	0.192857	5	7	-2.1233	162	0.12303	0.36102	0.999716	162	3	-2.1233
CYP27B1	10	6	0.00032044	0.00249	0.192857	6	8	-2.898	463	0.96113	0.98567	0.999716	463	2	-2.898
GLRX5	10	7	0.0003802	0.002818	0.192857	7	8	-2.861	448	0.88804	0.96596	0.999716	448	2	-2.861
HSCB	10	8	0.00043715	0.003236	0.19375	8	8	-2.754	383	0.60571	0.8474	0.999716	383	2	-2.754
POLE	10	9	0.00050237	0.003693	0.196561	9	7	-2.3245	466	0.96874	0.98823	0.999716	466	2	-2.3245
CIAO1	10	10	0.00073063	0.005403	0.237446	10	8	-2.8841	438	0.8416	0.9494	0.999716	438	1	-2.8841
ATP6V0D1	10	11	0.00074309	0.005453	0.237446	11	9	-2.8029	413	0.74698	0.91931	0.999716	413	1	-2.8029
UTY	10	12	0.00086666	0.006367	0.246577	12	5	0.19442	314	0.39484	0.6997	0.999716	314	5	0.19442
UQCRFS1	10	13	0.0010594	0.0077	0.246577	13	8	-2.9614	265	0.27499	0.58812	0.999716	265	2	-2.9614
UQCRC2	10	14	0.0010684	0.007779	0.246577	14	8	-2.815	467	0.96979	0.98853	0.999716	467	2	-2.815
POLA1	10	15	0.0011257	0.008067	0.246577	15	9	-2.868	446	0.87679	0.96167	0.999716	446	1	-2.868
ALOX5	10	16	0.0011458	0.008236	0.246577	16	6	-1.3861	233	0.21064	0.49939	0.999716	233	3	-1.3861
EXO5	10	17	0.0013358	0.009161	0.247487	17	8	-2.9245	308	0.38707	0.69247	0.999716	308	2	-2.9245
P4HTM	10	18	0.0013616	0.0093	0.247487	18	9	-2.3795	474	0.99231	0.99523	0.999716	474	1	-2.3795
KAT5	10	19	0.0017501	0.011607	0.269742	19	8	-2.7255	462	0.95862	0.98496	0.999716	462	2	-2.7255
ATP6AP1	10	20	0.0017682	0.011716	0.269742	20	8	-2.7653	102	0.068049	0.23687	0.999716	102	2	-2.7653
FDXR	10	21	0.0018731	0.012322	0.269742	21	9	-2.763	455	0.92147	0.97599	0.999716	455	1	-2.763
CCDC115	10	22	0.0019794	0.012939	0.269742	22	9	-2.5005	475	0.99275	0.99537	0.999716	475	1	-2.5005
DOHH	10	23	0.002018	0.013287	0.269742	23	6	-1.8875	388	0.63398	0.86188	0.999716	388	4	-1.8875
ERCC2	10	24	0.0020496	0.013515	0.269742	24	8	-2.966	468	0.97136	0.98916	0.999716	468	2	-2.966
DPH1	10	25	0.0023154	0.015245	0.28196	25	8	-2.7467	472	0.98731	0.99374	0.999716	472	1	-2.7467
COQ6	10	26	0.0023295	0.015305	0.28196	26	9	-2.7643	473	0.99025	0.99453	0.999716	473	1	-2.7643
CYP2E1	10	27	0.0026967	0.017333	0.307496	27	7	-2.8947	458	0.9283	0.97727	0.999716	458	2	-2.8947
DGCR8	10	28	0.003336	0.020892	0.357398	28	5	-0.75885	353	0.48718	0.77693	0.999716	353	5	-0.75885
SET	10	29	0.0035776	0.022214	0.366103	29	7	-2.7986	13	0.0035525	0.021826	0.752211	13	3	-2.7986
PPAT	10	30	0.0041181	0.024998	0.366103	30	9	-2.5605	385	0.60796	0.84851	0.999716	385	1	-2.5605
JHDM1D	10	31	0.0043266	0.025872	0.366103	31	6	-2.1304	362	0.52261	0.80275	0.999716	362	3	-2.1304
THAP4	10	32	0.0043728	0.02622	0.366103	32	7	-2.7113	404	0.71566	0.90373	0.999716	404	2	-2.7113
OSGEP	10	33	0.0043908	0.02632	0.366103	33	8	-2.6992	478	0.99757	0.99763	0.999716	478	1	-2.6992
SOD2	10	34	0.0044426	0.026558	0.366103	34	5	0.65849	217	0.18901	0.4677	0.999716	217	5	0.65849
CYP46A1	10	35	0.0046104	0.027423	0.366103	35	5	-0.15814	181	0.14525	0.39829	0.999716	181	5	-0.15814
NDOR1	10	36	0.0047379	0.028278	0.366103	36	8	-2.5817	465	0.96721	0.98774	0.999716	465	2	-2.5817
CYP2S1	10	37	0.0048345	0.028666	0.366103	37	6	-1.8165	329	0.4304	0.73004	0.999716	329	4	-1.8165
TMEM199	10	38	0.0049076	0.029044	0.366103	38	8	-2.5274	417	0.76284	0.92433	0.999716	417	2	-2.5274
NDUFS1	10	39	0.0054287	0.031658	0.388828	39	8	-2.6448	454	0.91318	0.97449	0.999716	454	2	-2.6448
SLC39A12	10	40	0.005755	0.033448	0.400536	40	8	-2.5763	339	0.45688	0.75252	0.999716	339	1	-2.5763
NIF3L1	10	41	0.0059589	0.034661	0.403293	41	7	-1.927	423	0.77997	0.9297	0.999716	423	2	-1.927
TET2	10	42	0.0062002	0.036082	0.403293	42	6	-2.7418	196	0.15972	0.42227	0.999716	196	4	-2.7418
COX5A	10	43	0.0063512	0.036947	0.403293	43	8	-2.5342	425	0.78053	0.92987	0.999716	425	2	-2.5342
SLC39A13	10	44	0.0067121	0.038905	0.403293	44	5	-0.38158	202	0.16582	0.4319	0.999716	202	5	-0.38158
SLC39A7	10	45	0.006718	0.038935	0.403293	45	9	-2.4358	286	0.31671	0.62904	0.999716	286	1	-2.4358
SDHC	10	46	0.0068	0.039412	0.403293	46	9	-2.8442	452	0.90848	0.97342	0.999716	452	1	-2.8442
NDUFV1	10	47	0.0068305	0.039572	0.403293	47	7	-2.7657	391	0.63873	0.86418	0.999716	391	3	-2.7657
RSBN1L	10	48	0.0071724	0.041301	0.412153	48	7	-2.3884	428	0.81285	0.93971	0.999716	428	3	-2.3884
TSHZ3	10	49	0.0077757	0.044453	0.434548	49	6	-1.01	225	0.20317	0.48922	0.999716	225	4	-1.01
KDM4B	10	50	0.0086821	0.049523	0.474429	50	6	-2.2443	10	0.002598	0.016458	0.752211	10	4	-2.2443
TPH1	10	51	0.0092271	0.052177	0.490056	51	9	-1.9731	461	0.951	0.98265	0.999716	461	1	-1.9731
PTGES2	10	52	0.0095255	0.053837	0.495925	52	8	-2.2885	430	0.8225	0.94278	0.999716	430	2	-2.2885
CYC1	10	53	0.01026	0.057595	0.52053	53	7	-1.6019	376	0.58594	0.83664	0.999716	376	3	-1.6019
ALKBH1	10	54	0.010586	0.059057	0.523854	54	8	-2.3054	63	0.03162	0.13661	0.999716	63	1	-2.3054
ALKBH5	10	55	0.011214	0.061641	0.53684	55	7	-2.4552	371	0.57384	0.8303	0.999716	371	3	-2.4552
SDHD	10	56	0.011839	0.064256	0.549617	56	7	-2.3604	426	0.79093	0.93267	0.999716	426	3	-2.3604
RTEL1	10	57	0.012438	0.066672	0.560276	57	7	-2.7349	437	0.84112	0.94929	0.999716	437	2	-2.7349
IBA57	10	58	0.01317	0.069565	0.574507	58	7	-2.4977	424	0.78009	0.92976	0.999716	424	2	-2.4977
KDM6B	10	59	0.014642	0.07534	0.61123	59	3	1.9607	147	0.10368	0.3234	0.999716	147	7	1.9607
MB	10	60	0.014915	0.076563	0.61123	60	4	1.6042	254	0.25146	0.55696	0.999716	254	6	1.6042
PPP1CA	10	61	0.01586	0.080083	0.619931	61	7	-2.1398	378	0.59347	0.84096	0.999716	378	3	-2.1398
ISCU	10	62	0.015895	0.080242	0.619931	62	6	-2.7345	272	0.28499	0.59784	0.999716	272	4	-2.7345
SLC11A2	10	63	0.017108	0.084725	0.64418	63	8	-2.508	249	0.23917	0.54003	0.999716	249	1	-2.508
SCD	10	64	0.018059	0.088085	0.655007	64	6	-2.1926	414	0.74749	0.91959	0.999716	414	4	-2.1926
CYP26B1	10	65	0.018408	0.089407	0.655007	65	7	-1.9467	394	0.66179	0.87597	0.999716	394	3	-1.9467
FADS2	10	66	0.018891	0.091356	0.655007	66	8	-2.4973	457	0.92407	0.97643	0.999716	457	2	-2.4973
MPO	10	67	0.019111	0.092141	0.655007	67	6	-2.7112	155	0.11729	0.35046	0.999716	155	3	-2.7112
TH	10	68	0.01926	0.092986	0.655007	68	6	-1.6541	47	0.019646	0.092281	0.940476	47	3	-1.6541
STEAP1	10	69	0.020485	0.09744	0.664688	69	8	-2.5185	471	0.97836	0.99209	0.999716	471	2	-2.5185
GUCY1A3	10	70	0.020716	0.098385	0.664688	70	4	2.5392	141	0.099031	0.31273	0.999716	141	6	2.5392
GRXCR1	10	71	0.020742	0.098524	0.664688	71	6	-2.6247	422	0.77964	0.92955	0.999716	422	3	-2.6247
SLC39A3	10	72	0.021312	0.1006	0.669279	72	6	-1.1209	291	0.3254	0.63771	0.999716	291	4	-1.1209
SP1	10	73	0.023993	0.11044	0.691825	73	6	-2.3645	443	0.85825	0.95504	0.999716	443	3	-2.3645
CYP2J2	10	74	0.02408	0.11076	0.691825	74	6	-2.4953	400	0.69369	0.89246	0.999716	400	4	-2.4953
FDX1L	10	75	0.024583	0.11266	0.691825	75	7	-2.3543	460	0.94209	0.98032	0.999716	460	2	-2.3543
ABCB7	10	76	0.024593	0.11269	0.691825	76	6	-2.3096	49	0.021612	0.10013	0.966714	49	3	-2.3096
KDM4C	10	77	0.02486	0.1136	0.691825	77	3	2.4055	75	0.040797	0.1626	0.999716	75	6	2.4055
LRP2	10	78	0.026992	0.1213	0.691825	78	7	-1.8192	144	0.10009	0.31529	0.999716	144	2	-1.8192
CLSTN3	10	79	0.027149	0.12196	0.691825	79	7	-2.4022	396	0.67143	0.88118	0.999716	396	3	-2.4022
SETD7	10	80	0.027216	0.12221	0.691825	80	6	-1.8937	149	0.10848	0.33422	0.999716	149	4	-1.8937
KIAA1456	10	81	0.027324	0.12255	0.691825	81	5	0.66686	319	0.4108	0.71343	0.999716	319	5	0.66686
TBXAS1	10	82	0.027487	0.12317	0.691825	82	8	-2.4979	449	0.89541	0.96852	0.999716	449	2	-2.4979
FDX1	10	83	0.027704	0.12399	0.691825	83	7	-2.577	429	0.81562	0.94058	0.999716	429	2	-2.577
FTH1	10	84	0.02813	0.12548	0.691825	84	8	-2.4321	433	0.83038	0.94552	0.999716	433	2	-2.4321
METAP2	10	85	0.028386	0.12643	0.691825	85	7	-2.2461	296	0.34328	0.65383	0.999716	296	3	-2.2461
HEBP2	10	86	0.028842	0.12808	0.691825	86	6	-2.4882	374	0.58357	0.83541	0.999716	374	4	-2.4882
KDM2A	10	87	0.028862	0.1281	0.691825	87	8	-2.3708	419	0.76781	0.92579	0.999716	419	2	-2.3708
CYP1B1	10	88	0.028938	0.12835	0.691825	88	7	-2.548	397	0.67147	0.88118	0.999716	397	3	-2.548
MIPEP	10	89	0.02918	0.12924	0.691825	89	7	-2.4616	476	0.99584	0.99659	0.999716	476	1	-2.4616
EGLN1	10	90	0.029377	0.12999	0.691825	90	8	-2.0485	441	0.85706	0.95459	0.999716	441	1	-2.0485
MMS19	10	91	0.029979	0.1322	0.695892	91	8	-1.9168	435	0.8335	0.94661	0.999716	435	2	-1.9168
RRM2	10	92	0.030755	0.13493	0.70251	92	7	-2.4263	436	0.84094	0.94919	0.999716	436	3	-2.4263
CYB5R2	10	93	0.03162	0.13802	0.710501	93	4	3.4849	97	0.064038	0.22688	0.999716	97	6	3.4849
CYGB	10	94	0.032318	0.14061	0.710501	94	5	0.32112	82	0.047856	0.18304	0.999716	82	5	0.32112

CYP4F2	10	95	0.032398	0.14091	0.710501	95	6	-2.1533	236	0.21484	0.5057	0.999716	236	4	-2.1533
FTO	10	96	0.033056	0.14286	0.710825	96	8	-2.2252	407	0.72665	0.909	0.999716	407	2	-2.2252
NFS1	10	97	0.033381	0.14395	0.710825	97	6	-2.2755	328	0.42912	0.72893	0.999716	328	3	-2.2755
EIF2AK1	10	98	0.034984	0.14966	0.722984	98	3	2.2036	229	0.20474	0.49146	0.999716	229	6	2.2036
NDUFS8	10	99	0.035032	0.14975	0.722984	99	6	-2.5476	214	0.18681	0.46393	0.999716	214	4	-2.5476
AIFM3	10	100	0.035817	0.15211	0.722984	100	7	-2.5805	356	0.48984	0.77917	0.999716	356	3	-2.5805
MYLIP	10	101	0.035914	0.15245	0.722984	101	7	-2.0711	321	0.41762	0.71905	0.999716	321	2	-2.0711
ACO2	10	102	0.037109	0.15637	0.726368	102	7	-2.0787	28	0.011214	0.05846	0.940476	28	3	-2.0787
FABP7	10	103	0.037194	0.15661	0.726368	103	6	-2.4804	241	0.22687	0.52303	0.999716	241	3	-2.4804
ASPH	10	104	0.037842	0.15874	0.726368	104	6	-2.5921	409	0.73629	0.91375	0.999716	409	4	-2.5921
ALOX12	10	105	0.038484	0.16084	0.726368	105	7	-2.3836	372	0.57667	0.83164	0.999716	372	3	-2.3836
CYB5R1	10	106	0.038861	0.16207	0.726368	106	6	-1.9085	274	0.29169	0.60442	0.999716	274	4	-1.9085
ATP6V0A2	10	107	0.038925	0.16226	0.726368	107	8	-2.3993	470	0.97814	0.99198	0.999716	470	2	-2.3993
BCMO1	10	108	0.04168	0.1711	0.75487	108	4	1.584	287	0.31807	0.63041	0.999716	287	5	1.584
ADI1	10	109	0.042303	0.17318	0.75487	109	8	-2.1977	365	0.5414	0.81281	0.999716	365	1	-2.1977
SDHB	10	110	0.042352	0.17335	0.75487	110	6	-2.3466	164	0.12769	0.36941	0.999716	164	4	-2.3466
CYB5RL	10	111	0.043348	0.17687	0.758648	111	5	1.1196	212	0.18435	0.46013	0.999716	212	5	1.1196
FTL	10	112	0.044937	0.18208	0.758648	112	7	-1.8282	279	0.30816	0.62046	0.999716	279	3	-1.8282
FLVCR1	10	113	0.045012	0.18239	0.758648	113	4	1.2904	263	0.27325	0.58599	0.999716	263	6	1.2904
FXN	10	114	0.045999	0.18565	0.758648	114	6	-2.1878	98	0.064286	0.22743	0.999716	98	4	-2.1878
ADO	10	115	0.046228	0.18637	0.758648	115	8	-2.0476	418	0.765	0.92497	0.999716	418	2	-2.0476
TF	10	116	0.046675	0.18782	0.758648	116	4	2.2503	195	0.15815	0.41968	0.999716	195	6	2.2503
HPD	10	117	0.046855	0.18828	0.758648	117	4	1.3127	77	0.04168	0.16515	0.999716	77	5	1.3127
SLC39A8	10	118	0.047145	0.18925	0.758648	118	7	-2.4188	228	0.20458	0.4912	0.999716	228	3	-2.4188
NDFIP1	10	119	0.047416	0.19029	0.758648	119	8	-2.0461	432	0.82682	0.94413	0.999716	432	1	-2.0461
CBS	10	120	0.047744	0.19148	0.758648	120	8	-2.273	369	0.55764	0.82162	0.999716	369	2	-2.273
FBXL5	10	121	0.04817	0.19275	0.758648	121	6	-2.4183	153	0.11263	0.34264	0.999716	153	4	-2.4183
MDM4	10	122	0.048568	0.19427	0.758648	122	5	0.27578	242	0.22727	0.52359	0.999716	242	5	0.27578
LTF	10	123	0.048948	0.19544	0.758648	123	6	-2.4645	285	0.31578	0.62817	0.999716	285	4	-2.4645
HBM	10	124	0.049762	0.19786	0.758648	124	6	-1.9434	284	0.31546	0.6279	0.999716	284	4	-1.9434
ADHFE1	10	125	0.049811	0.19798	0.758648	125	4	0.55918	151	0.10995	0.33764	0.999716	151	4	0.55918
CYP2C18	10	126	0.050593	0.20038	0.761772	126	7	-2.2634	431	0.82496	0.94352	0.999716	431	3	-2.2634
FOLH1	10	127	0.051646	0.20371	0.764729	127	3	4.5748	19	0.0056603	0.032424	0.817419	19	7	4.5748
P4HA2	10	128	0.051953	0.20458	0.764729	128	7	-2.5235	270	0.27758	0.59103	0.999716	270	3	-2.5235
DDX11	10	129	0.052501	0.20595	0.764729	129	4	0.79773	420	0.77151	0.92674	0.999716	420	2	0.79773
CACNA1C	10	130	0.055839	0.21501	0.783071	130	6	-2.1703	382	0.59963	0.84409	0.999716	382	3	-2.1703
MPPED1	10	131	0.056275	0.21617	0.783071	131	6	-2.063	280	0.31024	0.62258	0.999716	280	4	-2.063
ABCE1	10	132	0.056769	0.2173	0.783071	132	6	-1.928	459	0.94099	0.98004	0.999716	459	1	-1.928
FECH	10	133	0.057372	0.21887	0.783071	133	7	-2.5086	447	0.87696	0.96172	0.999716	447	3	-2.5086
COQ7	10	134	0.058774	0.22226	0.783071	134	5	-0.5077	293	0.33013	0.64195	0.999716	293	5	-0.5077
NR1D2	10	135	0.059252	0.22338	0.783071	135	5	-0.42755	11	0.0028024	0.017591	0.752211	11	4	-0.42755
GSTP1	10	136	0.059438	0.22374	0.783071	136	7	-2.3287	384	0.60646	0.84771	0.999716	384	3	-2.3287
KIF4B	10	137	0.05988	0.22494	0.783071	137	4	4.0373	17	0.0051445	0.030097	0.810185	17	6	4.0373
PHYHD1	10	138	0.060797	0.22726	0.783071	138	7	-2.0147	351	0.47561	0.76795	0.999716	351	3	-2.0147
CYP4V2	10	139	0.061519	0.22911	0.783071	139	5	1.3995	161	0.12145	0.35794	0.999716	161	5	1.3995
HSPA6	10	140	0.063101	0.2329	0.783071	140	6	-1.5183	302	0.36621	0.6741	0.999716	302	4	-1.5183
PAPL	10	141	0.063155	0.23307	0.783071	141	6	-0.85181	337	0.45441	0.75072	0.999716	337	3	-0.85181
HCCS	10	142	0.064443	0.23633	0.783071	142	7	-2.2439	124	0.080987	0.26942	0.999716	124	3	-2.2439
ALKBH7	10	143	0.064511	0.23651	0.783071	143	7	-2.3138	403	0.71059	0.90107	0.999716	403	3	-2.3138
SRC	10	144	0.064589	0.23665	0.783071	144	7	-2.1966	444	0.86773	0.95856	0.999716	444	3	-2.1966
FA2H	10	145	0.064789	0.23705	0.783071	145	4	2.5979	115	0.074097	0.25225	0.999716	115	6	2.5979
CYCS	10	146	0.06642	0.24113	0.791112	146	5	1.1013	262	0.2702	0.58221	0.999716	262	5	1.1013
CYP2B6	10	147	0.069535	0.24839	0.809378	147	4	1.6807	201	0.16449	0.42974	0.999716	201	6	1.6807
CYP4F22	10	148	0.071727	0.25369	0.81884	148	6	-2.3117	338	0.45588	0.75178	0.999716	338	4	-2.3117
BRIP1	10	149	0.072161	0.25471	0.81884	149	7	-2.1305	360	0.50477	0.7907	0.999716	360	3	-2.1305
COX10	10	150	0.073786	0.25875	0.820849	150	7	-2.3202	442	0.85776	0.95478	0.999716	442	3	-2.3202
HBZ	10	151	0.074093	0.25947	0.820849	151	5	-1.1364	377	0.58804	0.83793	0.999716	377	4	-1.1364
CYP3A5	10	152	0.074538	0.26048	0.820849	152	5	-0.47624	275	0.29205	0.60477	0.999716	275	4	-0.47624
ALKBH6	10	153	0.075577	0.26297	0.821152	153	6	-1.6032	311	0.39261	0.69757	0.999716	311	4	-1.6032
ASAH2	10	154	0.076537	0.26523	0.821152	154	4	2.0721	131	0.092114	0.29614	0.999716	131	6	2.0721
EGLN2	10	155	0.076773	0.26572	0.821152	155	5	0.84466	174	0.13895	0.38814	0.999716	174	5	0.84466
CLSTN1	10	156	0.077768	0.26821	0.82355	156	7	-1.5966	408	0.73237	0.91188	0.999716	408	3	-1.5966
PXDNL	10	157	0.080202	0.27398	0.829438	157	6	-2.2656	434	0.83067	0.94566	0.999716	434	2	-2.2656
MARK3	10	158	0.080987	0.27575	0.829438	158	6	-1.392	86	0.052767	0.19646	0.999716	86	4	-1.392
METAP1D	10	159	0.081598	0.2772	0.829438	159	5	-0.60043	103	0.068352	0.23747	0.999716	103	4	-0.60043
ELP3	10	160	0.082622	0.27959	0.829438	160	6	-1.3016	450	0.89993	0.97027	0.999716	450	3	-1.3016
NR1D1	10	161	0.083109	0.2809	0.829438	161	7	-2.0019	345	0.46285	0.7573	0.999716	345	3	-2.0019
DDX5	10	162	0.08362	0.2821	0.829438	162	5	-0.24485	359	0.50077	0.78739	0.999716	359	4	-0.24485
BCO2	10	163	0.085753	0.28695	0.829438	163	7	-1.9343	297	0.34687	0.65706	0.999716	297	3	-1.9343
COX15	10	164	0.086647	0.28911	0.829438	164	7	-2.1388	469	0.97519	0.9906	0.999716	469	2	-2.1388
HGD	10	165	0.087254	0.29044	0.829438	165	5	0.20659	218	0.18909	0.4678	0.999716	218	5	0.20659
GLRX2	10	166	0.087395	0.29077	0.829438	166	6	-1.4507	126	0.082617	0.27341	0.999716	126	3	-1.4507
REV3L	10	167	0.088146	0.29247	0.829438	167	6	-2.2586	320	0.41197	0.71443	0.999716	320	3	-2.2586
FADS1	10	168	0.089709	0.29591	0.829438	168	5	-0.73079	312	0.39285	0.69776	0.999716	312	4	-0.73079
CYB5R4	10	169	0.089866	0.29615	0.829438	169	5	-0.41424	69	0.038337	0.156	0.999716	69	4	-0.41424
HTT	10	170	0.090584	0.2976	0.829438	170	6	-1.1758	20	0.0060525	0.034422	0.824405	20	4	-1.1758
MCOLN1	10	171	0.090798	0.29804	0.829438	171	4	2.1574	251	0.24036	0.54164	0.999716	251	6	2.1574
TMEM62	10	172	0.09106	0.29866	0.829438	172	5	-0.71193	352	0.4774	0.76927	0.999716	352	4	-0.71193
CYP7A1	10	173	0.091519	0.29957	0.829438	173	6	-1.4317	323	0.42081	0.72177	0.999716	323	2	-1.4317
CNNM2	10	174	0.092829	0.30273	0.82964	174	7	-2.214	73	0.040554	0.16192	0.999716	73	3	-2.214
SLC39A14	10	175	0.093565	0.30454	0.82964	175	4	2.1661	200	0.16441	0.42963	0.999716	200	5	2.1661
ENAH	10	176	0.093763	0.30484	0.82964	176	3	3.3827	99	0.066428	0.23302	0.999716	99	7	3.3827
ETHE1	10	177	0.095349	0.30867	0.835338	177	5	-0.34772	213	0.18576	0.46235	0.999716	213	5	-0.34772
POLD1	10	178	0.099125	0.31709	0.852041	178	5	0.017061	269	0.27693	0.59043	0.999716	269	5	0.017061
SUOX	10	179	0.10165	0.32272	0.852041	179	5	-0.20568	453	0.90925	0.97359	0.999716	453	2	-0.20568
P4HA3	10	180	0.1022	0.32385	0.852041	180	4	1.9835	247	0.23683	0.53669	0.999716	247	6	1.9835
HDAC1	10	181	0.10246	0.3244	0.852041	181	6	-0.93562	198	0.16245	0.42661	0.999716	198	4	-0.93562
ISCA2	10	182	0.10272	0.32494	0.852041	182	6	-2.2009	456	0.92399	0.97641	0.999716	456	2	-2.2009
ETFDH	10	183	0.10523	0.33042	0.852041	183	6	-1.8762	72	0.04033	0.16136	0.999716	72	4	-1.8762
SLC39A11	10	184	0.10533	0.33066	0.852041	184	7	-2.1499	186	0.1493	0.40527	0.999716	186	3	-2.1499
KDM5B	10	185	0.10594	0.33183	0.852041	185	7	-1.8293	445	0.87547	0.96135	0.999716	445	1	-1.8293
TYW5	10	186	0.10702	0.33422	0.852041	186	5	0.13224	215	0.18748	0.46502	0.999716	215	5	0.13224
LEPRE1	10	187	0.10756	0.33528	0.852041	187	5	-0.68448	380	0.59859	0.84361	0.999716	380	4	-0.68448
PGRMC1	10	188	0.10794	0.33603	0.852041	188	6	-1.0998	173	0.13723	0.38532	0.999716	173	4	-1.0998
KDM8	10	189	0.10802	0.33619	0.852041	189	7	-2.0797	410	0.74139	0.91651	0.999716	410	2	-2.0797
APP	10	190	0.10951	0.33956	0.852618	190	5	-0.40399	178	0.14312	0.39526	0.999716	178	5	-0.40399

ALB	10	191	0.1097	0.33998	0.852618	191	7	-2.156	440	0.8565	0.95442	0.999716	440	3	-2.156
NUBP1	10	192	0.11133	0.34351	0.856982	192	6	-1.9561	439	0.84752	0.95131	0.999716	439	3	-1.9561
NOS3	10	193	0.11332	0.34752	0.857912	193	5	0.17293	294	0.33495	0.64613	0.999716	294	5	0.17293
NEK6	10	194	0.11377	0.34851	0.857912	194	7	-1.7901	405	0.71973	0.90567	0.999716	405	3	-1.7901
MUTYH	10	195	0.11418	0.34925	0.857912	195	7	-1.4436	203	0.16712	0.43383	0.999716	203	2	-1.4436
NDUFS7	10	196	0.11574	0.35272	0.862014	196	6	-1.5775	451	0.90336	0.97166	0.999716	451	3	-1.5775
CISD3	10	197	0.11729	0.35576	0.863504	197	5	0.29778	350	0.47535	0.76777	0.999716	350	5	0.29778
KDM2B	10	198	0.11786	0.35694	0.863504	198	5	-0.76917	106	0.069833	0.24132	0.999716	106	4	-0.76917
CYB5D2	10	199	0.11884	0.3591	0.864358	199	3	2.4182	180	0.14497	0.39774	0.999716	180	6	2.4182
NOX3	10	200	0.11974	0.36094	0.86444	200	6	-1.6034	363	0.52471	0.80399	0.999716	363	3	-1.6034
NARFL	10	201	0.12139	0.36432	0.868195	201	6	-2.0371	366	0.55045	0.81762	0.999716	366	4	-2.0371
MAP2	10	202	0.12428	0.37074	0.870137	202	7	-1.4643	261	0.26914	0.58084	0.999716	261	3	-1.4643
CYP8B1	10	203	0.12594	0.37429	0.870137	203	4	2.8016	138	0.098259	0.31102	0.999716	138	6	2.8016
ACP5	10	204	0.12594	0.3743	0.870137	204	6	-1.7063	375	0.58576	0.83652	0.999716	375	3	-1.7063
JMJD7	10	205	0.12614	0.37471	0.870137	205	7	-1.8841	401	0.69521	0.89315	0.999716	401	3	-1.8841
ASPHD2	10	206	0.12712	0.37666	0.870137	206	4	3.0004	139	0.098469	0.31152	0.999716	139	6	3.0004
LIAS	10	207	0.12816	0.37883	0.870137	207	5	-0.35844	324	0.42151	0.72237	0.999716	324	5	-0.35844
COPA	10	208	0.13072	0.38414	0.870137	208	6	-2.0848	152	0.11107	0.33998	0.999716	152	4	-2.0848
SLC39A1	10	209	0.13114	0.38514	0.870137	209	5	-0.42988	94	0.05988	0.21586	0.999716	94	4	-0.42988
BBOX1	10	210	0.1312	0.38521	0.870137	210	4	2.0358	224	0.20205	0.48758	0.999716	224	6	2.0358
CYP4F12	10	211	0.132	0.38685	0.870137	211	6	-2.1079	292	0.3299	0.64162	0.999716	292	3	-2.1079
PDE4D	10	212	0.13266	0.38808	0.870137	212	5	0.21041	135	0.096667	0.30709	0.999716	135	5	0.21041
JMJD4	10	213	0.1335	0.38975	0.870137	213	6	-2.2396	146	0.1033	0.32257	0.999716	146	4	-2.2396
CYP11B1	10	214	0.13419	0.39092	0.870137	214	4	2.0155	210	0.18316	0.45829	0.999716	210	6	2.0155
LEPREL1	10	215	0.1358	0.39402	0.870137	215	6	-2.0529	127	0.085504	0.28074	0.999716	127	4	-2.0529
CYB561	10	216	0.13714	0.3966	0.870137	216	7	-1.3205	301	0.36496	0.67305	0.999716	301	3	-1.3205
MSMO1	10	217	0.13734	0.39698	0.870137	217	6	-2.0267	209	0.18095	0.45503	0.999716	209	3	-2.0267
JMJD8	10	218	0.13765	0.3974	0.870137	218	5	-0.37717	411	0.74436	0.91806	0.999716	411	3	-0.37717
APBB2	10	219	0.13792	0.3979	0.870137	219	5	-0.05354	347	0.4711	0.7645	0.999716	347	4	-0.05354
SCARA5	10	220	0.13875	0.39976	0.870137	220	4	1.3534	167	0.13114	0.37563	0.999716	167	5	1.3534
PER1	10	221	0.13961	0.40157	0.870137	221	6	-1.8934	386	0.62247	0.8557	0.999716	386	4	-1.8934
IDO1	10	222	0.14061	0.40366	0.870137	222	7	-1.7965	244	0.23236	0.53047	0.999716	244	3	-1.7965
KDM4A	10	223	0.14132	0.40509	0.870137	223	7	-2.0441	464	0.96494	0.98691	0.999716	464	2	-2.0441
TET3	10	224	0.14363	0.40957	0.875818	224	7	-1.9072	235	0.2144	0.50506	0.999716	235	2	-1.9072
MRE11A	10	225	0.14618	0.41398	0.881323	225	7	-1.695	252	0.24187	0.54367	0.999716	252	3	-1.695
CYP4X1	10	226	0.15058	0.42261	0.891976	226	7	-1.1085	278	0.30348	0.61606	0.999716	278	3	-1.1085
JMJD6	10	227	0.15065	0.42271	0.891976	227	6	-1.94	246	0.23506	0.53429	0.999716	246	4	-1.94
AMDHD1	10	228	0.15379	0.42901	0.895187	228	3	4.1126	24	0.0080359	0.043816	0.874504	24	7	4.1126
ALOX12B	10	229	0.15481	0.43093	0.895187	229	7	-0.99213	364	0.54016	0.81242	0.999716	364	3	-0.99213
MIOX	10	230	0.15528	0.4319	0.895187	230	6	-1.508	148	0.10794	0.33289	0.999716	148	3	-1.508
NENF	10	231	0.15546	0.43231	0.895187	231	5	0.00228	41	0.018059	0.085829	0.940476	41	5	0.00228
PHYH	10	232	0.15614	0.43358	0.895187	232	7	-1.6749	170	0.13419	0.3803	0.999716	170	3	-1.6749
ATP6V1G1	10	233	0.15964	0.4399	0.9011	233	7	-1.7155	343	0.45888	0.75415	0.999716	343	3	-1.7155
KLKB1	10	234	0.1627	0.44542	0.9011	234	4	1.3213	91	0.054947	0.20294	0.999716	91	5	1.3213
APBB3	10	235	0.16565	0.45069	0.9011	235	2	4.2893	27	0.010036	0.053291	0.940476	27	7	4.2893
CA6	10	236	0.16712	0.45254	0.9011	236	5	-0.72373	335	0.45024	0.74728	0.999716	335	4	-0.72373
MINA	10	237	0.1677	0.45332	0.9011	237	3	3.9053	34	0.014687	0.072766	0.940476	34	7	3.9053
NARF	10	238	0.16832	0.45425	0.9011	238	4	1.4388	256	0.25528	0.56258	0.999716	256	5	1.4388
HMOX2	10	239	0.16881	0.45491	0.9011	239	5	-0.58114	67	0.035797	0.14863	0.999716	67	4	-0.58114
CYP2D6	10	240	0.1704	0.4573	0.9011	240	5	-0.73638	373	0.58267	0.83489	0.999716	373	4	-0.73638
NUBP2	10	241	0.17209	0.45967	0.9011	241	6	-1.4487	387	0.62633	0.85776	0.999716	387	4	-1.4487
GUCY1A2	10	242	0.17272	0.46047	0.9011	242	5	0.46283	183	0.14674	0.40082	0.999716	183	5	0.46283
ISCA1	10	243	0.17298	0.4609	0.9011	243	4	1.2485	299	0.35637	0.66543	0.999716	299	5	1.2485
NDUFS2	10	244	0.17423	0.46253	0.9011	244	6	-1.5653	171	0.13475	0.38139	0.999716	171	3	-1.5653
CYB561D1	10	245	0.1743	0.4626	0.9011	245	5	-0.11456	340	0.45742	0.75294	0.999716	340	5	-0.11456
CYP26A1	10	246	0.17618	0.46486	0.9011	246	6	-1.8139	113	0.07292	0.24943	0.999716	113	4	-1.8139
CYP24A1	10	247	0.17736	0.4664	0.9011	247	6	-1.8649	281	0.31053	0.62283	0.999716	281	4	-1.8649
PHF8	10	248	0.17833	0.46776	0.9011	248	6	-1.9403	427	0.80058	0.93562	0.999716	427	3	-1.9403
C16orf52	10	249	0.1787	0.46842	0.9011	249	4	2.4732	65	0.033303	0.14158	0.999716	65	6	2.4732
BACH1	10	250	0.1804	0.47071	0.901876	250	6	-2.079	288	0.31842	0.63074	0.999716	288	4	-2.079
HIF1AN	10	251	0.18226	0.47318	0.903007	251	5	0.77349	226	0.20335	0.4895	0.999716	226	5	0.77349
PLOD2	10	252	0.18498	0.47701	0.906699	252	3	4.2213	33	0.014621	0.072497	0.940476	33	7	4.2213
SLC25A28	10	253	0.18864	0.48241	0.913335	253	4	1.3031	348	0.47185	0.76516	0.999716	348	5	1.3031
RSAD1	10	254	0.1951	0.49118	0.926275	254	5	-0.16584	313	0.39351	0.69836	0.999716	313	5	-0.16584
SDHAF1	10	255	0.19957	0.49767	0.926852	255	4	3.8951	57	0.028024	0.12385	0.999716	57	6	3.8951
CD163	10	256	0.19989	0.49814	0.926852	256	6	-1.7567	110	0.071299	0.24501	0.999716	110	4	-1.7567
RSBN1	10	257	0.2018	0.50066	0.926852	257	5	-0.7995	402	0.70463	0.89813	0.999716	402	4	-0.7995
CYP11A1	10	258	0.20207	0.50106	0.926852	258	6	-1.7202	315	0.39967	0.7038	0.999716	315	4	-1.7202
DUOX1	10	259	0.20217	0.50116	0.926852	259	6	-1.517	342	0.4586	0.75396	0.999716	342	3	-1.517
ALOX15B	10	260	0.20477	0.50463	0.929679	260	4	2.7598	134	0.09391	0.30052	0.999716	134	6	2.7598
GUCY1B3	10	261	0.20724	0.50744	0.931281	261	5	-0.6969	140	0.098933	0.31252	0.999716	140	4	-0.6969
SLC11A1	10	262	0.20956	0.51045	0.933233	262	6	-1.7508	9	0.0023134	0.014917	0.752211	9	4	-1.7508
PLOD1	10	263	0.21248	0.51431	0.936679	263	5	0.038837	74	0.04077	0.16252	0.999716	74	5	0.038837
CYP2C9	10	264	0.21391	0.51625	0.936679	264	4	2.5953	179	0.14491	0.3977	0.999716	179	6	2.5953
CYP26C1	10	265	0.2158	0.51882	0.937145	265	4	2.5264	176	0.14177	0.39313	0.999716	176	6	2.5264
MFI2	10	266	0.21838	0.52227	0.937145	266	5	-0.59008	322	0.41959	0.72083	0.999716	322	4	-0.59008
BOLA1	10	267	0.21982	0.52415	0.937145	267	5	-0.11314	112	0.071654	0.24596	0.999716	112	5	-0.11314
CISD1	10	268	0.21997	0.52433	0.937145	268	3	3.6789	39	0.016585	0.08049	0.940476	39	7	3.6789
HPDL	10	269	0.22435	0.5303	0.944282	269	6	-1.8084	205	0.17139	0.44073	0.999716	205	4	-1.8084
FADS3	10	270	0.22762	0.53441	0.944922	270	5	-0.44225	358	0.49467	0.78294	0.999716	358	5	-0.44225
RORC	10	271	0.22774	0.5346	0.944922	271	4	1.6564	258	0.26353	0.57333	0.999716	258	5	1.6564
NOX1	10	272	0.23019	0.5376	0.946735	272	5	-0.46275	271	0.2808	0.5939	0.999716	271	5	-0.46275
HRG	10	273	0.23683	0.54594	0.9472	273	6	-1.6843	185	0.14825	0.40342	0.999716	185	4	-1.6843
PPP3CA	10	274	0.23769	0.54705	0.9472	274	3	4.6228	22	0.0077322	0.042305	0.874504	22	7	4.6228
PPP3CB	10	275	0.23837	0.54786	0.9472	275	6	-1.1514	182	0.1463	0.40003	0.999716	182	4	-1.1514
CYBRD1	10	276	0.24052	0.55067	0.9472	276	3	1.4757	255	0.2517	0.55738	0.999716	255	6	1.4757
JMJD1C	10	277	0.24322	0.55414	0.9472	277	3	2.5326	158	0.11924	0.35396	0.999716	158	6	2.5326
HDAC2	10	278	0.24726	0.55923	0.9472	278	5	0.97216	109	0.070684	0.24345	0.999716	109	5	0.97216
CTRL	1004	279	0.24861	0.56089	0.9472	279	394	2.0451	1	5.41E-12	4.97E-06	0.002381	1	567	2.0451
CDO1	10	280	0.24965	0.56207	0.9472	280	6	-1.6561	40	0.016655	0.080739	0.940476	40	3	-1.6561
IRG1	10	281	0.25699	0.5717	0.9472	281	4	0.9711	117	0.074538	0.25334	0.999716	117	4	0.9711
BOLA3	10	282	0.25811	0.57299	0.9472	282	5	-0.49658	381	0.59916	0.8439	0.999716	381	3	-0.49658
METAP1	10	283	0.25813	0.57299	0.9472	283	5	0.26517	336	0.45157	0.74833	0.999716	336	5	0.26517
TET1	10	284	0.25926	0.57436	0.9472	284	5	0.89837	221	0.20058	0.48545	0.999716	221	5	0.89837
DPH3	10	285	0.25984	0.57492	0.9472	285	4	1.4522	61	0.029934	0.13107	0.999716	61	5	1.4522
ABAT	10	286	0.26022	0.57542	0.9472	286	6	-1.4985	283	0.31352	0.62602	0.999716	283	4	-1.4985

STEAP3	10	287	0.26026	0.57545	0.9472	287	5	0.10605	325	0.42195	0.72276	0.999716	325	5	0.10605
NOS2	10	288	0.26108	0.57647	0.9472	288	5	-0.23549	101	0.067804	0.23618	0.999716	101	5	-0.23549
PAH	10	289	0.26243	0.57811	0.9472	289	6	-1.61	289	0.32162	0.63381	0.999716	289	4	-1.61
HAMP	10	290	0.2631	0.57886	0.9472	290	5	1.2917	184	0.14802	0.403	0.999716	184	5	1.2917
PER3	10	291	0.26377	0.5798	0.9472	291	3	3.0164	123	0.080183	0.26749	0.999716	123	7	3.0164
DUOX2	10	292	0.26614	0.58252	0.9472	292	6	-1.6937	93	0.056594	0.20723	0.999716	93	4	-1.6937
CYP20A1	10	293	0.26716	0.58368	0.9472	293	5	-0.87123	290	0.32528	0.63754	0.999716	290	2	-0.87123
KIF4A	10	294	0.268	0.5848	0.9472	294	6	-1.5453	392	0.64288	0.86614	0.999716	392	3	-1.5453
HAAO	10	295	0.26847	0.58538	0.9472	295	5	-0.5253	370	0.57305	0.8299	0.999716	370	4	-0.5253
SLC46A1	10	296	0.27099	0.58843	0.9472	296	5	-0.98496	389	0.63654	0.8632	0.999716	389	4	-0.98496
SLC39A2	10	297	0.27315	0.59085	0.9472	297	4	2.3311	208	0.17889	0.45184	0.999716	208	6	2.3311
CISD2	10	298	0.27369	0.59137	0.9472	298	3	1.3844	264	0.27362	0.58635	0.999716	264	6	1.3844
CYP4Z1	10	299	0.27621	0.59427	0.9472	299	4	3.3556	18	0.0052283	0.030445	0.810185	18	6	3.3556
SGK1	10	300	0.2771	0.59521	0.9472	300	5	-0.18419	219	0.19277	0.47327	0.999716	219	4	-0.18419
CYP27C1	10	301	0.28367	0.60254	0.953803	301	4	0.74566	257	0.26242	0.57194	0.999716	257	3	0.74566
NPAS2	10	302	0.28439	0.60335	0.953803	302	5	-0.10792	132	0.092175	0.29626	0.999716	132	4	-0.10792
LDHD	10	303	0.28644	0.60554	0.954128	303	6	-1.3585	355	0.48949	0.77879	0.999716	355	4	-1.3585
FTMT	10	304	0.29353	0.61443	0.954471	304	6	-1.2337	367	0.55069	0.81778	0.999716	367	3	-1.2337
CYB561A	10	305	0.29503	0.61629	0.954471	305	5	-0.7918	416	0.75541	0.9222	0.999716	416	3	-0.7918
LYRM7	8	306	0.2952	0.59164	0.9472	306	3	1.3288	341	0.45769	0.72443	0.999716	341	4	1.3288
CYP3A7	10	307	0.29676	0.61825	0.954471	307	4	0.95955	303	0.37503	0.6819	0.999716	303	4	0.95955
OGFOD2	10	308	0.30034	0.62279	0.954471	308	6	-1.5804	334	0.44167	0.73982	0.999716	334	3	-1.5804
SLC40A1	10	309	0.3018	0.62439	0.954471	309	2	4.8449	8	0.0016576	0.010702	0.640774	8	8	4.8449
ABL1	10	310	0.30193	0.62455	0.954471	310	5	0.58376	248	0.23722	0.53729	0.999716	248	5	0.58376
CYP1A1	10	311	0.30274	0.62546	0.954471	311	4	2.2736	216	0.18868	0.46715	0.999716	216	6	2.2736
PIR	10	312	0.30423	0.62728	0.954471	312	2	2.8885	31	0.012929	0.065628	0.940476	31	7	2.8885
HSPBAP1	10	313	0.3043	0.62734	0.954471	313	4	0.71033	260	0.26756	0.57869	0.999716	260	5	0.71033
CYP4F8	10	314	0.30555	0.62881	0.954471	314	4	2.9498	48	0.021465	0.099448	0.966714	48	6	2.9498
EVL	10	315	0.30939	0.63301	0.954471	315	5	-0.42812	222	0.2016	0.48684	0.999716	222	4	-0.42812
CYB5D1	10	316	0.30972	0.63349	0.954471	316	5	-0.29031	306	0.38147	0.68743	0.999716	306	4	-0.29031
SLC22A17	10	317	0.31504	0.63984	0.954471	317	5	0.36934	243	0.23054	0.52805	0.999716	243	5	0.36934
KAT2B	10	318	0.31604	0.64095	0.954471	318	5	0.4609	35	0.014915	0.073869	0.940476	35	5	0.4609
CYP11B2	10	319	0.31671	0.64169	0.954471	319	5	-0.28266	87	0.053298	0.19789	0.999716	87	4	-0.28266
INPPL1	10	320	0.31854	0.64384	0.954471	320	5	0.47224	239	0.22215	0.51646	0.999716	239	5	0.47224
TMLHE	10	321	0.31884	0.6441	0.954471	321	6	-1.1482	154	0.11574	0.34796	0.999716	154	4	-1.1482
CYB561D	10	322	0.32016	0.64546	0.954471	322	5	0.22268	310	0.39152	0.69648	0.999716	310	4	0.22268
CYP2W1	10	323	0.3218	0.64752	0.954471	323	6	-0.71842	412	0.74606	0.91885	0.999716	412	4	-0.71842
CYP39A1	10	324	0.32284	0.6488	0.954471	324	4	1.1973	238	0.2158	0.5071	0.999716	238	5	1.1973
CYP21A2	10	325	0.3233	0.64922	0.954471	325	5	0.54939	118	0.075348	0.25529	0.999716	118	5	0.54939
EGLN3	10	326	0.32652	0.65306	0.954471	326	2	3.8727	14	0.0035877	0.021985	0.752211	14	8	3.8727
ALKBH8	10	327	0.32907	0.65558	0.954471	327	3	2.4878	89	0.053573	0.19871	0.999716	89	7	2.4878
CYB5A	10	328	0.3314	0.65844	0.954471	328	6	-1.3075	150	0.10959	0.33679	0.999716	150	4	-1.3075
TSHZ2	10	329	0.33263	0.65963	0.954471	329	6	-1.1696	231	0.20881	0.49693	0.999716	231	4	-1.1696
FLVCR2	10	330	0.33643	0.66376	0.954471	330	4	1.8138	237	0.21521	0.5062	0.999716	237	6	1.8138
ALKBH4	10	331	0.33855	0.66605	0.954471	331	4	3.0755	111	0.07133	0.2451	0.999716	111	6	3.0755
PHF2	10	332	0.34001	0.66778	0.954471	332	4	3.0805	107	0.070131	0.24208	0.999716	107	6	3.0805
TMPPE	10	333	0.34002	0.66778	0.954471	333	6	-1.2566	129	0.086824	0.28395	0.999716	129	4	-1.2566
CAT	10	334	0.34093	0.66871	0.954471	334	5	-0.8645	220	0.19957	0.48398	0.999716	220	3	-0.8645
CYP2F1	10	335	0.34167	0.66952	0.954471	335	6	-1.2437	78	0.043348	0.17019	0.999716	78	3	-1.2437
APBB1	10	336	0.34856	0.67685	0.961358	336	4	1.3237	331	0.43404	0.73329	0.999716	331	5	1.3237
SC5D	10	337	0.35077	0.67908	0.961358	337	5	-0.77628	273	0.29159	0.60432	0.999716	273	4	-0.77628
FRAS1	10	338	0.35292	0.68178	0.961358	338	5	0.26457	277	0.30034	0.61317	0.999716	277	5	0.26457
XDH	10	339	0.35509	0.6839	0.961358	339	5	0.73734	197	0.161	0.42441	0.999716	197	5	0.73734
CYP19A1	10	340	0.35808	0.68694	0.961358	340	5	1.0401	116	0.074371	0.25297	0.999716	116	5	1.0401
LRP1	10	341	0.36211	0.69099	0.961358	341	6	-1.0546	145	0.10167	0.31895	0.999716	145	4	-1.0546
VWF	10	342	0.36497	0.69429	0.961358	342	5	-0.05284	309	0.39148	0.69644	0.999716	309	4	-0.05284
CYBB	10	343	0.36708	0.69636	0.961358	343	4	2.6017	68	0.037292	0.1529	0.999716	68	6	2.6017
MOXD1	10	344	0.36718	0.69645	0.961358	344	6	-0.69481	15	0.0043266	0.025654	0.801339	15	3	-0.69481
ACO1	10	345	0.37026	0.69855	0.961358	345	4	2.0642	156	0.11783	0.35142	0.999716	156	5	2.0642
CLSTN2	10	346	0.37468	0.70202	0.961358	346	3	3.3041	70	0.039441	0.15894	0.999716	70	7	3.3041
CYP51A1	10	347	0.3749	0.70215	0.961358	347	4	1.7009	38	0.016494	0.080073	0.940476	38	5	1.7009
SLC39A10	10	348	0.37811	0.70444	0.961358	348	5	-0.72917	295	0.33906	0.64991	0.999716	295	4	-0.72917
JAK2	10	349	0.38023	0.70606	0.961358	349	4	1.9819	250	0.24008	0.54129	0.999716	250	6	1.9819
APBA1	10	350	0.38321	0.70806	0.961358	350	5	-0.2971	330	0.43133	0.73092	0.999716	330	4	-0.2971
RAB6B	10	351	0.38371	0.70847	0.961358	351	4	1.2668	142	0.099836	0.31478	0.999716	142	5	1.2668
PTGIS	10	352	0.39285	0.71538	0.962856	352	4	3.5463	88	0.053389	0.19819	0.999716	88	6	3.5463
SLC25A37	10	353	0.39351	0.71593	0.962856	353	5	0.82682	175	0.1396	0.38935	0.999716	175	5	0.82682
KDM3A	10	354	0.3955	0.71751	0.962856	354	5	1.1698	157	0.11805	0.35172	0.999716	157	5	1.1698
CYP4A11	10	355	0.39814	0.71931	0.962856	355	2	3.8877	43	0.018628	0.088036	0.940476	43	8	3.8877
CH25H	10	356	0.40015	0.72095	0.962856	356	5	0.23888	298	0.34819	0.65825	0.999716	298	5	0.23888
CYP3A43	10	357	0.40414	0.72401	0.962856	357	5	-0.29586	346	0.46958	0.76344	0.999716	346	4	-0.29586
CYP4A22	10	358	0.40422	0.72405	0.962856	358	4	3.2393	104	0.069375	0.24021	0.999716	104	6	3.2393
CYP17A1	10	359	0.41011	0.72837	0.962856	359	5	0.27675	29	0.011224	0.05852	0.940476	29	5	0.27675
SLC39A5	10	360	0.41058	0.72862	0.962856	360	3	3.9307	54	0.026509	0.11806	0.999716	54	7	3.9307
GALT	10	361	0.41197	0.72968	0.962856	361	3	3.5244	23	0.0078759	0.043021	0.874504	23	7	3.5244
SLC48A1	10	362	0.41809	0.73408	0.966006	362	4	1.8364	192	0.15671	0.41752	0.999716	192	5	1.8364
ABCG2	10	363	0.42609	0.73988	0.969078	363	4	2.0949	193	0.15731	0.41841	0.999716	193	6	2.0949
FRRS1	10	364	0.42945	0.7425	0.969078	364	5	-0.10491	317	0.4047	0.70814	0.999716	317	5	-0.10491
HBQ1	10	365	0.43087	0.74362	0.969078	365	5	-0.38789	177	0.14191	0.39333	0.999716	177	4	-0.38789
HMOX1	10	366	0.43359	0.74562	0.969078	366	5	-0.25473	211	0.18362	0.45908	0.999716	211	4	-0.25473
ABCB6	10	367	0.44077	0.75101	0.969078	367	5	-0.36267	92	0.055396	0.20409	0.999716	92	4	-0.36267
PGRMC2	10	368	0.44212	0.75184	0.969078	368	5	-0.60376	399	0.68773	0.88966	0.999716	399	4	-0.60376
RPE65	10	369	0.4481	0.75624	0.969078	369	4	2.5246	187	0.15188	0.40945	0.999716	187	6	2.5246
CA9	10	370	0.44882	0.75672	0.969078	370	5	-0.4791	393	0.65967	0.87482	0.999716	393	4	-0.4791
HR	10	371	0.45987	0.76466	0.969078	371	4	2.4965	190	0.15489	0.41436	0.999716	190	6	2.4965
DPH2	10	372	0.4607	0.76533	0.969078	372	4	1.3438	332	0.43718	0.73605	0.999716	332	5	1.3438
NFU1	10	373	0.46354	0.76736	0.969078	373	4	2.6263	159	0.11988	0.35509	0.999716	159	6	2.6263
HBB	10	374	0.46678	0.77018	0.969078	374	4	2.2696	160	0.12038	0.35577	0.999716	160	6	2.2696
KDM5C	10	375	0.46685	0.77022	0.969078	375	4	1.8337	240	0.22435	0.51963	0.999716	240	6	1.8337
HBE1	10	376	0.46994	0.77254	0.969078	376	4	1.5167	318	0.40503	0.70849	0.999716	318	5	1.5167
DEGS2	10	377	0.47094	0.77301	0.969078	377	5	-0.10551	357	0.49395	0.78242	0.999716	357	5	-0.10551
DNAJC24	10	378	0.47271	0.77436	0.969078	378	4	1.5354	122	0.079667	0.26621	0.999716	122	5	1.5354
TPH2	10	379	0.47677	0.77702	0.969078	379	4	2.6489	56	0.02721	0.12096	0.999716	56	5	2.6489
MOCS1	10	380	0.47861	0.77804	0.969078	380	5	-0.0491	354	0.48944	0.77874	0.999716	354	5	-0.0491
CYP4B1	10	381	0.47865	0.77807	0.969078	381	5	0.15541	300	0.35861	0.66726	0.999716	300	5	0.15541
CYP2U1	10	382	0.47906	0.77841	0.969078	382	5	-0.88747	349	0.47252	0.76563	0.999716	349	4	-0.88747

KDM3B	10	383	0.48139	0.77977	0.969078	383	4	1.7024	133	0.092199	0.29631	0.999716	133	6	1.7024
FRRS1L	10	384	0.48363	0.78136	0.969078	384	5	0.29274	207	0.1732	0.44345	0.999716	207	4	0.29274
LPO	10	385	0.48617	0.7833	0.969078	385	3	2.3593	81	0.047854	0.18304	0.999716	81	7	2.3593
CP	10	386	0.48872	0.78497	0.969078	386	4	0.91473	390	0.63824	0.86395	0.999716	390	4	0.91473
TPO	10	387	0.4928	0.78812	0.970455	387	5	0.15228	282	0.31232	0.62484	0.999716	282	4	0.15228
NUBPL	10	388	0.49861	0.79166	0.972326	388	3	1.3127	266	0.27536	0.5886	0.999716	266	5	1.3127
LYRM4	10	389	0.50686	0.79772	0.97531	389	4	0.17466	395	0.66422	0.87732	0.999716	395	4	0.17466
HERC2	10	390	0.50733	0.79817	0.97531	390	4	0.27826	398	0.67903	0.88526	0.999716	398	4	0.27826
HBG2	3	391	0.51292	0.65487	0.954471	391	1	1.794	305	0.38036	0.53055	0.999716	305	2	1.794
AOX1	10	392	0.51807	0.80568	0.981988	392	4	2.8322	130	0.091904	0.29563	0.999716	130	6	2.8322
DPYD	10	393	0.52645	0.81112	0.984772	393	5	-0.5963	379	0.59476	0.84164	0.999716	379	3	-0.5963
CDK5RAP	10	394	0.52949	0.81319	0.984772	394	5	-0.30588	232	0.21002	0.49865	0.999716	232	4	-0.30588
KDM4E	10	395	0.53263	0.81532	0.984772	395	5	0.39346	25	0.0094962	0.050616	0.940476	25	5	0.39346
APLP1	10	396	0.54402	0.82276	0.984772	396	4	3.0767	120	0.078685	0.26377	0.999716	120	6	3.0767
KDM5A	10	397	0.54423	0.8229	0.984772	397	4	2.0223	26	0.0097641	0.052038	0.940476	26	5	2.0223
CIAPIN1	10	398	0.54653	0.82434	0.984772	398	4	5.0667	4	0.00077063	0.005363	0.509921	4	6	5.0667
CYP2A7	10	399	0.54907	0.82592	0.984772	399	2	4.426	3	0.00052426	0.003683	0.509921	3	8	4.426
NDUFV2	10	400	0.55272	0.82857	0.984772	400	5	0.3104	333	0.43808	0.73692	0.999716	333	5	0.3104
CYP2A6	10	401	0.55359	0.8292	0.984772	401	4	1.8485	223	0.20162	0.48688	0.999716	223	5	1.8485
GLRX3	10	402	0.55497	0.83016	0.984772	402	2	3.2986	53	0.024642	0.11139	0.999716	53	8	3.2986
CYP3A4	10	403	0.55584	0.83074	0.984772	403	5	0.37266	326	0.42464	0.72512	0.999716	326	5	0.37266
IREB2	10	404	0.56409	0.83607	0.984772	404	3	3.1569	55	0.026554	0.1182	0.999716	55	7	3.1569
HBD	10	405	0.56499	0.83661	0.984772	405	4	1.656	191	0.15528	0.41515	0.999716	191	5	1.656
HEBP1	10	406	0.57142	0.84115	0.984772	406	4	1.5108	245	0.23369	0.53243	0.999716	245	5	1.5108
TDO2	10	407	0.57304	0.8421	0.984772	407	5	1.0607	267	0.27653	0.58998	0.999716	267	5	1.0607
CYP27A1	10	408	0.57486	0.84335	0.984772	408	5	0.33244	344	0.46193	0.75658	0.999716	344	5	0.33244
CYP2C8	10	409	0.57545	0.84373	0.984772	409	4	1.2414	166	0.12944	0.37269	0.999716	166	5	1.2414
SLC39A9	10	410	0.57908	0.84594	0.984772	410	5	0.072201	80	0.047585	0.18226	0.999716	80	4	0.072201
TSHZ1	10	411	0.58058	0.84703	0.984772	411	5	0.70853	227	0.20414	0.49065	0.999716	227	5	0.70853
PARN	10	412	0.59044	0.85361	0.987209	412	3	2.116	100	0.066499	0.2332	0.999716	100	7	2.116
STC2	10	413	0.59585	0.85711	0.987209	413	3	3.8167	62	0.030155	0.13198	0.999716	62	7	3.8167
NOS1	10	414	0.59695	0.8577	0.987209	414	3	3.4541	114	0.072923	0.24945	0.999716	114	6	3.4541
STEAP1B	10	415	0.5975	0.85809	0.987209	415	4	2.7904	105	0.069803	0.24124	0.999716	105	6	2.7904
NTHL1	10	416	0.60119	0.86063	0.987209	416	3	3.1238	44	0.018742	0.088543	0.940476	44	7	3.1238
PTGS2	10	417	0.60721	0.86427	0.987209	417	2	2.7656	42	0.018128	0.086117	0.940476	42	7	2.7656
HBG1	3	418	0.60832	0.69802	0.961358	418	1	2.5216	204	0.17056	0.29825	0.999716	204	2	2.5216
LYZ	10	419	0.61017	0.86571	0.987209	419	4	3.1482	96	0.060642	0.2178	0.999716	96	6	3.1482
CYP2R1	10	420	0.6119	0.86672	0.987209	420	5	0.28869	259	0.26585	0.57638	0.999716	259	4	0.28869
LCN2	10	421	0.61628	0.86869	0.987209	421	4	1.4587	84	0.048334	0.18439	0.999716	84	5	1.4587
SLC39A6	10	422	0.61879	0.86973	0.987209	422	4	2.271	189	0.15479	0.41423	0.999716	189	6	2.271
RORB	10	423	0.62979	0.87432	0.987229	423	2	4.0017	12	0.0035481	0.021816	0.752211	12	8	4.0017
NOX4	10	424	0.63332	0.87596	0.987229	424	3	3.3361	59	0.028318	0.12506	0.999716	59	7	3.3361
DLG3	10	425	0.63542	0.87689	0.987229	425	4	0.71854	108	0.070201	0.24221	0.999716	108	5	0.71854
RPE	10	426	0.63974	0.87862	0.987229	426	4	1.9541	253	0.24355	0.54621	0.999716	253	6	1.9541
OGFOD3	10	427	0.64577	0.88091	0.987229	427	4	1.5577	276	0.29381	0.6067	0.999716	276	5	1.5577
TYW1	10	428	0.65175	0.88327	0.987229	428	4	2.0242	95	0.059883	0.21587	0.999716	95	6	2.0242
CYP4F11	10	429	0.65387	0.88418	0.987229	429	4	2.3263	128	0.086222	0.28244	0.999716	128	5	2.3263
UBE2D1	10	430	0.66008	0.88683	0.98789	430	4	1.2998	307	0.38556	0.69117	0.999716	307	5	1.2998
HEPHL1	10	431	0.6716	0.89173	0.99035	431	4	1.6124	304	0.37943	0.68578	0.999716	304	5	1.6124
RSAD2	10	432	0.67982	0.8953	0.99035	432	3	2.0936	230	0.20693	0.49438	0.999716	230	6	2.0936
PLOD3	10	433	0.68213	0.89606	0.99035	433	4	2.6563	188	0.15406	0.41313	0.999716	188	6	2.6563
RASD1	10	434	0.68493	0.89731	0.99035	434	4	1.3307	268	0.27665	0.59008	0.999716	268	6	1.3307
EML6	10	435	0.69249	0.90033	0.991075	435	4	2.8157	136	0.0968	0.30742	0.999716	136	6	2.8157
STEAP4	10	436	0.70312	0.90453	0.991075	436	2	2.6334	194	0.15759	0.41889	0.999716	194	6	2.6334
IDO2	9	437	0.711	0.90315	0.991075	437	2	0.93804	421	0.77729	0.91823	0.999716	421	2	0.93804
ALOX15	10	438	0.71234	0.90831	0.991075	438	3	3.9014	64	0.031811	0.1372	0.999716	64	7	3.9014
CYP7B1	10	439	0.71387	0.90889	0.991075	439	4	0.7215	361	0.51197	0.79688	0.999716	361	5	0.7215
SCD5	10	440	0.72082	0.91186	0.991075	440	3	2.1932	58	0.028245	0.12475	0.999716	58	6	2.1932
PPP3CC	10	441	0.7223	0.91245	0.991075	441	3	2.6342	30	0.011984	0.06179	0.940476	30	6	2.6342
CDKAL1	10	442	0.74066	0.92004	0.993898	442	4	1.8734	169	0.13356	0.37946	0.999716	169	6	1.8734
ALKBH3	10	443	0.74998	0.92404	0.993898	443	3	3.8741	66	0.035194	0.14709	0.999716	66	7	3.8741
CYP4F3	10	444	0.7507	0.9244	0.993898	444	4	1.2225	327	0.42598	0.72626	0.999716	327	5	1.2225
ALOXE3	10	445	0.75197	0.92491	0.993898	445	4	0.92337	406	0.72295	0.90742	0.999716	406	4	0.92337
CYB5R3	10	446	0.75345	0.92542	0.993898	446	2	2.6266	5	0.00086666	0.00596	0.509921	5	8	2.6266
RRM2B	10	447	0.7656	0.93036	0.994903	447	4	2.9125	165	0.12843	0.37074	0.999716	165	6	2.9125
CYBA	10	448	0.76588	0.93051	0.994903	448	3	2.7725	50	0.021835	0.10091	0.966714	50	6	2.7725
KDM4D	10	449	0.77197	0.93287	0.99518	449	3	3.1454	46	0.019547	0.091943	0.940476	46	6	3.1454
AFM	10	450	0.78148	0.93715	0.99518	450	4	2.4301	168	0.13184	0.37663	0.999716	168	6	2.4301
KDM5D	10	451	0.78428	0.93823	0.99518	451	3	1.3691	199	0.16387	0.4289	0.999716	199	5	1.3691
CYP1A2	10	452	0.78677	0.93908	0.99518	452	3	2.6878	76	0.041456	0.16441	0.999716	76	7	2.6878
AMBP	10	453	0.79568	0.94245	0.996061	453	4	3.0655	119	0.077485	0.26084	0.999716	119	6	3.0655
CYP2A13	10	454	0.79966	0.94407	0.996061	454	3	5.0338	7	0.0013699	0.008922	0.610544	7	7	5.0338
EPX	10	455	0.81502	0.95017	0.997234	455	4	1.1897	172	0.13635	0.38394	0.999716	172	5	1.1897
RFESD	10	456	0.82538	0.95394	0.997234	456	1	4.2044	16	0.0045182	0.026767	0.801339	16	8	4.2044
PXDN	10	457	0.82708	0.95431	0.997234	457	2	2.3202	206	0.1723	0.44207	0.999716	206	6	2.3202
NGB	10	458	0.84226	0.95814	0.997234	458	2	3.8	37	0.016463	0.079923	0.940476	37	8	3.8
AGMO	10	459	0.84617	0.959	0.997234	459	2	3.125	125	0.08115	0.2698	0.999716	125	7	3.125
NIPAL1	10	460	0.86441	0.96342	0.997234	460	2	2.0577	137	0.098094	0.31053	0.999716	137	7	2.0577
NOX5	10	461	0.86558	0.9637	0.997234	461	3	2.3931	71	0.040094	0.16081	0.999716	71	7	2.3931
STEAP2	10	462	0.86654	0.96397	0.997234	462	3	2.5126	79	0.044154	0.1724	0.999716	79	7	2.5126
HPX	10	463	0.87087	0.96517	0.997234	463	3	2.3224	121	0.078832	0.26424	0.999716	121	7	2.3224
SLC39A4	10	464	0.87401	0.96601	0.997234	464	3	1.7978	234	0.21288	0.5027	0.999716	234	6	1.7978
HIF1A	10	465	0.8855	0.96926	0.998033	465	3	2.8399	90	0.05421	0.20069	0.999716	90	7	2.8399
FTHL17	10	466	0.89265	0.97095	0.998033	466	3	2.231	143	0.10007	0.31525	0.999716	143	7	2.231
BLVRB	10	467	0.91191	0.97648	0.999059	467	3	3.462	83	0.04817	0.18381	0.999716	83	7	3.462
DEGS1	10	468	0.91331	0.97687	0.999059	468	3	1.5256	316	0.40325	0.70701	0.999716	316	5	1.5256
KDM6A	10	469	0.9223	0.97923	0.999059	469	3	2.5853	163	0.12331	0.36148	0.999716	163	6	2.5853
PTGS1	10	470	0.92714	0.9808	0.999059	470	3	1.7841	85	0.051325	0.19269	0.999716	85	6	1.7841
CYP2C19	10	471	0.943	0.98562	0.999059	471	1	2.4478	45	0.01918	0.090342	0.940476	45	8	2.4478
ATOX1	10	472	0.94507	0.98596	0.999059	472	2	3.5111	36	0.015172	0.074754	0.940476	36	6	3.5111
NIPA1	10	473	0.94925	0.98654	0.999059	473	2	4.3892	21	0.0075022	0.041281	0.874504	21	7	4.3892
RORA	10	474	0.97981	0.99331	0.999896	474	2	1.5644	60	0.029903	0.13092	0.999716	60	8	1.5644
P4HA1	10	475	0.99248	0.99679	0.999896	475	1	2.2084	32	0.014116	0.07042	0.940476	32	8	2.2084
HEPH	10	476	0.99642	0.99771	0.999896	476	1	4.4856	6	0.0009336	0.006387	0.509921	6	9	4.4856
ALKBH2	10	477	0.99672	0.99779	0.999896	477	1	3.3535	51	0.024191	0.10966	0.999716	51	8	3.3535
AFP	10	478	0.99745	0.99804	0.999896	478	1	2.9152	52	0.024577	0.11113	0.999716	52	8	2.9152

HFE	10	479	0.99983	0.9999	0.999896	479	0	4.9538	2	0.00017106	0.001337	0.320238	2	10	4.9538

Total dropped out sgRNAs
2861

Supplemental Table S5. GSEA analysis using MSigDB-HALLMARK of Mouse #1

id	num	neg|score	neg|rra	neg|p-value	neg|fdr	neg|rank	neg|goodgene	neg|lfc	pos|score	pos|rra	pos|p-value	pos|fdr	pos|rank	pos|goodgene	pos|lfc
HALLMARK_OXIDATIVE_PHOSPHORYLATION	200	0.539094	5.67E-05	0.001	0.0083333	6	17	0	0.177258	0.162013	0.541	0.567708	46	7	0
HALLMARK_ESTROGEN_RESPONSE_EARLY	200	0.639475	0.0043129	0.004	0.0285714	7	3	0	0.334425	0.016986	0.313	0.456579	32	1	0
HALLMARK_FATTY_ACID_METABOLISM	158	0.555583	0.0200083	0.015	0.09375	8	5	0	0.325355	0.325607	0.244	0.456579	24	3	0
HALLMARK_DNA_REPAIR	150	0.633398	0.0011436	0.019	0.095	9	4	0	0.217156	0	0.594	0.606122	49	0	0
HALLMARK_P53_PATHWAY	200	0.586972	0.0013658	0.019	0.095	10	1	0	0.269161	0.016986	0.412	0.47907	43	1	0
HALLMARK_E2F_TARGETS	200	0.692632	0.0029802	0.023	0.104545	11	4	0	0.376075	0	0.338	0.456579	35	0	0
HALLMARK_PEROXISOME	104	0.656561	0.097034	0.045	0.166667	12	2	0	0.543963	0.08146	0.128	0.32	20	1	0
HALLMARK_ADIPOGENESIS	200	0.54762	0.0163511	0.047	0.166667	13	4	0	0.346754	0.056229	0.268	0.456579	29	3	0
HALLMARK_MITOTIC_SPINDLE	199	0.676321	0.0030556	0.071	0.221875	16	1	0	0.418261	0	0.345	0.456579	37	0	0
HALLMARK_MYC_TARGETS_V1	200	0.569151	0.0109408	0.082	0.233333	17	2	0	0.25026	0	0.545	0.567708	48	0	0
HALLMARK_G2M_CHECKPOINT	200	0.564208	0.0074085	0.084	0.233333	18	1	0	0.387903	0	0.266	0.456579	27	0	0
HALLMARK_ALLOGRAFT_REJECTION	200	0.478333	0.008045	0.091	0.239474	19	2	0	0.355769	0	0.246	0.456579	25	0	0

Supplemental Table S6. GSEA analysis using MSigDB-HALLMARK of Mouse #2

id	num	neg|score	neg|rra	neg|p-value	neg|fdr	neg|rank	neg|goodgene	neg|lfc	pos|score	pos|rra	pos|p-value	pos|fdr	pos|rank	pos|goodgene	pos|lfc
HALLMARK_OXIDATIVE_PHOSPHORYLATION	200	0.541415	3.74E-05	0	0	6	13	0	0.102057	0.406635	0.809	0.809	50	3	0
HALLMARK_ADIPOGENESIS	200	0.647717	0.0156073	0.006	0.0428571	7	4	0	0.378872	0.072507	0.202	0.336667	30	3	0
HALLMARK_TGF_BETA_SIGNALING	54	0.863193	0.0098113	0.009	0.05625	8	1	0	0.615603	0.009136	0.176	0.319643	25	1	0
HALLMARK_PI3K_AKT_MTOR_SIGNALING	105	0.943515	0.0034028	0.013	0.0722222	9	1	0	0.518319	0	0.368	0.44878	41	0	0
HALLMARK_E2F_TARGETS	200	0.701747	0.0062018	0.026	0.127273	10	3	0	0.278196	0	0.497	0.542391	45	0	0
HALLMARK_APICAL_SURFACE	44	0.878661	0.0151085	0.028	0.127273	11	1	0	0.561916	0	0.317	0.428947	36	0	0
HALLMARK_ANGIOGENESIS	36	0.878661	0.0151085	0.049	0.204167	12	1	0	0.561916	0	0.326	0.428947	38	0	0
HALLMARK_DNA_REPAIR	150	0.570584	0.004672	0.056	0.21	13	4	0	0.478329	0.052535	0.137	0.319643	18	2	0
HALLMARK_UV_RESPONSE_DN	144	0.725131	0.108031	0.06	0.21	14	1	0	0.637317	0.048461	0.143	0.319643	20	2	0
HALLMARK_MITOTIC_SPINDLE	199	0.675672	0.0273365	0.063	0.21	15	1	0	0.435688	0	0.316	0.428947	34	0	0
HALLMARK_INTERFERON_ALPHA_RESPONSE	97	0.737945	0.018522	0.071	0.221875	16	2	0	0.454202	0	0.347	0.444872	39	0	0
HALLMARK_FATTY_ACID_METABOLISM	158	0.420372	0.0876958	0.08	0.230556	17	3	0	0.135914	0	0.723	0.737755	49	0	0
HALLMARK_BILE_ACID_METABOLISM	112	0.408265	0.0929134	0.083	0.230556	18	4	0	0.443297	0.014038	0.05	0.208333	12	5	0

Supplemental Table S7. GSEA analysis using MSigDB-HALLMARK of Mouse #3

id	num	neg|score	neg|rra	neg|p-value	neg|fdr	neg|rank	neg|goodgene	neg|lfc	pos|score	pos|rra	pos|p-value	pos|fdr	pos|rank	pos|goodgene	pos|lfc
HALLMARK_OXIDATIVE_PHOSPHORYLATION	200	0.565437	4.43E-05	0	0	6	17	0	0.0972584	0.041327	0.818	0.818	50	1	0
HALLMARK_MYC_TARGETS_V1	200	0.74599	0.0018538	0.001	0.0071429	7	3	0	0.385931	0.010232	0.266	0.369444	36	1	0
HALLMARK_MITOTIC_SPINDLE	199	0.79602	2.60E-05	0.011	0.0611111	8	1	0	0.376959	0	0.378	0.45	42	0	0
HALLMARK_ADIPOGENESIS	200	0.600256	0.0063373	0.011	0.0611111	9	5	0	0.301203	0.093205	0.353	0.430488	40	1	0
HALLMARK_DNA_REPAIR	150	0.670378	0.0003512	0.016	0.0772727	10	3	0	0.248066	0	0.502	0.534043	47	0	0
HALLMARK_UV_RESPONSE_UP	158	0.6416	0.0013967	0.017	0.0772727	11	3	0	0.452567	0.138902	0.149	0.275926	27	1	0
HALLMARK_P53_PATHWAY	200	0.588463	0.0355271	0.02	0.0833333	12	3	0	0.237063	0	0.492	0.534043	46	0	0
HALLMARK_G2M_CHECKPOINT	200	0.657092	0.0001264	0.03	0.115385	13	2	0	0.237179	0	0.593	0.613265	48	0	0
HALLMARK_PI3K_AKT_MTOR_SIGNALING	105	0.874477	0.0161502	0.033	0.117857	14	1	0	0.636243	0	0.241	0.365152	33	0	0
HALLMARK_E2F_TARGETS	200	0.653025	0.0517197	0.044	0.1375	16	2	0	0.285799	0	0.465	0.516667	45	0	0
HALLMARK_MTORC1_SIGNALING	200	0.477441	0.0158046	0.057	0.140476	18	5	0	0.440104	0.001532	0.095	0.25	19	4	0
HALLMARK_TGF_BETA_SIGNALING	54	0.750126	0.0443457	0.058	0.140476	19	1	0	0.571938	0	0.201	0.324194	31	0	0
HALLMARK_TNFA_SIGNALING_VIA_NFKB	200	0.59072	0.0278453	0.058	0.140476	20	2	0	0.520694	0.090564	0.125	0.265385	23	1	0
HALLMARK_REACTIVE_OXYGEN_SPECIES_PATHWAY	49	0.4386	0.0554797	0.086	0.195455	22	3	0	0.396635	0.060284	0.136	0.265385	25	6	0
HALLMARK_PEROXISOME	104	0.605243	0.0434362	0.091	0.197826	23	1	0	0.432943	0	0.257	0.367143	35	0	0
HALLMARK_MYOGENESIS	200	0.487656	0.014141	0.108	0.225	24	3	0	0.537207	0.021951	0.067	0.223333	15	4	0
HALLMARK_APOPTOSIS	161	0.473194	0.0151805	0.113	0.226	25	2	0	0.28063	0	0.403	0.468605	43	0	0

Supplemental Table S8. Transcripts Per Million (TPM) counts of non-treated and treated NK92 cells in connectivity scoring analysis shown in Figure 2

Gene_ID	Transcript_ID	Gene_Symbol	Description	gene_biotype	NK92-non-treat_TPM	NK92-treat_TPM	[NK92-non-treat_TPM]+1	[NK92-treat_TPM]+1	FC (NK92-treat/non-treat)
1	NM_130786	**A1BG**	alpha-1-B glycoprotein	protein_coding	0	0	1	1	1.000
2	NM_000014,NM_00	**A2M**	alpha-2-macroglobulin	protein_coding	0	0	1	1	0.981
3	NR_040112	**A2MP1**	alpha-2-macroglobulin p	transcribed_pseu	0	0	1	1	1.000
9	NM_000662,NM_00	**NAT1**	N-acetyltransferase 1	protein_coding	8	9	9	10	1.039
10	NM_000015,XM_01	**NAT2**	N-acetyltransferase 2	protein_coding	0	0	1	1	1.000
12	NM_001085	**SERPINA3**	serpin family A member 3	protein_coding	0	0	1	1	1.000
13	NM_001086,XM_00	**AADAC**	arylacetamide deacetylas	protein_coding	0	0	1	1	1.000
14	NM_001087,NM_00	**AAMP**	angio associated migratc	protein_coding	291	284	292	285	0.977
15	NM_001088,NM_00	**AANAT**	aralkylamine N-acetyltrar	protein_coding	0	0	1	1	1.047
16	NM_001605,XR_93	**AARS1**	alanyl-tRNA synthetase 1	protein_coding	78	78	79	79	0.995
18	NM_000663,NM_00	**ABAT**	4-aminobutyrate aminotra	protein_coding	0	0	1	1	1.025
19	NM_005502,XM_00	**ABCA1**	ATP binding cassette sul	protein_coding	0	0	1	1	1.024
20	NM_001606,NM_21	**ABCA2**	ATP binding cassette sul	protein_coding	22	22	23	23	1.004
21	NM_001089	**ABCA3**	ATP binding cassette sul	protein_coding	0	0	1	1	1.030
22	NM_001271696,NM	**ABCB7**	ATP binding cassette sul	protein_coding	19	17	20	18	0.898
23	NM_001025091,NM	**ABCF1**	ATP binding cassette sul	protein_coding	157	155	158	156	0.983
24	NM_000350	**ABCA4**	ATP binding cassette sul	protein_coding	0	0	1	1	1.000
25	NM_005157,NM_00	**ABL1**	ABL proto-oncogene 1, r	protein_coding	26	25	27	26	0.962
26	NM_001091,NM_00	**AOC1**	amine oxidase copper cc	protein_coding	0	0	1	1	1.000
27	NM_001136000,NM	**ABL2**	ABL proto-oncogene 2, r	protein_coding	11	8	12	9	0.762
28	NM_020469	**ABO**	ABO, alpha 1-3-N-acetyl	protein_coding	0	0	1	1	1.000
29	NM_001092,NM_00	**ABR**	ABR activator of RhoGEI	protein_coding	21	21	22	22	1.006
30	NM_001130410,NM	**ACAA1**	acetyl-CoA acyltransfera	protein_coding	36	37	37	38	1.049
31	NM_198834,NM_19	**ACACA**	acetyl-CoA carboxylase a	protein_coding	22	19	23	20	0.848
32	NM_001093,XM_00	**ACACB**	acetyl-CoA carboxylase b	protein_coding	0	0	1	1	1.017
33	NM_001608,XM_00	**ACADL**	acyl-CoA dehydrogenase	protein_coding	0	0	1	1	1.000
34	NM_000016,NM_00	**ACADM**	acyl-CoA dehydrogenase	protein_coding	125	113	126	114	0.901
35	NM_000017,NM_00	**ACADS**	acyl-CoA dehydrogenase	protein_coding	0	0	1	1	1.149
36	NM_001330174,NM	**ACADSB**	acyl-CoA dehydrogenase	protein_coding	5	5	6	6	0.974
37	NM_000018,NM_00	**ACADVL**	acyl-CoA dehydrogenase	protein_coding	60	57	61	58	0.953
38	NM_000019,XM_00	**ACAT1**	acetyl-CoA acetyltransfer	protein_coding	134	131	135	132	0.973
39	NM_001303253,NM	**ACAT2**	acetyl-CoA acetyltransfer	protein_coding	104	94	105	95	0.910
40	NM_001094,NM_18	**ASIC2**	acid sensing ion channel	protein_coding	0	0	1	1	1.000
41	NM_001095,NM_00	**ASIC1**	acid sensing ion channel	protein_coding	0	0	1	1	1.029
43	NM_000665,NM_00	**ACHE**	acetylcholinesterase (Ca	protein_coding	0	0	1	1	1.000
47	NM_001096,NM_00	**ACLY**	ATP citrate lyase	protein_coding	260	249	261	250	0.959
48	NM_001278352,NM	**ACO1**	aconitase 1	protein_coding	1	1	2	2	0.906
49	NM_001097	**ACR**	acrosin	protein_coding	0	0	1	1	1.000
50	NM_001098,XM_01	**ACO2**	aconitase 2	protein_coding	157	161	158	162	1.021
51	NM_001185039,NM	**ACOX1**	acyl-CoA oxidase 1	protein_coding	8	8	9	9	0.951
52	NM_001040649,NM	**ACP1**	acid phosphatase 1	protein_coding	185	177	186	178	0.954
53	NM_001302489,NM	**ACP2**	acid phosphatase 2, lysc	protein_coding	31	38	32	39	1.224
54	NM_001111034,NM	**ACP5**	acid phosphatase 5, tartı	protein_coding	0	0	1	1	0.914
55	NM_001099,NM_00	**ACP3**	acid phosphatase 3	protein_coding	0	0	1	1	1.000
56	NM_001612,NM_02	**ACRV1**	acrosomal vesicle proteir	protein_coding	0	0	1	1	1.000
58	NM_001100	**ACTA1**	actin alpha 1, skeletal m	protein_coding	0	0	1	1	0.978
59	NM_001141945,NM	**ACTA2**	actin alpha 2, smooth mu	protein_coding	0	1	1	2	1.133
60	NM_001101	**ACTB**	actin beta	protein_coding	7,775	8,598	7,776	8,599	1.106
62	gene-ACTBP2	**ACTBP2**	ACTB pseudogene 2	pseudogene	0	0	1	1	0.886
64	gene-ACTBP4	**ACTBP4**	ACTB pseudogene 4	pseudogene	0	0	1	1	1.062
67	gene-ACTBP7	**ACTBP7**	ACTB pseudogene 7	pseudogene	0	0	1	1	1.023
68	gene-ACTBP8	**ACTBP8**	ACTB pseudogene 8	pseudogene	0	0	1	1	0.983
69	gene-ACTBP9	**ACTBP9**	ACTB pseudogene 9	pseudogene	0	0	1	1	0.967
70	NM_005159	**ACTC1**	actin alpha cardiac musc	protein_coding	0	0	1	1	1.000
71	NM_001199954,NM	**ACTG1**	actin gamma 1	protein_coding	5,306	5,508	5,307	5,509	1.038
72	NM_001199893,NM	**ACTG2**	actin gamma 2, smooth r	protein_coding	0	0	1	1	1.000
73	gene-ACTG1P1	**ACTG1P1**	actin gamma 1 pseudoge	pseudogene	0	0	1	1	1.047
75	gene-ACTG1P3	**ACTG1P3**	actin gamma 1 pseudoge	pseudogene	0	0	1	1	0.934
81	NM_001322033,NM	**ACTN4**	actinin alpha 4	protein_coding	264	274	265	275	1.036
82	gene-ACTG1P9	**ACTG1P9**	actin gamma 1 pseudoge	pseudogene	0	0	1	1	0.987
83	gene-ACTG1P10	**ACTG1P10**	actin gamma 1 pseudoge	pseudogene	0	0	1	1	1.000
86	NM_004301,NM_17	**ACTL6A**	actin like 6A	protein_coding	123	116	124	117	0.949
87	NM_001102,NM_00	**ACTN1**	actinin alpha 1	protein_coding	0	0	1	1	1.000
88	NM_001103,NM_00	**ACTN2**	actinin alpha 2	protein_coding	0	0	1	1	1.051
89	NM_001104,NM_00	**ACTN3**	actinin alpha 3	protein_coding	0	0	1	1	1.000
90	NM_001105,NM_00	**ACVR1**	activin A receptor type 1	protein_coding	4	4	5	5	0.925
91	NM_004302,NM_02	**ACVR1B**	activin A receptor type 1I	protein_coding	19	15	20	16	0.783
92	NM_001278579,NM	**ACVR2A**	activin A receptor type 2/	protein_coding	1	1	2	2	0.943
93	NM_001106,XM_00	**ACVR2B**	activin A receptor type 2I	protein_coding	0	0	1	1	0.998
94	NM_000020,NM_00	**ACVRL1**	activin A receptor like typ	protein_coding	0	0	1	1	0.978
95	NM_000666,NM_00	**ACY1**	aminoacylase 1	protein_coding	4	4	5	5	0.938
97	NM_001107,NM_00	**ACYP1**	acylphosphatase 1	protein_coding	72	75	73	76	1.033
98	NM_001320586,NM	**ACYP2**	acylphosphatase 2	protein_coding	4	5	5	6	1.129
100	NM_000022,NM_00	**ADA**	adenosine deaminase	protein_coding	13	14	14	15	1.082
101	NM_001109,NM_00	**ADAM8**	ADAM metallopeptidase	protein_coding	2	1	3	2	0.755
102	NM_001110,NM_00	**ADAM10**	ADAM metallopeptidase	protein_coding	40	45	41	46	1.132
103	NM_001025107,NM	**ADAR**	adenosine deaminase RI	protein_coding	217	190	218	191	0.876
104	NM_001112,NM_00	**ADARB1**	adenosine deaminase RI	protein_coding	47	47	48	48	1.011
105	NM_018702	**ADARB2**	adenosine deaminase RI	protein_coding	0	0	1	1	1.000
107	NM_001281768,NM	**ADCY1**	adenylate cyclase 1	protein_coding	0	0	1	1	1.000
108	NM_020546,XM_01	**ADCY2**	adenylate cyclase 2	protein_coding	0	0	1	1	1.000
109	NM_001320613,NM	**ADCY3**	adenylate cyclase 3	protein_coding	145	129	146	130	0.886
111	NM_001199642,NM	**ADCY5**	adenylate cyclase 5	protein_coding	0	0	1	1	0.929
112	NM_015270,XM_00	**ADCY6**	adenylate cyclase 6	protein_coding	0	0	1	1	1.016
113	NM_001114,NM_00	**ADCY7**	adenylate cyclase 7	protein_coding	35	30	36	31	0.872
114	NM_001115,XM_00	**ADCY8**	adenylate cyclase 8	protein_coding	0	0	1	1	1.000
115	NM_001116,XM_00	**ADCY9**	adenylate cyclase 9	protein_coding	0	0	1	1	1.000
116	NM_001099733,NM	**ADCYAP1**	adenylate cyclase activa	protein_coding	0	0	1	1	1.000
117	NM_001118,NM_00	**ADCYAP1R1**	ADCYAP receptor type I	protein_coding	2	2	3	3	1.026
118	NM_001119,NM_00	**ADD1**	adducin 1	protein_coding	113	109	114	110	0.962
119	NM_001185054,NM	**ADD2**	adducin 2	protein_coding	0	0	1	1	1.000
120	NM_001121,NM_00	**ADD3**	adducin 3	protein_coding	105	99	106	100	0.943
123	NM_001122,NR_03	**PLIN2**	perilipin 2	protein_coding	9	8	10	9	0.852
124	NM_000667	**ADH1A**	alcohol dehydrogenase 1	protein_coding	0	0	1	1	1.000
125	NM_000668,NM_00	**ADH1B**	alcohol dehydrogenase 1	protein_coding	0	0	1	1	1.000
126	NM_000669,NR_13	**ADH1C**	alcohol dehydrogenase 1	protein_coding	0	0	1	1	1.000
127	NM_000670,NM_00	**ADH4**	alcohol dehydrogenase 4	protein_coding	0	0	1	1	1.000
128	NM_000671	**ADH5**	alcohol dehydrogenase 5	protein_coding	72	77	73	78	1.059
130	NM_000672,NM_00	**ADH6**	alcohol dehydrogenase 6	protein_coding	0	0	1	1	1.000
131	NM_000673,NM_00	**ADH7**	alcohol dehydrogenase 7	protein_coding	0	0	1	1	1.000
132	NM_001123,NM_00	**ADK**	adenosine kinase	protein_coding	64	57	65	58	0.900
133	NM_001124	**ADM**	adrenomedullin	protein_coding	0	0	1	1	1.000
134	NM_000674,NM_00	**ADORA1**	adenosine A1 receptor	protein_coding	0	0	1	1	1.000
135	NM_000675,NM_00	**ADORA2A**	adenosine A2a receptor	protein_coding	28	32	29	33	1.135
136	NM_000676,XM_01	**ADORA2B**	adenosine A2b receptor	protein_coding	0	0	1	1	1.000
137	gene-ADORA2BP1	**ADORA2BP1**	adenosine A2b receptor	pseudogene	0	0	1	1	1.000
140	NM_000677,NM_00	**ADORA3**	adenosine A3 receptor	protein_coding	0	0	1	1	1.000
141	NM_001125,NM_00	**ADPRH**	ADP-ribosylarginine hydı	protein_coding	0	0	1	1	1.027
142	NM_001618	**PARP1**	poly(ADP-ribose) polyme	protein_coding	231	219	232	220	0.949
143	NM_006437,XM_01	**PARP4**	poly(ADP-ribose) polyme	protein_coding	58	51	59	52	0.884
144	gene-PARP1P1	**PARP1P1**	poly(ADP-ribose) polyme	pseudogene	0	0	1	1	1.000
145	gene-PARP1P2	**PARP1P2**	poly(ADP-ribose) polyme	pseudogene	0	0	1	1	1.000
146	NM_000678	**ADRA1D**	adrenoceptor alpha 1D	protein_coding	0	0	1	1	1.000
147	NM_000679,XM_00	**ADRA1B**	adrenoceptor alpha 1B	protein_coding	0	0	1	1	1.000
148	NM_000680,NM_00	**ADRA1A**	adrenoceptor alpha 1A	protein_coding	0	0	1	1	1.000
150	NM_000681	**ADRA2A**	adrenoceptor alpha 2A	protein_coding	0	0	1	1	1.000
151	NM_000682	**ADRA2B**	adrenoceptor alpha 2B	protein_coding	0	0	1	1	1.000
152	NM_000683	**ADRA2C**	adrenoceptor alpha 2C	protein_coding	0	0	1	1	1.000
153	NM_000684	**ADRB1**	adrenoceptor beta 1	protein_coding	7	7	8	8	0.927
154	NM_000024	**ADRB2**	adrenoceptor beta 2	protein_coding	0	0	1	1	0.960
155	NM_000025	**ADRB3**	adrenoceptor beta 3	protein_coding	0	0	1	1	1.000
156	NM_001619,XM_01	**GRK2**	G protein-coupled recept	protein_coding	314	330	315	331	1.051

157	NM_001362778,NM	**GRK3**	G protein-coupled recept	protein_coding	0	0	1	1	1.000
158	NM_000026,NM_00	**ADSL**	adenylosuccinate lyase	protein_coding	124	111	125	112	0.899
159	NM_001126,NM_00	**ADSS2**	adenylosuccinate syntha	protein_coding	116	110	117	111	0.949
160	NM_014203,NM_13	**AP2A1**	adaptor related protein c	protein_coding	79	79	80	80	1.001
161	NM_001242837,NM	**AP2A2**	adaptor related protein c	protein_coding	21	19	22	20	0.907
162	NM_001127,NM_00	**AP1B1**	adaptor related protein c	protein_coding	96	99	97	100	1.034
163	NM_001030006,NM	**AP2B1**	adaptor related protein c	protein_coding	229	210	230	211	0.920
164	NM_001030007,NM	**AP1G1**	adaptor related protein c	protein_coding	52	45	53	46	0.874
165	NM_001129,XM_01	**AEBP1**	AE binding protein 1	protein_coding	0	0	1	1	1.000
166	NM_001130,NM_19	**TLE5**	TLE family member 5, tra	protein_coding	169	170	170	171	1.003
167	NM_001131,NM_00	**CRISP1**	cysteine rich secretory pr	protein_coding	0	0	1	1	1.000
172	NR_003226,NR_00	**AFG3L1P**	AFG3 like matrix AAA pe	transcribed_pseu	16	19	17	20	1.162
173	NM_001133,XM_01	**AFM**	afamin	protein_coding	0	0	1	1	1.000
174	NM_001134,NM_00	**AFP**	alpha fetoprotein	protein_coding	0	0	1	1	1.000
175	NM_000027,NM_00	**AGA**	aspartylglucosaminidase	protein_coding	7	8	8	9	1.045
176	NM_001135,NM_00	**ACAN**	aggrecan	protein_coding	0	0	1	1	1.000
177	NM_001136,NM_00	**AGER**	advanced glycosylation e	protein_coding	4	4	5	5	0.913
178	NM_000028,NM_00	**AGL**	amylo-alpha-1, 6-glucosi	protein_coding	21	16	22	17	0.779
181	NM_001138	**AGRP**	agouti related neuropept	protein_coding	0	0	1	1	1.000
182	NM_000214	**JAG1**	jagged canonical Notch l	protein_coding	0	0	1	1	0.981
183	NM_000029,NM_00	**AGT**	angiotensinogen	protein_coding	0	0	1	1	1.000
185	NM_000685,NM_00	**AGTR1**	angiotensin II receptor ty	protein_coding	0	0	1	1	1.000
186	NM_000686,XM_01	**AGTR2**	angiotensin II receptor ty	protein_coding	0	0	1	1	1.000
187	NM_005161,NR_02	**APLNR**	apelin receptor	protein_coding	0	0	1	1	1.000
189	NM_000030	**AGXT**	alanine--glyoxylate and s	protein_coding	0	0	1	1	1.000
190	NM_000475	**NR0B1**	nuclear receptor subfam	protein_coding	0	0	1	1	1.000
191	NM_000687,NM_00	**AHCY**	adenosylhomocysteinase	protein_coding	471	466	472	467	0.988
196	NM_001621	**AHR**	aryl hydrocarbon recepto	protein_coding	4	4	5	5	1.042
197	NM_001354571,NM	**AHSG**	alpha 2-HS glycoprotein	protein_coding	0	0	1	1	1.000
199	NM_001318970,NM	**AIF1**	allograft inflammatory fac	protein_coding	2	3	3	4	1.247
202	NM_001371242,NM	**CRYBG1**	crystallin beta-gamma do	protein_coding	29	24	30	25	0.836
203	NM_000476,NM_00	**AK1**	adenylate kinase 1	protein_coding	1	1	2	2	0.841
204	NM_001199199,NM	**AK2**	adenylate kinase 2	protein_coding	384	352	385	353	0.915
205	NM_001005353,NM	**AK4**	adenylate kinase 4	protein_coding	0	0	1	1	0.989
206	gene-AK4P1	**AK4P1**	adenylate kinase 4 pseu	pseudogene	0	0	1	1	0.925
207	NM_001014431,NM	**AKT1**	AKT serine/threonine kin	protein_coding	112	110	113	111	0.979
208	NM_001243027,NM	**AKT2**	AKT serine/threonine kin	protein_coding	77	81	78	82	1.045
210	NM_000031,NM_00	**ALAD**	aminolevulinate dehydra	protein_coding	13	12	14	13	0.863
211	NM_000688,NM_00	**ALAS1**	5'-aminolevulinate syntha	protein_coding	47	51	48	52	1.081
212	NM_000032,NM_00	**ALAS2**	5'-aminolevulinate syntha	protein_coding	0	0	1	1	1.000
213	NM_000477	**ALB**	albumin	protein_coding	0	0	1	1	1.000
214	NM_001243280,NM	**ALCAM**	activated leukocyte cell a	protein_coding	6	6	7	7	0.937
215	NM_000033,XR_93	**ABCD1**	ATP binding cassette sul	protein_coding	2	2	3	3	1.103
216	NM_000689	**ALDH1A1**	aldehyde dehydrogenase	protein_coding	2	2	3	3	0.967
217	NM_000690,NM_00	**ALDH2**	aldehyde dehydrogenase	protein_coding	0	0	1	1	0.975
218	NM_000691,NM_00	**ALDH3A1**	aldehyde dehydrogenase	protein_coding	0	0	1	1	1.000
219	NM_000692,XM_01	**ALDH1B1**	aldehyde dehydrogenase	protein_coding	31	29	32	30	0.953
220	NM_000693,NM_00	**ALDH1A3**	aldehyde dehydrogenase	protein_coding	0	0	1	1	1.000
221	NM_000694,NM_00	**ALDH3B1**	aldehyde dehydrogenase	protein_coding	0	0	1	1	1.171
222	NM_001031615,NM	**ALDH3B2**	aldehyde dehydrogenase	protein_coding	0	0	1	1	1.000
223	NM_000696,NM_00	**ALDH9A1**	aldehyde dehydrogenase	protein_coding	89	85	90	86	0.954
224	NM_000382,NM_00	**ALDH3A2**	aldehyde dehydrogenase	protein_coding	18	17	19	18	0.964
225	NM_005164,XM_01	**ABCD2**	ATP binding cassette sul	protein_coding	1	1	2	2	0.806
226	NM_001127617,NM	**ALDOA**	aldolase, fructose-bisphc	protein_coding	1,441	1,546	1,442	1,547	1.073
228	gene-ALDOAP2	**ALDOAP2**	ALDOA pseudogene 2	pseudogene	0	0	1	1	1.000
229	NM_000035	**ALDOB**	aldolase, fructose-bisphc	protein_coding	0	0	1	1	1.000
230	NM_005165,XM_00	**ALDOC**	aldolase, fructose-bisphc	protein_coding	2	2	3	3	0.908
231	NM_001346142,NM	**AKR1B1**	aldo-keto reductase fami	protein_coding	243	266	244	267	1.093
236	gene-AKR1B1P2	**AKR1B1P2**	aldo-keto reductase fami	pseudogene	0	0	1	1	1.000
238	NM_001353765,NM	**ALK**	ALK receptor tyrosine kin	protein_coding	0	0	1	1	1.000
239	NM_000697,XM_01	**ALOX12**	arachidonate 12-lipoxyge	protein_coding	0	0	1	1	1.000
240	NM_000698,NM_00	**ALOX5**	arachidonate 5-lipoxyger	protein_coding	0	0	1	1	1.000
241	NM_001204406,NM	**ALOX5AP**	arachidonate 5-lipoxyger	protein_coding	394	354	395	355	0.900
242	NM_001139	**ALOX12B**	arachidonate 12-lipoxyge	protein_coding	0	0	1	1	0.898
243	gene-ALOX12P1	**ALOX12P1**	arachidonate 12-lipoxyge	pseudogene	0	0	1	1	1.000
245	NR_002710,NR_12	**ALOX12P2**	arachidonate 12-lipoxyge	transcribed_pseu	0	0	1	1	1.000
246	NM_001140	**ALOX15**	arachidonate 15-lipoxyge	protein_coding	0	0	1	1	1.000
247	NM_001039130,NM	**ALOX15B**	arachidonate 15-lipoxyge	protein_coding	0	0	1	1	1.037
248	NM_001631	**ALPI**	alkaline phosphatase, int	protein_coding	6	10	7	11	1.542
249	NM_000478,NM_00	**ALPL**	alkaline phosphatase, bi	protein_coding	0	0	1	1	1.039
250	NM_001632	**ALPP**	alkaline phosphatase, pl	protein_coding	0	0	1	1	1.000
251	NM_031313	**ALPG**	alkaline phosphatase, ge	protein_coding	0	0	1	1	1.000
257	NM_006492	**ALX3**	ALX homeobox 3	protein_coding	0	0	1	1	1.000
258	NM_016519	**AMBN**	ameloblastin	protein_coding	0	0	1	1	1.000
259	NM_001633	**AMBP**	alpha-1-microglobulin/bil	protein_coding	0	0	1	1	1.000
262	NM_001287214,NM	**AMD1**	adenosylmethionine dec	protein_coding	167	141	168	142	0.845
263	gene-AMD1P2,gene	**AMD1P2**	adenosylmethionine dec	pseudogene	0	0	1	1	1.000
265	NM_001142,NM_18	**AMELX**	amelogenin X-linked	protein_coding	0	0	1	1	1.000
266	NM_001143,NM_00	**AMELY**	amelogenin Y-linked	protein_coding	0	0	1	1	1.000
267	NM_001144,NM_00	**AMFR**	autocrine motility factor r	protein_coding	24	24	25	25	0.985
268	NM_000479	**AMH**	anti-Mullerian hormone	protein_coding	2	2	3	3	0.887
269	NM_001164690,NM	**AMHR2**	anti-Mullerian hormone r	protein_coding	0	0	1	1	1.000
270	NM_000036,NM_00	**AMPD1**	adenosine monophospha	protein_coding	0	0	1	1	1.000
271	NM_001257361,NM	**AMPD2**	adenosine monophospha	protein_coding	75	73	76	74	0.977
272	NM_000480,NM_00	**AMPD3**	adenosine monophospha	protein_coding	16	14	17	15	0.901
273	NM_001635,NM_13	**AMPH**	amphiphysin	protein_coding	0	0	1	1	1.010
274	NM_001320632,NM	**BIN1**	bridging integrator 1	protein_coding	79	83	80	84	1.050
275	NM_000481,NM_00	**AMT**	aminomethyltransferase	protein_coding	0	0	1	1	1.263
276	NM_001008221,NM	**AMY1A**	amylase alpha 1A	protein_coding	0	0	1	1	1.066
277	NM_001008218,XM	**AMY1B**	amylase alpha 1B	protein_coding	0	0	1	1	1.000
278	NM_001008219,NM	**AMY1C**	amylase alpha 1C	protein_coding	0	0	1	1	1.070
279	NM_000699	**AMY2A**	amylase alpha 2A	protein_coding	0	0	1	1	1.000
280	NM_020978	**AMY2B**	amylase alpha 2B	protein_coding	2	2	3	3	1.005
281	gene-AMYP1	**AMYP1**	amylase alpha pseudoge	pseudogene	0	0	1	1	1.000
283	NM_001097577,NM	**ANG**	angiogenin	protein_coding	0	0	1	1	0.888
284	NM_001146,NM_00	**ANGPT1**	angiopoietin 1	protein_coding	0	0	1	1	1.000
285	NM_001118887,NM	**ANGPT2**	angiopoietin 2	protein_coding	0	0	1	1	1.000
286	NM_000037,NM_00	**ANK1**	ankyrin 1	protein_coding	0	0	1	1	1.000
287	NM_001127493,NM	**ANK2**	ankyrin 2	protein_coding	0	0	1	1	0.986
288	NM_001149,NM_00	**ANK3**	ankyrin 3	protein_coding	0	0	1	1	0.912
290	NM_001150,NM_00	**ANPEP**	alanyl aminopeptidase, m	protein_coding	0	0	1	1	1.027
291	NM_001151	**SLC25A4**	solute carrier family 25 m	protein_coding	0	0	1	1	1.000
292	NM_001152	**SLC25A5**	solute carrier family 25 m	protein_coding	1,131	1,129	1,132	1,130	0.998
293	NM_001636,NM_00	**SLC25A6**	solute carrier family 25 m	protein_coding	442	464	443	465	1.049
301	NM_000700,XM_01	**ANXA1**	annexin A1	protein_coding	26	31	27	32	1.180
302	NM_001002857,NM	**ANXA2**	annexin A2	protein_coding	394	440	395	441	1.118
303	NR_001562	**ANXA2P1**	annexin A2 pseudogene	transcribed_pseu	0	0	1	1	1.000
304	NR_003573	**ANXA2P2**	annexin A2 pseudogene	transcribed_pseu	0	1	1	2	1.366
305	NR_001446	**ANXA2P3**	annexin A2 pseudogene	transcribed_pseu	0	0	1	1	1.000
306	NM_005139,XR_00	**ANXA3**	annexin A3	protein_coding	0	0	1	1	1.000
307	NM_001153,NM_00	**ANXA4**	annexin A4	protein_coding	18	18	19	19	1.004
308	NM_001154,XM_01	**ANXA5**	annexin A5	protein_coding	208	209	209	210	1.007
309	NM_001155,NM_00	**ANXA6**	annexin A6	protein_coding	138	131	139	132	0.950
310	NM_001156,NM_00	**ANXA7**	annexin A7	protein_coding	62	62	63	63	0.990
311	NM_001157,NM_00	**ANXA11**	annexin A11	protein_coding	46	53	47	54	1.145
312	NM_001003954,NM	**ANXA13**	annexin A13	protein_coding	0	0	1	1	1.000
313	NM_001177506,NM	**AOAH**	acyloxyacyl hydrolase	protein_coding	2	1	3	2	0.725
314	NM_001158,NM_00	**AOC2**	amine oxidase copper co	protein_coding	4	4	5	5	1.095
316	NM_001159,XM_01	**AOX1**	aldehyde oxidase 1	protein_coding	0	0	1	1	0.994
317	NM_001160,NM_01	**APAF1**	apoptotic peptidase activ	protein_coding	12	11	13	12	0.876
318	NM_001161,NM_00	**NUDT2**	nudix hydrolase 2	protein_coding	30	30	31	31	1.002
319	NM_001638	**APOF**	apolipoprotein F	protein_coding	0	0	1	1	1.000
320	NM_001163,XM_00	**APBA1**	amyloid beta precursor p	protein_coding	4	3	5	4	0.695
321	NM_001130414,NM	**APBA2**	amyloid beta precursor p	protein_coding	0	0	1	1	1.051
322	NM_001164,NM_00	**APBB1**	amyloid beta precursor p	protein_coding	46	42	47	43	0.903
323	NM_001166050,NM	**APBB2**	amyloid beta precursor p	protein_coding	25	24	26	25	0.983

324	NM_000038,NM_00	**APC**	APC regulator of WNT si	protein_coding	14	11	15	12	0.826
325	NM_001639	**APCS**	amyloid P component, se	protein_coding	0	0	1	1	1.000
326	NM_000383	**AIRE**	autoimmune regulator	protein_coding	0	0	1	1	1.000
327	NM_001640,XM_00	**APEH**	acylaminoacyl-peptide hy	protein_coding	66	66	67	67	0.990
328	NM_001244249,NM	**APEX1**	apurinic/apyrimidinic end	protein_coding	248	265	249	266	1.069
329	NM_001166,NM_00	**BIRC2**	baculoviral IAP repeat cc	protein_coding	34	27	35	28	0.808
330	NM_001165,NM_18	**BIRC3**	baculoviral IAP repeat cc	protein_coding	20	16	21	17	0.804
331	NM_001167,NM_00	**XIAP**	X-linked inhibitor of apop	protein_coding	18	16	19	17	0.851
332	NM_001012270,NM	**BIRC5**	baculoviral IAP repeat cc	protein_coding	207	193	208	194	0.932
333	NM_001024807,NM	**APLP1**	amyloid beta precursor li	protein_coding	0	0	1	1	1.000
334	NM_001142276,NM	**APLP2**	amyloid beta precursor li	protein_coding	111	107	112	108	0.968
335	NM_000039,NM_00	**APOA1**	apolipoprotein A1	protein_coding	0	0	1	1	1.000
336	NM_001643	**APOA2**	apolipoprotein A2	protein_coding	0	0	1	1	1.000
337	NM_000482	**APOA4**	apolipoprotein A4	protein_coding	0	0	1	1	1.000
338	NM_000384	**APOB**	apolipoprotein B	protein_coding	0	0	1	1	1.000
339	NM_001304566,NM	**APOBEC1**	apolipoprotein B mRNA e	protein_coding	0	0	1	1	1.000
341	NM_001321065,NM	**APOC1**	apolipoprotein C1	protein_coding	0	0	1	1	0.825
342	NR_028412,NR_028	**APOC1P1**	apolipoprotein C1 pseud	transcribed_pseu	0	0	1	1	1.000
343	NM_001169,XM_01	**AQP8**	aquaporin 8	protein_coding	0	0	1	1	1.000
344	NM_000483	**APOC2**	apolipoprotein C2	protein_coding	0	0	1	1	1.000
345	NM_000040	**APOC3**	apolipoprotein C3	protein_coding	0	0	1	1	1.000
346	NM_001646	**APOC4**	apolipoprotein C4	protein_coding	0	0	1	1	1.000
347	NM_001647	**APOD**	apolipoprotein D	protein_coding	0	0	1	1	1.000
348	NM_000041,NM_00	**APOE**	apolipoprotein E	protein_coding	0	0	1	1	0.808
350	NM_000042	**APOH**	apolipoprotein H	protein_coding	0	0	1	1	1.083
351	NM_000484,NM_00	**APP**	amyloid beta precursor p	protein_coding	0	0	1	1	1.000
353	NM_000485,NM_00	**APRT**	adenine phosphoribosylt	protein_coding	179	198	180	199	1.111
354	NM_001030047,NM	**KLK3**	kallikrein related peptida	protein_coding	0	0	1	1	1.000
355	NM_000043,NM_00	**FAS**	Fas cell surface death re	protein_coding	11	9	12	10	0.867
356	NM_000639,NM_00	**FASLG**	Fas ligand	protein_coding	145	132	146	133	0.907
357	NM_001320663,NM	**SHROOM2**	shroom family member 2	protein_coding	0	0	1	1	1.000
358	NM_001329872,NM	**AQP1**	aquaporin 1 (Colton bloc	protein_coding	0	0	1	1	1.000
359	NM_000486	**AQP2**	aquaporin 2	protein_coding	0	0	1	1	1.000
360	NM_001318144,NM	**AQP3**	aquaporin 3 (Gill blood g	protein_coding	0	0	1	1	1.000
361	NM_001317384,NM	**AQP4**	aquaporin 4	protein_coding	0	0	1	1	1.000
362	NM_001651,XM_00	**AQP5**	aquaporin 5	protein_coding	0	0	1	1	1.000
363	NM_001652	**AQP6**	aquaporin 6	protein_coding	0	0	1	1	1.000
364	NM_001170,NM_00	**AQP7**	aquaporin 7	protein_coding	0	0	1	1	1.000
366	NM_001320635,NM	**AQP9**	aquaporin 9	protein_coding	0	0	1	1	1.000
367	NM_000044,NM_00	**AR**	androgen receptor	protein_coding	0	0	1	1	1.000
368	NM_001079528,NM	**ABCC6**	ATP binding cassette sul	protein_coding	0	0	1	1	1.000
369	NM_001256196,NM	**ARAF**	A-Raf proto-oncogene, s	protein_coding	62	61	63	62	0.998
372	NM_001142281,NM	**ARCN1**	archain 1	protein_coding	191	158	192	159	0.827
373	NM_001656,NM_03	**TRIM23**	tripartite motif containing	protein_coding	6	5	7	6	0.791
374	NM_001657	**AREG**	amphiregulin	protein_coding	0	0	1	1	0.709
375	NM_001024226,NM	**ARF1**	ADP ribosylation factor 1	protein_coding	393	383	394	384	0.975
377	NM_001659,XM_00	**ARF3**	ADP ribosylation factor 3	protein_coding	159	156	160	157	0.979
378	NM_001660	**ARF4**	ADP ribosylation factor 4	protein_coding	164	155	165	156	0.943
379	NM_001661,XM_01	**ARL4D**	ADP ribosylation factor li	protein_coding	0	0	1	1	1.000
380	gene-ARF4P1	**ARF4P1**	ADP ribosylation factor 4	pseudogene	0	0	1	1	1.000
381	NM_001662	**ARF5**	ADP ribosylation factor 5	protein_coding	154	166	155	167	1.077
382	NM_001663	**ARF6**	ADP ribosylation factor 6	protein_coding	152	147	153	148	0.968
383	NM_000045,NM_00	**ARG1**	arginase 1	protein_coding	0	0	1	1	1.000
384	NM_001172	**ARG2**	arginase 2	protein_coding	10	7	11	8	0.729
387	NM_001313941,NM	**RHOA**	ras homolog family mem	protein_coding	577	567	578	568	0.983
388	NM_004040	**RHOB**	ras homolog family mem	protein_coding	6	7	7	8	1.207
389	NM_001042678,NM	**RHOC**	ras homolog family mem	protein_coding	217	236	218	237	1.085
390	NM_001254738,NM	**RND3**	Rho family GTPase 3	protein_coding	0	0	1	1	1.000
391	NM_001665,XM_00	**RHOG**	ras homolog family mem	protein_coding	219	229	220	230	1.047
392	NM_004308,XM_01	**ARHGAP1**	Rho GTPase activating p	protein_coding	62	69	63	70	1.116
393	NM_001164741,NM	**ARHGAP4**	Rho GTPase activating p	protein_coding	79	88	80	89	1.119
394	NM_001030055,NM	**ARHGAP5**	Rho GTPase activating p	protein_coding	0	0	1	1	1.008
395	NM_001287242,NM	**ARHGAP6**	Rho GTPase activating p	protein_coding	0	0	1	1	1.000
396	NM_001185077,NM	**ARHGDIA**	Rho GDP dissociation inl	protein_coding	519	527	520	528	1.017
397	NM_001175,NM_00	**ARHGDIB**	Rho GDP dissociation inl	protein_coding	608	604	609	605	0.994
398	NM_001176	**ARHGDIG**	Rho GDP dissociation inl	protein_coding	0	0	1	1	1.000
399	NM_001278359,NM	**RHOH**	ras homolog family mem	protein_coding	10	11	11	12	1.026
400	NM_001177,NM_00	**ARL1**	ADP ribosylation factor li	protein_coding	35	31	36	32	0.874
401	NM_005169	**PHOX2A**	paired like homeobox 2A	protein_coding	0	0	1	1	1.000
402	NM_001199745,NM	**ARL2**	ADP ribosylation factor li	protein_coding	47	50	48	51	1.054
403	NM_004311,XM_01	**ARL3**	ADP ribosylation factor li	protein_coding	5	5	6	6	1.004
405	NM_001197325,NM	**ARNT**	aryl hydrocarbon recepto	protein_coding	22	20	23	21	0.898
406	NM_001030272,NM	**ARNTL**	aryl hydrocarbon recepto	protein_coding	17	17	18	18	1.012
407	NM_004312,XM_01	**ARR3**	arrestin 3	protein_coding	0	0	1	1	1.000
408	NM_004041,NM_02	**ARRB1**	arrestin beta 1	protein_coding	0	0	1	1	1.020
409	NM_001257328,NM	**ARRB2**	arrestin beta 2	protein_coding	27	26	28	27	0.961
410	NM_000487,NM_00	**ARSA**	arylsulfatase A	protein_coding	6	5	7	6	0.891
411	NM_000046,NM_19	**ARSB**	arylsulfatase B	protein_coding	26	24	27	25	0.915
412	NM_000351,NM_00	**STS**	steroid sulfatase	protein_coding	5	4	6	5	0.940
414	NM_001669,NM_00	**ARSD**	arylsulfatase D	protein_coding	4	4	5	5	0.905
415	NM_000047,NM_00	**ARSL**	arylsulfatase L	protein_coding	0	0	1	1	1.000
416	NM_001201538,NM	**ARSF**	arylsulfatase F	protein_coding	0	0	1	1	1.000
417	NM_004314,XM_01	**ART1**	ADP-ribosyltransferase 1	protein_coding	0	0	1	1	1.000
419	NM_001130016,NM	**ART3**	ADP-ribosyltransferase 3	protein_coding	0	0	1	1	1.000
420	NM_001354646,NM	**ART4**	ADP-ribosyltransferase 4	protein_coding	0	0	1	1	1.000
421	NM_001670,XM_00	**ARVCF**	ARVCF delta catenin fan	protein_coding	0	0	1	1	1.128
427	NM_001127505,NM	**ASAH1**	N-acylsphingosine amido	protein_coding	14	14	15	15	0.995
429	NM_004316	**ASCL1**	achaete-scute family bHl	protein_coding	0	0	1	1	1.000
430	NM_005170	**ASCL2**	achaete-scute family bHl	protein_coding	0	0	1	1	1.000
432	NM_001197216,NM	**ASGR1**	asialoglycoprotein recept	protein_coding	0	0	1	1	1.000
433	NM_001181,NM_00	**ASGR2**	asialoglycoprotein recept	protein_coding	0	0	1	1	1.000
434	NM_001672,XM_00	**ASIP**	agouti signaling protein	protein_coding	0	0	1	1	1.000
435	NM_000048,NM_00	**ASL**	argininosuccinate lyase	protein_coding	27	28	28	29	1.064
436	gene-ASLP1	**ASLP1**	argininosuccinate lyase p	pseudogene	2	1	3	2	0.851
438	NM_001171038,NM	**ASMT**	acetylserotonin O-methy	protein_coding	0	0	1	1	1.000
439	NM_001371488,NM	**GET3**	guided entry of tail-anch	protein_coding	125	145	126	146	1.162
440	NM_001178075,NM	**ASNS**	asparagine synthetase (g	protein_coding	2	0	3	1	0.492
443	NM_000049,NM_00	**ASPA**	aspartoacylase	protein_coding	0	0	1	1	1.000
444	NM_001164750,NM	**ASPH**	aspartate beta-hydroxyla	protein_coding	0	0	1	1	0.966
445	NM_000050,NM_05	**ASS1**	argininosuccinate syntha	protein_coding	0	0	1	1	1.000
447	gene-ASS1P2	**ASS1P2**	argininosuccinate synthe	pseudogene	0	0	1	1	0.911
449	gene-ASS1P4	**ASS1P4**	argininosuccinate synthe	pseudogene	0	0	1	1	1.000
450	gene-ASS1P5	**ASS1P5**	argininosuccinate synthe	pseudogene	0	0	1	1	1.000
451	gene-ASS1P6	**ASS1P6**	argininosuccinate synthe	pseudogene	0	0	1	1	1.000
453	gene-ASS1P8	**ASS1P8**	argininosuccinate synthe	pseudogene	0	0	1	1	1.000
454	gene-ASS1P9	**ASS1P9**	argininosuccinate synthe	pseudogene	0	0	1	1	1.000
455	gene-ASS1P10	**ASS1P10**	argininosuccinate synthe	pseudogene	0	0	1	1	1.000
459	gene-ASS1P14	**ASS1P14**	argininosuccinate synthe	pseudogene	0	0	1	1	1.000
460	NM_001286164,NM	**ASTN1**	astrotactin 1	protein_coding	0	0	1	1	1.000
462	NM_000488,NM_00	**SERPINC1**	serpin family C member	protein_coding	0	0	1	1	0.913
463	NM_001164766,NM	**ZFHX3**	zinc finger homeobox 3	protein_coding	0	0	1	1	1.071
466	NM_005171,XM_01	**ATF1**	activating transcription fa	protein_coding	31	25	32	26	0.809
467	NM_001030287,NM	**ATF3**	activating transcription fa	protein_coding	6	5	7	6	0.896
468	NM_001675,NM_18	**ATF4**	activating transcription fa	protein_coding	446	402	447	403	0.903
469	gene-ATF4P1	**ATF4P1**	activating transcription fa	pseudogene	0	0	1	1	1.070
471	NM_004044,XM_01	**ATIC**	5-aminoimidazole-4-carb	protein_coding	493	467	494	468	0.948
472	NM_000051,NM_00	**ATM**	ATM serine/threonine kin	protein_coding	20	15	21	16	0.764
473	NM_001042681,NM	**RERE**	arginine-glutamic acid di	protein_coding	5	5	6	6	0.972
474	NM_005172	**ATOH1**	atonal bHLH transcriptio	protein_coding	0	0	1	1	1.000
475	NM_004045	**ATOX1**	antioxidant 1 copper cha	protein_coding	98	110	99	111	1.129
476	NM_000701,NM_00	**ATP1A1**	ATPase Na+/K+ transpor	protein_coding	421	395	422	396	0.938
477	NM_000702	**ATP1A2**	ATPase Na+/K+ transpor	protein_coding	0	0	1	1	1.000
478	NM_001256213,NM	**ATP1A3**	ATPase Na+/K+ transpor	protein_coding	44	43	45	44	0.975
479	NM_001185085,NM	**ATP12A**	ATPase H+/K+ transporti	protein_coding	0	0	1	1	1.000
480	NM_001001734,NM	**ATP1A4**	ATPase Na+/K+ transpor	protein_coding	0	0	1	1	1.000
481	NM_001677	**ATP1B1**	ATPase Na+/K+ transpor	protein_coding	0	0	1	1	1.000

482	NM_001303263,NM	**ATP1B2**	ATPase Na+/K+ transpor	protein_coding	0	0	1	1	1.000
483	NM_001679	**ATP1B3**	ATPase Na+/K+ transpor	protein_coding	245	246	246	247	1.005
484	gene-ATP1B3P1	**ATP1B3P1**	ATPase Na+/K+ transpor	pseudogene	0	0	1	1	0.947
485	gene-ATP1B1P1	**ATP1B1P1**	ATPase Na+/K+ transpor	pseudogene	0	0	1	1	0.951
486	NM_001680,NM_02	**FXYD2**	FXYD domain containing	protein_coding	6	5	7	6	0.793
487	NM_001286075,NM	**ATP2A1**	ATPase sarcoplasmic/en	protein_coding	1	1	2	2	1.213
488	NM_001681,NM_17	**ATP2A2**	ATPase sarcoplasmic/en	protein_coding	131	125	132	126	0.953
489	NM_005173,NM_17	**ATP2A3**	ATPase sarcoplasmic/en	protein_coding	67	65	68	66	0.967
490	NM_001001323,NM	**ATP2B1**	ATPase plasma membra	protein_coding	23	20	24	21	0.880
491	NM_001001331,NM	**ATP2B2**	ATPase plasma membra	protein_coding	0	0	1	1	1.000
492	NM_001001344,NM	**ATP2B3**	ATPase plasma membra	protein_coding	0	0	1	1	1.000
493	NM_001001396,NM	**ATP2B4**	ATPase plasma membra	protein_coding	33	28	34	29	0.869
495	NM_000704	**ATP4A**	ATPase H+/K+ transporti	protein_coding	0	0	1	1	1.000
496	NM_000705	**ATP4B**	ATPase H+/K+ transporti	protein_coding	0	0	1	1	1.000
498	NM_001001935,NM	**ATP5F1A**	ATP synthase F1 subuni	protein_coding	695	652	696	653	0.938
501	NM_001182,NM_00	**ALDH7A1**	aldehyde dehydrogenase	protein_coding	0	0	1	1	1.000
504	gene-ATP5F1AP2	**ATP5F1AP2**	ATP synthase F1 subuni	pseudogene	0	0	1	1	1.000
506	NM_001686	**ATP5F1B**	ATP synthase F1 subuni	protein_coding	1,341	1,320	1,342	1,321	0.984
507	gene-ATP5F1BP1	**ATP5F1BP1**	ATP synthase F1 subuni	pseudogene	0	0	1	1	1.000
509	NM_001001973,NM	**ATP5F1C**	ATP synthase F1 subuni	protein_coding	108	112	109	113	1.039
513	NM_001001975,NM	**ATP5F1D**	ATP synthase F1 subuni	protein_coding	126	141	127	142	1.122
514	NM_006886	**ATP5F1E**	ATP synthase F1 subuni	protein_coding	201	206	202	207	1.027
515	NM_001688	**ATP5PB**	ATP synthase peripheral	protein_coding	268	273	269	274	1.016
516	NM_001002027,NM	**ATP5MC1**	ATP synthase membrane	protein_coding	532	541	533	542	1.016
517	NM_001002031,NM	**ATP5MC2**	ATP synthase membrane	protein_coding	149	166	150	167	1.108
518	NM_001002258,NM	**ATP5MC3**	ATP synthase membrane	protein_coding	328	351	329	352	1.069
520	gene-ATP5MC2P1	**ATP5MC2P1**	ATP synthase membrane	pseudogene	0	0	1	1	1.000
521	NM_007100,NR_03	**ATP5ME**	ATP synthase membrane	protein_coding	421	472	422	473	1.122
522	NM_001003696,NM	**ATP5PF**	ATP synthase peripheral	protein_coding	146	148	147	149	1.011
523	NM_001690	**ATP6V1A**	ATPase H+ transporting	protein_coding	64	56	65	57	0.891
525	NM_001692,XM_01	**ATP6V1B1**	ATPase H+ transporting	protein_coding	0	0	1	1	0.957
526	NM_001693,XR_00	**ATP6V1B2**	ATPase H+ transporting	protein_coding	57	56	58	57	0.982
527	NM_001198569,NM	**ATP6V0C**	ATPase H+ transporting	protein_coding	177	200	178	201	1.126
528	NM_001695	**ATP6V1C1**	ATPase H+ transporting	protein_coding	39	34	40	35	0.878
529	NM_001039366,NM	**ATP6V1E1**	ATPase H+ transporting	protein_coding	124	130	125	131	1.045
533	NM_001039457,NM	**ATP6V0B**	ATPase H+ transporting	protein_coding	203	220	204	221	1.086
534	NM_001204078,NM	**ATP6V1G2**	ATPase H+ transporting	protein_coding	4	3	5	4	0.804
535	NM_001130020,NM	**ATP6V0A1**	ATPase H+ transporting	protein_coding	0	0	1	1	1.000
537	NM_001183,XM_01	**ATP6AP1**	ATPase H+ transporting	protein_coding	39	44	40	45	1.125
538	NM_000052,NM_00	**ATP7A**	ATPase copper transport	protein_coding	7	5	8	6	0.706
539	NM_001697	**ATP5PO**	ATP synthase peripheral	protein_coding	538	541	539	542	1.005
540	NM_000053,NM_00	**ATP7B**	ATPase copper transport	protein_coding	0	0	1	1	0.913
541	gene-ALDH7A1P1	**ALDH7A1P1**	aldehyde dehydrogenase	pseudogene	0	0	1	1	1.000
543	gene-ALDH7A1P3	**ALDH7A1P3**	aldehyde dehydrogenase	pseudogene	1	0	2	1	0.538
544	gene-ALDH7A1P4	**ALDH7A1P4**	aldehyde dehydrogenase	pseudogene	0	0	1	1	1.000
545	NM_001184,NM_00	**ATR**	ATR serine/threonine kin	protein_coding	21	20	22	21	0.952
546	NM_000489,NM_13	**ATRX**	ATRX chromatin remode	protein_coding	19	14	20	15	0.745
547	NM_001244008,NM	**KIF1A**	kinesin family member 1	protein_coding	0	0	1	1	0.874
549	NM_001306190,NM	**AUH**	AU RNA binding methylg	protein_coding	20	18	21	19	0.911
550	NM_181575,NR_12	**AUP1**	AUP1 lipid droplet regula	protein_coding	205	197	206	198	0.964
551	NM_000490,XM_01	**AVP**	arginine vasopressin	protein_coding	0	0	1	1	1.000
552	NM_000706	**AVPR1A**	arginine vasopressin rec	protein_coding	0	0	1	1	1.000
553	NM_000707	**AVPR1B**	arginine vasopressin rec	protein_coding	0	0	1	1	1.000
554	NM_000054,NM_00	**AVPR2**	arginine vasopressin rec	protein_coding	0	0	1	1	1.000
558	NM_001278599,NM	**AXL**	AXL receptor tyrosine kin	protein_coding	0	0	1	1	1.000
563	NM_001185	**AZGP1**	alpha-2-glycoprotein 1, z	protein_coding	0	0	1	1	1.085
566	NM_001700	**AZU1**	azurocidin 1	protein_coding	0	0	1	1	1.000
567	NM_004048,XM_00	**B2M**	beta-2-microglobulin	protein_coding	7,900	7,791	7,901	7,792	0.986
570	NM_001127610,NM	**BAAT**	bile acid-CoA:amino acid	protein_coding	0	0	1	1	1.104
571	NM_001186,NM_20	**BACH1**	BTB domain and CNC ho	protein_coding	11	8	12	9	0.801
572	NM_004322,NM_03	**BAD**	BCL2 associated agonist	protein_coding	49	52	50	53	1.047
573	NM_001172415,NM	**BAG1**	BAG cochaperone 1	protein_coding	65	69	66	70	1.058
575	NM_001702,XM_01	**ADGRB1**	adhesion G protein-coup	protein_coding	0	0	1	1	1.000
576	NM_001294335,NM	**ADGRB2**	adhesion G protein-coup	protein_coding	3	3	4	4	0.921
577	NM_001704	**ADGRB3**	adhesion G protein-coup	protein_coding	0	0	1	1	1.000
578	NM_001188,XM_01	**BAK1**	BCL2 antagonist/killer 1	protein_coding	117	129	118	130	1.103
579	NM_001189	**NKX3-2**	NK3 homeobox 2	protein_coding	0	0	1	1	1.000
580	NM_000465,NM_00	**BARD1**	BRCA1 associated RING	protein_coding	51	42	52	43	0.830
581	NM_001291428,NM	**BAX**	BCL2 associated X, apop	protein_coding	110	125	111	126	1.136
582	NM_024649	**BBS1**	Bardet-Biedl syndrome 1	protein_coding	5	5	6	6	0.959
583	NM_001377456,NM	**BBS2**	Bardet-Biedl syndrome 2	protein_coding	4	4	5	5	0.908
585	NM_001252678,NM	**BBS4**	Bardet-Biedl syndrome 4	protein_coding	16	17	17	18	1.061
586	NM_001178091,NM	**BCAT1**	branched chain amino ac	protein_coding	22	16	23	17	0.765
587	NM_001164773,NM	**BCAT2**	branched chain amino ac	protein_coding	49	49	50	50	0.993
590	NM_000055,NR_13	**BCHE**	butyrylcholinesterase	protein_coding	0	0	1	1	1.000
593	NM_000709,NM_00	**BCKDHA**	branched chain keto acic	protein_coding	57	63	58	64	1.115
594	NM_000056,NM_00	**BCKDHB**	branched chain keto acic	protein_coding	16	17	17	18	1.032
595	NM_053056	**CCND1**	cyclin D1	protein_coding	0	0	1	1	1.000
596	NM_000633,NM_00	**BCL2**	BCL2 apoptosis regulato	protein_coding	61	53	62	54	0.875
597	NM_001114735,NM	**BCL2A1**	BCL2 related protein A1	protein_coding	3	2	4	3	0.876
598	NM_001191,NM_00	**BCL2L1**	BCL2 like 1	protein_coding	141	139	142	140	0.987
599	NM_001199839,NM	**BCL2L2**	BCL2 like 2	protein_coding	14	15	15	16	1.014
600	gene-BAK1P1	**BAK1P1**	BCL2 antagonist/killer 1	pseudogene	8	10	9	11	1.184
601	gene-BAK1P2	**BAK1P2**	BCL2 antagonist/killer 1	pseudogene	0	0	1	1	1.000
602	NM_005178,XM_01	**BCL3**	BCL3 transcription coact	protein_coding	65	66	66	67	1.016
604	NM_001130845,NM	**BCL6**	BCL6 transcription repre	protein_coding	1	1	2	2	1.326
605	NM_001024808,NM	**BCL7A**	BAF chromatin remodelir	protein_coding	0	0	1	1	1.000
606	NR_027992	**NBEAP1**	neurobeachin pseudoger	transcribed_pseu	0	0	1	1	1.000
607	NM_004326,XM_00	**BCL9**	BCL9 transcription coact	protein_coding	16	16	17	17	0.977
608	NM_001192	**TNFRSF17**	TNF receptor superfamily	protein_coding	0	0	1	1	1.000
610	NM_001194	**HCN2**	hyperpolarization activate	protein_coding	0	0	1	1	0.975
611	NM_001708	**OPN1SW**	opsin 1, short wave sens	protein_coding	0	0	1	1	1.000
613	NM_004327,NM_02	**BCR**	BCR activator of RhoGEI	protein_coding	0	0	1	1	0.962
617	NM_001079866,NM	**BCS1L**	BCS1 homolog, ubiquinc	protein_coding	73	74	74	75	1.012
618	NR_001568	**BCYRN1**	brain cytoplasmic RNA 1	lncRNA	0	0	1	1	1.135
619	gene-BCYRN1P1	**BCYRN1P1**	brain cytoplasmic RNA 1	pseudogene	0	0	1	1	1.194
622	NM_004051,NM_20	**BDH1**	3-hydroxybutyrate dehyd	protein_coding	28	28	29	29	0.995
623	NM_000710	**BDKRB1**	bradykinin receptor B1	protein_coding	0	0	1	1	1.000
624	NM_000623,NM_00	**BDKRB2**	bradykinin receptor B2	protein_coding	0	0	1	1	1.000
627	NM_001143805,NM	**BDNF**	brain derived neurotroph	protein_coding	0	0	1	1	1.000
629	NM_001710	**CFB**	complement factor B	protein_coding	0	0	1	1	1.027
631	NM_001161705,NM	**BFSP1**	beaded filament structur	protein_coding	0	0	1	1	0.944
632	NM_199173	**BGLAP**	bone gamma-carboxyglu	protein_coding	0	0	1	1	1.000
633	NM_001711,XM_01	**BGN**	biglycan	protein_coding	0	0	1	1	1.000
634	NM_001024912,NM	**CEACAM1**	CEA cell adhesion molec	protein_coding	207	170	208	171	0.822
635	NM_001713	**BHMT**	betaine--homocysteine S	protein_coding	0	0	1	1	1.041
636	NM_001003398,NM	**BICD1**	BICD cargo adaptor 1	protein_coding	9	7	10	8	0.811
637	NM_001196,NM_00	**BID**	BH3 interacting domain c	protein_coding	82	81	83	82	0.995
638	NM_001197	**BIK**	BCL2 interacting killer	protein_coding	0	0	1	1	1.000
639	NM_001198,NM_18	**PRDM1**	PR/SET domain 1	protein_coding	39	40	40	41	1.011
640	NM_001330465,NM	**BLK**	BLK proto-oncogene, Src	protein_coding	0	0	1	1	1.000
641	NM_000057,NM_00	**BLM**	BLM RecQ like helicase	protein_coding	43	36	44	37	0.840
642	NM_000386	**BLMH**	bleomycin hydrolase	protein_coding	191	177	192	178	0.925
643	NM_001716,NM_03	**CXCR5**	C-X-C motif chemokine r	protein_coding	0	0	1	1	1.000
644	NM_000712,NM_00	**BLVRA**	biliverdin reductase A	protein_coding	0	0	1	1	1.000
645	NM_000713	**BLVRB**	biliverdin reductase B	protein_coding	0	1	1	2	1.158
646	NM_001301206,NM	**BNC1**	basonuclin 1	protein_coding	0	0	1	1	1.000
648	NM_005180	**BMI1**	BMI1 proto-oncogene, pc	protein_coding	22	17	23	18	0.787
649	NM_001199,NM_00	**BMP1**	bone morphogenetic pro	protein_coding	3	2	4	3	0.786
650	NM_001200	**BMP2**	bone morphogenetic pro	protein_coding	0	0	1	1	1.000
651	NM_001201,XM_00	**BMP3**	bone morphogenetic pro	protein_coding	0	0	1	1	1.000
652	NM_001202,NM_00	**BMP4**	bone morphogenetic pro	protein_coding	0	0	1	1	1.000
653	NM_001329754,NM	**BMP5**	bone morphogenetic pro	protein_coding	0	0	1	1	1.000
654	NM_001718	**BMP6**	bone morphogenetic pro	protein_coding	0	0	1	1	1.000
655	NM_001719	**BMP7**	bone morphogenetic pro	protein_coding	0	0	1	1	1.000
656	NM_001720,XM_00	**BMP8B**	bone morphogenetic pro	protein_coding	1	1	2	2	1.193

657	NM_004329,XM_01	**BMPR1A**	bone morphogenetic pro	protein_coding	5	5	6	6	1.005
658	NM_001203,NM_00	**BMPR1B**	bone morphogenetic pro	protein_coding	0	0	1	1	1.000
659	NM_001204,XM_01	**BMPR2**	bone morphogenetic pro	protein_coding	2	1	3	2	0.819
660	NM_001320866,NM	**BMX**	BMX non-receptor tyrosi	protein_coding	0	0	1	1	1.000
661	NM_001722,XR_94	**POLR3D**	RNA polymerase III subu	protein_coding	19	19	20	20	0.986
662	NM_001205,NM_01	**BNIP1**	BCL2 interacting protein	protein_coding	30	29	31	30	0.952
663	NM_001320674,NM	**BNIP2**	BCL2 interacting protein	protein_coding	50	46	51	47	0.906
664	NM_004052	**BNIP3**	BCL2 interacting protein	protein_coding	0	0	1	1	1.000
665	NM_001330491,NM	**BNIP3L**	BCL2 interacting protein	protein_coding	5	5	6	6	0.999
666	NM_032515,XM_01	**BOK**	BCL2 family apoptosis re	protein_coding	0	0	1	1	1.000
667	NM_001144769,NM	**DST**	dystonin	protein_coding	0	0	1	1	0.966
668	NM_023067	**FOXL2**	forkhead box L2	protein_coding	0	0	1	1	1.000
669	NM_001293085,NM	**BPGM**	bisphosphoglycerate mu	protein_coding	37	35	38	36	0.940
670	NM_001302777,NM	**BPHL**	biphenyl hydrolase like	protein_coding	2	2	3	3	0.929
671	NM_001725,XM_02	**BPI**	bactericidal permeability	protein_coding	0	0	1	1	1.000
672	NM_007294,NM_00	**BRCA1**	BRCA1 DNA repair asso	protein_coding	50	45	51	46	0.903
673	NM_001354609,NM	**BRAF**	B-Raf proto-oncogene, s	protein_coding	16	13	17	14	0.834
675	NM_000059	**BRCA2**	BRCA2 DNA repair asso	protein_coding	16	12	17	13	0.756
676	NM_001242805,NM	**BRDT**	bromodomain testis asso	protein_coding	0	0	1	1	1.000
677	NM_001244698,NM	**ZFP36L1**	ZFP36 ring finger protei	protein_coding	32	30	33	31	0.934
678	NM_006887	**ZFP36L2**	ZFP36 ring finger protei	protein_coding	67	63	68	64	0.938
680	NM_001727	**BRS3**	bombesin receptor subty	protein_coding	0	0	1	1	1.000
682	NM_001322243,NM	**BSG**	basigin (Ok blood group)	protein_coding	335	350	336	351	1.044
683	NM_004334,XM_00	**BST1**	bone marrow stromal cel	protein_coding	0	0	1	1	1.091
684	NM_004335	**BST2**	bone marrow stromal cel	protein_coding	549	564	550	565	1.027
685	NM_001316963,NM	**BTC**	betacellulin	protein_coding	0	0	1	1	1.000
686	NM_001281723,NM	**BTD**	biotinidase	protein_coding	1	1	2	2	1.176
687	NM_001206	**KLF9**	Kruppel like factor 9	protein_coding	0	0	1	1	1.000
688	NM_001286818,NM	**KLF5**	Kruppel like factor 5	protein_coding	0	0	1	1	1.227
689	NM_001037637,NM	**BTF3**	basic transcription factor	protein_coding	1,183	1,192	1,184	1,193	1.008
690	NR_026983	**BTF3P11**	basic transcription factor	transcribed_pseu	0	0	1	1	1.000
693	gene-BTF3P1	**BTF3P1**	BTF3 pseudogene 1	pseudogene	0	0	1	1	1.000
694	NM_001731	**BTG1**	BTG anti-proliferation fac	protein_coding	8	7	9	8	0.881
695	NM_000061,NM_00	**BTK**	Bruton tyrosine kinase	protein_coding	0	0	1	1	1.054
696	NM_001732	**BTN1A1**	butyrophilin subfamily 1	protein_coding	0	0	1	1	1.000
699	NM_001278616,NM	**BUB1**	BUB1 mitotic checkpoint	protein_coding	182	139	183	140	0.764
701	NM_001211	**BUB1B**	BUB1 mitotic checkpoint	protein_coding	162	137	163	138	0.847
705	NM_004053	**BYSL**	bystin like	protein_coding	138	145	139	146	1.049
706	NM_000714,NM_00	**TSPO**	translocator protein	protein_coding	0	0	1	1	1.000
708	NM_001212	**C1QBP**	complement C1q binding	protein_coding	318	331	319	332	1.043
710	NM_000062,NM_00	**SERPING1**	serpin family G member	protein_coding	0	0	1	1	1.000
711	NR_024615	**ERC2-IT1**	ERC2 intronic transcript	lncRNA	0	0	1	1	1.000
712	NM_001347465,NM	**C1QA**	complement C1q A chai	protein_coding	0	0	1	1	1.000
713	NM_000491,NM_00	**C1QB**	complement C1q B chai	protein_coding	0	0	1	1	1.000
714	NM_001114101,NM	**C1QC**	complement C1q C chai	protein_coding	0	0	1	1	1.000
715	NM_001354346,NM	**C1R**	complement C1r	protein_coding	2	3	3	4	1.259
716	NM_001346850,NM	**C1S**	complement C1s	protein_coding	0	0	1	1	0.955
717	NM_000063,NM_00	**C2**	complement C2	protein_coding	1	0	2	1	0.685
718	NM_000064	**C3**	complement C3	protein_coding	1	0	2	1	0.899
719	NM_001326475,NM	**C3AR1**	complement C3a recept	protein_coding	4	3	5	4	0.851
720	NM_001252204,NM	**C4A**	complement C4A (Rodg	protein_coding	0	0	1	1	1.103
721	NM_001002029	**C4B**	complement C4B (Chido	protein_coding	0	0	1	1	1.106
722	NM_000715,XM_00	**C4BPA**	complement component	protein_coding	0	0	1	1	1.000
725	NM_000716,NM_00	**C4BPB**	complement component	protein_coding	0	0	1	1	1.000
726	NM_004055,XM_01	**CAPN5**	calpain 5	protein_coding	0	0	1	1	1.000
727	NM_001317163,NM	**C5**	complement C5	protein_coding	1	1	2	2	0.933
728	NM_001736,XM_00	**C5AR1**	complement C5a recept	protein_coding	0	0	1	1	0.960
729	NM_000065,NM_00	**C6**	complement C6	protein_coding	0	0	1	1	1.000
730	NM_000587	**C7**	complement C7	protein_coding	0	0	1	1	1.000
731	NM_000562,XM_01	**C8A**	complement C8 alpha ch	protein_coding	0	0	1	1	0.934
732	NM_000066,NM_00	**C8B**	complement C8 beta cha	protein_coding	2	2	3	3	0.903
733	NM_000606,XR_24	**C8G**	complement C8 gamma	protein_coding	0	0	1	1	1.066
734	NM_001126111,NM	**OSGIN2**	oxidative stress induced	protein_coding	38	34	39	35	0.906
735	NM_001737	**C9**	complement C9	protein_coding	0	0	1	1	1.000
738	NM_013265,NR_07	**VPS51**	VPS51 subunit of GARP	protein_coding	59	66	60	67	1.125
740	NM_004927,NR_03	**MRPL49**	mitochondrial ribosomal	protein_coding	138	137	139	138	0.993
741	NM_014205	**ZNHIT2**	zinc finger HIT-type cont	protein_coding	9	10	10	11	1.152
744	NM_001145399,NM	**MPPED2**	metallophosphoesterase	protein_coding	0	0	1	1	1.000
745	NM_001127392,NM	**MYRF**	myelin regulatory factor	protein_coding	3	4	4	5	1.304
746	NM_014206	**TMEM258**	transmembrane protein 2	protein_coding	181	177	182	178	0.978
747	NM_006133,XM_01	**DAGLA**	diacylglycerol lipase alpl	protein_coding	0	0	1	1	1.000
750	NR_122031	**GAS8-AS1**	GAS8 antisense RNA 1	lncRNA	0	0	1	1	1.000
752	NM_005892,XM_00	**FMNL1**	formin like 1	protein_coding	118	115	119	116	0.974
753	NM_001003674,NM	**LDLRAD4**	low density lipoprotein re	protein_coding	6	3	7	4	0.580
754	NM_001286822,NM	**PTTG1IP**	PTTG1 interacting protei	protein_coding	62	61	63	62	0.982
755	NM_001271440,NM	**CFAP410**	cilia and flagella associa	protein_coding	8	9	9	10	1.109
757	NM_006134,NR_04	**TMEM50B**	transmembrane protein 5	protein_coding	31	31	32	32	1.014
758	NM_001044370,NM	**MPPED1**	metallophosphoesterase	protein_coding	0	0	1	1	1.000
759	NM_001128829,NM	**CA1**	carbonic anhydrase 1	protein_coding	0	0	1	1	1.000
760	NM_000067,NM_00	**CA2**	carbonic anhydrase 2	protein_coding	0	0	1	1	1.000
761	NM_005181	**CA3**	carbonic anhydrase 3	protein_coding	0	0	1	1	0.947
762	NM_000717,NR_13	**CA4**	carbonic anhydrase 4	protein_coding	0	0	1	1	0.998
763	NM_001367225,NM	**CA5A**	carbonic anhydrase 5A	protein_coding	0	0	1	1	0.927
764	gene-CA5AP1	**CA5AP1**	carbonic anhydrase 5A p	pseudogene	0	0	1	1	1.000
765	NM_001215,NM_00	**CA6**	carbonic anhydrase 6	protein_coding	0	0	1	1	1.123
766	NM_001014435,NM	**CA7**	carbonic anhydrase 7	protein_coding	0	0	1	1	1.000
767	NM_001321837,NM	**CA8**	carbonic anhydrase 8	protein_coding	0	0	1	1	1.000
768	NM_001216,XR_00	**CA9**	carbonic anhydrase 9	protein_coding	0	0	1	1	1.000
770	NM_001217,NR_13	**CA11**	carbonic anhydrase 11	protein_coding	3	4	4	5	1.309
771	NM_001218,NM_00	**CA12**	carbonic anhydrase 12	protein_coding	0	0	1	1	1.000
773	NM_000068,NM_00	**CACNA1A**	calcium voltage-gated ch	protein_coding	0	0	1	1	0.986
774	NM_000718,NM_00	**CACNA1B**	calcium voltage-gated ch	protein_coding	0	0	1	1	1.000
775	NM_000719,NM_00	**CACNA1C**	calcium voltage-gated ch	protein_coding	0	0	1	1	1.000
776	NM_000720,NM_00	**CACNA1D**	calcium voltage-gated ch	protein_coding	0	0	1	1	1.000
777	NM_000721,NM_00	**CACNA1E**	calcium voltage-gated ch	protein_coding	0	0	1	1	1.000
778	NM_001256789,NM	**CACNA1F**	calcium voltage-gated ch	protein_coding	0	0	1	1	1.000
779	NM_000069,XM_00	**CACNA1S**	calcium voltage-gated ch	protein_coding	0	0	1	1	1.048
780	NM_001202521,NM	**DDR1**	discoidin domain recept	protein_coding	0	0	1	1	1.000
781	NM_000722,NM_00	**CACNA2D1**	calcium voltage-gated ch	protein_coding	0	0	1	1	1.000
782	NM_000723,NM_19	**CACNB1**	calcium voltage-gated ch	protein_coding	13	12	14	13	0.939
783	NM_000724,NM_00	**CACNB2**	calcium voltage-gated ch	protein_coding	0	0	1	1	1.000
784	NM_000725,NM_00	**CACNB3**	calcium voltage-gated ch	protein_coding	0	0	1	1	1.000
785	NM_000726,NM_00	**CACNB4**	calcium voltage-gated ch	protein_coding	0	0	1	1	1.026
786	NM_000727	**CACNG1**	calcium voltage-gated ch	protein_coding	0	0	1	1	1.000
788	NM_000387	**SLC25A20**	solute carrier family 25 m	protein_coding	73	66	74	67	0.909
789	gene-SLC25A20P1	**SLC25A20P1**	solute carrier family 25 m	pseudogene	0	0	1	1	1.000
790	NM_001306079,NM	**CAD**	carbamoyl-phosphate sy	protein_coding	53	55	54	56	1.041
793	NM_001366795,NM	**CALB1**	calbindin 1	protein_coding	0	0	1	1	1.000
794	NM_001740,NM_00	**CALB2**	calbindin 2	protein_coding	0	0	1	1	0.999
795	NM_004057,XM_01	**S100G**	S100 calcium binding pr	protein_coding	0	0	1	1	1.000
796	NM_001033952,NM	**CALCA**	calcitonin related polype	protein_coding	0	0	1	1	1.000
797	NM_000728	**CALCB**	calcitonin related polype	protein_coding	0	0	1	1	1.000
799	NM_001164737,NM	**CALCR**	calcitonin receptor	protein_coding	0	0	1	1	1.000
800	NM_004342,NM_03	**CALD1**	caldesmon 1	protein_coding	0	0	1	1	0.952
801	NM_001363669,NM	**CALM1**	calmodulin 1	protein_coding	261	268	262	269	1.027
802	gene-CALM1P1	**CALM1P1**	calmodulin 1 pseudogen	pseudogene	0	0	1	1	1.000
804	gene-CALM1P2	**CALM1P2**	calmodulin 1 pseudogen	pseudogene	0	0	1	1	1.000
805	NM_001305624,NM	**CALM2**	calmodulin 2	protein_coding	815	783	816	784	0.960
806	gene-CALM2P2	**CALM2P2**	calmodulin 2 pseudogen	pseudogene	0	0	1	1	0.927
807	gene-CALM2P3	**CALM2P3**	calmodulin 2 pseudogen	pseudogene	0	0	1	1	0.902
808	NM_001329921,NM	**CALM3**	calmodulin 3	protein_coding	560	589	561	590	1.051
810	NM_005185	**CALML3**	calmodulin like 3	protein_coding	0	0	1	1	1.000
811	NM_004343	**CALR**	calreticulin	protein_coding	1,468	1,621	1,469	1,622	1.104
813	NM_001130674,NM	**CALU**	calumenin	protein_coding	101	88	102	89	0.872
814	NM_001323374,NM	**CAMK4**	calcium/calmodulin depe	protein_coding	0	0	1	1	0.997
815	NM_001363989,NM	**CAMK2A**	calcium/calmodulin depe	protein_coding	0	0	1	1	1.000

816	NM_001220,NM_00	**CAMK2B**	calcium/calmodulin depe	protein_coding	0	0	1	1	1.000
817	NM_001221,NM_00	**CAMK2D**	calcium/calmodulin depe	protein_coding	6	6	7	7	0.972
818	NM_001204492,NM	**CAMK2G**	calcium/calmodulin depe	protein_coding	17	16	18	17	0.922
819	NM_001745	**CAMLG**	calcium modulating ligar	protein_coding	42	41	43	42	0.958
820	NM_004345	**CAMP**	cathelicidin antimicrobial	protein_coding	0	0	1	1	1.000
821	NM_001024649,NM	**CANX**	calnexin	protein_coding	518	478	519	479	0.923
822	NM_001256139,NM	**CAPG**	capping actin protein, ge	protein_coding	2	2	3	3	0.934
823	NM_001198868,NM	**CAPN1**	calpain 1	protein_coding	44	42	45	43	0.959
824	NM_001146068,NM	**CAPN2**	calpain 2	protein_coding	184	156	185	157	0.848
825	NM_000070,NM_02	**CAPN3**	calpain 3	protein_coding	31	35	32	36	1.095
826	NM_001003962,NM	**CAPNS1**	calpain small subunit 1	protein_coding	224	242	225	243	1.081
827	NM_014289	**CAPN6**	calpain 6	protein_coding	0	0	1	1	1.000
828	NM_004058,NM_08	**CAPS**	calcyphosine	protein_coding	1	1	2	2	0.924
829	NM_006135,XM_01	**CAPZA1**	capping actin protein of r	protein_coding	418	394	419	395	0.941
830	NM_006136	**CAPZA2**	capping actin protein of r	protein_coding	73	64	74	65	0.880
831	NM_001042440,NM	**CAST**	calpastatin	protein_coding	64	61	65	62	0.946
832	NM_001206540,NM	**CAPZB**	capping actin protein of r	protein_coding	292	294	293	295	1.007
833	NM_001014437,NM	**CARS1**	cysteinyl-tRNA synthetas	protein_coding	60	51	61	52	0.855
834	NM_001223,NM_00	**CASP1**	caspase 1	protein_coding	74	68	75	69	0.925
835	NM_001224,NM_03	**CASP2**	caspase 2	protein_coding	53	52	54	53	0.975
836	NM_001354777,NM	**CASP3**	caspase 3	protein_coding	142	131	143	132	0.925
837	NM_001225,NM_03	**CASP4**	caspase 4	protein_coding	133	122	134	123	0.915
838	NM_001136109,NM	**CASP5**	caspase 5	protein_coding	0	0	1	1	1.000
839	NM_001226,NM_03	**CASP6**	caspase 6	protein_coding	1	0	2	1	0.728
840	NM_001227,NM_00	**CASP7**	caspase 7	protein_coding	66	62	67	63	0.932
841	NM_001080124,NM	**CASP8**	caspase 8	protein_coding	77	76	78	77	0.986
842	NM_001229,NM_00	**CASP9**	caspase 9	protein_coding	11	11	12	12	1.030
843	NM_001206524,NM	**CASP10**	caspase 10	protein_coding	17	15	18	16	0.884
844	NM_001231	**CASQ1**	calsequestrin 1	protein_coding	0	0	1	1	1.000
845	NM_001232	**CASQ2**	calsequestrin 2	protein_coding	0	0	1	1	1.000
846	NM_000388,NM_00	**CASR**	calcium sensing receptor	protein_coding	0	0	1	1	1.000
847	NM_001752	**CAT**	catalase	protein_coding	47	47	48	48	1.009
857	NM_001172895,NM	**CAV1**	caveolin 1	protein_coding	0	0	1	1	0.963
858	NM_001206747,NM	**CAV2**	caveolin 2	protein_coding	0	0	1	1	1.000
859	NM_001234,NM_03	**CAV3**	caveolin 3	protein_coding	0	0	1	1	1.000
860	NM_001015051,NM	**RUNX2**	RUNX family transcriptio	protein_coding	1	1	2	2	0.732
861	NM_001001890,NM	**RUNX1**	RUNX family transcriptio	protein_coding	45	42	46	43	0.921
862	NM_001198625,NM	**RUNX1T1**	RUNX1 partner transcrip	protein_coding	1	1	2	2	0.773
863	NM_005187,NM_17	**CBFA2T3**	CBFA2/RUNX1 partner tr	protein_coding	0	0	1	1	1.000
864	NM_001031680,NM	**RUNX3**	RUNX family transcriptio	protein_coding	115	110	116	111	0.962
865	NM_001368707,NM	**CBFB**	core-binding factor subu	protein_coding	218	200	219	201	0.916
866	NM_001756	**SERPINA6**	serpin family A member 6	protein_coding	0	0	1	1	1.000
867	NM_005188	**CBL**	Cbl proto-oncogene	protein_coding	42	33	43	34	0.781
868	NM_001321786,NM	**CBLB**	Cbl proto-oncogene B	protein_coding	18	19	19	20	1.040
869	NM_004352	**CBLN1**	cerebellin 1 precursor	protein_coding	0	0	1	1	1.000
871	NM_001207014,NM	**SERPINH1**	serpin family H member	protein_coding	1	0	2	1	0.982
873	NM_001286789,NM	**CBR1**	carbonyl reductase 1	protein_coding	47	52	48	53	1.097
874	NM_001236,XM_01	**CBR3**	carbonyl reductase 3	protein_coding	0	0	1	1	1.000
875	NM_000071,NM_00	**CBS**	cystathionine beta-synth	protein_coding	0	0	1	1	1.000
881	NM_005893	**CCIN**	calicin	protein_coding	0	0	1	1	1.000
883	NM_001122671,NM	**KYAT1**	kynurenine aminotransfe	protein_coding	12	11	13	12	0.931
885	NM_000729,NM_00	**CCK**	cholecystokinin	protein_coding	0	0	1	1	1.000
886	NM_000730	**CCKAR**	cholecystokinin A recept	protein_coding	0	0	1	1	1.000
887	NM_001318029,NM	**CCKBR**	cholecystokinin B recept	protein_coding	0	0	1	1	1.000
889	NM_001013406,NM	**KRIT1**	KRIT1 ankyrin repeat cor	protein_coding	26	24	27	25	0.942
890	NM_001237	**CCNA2**	cyclin A2	protein_coding	169	157	170	158	0.931
891	NM_001354844,NM	**CCNB1**	cyclin B1	protein_coding	239	227	240	228	0.950
892	NM_001013399,NM	**CCNC**	cyclin C	protein_coding	90	74	91	75	0.827
894	NM_001759	**CCND2**	cyclin D2	protein_coding	178	171	179	172	0.957
895	gene-CCND2P1	**CCND2P1**	cyclin D2 pseudogene 1	pseudogene	0	0	1	1	1.000
896	NM_001136017,NM	**CCND3**	cyclin D3	protein_coding	275	294	276	295	1.068
897	gene-CCND3P1	**CCND3P1**	cyclin D3 pseudogene 1	pseudogene	0	0	1	1	0.867
898	NM_001238,NM_00	**CCNE1**	cyclin E1	protein_coding	10	10	11	11	1.051
899	NM_001323538,NM	**CCNF**	cyclin F	protein_coding	58	59	59	60	1.012
900	NM_001364015,NM	**CCNG1**	cyclin G1	protein_coding	56	45	57	46	0.800
901	NM_004354,XM_01	**CCNG2**	cyclin G2	protein_coding	1	1	2	2	0.921
902	NM_001199189,NM	**CCNH**	cyclin H	protein_coding	62	55	63	56	0.889
904	NM_001240,NM_00	**CCNT1**	cyclin T1	protein_coding	32	26	33	27	0.827
905	NM_001241,NM_00	**CCNT2**	cyclin T2	protein_coding	22	17	23	18	0.791
908	NM_001009186,NM	**CCT6A**	chaperonin containing TC	protein_coding	317	294	318	295	0.928
909	NM_001320652,NM	**CD1A**	CD1a molecule	protein_coding	1	0	2	1	0.895
910	NM_001764,XM_01	**CD1B**	CD1b molecule	protein_coding	0	0	1	1	1.000
911	NM_001765,XM_00	**CD1C**	CD1c molecule	protein_coding	0	0	1	1	0.944
912	NM_001319145,NM	**CD1D**	CD1d molecule	protein_coding	0	0	1	1	1.000
913	NM_001042583,NM	**CD1E**	CD1e molecule	protein_coding	0	0	1	1	1.000
914	NM_001328609,NM	**CD2**	CD2 molecule	protein_coding	56	49	57	50	0.872
915	NM_000732,NM_00	**CD3D**	CD3d molecule	protein_coding	0	0	1	1	1.000
916	NM_000733	**CD3E**	CD3e molecule	protein_coding	673	698	674	699	1.037
917	NM_000073,XM_00	**CD3G**	CD3g molecule	protein_coding	0	0	1	1	1.000
919	NM_000734,NM_00	**CD247**	CD247 molecule	protein_coding	205	216	206	217	1.050
920	NM_000616,NM_00	**CD4**	CD4 molecule	protein_coding	0	0	1	1	0.944
921	NM_001346456,NM	**CD5**	CD5 molecule	protein_coding	0	0	1	1	1.000
922	NM_001347698,NM	**CD5L**	CD5 molecule like	protein_coding	1	1	2	2	1.059
923	NM_001254750,NM	**CD6**	CD6 molecule	protein_coding	9	7	10	8	0.824
924	NM_006137,XM_01	**CD7**	CD7 molecule	protein_coding	12	14	13	15	1.151
925	NM_001145873,NM	**CD8A**	CD8a molecule	protein_coding	0	0	1	1	1.000
926	NM_001178100,NM	**CD8B**	CD8b molecule	protein_coding	0	0	1	1	1.000
927	NM_001349727,NM	**CD8B2**	CD8b2 molecule	protein_coding	0	0	1	1	1.000
928	NM_001330312,NM	**CD9**	CD9 molecule	protein_coding	0	0	1	1	1.000
929	NM_000591,NM_00	**CD14**	CD14 molecule	protein_coding	0	0	1	1	1.191
930	NM_001178098,NM	**CD19**	CD19 molecule	protein_coding	0	0	1	1	1.000
931	NM_021950,NM_15	**MS4A1**	membrane spanning 4-d	protein_coding	0	0	1	1	1.000
932	NM_001031666,NM	**MS4A3**	membrane spanning 4-d	protein_coding	0	0	1	1	1.000
933	NM_001185099,NM	**CD22**	CD22 molecule	protein_coding	0	0	1	1	1.000
935	gene-CD24P1	**CD24P1**	CD24 molecule pseudog	pseudogene	0	0	1	1	1.000
936	gene-CD24P2	**CD24P2**	CD24 molecule pseudog	pseudogene	0	0	1	1	1.000
938	gene-CD24P4	**CD24P4**	CD24 molecule pseudog	pseudogene	0	0	1	1	1.000
939	NM_001242,XM_01	**CD27**	CD27 molecule	protein_coding	0	0	1	1	1.000
940	NM_001243077,NM	**CD28**	CD28 molecule	protein_coding	21	18	22	19	0.858
941	NM_005191	**CD80**	CD80 molecule	protein_coding	3	2	4	3	0.810
942	NM_001206924,NM	**CD86**	CD86 molecule	protein_coding	15	16	16	17	1.054
943	NM_001243,NM_00	**TNFRSF8**	TNF receptor superfamily	protein_coding	44	46	45	47	1.038
944	NM_001244,NM_00	**TNFSF8**	TNF superfamily membe	protein_coding	0	0	1	1	1.053
945	NM_001082618,NM	**CD33**	CD33 molecule	protein_coding	0	0	1	1	1.079
946	NM_001177547,NM	**SIGLEC6**	sialic acid binding Ig like	protein_coding	31	24	32	25	0.769
947	NM_001025109,NM	**CD34**	CD34 molecule	protein_coding	0	0	1	1	1.000
948	NM_000072,NM_00	**CD36**	CD36 molecule	protein_coding	0	0	1	1	1.000
949	NM_001082959,NM	**SCARB1**	scavenger receptor class	protein_coding	27	29	28	30	1.070
950	NM_001204255,NM	**SCARB2**	scavenger receptor class	protein_coding	15	13	16	14	0.904
951	NM_001040031,NM	**CD37**	CD37 molecule	protein_coding	56	63	57	64	1.117
952	NM_001775,NR_13	**CD38**	CD38 molecule	protein_coding	94	68	95	69	0.726
953	NM_001098175,NM	**ENTPD1**	ectonucleoside triphosph	protein_coding	0	0	1	1	0.977
954	NM_001246,NM_20	**ENTPD2**	ectonucleoside triphosph	protein_coding	0	0	1	1	0.956
955	NM_001114089,NM	**ENTPD6**	ectonucleoside triphosph	protein_coding	40	45	41	46	1.116
956	NM_001248,NM_00	**ENTPD3**	ectonucleoside triphosph	protein_coding	0	0	1	1	1.000
957	NM_001249,NM_00	**ENTPD5**	ectonucleoside triphosph	protein_coding	7	7	8	8	0.996
958	NM_001250,NM_00	**CD40**	CD40 molecule	protein_coding	0	0	1	1	1.000
959	NM_000074	**CD40LG**	CD40 ligand	protein_coding	0	0	1	1	1.000
960	NM_000610,NM_00	**CD44**	CD44 molecule (Indian b	protein_coding	90	84	91	85	0.940
961	NM_001382306,NM	**CD47**	CD47 molecule	protein_coding	61	55	62	56	0.909
962	NM_001256030,NM	**CD48**	CD48 molecule	protein_coding	659	604	660	605	0.916
963	NM_000560,NM_00	**CD53**	CD53 molecule	protein_coding	475	488	476	489	1.026
965	NM_001144822,NM	**CD58**	CD58 molecule	protein_coding	24	22	25	23	0.925
966	NM_000611,NM_00	**CD59**	CD59 molecule (CD59 bl	protein_coding	0	0	1	1	1.000
967	NM_001257389,NM	**CD63**	CD63 molecule	protein_coding	377	376	378	377	0.998
968	NM_001040059,NM	**CD68**	CD68 molecule	protein_coding	5	8	6	9	1.415

969	NM_001781	CD69	CD69 molecule	protein_coding	9	7	10	8	0.875
970	NM_001252,NM_00	CD70	CD70 molecule	protein_coding	0	1	1	2	1.202
971	NM_001782,XM_00	CD72	CD72 molecule	protein_coding	0	0	1	1	0.899
972	NM_001025158,NM	CD74	CD74 molecule	protein_coding	70	71	71	72	1.019
973	NM_001783,NM_02	CD79A	CD79a molecule	protein_coding	1	1	2	2	0.933
974	NM_000626,NM_00	CD79B	CD79b molecule	protein_coding	0	0	1	1	0.929
975	NM_001297649,NM	CD81	CD81 molecule	protein_coding	294	308	295	309	1.049
976	NM_001025160,NM	ADGRE5	adhesion G protein-coup	protein_coding	144	151	145	152	1.044
977	NM_001039490,NM	CD151	CD151 molecule (Raph b	protein_coding	37	42	38	43	1.129
978	NM_001785	CDA	cytidine deaminase	protein_coding	0	0	1	1	1.000
983	NM_001170406,NM	CDK1	cyclin dependent kinase	protein_coding	151	134	152	135	0.887
984	NM_001291345,NM	CDK11B	cyclin dependent kinase	protein_coding	77	85	78	86	1.091
987	NM_001199282,NM	LRBA	LPS responsive beige-lik	protein_coding	24	20	25	21	0.817
988	NM_001253	CDC5L	cell division cycle 5 like	protein_coding	40	38	41	39	0.971
989	NM_001011553,NM	SEPTIN7	septin 7	protein_coding	101	92	102	93	0.909
990	NM_001254,XM_01	CDC6	cell division cycle 6	protein_coding	57	51	58	52	0.900
991	NM_001255	CDC20	cell division cycle 20	protein_coding	343	338	344	339	0.986
993	NM_001789,NM_20	CDC25A	cell division cycle 25A	protein_coding	99	100	100	101	1.011
994	NM_001287516,NM	CDC25B	cell division cycle 25B	protein_coding	98	114	99	115	1.158
995	NM_001287582,NM	CDC25C	cell division cycle 25C	protein_coding	21	19	22	20	0.905
996	NM_001114091,NM	CDC27	cell division cycle 27	protein_coding	94	79	95	80	0.836
997	NM_004359,XM_00	CDC34	cell division cycle 34, ubi	protein_coding	84	85	85	86	1.016
998	NM_001039802,NM	CDC42	cell division cycle 42	protein_coding	97	92	98	93	0.956
999	NM_001317184,NM	CDH1	cadherin 1	protein_coding	0	0	1	1	1.000
1000	NM_001308176,NM	CDH2	cadherin 2	protein_coding	0	0	1	1	1.000
1001	NM_001317195,NM	CDH3	cadherin 3	protein_coding	0	0	1	1	1.000
1002	NM_001252338,NM	CDH4	cadherin 4	protein_coding	0	0	1	1	1.000
1003	NM_001795,XM_01	CDH5	cadherin 5	protein_coding	0	0	1	1	1.000
1004	NM_001362435,NM	CDH6	cadherin 6	protein_coding	0	0	1	1	1.000
1005	NM_001317214,NM	CDH7	cadherin 7	protein_coding	0	0	1	1	1.000
1006	NM_001796,XM_00	CDH8	cadherin 8	protein_coding	0	0	1	1	1.000
1007	NM_016279	CDH9	cadherin 9	protein_coding	0	0	1	1	1.000
1008	NM_001317222,NM	CDH10	cadherin 10	protein_coding	0	0	1	1	1.000
1009	NM_001308392,NM	CDH11	cadherin 11	protein_coding	0	0	1	1	1.000
1010	NM_001317227,NM	CDH12	cadherin 12	protein_coding	0	0	1	1	1.000
1011	gene-CDH12P2	CDH12P2	cadherin 12 pseudogene	pseudogene	0	0	1	1	1.000
1012	NM_001220488,NM	CDH13	cadherin 13	protein_coding	0	0	1	1	1.000
1013	NM_004933	CDH15	cadherin 15	protein_coding	0	0	1	1	1.000
1014	NM_001204744,NM	CDH16	cadherin 16	protein_coding	0	0	1	1	1.000
1015	NM_001144663,NM	CDH17	cadherin 17	protein_coding	74	69	75	70	0.931
1016	NM_001167667,NM	CDH18	cadherin 18	protein_coding	0	0	1	1	1.000
1017	NM_001290230,NM	CDK2	cyclin dependent kinase	protein_coding	126	108	127	109	0.858
1018	NM_001258	CDK3	cyclin dependent kinase	protein_coding	0	1	1	2	1.429
1019	NM_000075	CDK4	cyclin dependent kinase	protein_coding	234	250	235	251	1.067
1020	NM_001164410,NM	CDK5	cyclin dependent kinase	protein_coding	21	24	22	25	1.158
1021	NM_001145306,NM	CDK6	cyclin dependent kinase	protein_coding	94	80	95	81	0.851
1022	NM_001324069,NM	CDK7	cyclin dependent kinase	protein_coding	69	59	70	60	0.857
1024	NM_001260,NM_00	CDK8	cyclin dependent kinase	protein_coding	35	32	36	33	0.936
1025	NM_001261	CDK9	cyclin dependent kinase	protein_coding	61	53	62	54	0.868
1026	NM_000389,NM_00	CDKN1A	cyclin dependent kinase	protein_coding	0	0	1	1	1.041
1027	NM_004064	CDKN1B	cyclin dependent kinase	protein_coding	34	29	35	30	0.866
1028	NM_000076,NM_00	CDKN1C	cyclin dependent kinase	protein_coding	0	0	1	1	1.000
1029	NM_000077,NM_00	CDKN2A	cyclin dependent kinase	protein_coding	351	403	352	404	1.148
1030	NM_004936,NM_07	CDKN2B	cyclin dependent kinase	protein_coding	0	0	1	1	1.000
1031	NM_001262,NM_07	CDKN2C	cyclin dependent kinase	protein_coding	69	71	70	72	1.033
1032	NM_001800,NM_07	CDKN2D	cyclin dependent kinase	protein_coding	15	21	16	22	1.372
1033	NM_001130851,NM	CDKN3	cyclin dependent kinase	protein_coding	46	50	47	51	1.090
1036	NM_001323565,NM	CDO1	cysteine dioxygenase typ	protein_coding	0	0	1	1	1.000
1038	NM_004065	CDR1	cerebellar degeneration	protein_coding	0	0	1	1	1.000
1039	NM_001802,XM_02	CDR2	cerebellar degeneration	protein_coding	28	29	29	30	1.005
1040	NM_001263,XM_00	CDS1	CDP-diacylglycerol synth	protein_coding	0	0	1	1	1.000
1041	NM_001264	CDSN	corneodesmosin	protein_coding	0	0	1	1	1.000
1043	NM_001803	CD52	CD52 molecule	protein_coding	66	76	67	77	1.140
1044	NM_001804	CDX1	caudal type homeobox 1	protein_coding	0	0	1	1	1.000
1045	NM_001265,NM_00	CDX2	caudal type homeobox 2	protein_coding	0	0	1	1	1.000
1046	NM_005193	CDX4	caudal type homeobox 4	protein_coding	0	0	1	1	1.000
1047	NM_001130675,NM	CLGN	calmegin	protein_coding	0	0	1	1	1.000
1048	NM_001291484,NM	CEACAM5	CEA cell adhesion molec	protein_coding	0	0	1	1	1.000
1050	NM_001285829,NM	CEBPA	CCAAT enhancer binding	protein_coding	0	0	1	1	1.000
1051	NM_001285878,NM	CEBPB	CCAAT enhancer binding	protein_coding	75	64	76	65	0.850
1052	NM_005195	CEBPD	CCAAT enhancer binding	protein_coding	3	2	4	3	0.737
1053	NM_001805	CEBPE	CCAAT enhancer binding	protein_coding	0	0	1	1	1.000
1054	NM_001252296,NM	CEBPG	CCAAT enhancer binding	protein_coding	50	46	51	47	0.912
1056	NM_001807	CEL	carboxyl ester lipase	protein_coding	1	1	2	2	1.018
1057	NR_001275	CELP	carboxyl ester lipase pse	transcribed_pseu	1	0	2	1	0.924
1058	NM_001042426,NM	CENPA	centromere protein A	protein_coding	41	35	42	36	0.843
1059	NM_001810	CENPB	centromere protein B	protein_coding	50	54	51	55	1.066
1060	NM_001362481,NM	CENPC	centromere protein C	protein_coding	10	10	11	11	1.001
1061	gene-CENPCP1	CENPCP1	centromere protein C ps	pseudogene	0	0	1	1	1.000
1062	NM_001286734,NM	CENPE	centromere protein E	protein_coding	39	29	40	30	0.758
1063	NM_016343,XM_01	CENPF	centromere protein F	protein_coding	80	56	81	57	0.698
1066	NM_001025194,NM	CES1	carboxylesterase 1	protein_coding	0	0	1	1	1.000
1068	NM_004066	CETN1	centrin 1	protein_coding	0	0	1	1	1.000
1069	NM_004344	CETN2	centrin 2	protein_coding	30	33	31	34	1.106
1070	NM_001297765,NM	CETN3	centrin 3	protein_coding	42	43	43	44	1.040
1071	NM_000078,NM_00	CETP	cholesteryl ester transfer	protein_coding	0	0	1	1	1.000
1072	NM_005507	CFL1	cofilin 1	protein_coding	2,184	2,353	2,185	2,354	1.078
1073	NM_001243645,NM	CFL2	cofilin 2	protein_coding	6	5	7	6	0.793
1074	gene-CFL1P5	CFL1P5	cofilin 1 pseudogene 5	pseudogene	0	0	1	1	1.091
1075	NM_001114173,NM	CTSC	cathepsin C	protein_coding	1,629	1,610	1,630	1,611	0.988
1080	NM_000492	CFTR	CF transmembrane cond	protein_coding	0	0	1	1	0.996
1081	NM_000735,NM_00	CGA	glycoprotein hormones, a	protein_coding	0	0	1	1	1.000
1082	NM_000737	CGB3	chorionic gonadotropin s	protein_coding	0	0	1	1	1.000
1084	NM_001277163,NM	CEACAM3	CEA cell adhesion molec	protein_coding	0	0	1	1	1.108
1087	NM_001291485,NM	CEACAM7	CEA cell adhesion molec	protein_coding	0	0	1	1	1.000
1088	NM_001816,XM_01	CEACAM8	CEA cell adhesion molec	protein_coding	0	0	1	1	1.000
1089	NM_001362492,NM	CEACAM4	CEA cell adhesion molec	protein_coding	0	0	1	1	1.000
1091	gene-CEACAMP2	CEACAMP2	CEA cell adhesion molec	pseudogene	0	0	1	1	1.000
1101	NM_001267,XM_01	CHAD	chondroadherin	protein_coding	0	0	1	1	1.000
1102	NM_001268,NM_00	RCBTB2	RCC1 and BTB domain c	protein_coding	121	114	122	115	0.946
1103	NM_001142929,NM	CHAT	choline O-acetyltransfera	protein_coding	0	0	1	1	1.000
1104	NM_001048194,NM	RCC1	regulator of chromosome	protein_coding	200	192	201	193	0.960
1105	NM_001270,NM_00	CHD1	chromodomain helicase	protein_coding	45	39	46	40	0.871
1106	NM_001042572,NM	CHD2	chromodomain helicase	protein_coding	42	34	43	35	0.812
1107	NM_001005271,NM	CHD3	chromodomain helicase	protein_coding	34	32	35	33	0.935
1108	NM_001273,NM_00	CHD4	chromodomain helicase	protein_coding	159	155	160	156	0.977
1109	NM_001818	AKR1C4	aldo-keto reductase fami	protein_coding	0	0	1	1	1.000
1111	NM_001114121,NM	CHEK1	checkpoint kinase 1	protein_coding	94	87	95	88	0.930
1112	NM_001085471,NM	FOXN3	forkhead box N3	protein_coding	32	27	33	28	0.841
1113	NM_001275,NM_00	CHGA	chromogranin A	protein_coding	0	0	1	1	1.000
1114	NM_001819	CHGB	chromogranin B	protein_coding	0	0	1	1	1.000
1116	NM_001276	CHI3L1	chitinase 3 like 1	protein_coding	0	0	1	1	0.948
1117	NM_001025197,NM	CHI3L2	chitinase 3 like 2	protein_coding	0	0	1	1	0.812
1118	NM_001256125,NM	CHIT1	chitinase 1	protein_coding	0	0	1	1	1.000
1119	NM_001277,NM_00	CHKA	choline kinase alpha	protein_coding	17	13	18	14	0.809
1120	NM_005198	CHKB	choline kinase beta	protein_coding	4	2	5	3	0.665
1121	NM_000390,NM_00	CHM	CHM Rab escort protein	protein_coding	12	10	13	11	0.881
1122	NM_001381853,NM	CHML	CHM like Rab escort pro	protein_coding	2	1	3	2	0.818
1123	NM_001025201,NM	CHN1	chimerin 1	protein_coding	1	1	2	2	1.004
1124	NM_001039936,NM	CHN2	chimerin 2	protein_coding	13	14	14	15	1.066
1128	NM_000738,XM_01	CHRM1	cholinergic receptor mus	protein_coding	0	0	1	1	1.000
1129	NM_000739,NM_00	CHRM2	cholinergic receptor mus	protein_coding	0	0	1	1	1.000
1130	NM_000081,NM_00	LYST	lysosomal trafficking reg	protein_coding	14	11	15	12	0.810
1131	NM_000740,NM_00	CHRM3	cholinergic receptor mus	protein_coding	0	0	1	1	1.000
1132	NM_000741,NM_00	CHRM4	cholinergic receptor mus	protein_coding	0	0	1	1	1.000
1133	NM_001320917,NM	CHRM5	cholinergic receptor mus	protein_coding	0	0	1	1	1.000

1134	NM_000079,NM_00	**CHRNA1**	cholinergic receptor nico	protein_coding	0	0	1	1	1.000
1135	NM_000742,NM_00	**CHRNA2**	cholinergic receptor nico	protein_coding	0	0	1	1	1.000
1136	NM_000743,NM_00	**CHRNA3**	cholinergic receptor nico	protein_coding	0	0	1	1	1.000
1137	NM_000744,NM_00	**CHRNA4**	cholinergic receptor nico	protein_coding	0	0	1	1	1.000
1138	NM_000745,NM_00	**CHRNA5**	cholinergic receptor nico	protein_coding	8	7	9	8	0.901
1139	NM_000746,NM_00	**CHRNA7**	cholinergic receptor nico	protein_coding	0	0	1	1	1.000
1140	NM_000747	**CHRNB1**	cholinergic receptor nico	protein_coding	4	3	5	4	0.981
1141	NM_000748,XM_01	**CHRNB2**	cholinergic receptor nico	protein_coding	0	0	1	1	1.024
1142	NM_000749,NM_00	**CHRNB3**	cholinergic receptor nico	protein_coding	0	0	1	1	1.000
1143	NM_000750,NM_00	**CHRNB4**	cholinergic receptor nico	protein_coding	0	0	1	1	1.000
1144	NM_000751,NM_00	**CHRND**	cholinergic receptor nico	protein_coding	0	0	1	1	1.000
1145	NM_000080,XM_01	**CHRNE**	cholinergic receptor nico	protein_coding	0	0	1	1	1.000
1146	NM_005199	**CHRNG**	cholinergic receptor nico	protein_coding	0	0	1	1	1.000
1147	NM_001278,NM_00	**CHUK**	component of inhibitor of	protein_coding	64	53	65	54	0.830
1149	NM_001279,NM_00	**CIDEA**	cell death inducing DFF/	protein_coding	0	0	1	1	1.000
1152	NM_001362531,NM	**CKB**	creatine kinase B	protein_coding	0	0	1	1	1.000
1153	NM_001280,NM_00	**CIRBP**	cold inducible RNA bindi	protein_coding	212	231	213	232	1.089
1154	NM_013324,NM_14	**CISH**	cytokine inducible SH2 c	protein_coding	272	275	273	276	1.010
1155	NM_001281,NM_00	**TBCB**	tubulin folding cofactor E	protein_coding	122	140	123	141	1.148
1157	gene-CKBP1	**CKBP1**	creatine kinase B pseudo	pseudogene	0	0	1	1	1.000
1158	NM_001824	**CKM**	creatine kinase, M-type	protein_coding	0	0	1	1	1.000
1159	NM_001375484,NM	**CKMT1B**	creatine kinase, mitochor	protein_coding	0	0	1	1	1.021
1160	NM_001099735,NM	**CKMT2**	creatine kinase, mitochor	protein_coding	0	0	1	1	1.000
1161	NM_000082,NM_00	**ERCC8**	ERCC excision repair 8,	protein_coding	8	14	9	15	1.584
1163	NM_001826,NR_02	**CKS1B**	CDC28 protein kinase re	protein_coding	313	312	314	313	0.997
1164	NM_001827	**CKS2**	CDC28 protein kinase re	protein_coding	483	469	484	470	0.971
1173	NM_001025205,NM	**AP2M1**	adaptor related protein c	protein_coding	288	297	289	298	1.032
1174	NM_001283	**AP1S1**	adaptor related protein c	protein_coding	55	54	56	55	0.979
1175	NM_001301076,NM	**AP2S1**	adaptor related protein c	protein_coding	159	171	160	172	1.076
1176	NM_001002924,NM	**AP3S1**	adaptor related protein c	protein_coding	111	101	112	102	0.908
1178	NM_001828	**CLC**	Charcot-Leyden crystal g	protein_coding	0	0	1	1	1.000
1179	NM_001285	**CLCA1**	chloride channel access	protein_coding	0	0	1	1	1.000
1180	NM_000083,NR_04	**CLCN1**	chloride voltage-gated cl	protein_coding	0	0	1	1	1.000
1181	NM_001171087,NM	**CLCN2**	chloride voltage-gated cl	protein_coding	1	1	2	2	1.050
1182	NM_001243372,NM	**CLCN3**	chloride voltage-gated cl	protein_coding	27	23	28	24	0.854
1183	NM_001256944,NM	**CLCN4**	chloride voltage-gated cl	protein_coding	0	0	1	1	1.000
1184	NM_000084,NM_00	**CLCN5**	chloride voltage-gated cl	protein_coding	6	5	7	6	0.784
1185	NM_001256959,NM	**CLCN6**	chloride voltage-gated cl	protein_coding	6	6	7	7	0.907
1186	NM_001114331,NM	**CLCN7**	chloride voltage-gated cl	protein_coding	15	17	16	18	1.145
1187	NM_001042704,NM	**CLCNKA**	chloride voltage-gated cl	protein_coding	0	0	1	1	1.000
1188	NM_000085,NM_00	**CLCNKB**	chloride voltage-gated cl	protein_coding	0	0	1	1	1.000
1191	NM_001831,NR_03	**CLU**	clusterin	protein_coding	23	23	24	24	1.018
1192	NM_001287593,NM	**CLIC1**	chloride intracellular cha	protein_coding	1,279	1,330	1,280	1,331	1.040
1193	NM_001289	**CLIC2**	chloride intracellular cha	protein_coding	0	0	1	1	1.000
1195	NM_001162407,NM	**CLK1**	CDC like kinase 1	protein_coding	60	59	61	60	0.981
1196	NM_001294338,NM	**CLK2**	CDC like kinase 2	protein_coding	40	36	41	37	0.915
1197	NR_002711	**CLK2P1**	CDC like kinase 2, pseuc	transcribed_pseu	0	0	1	1	1.000
1198	NM_001130028,NM	**CLK3**	CDC like kinase 3	protein_coding	31	26	32	27	0.866
1200	NM_000391	**TPP1**	tripeptidyl peptidase 1	protein_coding	17	15	18	16	0.901
1201	NM_000086,NM_00	**CLN3**	CLN3 lysosomal/endoso	protein_coding	44	44	45	45	0.997
1203	NM_001366624,NM	**CLN5**	CLN5 intracellular traffic	protein_coding	2	1	3	2	0.880
1204	gene-CLNS1AP1	**CLNS1AP1**	chloride nucleotide-sens	pseudogene	0	0	1	1	1.000
1207	NM_001293,NM_00	**CLNS1A**	chloride nucleotide-sens	protein_coding	99	100	100	101	1.013
1208	NM_001252597,NM	**CLPS**	colipase	protein_coding	0	0	1	1	1.000
1209	NM_001282175,NM	**CLPTM1**	CLPTM1 regulator of GA	protein_coding	117	120	118	121	1.025
1211	NM_001076677,NM	**CLTA**	clathrin light chain A	protein_coding	185	200	186	201	1.080
1212	NM_001364126,NM	**CLTB**	clathrin light chain B	protein_coding	129	142	130	143	1.097
1213	NM_001288653,NM	**CLTC**	clathrin heavy chain	protein_coding	173	150	174	151	0.867
1215	NM_001308083,NM	**CMA1**	chymase 1	protein_coding	0	0	1	1	1.000
1230	NM_001295	**CCR1**	C-C motif chemokine rec	protein_coding	369	324	370	325	0.877
1232	NM_001164680,NM	**CCR3**	C-C motif chemokine rec	protein_coding	1	1	2	2	1.022
1233	NM_005508,XM_01	**CCR4**	C-C motif chemokine rec	protein_coding	0	0	1	1	0.996
1234	NM_000579,NM_00	**CCR5**	C-C motif chemokine rec	protein_coding	58	50	59	51	0.868
1235	NM_004367,NM_03	**CCR6**	C-C motif chemokine rec	protein_coding	1	1	2	2	0.954
1236	NM_001301714,NM	**CCR7**	C-C motif chemokine rec	protein_coding	5	5	6	6	0.997
1237	NM_005201	**CCR8**	C-C motif chemokine rec	protein_coding	0	0	1	1	1.000
1238	NM_001296	**ACKR2**	atypical chemokine recep	protein_coding	1	0	2	1	0.681
1240	NM_001142343,NM	**CMKLR1**	chemerin chemokine-like	protein_coding	0	0	1	1	1.024
1241	NM_001143919,NM	**LTB4R**	leukotriene B4 receptor	protein_coding	7	7	8	8	1.027
1244	NM_000392,XM_00	**ABCC2**	ATP binding cassette sul	protein_coding	0	0	1	1	1.103
1258	NM_001135639,NM	**CNGB1**	cyclic nucleotide gated c	protein_coding	0	0	1	1	1.000
1259	NM_000087,NM_00	**CNGA1**	cyclic nucleotide gated c	protein_coding	0	0	1	1	1.000
1260	NM_005140	**CNGA2**	cyclic nucleotide gated c	protein_coding	0	0	1	1	1.000
1261	NM_001079878,NM	**CNGA3**	cyclic nucleotide gated c	protein_coding	0	0	1	1	1.000
1262	NM_001037329,XM	**CNGA4**	cyclic nucleotide gated c	protein_coding	0	0	1	1	1.166
1263	NM_004073,XR_24	**PLK3**	polo like kinase 3	protein_coding	7	8	8	9	1.081
1264	NM_001299,NM_00	**CNN1**	calponin 1	protein_coding	0	0	1	1	1.000
1265	NM_001303499,NM	**CNN2**	calponin 2	protein_coding	426	426	427	427	0.999
1266	NM_001286055,NM	**CNN3**	calponin 3	protein_coding	0	0	1	1	0.949
1267	NM_001330216,NM	**CNP**	2',3'-cyclic nucleotide 3'	protein_coding	54	55	55	56	1.030
1268	NM_001160226,NM	**CNR1**	cannabinoid receptor 1	protein_coding	0	0	1	1	1.000
1269	NM_001841,XM_01	**CNR2**	cannabinoid receptor 2	protein_coding	1	0	2	1	0.949
1270	NM_000614	**CNTF**	ciliary neurotrophic facto	protein_coding	2	2	3	3	1.070
1271	NM_001207011,NM	**CNTFR**	ciliary neurotrophic facto	protein_coding	0	0	1	1	1.000
1272	NM_001256063,NM	**CNTN1**	contactin 1	protein_coding	0	0	1	1	1.000
1277	NM_000088,XM_00	**COL1A1**	collagen type I alpha 1 c	protein_coding	0	0	1	1	1.000
1278	NM_000089	**COL1A2**	collagen type I alpha 2 c	protein_coding	0	0	1	1	1.000
1280	NM_001844,NM_03	**COL2A1**	collagen type II alpha 1	protein_coding	0	0	1	1	0.983
1281	NM_000090	**COL3A1**	collagen type III alpha 1	protein_coding	0	0	1	1	1.000
1282	NM_001303110,NM	**COL4A1**	collagen type IV alpha 1	protein_coding	0	0	1	1	1.000
1284	NM_001846	**COL4A2**	collagen type IV alpha 2	protein_coding	0	0	1	1	1.008
1285	NM_000091,XM_00	**COL4A3**	collagen type IV alpha 3	protein_coding	0	0	1	1	1.000
1286	NM_000092,XM_00	**COL4A4**	collagen type IV alpha 4	protein_coding	0	0	1	1	1.000
1287	NM_000495,NM_03	**COL4A5**	collagen type IV alpha 5	protein_coding	0	0	1	1	1.000
1288	NM_001287758,NM	**COL4A6**	collagen type IV alpha 6	protein_coding	0	0	1	1	1.000
1289	NM_000093,NM_00	**COL5A1**	collagen type V alpha 1	protein_coding	0	0	1	1	1.000
1290	NM_000393,XM_01	**COL5A2**	collagen type V alpha 2	protein_coding	60	50	61	51	0.841
1291	NM_001848	**COL6A1**	collagen type VI alpha 1	protein_coding	0	0	1	1	1.000
1292	NM_001849,NM_05	**COL6A2**	collagen type VI alpha 2	protein_coding	0	0	1	1	1.000
1293	NM_004369,NM_05	**COL6A3**	collagen type VI alpha 3	protein_coding	0	0	1	1	1.000
1294	NM_000094,XM_01	**COL7A1**	collagen type VII alpha 1	protein_coding	0	0	1	1	1.001
1295	NM_001850,NM_02	**COL8A1**	collagen type VIII alpha 1	protein_coding	0	0	1	1	1.000
1296	NM_001294347,NM	**COL8A2**	collagen type VIII alpha 2	protein_coding	0	0	1	1	1.000
1297	NM_001377289,NM	**COL9A1**	collagen type IX alpha 1	protein_coding	0	0	1	1	1.000
1298	NM_001852,XM_00	**COL9A2**	collagen type IX alpha 2	protein_coding	0	0	1	1	1.031
1299	NM_001853,XM_01	**COL9A3**	collagen type IX alpha 3	protein_coding	0	0	1	1	1.000
1300	NM_000493,XM_00	**COL10A1**	collagen type X alpha 1	protein_coding	0	0	1	1	1.000
1301	NM_001190709,NM	**COL11A1**	collagen type XI alpha 1	protein_coding	0	0	1	1	1.000
1302	NM_001163771,NM	**COL11A2**	collagen type XI alpha 2	protein_coding	1	0	2	1	0.902
1303	NM_004370,NM_08	**COL12A1**	collagen type XII alpha 1	protein_coding	0	0	1	1	0.985
1305	NM_001130103,NM	**COL13A1**	collagen type XIII alpha 1	protein_coding	0	0	1	1	1.000
1306	NM_001855,XM_01	**COL15A1**	collagen type XV alpha 1	protein_coding	1	1	2	2	0.990
1307	NM_001856,XM_00	**COL16A1**	collagen type XVI alpha	protein_coding	0	0	1	1	1.000
1308	NM_000494	**COL17A1**	collagen type XVII alpha	protein_coding	0	0	1	1	1.000
1310	NM_001858,XM_01	**COL19A1**	collagen type XIX alpha	protein_coding	0	0	1	1	1.000
1311	NM_000095	**COMP**	cartilage oligomeric matr	protein_coding	0	0	1	1	1.000
1312	NM_000754,NM_00	**COMT**	catechol-O-methyltransfe	protein_coding	15	17	16	18	1.126
1314	NM_001098398,NM	**COPA**	COPI coat complex subu	protein_coding	107	96	108	97	0.905
1315	NM_001144061,NM	**COPB1**	COPI coat complex subu	protein_coding	140	120	141	121	0.857
1316	NM_001160124,NM	**KLF6**	Kruppel like factor 6	protein_coding	42	40	43	41	0.941
1317	NM_001859	**SLC31A1**	solute carrier family 31 m	protein_coding	17	17	18	18	0.989
1318	NM_001860,XM_01	**SLC31A2**	solute carrier family 31 m	protein_coding	0	0	1	1	0.845
1325	NM_001302	**CORT**	cortistatin	protein_coding	0	0	1	1	1.010
1326	NM_001244134,NM	**MAP3K8**	mitogen-activated proteir	protein_coding	4	5	5	6	1.134
1327	NM_001318786,NM	**COX4I1**	cytochrome c oxidase su	protein_coding	623	669	624	670	1.073
1329	NM_001862	**COX5B**	cytochrome c oxidase su	protein_coding	369	425	370	426	1.151

1330	gene-COX5BP1	**COX5BP1**	cytochrome c oxidase su	pseudogene	0	0	1	1	1.000
1332	gene-COX5BP3	**COX5BP3**	cytochrome c oxidase su	pseudogene	0	0	1	1	1.000
1333	gene-COX5BP4	**COX5BP4**	cytochrome c oxidase su	pseudogene	0	0	1	1	1.000
1336	gene-COX5BP7	**COX5BP7**	cytochrome c oxidase su	pseudogene	0	0	1	1	1.000
1337	NM_004373	**COX6A1**	cytochrome c oxidase su	protein_coding	823	892	824	893	1.084
1338	gene-COX6A1P1	**COX6A1P1**	cytochrome c oxidase su	pseudogene	0	0	1	1	1.000
1339	NM_005205	**COX6A2**	cytochrome c oxidase su	protein_coding	0	0	1	1	1.000
1340	NM_001863	**COX6B1**	cytochrome c oxidase su	protein_coding	764	789	765	790	1.033
1345	NM_004374,XM_01	**COX6C**	cytochrome c oxidase su	protein_coding	479	496	480	497	1.035
1346	NM_001864	**COX7A1**	cytochrome c oxidase su	protein_coding	0	0	1	1	1.000
1347	NM_001366292,NM	**COX7A2**	cytochrome c oxidase su	protein_coding	368	356	369	357	0.968
1348	gene-COX7A2P2	**COX7A2P2**	cytochrome c oxidase su	pseudogene	0	0	1	1	1.000
1349	NM_001866	**COX7B**	cytochrome c oxidase su	protein_coding	143	156	144	157	1.093
1350	NM_001867	**COX7C**	cytochrome c oxidase su	protein_coding	624	616	625	617	0.987
1351	NM_004074	**COX8A**	cytochrome c oxidase su	protein_coding	837	875	838	876	1.046
1352	NM_001303	**COX10**	cytochrome c oxidase as	protein_coding	25	21	26	22	0.851
1353	NM_001162861,NM	**COX11**	cytochrome c oxidase co	protein_coding	46	41	47	42	0.894
1355	NM_001320974,NM	**COX15**	cytochrome c oxidase as	protein_coding	28	30	29	31	1.081
1356	NM_000096,NR_04	**CP**	ceruloplasmin	protein_coding	0	0	1	1	1.000
1357	NM_001868	**CPA1**	carboxypeptidase A1	protein_coding	0	0	1	1	1.000
1358	NM_001869	**CPA2**	carboxypeptidase A2	protein_coding	0	0	1	1	1.000
1359	NM_001870	**CPA3**	carboxypeptidase A3	protein_coding	0	0	1	1	1.000
1360	NM_001871	**CPB1**	carboxypeptidase B1	protein_coding	0	0	1	1	1.000
1361	NM_001278541,NM	**CPB2**	carboxypeptidase B2	protein_coding	0	0	1	1	1.000
1362	NM_001199775,NM	**CPD**	carboxypeptidase D	protein_coding	54	46	55	47	0.857
1363	NM_001873	**CPE**	carboxypeptidase E	protein_coding	0	0	1	1	1.000
1364	NM_001305	**CLDN4**	claudin 4	protein_coding	0	0	1	1	1.059
1365	NM_001306	**CLDN3**	claudin 3	protein_coding	0	0	1	1	1.000
1366	NM_001185022,NM	**CLDN7**	claudin 7	protein_coding	0	0	1	1	1.000
1368	NM_001005502,NM	**CPM**	carboxypeptidase M	protein_coding	0	0	1	1	1.000
1369	NM_001308	**CPN1**	carboxypeptidase N subu	protein_coding	0	0	1	1	1.000
1370	NM_001080513,NM	**CPN2**	carboxypeptidase N subu	protein_coding	0	0	1	1	1.000
1371	NM_000097,XM_00	**CPOX**	coproporphyrinogen oxid	protein_coding	133	120	134	121	0.906
1373	NM_001122633,NM	**CPS1**	carbamoyl-phosphate sy	protein_coding	2	2	3	3	0.977
1374	NM_001031847,NM	**CPT1A**	carnitine palmitoyltransfe	protein_coding	0	0	1	1	1.000
1375	NM_001145134,NM	**CPT1B**	carnitine palmitoyltransfe	protein_coding	5	4	6	5	0.781
1376	NM_000098,NM_00	**CPT2**	carnitine palmitoyltransfe	protein_coding	14	15	15	16	1.080
1378	NM_000651	**CR1**	complement C3b/C4b re	protein_coding	0	0	1	1	1.000
1379	NM_175710	**CR1L**	complement C3b/C4b re	protein_coding	0	0	1	1	1.000
1380	NM_001006658,NM	**CR2**	complement C3d recepto	protein_coding	0	0	1	1	1.000
1381	NM_004378	**CRABP1**	cellular retinoic acid bind	protein_coding	0	0	1	1	1.000
1382	NM_001199723,NM	**CRABP2**	cellular retinoic acid bind	protein_coding	0	0	1	1	0.921
1384	NM_000755,NM_00	**CRAT**	carnitine O-acetyltransfe	protein_coding	0	0	1	1	1.000
1385	NM_001320793,NM	**CREB1**	cAMP responsive elemer	protein_coding	23	18	24	19	0.801
1386	NM_001256090,NM	**ATF2**	activating transcription fa	protein_coding	47	38	48	39	0.811
1387	NM_001079846,NM	**CREBBP**	CREB binding protein	protein_coding	38	34	39	35	0.887
1388	NM_001136153,NM	**ATF6B**	activating transcription fa	protein_coding	98	96	99	97	0.982
1389	NM_001310	**CREBL2**	cAMP responsive elemer	protein_coding	9	8	10	9	0.932
1390	NM_001267562,NM	**CREM**	cAMP responsive elemer	protein_coding	19	16	20	17	0.885
1392	NM_000756	**CRH**	corticotropin releasing ho	protein_coding	0	0	1	1	1.000
1393	NM_001882,XR_94	**CRHBP**	corticotropin releasing ho	protein_coding	0	0	1	1	1.000
1394	NM_001145146,NM	**CRHR1**	corticotropin releasing ho	protein_coding	0	0	1	1	1.000
1395	NM_001202475,NM	**CRHR2**	corticotropin releasing ho	protein_coding	0	0	1	1	1.000
1396	NM_001311	**CRIP1**	cysteine rich protein 1	protein_coding	60	75	61	76	1.248
1397	NM_001270837,NM	**CRIP2**	cysteine rich protein 2	protein_coding	0	0	1	1	1.000
1398	NM_005206,NM_01	**CRK**	CRK proto-oncogene, ad	protein_coding	18	16	19	17	0.879
1399	NM_005207,NR_15	**CRKL**	CRK like proto-oncogene	protein_coding	81	71	82	72	0.876
1400	NM_001014809,NM	**CRMP1**	collapsin response medi	protein_coding	2	2	3	3	0.953
1401	NM_000567,NM_00	**CRP**	C-reactive protein	protein_coding	0	0	1	1	1.000
1404	NM_001884,XM_01	**HAPLN1**	hyaluronan and proteogl	protein_coding	0	0	1	1	1.000
1406	NM_000554	**CRX**	cone-rod homeobox	protein_coding	0	0	1	1	1.000
1407	NM_004075,XM_01	**CRY1**	cryptochrome circadian r	protein_coding	4	4	5	5	1.091
1408	NM_001127457,NM	**CRY2**	cryptochrome circadian r	protein_coding	19	18	20	19	0.923
1409	NM_000394,NM_00	**CRYAA**	crystallin alpha A	protein_coding	0	0	1	1	1.000
1410	NM_001289807,NM	**CRYAB**	crystallin alpha B	protein_coding	0	0	1	1	1.000
1411	NM_005208,XM_01	**CRYBA1**	crystallin beta A1	protein_coding	0	0	1	1	1.000
1412	NM_057093,NM_05	**CRYBA2**	crystallin beta A2	protein_coding	0	0	1	1	1.000
1413	NM_001886,XM_00	**CRYBA4**	crystallin beta A4	protein_coding	1	0	2	1	0.843
1414	NM_001887,XM_01	**CRYBB1**	crystallin beta B1	protein_coding	0	0	1	1	0.866
1415	NM_000496,XM_00	**CRYBB2**	crystallin beta B2	protein_coding	1	1	2	2	1.015
1416	NR_033733,NR_03	**CRYBB2P1**	crystallin beta B2 pseud	transcribed_pseu	27	25	28	26	0.919
1417	NM_004076,XM_01	**CRYBB3**	crystallin beta B3	protein_coding	1	1	2	2	1.472
1418	NM_014617	**CRYGA**	crystallin gamma A	protein_coding	0	0	1	1	1.000
1419	NM_005210,XM_01	**CRYGB**	crystallin gamma B	protein_coding	0	0	1	1	1.000
1420	NM_020989,XM_01	**CRYGC**	crystallin gamma C	protein_coding	0	0	1	1	1.000
1421	NM_006891	**CRYGD**	crystallin gamma D	protein_coding	0	0	1	1	1.000
1423	gene-CRYGFP	**CRYGFP**	crystallin gamma F, pseu	pseudogene	0	0	1	1	1.000
1424	gene-CRYGGP	**CRYGGP**	crystallin gamma G, pse	pseudogene	0	0	1	1	1.000
1427	NM_017541	**CRYGS**	crystallin gamma S	protein_coding	1	0	2	1	0.781
1428	NM_001376256,NM	**CRYM**	crystallin mu	protein_coding	0	0	1	1	1.000
1429	NM_001130042,NM	**CRYZ**	crystallin zeta	protein_coding	63	51	64	52	0.823
1431	NM_004077	**CS**	citrate synthase	protein_coding	168	158	169	159	0.940
1432	NM_001315,NM_13	**MAPK14**	mitogen-activated protei	protein_coding	50	44	51	45	0.889
1434	NM_001256135,NM	**CSE1L**	chromosome segregatior	protein_coding	221	206	222	207	0.932
1435	NM_000757,NM_17	**CSF1**	colony stimulating factor	protein_coding	339	247	340	248	0.730
1436	NM_001288705,NM	**CSF1R**	colony stimulating factor	protein_coding	0	0	1	1	0.882
1437	NM_000758	**CSF2**	colony stimulating factor	protein_coding	0	0	1	1	1.000
1438	NM_001161529,NM	**CSF2RA**	colony stimulating factor	protein_coding	0	0	1	1	1.000
1439	NM_000395,XM_00	**CSF2RB**	colony stimulating factor	protein_coding	36	34	37	35	0.941
1440	NM_000759,NM_00	**CSF3**	colony stimulating factor	protein_coding	0	0	1	1	1.000
1441	NM_000760,NM_15	**CSF3R**	colony stimulating factor	protein_coding	0	0	1	1	1.000
1442	NM_001317	**CSH1**	chorionic somatomammo	protein_coding	0	0	1	1	1.000
1443	NM_020991,NM_02	**CSH2**	chorionic somatomammo	protein_coding	0	0	1	1	1.000
1444	NM_001318,NM_00	**CSHL1**	chorionic somatomammo	protein_coding	0	0	1	1	1.000
1445	NM_001127190,NM	**CSK**	C-terminal Src kinase	protein_coding	300	314	301	315	1.047
1446	NM_001025104,NM	**CSN1S1**	casein alpha s1	protein_coding	0	0	1	1	1.000
1447	NM_001302770,NM	**CSN2**	casein beta	protein_coding	0	0	1	1	1.000
1448	NM_005212,XM_01	**CSN3**	casein kappa	protein_coding	0	0	1	1	1.000
1452	NM_001025105,NM	**CSNK1A1**	casein kinase 1 alpha 1	protein_coding	121	113	122	114	0.930
1453	NM_001363749,NM	**CSNK1D**	casein kinase 1 delta	protein_coding	89	95	90	96	1.066
1454	NM_001894,NM_15	**CSNK1E**	casein kinase 1 epsilon	protein_coding	52	50	53	51	0.968
1455	NM_001319,XM_00	**CSNK1G2**	casein kinase 1 gamma 2	protein_coding	76	77	77	78	1.011
1456	NM_001031812,NM	**CSNK1G3**	casein kinase 1 gamma 3	protein_coding	13	12	14	13	0.880
1457	NM_001362770,NM	**CSNK2A1**	casein kinase 2 alpha 1	protein_coding	59	59	60	60	0.989
1459	NM_001896,XM_00	**CSNK2A2**	casein kinase 2 alpha 2	protein_coding	72	66	73	67	0.914
1460	NM_001282385,NM	**CSNK2B**	casein kinase 2 beta	protein_coding	461	461	462	462	1.000
1462	NM_001126336,NM	**VCAN**	versican	protein_coding	0	0	1	1	1.008
1463	NM_004386	**NCAN**	neurocan	protein_coding	0	0	1	1	1.000
1464	NM_001897	**CSPG4**	chondroitin sulfate prote	protein_coding	0	0	1	1	0.966
1465	NM_001193570,NM	**CSRP1**	cysteine and glycine rich	protein_coding	118	117	119	118	0.992
1466	NM_001300965,NM	**CSRP2**	cysteine and glycine rich	protein_coding	0	0	1	1	1.000
1467	gene-CSRP2P1	**CSRP2P1**	cysteine and glycine rich	pseudogene	0	0	1	1	0.854
1468	NM_001270888,NM	**SLC25A10**	solute carrier family 25 m	protein_coding	20	21	21	22	1.065
1469	NM_001898	**CST1**	cystatin SN	protein_coding	0	0	1	1	1.000
1470	NM_001322	**CST2**	cystatin SA	protein_coding	0	0	1	1	1.000
1471	NM_000099,NM_00	**CST3**	cystatin C	protein_coding	0	0	1	1	1.000
1472	NM_001899	**CST4**	cystatin S	protein_coding	0	0	1	1	1.000
1473	NM_001900	**CST5**	cystatin D	protein_coding	0	0	1	1	1.000
1474	NM_001323	**CST6**	cystatin E/M	protein_coding	0	0	1	1	1.000
1475	NM_005213	**CSTA**	cystatin A	protein_coding	0	0	1	1	1.000
1476	NM_000100	**CSTB**	cystatin B	protein_coding	147	147	148	148	1.003
1477	NM_001033521,NM	**CSTF1**	cleavage stimulation fact	protein_coding	59	57	60	58	0.967
1478	NM_001306206,NM	**CSTF2**	cleavage stimulation fact	protein_coding	38	37	39	38	0.965
1479	NM_001033505,NM	**CSTF3**	cleavage stimulation fact	protein_coding	77	76	78	77	0.985
1482	NM_001166175,NM	**NKX2-5**	NK2 homeobox 5	protein_coding	0	0	1	1	1.000
1485	NM_001327	**CTAG1B**	cancer/testis antigen 1B	protein_coding	0	0	1	1	1.000

1486	NM_004388	**CTBS**	chitobiase	protein_coding	5	5	6	6	0.972
1487	NM_001012614,NM	**CTBP1**	C-terminal binding protei	protein_coding	106	122	107	123	1.151
1488	NM_001083914,NM	**CTBP2**	C-terminal binding protei	protein_coding	0	0	1	1	1.000
1489	NM_001142544,NM	**CTF1**	cardiotrophin 1	protein_coding	0	0	1	1	1.000
1490	NM_001901	**CCN2**	cellular communication n	protein_coding	0	0	1	1	1.000
1491	NM_001190463,NM	**CTH**	cystathionine gamma-lya	protein_coding	3	3	4	4	1.107
1493	NM_001037631,NM	**CTLA4**	cytotoxic T-lymphocyte a	protein_coding	2	2	3	3	1.003
1495	NM_001290307,NM	**CTNNA1**	catenin alpha 1	protein_coding	20	17	21	18	0.860
1496	NM_001164883,NM	**CTNNA2**	catenin alpha 2	protein_coding	0	0	1	1	1.000
1497	NM_001031681,NM	**CTNS**	cystinosin, lysosomal cys	protein_coding	3	3	4	4	1.166
1498	gene-CTNNA1P1	**CTNNA1P1**	catenin alpha 1 pseudog	pseudogene	0	0	1	1	1.000
1499	NM_001098209,NM	**CTNNB1**	catenin beta 1	protein_coding	125	123	126	124	0.982
1500	NM_001085458,NM	**CTNND1**	catenin delta 1	protein_coding	2	2	3	3	0.850
1501	NM_001288715,NM	**CTNND2**	catenin delta 2	protein_coding	0	0	1	1	1.000
1503	NM_001301237,NM	**CTPS1**	CTP synthase 1	protein_coding	172	173	173	174	1.001
1504	NM_001329190,NM	**CTRB1**	chymotrypsinogen B1	protein_coding	0	0	1	1	1.000
1506	NM_001907	**CTRL**	chymotrypsin like	protein_coding	6	6	7	7	0.892
1508	NM_001317237,NM	**CTSB**	cathepsin B	protein_coding	0	0	1	1	1.000
1509	NM_001909	**CTSD**	cathepsin D	protein_coding	175	195	176	196	1.112
1510	NM_001317331,NM	**CTSE**	cathepsin E	protein_coding	0	0	1	1	1.000
1511	NM_001911,XM_01	**CTSG**	cathepsin G	protein_coding	0	0	1	1	1.000
1512	NM_001319137,NM	**CTSH**	cathepsin H	protein_coding	0	0	1	1	1.000
1513	NM_000396	**CTSK**	cathepsin K	protein_coding	2	1	3	2	0.815
1514	NM_001257971,NM	**CTSL**	cathepsin L	protein_coding	0	0	1	1	1.000
1515	NM_001201575,NM	**CTSV**	cathepsin V	protein_coding	0	0	1	1	1.000
1517	NR_033407	**CTSLP2**	cathepsin L pseudogene	transcribed_pseu	0	0	1	1	0.889
1518	NR_033405	**CTSLP8**	cathepsin L pseudogene	transcribed_pseu	0	0	1	1	1.000
1519	NM_001334	**CTSO**	cathepsin O	protein_coding	0	0	1	1	0.939
1520	NM_001199739,NM	**CTSS**	cathepsin S	protein_coding	34	30	35	31	0.880
1521	NM_001335	**CTSW**	cathepsin W	protein_coding	1,333	1,392	1,334	1,393	1.044
1522	NM_001336	**CTSZ**	cathepsin Z	protein_coding	22	24	23	25	1.071
1523	NM_001202543,NM	**CUX1**	cut like homeobox 1	protein_coding	10	9	11	10	0.872
1524	NM_001171171,NM	**CX3CR1**	C-X3-C motif chemokine	protein_coding	6	8	7	9	1.243
1525	NM_001207063,NM	**CXADR**	CXADR Ig-like cell adhes	protein_coding	0	0	1	1	1.072
1527	NM_001205201,NM	**TEX28**	testis expressed 28	protein_coding	0	0	1	1	1.000
1528	NM_001190807,NM	**CYB5A**	cytochrome b5 type A	protein_coding	0	0	1	1	1.000
1531	gene-CYB5AP3	**CYB5AP3**	cytochrome b5 type A ps	pseudogene	0	0	1	1	1.000
1532	gene-CYB5AP4	**CYB5AP4**	cytochrome b5 type A ps	pseudogene	0	0	1	1	1.000
1534	NM_001017916,NM	**CYB561**	cytochrome b561	protein_coding	0	0	1	1	1.000
1535	NM_000101,XM_01	**CYBA**	cytochrome b-245 alpha	protein_coding	438	484	439	485	1.105
1536	NM_000397	**CYBB**	cytochrome b-245 beta c	protein_coding	2	2	3	3	1.058
1537	NM_001916,XM_01	**CYC1**	cytochrome c1	protein_coding	162	166	163	167	1.027
1538	NM_001271680,NM	**CYLC1**	cylicin 1	protein_coding	0	0	1	1	1.000
1539	NM_001340	**CYLC2**	cylicin 2	protein_coding	0	0	1	1	1.000
1540	NM_001042355,NM	**CYLD**	CYLD lysine 63 deubiqui	protein_coding	12	11	13	12	0.952
1543	NM_000499,NM_00	**CYP1A1**	cytochrome P450 family	protein_coding	0	0	1	1	1.000
1544	NM_000761	**CYP1A2**	cytochrome P450 family	protein_coding	0	0	1	1	1.000
1545	NM_000104	**CYP1B1**	cytochrome P450 family	protein_coding	0	0	1	1	1.000
1548	NM_000762	**CYP2A6**	cytochrome P450 family	protein_coding	0	0	1	1	1.000
1549	NM_000764,NM_03	**CYP2A7**	cytochrome P450 family	protein_coding	0	0	1	1	1.000
1550	gene-CYP2A7P1	**CYP2A7P1**	cytochrome P450 family	pseudogene	0	0	1	1	1.000
1551	NM_000765	**CYP3A7**	cytochrome P450 family	protein_coding	0	0	1	1	1.000
1553	NM_000766	**CYP2A13**	cytochrome P450 family	protein_coding	0	0	1	1	1.000
1555	NM_000767	**CYP2B6**	cytochrome P450 family	protein_coding	0	0	1	1	1.000
1556	NR_001278	**CYP2B7P**	cytochrome P450 family	transcribed_pseu	0	0	1	1	1.000
1557	NM_000769	**CYP2C19**	cytochrome P450 family	protein_coding	0	0	1	1	1.000
1558	NM_000770,NM_00	**CYP2C8**	cytochrome P450 family	protein_coding	0	0	1	1	1.000
1559	NM_000771	**CYP2C9**	cytochrome P450 family	protein_coding	0	0	1	1	1.000
1562	NM_000772,NM_00	**CYP2C18**	cytochrome P450 family	protein_coding	0	0	1	1	1.043
1564	NM_001348386,NR	**CYP2D7**	cytochrome P450 family	protein_coding	0	0	1	1	1.050
1565	NM_000106,NM_00	**CYP2D6**	cytochrome P450 family	protein_coding	1	0	2	1	0.741
1568	gene-CYP2D8P	**CYP2D8P**	ccytochrome P450 family	pseudogene	0	0	1	1	1.000
1571	NM_000773	**CYP2E1**	cytochrome P450 family	protein_coding	0	0	1	1	1.013
1572	NM_000774,NR_13	**CYP2F1**	cytochrome P450 family	protein_coding	0	0	1	1	1.000
1573	NM_000775,NR_13	**CYP2J2**	cytochrome P450 family	protein_coding	0	0	1	1	1.000
1576	NM_001202855,NM	**CYP3A4**	cytochrome P450 family	protein_coding	0	0	1	1	1.000
1577	NM_000777,NM_00	**CYP3A5**	cytochrome P450 family	protein_coding	0	0	1	1	1.000
1579	NM_000778,NM_00	**CYP4A11**	cytochrome P450 family	protein_coding	0	0	1	1	1.000
1580	NM_000779,NM_00	**CYP4B1**	cytochrome P450 family	protein_coding	0	0	1	1	1.000
1581	NM_000780	**CYP7A1**	cytochrome P450 family	protein_coding	0	0	1	1	1.000
1582	NM_004391	**CYP8B1**	cytochrome P450 family	protein_coding	0	0	1	1	1.000
1583	NM_000781,NM_00	**CYP11A1**	cytochrome P450 family	protein_coding	0	0	1	1	1.000
1584	NM_000497,NM_00	**CYP11B1**	cytochrome P450 family	protein_coding	0	0	1	1	1.000
1585	NM_000498	**CYP11B2**	cytochrome P450 family	protein_coding	0	0	1	1	1.000
1586	NM_000102	**CYP17A1**	cytochrome P450 family	protein_coding	0	0	1	1	1.000
1587	NR_001569,NR_02	**ADAM3A**	ADAM metallopeptidase	transcribed_pseu	0	0	1	1	1.000
1588	NM_000103,NM_00	**CYP19A1**	cytochrome P450 family	protein_coding	0	0	1	1	0.896
1589	NM_000500,NM_00	**CYP21A2**	cytochrome P450 family	protein_coding	0	0	1	1	1.000
1590	NR_040090	**CYP21A1P**	cytochrome P450 family	transcribed_pseu	0	0	1	1	1.000
1591	NM_000782,NM_00	**CYP24A1**	cytochrome P450 family	protein_coding	0	0	1	1	1.000
1592	NM_000783,NM_05	**CYP26A1**	cytochrome P450 family	protein_coding	0	0	1	1	1.000
1593	NM_000784,XM_01	**CYP27A1**	cytochrome P450 family	protein_coding	0	0	1	1	1.000
1594	NM_000785	**CYP27B1**	cytochrome P450 family	protein_coding	4	4	5	5	0.873
1595	NM_000786,NM_00	**CYP51A1**	cytochrome P450 family	protein_coding	75	60	76	61	0.802
1596	NR_144553	**ADAM3B**	ADAM metallopeptidase	transcribed_pseu	0	0	1	1	1.000
1600	NM_001353980,NM	**DAB1**	DAB adaptor protein 1	protein_coding	0	0	1	1	0.941
1601	NM_001244871,NM	**DAB2**	DAB adaptor protein 2	protein_coding	0	0	1	1	1.000
1602	NM_001366712,NM	**DACH1**	dachshund family transc	protein_coding	0	0	1	1	1.000
1603	NM_001344	**DAD1**	defender against cell dea	protein_coding	193	207	194	208	1.075
1604	NM_000574,NM_00	**CD55**	CD55 molecule (Cromer	protein_coding	0	0	1	1	1.000
1605	NM_001165928,NM	**DAG1**	dystroglycan 1	protein_coding	18	17	19	18	0.967
1606	NM_001345,NM_00	**DGKA**	diacylglycerol kinase alp	protein_coding	58	57	59	58	0.985
1607	NM_001350705,NM	**DGKB**	diacylglycerol kinase bet	protein_coding	0	0	1	1	1.000
1608	NM_001080744,NM	**DGKG**	diacylglycerol kinase gar	protein_coding	0	0	1	1	1.000
1609	NM_001347,XM_01	**DGKQ**	diacylglycerol kinase the	protein_coding	1	2	2	3	1.111
1610	NM_001917,XM_00	**DAO**	D-amino acid oxidase	protein_coding	0	0	1	1	1.000
1611	NM_001291963,NM	**DAP**	death associated protein	protein_coding	62	65	63	66	1.041
1612	NM_001288729,NM	**DAPK1**	death associated protein	protein_coding	0	0	1	1	1.000
1613	NM_001348,NM_00	**DAPK3**	death associated protein	protein_coding	47	50	48	51	1.059
1615	NM_001293312,NM	**DARS1**	aspartyl-tRNA synthetase	protein_coding	137	125	138	126	0.916
1616	NM_001141969,NM	**DAXX**	death domain associated	protein_coding	122	121	123	122	0.992
1617	NM_004081,XM_01	**DAZ1**	deleted in azoospermia 1	protein_coding	0	0	1	1	1.000
1618	NM_001190811,NM	**DAZL**	deleted in azoospermia l	protein_coding	0	0	1	1	1.062
1620	NM_014618	**BRINP1**	BMP/retinoic acid inducit	protein_coding	0	0	1	1	1.000
1621	NM_000787	**DBH**	dopamine beta-hydroxyla	protein_coding	0	0	1	1	1.000
1622	NM_001079862,NM	**DBI**	diazepam binding inhibit	protein_coding	581	611	582	612	1.053
1624	gene-DBIP1	**DBIP1**	DBI pseudogene 1	pseudogene	0	0	1	1	1.000
1627	NM_001363541,NM	**DBN1**	drebrin 1	protein_coding	0	0	1	1	0.966
1628	NM_001352,XM_01	**DBP**	D-box binding PAR bZIP	protein_coding	2	2	3	3	1.032
1629	NM_001918,XM_00	**DBT**	dihydrolipoamide branch	protein_coding	9	7	10	8	0.867
1630	NM_005215,XM_01	**DCC**	DCC netrin 1 receptor	protein_coding	0	0	1	1	1.000
1632	NM_001178029,NM	**ECI1**	enoyl-CoA delta isomera	protein_coding	24	23	25	24	0.963
1633	NM_000788	**DCK**	deoxycytidine kinase	protein_coding	139	122	140	123	0.883
1634	NM_001920,NM_13	**DCN**	decorin	protein_coding	0	0	1	1	0.924
1635	NM_001012732,NM	**DCTD**	dCMP deaminase	protein_coding	84	84	85	85	0.994
1636	NM_000789,NM_00	**ACE**	angiotensin I converting	protein_coding	0	0	1	1	1.000
1638	NM_001129889,NM	**DCT**	dopachrome tautomerase	protein_coding	0	0	1	1	1.000
1639	NM_001135040,NM	**DCTN1**	dynactin subunit 1	protein_coding	163	162	164	163	0.993
1641	NM_000555,NM_00	**DCX**	doublecortin	protein_coding	0	0	1	1	1.000
1642	NM_001923	**DDB1**	damage specific DNA bin	protein_coding	292	287	293	288	0.984
1643	NM_000107,NM_00	**DDB2**	damage specific DNA bin	protein_coding	19	23	20	24	1.199
1644	NM_000790,NM_00	**DDC**	dopa decarboxylase	protein_coding	1	1	2	2	0.959
1645	NM_001353,XM_01	**AKR1C1**	aldo-keto reductase fami	protein_coding	0	0	1	1	1.000
1646	NM_001135241,NM	**AKR1C2**	aldo-keto reductase fami	protein_coding	0	0	1	1	1.000
1647	NM_001199741,NM	**GADD45A**	growth arrest and DNA d	protein_coding	4	4	5	5	1.000
1648	gene-GADD45AP1	**GADD45AP1**	growth arrest and DNA d	pseudogene	0	0	1	1	1.000

1649	NM_001195053,NM	**DDIT3**	DNA damage inducible t	protein_coding	26	29	27	30	1.091
1650	NM_005216	**DDOST**	dolichyl-diphosphooligos	protein_coding	366	386	367	387	1.055
1652	NM_001084392,NM	**DDT**	D-dopachrome tautomer	protein_coding	155	157	156	158	1.014
1653	NM_004939	**DDX1**	DEAD-box helicase 1	protein_coding	147	143	148	144	0.972
1654	NM_001193416,NM	**DDX3X**	DEAD-box helicase 3 X-l	protein_coding	0	0	1	1	1.000
1655	NM_001320595,NM	**DDX5**	DEAD-box helicase 5	protein_coding	413	413	414	414	1.001
1656	NM_001257191,NM	**DDX6**	DEAD-box helicase 6	protein_coding	96	79	97	80	0.828
1657	NM_001290321,NM	**DMXL1**	Dmx like 1	protein_coding	10	8	11	9	0.792
1659	NM_001302623,NM	**DHX8**	DEAH-box helicase 8	protein_coding	43	39	44	40	0.919
1660	NM_001357,NR_03	**DHX9**	DExH-box helicase 9	protein_coding	292	270	293	271	0.927
1661	gene-DHX9P1	**DHX9P1**	DEAH-box helicase 9 ps	pseudogene	0	0	1	1	1.000
1662	NM_004398	**DDX10**	DEAD-box helicase 10	protein_coding	60	54	61	55	0.910
1663	NM_001257144,NM	**DDX11**	DEAD/H-box helicase 11	protein_coding	32	32	33	33	0.995
1665	NM_001358,XR_00	**DHX15**	DEAH-box helicase 15	protein_coding	258	222	259	223	0.859
1666	NM_001330575,NM	**DECR1**	2,4-dienoyl-CoA reducta	protein_coding	44	42	45	43	0.966
1667	NM_004084,XM_01	**DEFA1**	defensin alpha 1	protein_coding	0	0	1	1	1.000
1668	NM_005217,XM_01	**DEFA3**	defensin alpha 3	protein_coding	0	0	1	1	1.000
1669	NM_001925	**DEFA4**	defensin alpha 4	protein_coding	0	0	1	1	1.000
1670	NM_021010	**DEFA5**	defensin alpha 5	protein_coding	0	0	1	1	1.000
1671	NM_001926	**DEFA6**	defensin alpha 6	protein_coding	0	0	1	1	1.000
1672	NM_005218	**DEFB1**	defensin beta 1	protein_coding	0	0	1	1	1.000
1673	NM_004942	**DEFB4A**	defensin beta 4A	protein_coding	0	0	1	1	1.000
1674	NM_001382708,NM	**DES**	desmin	protein_coding	1	1	2	2	1.102
1675	NM_001317335,NM	**CFD**	complement factor D	protein_coding	0	0	1	1	1.000
1676	NM_004401,NM_21	**DFFA**	DNA fragmentation facto	protein_coding	114	101	115	102	0.891
1677	NM_001282669,NM	**DFFB**	DNA fragmentation facto	protein_coding	6	4	7	5	0.744
1678	NM_001145951,NM	**TIMM8A**	translocase of inner mito	protein_coding	46	38	47	39	0.838
1687	NM_001127453,NM	**GSDME**	gasdermin E	protein_coding	0	0	1	1	0.906
1690	NM_001135058,NM	**COCH**	cochlin	protein_coding	0	0	1	1	1.000
1716	NM_001318859,NM	**DGUOK**	deoxyguanosine kinase	protein_coding	137	146	138	147	1.060
1717	NM_001163817,NM	**DHCR7**	7-dehydrocholesterol rec	protein_coding	67	66	68	67	0.992
1718	NM_014762	**DHCR24**	24-dehydrocholesterol re	protein_coding	127	131	128	132	1.031
1719	NM_000791,NM_00	**DHFR**	dihydrofolate reductase	protein_coding	159	149	160	150	0.934
1720	NR_033423	**DHFRP3**	dihydrofolate reductase	transcribed_pseu	0	0	1	1	1.000
1723	NM_001361,XM_00	**DHODH**	dihydroorotate dehydrog	protein_coding	10	11	11	12	1.147
1725	NM_001206974,NM	**DHPS**	deoxyhypusine synthase	protein_coding	70	74	71	75	1.047
1727	NM_000398,NM_00	**CYB5R3**	cytochrome b5 reductase	protein_coding	33	33	34	34	1.026
1728	NM_000903,NM_00	**NQO1**	NAD(P)H quinone dehyd	protein_coding	11	13	12	14	1.146
1729	NM_001079812,NM	**DIAPH1**	diaphanous related form	protein_coding	209	195	210	196	0.934
1730	NM_006729,NM_00	**DIAPH2**	diaphanous related form	protein_coding	5	4	6	5	0.827
1731	NM_001365977,NM	**SEPTIN1**	septin 1	protein_coding	196	214	197	215	1.093
1733	NM_000792,NM_00	**DIO1**	iodothyronine deiodinase	protein_coding	0	0	1	1	0.924
1734	NM_000793,NM_00	**DIO2**	iodothyronine deiodinase	protein_coding	0	0	1	1	1.000
1735	NM_001362	**DIO3**	iodothyronine deiodinase	protein_coding	0	0	1	1	1.000
1736	NM_001142463,NM	**DKC1**	dyskerin pseudouridine s	protein_coding	333	323	334	324	0.970
1737	NM_001372031,NM	**DLAT**	dihydrolipoamide S-acety	protein_coding	88	79	89	80	0.907
1738	NM_000108,NM_00	**DLD**	dihydrolipoamide dehydr	protein_coding	81	74	82	75	0.910
1739	NM_001098424,NM	**DLG1**	discs large MAGUK scaf	protein_coding	23	21	24	22	0.915
1740	NM_001142699,NM	**DLG2**	discs large MAGUK scaf	protein_coding	1	1	2	2	0.978
1741	NM_001166278,NM	**DLG3**	discs large MAGUK scaf	protein_coding	25	22	26	23	0.903
1742	NM_001128827,NM	**DLG4**	discs large MAGUK scaf	protein_coding	0	0	1	1	0.980
1743	NM_001244883,NM	**DLST**	dihydrolipoamide S-succ	protein_coding	70	70	71	71	1.004
1744	NR_130749	**DLSTP1**	dihydrolipoamide S-succ	transcribed_pseu	1	1	2	2	0.851
1745	NM_001038493,NM	**DLX1**	distal-less homeobox 1	protein_coding	0	0	1	1	0.751
1746	NM_004405	**DLX2**	distal-less homeobox 2	protein_coding	0	0	1	1	1.000
1747	NM_005220	**DLX3**	distal-less homeobox 3	protein_coding	0	0	1	1	1.000
1748	NM_001934,NM_13	**DLX4**	distal-less homeobox 4	protein_coding	1	1	2	2	0.836
1749	NM_005221,XM_00	**DLX5**	distal-less homeobox 5	protein_coding	0	0	1	1	1.000
1750	NM_005222	**DLX6**	distal-less homeobox 6	protein_coding	0	0	1	1	1.000
1755	NM_001320644,NM	**DMBT1**	deleted in malignant bra	protein_coding	0	0	1	1	1.000
1756	NM_000109,NM_00	**DMD**	dystrophin	protein_coding	1	1	2	2	0.987
1757	NM_001134707,NM	**SARDH**	sarcosine dehydrogenas	protein_coding	0	0	1	1	1.000
1758	NM_001079911,NM	**DMP1**	dentin matrix acidic phos	protein_coding	0	0	1	1	0.973
1759	NM_001005336,NM	**DNM1**	dynamin 1	protein_coding	0	0	1	1	1.000
1760	NM_001081560,NM	**DMPK**	DM1 protein kinase	protein_coding	23	21	24	22	0.915
1761	NM_001363767,NM	**DMRT1**	doublesex and mab-3 rel	protein_coding	9	9	10	10	0.978
1762	NM_004943	**DMWD**	DM1 locus, WD repeat c	protein_coding	43	41	44	42	0.951
1763	NM_001080449,NR	**DNA2**	DNA replication helicase	protein_coding	50	41	51	42	0.832
1767	NM_001369,XM_00	**DNAH5**	dynein axonemal heavy c	protein_coding	0	0	1	1	1.000
1768	NM_001370,XM_00	**DNAH6**	dynein axonemal heavy c	protein_coding	0	0	1	1	0.973
1769	NM_001206927,NM	**DNAH8**	dynein axonemal heavy c	protein_coding	0	0	1	1	1.026
1770	NM_001372,NM_00	**DNAH9**	dynein axonemal heavy c	protein_coding	0	0	1	1	1.000
1773	NM_001351825,NM	**DNASE1**	deoxyribonuclease 1	protein_coding	8	8	9	9	1.011
1774	NM_001009932,NM	**DNASE1L1**	deoxyribonuclease 1 like	protein_coding	6	6	7	7	0.957
1775	NM_001301680,NM	**DNASE1L2**	deoxyribonuclease 1 like	protein_coding	2	3	3	4	1.178
1776	NM_001256560,NM	**DNASE1L3**	deoxyribonuclease 1 like	protein_coding	0	0	1	1	1.000
1777	NM_001375	**DNASE2**	deoxyribonuclease 2, lys	protein_coding	10	8	11	9	0.844
1778	NM_001376	**DYNC1H1**	dynein cytoplasmic 1 hea	protein_coding	40	34	41	35	0.856
1780	NM_001135556,NM	**DYNC1I1**	dynein cytoplasmic 1 inte	protein_coding	0	0	1	1	0.977
1781	NM_001271785,NM	**DYNC1I2**	dynein cytoplasmic 1 inte	protein_coding	24	26	25	27	1.072
1783	NM_001286157,NM	**DYNC1LI2**	dynein cytoplasmic 1 ligh	protein_coding	46	40	47	41	0.871
1785	NM_001005360,NM	**DNM2**	dynamin 2	protein_coding	147	151	148	152	1.025
1786	NM_001130823,NM	**DNMT1**	DNA methyltransferase 1	protein_coding	207	194	208	195	0.935
1787	NM_001321006,NM	**TRDMT1**	tRNA aspartic acid methy	protein_coding	1	1	2	2	0.776
1788	NM_001320892,NM	**DNMT3A**	DNA methyltransferase 3	protein_coding	2	2	3	3	1.107
1789	NM_001207055,NM	**DNMT3B**	DNA methyltransferase 3	protein_coding	38	33	39	34	0.868
1791	NM_001017520,NM	**DNTT**	DNA nucleotidylexotrans	protein_coding	0	0	1	1	1.000
1793	NM_001290223,NM	**DOCK1**	dedicator of cytokinesis 1	protein_coding	0	0	1	1	1.000
1794	NM_004946,NR_15	**DOCK2**	dedicator of cytokinesis 2	protein_coding	148	136	149	137	0.925
1795	NM_004947,XM_00	**DOCK3**	dedicator of cytokinesis 3	protein_coding	0	0	1	1	1.000
1796	NM_001197260,NM	**DOK1**	docking protein 1	protein_coding	12	12	13	13	1.041
1797	NM_001371205,NM	**DXO**	decapping exoribonuclea	protein_coding	8	9	9	10	1.088
1798	NM_001382,XM_00	**DPAGT1**	dolichyl-phosphate N-ac	protein_coding	60	56	61	57	0.945
1800	NM_001128141,NM	**DPEP1**	dipeptidase 1	protein_coding	0	0	1	1	1.000
1801	NM_001346574,NM	**DPH1**	diphthamide biosynthesi	protein_coding	19	17	20	18	0.877
1802	NM_001039589,NM	**DPH2**	diphthamide biosynthesi	protein_coding	105	98	106	99	0.937
1803	NM_001379604,NM	**DPP4**	dipeptidyl peptidase 4	protein_coding	231	222	232	223	0.961
1804	NM_001039350,NM	**DPP6**	dipeptidyl peptidase like	protein_coding	0	0	1	1	1.000
1805	NM_001937	**DPT**	dermatopontin	protein_coding	0	0	1	1	1.000
1806	NM_000110,NM_00	**DPYD**	dihydropyrimidine dehyd	protein_coding	20	17	21	18	0.847
1807	NM_001385,XM_00	**DPYS**	dihydropyrimidinase	protein_coding	0	0	1	1	1.000
1808	NM_001197293,NM	**DPYSL2**	dihydropyrimidinase like	protein_coding	33	36	34	37	1.079
1809	NM_001197294,NM	**DPYSL3**	dihydropyrimidinase like	protein_coding	0	0	1	1	1.000
1810	NM_001938	**DR1**	down-regulator of transc	protein_coding	52	46	53	47	0.891
1811	NM_000111	**SLC26A3**	solute carrier family 26 m	protein_coding	0	0	1	1	1.000
1812	NM_000794	**DRD1**	dopamine receptor D1	protein_coding	0	0	1	1	1.000
1813	NM_000795,NM_01	**DRD2**	dopamine receptor D2	protein_coding	0	0	1	1	1.035
1814	NM_000796,NM_00	**DRD3**	dopamine receptor D3	protein_coding	0	0	1	1	1.000
1815	NM_000797	**DRD4**	dopamine receptor D4	protein_coding	0	0	1	1	1.301
1816	NM_000798	**DRD5**	dopamine receptor D5	protein_coding	0	0	1	1	1.000
1817	gene-DRD5P1	**DRD5P1**	dopamine receptor D5 ps	pseudogene	0	0	1	1	0.980
1818	NR_111001	**DRD5P2**	dopamine receptor D5 ps	transcribed_pseu	0	0	1	1	0.995
1819	NM_001330144,NM	**DRG2**	developmentally regulate	protein_coding	30	33	31	34	1.097
1820	NM_005224,XM_00	**ARID3A**	AT-rich interaction doma	protein_coding	4	3	5	4	0.770
1821	NM_001171184,NM	**DRP2**	dystrophin related protei	protein_coding	1	1	2	2	1.051
1822	NM_001007026,NM	**ATN1**	atrophin 1	protein_coding	18	17	19	18	0.950
1823	NM_004948,NM_02	**DSC1**	desmocollin 1	protein_coding	0	0	1	1	1.000
1824	NM_004949,NM_02	**DSC2**	desmocollin 2	protein_coding	0	0	1	1	1.000
1825	NM_001941,NM_02	**DSC3**	desmocollin 3	protein_coding	0	0	1	1	1.000
1826	NM_001271534,NM	**DSCAM**	DS cell adhesion molecu	protein_coding	0	0	1	1	1.000
1827	NM_001285389,NM	**RCAN1**	regulator of calcineurin 1	protein_coding	9	7	10	8	0.819
1828	NM_001942	**DSG1**	desmoglein 1	protein_coding	0	0	1	1	1.000
1829	NM_001943,XM_02	**DSG2**	desmoglein 2	protein_coding	0	0	1	1	1.000
1830	NM_001944,XM_01	**DSG3**	desmoglein 3	protein_coding	0	0	1	1	1.000
1831	NM_001015881,NM	**TSC22D3**	TSC22 domain family me	protein_coding	10	12	11	13	1.152
1832	NM_001008844,NM	**DSP**	desmoplakin	protein_coding	0	0	1	1	1.000

1833	NM_004950,XM_01	**EPYC**	epiphycan	protein_coding	0	0	1	1	0.824
1834	NM_014208	**DSPP**	dentin sialophosphoprote	protein_coding	0	0	1	1	1.000
1836	NM_000112,XM_01	**SLC26A2**	solute carrier family 26 m	protein_coding	14	12	15	13	0.894
1837	NM_001128175,NM	**DTNA**	dystrobrevin alpha	protein_coding	0	0	1	1	1.000
1838	NM_001256303,NM	**DTNB**	dystrobrevin beta	protein_coding	7	6	8	7	0.926
1839	NM_001945	**HBEGF**	heparin binding EGF like	protein_coding	6	7	7	8	1.147
1840	NM_004416,XM_01	**DTX1**	deltex E3 ubiquitin ligase	protein_coding	0	0	1	1	0.953
1841	NM_001165031,NM	**DTYMK**	deoxythymidylate kinase	protein_coding	252	268	253	269	1.063
1842	NM_001197295,NM	**ECM2**	extracellular matrix prote	protein_coding	0	0	1	1	1.000
1843	NM_004417	**DUSP1**	dual specificity phosphat	protein_coding	4	4	5	5	0.853
1844	NM_004418,XM_01	**DUSP2**	dual specificity phosphat	protein_coding	34	31	35	32	0.913
1845	NM_004090	**DUSP3**	dual specificity phosphat	protein_coding	0	0	1	1	0.959
1846	NM_001394,NM_05	**DUSP4**	dual specificity phosphat	protein_coding	0	0	1	1	0.878
1847	NM_004419	**DUSP5**	dual specificity phosphat	protein_coding	295	304	296	305	1.029
1848	NM_001946,NM_02	**DUSP6**	dual specificity phosphat	protein_coding	14	11	15	12	0.802
1849	NM_001947	**DUSP7**	dual specificity phosphat	protein_coding	22	22	23	23	1.006
1850	NM_004420,XM_01	**DUSP8**	dual specificity phosphat	protein_coding	0	0	1	1	1.000
1852	NM_001318503,NM	**DUSP9**	dual specificity phosphat	protein_coding	0	0	1	1	1.000
1854	NM_001025248,NM	**DUT**	deoxyuridine triphosphat	protein_coding	484	477	485	478	0.987
1855	NM_001330311,NM	**DVL1**	dishevelled segment pol	protein_coding	31	28	32	29	0.903
1856	NM_004422,XM_00	**DVL2**	dishevelled segment pol	protein_coding	23	25	24	26	1.088
1857	NM_004423,XM_00	**DVL3**	dishevelled segment pol	protein_coding	38	34	39	35	0.897
1859	NM_001347721,NM	**DYRK1A**	dual specificity tyrosine p	protein_coding	23	21	24	22	0.929
1861	NM_000113,XR_92	**TOR1A**	torsin family 1 member A	protein_coding	67	69	68	70	1.017
1869	NM_005225	**E2F1**	E2F transcription factor 1	protein_coding	100	100	101	101	1.004
1870	NM_004091,XM_00	**E2F2**	E2F transcription factor 2	protein_coding	53	51	54	52	0.970
1871	NM_001243076,NM	**E2F3**	E2F transcription factor 3	protein_coding	47	40	48	41	0.854
1872	gene-E2F3P1	**E2F3P1**	E2F transcription factor 3	pseudogene	0	0	1	1	1.354
1874	NM_001950,XR_00	**E2F4**	E2F transcription factor 4	protein_coding	204	226	205	227	1.107
1875	NM_001083588,NM	**E2F5**	E2F transcription factor 5	protein_coding	47	40	48	41	0.862
1876	NM_001278275,NM	**E2F6**	E2F transcription factor 6	protein_coding	29	30	30	31	1.034
1877	NM_001288776,NM	**E4F1**	E4F transcription factor 1	protein_coding	26	28	27	29	1.047
1879	NM_001290360,NM	**EBF1**	EBF transcription factor 1	protein_coding	0	0	1	1	1.000
1880	NM_004951,XM_01	**GPR183**	G protein-coupled recept	protein_coding	0	0	1	1	1.119
1889	NM_001113347,NM	**ECE1**	endothelin converting en	protein_coding	31	28	32	29	0.902
1890	NM_001113755,NM	**TYMP**	thymidine phosphorylase	protein_coding	39	40	40	41	1.015
1891	NM_001398,XM_01	**ECH1**	enoyl-CoA hydratase 1	protein_coding	102	105	103	106	1.022
1892	NM_004092,XR_00	**ECHS1**	enoyl-CoA hydratase, sh	protein_coding	95	110	96	111	1.146
1893	NM_001202858,NM	**ECM1**	extracellular matrix prote	protein_coding	2	1	3	2	0.770
1894	NM_001258315,NM	**ECT2**	epithelial cell transformir	protein_coding	75	62	76	63	0.820
1896	NM_001005609,NM	**EDA**	ectodysplasin A	protein_coding	0	0	1	1	1.000
1901	NM_001320730,NM	**S1PR1**	sphingosine-1-phosphate	protein_coding	14	13	15	14	0.951
1902	NM_001351397,NM	**LPAR1**	lysophosphatidic acid rec	protein_coding	0	0	1	1	1.000
1903	NM_005226	**S1PR3**	sphingosine-1-phosphate	protein_coding	0	0	1	1	1.000
1906	NM_001168319,NM	**EDN1**	endothelin 1	protein_coding	0	0	1	1	1.000
1907	NM_001302269,NM	**EDN2**	endothelin 2	protein_coding	0	0	1	1	1.000
1908	NM_001302455,NM	**EDN3**	endothelin 3	protein_coding	0	0	1	1	0.966
1909	NM_001166055,NM	**EDNRA**	endothelin receptor type	protein_coding	0	0	1	1	1.000
1910	NM_000115,NM_00	**EDNRB**	endothelin receptor type	protein_coding	0	0	1	1	1.000
1911	NM_004426,XM_00	**PHC1**	polyhomeotic homolog 1	protein_coding	6	5	7	6	0.803
1912	NM_001330488,NM	**PHC2**	polyhomeotic homolog 2	protein_coding	42	43	43	44	1.031
1915	NM_001402	**EEF1A1**	eukaryotic translation elo	protein_coding	5,363	5,472	5,364	5,473	1.020
1917	NM_001958	**EEF1A2**	eukaryotic translation elo	protein_coding	0	0	1	1	1.000
1918	gene-EEF1A1P43	**EEF1A1P43**	eukaryotic translation elo	pseudogene	0	0	1	1	1.000
1932	gene-EEF1B2P1	**EEF1B2P1**	eukaryotic translation elo	pseudogene	0	0	1	1	1.000
1933	NM_001037663,NM	**EEF1B2**	eukaryotic translation elo	protein_coding	1,602	1,641	1,603	1,642	1.024
1934	gene-EEF1B2P2	**EEF1B2P2**	eukaryotic translation elo	pseudogene	0	0	1	1	1.017
1936	NM_001130053,NM	**EEF1D**	eukaryotic translation elo	protein_coding	333	337	334	338	1.012
1937	NM_001404	**EEF1G**	eukaryotic translation elo	protein_coding	1,962	1,973	1,963	1,974	1.006
1938	NM_001961	**EEF2**	eukaryotic translation elo	protein_coding	1,954	1,970	1,955	1,971	1.008
1939	NM_001201478,NM	**EIF2D**	eukaryotic translation init	protein_coding	57	60	58	61	1.056
1942	NM_004428,NM_18	**EFNA1**	ephrin A1	protein_coding	0	0	1	1	1.000
1943	NM_001405,XM_01	**EFNA2**	ephrin A2	protein_coding	0	0	1	1	1.000
1944	NM_004952	**EFNA3**	ephrin A3	protein_coding	1	2	2	3	1.213
1945	NM_005227,NM_18	**EFNA4**	ephrin A4	protein_coding	1	1	2	2	0.885
1946	NM_001962,XM_00	**EFNA5**	ephrin A5	protein_coding	1	1	2	2	0.983
1947	NM_004429	**EFNB1**	ephrin B1	protein_coding	5	6	6	7	1.210
1948	NM_001372056,NM	**EFNB2**	ephrin B2	protein_coding	0	0	1	1	1.003
1949	NM_001406	**EFNB3**	ephrin B3	protein_coding	0	0	1	1	1.000
1950	NM_001178130,NM	**EGF**	epidermal growth factor	protein_coding	0	0	1	1	1.000
1951	NM_001407	**CELSR3**	cadherin EGF LAG sever	protein_coding	14	13	15	14	0.955
1952	NM_001408	**CELSR2**	cadherin EGF LAG sever	protein_coding	0	0	1	1	1.000
1953	NM_001409,XM_00	**MEGF6**	multiple EGF like domair	protein_coding	0	0	1	1	1.006
1954	NM_001271938,NM	**MEGF8**	multiple EGF like domair	protein_coding	8	7	9	8	0.891
1955	NM_001080497	**MEGF9**	multiple EGF like domair	protein_coding	6	5	7	6	0.916
1956	NM_001346897,NM	**EGFR**	epidermal growth factor r	protein_coding	0	0	1	1	1.000
1958	NM_001964	**EGR1**	early growth response 1	protein_coding	4	3	5	4	0.888
1959	NM_000399,NM_00	**EGR2**	early growth response 2	protein_coding	3	2	4	3	0.811
1960	NM_001199880,NM	**EGR3**	early growth response 3	protein_coding	0	0	1	1	1.000
1961	NM_001965	**EGR4**	early growth response 4	protein_coding	1	1	2	2	1.213
1962	NM_001166415,NM	**EHHADH**	enoyl-CoA hydratase and	protein_coding	7	7	8	8	0.946
1964	NM_001412	**EIF1AX**	eukaryotic translation init	protein_coding	147	137	148	138	0.936
1965	NM_004094	**EIF2S1**	eukaryotic translation init	protein_coding	117	111	118	112	0.946
1967	NM_001414	**EIF2B1**	eukaryotic translation init	protein_coding	99	99	100	100	1.006
1968	NM_001415	**EIF2S3**	eukaryotic translation init	protein_coding	290	267	291	268	0.922
1969	NM_001329090,NM	**EPHA2**	EPH receptor A2	protein_coding	0	0	1	1	0.976
1973	NM_001204510,NM	**EIF4A1**	eukaryotic translation init	protein_coding	975	993	976	994	1.018
1974	NM_001967	**EIF4A2**	eukaryotic translation init	protein_coding	515	528	516	529	1.026
1975	NM_001300821,NM	**EIF4B**	eukaryotic translation init	protein_coding	230	205	231	206	0.894
1977	NM_001130678,NM	**EIF4E**	eukaryotic translation init	protein_coding	30	27	31	28	0.906
1978	NM_004095	**EIF4EBP1**	eukaryotic translation init	protein_coding	29	22	30	23	0.770
1979	NM_004096	**EIF4EBP2**	eukaryotic translation init	protein_coding	63	58	64	59	0.915
1980	gene-EIF4EP1	**EIF4EP1**	eukaryotic translation init	pseudogene	0	0	1	1	1.000
1981	NM_001194946,NM	**EIF4G1**	eukaryotic translation init	protein_coding	523	493	524	494	0.944
1982	NM_001042559,NM	**EIF4G2**	eukaryotic translation init	protein_coding	901	838	902	839	0.930
1983	NM_001969,NM_18	**EIF5**	eukaryotic translation init	protein_coding	126	117	127	118	0.934
1984	NM_001143760,NM	**EIF5A**	eukaryotic translation init	protein_coding	832	911	833	912	1.096
1987	gene-EIF5AP3	**EIF5AP3**	eukaryotic translation init	pseudogene	0	0	1	1	1.122
1990	NM_001971	**CELA1**	chymotrypsin like elastas	protein_coding	0	0	1	1	1.000
1991	NM_001972,XM_01	**ELANE**	elastase, neutrophil expr	protein_coding	0	0	1	1	1.000
1992	NM_030666,NR_07	**SERPINB1**	serpin family B member	protein_coding	127	131	128	132	1.032
1993	NM_001171195,NM	**ELAVL2**	ELAV like RNA binding p	protein_coding	0	0	1	1	1.000
1994	NM_001419,XR_00	**ELAVL1**	ELAV like RNA binding p	protein_coding	78	74	79	75	0.946
1995	NM_001420,NM_03	**ELAVL3**	ELAV like RNA binding p	protein_coding	0	0	1	1	1.000
1996	NM_001144774,NM	**ELAVL4**	ELAV like RNA binding p	protein_coding	0	0	1	1	1.000
1997	NM_001145353,NM	**ELF1**	E74 like ETS transcriptio	protein_coding	74	64	75	65	0.864
1998	NM_001276457,NM	**ELF2**	E74 like ETS transcriptio	protein_coding	19	17	20	18	0.924
1999	NM_001114309,NM	**ELF3**	E74 like ETS transcriptio	protein_coding	0	0	1	1	1.000
2000	NM_001127197,NM	**ELF4**	E74 like ETS transcriptio	protein_coding	84	87	85	88	1.027
2001	NM_001243080,NM	**ELF5**	E74 like ETS transcriptio	protein_coding	0	0	1	1	1.000
2002	NM_001114123,NM	**ELK1**	ETS transcription factor E	protein_coding	53	54	54	55	1.014
2003	NR_046211	**ELK2AP**	ETS transcription factor E	transcribed_pseu	0	0	1	1	1.000
2004	NM_001303511,NM	**ELK3**	ETS transcription factor E	protein_coding	5	5	6	6	0.913
2005	NM_001973,NM_02	**ELK4**	ETS transcription factor E	protein_coding	24	18	25	19	0.756
2006	NM_000501,NM_00	**ELN**	elastin	protein_coding	0	0	1	1	1.079
2009	NM_001008707,NM	**EML1**	EMAP like 1	protein_coding	0	0	1	1	1.000
2010	NM_000117,XM_02	**EMD**	emerin	protein_coding	138	154	139	155	1.112
2011	NM_001039469,NM	**MARK2**	microtubule affinity regul	protein_coding	56	39	57	40	0.696
2012	NM_001423,XM_02	**EMP1**	epithelial membrane prot	protein_coding	1	1	2	2	0.978
2013	NM_001424,XM_00	**EMP2**	epithelial membrane prot	protein_coding	0	0	1	1	1.000
2014	NM_001313905,NM	**EMP3**	epithelial membrane prot	protein_coding	166	175	167	176	1.051
2015	NM_001256252,NM	**ADGRE1**	adhesion G protein-coup	protein_coding	0	0	1	1	1.000
2016	NM_004097,XM_00	**EMX1**	empty spiracles homeob	protein_coding	0	0	1	1	1.000
2017	NM_001184740,NM	**CTTN**	cortactin	protein_coding	0	0	1	1	1.000
2018	NM_001165924,NM	**EMX2**	empty spiracles homeob	protein_coding	0	0	1	1	1.000
2019	NM_001426	**EN1**	engrailed homeobox 1	protein_coding	0	0	1	1	1.000

2020	NM_001427	**EN2**	engrailed homeobox 2	protein_coding	0	0	1	1	1.000
2021	NM_004435,XM_01	**ENDOG**	endonuclease G	protein_coding	3	4	4	5	1.174
2022	NM_000118,NM_00	**ENG**	endoglin	protein_coding	0	0	1	1	1.000
2023	NM_001201483,NM	**ENO1**	enolase 1	protein_coding	4,083	4,284	4,084	4,285	1.049
2025	gene-ENO1P1	**ENO1P1**	enolase 1 pseudogene 1	pseudogene	0	0	1	1	1.000
2026	NM_001975	**ENO2**	enolase 2	protein_coding	0	0	1	1	1.000
2027	NM_001193503,NM	**ENO3**	enolase 3	protein_coding	6	4	7	5	0.762
2028	NM_001379611,NM	**ENPEP**	glutamyl aminopeptidase	protein_coding	26	25	27	26	0.979
2029	NM_004436,NM_20	**ENSA**	endosulfine alpha	protein_coding	392	415	393	416	1.059
2030	NM_001078175,NM	**SLC29A1**	solute carrier family 29 m	protein_coding	126	135	127	136	1.067
2033	NM_001362843,NM	**EP300**	E1A binding protein p300	protein_coding	39	32	40	33	0.826
2034	NM_001430,XM_01	**EPAS1**	endothelial PAS domain	protein_coding	0	0	1	1	1.000
2035	NM_001166005,NM	**EPB41**	erythrocyte membrane p	protein_coding	93	86	94	87	0.924
2036	NM_001258329,NM	**EPB41L1**	erythrocyte membrane p	protein_coding	0	0	1	1	1.099
2037	NM_001135554,NM	**EPB41L2**	erythrocyte membrane p	protein_coding	0	0	1	1	1.000
2038	NM_000119,NM_00	**EPB42**	erythrocyte membrane p	protein_coding	0	0	1	1	1.000
2039	NM_001114135,NM	**DMTN**	dematin actin binding pro	protein_coding	0	0	1	1	1.000
2040	NM_001270526,NM	**STOM**	stomatin	protein_coding	163	148	164	149	0.908
2041	NM_005232,XM_00	**EPHA1**	EPH receptor A1	protein_coding	13	3	14	4	0.317
2042	NM_005233,NM_18	**EPHA3**	EPH receptor A3	protein_coding	0	0	1	1	1.000
2043	NM_001304536,NM	**EPHA4**	EPH receptor A4	protein_coding	0	0	1	1	0.961
2044	NM_001281765,NM	**EPHA5**	EPH receptor A5	protein_coding	0	0	1	1	1.000
2045	NM_001288629,NM	**EPHA7**	EPH receptor A7	protein_coding	0	0	1	1	1.000
2046	NM_001006943,NM	**EPHA8**	EPH receptor A8	protein_coding	0	0	1	1	1.000
2047	NM_004441,XM_01	**EPHB1**	EPH receptor B1	protein_coding	0	0	1	1	1.054
2048	NM_001309192,NM	**EPHB2**	EPH receptor B2	protein_coding	0	0	1	1	1.009
2049	NM_004443	**EPHB3**	EPH receptor B3	protein_coding	0	0	1	1	1.000
2050	NM_004444,XM_01	**EPHB4**	EPH receptor B4	protein_coding	0	1	1	2	1.152
2051	NM_001280794,NM	**EPHB6**	EPH receptor B6	protein_coding	0	0	1	1	1.000
2052	NM_000120,NM_00	**EPHX1**	epoxide hydrolase 1	protein_coding	0	0	1	1	0.873
2053	NM_001256482,NM	**EPHX2**	epoxide hydrolase 2	protein_coding	0	0	1	1	1.038
2054	NM_001351049,NM	**STX2**	syntaxin 2	protein_coding	3	3	4	4	0.954
2055	NM_018941,XM_00	**CLN8**	CLN8 transmembrane El	protein_coding	8	6	9	7	0.809
2056	NM_000799	**EPO**	erythropoietin	protein_coding	0	0	1	1	1.000
2057	NM_000121,NR_03	**EPOR**	erythropoietin receptor	protein_coding	3	3	4	4	0.870
2058	NM_004446,XM_01	**EPRS1**	glutamyl-prolyl-tRNA syn	protein_coding	144	131	145	132	0.914
2059	NM_004447,XM_01	**EPS8**	epidermal growth factor r	protein_coding	0	0	1	1	1.049
2060	NM_001159969,NM	**EPS15**	epidermal growth factor r	protein_coding	38	35	39	36	0.913
2063	NM_005234	**NR2F6**	nuclear receptor subfam	protein_coding	2	2	3	3	1.077
2064	NM_001005862,NM	**ERBB2**	erb-b2 receptor tyrosine	protein_coding	2	3	3	4	1.370
2065	NM_001005915,NM	**ERBB3**	erb-b2 receptor tyrosine	protein_coding	0	0	1	1	0.952
2066	NM_001042599,NM	**ERBB4**	erb-b2 receptor tyrosine	protein_coding	0	0	1	1	1.000
2067	NM_001166049,NM	**ERCC1**	ERCC excision repair 1,	protein_coding	49	53	50	54	1.080
2068	NM_000400,NM_00	**ERCC2**	ERCC excision repair 2,	protein_coding	8	7	9	8	0.911
2069	NM_001432	**EREG**	epiregulin	protein_coding	0	0	1	1	1.000
2070	NM_001301012,NM	**EYA4**	EYA transcriptional coac	protein_coding	0	0	1	1	0.976
2071	NM_000122,NM_00	**ERCC3**	ERCC excision repair 3,	protein_coding	70	63	71	64	0.909
2072	NM_005236,XM_01	**ERCC4**	ERCC excision repair 4,	protein_coding	4	3	5	4	0.871
2073	NM_000123	**ERCC5**	ERCC excision repair 5,	protein_coding	34	30	35	31	0.902
2074	NM_000124,NM_00	**ERCC6**	ERCC excision repair 6,	protein_coding	3	3	4	4	0.964
2077	NM_001301035,NM	**ERF**	ETS2 repressor factor	protein_coding	37	39	38	40	1.052
2078	NM_001136154,NM	**ERG**	ETS transcription factor	protein_coding	0	0	1	1	1.000
2079	NM_004450	**ERH**	ERH mRNA splicing and	protein_coding	614	638	615	639	1.039
2081	NM_001433,XM_01	**ERN1**	endoplasmic reticulum to	protein_coding	2	1	3	2	0.901
2086	NM_001007253,NR	**ERV3-1**	endogenous retrovirus gr	protein_coding	9	9	10	10	1.026
2091	NM_001436,XM_00	**FBL**	fibrillarin	protein_coding	718	740	719	741	1.030
2098	NM_001984,XM_00	**ESD**	esterase D	protein_coding	0	0	1	1	1.000
2099	NM_000125,NM_00	**ESR1**	estrogen receptor 1	protein_coding	0	0	1	1	1.064
2100	NM_001040275,NM	**ESR2**	estrogen receptor 2	protein_coding	1	1	2	2	0.924
2101	NM_001282450,NM	**ESRRA**	estrogen related recepto	protein_coding	68	68	69	69	1.012
2103	NM_001379180,NM	**ESRRB**	estrogen related recepto	protein_coding	0	0	1	1	1.001
2104	NM_001134285,NM	**ESRRG**	estrogen related recepto	protein_coding	0	0	1	1	1.000
2107	NM_001256302,NM	**ETF1**	eukaryotic translation ter	protein_coding	237	227	238	228	0.956
2108	NM_000126,NM_00	**ETFA**	electron transfer flavopro	protein_coding	188	185	189	186	0.984
2109	NM_001014763,NM	**ETFB**	electron transfer flavopro	protein_coding	155	182	156	183	1.178
2110	NM_001281737,NM	**ETFDH**	electron transfer flavopro	protein_coding	11	8	12	9	0.769
2113	NM_001143820,NM	**ETS1**	ETS proto-oncogene 1, t	protein_coding	284	278	285	279	0.978
2114	NM_001256295,NM	**ETS2**	ETS proto-oncogene 2, t	protein_coding	26	22	27	23	0.837
2115	NM_001163147,NM	**ETV1**	ETS variant transcription	protein_coding	1	1	2	2	0.817
2116	NM_001300974,NM	**ETV2**	ETS variant transcription	protein_coding	0	0	1	1	1.005
2117	NM_001145312,NM	**ETV3**	ETS variant transcription	protein_coding	20	19	21	20	0.942
2118	NM_001079675,NM	**ETV4**	ETS variant transcription	protein_coding	0	0	1	1	1.000
2119	NM_004454	**ETV5**	ETS variant transcription	protein_coding	8	7	9	8	0.945
2120	NM_001987,XM_01	**ETV6**	ETS variant transcription	protein_coding	5	4	6	5	0.938
2121	NM_001306090,NM	**EVC**	EvC ciliary complex subu	protein_coding	0	0	1	1	1.000
2122	NM_001105077,NM	**MECOM**	MDS1 and EVI1 complex	protein_coding	0	0	1	1	1.000
2123	NM_001003927,NM	**EVI2A**	ecotropic viral integration	protein_coding	21	18	22	19	0.858
2124	NM_006495,XM_00	**EVI2B**	ecotropic viral integration	protein_coding	88	79	89	80	0.901
2125	NM_001320747,NM	**EVPL**	envoplakin	protein_coding	0	0	1	1	1.015
2128	NM_001304519,NM	**EVX1**	even-skipped homeobox	protein_coding	0	0	1	1	1.000
2130	NM_001163285,NM	**EWSR1**	EWS RNA binding protei	protein_coding	429	441	430	442	1.028
2131	NM_000127	**EXT1**	exostosin glycosyltransfe	protein_coding	2	2	3	3	1.071
2132	NM_000401,NM_00	**EXT2**	exostosin glycosyltransfe	protein_coding	17	16	18	17	0.959
2134	NM_004455,XM_00	**EXTL1**	exostosin like glycosyltra	protein_coding	0	0	1	1	1.025
2135	NM_001033025,NM	**EXTL2**	exostosin like glycosyltra	protein_coding	12	10	13	11	0.820
2137	NM_001440,NR_07	**EXTL3**	exostosin like glycosyltra	protein_coding	5	4	6	5	0.811
2138	NM_000503,NM_00	**EYA1**	EYA transcriptional coac	protein_coding	0	0	1	1	1.000
2139	NM_005244,NM_17	**EYA2**	EYA transcriptional coac	protein_coding	0	0	1	1	1.000
2140	NM_001282560,NM	**EYA3**	EYA transcriptional coac	protein_coding	55	49	56	50	0.888
2145	NM_001321079,NM	**EZH1**	enhancer of zeste 1 poly	protein_coding	10	9	11	10	0.881
2146	NM_001203247,NM	**EZH2**	enhancer of zeste 2 poly	protein_coding	103	100	104	101	0.977
2147	NM_000506	**F2**	coagulation factor II, thro	protein_coding	0	0	1	1	1.050
2149	NM_001311313,NM	**F2R**	coagulation factor II throm	protein_coding	86	79	87	80	0.920
2150	NM_005242,XM_01	**F2RL1**	F2R like trypsin receptor	protein_coding	0	0	1	1	0.928
2151	NM_001256566,NM	**F2RL2**	coagulation factor II throm	protein_coding	0	0	1	1	1.000
2152	NM_001178096,NM	**F3**	coagulation factor III, tiss	protein_coding	0	0	1	1	1.000
2153	NM_000130,XM_01	**F5**	coagulation factor V	protein_coding	0	0	1	1	1.000
2155	NM_000131,NM_00	**F7**	coagulation factor VII	protein_coding	0	0	1	1	1.000
2157	NM_000132,NM_01	**F8**	coagulation factor VIII	protein_coding	2	1	3	2	0.597
2158	NM_000133,NM_00	**F9**	coagulation factor IX	protein_coding	0	0	1	1	1.000
2159	NM_000504,NM_00	**F10**	coagulation factor X	protein_coding	0	0	1	1	1.000
2160	NM_000128,NM_00	**F11**	coagulation factor XI	protein_coding	0	0	1	1	1.023
2161	NM_000505,XM_01	**F12**	coagulation factor XII	protein_coding	0	0	1	1	1.000
2162	NM_000129	**F13A1**	coagulation factor XIII A	protein_coding	0	0	1	1	1.000
2165	NM_001994,XM_01	**F13B**	coagulation factor XIII B	protein_coding	0	0	1	1	0.992
2166	NM_001441	**FAAH**	fatty acid amide hydrolas	protein_coding	1	1	2	2	0.918
2167	NM_001442	**FABP4**	fatty acid binding protein	protein_coding	0	0	1	1	1.000
2168	NM_001443	**FABP1**	fatty acid binding protein	protein_coding	0	0	1	1	1.000
2169	NM_000134	**FABP2**	fatty acid binding protein	protein_coding	0	0	1	1	1.000
2170	NM_001320996,NM	**FABP3**	fatty acid binding protein	protein_coding	0	0	1	1	1.000
2171	NM_001444	**FABP5**	fatty acid binding protein	protein_coding	661	663	662	664	1.002
2172	NM_001040442,NM	**FABP6**	fatty acid binding protein	protein_coding	0	0	1	1	1.000
2173	NM_001319039,NM	**FABP7**	fatty acid binding protein	protein_coding	0	0	1	1	1.000
2175	NM_000135,NM_00	**FANCA**	FA complementation gro	protein_coding	26	29	27	30	1.092
2176	NM_000136,NM_00	**FANCC**	FA complementation gro	protein_coding	15	14	16	15	0.954
2177	NM_001018115,NM	**FANCD2**	FA complementation gro	protein_coding	30	29	31	30	0.951
2178	NM_021922,XM_00	**FANCE**	FA complementation gro	protein_coding	20	23	21	24	1.125
2180	NM_001286708,NM	**ACSL1**	acyl-CoA synthetase lon	protein_coding	88	70	89	71	0.804
2181	NM_001354158,NM	**ACSL3**	acyl-CoA synthetase lon	protein_coding	55	51	56	52	0.928
2182	NM_001318509,NM	**ACSL4**	acyl-CoA synthetase lon	protein_coding	69	60	70	61	0.866
2184	NM_000137,NM_00	**FAH**	fumarylacetoacetate hyd	protein_coding	147	145	148	146	0.984
2185	NM_004103,NM_17	**PTK2B**	protein tyrosine kinase 2	protein_coding	102	103	103	104	1.013
2186	NM_004459,NM_18	**BPTF**	bromodomain PHD finge	protein_coding	25	21	26	22	0.850
2187	NM_001018113,NM	**FANCB**	FA complementation gro	protein_coding	13	8	14	9	0.685
2188	NM_022725	**FANCF**	FA complementation gro	protein_coding	31	29	32	30	0.941
2189	NM_004629	**FANCG**	FA complementation gro	protein_coding	29	29	30	30	1.014

2191	NM_001291807,NM	**FAP**	fibroblast activation prote	protein_coding	0	0	1	1	0.875
2192	NM_001996,NM_00	**FBLN1**	fibulin 1	protein_coding	0	0	1	1	1.000
2193	NM_004461	**FARSA**	phenylalanyl-tRNA synth	protein_coding	232	247	233	248	1.067
2194	NM_004104,XM_01	**FASN**	fatty acid synthase	protein_coding	117	119	118	120	1.018
2195	NM_005245,XM_00	**FAT1**	FAT atypical cadherin 1	protein_coding	0	0	1	1	1.000
2196	NM_001447,XM_00	**FAT2**	FAT atypical cadherin 2	protein_coding	0	0	1	1	1.013
2197	NM_001997	**FAU**	FAU ubiquitin like and rib	protein_coding	1,444	1,521	1,445	1,522	1.054
2199	NM_001004019,NM	**FBLN2**	fibulin 2	protein_coding	0	0	1	1	1.000
2200	NM_000138	**FBN1**	fibrillin 1	protein_coding	1	1	2	2	0.847
2201	NM_001999,XM_01	**FBN2**	fibrillin 2	protein_coding	0	0	1	1	1.000
2202	NM_001039348,NM	**EFEMP1**	EGF containing fibulin ex	protein_coding	0	0	1	1	1.000
2203	NM_000507,NM_00	**FBP1**	fructose-bisphosphatase	protein_coding	0	0	1	1	1.000
2204	NM_002000,NM_13	**FCAR**	Fc fragment of IgA recep	protein_coding	0	0	1	1	0.910
2205	NM_002001	**FCER1A**	Fc fragment of IgE recep	protein_coding	0	0	1	1	1.000
2206	NM_000139,NM_00	**MS4A2**	membrane spanning 4-d	protein_coding	0	0	1	1	1.000
2207	NM_004106	**FCER1G**	Fc fragment of IgE recep	protein_coding	8	7	9	8	0.918
2208	NM_001207019,NM	**FCER2**	Fc fragment of IgE recep	protein_coding	0	0	1	1	1.000
2209	NM_000566,NM_00	**FCGR1A**	Fc fragment of IgG recep	protein_coding	0	0	1	1	0.911
2210	NR_045213,NR_16	**FCGR1B**	Fc fragment of IgG recep	transcribed_pseu	0	0	1	1	1.000
2212	NM_001136219,NM	**FCGR2A**	Fc fragment of IgG recep	protein_coding	0	0	1	1	1.000
2213	NM_001002273,NM	**FCGR2B**	Fc fragment of IgG recep	protein_coding	0	0	1	1	1.080
2214	NM_000569,NM_00	**FCGR3A**	Fc fragment of IgG recep	protein_coding	0	0	1	1	0.966
2215	NM_000570,NM_00	**FCGR3B**	Fc fragment of IgG recep	protein_coding	0	0	1	1	0.969
2216	gene-FDX1P2	**FDX1P2**	ferredoxin 1 pseudogene	pseudogene	0	0	1	1	1.000
2217	NM_001136019,NM	**FCGRT**	Fc fragment of IgG recep	protein_coding	0	0	1	1	1.000
2218	NM_001079802,NM	**FKTN**	fukutin	protein_coding	11	10	12	11	0.867
2219	NM_002003	**FCN1**	ficolin 1	protein_coding	0	0	1	1	1.000
2220	NM_004108,NM_01	**FCN2**	ficolin 2	protein_coding	0	0	1	1	1.000
2222	NM_001287742,NM	**FDFT1**	farnesyl-diphosphate far	protein_coding	117	110	118	111	0.935
2224	NM_001135821,NM	**FDPS**	farnesyl diphosphate syr	protein_coding	323	329	324	330	1.020
2227	gene-FDPSP3	**FDPSP3**	farnesyl diphosphate syr	pseudogene	0	0	1	1	1.195
2228	gene-FDPSP4	**FDPSP4**	farnesyl diphosphate syr	pseudogene	0	0	1	1	0.926
2229	gene-FDPSP5	**FDPSP5**	farnesyl diphosphate syr	pseudogene	0	0	1	1	1.000
2230	NM_004109	**FDX1**	ferredoxin 1	protein_coding	25	23	26	24	0.918
2231	gene-FDX1P1	**FDX1P1**	ferredoxin 1 pseudogene	pseudogene	0	0	1	1	1.032
2232	NM_001258012,NM	**FDXR**	ferredoxin reductase	protein_coding	11	11	12	12	0.927
2235	NM_000140,NM_00	**FECH**	ferrochelatase	protein_coding	9	8	10	9	0.894
2236	gene-FECHP1	**FECHP1**	ferrochelatase pseudoge	pseudogene	0	0	1	1	1.000
2237	NM_004111	**FEN1**	flap structure-specific en	protein_coding	281	294	282	295	1.047
2239	NM_001448	**GPC4**	glypican 4	protein_coding	0	0	1	1	1.000
2241	NM_001308028,NM	**FER**	FER tyrosine kinase	protein_coding	14	12	15	13	0.901
2242	NM_001143783,NM	**FES**	FES proto-oncogene, tyr	protein_coding	0	0	1	1	1.104
2243	NM_000508,NM_02	**FGA**	fibrinogen alpha chain	protein_coding	0	0	1	1	1.000
2244	NM_001184741,NM	**FGB**	fibrinogen beta chain	protein_coding	0	0	1	1	1.000
2245	NM_004463	**FGD1**	FYVE, RhoGEF and PH	protein_coding	0	0	1	1	0.999
2246	NM_000800,NM_00	**FGF1**	fibroblast growth factor 1	protein_coding	0	0	1	1	1.000
2247	NM_001361665,NM	**FGF2**	fibroblast growth factor 2	protein_coding	0	0	1	1	1.000
2248	NM_005247	**FGF3**	fibroblast growth factor 3	protein_coding	0	0	1	1	1.000
2249	NM_002007	**FGF4**	fibroblast growth factor 4	protein_coding	0	0	1	1	1.000
2250	NM_001291812,NM	**FGF5**	fibroblast growth factor 5	protein_coding	0	0	1	1	1.000
2251	NM_020996,XM_01	**FGF6**	fibroblast growth factor 6	protein_coding	0	0	1	1	1.000
2252	NM_002009	**FGF7**	fibroblast growth factor 7	protein_coding	0	0	1	1	0.904
2253	NM_001206389,NM	**FGF8**	fibroblast growth factor 8	protein_coding	0	0	1	1	1.000
2254	NM_002010,XM_01	**FGF9**	fibroblast growth factor 9	protein_coding	0	0	1	1	0.997
2255	NM_004465,XM_00	**FGF10**	fibroblast growth factor 1	protein_coding	0	0	1	1	1.000
2256	NM_001303460,NM	**FGF11**	fibroblast growth factor 1	protein_coding	0	0	1	1	0.971
2257	NM_001377292,NM	**FGF12**	fibroblast growth factor 1	protein_coding	0	0	1	1	1.000
2258	NM_001139498,NM	**FGF13**	fibroblast growth factor 1	protein_coding	0	0	1	1	1.000
2259	NM_001321931,NM	**FGF14**	fibroblast growth factor 1	protein_coding	0	0	1	1	0.988
2260	NM_001174063,NM	**FGFR1**	fibroblast growth factor r	protein_coding	0	0	1	1	1.000
2261	NM_000142,NM_00	**FGFR3**	fibroblast growth factor r	protein_coding	0	0	1	1	1.000
2262	NM_004466,XM_01	**GPC5**	glypican 5	protein_coding	0	0	1	1	1.000
2263	NM_000141,NM_00	**FGFR2**	fibroblast growth factor r	protein_coding	0	0	1	1	1.000
2264	NM_001291980,NM	**FGFR4**	fibroblast growth factor r	protein_coding	0	0	1	1	1.000
2266	NM_000509,NM_02	**FGG**	fibrinogen gamma chain	protein_coding	0	0	1	1	1.000
2267	NM_004467,NM_14	**FGL1**	fibrinogen like 1	protein_coding	0	0	1	1	1.000
2268	NM_001042729,NM	**FGR**	FGR proto-oncogene, Sr	protein_coding	205	200	206	201	0.978
2271	NM_000143,XM_01	**FH**	fumarate hydratase	protein_coding	144	141	145	142	0.980
2272	NM_001166243,NM	**FHIT**	fragile histidine triad diac	protein_coding	0	0	1	1	1.035
2273	NM_001159699,NM	**FHL1**	four and a half LIM doma	protein_coding	0	0	1	1	1.000
2274	NM_001039492,NM	**FHL2**	four and a half LIM doma	protein_coding	0	0	1	1	1.068
2275	NM_001243878,NM	**FHL3**	four and a half LIM doma	protein_coding	0	0	1	1	1.108
2277	NM_004469	**VEGFD**	vascular endothelial grov	protein_coding	0	0	1	1	1.000
2280	NM_000801,NM_00	**FKBP1A**	FKBP prolyl isomerase 1	protein_coding	661	680	662	681	1.029
2281	NM_001322963,NM	**FKBP1B**	FKBP prolyl isomerase 1	protein_coding	0	0	1	1	1.000
2282	NR_024162	**FKBP1AP1**	FKBP prolyl isomerase 1	transcribed_pseu	0	0	1	1	1.000
2283	gene-FKBP1AP2	**FKBP1AP2**	FKBP prolyl isomerase 1	pseudogene	0	0	1	1	1.000
2284	gene-FKBP1AP3	**FKBP1AP3**	FKBP prolyl isomerase 1	pseudogene	0	0	1	1	1.000
2285	gene-FKBP1AP4	**FKBP1AP4**	FKBP prolyl isomerase 1	pseudogene	0	0	1	1	1.002
2286	NM_001135208,NM	**FKBP2**	FKBP prolyl isomerase 2	protein_coding	105	95	106	96	0.910
2287	NM_002013,XM_01	**FKBP3**	FKBP prolyl isomerase 3	protein_coding	120	117	121	118	0.978
2288	NM_002014,XM_01	**FKBP4**	FKBP prolyl isomerase 4	protein_coding	114	115	115	116	1.007
2289	NM_001145775,NM	**FKBP5**	FKBP prolyl isomerase 5	protein_coding	202	186	203	187	0.923
2290	NM_005249	**FOXG1**	forkhead box G1	protein_coding	0	0	1	1	1.000
2294	NM_001451	**FOXF1**	forkhead box F1	protein_coding	0	0	1	1	1.000
2295	NM_001452	**FOXF2**	forkhead box F2	protein_coding	0	0	1	1	1.000
2296	NM_001453	**FOXC1**	forkhead box C1	protein_coding	0	0	1	1	1.000
2297	NM_004472	**FOXD1**	forkhead box D1	protein_coding	3	3	4	4	1.031
2298	NM_207305	**FOXD4**	forkhead box D4	protein_coding	3	3	4	4	1.175
2299	NM_012188,NM_14	**FOXI1**	forkhead box I1	protein_coding	0	0	1	1	0.924
2300	NM_005250	**FOXL1**	forkhead box L1	protein_coding	0	0	1	1	1.000
2301	NM_012186	**FOXE3**	forkhead box E3	protein_coding	0	0	1	1	0.982
2302	NM_001454	**FOXJ1**	forkhead box J1	protein_coding	2	1	3	2	0.818
2303	NM_005251	**FOXC2**	forkhead box C2	protein_coding	0	0	1	1	1.000
2304	NM_004473	**FOXE1**	forkhead box E1	protein_coding	0	0	1	1	1.000
2305	NM_001243088,NM	**FOXM1**	forkhead box M1	protein_coding	67	67	68	68	0.996
2306	NM_004474	**FOXD2**	forkhead box D2	protein_coding	0	0	1	1	0.962
2307	NM_004118	**FOXS1**	forkhead box S1	protein_coding	0	0	1	1	1.060
2308	NM_002015,XM_01	**FOXO1**	forkhead box O1	protein_coding	0	1	1	2	1.209
2309	NM_001455,NM_20	**FOXO3**	forkhead box O3	protein_coding	16	14	17	15	0.838
2310	NM_001368134,NM	**FOXO3B**	forkhead box O3B	protein_coding	3	3	4	4	1.027
2311	gene-FOXO1B	**FOXO1B**	forkhead box O1B (pseu	pseudogene	0	0	1	1	1.000
2312	NM_002016	**FLG**	filaggrin	protein_coding	0	0	1	1	1.000
2313	NM_001167681,NM	**FLI1**	Fli-1 proto-oncogene, ET	protein_coding	54	52	55	53	0.952
2314	NM_001256264,NM	**FLII**	FLII actin remodeling prc	protein_coding	36	34	37	35	0.961
2315	NM_005511	**MLANA**	melan-A	protein_coding	0	0	1	1	1.000
2316	NM_001110556,NM	**FLNA**	filamin A	protein_coding	608	611	609	612	1.004
2317	NM_001164317,NM	**FLNB**	filamin B	protein_coding	9	8	10	9	0.929
2318	NM_001127487,NM	**FLNC**	filamin C	protein_coding	0	0	1	1	1.024
2319	NM_001330170,NM	**FLOT2**	flotillin 2	protein_coding	87	92	88	93	1.052
2321	NM_001159920,NM	**FLT1**	fms related receptor tyro	protein_coding	0	0	1	1	1.000
2322	NM_004119,NR_13	**FLT3**	fms related receptor tyro	protein_coding	0	0	1	1	1.000
2323	NM_001204502,NM	**FLT3LG**	fms related receptor tyro	protein_coding	87	93	88	94	1.069
2324	NM_001354989,NM	**FLT4**	fms related receptor tyro	protein_coding	0	0	1	1	1.000
2326	NM_001282692,NM	**FMO1**	flavin containing dimethy	protein_coding	0	0	1	1	1.000
2327	NM_001301347,NM	**FMO2**	flavin containing dimethy	protein_coding	0	0	1	1	1.000
2328	NM_001002294,NM	**FMO3**	flavin containing dimethy	protein_coding	0	0	1	1	1.000
2329	NM_002022,XM_00	**FMO4**	flavin containing dimethy	protein_coding	2	2	3	3	0.972
2330	NM_001144829,NM	**FMO5**	flavin containing dimethy	protein_coding	0	0	1	1	1.000
2331	NM_002023,NR_10	**FMOD**	fibromodulin	protein_coding	0	0	1	1	1.000
2332	NM_001185075,NM	**FMR1**	FMRP translational regul	protein_coding	47	41	48	42	0.880
2334	NM_001169122,NM	**AFF2**	AF4/FMR2 family membe	protein_coding	0	0	1	1	0.993
2335	NM_001306129,NM	**FN1**	fibronectin 1	protein_coding	33	29	34	30	0.886
2339	NM_002027,NR_03	**FNTA**	farnesyltransferase, CAA	protein_coding	112	111	113	112	0.985
2341	gene-FNTAP2	**FNTAP2**	farnesyltransferase, CAA	pseudogene	0	0	1	1	1.000
2342	NM_002028	**FNTB**	farnesyltransferase, CAA	protein_coding	7	7	8	8	1.008

2346	NM_001014986,NM	**FOLH1**	folate hydrolase 1	protein_coding	0	0	1	1	1.000
2348	NM_000802,NM_01	**FOLR1**	folate receptor alpha	protein_coding	0	0	1	1	1.000
2350	NM_000803,NM_00	**FOLR2**	folate receptor beta	protein_coding	0	0	1	1	1.000
2352	NM_000804,NM_00	**FOLR3**	folate receptor gamma	protein_coding	0	0	1	1	1.000
2353	NM_005252	**FOS**	Fos proto-oncogene, AP	protein_coding	3	3	4	4	0.970
2354	NM_001114171,NM	**FOSB**	FosB proto-oncogene, A	protein_coding	1	1	2	2	1.060
2355	NM_005253,XM_00	**FOSL2**	FOS like 2, AP-1 transcri	protein_coding	45	39	46	40	0.874
2356	NM_001018078,NM	**FPGS**	folylpolyglutamate synth	protein_coding	63	67	64	68	1.064
2357	NM_001193306,NM	**FPR1**	formyl peptide receptor 1	protein_coding	0	0	1	1	1.000
2358	NM_001005738,NM	**FPR2**	formyl peptide receptor 2	protein_coding	0	0	1	1	1.000
2359	NM_002030,XM_01	**FPR3**	formyl peptide receptor 3	protein_coding	0	0	1	1	1.000
2395	NM_000144,NM_18	**FXN**	frataxin	protein_coding	7	7	8	8	1.034
2444	NM_002031,XM_00	**FRK**	fyn related Src family tyr	protein_coding	0	0	1	1	1.015
2475	NM_004958,XM_00	**MTOR**	mechanistic target of rap	protein_coding	51	48	52	49	0.926
2483	NM_004477,XM_01	**FRG1**	FSHD region gene 1	protein_coding	151	151	152	152	1.002
2487	NM_001463	**FRZB**	frizzled related protein	protein_coding	0	0	1	1	1.000
2488	NM_000510,NM_00	**FSHB**	follicle stimulating hormo	protein_coding	0	0	1	1	1.000
2491	NM_001318521,NM	**CENPI**	centromere protein I	protein_coding	18	18	19	19	0.997
2492	NM_000145,NM_18	**FSHR**	follicle stimulating hormo	protein_coding	0	0	1	1	1.000
2494	NM_001276464,NM	**NR5A2**	nuclear receptor subfam	protein_coding	0	0	1	1	1.000
2495	NM_002032	**FTH1**	ferritin heavy chain 1	protein_coding	411	440	412	441	1.071
2496	gene-FTH1P1	**FTH1P1**	ferritin heavy chain 1 pse	pseudogene	0	0	1	1	1.000
2497	gene-FTH1P2	**FTH1P2**	ferritin heavy chain 1 pse	pseudogene	2	1	3	2	0.627
2498	NR_002201	**FTH1P3**	ferritin heavy chain 1 pse	transcribed_pseu	0	0	1	1	1.082
2499	gene-FTH1P4	**FTH1P4**	ferritin heavy chain 1 pse	pseudogene	0	0	1	1	1.000
2500	gene-FTH1P7	**FTH1P7**	ferritin heavy chain 1 pse	pseudogene	0	0	1	1	0.943
2501	gene-FTH1P8	**FTH1P8**	ferritin heavy chain 1 pse	pseudogene	0	0	1	1	0.992
2502	gene-FTH1P10	**FTH1P10**	ferritin heavy chain 1 pse	pseudogene	0	0	1	1	1.119
2503	gene-FTH1P11	**FTH1P11**	ferritin heavy chain 1 pse	pseudogene	0	0	1	1	1.000
2504	gene-FTH1P12	**FTH1P12**	ferritin heavy chain 1 pse	pseudogene	0	0	1	1	1.295
2507	gene-FTH1P15	**FTH1P15**	ferritin heavy chain 1 pse	pseudogene	0	0	1	1	1.121
2508	gene-FTH1P16	**FTH1P16**	ferritin heavy chain 1 pse	pseudogene	0	0	1	1	1.026
2509	gene-FTH1P5	**FTH1P5**	ferritin heavy chain 1 pse	pseudogene	0	0	1	1	0.973
2510	gene-FTH1P21	**FTH1P21**	ferritin heavy chain 1 pse	pseudogene	0	0	1	1	1.000
2512	NM_000146,XM_02	**FTL**	ferritin light chain	protein_coding	732	816	733	817	1.115
2515	NM_001278113,NM	**ADAM2**	ADAM metallopeptidase	protein_coding	0	0	1	1	1.000
2516	NM_004959	**NR5A1**	nuclear receptor subfam	protein_coding	0	0	1	1	1.000
2517	NM_000147,XM_00	**FUCA1**	alpha-L-fucosidase 1	protein_coding	1	2	2	3	1.112
2518	gene-FUCA1P1	**FUCA1P1**	alpha-L-fucosidase 1 pse	pseudogene	0	0	1	1	1.000
2519	NM_032020	**FUCA2**	alpha-L-fucosidase 2	protein_coding	21	20	22	21	0.934
2520	NM_000805	**GAST**	gastrin	protein_coding	0	0	1	1	1.000
2521	NM_001170634,NM	**FUS**	FUS RNA binding protei	protein_coding	641	837	642	838	1.305
2523	NM_000148,NM_00	**FUT1**	fucosyltransferase 1 (H b	protein_coding	0	0	1	1	1.000
2524	NM_000511,NM_00	**FUT2**	fucosyltransferase 2	protein_coding	0	0	1	1	1.000
2525	NM_000149,NM_00	**FUT3**	fucosyltransferase 3 (Lev	protein_coding	0	0	1	1	1.000
2526	NM_002033	**FUT4**	fucosyltransferase 4	protein_coding	0	0	1	1	1.014
2527	NM_002034	**FUT5**	fucosyltransferase 5	protein_coding	0	0	1	1	1.101
2528	NM_000150,NM_00	**FUT6**	fucosyltransferase 6	protein_coding	0	0	1	1	1.000
2529	NM_004479	**FUT7**	fucosyltransferase 7	protein_coding	12	14	13	15	1.174
2530	NM_001371533,NM	**FUT8**	fucosyltransferase 8	protein_coding	12	13	13	14	1.107
2531	NM_002035,XM_00	**KDSR**	3-ketodihydrosphingosin	protein_coding	9	8	10	9	0.844
2532	NM_001122951,NM	**ACKR1**	atypical chemokine recep	protein_coding	0	0	1	1	1.000
2533	NM_001243093,NM	**FYB1**	FYN binding protein 1	protein_coding	57	50	58	51	0.868
2534	NM_001370529,NM	**FYN**	FYN proto-oncogene, Sr	protein_coding	118	122	119	123	1.034
2535	NM_001466	**FZD2**	frizzled class receptor 2	protein_coding	0	0	1	1	0.844
2537	NM_002038,NM_02	**IFI6**	interferon alpha inducibl	protein_coding	849	880	850	881	1.037
2538	NM_000151,NM_00	**G6PC**	glucose-6-phosphatase	protein_coding	0	0	1	1	1.024
2539	NM_000402,NM_00	**G6PD**	glucose-6-phosphate de	protein_coding	291	309	292	310	1.059
2542	NM_001164277,NM	**SLC37A4**	solute carrier family 37 m	protein_coding	27	27	28	28	1.018
2543	NM_001040663,NR	**GAGE1**	G antigen 1	protein_coding	0	0	1	1	1.000
2547	NM_001288976,NM	**XRCC6**	X-ray repair cross compl	protein_coding	684	669	685	670	0.978
2548	NM_000152,NM_00	**GAA**	glucosidase alpha, acid	protein_coding	1	2	2	3	1.388
2549	NM_002039,NM_20	**GAB1**	GRB2 associated binding	protein_coding	0	0	1	1	1.000
2550	NM_001319053,NM	**GABBR1**	gamma-aminobutyric aci	protein_coding	0	0	1	1	1.000
2551	NM_001197297,NM	**GABPA**	GA binding protein trans	protein_coding	19	16	20	17	0.856
2553	NM_001320910,NM	**GABPB1**	GA binding protein trans	protein_coding	58	47	59	48	0.816
2554	NM_000806,NM_00	**GABRA1**	gamma-aminobutyric aci	protein_coding	0	0	1	1	1.000
2555	NM_000807,NM_00	**GABRA2**	gamma-aminobutyric aci	protein_coding	0	0	1	1	0.989
2556	NM_000808,XM_00	**GABRA3**	gamma-aminobutyric aci	protein_coding	0	0	1	1	1.000
2557	NM_000809,NM_00	**GABRA4**	gamma-aminobutyric aci	protein_coding	0	0	1	1	1.015
2558	NM_000810,NM_00	**GABRA5**	gamma-aminobutyric aci	protein_coding	0	0	1	1	1.000
2559	NM_000811	**GABRA6**	gamma-aminobutyric aci	protein_coding	0	0	1	1	1.000
2560	NM_000812,XM_01	**GABRB1**	gamma-aminobutyric aci	protein_coding	0	0	1	1	1.000
2561	NM_000813,NM_00	**GABRB2**	gamma-aminobutyric aci	protein_coding	0	0	1	1	1.000
2562	NM_000814,NM_00	**GABRB3**	gamma-aminobutyric aci	protein_coding	0	0	1	1	1.000
2563	NM_000815,XM_01	**GABRD**	gamma-aminobutyric aci	protein_coding	0	0	1	1	1.000
2564	NM_004961,XM_01	**GABRE**	gamma-aminobutyric aci	protein_coding	0	0	1	1	1.000
2565	NM_173536,XM_01	**GABRG1**	gamma-aminobutyric aci	protein_coding	0	0	1	1	1.000
2566	NM_000816,NM_00	**GABRG2**	gamma-aminobutyric aci	protein_coding	0	0	1	1	1.000
2567	NM_001270873,NM	**GABRG3**	gamma-aminobutyric aci	protein_coding	0	0	1	1	1.000
2568	NM_001291985,NM	**GABRP**	gamma-aminobutyric aci	protein_coding	0	0	1	1	1.000
2569	NM_001256703,NM	**GABRR1**	gamma-aminobutyric aci	protein_coding	0	0	1	1	1.000
2570	NM_002043,XM_01	**GABRR2**	gamma-aminobutyric aci	protein_coding	0	0	1	1	0.831
2571	NM_000817,NM_01	**GAD1**	glutamate decarboxylase	protein_coding	1	1	2	2	0.975
2572	NM_000818,NM_00	**GAD2**	glutamate decarboxylase	protein_coding	0	0	1	1	1.000
2580	NM_001318134,NM	**GAK**	cyclin G associated kinas	protein_coding	48	47	49	48	0.974
2581	NM_000153,NM_00	**GALC**	galactosylceramidase	protein_coding	6	7	7	8	1.110
2582	NM_000403,NM_00	**GALE**	UDP-galactose-4-epimer	protein_coding	55	54	56	55	0.988
2583	NM_001276468,NM	**B4GALNT1**	beta-1,4-N-acetyl-galact	protein_coding	0	0	1	1	1.080
2584	NM_000154,NM_00	**GALK1**	galactokinase 1	protein_coding	0	0	1	1	1.000
2585	NM_001001556,NM	**GALK2**	galactokinase 2	protein_coding	6	5	7	6	0.893
2587	NM_001480,XM_01	**GALR1**	galanin receptor 1	protein_coding	0	0	1	1	1.000
2588	NM_000512,NM_00	**GALNS**	galactosamine (N-acetyl)	protein_coding	2	1	3	2	0.741
2589	NM_020474	**GALNT1**	polypeptide N-acetylgala	protein_coding	71	61	72	62	0.865
2590	NM_001291866,NM	**GALNT2**	polypeptide N-acetylgala	protein_coding	25	21	26	22	0.849
2591	NM_004482,XM_00	**GALNT3**	polypeptide N-acetylgala	protein_coding	14	12	15	13	0.878
2592	NM_000155,NM_00	**GALT**	galactose-1-phosphate u	protein_coding	4	5	5	6	1.173
2593	NM_000156,NM_13	**GAMT**	guanidinoacetate N-meth	protein_coding	15	16	16	17	1.070
2595	NM_001301409,NM	**GANC**	glucosidase alpha, neutr	protein_coding	19	19	20	20	0.980
2596	NM_001130064,NM	**GAP43**	growth associated protei	protein_coding	0	0	1	1	1.000
2597	NM_001256799,NM	**GAPDH**	glyceraldehyde-3-phospl	protein_coding	4,381	4,619	4,382	4,620	1.054
2601	gene-GAPDHP60	**GAPDHP60**	glyceraldehyde-3-phospl	pseudogene	0	0	1	1	1.000
2609	gene-GAPDHP67	**GAPDHP67**	glyceraldehyde 3 phospl	pseudogene	0	0	1	1	1.000
2611	gene-GAPDHP69	**GAPDHP69**	glyceraldehyde 3 phospl	pseudogene	0	0	1	1	1.000
2613	gene-GAPDHP71	**GAPDHP71**	glyceraldehyde-3-phospl	pseudogene	0	0	1	1	1.000
2615	NM_001128922,NM	**LRRC32**	leucine rich repeat conta	protein_coding	0	0	1	1	1.000
2617	NM_001316772,NM	**GARS1**	glycyl-tRNA synthetase 1	protein_coding	114	108	115	109	0.943
2618	NM_000819,NM_00	**GART**	phosphoribosylglycinami	protein_coding	181	159	182	160	0.882
2619	NM_002048	**GAS1**	growth arrest specific 1	protein_coding	0	0	1	1	1.000
2620	NM_001143830,NM	**GAS2**	growth arrest specific 2	protein_coding	1	0	2	1	0.764
2621	NM_000820	**GAS6**	growth arrest specific 6	protein_coding	0	0	1	1	1.000
2622	NM_001286205,NM	**GAS8**	growth arrest specific 8	protein_coding	2	2	3	3	1.085
2623	NM_002049,XM_01	**GATA1**	GATA binding protein 1	protein_coding	0	0	1	1	1.000
2624	NM_001145661,NM	**GATA2**	GATA binding protein 2	protein_coding	0	0	1	1	1.000
2625	NM_001002295,NM	**GATA3**	GATA binding protein 3	protein_coding	10	12	11	13	1.139
2626	NM_001308093,NM	**GATA4**	GATA binding protein 4	protein_coding	0	0	1	1	1.000
2627	NM_005257	**GATA6**	GATA binding protein 6	protein_coding	0	0	1	1	1.000
2628	NM_001321015,NM	**GATM**	glycine amidinotransfera	protein_coding	3	3	4	4	0.972
2629	NM_000157,NM_00	**GBA**	glucosylceramidase beta	protein_coding	36	43	37	44	1.182
2630	NR_002188	**GBAP1**	glucosylceramidase beta	transcribed_pseu	12	12	13	13	1.069
2631	NM_001202469,NM	**NIPSNAP2**	nipsnap homolog 2	protein_coding	18	18	19	19	0.991
2632	NM_000158	**GBE1**	1,4-alpha-glucan branch	protein_coding	23	21	24	22	0.916
2633	NM_002053,XM_02	**GBP1**	guanylate binding protei	protein_coding	166	144	167	145	0.869
2634	NM_004120	**GBP2**	guanylate binding protei	protein_coding	55	52	56	53	0.952
2635	NM_001319179,NM	**GBP3**	guanylate binding protei	protein_coding	26	22	27	23	0.865
2636	NM_001098834	**GBX1**	gastrulation brain homeo	protein_coding	0	0	1	1	1.000

2637	NM_001301687,NM	**GBX2**	gastrulation brain homeo	protein_coding	0	0	1	1	1.000
2638	NM_000583,NM_00	**GC**	GC vitamin D binding pro	protein_coding	0	0	1	1	1.000
2639	NM_000159,NM_01	**GCDH**	glutaryl-CoA dehydroger	protein_coding	20	22	21	23	1.086
2641	NM_002054	**GCG**	glucagon	protein_coding	0	0	1	1	1.000
2642	NM_000160,XM_00	**GCGR**	glucagon receptor	protein_coding	0	0	1	1	1.000
2643	NM_000161,NM_00	**GCH1**	GTP cyclohydrolase 1	protein_coding	154	127	155	128	0.827
2644	NM_005258	**GCHFR**	GTP cyclohydrolase I fee	protein_coding	3	3	4	4	1.022
2645	NM_000162,NM_00	**GCK**	glucokinase	protein_coding	0	0	1	1	1.000
2646	NM_001486,XM_01	**GCKR**	glucokinase regulator	protein_coding	0	0	1	1	1.000
2647	NM_001487,NR_03	**BLOC1S1**	biogenesis of lysosomal	protein_coding	64	68	65	69	1.071
2648	NM_001376227,NM	**KAT2A**	lysine acetyltransferase 2	protein_coding	71	70	72	71	0.993
2649	NM_001278546,NM	**NR6A1**	nuclear receptor subfam	protein_coding	10	8	11	9	0.823
2650	NM_001097633,NM	**GCNT1**	glucosaminyl (N-acetyl) t	protein_coding	22	18	23	19	0.822
2651	NM_001374747,NM	**GCNT2**	glucosaminyl (N-acetyl) t	protein_coding	0	0	1	1	0.914
2652	NM_000513	**OPN1MW**	opsin 1, medium wave se	protein_coding	0	0	1	1	1.000
2653	NM_004483,NR_03	**GCSH**	glycine cleavage system	protein_coding	39	41	40	42	1.048
2657	NM_001492	**GDF1**	growth differentiation fac	protein_coding	0	0	1	1	1.000
2658	NM_016204	**GDF2**	growth differentiation fac	protein_coding	0	0	1	1	1.000
2660	NM_005259	**MSTN**	myostatin	protein_coding	0	0	1	1	0.935
2661	NM_001288824,NM	**GDF9**	growth differentiation fac	protein_coding	1	0	2	1	0.760
2662	NM_004962	**GDF10**	growth differentiation fac	protein_coding	0	0	1	1	1.000
2664	NM_001493	**GDI1**	GDP dissociation inhibito	protein_coding	97	97	98	98	0.999
2665	NM_001115156,NM	**GDI2**	GDP dissociation inhibito	protein_coding	321	305	322	306	0.952
2667	gene-GDI2P1	**GDI2P1**	GDP dissociation inhibito	pseudogene	0	0	1	1	1.000
2668	NM_000514,NM_00	**GDNF**	glial cell derived neurotro	protein_coding	0	0	1	1	1.000
2669	NM_005261,NM_18	**GEM**	GTP binding protein over	protein_coding	86	77	87	78	0.890
2670	NM_001131019,NM	**GFAP**	glial fibrillary acidic prote	protein_coding	0	0	1	1	1.000
2671	NM_005262	**GFER**	growth factor, augmente	protein_coding	23	26	24	27	1.118
2672	NM_001127215,NM	**GFI1**	growth factor independe	protein_coding	19	17	20	18	0.906
2673	NM_001244710,NM	**GFPT1**	glutamine--fructose-6-ph	protein_coding	32	27	33	28	0.832
2674	NM_001145453,NM	**GFRA1**	GDNF family receptor alp	protein_coding	0	0	1	1	1.000
2675	NM_001165038,NM	**GFRA2**	GDNF family receptor alp	protein_coding	0	0	1	1	1.020
2676	NM_001496	**GFRA3**	GDNF family receptor alp	protein_coding	0	0	1	1	0.954
2677	NM_000821,NM_00	**GGCX**	gamma-glutamyl carboxy	protein_coding	8	8	9	9	0.970
2678	NM_001288833,NM	**GGT1**	gamma-glutamyltransfer	protein_coding	3	3	4	4	1.041
2679	NR_003267	**GGT3P**	gamma-glutamyltransfer	transcribed_pseu	0	0	1	1	1.000
2681	NM_001382584,NM	**GGTA1**	glycoprotein alpha-galac	protein_coding	0	0	1	1	1.000
2683	NM_001378495,NM	**B4GALT1**	beta-1,4-galactosyltransf	protein_coding	30	28	31	29	0.934
2686	NM_001351702,NM	**GGT7**	gamma-glutamyltransfer	protein_coding	7	7	8	8	1.011
2687	NM_001099781,NM	**GGT5**	gamma-glutamyltransfer	protein_coding	0	0	1	1	1.000
2688	NM_000515,NM_02	**GH1**	growth hormone 1	protein_coding	0	0	1	1	1.000
2689	NM_002059,NM_02	**GH2**	growth hormone 2	protein_coding	0	0	1	1	1.000
2690	NM_000163,NM_00	**GHR**	growth hormone recepto	protein_coding	0	0	1	1	1.000
2691	NM_001184731,NM	**GHRH**	growth hormone releasin	protein_coding	0	0	1	1	1.000
2692	NM_000823	**GHRHR**	growth hormone releasin	protein_coding	0	0	1	1	1.000
2693	NM_004122,NM_19	**GHSR**	growth hormone secreta	protein_coding	0	0	1	1	1.000
2694	NM_005142,XM_01	**CBLIF**	cobalamin binding intrins	protein_coding	0	0	1	1	1.000
2695	NM_004123	**GIP**	gastric inhibitory polypep	protein_coding	0	0	1	1	1.000
2696	NM_000164,NM_00	**GIPR**	gastric inhibitory polypep	protein_coding	1	1	2	2	0.955
2697	NM_000165	**GJA1**	gap junction protein alph	protein_coding	0	0	1	1	1.000
2698	gene-GJA1P1	**GJA1P1**	gap junction protein alph	pseudogene	0	0	1	1	1.000
2700	NM_021954,XM_01	**GJA3**	gap junction protein alph	protein_coding	0	0	1	1	1.000
2701	NM_002060,XM_00	**GJA4**	gap junction protein alph	protein_coding	0	0	1	1	1.000
2702	NM_005266,NM_18	**GJA5**	gap junction protein alph	protein_coding	0	0	1	1	1.000
2703	NM_005267,XM_01	**GJA8**	gap junction protein alph	protein_coding	0	0	1	1	1.000
2705	NM_000166,NM_00	**GJB1**	gap junction protein beta	protein_coding	0	0	1	1	1.000
2706	NM_004004,XM_01	**GJB2**	gap junction protein beta	protein_coding	0	0	1	1	1.000
2707	NM_001005752,NM	**GJB3**	gap junction protein beta	protein_coding	0	0	1	1	1.000
2709	NM_005268,XM_00	**GJB5**	gap junction protein beta	protein_coding	0	0	1	1	1.000
2710	NM_000167,NM_00	**GK**	glycerol kinase	protein_coding	6	6	7	7	0.977
2712	NM_033214	**GK2**	glycerol kinase 2	protein_coding	0	0	1	1	1.000
2713	NR_026575	**GK3P**	glycerol kinase 3 pseudo	transcribed_pseu	0	0	1	1	1.000
2717	NM_000169,NR_16	**GLA**	galactosidase alpha	protein_coding	32	36	33	37	1.126
2719	NM_001164617,NM	**GPC3**	glypican 3	protein_coding	0	0	1	1	1.000
2720	NM_000404,NM_00	**GLB1**	galactosidase beta 1	protein_coding	22	22	23	23	0.996
2729	NM_001197115,NM	**GCLC**	glutamate-cysteine ligase	protein_coding	44	39	45	40	0.902
2730	NM_001308253,NM	**GCLM**	glutamate-cysteine ligase	protein_coding	16	15	17	16	0.911
2731	NM_000170	**GLDC**	glycine decarboxylase	protein_coding	38	38	39	39	1.004
2732	gene-GLDCP1	**GLDCP1**	glycine decarboxylase ps	pseudogene	0	0	1	1	1.023
2733	NM_001003722,NM	**GLE1**	GLE1 RNA export media	protein_coding	63	63	64	64	1.004
2734	NM_001145666,NM	**GLG1**	golgi glycoprotein 1	protein_coding	26	25	27	26	0.963
2735	NM_001160045,NM	**GLI1**	GLI family zinc finger 1	protein_coding	0	0	1	1	0.998
2736	NM_001371271,NM	**GLI2**	GLI family zinc finger 2	protein_coding	0	0	1	1	1.000
2737	NM_000168,XM_01	**GLI3**	GLI family zinc finger 3	protein_coding	0	0	1	1	0.924
2738	NM_138465	**GLI4**	GLI family zinc finger 4	protein_coding	4	4	5	5	0.988
2739	NM_006708	**GLO1**	glyoxalase I	protein_coding	399	351	400	352	0.881
2740	NM_002062,NR_13	**GLP1R**	glucagon like peptide 1 r	protein_coding	1	1	2	2	0.946
2741	NM_000171,NM_00	**GLRA1**	glycine receptor alpha 1	protein_coding	0	0	1	1	1.000
2742	NM_001118885,NM	**GLRA2**	glycine receptor alpha 2	protein_coding	0	0	1	1	1.000
2743	NM_000824,NM_00	**GLRB**	glycine receptor beta	protein_coding	0	0	1	1	1.000
2744	NM_001256310,NM	**GLS**	glutaminase	protein_coding	155	117	156	118	0.753
2745	NM_001118890,NM	**GLRX**	glutaredoxin	protein_coding	102	103	103	104	1.007
2746	NM_001318900,NM	**GLUD1**	glutamate dehydrogenas	protein_coding	160	161	161	162	1.005
2747	NM_012084	**GLUD2**	glutamate dehydrogenas	protein_coding	0	1	1	2	1.055
2749	NR_048575	**GLUD1P3**	glutamate dehydrogenas	transcribed_pseu	3	3	4	4	0.879
2750	gene-GLUD1P4	**GLUD1P4**	glutamate dehydrogenas	pseudogene	0	0	1	1	1.000
2752	NM_001033044,NM	**GLUL**	glutamate-ammonia ligas	protein_coding	106	108	107	109	1.021
2753	gene-GLULP1	**GLULP1**	glutamate-ammonia ligas	pseudogene	0	0	1	1	1.000
2754	gene-GLULP2	**GLULP2**	glutamate-ammonia ligas	pseudogene	0	0	1	1	1.000
2760	NM_000405,NM_00	**GM2A**	GM2 ganglioside activato	protein_coding	28	28	29	29	1.001
2761	gene-GM2AP1	**GM2AP1**	GM2 ganglioside activato	pseudogene	0	0	1	1	1.000
2762	NM_001253846,NM	**GMDS**	GDP-mannose 4,6-dehy	protein_coding	35	35	36	36	0.999
2764	NM_004124	**GMFB**	glia maturation factor bet	protein_coding	52	44	53	45	0.865
2765	NM_002066	**GML**	glycosylphosphatidylinos	protein_coding	1	0	2	1	0.643
2766	NM_006877,XM_01	**GMPR**	guanosine monophospha	protein_coding	0	0	1	1	1.000
2767	NM_002067	**GNA11**	G protein subunit alpha	protein_coding	0	0	1	1	1.000
2768	NM_001282440,NM	**GNA12**	G protein subunit alpha	protein_coding	21	17	22	18	0.811
2769	NM_002068	**GNA15**	G protein subunit alpha	protein_coding	0	0	1	1	0.922
2770	NM_001256414,NM	**GNAI1**	G protein subunit alpha i	protein_coding	0	0	1	1	1.000
2771	NM_001166425,NM	**GNAI2**	G protein subunit alpha i	protein_coding	307	322	308	323	1.048
2772	gene-GNAI2P1	**GNAI2P1**	G protein subunit alpha i	pseudogene	0	0	1	1	1.000
2773	NM_006496	**GNAI3**	G protein subunit alpha i	protein_coding	57	51	58	52	0.901
2774	NM_001142339,NM	**GNAL**	G protein subunit alpha	protein_coding	0	0	1	1	1.000
2775	NM_020988,NM_13	**GNAO1**	G protein subunit alpha c	protein_coding	4	4	5	5	0.949
2776	NM_002072,XM_01	**GNAQ**	G protein subunit alpha c	protein_coding	0	0	1	1	1.008
2777	gene-GNAQP1	**GNAQP1**	G protein subunit alpha c	pseudogene	0	0	1	1	1.000
2778	NM_000516,NM_00	**GNAS**	GNAS complex locus	protein_coding	1,098	1,140	1,099	1,141	1.038
2779	NM_000172,NM_14	**GNAT1**	G protein subunit alpha t	protein_coding	0	0	1	1	1.000
2780	NM_001377295,NM	**GNAT2**	G protein subunit alpha t	protein_coding	0	1	1	2	1.056
2781	NM_002073,XM_01	**GNAZ**	G protein subunit alpha z	protein_coding	0	0	1	1	1.000
2782	NM_001282538,NM	**GNB1**	G protein subunit beta 1	protein_coding	306	298	307	299	0.975
2783	NM_005273	**GNB2**	G protein subunit beta 2	protein_coding	205	203	206	204	0.992
2784	NM_001297571,NM	**GNB3**	G protein subunit beta 3	protein_coding	0	0	1	1	1.000
2785	NM_012202,XM_00	**GNG3**	G protein subunit gamma	protein_coding	0	0	1	1	1.000
2786	NM_001098721,NM	**GNG4**	G protein subunit gamma	protein_coding	0	0	1	1	1.000
2787	NM_005274	**GNG5**	G protein subunit gamma	protein_coding	403	421	404	422	1.045
2788	NM_052847,XM_01	**GNG7**	G protein subunit gamma	protein_coding	0	0	1	1	0.960
2789	gene-GNG5P1	**GNG5P1**	G protein subunit gamma	pseudogene	0	0	1	1	1.000
2790	NM_001017998,NM	**GNG10**	G protein subunit gamma	protein_coding	3	3	4	4	1.024
2791	NM_004126	**GNG11**	G protein subunit gamma	protein_coding	0	0	1	1	1.000
2792	NM_001329426,NM	**GNGT1**	G protein subunit gamma	protein_coding	0	0	1	1	1.000
2793	NM_001198754,NM	**GNGT2**	G protein subunit gamma	protein_coding	57	52	58	53	0.917
2794	NM_005275,XM_00	**GNL1**	G protein nucleolar 1 (pu	protein_coding	29	28	30	29	0.996
2796	NM_000825,NM_00	**GNRH1**	gonadotropin releasing h	protein_coding	1	1	2	2	0.927
2797	NM_001310220,NM	**GNRH2**	gonadotropin releasing h	protein_coding	1	0	2	1	0.837
2798	NM_000406,NM_00	**GNRHR**	gonadotropin releasing h	protein_coding	0	0	1	1	1.000

2799	NM_002076	**GNS**	glucosamine (N-acetyl)-6	protein_coding	23	23	24	24	0.997
2800	NM_002077,XM_00!	**GOLGA1**	golgin A1	protein_coding	9	9	10	10	1.000
2801	NM_001366244,NM	**GOLGA2**	golgin A2	protein_coding	26	24	27	25	0.924
2802	NM_001172557,NM	**GOLGA3**	golgin A3	protein_coding	12	10	13	11	0.892
2803	NM_001172713,NM	**GOLGA4**	golgin A4	protein_coding	19	15	20	16	0.790
2804	NM_001256486,NM	**GOLGB1**	golgin B1	protein_coding	11	10	12	11	0.869
2805	NM_002079	**GOT1**	glutamic-oxaloacetic tran	protein_coding	96	94	97	95	0.976
2806	NM_001286220,NM	**GOT2**	glutamic-oxaloacetic tran	protein_coding	86	85	87	86	0.983
2810	NM_006142	**SFN**	stratifin	protein_coding	0	0	1	1	0.943
2811	NM_000173	**GP1BA**	glycoprotein Ib platelet s	protein_coding	0	0	1	1	1.000
2812	NM_000407	**GP1BB**	glycoprotein Ib platelet s	protein_coding	0	0	1	1	1.000
2813	NM_001007240,NM	**GP2**	glycoprotein 2	protein_coding	0	0	1	1	1.000
2814	NM_004488	**GP5**	glycoprotein V platelet	protein_coding	0	0	1	1	1.000
2815	NM_000174,XM_00!	**GP9**	glycoprotein IX platelet	protein_coding	0	0	1	1	1.000
2817	NM_002081,XM_01	**GPC1**	glypican 1	protein_coding	0	0	1	1	1.002
2819	NM_001257199,NM	**GPD1**	glycerol-3-phosphate de	protein_coding	0	0	1	1	1.006
2820	NM_000408,NM_00	**GPD2**	glycerol-3-phosphate de	protein_coding	22	18	23	19	0.815
2821	NM_000175,NM_00	**GPI**	glucose-6-phosphate iso	protein_coding	404	409	405	410	1.013
2822	NM_001503,XM_01	**GPLD1**	glycosylphosphatidylinos	protein_coding	0	0	1	1	1.000
2823	NM_001261447,NM	**GPM6A**	glycoprotein M6A	protein_coding	0	0	1	1	1.000
2824	NM_001001994,NM	**GPM6B**	glycoprotein M6B	protein_coding	0	0	1	1	1.000
2825	NM_001098199,NM	**GPR1**	G protein-coupled recep	protein_coding	0	0	1	1	1.000
2826	NM_016602	**CCR10**	C-C motif chemokine rec	protein_coding	1	0	2	1	0.675
2827	NM_005281	**GPR3**	G protein-coupled recep	protein_coding	5	4	6	5	0.864
2828	NM_005282,XM_01	**GPR4**	G protein-coupled recep	protein_coding	0	0	1	1	1.001
2829	NM_001024644,NM	**XCR1**	X-C motif chemokine rec	protein_coding	47	44	48	45	0.937
2830	NM_001286099,NM	**GPR6**	G protein-coupled recep	protein_coding	0	0	1	1	1.000
2831	NM_005285	**NPBWR1**	neuropeptides B and W	protein_coding	0	0	1	1	0.978
2832	NM_005286	**NPBWR2**	neuropeptides B and W	protein_coding	0	0	1	1	1.000
2833	NM_001142797,NM	**CXCR3**	C-X-C motif chemokine r	protein_coding	168	155	169	156	0.923
2834	NM_004248	**PRLHR**	prolactin releasing horm	protein_coding	0	0	1	1	1.000
2835	NM_005288,XM_00!	**GPR12**	G protein-coupled recep	protein_coding	0	0	1	1	1.000
2837	NM_001381897,NM	**UTS2R**	urotensin 2 receptor	protein_coding	0	0	1	1	1.025
2838	NM_005290	**GPR15**	G protein-coupled recep	protein_coding	192	160	193	161	0.833
2840	NM_001161415,NM	**GPR17**	G protein-coupled recep	protein_coding	0	0	1	1	1.000
2841	NM_001098200,NM	**GPR18**	G protein-coupled recep	protein_coding	26	21	27	22	0.824
2842	NM_006143,XM_01	**GPR19**	G protein-coupled recep	protein_coding	2	3	3	4	1.324
2843	NM_005293	**GPR20**	G protein-coupled recep	protein_coding	0	0	1	1	1.000
2844	NM_005294,XM_00!	**GPR21**	G protein-coupled recep	protein_coding	0	0	1	1	1.000
2845	NM_005295,XM_01	**GPR22**	G protein-coupled recep	protein_coding	0	0	1	1	1.000
2846	NM_001278000,NM	**LPAR4**	lysophosphatidic acid re	protein_coding	0	0	1	1	0.937
2847	NM_005297	**MCHR1**	melanin concentrating h	protein_coding	0	0	1	1	1.000
2848	NM_005298	**GPR25**	G protein-coupled recep	protein_coding	0	0	1	1	0.809
2849	NM_153442	**GPR26**	G protein-coupled recep	protein_coding	0	0	1	1	1.000
2850	NM_018971	**GPR27**	G protein-coupled recep	protein_coding	0	0	1	1	1.063
2852	NM_001039966,NM	**GPER1**	G protein-coupled estrog	protein_coding	0	0	1	1	1.000
2853	NM_005299	**GPR31**	G protein-coupled recep	protein_coding	0	0	1	1	1.000
2854	NM_001506	**GPR32**	G protein-coupled recep	protein_coding	0	0	1	1	1.000
2855	gene-GPR32P1	**GPR32P1**	GPR32 pseudogene 1	pseudogene	0	0	1	1	1.000
2856	NM_001197184	**GPR33**	G protein-coupled recep	protein_coding	0	0	1	1	1.000
2857	NM_001097579,NM	**GPR34**	G protein-coupled recep	protein_coding	0	0	1	1	1.000
2859	NM_001195381,NM	**GPR35**	G protein-coupled recep	protein_coding	1	1	2	2	1.082
2861	NM_005302	**GPR37**	G protein-coupled recep	protein_coding	0	0	1	1	1.000
2862	NM_001507	**MLNR**	motilin receptor	protein_coding	0	0	1	1	1.000
2863	NM_001508	**GPR39**	G protein-coupled recep	protein_coding	0	0	1	1	1.000
2864	NM_005303	**FFAR1**	free fatty acid receptor 1	protein_coding	0	0	1	1	1.000
2865	NM_005304,XM_01	**FFAR3**	free fatty acid receptor 3	protein_coding	0	0	1	1	1.000
2866	NM_001348195	**GPR42**	G protein-coupled recep	protein_coding	0	0	1	1	1.000
2867	NM_001370087,NM	**FFAR2**	free fatty acid receptor 2	protein_coding	0	0	1	1	1.000
2868	NM_001004056,NM	**GRK4**	G protein-coupled recep	protein_coding	1	1	2	2	0.829
2869	NM_005308,XM_00!	**GRK5**	G protein-coupled recep	protein_coding	19	19	20	20	1.038
2870	NM_001004105,NM	**GRK6**	G protein-coupled recep	protein_coding	114	122	115	123	1.072
2871	NR_144554	**GRK6P1**	G protein-coupled recep	transcribed_pseu	0	0	1	1	1.006
2872	NM_017572,NM_19	**MKNK2**	MAPK interacting serine/	protein_coding	48	50	49	51	1.038
2873	NM_001321089,NM	**GPS1**	G protein pathway suppr	protein_coding	110	117	111	118	1.057
2874	NM_004489	**GPS2**	G protein pathway suppr	protein_coding	70	83	71	84	1.187
2875	NM_001382664,NM	**GPT**	glutamic--pyruvic transar	protein_coding	0	0	1	1	0.961
2876	NM_000581,NM_00	**GPX1**	glutathione peroxidase 1	protein_coding	153	160	154	161	1.044
2877	NM_002083,NR_04	**GPX2**	glutathione peroxidase 2	protein_coding	0	0	1	1	1.000
2878	NM_001329790,NM	**GPX3**	glutathione peroxidase 3	protein_coding	0	0	1	1	1.000
2879	NM_001039847,NM	**GPX4**	glutathione peroxidase 4	protein_coding	100	108	101	109	1.081
2880	NM_001509,NM_00	**GPX5**	glutathione peroxidase 5	protein_coding	0	0	1	1	1.000
2882	NM_015696	**GPX7**	glutathione peroxidase 7	protein_coding	0	0	1	1	1.000
2884	gene-GPX1P2	**GPX1P2**	glutathione peroxidase p	pseudogene	0	0	1	1	1.000
2885	NM_002086,NM_20	**GRB2**	growth factor receptor bo	protein_coding	142	133	143	134	0.935
2886	NM_001030002,NM	**GRB7**	growth factor receptor bo	protein_coding	0	0	1	1	0.841
2887	NM_001001549,NM	**GRB10**	growth factor receptor bo	protein_coding	0	0	1	1	1.000
2888	NM_001303422,NM	**GRB14**	growth factor receptor bo	protein_coding	0	0	1	1	1.000
2889	NM_001304275,NM	**RAPGEF1**	Rap guanine nucleotide	protein_coding	73	66	74	67	0.898
2890	NM_000827,NM_00	**GRIA1**	glutamate ionotropic rece	protein_coding	0	0	1	1	1.000
2891	NM_000826,NM_00	**GRIA2**	glutamate ionotropic rece	protein_coding	0	0	1	1	1.000
2892	NM_000828,NM_00	**GRIA3**	glutamate ionotropic rece	protein_coding	0	0	1	1	1.000
2893	NM_000829,NM_00	**GRIA4**	glutamate ionotropic rece	protein_coding	0	0	1	1	1.000
2894	NM_017551,XM_01	**GRID1**	glutamate ionotropic rece	protein_coding	0	0	1	1	1.000
2895	NM_001286838,NM	**GRID2**	glutamate ionotropic rece	protein_coding	0	0	1	1	1.000
2896	NM_002087,XM_00!	**GRN**	granulin precursor	protein_coding	33	35	34	36	1.080
2897	NM_000830,NM_00	**GRIK1**	glutamate ionotropic rece	protein_coding	0	0	1	1	1.000
2898	NM_001166247,NM	**GRIK2**	glutamate ionotropic rece	protein_coding	0	0	1	1	1.000
2899	NM_000831	**GRIK3**	glutamate ionotropic rece	protein_coding	0	0	1	1	1.012
2900	NM_001282470,NM	**GRIK4**	glutamate ionotropic rece	protein_coding	0	0	1	1	0.979
2901	NM_001301030,NM	**GRIK5**	glutamate ionotropic rece	protein_coding	0	0	1	1	0.835
2902	NM_000832,NM_00	**GRIN1**	glutamate ionotropic rece	protein_coding	0	0	1	1	1.000
2903	NM_000833,NM_00	**GRIN2A**	glutamate ionotropic rece	protein_coding	0	0	1	1	1.000
2904	NM_000834,XM_00!	**GRIN2B**	glutamate ionotropic rece	protein_coding	0	0	1	1	1.000
2905	NM_000835,NM_00	**GRIN2C**	glutamate ionotropic rece	protein_coding	0	0	1	1	0.976
2906	NM_000836,XM_01	**GRIN2D**	glutamate ionotropic rece	protein_coding	0	0	1	1	1.239
2907	NM_000837,NM_00	**GRINA**	glutamate ionotropic rece	protein_coding	5	5	6	6	0.955
2908	NM_000176,NM_00	**NR3C1**	nuclear receptor subfam	protein_coding	24	20	25	21	0.843
2909	NM_004491,XM_02	**ARHGAP35**	Rho GTPase activating p	protein_coding	27	24	28	25	0.875
2910	gene-NR3C1P1	**NR3C1P1**	nuclear receptor subfam	pseudogene	0	0	1	1	1.000
2911	NM_001278064,NM	**GRM1**	glutamate metabotropic r	protein_coding	0	0	1	1	1.023
2912	NM_000839,NM_00	**GRM2**	glutamate metabotropic r	protein_coding	0	0	1	1	0.975
2913	NM_000840,NM_00	**GRM3**	glutamate metabotropic r	protein_coding	0	0	1	1	1.000
2914	NM_000841,NM_00	**GRM4**	glutamate metabotropic r	protein_coding	0	0	1	1	1.000
2915	NM_000842,NM_00	**GRM5**	glutamate metabotropic r	protein_coding	0	0	1	1	0.961
2916	NM_000843	**GRM6**	glutamate metabotropic r	protein_coding	0	0	1	1	1.000
2917	NM_000844,NM_18	**GRM7**	glutamate metabotropic r	protein_coding	0	0	1	1	1.000
2918	NM_000845,NM_00	**GRM8**	glutamate metabotropic r	protein_coding	0	0	1	1	1.028
2919	NM_001511,NR_04	**CXCL1**	C-X-C motif chemokine li	protein_coding	0	0	1	1	1.000
2920	NM_002089	**CXCL2**	C-X-C motif chemokine li	protein_coding	0	0	1	1	1.000
2921	NM_002090	**CXCL3**	C-X-C motif chemokine li	protein_coding	0	0	1	1	1.000
2922	NM_001012512,NM	**GRP**	gastrin releasing peptide	protein_coding	0	0	1	1	1.000
2923	NM_005313	**PDIA3**	protein disulfide isomera	protein_coding	466	470	467	471	1.008
2925	NM_005314	**GRPR**	gastrin releasing peptide	protein_coding	0	0	1	1	0.978
2926	NM_001098477,NM	**GRSF1**	G-rich RNA sequence bi	protein_coding	44	42	45	43	0.953
2928	NM_005315	**GSC2**	goosecoid homeobox 2	protein_coding	0	0	1	1	1.000
2931	NM_019884,XR_00	**GSK3A**	glycogen synthase kinas	protein_coding	111	119	112	120	1.069
2932	NM_001146156,NM	**GSK3B**	glycogen synthase kinas	protein_coding	27	21	28	22	0.797
2934	NM_000177,NM_00	**GSN**	gelsolin	protein_coding	1	2	2	3	1.608
2935	NM_001130006,NM	**GSPT1**	G1 to S phase transition	protein_coding	91	82	92	83	0.910
2936	NM_000637,NM_00	**GSR**	glutathione-disulfide red	protein_coding	165	163	166	164	0.986
2937	NM_000178,NM_00	**GSS**	glutathione synthetase	protein_coding	100	98	101	99	0.981
2938	NM_001319059,NM	**GSTA1**	glutathione S-transferase	protein_coding	0	0	1	1	1.000
2939	NM_000846,XM_01	**GSTA2**	glutathione S-transferase	protein_coding	0	0	1	1	1.000
2940	NM_000847,NM_00	**GSTA3**	glutathione S-transferase	protein_coding	0	0	1	1	1.000
2941	NM_001512,XM_00!	**GSTA4**	glutathione S-transferase	protein_coding	1	1	2	2	0.806

2944	NM_000561,NM_14	**GSTM1**	glutathione S-transferase	protein_coding	0	0	1	1	1.166
2946	NM_000848,NM_00	**GSTM2**	glutathione S-transferase	protein_coding	0	0	1	1	1.000
2947	NM_000849,NR_024	**GSTM3**	glutathione S-transferase	protein_coding	0	0	1	1	1.000
2948	NM_000850,NM_14	**GSTM4**	glutathione S-transferase	protein_coding	9	10	10	11	1.137
2949	NM_000851,XM_00!	**GSTM5**	glutathione S-transferase	protein_coding	0	0	1	1	1.000
2950	NM_000852	**GSTP1**	glutathione S-transferase	protein_coding	905	928	906	929	1.025
2951	gene-GSTP1P1	**GSTP1P1**	glutathione S-transferase	pseudogene	0	0	1	1	1.000
2953	NR_126445	**GSTT2**	glutathione S-transferase	transcribed_pseu	0	0	1	1	1.000
2954	NM_001312660,NM	**GSTZ1**	glutathione S-transferase	protein_coding	13	10	14	11	0.806
2956	NM_000179,NM_00	**MSH6**	mutS homolog 6	protein_coding	69	65	70	66	0.948
2957	NM_001278940,NM	**GTF2A1**	general transcription fact	protein_coding	25	21	26	22	0.852
2958	NM_001320929,NM	**GTF2A2**	general transcription fact	protein_coding	138	134	139	135	0.970
2959	NM_001514,XM_01	**GTF2B**	general transcription fact	protein_coding	79	77	80	78	0.971
2960	NM_005513	**GTF2E1**	general transcription fact	protein_coding	35	33	36	34	0.946
2961	NM_001348353,NM	**GTF2E2**	general transcription fact	protein_coding	102	91	103	92	0.900
2962	NM_002096,XM_024	**GTF2F1**	general transcription fact	protein_coding	165	174	166	175	1.057
2963	NM_004128,XM_01	**GTF2F2**	general transcription fact	protein_coding	71	67	72	68	0.946
2964	gene-GTF2F2P1	**GTF2F2P1**	general transcription fact	pseudogene	0	0	1	1	1.000
2965	NM_001142307,NM	**GTF2H1**	general transcription fact	protein_coding	70	65	71	66	0.934
2966	NM_001364567,NM	**GTF2H2**	general transcription fact	protein_coding	105	91	106	92	0.868
2967	NM_001271866,NM	**GTF2H3**	general transcription fact	protein_coding	51	51	52	52	0.992
2968	NM_001517	**GTF2H4**	general transcription fact	protein_coding	34	34	35	35	0.993
2969	NM_001163636,NM	**GTF2I**	general transcription fact	protein_coding	79	71	80	72	0.894
2970	NR_002206	**GTF2IP1**	general transcription fact	transcribed_pseu	38	33	39	34	0.879
2971	NM_002097	**GTF3A**	general transcription fact	protein_coding	471	483	472	484	1.025
2972	NM_001242786,NM	**BRF1**	BRF1 RNA polymerase I	protein_coding	8	10	9	11	1.247
2974	NR_003923	**GUCY1B2**	guanylate cyclase 1 solu	transcribed_pseu	0	0	1	1	1.000
2975	NM_001286242,NM	**GTF3C1**	general transcription fact	protein_coding	50	51	51	52	1.007
2976	NM_001035521,NM	**GTF3C2**	general transcription fact	protein_coding	58	53	59	54	0.912
2977	NM_000855,NM_00	**GUCY1A2**	guanylate cyclase 1 solu	protein_coding	0	0	1	1	1.000
2978	NM_000409,NM_00	**GUCA1A**	guanylate cyclase activa	protein_coding	0	0	1	1	1.000
2979	NM_002098	**GUCA1B**	guanylate cyclase activa	protein_coding	3	3	4	4	0.997
2980	NM_033553	**GUCA2A**	guanylate cyclase activa	protein_coding	0	0	1	1	1.000
2981	NM_007102	**GUCA2B**	guanylate cyclase activa	protein_coding	0	0	1	1	1.000
2982	NM_000856,NM_00	**GUCY1A1**	guanylate cyclase 1 solu	protein_coding	0	0	1	1	1.000
2983	NM_000857,NM_00	**GUCY1B1**	guanylate cyclase 1 solu	protein_coding	0	0	1	1	1.000
2984	NM_004963,XM_01	**GUCY2C**	guanylate cyclase 2C	protein_coding	0	0	1	1	1.000
2986	NM_001522,XR_24	**GUCY2F**	guanylate cyclase 2F, re	protein_coding	0	0	1	1	1.000
2987	NM_000858,NM_00	**GUK1**	guanylate kinase 1	protein_coding	217	256	218	257	1.176
2990	NM_000181,NM_00	**GUSB**	glucuronidase beta	protein_coding	54	49	55	50	0.917
2992	NM_001184720,NM	**GYG1**	glycogenin 1	protein_coding	264	287	265	288	1.088
2993	NM_001308187,NM	**GYPA**	glycophorin A (MNS bloo	protein_coding	0	0	1	1	1.000
2994	NM_001304382,NM	**GYPB**	glycophorin B (MNS bloo	protein_coding	0	0	1	1	1.000
2995	NM_001256584,NM	**GYPC**	glycophorin C (Gerbich b	protein_coding	98	95	99	96	0.965
2996	NM_002102,NM_19	**GYPE**	glycophorin E (MNS bloo	protein_coding	0	0	1	1	1.000
2997	NM_001161587,NM	**GYS1**	glycogen synthase 1	protein_coding	46	48	47	49	1.042
2998	NM_021957,XM_00	**GYS2**	glycogen synthase 2	protein_coding	0	0	1	1	1.000
2999	NM_001270780,NM	**GZMH**	granzyme H	protein_coding	141	158	142	159	1.121
3000	NM_000180,XM_01	**GUCY2D**	guanylate cyclase 2D, re	protein_coding	0	0	1	1	1.000
3001	NM_006144	**GZMA**	granzyme A	protein_coding	1,284	1,261	1,285	1,262	0.982
3002	NM_001346011,NM	**GZMB**	granzyme B	protein_coding	1,850	2,149	1,851	2,150	1.161
3003	NM_002104	**GZMK**	granzyme K	protein_coding	247	240	248	241	0.969
3004	NM_001258351,NM	**GZMM**	granzyme M	protein_coding	163	191	164	192	1.169
3005	NM_005318	**H1-0**	H1.0 linker histone	protein_coding	0	0	1	1	1.000
3006	NM_005319	**H1-2**	H1.2 linker histone, clust	protein_coding	348	329	349	330	0.945
3007	NM_005320	**H1-3**	H1.3 linker histone, clust	protein_coding	1	1	2	2	1.125
3008	NM_005321	**H1-4**	H1.4 linker histone, clust	protein_coding	1	1	2	2	0.822
3009	NM_005322	**H1-5**	H1.5 linker histone, clust	protein_coding	0	0	1	1	0.669
3010	NM_005323	**H1-6**	H1.6 linker histone, clust	protein_coding	0	0	1	1	1.000
3012	NM_021052	**H2AC8**	H2A clustered histone 8	protein_coding	0	0	1	1	1.000
3013	NM_021065	**H2AC7**	H2A clustered histone 7	protein_coding	0	1	1	2	1.854
3014	NM_002105	**H2AX**	H2A.X variant histone	protein_coding	232	281	233	282	1.208
3015	NM_002106	**H2AZ1**	H2A.Z variant histone 1	protein_coding	1,148	1,181	1,149	1,182	1.029
3017	NM_021063,NM_13	**H2BC5**	H2B clustered histone 5	protein_coding	43	48	44	49	1.111
3018	NM_021062	**H2BC3**	H2B clustered histone 3	protein_coding	0	0	1	1	1.000
3020	NM_001379043,NM	**H3-3A**	H3.3 histone A	protein_coding	373	363	374	364	0.971
3021	NM_005324	**H3-3B**	H3.3 histone B	protein_coding	499	514	500	515	1.030
3024	NM_005325	**H1-1**	H1.1 linker histone, clust	protein_coding	0	0	1	1	1.000
3026	NM_001177660,NM	**HABP2**	hyaluronan binding prote	protein_coding	0	0	1	1	1.000
3028	NM_001037811,NM	**HSD17B10**	hydroxysteroid 17-beta d	protein_coding	340	381	341	382	1.123
3029	NM_001040427,NM	**HAGH**	hydroxyacylglutathione h	protein_coding	21	23	22	24	1.097
3030	NM_000182	**HADHA**	hydroxyacyl-CoA dehydr	protein_coding	111	108	112	109	0.969
3031	gene-HADHAP1	**HADHAP1**	HADHA pseudogene 1	pseudogene	0	0	1	1	1.000
3032	NM_000183,NM_00	**HADHB**	hydroxyacyl-CoA dehydr	protein_coding	56	58	57	59	1.040
3033	NM_001184705,NM	**HADH**	hydroxyacyl-CoA dehydr	protein_coding	60	61	61	62	1.016
3034	NM_001258333,NM	**HAL**	histidine ammonia-lyase	protein_coding	0	0	1	1	0.910
3035	NM_001258040,NM	**HARS1**	histidyl-tRNA synthetase	protein_coding	159	164	160	165	1.029
3036	NM_001297436,NM	**HAS1**	hyaluronan synthase 1	protein_coding	0	0	1	1	1.000
3037	NM_005328	**HAS2**	hyaluronan synthase 2	protein_coding	0	0	1	1	1.000
3038	NM_001199280,NM	**HAS3**	hyaluronan synthase 3	protein_coding	0	0	1	1	1.011
3039	NM_000558	**HBA1**	hemoglobin subunit alph	protein_coding	0	0	1	1	1.000
3040	NM_000517	**HBA2**	hemoglobin subunit alph	protein_coding	0	0	1	1	1.000
3042	NM_001003938	**HBM**	hemoglobin subunit mu	protein_coding	0	0	1	1	1.000
3043	NM_000518	**HBB**	hemoglobin subunit beta	protein_coding	0	0	1	1	1.000
3044	NR_001589	**HBBP1**	hemoglobin subunit beta	transcribed_pseu	0	0	1	1	1.000
3045	NM_000519	**HBD**	hemoglobin subunit delta	protein_coding	0	1	1	2	1.391
3046	NM_005330	**HBE1**	hemoglobin subunit epsi	protein_coding	0	0	1	1	1.000
3047	NM_000559	**HBG1**	hemoglobin subunit gam	protein_coding	0	0	1	1	1.000
3048	NM_000184	**HBG2**	hemoglobin subunit gam	protein_coding	0	0	1	1	1.000
3049	NM_005331	**HBQ1**	hemoglobin subunit theta	protein_coding	0	0	1	1	1.000
3050	NM_005332	**HBZ**	hemoglobin subunit zeta	protein_coding	0	0	1	1	1.094
3051	gene-HBZP1	**HBZP1**	hemoglobin subunit zeta	pseudogene	0	0	1	1	1.000
3052	NM_001122608,NM	**HCCS**	holocytochrome c syntha	protein_coding	40	38	41	39	0.954
3053	NM_000185	**SERPIND1**	serpin family D member	protein_coding	0	0	1	1	1.000
3054	NM_005334,XM_00	**HCFC1**	host cell factor C1	protein_coding	86	83	87	84	0.962
3055	NM_001172129,NM	**HCK**	HCK proto-oncogene, Sr	protein_coding	0	0	1	1	1.000
3059	NM_001292041,NM	**HCLS1**	hematopoietic cell-specif	protein_coding	449	456	450	457	1.015
3060	NM_001524	**HCRT**	hypocretin neuropeptide	protein_coding	0	0	1	1	1.000
3061	NM_001525,XM_01	**HCRTR1**	hypocretin receptor 1	protein_coding	0	0	1	1	1.000
3062	NM_001526,XM_01	**HCRTR2**	hypocretin receptor 2	protein_coding	0	0	1	1	1.000
3064	NM_002111	**HTT**	huntingtin	protein_coding	22	19	23	20	0.861
3065	NM_004964,XM_01	**HDAC1**	histone deacetylase 1	protein_coding	331	322	332	323	0.973
3066	NM_001527,NR_03	**HDAC2**	histone deacetylase 2	protein_coding	55	54	56	55	0.983
3067	NM_001306146,NM	**HDC**	histidine decarboxylase	protein_coding	0	0	1	1	0.907
3068	NM_001126050,NM	**HDGF**	heparin binding growth f	protein_coding	446	448	447	449	1.005
3069	NM_001243900,NM	**HDLBP**	high density lipoprotein b	protein_coding	163	161	164	162	0.990
3070	NM_001289067,NM	**HELLS**	helicase, lymphoid speci	protein_coding	32	27	33	28	0.855
3071	NM_001184976,NM	**NCKAP1L**	NCK associated protein 1	protein_coding	20	18	21	19	0.913
3073	NM_000520,NM_00	**HEXA**	hexosaminidase subunit	protein_coding	31	33	32	34	1.044
3074	NM_000521,NM_00	**HEXB**	hexosaminidase subunit	protein_coding	48	43	49	44	0.884
3075	NM_000186,NM_00	**CFH**	complement factor H	protein_coding	26	26	27	27	0.996
3077	NM_000410,NM_00	**HFE**	homeostatic iron regulato	protein_coding	0	0	1	1	1.000
3078	NM_001379306,NM	**CFHR1**	complement factor H rela	protein_coding	12	8	13	9	0.737
3080	NM_001312672,NM	**CFHR2**	complement factor H rela	protein_coding	0	0	1	1	1.000
3081	NM_000187,XM_00	**HGD**	homogentisate 1,2-dioxy	protein_coding	0	0	1	1	1.296
3082	NM_000601,NM_00	**HGF**	hepatocyte growth factor	protein_coding	0	0	1	1	1.000
3083	NM_001297439,NM	**HGFAC**	HGF activator	protein_coding	0	0	1	1	1.000
3084	NM_001159995,NM	**NRG1**	neuregulin 1	protein_coding	0	0	1	1	1.000
3087	NM_002729	**HHEX**	hematopoietically expres	protein_coding	0	0	1	1	1.000
3090	NM_001098202,NM	**HIC1**	HIC ZBTB transcriptional	protein_coding	6	6	7	7	0.976
3091	NM_001243084,NM	**HIF1A**	hypoxia inducible factor 1	protein_coding	74	66	75	67	0.895
3092	NM_001243198,NM	**HIP1**	huntingtin interacting pro	protein_coding	3	2	4	3	0.921
3093	NM_001111112,NM	**UBE2K**	ubiquitin conjugating enz	protein_coding	79	71	80	72	0.902
3094	NM_005340,NR_024	**HINT1**	histidine triad nucleotide	protein_coding	456	475	457	476	1.043
3096	NM_002114,XM_01	**HIVEP1**	HIVEP zinc finger 1	protein_coding	2	1	3	2	0.850
3097	NM_006734,XM_01	**HIVEP2**	HIVEP zinc finger 2	protein_coding	11	8	12	9	0.759

3098	NM_000188,NM_00	**HK1**	hexokinase 1	protein_coding	138	128	139	129	0.927
3099	NM_000189,NM_00	**HK2**	hexokinase 2	protein_coding	64	64	65	65	0.997
3101	NM_002115,XM_01	**HK3**	hexokinase 3	protein_coding	0	0	1	1	1.000
3104	NM_001278647,NM	**ZBTB48**	zinc finger and BTB dom	protein_coding	16	17	17	18	1.069
3105	NM_002116	**HLA-A**	major histocompatibility c	protein_coding	2,294	2,390	2,295	2,391	1.042
3106	NM_005514	**HLA-B**	major histocompatibility c	protein_coding	2,531	2,699	2,532	2,700	1.066
3107	NM_002117	**HLA-C**	major histocompatibility c	protein_coding	1,651	1,732	1,652	1,733	1.049
3108	NM_006120	**HLA-DMA**	major histocompatibility c	protein_coding	4	6	5	7	1.309
3109	NM_002118	**HLA-DMB**	major histocompatibility c	protein_coding	10	11	11	12	1.050
3110	NM_001165255,NM	**MNX1**	motor neuron and pancre	protein_coding	1	0	2	1	0.843
3111	NM_002119	**HLA-DOA**	major histocompatibility c	protein_coding	0	0	1	1	1.054
3112	NM_002120	**HLA-DOB**	major histocompatibility c	protein_coding	6	6	7	7	1.114
3113	NM_001242524,NM	**HLA-DPA1**	major histocompatibility c	protein_coding	3	2	4	3	0.802
3115	NM_002121	**HLA-DPB1**	major histocompatibility c	protein_coding	3	3	4	4	1.064
3116	NR_001435	**HLA-DPB2**	major histocompatibility c	transcribed_pseu	0	0	1	1	1.000
3117	NM_002122,XM_00	**HLA-DQA1**	major histocompatibility c	protein_coding	0	0	1	1	1.000
3118	NM_020056	**HLA-DQA2**	major histocompatibility c	protein_coding	0	0	1	1	1.000
3119	NM_001243961,NM	**HLA-DQB1**	major histocompatibility c	protein_coding	14	10	15	11	0.783
3120	NM_001198858,NM	**HLA-DQB2**	major histocompatibility c	protein_coding	0	0	1	1	1.000
3122	NM_019111	**HLA-DRA**	major histocompatibility c	protein_coding	0	0	1	1	0.952
3123	NM_002124	**HLA-DRB1**	major histocompatibility c	protein_coding	1	1	2	2	1.208
3127	NM_002125,XM_01	**HLA-DRB5**	major histocompatibility c	protein_coding	20	22	21	23	1.096
3128	NR_001298	**HLA-DRB6**	major histocompatibility c	transcribed_pseu	0	0	1	1	1.000
3131	NM_001330375,NM	**HLF**	HLF transcription factor,	protein_coding	0	0	1	1	1.000
3133	NM_005516,XM_01	**HLA-E**	major histocompatibility c	protein_coding	422	389	423	390	0.922
3134	NM_001098478,NM	**HLA-F**	major histocompatibility c	protein_coding	189	165	190	166	0.876
3135	NM_001363567,NM	**HLA-G**	major histocompatibility c	protein_coding	0	1	1	2	2.345
3136	NR_001434	**HLA-H**	major histocompatibility c	transcribed_pseu	139	158	140	159	1.129
3137	NR_024240	**HLA-J**	major histocompatibility c	transcribed_pseu	2	2	3	3	0.944
3138	gene-HLA-K	**HLA-K**	major histocompatibility c	pseudogene	8	8	9	9	1.037
3139	NR_027822	**HLA-L**	major histocompatibility c	transcribed_pseu	0	0	1	1	1.108
3140	NM_001194999,NM	**MR1**	major histocompatibility c	protein_coding	18	17	19	18	0.914
3141	NM_000411,NM_00	**HLCS**	holocarboxylase syntheta	protein_coding	5	5	6	6	1.057
3142	NM_021958	**HLX**	H2.0 like homeobox	protein_coding	0	0	1	1	1.000
3145	NM_000190,NM_00	**HMBS**	hydroxymethylbilane syn	protein_coding	95	96	96	97	1.010
3146	NM_001313892,NM	**HMGB1**	high mobility group box 1	protein_coding	433	410	434	411	0.947
3148	NM_001130688,NM	**HMGB2**	high mobility group box 2	protein_coding	1,056	1,118	1,057	1,119	1.059
3149	NM_001301228,NM	**HMGB3**	high mobility group box 3	protein_coding	12	12	13	13	0.945
3150	NM_004965,XM_01	**HMGN1**	high mobility group nucle	protein_coding	565	542	566	543	0.960
3151	NM_005517	**HMGN2**	high mobility group nucle	protein_coding	1,124	1,177	1,125	1,178	1.047
3152	gene-HMGN2P11	**HMGN2P11**	high mobility group nucle	pseudogene	0	0	1	1	1.000
3153	gene-HMGN2P12	**HMGN2P12**	high mobility group nucle	pseudogene	0	0	1	1	1.116
3155	NM_000191,NM_00	**HMGCL**	3-hydroxy-3-methylglutar	protein_coding	17	16	18	17	0.935
3156	NM_000859,NM_00	**HMGCR**	3-hydroxy-3-methylglutar	protein_coding	116	105	117	106	0.901
3157	NM_001098272,NM	**HMGCS1**	3-hydroxy-3-methylglutar	protein_coding	110	103	111	104	0.935
3158	NM_001166107,NM	**HMGCS2**	3-hydroxy-3-methylglutar	protein_coding	0	0	1	1	1.000
3159	NM_001319077,NM	**HMGA1**	high mobility group AT-h	protein_coding	511	514	512	515	1.006
3161	NM_001142556,NM	**HMMR**	hyaluronan mediated mo	protein_coding	114	101	115	102	0.887
3162	NM_002133	**HMOX1**	heme oxygenase 1	protein_coding	0	0	1	1	1.000
3163	NM_001127204,NM	**HMOX2**	heme oxygenase 2	protein_coding	119	117	120	118	0.980
3164	NM_001202233,NM	**NR4A1**	nuclear receptor subfam	protein_coding	1	0	2	1	0.702
3166	NM_001306142,NM	**HMX1**	H6 family homeobox 1	protein_coding	0	0	1	1	1.000
3167	NM_005519,XM_00	**HMX2**	H6 family homeobox 2	protein_coding	0	0	1	1	1.000
3169	NM_004496,XM_01	**FOXA1**	forkhead box A1	protein_coding	0	0	1	1	1.000
3170	NM_021784,NM_15	**FOXA2**	forkhead box A2	protein_coding	0	0	1	1	1.000
3171	NM_004497	**FOXA3**	forkhead box A3	protein_coding	0	0	1	1	1.104
3172	NM_000457,NM_00	**HNF4A**	hepatocyte nuclear facto	protein_coding	0	0	1	1	1.000
3174	NM_001330561,NM	**HNF4G**	hepatocyte nuclear facto	protein_coding	0	0	1	1	0.942
3175	NM_004498,NR_07	**ONECUT1**	one cut homeobox 1	protein_coding	0	0	1	1	1.000
3176	NM_001024074,NM	**HNMT**	histamine N-methyltrans	protein_coding	0	0	1	1	1.000
3177	NM_001300868,NM	**SLC29A2**	solute carrier family 29 m	protein_coding	0	0	1	1	1.000
3178	NM_002136,NM_03	**HNRNPA1**	heterogeneous nuclear r	protein_coding	1,192	1,155	1,193	1,156	0.969
3181	NM_002137,NM_03	**HNRNPA2B1**	heterogeneous nuclear r	protein_coding	1,795	1,596	1,796	1,597	0.889
3182	NM_004499,NM_03	**HNRNPAB**	heterogeneous nuclear r	protein_coding	713	725	714	726	1.017
3183	NM_001077442,NM	**HNRNPC**	heterogeneous nuclear r	protein_coding	775	750	776	751	0.968
3184	NM_001003810,NM	**HNRNPD**	heterogeneous nuclear r	protein_coding	499	522	500	523	1.045
3185	NM_001098204,NM	**HNRNPF**	heterogeneous nuclear r	protein_coding	453	464	454	465	1.023
3186	gene-RBMXP1	**RBMXP1**	RBMX pseudogene 1	pseudogene	0	0	1	1	1.000
3187	NM_001257293,NM	**HNRNPH1**	heterogeneous nuclear r	protein_coding	661	670	662	671	1.014
3188	NM_001032393,NM	**HNRNPH2**	heterogeneous nuclear r	protein_coding	87	85	88	86	0.970
3189	NM_001322434,NM	**HNRNPH3**	heterogeneous nuclear r	protein_coding	317	333	318	334	1.051
3190	NM_001318186,NM	**HNRNPK**	heterogeneous nuclear r	protein_coding	816	767	817	768	0.940
3191	NM_001005335,NM	**HNRNPL**	heterogeneous nuclear r	protein_coding	421	424	422	425	1.007
3192	NM_004501,NM_03	**HNRNPU**	heterogeneous nuclear r	protein_coding	419	397	420	398	0.946
3195	NM_001195517,NM	**TLX1**	T cell leukemia homeobo	protein_coding	0	0	1	1	1.000
3196	NM_016170	**TLX2**	T cell leukemia homeobo	protein_coding	0	0	1	1	1.000
3198	NM_005522,NM_15	**HOXA1**	homeobox A1	protein_coding	7	7	8	8	0.925
3199	NM_006735	**HOXA2**	homeobox A2	protein_coding	0	0	1	1	0.974
3200	NM_030661,NM_15	**HOXA3**	homeobox A3	protein_coding	1	1	2	2	0.986
3201	NM_002141	**HOXA4**	homeobox A4	protein_coding	2	3	3	4	1.161
3202	NM_019102	**HOXA5**	homeobox A5	protein_coding	4	3	5	4	0.739
3203	NM_024014	**HOXA6**	homeobox A6	protein_coding	2	1	3	2	0.789
3204	NM_006896	**HOXA7**	homeobox A7	protein_coding	14	14	15	15	1.028
3205	NM_152739	**HOXA9**	homeobox A9	protein_coding	31	32	32	33	1.021
3206	NM_018951,NR_03	**HOXA10**	homeobox A10	protein_coding	27	23	28	24	0.873
3207	NM_005523	**HOXA11**	homeobox A11	protein_coding	1	1	2	2	0.893
3208	NM_002143,XM_00	**HPCA**	hippocalcin	protein_coding	0	0	1	1	1.008
3209	NM_000522	**HOXA13**	homeobox A13	protein_coding	0	0	1	1	1.000
3211	NM_002144	**HOXB1**	homeobox B1	protein_coding	0	0	1	1	1.000
3212	NM_002145,XM_00	**HOXB2**	homeobox B2	protein_coding	105	114	106	115	1.085
3213	NM_001330322,NM	**HOXB3**	homeobox B3	protein_coding	11	10	12	11	0.937
3214	NM_024015	**HOXB4**	homeobox B4	protein_coding	57	59	58	60	1.042
3215	NM_002147	**HOXB5**	homeobox B5	protein_coding	0	0	1	1	0.950
3216	NM_001369397,NM	**HOXB6**	homeobox B6	protein_coding	0	0	1	1	1.000
3217	NM_004502	**HOXB7**	homeobox B7	protein_coding	42	43	43	44	1.038
3218	NM_024016,XM_00	**HOXB8**	homeobox B8	protein_coding	0	0	1	1	1.000
3219	NM_024017	**HOXB9**	homeobox B9	protein_coding	0	0	1	1	1.000
3221	NM_014620,NM_15	**HOXC4**	homeobox C4	protein_coding	0	0	1	1	0.981
3222	NM_018953,NR_00	**HOXC5**	homeobox C5	protein_coding	0	0	1	1	1.277
3223	NM_004503,NM_15	**HOXC6**	homeobox C6	protein_coding	0	0	1	1	0.847
3224	NM_022658	**HOXC8**	homeobox C8	protein_coding	0	0	1	1	1.032
3225	NM_006897	**HOXC9**	homeobox C9	protein_coding	0	0	1	1	1.000
3226	NM_017409	**HOXC10**	homeobox C10	protein_coding	0	0	1	1	1.000
3227	NM_014212	**HOXC11**	homeobox C11	protein_coding	0	0	1	1	1.000
3228	NM_173860	**HOXC12**	homeobox C12	protein_coding	0	0	1	1	1.000
3229	NM_017410	**HOXC13**	homeobox C13	protein_coding	0	0	1	1	1.000
3231	NM_024501	**HOXD1**	homeobox D1	protein_coding	0	0	1	1	1.000
3232	NM_006898,XM_00	**HOXD3**	homeobox D3	protein_coding	0	0	1	1	1.000
3233	NM_014621,XM_00	**HOXD4**	homeobox D4	protein_coding	0	0	1	1	1.000
3234	NM_001199746,NM	**HOXD8**	homeobox D8	protein_coding	0	0	1	1	1.000
3235	NM_014213	**HOXD9**	homeobox D9	protein_coding	0	0	1	1	1.000
3236	NM_002148	**HOXD10**	homeobox D10	protein_coding	0	0	1	1	1.000
3237	NM_021192	**HOXD11**	homeobox D11	protein_coding	0	0	1	1	1.000
3238	NM_021193	**HOXD12**	homeobox D12	protein_coding	0	0	1	1	1.088
3239	NM_000523,XM_01	**HOXD13**	homeobox D13	protein_coding	0	0	1	1	1.000
3240	NM_001126102,NM	**HP**	haptoglobin	protein_coding	0	0	1	1	1.000
3241	NM_001258357,NM	**HPCAL1**	hippocalcin like 1	protein_coding	7	7	8	8	0.937
3242	NM_001171993,NM	**HPD**	4-hydroxyphenylpyruvate	protein_coding	0	0	1	1	1.000
3248	NM_000860,NM_00	**HPGD**	15-hydroxyprostaglandin	protein_coding	21	22	22	23	1.033
3249	NM_001375441,NM	**HPN**	hepsin	protein_coding	0	0	1	1	1.000
3250	NM_020995,XM_02	**HPR**	haptoglobin-related prote	protein_coding	0	0	1	1	1.000
3251	NM_000194	**HPRT1**	hypoxanthine phosphoril	protein_coding	156	147	157	148	0.941
3255	gene-HPRT1P3	**HPRT1P3**	hypoxanthine phosphoril	pseudogene	0	0	1	1	1.000
3257	NM_000195,NM_00	**HPS1**	HPS1 biogenesis of lyso	protein_coding	17	17	18	18	0.995
3263	NM_000613	**HPX**	hemopexin	protein_coding	0	0	1	1	1.000
3265	NM_001130442,NM	**HRAS**	HRas proto-oncogene, G	protein_coding	48	50	49	51	1.045

3266	NM_181532	**ERAS**	ES cell expressed Ras	protein_coding	0	0	1	1	1.000
3267	NM_001135187,NM	**AGFG1**	ArfGAP with FG repeats	protein_coding	34	33	35	34	0.969
3268	NM_006076,XM_00	**AGFG2**	ArfGAP with FG repeats	protein_coding	18	21	19	22	1.114
3269	NM_000861,NM_00	**HRH1**	histamine receptor H1	protein_coding	0	0	1	1	0.931
3270	NM_002152,XM_01	**HRC**	histidine rich calcium bin	protein_coding	0	0	1	1	1.000
3273	NM_000412,XM_00	**HRG**	histidine rich glycoprotei	protein_coding	0	0	1	1	1.050
3274	NM_001131055,NM	**HRH2**	histamine receptor H2	protein_coding	0	0	1	1	1.000
3275	NM_001242864,NM	**PRMT2**	protein arginine methyltr	protein_coding	45	43	46	44	0.955
3276	NM_001207042,NM	**PRMT1**	protein arginine methyltr	protein_coding	586	608	587	609	1.037
3280	NM_005524	**HES1**	hes family bHLH transcri	protein_coding	0	0	1	1	1.000
3281	NM_001537	**HSBP1**	heat shock factor binding	protein_coding	18	16	19	17	0.922
3283	NM_000862,NM_00	**HSD3B1**	hydroxy-delta-5-steroid d	protein_coding	0	0	1	1	1.000
3284	NM_000198,NM_00	**HSD3B2**	hydroxy-delta-5-steroid d	protein_coding	0	0	1	1	1.000
3290	NM_001206741,NM	**HSD11B1**	hydroxysteroid 11-beta d	protein_coding	0	0	1	1	1.000
3291	NM_000196	**HSD11B2**	hydroxysteroid 11-beta d	protein_coding	0	0	1	1	1.000
3292	NM_000413,NM_00	**HSD17B1**	hydroxysteroid 17-beta d	protein_coding	0	1	1	2	1.486
3293	NM_000197,XM_01	**HSD17B3**	hydroxysteroid 17-beta d	protein_coding	0	0	1	1	1.000
3294	NM_002153,XR_00	**HSD17B2**	hydroxysteroid 17-beta d	protein_coding	0	0	1	1	1.000
3295	NM_000414,NM_00	**HSD17B4**	hydroxysteroid 17-beta d	protein_coding	48	45	49	46	0.950
3297	NM_005526,XM_00	**HSF1**	heat shock transcription	protein_coding	38	37	39	38	0.965
3298	NM_001135564,NM	**HSF2**	heat shock transcription	protein_coding	22	20	23	21	0.914
3299	NM_001040667,NM	**HSF4**	heat shock transcription	protein_coding	1	1	2	2	1.268
3300	NM_001039550,NM	**DNAJB2**	DnaJ heat shock protein	protein_coding	35	33	36	34	0.952
3301	NM_001314039,NM	**DNAJA1**	DnaJ heat shock protein	protein_coding	509	495	510	496	0.972
3303	NM_005345	**HSPA1A**	heat shock protein family	protein_coding	5	5	6	6	1.052
3304	NM_005346	**HSPA1B**	heat shock protein family	protein_coding	8	9	9	10	1.109
3305	NM_005527	**HSPA1L**	heat shock protein family	protein_coding	3	3	4	4	0.837
3306	NM_021979	**HSPA2**	heat shock protein family	protein_coding	1	1	2	2	1.100
3308	NM_002154	**HSPA4**	heat shock protein family	protein_coding	163	155	164	156	0.951
3309	NM_005347	**HSPA5**	heat shock protein family	protein_coding	495	552	496	553	1.117
3310	NM_002155	**HSPA6**	heat shock protein family	protein_coding	27	29	28	30	1.083
3311	NR_024151	**HSPA7**	heat shock protein family	transcribed_pseu	3	4	4	5	1.209
3312	NM_006597,NM_15	**HSPA8**	heat shock protein family	protein_coding	4,691	5,107	4,692	5,108	1.089
3313	NM_004134	**HSPA9**	heat shock protein family	protein_coding	494	460	495	461	0.932
3314	gene-HSPA8P1	**HSPA8P1**	heat shock protein family	pseudogene	0	0	1	1	0.963
3315	NM_001540	**HSPB1**	heat shock protein family	protein_coding	0	0	1	1	1.000
3316	NM_001541	**HSPB2**	heat shock protein family	protein_coding	0	0	1	1	1.119
3320	NM_001017963,NM	**HSP90AA1**	heat shock protein 90 alp	protein_coding	1,887	1,750	1,888	1,751	0.927
3321	NM_001007237,NM	**IGSF3**	immunoglobulin superfar	protein_coding	0	0	1	1	0.987
3322	gene-HSP90AA3P	**HSP90AA3P**	heat shock protein 90 alp	pseudogene	0	0	1	1	1.036
3323	gene-HSP90AA4P	**HSP90AA4P**	heat shock protein 90 alp	pseudogene	0	0	1	1	0.971
3324	gene-HSP90AA2P	**HSP90AA2P**	heat shock protein 90 alp	pseudogene	1	1	2	2	0.986
3326	NM_001271969,NM	**HSP90AB1**	heat shock protein 90 alp	protein_coding	3,108	3,033	3,109	3,034	0.976
3327	gene-HSP90AB3P	**HSP90AB3P**	heat shock protein 90 alp	pseudogene	1	1	2	2	1.021
3329	NM_002156,NM_19	**HSPD1**	heat shock protein family	protein_coding	2,281	2,148	2,282	2,149	0.942
3332	gene-HSPD1P3	**HSPD1P3**	heat shock protein family	pseudogene	0	0	1	1	0.985
3336	NM_002157	**HSPE1**	heat shock protein family	protein_coding	2,309	2,394	2,310	2,395	1.037
3337	NM_001300914,NM	**DNAJB1**	DnaJ heat shock protein	protein_coding	211	216	212	217	1.020
3338	NM_001307980,NM	**DNAJC4**	DnaJ heat shock protein	protein_coding	7	9	8	10	1.259
3339	NM_001291860,NM	**HSPG2**	heparan sulfate proteogl	protein_coding	0	0	1	1	0.981
3340	NM_001301063,NM	**NDST1**	N-deacetylase and N-sul	protein_coding	72	72	73	73	1.005
3344	NM_001375442,NM	**FOXN2**	forkhead box N2	protein_coding	36	28	37	29	0.797
3346	NM_001368990,NM	**HTN1**	histatin 1	protein_coding	0	0	1	1	1.000
3347	NM_000200	**HTN3**	histatin 3	protein_coding	0	0	1	1	1.000
3350	NM_000524	**HTR1A**	5-hydroxytryptamine rece	protein_coding	0	0	1	1	1.000
3351	NM_000863	**HTR1B**	5-hydroxytryptamine rece	protein_coding	0	0	1	1	1.039
3352	NM_000864	**HTR1D**	5-hydroxytryptamine rece	protein_coding	0	0	1	1	1.000
3354	NM_000865,XM_01	**HTR1E**	5-hydroxytryptamine rece	protein_coding	0	0	1	1	1.000
3355	NM_000866,NM_00	**HTR1F**	5-hydroxytryptamine rece	protein_coding	0	0	1	1	1.000
3356	NM_000621,NM_00	**HTR2A**	5-hydroxytryptamine rece	protein_coding	0	0	1	1	1.000
3357	NM_000867,NM_00	**HTR2B**	5-hydroxytryptamine rece	protein_coding	0	0	1	1	1.000
3358	NM_000868,NM_00	**HTR2C**	5-hydroxytryptamine rece	protein_coding	0	0	1	1	1.000
3359	NM_000869,NM_00	**HTR3A**	5-hydroxytryptamine rece	protein_coding	0	0	1	1	1.000
3360	NM_000870,NM_00	**HTR4**	5-hydroxytryptamine rece	protein_coding	0	0	1	1	1.000
3361	NM_024012	**HTR5A**	5-hydroxytryptamine rece	protein_coding	0	0	1	1	1.000
3362	NM_000871	**HTR6**	5-hydroxytryptamine rece	protein_coding	0	0	1	1	1.000
3363	NM_000872,NM_01	**HTR7**	5-hydroxytryptamine rece	protein_coding	0	0	1	1	1.000
3364	NM_001363683,NM	**HUS1**	HUS1 checkpoint clamp	protein_coding	10	9	11	10	0.957
3371	NM_002160,XM_00	**TNC**	tenascin C	protein_coding	0	0	1	1	1.000
3373	NM_033159,NM_15	**HYAL1**	hyaluronidase 1	protein_coding	0	0	1	1	1.000
3375	NM_000415,NM_00	**IAPP**	islet amyloid polypeptide	protein_coding	0	0	1	1	1.000
3376	NM_001374299,NM	**IARS1**	isoleucyl-tRNA synthetas	protein_coding	150	132	151	133	0.883
3381	NM_004967	**IBSP**	integrin binding sialoprot	protein_coding	0	0	1	1	1.000
3382	NM_001136020,NM	**ICA1**	islet cell autoantigen 1	protein_coding	4	4	5	5	0.898
3383	NM_000201	**ICAM1**	intercellular adhesion mo	protein_coding	96	103	97	104	1.070
3384	NM_000873,NM_00	**ICAM2**	intercellular adhesion mo	protein_coding	11	10	12	11	0.955
3385	NM_001320605,NM	**ICAM3**	intercellular adhesion mo	protein_coding	0	0	1	1	0.900
3386	NM_001039132,NM	**ICAM4**	intercellular adhesion mo	protein_coding	0	0	1	1	1.253
3394	NM_001363907,NM	**IRF8**	interferon regulatory fact	protein_coding	71	62	72	63	0.884
3396	NM_001303265,NM	**MRPL58**	mitochondrial ribosomal	protein_coding	113	111	114	112	0.977
3397	NM_002165,NM_18	**ID1**	inhibitor of DNA binding	protein_coding	1	0	2	1	0.687
3398	NM_002166	**ID2**	inhibitor of DNA binding	protein_coding	477	483	478	484	1.013
3399	NM_002167	**ID3**	inhibitor of DNA binding	protein_coding	53	54	54	55	1.011
3400	NM_001546	**ID4**	inhibitor of DNA binding	protein_coding	0	0	1	1	1.000
3416	NM_001165946,NM	**IDE**	insulin degrading enzym	protein_coding	19	17	20	18	0.884
3417	NM_001282386,NM	**IDH1**	isocitrate dehydrogenase	protein_coding	44	44	45	45	1.004
3418	NM_001289910,NM	**IDH2**	isocitrate dehydrogenase	protein_coding	102	105	103	106	1.033
3419	NM_005530,XM_00	**IDH3A**	isocitrate dehydrogenase	protein_coding	207	207	208	208	1.002
3420	NM_001258384,NM	**IDH3B**	isocitrate dehydrogenase	protein_coding	285	305	286	306	1.069
3421	NM_004135,NM_17	**IDH3G**	isocitrate dehydrogenase	protein_coding	63	73	64	74	1.159
3422	NM_001317955,NM	**IDI1**	isopentenyl-diphosphate	protein_coding	99	89	100	90	0.899
3423	NM_000202,NM_00	**IDS**	iduronate 2-sulfatase	protein_coding	91	83	92	84	0.917
3425	NM_000203,NM_00	**IDUA**	alpha-L-iduronidase	protein_coding	0	0	1	1	0.831
3426	NM_000204,NM_00	**CFI**	complement factor I	protein_coding	0	0	1	1	1.000
3428	NM_001206567,NM	**IFI16**	interferon gamma inducil	protein_coding	564	502	565	503	0.889
3429	NM_001288954,NM	**IFI27**	interferon alpha inducible	protein_coding	842	850	843	851	1.010
3430	NM_001330230,NM	**IFI35**	interferon induced protei	protein_coding	67	70	68	71	1.034
3431	NM_001185015,NM	**SP110**	SP110 nuclear body prot	protein_coding	66	58	67	59	0.871
3433	NM_001547	**IFIT2**	interferon induced protei	protein_coding	47	43	48	44	0.920
3434	NM_001270927,NM	**IFIT1**	interferon induced protei	protein_coding	31	27	32	28	0.881
3437	NM_001031683,NM	**IFIT3**	interferon induced protei	protein_coding	124	86	125	87	0.693
3439	NM_024013	**IFNA1**	interferon alpha 1	protein_coding	0	0	1	1	1.000
3440	NM_000605	**IFNA2**	interferon alpha 2	protein_coding	0	0	1	1	1.000
3441	NM_021068	**IFNA4**	interferon alpha 4	protein_coding	0	0	1	1	1.000
3442	NM_002169	**IFNA5**	interferon alpha 5	protein_coding	0	0	1	1	1.000
3443	NM_021002	**IFNA6**	interferon alpha 6	protein_coding	0	0	1	1	1.000
3444	NM_021057	**IFNA7**	interferon alpha 7	protein_coding	0	0	1	1	1.000
3445	NM_002170	**IFNA8**	interferon alpha 8	protein_coding	0	0	1	1	1.000
3446	NM_002171	**IFNA10**	interferon alpha 10	protein_coding	0	0	1	1	1.000
3447	NM_006900	**IFNA13**	interferon alpha 13	protein_coding	0	0	1	1	1.000
3448	NM_002172	**IFNA14**	interferon alpha 14	protein_coding	0	0	1	1	1.000
3449	NM_002173	**IFNA16**	interferon alpha 16	protein_coding	0	0	1	1	1.000
3451	NM_021268	**IFNA17**	interferon alpha 17	protein_coding	0	0	1	1	1.000
3452	NM_002175	**IFNA21**	interferon alpha 21	protein_coding	0	0	1	1	1.000
3453	NR_036676	**IFNA22P**	interferon alpha 22, pseu	transcribed_pseu	0	0	1	1	1.000
3454	NM_000629,XM_00	**IFNAR1**	interferon alpha and beta	protein_coding	26	22	27	23	0.845
3455	NM_000874,NM_00	**IFNAR2**	interferon alpha and beta	protein_coding	37	34	38	35	0.925
3456	NM_002176	**IFNB1**	interferon beta 1	protein_coding	1	1	2	2	0.943
3458	NM_000619	**IFNG**	interferon gamma	protein_coding	61	55	62	56	0.899
3459	NM_000416,NM_00	**IFNGR1**	interferon gamma recept	protein_coding	27	22	28	23	0.813
3460	NM_001329128,NM	**IFNGR2**	interferon gamma recept	protein_coding	0	0	1	1	1.000
3467	NM_002177	**IFNW1**	interferon omega 1	protein_coding	0	0	1	1	1.157
3475	NM_001007245,NM	**IFRD1**	interferon related develo	protein_coding	34	32	35	33	0.932
3476	NM_001370192,NM	**IGBP1**	immunoglobulin binding	protein_coding	49	43	50	44	0.881
3479	NM_000618,NM_00	**IGF1**	insulin like growth factor	protein_coding	2	1	3	2	0.942
3480	NM_000875,NM_00	**IGF1R**	insulin like growth factor	protein_coding	0	0	1	1	0.943

3481	NM_000612,NM_00	**IGF2**	insulin like growth factor	protein_coding	0	0	1	1	1.000
3482	NM_000876	**IGF2R**	insulin like growth factor	protein_coding	40	40	41	41	0.992
3483	NM_001146006,NM	**IGFALS**	insulin like growth factor	protein_coding	0	0	1	1	1.000
3484	NM_000596	**IGFBP1**	insulin like growth factor	protein_coding	0	0	1	1	1.000
3485	NM_000597,NM_00	**IGFBP2**	insulin like growth factor	protein_coding	0	0	1	1	1.000
3486	NM_000598,NM_00	**IGFBP3**	insulin like growth factor	protein_coding	0	0	1	1	0.903
3487	NM_001552	**IGFBP4**	insulin like growth factor	protein_coding	0	0	1	1	1.000
3488	NM_000599	**IGFBP5**	insulin like growth factor	protein_coding	0	0	1	1	1.032
3489	NM_002178	**IGFBP6**	insulin like growth factor	protein_coding	2	1	3	2	0.700
3490	NM_001253835,NM	**IGFBP7**	insulin like growth factor	protein_coding	15	14	16	15	0.949
3491	NM_001554	**CCN1**	cellular communication r	protein_coding	0	0	1	1	1.000
3493	id-IGHA1	**IGHA1**	immunoglobulin heavy co	C_region	1	1	2	2	1.098
3494	id-IGHA2	**IGHA2**	immunoglobulin heavy co	C_region	0	0	1	1	1.000
3495	id-IGHD	**IGHD**	immunoglobulin heavy co	C_region	0	0	1	1	1.281
3497	id-IGHE	**IGHE**	immunoglobulin heavy co	C_region	0	0	1	1	1.000
3498	id-IGHEP1	**IGHEP1**	immunoglobulin heavy co	C_region_pseudo	0	0	1	1	1.000
3500	id-IGHG1	**IGHG1**	immunoglobulin heavy co	C_region	0	0	1	1	1.000
3501	id-IGHG2	**IGHG2**	immunoglobulin heavy co	C_region	0	0	1	1	1.000
3502	id-IGHG3,id-IGHG3	**IGHG3**	immunoglobulin heavy co	C_region	2	2	3	3	0.995
3503	id-IGHG4	**IGHG4**	immunoglobulin heavy co	C_region	0	0	1	1	1.072
3505	id-IGHGP	**IGHGP**	immunoglobulin heavy co	C_region_pseudo	0	0	1	1	1.000
3507	id-IGHM,id-IGHM-2	**IGHM**	immunoglobulin heavy co	C_region	64	69	65	70	1.069
3508	NM_002180,XM_00	**IGHMBP2**	immunoglobulin mu DNA	protein_coding	14	15	15	16	1.054
3512	NM_144646	**JCHAIN**	joining chain of multimer	protein_coding	1	1	2	2	1.035
3513	gene-JCHAINP1	**JCHAINP1**	joining chain of multimer	pseudogene	0	0	1	1	1.000
3514	id-IGKC	**IGKC**	immunoglobulin kappa c	C_region	0	0	1	1	1.000
3516	NM_001363577,NM	**RBPJ**	recombination signal bin	protein_coding	50	35	51	36	0.702
3537	id-IGLC1	**IGLC1**	immunoglobulin lambda	C_region	0	0	1	1	1.000
3538	id-IGLC2	**IGLC2**	immunoglobulin lambda	C_region	0	0	1	1	1.000
3539	id-IGLC3	**IGLC3**	immunoglobulin lambda	C_region	0	0	1	1	1.000
3540	id-IGLC4	**IGLC4**	immunoglobulin lambda	C_region_pseudo	0	0	1	1	1.000
3541	id-IGLC5	**IGLC5**	immunoglobulin lambda	C_region_pseudo	0	0	1	1	1.000
3542	id-IGLC6	**IGLC6**	immunoglobulin lambda	C_region_pseudo	0	0	1	1	1.000
3543	NM_001369906,NM	**IGLL1**	immunoglobulin lambda	protein_coding	0	0	1	1	1.000
3547	NM_001170961,NM	**IGSF1**	immunoglobulin superfa	protein_coding	0	0	1	1	1.000
3549	NM_002181	**IHH**	Indian hedgehog signalir	protein_coding	0	0	1	1	0.932
3550	NM_006083,XR_00	**IK**	IK cytokine	protein_coding	139	134	140	135	0.964
3551	NM_001190720,NM	**IKBKB**	inhibitor of nuclear factor	protein_coding	22	23	23	24	1.066
3552	NM_000575,NM_00	**IL1A**	interleukin 1 alpha	protein_coding	0	0	1	1	1.000
3553	NM_000576,XM_01	**IL1B**	interleukin 1 beta	protein_coding	0	0	1	1	1.000
3554	NM_000877,NM_00	**IL1R1**	interleukin 1 receptor typ	protein_coding	0	0	1	1	1.026
3556	NM_001167928,NM	**IL1RAP**	interleukin 1 receptor acc	protein_coding	0	0	1	1	1.000
3557	NM_000577,NM_00	**IL1RN**	interleukin 1 receptor ant	protein_coding	33	19	34	20	0.596
3558	NM_000586	**IL2**	interleukin 2	protein_coding	0	0	1	1	1.000
3559	NM_000417,NM_00	**IL2RA**	interleukin 2 receptor sul	protein_coding	49	44	50	45	0.913
3560	NM_000878,NM_00	**IL2RB**	interleukin 2 receptor sul	protein_coding	669	696	670	697	1.039
3561	NM_000206	**IL2RG**	interleukin 2 receptor sul	protein_coding	428	427	429	428	0.998
3562	NM_000588	**IL3**	interleukin 3	protein_coding	0	0	1	1	1.000
3563	NM_001267713,NM	**IL3RA**	interleukin 3 receptor sul	protein_coding	4	4	5	5	1.039
3565	NM_000589,NM_00	**IL4**	interleukin 4	protein_coding	0	0	1	1	1.000
3566	NM_000418,NM_00	**IL4R**	interleukin 4 receptor	protein_coding	52	44	53	45	0.848
3567	NM_000879,XM_00	**IL5**	interleukin 5	protein_coding	0	0	1	1	1.000
3568	NM_000564,NM_00	**IL5RA**	interleukin 5 receptor sul	protein_coding	0	0	1	1	1.000
3569	NM_000600,NM_00	**IL6**	interleukin 6	protein_coding	0	0	1	1	0.910
3570	NM_000565,NM_00	**IL6R**	interleukin 6 receptor	protein_coding	0	0	1	1	1.000
3572	NM_001190981,NM	**IL6ST**	interleukin 6 signal trans	protein_coding	10	8	11	9	0.823
3573	gene-IL6STP1	**IL6STP1**	interleukin 6 signal trans	pseudogene	0	0	1	1	1.000
3574	NM_000880,NM_00	**IL7**	interleukin 7	protein_coding	0	0	1	1	1.000
3575	NM_002185,NR_12	**IL7R**	interleukin 7 receptor	protein_coding	0	0	1	1	1.000
3576	NM_000584,NM_00	**CXCL8**	C-X-C motif chemokine li	protein_coding	0	0	1	1	1.000
3577	NM_000634	**CXCR1**	C-X-C motif chemokine r	protein_coding	3	3	4	4	0.936
3578	NM_000590	**IL9**	interleukin 9	protein_coding	0	0	1	1	1.000
3579	NM_001168298,NM	**CXCR2**	C-X-C motif chemokine r	protein_coding	0	0	1	1	1.000
3580	NR_002712	**CXCR2P1**	C-X-C motif chemokine r	transcribed_pseu	0	0	1	1	1.000
3581	NM_002186,NM_00	**IL9R**	interleukin 9 receptor	protein_coding	12	7	13	8	0.624
3586	NM_000572,NM_00	**IL10**	interleukin 10	protein_coding	37	47	38	48	1.261
3587	NM_001558,NR_02	**IL10RA**	interleukin 10 receptor su	protein_coding	10	10	11	11	1.016
3588	NM_000628	**IL10RB**	interleukin 10 receptor su	protein_coding	43	42	44	43	0.990
3589	NM_000641,NM_00	**IL11**	interleukin 11	protein_coding	0	0	1	1	1.071
3590	NM_001142784,NR	**IL11RA**	interleukin 11 receptor su	protein_coding	0	0	1	1	1.026
3592	NM_000882,NM_00	**IL12A**	interleukin 12A	protein_coding	2	2	3	3	0.946
3593	NM_002187	**IL12B**	interleukin 12B	protein_coding	0	0	1	1	1.000
3594	NM_001290023,NM	**IL12RB1**	interleukin 12 receptor su	protein_coding	45	50	46	51	1.089
3595	NM_001258214,NM	**IL12RB2**	interleukin 12 receptor su	protein_coding	80	76	81	77	0.950
3596	NM_001354991,NM	**IL13**	interleukin 13	protein_coding	0	0	1	1	1.000
3597	NM_001560,XM_01	**IL13RA1**	interleukin 13 receptor su	protein_coding	0	0	1	1	1.000
3598	NM_000640	**IL13RA2**	interleukin 13 receptor su	protein_coding	0	0	1	1	1.000
3600	NM_000585,NM_17	**IL15**	interleukin 15	protein_coding	6	4	7	5	0.799
3601	NM_001243539,NM	**IL15RA**	interleukin 15 receptor su	protein_coding	36	34	37	35	0.937
3603	NM_001172128,NM	**IL16**	interleukin 16	protein_coding	30	30	31	31	0.980
3604	NM_001561,XM_00	**TNFRSF9**	TNF receptor superfamily	protein_coding	0	0	1	1	1.104
3605	NM_002190	**IL17A**	interleukin 17A	protein_coding	0	0	1	1	1.000
3606	NM_001243211,NM	**IL18**	interleukin 18	protein_coding	0	0	1	1	1.000
3607	NM_004514,XM_02	**FOXK2**	forkhead box K2	protein_coding	72	71	73	72	0.993
3608	NM_001267809,NM	**ILF2**	interleukin enhancer bind	protein_coding	503	505	504	506	1.004
3609	NM_001137673,NM	**ILF3**	interleukin enhancer bind	protein_coding	340	326	341	327	0.958
3611	NM_001014794,NM	**ILK**	integrin linked kinase	protein_coding	39	45	40	46	1.132
3612	NM_001144878,NM	**IMPA1**	inositol monophosphatas	protein_coding	61	49	62	50	0.819
3613	NM_014214,XM_01	**IMPA2**	inositol monophosphatas	protein_coding	0	0	1	1	1.000
3614	NM_000883,NM_00	**IMPDH1**	inosine monophosphate	protein_coding	46	46	47	47	1.012
3615	NM_000884,XM_00	**IMPDH2**	inosine monophosphate	protein_coding	401	379	402	380	0.945
3617	NM_001282368,NM	**IMPG1**	interphotoreceptor matrix	protein_coding	0	0	1	1	1.000
3619	NM_001040694,NM	**INCENP**	inner centromere protein	protein_coding	54	51	55	52	0.938
3620	NM_002164	**IDO1**	indoleamine 2,3-dioxyge	protein_coding	0	0	1	1	1.000
3621	NM_001267728,NM	**ING1**	inhibitor of growth family	protein_coding	23	20	24	21	0.851
3622	NM_001291959,NM	**ING2**	inhibitor of growth family	protein_coding	9	8	10	9	0.973
3623	NM_002191	**INHA**	inhibin subunit alpha	protein_coding	0	0	1	1	1.000
3624	NM_002192,XM_01	**INHBA**	inhibin subunit beta A	protein_coding	0	0	1	1	1.000
3625	NM_002193	**INHBB**	inhibin subunit beta B	protein_coding	0	0	1	1	1.000
3626	NM_005538	**INHBC**	inhibin subunit beta C	protein_coding	0	0	1	1	1.053
3627	NM_001565,NR_16	**CXCL10**	C-X-C motif chemokine li	protein_coding	1	2	2	3	1.239
3628	NM_001128928,NM	**INPP1**	inositol polyphosphate-1	protein_coding	33	34	34	35	1.037
3630	NM_000207,NM_00	**INS**	insulin	protein_coding	0	0	1	1	1.000
3631	NM_001134224,NM	**INPP4A**	inositol polyphosphate-4	protein_coding	34	29	35	30	0.844
3632	NM_001321042,NM	**INPP5A**	inositol polyphosphate-5	protein_coding	3	2	4	3	0.789
3633	NM_001297434,NM	**INPP5B**	inositol polyphosphate-5	protein_coding	14	12	15	13	0.909
3635	NM_001017915,NM	**INPP5D**	inositol polyphosphate-5	protein_coding	105	98	106	99	0.940
3636	NM_001567,XM_00	**INPPL1**	inositol polyphosphate p	protein_coding	41	36	42	37	0.879
3638	NM_001346590,NM	**INSIG1**	insulin induced gene 1	protein_coding	58	58	59	59	0.989
3640	NM_001265587,NM	**INSL3**	insulin like 3	protein_coding	0	0	1	1	1.333
3641	NM_002195	**INSL4**	insulin like 4	protein_coding	0	0	1	1	1.000
3642	NM_002196	**INSM1**	INSM transcriptional repr	protein_coding	0	0	1	1	1.000
3643	NM_000208,NM_00	**INSR**	insulin receptor	protein_coding	0	0	1	1	1.000
3645	NM_014215	**INSRR**	insulin receptor related r	protein_coding	0	0	1	1	1.000
3646	NM_001568,XM_01	**EIF3E**	eukaryotic translation init	protein_coding	345	309	346	310	0.897
3647	gene-EIF3EP1	**EIF3EP1**	eukaryotic translation init	pseudogene	6	4	7	5	0.771
3651	NM_000209,XR_94	**PDX1**	pancreatic and duodenal	protein_coding	0	0	1	1	1.000
3652	NM_001145349,NM	**IPP**	intracisternal A particle-p	protein_coding	11	11	12	12	0.997
3653	NR_023915	**IPW**	imprinted in Prader-Willi	lncRNA	2	2	3	3	0.987
3654	NM_001025242,NM	**IRAK1**	interleukin 1 receptor as	protein_coding	189	190	190	191	1.002
3655	NM_000210,NM_00	**ITGA6**	integrin subunit alpha 6	protein_coding	26	26	27	27	0.985
3656	NM_001570	**IRAK2**	interleukin 1 receptor as	protein_coding	1	1	2	2	1.061
3658	NM_001320941,NM	**IREB2**	iron responsive element	protein_coding	73	62	74	63	0.842
3659	NM_001354924,NM	**IRF1**	interferon regulatory fact	protein_coding	120	134	121	135	1.114
3660	NM_002199,XM_02	**IRF2**	interferon regulatory fact	protein_coding	30	27	31	28	0.925
3661	NM_001197122,NM	**IRF3**	interferon regulatory fact	protein_coding	74	79	75	80	1.075

3662	NM_001195286,NM	**IRF4**	interferon regulatory fact	protein_coding	56	61	57	62	1.080
3663	NM_001098627,NM	**IRF5**	interferon regulatory fact	protein_coding	0	0	1	1	1.000
3664	NM_001206696,NM	**IRF6**	interferon regulatory fact	protein_coding	1	1	2	2	1.007
3665	NM_001572,NM_00	**IRF7**	interferon regulatory fact	protein_coding	37	33	38	34	0.913
3667	NM_005544	**IRS1**	insulin receptor substrate	protein_coding	1	1	2	2	0.815
3669	NM_001303233,NM	**ISG20**	interferon stimulated exo	protein_coding	53	58	54	59	1.088
3670	NM_002202,XM_01	**ISL1**	ISL LIM homeobox 1	protein_coding	0	0	1	1	1.000
3671	NM_005545,NM_20	**ISLR**	immunoglobulin superfa	protein_coding	0	0	1	1	1.000
3672	NM_181501	**ITGA1**	integrin subunit alpha 1	protein_coding	3	2	4	3	0.658
3673	NM_002203,NR_07	**ITGA2**	integrin subunit alpha 2	protein_coding	0	0	1	1	1.000
3674	NM_000419,XM_01	**ITGA2B**	integrin subunit alpha 2b	protein_coding	0	0	1	1	0.997
3675	NM_002204,XM_00	**ITGA3**	integrin subunit alpha 3	protein_coding	0	0	1	1	1.000
3676	NM_000885,NM_00	**ITGA4**	integrin subunit alpha 4	protein_coding	309	276	310	277	0.891
3678	NM_002205,XM_02	**ITGA5**	integrin subunit alpha 5	protein_coding	0	0	1	1	1.000
3679	NM_001144996,NM	**ITGA7**	integrin subunit alpha 7	protein_coding	0	0	1	1	1.036
3680	NM_002207	**ITGA9**	integrin subunit alpha 9	protein_coding	0	0	1	1	0.965
3681	NM_001318185,NM	**ITGAD**	integrin subunit alpha D	protein_coding	60	60	61	61	0.996
3682	NM_002208,XM_01	**ITGAE**	integrin subunit alpha E	protein_coding	5	4	6	5	0.861
3683	NM_001114380,NM	**ITGAL**	integrin subunit alpha L	protein_coding	229	224	230	225	0.976
3684	NM_000632,NM_00	**ITGAM**	integrin subunit alpha M	protein_coding	3	2	4	3	0.969
3685	NM_001144999,NM	**ITGAV**	integrin subunit alpha V	protein_coding	0	0	1	1	1.000
3687	NM_000887,NM_00	**ITGAX**	integrin subunit alpha X	protein_coding	15	15	16	16	1.005
3688	NM_002211,NM_03	**ITGB1**	integrin subunit beta 1	protein_coding	62	52	63	53	0.838
3689	NM_000211,NM_00	**ITGB2**	integrin subunit beta 2	protein_coding	431	427	432	428	0.991
3690	NM_000212	**ITGB3**	integrin subunit beta 3	protein_coding	0	0	1	1	1.000
3691	NM_000213,NM_00	**ITGB4**	integrin subunit beta 4	protein_coding	0	0	1	1	1.000
3692	NM_001267810,NM	**EIF6**	eukaryotic translation init	protein_coding	455	461	456	462	1.013
3693	NM_001354764,NM	**ITGB5**	integrin subunit beta 5	protein_coding	9	7	10	8	0.857
3694	NM_000888,NM_00	**ITGB6**	integrin subunit beta 6	protein_coding	0	0	1	1	1.000
3695	NM_000889,NR_10	**ITGB7**	integrin subunit beta 7	protein_coding	157	160	158	161	1.021
3696	NM_002214,XM_01	**ITGB8**	integrin subunit beta 8	protein_coding	0	0	1	1	1.000
3697	NM_001166434,NM	**ITIH1**	inter-alpha-trypsin inhibit	protein_coding	0	0	1	1	1.000
3698	NM_002216	**ITIH2**	inter-alpha-trypsin inhibit	protein_coding	0	0	1	1	1.000
3699	NM_002217,XM_00	**ITIH3**	inter-alpha-trypsin inhibit	protein_coding	0	0	1	1	1.000
3700	NM_001166449,NM	**ITIH4**	inter-alpha-trypsin inhibit	protein_coding	1	1	2	2	1.087
3702	NM_005546,XM_01	**ITK**	IL2 inducible T cell kinas	protein_coding	72	69	73	70	0.956
3703	NM_001278503,NM	**STT3A**	STT3 oligosaccharyltrans	protein_coding	108	101	109	102	0.935
3704	NM_001267623,NM	**ITPA**	inosine triphosphatase	protein_coding	150	150	151	151	0.999
3705	NM_001142593,NM	**ITPK1**	inositol-tetrakisphosphat	protein_coding	79	88	80	89	1.103
3706	NM_002220,XM_01	**ITPKA**	inositol-trisphosphate 3-k	protein_coding	0	0	1	1	1.000
3707	NM_002221,XM_00	**ITPKB**	inositol-trisphosphate 3-k	protein_coding	10	8	11	9	0.868
3708	NM_001099952,NM	**ITPR1**	inositol 1,4,5-trisphospha	protein_coding	22	16	23	17	0.745
3709	NM_002223,XM_01	**ITPR2**	inositol 1,4,5-trisphospha	protein_coding	2	2	3	3	0.872
3710	NM_002224,XM_01	**ITPR3**	inositol 1,4,5-trisphospha	protein_coding	49	45	50	46	0.921
3712	NM_001159508,NM	**IVD**	isovaleryl-CoA dehydrog	protein_coding	53	55	54	56	1.031
3713	NM_005547	**IVL**	involucrin	protein_coding	0	0	1	1	1.000
3714	NM_002226,NM_14	**JAG2**	jagged canonical Notch l	protein_coding	0	0	1	1	1.000
3716	NM_001320923,NM	**JAK1**	Janus kinase 1	protein_coding	115	102	116	103	0.891
3717	NM_001322194,NM	**JAK2**	Janus kinase 2	protein_coding	5	5	6	6	0.968
3718	NM_000215,XM_01	**JAK3**	Janus kinase 3	protein_coding	20	22	21	23	1.076
3720	NM_001267040,NM	**JARID2**	jumonji and AT-rich inter	protein_coding	22	20	23	21	0.903
3725	NM_002228	**JUN**	Jun proto-oncogene, AP	protein_coding	19	21	20	22	1.112
3726	NM_002229	**JUNB**	JunB proto-oncogene, A	protein_coding	48	58	49	59	1.194
3727	NM_001286968,NM	**JUND**	JunD proto-oncogene, A	protein_coding	167	170	168	171	1.021
3728	NM_001352773,NM	**JUP**	junction plakoglobin	protein_coding	0	0	1	1	1.064
3730	NM_000216,XM_00	**ANOS1**	anosmin 1	protein_coding	0	0	1	1	1.000
3732	NM_001024844,NM	**CD82**	CD82 molecule	protein_coding	0	0	1	1	1.075
3734	gene-ANOS2P	**ANOS2P**	anosmin 2, pseudogene	pseudogene	0	0	1	1	0.985
3735	NM_001130089,NM	**KARS1**	lysyl-tRNA synthetase 1	protein_coding	340	320	341	321	0.943
3736	NM_000217	**KCNA1**	potassium voltage-gated	protein_coding	0	0	1	1	1.000
3737	NM_001204269,NM	**KCNA2**	potassium voltage-gated	protein_coding	2	1	3	2	0.813
3738	NM_002232,NR_10	**KCNA3**	potassium voltage-gated	protein_coding	141	113	142	114	0.803
3739	NM_002233	**KCNA4**	potassium voltage-gated	protein_coding	0	0	1	1	1.000
3741	NM_002234	**KCNA5**	potassium voltage-gated	protein_coding	0	0	1	1	1.000
3742	NM_002235,XM_00	**KCNA6**	potassium voltage-gated	protein_coding	0	0	1	1	1.000
3743	NM_031886	**KCNA7**	potassium voltage-gated	protein_coding	0	0	1	1	1.000
3744	NM_005549	**KCNA10**	potassium voltage-gated	protein_coding	0	0	1	1	1.000
3745	NM_004975,XM_00	**KCNB1**	potassium voltage-gated	protein_coding	0	0	1	1	1.000
3746	NM_001112741,NM	**KCNC1**	potassium voltage-gated	protein_coding	0	0	1	1	1.015
3747	NM_001260497,NM	**KCNC2**	potassium voltage-gated	protein_coding	0	0	1	1	1.000
3748	NM_001372305,NM	**KCNC3**	potassium voltage-gated	protein_coding	0	0	1	1	1.017
3749	NM_001039574,NM	**KCNC4**	potassium voltage-gated	protein_coding	3	2	4	3	0.784
3750	NM_004979,XM_01	**KCND1**	potassium voltage-gated	protein_coding	0	0	1	1	1.004
3751	NM_012281,XM_01	**KCND2**	potassium voltage-gated	protein_coding	0	0	1	1	1.001
3752	NM_001378969,NM	**KCND3**	potassium voltage-gated	protein_coding	0	0	1	1	1.000
3753	NM_000219,NM_00	**KCNE1**	potassium voltage-gated	protein_coding	2	1	3	2	0.823
3754	NM_002236	**KCNF1**	potassium voltage-gated	protein_coding	0	0	1	1	1.000
3755	NM_002237,XM_00	**KCNG1**	potassium voltage-gated	protein_coding	0	0	1	1	1.000
3756	NM_002238,NM_17	**KCNH1**	potassium voltage-gated	protein_coding	0	0	1	1	1.000
3757	NM_000238,NM_00	**KCNH2**	potassium voltage-gated	protein_coding	0	0	1	1	1.000
3758	NM_000220,NM_15	**KCNJ1**	potassium inwardly rectif	protein_coding	0	0	1	1	1.000
3759	NM_000891	**KCNJ2**	potassium inwardly rectif	protein_coding	0	0	1	1	1.000
3760	NM_001260508,NM	**KCNJ3**	potassium inwardly rectif	protein_coding	0	0	1	1	1.000
3761	NM_004981,NM_15	**KCNJ4**	potassium inwardly rectif	protein_coding	0	0	1	1	1.000
3762	NM_000890,NM_00	**KCNJ5**	potassium inwardly rectif	protein_coding	0	0	1	1	1.000
3763	NM_002240	**KCNJ6**	potassium inwardly rectif	protein_coding	0	0	1	1	1.000
3764	NM_004982,XM_00	**KCNJ8**	potassium inwardly rectif	protein_coding	0	0	1	1	1.000
3765	NM_004983	**KCNJ9**	potassium inwardly rectif	protein_coding	0	0	1	1	1.000
3766	NM_002241	**KCNJ10**	potassium inwardly rectif	protein_coding	0	0	1	1	1.000
3767	NM_000525,NM_00	**KCNJ11**	potassium inwardly rectif	protein_coding	0	0	1	1	0.988
3768	NM_021012,XM_00	**KCNJ12**	potassium inwardly rectif	protein_coding	0	0	1	1	1.000
3769	NM_001172416,NM	**KCNJ13**	potassium inwardly rectif	protein_coding	0	0	1	1	1.000
3770	NM_013348	**KCNJ14**	potassium inwardly rectif	protein_coding	1	1	2	2	1.153
3772	NM_001276435,NM	**KCNJ15**	potassium inwardly rectif	protein_coding	1	1	2	2	1.081
3773	NM_001270422,NM	**KCNJ16**	potassium inwardly rectif	protein_coding	0	0	1	1	1.000
3775	NM_002245,XM_01	**KCNK1**	potassium two pore dom	protein_coding	0	0	1	1	1.000
3776	NM_001017424,NM	**KCNK2**	potassium two pore dom	protein_coding	0	0	1	1	1.000
3777	NM_002246,XM_00	**KCNK3**	potassium two pore dom	protein_coding	0	0	1	1	1.000
3778	NM_001014797,NM	**KCNMA1**	potassium calcium-activa	protein_coding	0	0	1	1	1.000
3779	NM_004137	**KCNMB1**	potassium calcium-activa	protein_coding	0	0	1	1	1.000
3780	NM_002248,XM_00	**KCNN1**	potassium calcium-activa	protein_coding	0	0	1	1	1.000
3781	NM_001278204,NM	**KCNN2**	potassium calcium-activa	protein_coding	0	0	1	1	1.000
3782	NM_001204087,NM	**KCNN3**	potassium calcium-activa	protein_coding	0	0	1	1	1.000
3783	NM_002250,XM_00	**KCNN4**	potassium calcium-activa	protein_coding	0	0	1	1	1.000
3784	NM_000218,NM_18	**KCNQ1**	potassium voltage-gated	protein_coding	0	0	1	1	1.000
3785	NM_001382235,NM	**KCNQ2**	potassium voltage-gated	protein_coding	0	0	1	1	1.000
3786	NM_001204824,NM	**KCNQ3**	potassium voltage-gated	protein_coding	0	0	1	1	1.000
3787	NM_001322799,NM	**KCNS1**	potassium voltage-gated	protein_coding	0	0	1	1	1.000
3788	NM_020697	**KCNS2**	potassium voltage-gated	protein_coding	0	0	1	1	1.000
3790	NM_001282428,NM	**KCNS3**	potassium voltage-gated	protein_coding	0	0	1	1	1.000
3791	NM_002253	**KDR**	kinase insert domain rec	protein_coding	0	0	1	1	1.000
3792	NM_000420,XM_00	**KEL**	Kell metallo-endopeptida	protein_coding	0	0	1	1	1.000
3795	NM_000221,NM_00	**KHK**	ketohexokinase	protein_coding	3	2	4	3	0.823
3796	NM_001098511,NM	**KIF2A**	kinesin family member 2	protein_coding	143	124	144	125	0.867
3797	NM_002254,XM_00	**KIF3C**	kinesin family member 3	protein_coding	1	1	2	2	1.061
3798	NM_001354705,NM	**KIF5A**	kinesin family member 5	protein_coding	0	0	1	1	1.000
3799	NM_004521,XM_01	**KIF5B**	kinesin family member 5	protein_coding	33	28	34	29	0.847
3800	NM_004522,NR_11	**KIF5C**	kinesin family member 5	protein_coding	0	0	1	1	1.014
3801	NM_001130099,NM	**KIFC3**	kinesin family member C	protein_coding	0	0	1	1	1.000
3802	NM_014218,XM_01	**KIR2DL1**	killer cell immunoglobuli	protein_coding	0	0	1	1	1.000
3804	NM_015868	**KIR2DL3**	killer cell immunoglobuli	protein_coding	0	0	1	1	0.859
3805	NM_001080770,NM	**KIR2DL4**	killer cell immunoglobuli	protein_coding	109	96	110	97	0.887
3809	NM_001281971,NM	**KIR2DS4**	killer cell immunoglobuli	protein_coding	0	0	1	1	1.127
3811	NM_013289	**KIR3DL1**	killer cell immunoglobuli	protein_coding	1	1	2	2	0.950
3812	NM_001242867,NM	**KIR3DL2**	killer cell immunoglobuli	protein_coding	0	0	1	1	1.000
3814	NM_002256	**KISS1**	KiSS-1 metastasis suppr	protein_coding	0	0	1	1	1.000

3815	NM_000222,NM_00	**KIT**	KIT proto-oncogene, rec	protein_coding	0	0	1	1	0.931
3816	NM_002257,XM_01	**KLK1**	kallikrein 1	protein_coding	0	0	1	1	1.000
3817	NM_001002231,NM	**KLK2**	kallikrein related peptida	protein_coding	0	0	1	1	1.000
3818	NM_000892,NM_00	**KLKB1**	kallikrein B1	protein_coding	0	0	1	1	1.049
3820	NM_002258	**KLRB1**	killer cell lectin like recep	protein_coding	0	0	1	1	1.000
3821	NM_001304448,NM	**KLRC1**	killer cell lectin like recep	protein_coding	202	182	203	183	0.901
3822	NM_002260	**KLRC2**	killer cell lectin like recep	protein_coding	10	10	11	11	0.971
3823	NM_002261,NM_00	**KLRC3**	killer cell lectin like recep	protein_coding	3	4	4	5	1.202
3824	NM_001114396,NM	**KLRD1**	killer cell lectin like recep	protein_coding	8	7	9	8	0.905
3827	NM_000893,NM_00	**KNG1**	kininogen 1	protein_coding	0	0	1	1	1.000
3831	NM_001130107,NM	**KLC1**	kinesin light chain 1	protein_coding	66	59	67	60	0.892
3832	NM_004523	**KIF11**	kinesin family member 1	protein_coding	89	79	90	80	0.883
3833	NM_002263,XM_01	**KIFC1**	kinesin family member C	protein_coding	173	185	174	186	1.069
3834	NM_005355,NM_03	**KIF25**	kinesin family member 2	protein_coding	0	0	1	1	1.000
3835	NM_001256269,NM	**KIF22**	kinesin family member 2	protein_coding	158	161	159	162	1.023
3836	NM_002264,NR_02	**KPNA1**	karyopherin subunit alph	protein_coding	54	39	55	40	0.729
3837	NM_001276453,NM	**KPNB1**	karyopherin subunit beta	protein_coding	385	354	386	355	0.918
3838	NM_001320611,NM	**KPNA2**	karyopherin subunit alph	protein_coding	607	618	608	619	1.019
3839	NM_002267,XM_01	**KPNA3**	karyopherin subunit alph	protein_coding	113	98	114	99	0.876
3840	NM_002268	**KPNA4**	karyopherin subunit alph	protein_coding	50	45	51	46	0.909
3841	NM_001366304,NM	**KPNA5**	karyopherin subunit alph	protein_coding	3	3	4	4	0.919
3842	NM_001364292,NM	**TNPO1**	transportin 1	protein_coding	88	71	89	72	0.817
3843	NM_002271,XM_00	**IPO5**	importin 5	protein_coding	229	205	230	206	0.896
3844	gene-KRASP1	**KRASP1**	KRAS proto-oncogene, G	pseudogene	0	0	1	1	0.968
3845	NM_001369786,NM	**KRAS**	KRAS proto-oncogene, G	protein_coding	67	56	68	57	0.839
3846	NM_005553	**KRTAP5-9**	keratin associated protei	protein_coding	0	0	1	1	1.000
3848	NM_006121	**KRT1**	keratin 1	protein_coding	0	0	1	1	1.000
3849	NM_000423	**KRT2**	keratin 2	protein_coding	0	0	1	1	1.000
3850	NM_057088,XM_02	**KRT3**	keratin 3	protein_coding	0	0	1	1	1.000
3851	NM_002272	**KRT4**	keratin 4	protein_coding	0	0	1	1	1.000
3852	NM_000424	**KRT5**	keratin 5	protein_coding	1	1	2	2	1.226
3853	NM_005554	**KRT6A**	keratin 6A	protein_coding	0	0	1	1	1.000
3854	NM_005555	**KRT6B**	keratin 6B	protein_coding	0	0	1	1	1.000
3855	NM_005556,XM_01	**KRT7**	keratin 7	protein_coding	0	0	1	1	1.000
3856	NM_001256282,NM	**KRT8**	keratin 8	protein_coding	0	0	1	1	0.957
3857	NM_000226	**KRT9**	keratin 9	protein_coding	0	0	1	1	1.000
3858	NM_000421,NM_00	**KRT10**	keratin 10	protein_coding	12	13	13	14	1.116
3859	NM_000223	**KRT12**	keratin 12	protein_coding	0	0	1	1	1.000
3860	NM_002274,NM_15	**KRT13**	keratin 13	protein_coding	0	0	1	1	1.000
3861	NM_000526	**KRT14**	keratin 14	protein_coding	0	0	1	1	1.000
3866	NM_002275,XM_01	**KRT15**	keratin 15	protein_coding	0	0	1	1	1.000
3868	NM_005557	**KRT16**	keratin 16	protein_coding	0	0	1	1	1.000
3872	NM_000422	**KRT17**	keratin 17	protein_coding	0	0	1	1	1.000
3875	NM_000224,NM_19	**KRT18**	keratin 18	protein_coding	0	0	1	1	1.000
3876	gene-KRT18P11	**KRT18P11**	keratin 18 pseudogene 1	pseudogene	0	0	1	1	1.000
3879	gene-KRT18P1	**KRT18P1**	keratin 18 pseudogene 1	pseudogene	0	0	1	1	1.000
3880	NM_002276	**KRT19**	keratin 19	protein_coding	0	0	1	1	1.000
3881	NM_002277	**KRT31**	keratin 31	protein_coding	0	0	1	1	1.000
3882	NM_002278,XR_93	**KRT32**	keratin 32	protein_coding	0	0	1	1	1.000
3883	NM_004138,XM_01	**KRT33A**	keratin 33A	protein_coding	0	0	1	1	1.000
3884	NM_002279	**KRT33B**	keratin 33B	protein_coding	0	0	1	1	1.000
3885	NM_021013,XM_01	**KRT34**	keratin 34	protein_coding	0	0	1	1	1.000
3886	NM_002280,XM_01	**KRT35**	keratin 35	protein_coding	0	0	1	1	1.000
3887	NM_002281	**KRT81**	keratin 81	protein_coding	138	115	139	116	0.831
3888	NM_033033	**KRT82**	keratin 82	protein_coding	0	0	1	1	1.000
3889	NM_002282	**KRT83**	keratin 83	protein_coding	0	0	1	1	0.995
3890	NM_033045,XM_01	**KRT84**	keratin 84	protein_coding	0	0	1	1	1.000
3891	NM_001300810,NM	**KRT85**	keratin 85	protein_coding	0	0	1	1	0.957
3892	NM_001320198,XM	**KRT86**	keratin 86	protein_coding	23	20	24	21	0.874
3895	NM_001079521,NM	**KTN1**	kinectin 1	protein_coding	62	53	63	54	0.856
3897	NM_000425,NM_00	**L1CAM**	L1 cell adhesion molecul	protein_coding	0	0	1	1	1.000
3898	NM_005558	**LAD1**	ladinin 1	protein_coding	0	0	1	1	1.000
3899	NM_001025108,NM	**AFF3**	AF4/FMR2 family membe	protein_coding	0	0	1	1	1.000
3902	NM_002286,XM_01	**LAG3**	lymphocyte activating 3	protein_coding	14	15	15	16	1.077
3903	NM_001289023,NM	**LAIR1**	leukocyte associated imr	protein_coding	0	0	1	1	1.055
3904	NM_002288,NM_02	**LAIR2**	leukocyte associated imr	protein_coding	25	31	26	32	1.239
3906	NM_002289,XM_00	**LALBA**	lactalbumin alpha	protein_coding	0	0	1	1	1.000
3908	NM_000426,NM_00	**LAMA2**	laminin subunit alpha 2	protein_coding	0	0	1	1	0.961
3909	NM_000227,NM_00	**LAMA3**	laminin subunit alpha 3	protein_coding	0	0	1	1	0.948
3910	NM_001105206,NM	**LAMA4**	laminin subunit alpha 4	protein_coding	0	0	1	1	1.000
3911	NM_005560,XM_00	**LAMA5**	laminin subunit alpha 5	protein_coding	0	0	1	1	1.000
3912	NM_002291,XM_01	**LAMB1**	laminin subunit beta 1	protein_coding	0	0	1	1	1.000
3913	NM_002292,XM_00	**LAMB2**	laminin subunit beta 2	protein_coding	0	0	1	1	1.000
3914	NM_000228,NM_00	**LAMB3**	laminin subunit beta 3	protein_coding	1	1	2	2	0.905
3915	NM_002293	**LAMC1**	laminin subunit gamma 1	protein_coding	0	0	1	1	1.000
3916	NM_005561,XM_01	**LAMP1**	lysosomal associated me	protein_coding	98	106	99	107	1.086
3918	NM_005562,NM_01	**LAMC2**	laminin subunit gamma 2	protein_coding	0	0	1	1	1.000
3920	NM_001122606,NM	**LAMP2**	lysosomal associated me	protein_coding	72	64	73	65	0.886
3921	NM_001304288,NM	**RPSA**	ribosomal protein SA	protein_coding	1,868	1,881	1,869	1,882	1.007
3923	gene-RPSAP13	**RPSAP13**	ribosomal protein SA pse	pseudogene	0	0	1	1	0.983
3924	gene-RPSAP14	**RPSAP14**	ribosomal protein SA pse	pseudogene	0	0	1	1	1.078
3925	NM_001145454,NM	**STMN1**	stathmin 1	protein_coding	509	511	510	512	1.003
3927	NM_001271608,NM	**LASP1**	LIM and SH3 protein 1	protein_coding	439	443	440	444	1.009
3929	NM_004139	**LBP**	lipopolysaccharide bindi	protein_coding	0	0	1	1	1.000
3930	NM_002296,NM_19	**LBR**	lamin B receptor	protein_coding	278	241	279	242	0.867
3931	NM_000229	**LCAT**	lecithin-cholesterol acylt	protein_coding	0	0	1	1	1.029
3932	NM_001042771,NM	**LCK**	LCK proto-oncogene, Sr	protein_coding	75	74	76	75	0.984
3933	NM_001252617,NM	**LCN1**	lipocalin 1	protein_coding	0	0	1	1	1.000
3934	NM_005564	**LCN2**	lipocalin 2	protein_coding	0	0	1	1	1.000
3936	NM_002298,XM_00	**LCP1**	lymphocyte cytosolic pro	protein_coding	1,500	1,358	1,501	1,359	0.905
3937	NM_005565	**LCP2**	lymphocyte cytosolic pro	protein_coding	182	173	183	174	0.949
3938	NM_002299,XM_01	**LCT**	lactase	protein_coding	1	0	2	1	0.858
3939	NM_001135239,NM	**LDHA**	lactate dehydrogenase A	protein_coding	2,120	1,945	2,121	1,946	0.918
3940	gene-LDHAP1	**LDHAP1**	lactate dehydrogenase A	pseudogene	0	0	1	1	1.000
3945	NM_001174097,NM	**LDHB**	lactate dehydrogenase B	protein_coding	856	827	857	828	0.966
3947	gene-LDHBP2	**LDHBP2**	lactate dehydrogenase B	pseudogene	0	0	1	1	1.010
3948	NM_002301,NM_01	**LDHC**	lactate dehydrogenase C	protein_coding	10	10	11	11	0.982
3949	NM_000527,NM_00	**LDLR**	low density lipoprotein re	protein_coding	68	68	69	69	0.998
3950	NM_002302	**LECT2**	leukocyte cell derived ch	protein_coding	0	0	1	1	1.000
3952	NM_000230,XM_00	**LEP**	leptin	protein_coding	49	47	50	48	0.942
3953	NM_001003679,NM	**LEPR**	leptin receptor	protein_coding	3	3	4	4	0.836
3954	NM_012318,XM_00	**LETM1**	leucine zipper and EF-ha	protein_coding	48	49	49	50	1.019
3955	NM_001040167,NM	**LFNG**	LFNG O-fucosylpeptide	protein_coding	13	13	14	14	1.038
3956	NM_002305	**LGALS1**	galectin 1	protein_coding	2,417	2,746	2,418	2,747	1.136
3957	NM_006498	**LGALS2**	galectin 2	protein_coding	0	0	1	1	1.000
3958	NM_001357678,NM	**LGALS3**	galectin 3	protein_coding	62	62	63	63	1.000
3959	NM_005567	**LGALS3BP**	galectin 3 binding protei	protein_coding	83	83	84	84	1.001
3960	NM_006149,XM_01	**LGALS4**	galectin 4	protein_coding	0	0	1	1	1.250
3963	NM_002307	**LGALS7**	galectin 7	protein_coding	0	0	1	1	1.000
3964	NM_006499,NM_20	**LGALS8**	galectin 8	protein_coding	18	18	19	19	0.999
3965	NM_001330163,NM	**LGALS9**	galectin 9	protein_coding	3	4	4	5	1.160
3972	NM_000894	**LHB**	luteinizing hormone subu	protein_coding	0	0	1	1	1.000
3973	NM_000233,XM_00	**LHCGR**	luteinizing hormone/chor	protein_coding	0	0	1	1	1.000
3975	NM_005568	**LHX1**	LIM homeobox 1	protein_coding	0	0	1	1	1.000
3976	NM_001257135,NM	**LIF**	LIF interleukin 6 family c	protein_coding	0	0	1	1	1.000
3977	NM_001127671,NM	**LIFR**	LIF receptor subunit alph	protein_coding	0	0	1	1	1.021
3978	NM_000234,NM_00	**LIG1**	DNA ligase 1	protein_coding	81	84	82	85	1.039
3980	NM_002311,NM_01	**LIG3**	DNA ligase 3	protein_coding	27	24	28	25	0.898
3981	NM_001098268,NM	**LIG4**	DNA ligase 4	protein_coding	22	18	23	19	0.842
3982	NM_001161748,NM	**LIM2**	lens intrinsic membrane	protein_coding	61	78	62	79	1.272
3983	NM_001003407,NM	**ABLIM1**	actin binding LIM protein	protein_coding	43	36	44	37	0.844
3984	NM_001204426,NM	**LIMK1**	LIM domain kinase 1	protein_coding	102	102	103	103	0.999
3985	NM_001031801,NM	**LIMK2**	LIM domain kinase 2	protein_coding	29	23	30	24	0.820
3987	NM_001193482,NM	**LIMS1**	LIM zinc finger domain c	protein_coding	87	77	88	78	0.884
3988	NM_000235,NM_00	**LIPA**	lipase A, lysosomal acid	protein_coding	56	50	57	51	0.890
3990	NM_000236,XM_00	**LIPC**	lipase C, hepatic type	protein_coding	0	0	1	1	1.000

3991	NM_005357,XM_00!	**LIPE**	lipase E, hormone sensit	protein_coding	17	17	18	18	0.970
3992	NM_013402,XM_01	**FADS1**	fatty acid desaturase 1	protein_coding	2	2	3	3	0.974
3993	NM_001015002,NM	**LLGL2**	LLGL scribble cell polarit	protein_coding	0	0	1	1	0.991
3995	NM_021727,XM_01	**FADS3**	fatty acid desaturase 3	protein_coding	1	0	2	1	0.856
3996	NM_004140,XM_01	**LLGL1**	LLGL scribble cell polarit	protein_coding	12	13	13	14	1.027
3998	NM_005570	**LMAN1**	lectin, mannose binding	protein_coding	157	125	158	126	0.798
4000	NM_001257374,NM	**LMNA**	lamin A/C	protein_coding	0	1	1	2	1.216
4001	NM_001198557,NM	**LMNB1**	lamin B1	protein_coding	554	514	555	515	0.928
4004	NM_001270428,NM	**LMO1**	LIM domain only 1	protein_coding	0	0	1	1	1.000
4005	NM_001142315,NM	**LMO2**	LIM domain only 2	protein_coding	0	0	1	1	1.000
4007	NM_001307979,NM	**PRICKLE3**	prickle planar cell polarit	protein_coding	0	0	1	1	1.002
4008	NM_001306080,NM	**LMO7**	LIM domain 7	protein_coding	3	3	4	4	1.103
4009	NM_001174069,NM	**LMX1A**	LIM homeobox transcript	protein_coding	0	0	1	1	1.000
4010	NM_001174146,NM	**LMX1B**	LIM homeobox transcript	protein_coding	0	0	1	1	1.000
4012	NM_005575,NM_17	**LNPEP**	leucyl and cystinyl aminc	protein_coding	81	64	82	65	0.793
4013	NM_001130142,NM	**VWA5A**	von Willebrand factor A c	protein_coding	1	1	2	2	1.278
4014	NM_000427,XM_02	**LORICRIN**	loricrin cornified envelop	protein_coding	0	0	1	1	1.000
4015	NM_001178102,NM	**LOX**	lysyl oxidase	protein_coding	0	0	1	1	1.000
4016	NM_005576,XM_01	**LOXL1**	lysyl oxidase like 1	protein_coding	0	0	1	1	1.159
4017	NM_002318	**LOXL2**	lysyl oxidase like 2	protein_coding	0	0	1	1	1.021
4018	NM_005577	**LPA**	lipoprotein(a)	protein_coding	0	0	1	1	1.000
4023	NM_000237	**LPL**	lipoprotein lipase	protein_coding	0	0	1	1	1.000
4025	NM_001160102,NM	**LPO**	lactoperoxidase	protein_coding	0	0	1	1	1.000
4026	NM_001167671,NM	**LPP**	LIM domain containing p	protein_coding	2	2	3	3	0.918
4033	NM_001204126,NM	**IRAG2**	inositol 1,4,5-triphosphat	protein_coding	4	6	5	7	1.296
4034	NM_001289934,NM	**LRCH4**	leucine rich repeats and	protein_coding	46	45	47	46	0.979
4035	NM_002332,XM_01	**LRP1**	LDL receptor related pro	protein_coding	0	0	1	1	1.036
4036	NM_004525,XM_01	**LRP2**	LDL receptor related pro	protein_coding	0	0	1	1	0.996
4037	NM_002333,XM_00!	**LRP3**	LDL receptor related pro	protein_coding	0	0	1	1	1.000
4038	NM_002334,XM_01	**LRP4**	LDL receptor related pro	protein_coding	0	0	1	1	1.011
4040	NM_002336,XM_00	**LRP6**	LDL receptor related pro	protein_coding	0	0	1	1	1.000
4041	NM_001291902,NM	**LRP5**	LDL receptor related pro	protein_coding	0	0	1	1	1.000
4043	NM_002337,NR_11	**LRPAP1**	LDL receptor related pro	protein_coding	28	29	29	30	1.030
4045	NM_001318915,NM	**LSAMP**	limbic system associated	protein_coding	0	0	1	1	1.000
4046	NM_001013253,NM	**LSP1**	lymphocyte specific prote	protein_coding	820	876	821	877	1.069
4047	NM_001001438,NM	**LSS**	lanosterol synthase	protein_coding	14	15	15	16	1.068
4048	NM_000895,NM_00	**LTA4H**	leukotriene A4 hydrolase	protein_coding	71	67	72	68	0.955
4049	NM_000595,NM_00	**LTA**	lymphotoxin alpha	protein_coding	389	367	390	368	0.942
4050	NM_002341,NM_00	**LTB**	lymphotoxin beta	protein_coding	792	855	793	856	1.079
4051	NM_000896,NM_00	**CYP4F3**	cytochrome P450 family	protein_coding	0	0	1	1	1.000
4052	NM_000627,NM_00	**LTBP1**	latent transforming grow	protein_coding	0	0	1	1	1.026
4053	NM_000428,XM_01	**LTBP2**	latent transforming grow	protein_coding	0	0	1	1	0.990
4054	NM_001130144,NM	**LTBP3**	latent transforming grow	protein_coding	1	1	2	2	1.119
4055	NM_001270987,NM	**LTBR**	lymphotoxin beta recepto	protein_coding	0	0	1	1	1.000
4056	NM_145867	**LTC4S**	leukotriene C4 synthase	protein_coding	0	0	1	1	1.000
4057	NM_001199149,NM	**LTF**	lactotransferrin	protein_coding	0	0	1	1	1.000
4058	NM_001135685,NM	**LTK**	leukocyte receptor tyrosi	protein_coding	0	0	1	1	1.127
4059	NM_001013257,NM	**BCAM**	basal cell adhesion mole	protein_coding	0	0	1	1	1.000
4060	NM_002345	**LUM**	lumican	protein_coding	0	0	1	1	1.000
4061	NM_001127213,NM	**LY6E**	lymphocyte antigen 6 far	protein_coding	475	497	476	498	1.046
4062	NM_001130478,NM	**LY6H**	lymphocyte antigen 6 far	protein_coding	0	0	1	1	1.000
4063	NM_001033667,NM	**LY9**	lymphocyte antigen 9	protein_coding	1	2	2	3	1.228
4064	NM_005582,XM_00!	**CD180**	CD180 molecule	protein_coding	0	0	1	1	0.966
4065	NM_002349	**LY75**	lymphocyte antigen 75	protein_coding	16	11	17	12	0.742
4066	NM_005583	**LYL1**	LYL1 basic helix-loop-he	protein_coding	0	0	1	1	1.000
4067	NM_001111097,NM	**LYN**	LYN proto-oncogene, Sr	protein_coding	74	68	75	69	0.917
4068	NM_001114937,NM	**SH2D1A**	SH2 domain containing 1	protein_coding	246	245	247	246	0.994
4069	NM_000239	**LYZ**	lysozyme	protein_coding	0	0	1	1	0.939
4070	NM_002353	**TACSTD2**	tumor associated calciun	protein_coding	0	0	1	1	1.000
4071	NM_014220,XM_01	**TM4SF1**	transmembrane 4 L six fa	protein_coding	0	0	1	1	1.000
4072	NM_002354	**EPCAM**	epithelial cell adhesion n	protein_coding	0	0	1	1	1.000
4074	NM_001207024,NM	**M6PR**	mannose-6-phosphate re	protein_coding	136	126	137	127	0.932
4076	NM_005898,NM_20	**CAPRIN1**	cell cycle associated pro	protein_coding	249	241	250	242	0.966
4077	NM_001291571,NM	**NBR1**	NBR1 autophagy cargo r	protein_coding	42	38	43	39	0.912
4081	NM_005584	**MAB21L1**	mab-21 like 1	protein_coding	0	0	1	1	1.000
4082	NM_002356	**MARCKS**	myristoylated alanine ricl	protein_coding	0	0	1	1	1.000
4083	gene-MARCKSP1	**MARCKSP1**	myristoylated alanine ricl	pseudogene	0	0	1	1	1.000
4084	NM_001202513,NM	**MXD1**	MAX dimerization protein	protein_coding	5	5	6	6	1.062
4085	NM_002358	**MAD2L1**	mitotic arrest deficient 2	protein_coding	65	60	66	61	0.930
4086	NM_001003688,NM	**SMAD1**	SMAD family member 1	protein_coding	0	0	1	1	1.000
4087	NM_001003652,NM	**SMAD2**	SMAD family member 2	protein_coding	16	14	17	15	0.906
4088	NM_001145102,NM	**SMAD3**	SMAD family member 3	protein_coding	7	9	8	10	1.183
4089	NM_005359	**SMAD4**	SMAD family member 4	protein_coding	12	10	13	11	0.856
4090	NM_001001419,NM	**SMAD5**	SMAD family member 5	protein_coding	11	9	12	10	0.826
4091	NM_005585,NR_02	**SMAD6**	SMAD family member 6	protein_coding	1	1	2	2	0.992
4092	NM_001190821,NM	**SMAD7**	SMAD family member 7	protein_coding	15	13	16	14	0.859
4093	NM_001127217,NM	**SMAD9**	SMAD family member 9	protein_coding	0	0	1	1	1.000
4094	NM_001031804,NM	**MAF**	MAF bZIP transcription fa	protein_coding	0	0	1	1	1.055
4097	NM_002359,NM_03	**MAFG**	MAF bZIP transcription fa	protein_coding	13	11	14	12	0.824
4099	NM_001199216,NM	**MAG**	myelin associated glycop	protein_coding	0	0	1	1	1.000
4100	NM_004988	**MAGEA1**	MAGE family member A1	protein_coding	0	0	1	1	1.000
4101	NM_001282501,NM	**MAGEA2**	MAGE family member A2	protein_coding	0	0	1	1	0.977
4102	NM_005362,XM_00!	**MAGEA3**	MAGE family member A3	protein_coding	0	0	1	1	1.000
4103	NM_001011548,NM	**MAGEA4**	MAGE family member A4	protein_coding	0	0	1	1	1.000
4104	NM_021049	**MAGEA5**	MAGE family member A5	protein_coding	0	0	1	1	1.000
4105	NM_005363,NM_17	**MAGEA6**	MAGE family member A6	protein_coding	0	0	1	1	1.000
4106	gene-MAGEA7P	**MAGEA7P**	MAGE family member A7	pseudogene	0	0	1	1	1.000
4107	NM_001166400,NM	**MAGEA8**	MAGE family member A8	protein_coding	0	0	1	1	1.000
4108	NM_005365,XM_00!	**MAGEA9**	MAGE family member A9	protein_coding	0	0	1	1	1.078
4109	NM_001011543,NM	**MAGEA10**	MAGE family member A1	protein_coding	0	0	1	1	1.000
4110	NM_001011544,NM	**MAGEA11**	MAGE family member A1	protein_coding	0	0	1	1	1.000
4111	NM_001166386,NM	**MAGEA12**	MAGE family member A1	protein_coding	0	0	1	1	1.000
4112	NM_002363,NM_17	**MAGEB1**	MAGE family member B1	protein_coding	0	0	1	1	1.000
4113	NM_002364,XM_01	**MAGEB2**	MAGE family member B2	protein_coding	0	0	1	1	1.000
4114	NM_002365,XM_01	**MAGEB3**	MAGE family member B3	protein_coding	0	0	1	1	1.000
4115	NM_002367	**MAGEB4**	MAGE family member B4	protein_coding	0	0	1	1	1.000
4116	NM_002370	**MAGOH**	mago homolog, exon jun	protein_coding	443	447	444	448	1.009
4117	NM_001242385,NM	**MAK**	male germ cell associate	protein_coding	0	0	1	1	1.000
4118	NM_002371,NM_02	**MAL**	mal, T cell differentiation	protein_coding	3	3	4	4	0.987
4121	NM_005907,XM_00!	**MAN1A1**	mannosidase alpha clas:	protein_coding	81	62	82	63	0.761
4122	NM_001320977,NM	**MAN2A2**	mannosidase alpha clas:	protein_coding	0	0	1	1	0.928
4123	NM_001256494,NM	**MAN2C1**	mannosidase alpha clas:	protein_coding	11	11	12	12	0.949
4124	NM_002372,XM_01	**MAN2A1**	mannosidase alpha clas:	protein_coding	24	20	25	21	0.833
4125	NM_000528,NM_00	**MAN2B1**	mannosidase alpha clas:	protein_coding	30	28	31	29	0.908
4126	NM_005908,XM_01	**MANBA**	mannosidase beta	protein_coding	5	4	6	5	0.885
4128	NM_000240,NM_00	**MAOA**	monoamine oxidase A	protein_coding	0	0	1	1	1.000
4129	NM_000898,XM_00!	**MAOB**	monoamine oxidase B	protein_coding	0	0	1	1	1.000
4130	NM_002373	**MAP1A**	microtubule associated p	protein_coding	2	3	3	4	1.074
4131	NM_001324255,NM	**MAP1B**	microtubule associated p	protein_coding	0	0	1	1	0.992
4133	NM_001039538,NM	**MAP2**	microtubule associated p	protein_coding	0	0	1	1	1.013
4134	NM_001134364,NM	**MAP4**	microtubule associated p	protein_coding	55	54	56	55	0.999
4135	NM_033063,NM_20	**MAP6**	microtubule associated p	protein_coding	0	0	1	1	1.000
4137	NM_001123066,NM	**MAPT**	microtubule associated p	protein_coding	0	0	1	1	1.000
4139	NM_001286124,NM	**MARK1**	microtubule affinity regul	protein_coding	0	0	1	1	1.000
4140	NM_001128918,NM	**MARK3**	microtubule affinity regul	protein_coding	51	44	52	45	0.876
4141	NM_004990,XM_00	**MARS1**	methionyl-tRNA syntheta	protein_coding	168	154	169	155	0.917
4142	NM_001366704,NM	**MAS1**	MAS1 proto-oncogene, C	protein_coding	0	0	1	1	1.000
4143	NM_000429	**MAT1A**	methionine adenosyltran	protein_coding	0	0	1	1	0.930
4144	NM_005911	**MAT2A**	methionine adenosyltran	protein_coding	408	422	409	423	1.035
4145	NM_002378,NM_13	**MATK**	megakaryocyte-associate	protein_coding	82	89	83	90	1.090
4146	NM_002379	**MATN1**	matrilin 1	protein_coding	0	0	1	1	0.910
4147	NM_001317748,NM	**MATN2**	matrilin 2	protein_coding	0	0	1	1	1.000
4148	NM_002381	**MATN3**	matrilin 3	protein_coding	0	0	1	1	1.000
4149	NM_001271068,NM	**MAX**	MYC associated factor X	protein_coding	87	86	88	87	0.985
4150	NM_001042539,NM	**MAZ**	MYC associated zinc fing	protein_coding	347	388	348	389	1.120
4151	NM_001362846,NM	**MB**	myoglobin	protein_coding	0	0	1	1	1.000

4152	NM_001204136,NM	**MBD1**	methyl-CpG binding dom	protein_coding	71	62	72	63	0.873
4153	NM_000242,NM_00	**MBL2**	mannose binding lectin 2	protein_coding	0	0	1	1	1.000
4154	NM_001314057,NM	**MBNL1**	muscleblind like splicing	protein_coding	128	109	129	110	0.851
4155	NM_001025081,NM	**MBP**	myelin basic protein	protein_coding	68	68	69	69	1.003
4157	NM_002386	**MC1R**	melanocortin 1 receptor	protein_coding	2	3	3	4	1.355
4158	NM_000529,NM_00	**MC2R**	melanocortin 2 receptor	protein_coding	0	0	1	1	1.000
4159	NM_019888	**MC3R**	melanocortin 3 receptor	protein_coding	0	0	1	1	1.000
4160	NM_005912	**MC4R**	melanocortin 4 receptor	protein_coding	0	0	1	1	1.000
4161	NM_005913	**MC5R**	melanocortin 5 receptor	protein_coding	0	0	1	1	1.000
4162	NM_006500,XM_01	**MCAM**	melanoma cell adhesion	protein_coding	0	0	1	1	1.000
4163	NM_001085377,NM	**MCC**	MCC regulator of WNT s	protein_coding	0	0	1	1	1.000
4166	NM_021615,NR_16	**CHST6**	carbohydrate sulfotransf	protein_coding	0	0	1	1	1.000
4168	NM_001099855,NM	**MCF2**	MCF.2 cell line derived t	protein_coding	0	0	1	1	1.054
4170	NM_001197320,NM	**MCL1**	MCL1 apoptosis regulato	protein_coding	203	194	204	195	0.955
4171	NM_004526,NR_07	**MCM2**	minichromosome mainte	protein_coding	357	377	358	378	1.059
4172	NM_001270472,NM	**MCM3**	minichromosome mainte	protein_coding	478	469	479	470	0.983
4173	NM_005914,NM_18	**MCM4**	minichromosome mainte	protein_coding	391	372	392	373	0.951
4174	NM_006739,XM_00	**MCM5**	minichromosome mainte	protein_coding	168	178	169	179	1.056
4175	NM_005915	**MCM6**	minichromosome mainte	protein_coding	197	190	198	191	0.966
4176	NM_001278595,NM	**MCM7**	minichromosome mainte	protein_coding	661	678	662	679	1.026
4179	NM_002389,NM_15	**CD46**	CD46 molecule	protein_coding	52	43	53	44	0.830
4182	gene-CD46P1	**CD46P1**	CD46 molecule pseudog	pseudogene	0	0	1	1	1.000
4184	NM_030663	**SMCP**	sperm mitochondria ass	protein_coding	0	0	1	1	1.000
4185	NM_001318933,NM	**ADAM11**	ADAM metallopeptidase	protein_coding	0	0	1	1	0.964
4188	NM_001300804,NM	**MDFI**	MyoD family inhibitor	protein_coding	0	0	1	1	1.000
4189	NM_012328	**DNAJB9**	DnaJ heat shock protein	protein_coding	19	15	20	16	0.825
4190	NM_001199111,NM	**MDH1**	malate dehydrogenase 1	protein_coding	261	251	262	252	0.961
4191	NM_001282403,NM	**MDH2**	malate dehydrogenase 2	protein_coding	330	332	331	333	1.007
4192	NM_001012333,NM	**MDK**	midkine	protein_coding	3	6	4	7	1.466
4193	NM_001145337,NM	**MDM2**	MDM2 proto-oncogene	protein_coding	38	33	39	34	0.852
4194	NM_001204171,NM	**MDM4**	MDM4 regulator of p53	protein_coding	6	5	7	6	0.835
4199	NM_002395,XM_01	**ME1**	malic enzyme 1	protein_coding	0	0	1	1	0.951
4200	NM_001168335,NM	**ME2**	malic enzyme 2	protein_coding	56	55	57	56	0.980
4201	NM_001318942,NM	**MEA1**	male-enhanced antigen	protein_coding	204	236	205	237	1.154
4204	NM_001110792,NM	**MECP2**	methyl-CpG binding prot	protein_coding	21	20	22	21	0.941
4205	NM_001130926,NM	**MEF2A**	myocyte enhancer factor	protein_coding	43	35	44	36	0.822
4206	gene-MEF2AP1	**MEF2AP1**	myocyte enhancer factor	pseudogene	0	0	1	1	1.000
4207	NM_005919,NR_02	**BORCS8-MEF2B**	BORCS8-MEF2B readth	protein_coding	5	5	6	6	1.009
4208	NM_001131005,NM	**MEF2C**	myocyte enhancer factor	protein_coding	1	1	2	2	0.788
4209	NM_001271629,NM	**MEF2D**	myocyte enhancer factor	protein_coding	48	51	49	52	1.067
4210	NM_000243,NM_00	**MEFV**	MEFV innate immuity reg	protein_coding	1	0	2	1	0.927
4211	NM_002398	**MEIS1**	Meis homeobox 1	protein_coding	1	2	2	3	1.133
4212	NM_001220482,NM	**MEIS2**	Meis homeobox 2	protein_coding	0	0	1	1	1.000
4213	NR_002211	**MEIS3P1**	Meis homeobox 3 pseud	transcribed_pseu	0	0	1	1	1.000
4214	NM_005921,XM_01	**MAP3K1**	mitogen-activated protei	protein_coding	16	15	17	16	0.961
4215	NM_001330431,NM	**MAP3K3**	mitogen-activated protei	protein_coding	17	18	18	19	1.070
4216	NM_001291958,NM	**MAP3K4**	mitogen-activated protei	protein_coding	16	14	17	15	0.903
4217	NM_005923,XM_01	**MAP3K5**	mitogen-activated protei	protein_coding	6	7	7	8	1.050
4218	NM_005370	**RAB8A**	RAB8A, member RAS on	protein_coding	222	219	223	220	0.987
4221	NM_000244,NM_00	**MEN1**	menin 1	protein_coding	38	40	39	41	1.063
4222	NM_001040002,NM	**MEOX1**	mesenchyme homeobox	protein_coding	0	0	1	1	1.000
4223	NM_005924	**MEOX2**	mesenchyme homeobox	protein_coding	0	0	1	1	1.000
4224	NM_005588,XM_01	**MEP1A**	meprin A subunit alpha	protein_coding	0	0	1	1	1.000
4225	NM_001308171,NM	**MEP1B**	meprin A subunit beta	protein_coding	0	0	1	1	1.069
4232	NM_001253900,NM	**MEST**	mesoderm specific trans	protein_coding	0	0	1	1	1.000
4233	NM_000245,NM_00	**MET**	MET proto-oncogene, re	protein_coding	0	0	1	1	1.000
4234	NM_005371,NM_02	**METTL1**	methyltransferase like 1	protein_coding	28	28	29	29	1.007
4236	NM_005926	**MFAP1**	microfibril associated pro	protein_coding	142	139	143	140	0.979
4237	NM_001135247,NM	**MFAP2**	microfibril associated pro	protein_coding	0	0	1	1	1.000
4238	NM_001135037,NM	**MFAP3**	microfibril associated pro	protein_coding	36	29	37	30	0.814
4239	NM_001198695,NM	**MFAP4**	microfibril associated pro	protein_coding	0	0	1	1	1.000
4240	NM_001114614,NM	**MFGE8**	milk fat globule EGF anc	protein_coding	3	3	4	4	1.139
4241	NM_005929,NM_03	**MELTF**	melanotransferrin	protein_coding	0	0	1	1	1.064
4242	NM_001166343,NM	**MFNG**	MFNG O-fucosylpeptide	protein_coding	165	159	166	160	0.963
4245	NM_001114617,NM	**MGAT1**	alpha-1,3-mannosyl-glyc	protein_coding	91	87	92	88	0.956
4246	NM_002407	**SCGB2A1**	secretoglobin family 2A r	protein_coding	0	0	1	1	1.000
4247	NM_002408	**MGAT2**	alpha-1,6-mannosyl-glyc	protein_coding	97	93	98	94	0.954
4248	NM_001098270,NM	**MGAT3**	beta-1,4-mannosyl-glyco	protein_coding	0	0	1	1	0.930
4249	NM_001371457,NM	**MGAT5**	alpha-1,6-mannosylglyco	protein_coding	15	12	16	13	0.815
4250	NM_002411,XM_00	**SCGB2A2**	secretoglobin family 2A r	protein_coding	0	0	1	1	1.000
4253	NM_001247988,NM	**MIA2**	MIA SH3 domain ER exp	protein_coding	29	24	30	25	0.837
4254	NM_000899,NM_00	**KITLG**	KIT ligand	protein_coding	0	0	1	1	1.000
4255	NM_002412,XM_00	**MGMT**	O-6-methylguanine-DNA	protein_coding	1	2	2	3	1.325
4256	NM_000900,NM_00	**MGP**	matrix Gla protein	protein_coding	0	0	1	1	1.000
4257	NM_001260511,NM	**MGST1**	microsomal glutathione S	protein_coding	0	0	1	1	1.000
4258	NM_001204366,NM	**MGST2**	microsomal glutathione S	protein_coding	0	0	1	1	1.193
4259	NM_004528,XM_00	**MGST3**	microsomal glutathione S	protein_coding	16	18	17	19	1.161
4261	NM_000246,NM_00	**CIITA**	class II major histocompa	protein_coding	22	22	23	23	0.979
4267	NM_001122898,NM	**CD99**	CD99 molecule (Xg bloo	protein_coding	69	70	70	71	1.011
4277	NM_001289160,NM	**MICB**	MHC class I polypeptide-	protein_coding	84	78	85	79	0.928
4280	gene-MICE	**MICE**	MHC class I polypeptide-	pseudogene	1	2	2	3	1.400
4281	NM_000381,NM_00	**MID1**	midline 1	protein_coding	0	0	1	1	1.015
4282	NM_002415	**MIF**	macrophage migration in	protein_coding	1,088	1,254	1,089	1,255	1.153
4283	NM_002416	**CXCL9**	C-X-C motif chemokine li	protein_coding	0	0	1	1	0.966
4284	NM_012064,XM_01	**MIP**	major intrinsic protein of	protein_coding	1	1	2	2	1.187
4285	NM_005932,XM_01	**MIPEP**	mitochondrial intermedia	protein_coding	19	16	20	17	0.839
4286	NM_000248,NM_00	**MITF**	melanocyte inducing tran	protein_coding	0	0	1	1	1.000
4287	NM_001127696,NM	**ATXN3**	ataxin 3	protein_coding	5	5	6	6	1.052
4288	NM_001145966,NM	**MKI67**	marker of proliferation Ki	protein_coding	158	128	159	129	0.809
4289	NM_001145354,NM	**MKLN1**	muskelin 1	protein_coding	17	13	18	14	0.779
4291	NM_001130156,NM	**MLF1**	myeloid leukemia factor	protein_coding	0	0	1	1	1.000
4292	NM_000249,NM_00	**MLH1**	mutL homolog 1	protein_coding	50	53	51	54	1.066
4293	NM_001284230,NM	**MAP3K9**	mitogen-activated protei	protein_coding	1	1	2	2	0.883
4294	NM_002446,XM_00	**MAP3K10**	mitogen-activated protei	protein_coding	12	12	13	13	0.989
4295	NM_001040109,NM	**MLN**	motilin	protein_coding	0	0	1	1	1.000
4296	NM_002419,XR_42	**MAP3K11**	mitogen-activated protei	protein_coding	50	52	51	53	1.036
4297	NM_001197104,NM	**KMT2A**	lysine methyltransferase	protein_coding	27	22	28	23	0.819
4298	NM_005934,XM_01	**MLLT1**	MLLT1 super elongation	protein_coding	57	60	58	61	1.052
4299	NM_001166693,NM	**AFF1**	AF4/FMR2 family membe	protein_coding	5	4	6	5	0.847
4300	NM_001286691,NM	**MLLT3**	MLLT3 super elongation	protein_coding	18	16	19	17	0.924
4301	NM_001040000,NM	**AFDN**	afadin, adherens junctio	protein_coding	0	0	1	1	1.000
4302	NM_005937	**MLLT6**	MLLT6, PHD finger conta	protein_coding	221	203	222	204	0.919
4303	NM_001170931,NM	**FOXO4**	forkhead box O4	protein_coding	6	6	7	7	1.006
4306	NM_000901,NM_00	**NR3C2**	nuclear receptor subfam	protein_coding	0	0	1	1	1.000
4308	NM_001252020,NM	**TRPM1**	transient receptor potent	protein_coding	0	0	1	1	1.044
4311	NM_000902,NM_00	**MME**	membrane metalloendop	protein_coding	0	0	1	1	1.000
4312	NM_001145938,NM	**MMP1**	matrix metallopeptidase	protein_coding	0	0	1	1	1.000
4313	NM_001127891,NM	**MMP2**	matrix metallopeptidase	protein_coding	0	0	1	1	1.000
4314	NM_002422	**MMP3**	matrix metallopeptidase	protein_coding	0	0	1	1	1.000
4316	NM_002423	**MMP7**	matrix metallopeptidase	protein_coding	0	0	1	1	1.000
4317	NM_001304441,NM	**MMP8**	matrix metallopeptidase	protein_coding	0	0	1	1	1.000
4318	NM_004994	**MMP9**	matrix metallopeptidase	protein_coding	1	0	2	1	0.820
4319	NM_002425	**MMP10**	matrix metallopeptidase	protein_coding	0	0	1	1	1.000
4320	NM_005940,NR_13	**MMP11**	matrix metallopeptidase	protein_coding	0	0	1	1	1.000
4321	NM_002426	**MMP12**	matrix metallopeptidase	protein_coding	0	0	1	1	1.000
4322	NM_002427	**MMP13**	matrix metallopeptidase	protein_coding	0	0	1	1	1.000
4323	NM_004995	**MMP14**	matrix metallopeptidase	protein_coding	1	1	2	2	1.010
4324	NM_002428	**MMP15**	matrix metallopeptidase	protein_coding	0	0	1	1	1.000
4325	NM_005941,XM_02	**MMP16**	matrix metallopeptidase	protein_coding	0	0	1	1	1.000
4326	NM_016155,XM_01	**MMP17**	matrix metallopeptidase	protein_coding	0	0	1	1	1.000
4327	NM_001272101,NM	**MMP19**	matrix metallopeptidase	protein_coding	0	0	1	1	1.000
4329	NM_001278593,NM	**ALDH6A1**	aldehyde dehydrogenase	protein_coding	1	1	2	2	1.046
4330	NM_002430	**MN1**	MN1 proto-oncogene, tra	protein_coding	0	0	1	1	1.019
4331	NM_001177963,NM	**MNAT1**	MNAT1 component of CD	protein_coding	27	27	28	28	1.002
4332	NM_002432	**MNDA**	myeloid cell nuclear diffe	protein_coding	0	0	1	1	1.000
4335	NM_020310,XM_01	**MNT**	MAX network transcriptio	protein_coding	5	6	6	7	1.070

4336	NM_001278322,NM	**MOBP**	myelin associated oligod	protein_coding	0	0	1	1	1.001
4337	NM_001075098,NM	**MOCS1**	molybdenum cofactor sy	protein_coding	0	0	1	1	1.000
4338	NM_004531,NM_17	**MOCS2**	molybdenum cofactor sy	protein_coding	34	33	35	34	0.953
4340	NM_001008228,NM	**MOG**	myelin oligodendrocyte g	protein_coding	0	0	1	1	1.000
4342	NM_005372	**MOS**	MOS proto-oncogene, se	protein_coding	0	0	1	1	1.000
4343	NM_001130079,NM	**MOV10**	Mov10 RISC complex RN	protein_coding	51	51	52	52	0.999
4345	NM_001004196,NM	**CD200**	CD200 molecule	protein_coding	0	0	1	1	1.000
4350	NM_001015052,NM	**MPG**	N-methylpurine DNA glyc	protein_coding	58	64	59	65	1.102
4351	NM_001289155,NM	**MPI**	mannose phosphate isor	protein_coding	17	18	18	19	1.047
4352	NM_005373,XM_01	**MPL**	MPL proto-oncogene, thr	protein_coding	0	0	1	1	0.974
4353	NM_000250	**MPO**	myeloperoxidase	protein_coding	0	0	1	1	1.000
4354	NM_001166460,NM	**MPP1**	membrane palmitoylated	protein_coding	9	10	10	11	1.086
4355	NM_001278370,NM	**MPP2**	membrane palmitoylated	protein_coding	1	1	2	2	0.935
4356	NM_001330233,NM	**MPP3**	membrane palmitoylated	protein_coding	0	0	1	1	1.000
4357	NM_001013436,NM	**MPST**	mercaptopyruvate sulfurt	protein_coding	38	40	39	41	1.075
4358	NM_002437,XM_00	**MPV17**	mitochondrial inner mem	protein_coding	39	38	40	39	0.974
4359	NM_000530,NM_00	**MPZ**	myelin protein zero	protein_coding	0	0	1	1	0.979
4360	NM_002438	**MRC1**	mannose receptor C-type	protein_coding	0	0	1	1	1.029
4361	NM_001330347,NM	**MRE11**	MRE11 homolog, double	protein_coding	27	24	28	25	0.895
4362	gene-MRE11P1	**MRE11P1**	MRE11 homolog, double	pseudogene	0	0	1	1	1.000
4363	NM_004996,XM_01	**ABCC1**	ATP binding cassette sul	protein_coding	14	12	15	13	0.899
4430	NM_001130158,NM	**MYO1B**	myosin IB	protein_coding	0	0	1	1	1.000
4435	NM_001144885,NM	**CITED1**	Cbp/p300 interacting trar	protein_coding	0	0	1	1	0.953
4436	NM_000251,NM_00	**MSH2**	mutS homolog 2	protein_coding	93	86	94	87	0.922
4437	NM_002439	**MSH3**	mutS homolog 3	protein_coding	12	12	13	13	0.957
4438	NM_002440	**MSH4**	mutS homolog 4	protein_coding	0	0	1	1	1.127
4439	NM_002441,NM_02	**MSH5**	mutS homolog 5	protein_coding	3	3	4	4	0.915
4440	NM_002442,XM_00	**MSI1**	musashi RNA binding pr	protein_coding	0	0	1	1	1.000
4477	NM_002443,NM_13	**MSMB**	microseminoprotein beta	protein_coding	0	0	1	1	1.000
4478	NM_002444,XM_00	**MSN**	moesin	protein_coding	914	896	915	897	0.981
4479	gene-MSNP1	**MSNP1**	moesin pseudogene 1	pseudogene	0	0	1	1	1.082
4481	NM_001363744,NM	**MSR1**	macrophage scavenger r	protein_coding	0	0	1	1	1.000
4482	NM_001135670,NM	**MSRA**	methionine sulfoxide red	protein_coding	1	1	2	2	1.246
4485	NM_020998,NR_14	**MST1**	macrophage stimulating	protein_coding	1	1	2	2	1.004
4486	NM_001244937,NM	**MST1R**	macrophage stimulating	protein_coding	0	0	1	1	1.021
4487	NM_002448	**MSX1**	msh homeobox 1	protein_coding	0	0	1	1	0.840
4488	NM_001363626,NM	**MSX2**	msh homeobox 2	protein_coding	0	0	1	1	1.000
4489	NM_005946	**MT1A**	metallothionein 1A	protein_coding	0	0	1	1	1.000
4490	NM_005947	**MT1B**	metallothionein 1B	protein_coding	0	0	1	1	1.000
4493	NM_001363555,NM	**MT1E**	metallothionein 1E	protein_coding	103	145	104	146	1.413
4494	NM_001301272,NM	**MT1F**	metallothionein 1F	protein_coding	0	0	1	1	1.000
4495	NM_001301267,NM	**MT1G**	metallothionein 1G	protein_coding	0	0	1	1	1.000
4496	NM_005951	**MT1H**	metallothionein 1H	protein_coding	0	0	1	1	1.000
4498	NR_036677	**MT1JP**	metallothionein 1J, pseu	transcribed_pseu	0	0	1	1	1.000
4499	NM_176870	**MT1M**	metallothionein 1M	protein_coding	0	0	1	1	1.000
4500	NR_001447	**MT1L**	metallothionein 1L, pseu	transcribed_pseu	0	0	1	1	1.000
4501	NM_005952	**MT1X**	metallothionein 1X	protein_coding	170	235	171	236	1.381
4502	NM_005953	**MT2A**	metallothionein 2A	protein_coding	295	399	296	400	1.351
4503	gene-MT2P1	**MT2P1**	metallothionein 2 pseudo	pseudogene	0	1	1	2	1.512
4504	NM_005954	**MT3**	metallothionein 3	protein_coding	0	0	1	1	1.000
4507	NM_002451	**MTAP**	methylthioadenosine pho	protein_coding	29	25	30	26	0.882
4508	gene-ATP6	**ATP6**	ATP synthase F0 subuni	protein_coding	2,267	2,286	2,268	2,287	1.008
4509	gene-ATP8	**ATP8**	ATP synthase F0 subuni	protein_coding	3,373	3,132	3,374	3,133	0.929
4511	rna-TRNC	**TRNC**	tRNA-Cys	tRNA	32	35	33	36	1.083
4512	gene-COX1	**COX1**	cytochrome c oxidase su	protein_coding	2,646	2,961	2,647	2,962	1.119
4513	gene-COX2	**COX2**	cytochrome c oxidase su	protein_coding	2,030	2,270	2,031	2,271	1.118
4514	gene-COX3	**COX3**	cytochrome c oxidase su	protein_coding	3,239	3,487	3,240	3,488	1.077
4515	NM_001018025	**MTCP1**	mature T cell proliferatior	protein_coding	3	4	4	5	1.156
4519	gene-CYTB	**CYTB**	cytochrome b	protein_coding	842	899	843	900	1.067
4520	NM_005955,XM_01	**MTF1**	metal regulatory transcrip	protein_coding	20	18	21	19	0.892
4521	NM_001367553,NM	**NUDT1**	nudix hydrolase 1	protein_coding	50	59	51	60	1.164
4522	NM_001364837,NM	**MTHFD1**	methylenetetrahydrofolat	protein_coding	235	225	236	226	0.955
4523	gene-MTHFD1P1	**MTHFD1P1**	methylenetetrahydrofolat	pseudogene	0	0	1	1	0.985
4524	NM_001330358,NM	**MTHFR**	methylenetetrahydrofolat	protein_coding	11	11	12	12	1.011
4528	NM_001005369,NM	**MTIF2**	mitochondrial translation	protein_coding	51	50	52	51	0.984
4534	NM_000252,NM_00	**MTM1**	myotubularin 1	protein_coding	13	13	14	14	1.011
4535	gene-ND1	**ND1**	NADH dehydrogenase su	protein_coding	953	1,049	954	1,050	1.101
4536	gene-ND2	**ND2**	NADH dehydrogenase su	protein_coding	1,311	1,306	1,312	1,307	0.997
4537	gene-ND3	**ND3**	NADH dehydrogenase su	protein_coding	1,025	1,089	1,026	1,090	1.062
4538	gene-ND4	**ND4**	NADH dehydrogenase su	protein_coding	1,979	2,179	1,980	2,180	1.101
4539	gene-ND4L	**ND4L**	NADH dehydrogenase su	protein_coding	2,812	2,829	2,813	2,830	1.006
4540	gene-ND5	**ND5**	NADH dehydrogenase su	protein_coding	468	468	469	469	1.001
4541	gene-ND6	**ND6**	NADH dehydrogenase su	protein_coding	628	555	629	556	0.885
4542	NM_001348355,NM	**MYO1F**	myosin IF	protein_coding	132	139	133	140	1.058
4543	NM_005958,XM_01	**MTNR1A**	melatonin receptor 1A	protein_coding	0	0	1	1	1.000
4544	NM_005959,XM_01	**MTNR1B**	melatonin receptor 1B	protein_coding	0	0	1	1	1.000
4547	NM_000253,NM_00	**MTTP**	microsomal triglyceride t	protein_coding	1	1	2	2	1.036
4548	NM_000254,NM_00	**MTR**	5-methyltetrahydrofolate	protein_coding	19	17	20	18	0.930
4549	rna-RNR1	**RNR1**	s-rRNA	rRNA	212	212	213	213	1.000
4550	rna-RNR2	**RNR2**	l-rRNA	rRNA	463	485	464	486	1.049
4552	NM_001364440,NM	**MTRR**	5-methyltetrahydrofolate	protein_coding	40	37	41	38	0.912
4553	rna-TRNA	**TRNA**	tRNA-Ala	tRNA	22	22	23	23	1.007
4555	rna-TRND	**TRND**	tRNA-Asp	tRNA	25	26	26	27	1.014
4556	rna-TRNE	**TRNE**	tRNA-Glu	tRNA	145	141	146	142	0.973
4558	rna-TRNF	**TRNF**	tRNA-Phe	tRNA	70	77	71	78	1.100
4563	rna-TRNG	**TRNG**	tRNA-Gly	tRNA	1	3	2	4	1.617
4564	rna-TRNH	**TRNH**	tRNA-His	tRNA	6	2	7	3	0.437
4565	rna-TRNI	**TRNI**	tRNA-Ile	tRNA	39	51	40	52	1.313
4566	rna-TRNK	**TRNK**	tRNA-Lys	tRNA	14	7	15	8	0.534
4567	rna-TRNL1	**TRNL1**	tRNA-Leu	tRNA	218	202	219	203	0.925
4568	rna-TRNL2	**TRNL2**	tRNA-Leu	tRNA	1	2	2	3	1.246
4569	rna-TRNM	**TRNM**	tRNA-Met	tRNA	46	48	47	49	1.040
4570	rna-TRNN	**TRNN**	tRNA-Asn	tRNA	11	13	12	14	1.248
4571	rna-TRNP	**TRNP**	tRNA-Pro	tRNA	26	22	27	23	0.879
4572	rna-TRNQ	**TRNQ**	tRNA-Gln	tRNA	44	58	45	59	1.314
4573	rna-TRNR	**TRNR**	tRNA-Arg	tRNA	2	2	3	3	0.896
4574	rna-TRNS1	**TRNS1**	tRNA-Ser	tRNA	302	303	303	304	1.002
4575	rna-TRNS2	**TRNS2**	tRNA-Ser	tRNA	4	2	5	3	0.629
4576	rna-TRNT	**TRNT**	tRNA-Thr	tRNA	29	21	30	22	0.733
4577	rna-TRNV	**TRNV**	tRNA-Val	tRNA	33	33	34	34	0.998
4578	rna-TRNW	**TRNW**	tRNA-Trp	tRNA	19	20	20	21	1.037
4579	rna-TRNY	**TRNY**	tRNA-Tyr	tRNA	358	340	359	341	0.949
4580	NM_002455,NM_19	**MTX1**	metaxin 1	protein_coding	60	58	61	59	0.980
4582	NM_001018016,NM	**MUC1**	mucin 1, cell surface ass	protein_coding	0	0	1	1	1.000
4583	NM_002457	**MUC2**	mucin 2, oligomeric muc	protein_coding	0	0	1	1	1.000
4584	NM_005960	**MUC3A**	mucin 3A, cell surface as	protein_coding	0	0	1	1	1.000
4585	NM_004532,NM_01	**MUC4**	mucin 4, cell surface ass	protein_coding	0	0	1	1	1.000
4586	NM_001304359	**MUC5AC**	mucin 5AC, oligomeric m	protein_coding	0	0	1	1	1.000
4588	NM_005961	**MUC6**	mucin 6, oligomeric muc	protein_coding	0	0	1	1	1.000
4589	NM_001145006,NM	**MUC7**	mucin 7, secreted	protein_coding	0	0	1	1	1.000
4591	NM_001005207,NM	**TRIM37**	tripartite motif containing	protein_coding	33	30	34	31	0.910
4593	NM_001166280,NM	**MUSK**	muscle associated recep	protein_coding	0	0	1	1	1.000
4594	NM_000255,XM_00	**MMUT**	methylmalonyl-CoA muta	protein_coding	20	20	21	21	0.992
4595	NM_001048171,NM	**MUTYH**	mutY DNA glycosylase	protein_coding	38	47	39	48	1.232
4597	NM_002461,XM_01	**MVD**	mevalonate diphosphate	protein_coding	52	53	53	54	1.018
4598	NM_000431,NM_00	**MVK**	mevalonate kinase	protein_coding	18	16	19	17	0.883
4599	NM_001144925,NM	**MX1**	MX dynamin like GTPase	protein_coding	236	198	237	199	0.837
4600	NM_002463,XM_00	**MX2**	MX dynamin like GTPase	protein_coding	2	2	3	3	0.868
4601	NM_001008541,NM	**MXI1**	MAX interactor 1, dimeriz	protein_coding	8	9	9	10	1.151
4602	NM_001130172,NM	**MYB**	MYB proto-oncogene, tra	protein_coding	6	7	7	8	1.091
4603	NM_001080416,NM	**MYBL1**	MYB proto-oncogene like	protein_coding	48	45	49	46	0.941
4604	NM_001254718,NM	**MYBPC1**	myosin binding protein C	protein_coding	0	0	1	1	1.000
4605	NM_001278610,NM	**MYBL2**	MYB proto-oncogene like	protein_coding	184	189	185	190	1.027
4606	NM_004533	**MYBPC2**	myosin binding protein C	protein_coding	0	0	1	1	1.000
4607	NM_000256	**MYBPC3**	myosin binding protein C	protein_coding	0	0	1	1	1.000
4608	NM_004997	**MYBPH**	myosin binding protein H	protein_coding	0	0	1	1	1.000

4609	NM_001354870,NM	**MYC**	MYC proto-oncogene, bH	protein_coding	178	194	179	195	1.090
4610	NM_001033081,NM	**MYCL**	MYCL proto-oncogene, b	protein_coding	0	0	1	1	1.000
4611	gene-MYCLP1	**MYCLP1**	MYCL pseudogene 1	pseudogene	0	0	1	1	1.000
4613	NM_001293228,NM	**MYCN**	MYCN proto-oncogene, b	protein_coding	0	0	1	1	1.000
4615	NM_001172566,NM	**MYD88**	MYD88 innate immune s	protein_coding	98	96	99	97	0.987
4616	NM_015675,XM_01	**GADD45B**	growth arrest and DNA d	protein_coding	128	147	129	148	1.152
4617	NM_005593	**MYF5**	myogenic factor 5	protein_coding	0	0	1	1	1.000
4618	NM_002469	**MYF6**	myogenic factor 6	protein_coding	0	0	1	1	1.000
4619	NM_005963,XM_01	**MYH1**	myosin heavy chain 1	protein_coding	0	0	1	1	1.000
4620	NM_001100112,NM	**MYH2**	myosin heavy chain 2	protein_coding	0	0	1	1	1.000
4621	NM_002470,XM_01	**MYH3**	myosin heavy chain 3	protein_coding	1	1	2	2	1.158
4622	NM_017533,XM_01	**MYH4**	myosin heavy chain 4	protein_coding	0	0	1	1	1.000
4624	NM_002471	**MYH6**	myosin heavy chain 6	protein_coding	0	0	1	1	1.000
4625	NM_000257,XM_01	**MYH7**	myosin heavy chain 7	protein_coding	0	0	1	1	1.000
4626	NM_002472	**MYH8**	myosin heavy chain 8	protein_coding	0	0	1	1	1.000
4627	NM_002473	**MYH9**	myosin heavy chain 9	protein_coding	338	335	339	336	0.990
4628	NM_001256012,NM	**MYH10**	myosin heavy chain 10	protein_coding	0	0	1	1	1.000
4629	NM_001040113,NM	**MYH11**	myosin heavy chain 11	protein_coding	0	0	1	1	0.930
4632	NM_079420,NM_07	**MYL1**	myosin light chain 1	protein_coding	0	0	1	1	1.000
4633	NM_000432	**MYL2**	myosin light chain 2	protein_coding	0	0	1	1	1.000
4634	NM_000258	**MYL3**	myosin light chain 3	protein_coding	0	0	1	1	1.000
4635	NM_001002841,NM	**MYL4**	myosin light chain 4	protein_coding	0	1	1	2	1.697
4636	NM_001363650,NM	**MYL5**	myosin light chain 5	protein_coding	4	5	5	6	1.194
4637	NM_021019,NM_07	**MYL6**	myosin light chain 6	protein_coding	798	871	799	872	1.091
4638	NM_001321309,NM	**MYLK**	myosin light chain kinase	protein_coding	0	0	1	1	1.027
4640	NM_001256041,NM	**MYO1A**	myosin IA	protein_coding	0	0	1	1	1.073
4641	NM_001080779,NM	**MYO1C**	myosin IC	protein_coding	2	2	3	3	1.037
4642	NM_001303279,NM	**MYO1D**	myosin ID	protein_coding	7	5	8	6	0.839
4643	NM_004998	**MYO1E**	myosin IE	protein_coding	0	0	1	1	0.989
4644	NM_000259,NM_00	**MYO5A**	myosin VA	protein_coding	13	11	14	12	0.844
4645	NM_001080467	**MYO5B**	myosin VB	protein_coding	6	2	7	3	0.491
4646	NM_001300899,NM	**MYO6**	myosin VI	protein_coding	13	11	14	12	0.816
4647	NM_000260,NM_00	**MYO7A**	myosin VIIA	protein_coding	0	0	1	1	1.000
4648	NM_001080527,XM	**MYO7B**	myosin VIIB	protein_coding	0	0	1	1	1.000
4649	NM_006901,XM_00	**MYO9A**	myosin IXA	protein_coding	5	4	6	5	0.791
4650	NM_001130065,NM	**MYO9B**	myosin IXB	protein_coding	64	58	65	59	0.900
4651	NM_012334,XM_00	**MYO10**	myosin X	protein_coding	0	0	1	1	1.000
4653	NM_000261	**MYOC**	myocilin	protein_coding	0	0	1	1	1.213
4654	NM_002478	**MYOD1**	myogenic differentiation	protein_coding	0	0	1	1	1.000
4656	NM_002479	**MYOG**	myogenin	protein_coding	0	0	1	1	1.000
4659	NM_001143885,NM	**PPP1R12A**	protein phosphatase 1 re	protein_coding	54	49	55	50	0.902
4660	NM_001167857,NM	**PPP1R12B**	protein phosphatase 1 re	protein_coding	4	4	5	5	0.946
4661	NM_004535	**MYT1**	myelin transcription facto	protein_coding	1	1	2	2	1.145
4664	NM_001321312,NM	**NAB1**	NGFI-A binding protein 1	protein_coding	25	21	26	22	0.822
4665	NM_001330305,NM	**NAB2**	NGFI-A binding protein 2	protein_coding	8	9	9	10	1.075
4666	NM_001113201,NM	**NACA**	nascent polypeptide ass	protein_coding	1,400	1,366	1,401	1,367	0.976
4668	NM_000262,NM_00	**NAGA**	alpha-N-acetylgalactosar	protein_coding	14	15	15	16	1.013
4669	NM_000263,XM_00	**NAGLU**	N-acetyl-alpha-glucosam	protein_coding	14	14	15	15	0.993
4670	NM_001297418,NM	**HNRNPM**	heterogeneous nuclear r	protein_coding	535	544	536	545	1.017
4671	NM_001346870,NM	**NAIP**	NLR family apoptosis inh	protein_coding	1	1	2	2	1.032
4673	NM_001307924,NM	**NAP1L1**	nucleosome assembly pr	protein_coding	232	210	233	211	0.907
4674	NM_021963	**NAP1L2**	nucleosome assembly pr	protein_coding	0	0	1	1	1.000
4675	NM_004538	**NAP1L3**	nucleosome assembly pr	protein_coding	0	0	1	1	1.000
4676	NM_001369375,NM	**NAP1L4**	nucleosome assembly pr	protein_coding	219	226	220	227	1.032
4677	NM_004539,XM_00	**NARS1**	asparaginyl-tRNA synthe	protein_coding	236	215	237	216	0.911
4678	NM_001195193,NM	**NASP**	nuclear autoantigenic sp	protein_coding	352	322	353	323	0.914
4680	NM_002483,XM_01	**CEACAM6**	CEA cell adhesion molec	protein_coding	0	0	1	1	1.000
4681	NM_001204084,NM	**NBL1**	NBL1, DAN family BMP a	protein_coding	0	0	1	1	1.000
4682	NM_001278506,NM	**NUBP1**	nucleotide binding protei	protein_coding	73	74	74	75	1.004
4683	NM_001024688,NM	**NBN**	nibrin	protein_coding	69	62	70	63	0.897
4684	NM_000615,NM_00	**NCAM1**	neural cell adhesion mol	protein_coding	89	82	90	83	0.914
4685	NM_001352591,NM	**NCAM2**	neural cell adhesion mol	protein_coding	1	1	2	2	1.090
4686	NM_001351504,NM	**NCBP1**	nuclear cap binding prot	protein_coding	60	54	61	55	0.908
4688	NM_000433,NM_00	**NCF2**	neutrophil cytosolic facto	protein_coding	3	2	4	3	0.735
4689	NM_000631,NM_01	**NCF4**	neutrophil cytosolic facto	protein_coding	13	13	14	14	1.059
4690	NM_001190796,NM	**NCK1**	NCK adaptor protein 1	protein_coding	22	21	23	22	0.942
4691	NM_005381	**NCL**	nucleolin	protein_coding	2,207	2,161	2,208	2,162	0.979
4692	NM_002487	**NDN**	necdin, MAGE family me	protein_coding	0	0	1	1	1.000
4693	NM_000266	**NDP**	norrin cystine knot growt	protein_coding	0	0	1	1	1.117
4694	NM_004541	**NDUFA1**	NADH:ubiquinone oxidor	protein_coding	409	432	410	433	1.058
4695	NM_001185012,NM	**NDUFA2**	NADH:ubiquinone oxidor	protein_coding	209	211	210	212	1.012
4696	NM_004542,XM_01	**NDUFA3**	NADH:ubiquinone oxidor	protein_coding	120	143	121	144	1.196
4697	NM_002489	**NDUFA4**	NDUFA4 mitochondrial c	protein_coding	117	109	118	110	0.929
4698	NM_001282419,NM	**NDUFA5**	NADH:ubiquinone oxidor	protein_coding	49	43	50	44	0.894
4699	gene-NDUFA5P1	**NDUFA5P1**	NADH:ubiquinone oxidor	pseudogene	0	0	1	1	1.000
4700	NM_002490	**NDUFA6**	NADH:ubiquinone oxidor	protein_coding	104	102	105	103	0.987
4701	NM_005001,NR_13	**NDUFA7**	NADH:ubiquinone oxidor	protein_coding	153	186	154	187	1.217
4702	NM_001318195,NM	**NDUFA8**	NADH:ubiquinone oxidor	protein_coding	107	118	108	119	1.102
4703	NM_001164507,NM	**NEB**	nebulin	protein_coding	0	0	1	1	0.989
4704	NM_005002	**NDUFA9**	NADH:ubiquinone oxidor	protein_coding	20	20	21	21	1.020
4705	NM_001322019,NM	**NDUFA10**	NADH:ubiquinone oxidor	protein_coding	64	67	65	68	1.042
4706	NM_005003,XM_01	**NDUFAB1**	NADH:ubiquinone oxidor	protein_coding	420	434	421	435	1.032
4707	NM_004545	**NDUFB1**	NADH:ubiquinone oxidor	protein_coding	262	245	263	246	0.934
4708	NM_004546	**NDUFB2**	NADH:ubiquinone oxidor	protein_coding	214	217	215	218	1.012
4709	NM_001257102,NM	**NDUFB3**	NADH:ubiquinone oxidor	protein_coding	542	588	543	589	1.086
4710	NM_001168331,NM	**NDUFB4**	NADH:ubiquinone oxidor	protein_coding	293	294	294	295	1.004
4711	NM_001199957,NM	**NDUFB5**	NADH:ubiquinone oxidor	protein_coding	53	54	54	55	1.022
4712	NM_001199987,NM	**NDUFB6**	NADH:ubiquinone oxidor	protein_coding	80	78	81	79	0.977
4713	NM_004146,XM_01	**NDUFB7**	NADH:ubiquinone oxidor	protein_coding	260	286	261	287	1.098
4714	NM_001284367,NM	**NDUFB8**	NADH:ubiquinone oxidor	protein_coding	461	480	462	481	1.041
4715	NM_001278645,NM	**NDUFB9**	NADH:ubiquinone oxidor	protein_coding	304	314	305	315	1.032
4716	NM_004548	**NDUFB10**	NADH:ubiquinone oxidor	protein_coding	176	194	177	195	1.105
4717	NM_001184986,NM	**NDUFC1**	NADH:ubiquinone oxidor	protein_coding	60	57	61	58	0.967
4718	NM_001204054,NM	**NDUFC2**	NADH:ubiquinone oxidor	protein_coding	90	98	91	99	1.084
4719	NM_001199981,NM	**NDUFS1**	NADH:ubiquinone oxidor	protein_coding	46	43	47	44	0.924
4720	NM_001166159,NM	**NDUFS2**	NADH:ubiquinone oxidor	protein_coding	155	154	156	155	0.997
4722	NM_004551	**NDUFS3**	NADH:ubiquinone oxidor	protein_coding	221	236	222	237	1.067
4723	NM_001166102,NM	**NDUFV1**	NADH:ubiquinone oxidor	protein_coding	267	275	268	276	1.030
4724	NM_001318051,NM	**NDUFS4**	NADH:ubiquinone oxidor	protein_coding	91	89	92	90	0.983
4725	NM_001184979,NM	**NDUFS5**	NADH:ubiquinone oxidor	protein_coding	503	550	504	551	1.094
4726	NM_004553	**NDUFS6**	NADH:ubiquinone oxidor	protein_coding	463	487	464	488	1.051
4728	NM_002496	**NDUFS8**	NADH:ubiquinone oxidor	protein_coding	233	255	234	256	1.093
4729	NM_021074,XM_01	**NDUFV2**	NADH:ubiquinone oxidor	protein_coding	193	188	194	189	0.973
4730	gene-NDUFV2P1	**NDUFV2P1**	NADH:ubiquinone oxidor	pseudogene	4	4	5	5	0.887
4731	NM_001001503,NM	**NDUFV3**	NADH:ubiquinone oxidor	protein_coding	12	14	13	15	1.122
4733	NM_004147	**DRG1**	developmentally regulate	protein_coding	129	125	130	126	0.974
4734	NM_001284338,NM	**NEDD4**	NEDD4 E3 ubiquitin prot	protein_coding	0	0	1	1	1.000
4735	NM_001008491,NM	**SEPTIN2**	septin 2	protein_coding	447	413	448	414	0.926
4736	NM_007104,XM_01	**RPL10A**	ribosomal protein L10a	protein_coding	1,678	1,784	1,679	1,785	1.063
4738	NM_006156	**NEDD8**	NEDD8 ubiquitin like mo	protein_coding	317	365	318	366	1.149
4739	NM_001142393,NM	**NEDD9**	neural precursor cell exp	protein_coding	96	97	97	98	1.013
4741	NM_001105541,NM	**NEFM**	neurofilament medium	protein_coding	0	0	1	1	1.000
4744	NM_021076,XM_01	**NEFH**	neurofilament heavy	protein_coding	0	0	1	1	1.000
4745	NM_001288713,NM	**NELL1**	neural EGFL like 1	protein_coding	0	0	1	1	1.000
4747	NM_006158	**NEFL**	neurofilament light	protein_coding	0	0	1	1	1.000
4750	NM_001199397,NM	**NEK1**	NIMA related kinase 1	protein_coding	13	11	14	12	0.836
4751	NM_001204182,NM	**NEK2**	NIMA related kinase 2	protein_coding	45	39	46	40	0.874
4752	NM_002498,NM_15	**NEK3**	NIMA related kinase 3	protein_coding	0	0	1	1	1.091
4753	NM_001145107,NM	**NELL2**	neural EGFL like 2	protein_coding	0	0	1	1	1.000
4756	NM_001172623,NM	**NEO1**	neogenin 1	protein_coding	0	0	1	1	1.000
4758	NM_000434	**NEU1**	neuraminidase 1	protein_coding	33	36	34	37	1.086
4759	NM_005383	**NEU2**	neuraminidase 2	protein_coding	0	0	1	1	1.000
4760	NM_002500,NR_14	**NEUROD1**	neuronal differentiation 1	protein_coding	0	0	1	1	1.000
4761	NM_006160	**NEUROD2**	neuronal differentiation 2	protein_coding	0	0	1	1	0.970
4762	NM_006161	**NEUROG1**	neurogenin 1	protein_coding	0	0	1	1	1.000
4763	NM_000267,NM_00	**NF1**	neurofibromin 1	protein_coding	18	14	19	15	0.785

4764	gene-NF1P3	**NF1P3**	neurofibromin 1 pseudog	pseudogene	0	0	1	1	1.000
4768	gene-NF1P5	**NF1P5**	neurofibromin 1 pseudog	pseudogene	0	0	1	1	1.000
4771	NM_000268,NM_01	**NF2**	neurofibromin 2	protein_coding	30	28	31	29	0.940
4772	NM_001278669,NM	**NFATC1**	nuclear factor of activate	protein_coding	0	0	1	1	0.981
4773	NM_001136021,NM	**NFATC2**	nuclear factor of activate	protein_coding	51	43	52	44	0.852
4774	NM_001134673,NM	**NFIA**	nuclear factor I A	protein_coding	0	1	1	2	1.155
4775	NM_004555,NM_17	**NFATC3**	nuclear factor of activate	protein_coding	39	37	40	38	0.954
4776	NM_001136022,NM	**NFATC4**	nuclear factor of activate	protein_coding	0	0	1	1	1.000
4778	NM_001136023,NM	**NFE2**	nuclear factor, erythroid	protein_coding	1	1	2	2	1.183
4779	NM_001330261,NM	**NFE2L1**	nuclear factor, erythroid	protein_coding	120	117	121	118	0.981
4780	NM_001145412,NM	**NFE2L2**	nuclear factor, erythroid	protein_coding	71	69	72	70	0.974
4781	NM_001190737,NM	**NFIB**	nuclear factor I B	protein_coding	1	1	2	2	1.119
4782	NM_001245002,NM	**NFIC**	nuclear factor I C	protein_coding	1	1	2	2	1.153
4783	NM_001289999,NM	**NFIL3**	nuclear factor, interleukin	protein_coding	49	47	50	48	0.953
4784	NM_001271043,NM	**NFIX**	nuclear factor I X	protein_coding	0	0	1	1	1.000
4790	NM_001165412,NM	**NFKB1**	nuclear factor kappa B s	protein_coding	60	58	61	59	0.958
4791	NM_001077494,NM	**NFKB2**	nuclear factor kappa B s	protein_coding	14	15	15	16	1.062
4792	NM_020529	**NFKBIA**	NFKB inhibitor alpha	protein_coding	72	70	73	71	0.967
4793	NM_001243116,NM	**NFKBIB**	NFKB inhibitor beta	protein_coding	126	125	127	126	0.995
4794	NM_004556	**NFKBIE**	NFKB inhibitor epsilon	protein_coding	10	12	11	13	1.170
4795	NM_001144961,NM	**NFKBIL1**	NFKB inhibitor like 1	protein_coding	36	38	37	39	1.074
4796	NM_013432,XM_01	**TONSL**	tonsoku like, DNA repair	protein_coding	21	20	22	21	0.960
4798	NM_001143835,NM	**NFRKB**	nuclear factor related to	protein_coding	22	20	23	21	0.934
4799	NM_001318758,NM	**NFX1**	nuclear transcription fact	protein_coding	41	40	42	41	0.976
4800	NM_002505,NM_02	**NFYA**	nuclear transcription fact	protein_coding	30	28	31	29	0.935
4801	NM_006166,XM_00	**NFYB**	nuclear transcription fact	protein_coding	32	32	33	33	1.012
4802	NM_001142587,NM	**NFYC**	nuclear transcription fact	protein_coding	61	62	62	63	1.025
4803	NM_002506,XM_00	**NGF**	nerve growth factor	protein_coding	0	0	1	1	1.000
4804	NM_002507	**NGFR**	nerve growth factor recep	protein_coding	5	5	6	6	1.075
4807	NM_005598	**NHLH1**	nescient helix-loop-helix	protein_coding	0	0	1	1	1.032
4808	NM_001111061,NM	**NHLH2**	nescient helix-loop-helix	protein_coding	0	0	1	1	1.000
4809	NM_001003796,NM	**SNU13**	small nuclear ribonucleo	protein_coding	236	254	237	255	1.074
4810	NM_001136024,NM	**NHS**	NHS actin remodeling re	protein_coding	0	0	1	1	1.000
4811	NM_002508,XM_01	**NID1**	nidogen 1	protein_coding	0	0	1	1	1.000
4814	NM_004148,XM_01	**NINJ1**	ninjurin 1	protein_coding	0	0	1	1	1.000
4815	NM_001294345,NM	**NINJ2**	ninjurin 2	protein_coding	2	2	3	3	0.961
4817	NM_001185092,NM	**NIT1**	nitrilase 1	protein_coding	23	27	24	28	1.181
4818	NM_001363693,NM	**NKG7**	natural killer cell granule	protein_coding	3,215	3,445	3,216	3,446	1.071
4820	NM_001349124,NM	**NKTR**	natural killer cell triggerin	protein_coding	30	26	31	27	0.870
4821	NM_002509,XM_00	**NKX2-2**	NK2 homeobox 2	protein_coding	0	0	1	1	1.000
4824	NM_001256339,NM	**NKX3-1**	NK3 homeobox 1	protein_coding	1	1	2	2	0.942
4825	NM_006168	**NKX6-1**	NK6 homeobox 1	protein_coding	0	0	1	1	1.000
4826	NM_001322802,NM	**NNAT**	neuronatin	protein_coding	0	0	1	1	1.000
4828	NM_021077,NM_20	**NMB**	neuromedin B	protein_coding	0	1	1	2	1.429
4829	NM_001324307,NM	**NMBR**	neuromedin B receptor	protein_coding	0	0	1	1	0.963
4830	NM_000269,NM_19	**NME1**	NME/NM23 nucleoside d	protein_coding	737	744	738	745	1.010
4831	NM_001018137,NM	**NME2**	NME/NM23 nucleoside d	protein_coding	1,412	1,510	1,413	1,511	1.069
4832	NM_002513,XM_00	**NME3**	NME/NM23 nucleoside d	protein_coding	1	1	2	2	0.941
4833	NM_001286433,NM	**NME4**	NME/NM23 nucleoside d	protein_coding	0	0	1	1	0.953
4835	NM_000904,NM_00	**NQO2**	N-ribosyldihydronicotinar	protein_coding	24	21	25	22	0.870
4836	NM_021079,XR_00	**NMT1**	N-myristoyltransferase 1	protein_coding	81	79	82	80	0.973
4837	NM_001372045,NM	**NNMT**	nicotinamide N-methyltra	protein_coding	0	0	1	1	1.000
4838	NM_001329906,NM	**NODAL**	nodal growth differentiati	protein_coding	0	0	1	1	1.000
4839	NM_001033714,NM	**NOP2**	NOP2 nucleolar protein	protein_coding	81	80	82	81	0.986
4841	NM_001145408,NM	**NONO**	non-POU domain contai	protein_coding	598	604	599	605	1.010
4842	NM_000620,NM_00	**NOS1**	nitric oxide synthase 1	protein_coding	0	0	1	1	1.000
4843	NM_000625	**NOS2**	nitric oxide synthase 2	protein_coding	0	0	1	1	1.000
4846	NM_000603,NM_00	**NOS3**	nitric oxide synthase 3	protein_coding	0	0	1	1	1.043
4848	NM_001199302,NM	**CNOT2**	CCR4-NOT transcription	protein_coding	34	31	35	32	0.910
4849	NM_014516,XM_00	**CNOT3**	CCR4-NOT transcription	protein_coding	54	55	55	56	1.031
4850	NM_001008225,NM	**CNOT4**	CCR4-NOT transcription	protein_coding	18	17	19	18	0.956
4851	NM_017617,XM_01	**NOTCH1**	notch receptor 1	protein_coding	8	8	9	9	1.022
4852	NM_000905	**NPY**	neuropeptide Y	protein_coding	0	0	1	1	1.000
4853	NM_001200001,NM	**NOTCH2**	notch receptor 2	protein_coding	51	43	52	44	0.850
4854	NM_000435,XM_00	**NOTCH3**	notch receptor 3	protein_coding	0	0	1	1	1.000
4855	NM_004557,NR_13	**NOTCH4**	notch receptor 4	protein_coding	0	0	1	1	0.934
4856	NM_002514	**CCN3**	cellular communication n	protein_coding	0	0	1	1	1.000
4857	NM_001366390,NM	**NOVA1**	NOVA alternative splicing	protein_coding	0	0	1	1	1.000
4858	NM_002516,XM_00	**NOVA2**	NOVA alternative splicing	protein_coding	0	0	1	1	1.000
4860	NM_000270	**PNP**	purine nucleoside phosp	protein_coding	201	197	202	198	0.979
4861	NM_001321086,NM	**NPAS1**	neuronal PAS domain pr	protein_coding	1	1	2	2	1.116
4862	NM_002518,XM_00	**NPAS2**	neuronal PAS domain pr	protein_coding	0	0	1	1	1.000
4863	NM_001321307,NM	**NPAT**	nuclear protein, coactiva	protein_coding	18	16	19	17	0.886
4864	NM_000271,XM_00	**NPC1**	NPC intracellular cholest	protein_coding	24	22	25	23	0.923
4867	NM_000272,NM_00	**NPHP1**	nephrocystin 1	protein_coding	1	1	2	2	1.146
4868	NM_004646	**NPHS1**	NPHS1 adhesion molecu	protein_coding	0	0	1	1	1.000
4869	NM_001037738,NM	**NPM1**	nucleophosmin 1	protein_coding	4,527	4,327	4,528	4,328	0.956
4878	NM_006172	**NPPA**	natriuretic peptide A	protein_coding	0	0	1	1	1.000
4879	NM_002521	**NPPB**	natriuretic peptide B	protein_coding	0	0	1	1	1.000
4880	NM_024409,XM_01	**NPPC**	natriuretic peptide C	protein_coding	0	0	1	1	1.000
4881	NM_000906,XM_00	**NPR1**	natriuretic peptide recept	protein_coding	0	0	1	1	1.000
4882	NM_001378923,NM	**NPR2**	natriuretic peptide recept	protein_coding	1	1	2	2	1.002
4883	NM_000908,NM_00	**NPR3**	natriuretic peptide recept	protein_coding	0	0	1	1	1.002
4884	NM_002522	**NPTX1**	neuronal pentraxin 1	protein_coding	0	0	1	1	1.068
4885	NM_002523	**NPTX2**	neuronal pentraxin 2	protein_coding	0	0	1	1	1.000
4886	NM_000909,XM_00	**NPY1R**	neuropeptide Y receptor	protein_coding	0	0	1	1	1.000
4887	NM_000910,NM_00	**NPY2R**	neuropeptide Y receptor	protein_coding	0	0	1	1	1.000
4888	NR_002713	**NPY6R**	neuropeptide Y receptor	transcribed_pseu	0	0	1	1	1.000
4889	NM_001317091,NM	**NPY5R**	neuropeptide Y receptor	protein_coding	0	0	1	1	1.000
4891	NM_000617,NM_00	**SLC11A2**	solute carrier family 11 m	protein_coding	16	15	17	16	0.979
4892	NM_001261463,NM	**NRAP**	nebulin related anchoring	protein_coding	0	0	1	1	1.018
4893	NM_002524	**NRAS**	NRAS proto-oncogene, G	protein_coding	127	116	128	117	0.913
4897	NM_001037132,NM	**NRCAM**	neuronal cell adhesion m	protein_coding	0	0	1	1	1.000
4898	NM_001101662,NM	**NRDC**	nardilysin convertase	protein_coding	121	112	122	113	0.924
4899	NM_001040110,NM	**NRF1**	nuclear respiratory factor	protein_coding	21	19	22	20	0.907
4900	NM_001126181,NM	**NRGN**	neurogranin	protein_coding	0	0	1	1	1.000
4901	NM_001354768,NM	**NRL**	neural retina leucine zipp	protein_coding	1	1	2	2	0.853
4902	NM_004558,XM_01	**NRTN**	neurturin	protein_coding	0	0	1	1	1.000
4904	NM_004559,NR_13	**YBX1**	Y-box binding protein 1	protein_coding	878	936	879	937	1.066
4905	NM_006178,NR_04	**NSF**	N-ethylmaleimide sensiti	protein_coding	41	37	42	38	0.911
4907	NM_001204813,NM	**NT5E**	5'-nucleotidase ecto	protein_coding	0	0	1	1	1.000
4908	NM_001102654,NM	**NTF3**	neurotrophin 3	protein_coding	0	0	1	1	1.000
4909	NM_006179,XM_00	**NTF4**	neurotrophin 4	protein_coding	0	0	1	1	1.000
4910	gene-NTF6A	**NTF6A**	neurotrophin 6 alpha (ps	pseudogene	0	0	1	1	1.000
4911	gene-NTF6B	**NTF6B**	neurotrophin 6 beta (pse	pseudogene	0	0	1	1	1.000
4912	gene-NTF6G	**NTF6G**	neurotrophin 6 gamma (p	pseudogene	0	0	1	1	1.000
4913	NM_001318193,NM	**NTHL1**	nth like DNA glycosylase	protein_coding	65	67	66	68	1.029
4914	NM_001007792,NM	**NTRK1**	neurotrophic receptor tyr	protein_coding	0	0	1	1	0.993
4915	NM_001007097,NM	**NTRK2**	neurotrophic receptor tyr	protein_coding	0	0	1	1	1.050
4916	NM_001007156,NM	**NTRK3**	neurotrophic receptor tyr	protein_coding	0	0	1	1	1.000
4917	NM_006181	**NTN3**	netrin 3	protein_coding	0	0	1	1	0.851
4919	NM_001083592,NM	**ROR1**	receptor tyrosine kinase	protein_coding	0	0	1	1	1.000
4920	NM_001318204,NM	**ROR2**	receptor tyrosine kinase	protein_coding	0	0	1	1	1.162
4921	NM_001014796,NM	**DDR2**	discoidin domain recepto	protein_coding	0	0	1	1	1.038
4922	NM_006183	**NTS**	neurotensin	protein_coding	0	0	1	1	0.866
4923	NM_002531,XM_01	**NTSR1**	neurotensin receptor 1	protein_coding	0	0	1	1	1.000
4924	NM_006184,XM_01	**NUCB1**	nucleobindin 1	protein_coding	73	84	74	85	1.154
4925	NM_001330227,NM	**NUCB2**	nucleobindin 2	protein_coding	183	175	184	176	0.956
4926	NM_001286561,NM	**NUMA1**	nuclear mitotic apparatus	protein_coding	62	62	63	63	1.004
4927	NM_001320653,NM	**NUP88**	nucleoporin 88	protein_coding	34	33	35	34	0.982
4928	NM_001365125,NM	**NUP98**	nucleoporin 98 and 96 p	protein_coding	115	101	116	102	0.878
4929	NM_006186,NM_17	**NR4A2**	nuclear receptor subfam	protein_coding	28	25	29	26	0.879
4931	NM_001243146,NM	**NVL**	nuclear VCP like	protein_coding	28	26	29	27	0.937
4935	NM_000273,XM_00	**GPR143**	G protein-coupled recept	protein_coding	0	0	1	1	1.000
4938	NM_001032409,NM	**OAS1**	2'-5'-oligoadenylate synt	protein_coding	60	50	61	51	0.837
4939	NM_001032731,NM	**OAS2**	2'-5'-oligoadenylate synt	protein_coding	105	85	106	86	0.817

4940	NM_006187,XM_00!	**OAS3**	2'-5'-oligoadenylate syntl	protein_coding	15	13	16	14	0.876
4942	NM_000274,NM_00	**OAT**	ornithine aminotransfera:	protein_coding	0	0	1	1	1.000
4943	NM_001348262,NM	**TBC1D25**	TBC1 domain family mer	protein_coding	20	19	21	20	0.949
4946	NM_001301020,NM	**OAZ1**	ornithine decarboxylase	protein_coding	928	986	929	987	1.062
4947	NM_001301302,NM	**OAZ2**	ornithine decarboxylase	protein_coding	86	84	87	85	0.974
4948	NM_000275,NM_00	**OCA2**	OCA2 melanosomal tran	protein_coding	0	0	1	1	1.000
4951	NM_006188	**OCM2**	oncomodulin 2	protein_coding	0	0	1	1	1.000
4952	NM_000276,NM_00	**OCRL**	OCRL inositol polyphosp	protein_coding	0	0	1	1	1.000
4953	NM_001287188,NM	**ODC1**	ornithine decarboxylase	protein_coding	566	621	567	622	1.097
4954	gene-ODCP	**ODCP**	ornithine decarboxylase	pseudogene	0	0	1	1	1.000
4956	NM_024410	**ODF1**	outer dense fiber of sper	protein_coding	0	0	1	1	1.097
4957	NM_001242352,NM	**ODF2**	outer dense fiber of sper	protein_coding	61	63	62	64	1.044
4958	NM_005014	**OMD**	osteomodulin	protein_coding	0	0	1	1	1.000
4967	NM_001003941,NM	**OGDH**	oxoglutarate dehydroger	protein_coding	56	58	57	59	1.039
4968	NM_001354648,NM	**OGG1**	8-oxoguanine DNA glyco	protein_coding	32	34	33	35	1.077
4969	NM_014057,NM_02	**OGN**	osteoglycin	protein_coding	0	0	1	1	1.000
4973	NM_001172632,NM	**OLR1**	oxidized low density lipo	protein_coding	0	0	1	1	1.000
4974	NM_002544	**OMG**	oligodendrocyte myelin g	protein_coding	0	0	1	1	1.175
4975	NM_006189	**OMP**	olfactory marker protein	protein_coding	0	0	1	1	1.000
4976	NM_001354663,NM	**OPA1**	OPA1 mitochondrial dyn	protein_coding	69	61	70	62	0.890
4978	NM_001012393,NM	**OPCML**	opioid binding protein/ce	protein_coding	0	0	1	1	0.992
4982	NM_002546	**TNFRSF11B**	TNF receptor superfamily	protein_coding	0	0	1	1	1.000
4983	NM_002547,XM_00!	**OPHN1**	oligophrenin 1	protein_coding	0	0	1	1	1.023
4985	NM_000911	**OPRD1**	opioid receptor delta 1	protein_coding	0	0	1	1	1.000
4986	NM_000912,NM_00	**OPRK1**	opioid receptor kappa 1	protein_coding	0	0	1	1	1.000
4987	NM_000913,NM_00	**OPRL1**	opioid related nociceptin	protein_coding	0	0	1	1	1.000
4988	NM_000914,NM_00	**OPRM1**	opioid receptor mu 1	protein_coding	0	0	1	1	1.000
4990	NM_007374	**SIX6**	SIX homeobox 6	protein_coding	0	0	1	1	1.000
4991	NM_002548	**OR1D2**	olfactory receptor family	protein_coding	0	0	1	1	1.000
4992	NM_001370639,NM	**OR1F1**	olfactory receptor family	protein_coding	0	0	1	1	1.000
4993	NM_012368	**OR2C1**	olfactory receptor family	protein_coding	0	0	1	1	1.000
4994	NM_002550,XM_01	**OR3A1**	olfactory receptor family	protein_coding	0	0	1	1	1.000
4995	NM_002551,XM_01	**OR3A2**	olfactory receptor family	protein_coding	0	0	1	1	1.000
4998	NM_001190818,NM	**ORC1**	origin recognition comple	protein_coding	77	77	78	78	0.995
4999	NM_006190,NR_03	**ORC2**	origin recognition comple	protein_coding	44	39	45	40	0.882
5000	NM_001190879,NM	**ORC4**	origin recognition comple	protein_coding	10	10	11	11	0.941
5001	NM_002553,NM_18	**ORC5**	origin recognition comple	protein_coding	66	63	67	64	0.950
5002	NM_001315501,NM	**SLC22A18**	solute carrier family 22 m	protein_coding	1	1	2	2	1.398
5003	NM_001302862,NM	**SLC22A18AS**	solute carrier family 22 m	protein_coding	0	0	1	1	1.000
5004	NM_000607	**ORM1**	orosomucoid 1	protein_coding	0	0	1	1	1.000
5005	NM_000608	**ORM2**	orosomucoid 2	protein_coding	0	0	1	1	1.000
5007	NM_002556	**OSBP**	oxysterol binding protein	protein_coding	47	47	48	48	0.987
5008	NM_001319108,NM	**OSM**	oncostatin M	protein_coding	15	15	16	16	1.005
5009	NM_000531,XM_01	**OTC**	ornithine carbamoyltrans	protein_coding	0	0	1	1	1.000
5010	NM_001185056,NM	**CLDN11**	claudin 11	protein_coding	0	0	1	1	1.008
5013	NM_001199770,NM	**OTX1**	orthodenticle homeobox	protein_coding	0	0	1	1	1.031
5015	NM_001270523,NM	**OTX2**	orthodenticle homeobox	protein_coding	0	0	1	1	1.000
5016	NM_002557	**OVGP1**	oviductal glycoprotein 1	protein_coding	1	1	2	2	0.941
5017	NM_004561,XM_00!	**OVOL1**	ovo like transcriptional re	protein_coding	0	0	1	1	1.000
5018	NM_005015	**OXA1L**	OXA1L mitochondrial inn	protein_coding	60	68	61	69	1.118
5019	NM_000436,NM_00	**OXCT1**	3-oxoacid CoA-transfera:	protein_coding	133	135	134	136	1.018
5020	NM_000915,XM_01	**OXT**	oxytocin/neurophysin I p	protein_coding	0	0	1	1	1.000
5021	NM_000916,NM_00	**OXTR**	oxytocin receptor	protein_coding	2	1	3	2	0.761
5023	NM_002558,XM_00	**P2RX1**	purinergic receptor P2X	protein_coding	0	0	1	1	1.028
5024	NM_002559,XM_01	**P2RX3**	purinergic receptor P2X	protein_coding	0	0	1	1	1.037
5025	NM_001256796,NM	**P2RX4**	purinergic receptor P2X	protein_coding	11	11	12	12	0.946
5026	NM_001204519,NM	**P2RX5**	purinergic receptor P2X	protein_coding	1	1	2	2	0.875
5027	NM_002562,NR_03	**P2RX7**	purinergic receptor P2X	protein_coding	5	5	6	6	0.979
5028	NM_002563	**P2RY1**	purinergic receptor P2Y1	protein_coding	0	0	1	1	1.000
5029	NM_002564,NM_17	**P2RY2**	purinergic receptor P2Y2	protein_coding	0	0	1	1	1.000
5030	NM_002565	**P2RY4**	pyrimidinergic receptor F	protein_coding	0	0	1	1	0.950
5031	NM_001277204,NM	**P2RY6**	pyrimidinergic receptor F	protein_coding	0	0	1	1	1.000
5032	NM_002566	**P2RY11**	purinergic receptor P2Y1	protein_coding	0	0	1	1	0.872
5033	NM_000917,NM_00	**P4HA1**	prolyl 4-hydroxylase sub	protein_coding	14	12	15	13	0.881
5034	NM_000918,XM_02	**P4HB**	prolyl 4-hydroxylase sub	protein_coding	406	405	407	406	0.998
5036	NM_006191	**PA2G4**	proliferation-associated 2	protein_coding	465	454	466	455	0.975
5037	NM_002567	**PEBP1**	phosphatidylethanolamir	protein_coding	249	263	250	264	1.058
5042	NM_030979	**PABPC3**	poly(A) binding protein c	protein_coding	0	0	1	1	1.119
5045	NM_001289823,NM	**FURIN**	furin, paired basic amino	protein_coding	5	6	6	7	1.103
5046	NM_001291309,NM	**PCSK6**	proprotein convertase su	protein_coding	0	0	1	1	0.997
5047	NM_001018048,NM	**PAEP**	progestagen associated	protein_coding	0	0	1	1	1.000
5048	NM_000430,XM_01	**PAFAH1B1**	platelet activating factor	protein_coding	51	44	52	45	0.871
5049	NM_001184746,NM	**PAFAH1B2**	platelet activating factor	protein_coding	49	45	50	46	0.915
5050	NM_001145939,NM	**PAFAH1B3**	platelet activating factor	protein_coding	67	70	68	71	1.043
5051	NM_000437,XM_00	**PAFAH2**	platelet activating factor	protein_coding	13	13	14	14	1.053
5052	NM_001202431,NM	**PRDX1**	peroxiredoxin 1	protein_coding	508	487	509	488	0.960
5053	NM_000277,NM_00	**PAH**	phenylalanine hydroxyla:	protein_coding	0	0	1	1	1.000
5054	NM_000602	**SERPINE1**	serpin family E member	protein_coding	3	4	4	5	1.264
5055	NM_001143818,NM	**SERPINB2**	serpin family B member	protein_coding	0	0	1	1	1.000
5058	NM_001128620,NM	**PAK1**	p21 (RAC1) activated kin	protein_coding	0	0	1	1	0.929
5062	NM_002577,XM_01	**PAK2**	p21 (RAC1) activated kin	protein_coding	103	91	104	92	0.876
5063	NM_001128166,NM	**PAK3**	p21 (RAC1) activated kin	protein_coding	0	0	1	1	0.994
5064	NM_001040134,NM	**PALM**	paralemmin	protein_coding	0	0	1	1	1.000
5066	NM_000919,NM_00	**PAM**	peptidylglycine alpha-am	protein_coding	7	5	8	6	0.798
5067	NM_020872,XM_00!	**CNTN3**	contactin 3	protein_coding	0	0	1	1	1.000
5068	NM_002580,NM_13	**REG3A**	regenerating family mem	protein_coding	0	0	1	1	1.000
5069	NM_002581,XM_00	**PAPPA**	pappalysin 1	protein_coding	0	0	1	1	1.009
5071	NM_004562,NM_01	**PRKN**	parkin RBR E3 ubiquitin	protein_coding	0	0	1	1	1.000
5073	NM_001134477,NM	**PARN**	poly(A)-specific ribonucle	protein_coding	61	58	62	59	0.947
5074	NM_001354732,NM	**PAWR**	pro-apoptotic WT1 regul:	protein_coding	0	0	1	1	1.000
5075	NM_001257096,NM	**PAX1**	paired box 1	protein_coding	0	0	1	1	1.000
5076	NM_000278,NM_00	**PAX2**	paired box 2	protein_coding	0	0	1	1	1.318
5077	NM_000438,NM_00	**PAX3**	paired box 3	protein_coding	0	0	1	1	1.000
5078	NM_001366110,NM	**PAX4**	paired box 4	protein_coding	0	0	1	1	1.000
5079	NM_001280547,NM	**PAX5**	paired box 5	protein_coding	1	0	2	1	0.749
5080	NM_000280,NM_00	**PAX6**	paired box 6	protein_coding	0	0	1	1	1.000
5081	NM_001135254,NM	**PAX7**	paired box 7	protein_coding	0	0	1	1	1.000
5082	NM_005388	**PDCL**	phosducin like	protein_coding	15	17	16	18	1.129
5083	NM_001372076,NM	**PAX9**	paired box 9	protein_coding	2	2	3	3	1.168
5087	NM_001204961,NM	**PBX1**	PBX homeobox 1	protein_coding	0	0	1	1	1.000
5088	gene-PBX2P1	**PBX2P1**	PBX homeobox 2 pseud	pseudogene	1	1	2	2	1.006
5089	NM_002586	**PBX2**	PBX homeobox 2	protein_coding	64	59	65	60	0.920
5090	NM_001134778,NM	**PBX3**	PBX homeobox 3	protein_coding	8	8	9	9	1.002
5091	NM_000920,NM_00	**PC**	pyruvate carboxylase	protein_coding	0	0	1	1	1.003
5092	NM_000281,NM_00	**PCBD1**	pterin-4 alpha-carbinolar	protein_coding	9	9	10	10	0.968
5093	NM_006196	**PCBP1**	poly(rC) binding protein	protein_coding	446	440	447	441	0.987
5094	NM_001098620,NM	**PCBP2**	poly(rC) binding protein	protein_coding	269	284	270	285	1.057
5095	NM_000282,NM_00	**PCCA**	propionyl-CoA carboxyla:	protein_coding	14	13	15	14	0.943
5096	NM_000532,NM_00	**PCCB**	propionyl-CoA carboxyla:	protein_coding	66	62	67	63	0.944
5097	NM_001278613,NM	**PCDH1**	protocadherin 1	protein_coding	0	0	1	1	1.000
5098	NM_002588,NM_03	**PCDHGC3**	protocadherin gamma su	protein_coding	0	0	1	1	1.000
5099	NM_001173523,NM	**PCDH7**	protocadherin 7	protein_coding	0	0	1	1	1.005
5100	NM_002590,NM_03	**PCDH8**	protocadherin 8	protein_coding	0	0	1	1	1.000
5101	NM_001318372,NM	**PCDH9**	protocadherin 9	protein_coding	0	0	1	1	1.000
5104	NM_000624	**SERPINA5**	serpin family A member !	protein_coding	0	0	1	1	1.000
5105	NM_002591,XM_02	**PCK1**	phosphoenolpyruvate ca	protein_coding	0	0	1	1	1.000
5106	NM_001018073,NM	**PCK2**	phosphoenolpyruvate ca	protein_coding	32	32	33	33	1.001
5108	NM_001315507,NM	**PCM1**	pericentriolar material 1	protein_coding	37	35	38	36	0.941
5110	NM_001252049,NM	**PCMT1**	protein-L-isoaspartate (D	protein_coding	151	139	152	140	0.923
5111	NM_002592,NM_18	**PCNA**	proliferating cell nuclear	protein_coding	552	538	553	539	0.976
5116	NM_001315529,NM	**PCNT**	pericentrin	protein_coding	19	17	20	18	0.925
5118	NM_002593,XM_02	**PCOLCE**	procollagen C-endopepti	protein_coding	5	4	6	5	0.954
5119	NM_001083314,NM	**CHMP1A**	charged multivesicular b	protein_coding	103	109	104	110	1.057
5121	NM_006198	**PCP4**	Purkinje cell protein 4	protein_coding	0	0	1	1	1.000
5122	NM_000439,NM_00	**PCSK1**	proprotein convertase su	protein_coding	0	0	1	1	1.000
5125	NM_001190482,NM	**PCSK5**	proprotein convertase su	protein_coding	0	0	1	1	1.000

5126	NM_001201528,NM	**PCSK2**	proprotein convertase su	protein_coding	0	0	1	1	1.000
5127	NM_001170460,NM	**CDK16**	cyclin dependent kinase	protein_coding	35	34	36	35	0.982
5128	NM_001170464,NM	**CDK17**	cyclin dependent kinase	protein_coding	26	22	27	23	0.858
5129	NM_002596,NM_21	**CDK18**	cyclin dependent kinase	protein_coding	0	0	1	1	1.000
5130	NM_001312673,NM	**PCYT1A**	phosphate cytidylyltransf	protein_coding	37	39	38	40	1.052
5132	NM_002597,NM_02	**PDC**	phosducin	protein_coding	0	0	1	1	1.000
5133	NM_005018,XM_00	**PDCD1**	programmed cell death 1	protein_coding	0	0	1	1	1.000
5134	NM_001199461,NM	**PDCD2**	programmed cell death 2	protein_coding	94	88	95	89	0.940
5136	NM_001003683,NM	**PDE1A**	phosphodiesterase 1A	protein_coding	0	0	1	1	1.000
5137	NM_001191056,NM	**PDE1C**	phosphodiesterase 1C	protein_coding	0	0	1	1	1.000
5138	NM_001143839,NM	**PDE2A**	phosphodiesterase 2A	protein_coding	0	0	1	1	0.966
5139	NM_000921,NM_00	**PDE3A**	phosphodiesterase 3A	protein_coding	7	6	8	7	0.854
5140	NM_000922,NM_00	**PDE3B**	phosphodiesterase 3B	protein_coding	10	8	11	9	0.873
5141	NM_001111307,NM	**PDE4A**	phosphodiesterase 4A	protein_coding	15	13	16	14	0.862
5142	NM_001037339,NM	**PDE4B**	phosphodiesterase 4B	protein_coding	7	6	8	7	0.872
5143	NM_000923,NM_00	**PDE4C**	phosphodiesterase 4C	protein_coding	0	0	1	1	1.000
5144	NM_001104631,NM	**PDE4D**	phosphodiesterase 4D	protein_coding	26	21	27	22	0.808
5145	NM_000440,XM_01	**PDE6A**	phosphodiesterase 6A	protein_coding	0	0	1	1	1.000
5146	NM_006204	**PDE6C**	phosphodiesterase 6C	protein_coding	0	0	1	1	1.000
5147	NM_001291018,NM	**PDE6D**	phosphodiesterase 6D	protein_coding	31	36	32	37	1.173
5148	NM_001365724,NM	**PDE6G**	phosphodiesterase 6G	protein_coding	1	0	2	1	0.944
5149	NM_006205,XM_01	**PDE6H**	phosphodiesterase 6H	protein_coding	0	0	1	1	1.101
5150	NM_001242318,NM	**PDE7A**	phosphodiesterase 7A	protein_coding	32	31	33	32	0.964
5151	NM_001243137,NM	**PDE8A**	phosphodiesterase 8A	protein_coding	0	0	1	1	0.939
5152	NM_001001567,NM	**PDE9A**	phosphodiesterase 9A	protein_coding	0	0	1	1	1.000
5153	NM_000924,NM_00	**PDE1B**	phosphodiesterase 1B	protein_coding	3	3	4	4	1.015
5154	NM_002607,NM_03	**PDGFA**	platelet derived growth fa	protein_coding	0	0	1	1	1.000
5155	NM_002608,NM_03	**PDGFB**	platelet derived growth fa	protein_coding	0	0	1	1	1.000
5156	NM_001347827,NM	**PDGFRA**	platelet derived growth fa	protein_coding	0	0	1	1	1.000
5157	NM_001372073,NM	**PDGFRL**	platelet derived growth fa	protein_coding	0	0	1	1	1.000
5158	NM_000283,NM_00	**PDE6B**	phosphodiesterase 6B	protein_coding	2	2	3	3	0.951
5159	NM_001355016,NM	**PDGFRB**	platelet derived growth fa	protein_coding	80	75	81	76	0.931
5160	NM_000284,NM_00	**PDHA1**	pyruvate dehydrogenase	protein_coding	114	111	115	112	0.969
5161	NM_005390	**PDHA2**	pyruvate dehydrogenase	protein_coding	0	0	1	1	1.000
5162	NM_000925,NM_00	**PDHB**	pyruvate dehydrogenase	protein_coding	118	110	119	111	0.930
5163	NM_001278549,NM	**PDK1**	pyruvate dehydrogenase	protein_coding	6	4	7	5	0.642
5164	NM_001199898,NM	**PDK2**	pyruvate dehydrogenase	protein_coding	9	11	10	12	1.170
5165	NM_001142386,NM	**PDK3**	pyruvate dehydrogenase	protein_coding	7	6	8	7	0.908
5166	NM_002612	**PDK4**	pyruvate dehydrogenase	protein_coding	0	0	1	1	1.000
5167	NM_006208	**ENPP1**	ectonucleotide pyrophos	protein_coding	0	0	1	1	1.000
5168	NM_001040092,NM	**ENPP2**	ectonucleotide pyrophos	protein_coding	0	0	1	1	1.000
5169	NM_005021,NR_13	**ENPP3**	ectonucleotide pyrophos	protein_coding	0	0	1	1	1.290
5170	NM_001261816,NM	**PDPK1**	3-phosphoinositide depe	protein_coding	37	33	38	34	0.882
5172	NM_000441,XM_00	**SLC26A4**	solute carrier family 26 m	protein_coding	0	0	1	1	0.824
5173	NM_001190892,NM	**PDYN**	prodynorphin	protein_coding	0	0	1	1	1.000
5174	NM_001201325,NM	**PDZK1**	PDZ domain containing 1	protein_coding	0	0	1	1	0.938
5175	NM_000442,XM_00	**PECAM1**	platelet and endothelial c	protein_coding	0	0	1	1	0.986
5176	NM_001329903,NM	**SERPINF1**	serpin family F member 1	protein_coding	0	0	1	1	1.000
5178	NM_001146184,NM	**PEG3**	paternally expressed 3	protein_coding	0	0	1	1	1.000
5179	NM_001135690	**PENK**	proenkephalin	protein_coding	0	0	1	1	1.000
5184	NM_000285,NM_00	**PEPD**	peptidase D	protein_coding	9	11	10	12	1.239
5187	NM_002616,XM_00	**PER1**	period circadian regulato	protein_coding	2	2	3	3	1.009
5188	NM_001363341,NM	**GATB**	glutamyl-tRNA amidotran	protein_coding	22	23	23	24	1.067
5189	NM_000466,NM_00	**PEX1**	peroxisomal biogenesis f	protein_coding	10	12	11	13	1.116
5190	NM_000287,NM_00	**PEX6**	peroxisomal biogenesis f	protein_coding	18	18	19	19	1.000
5191	NM_000288,XM_00	**PEX7**	peroxisomal biogenesis f	protein_coding	4	3	5	4	0.901
5192	NM_001374425,NM	**PEX10**	peroxisomal biogenesis f	protein_coding	12	13	13	14	1.039
5193	NM_000286	**PEX12**	peroxisomal biogenesis f	protein_coding	11	12	12	13	1.114
5194	NM_002618	**PEX13**	peroxisomal biogenesis f	protein_coding	11	9	12	10	0.873
5195	NM_004565,XM_00	**PEX14**	peroxisomal biogenesis f	protein_coding	37	36	38	37	0.964
5196	NM_001363352,NM	**PF4**	platelet factor 4	protein_coding	0	0	1	1	1.000
5197	NM_002620	**PF4V1**	platelet factor 4 variant 1	protein_coding	0	0	1	1	1.000
5198	NM_012393,XM_00	**PFAS**	phosphoribosylformylgly	protein_coding	32	31	33	32	0.967
5199	NM_001145252,NM	**CFP**	complement factor prope	protein_coding	0	0	1	1	0.822
5201	NM_002622,XM_00	**PFDN1**	prefoldin subunit 1	protein_coding	187	174	188	175	0.931
5202	NM_012394,XM_01	**PFDN2**	prefoldin subunit 2	protein_coding	376	386	377	387	1.026
5203	NM_002623,XM_01	**PFDN4**	prefoldin subunit 4	protein_coding	99	91	100	92	0.914
5204	NM_002624,NM_14	**PFDN5**	prefoldin subunit 5	protein_coding	327	356	328	357	1.088
5205	NM_001374385,NM	**ATP8B1**	ATPase phospholipid tra	protein_coding	0	0	1	1	1.000
5207	NM_001271804,NM	**PFKFB1**	6-phosphofructo-2-kinas	protein_coding	0	0	1	1	0.815
5208	NM_001018053,NM	**PFKFB2**	6-phosphofructo-2-kinas	protein_coding	0	0	1	1	1.000
5209	NM_001145443,NM	**PFKFB3**	6-phosphofructo-2-kinas	protein_coding	16	16	17	17	1.020
5210	NM_001317134,NM	**PFKFB4**	6-phosphofructo-2-kinas	protein_coding	1	1	2	2	0.778
5211	NM_001002021,NM	**PFKL**	phosphofructokinase, live	protein_coding	52	55	53	56	1.049
5212	NM_001177969,NM	**VIT**	vitrin	protein_coding	0	0	1	1	1.000
5213	NM_000289,NM_00	**PFKM**	phosphofructokinase, mu	protein_coding	4	4	5	5	0.997
5214	NM_001242339,NM	**PFKP**	phosphofructokinase, pla	protein_coding	148	146	149	147	0.986
5216	NM_001375991,NM	**PFN1**	profilin 1	protein_coding	1,750	1,967	1,751	1,968	1.124
5217	NM_002628,NM_05	**PFN2**	profilin 2	protein_coding	0	0	1	1	1.000
5218	NM_001287135,NM	**CDK14**	cyclin dependent kinase	protein_coding	25	20	26	21	0.812
5222	NM_014224	**PGA5**	pepsinogen A5	protein_coding	0	0	1	1	1.000
5223	NM_001317079,NM	**PGAM1**	phosphoglycerate mutas	protein_coding	468	481	469	482	1.026
5224	NM_000290	**PGAM2**	phosphoglycerate mutas	protein_coding	0	0	1	1	1.000
5225	NM_001166424,NM	**PGC**	progastricsin	protein_coding	0	0	1	1	1.000
5226	NM_001304451,NM	**PGD**	phosphogluconate dehyd	protein_coding	183	192	184	193	1.048
5228	NM_001207012,NM	**PGF**	placental growth factor	protein_coding	0	0	1	1	1.000
5229	NM_005023,XM_00	**PGGT1B**	protein geranylgeranyltra	protein_coding	1	1	2	2	0.878
5230	NM_000291	**PGK1**	phosphoglycerate kinase	protein_coding	345	330	346	331	0.954
5231	gene-PGK1P1	**PGK1P1**	phosphoglycerate kinase	pseudogene	1	1	2	2	1.121
5232	NM_138733	**PGK2**	phosphoglycerate kinase	protein_coding	0	0	1	1	1.000
5233	gene-PGK1P2	**PGK1P2**	phosphoglycerate kinase	pseudogene	0	0	1	1	0.962
5236	NM_001172818,NM	**PGM1**	phosphoglucomutase 1	protein_coding	0	0	1	1	1.039
5238	NM_001199917,NM	**PGM3**	phosphoglucomutase 3	protein_coding	5	5	6	6	0.962
5239	NM_021965,XM_01	**PGM5**	phosphoglucomutase 5	protein_coding	0	0	1	1	1.000
5241	NM_000926,NM_00	**PGR**	progesterone receptor	protein_coding	0	0	1	1	1.000
5243	NM_000927,NM_00	**ABCB1**	ATP binding cassette sul	protein_coding	15	13	16	14	0.844
5244	NM_000443,NM_01	**ABCB4**	ATP binding cassette sul	protein_coding	0	0	1	1	0.960
5245	NM_001281496,NM	**PHB**	prohibitin	protein_coding	487	519	488	520	1.065
5246	gene-PHBP1	**PHBP1**	prohibitin pseudogene 1	pseudogene	0	0	1	1	1.000
5250	NM_002635,NM_00	**SLC25A3**	solute carrier family 25 m	protein_coding	122	131	123	132	1.074
5251	NM_000444,NM_00	**PHEX**	phosphate regulating en	protein_coding	0	0	1	1	1.000
5252	NM_002636,NM_02	**PHF1**	PHD finger protein 1	protein_coding	21	21	22	22	1.021
5253	NM_005392,XM_00	**PHF2**	PHD finger protein 2	protein_coding	9	9	10	10	1.004
5255	NM_001122670,NM	**PHKA1**	phosphorylase kinase re	protein_coding	0	0	1	1	1.000
5256	NM_000292,XM_00	**PHKA2**	phosphorylase kinase re	protein_coding	7	6	8	7	0.868
5257	NM_000293,NM_00	**PHKB**	phosphorylase kinase re	protein_coding	18	17	19	18	0.926
5260	NM_001258459,NM	**PHKG1**	phosphorylase kinase ca	protein_coding	0	1	1	2	1.163
5261	NM_000294,NM_00	**PHKG2**	phosphorylase kinase ca	protein_coding	13	13	14	14	0.983
5262	gene-PHKG1P3	**PHKG1P3**	phosphorylase kinase ca	pseudogene	0	0	1	1	1.000
5264	NM_001037537,NM	**PHYH**	phytanoyl-CoA 2-hydroxy	protein_coding	3	3	4	4	0.992
5265	NM_000295,NM_00	**SERPINA1**	serpin family A member 1	protein_coding	0	0	1	1	1.000
5266	NM_002638	**PI3**	peptidase inhibitor 3	protein_coding	0	0	1	1	1.000
5267	NM_001289032,NM	**SERPINA4**	serpin family A member 4	protein_coding	0	0	1	1	1.000
5268	NM_002639,XM_00	**SERPINB5**	serpin family B member 5	protein_coding	0	0	1	1	1.000
5269	NM_001195291,NM	**SERPINB6**	serpin family B member 6	protein_coding	5	6	6	7	1.145
5270	NM_001136528,NM	**SERPINE2**	serpin family E member 2	protein_coding	0	0	1	1	1.000
5271	NM_001031848,NM	**SERPINB8**	serpin family B member 8	protein_coding	0	0	1	1	1.000
5272	NM_004155,XM_00	**SERPINB9**	serpin family B member 9	protein_coding	180	179	181	180	0.994
5273	NM_005024,XM_01	**SERPINB10**	serpin family B member	protein_coding	0	0	1	1	1.000
5274	NM_001122752,NM	**SERPINI1**	serpin family I member 1	protein_coding	7	7	8	8	0.998
5275	NM_001307923,NM	**SERPINB13**	serpin family B member	protein_coding	0	0	1	1	1.000
5276	NM_001012303,NM	**SERPINI2**	serpin family I member 2	protein_coding	0	0	1	1	1.000
5277	NM_002641,NM_02	**PIGA**	phosphatidylinositol glyc	protein_coding	16	14	17	15	0.906
5278	gene-PIGAP1	**PIGAP1**	phosphatidylinositol glyc	pseudogene	0	0	1	1	1.000
5279	NM_002642,NM_15	**PIGC**	phosphatidylinositol glyc	protein_coding	46	39	47	40	0.845
5280	gene-PIGCP1	**PIGCP1**	phosphatidylinositol glyc	pseudogene	0	0	1	1	0.939

5281	NM_002643,NM_17	**PIGF**	phosphatidylinositol glyc	protein_coding	21	21	22	22	1.004
5282	gene-PIGFP1	**PIGFP1**	phosphatidylinositol glyc	pseudogene	0	0	1	1	1.224
5283	NM_001363694,NM	**PIGH**	phosphatidylinositol glyc	protein_coding	28	25	29	26	0.892
5284	NM_002644,XM_01	**PIGR**	polymeric immunoglobul	protein_coding	0	0	1	1	1.021
5286	NM_001321378,NM	**PIK3C2A**	phosphatidylinositol-4-pł	protein_coding	19	16	20	17	0.847
5287	NM_001377334,NM	**PIK3C2B**	phosphatidylinositol-4-pł	protein_coding	1	1	2	2	0.889
5288	NM_001288772,NM	**PIK3C2G**	phosphatidylinositol-4-pł	protein_coding	0	0	1	1	1.000
5289	NM_001308020,NM	**PIK3C3**	phosphatidylinositol 3-kir	protein_coding	6	6	7	7	0.993
5290	NM_006218,XM_00	**PIK3CA**	phosphatidylinositol-4,5-	protein_coding	9	8	10	9	0.886
5291	NM_001256045,NM	**PIK3CB**	phosphatidylinositol-4,5-	protein_coding	14	13	15	14	0.919
5292	NM_001243186,NM	**PIM1**	Pim-1 proto-oncogene, s	protein_coding	269	244	270	245	0.907
5293	NM_001350234,NM	**PIK3CD**	phosphatidylinositol-4,5-	protein_coding	188	190	189	191	1.012
5294	NM_001282426,NM	**PIK3CG**	phosphatidylinositol-4,5-	protein_coding	66	53	67	54	0.799
5295	NM_001242466,NM	**PIK3R1**	phosphoinositide-3-kinas	protein_coding	38	35	39	36	0.942
5296	NM_005027,NR_07	**PIK3R2**	phosphoinositide-3-kinas	protein_coding	16	16	17	17	1.003
5297	NM_001362862,NM	**PI4KA**	phosphatidylinositol 4-kir	protein_coding	31	29	32	30	0.948
5298	NM_001198773,NM	**PI4KB**	phosphatidylinositol 4-kir	protein_coding	49	47	50	48	0.960
5300	NM_006221,NR_03	**PIN1**	peptidylprolyl cis/trans is	protein_coding	209	245	210	246	1.170
5301	NR_023916	**PIN1P1**	peptidylprolyl cis/trans is	transcribed_pseu	0	0	1	1	1.000
5303	NM_001170747,NM	**PIN4**	peptidylprolyl cis/trans is	protein_coding	46	45	47	46	0.976
5304	NM_002652	**PIP**	prolactin induced protein	protein_coding	0	0	1	1	1.000
5305	NM_001330062,NM	**PIP4K2A**	phosphatidylinositol-5-pł	protein_coding	25	25	26	26	0.995
5306	NM_006224,XM_01	**PITPNA**	phosphatidylinositol tran	protein_coding	22	22	23	23	0.975
5307	NM_002653	**PITX1**	paired like homeodomair	protein_coding	0	0	1	1	1.000
5308	NM_000325,NM_00	**PITX2**	paired like homeodomair	protein_coding	0	0	1	1	1.000
5309	NM_005029	**PITX3**	paired like homeodomair	protein_coding	0	0	1	1	1.000
5310	NM_000296,NM_00	**PKD1**	polycystin 1, transient re	protein_coding	10	10	11	11	0.993
5311	NM_000297,NR_15	**PKD2**	polycystin 2, transient re	protein_coding	2	2	3	3	1.125
5313	NM_000298,NM_18	**PKLR**	pyruvate kinase L/R	protein_coding	0	0	1	1	0.966
5314	NM_138694,NM_17	**PKHD1**	PKHD1 ciliary IPT domai	protein_coding	0	0	1	1	1.000
5315	NM_001206796,NM	**PKM**	pyruvate kinase M1/2	protein_coding	2,206	2,235	2,207	2,236	1.013
5316	NM_001286258,NM	**PKNOX1**	PBX/knotted 1 homeobo	protein_coding	12	12	13	13	1.020
5317	NM_000299,NM_00	**PKP1**	plakophilin 1	protein_coding	0	0	1	1	1.000
5318	NM_001005242,NM	**PKP2**	plakophilin 2	protein_coding	0	0	1	1	1.000
5319	NM_000928	**PLA2G1B**	phospholipase A2 group	protein_coding	0	0	1	1	1.000
5320	NM_000300,NM_00	**PLA2G2A**	phospholipase A2 group	protein_coding	0	0	1	1	1.000
5321	NM_001311193,NM	**PLA2G4A**	phospholipase A2 group	protein_coding	0	0	1	1	1.000
5322	NM_000929,XM_00	**PLA2G5**	phospholipase A2 group	protein_coding	0	0	1	1	1.000
5324	NM_001114634,NM	**PLAG1**	PLAG1 zinc finger	protein_coding	0	0	1	1	1.001
5325	NM_001080951,NM	**PLAGL1**	PLAG1 like zinc finger 1	protein_coding	1	0	2	1	0.913
5326	NM_002657,XM_00	**PLAGL2**	PLAG1 like zinc finger 2	protein_coding	75	69	76	70	0.924
5327	NM_000930,NM_00	**PLAT**	plasminogen activator, ti	protein_coding	0	0	1	1	1.000
5328	NM_001145031,NM	**PLAU**	plasminogen activator, u	protein_coding	10	6	11	7	0.679
5329	NM_001005376,NM	**PLAUR**	plasminogen activator, u	protein_coding	1	1	2	2	0.954
5330	NM_001284297,NM	**PLCB2**	phospholipase C beta 2	protein_coding	34	33	35	34	0.974
5331	NM_000932,NM_00	**PLCB3**	phospholipase C beta 3	protein_coding	2	2	3	3	0.944
5332	NM_000933,NM_00	**PLCB4**	phospholipase C beta 4	protein_coding	0	0	1	1	1.000
5333	NM_001130964,NM	**PLCD1**	phospholipase C delta 1	protein_coding	0	0	1	1	1.052
5334	NM_006226,XM_00	**PLCL1**	phospholipase C like 1 (i	protein_coding	0	0	1	1	1.000
5335	NM_002660,NM_18	**PLCG1**	phospholipase C gamma	protein_coding	63	65	64	66	1.044
5336	NM_002661	**PLCG2**	phospholipase C gamma	protein_coding	18	17	19	18	0.971
5337	NM_001130081,NM	**PLD1**	phospholipase D1	protein_coding	0	0	1	1	1.076
5338	NM_001243108,NM	**PLD2**	phospholipase D2	protein_coding	0	0	1	1	1.177
5339	NM_000445,NM_20	**PLEC**	plectin	protein_coding	4	4	5	5	0.903
5340	NM_000301,NM_00	**PLG**	plasminogen	protein_coding	0	0	1	1	0.996
5341	NM_002664	**PLEK**	pleckstrin	protein_coding	223	229	224	230	1.027
5342	NM_002665	**PLGLB2**	plasminogen like B2	protein_coding	0	0	1	1	1.077
5343	NM_001032392	**PLGLB1**	plasminogen like B1	protein_coding	0	0	1	1	1.040
5345	NM_000934,NM_00	**SERPINF2**	serpin family F member 2	protein_coding	0	0	1	1	1.000
5346	NM_001145311,NM	**PLIN1**	perilipin 1	protein_coding	0	0	1	1	0.968
5347	NM_005030	**PLK1**	polo like kinase 1	protein_coding	123	122	124	123	0.988
5348	NM_001278717,NM	**FXYD1**	FXYD domain containing	protein_coding	0	0	1	1	1.000
5349	NM_001136007,NM	**FXYD3**	FXYD domain containing	protein_coding	0	0	1	1	1.000
5350	NM_002667	**PLN**	phospholamban	protein_coding	0	0	1	1	1.000
5351	NM_000302,NM_00	**PLOD1**	procollagen-lysine,2-oxo	protein_coding	34	30	35	31	0.887
5352	NM_000935,NM_18	**PLOD2**	procollagen-lysine,2-oxo	protein_coding	1	0	2	1	0.907
5354	NM_000533,NM_00	**PLP1**	proteolipid protein 1	protein_coding	0	0	1	1	1.000
5355	NM_002668	**PLP2**	proteolipid protein 2	protein_coding	84	90	85	91	1.067
5356	NM_001201564,NM	**PLRG1**	pleiotropic regulator 1	protein_coding	47	45	48	46	0.966
5357	NM_001145319,NM	**PLS1**	plastin 1	protein_coding	0	0	1	1	0.978
5358	NM_001136025,NM	**PLS3**	plastin 3	protein_coding	0	0	1	1	1.000
5359	NM_001363872,NM	**PLSCR1**	phospholipid scramblase	protein_coding	114	83	115	84	0.736
5360	NM_001242920,NM	**PLTP**	phospholipid transfer pro	protein_coding	0	0	1	1	1.145
5361	NM_032242,XM_01	**PLXNA1**	plexin A1	protein_coding	1	1	2	2	0.936
5362	NM_025179,XM_00	**PLXNA2**	plexin A2	protein_coding	0	0	1	1	1.000
5364	NM_001130082,NM	**PLXNB1**	plexin B1	protein_coding	0	0	1	1	1.000
5365	NM_001163257,NM	**PLXNB3**	plexin B3	protein_coding	0	0	1	1	1.000
5366	NM_001382615,NM	**PMAIP1**	phorbol-12-myristate-13-	protein_coding	95	75	96	76	0.794
5367	NM_002674,XM_01	**PMCH**	pro-melanin concentratin	protein_coding	0	0	1	1	0.928
5368	NM_001284244,NM	**PNOC**	prepronociceptin	protein_coding	0	0	1	1	1.000
5369	NR_003921	**PMCHL1**	pro-melanin concentratin	transcribed_pseu	0	0	1	1	1.000
5370	NR_003922	**PMCHL2**	pro-melanin concentratin	transcribed_pseu	0	0	1	1	1.000
5371	NM_002675,NM_03	**PML**	PML nuclear body scaffo	protein_coding	55	58	56	59	1.060
5372	NM_002676,XM_00	**PMM1**	phosphomannomutase 1	protein_coding	29	30	30	31	1.035
5373	NM_000303,XM_00	**PMM2**	phosphomannomutase 2	protein_coding	61	62	62	63	1.008
5375	NM_001348381,NM	**PMP2**	peripheral myelin protein	protein_coding	0	0	1	1	1.000
5376	NM_000304,NM_00	**PMP22**	peripheral myelin protein	protein_coding	0	0	1	1	1.000
5378	NM_000534,NM_00	**PMS1**	PMS1 homolog 1, misma	protein_coding	48	45	49	46	0.941
5379	NR_003613	**PMS2P1**	PMS1 homolog 2, misma	transcribed_pseu	47	50	48	51	1.056
5380	NR_003614	**PMS2P2**	PMS1 homolog 2, misma	transcribed_pseu	5	5	6	6	0.926
5382	NR_022007,NR_04	**PMS2P4**	PMS1 homolog 2, misma	transcribed_pseu	9	9	10	10	1.044
5383	NR_027775,NR_02	**PMS2P5**	PMS1 homolog 2, misma	transcribed_pseu	16	13	17	14	0.827
5387	NR_028059	**PMS2P3**	PMS1 homolog 2, misma	transcribed_pseu	13	13	14	14	0.976
5393	NM_001034194,NM	**EXOSC9**	exosome component 9	protein_coding	77	71	78	72	0.919
5394	NM_001001998,NM	**EXOSC10**	exosome component 10	protein_coding	94	83	95	84	0.887
5395	NM_000535,NM_00	**PMS2**	PMS1 homolog 2, misma	protein_coding	15	14	16	15	0.943
5396	NM_006902,NM_02	**PRRX1**	paired related homeobox	protein_coding	0	0	1	1	1.000
5406	NM_000936	**PNLIP**	pancreatic lipase	protein_coding	0	0	1	1	1.000
5407	NM_001303135,NM	**PNLIPRP1**	pancreatic lipase related	protein_coding	0	0	1	1	1.000
5408	NR_103727	**PNLIPRP2**	pancreatic lipase related	transcribed_pseu	0	0	1	1	1.000
5409	NM_002686,NR_07	**PNMT**	phenylethanolamine N-m	protein_coding	0	0	1	1	1.000
5411	NM_002687	**PNN**	pinin, desmosome assoc	protein_coding	149	146	150	147	0.982
5412	NM_007106	**UBL3**	ubiquitin like 3	protein_coding	8	8	9	9	0.923
5413	NM_001009939,NM	**SEPTIN5**	septin 5	protein_coding	0	0	1	1	1.000
5414	NM_001038704,NM	**SEPTIN4**	septin 4	protein_coding	0	0	1	1	1.000
5420	NM_001018111,NM	**PODXL**	podocalyxin like	protein_coding	0	0	1	1	1.017
5422	NM_001330360,NM	**POLA1**	DNA polymerase alpha 1	protein_coding	47	42	48	43	0.890
5423	NM_002690,XM_00	**POLB**	DNA polymerase beta	protein_coding	55	55	56	56	1.011
5424	NM_001256849,NM	**POLD1**	DNA polymerase delta 1,	protein_coding	93	98	94	99	1.048
5425	NM_001127218,NM	**POLD2**	DNA polymerase delta 2,	protein_coding	91	91	92	92	0.997
5426	NM_006231,XM_01	**POLE**	DNA polymerase epsilon	protein_coding	23	21	24	22	0.918
5427	NM_001197330,NM	**POLE2**	DNA polymerase epsilon	protein_coding	22	20	23	21	0.912
5428	NM_001126131,NM	**POLG**	DNA polymerase gamma	protein_coding	55	52	56	53	0.948
5429	NM_001291969,NM	**POLH**	DNA polymerase eta	protein_coding	22	18	23	19	0.849
5430	NM_000937	**POLR2A**	RNA polymerase II subur	protein_coding	64	67	65	68	1.033
5431	NM_000938,NM_00	**POLR2B**	RNA polymerase II subur	protein_coding	133	120	134	121	0.905
5432	NM_032940	**POLR2C**	RNA polymerase II subur	protein_coding	128	128	129	129	1.004
5433	NM_004805	**POLR2D**	RNA polymerase II subur	protein_coding	80	76	81	77	0.950
5434	NM_001316323,NM	**POLR2E**	RNA polymerase II subur	protein_coding	140	145	141	146	1.035
5435	NM_001301129,NM	**POLR2F**	RNA polymerase II subur	protein_coding	68	74	69	75	1.085
5436	NM_002696	**POLR2G**	RNA polymerase II subur	protein_coding	194	186	195	187	0.959
5437	NM_001278698,NM	**POLR2H**	RNA polymerase II subur	protein_coding	134	130	135	131	0.966
5438	NM_006233	**POLR2I**	RNA polymerase II subur	protein_coding	111	134	112	135	1.206
5439	NM_001371100,NM	**POLR2J**	RNA polymerase II subur	protein_coding	105	114	106	115	1.087
5440	NM_005034	**POLR2K**	RNA polymerase II subur	protein_coding	195	183	196	184	0.941
5441	NM_021128	**POLR2L**	RNA polymerase II subur	protein_coding	171	193	172	194	1.131

5442	NM_005035,XM_00!	**POLRMT**	RNA polymerase mitocho	protein_coding	59	64	60	65	1.073
5443	NM_000939,NM_00	**POMC**	proopiomelanocortin	protein_coding	15	19	16	20	1.238
5444	NM_000446	**PON1**	paraoxonase 1	protein_coding	0	0	1	1	1.000
5445	NM_000305,NM_00	**PON2**	paraoxonase 2	protein_coding	22	21	23	22	0.931
5446	NM_000940	**PON3**	paraoxonase 3	protein_coding	0	0	1	1	1.000
5447	NM_000941,NM_00	**POR**	cytochrome p450 oxidore	protein_coding	44	38	45	39	0.865
5449	NM_000306,NM_00	**POU1F1**	POU class 1 homeobox 1	protein_coding	0	0	1	1	1.000
5450	NM_006235,XM_00!	**POU2AF1**	POU class 2 homeobox a	protein_coding	0	0	1	1	1.000
5451	NM_001198783,NM	**POU2F1**	POU class 2 homeobox 1	protein_coding	14	11	15	12	0.782
5452	NM_001207025,NM	**POU2F2**	POU class 2 homeobox 2	protein_coding	4	3	5	4	0.918
5453	NM_002699	**POU3F1**	POU class 3 homeobox 1	protein_coding	0	0	1	1	1.000
5454	NM_005604	**POU3F2**	POU class 3 homeobox 2	protein_coding	0	0	1	1	1.000
5455	NM_006236	**POU3F3**	POU class 3 homeobox 3	protein_coding	0	0	1	1	1.000
5456	NM_000307	**POU3F4**	POU class 3 homeobox 4	protein_coding	0	0	1	1	1.000
5457	NM_006237	**POU4F1**	POU class 4 homeobox 1	protein_coding	0	0	1	1	1.000
5458	NM_004575	**POU4F2**	POU class 4 homeobox 2	protein_coding	0	0	1	1	1.000
5459	NM_002700	**POU4F3**	POU class 4 homeobox 3	protein_coding	0	0	1	1	1.094
5460	NM_001173531,NM	**POU5F1**	POU class 5 homeobox 1	protein_coding	0	0	1	1	1.000
5462	NM_001159542	**POU5F1B**	POU class 5 homeobox 1	protein_coding	0	0	1	1	1.000
5463	NM_001330422,NM	**POU6F1**	POU class 6 homeobox 1	protein_coding	1	0	2	1	0.739
5464	NM_021129,XR_00:	**PPA1**	inorganic pyrophosphata	protein_coding	462	326	463	327	0.707
5465	NM_001001928,NM	**PPARA**	peroxisome proliferator a	protein_coding	1	1	2	2	0.946
5467	NM_001171818,NM	**PPARD**	peroxisome proliferator a	protein_coding	18	16	19	17	0.901
5468	NM_001330615,NM	**PPARG**	peroxisome proliferator a	protein_coding	0	0	1	1	1.000
5469	NM_004774,XM_00!	**MED1**	mediator complex subun	protein_coding	83	66	84	67	0.796
5470	NM_006239,XM_01:	**PPEF2**	protein phosphatase with	protein_coding	0	0	1	1	1.000
5471	NM_002703,NR_15!	**PPAT**	phosphoribosyl pyrophos	protein_coding	96	81	97	82	0.841
5473	NM_002704	**PPBP**	pro-platelet basic protein	protein_coding	0	0	1	1	1.000
5475	NM_001377986,NM	**PPEF1**	protein phosphatase with	protein_coding	0	0	1	1	1.000
5476	NM_000308,NM_00	**CTSA**	cathepsin A	protein_coding	86	82	87	83	0.957
5478	NM_001300981,NM	**PPIA**	peptidylprolyl isomerase	protein_coding	1,229	1,211	1,230	1,212	0.985
5479	NM_000942	**PPIB**	peptidylprolyl isomerase	protein_coding	1,137	1,201	1,138	1,202	1.056
5480	NM_000943	**PPIC**	peptidylprolyl isomerase	protein_coding	0	0	1	1	1.000
5481	NM_005038	**PPID**	peptidylprolyl isomerase	protein_coding	31	25	32	26	0.827
5483	gene-PPIAP11	**PPIAP11**	peptidylprolyl isomerase	pseudogene	1	2	2	3	1.491
5485	gene-PPIAP13	**PPIAP13**	peptidylprolyl isomerase	pseudogene	0	0	1	1	1.000
5486	gene-PPIAP14	**PPIAP14**	peptidylprolyl isomerase	pseudogene	0	0	1	1	1.000
5487	gene-PPIAP15	**PPIAP15**	peptidylprolyl isomerase	pseudogene	0	0	1	1	1.003
5488	gene-PPIAP16	**PPIAP16**	peptidylprolyl isomerase	pseudogene	0	0	1	1	1.000
5489	gene-PPIAP7	**PPIAP7**	peptidylprolyl isomerase	pseudogene	0	0	1	1	1.000
5491	gene-PPIAP9	**PPIAP9**	peptidylprolyl isomerase	pseudogene	0	0	1	1	1.000
5493	NM_002705,XM_00(	**PPL**	periplakin	protein_coding	0	0	1	1	1.000
5494	NM_021003,NM_17	**PPM1A**	protein phosphatase, Mg	protein_coding	17	15	18	16	0.871
5495	NM_001033557,NM	**PPM1B**	protein phosphatase, Mg	protein_coding	55	51	56	52	0.940
5496	NM_177983	**PPM1G**	protein phosphatase, Mg	protein_coding	320	327	321	328	1.023
5498	NM_000309,NM_00	**PPOX**	protoporphyrinogen oxid	protein_coding	12	11	13	12	0.944
5499	NM_001008709,NM	**PPP1CA**	protein phosphatase 1 ca	protein_coding	398	439	399	440	1.104
5500	NM_002709,NM_20	**PPP1CB**	protein phosphatase 1 ca	protein_coding	103	94	104	95	0.914
5501	NM_001244974,NM	**PPP1CC**	protein phosphatase 1 ca	protein_coding	334	298	335	299	0.891
5502	NM_006741,XM_00!	**PPP1R1A**	protein phosphatase 1 re	protein_coding	0	0	1	1	1.000
5504	NM_001291504,NM	**PPP1R2**	protein phosphatase 1 re	protein_coding	32	30	33	31	0.945
5506	NM_002711,XM_00!	**PPP1R3A**	protein phosphatase 1 re	protein_coding	0	0	1	1	1.000
5507	NM_005398	**PPP1R3C**	protein phosphatase 1 re	protein_coding	0	0	1	1	1.000
5509	NM_006242	**PPP1R3D**	protein phosphatase 1 re	protein_coding	3	3	4	4	1.115
5510	NM_001282409,NM	**PPP1R7**	protein phosphatase 1 re	protein_coding	197	204	198	205	1.036
5511	NM_002713,NM_01	**PPP1R8**	protein phosphatase 1 re	protein_coding	146	135	147	136	0.929
5512	gene-PPP1R8P1	**PPP1R8P1**	protein phosphatase 1 re	pseudogene	0	0	1	1	1.000
5514	NM_001376195,NM	**PPP1R10**	protein phosphatase 1 re	protein_coding	45	44	46	45	0.987
5515	NM_001355019,NM	**PPP2CA**	protein phosphatase 2 ca	protein_coding	166	163	167	164	0.984
5516	NM_001009552	**PPP2CB**	protein phosphatase 2 ca	protein_coding	149	151	150	152	1.010
5517	gene-PPP2CBP1	**PPP2CBP1**	protein phosphatase 2 ca	pseudogene	0	0	1	1	1.035
5518	NM_001363656,NM	**PPP2R1A**	protein phosphatase 2 sc	protein_coding	79	84	80	85	1.067
5519	NM_001177562,NM	**PPP2R1B**	protein phosphatase 2 sc	protein_coding	80	82	81	83	1.025
5520	NM_001177591,NM	**PPP2R2A**	protein phosphatase 2 re	protein_coding	38	35	39	36	0.923
5521	NM_001271899,NM	**PPP2R2B**	protein phosphatase 2 re	protein_coding	12	13	13	14	1.096
5522	NM_001206994,NM	**PPP2R2C**	protein phosphatase 2 re	protein_coding	0	0	1	1	1.176
5523	NM_001190447,NM	**PPP2R3A**	protein phosphatase 2 re	protein_coding	0	0	1	1	1.019
5524	NM_001193397,NM	**PTPA**	protein phosphatase 2 pl	protein_coding	102	102	103	103	1.001
5525	NM_001199756,NM	**PPP2R5A**	protein phosphatase 2 re	protein_coding	45	41	46	42	0.918
5526	NM_006244,XM_01:	**PPP2R5B**	protein phosphatase 2 re	protein_coding	5	5	6	6	1.088
5527	NM_001161725,NM	**PPP2R5C**	protein phosphatase 2 re	protein_coding	54	56	55	57	1.045
5528	NM_001270476,NM	**PPP2R5D**	protein phosphatase 2 re	protein_coding	48	46	49	47	0.969
5529	NM_001282179,NM	**PPP2R5E**	protein phosphatase 2 re	protein_coding	42	38	43	39	0.906
5530	NM_000944,NM_00	**PPP3CA**	protein phosphatase 3 ca	protein_coding	27	23	28	24	0.864
5531	NM_001303503,NM	**PPP4C**	protein phosphatase 4 ca	protein_coding	209	225	210	226	1.078
5532	NM_001142353,NM	**PPP3CB**	protein phosphatase 3 ca	protein_coding	37	33	38	34	0.891
5533	NM_001243974,NM	**PPP3CC**	protein phosphatase 3 ca	protein_coding	35	34	36	35	0.972
5534	NM_000945	**PPP3R1**	protein phosphatase 3 re	protein_coding	77	77	78	78	0.993
5535	NM_147180	**PPP3R2**	protein phosphatase 3 re	protein_coding	0	0	1	1	1.000
5536	NM_001204284,NM	**PPP5C**	protein phosphatase 5 ca	protein_coding	154	153	155	154	0.998
5537	NM_001123355,NM	**PPP6C**	protein phosphatase 6 ca	protein_coding	48	43	49	44	0.897
5538	NM_000310,NM_00	**PPT1**	palmitoyl-protein thioeste	protein_coding	255	237	256	238	0.930
5539	NM_001319209,NM	**PPY**	pancreatic polypeptide	protein_coding	0	0	1	1	1.000
5540	NM_001278794,NM	**NPY4R**	neuropeptide Y receptor	protein_coding	0	0	1	1	1.000
5542	NR_160307	**PRB1**	proline rich protein BstNI	transcribed_pseu	0	0	1	1	1.000
5544	NM_006249	**PRB3**	proline rich protein BstNI	protein_coding	0	0	1	1	1.000
5545	NM_001261399,NM	**PRB4**	proline rich protein BstNI	protein_coding	0	0	1	1	1.000
5546	NM_005973,XM_00!	**PRCC**	proline rich mitotic check	protein_coding	121	121	122	122	1.007
5547	NM_001319214,NM	**PRCP**	prolylcarboxypeptidase	protein_coding	12	10	13	11	0.874
5549	NM_002725,NM_20	**PRELP**	proline and arginine rich	protein_coding	0	0	1	1	1.000
5550	NM_002726,XM_00!	**PREP**	prolyl endopeptidase	protein_coding	31	31	32	32	0.994
5551	NM_001083116,NM	**PRF1**	perforin 1	protein_coding	442	473	443	474	1.072
5552	NM_001321053,NM	**SRGN**	serglycin	protein_coding	679	626	680	627	0.922
5553	NM_001243245,NM	**PRG2**	proteoglycan 2, pro eosir	protein_coding	2	2	3	3	0.996
5554	NM_001291314,NM	**PRH1**	proline rich protein HaeII	protein_coding	2	2	3	3	1.013
5555	NM_001110213	**PRH2**	proline rich protein HaeII	protein_coding	0	0	1	1	0.962
5557	NM_000946	**PRIM1**	DNA primase subunit 1	protein_coding	79	69	80	70	0.871
5558	NM_000947,NM_00	**PRIM2**	DNA primase subunit 2	protein_coding	45	40	46	41	0.898
5562	NM_001355028,NM	**PRKAA1**	protein kinase AMP-activ	protein_coding	46	40	47	41	0.880
5563	NM_006252,XM_01	**PRKAA2**	protein kinase AMP-activ	protein_coding	0	0	1	1	1.000
5564	NM_006253,XM_00!	**PRKAB1**	protein kinase AMP-activ	protein_coding	21	22	22	23	1.046
5565	NM_005399,NR_10:	**PRKAB2**	protein kinase AMP-activ	protein_coding	9	8	10	9	0.938
5566	NM_001304349,NM	**PRKACA**	protein kinase cAMP-acti	protein_coding	26	24	27	25	0.930
5567	NM_001242857,NM	**PRKACB**	protein kinase cAMP-acti	protein_coding	123	111	124	112	0.905
5568	NM_002732	**PRKACG**	protein kinase cAMP-acti	protein_coding	0	0	1	1	1.000
5569	NM_006823,NM_18	**PKIA**	cAMP-dependent protein	protein_coding	0	0	1	1	1.000
5570	NM_001270393,NM	**PKIB**	cAMP-dependent protein	protein_coding	0	0	1	1	0.933
5571	NM_001206709,NM	**PRKAG1**	protein kinase AMP-activ	protein_coding	63	61	64	62	0.980
5573	NM_001276289,NM	**PRKAR1A**	protein kinase cAMP-dep	protein_coding	195	164	196	165	0.840
5574	gene-PRKAR1AP1	**PRKAR1AP1**	protein kinase cAMP-dep	pseudogene	0	0	1	1	1.000
5575	NM_001164758,NM	**PRKAR1B**	protein kinase cAMP-dep	protein_coding	63	70	64	71	1.100
5576	NM_001321982,NM	**PRKAR2A**	protein kinase cAMP-dep	protein_coding	26	24	27	25	0.937
5577	NM_002736,XM_01	**PRKAR2B**	protein kinase cAMP-dep	protein_coding	5	4	6	5	0.898
5578	NM_002737,XM_01	**PRKCA**	protein kinase C alpha	protein_coding	29	28	30	29	0.969
5579	NM_002738,NM_21	**PRKCB**	protein kinase C beta	protein_coding	79	68	80	69	0.868
5580	NM_001316327,NM	**PRKCD**	protein kinase C delta	protein_coding	13	15	14	16	1.175
5581	NM_005400,XM_00!	**PRKCE**	protein kinase C epsilon	protein_coding	1	0	2	1	0.911
5582	NM_001316329,NM	**PRKCG**	protein kinase C gamma	protein_coding	0	0	1	1	1.000
5583	NM_006255,XM_01	**PRKCH**	protein kinase C eta	protein_coding	228	223	229	224	0.981
5584	NM_002740,XM_01	**PRKCI**	protein kinase C iota	protein_coding	11	11	12	12	0.951
5585	NM_002741,NM_21	**PKN1**	protein kinase N1	protein_coding	74	79	75	80	1.071
5586	NM_001320707,NM	**PKN2**	protein kinase N2	protein_coding	13	11	14	12	0.857
5587	NM_001330069,NM	**PRKD1**	protein kinase D1	protein_coding	0	0	1	1	1.000
5588	NM_001242413,NM	**PRKCQ**	protein kinase C theta	protein_coding	62	60	63	61	0.979
5589	NM_001001329,NM	**PRKCSH**	protein kinase C substra	protein_coding	206	214	207	215	1.036
5590	NM_001033581,NM	**PRKCZ**	protein kinase C zeta	protein_coding	0	0	1	1	1.000

5591	NM_001081640,NM	**PRKDC**	protein kinase, DNA-activ	protein_coding	196	159	197	160	0.811
5592	NM_001098512,NM	**PRKG1**	protein kinase cGMP-dep	protein_coding	0	0	1	1	1.011
5593	NM_001282480,NM	**PRKG2**	protein kinase cGMP-dep	protein_coding	0	0	1	1	1.000
5594	NM_002745,NM_13	**MAPK1**	mitogen-activated proteir	protein_coding	88	81	89	82	0.927
5595	NM_001040056,NM	**MAPK3**	mitogen-activated proteir	protein_coding	32	32	33	33	0.990
5596	NM_001292039,NM	**MAPK4**	mitogen-activated proteir	protein_coding	0	0	1	1	1.000
5597	NM_002748,XM_00	**MAPK6**	mitogen-activated proteir	protein_coding	112	97	113	98	0.872
5598	NM_002749,NM_13	**MAPK7**	mitogen-activated proteir	protein_coding	6	8	7	9	1.272
5599	NM_001278547,NM	**MAPK8**	mitogen-activated proteir	protein_coding	9	8	10	9	0.911
5600	NM_002751,NR_11	**MAPK11**	mitogen-activated proteir	protein_coding	13	12	14	13	0.928
5601	NM_001135044,NM	**MAPK9**	mitogen-activated proteir	protein_coding	25	22	26	23	0.880
5602	NM_001318067,NM	**MAPK10**	mitogen-activated proteir	protein_coding	0	0	1	1	0.979
5603	NM_002754,NR_07	**MAPK13**	mitogen-activated proteir	protein_coding	3	2	4	3	0.868
5604	NM_002755,XM_01	**MAP2K1**	mitogen-activated proteir	protein_coding	82	76	83	77	0.928
5605	NM_030662,XM_00	**MAP2K2**	mitogen-activated proteir	protein_coding	205	210	206	211	1.020
5606	NM_001316332,NM	**MAP2K3**	mitogen-activated proteir	protein_coding	83	83	84	84	1.003
5607	NM_001206804,NM	**MAP2K5**	mitogen-activated proteir	protein_coding	12	11	13	12	0.948
5608	NM_001330450,NM	**MAP2K6**	mitogen-activated proteir	protein_coding	0	0	1	1	1.009
5609	NM_001297555,NM	**MAP2K7**	mitogen-activated proteir	protein_coding	33	34	34	35	1.036
5610	NM_001135651,NM	**EIF2AK2**	eukaryotic translation init	protein_coding	48	35	49	36	0.735
5611	NM_006260,XM_01	**DNAJC3**	DnaJ heat shock protein	protein_coding	45	40	46	41	0.893
5612	NM_004705,NR_13	**THAP12**	THAP domain containing	protein_coding	111	102	112	103	0.912
5613	NM_005044	**PRKX**	protein kinase X-linked	protein_coding	5	4	6	5	0.860
5615	gene-PRKXP2	**PRKXP2**	PRKX pseudogene 2	pseudogene	0	0	1	1	0.945
5616	NR_028062	**PRKY**	protein kinase Y-linked (p	transcribed_pseu	4	4	5	5	0.934
5617	NM_000948,NM_00	**PRL**	prolactin	protein_coding	0	0	1	1	1.000
5618	NM_000949,NM_00	**PRLR**	prolactin receptor	protein_coding	0	0	1	1	1.000
5619	NM_002761	**PRM1**	protamine 1	protein_coding	0	0	1	1	1.000
5620	NM_001286356,NM	**PRM2**	protamine 2	protein_coding	0	0	1	1	1.000
5621	NM_000311,NM_00	**PRNP**	prion protein	protein_coding	82	69	83	70	0.847
5623	NM_004158	**PSPN**	persephin	protein_coding	0	0	1	1	1.113
5624	NM_000312,NM_00	**PROC**	protein C, inactivator of c	protein_coding	0	0	1	1	1.000
5625	NM_001195226,NM	**PRODH**	proline dehydrogenase 1	protein_coding	0	0	1	1	1.000
5626	NM_006261	**PROP1**	PROP paired-like homec	protein_coding	0	0	1	1	1.000
5627	NM_000313,NM_00	**PROS1**	protein S	protein_coding	0	0	1	1	1.000
5629	NM_001270616,NM	**PROX1**	prospero homeobox 1	protein_coding	0	0	1	1	1.000
5630	NM_006262,XM_00	**PRPH**	peripherin	protein_coding	0	0	1	1	1.000
5631	NM_001204402,NM	**PRPS1**	phosphoribosyl pyrophos	protein_coding	167	160	168	161	0.956
5634	NM_001039091,NM	**PRPS2**	phosphoribosyl pyrophos	protein_coding	69	63	70	64	0.914
5635	NM_001330503,NM	**PRPSAP1**	phosphoribosyl pyrophos	protein_coding	20	25	21	26	1.212
5636	NM_001243936,NM	**PRPSAP2**	phosphoribosyl pyrophos	protein_coding	31	28	32	29	0.920
5638	NM_000950,NM_00	**PRRG1**	proline rich and Gla dom	protein_coding	0	0	1	1	1.000
5639	NM_000951,NM_00	**PRRG2**	proline rich and Gla dom	protein_coding	2	2	3	3	1.111
5641	NM_001008530,NM	**LGMN**	legumain	protein_coding	0	1	1	2	1.024
5644	NM_002769,XM_01	**PRSS1**	serine protease 1	protein_coding	0	0	1	1	1.000
5645	NM_001303414,NM	**PRSS2**	serine protease 2	protein_coding	0	0	1	1	1.000
5646	NM_001197097,NM	**PRSS3**	serine protease 3	protein_coding	0	0	1	1	1.000
5648	NM_001031849,NM	**MASP1**	mannan binding lectin se	protein_coding	0	0	1	1	1.000
5649	NM_005045,NM_17	**RELN**	reelin	protein_coding	0	0	1	1	1.009
5650	NM_001207053,NM	**KLK7**	kallikrein related peptida	protein_coding	0	0	1	1	1.000
5651	NM_002772,XM_01	**TMPRSS15**	transmembrane serine p	protein_coding	0	0	1	1	1.000
5652	NM_002773	**PRSS8**	serine protease 8	protein_coding	0	0	1	1	1.000
5653	NM_001012964,NM	**KLK6**	kallikrein related peptida	protein_coding	0	0	1	1	1.000
5654	NM_002775	**HTRA1**	HtrA serine peptidase 1	protein_coding	0	0	1	1	1.000
5655	NM_001077500,NM	**KLK10**	kallikrein related peptida	protein_coding	0	0	1	1	1.000
5657	NM_002777,XM_01	**PRTN3**	proteinase 3	protein_coding	0	0	1	1	1.000
5660	NM_001042465,NM	**PSAP**	prosaposin	protein_coding	33	35	34	36	1.057
5662	NM_001270965,NM	**PSD**	pleckstrin and Sec7 dom	protein_coding	0	0	1	1	1.129
5663	NM_000021,NM_00	**PSEN1**	presenilin 1	protein_coding	23	19	24	20	0.834
5664	NM_000447,NM_01	**PSEN2**	presenilin 2	protein_coding	0	0	1	1	1.030
5669	NM_001184825,NM	**PSG1**	pregnancy specific beta-	protein_coding	0	0	1	1	1.000
5670	NM_031246	**PSG2**	pregnancy specific beta-	protein_coding	0	0	1	1	1.000
5671	NM_021016,XM_01	**PSG3**	pregnancy specific beta-	protein_coding	0	0	1	1	1.000
5672	NM_001276495,NM	**PSG4**	pregnancy specific beta-	protein_coding	0	0	1	1	1.000
5673	NM_001130014,NM	**PSG5**	pregnancy specific beta-	protein_coding	0	0	1	1	1.000
5675	NM_001031850,NM	**PSG6**	pregnancy specific beta-	protein_coding	0	0	1	1	1.000
5676	NM_001206650,NM	**PSG7**	pregnancy specific beta-	protein_coding	0	0	1	1	1.000
5678	NM_001301707,NM	**PSG9**	pregnancy specific beta-	protein_coding	0	0	1	1	1.000
5680	NM_001113410,NM	**PSG11**	pregnancy specific beta-	protein_coding	0	0	1	1	1.000
5681	NM_006742	**PSKH1**	protein serine kinase H1	protein_coding	13	13	14	14	1.012
5682	NM_001143937,NM	**PSMA1**	proteasome 20S subunit	protein_coding	532	508	533	509	0.955
5683	NM_002787	**PSMA2**	proteasome 20S subunit	protein_coding	196	195	197	196	0.993
5684	NM_002788,NM_15	**PSMA3**	proteasome 20S subunit	protein_coding	334	316	335	317	0.947
5685	NM_001102667,NM	**PSMA4**	proteasome 20S subunit	protein_coding	254	189	255	190	0.743
5686	NM_001199772,NM	**PSMA5**	proteasome 20S subunit	protein_coding	145	140	146	141	0.966
5687	NM_001282232,NM	**PSMA6**	proteasome 20S subunit	protein_coding	298	275	299	276	0.924
5688	NM_002792	**PSMA7**	proteasome 20S subunit	protein_coding	1,029	1,089	1,030	1,090	1.058
5689	NM_002793	**PSMB1**	proteasome 20S subunit	protein_coding	386	398	387	399	1.032
5690	NM_001199779,NM	**PSMB2**	proteasome 20S subunit	protein_coding	174	174	175	175	1.000
5691	NM_002795,NR_10	**PSMB3**	proteasome 20S subunit	protein_coding	526	540	527	541	1.027
5692	NM_002796	**PSMB4**	proteasome 20S subunit	protein_coding	590	589	591	590	0.998
5693	NM_001130725,NM	**PSMB5**	proteasome 20S subunit	protein_coding	145	152	146	153	1.045
5694	NM_001270481,NM	**PSMB6**	proteasome 20S subunit	protein_coding	184	183	185	184	0.998
5695	NM_002799	**PSMB7**	proteasome 20S subunit	protein_coding	320	315	321	316	0.985
5696	NM_004159,NM_14	**PSMB8**	proteasome 20S subunit	protein_coding	558	566	559	567	1.014
5697	NM_004160	**PYY**	peptide YY	protein_coding	0	0	1	1	1.000
5698	NM_002800	**PSMB9**	proteasome 20S subunit	protein_coding	374	406	375	407	1.086
5699	NM_002801	**PSMB10**	proteasome 20S subunit	protein_coding	238	254	239	255	1.067
5700	NM_001330212,NM	**PSMC1**	proteasome 26S subunit	protein_coding	68	69	69	70	1.028
5701	NM_001204453,NM	**PSMC2**	proteasome 26S subunit	protein_coding	140	131	141	132	0.936
5702	NM_002804,XM_01	**PSMC3**	proteasome 26S subunit	protein_coding	318	326	319	327	1.024
5703	gene-PSMC3P1	**PSMC3P1**	proteasome 26S subunit	pseudogene	0	0	1	1	1.000
5704	NM_006503,NM_15	**PSMC4**	proteasome 26S subunit	protein_coding	187	199	188	200	1.064
5705	NM_001199163,NM	**PSMC5**	proteasome 26S subunit	protein_coding	341	347	342	348	1.016
5706	NM_001366414,NM	**PSMC6**	proteasome 26S subunit	protein_coding	102	102	103	103	0.995
5707	NM_001191037,NM	**PSMD1**	proteasome 26S subunit	protein_coding	216	192	217	193	0.889
5708	NM_001278708,NM	**PSMD2**	proteasome 26S subunit	protein_coding	329	335	330	336	1.019
5709	NM_002809	**PSMD3**	proteasome 26S subunit	protein_coding	269	267	270	268	0.994
5710	NM_001330692,NM	**PSMD4**	proteasome 26S subunit	protein_coding	323	356	324	357	1.102
5711	NM_001270427,NM	**PSMD5**	proteasome 26S subunit	protein_coding	54	49	55	50	0.920
5713	NM_002811	**PSMD7**	proteasome 26S subunit	protein_coding	292	270	293	271	0.926
5714	NM_002812	**PSMD8**	proteasome 26S subunit	protein_coding	325	338	326	339	1.040
5715	NM_001261400,NM	**PSMD9**	proteasome 26S subunit	protein_coding	38	41	39	42	1.075
5716	NM_002814,NM_17	**PSMD10**	proteasome 26S subunit	protein_coding	49	44	50	45	0.891
5717	NM_001270482,NM	**PSMD11**	proteasome 26S subunit	protein_coding	136	127	137	128	0.935
5718	NM_001316341,NM	**PSMD12**	proteasome 26S subunit	protein_coding	78	70	79	71	0.902
5719	NM_002817,NM_17	**PSMD13**	proteasome 26S subunit	protein_coding	310	293	311	294	0.947
5720	NM_001281528,NM	**PSME1**	proteasome activator sub	protein_coding	541	548	542	549	1.014
5721	NM_002818,XM_00	**PSME2**	proteasome activator sub	protein_coding	973	959	974	960	0.986
5723	NM_001370503,NM	**PSPH**	phosphoserine phosphat	protein_coding	19	20	20	21	1.089
5724	NM_000952,NM_00	**PTAFR**	platelet activating factor	protein_coding	4	4	5	5	0.985
5725	NM_002819,NM_03	**PTBP1**	polypyrimidine tract bind	protein_coding	419	398	420	399	0.952
5726	NM_176817	**TAS2R38**	taste 2 receptor member	protein_coding	0	0	1	1	1.088
5727	NM_000264,NM_00	**PTCH1**	patched 1	protein_coding	0	0	1	1	0.976
5728	NM_000314,NM_00	**PTEN**	phosphatase and tensin	protein_coding	29	28	30	29	0.954
5729	NM_000953,NM_00	**PTGDR**	prostaglandin D2 recepto	protein_coding	8	7	9	8	0.970
5730	NM_000954	**PTGDS**	prostaglandin D2 syntha	protein_coding	0	0	1	1	1.000
5731	NM_000955	**PTGER1**	prostaglandin E receptor	protein_coding	0	0	1	1	1.000
5732	NM_000956	**PTGER2**	prostaglandin E receptor	protein_coding	99	91	100	92	0.919
5733	NM_001126044,NM	**PTGER3**	prostaglandin E receptor	protein_coding	26	30	27	31	1.142
5734	NM_000958,XM_01	**PTGER4**	prostaglandin E receptor	protein_coding	22	20	23	21	0.886
5737	NM_000959,NM_00	**PTGFR**	prostaglandin F receptor	protein_coding	0	0	1	1	1.000
5738	NM_020440,XM_01	**PTGFRN**	prostaglandin F2 recepto	protein_coding	0	0	1	1	1.000
5739	NM_000960,XM_00	**PTGIR**	prostaglandin I2 recepto	protein_coding	0	0	1	1	1.000
5740	NM_000961	**PTGIS**	prostaglandin I2 synthas	protein_coding	0	0	1	1	0.999
5741	NM_000315,NM_00	**PTH**	parathyroid hormone	protein_coding	0	0	1	1	1.000

5742	NM_000962,NM_00	**PTGS1**	prostaglandin-endoperox	protein_coding	0	0	1	1	0.990
5743	NM_000963	**PTGS2**	prostaglandin-endoperox	protein_coding	0	0	1	1	1.000
5744	NM_002820,NM_19	**PTHLH**	parathyroid hormone like	protein_coding	0	0	1	1	1.000
5745	NM_000316,NM_00	**PTH1R**	parathyroid hormone 1 re	protein_coding	0	0	1	1	1.000
5746	NM_001309516,NM	**PTH2R**	parathyroid hormone 2 re	protein_coding	0	0	1	1	1.000
5747	NM_001199649,NM	**PTK2**	protein tyrosine kinase 2	protein_coding	0	0	1	1	1.000
5753	NM_001256358,NM	**PTK6**	protein tyrosine kinase 6	protein_coding	0	0	1	1	1.000
5754	NM_001270398,NM	**PTK7**	protein tyrosine kinase 7	protein_coding	1	2	2	3	1.249
5756	NM_001242397,NM	**TWF1**	twinfilin actin binding pro	protein_coding	39	36	40	37	0.914
5757	NM_001099285,NM	**PTMA**	prothymosin alpha	protein_coding	8,584	8,792	8,585	8,793	1.024
5763	NM_001330333,NM	**PTMS**	parathymosin	protein_coding	29	34	30	35	1.168
5764	NM_001321386,NM	**PTN**	pleiotrophin	protein_coding	0	0	1	1	1.000
5767	gene-PTP4A2P1	**PTP4A2P1**	PTP4A2 pseudogene 1	pseudogene	1	2	2	3	1.268
5768	NM_001004128,NM	**QSOX1**	quiescin sulfhydryl oxida	protein_coding	35	23	36	24	0.664
5769	gene-PTP4A2P2	**PTP4A2P2**	PTP4A2 pseudogene 2	pseudogene	0	0	1	1	0.977
5770	NM_001278618,NM	**PTPN1**	protein tyrosine phospha	protein_coding	124	121	125	122	0.969
5771	NM_001207013,NM	**PTPN2**	protein tyrosine phospha	protein_coding	31	36	32	37	1.164
5774	NM_001145368,NM	**PTPN3**	protein tyrosine phospha	protein_coding	0	0	1	1	1.000
5775	NM_002830	**PTPN4**	protein tyrosine phospha	protein_coding	12	10	13	11	0.868
5777	NM_002831,NM_08	**PTPN6**	protein tyrosine phospha	protein_coding	5	4	6	5	0.986
5778	NM_001199797,NM	**PTPN7**	protein tyrosine phospha	protein_coding	203	219	204	220	1.079
5780	NM_002833	**PTPN9**	protein tyrosine phospha	protein_coding	21	20	22	21	0.934
5781	NM_001330437,NM	**PTPN11**	protein tyrosine phospha	protein_coding	122	104	123	105	0.852
5782	NM_001131008,NM	**PTPN12**	protein tyrosine phospha	protein_coding	19	15	20	16	0.770
5783	NM_006264,NM_08	**PTPN13**	protein tyrosine phospha	protein_coding	0	0	1	1	1.000
5784	NM_005401,XM_01	**PTPN14**	protein tyrosine phospha	protein_coding	0	0	1	1	0.973
5786	NM_002836,NM_08	**PTPRA**	protein tyrosine phospha	protein_coding	49	48	50	49	0.973
5787	NM_001109754,NM	**PTPRB**	protein tyrosine phospha	protein_coding	0	0	1	1	1.000
5788	NM_001267798,NM	**PTPRC**	protein tyrosine phospha	protein_coding	343	325	344	326	0.947
5789	NM_001040712,NM	**PTPRD**	protein tyrosine phospha	protein_coding	0	0	1	1	1.000
5790	NM_005608	**PTPRCAP**	protein tyrosine phospha	protein_coding	631	672	632	673	1.063
5791	NM_001316676,NM	**PTPRE**	protein tyrosine phospha	protein_coding	27	23	28	24	0.870
5792	NM_001329137,NM	**PTPRF**	protein tyrosine phospha	protein_coding	0	0	1	1	0.976
5793	NM_001375471,NM	**PTPRG**	protein tyrosine phospha	protein_coding	0	0	1	1	1.010
5794	NM_001161440,NM	**PTPRH**	protein tyrosine phospha	protein_coding	0	0	1	1	1.000
5795	NM_001098503,NM	**PTPRJ**	protein tyrosine phospha	protein_coding	39	35	40	36	0.895
5796	NM_001135648,NM	**PTPRK**	protein tyrosine phospha	protein_coding	0	0	1	1	0.956
5797	NM_001105244,NM	**PTPRM**	protein tyrosine phospha	protein_coding	0	0	1	1	1.006
5798	NM_001199763,NM	**PTPRN**	protein tyrosine phospha	protein_coding	0	0	1	1	1.000
5799	NM_001308267,NM	**PTPRN2**	protein tyrosine phospha	protein_coding	0	0	1	1	0.995
5800	NM_002848,NM_03	**PTPRO**	protein tyrosine phospha	protein_coding	0	0	1	1	1.000
5801	NM_001207015,NM	**PTPRR**	protein tyrosine phospha	protein_coding	1	0	2	1	0.971
5802	NM_002850,NM_13	**PTPRS**	protein tyrosine phospha	protein_coding	0	0	1	1	1.000
5803	NM_001206838,NM	**PTPRZ1**	protein tyrosine phospha	protein_coding	0	0	1	1	1.004
5805	NM_000317	**PTS**	6-pyruvoyltetrahydropter	protein_coding	43	41	44	42	0.939
5806	NM_002852	**PTX3**	pentraxin 3	protein_coding	0	0	1	1	1.106
5810	NM_002853,NR_02	**RAD1**	RAD1 checkpoint DNA e	protein_coding	41	35	42	36	0.874
5813	NM_005859	**PURA**	purine rich element bindi	protein_coding	11	10	12	11	0.951
5814	NM_033224	**PURB**	purine rich element bindi	protein_coding	22	19	23	20	0.876
5816	NM_001315532,NM	**PVALB**	parvalbumin	protein_coding	0	0	1	1	1.000
5817	NM_001135768,NM	**PVR**	PVR cell adhesion molec	protein_coding	0	0	1	1	1.000
5818	NM_002855,NM_20	**NECTIN1**	nectin cell adhesion mole	protein_coding	0	0	1	1	1.000
5819	NM_001042724,NM	**NECTIN2**	nectin cell adhesion mole	protein_coding	0	0	1	1	1.000
5820	NR_003367	**PVT1**	Pvt1 oncogene	lncRNA	14	13	15	14	0.942
5822	NM_005049,XM_01	**PWP2**	PWP2 small subunit proc	protein_coding	84	77	85	78	0.913
5824	NM_001193644,NM	**PEX19**	peroxisomal biogenesis f	protein_coding	60	56	61	57	0.930
5825	NM_001122674,NM	**ABCD3**	ATP binding cassette sul	protein_coding	42	38	43	39	0.909
5826	NM_001353591,NM	**ABCD4**	ATP binding cassette sul	protein_coding	12	12	13	13	1.008
5827	NM_018663	**PXMP2**	peroxisomal membrane p	protein_coding	20	23	21	24	1.122
5828	NM_000318,NM_00	**PEX2**	peroxisomal biogenesis f	protein_coding	32	29	33	30	0.931
5829	NM_001080855,NM	**PXN**	paxillin	protein_coding	207	207	208	208	1.002
5830	NM_000319,NM_00	**PEX5**	peroxisomal biogenesis f	protein_coding	10	9	11	10	0.945
5831	NM_001282279,NM	**PYCR1**	pyrroline-5-carboxylate re	protein_coding	112	105	113	106	0.939
5832	NM_001017423,NM	**ALDH18A1**	aldehyde dehydrogenase	protein_coding	118	119	119	120	1.008
5833	NM_001184917,NM	**PCYT2**	phosphate cytidylyltransf	protein_coding	12	14	13	15	1.192
5834	NM_002862,XR_00	**PYGB**	glycogen phosphorylase	protein_coding	67	64	68	65	0.954
5836	NM_001163940,NM	**PYGL**	glycogen phosphorylase	protein_coding	0	0	1	1	1.000
5837	NM_001164716,NM	**PYGM**	glycogen phosphorylase	protein_coding	0	1	1	2	1.178
5858	NM_002864,XM_01	**PZP**	PZP alpha-2-macroglobu	protein_coding	0	0	1	1	1.000
5859	NM_001272073,NM	**QARS1**	glutaminyl-tRNA syntheta	protein_coding	161	152	162	153	0.948
5860	NM_000320,NM_00	**QDPR**	quinoid dihydropteridine	protein_coding	0	0	1	1	1.000
5861	NM_004161,NM_01	**RAB1A**	RAB1A, member RAS on	protein_coding	74	69	75	70	0.933
5862	NM_001242644,NM	**RAB2A**	RAB2A, member RAS on	protein_coding	43	40	44	41	0.930
5863	NM_001243738,NM	**RGL2**	ral guanine nucleotide di	protein_coding	16	15	17	16	0.944
5864	NM_002866,XM_01	**RAB3A**	RAB3A, member RAS on	protein_coding	2	2	3	3	1.104
5865	NM_002867,XM_01	**RAB3B**	RAB3B, member RAS on	protein_coding	0	0	1	1	1.010
5866	NM_001271686,NM	**RAB3IL1**	RAB3A interacting protei	protein_coding	0	0	1	1	1.000
5867	NM_001271998,NM	**RAB4A**	RAB4A, member RAS on	protein_coding	22	20	23	21	0.899
5868	NM_001292048,NM	**RAB5A**	RAB5A, member RAS on	protein_coding	58	51	59	52	0.890
5869	NM_001252036,NM	**RAB5B**	RAB5B, member RAS on	protein_coding	29	30	30	31	1.016
5870	NM_001243718,NM	**RAB6A**	RAB6A, member RAS on	protein_coding	74	68	75	69	0.927
5871	NM_001307990,NM	**MAP4K2**	mitogen-activated protei	protein_coding	1	1	2	2	0.921
5872	NM_001272038,NM	**RAB13**	RAB13, member RAS on	protein_coding	0	1	1	2	1.200
5873	NM_004580,NM_18	**RAB27A**	RAB27A, member RAS o	protein_coding	164	148	165	149	0.903
5874	NM_001375327,NM	**RAB27B**	RAB27B, member RAS o	protein_coding	1	1	2	2	1.033
5875	NM_004581,NM_18	**RABGGTA**	Rab geranylgeranyltrans	protein_coding	19	18	20	19	0.968
5876	NM_004582,NR_07	**RABGGTB**	Rab geranylgeranyltrans	protein_coding	97	88	98	89	0.911
5877	NM_002871	**RABIF**	RAB interacting factor	protein_coding	13	12	14	13	0.925
5878	NM_001252039,NM	**RAB5C**	RAB5C, member RAS on	protein_coding	234	239	235	240	1.019
5879	NM_006908,NM_01	**RAC1**	Rac family small GTPase	protein_coding	140	130	141	131	0.927
5880	NM_002872,XM_00	**RAC2**	Rac family small GTPase	protein_coding	606	639	607	640	1.054
5881	NM_001316307,NM	**RAC3**	Rac family small GTPase	protein_coding	4	2	5	3	0.723
5883	NM_001243224,NM	**RAD9A**	RAD9 checkpoint clamp	protein_coding	21	22	22	23	1.073
5884	NM_001278622,NM	**RAD17**	RAD17 checkpoint clamp	protein_coding	62	58	63	59	0.933
5885	NM_006265	**RAD21**	RAD21 cohesin complex	protein_coding	314	271	315	272	0.864
5886	NM_001270362,NM	**RAD23A**	RAD23 homolog A, nucle	protein_coding	186	184	187	185	0.990
5887	NM_001244713,NM	**RAD23B**	RAD23 homolog B, nucle	protein_coding	215	199	216	200	0.925
5888	NM_001164269,NM	**RAD51**	RAD51 recombinase	protein_coding	51	53	52	54	1.035
5889	NM_002876,NM_05	**RAD51C**	RAD51 paralog C	protein_coding	85	96	86	97	1.136
5890	NM_001321809,NM	**RAD51B**	RAD51 paralog B	protein_coding	9	6	10	7	0.703
5891	NM_001272011,NM	**MOK**	MOK protein kinase	protein_coding	1	2	2	3	1.275
5892	NM_001142571,NM	**RAD51D**	RAD51 paralog D	protein_coding	20	22	21	23	1.060
5893	NM_001297419,NM	**RAD52**	RAD52 homolog, DNA re	protein_coding	5	5	6	6	1.008
5894	NM_001354689,NM	**RAF1**	Raf-1 proto-oncogene, se	protein_coding	71	65	72	66	0.925
5896	NM_000448,NM_00	**RAG1**	recombination activating	protein_coding	0	0	1	1	0.982
5897	NM_000536,NM_00	**RAG2**	recombination activating	protein_coding	0	0	1	1	1.000
5898	NM_005402,XM_00	**RALA**	RAS like proto-oncogene	protein_coding	37	35	38	36	0.939
5899	NM_001369400,NM	**RALB**	RAS like proto-oncogene	protein_coding	42	42	43	43	0.989
5900	NM_001042368,NM	**RALGDS**	ral guanine nucleotide di	protein_coding	10	12	11	13	1.193
5901	NM_001300796,NM	**RAN**	RAN, member RAS onco	protein_coding	694	699	695	700	1.007
5902	NM_001278639,NM	**RANBP1**	RAN binding protein 1	protein_coding	427	472	428	473	1.106
5903	NM_006267,XM_00	**RANBP2**	RAN binding protein 2	protein_coding	54	44	55	45	0.813
5905	NM_001278651,NM	**RANGAP1**	Ran GTPase activating p	protein_coding	140	133	141	134	0.949
5906	NM_001010935,NM	**RAP1A**	RAP1A, member of RAS	protein_coding	150	123	151	124	0.822
5907	gene-RAP1AP	**RAP1AP**	RAP1A, member of RAS	pseudogene	0	0	1	1	0.927
5908	NM_001010942,NM	**RAP1B**	RAP1B, member of RAS	protein_coding	37	34	38	35	0.930
5909	NM_001145657,NM	**RAP1GAP**	RAP1 GTPase activating	protein_coding	0	0	1	1	0.869
5910	NM_001100426,NM	**RAP1GDS1**	Rap1 GTPase-GDP diss	protein_coding	15	12	16	13	0.822
5911	NM_021033	**RAP2A**	RAP2A, member of RAS	protein_coding	119	108	120	109	0.901
5912	NM_002886	**RAP2B**	RAP2B, member of RAS	protein_coding	20	19	21	20	0.976
5913	NM_005055,NM_03	**RAPSN**	receptor associated prot	protein_coding	0	0	1	1	1.242
5914	NM_000964,NM_00	**RARA**	retinoic acid receptor alp	protein_coding	17	14	18	15	0.820
5915	NM_000965,NM_00	**RARB**	retinoic acid receptor bet	protein_coding	0	0	1	1	1.000
5916	NM_000966,NM_00	**RARG**	retinoic acid receptor gar	protein_coding	0	1	1	2	1.149
5917	NM_002887	**RARS1**	arginyl-tRNA synthetase	protein_coding	123	119	124	120	0.966
5918	NM_002888,NM_20	**RARRES1**	retinoic acid receptor res	protein_coding	0	0	1	1	1.000

5919	NM_002889,XM_01	**RARRES2**	retinoic acid receptor res	protein_coding	0	0	1	1	1.000
5920	NM_004585	**PLAAT4**	phospholipase A and acy	protein_coding	108	112	109	113	1.033
5921	NM_002890,NM_02	**RASA1**	RAS p21 protein activato	protein_coding	19	18	20	19	0.916
5922	NM_001303245,NM	**RASA2**	RAS p21 protein activato	protein_coding	29	24	30	25	0.834
5923	NM_001145648,NM	**RASGRF1**	Ras protein specific guar	protein_coding	0	0	1	1	1.000
5924	NM_006909,XM_00	**RASGRF2**	Ras protein specific guar	protein_coding	0	0	1	1	1.009
5925	NM_000321,XM_01	**RB1**	RB transcriptional corepr	protein_coding	133	116	134	117	0.872
5926	NM_002892,NM_02	**ARID4A**	AT-rich interaction domai	protein_coding	12	10	13	11	0.825
5927	NM_001042603	**KDM5A**	lysine demethylase 5A	protein_coding	14	12	15	13	0.866
5928	NM_001135255,NM	**RBBP4**	RB binding protein 4, chr	protein_coding	136	130	137	131	0.958
5929	NM_001193272,NM	**RBBP5**	RB binding protein 5, his	protein_coding	29	28	30	29	0.958
5930	NM_006910,NM_01	**RBBP6**	RB binding protein 6, ub	protein_coding	56	48	57	49	0.859
5931	NM_001198719,NM	**RBBP7**	RB binding protein 7, chr	protein_coding	244	248	245	249	1.018
5932	NM_002894,NM_20	**RBBP8**	RB binding protein 8, en	protein_coding	111	96	112	97	0.866
5933	NM_001323281,NM	**RBL1**	RB transcriptional corepr	protein_coding	32	28	33	29	0.891
5934	NM_001323608,NM	**RBL2**	RB transcriptional corepr	protein_coding	32	27	33	28	0.841
5935	NM_006743,XM_02	**RBM3**	RNA binding motif protei	protein_coding	390	372	391	373	0.953
5936	NM_001198843,NM	**RBM4**	RNA binding motif protei	protein_coding	134	144	135	145	1.072
5937	NM_002897,NM_01	**RBMS1**	RNA binding motif single	protein_coding	54	49	55	50	0.916
5938	gene-RBMS1P1	**RBMS1P1**	RNA binding motif single	pseudogene	0	0	1	1	1.108
5939	NM_002898,XM_00	**RBMS2**	RNA binding motif single	protein_coding	0	0	1	1	1.027
5940	NM_001320944,NM	**RBMY1A1**	RNA binding motif protei	protein_coding	0	0	1	1	1.000
5947	NM_001130992,NM	**RBP1**	retinol binding protein 1	protein_coding	0	0	1	1	1.000
5948	NM_004164	**RBP2**	retinol binding protein 2	protein_coding	0	0	1	1	1.000
5949	NM_002900	**RBP3**	retinol binding protein 3	protein_coding	0	0	1	1	1.000
5950	NM_001323517,NM	**RBP4**	retinol binding protein 4	protein_coding	0	0	1	1	1.000
5954	NM_002901	**RCN1**	reticulocalbin 1	protein_coding	21	23	22	24	1.091
5955	NM_001271837,NM	**RCN2**	reticulocalbin 2	protein_coding	35	33	36	34	0.944
5956	NM_020061	**OPN1LW**	opsin 1, long wave sensi	protein_coding	0	0	1	1	1.000
5957	NM_002903	**RCVRN**	recoverin	protein_coding	0	0	1	1	1.000
5959	NM_001199771,NM	**RDH5**	retinol dehydrogenase 5	protein_coding	0	0	1	1	0.844
5961	NM_000322,XR_42	**PRPH2**	peripherin 2	protein_coding	0	0	1	1	1.000
5962	NM_001260492,NM	**RDX**	radixin	protein_coding	17	14	18	15	0.873
5964	gene-RDXP2	**RDXP2**	radixin pseudogene 2	pseudogene	0	0	1	1	1.000
5965	NM_002907,NM_03	**RECQL**	RecQ like helicase	protein_coding	61	59	62	60	0.972
5966	NM_001291746,NM	**REL**	REL proto-oncogene, NF	protein_coding	6	5	7	6	0.776
5967	NM_002909	**REG1A**	regenerating family mem	protein_coding	0	0	1	1	1.000
5968	NM_006507	**REG1B**	regenerating family mem	protein_coding	0	0	1	1	1.000
5969	NR_002714	**REG1CP**	regenerating family mem	transcribed_pseu	0	0	1	1	1.000
5970	NM_001145138,NM	**RELA**	RELA proto-oncogene, N	protein_coding	70	76	71	77	1.073
5971	NM_006509,XM_00	**RELB**	RELB proto-oncogene, N	protein_coding	8	8	9	9	1.006
5972	NM_000537	**REN**	renin	protein_coding	0	0	1	1	1.000
5973	NM_002910,XM_01	**RENBP**	renin binding protein	protein_coding	0	0	1	1	1.000
5976	NM_001297549,NM	**UPF1**	UPF1 RNA helicase and	protein_coding	133	129	134	130	0.975
5977	NM_001330308,NM	**DPF2**	double PHD fingers 2	protein_coding	67	89	68	90	1.323
5978	NM_001193508,NM	**REST**	RE1 silencing transcripti	protein_coding	24	23	25	24	0.962
5979	NM_001355216,NM	**RET**	ret proto-oncogene	protein_coding	0	0	1	1	1.018
5980	NM_001286431,NM	**REV3L**	REV3 like, DNA directed	protein_coding	12	11	13	12	0.874
5981	NM_001204747,NM	**RFC1**	replication factor C subu	protein_coding	104	90	105	91	0.866
5982	NM_001278791,NM	**RFC2**	replication factor C subu	protein_coding	198	211	199	212	1.064
5983	NM_002915,NM_18	**RFC3**	replication factor C subu	protein_coding	162	146	163	147	0.898
5984	NM_002916,NM_18	**RFC4**	replication factor C subu	protein_coding	150	134	151	135	0.889
5985	NM_001130112,NM	**RFC5**	replication factor C subu	protein_coding	42	51	43	52	1.199
5986	NM_002917,XM_01	**RFNG**	RFNG O-fucosylpeptide	protein_coding	12	12	13	13	0.983
5987	NM_006510	**TRIM27**	tripartite motif containing	protein_coding	65	63	66	64	0.975
5988	NM_021026,XM_01	**RFPL1**	ret finger protein like 1	protein_coding	0	0	1	1	1.000
5989	NM_002918,XM_01	**RFX1**	regulatory factor X1	protein_coding	8	8	9	9	1.038
5990	NM_000635,NM_13	**RFX2**	regulatory factor X2	protein_coding	0	0	1	1	0.996
5991	NM_001282116,NM	**RFX3**	regulatory factor X3	protein_coding	4	4	5	5	1.025
5992	NM_001206691,NM	**RFX4**	regulatory factor X4	protein_coding	0	0	1	1	1.000
5993	NM_000449,NM_00	**RFX5**	regulatory factor X5	protein_coding	59	55	60	56	0.921
5994	NM_000538	**RFXAP**	regulatory factor X assoc	protein_coding	35	33	36	34	0.933
5995	NM_001012720,NM	**RGR**	retinal G protein coupled	protein_coding	0	0	1	1	1.000
5996	NM_002922	**RGS1**	regulator of G protein sig	protein_coding	11	7	12	8	0.681
5997	NM_002923	**RGS2**	regulator of G protein sig	protein_coding	6	6	7	7	0.936
5998	NM_001276260,NM	**RGS3**	regulator of G protein sig	protein_coding	33	38	34	39	1.131
5999	NM_001102445,NM	**RGS4**	regulator of G protein sig	protein_coding	7	4	8	5	0.667
6000	NM_001282773,NM	**RGS7**	regulator of G protein sig	protein_coding	0	0	1	1	1.000
6001	NM_001005339,NM	**RGS10**	regulator of G protein sig	protein_coding	0	0	1	1	1.000
6002	NM_002926,NM_19	**RGS12**	regulator of G protein sig	protein_coding	0	0	1	1	0.985
6003	NM_002927,NM_14	**RGS13**	regulator of G protein sig	protein_coding	1	1	2	2	0.718
6004	NM_002928,XM_02	**RGS16**	regulator of G protein sig	protein_coding	67	51	68	52	0.773
6005	NM_000324,XM_01	**RHAG**	Rh associated glycoprote	protein_coding	0	0	1	1	1.000
6006	NM_001330430,NM	**RHCE**	Rh blood group CcEe an	protein_coding	0	0	1	1	1.118
6007	NM_001127691,NM	**RHD**	Rh blood group D antige	protein_coding	0	0	1	1	1.000
6008	gene-RHEBP1	**RHEBP1**	RHEB pseudogene 1	pseudogene	1	1	2	2	1.043
6009	NM_005614,XM_01	**RHEB**	Ras homolog, mTORC1	protein_coding	70	66	71	67	0.951
6010	NM_000539	**RHO**	rhodopsin	protein_coding	0	0	1	1	1.000
6011	NM_002929,XM_01	**GRK1**	G protein-coupled recept	protein_coding	0	0	1	1	1.000
6013	NM_006911,XM_01	**RLN1**	relaxin 1	protein_coding	3	3	4	4	1.005
6014	NM_001272077,NM	**RIT2**	Ras like without CAAX 2	protein_coding	0	0	1	1	1.000
6015	NM_002931	**RING1**	ring finger protein 1	protein_coding	66	78	67	79	1.183
6016	NM_001256820,NM	**RIT1**	Ras like without CAAX 1	protein_coding	9	9	10	10	0.972
6017	NM_000326,XM_01	**RLBP1**	retinaldehyde binding pr	protein_coding	0	0	1	1	1.000
6018	NM_012421,XM_01	**RLF**	RLF zinc finger	protein_coding	20	17	21	18	0.893
6019	NM_001329191,NM	**RLN2**	relaxin 2	protein_coding	0	1	1	2	1.300
6023	NR_003051	**RMRP**	RNA component of mitoc	RNase_MRP_RN/	3	3	4	4	0.954
6029	NR_002715	**RN7SL1**	RNA component of signa	scRNA	25	12	26	13	0.487
6030	gene-RN7SL4P	**RN7SL4P**	RNA, 7SL, cytoplasmic 4	pseudogene	4	2	5	3	0.658
6035	NM_002933,NM_19	**RNASE1**	ribonuclease A family me	protein_coding	0	0	1	1	1.000
6036	NM_002934	**RNASE2**	ribonuclease A family me	protein_coding	0	0	1	1	1.000
6037	NM_002935	**RNASE3**	ribonuclease A family me	protein_coding	0	0	1	1	1.000
6038	NM_001282192,NM	**RNASE4**	ribonuclease A family me	protein_coding	0	0	1	1	0.956
6039	NM_005615,XM_01	**RNASE6**	ribonuclease A family me	protein_coding	0	0	1	1	1.004
6041	NM_021133,XR_00	**RNASEL**	ribonuclease L	protein_coding	8	8	9	9	0.991
6043	NR_002586	**SNORA63**	small nucleolar RNA, H/A	snoRNA	27	23	28	24	0.847
6044	NR_002324	**SNORA62**	small nucleolar RNA, H/A	snoRNA	13	9	14	10	0.758
6045	NM_007212,XM_00	**RNF2**	ring finger protein 2	protein_coding	29	25	30	26	0.859
6046	NM_001113182,NM	**BRD2**	bromodomain containing	protein_coding	191	186	192	187	0.977
6047	NM_001185009,NM	**RNF4**	ring finger protein 4	protein_coding	133	129	134	130	0.974
6048	NM_006913	**RNF5**	ring finger protein 5	protein_coding	115	127	116	128	1.100
6049	NM_005977,NM_18	**RNF6**	ring finger protein 6	protein_coding	79	66	80	67	0.841
6050	NM_002939,NM_20	**RNH1**	ribonuclease/angiogenin	protein_coding	124	147	125	148	1.182
6051	NM_001319182,NM	**RNPEP**	arginyl aminopeptidase	protein_coding	4	5	5	6	1.163
6059	NM_001040876,NM	**ABCE1**	ATP binding cassette sul	protein_coding	188	160	189	161	0.851
6060	NR_004421	**RNU1-4**	RNA, U1 small nuclear 4	snRNA	1	1	2	2	1.252
6063	NR_145576	**RNVU1-2A**	RNA, variant U1 small nu	snRNA	0	0	1	1	1.000
6066	NR_002716	**RNU2-1**	RNA, U2 small nuclear 1	snRNA	0	0	1	1	0.996
6074	gene-RNU7-24P	**RNU7-24P**	RNA, U7 small nuclear 2	pseudogene	0	0	1	1	1.000
6076	gene-SNORD13P1	**SNORD13P1**	small nucleolar RNA, C/I	pseudogene	0	0	1	1	1.000
6078	gene-SNORD13P3	**SNORD13P3**	small nucleolar RNA, C/I	pseudogene	0	0	1	1	1.000
6079	NR_000005	**SNORD15A**	small nucleolar RNA, C/I	snoRNA	3	2	4	3	0.775
6080	NR_002907	**SNORA73A**	small nucleolar RNA, H/A	snoRNA	6	8	7	9	1.272
6082	NR_002908	**SNORD20**	small nucleolar RNA, C/I	snoRNA	1	1	2	2	1.105
6083	NR_000006	**SNORD21**	small nucleolar RNA, C/I	snoRNA	4	3	5	4	0.700
6084	NR_004391	**RNY1**	RNA, Ro60-associated Y	Y_RNA	0	0	1	1	0.748
6085	NR_004392	**RNY3**	RNA, Ro60-associated Y	Y_RNA	0	0	1	1	1.000
6086	NR_004393	**RNY4**	RNA, Ro60-associated Y	Y_RNA	0	0	1	1	1.000
6087	gene-RNY4P1	**RNY4P1**	RNY4 pseudogene 1	pseudogene	0	0	1	1	1.000
6088	gene-RNY4P2	**RNY4P2**	RNY4 pseudogene 2	pseudogene	0	0	1	1	1.000
6090	NR_001571	**RNY5**	RNA, Ro60-associated Y	Y_RNA	0	0	1	1	1.000
6091	NM_001145845,NM	**ROBO1**	roundabout guidance rec	protein_coding	4	3	5	4	0.747
6092	NM_001128929,NM	**ROBO2**	roundabout guidance rec	protein_coding	0	0	1	1	1.000
6093	NM_005406	**ROCK1**	Rho associated coiled-co	protein_coding	47	40	48	41	0.853
6094	NM_000327	**ROM1**	retinal outer segment me	protein_coding	7	8	8	9	1.231
6095	NM_002943,NM_13	**RORA**	RAR related orphan rece	protein_coding	16	15	17	16	0.944

6096	NM_001365023,NM	**RORB**	RAR related orphan rece	protein_coding	0	0	1	1	0.936
6097	NM_001001523,NM	**RORC**	RAR related orphan rece	protein_coding	0	0	1	1	1.000
6098	NM_001378891,NM	**ROS1**	ROS proto-oncogene 1,	protein_coding	0	0	1	1	1.000
6100	NM_203288,XM_01	**RP9**	RP9 pre-mRNA splicing f	protein_coding	14	15	15	16	1.063
6101	NM_001375654,NM	**RP1**	RP1 axonemal microtubu	protein_coding	0	0	1	1	1.000
6102	NM_006915	**RP2**	RP2 activator of ARL3 G	protein_coding	23	20	24	21	0.906
6103	NM_000328,NM_00	**RPGR**	retinitis pigmentosa GTP	protein_coding	8	6	9	7	0.811
6117	NM_001355120,NM	**RPA1**	replication protein A1	protein_coding	68	62	69	63	0.919
6118	NM_001286076,NM	**RPA2**	replication protein A2	protein_coding	278	276	279	277	0.990
6119	NM_002947	**RPA3**	replication protein A3	protein_coding	47	55	48	56	1.162
6120	NM_001278282,NM	**RPE**	ribulose-5-phosphate-3-e	protein_coding	98	77	99	78	0.780
6121	NM_000329,XM_01	**RPE65**	retinoid isomerohydrolas	protein_coding	0	0	1	1	1.000
6122	NM_000967,NM_00	**RPL3**	ribosomal protein L3	protein_coding	2,229	2,267	2,230	2,268	1.017
6123	NM_005061,XM_01	**RPL3L**	ribosomal protein L3 like	protein_coding	0	0	1	1	1.000
6124	NM_000968	**RPL4**	ribosomal protein L4	protein_coding	1,618	1,607	1,619	1,608	0.993
6125	NM_000969,NR_14	**RPL5**	ribosomal protein L5	protein_coding	1,939	1,880	1,940	1,881	0.970
6126	gene-RPL9P7	**RPL9P7**	ribosomal protein L9 pse	pseudogene	25	22	26	23	0.881
6128	NM_000970,NM_00	**RPL6**	ribosomal protein L6	protein_coding	1,671	1,539	1,672	1,540	0.921
6129	NM_000971,NM_00	**RPL7**	ribosomal protein L7	protein_coding	1,994	2,012	1,995	2,013	1.009
6130	NM_000972	**RPL7A**	ribosomal protein L7a	protein_coding	2,452	2,514	2,453	2,515	1.025
6132	NM_000973,NM_00	**RPL8**	ribosomal protein L8	protein_coding	1,652	1,779	1,653	1,780	1.077
6133	NM_000661,NM_00	**RPL9**	ribosomal protein L9	protein_coding	2,414	2,325	2,415	2,326	0.963
6134	NM_001256577,NM	**RPL10**	ribosomal protein L10	protein_coding	1,009	1,005	1,010	1,006	0.996
6135	NM_000975,NM_00	**RPL11**	ribosomal protein L11	protein_coding	2,021	2,061	2,022	2,062	1.020
6136	NM_000976	**RPL12**	ribosomal protein L12	protein_coding	2,931	3,018	2,932	3,019	1.030
6137	NM_000977,NM_00	**RPL13**	ribosomal protein L13	protein_coding	598	661	599	662	1.105
6138	NM_001253379,NM	**RPL15**	ribosomal protein L15	protein_coding	901	884	902	885	0.981
6139	NM_000985,NM_00	**RPL17**	ribosomal protein L17	protein_coding	3,244	3,386	3,245	3,387	1.044
6141	NM_000979,NM_00	**RPL18**	ribosomal protein L18	protein_coding	2,005	2,072	2,006	2,073	1.033
6142	NM_000980	**RPL18A**	ribosomal protein L18a	protein_coding	2,703	2,802	2,704	2,803	1.037
6143	NM_000981,NM_00	**RPL19**	ribosomal protein L19	protein_coding	3,432	3,592	3,433	3,593	1.047
6144	NM_000982	**RPL21**	ribosomal protein L21	protein_coding	3,514	3,421	3,515	3,422	0.973
6145	gene-RPL21P1	**RPL21P1**	ribosomal protein L21 ps	pseudogene	0	0	1	1	0.853
6146	NM_000983	**RPL22**	ribosomal protein L22	protein_coding	685	670	686	671	0.979
6147	NM_000984	**RPL23A**	ribosomal protein L23a	protein_coding	1,792	1,924	1,793	1,925	1.074
6148	gene-RPL23AP1	**RPL23AP1**	ribosomal protein L23a p	pseudogene	0	0	1	1	1.006
6150	NM_021134,XM_00	**MRPL23**	mitochondrial ribosomal	protein_coding	164	183	165	184	1.119
6152	NM_000986	**RPL24**	ribosomal protein L24	protein_coding	2,270	2,304	2,271	2,305	1.015
6154	NM_000987,NM_00	**RPL26**	ribosomal protein L26	protein_coding	1,862	1,901	1,863	1,902	1.021
6155	NM_000988,NM_00	**RPL27**	ribosomal protein L27	protein_coding	2,856	2,968	2,857	2,969	1.039
6156	NM_000989	**RPL30**	ribosomal protein L30	protein_coding	2,551	2,724	2,552	2,725	1.068
6157	NM_000990	**RPL27A**	ribosomal protein L27a	protein_coding	460	459	461	460	0.998
6158	NM_000991,NM_00	**RPL28**	ribosomal protein L28	protein_coding	387	417	388	418	1.079
6159	NM_000992,XM_02	**RPL29**	ribosomal protein L29	protein_coding	1,534	1,645	1,535	1,646	1.073
6160	NM_000993,NM_00	**RPL31**	ribosomal protein L31	protein_coding	756	751	757	752	0.994
6161	NM_000994,NM_00	**RPL32**	ribosomal protein L32	protein_coding	549	574	550	575	1.045
6163	gene-RPL32P1	**RPL32P1**	ribosomal protein L32 ps	pseudogene	0	0	1	1	1.000
6164	NM_000995,NM_00	**RPL34**	ribosomal protein L34	protein_coding	522	518	523	519	0.992
6165	NM_000996,NM_00	**RPL35A**	ribosomal protein L35a	protein_coding	719	734	720	735	1.021
6166	NM_001001	**RPL36AL**	ribosomal protein L36a li	protein_coding	338	332	339	333	0.984
6167	NM_000997,NR_15	**RPL37**	ribosomal protein L37	protein_coding	347	379	348	380	1.093
6168	NM_000998	**RPL37A**	ribosomal protein L37a	protein_coding	563	593	564	594	1.053
6169	NM_000999,NM_00	**RPL38**	ribosomal protein L38	protein_coding	573	656	574	657	1.144
6170	NM_001000	**RPL39**	ribosomal protein L39	protein_coding	2,413	2,476	2,414	2,477	1.026
6171	NM_001035267,NM	**RPL41**	ribosomal protein L41	protein_coding	3,568	3,797	3,569	3,798	1.064
6172	gene-RPL41P5	**RPL41P5**	ribosomal protein L41 ps	pseudogene	90	99	91	100	1.090
6173	NM_021029	**RPL36A**	ribosomal protein L36a	protein_coding	1,041	1,174	1,042	1,175	1.127
6174	gene-RPL7P1	**RPL7P1**	ribosomal protein L7 pse	pseudogene	14	14	15	15	0.991
6175	NM_001002,NM_05	**RPLP0**	ribosomal protein lateral	protein_coding	3,251	3,306	3,252	3,307	1.017
6176	NM_001003,NM_21	**RPLP1**	ribosomal protein lateral	protein_coding	1,940	2,220	1,941	2,221	1.144
6181	NM_001004	**RPLP2**	ribosomal protein lateral	protein_coding	2,882	3,038	2,883	3,039	1.054
6182	NM_002949	**MRPL12**	mitochondrial ribosomal	protein_coding	311	319	312	320	1.025
6183	NM_021107,NM_03	**MRPS12**	mitochondrial ribosomal	protein_coding	239	253	240	254	1.060
6184	NM_002950	**RPN1**	ribophorin I	protein_coding	331	336	332	337	1.016
6185	NM_001135771,NM	**RPN2**	ribophorin II	protein_coding	350	354	351	355	1.011
6187	NM_002952	**RPS2**	ribosomal protein S2	protein_coding	6,007	6,784	6,008	6,785	1.129
6188	NM_001005,NM_00	**RPS3**	ribosomal protein S3	protein_coding	1,429	1,425	1,430	1,426	0.997
6189	NM_001006,NM_00	**RPS3A**	ribosomal protein S3A	protein_coding	2,103	2,023	2,104	2,024	0.962
6191	NM_001007	**RPS4X**	ribosomal protein S4 X-li	protein_coding	1,262	1,320	1,263	1,321	1.046
6192	NM_001008	**RPS4Y1**	ribosomal protein S4 Y-li	protein_coding	621	662	622	663	1.066
6193	NM_001009	**RPS5**	ribosomal protein S5	protein_coding	1,743	1,882	1,744	1,883	1.079
6194	NM_001010	**RPS6**	ribosomal protein S6	protein_coding	2,038	2,057	2,039	2,058	1.010
6195	NM_001006665,NM	**RPS6KA1**	ribosomal protein S6 kina	protein_coding	118	114	119	115	0.973
6196	NM_001006932,NM	**RPS6KA2**	ribosomal protein S6 kina	protein_coding	0	0	1	1	1.000
6197	NM_004586,XM_00	**RPS6KA3**	ribosomal protein S6 kina	protein_coding	85	76	86	77	0.891
6198	NM_001272042,NM	**RPS6KB1**	ribosomal protein S6 kina	protein_coding	31	28	32	29	0.913
6199	NM_003952,XM_00	**RPS6KB2**	ribosomal protein S6 kina	protein_coding	83	84	84	85	1.015
6201	NM_001011	**RPS7**	ribosomal protein S7	protein_coding	1,551	1,508	1,552	1,509	0.973
6202	NM_001012	**RPS8**	ribosomal protein S8	protein_coding	3,005	3,025	3,006	3,026	1.007
6203	NM_001013,NM_00	**RPS9**	ribosomal protein S9	protein_coding	1,905	1,979	1,906	1,980	1.039
6204	NM_001014,NM_00	**RPS10**	ribosomal protein S10	protein_coding	2,231	2,273	2,232	2,274	1.019
6205	NM_001015	**RPS11**	ribosomal protein S11	protein_coding	3,141	3,428	3,142	3,429	1.091
6206	NM_001016	**RPS12**	ribosomal protein S12	protein_coding	1,981	2,090	1,982	2,091	1.055
6207	NM_001017	**RPS13**	ribosomal protein S13	protein_coding	1,752	1,728	1,753	1,729	0.987
6208	NM_001025070,NM	**RPS14**	ribosomal protein S14	protein_coding	845	886	846	887	1.049
6209	NM_001018,NM_00	**RPS15**	ribosomal protein S15	protein_coding	2,114	2,252	2,115	2,253	1.066
6210	NM_001019,NM_00	**RPS15A**	ribosomal protein S15a	protein_coding	596	599	597	600	1.005
6217	NM_001020,NM_00	**RPS16**	ribosomal protein S16	protein_coding	2,064	2,223	2,065	2,224	1.077
6218	NM_001021,NR_11	**RPS17**	ribosomal protein S17	protein_coding	4,361	4,541	4,362	4,542	1.041
6219	gene-RPS17P2	**RPS17P2**	ribosomal protein S17 ps	pseudogene	0	0	1	1	0.982
6222	NM_022551	**RPS18**	ribosomal protein S18	protein_coding	8,226	8,538	8,227	8,539	1.038
6223	NM_001022,NM_00	**RPS19**	ribosomal protein S19	protein_coding	699	702	700	703	1.003
6224	NM_001023,NM_00	**RPS20**	ribosomal protein S20	protein_coding	2,258	2,222	2,259	2,223	0.984
6225	gene-RPS20P3	**RPS20P3**	ribosomal protein S20 ps	pseudogene	0	0	1	1	0.890
6226	gene-RPS20P4	**RPS20P4**	ribosomal protein S20 ps	pseudogene	0	0	1	1	1.000
6227	NM_001024,XM_02	**RPS21**	ribosomal protein S21	protein_coding	1,782	1,985	1,783	1,986	1.114
6228	NM_001025	**RPS23**	ribosomal protein S23	protein_coding	572	597	573	598	1.042
6229	NM_001026,NM_00	**RPS24**	ribosomal protein S24	protein_coding	3,045	3,097	3,046	3,098	1.017
6230	NM_001028	**RPS25**	ribosomal protein S25	protein_coding	2,164	2,216	2,165	2,217	1.024
6231	NM_001029	**RPS26**	ribosomal protein S26	protein_coding	528	545	529	546	1.033
6232	NM_001030,NM_00	**RPS27**	ribosomal protein S27	protein_coding	3,726	3,876	3,727	3,877	1.040
6233	NM_001135592,NM	**RPS27A**	ribosomal protein S27a	protein_coding	1,104	1,098	1,105	1,099	0.995
6234	NM_001031	**RPS28**	ribosomal protein S28	protein_coding	255	320	256	321	1.253
6235	NM_001030001,NM	**RPS29**	ribosomal protein S29	protein_coding	486	620	487	621	1.275
6236	NM_001128850,NM	**RRAD**	RRAD, Ras related glyco	protein_coding	0	0	1	1	1.000
6237	NM_006270	**RRAS**	RAS related	protein_coding	1	2	2	3	1.076
6238	NM_001042576,NM	**RRBP1**	ribosome binding protein	protein_coding	17	18	18	19	1.017
6239	NM_001003698,NM	**RREB1**	ras responsive element b	protein_coding	0	0	1	1	1.000
6240	NM_001033,NM_00	**RRM1**	ribonucleotide reductase	protein_coding	217	199	218	200	0.916
6241	NM_001034,NM_00	**RRM2**	ribonucleotide reductase	protein_coding	503	530	504	531	1.053
6242	NM_001015055,NM	**RTKN**	rhotekin	protein_coding	0	0	1	1	1.013
6244	gene-RRM2P2	**RRM2P2**	ribonucleotide reductase	pseudogene	0	0	1	1	1.000
6247	NM_000330	**RS1**	retinoschisin 1	protein_coding	0	0	1	1	1.000
6248	NM_006511	**RSC1A1**	regulator of solute carrie	protein_coding	0	0	1	1	0.962
6249	NM_001247997,NM	**CLIP1**	CAP-Gly domain contain	protein_coding	15	14	16	15	0.939
6251	NM_012425,NM_15	**RSU1**	Ras suppressor protein 1	protein_coding	18	17	19	18	0.948
6252	NM_001363702,NM	**RTN1**	reticulon 1	protein_coding	0	0	1	1	1.000
6253	NM_005619,NM_20	**RTN2**	reticulon 2	protein_coding	2	1	3	2	0.757
6256	NM_001291920,NM	**RXRA**	retinoid X receptor alpha	protein_coding	3	3	4	4	1.008
6257	NM_001270401,NM	**RXRB**	retinoid X receptor beta	protein_coding	30	29	31	30	0.977
6258	NM_001256570,NM	**RXRG**	retinoid X receptor gamm	protein_coding	0	0	1	1	1.000
6259	NM_001005861,NM	**RYK**	receptor like tyrosine kin	protein_coding	8	8	9	9	0.989
6260	gene-RYKP1	**RYKP1**	RYK pseudogene 1	pseudogene	0	0	1	1	1.000
6261	NM_000540,NM_00	**RYR1**	ryanodine receptor 1	protein_coding	0	0	1	1	1.041
6262	NM_001035,XM_00	**RYR2**	ryanodine receptor 2	protein_coding	0	0	1	1	0.998
6263	NM_001036,NM_00	**RYR3**	ryanodine receptor 3	protein_coding	0	0	1	1	1.011

6271	NM_006271	**S100A1**	S100 calcium binding pr	protein_coding	0	0	1	1	1.000
6272	NM_001205228,NM	**SORT1**	sortilin 1	protein_coding	16	13	17	14	0.801
6273	NM_001366406,NM	**S100A2**	S100 calcium binding pr	protein_coding	7	9	8	10	1.246
6274	NM_002960	**S100A3**	S100 calcium binding pr	protein_coding	0	0	1	1	1.179
6275	NM_002961,NM_01	**S100A4**	S100 calcium binding pr	protein_coding	28	23	29	24	0.839
6276	NM_002962,XM_01	**S100A5**	S100 calcium binding pr	protein_coding	1	0	2	1	0.843
6277	NM_014624,XM_01	**S100A6**	S100 calcium binding pr	protein_coding	968	1,050	969	1,051	1.085
6278	NM_002963	**S100A7**	S100 calcium binding pr	protein_coding	0	0	1	1	1.000
6279	NM_001319196,NM	**S100A8**	S100 calcium binding pr	protein_coding	19	15	20	16	0.805
6280	NM_002965	**S100A9**	S100 calcium binding pr	protein_coding	393	388	394	389	0.986
6281	NM_002966	**S100A10**	S100 calcium binding pr	protein_coding	97	110	98	111	1.131
6282	NM_005620	**S100A11**	S100 calcium binding pr	protein_coding	144	148	145	149	1.032
6283	NM_005621	**S100A12**	S100 calcium binding pr	protein_coding	4	4	5	5	0.998
6284	NM_001024210,NM	**S100A13**	S100 calcium binding pr	protein_coding	9	9	10	10	0.984
6285	NM_006272,XM_01	**S100B**	S100 calcium binding pr	protein_coding	0	0	1	1	0.966
6286	NM_005980	**S100P**	S100 calcium binding pr	protein_coding	0	0	1	1	1.202
6288	NM_000331,NM_00	**SAA1**	serum amyloid A1	protein_coding	0	0	1	1	1.000
6289	NM_001127380,NM	**SAA2**	serum amyloid A2	protein_coding	0	0	1	1	0.839
6290	NR_026576	**SAA3P**	serum amyloid A3, pseu	transcribed_pseu	0	0	1	1	1.000
6291	NM_006512	**SAA4**	serum amyloid A4, const	protein_coding	0	0	1	1	1.000
6293	NM_001289174,NM	**VPS52**	VPS52 subunit of GARP	protein_coding	28	30	29	31	1.071
6294	NM_001201338,NM	**SAFB**	scaffold attachment facto	protein_coding	172	178	173	179	1.036
6295	NM_000541,XM_01	**SAG**	S-antigen visual arrestin	protein_coding	0	0	1	1	1.000
6296	NM_005622,NM_20	**ACSM3**	acyl-CoA synthetase me	protein_coding	5	4	6	5	0.851
6297	NM_001291446,NM	**SALL2**	spalt like transcription fa	protein_coding	0	0	1	1	1.000
6299	NM_001127892,NM	**SALL1**	spalt like transcription fa	protein_coding	0	0	1	1	1.000
6300	NM_001303252,NM	**MAPK12**	mitogen-activated protei	protein_coding	31	28	32	29	0.924
6301	NM_001330669,NM	**SARS1**	seryl-tRNA synthetase 1	protein_coding	217	208	218	209	0.955
6302	NM_001330168,NM	**TSPAN31**	tetraspanin 31	protein_coding	6	5	7	6	0.796
6303	NM_002970,NR_02	**SAT1**	spermidine/spermine N1	protein_coding	35	35	36	36	1.015
6304	NM_001131010,NM	**SATB1**	SATB homeobox 1	protein_coding	0	0	1	1	1.000
6305	NM_001365819,NM	**SBF1**	SET binding factor 1	protein_coding	64	59	65	60	0.928
6307	NM_001017369,NM	**MSMO1**	methylsterol monooxyge	protein_coding	99	81	100	82	0.820
6308	gene-SC4MOP	**SC4MOP**	sterol-C4-methyl oxidase	pseudogene	0	0	1	1	1.000
6309	NM_001024956,NM	**SC5D**	sterol-C5-desaturase	protein_coding	15	13	16	14	0.894
6310	NM_000332,NM_00	**ATXN1**	ataxin 1	protein_coding	15	15	16	16	0.951
6311	NM_001310121,NM	**ATXN2**	ataxin 2	protein_coding	11	10	12	11	0.990
6314	NM_000333,NM_00	**ATXN7**	ataxin 7	protein_coding	14	12	15	13	0.869
6315	NR_002717	**ATXN8OS**	ATXN8 opposite strand l	antisense_RNA	0	0	1	1	1.000
6317	NM_006919	**SERPINB3**	serpin family B member	protein_coding	0	0	1	1	1.000
6318	NM_002974,NM_17	**SERPINB4**	serpin family B member	protein_coding	0	0	1	1	1.000
6319	NM_005063	**SCD**	stearoyl-CoA desaturase	protein_coding	159	150	160	151	0.948
6320	NM_002975	**CLEC11A**	C-type lectin domain con	protein_coding	3	4	4	5	1.317
6322	NM_001037535,NM	**SCML1**	Scm polycomb group pro	protein_coding	0	0	1	1	1.000
6323	NM_001165963,NM	**SCN1A**	sodium voltage-gated ch	protein_coding	0	0	1	1	1.009
6324	NM_001037,NM_00	**SCN1B**	sodium voltage-gated ch	protein_coding	0	0	1	1	0.741
6326	NM_001040142,NM	**SCN2A**	sodium voltage-gated ch	protein_coding	1	0	2	1	0.822
6327	NM_004588	**SCN2B**	sodium voltage-gated ch	protein_coding	0	0	1	1	1.000
6328	NM_001081676,NM	**SCN3A**	sodium voltage-gated ch	protein_coding	6	5	7	6	0.908
6329	NM_000334	**SCN4A**	sodium voltage-gated ch	protein_coding	0	0	1	1	1.064
6330	NM_001142348,NM	**SCN4B**	sodium voltage-gated ch	protein_coding	0	0	1	1	0.938
6331	NM_000335,NM_00	**SCN5A**	sodium voltage-gated ch	protein_coding	0	0	1	1	0.988
6332	NM_002976,NR_04	**SCN7A**	sodium voltage-gated ch	protein_coding	0	0	1	1	1.000
6334	NM_001177984,NM	**SCN8A**	sodium voltage-gated ch	protein_coding	1	0	2	1	0.748
6335	NM_001365536,NM	**SCN9A**	sodium voltage-gated ch	protein_coding	0	0	1	1	1.000
6336	NM_001293306,NM	**SCN10A**	sodium voltage-gated ch	protein_coding	0	0	1	1	1.000
6337	NM_001038,NM_00	**SCNN1A**	sodium channel epithelia	protein_coding	0	0	1	1	1.000
6338	NM_000336,XM_01	**SCNN1B**	sodium channel epithelia	protein_coding	0	0	1	1	1.081
6339	NM_001130413,NR	**SCNN1D**	sodium channel epithelia	protein_coding	1	0	2	1	0.850
6340	NM_001039	**SCNN1G**	sodium channel epithelia	protein_coding	0	0	1	1	1.000
6341	NM_004589	**SCO1**	synthesis of cytochrome	protein_coding	10	10	11	11	0.949
6342	NM_001007098,NM	**SCP2**	sterol carrier protein 2	protein_coding	159	150	160	151	0.949
6343	NM_021920,XM_01	**SCT**	secretin	protein_coding	0	0	1	1	1.000
6344	NM_002980,XM_00	**SCTR**	secretin receptor	protein_coding	0	0	1	1	1.000
6345	NM_001098814,NM	**SRL**	sarcalumenin	protein_coding	0	0	1	1	1.000
6346	NM_002981	**CCL1**	C-C motif chemokine lig	protein_coding	0	0	1	1	1.000
6347	NM_002982	**CCL2**	C-C motif chemokine lig	protein_coding	0	0	1	1	1.000
6348	NM_002983,NR_16	**CCL3**	C-C motif chemokine lig	protein_coding	4,622	4,941	4,623	4,942	1.069
6351	NM_002984	**CCL4**	C-C motif chemokine lig	protein_coding	1,608	1,499	1,609	1,500	0.932
6352	NM_001278736,NM	**CCL5**	C-C motif chemokine lig	protein_coding	991	1,072	992	1,073	1.082
6354	NM_006273	**CCL7**	C-C motif chemokine lig	protein_coding	0	0	1	1	1.000
6355	NM_005623	**CCL8**	C-C motif chemokine lig	protein_coding	0	0	1	1	1.000
6356	NM_002986	**CCL11**	C-C motif chemokine lig	protein_coding	0	0	1	1	1.000
6357	NM_005408	**CCL13**	C-C motif chemokine lig	protein_coding	0	0	1	1	1.000
6358	NM_032962,NM_03	**CCL14**	C-C motif chemokine lig	protein_coding	0	0	1	1	1.000
6359	NM_032965	**CCL15**	C-C motif chemokine lig	protein_coding	0	0	1	1	1.000
6360	NM_004590,XM_00	**CCL16**	C-C motif chemokine lig	protein_coding	0	0	1	1	1.000
6361	NM_002987,XM_01	**CCL17**	C-C motif chemokine lig	protein_coding	0	0	1	1	1.000
6362	NM_002988	**CCL18**	C-C motif chemokine lig	protein_coding	59	67	60	68	1.142
6363	NM_006274	**CCL19**	C-C motif chemokine lig	protein_coding	0	0	1	1	1.000
6364	NM_001130046,NM	**CCL20**	C-C motif chemokine lig	protein_coding	0	0	1	1	1.000
6366	NM_002989	**CCL21**	C-C motif chemokine lig	protein_coding	0	0	1	1	1.000
6367	NM_002990	**CCL22**	C-C motif chemokine lig	protein_coding	0	0	1	1	1.000
6368	NM_005064,NM_14	**CCL23**	C-C motif chemokine lig	protein_coding	0	0	1	1	1.000
6369	NM_001371193,NM	**CCL24**	C-C motif chemokine lig	protein_coding	0	0	1	1	1.000
6370	NM_001201359,NM	**CCL25**	C-C motif chemokine lig	protein_coding	0	0	1	1	1.000
6372	NM_002993	**CXCL6**	C-X-C motif chemokine li	protein_coding	0	0	1	1	1.000
6373	NM_001302123,NM	**CXCL11**	C-X-C motif chemokine li	protein_coding	0	0	1	1	0.887
6374	NM_002994	**CXCL5**	C-X-C motif chemokine li	protein_coding	0	0	1	1	1.000
6375	NM_002995,XM_01	**XCL1**	X-C motif chemokine liga	protein_coding	41	54	42	55	1.313
6376	NM_001304392,NM	**CX3CL1**	C-X3-C motif chemokine	protein_coding	0	0	1	1	1.000
6382	NM_001006946,NM	**SDC1**	syndecan 1	protein_coding	0	0	1	1	1.000
6383	NM_002998,XM_01	**SDC2**	syndecan 2	protein_coding	0	0	1	1	1.000
6385	NM_002999,XM_01	**SDC4**	syndecan 4	protein_coding	11	10	12	11	0.947
6386	NM_001007067,NM	**SDCBP**	syndecan binding protei	protein_coding	0	0	1	1	1.000
6387	NM_000609,NM_00	**CXCL12**	C-X-C motif chemokine li	protein_coding	0	0	1	1	1.000
6388	NM_006923,NR_04	**SDF2**	stromal cell derived facto	protein_coding	78	82	79	83	1.057
6389	NM_001294332,NM	**SDHA**	succinate dehydrogenas	protein_coding	73	92	74	93	1.260
6390	NM_003000	**SDHB**	succinate dehydrogenas	protein_coding	338	344	339	345	1.018
6391	NM_001035511,NM	**SDHC**	succinate dehydrogenas	protein_coding	41	38	42	39	0.943
6392	NM_001276503,NM	**SDHD**	succinate dehydrogenas	protein_coding	277	274	278	275	0.989
6396	NM_001136026,NM	**SEC13**	SEC13 homolog, nuclea	protein_coding	178	181	179	182	1.013
6397	NM_001039573,NM	**SEC14L1**	SEC14 like lipid binding	protein_coding	34	31	35	32	0.908
6398	NM_003004,XM_00	**SECTM1**	secreted and transmemb	protein_coding	0	0	1	1	1.000
6399	NM_001011658,NM	**TRAPPC2**	trafficking protein particle	protein_coding	17	17	18	18	1.029
6400	NM_001244984,NM	**SEL1L**	SEL1L adaptor subunit c	protein_coding	39	35	40	36	0.909
6401	NM_000450	**SELE**	selectin E	protein_coding	0	0	1	1	1.000
6402	NM_000655,NR_02	**SELL**	selectin L	protein_coding	2	3	3	4	1.018
6403	NM_003005,XM_00	**SELP**	selectin P	protein_coding	0	0	1	1	1.000
6404	NM_001206609,NM	**SELPLG**	selectin P ligand	protein_coding	108	108	109	109	1.000
6405	NM_001318798,NM	**SEMA3F**	semaphorin 3F	protein_coding	0	0	1	1	1.000
6406	NM_003007	**SEMG1**	semenogelin 1	protein_coding	0	0	1	1	1.000
6407	NM_003008	**SEMG2**	semenogelin 2	protein_coding	0	0	1	1	1.000
6414	NM_001085486,NM	**SELENOP**	selenoprotein P	protein_coding	0	0	1	1	0.890
6415	NM_003009	**SELENOW**	selenoprotein W	protein_coding	191	182	192	183	0.950
6416	NM_001281435,NM	**MAP2K4**	mitogen-activated protei	protein_coding	19	18	20	19	0.924
6418	NM_001122821,NM	**SET**	SET nuclear proto-oncog	protein_coding	690	660	691	661	0.957
6419	NM_001243723,NM	**SETMAR**	SET domain and mariner	protein_coding	11	11	12	12	1.018
6421	NM_005066,NR_13	**SFPQ**	splicing factor proline an	protein_coding	347	320	348	321	0.923
6422	NM_003012	**SFRP1**	secreted frizzled related	protein_coding	0	0	1	1	1.000
6423	NM_003013	**SFRP2**	secreted frizzled related	protein_coding	0	0	1	1	1.000
6424	NM_003014	**SFRP4**	secreted frizzled related	protein_coding	0	0	1	1	1.000
6425	NM_003015	**SFRP5**	secreted frizzled related	protein_coding	0	0	1	1	1.000
6426	NM_001078166,NM	**SRSF1**	serine and arginine rich s	protein_coding	317	303	318	304	0.955
6427	NM_001195427,NM	**SRSF2**	serine and arginine rich s	protein_coding	537	582	538	583	1.084
6428	NM_003017,NR_03	**SRSF3**	serine and arginine rich s	protein_coding	239	246	240	247	1.029

6429	NM_005626,XM_01	**SRSF4**	serine and arginine rich s	protein_coding	164	161	165	162	0.981
6430	NM_001039465,NM	**SRSF5**	serine and arginine rich s	protein_coding	269	281	270	282	1.041
6431	NM_006275,NR_03	**SRSF6**	serine and arginine rich s	protein_coding	139	138	140	139	0.992
6432	NM_001031684,NM	**SRSF7**	serine and arginine rich s	protein_coding	336	379	337	380	1.126
6433	NM_001261411,NM	**SFSWAP**	splicing factor SWAP	protein_coding	40	39	41	40	0.983
6434	NM_001243879,NM	**TRA2B**	transformer 2 beta homo	protein_coding	308	325	309	326	1.054
6439	NM_000542,NM_00	**SFTPB**	surfactant protein B	protein_coding	0	0	1	1	1.000
6440	NM_001172357,NM	**SFTPC**	surfactant protein C	protein_coding	0	0	1	1	1.000
6441	NM_003019,XM_01	**SFTPD**	surfactant protein D	protein_coding	0	0	1	1	1.000
6442	NM_000023,NM_00	**SGCA**	sarcoglycan alpha	protein_coding	0	0	1	1	1.000
6443	NM_000232	**SGCB**	sarcoglycan beta	protein_coding	0	0	1	1	1.000
6444	NM_000337,NM_00	**SGCD**	sarcoglycan delta	protein_coding	0	0	1	1	1.000
6445	NM_000231,NM_00	**SGCG**	sarcoglycan gamma	protein_coding	0	0	1	1	1.000
6446	NM_001143676,NM	**SGK1**	serum/glucocorticoid reg	protein_coding	0	0	1	1	0.952
6447	NM_001144757,NM	**SCG5**	secretogranin V	protein_coding	0	2	1	3	2.093
6448	NM_000199,NM_00	**SGSH**	N-sulfoglucosamine sulfo	protein_coding	3	3	4	4	0.807
6449	NM_003021,XM_01	**SGTA**	small glutamine rich tetra	protein_coding	179	179	180	180	1.001
6450	NM_001001713,NM	**SH3BGR**	SH3 domain binding glut	protein_coding	4	6	5	7	1.255
6451	NM_003022,XM_01	**SH3BGRL**	SH3 domain binding glut	protein_coding	60	57	61	58	0.949
6452	NM_001122681,NM	**SH3BP2**	SH3 domain binding prot	protein_coding	34	32	35	33	0.928
6453	NM_001001132,NM	**ITSN1**	intersectin 1	protein_coding	3	3	4	4	0.903
6455	NM_001199943,NM	**SH3GL1**	SH3 domain containing G	protein_coding	47	51	48	52	1.074
6456	NM_003026,XM_01	**SH3GL2**	SH3 domain containing G	protein_coding	0	0	1	1	1.000
6457	NM_001301108,NM	**SH3GL3**	SH3 domain containing G	protein_coding	0	0	1	1	1.000
6458	NR_033412	**SH3GL1P1**	SH3 domain containing G	transcribed_pseu	0	0	1	1	0.933
6459	NR_033420	**SH3GL1P2**	SH3 domain containing G	transcribed_pseu	0	0	1	1	0.963
6461	NM_003028	**SHB**	SH2 domain containing a	protein_coding	0	0	1	1	1.000
6462	NM_001040,NM_00	**SHBG**	sex hormone binding glo	protein_coding	0	0	1	1	1.000
6464	NM_001130040,NM	**SHC1**	SHC adaptor protein 1	protein_coding	41	40	42	41	0.984
6465	gene-SHC1P1	**SHC1P1**	SHC adaptor protein 1 ps	pseudogene	0	0	1	1	1.000
6466	gene-SHC1P2	**SHC1P2**	SHC adaptor protein 1 ps	pseudogene	0	0	1	1	1.000
6468	NM_001323541,NM	**FBXW4**	F-box and WD repeat do	protein_coding	13	15	14	16	1.144
6469	NM_000193,NM_00	**SHH**	sonic hedgehog signalin	protein_coding	0	0	1	1	1.000
6470	NM_001281786,NM	**SHMT1**	serine hydroxymethyltran	protein_coding	33	35	34	36	1.059
6471	gene-SHMT1P1	**SHMT1P1**	serine hydroxymethyltran	pseudogene	0	0	1	1	1.000
6472	NM_001166356,NM	**SHMT2**	serine hydroxymethyltran	protein_coding	285	268	286	269	0.939
6473	NM_000451,NM_00	**SHOX**	short stature homeobox	protein_coding	0	0	1	1	1.000
6474	NM_001163678,NM	**SHOX2**	short stature homeobox 2	protein_coding	0	0	1	1	1.000
6476	NM_001041,XM_01	**SI**	sucrase-isomaltase	protein_coding	0	0	1	1	1.000
6477	NM_001006610,NM	**SIAH1**	siah E3 ubiquitin protein	protein_coding	11	13	12	14	1.140
6478	NM_005067	**SIAH2**	siah E3 ubiquitin protein	protein_coding	39	39	40	40	0.982
6480	NM_001353916,NM	**ST6GAL1**	ST6 beta-galactoside alp	protein_coding	8	7	9	8	0.874
6482	NM_003033,NM_17	**ST3GAL1**	ST3 beta-galactoside alp	protein_coding	33	29	34	30	0.902
6483	NM_006927	**ST3GAL2**	ST3 beta-galactoside alp	protein_coding	11	10	12	11	0.891
6484	NM_001254757,NM	**ST3GAL4**	ST3 beta-galactoside alp	protein_coding	7	9	8	10	1.312
6487	NM_001270459,NM	**ST3GAL3**	ST3 beta-galactoside alp	protein_coding	3	2	4	3	0.899
6489	NM_001304450,NM	**ST8SIA1**	ST8 alpha-N-acetyl-neur	protein_coding	1	1	2	2	1.049
6490	NM_001200053,NM	**PMEL**	premelanosome protein	protein_coding	0	0	1	1	0.883
6491	NM_001048166,NM	**STIL**	STIL centriolar assembly	protein_coding	62	57	63	58	0.922
6492	NM_001374769,NM	**SIM1**	SIM bHLH transcription f	protein_coding	0	0	1	1	1.000
6493	NM_005069,NM_00	**SIM2**	SIM bHLH transcription f	protein_coding	0	0	1	1	1.000
6494	NM_006747,NM_15	**SIPA1**	signal-induced proliferati	protein_coding	56	57	57	58	1.027
6495	NM_005982,XM_01	**SIX1**	SIX homeobox 1	protein_coding	0	0	1	1	1.000
6496	NM_005413	**SIX3**	SIX homeobox 3	protein_coding	0	0	1	1	1.000
6497	NM_003036,XM_00	**SKI**	SKI proto-oncogene	protein_coding	36	24	37	25	0.685
6498	NM_001145097,NM	**SKIL**	SKI like proto-oncogene	protein_coding	13	8	14	9	0.656
6499	NM_006929,XM_01	**SKIV2L**	Ski2 like RNA helicase	protein_coding	25	25	26	26	1.014
6500	NM_006930,NM_17	**SKP1**	S-phase kinase associate	protein_coding	82	94	83	95	1.140
6502	NM_001243120,NM	**SKP2**	S-phase kinase associate	protein_coding	61	54	62	55	0.878
6503	NM_001045556,NM	**SLA**	Src like adaptor	protein_coding	35	33	36	34	0.959
6504	NM_001330754,NM	**SLAMF1**	signaling lymphocytic ac	protein_coding	14	13	15	14	0.988
6505	NM_004170,XM_01	**SLC1A1**	solute carrier family 1 me	protein_coding	0	0	1	1	0.979
6506	NM_001195728,NM	**SLC1A2**	solute carrier family 1 me	protein_coding	0	0	1	1	1.000
6507	NM_001166695,NM	**SLC1A3**	solute carrier family 1 me	protein_coding	0	0	1	1	1.000
6508	NM_001326559,NM	**SLC4A3**	solute carrier family 4 me	protein_coding	0	0	1	1	1.000
6509	NM_001193493,NM	**SLC1A4**	solute carrier family 1 me	protein_coding	38	35	39	36	0.924
6510	NM_001145144,NM	**SLC1A5**	solute carrier family 1 me	protein_coding	238	230	239	231	0.966
6511	NM_001272087,NM	**SLC1A6**	solute carrier family 1 me	protein_coding	0	0	1	1	1.000
6512	NM_001287595,NM	**SLC1A7**	solute carrier family 1 me	protein_coding	0	0	1	1	1.000
6513	NM_006516	**SLC2A1**	solute carrier family 2 me	protein_coding	69	76	70	77	1.101
6514	NM_000340,NM_00	**SLC2A2**	solute carrier family 2 me	protein_coding	0	0	1	1	1.000
6515	NM_006931	**SLC2A3**	solute carrier family 2 me	protein_coding	65	63	66	64	0.977
6517	NM_001042	**SLC2A4**	solute carrier family 2 me	protein_coding	0	0	1	1	1.000
6518	NM_001135585,NM	**SLC2A5**	solute carrier family 2 me	protein_coding	0	0	1	1	1.000
6519	NM_000341,XM_01	**SLC3A1**	solute carrier family 3 me	protein_coding	0	0	1	1	0.952
6520	NM_001012662,NM	**SLC3A2**	solute carrier family 3 me	protein_coding	212	212	213	213	1.000
6521	NM_000342,XM_00	**SLC4A1**	solute carrier family 4 me	protein_coding	0	0	1	1	1.000
6522	NM_001199692,NM	**SLC4A2**	solute carrier family 4 me	protein_coding	34	30	35	31	0.895
6523	NM_000343,NM_00	**SLC5A1**	solute carrier family 5 me	protein_coding	0	0	1	1	0.927
6524	NM_003041,NR_13	**SLC5A2**	solute carrier family 5 me	protein_coding	0	0	1	1	1.000
6525	NM_001207017,NM	**SMTN**	smoothelin	protein_coding	18	19	19	20	1.061
6526	NM_006933	**SLC5A3**	solute carrier family 5 me	protein_coding	23	19	24	20	0.802
6527	NM_014227,XM_00	**SLC5A4**	solute carrier family 5 me	protein_coding	0	0	1	1	1.000
6528	NM_000453,XM_01	**SLC5A5**	solute carrier family 5 me	protein_coding	0	0	1	1	1.000
6529	NM_001348250,NM	**SLC6A1**	solute carrier family 6 me	protein_coding	0	0	1	1	1.000
6530	NM_001043,NM_00	**SLC6A2**	solute carrier family 6 me	protein_coding	0	0	1	1	1.000
6531	NM_001044	**SLC6A3**	solute carrier family 6 me	protein_coding	0	0	1	1	1.000
6532	NM_001045	**SLC6A4**	solute carrier family 6 me	protein_coding	0	0	1	1	1.016
6533	NM_001134367,NM	**SLC6A6**	solute carrier family 6 me	protein_coding	43	26	44	27	0.620
6534	NM_014228,XM_01	**SLC6A7**	solute carrier family 6 me	protein_coding	0	0	1	1	1.000
6535	NM_001142805,NM	**SLC6A8**	solute carrier family 6 me	protein_coding	0	0	1	1	1.000
6536	NM_001024845,NM	**SLC6A9**	solute carrier family 6 me	protein_coding	0	0	1	1	1.000
6538	NM_001317406,NM	**SLC6A11**	solute carrier family 6 me	protein_coding	0	0	1	1	1.000
6539	NM_001122847,NM	**SLC6A12**	solute carrier family 6 me	protein_coding	0	0	1	1	1.000
6540	NM_001190997,NM	**SLC6A13**	solute carrier family 6 me	protein_coding	0	0	1	1	1.000
6541	NM_003045,XM_00	**SLC7A1**	solute carrier family 7 me	protein_coding	114	105	115	106	0.917
6542	NM_001008539,NM	**SLC7A2**	solute carrier family 7 me	protein_coding	0	0	1	1	1.000
6543	NM_015063,XM_00	**SLC8A2**	solute carrier family 8 me	protein_coding	0	0	1	1	1.000
6545	NM_004173	**SLC7A4**	solute carrier family 7 me	protein_coding	0	0	1	1	1.000
6546	NM_001112800,NM	**SLC8A1**	solute carrier family 8 me	protein_coding	0	0	1	1	1.000
6547	NM_001130417,NM	**SLC8A3**	solute carrier family 8 me	protein_coding	0	0	1	1	1.000
6548	NM_003047,NR_04	**SLC9A1**	solute carrier family 9 me	protein_coding	22	21	23	22	0.977
6549	NM_003048	**SLC9A2**	solute carrier family 9 me	protein_coding	0	0	1	1	1.000
6550	NM_001284351,NM	**SLC9A3**	solute carrier family 9 me	protein_coding	6	6	7	7	0.979
6551	gene-SLC9A3P1	**SLC9A3P1**	solute carrier family 9 me	pseudogene	0	0	1	1	1.000
6553	NM_001323971,NM	**SLC9A5**	solute carrier family 9 me	protein_coding	0	0	1	1	0.975
6554	NM_003049	**SLC10A1**	solute carrier family 10 m	protein_coding	0	0	1	1	1.000
6555	NM_000452	**SLC10A2**	solute carrier family 10 m	protein_coding	0	0	1	1	1.000
6556	NM_000578,XM_00	**SLC11A1**	solute carrier family 11 m	protein_coding	0	0	1	1	1.046
6557	NM_000338,NM_00	**SLC12A1**	solute carrier family 12 m	protein_coding	0	0	1	1	1.000
6558	NM_001046,NM_00	**SLC12A2**	solute carrier family 12 m	protein_coding	7	6	8	7	0.877
6559	NM_000339,NM_00	**SLC12A3**	solute carrier family 12 m	protein_coding	0	0	1	1	1.000
6560	NM_001145961,NM	**SLC12A4**	solute carrier family 12 m	protein_coding	1	0	2	1	0.856
6561	NM_001324400,NM	**SLC13A1**	solute carrier family 13 m	protein_coding	0	0	1	1	1.000
6563	NM_001128588,NM	**SLC14A1**	solute carrier family 14 m	protein_coding	0	0	1	1	1.077
6564	NM_005073	**SLC15A1**	solute carrier family 15 m	protein_coding	0	0	1	1	1.000
6565	NM_001145998,NM	**SLC15A2**	solute carrier family 15 m	protein_coding	0	1	1	2	1.155
6566	NM_001166496,NM	**SLC16A1**	solute carrier family 16 m	protein_coding	167	155	168	156	0.931
6567	NM_006517	**SLC16A2**	solute carrier family 16 m	protein_coding	0	0	1	1	1.000
6568	NM_005074,XM_01	**SLC17A1**	solute carrier family 17 m	protein_coding	0	0	1	1	1.000
6569	NM_001167579,NM	**SLC34A1**	solute carrier family 34 m	protein_coding	0	0	1	1	1.051
6570	NM_001135691,NM	**SLC18A1**	solute carrier family 18 m	protein_coding	0	0	1	1	1.000
6571	NM_003054	**SLC18A2**	solute carrier family 18 m	protein_coding	0	0	1	1	1.000
6572	NM_003055	**SLC18A3**	solute carrier family 18 m	protein_coding	0	0	1	1	1.000
6573	NM_001205206,NM	**SLC19A1**	solute carrier family 19 m	protein_coding	35	37	36	38	1.045
6574	NM_005415,XM_01	**SLC20A1**	solute carrier family 20 m	protein_coding	176	180	177	181	1.023

6575	NM_001257180,NM	**SLC20A2**	solute carrier family 20 m	protein_coding	23	23	24	24	0.994
6576	NM_001256534,NM	**SLC25A1**	solute carrier family 25 m	protein_coding	15	15	16	16	0.987
6578	NM_005630,XM_01	**SLCO2A1**	solute carrier organic ani	protein_coding	0	0	1	1	1.000
6579	NM_021094,NM_13	**SLCO1A2**	solute carrier organic ani	protein_coding	0	0	1	1	1.000
6580	NM_003057,NM_15	**SLC22A1**	solute carrier family 22 m	protein_coding	1	0	2	1	0.918
6581	NM_021977,XM_00	**SLC22A3**	solute carrier family 22 m	protein_coding	0	0	1	1	1.000
6582	NM_003058	**SLC22A2**	solute carrier family 22 m	protein_coding	0	0	1	1	1.000
6583	NM_003059,XM_00	**SLC22A4**	solute carrier family 22 m	protein_coding	1	0	2	1	0.891
6584	NM_001308122,NM	**SLC22A5**	solute carrier family 22 m	protein_coding	3	3	4	4	0.959
6585	NM_003061	**SLIT1**	slit guidance ligand 1	protein_coding	0	0	1	1	0.976
6586	NM_001271946,NM	**SLIT3**	slit guidance ligand 3	protein_coding	0	0	1	1	1.000
6588	NM_003063	**SLN**	sarcolipin	protein_coding	0	0	1	1	1.192
6590	NM_003064	**SLPI**	secretory leukocyte pept	protein_coding	2	1	3	2	0.662
6591	NM_003068	**SNAI2**	snail family transcription	protein_coding	0	0	1	1	1.000
6594	NM_001282874,NM	**SMARCA1**	SWI/SNF related, matrix	protein_coding	0	0	1	1	1.000
6595	NM_001289396,NM	**SMARCA2**	SWI/SNF related, matrix	protein_coding	33	33	34	34	0.974
6596	NM_001318934,NM	**HLTF**	helicase like transcription	protein_coding	65	46	66	47	0.719
6597	NM_001128844,NM	**SMARCA4**	SWI/SNF related, matrix	protein_coding	146	151	147	152	1.032
6598	NM_001007468,NM	**SMARCB1**	SWI/SNF related, matrix	protein_coding	21	23	22	24	1.071
6599	NM_003074	**SMARCC1**	SWI/SNF related, matrix	protein_coding	76	69	77	70	0.914
6601	NM_001130420,NM	**SMARCC2**	SWI/SNF related, matrix	protein_coding	58	61	59	62	1.041
6602	NM_003076,NM_13	**SMARCD1**	SWI/SNF related, matrix	protein_coding	72	70	73	71	0.977
6603	NM_001098426,NM	**SMARCD2**	SWI/SNF related, matrix	protein_coding	97	98	98	99	1.002
6604	NM_001003801,NM	**SMARCD3**	SWI/SNF related, matrix	protein_coding	0	0	1	1	1.000
6605	NM_003079	**SMARCE1**	SWI/SNF related, matrix	protein_coding	67	66	68	67	0.971
6606	NM_000344,NM_00	**SMN1**	survival of motor neuron	protein_coding	171	158	172	159	0.922
6607	NM_017411,NM_02	**SMN2**	survival of motor neuron	protein_coding	184	170	185	171	0.921
6608	NM_005631,XM_02	**SMO**	smoothened, frizzled cla	protein_coding	0	0	1	1	1.000
6609	NM_000543,NM_00	**SMPD1**	sphingomyelin phosphod	protein_coding	23	22	24	23	0.972
6610	NM_003080,XM_00	**SMPD2**	sphingomyelin phosphod	protein_coding	9	8	10	9	0.906
6611	NM_001258423,NM	**SMS**	spermine synthase	protein_coding	153	140	154	141	0.917
6612	NM_001286416,NM	**SUMO3**	small ubiquitin like modif	protein_coding	133	139	134	140	1.043
6613	NM_001005849,NM	**SUMO2**	small ubiquitin like modif	protein_coding	215	207	216	208	0.962
6614	NM_001367089,NM	**SIGLEC1**	sialic acid binding Ig like	protein_coding	0	0	1	1	1.000
6615	NM_005985	**SNAI1**	snail family transcription	protein_coding	0	0	1	1	1.000
6616	NM_001322902,NM	**SNAP25**	synaptosome associated	protein_coding	3	2	4	3	0.663
6617	NM_003082	**SNAPC1**	small nuclear RNA activa	protein_coding	12	12	13	13	0.933
6618	NM_003083,NR_03	**SNAPC2**	small nuclear RNA activa	protein_coding	19	21	20	22	1.135
6619	NM_001039697,NM	**SNAPC3**	small nuclear RNA activa	protein_coding	19	16	20	17	0.871
6620	NM_001001502,NM	**SNCB**	synuclein beta	protein_coding	0	0	1	1	1.000
6621	NM_003086,XM_00	**SNAPC4**	small nuclear RNA activa	protein_coding	9	8	10	9	0.950
6622	NM_000345,NM_00	**SNCA**	synuclein alpha	protein_coding	0	0	1	1	1.000
6623	NM_001330120,NM	**SNCG**	synuclein gamma	protein_coding	0	0	1	1	1.000
6624	NM_003088	**FSCN1**	fascin actin-bundling pro	protein_coding	0	0	1	1	1.000
6625	NM_001301069,NM	**SNRNP70**	small nuclear ribonucleo	protein_coding	267	291	268	292	1.091
6626	NM_004596	**SNRPA**	small nuclear ribonucleo	protein_coding	238	240	239	241	1.007
6627	NM_003090,NR_13	**SNRPA1**	small nuclear ribonucleo	protein_coding	679	674	680	675	0.993
6628	NM_003091,NM_19	**SNRPB**	small nuclear ribonucleo	protein_coding	686	710	687	711	1.036
6629	NM_003092,NM_19	**SNRPB2**	small nuclear ribonucleo	protein_coding	154	147	155	148	0.960
6631	NM_003093,NR_02	**SNRPC**	small nuclear ribonucleo	protein_coding	519	535	520	536	1.031
6632	NM_001291916,NM	**SNRPD1**	small nuclear ribonucleo	protein_coding	92	95	93	96	1.031
6633	NM_001369751,NM	**SNRPD2**	small nuclear ribonucleo	protein_coding	665	765	666	766	1.150
6634	NM_001278656,NM	**SNRPD3**	small nuclear ribonucleo	protein_coding	127	130	128	131	1.019
6635	NM_001304464,NM	**SNRPE**	small nuclear ribonucleo	protein_coding	121	129	122	130	1.069
6636	NM_003095	**SNRPF**	small nuclear ribonucleo	protein_coding	201	201	202	202	1.001
6637	NM_001317165,NM	**SNRPG**	small nuclear ribonucleo	protein_coding	845	830	846	831	0.983
6638	NM_001349454,NM	**SNRPN**	small nuclear ribonucleo	protein_coding	24	28	25	29	1.124
6640	NM_003098,XM_00	**SNTA1**	syntrophin alpha 1	protein_coding	0	0	1	1	1.000
6641	NM_021021,XM_01	**SNTB1**	syntrophin beta 1	protein_coding	27	26	28	27	0.965
6642	NM_001242933,NM	**SNX1**	sorting nexin 1	protein_coding	25	22	26	23	0.904
6643	NM_001278199,NM	**SNX2**	sorting nexin 2	protein_coding	6	7	7	8	1.089
6644	gene-SRIP1	**SRIP1**	sorcin pseudogene 1	pseudogene	0	0	1	1	1.000
6645	NM_006750	**SNTB2**	syntrophin beta 2	protein_coding	4	3	5	4	0.925
6646	NM_001252511,NM	**SOAT1**	sterol O-acyltransferase	protein_coding	39	36	40	37	0.923
6647	NM_000454	**SOD1**	superoxide dismutase 1	protein_coding	497	524	498	525	1.054
6648	NM_000636,NM_00	**SOD2**	superoxide dismutase 2	protein_coding	30	27	31	28	0.879
6649	NM_003102,XR_42	**SOD3**	superoxide dismutase 3	protein_coding	0	0	1	1	1.000
6650	NM_005632,XM_01	**CAPN15**	calpain 15	protein_coding	50	51	51	52	1.028
6651	NM_001291411,NM	**SON**	SON DNA and RNA bind	protein_coding	150	127	151	128	0.849
6652	NM_003104,NR_03	**SORD**	sorbitol dehydrogenase	protein_coding	11	11	12	12	0.981
6653	NM_003105	**SORL1**	sortilin related receptor 1	protein_coding	1	1	2	2	0.890
6654	NM_001382394,NM	**SOS1**	SOS Ras/Rac guanine n	protein_coding	18	18	19	19	0.970
6655	NM_006939	**SOS2**	SOS Ras/Rho guanine n	protein_coding	9	8	10	9	0.906
6656	NM_005986	**SOX1**	SRY-box transcription fa	protein_coding	0	0	1	1	1.000
6657	NM_003106	**SOX2**	SRY-box transcription fa	protein_coding	0	0	1	1	0.912
6658	NM_005634	**SOX3**	SRY-box transcription fa	protein_coding	0	0	1	1	1.000
6659	NM_003107	**SOX4**	SRY-box transcription fa	protein_coding	0	0	1	1	1.000
6660	NM_001261414,NM	**SOX5**	SRY-box transcription fa	protein_coding	0	0	1	1	1.000
6661	gene-SOX5P1	**SOX5P1**	SOX5 pseudogene 1	pseudogene	0	0	1	1	1.000
6662	NM_000346	**SOX9**	SRY-box transcription fa	protein_coding	0	0	1	1	1.000
6663	NM_006941	**SOX10**	SRY-box transcription fa	protein_coding	0	0	1	1	1.000
6664	NM_003108	**SOX11**	SRY-box transcription fa	protein_coding	0	0	1	1	1.000
6665	NM_006942	**SOX15**	SRY-box transcription fa	protein_coding	0	0	1	1	0.958
6666	NM_006943	**SOX12**	SRY-box transcription fa	protein_coding	0	0	1	1	0.943
6667	NM_001251825,NM	**SP1**	Sp1 transcription factor	protein_coding	34	28	35	29	0.831
6668	NM_003110,XM_00	**SP2**	Sp2 transcription factor	protein_coding	21	22	22	23	1.054
6670	NM_001017371,NM	**SP3**	Sp3 transcription factor	protein_coding	43	35	44	36	0.820
6671	NM_001326542,NM	**SP4**	Sp4 transcription factor	protein_coding	11	9	12	10	0.768
6672	NM_001080391,NM	**SP100**	SP100 nuclear antigen	protein_coding	127	90	128	91	0.707
6674	NM_001374321,NM	**SPAG1**	sperm associated antige	protein_coding	7	6	8	7	0.921
6675	NM_001324113,NM	**UAP1**	UDP-N-acetylglucosamir	protein_coding	59	57	60	58	0.960
6676	NM_001317931,NM	**SPAG4**	sperm associated antige	protein_coding	0	1	1	2	1.106
6677	NM_001174044,NM	**SPAM1**	sperm adhesion molecul	protein_coding	0	0	1	1	1.000
6678	NM_001309443,NM	**SPARC**	secreted protein acidic a	protein_coding	0	0	1	1	1.000
6683	NM_001363823,NM	**SPAST**	spastin	protein_coding	13	11	14	12	0.868
6687	NM_001363850,NM	**SPG7**	SPG7 matrix AAA peptid	protein_coding	20	23	21	24	1.150
6688	NM_001080547,NM	**SPI1**	Spi-1 proto-oncogene	protein_coding	0	0	1	1	1.000
6689	NM_001243998,NM	**SPIB**	Spi-B transcription factor	protein_coding	1	1	2	2	0.973
6690	NM_001354966,NM	**SPINK1**	serine peptidase inhibitor	protein_coding	0	0	1	1	1.000
6691	NM_001271718,NM	**SPINK2**	serine peptidase inhibitor	protein_coding	0	0	1	1	1.000
6692	NM_001032367,NM	**SPINT1**	serine peptidase inhibitor	protein_coding	0	0	1	1	1.000
6693	NM_001030288,NM	**SPN**	sialophorin	protein_coding	96	87	97	88	0.913
6694	NM_006944,XM_00	**SPP2**	secreted phosphoprotein	protein_coding	0	0	1	1	1.000
6695	NM_004598	**SPOCK1**	SPARC (osteonectin), cv	protein_coding	8	6	9	7	0.845
6696	NM_000582,NM_00	**SPP1**	secreted phosphoprotein	protein_coding	1	1	2	2	1.111
6697	NM_003124	**SPR**	sepiapterin reductase	protein_coding	0	0	1	1	1.000
6698	NM_001199828,NM	**SPRR1A**	small proline rich protein	protein_coding	0	0	1	1	1.000
6699	NM_003125	**SPRR1B**	small proline rich protein	protein_coding	0	0	1	1	1.000
6700	NM_005988	**SPRR2A**	small proline rich protein	protein_coding	0	0	1	1	1.000
6701	NM_001017418,XM	**SPRR2B**	small proline rich protein	protein_coding	0	0	1	1	1.000
6702	NR_003062	**SPRR2C**	small proline rich protein	transcribed_pseu	0	0	1	1	1.000
6703	NM_001382248,NM	**SPRR2D**	small proline rich protein	protein_coding	0	0	1	1	1.000
6704	NM_001024209	**SPRR2E**	small proline rich protein	protein_coding	0	0	1	1	1.000
6705	NM_001014450,NM	**SPRR2F**	small proline rich protein	protein_coding	0	0	1	1	1.000
6706	NM_001014291,XM	**SPRR2G**	small proline rich protein	protein_coding	0	0	1	1	1.000
6707	NM_001097589,NM	**SPRR3**	small proline rich protein	protein_coding	0	0	1	1	1.000
6708	NM_003126,XM_01	**SPTA1**	spectrin alpha, erythrocy	protein_coding	1	1	2	2	0.905
6709	NM_001130438,NM	**SPTAN1**	spectrin alpha, non-eryth	protein_coding	77	74	78	75	0.961
6710	NM_001024858,NM	**SPTB**	spectrin beta, erythrocyti	protein_coding	0	0	1	1	0.992
6711	NM_003128,NM_17	**SPTBN1**	spectrin beta, non-erythr	protein_coding	8	8	9	9	1.003
6712	NM_006946,XM_00	**SPTBN2**	spectrin beta, non-erythr	protein_coding	0	0	1	1	1.008
6713	NM_003129,XM_01	**SQLE**	squalene epoxidase	protein_coding	20	18	21	19	0.888
6714	NM_005417,NM_19	**SRC**	SRC proto-oncogene, nc	protein_coding	0	0	1	1	1.011
6715	NM_001047,NM_00	**SRD5A1**	steroid 5 alpha-reductas	protein_coding	7	8	8	9	1.106
6716	NM_000348,XM_01	**SRD5A2**	steroid 5 alpha-reductas	protein_coding	0	0	1	1	1.000
6717	NM_001256891,NM	**SRI**	sorcin	protein_coding	132	134	133	135	1.015

6718	NM_001190906,NM	**AKR1D1**	aldo-keto reductase fami	protein_coding	0	0	1	1	1.000
6719	NR_028597	**SRD5A1P1**	steroid 5 alpha-reductase	transcribed_pseu	0	0	1	1	1.000
6720	NM_001005291,NM	**SREBF1**	sterol regulatory element	protein_coding	2	3	3	4	1.305
6721	NM_004599,NR_10	**SREBF2**	sterol regulatory element	protein_coding	79	75	80	76	0.950
6722	NM_001292001,NM	**SRF**	serum response factor	protein_coding	80	80	81	81	0.998
6723	NM_003132,XM_01	**SRM**	spermidine synthase	protein_coding	550	563	551	564	1.023
6725	NM_080823	**SRMS**	src-related kinase lacking	protein_coding	0	0	1	1	1.000
6726	NM_001130440,NM	**SRP9**	signal recognition particle	protein_coding	468	438	469	439	0.936
6727	NM_001309434,NM	**SRP14**	signal recognition particle	protein_coding	709	744	710	745	1.049
6728	NM_001204193,NM	**SRP19**	signal recognition particle	protein_coding	58	51	59	52	0.874
6729	NM_001146282,NM	**SRP54**	signal recognition particle	protein_coding	94	89	95	90	0.953
6730	NM_001260502,NM	**SRP68**	signal recognition particle	protein_coding	112	119	113	120	1.063
6731	NM_001267722,NM	**SRP72**	signal recognition particle	protein_coding	115	105	116	106	0.915
6732	NM_003137,NR_03	**SRPK1**	SRSF protein kinase 1	protein_coding	134	115	135	116	0.860
6733	NM_001278273,NM	**SRPK2**	SRSF protein kinase 2	protein_coding	20	17	21	18	0.835
6734	NM_001177842,NM	**SRPRA**	SRP receptor subunit alp	protein_coding	154	155	155	156	1.006
6736	NM_003140	**SRY**	sex determining region Y	protein_coding	0	0	1	1	1.000
6737	NM_003141	**TRIM21**	tripartite motif containing	protein_coding	58	55	59	56	0.960
6738	NM_001042369,NM	**RO60**	Ro60, Y RNA binding pro	protein_coding	20	20	21	21	0.972
6741	NM_001294145,NM	**SSB**	small RNA binding exonu	protein_coding	483	419	484	420	0.869
6742	NM_001256510,NM	**SSBP1**	single stranded DNA bin	protein_coding	430	431	431	432	1.000
6744	NM_001130445,NM	**ITPRID2**	ITPR interacting domain	protein_coding	2	2	3	3	0.772
6745	NM_001292008,NM	**SSR1**	signal sequence recepto	protein_coding	72	64	73	65	0.893
6746	NM_003145	**SSR2**	signal sequence recepto	protein_coding	389	393	390	394	1.011
6747	NM_001308197,NM	**SSR3**	signal sequence recepto	protein_coding	83	75	84	76	0.905
6748	NM_001204526,NM	**SSR4**	signal sequence recepto	protein_coding	289	336	290	337	1.161
6749	NM_003146,XM_01	**SSRP1**	structure specific recogn	protein_coding	409	401	410	402	0.981
6750	NM_001048	**SST**	somatostatin	protein_coding	0	0	1	1	1.000
6751	NM_001049	**SSTR1**	somatostatin receptor 1	protein_coding	0	0	1	1	1.000
6752	NM_001050	**SSTR2**	somatostatin receptor 2	protein_coding	0	0	1	1	1.000
6753	NM_001051,NM_00	**SSTR3**	somatostatin receptor 3	protein_coding	0	0	1	1	0.977
6754	NM_001052	**SSTR4**	somatostatin receptor 4	protein_coding	0	0	1	1	1.000
6755	NM_001053,NM_00	**SSTR5**	somatostatin receptor 5	protein_coding	0	0	1	1	1.000
6756	NM_001278691,NM	**SSX1**	SSX family member 1	protein_coding	0	0	1	1	1.000
6757	NM_001278697,NM	**SSX2**	SSX family member 2	protein_coding	0	0	1	1	1.000
6758	NM_021015,NM_17	**SSX5**	SSX family member 5	protein_coding	0	0	1	1	1.000
6759	NM_005636,NM_17	**SSX4**	SSX family member 4	protein_coding	0	0	1	1	1.000
6760	NM_001007559,NM	**SS18**	SS18 subunit of BAF chr	protein_coding	62	57	63	58	0.927
6764	NM_001376495,NM	**DENND2B**	DENN domain containing	protein_coding	0	0	1	1	0.870
6767	NM_001278589,NM	**ST13**	ST13 Hsp70 interacting p	protein_coding	174	168	175	169	0.963
6768	NM_021978	**ST14**	suppression of tumorigen	protein_coding	0	0	1	1	1.030
6769	NM_001292049,NM	**STAC**	SH3 and cysteine rich do	protein_coding	0	0	1	1	1.000
6770	NM_000349	**STAR**	steroidogenic acute regu	protein_coding	0	0	1	1	1.078
6771	gene-STARP1	**STARP1**	steroidogenic acute regu	pseudogene	0	0	1	1	1.000
6772	NM_007315,NM_13	**STAT1**	signal transducer and ac	protein_coding	231	208	232	209	0.903
6773	NM_005419,NM_19	**STAT2**	signal transducer and ac	protein_coding	13	10	14	11	0.799
6774	NM_001369512,NM	**STAT3**	signal transducer and ac	protein_coding	52	46	53	47	0.893
6775	NM_001243835,NM	**STAT4**	signal transducer and ac	protein_coding	89	86	90	87	0.961
6776	NM_001288718,NM	**STAT5A**	signal transducer and ac	protein_coding	65	67	66	68	1.027
6777	NM_012448,XM_00	**STAT5B**	signal transducer and ac	protein_coding	42	36	43	37	0.881
6778	NM_001178078,NM	**STAT6**	signal transducer and ac	protein_coding	31	30	32	31	0.973
6779	NM_001009181,NM	**STATH**	statherin	protein_coding	0	0	1	1	1.000
6780	NM_001037328,NM	**STAU1**	staufen double-stranded	protein_coding	135	129	136	130	0.958
6781	NM_003155	**STC1**	stanniocalcin 1	protein_coding	0	0	1	1	1.000
6782	NM_006948	**HSPA13**	heat shock protein family	protein_coding	52	43	53	44	0.829
6783	NM_005420,XM_01	**SULT1E1**	sulfotransferase family 1	protein_coding	0	0	1	1	1.000
6785	NM_022726	**ELOVL4**	ELOVL fatty acid elongas	protein_coding	11	10	12	11	0.874
6786	NM_001277961,NM	**STIM1**	stromal interaction molec	protein_coding	56	57	57	58	1.021
6787	NM_001193533,NM	**NEK4**	NIMA related kinase 4	protein_coding	15	14	16	15	0.914
6788	NM_001256312,NM	**STK3**	serine/threonine kinase 3	protein_coding	3	3	4	4	0.952
6789	NM_001352385,NM	**STK4**	serine/threonine kinase 4	protein_coding	129	102	130	103	0.794
6790	NM_001323303,NM	**AURKA**	aurora kinase A	protein_coding	217	183	218	184	0.844
6791	NR_001587	**AURKAP1**	aurora kinase A pseudog	transcribed_pseu	1	2	2	3	1.121
6792	NM_001037343,NM	**CDKL5**	cyclin dependent kinase	protein_coding	1	0	2	1	0.763
6793	NM_005990,XM_01	**STK10**	serine/threonine kinase 1	protein_coding	119	124	120	125	1.045
6794	NM_000455	**STK11**	serine/threonine kinase 1	protein_coding	59	64	60	65	1.085
6795	NM_001015878,NM	**AURKC**	aurora kinase C	protein_coding	1	1	2	2	0.816
6799	NM_001054,NM_00	**SULT1A2**	sulfotransferase family 1	protein_coding	0	0	1	1	1.380
6801	NM_003162,XM_00	**STRN**	striatin	protein_coding	9	7	10	8	0.783
6803	gene-STSP1	**STSP1**	steroid sulfatase (micros	pseudogene	0	0	1	1	1.000
6804	NM_001165903,NM	**STX1A**	syntaxin 1A	protein_coding	0	0	1	1	1.000
6809	NM_001178040,NM	**STX3**	syntaxin 3	protein_coding	155	156	156	157	1.001
6810	NM_001272095,NM	**STX4**	syntaxin 4	protein_coding	32	36	33	37	1.130
6811	NM_001244666,NM	**STX5**	syntaxin 5	protein_coding	25	26	26	27	1.023
6812	NM_001032221,NM	**STXBP1**	syntaxin binding protein	protein_coding	8	8	9	9	0.953
6813	NM_001127396,NM	**STXBP2**	syntaxin binding protein	protein_coding	62	65	63	66	1.046
6814	NM_007269	**STXBP3**	syntaxin binding protein	protein_coding	64	56	65	57	0.869
6815	NM_001130701,NM	**STYX**	serine/threonine/tyrosine	protein_coding	20	17	21	18	0.885
6817	NM_001055,NM_17	**SULT1A1**	sulfotransferase family 1	protein_coding	7	8	8	9	1.101
6818	NM_177552	**SULT1A3**	sulfotransferase family 1	protein_coding	0	0	1	1	0.860
6819	NM_001056,NM_17	**SULT1C2**	sulfotransferase family 1	protein_coding	0	0	1	1	1.000
6820	NM_004605,NM_17	**SULT2B1**	sulfotransferase family 2	protein_coding	0	0	1	1	1.000
6821	NM_000456,NM_00	**SUOX**	sulfite oxidase	protein_coding	0	0	1	1	1.052
6822	NM_003167	**SULT2A1**	sulfotransferase family 2	protein_coding	0	0	1	1	1.000
6824	gene-ETF1P1	**ETF1P1**	eukaryotic translation ter	pseudogene	0	0	1	1	1.029
6825	gene-ETF1P2	**ETF1P2**	eukaryotic translation ter	pseudogene	0	0	1	1	0.986
6827	NM_003168,NR_07	**SUPT4H1**	SPT4 homolog, DSIF elo	protein_coding	107	109	108	110	1.017
6829	NM_001111020,NM	**SUPT5H**	SPT5 homolog, DSIF elo	protein_coding	179	192	180	193	1.070
6830	NM_001320755,NM	**SUPT6H**	SPT6 homolog, histone c	protein_coding	102	97	103	98	0.954
6832	NM_001301683,NM	**SUPV3L1**	Suv3 like RNA helicase	protein_coding	79	78	80	79	0.998
6833	NM_000352,NM_00	**ABCC8**	ATP binding cassette sul	protein_coding	0	0	1	1	1.000
6834	NM_001280787,NM	**SURF1**	SURF1 cytochrome c oxi	protein_coding	27	31	28	32	1.139
6835	NM_001278928,NM	**SURF2**	surfeit 2	protein_coding	54	57	55	58	1.057
6836	NM_001280788,NM	**SURF4**	surfeit 4	protein_coding	232	231	233	232	0.994
6837	NM_133640,NM_18	**MED22**	mediator complex subun	protein_coding	38	38	39	39	0.997
6838	NM_001278942,NM	**SURF6**	surfeit 6	protein_coding	27	26	28	27	0.980
6839	NM_001282166,NM	**SUV39H1**	suppressor of variegation	protein_coding	36	38	37	39	1.055
6840	NM_001323599,NM	**SVIL**	supervillin	protein_coding	0	0	1	1	0.979
6843	NM_001297438,NM	**VAMP1**	vesicle associated memb	protein_coding	11	10	12	11	0.935
6844	NM_001330125,NM	**VAMP2**	vesicle associated memb	protein_coding	18	22	19	23	1.187
6845	NM_001145149,NM	**VAMP7**	vesicle associated memb	protein_coding	44	36	45	37	0.818
6846	NM_003175	**XCL2**	X-C motif chemokine liga	protein_coding	110	127	111	128	1.153
6847	NM_001282541,NM	**SYCP1**	synaptonemal complex p	protein_coding	0	0	1	1	1.000
6850	NM_001135052,NM	**SYK**	spleen associated tyrosin	protein_coding	6	6	7	7	0.961
6853	NM_006950,NM_13	**SYN1**	synapsin I	protein_coding	0	0	1	1	0.944
6854	NM_003178,NM_13	**SYN2**	synapsin II	protein_coding	0	0	1	1	1.000
6855	NM_003179	**SYP**	synaptophysin	protein_coding	3	5	4	6	1.313
6856	NM_001381910,NM	**SYPL1**	synaptophysin like 1	protein_coding	110	98	111	99	0.899
6857	NM_001135805,NM	**SYT1**	synaptotagmin 1	protein_coding	3	3	4	4	0.930
6860	NM_020783	**SYT4**	synaptotagmin 4	protein_coding	0	0	1	1	1.000
6861	NM_001297774,NM	**SYT5**	synaptotagmin 5	protein_coding	0	1	1	2	1.242
6862	NM_001270484,NM	**TBXT**	T-box transcription factor	protein_coding	0	0	1	1	1.000
6863	NM_003182,NM_01	**TAC1**	tachykinin precursor 1	protein_coding	0	0	1	1	1.000
6865	NM_001057	**TACR2**	tachykinin receptor 2	protein_coding	0	0	1	1	1.000
6866	NM_001178054,NM	**TAC3**	tachykinin precursor 3	protein_coding	0	0	1	1	1.000
6867	NM_001122824,NM	**TACC1**	transforming acidic coilec	protein_coding	129	106	130	107	0.824
6868	NM_001382777,NM	**ADAM17**	ADAM metallopeptidase	protein_coding	25	23	26	24	0.917
6869	NM_001058,NM_01	**TACR1**	tachykinin receptor 1	protein_coding	0	0	1	1	1.000
6870	NM_001059	**TACR3**	tachykinin receptor 3	protein_coding	0	0	1	1	1.000
6871	NM_001166105,NM	**TADA2A**	transcriptional adaptor 2	protein_coding	14	15	15	16	1.049
6872	NM_001286074,NM	**TAF1**	TATA-box binding protein	protein_coding	32	30	33	31	0.931
6873	NM_003184,XM_00	**TAF2**	TATA-box binding protein	protein_coding	56	49	57	50	0.872
6874	NM_003185	**TAF4**	TATA-box binding protein	protein_coding	24	23	25	24	0.941
6875	NM_001293725,NM	**TAF4B**	TATA-box binding protein	protein_coding	14	11	15	12	0.806
6876	NM_001001522,NM	**TAGLN**	transgelin	protein_coding	0	0	1	1	0.889
6877	NM_006951,NM_13	**TAF5**	TATA-box binding protein	protein_coding	23	21	24	22	0.912

6878	NM_001190415,NM	**TAF6**	TATA-box binding protei	protein_coding	66	73	67	74	1.110
6879	NM_005642	**TAF7**	TATA-box binding protei	protein_coding	163	148	164	149	0.910
6880	NM_001015892,NM	**TAF9**	TATA-box binding protei	protein_coding	84	89	85	90	1.057
6881	NM_006284	**TAF10**	TATA-box binding protei	protein_coding	29	33	30	34	1.147
6882	NM_001270488,NM	**TAF11**	TATA-box binding protei	protein_coding	83	79	84	80	0.956
6883	NM_001135218,NM	**TAF12**	TATA-box binding protei	protein_coding	64	68	65	69	1.062
6884	NM_005645	**TAF13**	TATA-box binding protei	protein_coding	70	75	71	76	1.080
6885	NM_003188,NM_14	**MAP3K7**	mitogen-activated protei	protein_coding	43	40	44	41	0.920
6886	NM_001287347,NM	**TAL1**	TAL bHLH transcription f	protein_coding	0	0	1	1	1.001
6887	NM_005421	**TAL2**	TAL bHLH transcription f	protein_coding	0	1	1	2	1.160
6888	NM_006755	**TALDO1**	transaldolase 1	protein_coding	212	212	213	213	1.001
6889	gene-TALDO1P1	**TALDO1P1**	transaldolase 1 pseudog	pseudogene	0	0	1	1	1.000
6890	NM_000593,NM_00	**TAP1**	transporter 1, ATP bindir	protein_coding	309	301	310	302	0.975
6891	NM_001290043,NM	**TAP2**	transporter 2, ATP bindir	protein_coding	167	155	168	156	0.930
6892	NM_003190,NM_17	**TAPBP**	TAP binding protein	protein_coding	141	142	142	143	1.004
6894	NM_005646,XM_00!	**TARBP1**	TAR (HIV-1) RNA binding	protein_coding	12	11	13	12	0.908
6895	NM_004178,NM_13	**TARBP2**	TARBP2 subunit of RISC	protein_coding	37	42	38	43	1.123
6896	gene-TARBP2P1	**TARBP2P1**	TARBP2 pseudogene 1	pseudogene	0	0	1	1	1.000
6897	NM_001258437,NM	**TARS1**	threonyl-tRNA synthetas	protein_coding	287	251	288	252	0.874
6898	NM_000353	**TAT**	tyrosine aminotransferas	protein_coding	0	0	1	1	1.000
6899	NM_001379200,NM	**TBX1**	T-box transcription factor	protein_coding	0	0	1	1	1.000
6900	NM_001346083,NM	**CNTN2**	contactin 2	protein_coding	0	0	1	1	1.000
6901	NM_000116,NM_00	**TAZ**	tafazzin	protein_coding	18	20	19	21	1.093
6902	NM_001297738,NM	**TBCA**	tubulin folding cofactor A	protein_coding	433	435	434	436	1.004
6903	NM_003192	**TBCC**	tubulin folding cofactor C	protein_coding	39	42	40	43	1.093
6904	NM_005993,XM_00!	**TBCD**	tubulin folding cofactor D	protein_coding	21	24	22	25	1.116
6905	NM_001079515,NM	**TBCE**	tubulin folding cofactor E	protein_coding	12	13	13	14	1.092
6906	NM_000354,XM_00!	**SERPINA7**	serpin family A member 7	protein_coding	0	0	1	1	1.000
6907	NM_001139466,NM	**TBL1X**	transducin beta like 1 X-l	protein_coding	42	36	43	37	0.849
6908	NM_001172085,NM	**TBP**	TATA-box binding protei	protein_coding	34	33	35	34	0.971
6909	NM_005994	**TBX2**	T-box transcription factor	protein_coding	0	0	1	1	1.000
6910	NM_000192,NM_08	**TBX5**	T-box transcription factor	protein_coding	0	0	1	1	1.000
6911	NM_004608,XM_00!	**TBX6**	T-box transcription factor	protein_coding	0	0	1	1	1.079
6913	NM_001330677,NM	**TBX15**	T-box transcription factor	protein_coding	0	0	1	1	0.977
6915	NM_001060,NM_20	**TBXA2R**	thromboxane A2 recepto	protein_coding	0	0	1	1	1.000
6916	NM_001061,NM_00	**TBXAS1**	thromboxane A synthase	protein_coding	0	0	1	1	1.000
6917	NM_006756,NM_20	**TCEA1**	transcription elongation f	protein_coding	192	169	193	170	0.881
6918	gene-TCEA1P1	**TCEA1P1**	transcription elongation f	pseudogene	0	0	1	1	1.000
6919	NM_003195,NM_19	**TCEA2**	transcription elongation f	protein_coding	17	16	18	17	0.966
6920	NM_003196,XM_00	**TCEA3**	transcription elongation f	protein_coding	0	0	1	1	0.977
6921	NM_001204857,NM	**ELOC**	elongin C	protein_coding	135	136	136	137	1.006
6922	gene-SKP1P1	**SKP1P1**	S-phase kinase associat	pseudogene	1	1	2	2	0.866
6923	NM_007108,NM_20	**ELOB**	elongin B	protein_coding	199	208	200	209	1.049
6924	NM_003198	**ELOA**	elongin A	protein_coding	53	51	54	52	0.963
6925	NM_001083962,NM	**TCF4**	transcription factor 4	protein_coding	6	6	7	7	0.939
6926	NM_005996,NM_01	**TBX3**	T-box transcription factor	protein_coding	0	0	1	1	1.000
6927	NM_000545,NM_00	**HNF1A**	HNF1 homeobox A	protein_coding	0	0	1	1	1.000
6928	NM_000458,NM_00	**HNF1B**	HNF1 homeobox B	protein_coding	0	0	1	1	1.000
6929	NM_001136139,NM	**TCF3**	transcription factor 3	protein_coding	73	69	74	70	0.949
6932	NM_001134851,NM	**TCF7**	transcription factor 7	protein_coding	0	0	1	1	1.000
6934	NM_001146274,NM	**TCF7L2**	transcription factor 7 like	protein_coding	0	0	1	1	1.047
6935	NM_001128128,NM	**ZEB1**	zinc finger E-box binding	protein_coding	6	5	7	6	0.789
6936	NM_001201334,NM	**GCFC2**	GC-rich sequence DNA-l	protein_coding	0	0	1	1	1.000
6938	NM_001306219,NM	**TCF12**	transcription factor 12	protein_coding	42	37	43	38	0.883
6939	NM_004609	**TCF15**	transcription factor 15	protein_coding	0	0	1	1	1.000
6940	NM_001324339,NM	**ZNF354A**	zinc finger protein 354A	protein_coding	9	9	10	10	0.919
6941	NM_001077511,NM	**TCF19**	transcription factor 19	protein_coding	68	66	69	67	0.975
6942	NM_001378418,NM	**TCF20**	transcription factor 20	protein_coding	29	25	30	26	0.868
6943	NM_003206,NM_19	**TCF21**	transcription factor 21	protein_coding	0	0	1	1	1.000
6944	NM_001271087,NM	**VPS72**	vacuolar protein sorting 7	protein_coding	72	74	73	75	1.028
6945	NM_170607,NM_19	**MLX**	MAX dimerization protein	protein_coding	114	108	115	109	0.949
6947	NM_001062	**TCN1**	transcobalamin 1	protein_coding	0	0	1	1	1.000
6948	NM_000355,NM_00	**TCN2**	transcobalamin 2	protein_coding	0	0	1	1	1.000
6949	NM_000356,NM_00	**TCOF1**	treacle ribosome bioogene	protein_coding	206	198	207	199	0.963
6950	NM_001008897,NM	**TCP1**	t-complex 1	protein_coding	465	449	466	450	0.966
6952	gene-TCP1P2	**TCP1P2**	t-complex 1 pseudogene	pseudogene	0	0	1	1	1.000
6953	NR_163193,NR_16:	**TCP10L3**	t-complex 10 like 3, pseu	transcribed_pseu	0	0	1	1	1.000
6954	NM_001261817,NM	**TCP11**	t-complex 11	protein_coding	0	0	1	1	1.000
6956	id-TRAV6	**TRAV6**	T cell receptor alpha vari	V_segment	0	0	1	1	1.000
6958	id-TRBV29OR9-2	**TRBV29OR9-2**	T cell receptor beta varia	V_segment_pseu	0	0	1	1	1.000
6959	id-TRBV21OR9-2	**TRBV21OR9-2**	T cell receptor beta varia	V_segment_pseu	0	0	1	1	1.000
6960	id-TRBV25OR9-2	**TRBV25OR9-2**	T cell receptor beta varia	V_segment_pseu	0	0	1	1	1.000
6961	id-TRBV24OR9-2	**TRBV24OR9-2**	T cell receptor beta varia	V_segment_pseu	0	0	1	1	1.000
6962	id-TRBV20OR9-2	**TRBV20OR9-2**	T cell receptor beta varia	V_segment_pseu	0	0	1	1	1.000
6966	id-TRGC1	**TRGC1**	T cell receptor gamma co	C_region	2	0	3	1	0.411
6967	id-TRGC2	**TRGC2**	T cell receptor gamma co	C_region	3	6	4	7	1.590
6968	id-TRGJ1	**TRGJ1**	T cell receptor gamma jo	J_segment	0	0	1	1	1.005
6969	id-TRGJ2	**TRGJ2**	T cell receptor gamma jo	J_segment	1	1	2	2	1.068
6970	id-TRGJP	**TRGJP**	T cell receptor gamma jo	J_segment	0	0	1	1	1.005
6971	id-TRGJP1	**TRGJP1**	T cell receptor gamma jo	J_segment	0	0	1	1	1.011
6972	id-TRGJP2	**TRGJP2**	T cell receptor gamma jo	J_segment	0	0	1	1	1.000
6973	id-TRGV1	**TRGV1**	T cell receptor gamma va	V_segment_pseu	0	0	1	1	1.000
6974	id-TRGV2	**TRGV2**	T cell receptor gamma va	V_segment	0	0	1	1	1.000
6975	NM_058222	**TECTB**	tectorin beta	protein_coding	0	0	1	1	1.000
6976	id-TRGV3	**TRGV3**	T cell receptor gamma va	V_segment	0	0	1	1	0.881
6977	id-TRGV4	**TRGV4**	T cell receptor gamma va	V_segment	0	0	1	1	1.000
6978	id-TRGV5	**TRGV5**	T cell receptor gamma va	V_segment	0	0	1	1	1.000
6979	id-TRGV5P	**TRGV5P**	T cell receptor gamma va	V_segment_pseu	0	0	1	1	1.000
6980	id-TRGV6	**TRGV6**	T cell receptor gamma va	V_segment_pseu	0	0	1	1	1.000
6981	id-TRGV7	**TRGV7**	T cell receptor gamma va	V_segment_pseu	19	19	20	20	1.009
6982	id-TRGV8	**TRGV8**	T cell receptor gamma va	V_segment	7	7	8	8	0.967
6983	id-TRGV9	**TRGV9**	T cell receptor gamma va	V_segment	18	17	19	18	0.977
6984	id-TRGV10	**TRGV10**	T cell receptor gamma va	V_segment_pseu	9	10	10	11	1.102
6985	id-TRGV11	**TRGV11**	T cell receptor gamma va	V_segment_pseu	0	0	1	1	1.000
6986	id-TRGVA	**TRGVA**	T cell receptor gamma va	V_segment_pseu	0	1	1	2	1.559
6987	id-TRGVB	**TRGVB**	T cell receptor gamma va	V_segment_pseu	0	0	1	1	0.677
6988	NM_022171	**TCTA**	T cell leukemia transloca	protein_coding	48	51	49	52	1.054
6990	NM_006520	**DYNLT3**	dynein light chain Tctex-	protein_coding	24	18	25	19	0.768
6991	NM_174910,NR_13	**TCTE3**	t-complex-associated-tes	protein_coding	5	6	6	7	1.090
6992	NM_021959,XM_00	**PPP1R11**	protein phosphatase 1 re	protein_coding	74	76	75	77	1.028
6993	NM_001291602,NM	**DYNLT1**	dynein light chain Tctex-	protein_coding	84	88	85	89	1.055
6996	NM_001363612,NM	**TDG**	thymine DNA glycosylase	protein_coding	88	72	89	73	0.824
6997	NM_001174136,NM	**TDGF1**	teratocarcinoma-derived	protein_coding	0	0	1	1	1.000
6998	NR_002718	**TDGF1P3**	teratocarcinoma-derived	transcribed_pseu	0	0	1	1	1.000
6999	NM_005651	**TDO2**	tryptophan 2,3-dioxygena	protein_coding	0	0	1	1	1.000
7001	NM_005809	**PRDX2**	peroxiredoxin 2	protein_coding	0	0	1	1	1.000
7002	gene-PRDX1P1	**PRDX1P1**	peroxiredoxin 1 pseudog	pseudogene	0	0	1	1	1.000
7003	NM_021961	**TEAD1**	TEA domain transcriptior	protein_coding	0	0	1	1	1.000
7004	NM_003213,NM_20	**TEAD4**	TEA domain transcriptior	protein_coding	0	0	1	1	1.117
7005	NM_003214	**TEAD3**	TEA domain transcriptior	protein_coding	0	0	1	1	1.000
7006	NM_003215,XM_01	**TEC**	tec protein tyrosine kinas	protein_coding	2	1	3	2	0.676
7007	NM_005422	**TECTA**	tectorin alpha	protein_coding	0	0	1	1	1.000
7008	NM_001145398,NM	**TEF**	TEF transcription factor,	protein_coding	3	5	4	6	1.368
7009	NM_001098576,NM	**TMBIM6**	transmembrane BAX inh	protein_coding	313	299	314	300	0.956
7010	NM_000459,NM_00	**TEK**	TEK receptor tyrosine kir	protein_coding	0	0	1	1	1.000
7011	NM_001319035,NM	**TEP1**	telomerase associated pr	protein_coding	3	3	4	4	0.886
7012	NR_001566	**TERC**	telomerase RNA compon	telomerase_RNA	0	0	1	1	0.788
7013	NM_003218,NM_01	**TERF1**	telomeric repeat binding	protein_coding	43	42	44	43	0.977
7014	NM_005652,XM_00!	**TERF2**	telomeric repeat binding	protein_coding	37	35	38	36	0.942
7015	NM_001193376,NM	**TERT**	telomerase reverse trans	protein_coding	3	3	4	4	1.127
7016	NM_001318230,NM	**TESK1**	testis associated actin re	protein_coding	15	16	16	17	1.034
7018	NM_001063,NM_00	**TF**	transferrin	protein_coding	0	0	1	1	1.000
7019	NM_001270782,NM	**TFAM**	transcription factor A, mi	protein_coding	77	68	78	69	0.881
7020	NM_001032280,NM	**TFAP2A**	transcription factor AP-2	protein_coding	3	3	4	4	0.970
7021	NM_003221,XM_01	**TFAP2B**	transcription factor AP-2	protein_coding	0	0	1	1	1.000
7022	NM_003222	**TFAP2C**	transcription factor AP-2	protein_coding	0	0	1	1	1.000

7023	NM_003223,XM_01	**TFAP4**	transcription factor AP-4	protein_coding	30	32	31	33	1.058
7024	NM_001173452,NM	**TFCP2**	transcription factor CP2	protein_coding	36	34	37	35	0.955
7025	NM_005654,XM_01	**NR2F1**	nuclear receptor subfam	protein_coding	0	0	1	1	1.126
7026	NM_001145155,NM	**NR2F2**	nuclear receptor subfam	protein_coding	0	0	1	1	1.034
7027	NM_007111,NR_02	**TFDP1**	transcription factor Dp-1	protein_coding	429	414	430	415	0.966
7029	NM_001178138,NM	**TFDP2**	transcription factor Dp-2	protein_coding	7	6	8	7	0.922
7030	NM_001282142,NM	**TFE3**	transcription factor bindi	protein_coding	27	26	28	27	0.948
7031	NM_003225	**TFF1**	trefoil factor 1	protein_coding	0	0	1	1	1.000
7032	NM_005423	**TFF2**	trefoil factor 2	protein_coding	0	0	1	1	1.000
7033	NM_003226	**TFF3**	trefoil factor 3	protein_coding	0	0	1	1	1.000
7035	NM_001032281,NM	**TFPI**	tissue factor pathway inh	protein_coding	0	0	1	1	1.053
7036	NM_001206855,NM	**TFR2**	transferrin receptor 2	protein_coding	11	12	12	13	1.013
7037	NM_001128148,NM	**TFRC**	transferrin receptor	protein_coding	184	184	185	185	1.000
7038	NM_003235,XM_00	**TG**	thyroglobulin	protein_coding	0	0	1	1	0.967
7039	NM_001099691,NM	**TGFA**	transforming growth fact	protein_coding	1	1	2	2	0.693
7040	NM_000660,XM_01	**TGFB1**	transforming growth fact	protein_coding	202	216	203	217	1.067
7041	NM_001042454,NM	**TGFB1I1**	transforming growth fact	protein_coding	0	0	1	1	1.002
7042	NM_001135599,NM	**TGFB2**	transforming growth fact	protein_coding	0	0	1	1	0.993
7043	NM_001329938,NM	**TGFB3**	transforming growth fact	protein_coding	0	0	1	1	1.000
7044	NM_001172425,NM	**LEFTY2**	left-right determination fa	protein_coding	0	0	1	1	0.958
7045	NM_000358	**TGFBI**	transforming growth fact	protein_coding	0	0	1	1	1.000
7046	NM_001130916,NM	**TGFBR1**	transforming growth fact	protein_coding	43	37	44	38	0.867
7047	NM_003241,XM_01	**TGM4**	transglutaminase 4	protein_coding	0	0	1	1	1.000
7048	NM_001024847,NM	**TGFBR2**	transforming growth fact	protein_coding	19	17	20	18	0.873
7049	NM_001195683,NM	**TGFBR3**	transforming growth fact	protein_coding	5	4	6	5	0.930
7050	NM_001278682,NM	**TGIF1**	TGFB induced factor hor	protein_coding	13	13	14	14	0.961
7051	NM_000359	**TGM1**	transglutaminase 1	protein_coding	0	0	1	1	1.201
7052	NM_001323316,NM	**TGM2**	transglutaminase 2	protein_coding	0	0	1	1	1.000
7053	NM_003245	**TGM3**	transglutaminase 3	protein_coding	0	0	1	1	1.000
7054	NM_000360,NM_19	**TH**	tyrosine hydroxylase	protein_coding	0	0	1	1	1.000
7056	NM_000361	**THBD**	thrombomodulin	protein_coding	0	0	1	1	1.000
7057	NM_003246,XM_01	**THBS1**	thrombospondin 1	protein_coding	0	0	1	1	1.030
7058	NM_001381939,NM	**THBS2**	thrombospondin 2	protein_coding	0	0	1	1	1.000
7059	NM_001252607,NM	**THBS3**	thrombospondin 3	protein_coding	0	0	1	1	0.915
7060	NM_001306212,NM	**THBS4**	thrombospondin 4	protein_coding	0	0	1	1	0.995
7062	NM_007113	**TCHH**	trichohyalin	protein_coding	0	0	1	1	1.000
7064	NM_003249,XM_01	**THOP1**	thimet oligopeptidase 1	protein_coding	91	90	92	91	0.992
7066	NM_000460,NM_00	**THPO**	thrombopoietin	protein_coding	13	12	14	13	0.936
7067	NM_001190918,NM	**THRA**	thyroid hormone recepto	protein_coding	2	1	3	2	0.913
7068	NM_000461,NM_00	**THRB**	thyroid hormone recepto	protein_coding	0	0	1	1	1.000
7069	NM_003251	**THRSP**	thyroid hormone respons	protein_coding	0	0	1	1	1.000
7070	NM_001311160,NM	**THY1**	Thy-1 cell surface antige	protein_coding	0	0	1	1	1.000
7071	NM_001032282,NM	**KLF10**	Kruppel like factor 10	protein_coding	34	35	35	36	1.033
7072	NM_001351508,NM	**TIA1**	TIA1 cytotoxic granule as	protein_coding	56	48	57	49	0.858
7073	NM_001033925,NM	**TIAL1**	TIA1 cytotoxic granule as	protein_coding	62	60	63	61	0.967
7074	NM_001353684,NM	**TIAM1**	TIAM Rac1 associated G	protein_coding	7	6	8	7	0.840
7075	NM_001253357,NM	**TIE1**	tyrosine kinase with imm	protein_coding	2	2	3	3	1.062
7076	NM_003254,XM_01	**TIMP1**	TIMP metallopeptidase in	protein_coding	152	170	153	171	1.118
7077	NM_003255	**TIMP2**	TIMP metallopeptidase in	protein_coding	0	0	1	1	1.000
7078	NM_000362	**TIMP3**	TIMP metallopeptidase in	protein_coding	0	0	1	1	1.000
7079	NM_003256	**TIMP4**	TIMP metallopeptidase in	protein_coding	0	0	1	1	1.000
7080	NM_001079668,NM	**NKX2-1**	NK2 homeobox 1	protein_coding	0	0	1	1	1.000
7082	NM_001301025,NM	**TJP1**	tight junction protein 1	protein_coding	23	21	24	22	0.921
7083	NM_001346663,NM	**TK1**	thymidine kinase 1	protein_coding	166	191	167	192	1.148
7084	NM_001172643,NM	**TK2**	thymidine kinase 2	protein_coding	2	2	3	3	1.165
7086	NM_001064,NM_00	**TKT**	transketolase	protein_coding	264	266	265	267	1.008
7087	NM_003259,XM_01	**ICAM5**	intercellular adhesion mo	protein_coding	1	1	2	2	1.072
7088	NM_001303103,NM	**TLE1**	TLE family member 1, tra	protein_coding	0	0	1	1	1.024
7089	NM_001144761,NM	**TLE2**	TLE family member 2, tra	protein_coding	0	0	1	1	0.967
7090	NM_001105192,NM	**TLE3**	TLE family member 3, tra	protein_coding	36	30	37	31	0.853
7091	NM_001282748,NM	**TLE4**	TLE family member 4, tra	protein_coding	4	4	5	5	0.911
7092	NM_001204760,NM	**TLL1**	tolloid like 1	protein_coding	1	1	2	2	0.975
7093	NM_012465	**TLL2**	tolloid like 2	protein_coding	0	0	1	1	1.044
7094	NM_006289	**TLN1**	talin 1	protein_coding	85	79	86	80	0.931
7095	NM_003262,XM_01	**SEC62**	SEC62 homolog, preprot	protein_coding	38	32	39	33	0.830
7096	NM_003263,XM_00	**TLR1**	toll like receptor 1	protein_coding	1	1	2	2	0.893
7097	NM_001318787,NM	**TLR2**	toll like receptor 2	protein_coding	0	0	1	1	1.000
7098	NM_003265	**TLR3**	toll like receptor 3	protein_coding	6	4	7	5	0.831
7099	NM_003266,NM_13	**TLR4**	toll like receptor 4	protein_coding	0	0	1	1	1.000
7100	NM_003268,XM_00	**TLR5**	toll like receptor 5	protein_coding	0	0	1	1	1.000
7101	NM_001286102,NM	**NR2E1**	nuclear receptor subfam	protein_coding	0	0	1	1	1.000
7102	NM_004615	**TSPAN7**	tetraspanin 7	protein_coding	0	0	1	1	1.000
7103	NM_001369760,NM	**TSPAN8**	tetraspanin 8	protein_coding	0	0	1	1	1.000
7104	NM_004617	**TM4SF4**	transmembrane 4 L six fa	protein_coding	0	0	1	1	1.000
7105	NM_001278740,NM	**TSPAN6**	tetraspanin 6	protein_coding	0	0	1	1	1.000
7106	NM_001025234,NM	**TSPAN4**	tetraspanin 4	protein_coding	2	2	3	3	0.825
7107	NM_003272,XM_01	**GPR137B**	G protein-coupled recep	protein_coding	0	0	1	1	1.000
7108	NM_001277233,NM	**TM7SF2**	transmembrane 7 superf	protein_coding	4	5	5	6	1.056
7109	NM_001351709,NM	**TRAPPC10**	trafficking protein particle	protein_coding	30	26	31	27	0.880
7110	NM_001363879,NM	**TMF1**	TATA element modulator	protein_coding	13	12	14	13	0.947
7111	NM_001166116,NM	**TMOD1**	tropomodulin 1	protein_coding	0	0	1	1	1.000
7112	NM_001032283,NM	**TMPO**	thymopoietin	protein_coding	199	183	200	184	0.924
7113	NM_001135099,NM	**TMPRSS2**	transmembrane serine p	protein_coding	0	0	1	1	1.000
7114	NM_021109	**TMSB4X**	thymosin beta 4 X-linked	protein_coding	14,859	15,469	14,860	15,470	1.041
7115	gene-TMSB4XP1	**TMSB4XP1**	TMSB4X pseudogene 1	pseudogene	16	18	17	19	1.171
7116	gene-TMSB4XP2	**TMSB4XP2**	TMSB4X pseudogene 2	pseudogene	0	0	1	1	1.004
7117	gene-TMSB4XP8	**TMSB4XP8**	TMSB4X pseudogene 8	pseudogene	327	386	328	387	1.181
7118	gene-TMSB4XP4	**TMSB4XP4**	TMSB4X pseudogene 4	pseudogene	2	2	3	3	1.249
7119	gene-TMSB4XP5	**TMSB4XP5**	TMSB4X pseudogene 5	pseudogene	0	0	1	1	0.922
7120	gene-TMSB4XP6	**TMSB4XP6**	TMSB4X pseudogene 6	pseudogene	3	3	4	4	1.053
7121	gene-TMSB4XP7	**TMSB4XP7**	TMSB4X pseudogene 7	pseudogene	0	0	1	1	1.000
7122	NM_001130861,NM	**CLDN5**	claudin 5	protein_coding	0	0	1	1	1.000
7123	NM_001308394,NM	**CLEC3B**	C-type lectin domain fam	protein_coding	1	1	2	2	0.954
7124	NM_000594	**TNF**	tumor necrosis factor	protein_coding	0	1	1	2	1.060
7125	NM_003279,XM_01	**TNNC2**	troponin C2, fast skeletal	protein_coding	0	0	1	1	1.000
7126	NM_021137,XM_01	**TNFAIP1**	TNF alpha induced prote	protein_coding	11	12	12	13	1.064
7127	NM_001371220,NM	**TNFAIP2**	TNF alpha induced prote	protein_coding	0	0	1	1	1.000
7128	NM_001270507,NM	**TNFAIP3**	TNF alpha induced prote	protein_coding	6	5	7	6	0.896
7130	NM_007115	**TNFAIP6**	TNF alpha induced prote	protein_coding	0	0	1	1	1.000
7132	NM_001065,NM_00	**TNFRSF1A**	TNF receptor superfamily	protein_coding	79	84	80	85	1.063
7133	NM_001066,XM_01	**TNFRSF1B**	TNF receptor superfamily	protein_coding	395	409	396	410	1.036
7134	NM_003280	**TNNC1**	troponin C1, slow skeleta	protein_coding	0	1	1	2	2.255
7135	NM_003281	**TNNI1**	troponin I1, slow skeletal	protein_coding	0	0	1	1	1.000
7136	NM_001145829,NM	**TNNI2**	troponin I2, fast skeletal	protein_coding	1	1	2	2	0.758
7137	NM_000363	**TNNI3**	troponin I3, cardiac type	protein_coding	0	0	1	1	0.898
7138	NM_001126132,NM	**TNNT1**	troponin T1, slow skeleta	protein_coding	0	0	1	1	1.000
7139	NM_000364,NM_00	**TNNT2**	troponin T2, cardiac type	protein_coding	0	0	1	1	1.000
7140	NM_001042780,NM	**TNNT3**	troponin T3, fast skeletal	protein_coding	0	0	1	1	1.000
7141	NM_003284	**TNP1**	transition protein 1	protein_coding	0	0	1	1	1.000
7142	NM_005425	**TNP2**	transition protein 2	protein_coding	0	0	1	1	1.000
7143	NM_001328635,NM	**TNR**	tenascin R	protein_coding	0	0	1	1	1.000
7145	NM_001308022,NM	**TNS1**	tensin 1	protein_coding	0	0	1	1	0.871
7146	NR_001284	**TNXA**	tenascin XA (pseudogen	transcribed_pseu	0	0	1	1	1.000
7148	NM_001365276,NM	**TNXB**	tenascin XB	protein_coding	0	0	1	1	1.023
7150	NM_003286	**TOP1**	DNA topoisomerase I	protein_coding	288	257	289	258	0.892
7151	NR_002719	**TOP1P1**	DNA topoisomerase I pse	transcribed_pseu	0	0	1	1	1.045
7152	NR_001283	**TOP1P2**	DNA topoisomerase I pse	transcribed_pseu	0	0	1	1	1.000
7153	NM_001067,XM_00	**TOP2A**	DNA topoisomerase II alp	protein_coding	413	329	414	330	0.798
7155	NM_001068,NM_00	**TOP2B**	DNA topoisomerase II be	protein_coding	73	57	74	58	0.785
7156	NM_001320759,NM	**TOP3A**	DNA topoisomerase III al	protein_coding	19	18	20	19	0.923
7157	NM_000546,NM_00	**TP53**	tumor protein p53	protein_coding	7	8	8	9	1.093
7158	NM_001141979,NM	**TP53BP1**	tumor protein p53 bindin	protein_coding	37	32	38	33	0.891
7159	NM_001031685,NM	**TP53BP2**	tumor protein p53 bindin	protein_coding	25	22	26	23	0.880
7161	NM_001126240,NM	**TP73**	tumor protein p73	protein_coding	0	0	1	1	1.000
7162	NM_001166392,NM	**TPBG**	trophoblast glycoprotein	protein_coding	0	0	1	1	1.000
7163	NM_001025252,NM	**TPD52**	tumor protein D52	protein_coding	48	47	49	48	0.971

7164	NM_001003395,NM	**TPD52L1**	TPD52 like 1	protein_coding	0	0	1	1	1.000
7165	NM_001243891,NM	**TPD52L2**	TPD52 like 2	protein_coding	122	125	123	126	1.026
7166	NM_004179	**TPH1**	tryptophan hydroxylase 1	protein_coding	2	2	3	3	1.006
7167	NM_000365,NM_00	**TPI1**	triosephosphate isomera	protein_coding	933	946	934	947	1.014
7168	NM_000366,NM_00	**TPM1**	tropomyosin 1	protein_coding	5	4	6	5	0.769
7169	NM_001301226,NM	**TPM2**	tropomyosin 2	protein_coding	0	0	1	1	1.000
7170	NM_001043351,NM	**TPM3**	tropomyosin 3	protein_coding	527	534	528	535	1.013
7171	NM_001145160,NM	**TPM4**	tropomyosin 4	protein_coding	689	623	690	624	0.904
7172	NM_000367,NM_00	**TPMT**	thiopurine S-methyltrans	protein_coding	28	25	29	26	0.899
7173	NM_000547,NM_00	**TPO**	thyroid peroxidase	protein_coding	0	0	1	1	1.000
7174	NM_001330588,NM	**TPP2**	tripeptidyl peptidase 2	protein_coding	68	57	69	58	0.850
7175	NM_003292	**TPR**	translocated promoter re	protein_coding	73	66	74	67	0.901
7177	NM_003294	**TPSAB1**	tryptase alpha/beta 1	protein_coding	0	0	1	1	1.000
7178	NM_001286272,NM	**TPT1**	tumor protein, translatior	protein_coding	770	735	771	736	0.955
7179	NM_001290224,NM	**TPTE**	transmembrane phospha	protein_coding	0	0	1	1	1.000
7180	NM_001142407,NM	**CRISP2**	cysteine rich secretory pr	protein_coding	0	0	1	1	1.000
7181	NM_001032287,NM	**NR2C1**	nuclear receptor subfam	protein_coding	10	11	11	12	1.033
7182	NM_001291694,NM	**NR2C2**	nuclear receptor subfam	protein_coding	12	10	13	11	0.857
7184	NM_003299	**HSP90B1**	heat shock protein 90 be	protein_coding	674	679	675	680	1.007
7185	NM_001190945,NM	**TRAF1**	TNF receptor associated	protein_coding	21	21	22	22	1.029
7186	NM_021138,XM_01	**TRAF2**	TNF receptor associated	protein_coding	47	43	48	44	0.920
7187	NM_001199427,NM	**TRAF3**	TNF receptor associated	protein_coding	23	22	24	23	0.945
7188	NM_001033910,NM	**TRAF5**	TNF receptor associated	protein_coding	32	27	33	28	0.840
7189	NM_004620,NM_14	**TRAF6**	TNF receptor associated	protein_coding	5	5	6	6	0.934
7190	NR_073383	**HSP90B2P**	heat shock protein 90 be	transcribed_pseu	1	1	2	2	0.917
7193	rna-TRE-TTC3-1	**TRE-TTC3-1**	tRNA-Glu	tRNA	0	0	1	1	1.000
7195	rna-TRG-CCC1-1	**TRG-CCC1-1**	tRNA-Gly	tRNA	0	0	1	1	1.000
7196	rna-TRG-GCC2-6	**TRG-GCC2-6**	tRNA-Gly	tRNA	0	0	1	1	1.000
7197	rna-TRG-TCC1-1	**TRG-TCC1-1**	tRNA-Gly	tRNA	1	1	2	2	1.104
7198	rna-TRG-GCC3-1	**TRG-GCC3-1**	tRNA-Gly	tRNA	0	0	1	1	1.000
7200	NM_007117	**TRH**	thyrotropin releasing hor	protein_coding	0	0	1	1	1.000
7201	NM_003301	**TRHR**	thyrotropin releasing hor	protein_coding	0	0	1	1	1.000
7202	rna-TRI-AAT9-1	**TRI-AAT9-1**	tRNA-Ile	tRNA	0	0	1	1	1.000
7203	NM_001008800,NM	**CCT3**	chaperonin containing T(	protein_coding	883	873	884	874	0.988
7204	NM_007118,NR_13	**TRIO**	trio Rho guanine nucleot	protein_coding	3	2	4	3	0.793
7205	NM_003302	**TRIP6**	thyroid hormone recepto	protein_coding	1	1	2	2	1.035
7206	rna-TRK-TTT3-5	**TRK-TTT3-5**	tRNA-Lys	tRNA	0	0	1	1	1.442
7207	rna-TRL-AAG2-3	**TRL-AAG2-3**	tRNA-Leu	tRNA	0	0	1	1	1.000
7208	rna-TRL-TAG1-1	**TRL-TAG1-1**	tRNA-Leu	tRNA	0	0	1	1	1.000
7210	rna-TRX-CAT1-2	**TRX-CAT1-2**	tRNA-Met	tRNA	0	0	1	1	1.000
7212	rna-TRX-CAT2-1	**TRX-CAT2-1**	tRNA-Met	tRNA	0	0	1	1	1.000
7214	rna-TRN-GTT2-7	**TRN-GTT2-7**	tRNA-Asn	tRNA	0	0	1	1	1.007
7216	NM_001039705,NM	**TRO**	trophinin	protein_coding	0	0	1	1	1.000
7217	rna-TRP-AGG2-5	**TRP-AGG2-5**	tRNA-Pro	tRNA	0	0	1	1	1.000
7218	rna-TRP-AGG2-6	**TRP-AGG2-6**	tRNA-Pro	tRNA	0	0	1	1	1.000
7219	rna-TRP-TGG3-1	**TRP-TGG3-1**	tRNA-Pro	tRNA	0	0	1	1	1.117
7220	NM_001251845,NM	**TRPC1**	transient receptor potent	protein_coding	0	0	1	1	1.000
7221	NR_002720	**TRPC2**	transient receptor potent	transcribed_pseu	0	0	1	1	1.000
7222	NM_001130698,NM	**TRPC3**	transient receptor potent	protein_coding	0	0	1	1	1.000
7223	NM_001135955,NM	**TRPC4**	transient receptor potent	protein_coding	0	0	1	1	1.000
7224	NM_012471,XM_01	**TRPC5**	transient receptor potent	protein_coding	0	0	1	1	1.000
7225	NM_004621,XM_01	**TRPC6**	transient receptor potent	protein_coding	0	0	1	1	1.000
7226	NM_001320350,NM	**TRPM2**	transient receptor potent	protein_coding	9	8	10	9	0.903
7227	NM_001282902,NM	**TRPS1**	transcriptional repressor	protein_coding	2	2	3	3	0.908
7228	rna-TRQ-CTG1-5	**TRQ-CTG1-5**	tRNA-Gln	tRNA	0	0	1	1	1.434
7229	rna-TRQ-TTG1-1	**TRQ-TTG1-1**	tRNA-Gln	tRNA	0	0	1	1	1.000
7230	rna-TRR-TCT2-1	**TRR-TCT2-1**	tRNA-Arg	tRNA	0	0	1	1	1.000
7231	rna-TRR-ACG1-2	**TRR-ACG1-2**	tRNA-Arg	tRNA	0	0	1	1	0.751
7232	rna-TRR-TCG4-1	**TRR-TCG4-1**	tRNA-Arg	tRNA	0	0	1	1	1.000
7233	rna-TRR-CCG2-1	**TRR-CCG2-1**	tRNA-Arg	tRNA	0	0	1	1	1.000
7236	rna-TRT-TGT6-1	**TRT-TGT6-1**	tRNA-Thr	tRNA	0	0	1	1	1.000
7237	rna-TRT-TGT3-1	**TRT-TGT3-1**	tRNA-Thr	tRNA	0	0	1	1	1.000
7239	rna-TRV-AAC1-4	**TRV-AAC1-4**	tRNA-Val	tRNA	0	0	1	1	1.000
7240	rna-TRV-CAC1-2	**TRV-CAC1-2**	tRNA-Val	tRNA	0	0	1	1	1.000
7241	rna-TRV-AAC1-3	**TRV-AAC1-3**	tRNA-Val	tRNA	0	0	1	1	1.000
7247	NM_001261401,NM	**TSN**	translin	protein_coding	123	116	124	117	0.940
7248	NM_000368,NM_00	**TSC1**	TSC complex subunit 1	protein_coding	5	5	6	6	0.905
7249	NM_000548,NM_00	**TSC2**	TSC complex subunit 2	protein_coding	27	27	28	28	0.977
7251	NM_006292,XM_00	**TSG101**	tumor susceptibility 101	protein_coding	90	83	91	84	0.925
7252	NM_000549,NM_00	**TSHB**	thyroid stimulating horm	protein_coding	0	0	1	1	1.000
7253	NM_000369,NM_00	**TSHR**	thyroid stimulating horm	protein_coding	0	0	1	1	0.980
7257	NM_005999	**TSNAX**	translin associated facto	protein_coding	58	53	59	54	0.916
7258	NM_001197242,NM	**TSPY1**	testis specific protein Y-l	protein_coding	0	0	1	1	1.000
7259	NM_003309	**TSPYL1**	TSPY like 1	protein_coding	25	26	26	27	1.023
7260	NM_001330530,NM	**EIPR1**	EARP complex and GAR	protein_coding	29	37	30	38	1.263
7262	NM_003311	**PHLDA2**	pleckstrin homology like	protein_coding	0	0	1	1	0.827
7263	NM_001270483,NM	**TST**	thiosulfate sulfurtransfer	protein_coding	16	19	17	20	1.168
7264	NM_001317783,NM	**GFUS**	GDP-L-fucose synthase	protein_coding	39	45	40	46	1.151
7265	NM_001282500,NM	**TTC1**	tetratricopeptide repeat c	protein_coding	155	152	156	153	0.978
7266	NM_001144766,NM	**DNAJC7**	DnaJ heat shock protein	protein_coding	167	162	168	163	0.969
7267	NM_001001894,NM	**TTC3**	tetratricopeptide repeat c	protein_coding	27	20	28	21	0.772
7268	NM_001291333,NM	**TTC4**	tetratricopeptide repeat c	protein_coding	91	91	92	92	1.002
7270	NM_001205296,NM	**TTF1**	transcription termination	protein_coding	23	24	24	25	1.020
7272	NM_001166691,NM	**TTK**	TTK protein kinase	protein_coding	58	53	59	54	0.923
7273	NM_001256850,NM	**TTN**	titin	protein_coding	0	0	1	1	0.995
7274	NM_000370,XM_00	**TTPA**	alpha tocopherol transfe	protein_coding	0	0	1	1	1.000
7275	NM_003320,NM_17	**TUB**	TUB bipartite transcriptio	protein_coding	0	0	1	1	1.000
7276	NM_000371	**TTR**	transthyretin	protein_coding	0	0	1	1	1.000
7277	NM_001278552,NM	**TUBA4A**	tubulin alpha 4a	protein_coding	270	271	271	272	1.002
7278	NM_006001	**TUBA3C**	tubulin alpha 3c	protein_coding	19	15	20	16	0.816
7280	NM_001069,NM_00	**TUBB2A**	tubulin beta 2A class IIa	protein_coding	1	1	2	2	1.169
7283	NM_001070,XM_02	**TUBG1**	tubulin gamma 1	protein_coding	175	180	176	181	1.029
7284	NM_001365360,NM	**TUFM**	Tu translation elongation	protein_coding	455	472	456	473	1.037
7286	NM_001126337,NM	**TUFT1**	tuftelin 1	protein_coding	0	0	1	1	1.000
7287	NM_001289395,NM	**TULP1**	TUB like protein 1	protein_coding	0	0	1	1	1.000
7288	NM_003323,XM_00	**TULP2**	TUB like protein 2	protein_coding	0	0	1	1	1.000
7289	NM_001160408,NM	**TULP3**	TUB like protein 3	protein_coding	10	12	11	13	1.180
7290	NM_003325	**HIRA**	histone cell cycle regulat	protein_coding	59	62	60	63	1.050
7291	NM_000474,NR_14	**TWIST1**	twist family bHLH transcr	protein_coding	0	0	1	1	1.000
7292	NM_001297562,NM	**TNFSF4**	TNF superfamily membe	protein_coding	1	1	2	2	0.961
7293	NM_003327,XM_01	**TNFRSF4**	TNF receptor superfamily	protein_coding	0	0	1	1	1.010
7294	NM_003328,XM_01	**TXK**	TXK tyrosine kinase	protein_coding	57	46	58	47	0.807
7295	NM_001244938,NM	**TXN**	thioredoxin	protein_coding	613	651	614	652	1.061
7296	NM_001093771,NM	**TXNRD1**	thioredoxin reductase 1	protein_coding	166	149	167	150	0.898
7297	NM_003331,XM_01	**TYK2**	tyrosine kinase 2	protein_coding	60	59	61	60	0.985
7298	NM_001071,NM_00	**TYMS**	thymidylate synthetase	protein_coding	346	330	347	331	0.952
7299	NM_000372,XM_01	**TYR**	tyrosinase	protein_coding	0	0	1	1	1.000
7300	gene-TYRL	**TYRL**	tyrosinase like (pseudog	pseudogene	0	0	1	1	1.000
7301	NM_001330264,NM	**TYRO3**	TYRO3 protein tyrosine k	protein_coding	0	0	1	1	1.000
7302	NR_028510	**TYRO3P**	TYRO3P protein tyrosine	transcribed_pseu	0	0	1	1	1.000
7305	NM_001173514,NM	**TYROBP**	transmembrane immune	protein_coding	17	19	18	20	1.109
7306	NM_000550,XR_00	**TYRP1**	tyrosinase related protein	protein_coding	0	0	1	1	1.000
7307	NM_001025203,NM	**U2AF1**	U2 small nuclear RNA au	protein_coding	288	310	289	311	1.074
7310	gene-ZRSR2P1	**ZRSR2P1**	ZRSR2 pseudogene 1	pseudogene	0	0	1	1	0.960
7311	NM_001033930,NM	**UBA52**	ubiquitin A-52 residue rib	protein_coding	422	444	423	445	1.053
7312	gene-UBA52P1	**UBA52P1**	ubiquitin A-52 residue rib	pseudogene	0	0	1	1	1.000
7313	gene-UBA52P2	**UBA52P2**	ubiquitin A-52 residue rib	pseudogene	0	0	1	1	1.000
7314	NM_001281716,NM	**UBB**	ubiquitin B	protein_coding	899	965	900	966	1.074
7315	gene-UBBP1	**UBBP1**	ubiquitin B pseudogene	pseudogene	0	0	1	1	1.070
7316	NM_021009	**UBC**	ubiquitin C	protein_coding	1,132	1,238	1,133	1,239	1.094
7317	NM_003334,NM_15	**UBA1**	ubiquitin like modifier act	protein_coding	263	267	264	268	1.015
7318	NM_003335,XM_00	**UBA7**	ubiquitin like modifier act	protein_coding	1	2	2	3	1.078
7319	NM_001282161,NM	**UBE2A**	ubiquitin conjugating enz	protein_coding	149	131	150	132	0.883
7320	NM_003337	**UBE2B**	ubiquitin conjugating enz	protein_coding	50	50	51	51	1.011
7321	NM_001204880,NM	**UBE2D1**	ubiquitin conjugating enz	protein_coding	23	22	24	23	0.969
7322	NM_003339,NM_18	**UBE2D2**	ubiquitin conjugating enz	protein_coding	152	146	153	147	0.956

7323	NM_001300795,NM	**UBE2D3**	ubiquitin conjugating enz	protein_coding	203	188	204	189	0.925
7324	NM_001202476,NM	**UBE2E1**	ubiquitin conjugating enz	protein_coding	50	44	51	45	0.894
7325	NM_001370225,NM	**UBE2E2**	ubiquitin conjugating enz	protein_coding	0	0	1	1	1.000
7326	NM_003342	**UBE2G1**	ubiquitin conjugating enz	protein_coding	47	45	48	46	0.959
7327	NM_001202489,NM	**UBE2G2**	ubiquitin conjugating enz	protein_coding	129	119	130	120	0.923
7328	NM_001202498,NM	**UBE2H**	ubiquitin conjugating enz	protein_coding	24	21	25	22	0.851
7329	NM_003345,NM_19	**UBE2I**	ubiquitin conjugating enz	protein_coding	123	129	124	130	1.049
7332	NM_001256355,NM	**UBE2L3**	ubiquitin conjugating enz	protein_coding	143	143	144	144	0.998
7333	gene-UBE2L4	**UBE2L4**	ubiquitin conjugating enz	pseudogene	0	0	1	1	1.458
7334	NM_003348,XR_002	**UBE2N**	ubiquitin conjugating enz	protein_coding	115	107	116	108	0.925
7335	NM_001032288,NM	**UBE2V1**	ubiquitin conjugating enz	protein_coding	324	298	325	299	0.919
7336	NM_003350,XM_01	**UBE2V2**	ubiquitin conjugating enz	protein_coding	75	66	76	67	0.882
7337	NM_000462,NM_00	**UBE3A**	ubiquitin protein ligase E	protein_coding	61	54	62	55	0.882
7339	gene-UBE3AP2	**UBE3AP2**	ubiquitin protein ligase E	pseudogene	0	0	1	1	1.000
7341	NM_001005781,NM	**SUMO1**	small ubiquitin like modif	protein_coding	341	322	342	323	0.945
7342	NM_001128160,NM	**UBP1**	upstream binding protein	protein_coding	71	71	72	72	0.995
7343	NM_001076683,NM	**UBTF**	upstream binding transcr	protein_coding	114	120	115	121	1.055
7345	NM_004181	**UCHL1**	ubiquitin C-terminal hydr	protein_coding	0	0	1	1	1.000
7347	NM_001270952,NM	**UCHL3**	ubiquitin C-terminal hydr	protein_coding	118	111	119	112	0.948
7348	NM_006952	**UPK1B**	uroplakin 1B	protein_coding	0	0	1	1	1.000
7349	NM_003353	**UCN**	urocortin	protein_coding	1	1	2	2	1.085
7350	NM_021833,XM_00	**UCP1**	uncoupling protein 1	protein_coding	0	0	1	1	1.000
7351	NM_001381943,NM	**UCP2**	uncoupling protein 2	protein_coding	77	90	78	91	1.160
7352	NM_003356,NM_02	**UCP3**	uncoupling protein 3	protein_coding	1	1	2	2	0.933
7353	NM_001035247,NM	**UFD1**	ubiquitin recognition fact	protein_coding	75	80	76	81	1.068
7355	NM_001032289,NM	**SLC35A2**	solute carrier family 35 m	protein_coding	33	38	34	39	1.151
7356	NM_003357	**SCGB1A1**	secretoglobin family 1A r	protein_coding	0	0	1	1	1.000
7357	NM_003358,XM_00	**UGCG**	UDP-glucose ceramide g	protein_coding	32	26	33	27	0.828
7358	NM_001184700,NM	**UGDH**	UDP-glucose 6-dehydrog	protein_coding	34	32	35	33	0.950
7360	NM_001001521,NM	**UGP2**	UDP-glucose pyrophosp	protein_coding	135	130	136	131	0.967
7363	NM_001297615,NM	**UGT2B4**	UDP glucuronosyltransfe	protein_coding	0	0	1	1	1.000
7364	NM_001074,NM_00	**UGT2B7**	UDP glucuronosyltransfe	protein_coding	0	0	1	1	1.000
7365	NM_001075,NM_00	**UGT2B10**	UDP glucuronosyltransfe	protein_coding	0	0	1	1	1.000
7366	NM_001076	**UGT2B15**	UDP glucuronosyltransfe	protein_coding	0	0	1	1	1.000
7367	NM_001077,XM_02	**UGT2B17**	UDP glucuronosyltransfe	protein_coding	0	0	1	1	1.000
7368	NM_001128174,NM	**UGT8**	UDP glycosyltransferase	protein_coding	0	0	1	1	1.001
7369	NM_001008389,NM	**UMOD**	uromodulin	protein_coding	0	0	1	1	1.000
7371	NM_001363568,NM	**UCK2**	uridine-cytidine kinase 2	protein_coding	66	63	67	64	0.950
7372	NM_000373,NR_03	**UMPS**	uridine monophosphate s	protein_coding	43	43	44	44	1.003
7373	NM_021110,XM_00	**COL14A1**	collagen type XIV alpha	protein_coding	0	0	1	1	1.000
7374	NM_003362,NM_08	**UNG**	uracil DNA glycosylase	protein_coding	57	61	58	62	1.081
7375	NM_001251877,NM	**USP4**	ubiquitin specific peptida	protein_coding	21	20	22	21	0.935
7376	NM_001256647,NM	**NR1H2**	nuclear receptor subfam	protein_coding	25	28	26	29	1.101
7378	NM_001287426,NM	**UPP1**	uridine phosphorylase 1	protein_coding	85	91	86	92	1.070
7379	NM_006760	**UPK2**	uroplakin 2	protein_coding	0	0	1	1	1.000
7380	NM_001167574,NM	**UPK3A**	uroplakin 3A	protein_coding	0	0	1	1	1.000
7381	NM_001199975,NM	**UQCRB**	ubiquinol-cytochrome c r	protein_coding	83	83	84	84	1.010
7384	NM_003365,XR_002	**UQCRC1**	ubiquinol-cytochrome c r	protein_coding	261	269	262	270	1.031
7385	NM_003366	**UQCRC2**	ubiquinol-cytochrome c r	protein_coding	199	192	200	193	0.966
7386	NM_006003	**UQCRFS1**	ubiquinol-cytochrome c r	protein_coding	108	107	109	108	0.992
7388	NM_001297565,NM	**UQCRH**	ubiquinol-cytochrome c r	protein_coding	936	1,014	937	1,015	1.084
7389	NM_000374,NR_03	**UROD**	uroporphyrinogen decarb	protein_coding	59	67	60	68	1.139
7390	NM_000375,NM_00	**UROS**	uroporphyrinogen III synt	protein_coding	34	33	35	34	0.988
7391	NM_001276373,NM	**USF1**	upstream transcription fa	protein_coding	80	83	81	84	1.036
7392	NM_001321150,NM	**USF2**	upstream transcription fa	protein_coding	70	74	71	75	1.050
7398	NM_001017415,NM	**USP1**	ubiquitin specific peptida	protein_coding	171	152	172	153	0.889
7399	NM_007123,NM_20	**USH2A**	usherin	protein_coding	0	0	1	1	1.000
7401	NM_001195794,NM	**CLRN1**	clarin 1	protein_coding	0	0	1	1	1.000
7402	NM_001375323,NM	**UTRN**	utrophin	protein_coding	21	17	22	18	0.852
7403	NM_001291415,NM	**KDM6A**	lysine demethylase 6A	protein_coding	15	15	16	16	1.020
7404	NM_001258249,NM	**UTY**	ubiquitously transcribed	protein_coding	6	5	7	6	0.868
7405	NM_003369,XM_01	**UVRAG**	UV radiation resistance a	protein_coding	12	10	13	11	0.868
7407	NM_006295,XM_00	**VARS1**	valyl-tRNA synthetase 1	protein_coding	161	150	162	151	0.931
7408	NM_003370,XM_00	**VASP**	vasodilator stimulated ph	protein_coding	190	193	191	194	1.014
7409	NM_001258206,NM	**VAV1**	vav guanine nucleotide e	protein_coding	123	123	124	124	1.004
7410	NM_001134398,NM	**VAV2**	vav guanine nucleotide e	protein_coding	0	0	1	1	1.000
7411	NM_001303543,NM	**VBP1**	VHL binding protein 1	protein_coding	150	132	151	133	0.878
7412	NM_001078,NM_00	**VCAM1**	vascular cell adhesion m	protein_coding	0	0	1	1	1.000
7414	NM_003373,NM_01	**VCL**	vinculin	protein_coding	20	17	21	18	0.827
7415	NM_001354927,NM	**VCP**	valosin containing protein	protein_coding	172	171	173	172	0.994
7416	NM_003374,NR_03	**VDAC1**	voltage dependent anion	protein_coding	629	596	630	597	0.949
7417	NM_001184783,NM	**VDAC2**	voltage dependent anion	protein_coding	244	247	245	248	1.011
7418	gene-VDAC1P4	**VDAC1P4**	voltage dependent anion	pseudogene	0	0	1	1	0.987
7419	NM_001135694,NM	**VDAC3**	voltage dependent anion	protein_coding	444	444	445	445	1.000
7421	NM_000376,NM_00	**VDR**	vitamin D receptor	protein_coding	0	0	1	1	1.040
7422	NM_001025366,NM	**VEGFA**	vascular endothelial grov	protein_coding	0	0	1	1	0.852
7423	NM_001243733,NM	**VEGFB**	vascular endothelial grov	protein_coding	3	3	4	4	0.985
7424	NM_005429	**VEGFC**	vascular endothelial grov	protein_coding	0	0	1	1	1.000
7425	NM_003378,XM_00	**VGF**	VGF nerve growth factor	protein_coding	0	0	1	1	1.262
7428	NM_000551,NM_00	**VHL**	von Hippel-Lindau tumor	protein_coding	62	53	63	54	0.855
7429	NM_007127	**VIL1**	villin 1	protein_coding	0	0	1	1	0.971
7430	NM_001111077,NM	**EZR**	ezrin	protein_coding	167	171	168	172	1.020
7431	NM_003380,XM_00	**VIM**	vimentin	protein_coding	716	682	717	683	0.952
7432	NM_003381,NM_19	**VIP**	vasoactive intestinal pept	protein_coding	0	0	1	1	1.000
7433	NM_001251882,NM	**VIPR1**	vasoactive intestinal pept	protein_coding	0	0	1	1	0.974
7434	NM_001304522,NM	**VIPR2**	vasoactive intestinal pept	protein_coding	0	0	1	1	1.000
7436	NM_001018056,NM	**VLDLR**	very low density lipoprote	protein_coding	0	0	1	1	1.000
7439	NM_001139443,NM	**BEST1**	bestrophin 1	protein_coding	0	0	1	1	0.979
7441	NM_001303509,NM	**VPREB1**	V-set pre-B cell surrogate	protein_coding	0	0	1	1	1.000
7442	NM_018727,NM_08	**TRPV1**	transient receptor potent	protein_coding	0	0	1	1	1.070
7443	NM_003384,XM_00	**VRK1**	VRK serine/threonine kin	protein_coding	88	79	89	80	0.903
7444	NM_001130480,NM	**VRK2**	VRK serine/threonine kin	protein_coding	16	14	17	15	0.926
7447	NM_001366803,NM	**VSNL1**	visinin like 1	protein_coding	0	0	1	1	1.000
7448	NM_000638	**VTN**	vitronectin	protein_coding	0	0	1	1	1.000
7450	NM_000552	**VWF**	von Willebrand factor	protein_coding	0	0	1	1	1.000
7451	gene-VWFP1	**VWFP1**	von Willebrand factor ps	pseudogene	1	0	2	1	0.937
7453	NM_004184,NM_17	**WARS1**	tryptophanyl-tRNA synth	protein_coding	211	214	212	215	1.011
7454	NM_000377,XM_01	**WAS**	WASP actin nucleation p	protein_coding	173	174	174	175	1.006
7455	NM_003386,NM_17	**ZAN**	zonadhesin	protein_coding	0	0	1	1	1.000
7456	NM_001077269,NM	**WIPF1**	WAS/WASL interacting p	protein_coding	607	510	608	511	0.841
7458	NM_022170,NM_03	**EIF4H**	eukaryotic translation init	protein_coding	633	618	634	619	0.976
7461	NM_003388,NM_03	**CLIP2**	CAP-Gly domain contain	protein_coding	0	0	1	1	0.950
7462	NM_014146,NM_03	**LAT2**	linker for activation of T c	protein_coding	29	22	30	23	0.763
7464	NM_003389,NM_05	**CORO2A**	coronin 2A	protein_coding	0	0	1	1	0.950
7465	NM_001143976,NM	**WEE1**	WEE1 G2 checkpoint kin	protein_coding	36	30	37	31	0.857
7466	NM_001145853,NM	**WFS1**	wolframin ER transmemb	protein_coding	13	14	14	15	1.012
7468	NM_001042424,NM	**NSD2**	nuclear receptor binding	protein_coding	129	109	130	110	0.852
7469	NM_005663,XM_01	**NELFA**	negative elongation facto	protein_coding	63	59	64	60	0.929
7471	NM_005430	**WNT1**	Wnt family member 1	protein_coding	0	0	1	1	1.000
7472	NM_003391,NR_02	**WNT2**	Wnt family member 2	protein_coding	0	0	1	1	1.000
7473	NM_030753	**WNT3**	Wnt family member 3	protein_coding	0	0	1	1	1.000
7474	NM_001256105,NM	**WNT5A**	Wnt family member 5A	protein_coding	0	0	1	1	1.000
7475	NM_006522	**WNT6**	Wnt family member 6	protein_coding	0	0	1	1	1.045
7476	NM_004625,XM_01	**WNT7A**	Wnt family member 7A	protein_coding	0	0	1	1	1.000
7477	NM_058238,XM_01	**WNT7B**	Wnt family member 7B	protein_coding	0	0	1	1	1.000
7478	NM_001300938,NM	**WNT8A**	Wnt family member 8A	protein_coding	0	0	1	1	1.000
7479	NM_003393	**WNT8B**	Wnt family member 8B	protein_coding	0	0	1	1	1.000
7480	NM_003394,XM_01	**WNT10B**	Wnt family member 10B	protein_coding	17	18	18	19	1.084
7481	NM_004626,XM_00	**WNT11**	Wnt family member 11	protein_coding	0	0	1	1	1.000
7482	NM_001291880,NM	**WNT2B**	Wnt family member 2B	protein_coding	0	0	1	1	1.000
7483	NM_003395,XM_01	**WNT9A**	Wnt family member 9A	protein_coding	0	0	1	1	1.000
7484	NM_001320458,NM	**WNT9B**	Wnt family member 9B	protein_coding	0	0	1	1	1.000
7485	NM_001146218,NM	**GET1**	guided entry of tail-anch	protein_coding	39	37	40	38	0.932
7486	NM_000553,XM_01	**WRN**	WRN RecQ like helicase	protein_coding	23	20	24	21	0.889
7490	NM_000378,NM_00	**WT1**	WT1 transcription factor	protein_coding	0	0	1	1	1.000
7494	NM_001079539,NM	**XBP1**	X-box binding protein 1	protein_coding	95	86	96	87	0.903

7495	gene-XBP1P1	**XBP1P1**	X-box binding protein 1 p	pseudogene	0	0	1	1	1.006
7498	NM_000379,XM_01	**XDH**	xanthine dehydrogenase	protein_coding	0	0	1	1	1.000
7499	NM_001141919,NM	**XG**	Xg glycoprotein (Xg bloo	protein_coding	0	0	1	1	1.000
7503	NR_001564	**XIST**	X inactive specific transc	lncRNA	0	0	1	1	1.000
7504	NM_021083,XM_01	**XK**	X-linked Kx blood group	protein_coding	15	14	16	15	0.948
7507	NM_000380,NM_00	**XPA**	XPA, DNA damage recog	protein_coding	20	19	21	20	0.949
7508	NM_001354726,NM	**XPC**	XPC complex subunit, D	protein_coding	21	22	22	23	1.071
7511	NM_001167604,NM	**XPNPEP1**	X-prolyl aminopeptidase	protein_coding	47	49	48	50	1.027
7512	NM_003399	**XPNPEP2**	X-prolyl aminopeptidase	protein_coding	0	0	1	1	1.021
7514	NM_003400,XM_00	**XPO1**	exportin 1	protein_coding	246	215	247	216	0.874
7515	NM_006297	**XRCC1**	X-ray repair cross comple	protein_coding	61	60	62	61	0.974
7516	NM_005431	**XRCC2**	X-ray repair cross comple	protein_coding	29	22	30	23	0.767
7517	NM_001100118,NM	**XRCC3**	X-ray repair cross comple	protein_coding	51	51	52	52	0.989
7518	NM_001318012,NM	**XRCC4**	X-ray repair cross comple	protein_coding	22	20	23	21	0.922
7520	NM_021141	**XRCC5**	X-ray repair cross comple	protein_coding	628	574	629	575	0.915
7525	NM_005433,XM_00	**YES1**	YES proto-oncogene 1, S	protein_coding	4	3	5	4	0.837
7526	gene-YES1P1	**YES1P1**	YES1 pseudogene 1	pseudogene	0	0	1	1	1.000
7528	NM_003403	**YY1**	YY1 transcription factor	protein_coding	60	58	61	59	0.977
7529	NM_003404,NM_13	**YWHAB**	tyrosine 3-monooxygena	protein_coding	442	415	443	416	0.938
7531	NM_006761,NR_02	**YWHAE**	tyrosine 3-monooxygena	protein_coding	395	403	396	404	1.021
7532	NM_012479	**YWHAG**	tyrosine 3-monooxygena	protein_coding	277	254	278	255	0.920
7533	NM_003405	**YWHAH**	tyrosine 3-monooxygena	protein_coding	347	357	348	358	1.027
7534	NM_001135699,NM	**YWHAZ**	tyrosine 3-monooxygena	protein_coding	876	823	877	824	0.939
7535	NM_001079,NM_00	**ZAP70**	zeta chain of T cell recep	protein_coding	149	157	150	158	1.055
7536	NM_001178030,NM	**SF1**	splicing factor 1	protein_coding	268	255	269	256	0.949
7538	NM_003407	**ZFP36**	ZFP36 ring finger protein	protein_coding	67	77	68	78	1.152
7539	NM_001282515,NM	**ZFP37**	ZFP37 zinc finger protein	protein_coding	0	0	1	1	1.000
7541	NM_001143823,NM	**ZBTB14**	zinc finger and BTB dom	protein_coding	11	11	12	12	0.981
7542	NM_006782	**ZFPL1**	zinc finger protein like 1	protein_coding	24	30	25	31	1.228
7543	NM_001178084,NM	**ZFX**	zinc finger protein X-link	protein_coding	17	15	18	16	0.889
7544	NM_001145275,NM	**ZFY**	zinc finger protein Y-link	protein_coding	14	11	15	12	0.825
7545	NM_003412	**ZIC1**	Zic family member 1	protein_coding	0	0	1	1	1.000
7546	NM_007129	**ZIC2**	Zic family member 2	protein_coding	0	0	1	1	1.000
7547	NM_001330661,NM	**ZIC3**	Zic family member 3	protein_coding	0	0	1	1	1.000
7549	NM_001017396,NM	**ZNF2**	zinc finger protein 2	protein_coding	5	4	6	5	0.850
7551	NM_001278284,NM	**ZNF3**	zinc finger protein 3	protein_coding	21	21	22	22	1.008
7552	NM_001330574,NM	**ZNF711**	zinc finger protein 711	protein_coding	0	0	1	1	1.000
7553	NM_001282795,NM	**ZNF7**	zinc finger protein 7	protein_coding	9	10	10	11	1.078
7554	NM_021089	**ZNF8**	zinc finger protein 8	protein_coding	1	1	2	2	0.979
7555	NM_001127192,NM	**CNBP**	CCHC-type zinc finger nu	protein_coding	457	438	458	439	0.959
7556	NM_015394	**ZNF10**	zinc finger protein 10	protein_coding	0	0	1	1	1.000
7559	NM_006956,NM_01	**ZNF12**	zinc finger protein 12	protein_coding	12	10	13	11	0.872
7561	NM_021030	**ZNF14**	zinc finger protein 14	protein_coding	1	1	2	2	0.967
7562	NM_001297560,NM	**ZNF708**	zinc finger protein 708	protein_coding	0	0	1	1	1.000
7564	NM_001029976,NM	**ZNF16**	zinc finger protein 16	protein_coding	2	4	3	5	1.301
7565	NM_001330617,NM	**ZNF17**	zinc finger protein 17	protein_coding	10	11	11	12	1.099
7566	NM_001303281,NM	**ZNF18**	zinc finger protein 18	protein_coding	4	4	5	5	0.931
7567	NM_006961	**ZNF19**	zinc finger protein 19	protein_coding	1	1	2	2	0.986
7568	NM_001203250,NM	**ZNF20**	zinc finger protein 20	protein_coding	3	2	4	3	0.771
7569	NM_001007088,NM	**ZNF182**	zinc finger protein 182	protein_coding	12	12	13	13	0.954
7570	NM_006963	**ZNF22**	zinc finger protein 22	protein_coding	139	123	140	124	0.885
7571	NM_001304492,NM	**ZNF23**	zinc finger protein 23	protein_coding	12	13	13	14	1.054
7572	NM_001308123,NM	**ZNF24**	zinc finger protein 24	protein_coding	42	36	43	37	0.849
7574	NM_001256279,NM	**ZNF26**	zinc finger protein 26	protein_coding	0	0	1	1	1.151
7576	NM_001369761,NM	**ZNF28**	zinc finger protein 28	protein_coding	7	6	8	7	0.889
7579	NM_001377376,NM	**ZSCAN20**	zinc finger and SCAN do	protein_coding	2	2	3	3	1.011
7580	NM_001005368,NM	**ZNF32**	zinc finger protein 32	protein_coding	0	0	1	1	1.000
7581	NM_001278170,NM	**ZNF33A**	zinc finger protein 33A	protein_coding	7	6	8	7	0.888
7582	NM_001305033,NM	**ZNF33B**	zinc finger protein 33B	protein_coding	12	11	13	12	0.962
7584	NM_003420	**ZNF35**	zinc finger protein 35	protein_coding	16	15	17	16	0.941
7586	NM_001287054,NM	**ZKSCAN1**	zinc finger with KRAB an	protein_coding	20	17	21	18	0.862
7587	NM_001007094,NM	**ZNF37A**	zinc finger protein 37A	protein_coding	8	7	9	8	0.858
7589	NM_001362779,NM	**ZSCAN21**	zinc finger and SCAN do	protein_coding	12	11	13	12	0.882
7592	NM_001324139,NM	**ZNF41**	zinc finger protein 41	protein_coding	16	14	17	15	0.885
7593	NM_001267033,NM	**MZF1**	myeloid zinc finger 1	protein_coding	5	4	6	5	0.941
7594	NM_001256648,NM	**ZNF43**	zinc finger protein 43	protein_coding	0	0	1	1	1.000
7596	NM_003425,XM_01	**ZNF45**	zinc finger protein 45	protein_coding	14	12	15	13	0.873
7597	NM_001304507,NM	**ZBTB25**	zinc finger and BTB dom	protein_coding	5	5	6	6	0.896
7608	NM_001355194	**ZNF56**	zinc finger protein 56	protein_coding	1	1	2	2	0.908
7617	NM_001355197	**ZNF66**	zinc finger protein 66	protein_coding	0	0	1	1	1.000
7618	gene-ZNF726P1	**ZNF726P1**	zinc finger protein 726 ps	pseudogene	0	0	1	1	1.000
7620	NM_001321163,NM	**ZNF69**	zinc finger protein 69	protein_coding	0	0	1	1	1.000
7621	NM_021916,XM_02	**ZNF70**	zinc finger protein 70	protein_coding	3	2	4	3	0.655
7625	NM_001256523,NM	**ZNF74**	zinc finger protein 74	protein_coding	10	10	11	11	1.070
7626	NM_001185063,NM	**ZNF75D**	zinc finger protein 75D	protein_coding	4	3	5	4	0.909
7627	NM_001302109,NM	**ZNF75A**	zinc finger protein 75a	protein_coding	1	1	2	2	0.917
7628	gene-ZNF75BP	**ZNF75BP**	zinc finger protein 75B, p	pseudogene	0	0	1	1	1.000
7629	NM_001292032,NM	**ZNF76**	zinc finger protein 76	protein_coding	15	15	16	16	0.961
7633	NM_001286696,NM	**ZNF79**	zinc finger protein 79	protein_coding	5	6	6	7	1.120
7634	NM_007136	**ZNF80**	zinc finger protein 80	protein_coding	0	0	1	1	1.000
7637	NM_001127372,NM	**ZNF84**	zinc finger protein 84	protein_coding	1	1	2	2	0.899
7638	NM_001297588,NM	**ZNF221**	zinc finger protein 221	protein_coding	0	0	1	1	1.000
7639	NM_001256171,NM	**ZNF85**	zinc finger protein 85	protein_coding	0	0	1	1	1.000
7643	NM_007138,XR_00	**ZNF90**	zinc finger protein 90	protein_coding	0	0	1	1	1.000
7644	NM_001300951,NM	**ZNF91**	zinc finger protein 91	protein_coding	0	0	1	1	1.000
7652	NM_001080409	**ZNF99**	zinc finger protein 99	protein_coding	0	0	1	1	1.000
7661	gene-ZNF90P1	**ZNF90P1**	zinc finger protein 90 pse	pseudogene	0	0	1	1	1.000
7673	NM_001129996,NM	**ZNF222**	zinc finger protein 222	protein_coding	1	1	2	2	1.228
7675	NM_001008727,NM	**ZNF121**	zinc finger protein 121	protein_coding	26	22	27	23	0.854
7678	NM_001243740,NM	**ZNF124**	zinc finger protein 124	protein_coding	12	10	13	11	0.863
7681	NM_005664	**MKRN3**	makorin ring finger prote	protein_coding	0	0	1	1	1.021
7682	gene-MKRN4P	**MKRN4P**	makorin ring finger prote	pseudogene	0	0	1	1	1.000
7683	gene-MKRN5P	**MKRN5P**	makorin ring finger prote	pseudogene	0	0	1	1	1.032
7686	NR_026640	**MKRN7P**	makorin ring finger prote	transcribed_pseu	0	0	1	1	1.000
7690	NM_001297548,NM	**ZNF131**	zinc finger protein 131	protein_coding	44	41	45	42	0.942
7691	NM_003433	**ZNF132**	zinc finger protein 132	protein_coding	7	6	8	7	0.914
7692	NM_001083330,NM	**ZNF133**	zinc finger protein 133	protein_coding	3	4	4	5	1.034
7693	NM_003435	**ZNF134**	zinc finger protein 134	protein_coding	9	9	10	10	1.044
7694	NM_001164527,NM	**ZNF135**	zinc finger protein 135	protein_coding	0	0	1	1	1.000
7695	NM_001348013,NM	**ZNF136**	zinc finger protein 136	protein_coding	10	9	11	10	0.904
7696	NR_023311	**ZNF137P**	zinc finger protein 137, p	transcribed_pseu	0	0	1	1	1.000
7697	NM_001160183,NM	**ZNF138**	zinc finger protein 138	protein_coding	21	19	22	20	0.903
7699	NM_001300776,NM	**ZNF140**	zinc finger protein 140	protein_coding	0	0	1	1	1.000
7700	NM_001348277,NM	**ZNF141**	zinc finger protein 141	protein_coding	0	0	1	1	1.000
7701	NM_001105537,NM	**ZNF142**	zinc finger protein 142	protein_coding	32	31	33	32	0.966
7702	NM_001282656,NM	**ZNF143**	zinc finger protein 143	protein_coding	19	17	20	18	0.885
7703	NM_001369614,NM	**PCGF2**	polycomb group ring fing	protein_coding	5	3	6	4	0.764
7704	NM_001018011,NM	**ZBTB16**	zinc finger and BTB dom	protein_coding	0	0	1	1	1.000
7705	NM_001099638,NM	**ZNF146**	zinc finger protein 146	protein_coding	121	105	122	106	0.863
7706	NM_005082	**TRIM25**	tripartite motif containing	protein_coding	61	49	62	50	0.806
7707	NM_001348424,NM	**ZNF148**	zinc finger protein 148	protein_coding	25	20	26	21	0.803
7709	NM_001242884,NM	**ZBTB17**	zinc finger and BTB dom	protein_coding	17	16	18	17	0.941
7710	NM_001085384,NR	**ZNF154**	zinc finger protein 154	protein_coding	0	0	1	1	0.971
7711	NM_001260486,NM	**ZNF155**	zinc finger protein 155	protein_coding	0	0	1	1	1.000
7712	NM_003446	**ZNF157**	zinc finger protein 157	protein_coding	0	0	1	1	1.000
7716	NM_001330393,NM	**VEZF1**	vascular endothelial zinc	protein_coding	21	18	22	19	0.866
7718	NM_001376491,NM	**ZNF165**	zinc finger protein 165	protein_coding	1	1	2	2	1.040
7726	NM_001242783,NM	**TRIM26**	tripartite motif containing	protein_coding	114	115	115	116	1.006
7727	NM_001032292,NM	**ZNF174**	zinc finger protein 174	protein_coding	28	24	29	25	0.878
7728	NM_007147,NR_13	**ZNF175**	zinc finger protein 175	protein_coding	10	9	11	10	0.922
7730	NM_001172651,NM	**ZNF177**	zinc finger protein 177	protein_coding	1	0	2	1	0.667
7732	NM_007148,XM_00	**RNF112**	ring finger protein 112	protein_coding	0	0	1	1	1.040
7733	NM_001278508,NM	**ZNF180**	zinc finger protein 180	protein_coding	7	5	8	6	0.836
7737	NM_006978	**RNF113A**	ring finger protein 113A	protein_coding	32	29	33	30	0.926
7738	NM_001318891,NM	**ZNF184**	zinc finger protein 184	protein_coding	11	10	12	11	0.887
7739	NM_001178106,NM	**ZNF185**	zinc finger protein 185 w	protein_coding	1	1	2	2	1.088

7741	NM_001023560,NM	**ZSCAN26**	zinc finger and SCAN do	protein_coding	10	11	11	12	1.051
7743	NM_001278231,NM	**ZNF189**	zinc finger protein 189	protein_coding	10	10	11	11	0.989
7745	NM_001278119,NM	**ZKSCAN8**	zinc finger with KRAB an	protein_coding	26	21	27	22	0.806
7746	NM_001199479,NM	**ZSCAN9**	zinc finger and SCAN do	protein_coding	18	18	19	19	0.994
7748	NM_001130519,NM	**ZNF195**	zinc finger protein 195	protein_coding	22	21	23	22	0.942
7750	NM_001190964,NM	**ZMYM2**	zinc finger MYM-type cor	protein_coding	30	31	31	32	1.057
7752	NM_001145446,NM	**ZNF200**	zinc finger protein 200	protein_coding	38	38	39	39	0.979
7753	NM_001301779,NM	**ZNF202**	zinc finger protein 202	protein_coding	15	13	16	14	0.854
7754	NR_002722,NR_02	**ZNF204P**	zinc finger protein 204, p	transcribed_pseu	0	0	1	1	1.000
7755	NM_001042428,NM	**ZNF205**	zinc finger protein 205	protein_coding	0	0	1	1	1.000
7756	NM_001032293,NM	**ZNF207**	zinc finger protein 207	protein_coding	168	153	169	154	0.912
7757	NM_001329971,NM	**ZNF208**	zinc finger protein 208	protein_coding	0	0	1	1	1.000
7760	NM_001134655,NM	**ZNF213**	zinc finger protein 213	protein_coding	19	21	20	22	1.142
7761	NM_001354830,NM	**ZNF214**	zinc finger protein 214	protein_coding	0	0	1	1	1.000
7762	NM_001354853,NM	**ZNF215**	zinc finger protein 215	protein_coding	0	0	1	1	1.000
7763	NM_001102420,NM	**ZFAND5**	zinc finger AN1-type con	protein_coding	23	25	24	26	1.066
7764	NM_006526,XM_00	**ZNF217**	zinc finger protein 217	protein_coding	22	18	23	19	0.822
7766	NM_013361,XM_01	**ZNF223**	zinc finger protein 223	protein_coding	0	0	1	1	1.000
7767	NM_001321645,NM	**ZNF224**	zinc finger protein 224	protein_coding	6	5	7	6	0.905
7768	NM_001321685,NM	**ZNF225**	zinc finger protein 225	protein_coding	2	2	3	3	0.917
7769	NM_001032372,NM	**ZNF226**	zinc finger protein 226	protein_coding	6	5	7	6	0.899
7770	NM_001289166,NM	**ZNF227**	zinc finger protein 227	protein_coding	6	6	7	7	0.924
7771	NM_001083335,NM	**ZNF112**	zinc finger protein 112	protein_coding	0	0	1	1	1.000
7772	NM_001278510,NM	**ZNF229**	zinc finger protein 229	protein_coding	0	0	1	1	1.000
7773	NM_006300	**ZNF230**	zinc finger protein 230	protein_coding	1	0	2	1	0.845
7775	NM_001320952,NM	**ZNF232**	zinc finger protein 232	protein_coding	18	15	19	16	0.815
7776	NM_001306089,NM	**ZNF236**	zinc finger protein 236	protein_coding	4	4	5	5	0.939
7779	NM_021194	**SLC30A1**	solute carrier family 30 m	protein_coding	24	24	25	25	1.006
7780	NM_001004434,NM	**SLC30A2**	solute carrier family 30 m	protein_coding	0	0	1	1	1.000
7781	NM_001318949,NM	**SLC30A3**	solute carrier family 30 m	protein_coding	0	0	1	1	1.000
7782	NM_001321036,NM	**SLC30A4**	solute carrier family 30 m	protein_coding	3	3	4	4	0.987
7783	NM_001376231,NM	**ZP2**	zona pellucida glycoprot	protein_coding	0	0	1	1	1.000
7784	NM_001110354,NM	**ZP3**	zona pellucida glycoprot	protein_coding	1	1	2	2	1.078
7786	NM_001193511,NM	**MAP3K12**	mitogen-activated protei	protein_coding	6	6	7	7	0.958
7789	NM_007156	**ZXDA**	zinc finger X-linked dupli	protein_coding	0	0	1	1	0.986
7791	NM_001010972,NM	**ZYX**	zyxin	protein_coding	214	208	215	209	0.972
7798	NM_001142546,NM	**LUZP1**	leucine zipper protein 1	protein_coding	14	11	15	12	0.775
7799	NM_001007257,NM	**PRDM2**	PR/SET domain 2	protein_coding	41	39	42	40	0.957
7802	NM_003462	**DNALI1**	dynein axonemal light in	protein_coding	0	0	1	1	1.000
7803	NM_003463,XM_01	**PTP4A1**	protein tyrosine phospha	protein_coding	114	110	115	111	0.965
7804	NM_001018054,NM	**LRP8**	LDL receptor related pro	protein_coding	53	44	54	45	0.838
7805	NM_006762,XM_01	**LAPTM5**	lysosomal protein transm	protein_coding	96	86	97	87	0.891
7809	NM_057176	**BSND**	barttin CLCNK type acce	protein_coding	0	0	1	1	1.000
7812	NM_001007553,NM	**CSDE1**	cold shock domain conta	protein_coding	353	330	354	331	0.935
7813	NM_001308248,NM	**EVI5**	ecotropic viral integration	protein_coding	0	0	1	1	1.000
7818	NM_001199849,NM	**DAP3**	death associated protein	protein_coding	210	192	211	193	0.913
7827	NM_001297575,NM	**NPHS2**	NPHS2 stomatin family m	protein_coding	0	0	1	1	1.000
7832	NM_006763	**BTG2**	BTG anti-proliferation fac	protein_coding	22	21	23	22	0.963
7837	NM_012293,XM_00	**PXDN**	peroxidasin	protein_coding	0	0	1	1	1.000
7840	NM_001378454,NM	**ALMS1**	ALMS1 centrosome and	protein_coding	10	8	11	9	0.857
7841	NM_001146158,NM	**MOGS**	mannosyl-oligosacchario	protein_coding	84	84	85	85	1.003
7844	NM_001198951,NM	**RNF103**	ring finger protein 103	protein_coding	7	7	8	8	1.021
7845	gene-PAFAH1B1P1	**PAFAH1B1P1**	platelet activating factor a	pseudogene	0	0	1	1	1.000
7846	NM_001270399,NM	**TUBA1A**	tubulin alpha 1a	protein_coding	284	308	285	309	1.084
7849	NM_003466,NM_01	**PAX8**	paired box 8	protein_coding	2	2	3	3	1.290
7850	NM_001261419,NM	**IL1R2**	interleukin 1 receptor typ	protein_coding	0	0	1	1	0.976
7851	NM_001371559,NM	**MALL**	mal, T cell differentiation	protein_coding	0	0	1	1	1.000
7852	NM_001008540,NM	**CXCR4**	C-X-C motif chemokine r	protein_coding	239	245	240	246	1.026
7855	NM_003468,XM_02	**FZD5**	frizzled class receptor 5	protein_coding	1	0	2	1	0.765
7857	NM_003469	**SCG2**	secretogranin II	protein_coding	0	0	1	1	1.002
7862	NM_001003694,NM	**BRPF1**	bromodomain and PHD f	protein_coding	24	25	25	26	1.036
7866	NM_006764	**IFRD2**	interferon related develop	protein_coding	93	102	94	103	1.096
7867	NM_001243925,NM	**MAPKAPK3**	MAPK activated protein k	protein_coding	164	165	165	166	1.003
7869	NM_001005914,NM	**SEMA3B**	semaphorin 3B	protein_coding	0	0	1	1	1.000
7871	NM_001304420,NM	**SLMAP**	sarcolemma associated p	protein_coding	18	17	19	18	0.950
7873	NM_006010	**MANF**	mesencephalic astrocyte	protein_coding	353	358	354	359	1.015
7874	NM_001286457,NM	**USP7**	ubiquitin specific peptida	protein_coding	124	111	125	112	0.900
7879	NM_004637	**RAB7A**	RAB7A, member RAS on	protein_coding	268	268	269	269	1.001
7881	NM_001308217,NM	**KCNAB1**	potassium voltage-gated	protein_coding	0	0	1	1	0.985
7884	NM_001306074,NM	**SLBP**	stem-loop binding protein	protein_coding	99	130	100	131	1.318
7903	NM_005668,NM_17	**ST8SIA4**	ST8 alpha-N-acetyl-neur	protein_coding	36	28	37	29	0.777
7905	NM_005669,XM_01	**REEP5**	receptor accessory prote	protein_coding	113	106	114	107	0.939
7913	NM_001134709,NM	**DEK**	DEK proto-oncogene	protein_coding	554	471	555	472	0.851
7915	NM_001080,NM_00	**ALDH5A1**	aldehyde dehydrogenase	protein_coding	0	0	1	1	1.000
7916	NM_004638,NM_08	**PRRC2A**	proline rich coiled-coil 2A	protein_coding	258	238	259	239	0.925
7917	NM_001098534,NM	**BAG6**	BAG cochaperone 6	protein_coding	144	137	145	138	0.950
7918	NM_001199237,NM	**GPANK1**	G-patch domain and ank	protein_coding	50	44	51	45	0.884
7919	NM_004640,NM_08	**DDX39B**	DExD-box helicase 39B	protein_coding	296	302	297	303	1.019
7920	NM_001177515,NM	**ABHD16A**	abhydrolase domain con	protein_coding	55	50	56	51	0.908
7922	NM_001077516,NM	**SLC39A7**	solute carrier family 39 m	protein_coding	171	169	172	170	0.994
7923	NM_014234	**HSD17B8**	hydroxysteroid 17-beta d	protein_coding	8	9	9	10	1.118
7932	NM_007160,XM_01	**OR2H2**	olfactory receptor family	protein_coding	0	0	1	1	1.000
7936	NM_002904,XM_00	**NELFE**	negative elongation facto	protein_coding	141	137	142	138	0.973
7940	NM_001166538,NM	**LST1**	leukocyte specific transc	protein_coding	45	47	46	48	1.056
7941	NM_001168357,NM	**PLA2G7**	phospholipase A2 group	protein_coding	0	0	1	1	1.000
7942	NM_001167827,NM	**TFEB**	transcription factor EB	protein_coding	2	1	3	2	0.729
7955	NR_026876	**RNF217-AS1**	RNF217 antisense RNA	lncRNA	0	0	1	1	1.000
7957	NM_001018041,NM	**EPM2A**	EPM2A glucan phosphat	protein_coding	3	3	4	4	0.988
7965	NM_001326606,NM	**AIMP2**	aminoacyl tRNA syntheta	protein_coding	89	96	90	97	1.088
7975	NM_002360,XM_00	**MAFK**	MAF bZIP transcription fa	protein_coding	29	29	30	30	0.989
7976	NM_017412,NM_14	**FZD3**	frizzled class receptor 3	protein_coding	0	0	1	1	1.000
7978	NM_001301134,NM	**MTERF1**	mitochondrial transcriptio	protein_coding	19	21	20	22	1.125
7979	NM_006304,NR_03	**SEM1**	SEM1 26S proteasome c	protein_coding	361	359	362	360	0.994
7980	NM_001271003,NM	**TFPI2**	tissue factor pathway inh	protein_coding	0	0	1	1	1.000
7982	NM_001369598,NM	**ST7**	suppression of tumoriger	protein_coding	4	3	5	4	0.784
7984	NM_005435,XM_01	**ARHGEF5**	Rho guanine nucleotide	protein_coding	0	0	1	1	1.000
7988	NM_012256	**ZNF212**	zinc finger protein 212	protein_coding	12	12	13	13	1.000
7991	NM_001356429,NM	**TUSC3**	tumor suppressor candid	protein_coding	0	0	1	1	1.000
7993	NM_001282189,NM	**UBXN8**	UBX domain protein 8	protein_coding	36	36	37	37	1.001
7994	NM_001305878,NM	**KAT6A**	lysine acetyltransferase 6	protein_coding	44	36	45	37	0.820
8000	NM_005672,NR_03	**PSCA**	prostate stem cell antige	protein_coding	0	0	1	1	1.000
8001	NM_001042543,NM	**GLRA3**	glycine receptor alpha 3	protein_coding	0	0	1	1	1.012
8013	NM_006981,NM_17	**NR4A3**	nuclear receptor subfam	protein_coding	1	1	2	2	1.003
8019	NM_007371,XM_00	**BRD3**	bromodomain containing	protein_coding	11	10	12	11	0.891
8021	NM_001318324,NM	**NUP214**	nucleoporin 214	protein_coding	26	25	27	26	0.970
8022	NM_001363746,NM	**LHX3**	LIM homeobox 3	protein_coding	0	0	1	1	0.950
8027	NM_001324282,NM	**STAM**	signal transducing adapt	protein_coding	2	3	3	4	1.022
8028	NM_001195626,NM	**MLLT10**	MLLT10 histone lysine m	protein_coding	8	6	9	7	0.852
8029	NM_001081,XM_01	**CUBN**	cubilin	protein_coding	0	0	1	1	1.049
8030	NM_005436	**CCDC6**	coiled-coil domain contai	protein_coding	25	24	26	25	0.972
8031	NM_001145260,NM	**NCOA4**	nuclear receptor coactiva	protein_coding	147	134	148	135	0.914
8034	NM_001324312,NM	**SLC25A16**	solute carrier family 25 m	protein_coding	6	5	7	6	0.939
8036	NM_001269039,NM	**SHOC2**	SHOC2 leucine rich repe	protein_coding	32	27	33	28	0.849
8038	NM_001288973,NM	**ADAM12**	ADAM metallopeptidase	protein_coding	0	0	1	1	1.000
8045	NM_001143993,NM	**RASSF7**	Ras association domain	protein_coding	15	15	16	16	0.948
8048	NM_001369404,NM	**CSRP3**	cysteine and glycine rich	protein_coding	0	0	1	1	1.000
8050	NM_001135024,NM	**PDHX**	pyruvate dehydrogenase	protein_coding	57	50	58	51	0.872
8061	NM_001300844,NM	**FOSL1**	FOS like 1, AP-1 transcri	protein_coding	0	0	1	1	1.000
8065	NM_003478,XM_00	**CUL5**	cullin 5	protein_coding	23	19	24	20	0.841
8073	NM_001195100,NM	**PTP4A2**	protein tyrosine phospha	protein_coding	302	286	303	287	0.948
8074	NM_020638	**FGF23**	fibroblast growth factor 2	protein_coding	0	0	1	1	1.000
8076	NM_001297709,NM	**MFAP5**	microfibril associated pro	protein_coding	0	0	1	1	1.000
8078	NM_001098536,NM	**USP5**	ubiquitin specific peptida	protein_coding	53	57	54	58	1.071
8079	NM_001382225,NM	**MLF2**	myeloid leukemia factor 2	protein_coding	121	126	122	127	1.037
8082	NM_001135823,NM	**SSPN**	sarcospan	protein_coding	0	0	1	1	1.000
8085	NM_003482,XM_00	**KMT2D**	lysine methyltransferase	protein_coding	30	24	31	25	0.803

8086	NM_001173466,NM	**AAAS**	aladin WD repeat nucleo	protein_coding	57	61	58	62	1.061
8087	NM_001013438,NM	**FXR1**	FMR1 autosomal homolo	protein_coding	119	112	120	113	0.947
8089	NM_001300950,NM	**YEATS4**	YEATS domain containin	protein_coding	42	36	43	37	0.860
8091	NM_001300918,NM	**HMGA2**	high mobility group AT-h	protein_coding	0	0	1	1	1.000
8092	NM_006982,XM_01	**ALX1**	ALX homeobox 1	protein_coding	0	0	1	1	1.000
8099	NM_001270433,NM	**CDK2AP1**	cyclin dependent kinase	protein_coding	26	25	27	26	0.991
8100	NM_001318491,NM	**IFT88**	intraflagellar transport 88	protein_coding	10	9	11	10	0.867
8106	NM_001360551,NM	**PABPN1**	poly(A) binding protein n	protein_coding	164	194	165	195	1.183
8110	NM_001280542,NM	**DPF3**	double PHD fingers 3	protein_coding	13	12	14	13	0.872
8111	NM_001177676,NM	**GPR68**	G protein-coupled recept	protein_coding	34	34	35	35	1.015
8115	NM_001098725,NM	**TCL1A**	TCL1 family AKT coactiva	protein_coding	0	0	1	1	1.000
8120	NM_001278511,NM	**AP3B2**	adaptor related protein c	protein_coding	0	0	1	1	1.000
8123	NR_022008	**PWAR5**	Prader Willi/Angelman re	lncRNA	3	2	4	3	0.721
8125	NM_006305	**ANP32A**	acidic nuclear phosphop	protein_coding	520	524	521	525	1.006
8128	NM_001330416,NM	**ST8SIA2**	ST8 alpha-N-acetyl-neur	protein_coding	0	0	1	1	1.000
8131	NM_001039476,NM	**NPRL3**	NPR3 like, GATOR1 com	protein_coding	37	41	38	42	1.098
8139	NM_001377486,NM	**GAN**	gigaxonin	protein_coding	4	4	5	5	0.873
8140	NM_003486,XM_00	**SLC7A5**	solute carrier family 7 me	protein_coding	92	84	93	85	0.914
8148	NM_003487,NM_13	**TAF15**	TATA-box binding protein	protein_coding	629	658	630	659	1.044
8153	NM_005440,XM_01	**RND2**	Rho family GTPase 2	protein_coding	0	0	1	1	1.000
8161	NM_004645	**COIL**	coilin	protein_coding	36	36	37	37	0.981
8165	NM_001242902,NM	**AKAP1**	A-kinase anchoring prote	protein_coding	66	65	67	66	0.986
8170	NM_001242692,NM	**SLC14A2**	solute carrier family 14 m	protein_coding	5	5	6	6	1.043
8174	NM_130760,NM_13	**MADCAM1**	mucosal vascular addres	protein_coding	0	0	1	1	1.000
8175	NM_007165	**SF3A2**	splicing factor 3a subuni	protein_coding	207	210	208	211	1.013
8178	NM_006532,XM_01	**ELL**	elongation factor for RNA	protein_coding	27	26	28	27	0.958
8187	NM_001099282,NM	**ZNF239**	zinc finger protein 239	protein_coding	0	0	1	1	1.000
8189	NM_004819,XM_00	**SYMPK**	symplekin	protein_coding	71	69	72	70	0.974
8190	NM_001202553,NM	**MIA**	MIA SH3 domain contain	protein_coding	0	0	1	1	1.249
8192	NM_006012	**CLPP**	caseinolytic mitochondria	protein_coding	58	62	59	63	1.071
8193	NM_001135155,NM	**DPF1**	double PHD fingers 1	protein_coding	0	0	1	1	1.000
8195	NM_018848,NM_17	**MKKS**	McKusick-Kaufman synd	protein_coding	9	9	10	10	0.976
8200	NM_000557,NM_00	**GDF5**	growth differentiation fac	protein_coding	0	0	1	1	0.967
8202	NM_001174087,NM	**NCOA3**	nuclear receptor coactiva	protein_coding	37	29	38	30	0.793
8204	NM_003489,XM_00	**NRIP1**	nuclear receptor interacti	protein_coding	0	0	1	1	1.000
8208	NM_005441,XM_01	**CHAF1B**	chromatin assembly facto	protein_coding	22	20	23	21	0.906
8209	NM_001320383,NM	**GATD3A**	glutamine amidotransfera	protein_coding	56	60	57	61	1.069
8214	NM_005675,XM_01	**DGCR6**	DiGeorge syndrome critic	protein_coding	1	1	2	2	1.148
8215	gene-DVL1P1	**DVL1P1**	dishevelled segment pola	pseudogene	0	0	1	1	1.000
8216	NM_006767	**LZTR1**	leucine zipper like transc	protein_coding	15	14	16	15	0.957
8218	NM_001835,NM_00	**CLTCL1**	clathrin heavy chain like	protein_coding	0	0	1	1	1.000
8220	NM_022719,NR_13	**ESS2**	ess-2 splicing factor hom	protein_coding	17	15	18	16	0.879
8224	NM_001135774,NM	**SYN3**	synapsin III	protein_coding	0	0	1	1	1.000
8225	NM_012227,NM_01	**GTPBP6**	GTP binding protein 6 (p	protein_coding	29	31	30	32	1.069
8226	NM_001135565,NM	**PUDP**	pseudouridine 5'-phosph	protein_coding	49	48	50	49	0.980
8227	NM_005088,NM_00	**AKAP17A**	A-kinase anchoring prote	protein_coding	27	31	28	32	1.137
8228	NM_001142389,NM	**PNPLA4**	patatin like phospholipas	protein_coding	6	8	7	9	1.210
8233	NM_005089,XM_00	**ZRSR2**	zinc finger CCCH-type, F	protein_coding	21	17	22	18	0.794
8237	NM_001371072,XM	**USP11**	ubiquitin specific peptida	protein_coding	66	69	67	70	1.045
8239	NM_001039590,NM	**USP9X**	ubiquitin specific peptida	protein_coding	34	28	35	29	0.823
8241	NM_001204466,NM	**RBM10**	RNA binding motif protei	protein_coding	88	87	89	88	0.991
8242	NM_001146702,NM	**KDM5C**	lysine demethylase 5C	protein_coding	59	56	60	57	0.953
8243	NM_001281463,NM	**SMC1A**	structural maintenance o	protein_coding	84	78	85	79	0.934
8251	gene-HNRNPDP1	**HNRNPDP1**	heterogeneous nuclear r	pseudogene	0	0	1	1	1.000
8260	NM_001256119,NM	**NAA10**	N-alpha-acetyltransferas	protein_coding	106	100	107	101	0.943
8263	NM_012151	**F8A1**	coagulation factor VIII as	protein_coding	21	23	22	24	1.118
8266	NM_014235	**UBL4A**	ubiquitin like 4A	protein_coding	98	99	99	100	1.008
8269	NM_003492,XM_01	**TMEM187**	transmembrane protein 1	protein_coding	2	2	3	3	0.978
8270	NM_006014	**LAGE3**	L antigen family member	protein_coding	52	56	53	57	1.087
8273	NM_001142391,NM	**SLC10A3**	solute carrier family 10 m	protein_coding	45	50	46	51	1.116
8277	NM_001145933,NM	**TKTL1**	transketolase like 1	protein_coding	0	0	1	1	0.885
8284	NM_001146705,NM	**KDM5D**	lysine demethylase 5D	protein_coding	22	19	23	20	0.864
8287	NM_004654,XM_01	**USP9Y**	ubiquitin specific peptida	protein_coding	9	8	10	9	0.876
8288	NM_000502	**EPX**	eosinophil peroxidase	protein_coding	0	0	1	1	1.000
8289	NM_006015,NM_13	**ARID1A**	AT-rich interaction domai	protein_coding	78	72	79	73	0.922
8290	NM_003493	**H3-4**	H3.4 histone	protein_coding	0	0	1	1	1.000
8291	NM_001130455,NM	**DYSF**	dysferlin	protein_coding	34	30	35	31	0.894
8292	NM_005677,NM_08	**COLQ**	collagen like tail subunit	protein_coding	1	1	2	2	1.281
8293	NM_021967,NM_02	**SERF1A**	small EDRK-rich factor 1	protein_coding	20	21	21	22	1.011
8294	NM_003495	**H4C9**	H4 clustered histone 9	protein_coding	9	14	10	15	1.490
8295	NM_001244580,NM	**TRRAP**	transformation/transcripti	protein_coding	39	31	40	32	0.798
8301	NM_001008660,NM	**PICALM**	phosphatidylinositol bind	protein_coding	107	94	108	95	0.878
8302	NM_013431	**KLRC4**	killer cell lectin like recep	protein_coding	2	1	3	2	0.733
8303	NM_003498,XM_01	**SNN**	stannin	protein_coding	3	3	4	4	1.043
8309	NM_003500,XM_00	**ACOX2**	acyl-CoA oxidase 2	protein_coding	0	0	1	1	1.076
8310	NM_001101667,NM	**ACOX3**	acyl-CoA oxidase 3, prist	protein_coding	5	5	6	6	1.043
8312	NM_003502,NM_18	**AXIN1**	axin 1	protein_coding	92	90	93	91	0.984
8313	NM_001363813,NM	**AXIN2**	axin 2	protein_coding	0	0	1	1	1.000
8314	NM_004656,XM_01	**BAP1**	BRCA1 associated protei	protein_coding	61	60	62	61	0.990
8315	NM_006768,XM_00	**BRAP**	BRCA1 associated protei	protein_coding	24	24	25	25	0.965
8317	NM_001134419,NM	**CDC7**	cell division cycle 7	protein_coding	63	56	64	57	0.894
8318	NM_001178010,NM	**CDC45**	cell division cycle 45	protein_coding	73	73	74	74	1.006
8320	NM_001278182,NM	**EOMES**	eomesodermin	protein_coding	84	79	85	80	0.944
8321	NM_003505	**FZD1**	frizzled class receptor 1	protein_coding	0	0	1	1	0.986
8322	NM_012193	**FZD4**	frizzled class receptor 4	protein_coding	0	0	1	1	0.979
8323	NM_001164615,NM	**FZD6**	frizzled class receptor 6	protein_coding	0	0	1	1	1.000
8324	NM_003507	**FZD7**	frizzled class receptor 7	protein_coding	0	0	1	1	1.000
8325	NM_031866	**FZD8**	frizzled class receptor 8	protein_coding	0	0	1	1	1.000
8326	NM_003508	**FZD9**	frizzled class receptor 9	protein_coding	0	0	1	1	1.000
8327	gene-GABPAP	**GABPAP**	GABPA pseudogene	pseudogene	0	0	1	1	0.889
8328	NM_001135031,NM	**GFI1B**	growth factor independen	protein_coding	1	1	2	2	1.096
8329	NM_003509	**H2AC13**	H2A clustered histone 13	protein_coding	4	6	5	7	1.342
8330	NM_003510	**H2AC15**	H2A clustered histone 15	protein_coding	1	2	2	3	1.401
8331	NM_021066	**H2AC14**	H2A clustered histone 14	protein_coding	1	2	2	3	1.474
8332	NM_003511	**H2AC16**	H2A clustered histone 16	protein_coding	3	4	4	5	1.195
8334	NM_003512	**H2AC6**	H2A clustered histone 6	protein_coding	87	98	88	99	1.120
8335	NM_003513	**H2AC4**	H2A clustered histone 4	protein_coding	1	1	2	2	1.059
8336	NM_003514	**H2AC17**	H2A clustered histone 17	protein_coding	4	6	5	7	1.347
8337	NM_003516	**H2AC18**	H2A clustered histone 18	protein_coding	59	65	60	66	1.086
8338	NM_003517	**H2AC20**	H2A clustered histone 20	protein_coding	7	9	8	10	1.355
8339	NM_003518	**H2BC8**	H2B clustered histone 8	protein_coding	0	0	1	1	1.000
8340	NM_003519	**H2BC13**	H2B clustered histone 13	protein_coding	0	1	1	2	1.658
8341	NM_003520	**H2BC15**	H2B clustered histone 15	protein_coding	2	2	3	3	1.275
8342	NM_003521	**H2BC14**	H2B clustered histone 14	protein_coding	2	2	3	3	1.037
8343	NM_003522	**H2BC7**	H2B clustered histone 7	protein_coding	1	1	2	2	0.961
8344	NM_003523	**H2BC6**	H2B clustered histone 6	protein_coding	0	0	1	1	1.000
8345	NM_003524	**H2BC9**	H2B clustered histone 9	protein_coding	7	9	8	10	1.181
8346	NM_003525	**H2BC10**	H2B clustered histone 10	protein_coding	1	1	2	2	1.120
8347	NM_001381989,NM	**H2BC4**	H2B clustered histone 4	protein_coding	6	5	7	6	0.937
8348	NM_003527	**H2BC17**	H2B clustered histone 17	protein_coding	7	6	8	7	0.863
8349	NM_003528	**H2BC21**	H2B clustered histone 21	protein_coding	3	2	4	3	0.811
8350	NM_003529	**H3C1**	H3 clustered histone 1	protein_coding	0	1	1	2	1.592
8351	NM_001376937,NM	**H3C4**	H3 clustered histone 4	protein_coding	7	5	8	6	0.746
8352	NM_003531	**H3C3**	H3 clustered histone 3	protein_coding	0	0	1	1	1.000
8353	NM_001381999,NM	**H3C6**	H3 clustered histone 6	protein_coding	0	0	1	1	0.804
8354	NM_003533	**H3C11**	H3 clustered histone 11	protein_coding	3	3	4	4	1.067
8355	NM_003534	**H3C8**	H3 clustered histone 8	protein_coding	3	4	4	5	1.113
8356	NM_003535	**H3C12**	H3 clustered histone 12	protein_coding	0	0	1	1	0.872
8357	NM_003536	**H3C10**	H3 clustered histone 10	protein_coding	6	6	7	7	0.946
8358	NM_003537	**H3C2**	H3 clustered histone 2	protein_coding	1	0	2	1	0.700
8359	NM_003538	**H4C1**	H4 clustered histone 1	protein_coding	0	1	1	2	1.058
8360	NM_003539	**H4C4**	H4 clustered histone 4	protein_coding	2	1	3	2	0.831
8361	NM_003540	**H4C6**	H4 clustered histone 6	protein_coding	1	1	2	2	1.202
8362	NM_003541	**H4C12**	H4 clustered histone 12	protein_coding	8	8	9	9	1.020
8363	NM_021968	**H4C11**	H4 clustered histone 11	protein_coding	8	9	9	10	1.096
8364	NM_003542	**H4C3**	H4 clustered histone 3	protein_coding	3	2	4	3	0.705

8365	NM_003543	**H4C8**	H4 clustered histone 8	protein_coding	4	6	5	7	1.353
8366	NM_003544	**H4C2**	H4 clustered histone 2	protein_coding	0	0	1	1	1.000
8367	NM_003545	**H4C5**	H4 clustered histone 5	protein_coding	0	0	1	1	0.990
8368	NM_003546	**H4C13**	H4 clustered histone 13	protein_coding	0	0	1	1	0.965
8369	NM_003547	**H4C7**	H4 clustered histone 7	protein_coding	0	0	1	1	1.000
8370	NM_003548	**H4C14**	H4 clustered histone 14	protein_coding	11	13	12	14	1.132
8372	NM_001200029,NM	**HYAL3**	hyaluronidase 3	protein_coding	5	5	6	6	1.001
8373	gene-IFIT1P1	**IFIT1P1**	interferon induced protei	pseudogene	0	0	1	1	1.187
8379	NM_001013836,NM	**MAD1L1**	mitotic arrest deficient 1	protein_coding	64	73	65	74	1.131
8382	NM_003551,XM_00	**NME5**	NME/NM23 family memb	protein_coding	0	0	1	1	1.000
8383	NM_014565	**OR1A1**	olfactory receptor family	protein_coding	0	0	1	1	1.000
8386	NM_014566	**OR1D5**	olfactory receptor family	protein_coding	0	0	1	1	1.000
8387	NM_003553	**OR1E1**	olfactory receptor family	protein_coding	0	0	1	1	1.000
8388	NM_003554	**OR1E2**	olfactory receptor family	protein_coding	0	0	1	1	1.000
8389	NM_001348201	**OR1E3**	olfactory receptor family	protein_coding	0	0	1	1	1.000
8390	NM_003555	**OR1G1**	olfactory receptor family	protein_coding	0	0	1	1	1.000
8392	NM_012373,XM_01	**OR3A3**	olfactory receptor family	protein_coding	0	0	1	1	1.000
8394	NM_001135636,NM	**PIP5K1A**	phosphatidylinositol-4-ph	protein_coding	98	87	99	88	0.892
8395	NM_001278253,NM	**PIP5K1B**	phosphatidylinositol-4-ph	protein_coding	0	0	1	1	1.015
8396	NM_003559,XM_01	**PIP4K2B**	phosphatidylinositol-5-ph	protein_coding	66	61	67	62	0.931
8398	NM_001004426,NM	**PLA2G6**	phospholipase A2 group	protein_coding	2	2	3	3	0.988
8399	NM_003561,NR_13	**PLA2G10**	phospholipase A2 group	protein_coding	0	0	1	1	1.000
8402	NM_001165417,NM	**SLC25A11**	solute carrier family 25 m	protein_coding	59	64	60	65	1.089
8403	NM_004189	**SOX14**	SRY-box transcription fa	protein_coding	0	0	1	1	1.000
8404	NM_001128310,NM	**SPARCL1**	SPARC like 1	protein_coding	0	0	1	1	1.000
8405	NM_001007226,NM	**SPOP**	speckle type BTB/POZ p	protein_coding	38	37	39	38	0.964
8406	NM_001170750,NM	**SRPX**	sushi repeat containing	protein_coding	0	0	1	1	1.000
8407	NM_001277223,NM	**TAGLN2**	transgelin 2	protein_coding	985	1,057	986	1,058	1.073
8408	NM_003565,XM_01	**ULK1**	unc-51 like autophagy ac	protein_coding	6	6	7	7	1.049
8409	NM_004182,NM_15	**UXT**	ubiquitously expressed p	protein_coding	142	152	143	153	1.070
8410	gene-RPS9P1	**RPS9P1**	ribosomal protein S9 pse	pseudogene	0	0	1	1	1.000
8411	NM_003566,XM_01	**EEA1**	early endosome antigen	protein_coding	7	7	8	8	0.957
8412	NM_001261408,NM	**BCAR3**	BCAR3 adaptor protein,	protein_coding	0	0	1	1	1.174
8416	NM_003568,XM_01	**ANXA9**	annexin A9	protein_coding	2	3	3	4	1.188
8417	NM_001326578,NM	**STX7**	syntaxin 7	protein_coding	7	5	8	6	0.734
8418	NR_002174,NR_02	**CMAHP**	cytidine monophospho-N	transcribed_pseu	87	61	88	62	0.706
8419	NM_003571,XM_01	**BFSP2**	beaded filament structur	protein_coding	0	0	1	1	1.000
8420	NR_002909,NR_03	**SNHG3**	small nucleolar RNA hos	lncRNA	70	63	71	64	0.901
8424	NM_001376258,NM	**BBOX1**	gamma-butyrobetaine hy	protein_coding	0	0	1	1	1.000
8425	NM_001042544,NM	**LTBP4**	latent transforming grow	protein_coding	0	0	1	1	1.000
8427	NM_001303481,NM	**ZNF282**	zinc finger protein 282	protein_coding	15	17	16	18	1.068
8428	NM_001032296,NM	**STK24**	serine/threonine kinase 2	protein_coding	93	79	94	80	0.856
8431	NM_021969,XM_01	**NR0B2**	nuclear receptor subfam	protein_coding	0	0	1	1	1.000
8433	NM_003577	**UTF1**	undifferentiated embryon	protein_coding	0	0	1	1	1.000
8434	NM_001316345,NM	**RECK**	reversion inducing cystei	protein_coding	1	0	2	1	0.949
8435	NM_003578	**SOAT2**	sterol O-acyltransferase	protein_coding	1	1	2	2	1.130
8436	NM_004657	**CAVIN2**	caveolae associated prot	protein_coding	0	0	1	1	1.000
8437	NM_001193520,NM	**RASAL1**	RAS protein activator like	protein_coding	0	0	1	1	1.000
8438	NM_001142548,NM	**RAD54L**	RAD54 like	protein_coding	23	22	24	23	0.963
8439	NM_001144772,NM	**NSMAF**	neutral sphingomyelinas	protein_coding	63	58	64	59	0.937
8440	NM_001004720,NM	**NCK2**	NCK adaptor protein 2	protein_coding	5	4	6	5	0.762
8443	NM_001316350,NM	**GNPAT**	glyceronephosphate O-a	protein_coding	55	48	56	49	0.884
8444	NM_001004023,NM	**DYRK3**	dual specificity tyrosine	protein_coding	2	2	3	3	0.871
8445	NM_003583,NM_00	**DYRK2**	dual specificity tyrosine	protein_coding	10	8	11	9	0.799
8446	NM_003584	**DUSP11**	dual specificity phosphat	protein_coding	71	67	72	68	0.937
8447	NM_003585,XM_01	**DOC2B**	double C2 domain beta	protein_coding	0	0	1	1	1.000
8448	NM_001282062,NM	**DOC2A**	double C2 domain alpha	protein_coding	0	0	1	1	1.000
8449	NM_001164239,NM	**DHX16**	DEAH-box helicase 16	protein_coding	80	83	81	84	1.030
8450	NM_001079872,NM	**CUL4B**	cullin 4B	protein_coding	21	19	22	20	0.911
8451	NM_001008895,NM	**CUL4A**	cullin 4A	protein_coding	81	78	82	79	0.960
8452	NM_001257197,NM	**CUL3**	cullin 3	protein_coding	67	60	68	61	0.895
8453	NM_001198777,NM	**CUL2**	cullin 2	protein_coding	15	12	16	13	0.831
8454	NM_001370660,NM	**CUL1**	cullin 1	protein_coding	309	267	310	268	0.864
8455	NM_001207047,NM	**ATRN**	attractin	protein_coding	10	8	11	9	0.834
8456	NM_001369369,NM	**FOXN1**	forkhead box N1	protein_coding	0	0	1	1	1.000
8458	NM_003594,XM_00	**TTF2**	transcription termination	protein_coding	43	41	44	42	0.960
8459	NM_001008566,NM	**TPST2**	tyrosylprotein sulfotransf	protein_coding	31	34	32	35	1.079
8460	NM_003596,XM_00	**TPST1**	tyrosylprotein sulfotransf	protein_coding	0	1	1	2	1.176
8462	NM_001177716,NM	**KLF11**	Kruppel like factor 11	protein_coding	0	0	1	1	1.000
8463	NM_001256658,NM	**TEAD2**	TEA domain transcriptior	protein_coding	0	0	1	1	1.079
8464	NM_001261823,NM	**SUPT3H**	SPT3 homolog, SAGA ar	protein_coding	4	3	5	4	0.857
8467	NM_003601,XR_00	**SMARCA5**	SWI/SNF related, matrix	protein_coding	78	70	79	71	0.899
8468	NM_001135211,NM	**FKBP6**	FKBP prolyl isomerase 6	protein_coding	0	0	1	1	1.000
8470	NM_001145670,NM	**SORBS2**	sorbin and SH3 domain	protein_coding	0	0	1	1	1.000
8471	NM_001379150,NM	**IRS4**	insulin receptor substrate	protein_coding	0	0	1	1	1.000
8473	NM_181672,NM_18	**OGT**	O-linked N-acetylglucosa	protein_coding	111	101	112	102	0.909
8476	NM_001366010,NM	**CDC42BPA**	CDC42 binding protein k	protein_coding	0	0	1	1	1.000
8477	NM_003608	**GPR65**	G protein-coupled recept	protein_coding	11	11	12	12	0.934
8479	NM_001197323,NM	**HIRIP3**	HIRA interacting protein	protein_coding	75	80	76	81	1.059
8480	NM_001015885,NM	**RAE1**	ribonucleic acid export 1	protein_coding	106	113	107	114	1.065
8481	NM_001330209,NM	**OFD1**	OFD1 centriole and cent	protein_coding	27	19	28	20	0.722
8482	NM_001146029,NM	**SEMA7A**	semaphorin 7A (John Mil	protein_coding	71	78	72	79	1.095
8483	NM_003613,XM_01	**CILP**	cartilage intermediate lay	protein_coding	0	0	1	1	1.000
8484	NM_003614	**GALR3**	galanin receptor 3	protein_coding	0	0	1	1	1.000
8487	NM_001009182,NM	**GEMIN2**	gem nuclear organelle a	protein_coding	46	44	47	45	0.960
8488	gene-RPL3P1	**RPL3P1**	ribosomal protein L3 pse	pseudogene	0	0	1	1	1.000
8489	gene-RPL23AP3	**RPL23AP3**	ribosomal protein L23a p	pseudogene	0	0	1	1	1.000
8490	NM_001195303,NM	**RGS5**	regulator of G protein sig	protein_coding	4	5	5	6	1.162
8491	NM_001270425,NM	**MAP4K3**	mitogen-activated protei	protein_coding	7	5	8	6	0.779
8492	NM_003619,XM_00	**PRSS12**	serine protease 12	protein_coding	0	0	1	1	1.000
8493	NM_003620,XR_93	**PPM1D**	protein phosphatase, Mg	protein_coding	14	13	15	14	0.901
8495	NM_001256568,NM	**PPFIBP2**	PPFIA binding protein 2	protein_coding	0	0	1	1	0.978
8496	NM_001198915,NM	**PPFIBP1**	PPFIA binding protein 1	protein_coding	10	8	11	9	0.800
8497	NM_001304331,NM	**PPFIA4**	PTPRF interacting protei	protein_coding	0	0	1	1	1.000
8498	NM_001300865,NM	**RANBP3**	RAN binding protein 3	protein_coding	65	65	66	66	1.012
8499	NM_001220473,NM	**PPFIA2**	PTPRF interacting protei	protein_coding	0	0	1	1	0.983
8500	NM_001378006,NM	**PPFIA1**	PTPRF interacting protei	protein_coding	49	45	50	46	0.919
8501	NM_001198810,NM	**SLC43A1**	solute carrier family 43 m	protein_coding	1	1	2	2	0.977
8502	NM_001005476,NM	**PKP4**	plakophilin 4	protein_coding	35	30	36	31	0.853
8503	NM_001114172,NM	**PIK3R3**	phosphoinositide-3-kinas	protein_coding	16	14	17	15	0.886
8504	NM_003630	**PEX3**	peroxisomal biogenesis f	protein_coding	18	19	19	20	1.056
8505	NM_001303486,NM	**PARG**	poly(ADP-ribose) glycohy	protein_coding	28	26	29	27	0.928
8506	NM_003632,XM_00	**CNTNAP1**	contactin associated prot	protein_coding	0	0	1	1	0.922
8507	NM_001256574,NM	**ENC1**	ectodermal-neural cortex	protein_coding	0	0	1	1	1.000
8508	NM_001202502,NM	**NIPSNAP1**	nipsnap homolog 1	protein_coding	17	18	18	19	1.060
8509	NM_001330107,NM	**NDST2**	N-deacetylase and N-sul	protein_coding	12	12	13	13	0.975
8510	NM_006983,XM_01	**MMP23B**	matrix metallopeptidase	protein_coding	1	0	2	1	0.850
8511	NR_002946	**MMP23A**	matrix metallopeptidase	transcribed_pseu	0	0	1	1	0.969
8512	NR_002724	**MBL1P**	mannose binding lectin 1	transcribed_pseu	0	0	1	1	1.000
8513	NM_001198828,NM	**LIPF**	lipase F, gastric type	protein_coding	0	0	1	1	1.000
8514	NM_001199860,NM	**KCNAB2**	potassium voltage-gated	protein_coding	175	180	176	181	1.026
8515	NM_001303040,NM	**ITGA10**	integrin subunit alpha 10	protein_coding	1	1	2	2	0.982
8516	NM_001291494,NM	**ITGA8**	integrin subunit alpha 8	protein_coding	0	0	1	1	1.000
8517	NM_001099856,NM	**IKBKG**	inhibitor of nuclear factor	protein_coding	26	29	27	30	1.080
8518	NM_001318360,NM	**ELP1**	elongator complex protei	protein_coding	32	28	33	29	0.893
8519	NM_003641	**IFITM1**	interferon induced transr	protein_coding	809	816	810	817	1.009
8520	NM_003642,NR_02	**HAT1**	histone acetyltransferase	protein_coding	247	226	248	227	0.917
8521	NM_003643,XM_01	**GCM1**	glial cells missing transc	protein_coding	0	0	1	1	0.943
8522	NM_001130831,NM	**GAS7**	growth arrest specific 7	protein_coding	1	1	2	2	1.005
8525	NM_001105540,NM	**DGKZ**	diacylglycerol kinase zet	protein_coding	246	255	247	256	1.037
8526	NM_003647,XM_01	**DGKE**	diacylglycerol kinase eps	protein_coding	21	18	22	19	0.842
8527	NM_001377259,NM	**DGKD**	diacylglycerol kinase del	protein_coding	77	71	78	72	0.922
8528	NM_001368170,NM	**DDO**	D-aspartate oxidase	protein_coding	0	0	1	1	1.184
8529	NM_001082	**CYP4F2**	cytochrome P450 family	protein_coding	0	0	1	1	1.000
8530	NM_003650	**CST7**	cystatin F	protein_coding	225	230	226	231	1.024
8531	NM_001145426,NM	**YBX3**	Y-box binding protein 3	protein_coding	0	0	1	1	1.092

8532	NM_001014447,NM	**CPZ**	carboxypeptidase Z	protein_coding	0	0	1	1	1.000
8533	NM_001199125,NM	**COPS3**	COP9 signalosome subu	protein_coding	95	92	96	93	0.963
8534	NM_003654,XM_00	**CHST1**	carbohydrate sulfotransf	protein_coding	0	0	1	1	1.000
8535	NM_003655,XM_01	**CBX4**	chromobox 4	protein_coding	22	26	23	27	1.136
8536	NM_003656,XM_00	**CAMK1**	calcium/calmodulin depe	protein_coding	0	0	1	1	1.000
8537	NM_001316361,NM	**BCAS1**	brain enriched myelin as	protein_coding	0	0	1	1	1.000
8538	NM_003658,XM_01	**BARX2**	BARX homeobox 2	protein_coding	0	0	1	1	1.000
8539	NM_001142930,NM	**API5**	apoptosis inhibitor 5	protein_coding	182	161	183	162	0.886
8540	NM_003659,XM_01	**AGPS**	alkylglycerone phosphat	protein_coding	78	67	79	68	0.866
8541	NM_003660,NR_10	**PPFIA3**	PTPRF interacting protei	protein_coding	3	3	4	4	1.130
8542	NM_001136540,NM	**APOL1**	apolipoprotein L1	protein_coding	21	20	22	21	0.982
8543	NM_001369491,NM	**LMO4**	LIM domain only 4	protein_coding	56	58	57	59	1.037
8544	NM_001018109,NM	**PIR**	pirin	protein_coding	0	0	1	1	1.000
8545	NM_001008390,NM	**CGGBP1**	CGG triplet repeat bindir	protein_coding	83	71	84	72	0.850
8546	NM_001271769,NM	**AP3B1**	adaptor related protein c	protein_coding	56	49	57	50	0.866
8547	NM_003665,NM_17	**FCN3**	ficolin 3	protein_coding	0	0	1	1	1.000
8548	NM_001320972,NM	**BLZF1**	basic leucine zipper nucl	protein_coding	19	16	20	17	0.811
8549	NM_001277226,NM	**LGR5**	leucine rich repeat conta	protein_coding	0	0	1	1	1.000
8550	NM_001371479,NM	**MAPKAPK5**	MAPK activated protein k	protein_coding	10	11	11	12	1.093
8551	NR_002725	**INE2**	inactivation escape 2	lncRNA	0	0	1	1	1.000
8552	NR_024616	**INE1**	inactivation escape 1	lncRNA	1	1	2	2	1.488
8553	NM_003670,XM_02	**BHLHE40**	basic helix-loop-helix fam	protein_coding	180	173	181	174	0.957
8554	NM_001320687,NM	**PIAS1**	protein inhibitor of activa	protein_coding	24	22	25	23	0.929
8555	NM_001077181,NM	**CDC14B**	cell division cycle 14B	protein_coding	0	0	1	1	1.000
8556	NM_001319210,NM	**CDC14A**	cell division cycle 14A	protein_coding	1	1	2	2	0.845
8557	NM_003673	**TCAP**	titin-cap	protein_coding	2	0	3	1	0.517
8558	NM_001098533,NM	**CDK10**	cyclin dependent kinase	protein_coding	13	12	14	13	0.980
8559	NM_003675,XM_01	**PRPF18**	pre-mRNA processing fa	protein_coding	12	10	13	11	0.873
8560	NM_001321541,NM	**DEGS1**	delta 4-desaturase, sphir	protein_coding	119	120	120	121	1.010
8562	NM_003677	**DENR**	density regulated re-initia	protein_coding	100	87	101	88	0.867
8563	NM_001002877,NM	**THOC5**	THO complex 5	protein_coding	33	38	34	39	1.159
8564	NM_003679,XM_00	**KMO**	kynurenine 3-monooxyge	protein_coding	0	0	1	1	1.096
8565	NM_003680,XM_01	**YARS1**	tyrosyl-tRNA synthetase	protein_coding	324	315	325	316	0.972
8566	NM_001331030,NM	**PDXK**	pyridoxal kinase	protein_coding	26	24	27	25	0.920
8567	NM_001135943,NM	**MADD**	MAP kinase activating de	protein_coding	31	29	32	30	0.916
8568	NM_003683,XM_01	**RRP1**	ribosomal RNA processir	protein_coding	75	75	76	76	1.000
8569	NM_001135553,NM	**MKNK1**	MAPK interacting serine/	protein_coding	32	29	33	30	0.929
8570	NM_001366299,NM	**KHSRP**	KH-type splicing regulato	protein_coding	363	366	364	367	1.007
8572	NM_001131027,NM	**PDLIM4**	PDZ and LIM domain 4	protein_coding	0	0	1	1	1.000
8573	NM_001126054,NM	**CASK**	calcium/calmodulin depe	protein_coding	3	2	4	3	0.902
8574	NM_001320979,NM	**AKR7A2**	aldo-keto reductase fami	protein_coding	24	23	25	24	0.946
8575	NM_001139517,NM	**PRKRA**	protein activator of interfe	protein_coding	68	62	69	63	0.922
8576	NM_001008910,NM	**STK16**	serine/threonine kinase 1	protein_coding	26	32	27	33	1.234
8577	NM_003692	**TMEFF1**	transmembrane protein w	protein_coding	0	0	1	1	1.000
8578	NM_003693,NM_14	**SCARF1**	scavenger receptor class	protein_coding	0	1	1	2	1.079
8581	NM_003695	**LY6D**	lymphocyte antigen 6 far	protein_coding	0	0	1	1	1.000
8587	NR_045004	**OR7E2P**	olfactory receptor family	transcribed_pseu	0	0	1	1	1.000
8590	NM_003696	**OR6A2**	olfactory receptor family	protein_coding	0	0	1	1	1.000
8600	NM_003701,NM_03	**TNFSF11**	TNF superfamily membe	protein_coding	0	0	1	1	1.000
8601	NM_001286673,NM	**RGS20**	regulator of G protein sig	protein_coding	0	0	1	1	1.000
8602	NM_001291978,NM	**NOP14**	NOP14 nucleolar protein	protein_coding	127	123	128	124	0.963
8603	NM_001256666,NM	**FAM193A**	family with sequence sim	protein_coding	16	14	17	15	0.887
8604	NM_003705,NR_04	**SLC25A12**	solute carrier family 25 m	protein_coding	52	45	53	46	0.877
8605	NM_001159322,NM	**PLA2G4C**	phospholipase A2 group	protein_coding	0	0	1	1	1.000
8607	NM_001319084,NM	**RUVBL1**	RuvB like AAA ATPase 1	protein_coding	255	262	256	263	1.030
8608	NM_001320108,NM	**RDH16**	retinol dehydrogenase 16	protein_coding	0	0	1	1	1.020
8609	NM_001270942,NM	**KLF7**	Kruppel like factor 7	protein_coding	0	0	1	1	0.990
8611	NM_003711,NM_17	**PLPP1**	phospholipid phosphatas	protein_coding	3	2	4	3	0.835
8612	NM_003712,NM_17	**PLPP2**	phospholipid phosphatas	protein_coding	0	0	1	1	1.000
8613	NM_003713	**PLPP3**	phospholipid phosphatas	protein_coding	0	0	1	1	1.000
8614	NM_003714	**STC2**	stanniocalcin 2	protein_coding	0	0	1	1	1.019
8615	NM_001290049,NM	**USO1**	USO1 vesicle transport fa	protein_coding	52	48	53	49	0.921
8618	NM_003716,NM_18	**CADPS**	calcium dependent secre	protein_coding	0	0	1	1	1.000
8620	NM_001320296,NM	**NPFF**	neuropeptide FF-amide p	protein_coding	1	1	2	2	0.760
8621	NM_003718,NM_03	**CDK13**	cyclin dependent kinase	protein_coding	11	10	12	11	0.921
8622	NM_001029851,NM	**PDE8B**	phosphodiesterase 8B	protein_coding	0	0	1	1	0.968
8623	NM_001173473,NM	**ASMTL**	acetylserotonin O-methy	protein_coding	10	12	11	13	1.214
8624	NM_001261824,NM	**PSMG1**	proteasome assembly ch	protein_coding	113	113	114	114	1.000
8625	NM_001278727,NM	**RFXANK**	regulatory factor X assoc	protein_coding	73	78	74	79	1.059
8626	NM_001114978,NM	**TP63**	tumor protein p63	protein_coding	0	0	1	1	1.000
8629	NM_001077527,NM	**JRK**	Jrk helix-turn-helix protei	protein_coding	7	6	8	7	0.914
8630	NM_003725,XM_00	**HSD17B6**	hydroxysteroid 17-beta d	protein_coding	2	3	3	4	1.235
8631	NM_001075099,NM	**SKAP1**	src kinase associated ph	protein_coding	86	80	87	81	0.932
8632	NM_173628,XM_01	**DNAH17**	dynein axonemal heavy c	protein_coding	0	0	1	1	1.000
8633	NM_003728,XM_00	**UNC5C**	unc-5 netrin receptor C	protein_coding	0	0	1	1	1.000
8634	NM_001130841,NM	**RTCA**	RNA 3'-terminal phospha	protein_coding	67	62	68	63	0.926
8635	NM_003730,XM_01	**RNASET2**	ribonuclease T2	protein_coding	0	0	1	1	1.002
8636	NM_003731	**SSNA1**	SS nuclear autoantigen 1	protein_coding	103	112	104	113	1.087
8637	NM_003732	**EIF4EBP3**	eukaryotic translation init	protein_coding	0	0	1	1	0.915
8638	NM_001261825,NM	**OASL**	2'-5'-oligoadenylate syntl	protein_coding	115	100	116	101	0.866
8639	NM_001277731,NM	**AOC3**	amine oxidase copper co	protein_coding	1	1	2	2	0.946
8641	NM_003736,NM_03	**PCDHGB4**	protocadherin gamma su	protein_coding	0	0	1	1	0.918
8642	NM_003737	**DCHS1**	dachsous cadherin-relate	protein_coding	0	0	1	1	0.969
8643	NM_001166292,NM	**PTCH2**	patched 2	protein_coding	1	1	2	2	0.907
8644	NM_001253908,NM	**AKR1C3**	aldo-keto reductase fami	protein_coding	2	1	3	2	0.758
8645	NM_003740,XM_00	**KCNK5**	potassium two pore dom	protein_coding	0	0	1	1	1.075
8646	NM_001304472,NM	**CHRD**	chordin	protein_coding	2	2	3	3	1.211
8647	NM_003742,XM_00	**ABCB11**	ATP binding cassette sul	protein_coding	0	0	1	1	1.000
8648	NM_001362950,NM	**NCOA1**	nuclear receptor coactiva	protein_coding	21	19	22	20	0.920
8649	NM_001243736,NM	**LAMTOR3**	late endosomal/lysosoma	protein_coding	19	20	20	21	1.066
8650	NM_001005743,NM	**NUMB**	NUMB endocytic adaptor	protein_coding	25	23	26	24	0.923
8651	NM_003745	**SOCS1**	suppressor of cytokine si	protein_coding	3	5	4	6	1.428
8653	NM_001122665,NM	**DDX3Y**	DEAD-box helicase 3 Y-l	protein_coding	107	88	108	89	0.825
8654	NM_001083,NM_03	**PDE5A**	phosphodiesterase 5A	protein_coding	0	0	1	1	1.000
8655	NM_001037494,NM	**DYNLL1**	dynein light chain LC8-ty	protein_coding	482	544	483	545	1.129
8658	NM_003747,XM_00	**TNKS**	tankyrase	protein_coding	5	4	6	5	0.971
8659	NM_001161504,NM	**ALDH4A1**	aldehyde dehydrogenase	protein_coding	4	5	5	6	1.020
8660	NM_003749	**IRS2**	insulin receptor substrate	protein_coding	0	0	1	1	1.000
8661	NM_003750	**EIF3A**	eukaryotic translation init	protein_coding	286	255	287	256	0.890
8662	NM_001037283,NM	**EIF3B**	eukaryotic translation init	protein_coding	224	225	225	226	1.001
8663	NM_001037808,NM	**EIF3C**	eukaryotic translation init	protein_coding	459	459	460	460	1.002
8664	NM_003753,NR_15	**EIF3D**	eukaryotic translation init	protein_coding	291	266	292	267	0.915
8665	NM_003754	**EIF3F**	eukaryotic translation init	protein_coding	52	57	53	58	1.102
8666	NM_003755	**EIF3G**	eukaryotic translation init	protein_coding	383	394	384	395	1.028
8667	NM_003756	**EIF3H**	eukaryotic translation init	protein_coding	150	154	151	155	1.024
8668	NM_003757,XM_02	**EIF3I**	eukaryotic translation init	protein_coding	406	415	407	416	1.022
8669	NM_001284335,NM	**EIF3J**	eukaryotic translation init	protein_coding	309	281	310	282	0.910
8671	NM_001098484,NM	**SLC4A4**	solute carrier family 4 me	protein_coding	0	0	1	1	0.966
8672	NM_001198801,NM	**EIF4G3**	eukaryotic translation init	protein_coding	38	30	39	31	0.799
8673	NM_003761,XM_01	**VAMP8**	vesicle associated memb	protein_coding	108	110	109	111	1.017
8674	NM_001185127,NM	**VAMP4**	vesicle associated memb	protein_coding	8	8	9	9	0.997
8675	NM_001001433,NM	**STX16**	syntaxin 16	protein_coding	71	63	72	64	0.890
8676	NM_003764,XM_01	**STX11**	syntaxin 11	protein_coding	39	40	40	41	1.031
8677	NM_001271609,NM	**STX10**	syntaxin 10	protein_coding	75	78	76	79	1.046
8678	NM_001313998,NM	**BECN1**	beclin 1	protein_coding	137	133	138	134	0.974
8681	NM_001198588,NM	**JMJD7-PLA2G4B**	JMJD7-PLA2G4B readth	protein_coding	0	3	1	4	3.600
8682	NM_001297576,NM	**PEA15**	proliferation and apoptos	protein_coding	93	82	94	83	0.879
8683	NM_003769	**SRSF9**	serine and arginine rich s	protein_coding	244	250	245	251	1.027
8685	NM_006770,XM_01	**MARCO**	macrophage receptor wit	protein_coding	0	0	1	1	1.000
8686	gene-KRT41P	**KRT41P**	keratin 41 pseudogene	pseudogene	0	0	1	1	1.000
8687	NM_006771	**KRT38**	keratin 38	protein_coding	0	0	1	1	1.000
8688	NM_003770	**KRT37**	keratin 37	protein_coding	0	0	1	1	1.000
8689	NM_003771	**KRT36**	keratin 36	protein_coding	0	0	1	1	1.000
8690	NM_001261833,NM	**JRKL**	JRK like	protein_coding	14	13	15	14	0.927
8692	NM_003773,NM_03	**HYAL2**	hyaluronidase 2	protein_coding	16	15	17	16	0.905
8693	NM_003774	**GALNT4**	polypeptide N-acetylgala	protein_coding	5	3	6	4	0.717

8694	NM_012079,XM_01	**DGAT1**	diacylglycerol O-acyltran	protein_coding	3	4	4	5	1.056
8697	NM_004661	**CDC23**	cell division cycle 23	protein_coding	85	83	86	84	0.981
8698	NM_003775	**S1PR4**	sphingosine-1-phosphate	protein_coding	42	49	43	50	1.151
8701	NM_001277115	**DNAH11**	dynein axonemal heavy c	protein_coding	0	0	1	1	0.976
8702	NM_003778,NM_21	**B4GALT4**	beta-1,4-galactosyltransf	protein_coding	16	18	17	19	1.125
8703	NM_001199873,NM	**B4GALT3**	beta-1,4-galactosyltransf	protein_coding	68	65	69	66	0.962
8704	NM_001005417,NM	**B4GALT2**	beta-1,4-galactosyltransf	protein_coding	43	45	44	46	1.052
8705	NM_003782	**B3GALT4**	beta-1,3-galactosyltransf	protein_coding	0	0	1	1	1.019
8706	NM_001038628,NM	**B3GALNT1**	beta-1,3-N-acetylgalacto	protein_coding	0	0	1	1	1.000
8707	NM_003783	**B3GALT2**	beta-1,3-galactosyltransf	protein_coding	0	0	1	1	1.000
8708	NM_020981,XM_00	**B3GALT1**	beta-1,3-galactosyltransf	protein_coding	0	0	1	1	1.000
8710	NM_001040147,NM	**SERPINB7**	serpin family B member	protein_coding	0	0	1	1	1.000
8711	NM_001251902,NM	**TNK1**	tyrosine kinase non rece	protein_coding	0	0	1	1	1.000
8712	NM_003785,XM_01	**PAGE1**	PAGE family member 1	protein_coding	0	0	1	1	1.000
8714	NM_001144070,NM	**ABCC3**	ATP binding cassette sul	protein_coding	0	0	1	1	1.000
8715	NM_001198546,NM	**NOL4**	nucleolar protein 4	protein_coding	0	0	1	1	1.000
8717	NM_001323552,NM	**TRADD**	TNFRSF1A associated v	protein_coding	9	10	10	11	1.043
8718	NM_001039664,NM	**TNFRSF25**	TNF receptor superfamily	protein_coding	0	0	1	1	1.000
8720	NM_003791,XM_01	**MBTPS1**	membrane bound transc	protein_coding	51	51	52	52	1.004
8721	NM_001281297,NM	**EDF1**	endothelial differentiatior	protein_coding	420	458	421	459	1.089
8722	NM_003793,XM_01	**CTSF**	cathepsin F	protein_coding	0	0	1	1	1.000
8723	NM_003794,NR_07	**SNX4**	sorting nexin 4	protein_coding	65	57	66	58	0.888
8724	NM_001300928,NM	**SNX3**	sorting nexin 3	protein_coding	141	131	142	132	0.927
8725	NM_001252641,NM	**URI1**	URI1 prefoldin like chape	protein_coding	57	53	58	54	0.927
8726	NM_001308007,NM	**EED**	embryonic ectoderm dev	protein_coding	63	72	64	73	1.145
8727	NM_001286974,NM	**CTNNAL1**	catenin alpha like 1	protein_coding	27	22	28	23	0.808
8728	NM_033274,XM_01	**ADAM19**	ADAM metallopeptidase	protein_coding	0	0	1	1	1.000
8729	NM_001199378,NM	**GBF1**	golgi brefeldin A resistan	protein_coding	41	38	42	39	0.943
8731	NM_001308263,NM	**RNMT**	RNA guanine-7 methyltra	protein_coding	21	20	22	21	0.941
8732	NM_001286426,NM	**RNGTT**	RNA guanylyltransferase	protein_coding	27	23	28	24	0.871
8733	NM_003801	**GPAA1**	glycosylphosphatidylinos	protein_coding	34	34	35	35	1.017
8735	NM_003802	**MYH13**	myosin heavy chain 13	protein_coding	0	0	1	1	1.000
8736	NM_003803,NM_01	**MYOM1**	myomesin 1	protein_coding	1	1	2	2	0.965
8737	NM_001317061,NM	**RIPK1**	receptor interacting serin	protein_coding	21	22	22	23	1.027
8738	NM_001320099,NM	**CRADD**	CASP2 and RIPK1 doma	protein_coding	10	10	11	11	0.998
8739	NM_003806,NR_07	**HRK**	harakiri, BCL2 interacting	protein_coding	0	0	1	1	1.000
8740	NM_001376887,NM	**TNFSF14**	TNF superfamily membe	protein_coding	71	60	72	61	0.840
8741	NM_001198622,NM	**TNFSF13**	TNF superfamily membe	protein_coding	0	0	1	1	1.000
8742	NM_003809,NR_03	**TNFSF12**	TNF superfamily membe	protein_coding	7	6	8	7	0.836
8743	NM_001190942,NM	**TNFSF10**	TNF superfamily membe	protein_coding	39	34	40	35	0.890
8744	NM_003811	**TNFSF9**	TNF superfamily membe	protein_coding	0	0	1	1	1.000
8745	NM_003812,XM_00	**ADAM23**	ADAM metallopeptidase	protein_coding	0	0	1	1	1.000
8747	NM_003813	**ADAM21**	ADAM metallopeptidase	protein_coding	0	0	1	1	1.010
8748	NM_003814,XM_00	**ADAM20**	ADAM metallopeptidase	protein_coding	0	0	1	1	0.991
8749	NM_001190956,NM	**ADAM18**	ADAM metallopeptidase	protein_coding	0	0	1	1	1.000
8751	NM_001261464,NM	**ADAM15**	ADAM metallopeptidase	protein_coding	6	7	7	8	1.082
8754	NM_003816,NR_02	**ADAM9**	ADAM metallopeptidase	protein_coding	12	11	13	12	0.890
8755	NR_002224	**ADAM6**	ADAM metallopeptidase	transcribed_pseu	0	0	1	1	1.000
8756	NM_003817,XM_01	**ADAM7**	ADAM metallopeptidase	protein_coding	0	0	1	1	1.000
8760	NM_003818,XM_00	**CDS2**	CDP-diacylglycerol synth	protein_coding	16	14	17	15	0.909
8761	NM_001135653,NM	**PABPC4**	poly(A) binding protein c	protein_coding	117	108	118	109	0.921
8763	NM_001142401,NM	**CD164**	CD164 molecule	protein_coding	226	204	227	205	0.906
8764	NM_001297605,NM	**TNFRSF14**	TNF receptor superfamily	protein_coding	11	14	12	15	1.212
8766	NM_001206836,NM	**RAB11A**	RAB11A, member RAS o	protein_coding	58	55	59	56	0.946
8767	NM_001375360,NM	**RIPK2**	receptor interacting serin	protein_coding	27	24	28	25	0.882
8771	NM_003823	**TNFRSF6B**	TNF receptor superfamily	protein_coding	1	0	2	1	0.731
8772	NM_003824	**FADD**	Fas associated via death	protein_coding	61	62	62	63	1.017
8773	NM_003825,NM_13	**SNAP23**	synaptosome associated	protein_coding	105	95	106	96	0.912
8774	NM_003826,XM_01	**NAPG**	NSF attachment protein	protein_coding	19	17	20	18	0.934
8775	NM_003827,NR_03	**NAPA**	NSF attachment protein	protein_coding	132	151	133	152	1.137
8776	NM_001306144,NM	**MTMR1**	myotubularin related pro	protein_coding	52	53	53	54	1.023
8777	NM_001261406,NM	**MPDZ**	multiple PDZ domain cru	protein_coding	0	0	1	1	1.000
8778	NM_003830,XM_01	**SIGLEC5**	sialic acid binding Ig like	protein_coding	0	0	1	1	1.073
8780	NM_001348193,NM	**RIOK3**	RIO kinase 3	protein_coding	60	57	61	58	0.955
8781	gene-PSPHP1	**PSPHP1**	phosphoserine phosphat	pseudogene	0	0	1	1	1.385
8784	NM_004195,NM_14	**TNFRSF18**	TNF receptor superfamily	protein_coding	0	0	1	1	1.000
8785	NM_003833,NM_03	**MATN4**	matrilin 4	protein_coding	0	0	1	1	0.968
8786	NM_001286485,NM	**RGS11**	regulator of G protein sig	protein_coding	0	0	1	1	1.000
8787	NM_001081955,NM	**RGS9**	regulator of G protein sig	protein_coding	4	3	5	4	0.920
8788	NM_001317172,NM	**DLK1**	delta like non-canonical	protein_coding	0	0	1	1	1.000
8789	NM_003837	**FBP2**	fructose-bisphosphatase	protein_coding	0	0	1	1	1.000
8790	NM_001199328,NM	**FPGT**	fucose-1-phosphate gua	protein_coding	8	7	9	8	0.932
8792	NM_001270949,NM	**TNFRSF11A**	TNF receptor superfamily	protein_coding	0	0	1	1	1.000
8793	NM_003840	**TNFRSF10D**	TNF receptor superfamily	protein_coding	0	0	1	1	1.000
8794	NM_003841	**TNFRSF10C**	TNF receptor superfamily	protein_coding	1	0	2	1	0.827
8795	NM_003842,NM_14	**TNFRSF10B**	TNF receptor superfamily	protein_coding	20	15	21	16	0.784
8796	NM_001160706,NM	**SCEL**	sciellin	protein_coding	0	0	1	1	1.000
8797	NM_003844	**TNFRSF10A**	TNF receptor superfamily	protein_coding	2	1	3	2	0.789
8798	NM_001282285,NM	**DYRK4**	dual specificity tyrosine p	protein_coding	21	23	22	24	1.106
8799	NM_001184795,NM	**PEX11B**	peroxisomal biogenesis f	protein_coding	41	41	42	42	1.010
8800	NM_001271572,NM	**PEX11A**	peroxisomal biogenesis f	protein_coding	1	1	2	2	1.004
8801	NM_001177599,NM	**SUCLG2**	succinate-CoA ligase GD	protein_coding	41	41	42	42	0.988
8802	NM_003849	**SUCLG1**	succinate-CoA ligase GD	protein_coding	208	198	209	199	0.951
8803	NM_003850	**SUCLA2**	succinate-CoA ligase AD	protein_coding	81	76	82	77	0.941
8804	NM_003851	**CREG1**	cellular repressor of E1A	protein_coding	33	33	34	34	1.004
8805	NM_003852,NM_01	**TRIM24**	tripartite motif containing	protein_coding	16	16	17	17	1.005
8807	NM_003853,XM_01	**IL18RAP**	interleukin 18 receptor ac	protein_coding	1	2	2	3	1.131
8808	NM_001351446,NM	**IL1RL2**	interleukin 1 receptor like	protein_coding	0	0	1	1	0.947
8809	NM_001282399,NM	**IL18R1**	interleukin 18 receptor 1	protein_coding	8	7	9	8	0.895
8811	NM_003857,XM_01	**GALR2**	galanin receptor 2	protein_coding	1	2	2	3	1.020
8812	NM_001099402,XM	**CCNK**	cyclin K	protein_coding	71	69	72	70	0.969
8813	NM_001317034,NM	**DPM1**	dolichyl-phosphate mann	protein_coding	258	246	259	247	0.954
8814	NM_001282236,NM	**CDKL1**	cyclin dependent kinase	protein_coding	2	1	3	2	0.676
8815	NM_001143985,NM	**BANF1**	BAF nuclear assembly fa	protein_coding	428	451	429	452	1.053
8816	NM_001284206,NM	**DCAF5**	DDB1 and CUL4 associa	protein_coding	23	23	24	24	0.998
8817	NM_003862	**FGF18**	fibroblast growth factor 1	protein_coding	0	0	1	1	1.062
8818	NM_001378436,NM	**DPM2**	dolichyl-phosphate mann	protein_coding	90	93	91	94	1.024
8819	NM_003864	**SAP30**	Sin3A associated protein	protein_coding	39	42	40	43	1.058
8820	NM_001376058,NM	**HESX1**	HESX homeobox 1	protein_coding	1	2	2	3	1.355
8821	NM_001101669,NM	**INPP4B**	inositol polyphosphate-4	protein_coding	4	3	5	4	0.749
8822	NM_001304478,NM	**FGF17**	fibroblast growth factor 1	protein_coding	0	0	1	1	0.967
8823	NM_003868	**FGF16**	fibroblast growth factor 1	protein_coding	0	0	1	1	1.000
8824	NM_001365405,NM	**CES2**	carboxylesterase 2	protein_coding	22	22	23	23	0.982
8825	NM_001324423,NM	**LIN7A**	lin-7 homolog A, crumbs	protein_coding	0	0	1	1	1.000
8826	NM_003870,XR_00	**IQGAP1**	IQ motif containing GTPa	protein_coding	192	166	193	167	0.869
8828	NM_003872,NM_01	**NRP2**	neuropilin 2	protein_coding	0	0	1	1	0.964
8829	NM_001024628,NM	**NRP1**	neuropilin 1	protein_coding	0	0	1	1	1.000
8831	NM_001130066,NM	**SYNGAP1**	synaptic Ras GTPase ac	protein_coding	3	3	4	4	1.002
8832	NM_001184879,NM	**CD84**	CD84 molecule	protein_coding	0	0	1	1	0.991
8833	NM_003875,XM_01	**GMPS**	guanine monophosphate	protein_coding	47	38	48	39	0.821
8834	NM_003876,NR_02	**TMEM11**	transmembrane protein 1	protein_coding	76	79	77	80	1.039
8835	NM_001270467,NM	**SOCS2**	suppressor of cytokine s	protein_coding	67	65	68	66	0.975
8836	NM_003878,XM_01	**GGH**	gamma-glutamyl hydrola	protein_coding	90	88	91	89	0.981
8837	NM_001127183,NM	**CFLAR**	CASP8 and FADD like ap	protein_coding	65	57	66	58	0.880
8838	NM_003880,NM_19	**CCN6**	cellular communication n	protein_coding	0	0	1	1	1.068
8839	NM_001323369,NM	**CCN5**	cellular communication n	protein_coding	0	0	1	1	1.000
8840	NM_001204869,NM	**CCN4**	cellular communication n	protein_coding	0	0	1	1	1.000
8841	NM_001355039,NM	**HDAC3**	histone deacetylase 3	protein_coding	125	124	126	125	0.994
8842	NM_001145847,NM	**PROM1**	prominin 1	protein_coding	0	0	1	1	0.974
8843	NM_006018	**HCAR3**	hydroxycarboxylic acid re	protein_coding	0	0	1	1	0.956
8844	NM_001367810,NM	**KSR1**	kinase suppressor of ras	protein_coding	76	77	77	78	1.012
8846	NM_006020,XR_00	**ALKBH1**	alkB homolog 1, histone	protein_coding	20	20	21	21	0.985
8847	NR_152566,NR_15	**DLEU2**	deleted in lymphocytic le	lncRNA	109	103	110	104	0.946
8848	NM_001243797,NM	**TSC22D1**	TSC22 domain family me	protein_coding	11	11	12	12	0.974
8850	NM_003884,XM_00	**KAT2B**	lysine acetyltransferase	protein_coding	50	46	51	47	0.937
8851	NM_003885,XM_01	**CDK5R1**	cyclin dependent kinase	protein_coding	4	4	5	5	0.977

8852	NM_003886,NM_13	**AKAP4**	A-kinase anchoring prote	protein_coding	0	0	1	1	1.000
8853	NM_001135191,NM	**ASAP2**	ArfGAP with SH3 domair	protein_coding	0	0	1	1	1.000
8854	NM_001206897,NM	**ALDH1A2**	aldehyde dehydrogenase	protein_coding	0	0	1	1	1.000
8856	NM_003889,NM_02	**NR1I2**	nuclear receptor subfam	protein_coding	0	0	1	1	1.000
8857	NM_003890	**FCGBP**	Fc fragment of IgG bindi	protein_coding	0	0	1	1	0.966
8858	NM_001256134,NM	**PROZ**	protein Z, vitamin K depe	protein_coding	0	0	1	1	1.000
8859	NM_004197,NM_03	**STK19**	serine/threonine kinase 1	protein_coding	10	9	11	10	0.953
8861	NM_001113407,NM	**LDB1**	LIM domain binding 1	protein_coding	32	35	33	36	1.098
8862	NM_017413	**APLN**	apelin	protein_coding	0	0	1	1	1.000
8863	NM_001289861,NM	**PER3**	period circadian regulato	protein_coding	0	0	1	1	0.913
8864	NM_022817,XM_00	**PER2**	period circadian regulato	protein_coding	41	35	42	36	0.866
8867	NM_001160302,NM	**SYNJ1**	synaptojanin 1	protein_coding	7	6	8	7	0.905
8869	NM_001042437,NM	**ST3GAL5**	ST3 beta-galactoside alp	protein_coding	33	33	34	34	0.993
8870	NM_003897	**IER3**	immediate early respons	protein_coding	0	0	1	1	1.000
8871	NM_001178088,NM	**SYNJ2**	synaptojanin 2	protein_coding	0	0	1	1	1.028
8872	NM_006023,XM_00	**CDC123**	cell division cycle 123	protein_coding	92	94	93	95	1.029
8874	NM_001113511,NM	**ARHGEF7**	Rho guanine nucleotide	protein_coding	40	34	41	35	0.859
8875	NM_001242350,NM	**VNN2**	vanin 2	protein_coding	1	0	2	1	0.883
8876	NM_004666	**VNN1**	vanin 1	protein_coding	0	0	1	1	1.000
8877	NM_001142601,NM	**SPHK1**	sphingosine kinase 1	protein_coding	0	0	1	1	1.000
8878	NM_001142298,NM	**SQSTM1**	sequestosome 1	protein_coding	30	36	31	37	1.182
8879	NM_003901,XM_00	**SGPL1**	sphingosine-1-phosphate	protein_coding	22	21	23	22	0.948
8880	NM_001303433,NM	**FUBP1**	far upstream element bir	protein_coding	120	101	121	102	0.842
8881	NM_001078645,NM	**CDC16**	cell division cycle 16	protein_coding	71	72	72	73	1.006
8882	NM_001317086,NM	**ZPR1**	ZPR1 zinc finger	protein_coding	50	51	51	52	1.032
8883	NM_001018159,NM	**NAE1**	NEDD8 activating enzym	protein_coding	131	110	132	111	0.843
8884	NM_021095,NR_02	**SLC5A6**	solute carrier family 5 me	protein_coding	63	68	64	69	1.067
8886	NM_006773	**DDX18**	DEAD-box helicase 18	protein_coding	156	150	157	151	0.958
8887	NM_001079864,NM	**TAX1BP1**	Tax1 binding protein 1	protein_coding	31	29	32	30	0.949
8888	NM_003906,XM_00	**MCM3AP**	minichromosome mainte	protein_coding	56	51	57	52	0.925
8890	NM_001034116,NM	**EIF2B4**	eukaryotic translation init	protein_coding	41	49	42	50	1.179
8891	NM_001166588,NM	**EIF2B3**	eukaryotic translation init	protein_coding	73	67	74	68	0.921
8892	NM_014239	**EIF2B2**	eukaryotic translation init	protein_coding	25	25	26	26	0.986
8893	NM_003907,XM_01	**EIF2B5**	eukaryotic translation init	protein_coding	104	84	105	85	0.812
8894	NM_001316363,NM	**EIF2S2**	eukaryotic translation init	protein_coding	377	344	378	345	0.911
8895	NM_003909,XM_00	**CPNE3**	copine 3	protein_coding	0	0	1	1	1.015
8896	NM_001370407,NM	**BUD31**	BUD31 homolog	protein_coding	196	195	197	196	0.992
8897	NM_021090,NM_15	**MTMR3**	myotubularin related pro	protein_coding	7	7	8	8	0.922
8898	NM_001243571,NM	**MTMR2**	myotubularin related pro	protein_coding	44	39	45	40	0.892
8899	NM_003913,NR_14	**PRPF4B**	pre-mRNA processing fa	protein_coding	59	53	60	54	0.896
8900	NM_001111045,NM	**CCNA1**	cyclin A1	protein_coding	0	0	1	1	0.967
8904	NM_001198863,NM	**CPNE1**	copine 1	protein_coding	255	255	256	256	1.001
8905	NM_001272071,NM	**AP1S2**	adaptor related protein c	protein_coding	21	19	22	20	0.898
8906	NM_001282474,NM	**AP1G2**	adaptor related protein c	protein_coding	34	36	35	37	1.056
8907	NM_001130524,NM	**AP1M1**	adaptor related protein c	protein_coding	17	18	18	19	1.062
8908	NM_001079855,NM	**GYG2**	glycogenin 2	protein_coding	0	0	1	1	1.000
8909	NM_001172439,NM	**ENDOU**	endonuclease, poly(U) s	protein_coding	0	0	1	1	1.083
8910	NM_001099400,NM	**SGCE**	sarcoglycan epsilon	protein_coding	0	0	1	1	1.000
8911	NM_001003406,NM	**CACNA1I**	calcium voltage-gated ch	protein_coding	0	0	1	1	1.000
8912	NM_001005407,NM	**CACNA1H**	calcium voltage-gated ch	protein_coding	0	0	1	1	1.000
8913	NM_001256324,NM	**CACNA1G**	calcium voltage-gated ch	protein_coding	0	0	1	1	1.010
8914	NM_001330295,NM	**TIMELESS**	timeless circadian regula	protein_coding	56	54	57	55	0.966
8915	NM_001320715,NM	**BCL10**	BCL10 immune signaling	protein_coding	23	23	24	24	0.986
8916	NM_001271602,NM	**HERC3**	HECT and RLD domain c	protein_coding	0	0	1	1	1.000
8917	gene-HERC2P1	**HERC2P1**	hect domain and RLD 2 p	pseudogene	0	0	1	1	1.000
8918	gene-HERC2P6	**HERC2P6**	hect domain and RLD 2 p	pseudogene	0	0	1	1	1.000
8924	NM_004667,XM_00	**HERC2**	HECT and RLD domain c	protein_coding	16	13	17	14	0.833
8925	NM_003922,XM_01	**HERC1**	HECT and RLD domain c	protein_coding	32	23	33	24	0.734
8926	NM_005678,NM_02	**SNURF**	SNRPN upstream readin	protein_coding	5	3	6	4	0.627
8927	NM_003458	**BSN**	bassoon presynaptic cyto	protein_coding	0	0	1	1	0.928
8928	NM_003923	**FOXH1**	forkhead box H1	protein_coding	0	0	1	1	0.875
8929	NM_003924	**PHOX2B**	paired like homeobox 2B	protein_coding	0	0	1	1	1.000
8930	NM_001276270,NM	**MBD4**	methyl-CpG binding dom	protein_coding	70	71	71	72	1.006
8932	NM_003927,NM_01	**MBD2**	methyl-CpG binding dom	protein_coding	47	46	48	47	0.967
8933	NM_001078171	**RTL8C**	retrotransposon Gag like	protein_coding	0	0	1	1	1.000
8934	NM_001135662,NM	**RAB29**	RAB29, member RAS on	protein_coding	63	62	64	63	0.979
8935	NM_001303468,NM	**SKAP2**	src kinase associated ph	protein_coding	0	0	1	1	1.008
8936	NM_001024934,NM	**WASF1**	WASP family member 1	protein_coding	0	0	1	1	1.000
8938	NM_001199096,NM	**BAIAP3**	BAI1 associated protein 3	protein_coding	0	0	1	1	1.000
8939	NM_003934,XM_00	**FUBP3**	far upstream element bir	protein_coding	60	52	61	53	0.860
8940	NM_001282112,NM	**TOP3B**	DNA topoisomerase III be	protein_coding	13	13	14	14	0.993
8941	NM_003936	**CDK5R2**	cyclin dependent kinase	protein_coding	0	0	1	1	1.000
8942	NM_001032998,NM	**KYNU**	kynureninase	protein_coding	0	0	1	1	0.814
8943	NM_001261826,NM	**AP3D1**	adaptor related protein c	protein_coding	106	107	107	108	1.013
8944	NR_000007	**SNORD73A**	small nucleolar RNA, C/D	snoRNA	0	0	1	1	0.997
8945	NM_001256856,NM	**BTRC**	beta-transducin repeat c	protein_coding	9	8	10	9	0.886
8968	NM_021018	**H3C7**	H3 clustered histone 7	protein_coding	3	3	4	4	1.067
8969	NM_021064	**H2AC11**	H2A clustered histone 11	protein_coding	7	4	8	5	0.644
8970	NM_021058	**H2BC11**	H2B clustered histone 11	protein_coding	27	26	28	27	0.974
8971	NM_006026	**H1-10**	H1.10 linker histone	protein_coding	6	6	7	7	1.034
8972	NM_001365693,NM	**MGAM**	maltase-glucoamylase	protein_coding	0	0	1	1	0.951
8973	NM_001199279,NM	**CHRNA6**	cholinergic receptor nico	protein_coding	0	0	1	1	0.835
8974	NM_001017974,NM	**P4HA2**	prolyl 4-hydroxylase sub	protein_coding	0	0	1	1	1.020
8975	NM_003940,XM_01	**USP13**	ubiquitin specific peptida	protein_coding	10	9	11	10	0.975
8976	NM_003941	**WASL**	WASP like actin nucleati	protein_coding	3	2	4	3	0.867
8985	NM_001084	**PLOD3**	procollagen-lysine,2-oxo	protein_coding	8	9	9	10	1.129
8986	NM_001006944,NM	**RPS6KA4**	ribosomal protein S6 kina	protein_coding	45	50	46	51	1.110
8987	NM_003943	**STBD1**	starch binding domain 1	protein_coding	6	8	7	9	1.313
8988	NM_006308	**HSPB3**	heat shock protein family	protein_coding	0	0	1	1	1.000
8989	NM_007332,XM_01	**TRPA1**	transient receptor potent	protein_coding	0	0	1	1	1.000
8991	NM_001258288,NM	**SELENBP1**	selenium binding protein	protein_coding	0	0	1	1	1.000
8992	NM_003945	**ATP6V0E1**	ATPase H+ transporting	protein_coding	99	105	100	106	1.064
8993	NM_005091	**PGLYRP1**	peptidoglycan recognitio	protein_coding	0	0	1	1	1.000
8994	NM_014240,XM_01	**LIMD1**	LIM domains containing	protein_coding	23	22	24	23	0.939
8995	NM_005092	**TNFSF18**	TNF superfamily membe	protein_coding	0	0	1	1	1.000
8996	NM_001185057,NM	**NOL3**	nucleolar protein 3	protein_coding	1	2	2	3	1.835
8997	NM_001024660,NM	**KALRN**	kalirin RhoGEF kinase	protein_coding	1	1	2	2	0.983
8999	NM_001330724,NM	**CDKL2**	cyclin dependent kinase	protein_coding	0	0	1	1	0.983
9001	NM_001079870,NM	**HAP1**	huntingtin associated pro	protein_coding	0	0	1	1	1.000
9002	NM_003950,XM_00	**F2RL3**	F2R like thrombin or tryp	protein_coding	0	0	1	1	0.946
9013	NM_001243156,NM	**TAF1C**	TATA-box binding protein	protein_coding	13	15	14	16	1.126
9014	NM_001318976,NM	**TAF1B**	TATA-box binding protein	protein_coding	18	14	19	15	0.765
9015	NM_001201536,NM	**TAF1A**	TATA-box binding protein	protein_coding	25	25	26	26	1.027
9016	NM_001282195,NM	**SLC25A14**	solute carrier family 25 m	protein_coding	17	18	18	19	1.102
9019	NM_001146191,NM	**MPZL1**	myelin protein zero like 1	protein_coding	4	3	5	4	0.891
9020	NM_003954,XM_01	**MAP3K14**	mitogen-activated protein	protein_coding	9	9	10	10	0.938
9021	NM_001378932,NM	**SOCS3**	suppressor of cytokine si	protein_coding	0	0	1	1	0.944
9022	NM_004669,XM_01	**CLIC3**	chloride intracellular cha	protein_coding	0	0	1	1	1.000
9023	NM_003956	**CH25H**	cholesterol 25-hydroxyla	protein_coding	0	0	1	1	1.000
9024	NM_001256627,NM	**BRSK2**	BR serine/threonine kina	protein_coding	0	0	1	1	1.000
9025	NM_003958,NM_18	**RNF8**	ring finger protein 8	protein_coding	15	15	16	16	0.983
9026	NM_001303097,NM	**HIP1R**	huntingtin interacting pro	protein_coding	8	7	9	8	0.877
9027	NM_003960	**NAT8**	N-acetyltransferase 8 (pu	protein_coding	0	0	1	1	1.000
9028	NM_001278720,NM	**RHBDL1**	rhomboid like 1	protein_coding	1	1	2	2	1.179
9031	NM_001370402,NM	**BAZ1B**	bromodomain adjacent to	protein_coding	143	121	144	122	0.844
9032	NM_003963	**TM4SF5**	transmembrane 4 L six fa	protein_coding	0	0	1	1	1.000
9033	NM_001253837,NM	**PKD2L1**	polycystin 2 like 1, transi	protein_coding	0	0	1	1	1.015
9034	NM_001130910,NM	**CCRL2**	C-C motif chemokine rec	protein_coding	4	4	5	5	0.958
9037	NM_003966,XM_00	**SEMA5A**	semaphorin 5A	protein_coding	0	0	1	1	1.000
9038	NM_003967	**TAAR5**	trace amine associated re	protein_coding	0	0	1	1	1.000
9039	NM_001363861,NM	**UBA3**	ubiquitin like modifier act	protein_coding	86	78	87	79	0.915
9040	NM_003969	**UBE2M**	ubiquitin conjugating enz	protein_coding	161	162	162	163	1.005
9043	NM_001130527,NM	**SPAG9**	sperm associated antige	protein_coding	28	24	29	25	0.852
9044	NM_003972,NR_16	**BTAF1**	B-TFIID TATA-box bindin	protein_coding	25	22	26	23	0.888
9045	NM_001034996,NM	**RPL14**	ribosomal protein L14	protein_coding	217	216	218	217	0.995
9046	NM_001317800,NM	**DOK2**	docking protein 2	protein_coding	2	1	3	2	0.903

9047	NM_001161441,NM	**SH2D2A**	SH2 domain containing 2	protein_coding	210	215	211	216	1.021
9048	NM_001136215,NM	**ARTN**	artemin	protein_coding	0	0	1	1	1.000
9049	NM_001302959,NM	**AIP**	aryl hydrocarbon recepto	protein_coding	95	100	96	101	1.045
9050	NM_024430,XM_01	**PSTPIP2**	proline-serine-threonine	protein_coding	13	12	14	13	0.956
9051	NM_001321135,NM	**PSTPIP1**	proline-serine-threonine	protein_coding	82	81	83	82	0.995
9052	NM_003979	**GPRC5A**	G protein-coupled recept	protein_coding	0	0	1	1	1.000
9053	NM_001198608,NM	**MAP7**	microtubule associated p	protein_coding	0	0	1	1	1.000
9054	NM_001198989,NM	**NFS1**	NFS1 cysteine desulfura	protein_coding	27	28	28	29	1.036
9055	NM_001267580,NM	**PRC1**	protein regulator of cytok	protein_coding	89	79	90	80	0.886
9056	NM_001126105,NM	**SLC7A7**	solute carrier family 7 me	protein_coding	0	0	1	1	1.177
9057	NM_001076785,NM	**SLC7A6**	solute carrier family 7 me	protein_coding	35	31	36	32	0.895
9058	NM_001145975,NM	**SLC13A2**	solute carrier family 13 m	protein_coding	0	0	1	1	1.000
9060	NM_001015880,NM	**PAPSS2**	3'-phosphoadenosine 5'-	protein_coding	0	0	1	1	1.000
9061	NM_005443,XM_01	**PAPSS1**	3'-phosphoadenosine 5'-	protein_coding	23	22	24	23	0.989
9063	NM_001324046,NM	**PIAS2**	protein inhibitor of activa	protein_coding	0	0	1	1	1.000
9064	NM_001297609,NM	**MAP3K6**	mitogen-activated protein	protein_coding	0	0	1	1	1.000
9066	NM_001252065,NM	**SYT7**	synaptotagmin 7	protein_coding	0	0	1	1	1.000
9068	NM_001376763,NM	**ANGPTL1**	angiopoietin like 1	protein_coding	0	0	1	1	1.046
9069	NM_001185072,NM	**CLDN12**	claudin 12	protein_coding	6	5	7	6	0.893
9070	NM_001105214,NM	**ASH2L**	ASH2 like, histone lysine	protein_coding	63	74	64	75	1.175
9071	NM_001160100,NM	**CLDN10**	claudin 10	protein_coding	0	0	1	1	1.000
9073	NM_199328	**CLDN8**	claudin 8	protein_coding	0	0	1	1	1.000
9074	NM_021195	**CLDN6**	claudin 6	protein_coding	0	0	1	1	1.000
9075	NM_001171092,NM	**CLDN2**	claudin 2	protein_coding	0	0	1	1	1.000
9076	NM_021101	**CLDN1**	claudin 1	protein_coding	0	0	1	1	1.000
9077	NM_004675	**DIRAS3**	DIRAS family GTPase 3	protein_coding	0	0	1	1	1.000
9079	NM_001130834,NM	**LDB2**	LIM domain binding 2	protein_coding	9	8	10	9	0.863
9080	NM_020982	**CLDN9**	claudin 9	protein_coding	0	0	1	1	1.000
9081	NM_004676,XM_01	**PRY**	PTPN13 like Y-linked	protein_coding	0	0	1	1	1.000
9082	NM_004677	**XKRY**	XK related, Y-linked	protein_coding	0	0	1	1	1.000
9083	NM_004678	**BPY2**	basic charge Y-linked 2	protein_coding	0	0	1	1	1.000
9084	NM_004679	**VCY**	variable charge Y-linked	protein_coding	0	0	1	1	1.000
9085	NM_004680,NM_17	**CDY1**	chromodomain Y-linked	protein_coding	0	0	1	1	1.000
9086	NM_001278612,NM	**EIF1AY**	eukaryotic translation init	protein_coding	239	238	240	239	0.995
9087	NM_004202	**TMSB4Y**	thymosin beta 4 Y-linked	protein_coding	3	4	4	5	1.187
9088	NM_001258450,NM	**PKMYT1**	protein kinase, membran	protein_coding	106	106	107	107	0.998
9091	NM_004204,NM_14	**PIGQ**	phosphatidylinositol glyc	protein_coding	20	22	21	23	1.070
9092	NM_005146,XM_01	**SART1**	spliceosome associated f	protein_coding	131	142	132	143	1.083
9093	NM_001135110,NM	**DNAJA3**	DnaJ heat shock protein	protein_coding	148	150	149	151	1.013
9094	NM_001330166,NM	**UNC119**	unc-119 lipid binding cha	protein_coding	3	3	4	4	0.886
9095	NM_005149	**TBX19**	T-box transcription factor	protein_coding	3	2	4	3	0.804
9096	NM_001080508,XM	**TBX18**	T-box transcription factor	protein_coding	0	0	1	1	1.000
9097	NM_001037334,NM	**USP14**	ubiquitin specific peptida	protein_coding	63	57	64	58	0.909
9098	NM_001304284,NM	**USP6**	ubiquitin specific peptida	protein_coding	0	0	1	1	0.974
9099	NM_001243759,NM	**USP2**	ubiquitin specific peptida	protein_coding	1	1	2	2	0.900
9100	NM_001272075,NM	**USP10**	ubiquitin specific peptida	protein_coding	114	103	115	104	0.912
9101	NM_001128610,NM	**USP8**	ubiquitin specific peptida	protein_coding	62	61	63	62	0.988
9103	NR_047648	**FCGR2C**	Fc fragment of IgG recep	transcribed_pseu	0	0	1	1	1.048
9104	NM_001282848,NM	**RGN**	regucalcin	protein_coding	0	0	1	1	1.000
9107	NM_004685,XM_01	**MTMR6**	myotubularin related pro	protein_coding	43	37	44	38	0.855
9108	NM_004686,XM_00	**MTMR7**	myotubularin related pro	protein_coding	0	0	1	1	0.993
9110	NM_001378066,NM	**MTMR4**	myotubularin related pro	protein_coding	41	36	42	37	0.864
9111	NM_004688,XM_00	**NMI**	N-myc and STAT interac	protein_coding	180	170	181	171	0.944
9112	NM_001203258,NM	**MTA1**	metastasis associated 1	protein_coding	64	60	65	61	0.949
9113	NM_001270519,NM	**LATS1**	large tumor suppressor k	protein_coding	13	12	14	13	0.891
9114	NM_004691,XM_01	**ATP6V0D1**	ATPase H+ transporting	protein_coding	46	54	47	55	1.177
9117	NM_001201572,NM	**SEC22C**	SEC22 homolog C, vesic	protein_coding	29	25	30	26	0.848
9118	NM_032727	**INA**	internexin neuronal interi	protein_coding	0	0	1	1	1.000
9119	NM_004693	**KRT75**	keratin 75	protein_coding	0	0	1	1	1.000
9120	NM_001174166,NM	**SLC16A6**	solute carrier family 16 m	protein_coding	16	16	17	17	0.972
9121	NM_001271765,NM	**SLC16A5**	solute carrier family 16 m	protein_coding	0	0	1	1	1.000
9122	NM_001201546,NM	**SLC16A4**	solute carrier family 16 m	protein_coding	0	0	1	1	0.956
9123	NM_001042422,NM	**SLC16A3**	solute carrier family 16 m	protein_coding	0	0	1	1	1.000
9124	NM_020992,XM_01	**PDLIM1**	PDZ and LIM domain 1	protein_coding	75	85	76	86	1.131
9125	NM_001271634,NM	**CNOT9**	CCR4-NOT transcription	protein_coding	153	161	154	162	1.051
9126	NM_005445	**SMC3**	structural maintenance c	protein_coding	89	79	90	80	0.888
9127	NM_001159554,NM	**P2RX6**	purinergic receptor P2X l	protein_coding	0	0	1	1	0.905
9128	NM_001244926,NM	**PRPF4**	pre-mRNA processing fa	protein_coding	77	75	78	76	0.978
9129	NM_001350529,NM	**PRPF3**	pre-mRNA processing fa	protein_coding	64	63	65	64	0.977
9130	NM_004699	**FAM50A**	family with sequence sim	protein_coding	69	71	70	72	1.036
9131	NM_001130846,NM	**AIFM1**	apoptosis inducing facto	protein_coding	116	111	117	112	0.953
9132	NM_004700,NM_17	**KCNQ4**	potassium voltage-gated	protein_coding	0	0	1	1	1.023
9133	NM_004701	**CCNB2**	cyclin B2	protein_coding	152	152	153	153	1.002
9134	NM_057749,XM_01	**CCNE2**	cyclin E2	protein_coding	21	22	22	23	1.055
9135	NM_001083585,NM	**RABEP1**	rabaptin, RAB GTPase b	protein_coding	30	27	31	28	0.917
9136	NM_004704	**RRP9**	ribosomal RNA processin	protein_coding	133	143	134	144	1.079
9138	NM_004706,NM_19	**ARHGEF1**	Rho guanine nucleotide	protein_coding	171	172	172	173	1.010
9139	NM_001032999,NM	**CBFA2T2**	CBFA2/RUNX1 partner t	protein_coding	6	6	7	7	0.966
9140	NM_001277783,NM	**ATG12**	autophagy related 12	protein_coding	40	36	41	37	0.888
9141	NM_004708,XM_00	**PDCD5**	programmed cell death 5	protein_coding	351	349	352	350	0.996
9143	NM_004209	**SYNGR3**	synaptogyrin 3	protein_coding	0	0	1	1	1.000
9144	NM_001320523,NM	**SYNGR2**	synaptogyrin 2	protein_coding	160	171	161	172	1.071
9145	NM_004711,NM_14	**SYNGR1**	synaptogyrin 1	protein_coding	0	0	1	1	1.000
9146	NM_004712	**HGS**	hepatocyte growth factor	protein_coding	48	48	49	49	1.000
9147	NM_001301732,NM	**NEMF**	nuclear export mediator f	protein_coding	29	26	30	27	0.890
9148	NM_004210,XM_00	**NEURL1**	neuralized E3 ubiquitin p	protein_coding	0	0	1	1	1.000
9149	NM_004714,NM_00	**DYRK1B**	dual specificity tyrosine p	protein_coding	3	3	4	4	1.016
9150	NM_001202504,NM	**CTDP1**	CTD phosphatase subur	protein_coding	30	31	31	32	1.040
9152	NM_001318369,NM	**SLC6A5**	solute carrier family 6 me	protein_coding	0	0	1	1	0.898
9153	NM_004212,XM_01	**SLC28A2**	solute carrier family 28 m	protein_coding	0	0	1	1	1.000
9154	NM_001287761,NM	**SLC28A1**	solute carrier family 28 m	protein_coding	0	0	1	1	1.000
9156	NM_001319224,NM	**EXO1**	exonuclease 1	protein_coding	38	34	39	35	0.898
9158	NM_004214,NM_19	**FIBP**	FGF1 intracellular bindin	protein_coding	65	67	66	68	1.026
9159	NM_004716,XM_00	**PCSK7**	proprotein convertase su	protein_coding	64	66	65	67	1.027
9162	NM_001321708,NM	**DGKI**	diacylglycerol kinase iota	protein_coding	0	0	1	1	1.000
9166	NM_001278938,NM	**EBAG9**	estrogen receptor bindin	protein_coding	38	37	39	38	0.977
9167	NM_001319036,NM	**COX7A2L**	cytochrome c oxidase su	protein_coding	114	123	115	124	1.080
9168	NM_021103	**TMSB10**	thymosin beta 10	protein_coding	3,392	3,820	3,393	3,821	1.126
9169	NM_004719,XM_00	**SCAF11**	SR-related CTD associat	protein_coding	44	38	45	39	0.872
9170	NM_004720,XM_01	**LPAR2**	lysophosphatidic acid re	protein_coding	5	6	6	7	1.090
9172	NM_003970,XM_00	**MYOM2**	myomesin 2	protein_coding	0	0	1	1	1.000
9173	NM_001282408,NM	**IL1RL1**	interleukin 1 receptor like	protein_coding	0	0	1	1	0.956
9175	NM_001242314,NM	**MAP3K13**	mitogen-activated protein	protein_coding	25	23	26	24	0.932
9177	NM_001363563,NM	**HTR3B**	5-hydroxytryptamine rece	protein_coding	0	0	1	1	1.000
9179	NM_001363671,NM	**AP4M1**	adaptor related protein c	protein_coding	6	7	7	8	1.219
9180	NM_001168355,NM	**OSMR**	oncostatin M receptor	protein_coding	0	0	1	1	1.000
9181	NM_001162383,NM	**ARHGEF2**	Rho/Rac guanine nucleo	protein_coding	65	62	66	63	0.957
9182	NM_005447,XM_01	**RASSF9**	Ras association domain f	protein_coding	0	0	1	1	1.000
9183	NM_004724,XM_01	**ZW10**	zw10 kinetochore protein	protein_coding	49	45	50	46	0.928
9184	NM_001007793,NM	**BUB3**	BUB3 mitotic checkpoint	protein_coding	101	114	102	115	1.131
9185	NM_001080975,NM	**REPS2**	RALBP1 associated Eps	protein_coding	0	0	1	1	1.000
9187	NM_001254740,NM	**SLC24A1**	solute carrier family 24 m	protein_coding	1	1	2	2	1.059
9188	NM_001256910,NM	**DDX21**	DExD-box helicase 21	protein_coding	343	300	344	301	0.877
9189	NM_001171135,NM	**ZBED1**	zinc finger BED-type con	protein_coding	12	12	13	13	0.988
9191	NM_001039711,NM	**DEDD**	death effector domain co	protein_coding	31	36	32	37	1.158
9194	NM_001270622,NM	**SLC16A7**	solute carrier family 16 m	protein_coding	5	4	6	5	0.729
9196	NM_004732,XM_01	**KCNAB3**	potassium voltage-gated	protein_coding	1	2	2	3	1.265
9197	NM_001190992,NM	**SLC33A1**	solute carrier family 33 m	protein_coding	13	9	14	10	0.722
9200	NM_014241,XM_00	**HACD1**	3-hydroxyacyl-CoA dehy	protein_coding	0	0	1	1	1.000
9201	NM_001195415,NM	**DCLK1**	doublecortin like kinase 1	protein_coding	0	0	1	1	0.894
9202	NM_001350138,NM	**ZMYM4**	zinc finger MYM-type cor	protein_coding	31	27	32	28	0.895
9203	NM_001171162,NM	**ZMYM3**	zinc finger MYM-type cor	protein_coding	28	30	29	31	1.072
9204	NM_007167	**ZMYM6**	zinc finger MYM-type cor	protein_coding	13	12	14	13	0.978
9205	NM_001039649,NM	**ZMYM5**	zinc finger MYM-type cor	protein_coding	22	18	23	19	0.812
9206	gene-RAD17P2	**RAD17P2**	RAD17 pseudogene 2	pseudogene	0	0	1	1	1.000
9207	gene-RAD17P1	**RAD17P1**	RAD17 pseudogene 1	pseudogene	0	0	1	1	1.000

9208	NM_001137550,NM	**LRRFIP1**	LRR binding FLII interact	protein_coding	272	185	273	186	0.684
9209	NM_001134369,NM	**LRRFIP2**	LRR binding FLII interact	protein_coding	32	30	33	31	0.966
9210	NM_005448	**BMP15**	bone morphogenetic pro	protein_coding	0	0	1	1	1.000
9211	NM_001308275,NM	**LGI1**	leucine rich glioma inacti	protein_coding	0	0	1	1	1.000
9212	NM_001256834,NM	**AURKB**	aurora kinase B	protein_coding	120	122	121	123	1.018
9213	NM_001135669,NM	**XPR1**	xenotropic and polytropic	protein_coding	27	23	28	24	0.846
9214	NM_001142473,NM	**FCMR**	Fc fragment of IgM recep	protein_coding	11	14	12	15	1.194
9215	NM_001362949,NM	**LARGE1**	LARGE xylosyl- and gluc	protein_coding	0	0	1	1	1.000
9217	NM_001195677,NM	**VAPB**	VAMP associated protein	protein_coding	23	22	24	23	0.972
9218	NM_003574,NM_19	**VAPA**	VAMP associated protein	protein_coding	34	34	35	35	1.021
9219	NM_001330292,NM	**MTA2**	metastasis associated 1	protein_coding	221	230	222	231	1.041
9220	NM_004740	**TIAF1**	TGFB1-induced anti-apo	protein_coding	0	0	1	1	0.934
9221	NM_001284388,NM	**NOLC1**	nucleolar and coiled-bod	protein_coding	321	310	322	311	0.968
9223	NM_001033057,NM	**MAGI1**	membrane associated gu	protein_coding	0	0	1	1	1.054
9227	NM_001301645,NM	**LRAT**	lecithin retinol acyltransf	protein_coding	0	0	1	1	1.000
9228	NM_001346810,NR	**DLGAP2**	DLG associated protein 2	protein_coding	0	0	1	1	1.000
9229	NM_001003809,NM	**DLGAP1**	DLG associated protein 1	protein_coding	0	0	1	1	1.000
9230	NM_004218	**RAB11B**	RAB11B, member RAS o	protein_coding	81	95	82	96	1.163
9231	NM_004747,XM_00	**DLG5**	discs large MAGUK scaf	protein_coding	7	7	8	8	0.922
9232	NM_001282382,NM	**PTTG1**	PTTG1 regulator of siste	protein_coding	302	292	303	293	0.966
9235	NM_001012631,NM	**IL32**	interleukin 32	protein_coding	2,644	2,812	2,645	2,813	1.064
9236	NM_001204450,NM	**CCPG1**	cell cycle progression 1	protein_coding	12	10	13	11	0.857
9238	NM_001261834,NM	**TBRG4**	transforming growth fact	protein_coding	69	68	70	69	0.984
9240	NM_006029	**PNMA1**	PNMA family member 1	protein_coding	45	46	46	47	1.021
9241	NM_005450	**NOG**	noggin	protein_coding	0	0	1	1	1.000
9242	NM_005098	**MSC**	musculin	protein_coding	34	40	35	41	1.185
9244	NM_004750	**CRLF1**	cytokine receptor like fac	protein_coding	0	0	1	1	0.995
9245	NM_004751	**GCNT3**	glucosaminyl (N-acetyl) t	protein_coding	0	0	1	1	1.000
9246	NM_004223,NM_19	**UBE2L6**	ubiquitin conjugating enz	protein_coding	276	275	277	276	0.994
9247	NM_004752	**GCM2**	glial cells missing transc	protein_coding	0	0	1	1	1.000
9248	NM_004224,XM_01	**GPR50**	G protein-coupled recept	protein_coding	0	0	1	1	1.000
9249	NM_001319225,NM	**DHRS3**	dehydrogenase/reductas	protein_coding	0	0	1	1	1.015
9252	NM_001322227,NM	**RPS6KA5**	ribosomal protein S6 kina	protein_coding	7	5	8	6	0.828
9253	NM_001289979,NM	**NUMBL**	NUMB like endocytic ada	protein_coding	2	2	3	3	0.982
9254	NM_001005505,NM	**CACNA2D2**	calcium voltage-gated ch	protein_coding	0	0	1	1	1.000
9255	NM_001142415,NM	**AIMP1**	aminoacyl tRNA syntheta	protein_coding	64	59	65	60	0.919
9256	NM_001261835,NM	**TSPOAP1**	TSPO associated protein	protein_coding	4	5	5	6	1.156
9258	NM_004225,XM_01	**MFHAS1**	malignant fibrous histioc	protein_coding	0	0	1	1	1.000
9260	NM_005451,NM_20	**PDLIM7**	PDZ and LIM domain 7	protein_coding	6	5	7	6	0.884
9261	NM_004759,NM_03	**MAPKAPK2**	MAPK activated protein k	protein_coding	127	133	128	134	1.045
9262	NM_004226,XM_01	**STK17B**	serine/threonine kinase 1	protein_coding	200	155	201	156	0.774
9263	NM_004760,XM_01	**STK17A**	serine/threonine kinase 1	protein_coding	27	21	28	22	0.810
9265	NM_001367580,NM	**CYTH3**	cytohesin 3	protein_coding	4	3	5	4	0.862
9266	NM_004228,NM_01	**CYTH2**	cytohesin 2	protein_coding	21	21	22	22	1.023
9267	NM_001292018,NM	**CYTH1**	cytohesin 1	protein_coding	28	27	29	28	0.975
9270	NM_001319066,NM	**ITGB1BP1**	integrin subunit beta 1 b	protein_coding	41	47	42	48	1.130
9271	NM_001190971,NM	**PIWIL1**	piwi like RNA-mediated g	protein_coding	0	0	1	1	1.000
9274	NM_001286526,NM	**BCL7C**	BAF chromatin remodelin	protein_coding	36	45	37	46	1.235
9275	NM_001197244,NM	**BCL7B**	BAF chromatin remodelin	protein_coding	146	147	147	148	1.003
9276	NM_004766,NR_02	**COPB2**	COPI coat complex subu	protein_coding	148	128	149	129	0.870
9277	NM_001164267,NM	**WDR46**	WD repeat domain 46	protein_coding	131	134	132	135	1.024
9278	NM_001145338,NM	**ZBTB22**	zinc finger and BTB dom	protein_coding	2	3	3	4	1.273
9282	NM_004229,XM_00	**MED14**	mediator complex subun	protein_coding	31	23	32	24	0.742
9283	NM_004767,XM_01	**GPR37L1**	G protein-coupled recept	protein_coding	0	0	1	1	1.000
9284	NM_006985	**NPIPA1**	nuclear pore complex int	protein_coding	1	1	2	2	0.962
9287	NM_001033080,NM	**TAAR2**	trace amine associated r	protein_coding	0	0	1	1	1.000
9288	NR_028511	**TAAR3P**	trace amine associated r	transcribed_pseu	0	0	1	1	1.000
9289	NM_001145770,NM	**ADGRG1**	adhesion G protein-coup	protein_coding	1	1	2	2	1.194
9290	NM_005683,XM_00	**GPR55**	G protein-coupled recept	protein_coding	0	0	1	1	0.896
9293	NM_005684	**GPR52**	G protein-coupled recept	protein_coding	0	0	1	1	0.859
9294	NM_004230	**S1PR2**	sphingosine-1-phosphate	protein_coding	0	0	1	1	0.841
9295	NM_001190987,NM	**SRSF11**	serine and arginine rich s	protein_coding	206	178	207	179	0.865
9296	NM_001198909,NM	**ATP6V1F**	ATPase H+ transporting	protein_coding	289	312	290	313	1.079
9297	NR_002559	**SNORD29**	small nucleolar RNA, C/D	snoRNA	29	24	30	25	0.832
9298	NR_002560	**SNORD31**	small nucleolar RNA, C/D	snoRNA	19	23	20	24	1.162
9299	NR_002561	**SNORD30**	small nucleolar RNA, C/D	snoRNA	22	19	23	20	0.895
9300	NR_002562	**SNORD28**	small nucleolar RNA, C/D	snoRNA	19	17	20	18	0.895
9301	NR_002563	**SNORD27**	small nucleolar RNA, C/D	snoRNA	15	13	16	14	0.857
9302	NR_002564	**SNORD26**	small nucleolar RNA, C/D	snoRNA	21	17	22	18	0.804
9303	NR_002565	**SNORD25**	small nucleolar RNA, C/D	snoRNA	0	0	1	1	0.972
9304	NR_000008	**SNORD22**	small nucleolar RNA, C/D	snoRNA	28	28	29	29	0.998
9306	NM_004232,XM_00	**SOCS6**	suppressor of cytokine si	protein_coding	0	0	1	1	1.000
9308	NM_001040280,NM	**CD83**	CD83 molecule	protein_coding	11	10	12	11	0.951
9310	NM_004234,XM_00	**ZNF235**	zinc finger protein 235	protein_coding	0	0	1	1	1.000
9311	NM_004769,NM_02	**ASIC3**	acid sensing ion channel	protein_coding	0	0	1	1	1.203
9312	NM_004770,XM_01	**KCNB2**	potassium voltage-gated	protein_coding	0	0	1	1	1.000
9313	NM_004771	**MMP20**	matrix metallopeptidase 2	protein_coding	0	0	1	1	1.000
9314	NM_001314052,NM	**KLF4**	Kruppel like factor 4	protein_coding	0	0	1	1	1.000
9315	NM_001142474,NM	**NREP**	neuronal regeneration re	protein_coding	0	0	1	1	1.000
9317	NM_001001484,NM	**PTER**	phosphotriesterase relate	protein_coding	0	0	1	1	1.066
9318	NM_001143887,NM	**COPS2**	COP9 signalosome subu	protein_coding	76	70	77	71	0.924
9319	NM_001166260,NM	**TRIP13**	thyroid hormone recepto	protein_coding	84	82	85	83	0.973
9320	NM_001284214,NM	**TRIP12**	thyroid hormone recepto	protein_coding	100	85	101	86	0.859
9321	NM_001321851,NM	**TRIP11**	thyroid hormone recepto	protein_coding	9	8	10	9	0.884
9322	NM_001288962,NM	**TRIP10**	thyroid hormone recepto	protein_coding	9	7	10	8	0.788
9324	NM_001201362,NM	**HMGN3**	high mobility group nucle	protein_coding	0	0	1	1	1.000
9325	NM_001321924,NM	**TRIP4**	thyroid hormone recepto	protein_coding	27	26	28	27	0.963
9326	NM_001281432,NM	**ZNHIT3**	zinc finger HIT-type cont	protein_coding	136	148	137	149	1.086
9328	NM_001122823,NM	**GTF3C5**	general transcription fact	protein_coding	78	80	79	81	1.032
9329	NM_012204,NR_13	**GTF3C4**	general transcription fact	protein_coding	22	19	23	20	0.864
9330	NM_001206774,NM	**GTF3C3**	general transcription fact	protein_coding	46	43	47	44	0.948
9331	NM_001330570,NM	**B4GALT6**	beta-1,4-galactosyltransf	protein_coding	3	3	4	4	1.176
9332	NM_001370145,NM	**CD163**	CD163 molecule	protein_coding	0	0	1	1	0.982
9333	NM_004245,NM_20	**TGM5**	transglutaminase 5	protein_coding	0	0	1	1	1.000
9334	NM_004776	**B4GALT5**	beta-1,4-galactosyltransf	protein_coding	153	152	154	153	0.996
9337	NM_001301073,NM	**CNOT8**	CCR4-NOT transcription	protein_coding	52	50	53	51	0.963
9338	NM_001006639,NM	**TCEAL1**	transcription elongation f	protein_coding	0	0	1	1	1.000
9340	NM_004246,XM_00	**GLP2R**	glucagon like peptide 2 r	protein_coding	0	0	1	1	1.000
9341	NM_004781,XM_01	**VAMP3**	vesicle associated memb	protein_coding	22	23	23	24	1.042
9342	NM_004782	**SNAP29**	synaptosome associated	protein_coding	34	32	35	33	0.941
9343	NM_001142605,NM	**EFTUD2**	elongation factor Tu GTP	protein_coding	187	178	188	179	0.956
9344	NM_001252043,NM	**TAOK2**	TAO kinase 2	protein_coding	44	44	45	45	1.006
9348	NM_004784,NR_14	**NDST3**	N-deacetylase and N-sul	protein_coding	0	0	1	1	1.000
9349	NM_000978	**RPL23**	ribosomal protein L23	protein_coding	694	720	695	721	1.038
9350	NM_005454,XR_00	**CER1**	cerberus 1, DAN family B	protein_coding	0	0	1	1	1.000
9351	NM_001130012,NM	**SLC9A3R2**	SLC9A3 regulator 2	protein_coding	0	0	1	1	1.000
9352	NM_004786,NR_02	**TXNL1**	thioredoxin like 1	protein_coding	40	35	41	36	0.882
9353	NM_001289135,NM	**SLIT2**	slit guidance ligand 2	protein_coding	0	0	1	1	1.000
9354	NM_001204077,NM	**UBE4A**	ubiquitination factor E4A	protein_coding	44	41	45	42	0.931
9355	NM_004789,XM_00	**LHX2**	LIM homeobox 2	protein_coding	0	0	1	1	1.000
9356	NM_004790,NM_15	**SLC22A6**	solute carrier family 22 m	protein_coding	0	0	1	1	1.000
9358	NM_001271754,NM	**ITGBL1**	integrin subunit beta like	protein_coding	0	0	1	1	1.000
9360	NM_004792,XM_00	**PPIG**	peptidylprolyl isomerase	protein_coding	77	67	78	68	0.873
9361	NM_001276479,NM	**LONP1**	lon peptidase 1, mitocho	protein_coding	114	120	115	121	1.054
9362	NM_001280558,NM	**CPNE6**	copine 6	protein_coding	0	0	1	1	1.000
9363	NM_004794,XM_01	**RAB33A**	RAB33A, member RAS o	protein_coding	48	45	49	46	0.926
9364	NM_001017979,NM	**RAB28**	RAB28, member RAS on	protein_coding	38	34	39	35	0.913
9365	NM_004795,XM_00	**KL**	klotho	protein_coding	0	0	1	1	1.000
9366	NR_000039	**RAB9BP1**	RAB9B, member RAS on	transcribed_pseu	0	0	1	1	1.000
9367	NM_001195328,NM	**RAB9A**	RAB9A, member RAS on	protein_coding	21	19	22	20	0.922
9368	NM_004252,XR_00	**SLC9A3R1**	SLC9A3 regulator 1	protein_coding	414	403	415	404	0.973
9369	NM_001105250,NM	**NRXN3**	neurexin 3	protein_coding	0	0	1	1	0.971
9370	NM_001177800,NM	**ADIPOQ**	adiponectin, C1Q and cc	protein_coding	0	0	1	1	1.000
9371	NM_004798,XR_00	**KIF3B**	kinesin family member 3E	protein_coding	34	29	35	30	0.856
9372	NM_004799,NM_00	**ZFYVE9**	zinc finger FYVE-type cc	protein_coding	0	0	1	1	1.001
9373	NM_001031689,NM	**PLAA**	phospholipase A2 activat	protein_coding	35	33	36	34	0.939

9374	NM_001204103,NM	**PPT2**	palmitoyl-protein thioeste	protein_coding	4	3	5	4	0.877
9375	NM_004800	**TM9SF2**	transmembrane 9 superf	protein_coding	105	94	106	95	0.895
9376	NM_001184732,NM	**SLC22A8**	solute carrier family 22 m	protein_coding	0	0	1	1	1.000
9377	NM_004255	**COX5A**	cytochrome c oxidase su	protein_coding	280	303	281	304	1.085
9378	NM_001135659,NM	**NRXN1**	neurexin 1	protein_coding	0	0	1	1	0.990
9379	NM_001376262,NM	**NRXN2**	neurexin 2	protein_coding	1	1	2	2	0.918
9380	NM_012203,XM_00	**GRHPR**	glyoxylate and hydroxyp	protein_coding	44	51	45	52	1.167
9381	NM_001287489,NM	**OTOF**	otoferlin	protein_coding	0	0	1	1	1.000
9382	NM_018714	**COG1**	component of oligomeric	protein_coding	13	15	14	16	1.151
9383	NR_003255	**TSIX**	TSIX transcript, XIST ant	antisense_RNA	0	0	1	1	1.000
9384	gene-COX6CP1	**COX6CP1**	cytochrome c oxidase su	pseudogene	0	0	1	1	1.163
9385	gene-COX5AP1	**COX5AP1**	cytochrome c oxidase su	pseudogene	0	0	1	1	1.000
9386	gene-COX7CP1	**COX7CP1**	cytochrome c oxidase su	pseudogene	0	0	1	1	0.892
9388	NM_001308006,NM	**LIPG**	lipase G, endothelial typ	protein_coding	0	0	1	1	1.010
9389	NM_001320033,NM	**SLC22A14**	solute carrier family 22 m	protein_coding	0	0	1	1	1.000
9390	NM_004256	**SLC22A13**	solute carrier family 22 m	protein_coding	0	0	1	1	0.995
9391	NM_004804,XM_02	**CIAO1**	cytosolic iron-sulfur asse	protein_coding	81	76	82	77	0.941
9392	NM_001142621,NM	**TGFBRAP1**	transforming growth fact	protein_coding	37	32	38	33	0.879
9394	NM_004807	**HS6ST1**	heparan sulfate 6-O-sulf	protein_coding	0	0	1	1	1.000
9397	NM_001308295,NM	**NMT2**	N-myristoyltransferase 2	protein_coding	6	6	7	7	0.956
9398	NM_001256106,NM	**CD101**	CD101 molecule	protein_coding	0	0	1	1	0.945
9399	NM_001256672,NM	**STOML1**	stomatin like 1	protein_coding	6	9	7	10	1.496
9400	NM_001003715,NM	**RECQL5**	RecQ like helicase 5	protein_coding	16	19	17	20	1.154
9401	NM_004260,XM_01	**RECQL4**	RecQ like helicase 4	protein_coding	18	19	19	20	1.069
9402	NM_001291824,NM	**GRAP2**	GRB2 related adaptor pr	protein_coding	37	30	38	31	0.822
9403	NM_004261,NM_20	**SELENOF**	selenoprotein F	protein_coding	263	253	264	254	0.961
9404	NM_001143995,NM	**LPXN**	leupaxin	protein_coding	194	193	195	194	0.995
9406	NM_005455,NM_20	**ZRANB2**	zinc finger RANBP2-type	protein_coding	223	183	224	184	0.824
9407	NM_004262,XM_00	**TMPRSS11D**	transmembrane serine p	protein_coding	0	0	1	1	1.000
9409	NM_004813,NM_05	**PEX16**	peroxisomal biogenesis f	protein_coding	48	52	49	53	1.069
9410	NM_004814	**SNRNP40**	small nuclear ribonucleo	protein_coding	228	219	229	220	0.958
9411	NM_001328664,NM	**ARHGAP29**	Rho GTPase activating p	protein_coding	0	0	1	1	1.000
9412	NM_001271811,NM	**MED21**	mediator complex subun	protein_coding	32	28	33	29	0.859
9413	NM_001127608,NM	**FAM189A2**	family with sequence sim	protein_coding	0	0	1	1	1.000
9414	NM_001170414,NM	**TJP2**	tight junction protein 2	protein_coding	1	1	2	2	0.849
9415	NM_001281501,NM	**FADS2**	fatty acid desaturase 2	protein_coding	6	6	7	7	1.015
9416	NM_004818	**DDX23**	DEAD-box helicase 23	protein_coding	131	133	132	134	1.010
9419	NM_014171	**CRIPT**	CXXC repeat containing	protein_coding	3	4	4	5	1.136
9420	NM_001324112,NM	**CYP7B1**	cytochrome P450 family	protein_coding	0	0	1	1	1.000
9421	NM_004821,XM_00	**HAND1**	heart and neural crest de	protein_coding	0	0	1	1	1.000
9422	NM_003417,XM_01	**ZNF264**	zinc finger protein 264	protein_coding	9	7	10	8	0.772
9423	NM_004822,XM_00	**NTN1**	netrin 1	protein_coding	0	0	1	1	1.000
9424	NM_004823,XM_01	**KCNK6**	potassium two pore dom	protein_coding	0	0	1	1	1.000
9425	NM_001143970,NM	**CDYL**	chromodomain Y like	protein_coding	47	43	48	44	0.913
9426	NM_004825	**CDY2A**	chromodomain Y-linked 2	protein_coding	0	0	1	1	1.000
9427	NM_001290787,NM	**ECEL1**	endothelin converting en	protein_coding	0	0	1	1	1.000
9429	NM_001257386,NM	**ABCG2**	ATP binding cassette sul	protein_coding	0	0	1	1	1.000
9430	gene-MYLKP1	**MYLKP1**	myosin light chain kinase	pseudogene	0	0	1	1	1.000
9435	NM_004267	**CHST2**	carbohydrate sulfotransf	protein_coding	23	23	24	24	0.982
9436	NM_001199509,NM	**NCR2**	natural cytotoxicity trigge	protein_coding	31	35	32	36	1.107
9437	NM_001145457,NM	**NCR1**	natural cytotoxicity trigge	protein_coding	74	62	75	63	0.830
9439	NM_001270521,NM	**MED23**	mediator complex subun	protein_coding	13	12	14	13	0.943
9440	NM_004268	**MED17**	mediator complex subun	protein_coding	19	17	20	18	0.925
9441	NM_004831	**MED26**	mediator complex subun	protein_coding	21	21	22	22	1.001
9442	NM_001253881,NM	**MED27**	mediator complex subun	protein_coding	49	52	50	53	1.073
9443	NM_001100816,NM	**MED7**	mediator complex subun	protein_coding	24	22	25	23	0.910
9444	NM_001301085,NM	**QKI**	QKI, KH domain containi	protein_coding	11	8	12	9	0.754
9445	NM_021999	**ITM2B**	integral membrane prote	protein_coding	26	23	27	24	0.902
9446	NM_001191002,NM	**GSTO1**	glutathione S-transferase	protein_coding	137	132	138	133	0.962
9447	NM_001348247,NM	**AIM2**	absent in melanoma 2	protein_coding	42	38	43	39	0.915
9448	NM_001242559,NM	**MAP4K4**	mitogen-activated protein	protein_coding	11	11	12	12	1.002
9450	NM_004271,XM_01	**LY86**	lymphocyte antigen 86	protein_coding	0	0	1	1	1.000
9451	NM_001313915,NM	**EIF2AK3**	eukaryotic translation init	protein_coding	24	22	25	23	0.935
9452	NM_001171581,NM	**ITM2A**	integral membrane prote	protein_coding	0	0	1	1	1.000
9453	NM_001037277,NM	**GGPS1**	geranylgeranyl diphosph	protein_coding	14	13	15	14	0.961
9454	NM_001145721,NM	**HOMER3**	homer scaffold protein 3	protein_coding	0	0	1	1	1.000
9455	NM_004839,NM_19	**HOMER2**	homer scaffold protein 2	protein_coding	1	1	2	2	0.975
9456	NM_001277077,NM	**HOMER1**	homer scaffold protein 1	protein_coding	0	0	1	1	1.000
9457	NM_001170807,NM	**FHL5**	four and a half LIM doma	protein_coding	0	0	1	1	1.000
9459	NM_001306177,NM	**ARHGEF6**	Rac/Cdc42 guanine nucl	protein_coding	55	48	56	49	0.866
9462	NM_004841,NM_17	**RASAL2**	RAS protein activator like	protein_coding	0	0	1	1	1.000
9463	NM_001039583,NM	**PICK1**	protein interacting with P	protein_coding	20	22	21	23	1.071
9464	NM_021973	**HAND2**	heart and neural crest de	protein_coding	0	0	1	1	1.000
9465	NM_001376570,NM	**AKAP7**	A-kinase anchoring prote	protein_coding	0	0	1	1	0.924
9466	NM_004843	**IL27RA**	interleukin 27 receptor su	protein_coding	23	23	24	24	0.995
9467	NM_001018009,NM	**SH3BP5**	SH3 domain binding prot	protein_coding	12	14	13	15	1.156
9468	NM_001163264,NM	**PCYT1B**	phosphate cytidylyltransf	protein_coding	0	0	1	1	1.000
9469	NM_004273,XM_00	**CHST3**	carbohydrate sulfotransf	protein_coding	0	0	1	1	1.000
9470	NM_001276336,NM	**EIF4E2**	eukaryotic translation init	protein_coding	164	172	165	173	1.045
9472	NM_004274,XM_01	**AKAP6**	A-kinase anchoring prote	protein_coding	0	0	1	1	0.963
9473	NM_001039477,NM	**THEMIS2**	thymocyte selection asso	protein_coding	0	0	1	1	1.000
9474	NM_001286106,NM	**ATG5**	autophagy related 5	protein_coding	41	38	42	39	0.946
9475	NM_001321643,NM	**ROCK2**	Rho associated coiled-co	protein_coding	12	10	13	11	0.844
9476	NM_004851,XM_01	**NAPSA**	napsin A aspartic peptida	protein_coding	5	5	6	6	0.993
9477	NM_001305455,NM	**MED20**	mediator complex subun	protein_coding	50	52	51	53	1.056
9478	NM_001033677,NM	**CABP1**	calcium binding protein 1	protein_coding	0	0	1	1	1.000
9479	NM_005456	**MAPK8IP1**	mitogen-activated protein	protein_coding	0	0	1	1	0.992
9480	NM_004852	**ONECUT2**	one cut homeobox 2	protein_coding	0	0	1	1	1.000
9481	NM_001204051,NM	**SLC25A27**	solute carrier family 25 m	protein_coding	0	0	1	1	1.000
9482	NM_004853,NR_03	**STX8**	syntaxin 8	protein_coding	23	27	24	28	1.135
9486	NM_004854,XM_01	**CHST10**	carbohydrate sulfotransf	protein_coding	0	0	1	1	1.000
9487	NM_004278,XM_01	**PIGL**	phosphatidylinositol glyc	protein_coding	5	5	6	6	0.985
9488	NM_004855,XM_00	**PIGB**	phosphatidylinositol glyc	protein_coding	27	25	28	26	0.945
9489	NM_024419,NR_11	**PGS1**	phosphatidylglycerophos	protein_coding	21	19	22	20	0.914
9491	NM_001323407,NM	**PSMF1**	proteasome inhibitor sub	protein_coding	47	49	48	50	1.034
9493	NM_001281301,NM	**KIF23**	kinesin family member 23	protein_coding	85	79	86	80	0.934
9495	NM_004857	**AKAP5**	A-kinase anchoring prote	protein_coding	1	1	2	2	0.915
9496	NM_001321120,NM	**TBX4**	T-box transcription factor	protein_coding	0	0	1	1	1.000
9497	NM_001258379,NM	**SLC4A7**	solute carrier family 4 me	protein_coding	41	31	42	32	0.761
9498	NM_001039960,NM	**SLC4A8**	solute carrier family 4 me	protein_coding	6	4	7	5	0.760
9499	NM_001135940,NM	**MYOT**	myotilin	protein_coding	0	0	1	1	1.000
9500	NM_001005332,NM	**MAGED1**	MAGE family member D1	protein_coding	0	0	1	1	1.000
9501	NM_001190411,NM	**RPH3AL**	rabphilin 3A like (without	protein_coding	0	0	1	1	1.000
9502	NM_130777,XM_01	**XAGE2**	X antigen family member	protein_coding	0	0	1	1	1.000
9506	NM_001318877,NM	**PAGE4**	PAGE family member 4	protein_coding	0	0	1	1	1.000
9507	NM_001320336,NM	**ADAMTS4**	ADAM metallopeptidase	protein_coding	0	0	1	1	0.984
9508	NM_014243,XM_01	**ADAMTS3**	ADAM metallopeptidase	protein_coding	0	0	1	1	1.000
9509	NM_014244,NM_02	**ADAMTS2**	ADAM metallopeptidase	protein_coding	0	0	1	1	1.039
9510	NM_006988	**ADAMTS1**	ADAM metallopeptidase	protein_coding	0	0	1	1	1.000
9512	NM_004279,XM_00	**PMPCB**	peptidase, mitochondrial	protein_coding	57	57	58	58	1.002
9513	NM_004860,XR_24	**FXR2**	FMR1 autosomal homolo	protein_coding	21	21	22	22	1.039
9514	NM_001318103,NM	**GAL3ST1**	galactose-3-O-sulfotrans	protein_coding	0	0	1	1	1.000
9515	NM_001308330,NM	**STXBP5L**	syntaxin binding protein	protein_coding	0	0	1	1	1.010
9516	NM_001136472,NM	**LITAF**	lipopolysaccharide induc	protein_coding	22	23	23	24	1.031
9517	NM_004863,XM_01	**SPTLC2**	serine palmitoyltransfera	protein_coding	39	35	40	36	0.898
9518	NM_004864,XM_02	**GDF15**	growth differentiation fac	protein_coding	0	0	1	1	1.212
9519	NM_001253676,NM	**TBPL1**	TATA-box binding protein	protein_coding	22	22	23	23	0.995
9520	NM_001330257,NM	**NPEPPS**	aminopeptidase puromyc	protein_coding	68	64	69	65	0.937
9521	NM_001135650,NM	**EEF1E1**	eukaryotic translation elo	protein_coding	191	181	192	182	0.948
9522	NM_001290229,NM	**SCAMP1**	secretory carrier membra	protein_coding	14	12	15	13	0.832
9524	NM_001321170,NM	**TECR**	trans-2,3-enoyl-CoA redu	protein_coding	90	98	91	99	1.087
9525	NM_004869,XM_00	**VPS4B**	vacuolar protein sorting 4	protein_coding	64	56	65	57	0.885
9526	NM_001330073,NM	**MPDU1**	mannose-P-dolichol utiliz	protein_coding	75	73	76	74	0.979
9527	NM_001007024,NM	**GOSR1**	golgi SNAP receptor com	protein_coding	43	40	44	41	0.929
9528	NM_001305043,NM	**TMEM59**	transmembrane protein 5	protein_coding	43	39	44	40	0.915
9529	NM_001015048,NM	**BAG5**	BAG cochaperone 5	protein_coding	41	38	42	39	0.942

9530	NM_001204878,NM	**BAG4**	BAG cochaperone 4	protein_coding	41	38	42	39	0.948
9531	NM_004281,XM_00!	**BAG3**	BAG cochaperone 3	protein_coding	1	2	2	3	1.461
9532	NM_004282,XM_00!	**BAG2**	BAG cochaperone 2	protein_coding	12	11	13	12	0.964
9533	NM_001318876,NM	**POLR1C**	RNA polymerase I and III	protein_coding	82	92	83	93	1.109
9534	NM_001278661,NM	**ZNF254**	zinc finger protein 254	protein_coding	0	0	1	1	1.000
9535	NM_001301008,NM	**GMFG**	glia maturation factor gar	protein_coding	109	114	110	115	1.044
9536	NM_004878	**PTGES**	prostaglandin E synthase	protein_coding	0	0	1	1	1.000
9537	NM_001258320,NM	**TP53I11**	tumor protein p53 inducil	protein_coding	0	0	1	1	1.000
9538	NM_001290135,NM	**EI24**	EI24 autophagy associat	protein_coding	101	102	102	103	1.010
9540	NM_001206802,NM	**TP53I3**	tumor protein p53 inducil	protein_coding	0	0	1	1	1.000
9541	NM_004882	**CIR1**	corepressor interacting w	protein_coding	42	40	43	41	0.962
9542	NM_001184935,NM	**NRG2**	neuregulin 2	protein_coding	0	0	1	1	1.000
9543	NM_004884,XM_01	**IGDCC3**	immunoglobulin superfar	protein_coding	0	0	1	1	1.000
9545	NM_004283	**RAB3D**	RAB3D, member RAS or	protein_coding	0	0	1	1	0.968
9546	NM_004886,XM_00(	**APBA3**	amyloid beta precursor p	protein_coding	25	26	26	27	1.051
9547	NM_004887	**CXCL14**	C-X-C motif chemokine li	protein_coding	0	0	1	1	1.000
9550	NM_004888	**ATP6V1G1**	ATPase H+ transporting	protein_coding	113	114	114	115	1.011
9551	NM_001003713,NM	**ATP5MF**	ATP synthase membrane	protein_coding	869	887	870	888	1.020
9552	NM_004890,XM_00(	**SPAG7**	sperm associated antige	protein_coding	48	52	49	53	1.076
9553	NM_004891,NM_14	**MRPL33**	mitochondrial ribosomal	protein_coding	188	162	189	163	0.863
9554	NM_004892,XM_01	**SEC22B**	SEC22 homolog B, vesic	protein_coding	32	31	33	32	0.984
9555	NM_001040158,NM	**MACROH2A1**	macroH2A.1 histone	protein_coding	451	476	452	477	1.056
9556	NM_001127393,NM	**ATP5MPL**	ATP synthase membrane	protein_coding	204	216	205	217	1.057
9557	NM_001256336,NM	**CHD1L**	chromodomain helicase	protein_coding	43	40	44	41	0.927
9559	NM_001035260,NM	**VPS26A**	VPS26, retromer comple	protein_coding	50	47	51	48	0.936
9560	NM_001291468,NM	**CCL4L2**	C-C motif chemokine liga	protein_coding	264	230	265	231	0.870
9562	NM_001178117,NM	**MINPP1**	multiple inositol-polypho:	protein_coding	15	14	16	15	0.924
9563	NM_001282587,NM	**H6PD**	hexose-6-phosphate deh	protein_coding	1	1	2	2	0.958
9564	NM_001170714,NM	**BCAR1**	BCAR1 scaffold protein,	protein_coding	0	0	1	1	1.000
9567	NM_004286,XM_01	**GTPBP1**	GTP binding protein 1	protein_coding	48	48	49	49	0.994
9568	NM_005458,XM_00!	**GABBR2**	gamma-aminobutyric aci	protein_coding	0	0	1	1	1.000
9569	NM_001199207,NM	**GTF2IRD1**	GTF2I repeat domain co	protein_coding	0	0	1	1	1.000
9570	NM_001012511,NM	**GOSR2**	golgi SNAP receptor con	protein_coding	59	55	60	56	0.933
9572	NM_021724	**NR1D1**	nuclear receptor subfam	protein_coding	7	7	8	8	0.983
9573	NM_020634	**GDF3**	growth differentiation fac	protein_coding	0	0	1	1	1.000
9575	NM_001267843,NM	**CLOCK**	clock circadian regulator	protein_coding	6	6	7	7	0.932
9576	NM_001253854,NM	**SPAG6**	sperm associated antige	protein_coding	1	1	2	2	0.957
9577	NM_001261840,NM	**BABAM2**	BRISC and BRCA1 A cor	protein_coding	30	30	31	31	0.997
9578	NM_006035,XM_00!	**CDC42BPB**	CDC42 binding protein k	protein_coding	0	0	1	1	1.000
9580	NM_005686,XM_00!	**SOX13**	SRY-box transcription fa	protein_coding	0	0	1	1	1.054
9581	NM_001042385,NM	**PREPL**	prolyl endopeptidase like	protein_coding	39	35	40	36	0.902
9582	NM_001270411,NM	**APOBEC3B**	apolipoprotein B mRNA e	protein_coding	74	76	75	77	1.029
9583	NM_001128930,NM	**ENTPD4**	ectonucleoside triphosph	protein_coding	10	9	11	10	0.931
9584	NM_001242599,NM	**RBM39**	RNA binding motif protei	protein_coding	193	186	194	187	0.964
9585	NM_001284259,NM	**KIF20B**	kinesin family member 2(	protein_coding	47	34	48	35	0.744
9586	NM_001011666,NM	**CREB5**	cAMP responsive elemer	protein_coding	0	0	1	1	1.000
9587	NM_001003690,NM	**MAD2L1BP**	MAD2L1 binding protein	protein_coding	83	84	84	85	1.020
9588	NM_004905	**PRDX6**	peroxiredoxin 6	protein_coding	264	276	265	277	1.047
9589	NM_001270531,NM	**WTAP**	WT1 associated protein	protein_coding	163	157	164	158	0.961
9590	NM_001370346,NM	**AKAP12**	A-kinase anchoring prote	protein_coding	0	0	1	1	1.000
9592	NM_004907	**IER2**	immediate early respons	protein_coding	163	172	164	173	1.051
9595	NM_004288,XM_01	**CYTIP**	cytohesin 1 interacting p	protein_coding	191	179	192	180	0.940
9597	NR_026763	**SMAD5-AS1**	SMAD5 antisense RNA 1	lncRNA	0	0	1	1	1.000
9600	NM_001130848,NM	**PITPNM1**	phosphatidylinositol tran	protein_coding	37	38	38	39	1.033
9601	NM_001371244,NM	**PDIA4**	protein disulfide isomera	protein_coding	223	226	224	227	1.016
9603	NM_004289	**NFE2L3**	nuclear factor, erythroid	protein_coding	0	0	1	1	1.022
9604	NM_001201365,NM	**RNF14**	ring finger protein 14	protein_coding	34	30	35	31	0.885
9605	NM_004913,XM_00!	**VPS9D1**	VPS9 domain containing	protein_coding	4	3	5	4	0.868
9607	NM_004291	**CARTPT**	CART prepropeptide	protein_coding	0	0	1	1	1.000
9609	NM_001349877,NM	**RAB36**	RAB36, member RAS on	protein_coding	0	0	1	1	1.000
9610	NM_001363559,NM	**RIN1**	Ras and Rab interactor 1	protein_coding	0	0	1	1	0.983
9611	NM_001190438,NM	**NCOR1**	nuclear receptor corepre	protein_coding	34	27	35	28	0.802
9612	NM_001077261,NM	**NCOR2**	nuclear receptor corepre	protein_coding	51	44	52	45	0.879
9615	NM_001242505,NM	**GDA**	guanine deaminase	protein_coding	0	0	1	1	0.961
9616	NM_001201370,NM	**RNF7**	ring finger protein 7	protein_coding	72	74	73	75	1.026
9617	NM_001354073,NM	**MTRF1**	mitochondrial translation	protein_coding	17	16	18	17	0.933
9618	NM_004295,XM_01	**TRAF4**	TNF receptor associated	protein_coding	21	23	22	24	1.091
9619	NM_004915,NM_01	**ABCG1**	ATP binding cassette sul	protein_coding	6	5	7	6	0.910
9620	NM_001378328,NM	**CELSR1**	cadherin EGF LAG sever	protein_coding	0	0	1	1	0.969
9622	NM_001302961,NM	**KLK4**	kallikrein related peptida	protein_coding	0	0	1	1	1.000
9623	NM_004918	**TCL1B**	TCL1 family AKT coactiva	protein_coding	0	0	1	1	1.000
9625	NM_001080395,NM	**AATK**	apoptosis associated tyro	protein_coding	0	0	1	1	1.000
9626	NM_001363884,NM	**GUCA1C**	guanylate cyclase activa	protein_coding	0	0	1	1	1.000
9627	NM_001242935,NM	**SNCAIP**	synuclein alpha interacti	protein_coding	0	0	1	1	1.000
9628	NM_001204416,NM	**RGS6**	regulator of G protein sig	protein_coding	7	6	8	7	0.908
9629	NR_024604	**CLCA3P**	chloride channel access	transcribed_pseu	0	0	1	1	1.000
9630	NM_004297,XM_01	**GNA14**	G protein subunit alpha	protein_coding	0	0	1	1	1.040
9631	NM_001278312,NM	**NUP155**	nucleoporin 155	protein_coding	46	38	47	39	0.837
9632	NM_004922,NM_19	**SEC24C**	SEC24 homolog C, COP	protein_coding	93	92	94	93	0.981
9633	NM_001039656,NM	**TESMIN**	testis expressed metallot	protein_coding	0	0	1	1	1.000
9635	NM_006536,XM_01	**CLCA2**	chloride channel access	protein_coding	0	0	1	1	0.976
9636	NM_005101	**ISG15**	ISG15 ubiquitin like mod	protein_coding	337	347	338	348	1.030
9637	NM_001042548,NM	**FEZ2**	fasciculation and elongat	protein_coding	12	11	13	12	0.903
9638	NM_005103,NM_02	**FEZ1**	fasciculation and elongat	protein_coding	0	0	1	1	1.000
9639	NM_001308152,NM	**ARHGEF10**	Rho guanine nucleotide	protein_coding	0	0	1	1	0.999
9640	NM_014630,XM_00!	**ZNF592**	zinc finger protein 592	protein_coding	29	29	30	30	1.001
9641	NM_001193321,NM	**IKBKE**	inhibitor of nuclear factor	protein_coding	15	16	16	17	1.085
9643	NM_001142418,NM	**MORF4L2**	mortality factor 4 like 2	protein_coding	169	160	170	161	0.944
9644	NM_001365079,NM	**SH3PXD2A**	SH3 and PX domains 2A	protein_coding	0	0	1	1	0.986
9645	NM_001282663,NM	**MICAL2**	microtubule associated m	protein_coding	18	14	19	15	0.809
9646	NM_001346279,NM	**CTR9**	CTR9 homolog, Paf1/RN	protein_coding	97	90	98	91	0.924
9647	NM_014634,XM_01	**PPM1F**	protein phosphatase, Mg	protein_coding	13	11	14	12	0.903
9648	NM_181453,NR_02	**GCC2**	GRIP and coiled-coil dor	protein_coding	20	15	21	16	0.756
9649	NM_001190728,NM	**RALGPS1**	Ral GEF with PH domair	protein_coding	2	1	3	2	0.579
9650	NM_001145838,NM	**MTFR1**	mitochondrial fission regi	protein_coding	14	12	15	13	0.856
9651	NM_001303012,NM	**PLCH2**	phospholipase C eta 2	protein_coding	0	0	1	1	1.009
9652	NM_014639,XR_00	**TTC37**	tetratricopeptide repeat c	protein_coding	37	31	38	32	0.846
9653	NM_001134492,NM	**HS2ST1**	heparan sulfate 2-O-sulf	protein_coding	38	33	39	34	0.873
9654	NM_014640,XM_00(	**TTLL4**	tubulin tyrosine ligase lik	protein_coding	31	28	32	29	0.885
9655	NM_014011,NM_14	**SOCS5**	suppressor of cytokine si	protein_coding	7	6	8	7	0.809
9656	NM_014641,XM_00!	**MDC1**	mediator of DNA damage	protein_coding	34	35	35	36	1.014
9657	NM_001023570,NM	**IQCB1**	IQ motif containing B1	protein_coding	64	59	65	60	0.917
9658	NM_014643,XM_01	**ZNF516**	zinc finger protein 516	protein_coding	2	2	3	3	1.034
9659	NM_001002810,NM	**PDE4DIP**	phosphodiesterase 4D in	protein_coding	12	8	13	9	0.687
9662	NM_025009,XM_00!	**CEP135**	centrosomal protein 135	protein_coding	9	8	10	9	0.908
9663	NM_001375808,NM	**LPIN2**	lipin 2	protein_coding	37	32	38	33	0.876
9665	NM_001184998,NM	**MARF1**	meiosis regulator and ml	protein_coding	11	9	12	10	0.890
9666	NM_014648,XM_00!	**DZIP3**	DAZ interacting zinc fing	protein_coding	18	17	19	18	0.918
9667	NM_014649,XM_01	**SAFB2**	scaffold attachment facto	protein_coding	91	91	92	92	0.991
9668	NM_001322284,NM	**ZNF432**	zinc finger protein 432	protein_coding	0	0	1	1	1.000
9669	NM_015904	**EIF5B**	eukaryotic translation init	protein_coding	157	145	158	146	0.928
9670	NM_014652,XM_02	**IPO13**	importin 13	protein_coding	31	34	32	35	1.079
9671	NM_001304447,NM	**WSCD2**	WSC domain containing	protein_coding	0	0	1	1	1.000
9672	NM_014654,XM_01	**SDC3**	syndecan 3	protein_coding	0	0	1	1	1.000
9673	NM_001286184,NM	**SLC25A44**	solute carrier family 25 m	protein_coding	31	40	32	41	1.288
9674	NM_001162893,NM	**KIAA0040**	KIAA0040	protein_coding	26	24	27	25	0.913
9675	NM_001303457,NM	**TTI1**	TELO2 interacting protei	protein_coding	52	50	53	51	0.964
9677	NM_001130858,NM	**PPIP5K1**	diphosphoinositol pentak	protein_coding	15	14	16	15	0.943
9678	NM_001007157,NM	**PHF14**	PHD finger protein 14	protein_coding	34	23	35	24	0.686
9679	NM_014661	**FAM53B**	family with sequence sim	protein_coding	18	18	19	19	1.015
9681	NM_001007188,NM	**DEPDC5**	DEP domain containing !	protein_coding	12	11	13	12	0.920
9682	NM_014663,XM_00!	**KDM4A**	lysine demethylase 4A	protein_coding	26	26	27	27	1.004
9683	NM_153029,XM_01	**N4BP1**	NEDD4 binding protein 1	protein_coding	38	31	39	32	0.833
9684	NM_001272036,NM	**LRRC14**	leucine rich repeat conta	protein_coding	10	11	11	12	1.073
9685	NM_001195555,NM	**CLINT1**	clathrin interactor 1	protein_coding	94	89	95	90	0.945
9686	NM_001128219,NM	**VGLL4**	vestigial like family meml	protein_coding	13	12	14	13	0.920

9687	NM_014668,NM_03	**GREB1**	growth regulating estrog	protein_coding	2	2	3	3	0.789
9688	NM_001242795,NM	**NUP93**	nucleoporin 93	protein_coding	33	33	34	34	0.996
9689	NM_001207067,NM	**BZW1**	basic leucine zipper and	protein_coding	263	230	264	231	0.875
9690	NM_014671,XM_00	**UBE3C**	ubiquitin protein ligase E	protein_coding	73	70	74	71	0.961
9692	NM_001256678,NM	**PRORP**	protein only RNase P ca	protein_coding	28	18	29	19	0.641
9693	NM_001351724,NM	**RAPGEF2**	Rap guanine nucleotide	protein_coding	0	0	1	1	0.984
9694	NM_001329493,NM	**EMC2**	ER membrane protein cc	protein_coding	19	21	20	22	1.057
9695	NM_014674,XM_01	**EDEM1**	ER degradation enhanci	protein_coding	35	29	36	30	0.834
9696	NM_014675,XM_00	**CROCC**	ciliary rootlet coiled-coil,	protein_coding	1	1	2	2	0.960
9697	NM_012288,XM_01	**TRAM2**	translocation associated	protein_coding	0	0	1	1	1.000
9698	NM_001020658,NM	**PUM1**	pumilio RNA binding fam	protein_coding	45	38	46	39	0.840
9699	NM_001100117,NM	**RIMS2**	regulating synaptic mem	protein_coding	0	0	1	1	0.960
9700	NM_012291,XM_00	**ESPL1**	extra spindle pole bodies	protein_coding	43	42	44	43	0.983
9701	NM_001242898,NM	**PPP6R2**	protein phosphatase 6 re	protein_coding	29	31	30	32	1.067
9702	NM_001243776,NM	**CEP57**	centrosomal protein 57	protein_coding	56	50	57	51	0.893
9703	NM_001321560,NM	**KIAA0100**	KIAA0100	protein_coding	94	84	95	85	0.898
9704	NM_014681,XM_00	**DHX34**	DExH-box helicase 34	protein_coding	23	24	24	25	1.043
9705	NM_001352826,NM	**ST18**	ST18 C2H2C-type zinc fi	protein_coding	5	5	6	6	0.965
9706	NM_001142610,NM	**ULK2**	unc-51 like autophagy ac	protein_coding	0	0	1	1	1.000
9708	NM_014004,NM_03	**PCDHGA8**	protocadherin gamma su	protein_coding	0	0	1	1	0.919
9709	NM_001010989,NM	**HERPUD1**	homocysteine inducible	protein_coding	25	27	26	28	1.082
9710	NM_014686	**GARRE1**	granule associated Rac a	protein_coding	6	5	7	6	0.869
9711	NM_001145642,NM	**RUBCN**	rubicon autophagy regul	protein_coding	24	22	25	23	0.938
9712	NM_001080491,NM	**USP6NL**	USP6 N-terminal like	protein_coding	0	0	1	1	0.947
9715	NM_001031690,NM	**FAM131B**	family with sequence sim	protein_coding	0	0	1	1	1.000
9716	NM_014691	**AQR**	aquarius intron-binding s	protein_coding	35	30	36	31	0.872
9717	NM_014692,XM_02	**SEC14L5**	SEC14 like lipid binding	protein_coding	0	0	1	1	1.015
9718	NM_001037324,NM	**ECE2**	endothelin converting en	protein_coding	0	0	1	1	1.000
9719	NM_001145320,NM	**ADAMTSL2**	ADAMTS like 2	protein_coding	0	0	1	1	1.000
9720	NM_001382000,NM	**CCDC144A**	coiled-coil domain contai	protein_coding	5	3	6	4	0.646
9721	NM_014696,XM_00	**GPRIN2**	G protein regulated indu	protein_coding	0	0	1	1	1.000
9722	NM_001126060,NM	**NOS1AP**	nitric oxide synthase 1 ac	protein_coding	0	0	1	1	0.984
9723	NM_001178129,NM	**SEMA3E**	semaphorin 3E	protein_coding	0	0	1	1	1.000
9724	NM_021645	**UTP14C**	UTP14C small subunit p	protein_coding	2	2	3	3	1.078
9725	NM_014698,XM_00	**TMEM63A**	transmembrane protein 6	protein_coding	4	4	5	5	1.037
9726	NM_014699,XM_00	**ZNF646**	zinc finger protein 646	protein_coding	23	23	24	24	0.983
9727	NM_001142272,NM	**RAB11FIP3**	RAB11 family interacting	protein_coding	19	20	20	21	1.041
9728	NM_001193489,NM	**SECISBP2L**	SECIS binding protein 2	protein_coding	20	16	21	17	0.816
9729	NM_014702	**KIAA0408**	KIAA0408	protein_coding	0	0	1	1	1.000
9730	NM_001171904,NM	**DCAF1**	DDB1 and CUL4 associa	protein_coding	38	30	39	31	0.786
9731	NM_014704,XM_00	**CEP104**	centrosomal protein 104	protein_coding	16	16	17	17	1.030
9732	NM_001363540,NM	**DOCK4**	dedicator of cytokinesis 4	protein_coding	0	0	1	1	0.884
9733	NM_014706,XM_00	**SART3**	spliceosome associated	protein_coding	69	67	70	68	0.976
9734	NM_001204144,NM	**HDAC9**	histone deacetylase 9	protein_coding	25	18	26	19	0.738
9735	NM_014708,XM_00	**KNTC1**	kinetochore associated 1	protein_coding	35	28	36	29	0.793
9736	NM_014709	**USP34**	ubiquitin specific peptida	protein_coding	21	17	22	18	0.834
9737	NM_001099410,NM	**GPRASP1**	G protein-coupled recep	protein_coding	0	0	1	1	1.000
9738	NM_001199022,NM	**CCP110**	centriolar coiled-coil prot	protein_coding	42	37	43	38	0.871
9739	NM_014712,XM_00	**SETD1A**	SET domain containing 1	protein_coding	29	28	30	29	0.950
9741	NM_014713	**LAPTM4A**	lysosomal protein transm	protein_coding	48	50	49	51	1.030
9742	NM_014714,XM_00	**IFT140**	intraflagellar transport 14	protein_coding	2	2	3	3	0.890
9743	NM_001142685,NM	**ARHGAP32**	Rho GTPase activating p	protein_coding	0	0	1	1	1.010
9744	NM_014716,XM_02	**ACAP1**	ArfGAP with coiled-coil, a	protein_coding	43	46	44	47	1.064
9745	NM_001352260,NM	**ZNF536**	zinc finger protein 536	protein_coding	0	0	1	1	1.000
9746	NM_014718,XM_00	**CLSTN3**	calsyntenin 3	protein_coding	22	17	23	18	0.783
9747	NM_001206938,NM	**TCAF1**	TRPM8 channel associat	protein_coding	0	0	1	1	0.965
9748	NM_001304743,NM	**SLK**	STE20 like kinase	protein_coding	39	33	40	34	0.859
9749	NM_001100164,NM	**PHACTR2**	phosphatase and actin re	protein_coding	2	1	3	2	0.816
9750	NM_001286445,NM	**RIPOR2**	RHO family interacting c	protein_coding	16	14	17	15	0.861
9751	NM_001318234,NM	**SNPH**	syntaphilin	protein_coding	0	0	1	1	0.984
9752	NM_014005,NM_03	**PCDHA9**	protocadherin alpha 9	protein_coding	0	0	1	1	1.000
9753	NM_001163391,NM	**ZSCAN12**	zinc finger and SCAN do	protein_coding	5	6	6	7	1.112
9754	NM_001142503,NM	**STARD8**	StAR related lipid transfe	protein_coding	0	0	1	1	1.000
9755	NM_014726,XM_00	**TBKBP1**	TBK1 binding protein 1	protein_coding	3	5	4	6	1.340
9757	NM_014727,XM_01	**KMT2B**	lysine methyltransferase	protein_coding	28	27	29	28	0.947
9758	NM_001368395,NM	**FRMPD4**	FERM and PDZ domain	protein_coding	0	0	1	1	1.000
9759	NM_001378414,NM	**HDAC4**	histone deacetylase 4	protein_coding	12	11	13	12	0.907
9760	NM_014729,XM_01	**TOX**	thymocyte selection asso	protein_coding	92	86	93	87	0.940
9761	NM_001303627,NM	**MLEC**	malectin	protein_coding	92	89	93	90	0.970
9762	NM_001282533,NM	**LZTS3**	leucine zipper tumor sup	protein_coding	0	0	1	1	0.984
9764	NM_001286565,NM	**KIAA0513**	KIAA0513	protein_coding	2	1	3	2	0.838
9765	NM_001105251,NM	**ZFYVE16**	zinc finger FYVE-type cc	protein_coding	7	6	8	7	0.917
9766	NM_014734	**SUSD6**	sushi domain containing	protein_coding	32	28	33	29	0.892
9767	NM_001077445,NM	**JADE3**	jade family PHD finger 3	protein_coding	0	0	1	1	1.000
9768	NM_001029989,NM	**PCLAF**	PCNA clamp associated	protein_coding	235	226	236	227	0.962
9770	NM_014737,NM_17	**RASSF2**	Ras association domain	protein_coding	0	0	1	1	0.868
9771	NM_001367600,NM	**RAPGEF5**	Rap guanine nucleotide	protein_coding	0	0	1	1	1.000
9772	NM_001321148,NM	**TMEM94**	transmembrane protein 9	protein_coding	19	19	20	20	1.001
9774	NM_001077440,NM	**BCLAF1**	BCL2 associated transcr	protein_coding	83	88	84	89	1.062
9775	NM_014740,XM_01	**EIF4A3**	eukaryotic translation init	protein_coding	173	187	174	188	1.084
9776	NM_001142673,NM	**ATG13**	autophagy related 13	protein_coding	23	23	24	24	1.007
9777	NM_001363731,NM	**TM9SF4**	transmembrane 9 superf	protein_coding	62	57	63	58	0.928
9778	NM_001100590,NM	**KIAA0232**	KIAA0232	protein_coding	10	8	11	9	0.819
9779	NM_001134381,NM	**TBC1D5**	TBC1 domain family mer	protein_coding	19	16	20	17	0.852
9780	NM_001142864	**PIEZO1**	piezo type mechanosens	protein_coding	26	25	27	26	0.954
9781	NM_001349181,NM	**RNF144A**	ring finger protein 144A	protein_coding	0	0	1	1	0.845
9782	NM_001194954,NM	**MATR3**	matrin 3	protein_coding	234	215	235	216	0.916
9783	NM_014747,XM_01	**RIMS3**	regulating synaptic mem	protein_coding	0	0	1	1	1.004
9784	NM_001267059,NM	**SNX17**	sorting nexin 17	protein_coding	43	48	44	49	1.092
9785	NM_014003,XM_00	**DHX38**	DEAH-box helicase 38	protein_coding	63	64	64	65	1.020
9786	NM_001244189,NM	**KIAA0586**	KIAA0586	protein_coding	9	6	10	7	0.695
9787	NM_001146015,NM	**DLGAP5**	DLG associated protein 5	protein_coding	91	73	92	74	0.803
9788	NM_001282971,NM	**MTSS1**	MTSS I-BAR domain con	protein_coding	0	0	1	1	1.000
9789	NM_014752	**SPCS2**	signal peptidase comple	protein_coding	136	129	137	130	0.950
9790	NM_014753,XM_00	**BMS1**	BMS1 ribosome biogene	protein_coding	95	85	96	86	0.894
9791	NM_001290225,NM	**PTDSS1**	phosphatidylserine synth	protein_coding	161	159	162	160	0.987
9792	NM_014755,XM_00	**SERTAD2**	SERTA domain containin	protein_coding	10	9	11	10	0.951
9793	NM_001008938,NM	**CKAP5**	cytoskeleton associated	protein_coding	142	123	143	124	0.866
9794	NM_014757	**MAML1**	mastermind like transcrip	protein_coding	29	26	30	27	0.900
9796	NM_001099335,NM	**PHYHIP**	phytanoyl-CoA 2-hydrox	protein_coding	0	0	1	1	1.125
9797	NM_014760	**TATDN2**	TatD DNase domain con	protein_coding	52	46	53	47	0.889
9798	NM_001270975,NM	**IST1**	IST1 factor associated w	protein_coding	54	51	55	52	0.946
9801	NM_014763	**MRPL19**	mitochondrial ribosomal	protein_coding	20	18	21	19	0.887
9802	NM_001136264,NM	**DAZAP2**	DAZ associated protein 2	protein_coding	137	132	138	133	0.966
9804	NM_014765	**TOMM20**	translocase of outer mito	protein_coding	95	87	96	88	0.910
9805	NM_001145513,NM	**SCRN1**	secernin 1	protein_coding	0	0	1	1	1.000
9806	NM_001134434,NM	**SPOCK2**	SPARC (osteonectin), cv	protein_coding	2	2	3	3	0.906
9807	NM_001006115,NM	**IP6K1**	inositol hexakisphosphat	protein_coding	32	31	33	32	0.975
9808	NR_022006	**KIAA0087**	KIAA0087 lncRNA	lncRNA	0	0	1	1	1.000
9810	NM_001207033,NM	**RNF40**	ring finger protein 40	protein_coding	89	89	90	90	1.004
9811	NM_001142397,NM	**CTIF**	cap binding complex dep	protein_coding	18	18	19	19	1.039
9812	NM_001142603,NM	**DELE1**	DAP3 binding cell death	protein_coding	29	30	30	31	1.029
9813	NM_014774	**EFCAB14**	EF-hand calcium binding	protein_coding	50	46	51	47	0.927
9814	NM_001007467,NM	**SFI1**	SFI1 centrin binding prot	protein_coding	15	14	16	15	0.935
9815	NM_001135213,NM	**GIT2**	GIT ArfGAP 2	protein_coding	32	32	33	33	1.000
9816	NM_001314021,NM	**URB2**	URB2 ribosome biogene	protein_coding	39	37	40	38	0.956
9817	NM_012289,NM_20	**KEAP1**	kelch like ECH associate	protein_coding	148	160	149	161	1.085
9818	NM_001008564,NM	**NUP58**	nucleoporin 58	protein_coding	133	114	134	115	0.856
9819	NM_001303264,NM	**TSC22D2**	TSC22 domain family me	protein_coding	6	4	7	5	0.756
9820	NM_001168370,NM	**CUL7**	cullin 7	protein_coding	4	4	5	5	0.973
9821	NM_001083617,NM	**RB1CC1**	RB1 inducible coiled-coil	protein_coding	18	15	19	16	0.847
9823	NM_001282231,NM	**ARMCX2**	armadillo repeat containi	protein_coding	0	0	1	1	1.000
9824	NM_001286479,NM	**ARHGAP11A**	Rho GTPase activating p	protein_coding	145	119	146	120	0.824
9825	NM_001135773,NM	**SPATA2**	spermatogenesis associa	protein_coding	20	21	21	22	1.068
9826	NM_001377418,NM	**ARHGEF11**	Rho guanine nucleotide	protein_coding	1	1	2	2	1.047
9827	NM_001080496	**RGP1**	RGP1 homolog, RAB6A	protein_coding	17	16	18	17	0.904

9828	NM_014786,XM_01	**ARHGEF17**	Rho guanine nucleotide	protein_coding	0	0	1	1	0.916
9829	NM_001256864,NM	**DNAJC6**	DnaJ heat shock protein	protein_coding	13	10	14	11	0.777
9830	NM_014788,NM_03	**TRIM14**	tripartite motif containing	protein_coding	63	58	64	59	0.913
9831	NM_001082480,NM	**ZNF623**	zinc finger protein 623	protein_coding	5	5	6	6	0.942
9832	NM_001270934,NM	**JAKMIP2**	janus kinase and microtu	protein_coding	5	5	6	6	0.914
9833	NM_001256685,NM	**MELK**	maternal embryonic leuc	protein_coding	76	61	77	62	0.802
9834	NR_026800	**FAM30A**	family with sequence sim	lncRNA	0	0	1	1	0.939
9836	NM_014793	**LCMT2**	leucine carboxyl methyltr	protein_coding	13	13	14	14	0.975
9837	NM_021067,NR_13	**GINS1**	GINS complex subunit 1	protein_coding	66	59	67	60	0.892
9839	NM_001171653,NM	**ZEB2**	zinc finger E-box binding	protein_coding	6	5	7	6	0.837
9840	NM_001098815,NM	**TESPA1**	thymocyte expressed, po	protein_coding	0	0	1	1	0.960
9841	NM_001164313,NM	**ZBTB24**	zinc finger and BTB dom	protein_coding	19	15	20	16	0.818
9842	NM_001352825,NM	**PLEKHM1**	pleckstrin homology and	protein_coding	15	15	16	16	1.016
9843	NM_001130860,NM	**HEPH**	hephaestin	protein_coding	0	0	1	1	1.014
9844	NM_001039459,NM	**ELMO1**	engulfment and cell moti	protein_coding	26	27	27	28	1.036
9846	NM_012296,NM_08	**GAB2**	GRB2 associated binding	protein_coding	0	0	1	1	1.047
9847	NM_001286173,NM	**C2CD5**	C2 calcium dependent d	protein_coding	18	16	19	17	0.890
9848	NM_001009554,NM	**MFAP3L**	microfibril associated pro	protein_coding	0	0	1	1	1.000
9849	NM_001278524,NM	**ZNF518A**	zinc finger protein 518A	protein_coding	6	5	7	6	0.866
9851	NM_001351225,NM	**KIAA0753**	KIAA0753	protein_coding	6	7	7	8	1.047
9852	NM_014805	**EPM2AIP1**	EPM2A interacting protei	protein_coding	9	8	10	9	0.904
9853	NM_001135999,NM	**RUSC2**	RUN and SH3 domain co	protein_coding	0	0	1	1	1.000
9854	NM_001290474,NM	**C2CD2L**	C2CD2 like	protein_coding	16	16	17	17	1.000
9855	NM_001282983,NM	**FARP2**	FERM, ARH/RhoGEF an	protein_coding	11	5	12	6	0.456
9856	NM_001168374,NM	**KIAA0319**	KIAA0319	protein_coding	0	0	1	1	0.898
9857	NM_014810,XM_01	**CEP350**	centrosomal protein 350	protein_coding	25	19	26	20	0.772
9858	NM_014811,XM_00	**PPP1R26**	protein phosphatase 1 re	protein_coding	0	0	1	1	1.000
9859	NM_001042404,NM	**CEP170**	centrosomal protein 170	protein_coding	4	3	5	4	0.832
9860	NM_001312686,NM	**LRIG2**	leucine rich repeats and	protein_coding	8	7	9	8	0.854
9861	NM_001271779,NM	**PSMD6**	proteasome 26S subunit	protein_coding	182	172	183	173	0.947
9862	NM_001079518,NM	**MED24**	mediator complex subun	protein_coding	74	76	75	77	1.036
9863	NM_001301128,NM	**MAGI2**	membrane associated gu	protein_coding	0	0	1	1	1.000
9865	NM_014817	**TRIL**	TLR4 interactor with leuc	protein_coding	0	0	1	1	1.000
9866	NM_014818,XM_00	**TRIM66**	tripartite motif containing	protein_coding	4	4	5	5	0.985
9867	NM_014819,XM_01	**PJA2**	praja ring finger ubiquitin	protein_coding	74	67	75	68	0.902
9868	NM_014820	**TOMM70**	translocase of outer mito	protein_coding	113	100	114	101	0.881
9869	NM_001145415,NM	**SETDB1**	SET domain bifurcated h	protein_coding	22	21	23	22	0.966
9870	NM_001039479,XM	**AREL1**	apoptosis resistant E3 ub	protein_coding	27	24	28	25	0.907
9871	NM_001318066,NM	**SEC24D**	SEC24 homolog D, COP	protein_coding	19	19	20	20	0.980
9873	NM_014824,XM_01	**FCHSD2**	FCH and double SH3 do	protein_coding	32	29	33	30	0.902
9874	NM_001136554,NM	**TLK1**	tousled like kinase 1	protein_coding	53	47	54	48	0.895
9875	NM_014825	**URB1**	URB1 ribosome biogene	protein_coding	33	30	34	31	0.918
9877	NM_001319238,NM	**ZC3H11A**	zinc finger CCCH-type co	protein_coding	69	62	70	63	0.901
9878	NM_001303523,NM	**TOX4**	TOX high mobility group	protein_coding	42	40	43	41	0.955
9879	NM_001300860,NM	**DDX46**	DEAD-box helicase 46	protein_coding	95	79	96	80	0.824
9880	NM_014830	**ZBTB39**	zinc finger and BTB dom	protein_coding	11	10	12	11	0.872
9881	NM_001329998,NM	**TRANK1**	tetratricopeptide repeat a	protein_coding	5	4	6	5	0.935
9882	NM_001286658,NM	**TBC1D4**	TBC1 domain family mer	protein_coding	33	25	34	26	0.775
9883	NM_001257190,NM	**POM121**	POM121 transmembrane	protein_coding	77	76	78	77	0.988
9884	NM_014834	**LRRC37A**	leucine rich repeat conta	protein_coding	2	2	3	3	0.965
9885	NM_001278649,NM	**OSBPL2**	oxysterol binding protein	protein_coding	19	18	20	19	0.926
9886	NM_001242359,NM	**RHOBTB1**	Rho related BTB domain	protein_coding	0	0	1	1	1.000
9887	NM_001174061,NM	**SMG7**	SMG7 nonsense mediate	protein_coding	113	97	114	98	0.860
9889	NM_014838,XM_02	**ZBED4**	zinc finger BED-type con	protein_coding	21	18	22	19	0.839
9890	NM_001166252,NM	**PLPPR4**	phospholipid phosphatas	protein_coding	0	0	1	1	1.000
9891	NM_014840	**NUAK1**	NUAK family kinase 1	protein_coding	0	0	1	1	1.000
9892	NM_001242792,NM	**SNAP91**	synaptosome associated	protein_coding	0	0	1	1	1.000
9894	NM_001351846,NM	**TELO2**	telomere maintenance 2	protein_coding	35	40	36	41	1.129
9895	NM_001172631,NM	**TECPR2**	tectonin beta-propeller re	protein_coding	1	1	2	2	1.073
9896	NM_014845,XM_01	**FIG4**	FIG4 phosphoinositide 5	protein_coding	8	6	9	7	0.813
9897	NM_001330609,NM	**WASHC5**	WASH complex subunit	protein_coding	17	16	18	17	0.976
9898	NM_001127320,NM	**UBAP2L**	ubiquitin associated prot	protein_coding	159	159	160	160	0.997
9899	NM_001167580,NM	**SV2B**	synaptic vesicle glycopro	protein_coding	0	0	1	1	1.016
9900	NM_001278719,NM	**SV2A**	synaptic vesicle glycopro	protein_coding	42	41	43	42	0.982
9901	NM_001033117,NM	**SRGAP3**	SLIT-ROBO Rho GTPase	protein_coding	0	0	1	1	0.947
9902	NM_006039,XM_01	**MRC2**	mannose receptor C type	protein_coding	0	0	1	1	1.000
9903	NM_001324309,NM	**KLHL21**	kelch like family member	protein_coding	14	14	15	15	1.001
9904	NM_001146698,NM	**RBM19**	RNA binding motif protei	protein_coding	39	35	40	36	0.890
9905	NM_001098509,NM	**SGSM2**	small G protein signaling	protein_coding	8	8	9	9	1.077
9906	NM_001199787,NM	**SLC35E2A**	solute carrier family 35 m	protein_coding	7	7	8	8	1.021
9907	NM_001364858,NM	**AP5Z1**	adaptor related protein c	protein_coding	10	12	11	13	1.121
9908	NM_012297,NM_20	**G3BP2**	G3BP stress granule ass	protein_coding	161	147	162	148	0.909
9909	NM_001367466,NM	**DENND4B**	DENN domain containing	protein_coding	68	69	69	70	1.018
9910	NM_001035230,NM	**RABGAP1L**	RAB GTPase activating p	protein_coding	12	13	13	14	1.125
9911	NM_001242925,NM	**TMCC2**	transmembrane and coile	protein_coding	1	0	2	1	0.936
9912	NM_001321164,NM	**ARHGAP44**	Rho GTPase activating p	protein_coding	0	0	1	1	1.000
9913	NM_001282729,NM	**SUPT7L**	SPT7 like, STAGA compl	protein_coding	10	11	11	12	1.025
9914	NM_001286527,NM	**ATP2C2**	ATPase secretory pathwa	protein_coding	0	0	1	1	1.000
9915	NM_014862	**ARNT2**	aryl hydrocarbon recepto	protein_coding	0	0	1	1	1.000
9917	NM_001324310,NM	**FAM20B**	FAM20B glycosaminogly	protein_coding	22	20	23	21	0.897
9918	NM_014865	**NCAPD2**	non-SMC condensin I co	protein_coding	89	83	90	84	0.931
9919	NM_001276418,NM	**SEC16A**	SEC16 homolog A, endo	protein_coding	52	46	53	47	0.888
9920	NM_014867,XM_01	**KBTBD11**	kelch repeat and BTB do	protein_coding	0	0	1	1	1.000
9921	NM_001330474,NM	**RNF10**	ring finger protein 10	protein_coding	55	52	56	53	0.957
9922	NM_001134382,NM	**IQSEC1**	IQ motif and Sec7 domai	protein_coding	17	17	18	18	1.017
9923	NM_001083621,NM	**ZBTB40**	zinc finger and BTB dom	protein_coding	0	0	1	1	0.990
9924	NM_001127460,NM	**PAN2**	poly(A) specific ribonucle	protein_coding	5	5	6	6	1.066
9925	NM_014872,XM_00	**ZBTB5**	zinc finger and BTB dom	protein_coding	10	9	11	10	0.876
9926	NM_001320808,NM	**LPGAT1**	lysophosphatidylglycerol	protein_coding	13	10	14	11	0.836
9927	NM_001127660,NM	**MFN2**	mitofusin 2	protein_coding	134	131	135	132	0.978
9928	NM_001305792,NM	**KIF14**	kinesin family member 14	protein_coding	59	45	60	46	0.766
9929	NM_001360235,NM	**JOSD1**	Josephin domain contair	protein_coding	70	71	71	72	1.016
9931	NM_001330447,NM	**HELZ**	helicase with zinc finger	protein_coding	29	24	30	25	0.816
9933	NM_014878	**PUM3**	pumilio RNA binding fam	protein_coding	102	98	103	99	0.960
9934	NM_001081455,NM	**P2RY14**	purinergic receptor P2Y1	protein_coding	0	0	1	1	1.000
9935	NM_005461	**MAFB**	MAF bZIP transcription fa	protein_coding	0	0	1	1	1.000
9936	NM_001198763,NM	**CD302**	CD302 molecule	protein_coding	0	0	1	1	0.920
9937	NM_001271816,NM	**DCLRE1A**	DNA cross-link repair 1A	protein_coding	27	20	28	21	0.736
9938	NM_001007231,NM	**ARHGAP25**	Rho GTPase activating p	protein_coding	107	104	108	105	0.974
9939	NM_005105	**RBM8A**	RNA binding motif protei	protein_coding	144	150	145	151	1.042
9940	NM_001321153,NM	**DLEC1**	DLEC1 cilia and flagella	protein_coding	0	0	1	1	1.000
9941	NM_001145464,NM	**EXOG**	exo/endonuclease G	protein_coding	14	15	15	16	1.078
9942	NM_001349178,NM	**XYLB**	xylulokinase	protein_coding	2	2	3	3	1.107
9943	NM_005109,XM_00	**OXSR1**	oxidative stress responsi	protein_coding	52	44	53	45	0.859
9945	NM_005110	**GFPT2**	glutamine-fructose-6-pho	protein_coding	0	0	1	1	1.403
9946	NM_145858	**CRYZL1**	crystallin zeta like 1	protein_coding	11	12	12	13	1.063
9947	NM_005462,XM_01	**MAGEC1**	MAGE family member C1	protein_coding	0	0	1	1	1.000
9948	NM_005112,NM_01	**WDR1**	WD repeat domain 1	protein_coding	695	713	696	714	1.026
9949	NM_001025580,NM	**AMMECR1**	AMMECR nuclear protein	protein_coding	21	16	22	17	0.754
9950	NM_005113,XM_01	**GOLGA5**	golgin A5	protein_coding	25	24	26	25	0.991
9951	NM_006040	**HS3ST4**	heparan sulfate-glucosar	protein_coding	0	0	1	1	1.000
9953	NM_006041,NR_13	**HS3ST3B1**	heparan sulfate-glucosar	protein_coding	5	4	6	5	0.955
9955	NM_006042,XM_01	**HS3ST3A1**	heparan sulfate-glucosar	protein_coding	0	0	1	1	1.001
9956	NM_006043,XM_01	**HS3ST2**	heparan sulfate-glucosar	protein_coding	0	0	1	1	1.000
9957	NM_005114,XM_01	**HS3ST1**	heparan sulfate-glucosar	protein_coding	0	0	1	1	1.000
9958	NM_001252078,NM	**USP15**	ubiquitin specific peptida	protein_coding	20	19	21	20	0.968
9959	gene-USP12P1	**USP12P1**	USP12 pseudogene 1	pseudogene	0	0	1	1	0.878
9960	NM_001256702,NM	**USP3**	ubiquitin specific peptida	protein_coding	17	17	18	18	0.991
9961	NM_001293204,NM	**MVP**	major vault protein	protein_coding	0	0	1	1	0.977
9962	NM_005116,NM_20	**SLC23A2**	solute carrier family 23 m	protein_coding	18	15	19	16	0.825
9963	NM_005847,NM_15	**SLC23A1**	solute carrier family 23 m	protein_coding	1	1	2	2	0.894
9964	gene-MYCLP3	**MYCLP3**	MYCL pseudogene 3	pseudogene	0	0	1	1	1.000
9965	NM_005117	**FGF19**	fibroblast growth factor 1	protein_coding	0	0	1	1	1.000
9966	NM_001204344,NM	**TNFSF15**	TNF superfamily membe	protein_coding	0	0	1	1	0.986
9967	NM_001321471,NM	**THRAP3**	thyroid hormone recepto	protein_coding	163	150	164	151	0.926
9968	NM_005120	**MED12**	mediator complex subun	protein_coding	23	22	24	23	0.942

9969	NM_005121,XM_01	**MED13**	mediator complex subun	protein_coding	34	29	35	30	0.858
9970	NM_001077469,NM	**NR1I3**	nuclear receptor subfam	protein_coding	0	0	1	1	1.000
9971	NM_001206977,NM	**NR1H4**	nuclear receptor subfam	protein_coding	0	0	1	1	1.000
9972	NM_001278209,NM	**NUP153**	nucleoporin 153	protein_coding	122	105	123	106	0.857
9973	NM_005125,XR_24	**CCS**	copper chaperone for su	protein_coding	15	16	16	17	1.117
9975	NM_001145425,NM	**NR1D2**	nuclear receptor subfam	protein_coding	6	5	7	6	0.923
9976	NM_005127	**CLEC2B**	C-type lectin domain fam	protein_coding	20	18	21	19	0.872
9978	NM_014248	**RBX1**	ring-box 1	protein_coding	96	92	97	93	0.957
9980	NM_001320714,NM	**DOP1B**	DOP1 leucine zipper like	protein_coding	1	1	2	2	0.818
9982	NM_005130	**FGFBP1**	fibroblast growth factor b	protein_coding	0	0	1	1	1.000
9984	NM_005131,XM_01	**THOC1**	THO complex 1	protein_coding	33	27	34	28	0.824
9985	NM_001048205,NM	**REC8**	REC8 meiotic recombina	protein_coding	1	2	2	3	1.248
9986	NM_001032279,NM	**RCE1**	Ras converting CAAX en	protein_coding	31	33	32	34	1.081
9987	NM_001207000,NM	**HNRNPDL**	heterogeneous nuclear r	protein_coding	222	228	223	229	1.030
9988	NM_001142326,NM	**DMTF1**	cyclin D binding myb like	protein_coding	27	24	28	25	0.925
9989	NM_001042388,NM	**PPP4R1**	protein phosphatase 4 re	protein_coding	35	36	36	37	1.015
9990	NM_001042494,NM	**SLC12A6**	solute carrier family 12 m	protein_coding	2	2	3	3	1.026
9991	NM_001163788,NM	**PTBP3**	polypyrimidine tract bind	protein_coding	95	78	96	79	0.826
9992	NM_172201	**KCNE2**	potassium voltage-gated	protein_coding	0	1	1	2	1.158
9993	NM_001173533,NM	**DGCR2**	DiGeorge syndrome critic	protein_coding	28	29	29	30	1.023
9994	NM_001137667,NM	**CASP8AP2**	caspase 8 associated pr	protein_coding	23	19	24	20	0.812
9995	gene-ELK2BP	**ELK2BP**	ETS transcription factor I	pseudogene	0	0	1	1	1.000
9997	NM_001169109,NM	**SCO2**	synthesis of cytochrome	protein_coding	12	11	13	12	0.924
10000	NM_001206729,NM	**AKT3**	AKT serine/threonine kin	protein_coding	0	0	1	1	0.971
10001	NM_001284209,NM	**MED6**	mediator complex subun	protein_coding	38	41	39	42	1.089
10002	NM_014249,NM_01	**NR2E3**	nuclear receptor subfam	protein_coding	0	0	1	1	1.000
10003	NM_001300930,NM	**NAALAD2**	N-acetylated alpha-linke	protein_coding	0	0	1	1	1.000
10004	NM_005468,XM_01	**NAALADL1**	N-acetylated alpha-linke	protein_coding	0	0	1	1	0.968
10005	NM_005469,XM_00	**ACOT8**	acyl-CoA thioesterase 8	protein_coding	54	56	55	57	1.040
10006	NM_001012750,NM	**ABI1**	abl interactor 1	protein_coding	9	10	10	11	1.087
10007	NM_005471,XM_00	**GNPDA1**	glucosamine-6-phosphat	protein_coding	2	2	3	3	1.012
10008	NM_005472,XM_01	**KCNE3**	potassium voltage-gated	protein_coding	0	0	1	1	0.988
10009	NM_001184742,NM	**ZBTB33**	zinc finger and BTB dom	protein_coding	35	31	36	32	0.886
10010	NM_001199135,NM	**TANK**	TRAF family member ass	protein_coding	77	65	78	66	0.838
10011	NM_001035235,NM	**SRA1**	steroid receptor RNA act	protein_coding	57	56	58	57	0.986
10013	NM_001321225,NM	**HDAC6**	histone deacetylase 6	protein_coding	16	16	17	17	1.032
10014	NM_001015053,NM	**HDAC5**	histone deacetylase 5	protein_coding	2	3	3	4	1.250
10015	NM_001162429,NM	**PDCD6IP**	programmed cell death 6	protein_coding	53	47	54	48	0.899
10016	NM_001267556,NM	**PDCD6**	programmed cell death 6	protein_coding	109	112	110	113	1.031
10017	NM_001306168,NM	**BCL2L10**	BCL2 like 10	protein_coding	0	0	1	1	0.932
10018	NM_001204106,NM	**BCL2L11**	BCL2 like 11	protein_coding	7	7	8	8	1.007
10019	NM_001291424,NM	**SH2B3**	SH2B adaptor protein 3	protein_coding	0	0	1	1	1.013
10020	NM_001128227,NM	**GNE**	glucosamine (UDP-N-ac	protein_coding	9	9	10	10	0.987
10021	NM_005477,XM_01	**HCN4**	hyperpolarization activat	protein_coding	0	0	1	1	1.000
10022	NM_005478	**INSL5**	insulin like 5	protein_coding	0	0	1	1	1.000
10023	NM_005479	**FRAT1**	FRAT regulator of WNT s	protein_coding	1	1	2	2	1.012
10024	NM_001100620,NM	**TROAP**	trophinin associated prot	protein_coding	32	34	33	35	1.069
10025	NM_005481,XM_01	**MED16**	mediator complex subun	protein_coding	45	49	46	50	1.078
10026	NM_005482	**PIGK**	phosphatidylinositol glyc	protein_coding	39	35	40	36	0.910
10031	gene-SHROOM2P1	**SHROOM2P1**	shroom family member 2	pseudogene	0	0	1	1	1.000
10033	gene-ARSLP1	**ARSLP1**	arylsulfatase L pseudoge	pseudogene	0	0	1	1	1.000
10034	gene-ARSDP1	**ARSDP1**	arylsulfatase D pseudog	pseudogene	0	0	1	1	1.000
10036	NM_005483,XM_01	**CHAF1A**	chromatin assembly fact	protein_coding	113	107	114	108	0.946
10038	NM_001042618,NM	**PARP2**	poly(ADP-ribose) polyme	protein_coding	40	43	41	44	1.066
10039	NM_001003931,NM	**PARP3**	poly(ADP-ribose) polyme	protein_coding	11	12	12	13	1.035
10040	NM_001321173,NM	**TOM1L1**	target of myb1 like 1 mer	protein_coding	0	0	1	1	1.000
10042	NM_001003681,NM	**HMGXB4**	HMG-box containing 4	protein_coding	21	19	22	20	0.906
10043	NM_001135729,NM	**TOM1**	target of myb1 membran	protein_coding	6	6	7	7	0.975
10044	NM_001142531,NM	**SH2D3C**	SH2 domain containing 3	protein_coding	30	34	31	35	1.152
10045	NM_005490,XM_00	**SH2D3A**	SH2 domain containing 3	protein_coding	17	17	18	18	0.975
10046	NM_001177465,NM	**MAMLD1**	mastermind like domain	protein_coding	0	0	1	1	1.000
10047	NM_001281730,NM	**CST8**	cystatin 8	protein_coding	0	0	1	1	1.000
10048	NM_005493,XM_00	**RANBP9**	RAN binding protein 9	protein_coding	52	48	53	49	0.924
10049	NM_001363676,NM	**DNAJB6**	DnaJ heat shock protein	protein_coding	138	129	139	130	0.941
10050	NM_001286121,NM	**SLC17A4**	solute carrier family 17 m	protein_coding	0	0	1	1	1.000
10051	NM_001002800,NM	**SMC4**	structural maintenance c	protein_coding	252	209	253	210	0.831
10052	NM_001080383,NM	**GJC1**	gap junction protein gam	protein_coding	0	0	1	1	1.013
10053	NM_001300887,NM	**AP1M2**	adaptor related protein c	protein_coding	0	0	1	1	1.000
10054	NM_005499,XM_00	**UBA2**	ubiquitin like modifier act	protein_coding	210	192	211	193	0.915
10055	NM_001145713,NM	**SAE1**	SUMO1 activating enzym	protein_coding	320	311	321	312	0.971
10056	NM_005687,NR_13	**FARSB**	phenylalanyl-tRNA synth	protein_coding	157	151	158	152	0.967
10057	NM_001023587,NM	**ABCC5**	ATP binding cassette su	protein_coding	24	21	25	22	0.903
10058	NM_001349828,NM	**ABCB6**	ATP binding cassette su	protein_coding	0	0	1	1	0.952
10059	NM_001278463,NM	**DNM1L**	dynamin 1 like	protein_coding	69	60	70	61	0.868
10060	NM_001377273,NM	**ABCC9**	ATP binding cassette su	protein_coding	0	0	1	1	1.000
10061	NM_007189	**ABCF2**	ATP binding cassette su	protein_coding	74	74	75	75	1.003
10062	NM_001130101,NM	**NR1H3**	nuclear receptor subfam	protein_coding	13	15	14	16	1.155
10063	NM_001382002,NM	**COX17**	cytochrome c oxidase co	protein_coding	254	261	255	262	1.028
10064	gene-VDAC1P2	**VDAC1P2**	voltage dependent anion	pseudogene	0	0	1	1	1.130
10066	NM_001320778,NM	**SCAMP2**	secretory carrier membra	protein_coding	87	92	88	93	1.053
10067	NM_005698,NM_05	**SCAMP3**	secretory carrier membra	protein_coding	119	128	120	129	1.079
10068	NM_001039659,NM	**IL18BP**	interleukin 18 binding pr	protein_coding	4	4	5	5	1.148
10069	NM_001320724,NM	**RWDD2B**	RWD domain containing	protein_coding	18	19	19	20	1.051
10071	NM_001164462	**MUC12**	mucin 12, cell surface as	protein_coding	0	0	1	1	0.984
10072	NM_001256670,NM	**DPP3**	dipeptidyl peptidase 3	protein_coding	88	88	89	89	1.008
10073	NM_001042581,NM	**SNUPN**	snurportin 1	protein_coding	35	32	36	33	0.914
10075	NM_031407,XM_00	**HUWE1**	HECT, UBA and WWE d	protein_coding	99	82	100	83	0.835
10076	NM_001195001,NM	**PTPRU**	protein tyrosine phospha	protein_coding	0	0	1	1	0.949
10077	NM_139022,XM_00	**TSPAN32**	tetraspanin 32	protein_coding	2	1	3	2	0.676
10078	NM_001297658,NM	**TSSC4**	tumor suppressing subtr	protein_coding	68	75	69	76	1.098
10079	NM_006045,XM_01	**ATP9A**	ATPase phospholipid tra	protein_coding	0	0	1	1	1.034
10081	NM_005707	**PDCD7**	programmed cell death 7	protein_coding	51	53	52	54	1.036
10082	NM_005708,XM_01	**GPC6**	glypican 6	protein_coding	0	0	1	1	1.000
10083	NM_001297764,NM	**USH1C**	USH1 protein network cc	protein_coding	0	0	1	1	1.000
10084	NM_001032381,NM	**PQBP1**	polyglutamine binding pr	protein_coding	120	135	121	136	1.123
10085	NM_001278642,NM	**EDIL3**	EGF like repeats and dis	protein_coding	0	0	1	1	1.000
10086	NM_001145095	**HHLA1**	HERV-H LTR-associating	protein_coding	0	0	1	1	1.000
10087	NM_001130105,NM	**CERT1**	ceramide transporter 1	protein_coding	21	20	22	21	0.956
10089	NM_005714,NM_03	**KCNK7**	potassium two pore dom	protein_coding	0	1	1	2	1.074
10090	NM_005715,XM_01	**UST**	uronyl 2-sulfotransferase	protein_coding	0	0	1	1	1.000
10092	NM_001270439,NM	**ARPC5**	actin related protein 2/3	protein_coding	91	86	92	87	0.943
10093	NM_001024959,NM	**ARPC4**	actin related protein 2/3	protein_coding	161	170	162	171	1.060
10094	NM_001278556,NM	**ARPC3**	actin related protein 2/3	protein_coding	455	442	456	443	0.973
10095	NM_005720,XM_00	**ARPC1B**	actin related protein 2/3	protein_coding	471	479	472	480	1.018
10096	NM_001277140,NM	**ACTR3**	actin related protein 3	protein_coding	304	279	305	280	0.919
10097	NM_001005386,NM	**ACTR2**	actin related protein 2	protein_coding	440	379	441	380	0.861
10098	NM_005723,XM_00	**TSPAN5**	tetraspanin 5	protein_coding	12	12	13	13	0.937
10099	NM_001168412,NM	**TSPAN3**	tetraspanin 3	protein_coding	14	13	15	14	0.951
10100	NM_001308315,NM	**TSPAN2**	tetraspanin 2	protein_coding	5	5	6	6	0.987
10101	NM_001284501,NM	**NUBP2**	nucleotide binding protei	protein_coding	84	93	85	94	1.101
10102	NM_001172695,NM	**TSFM**	Ts translation elongation	protein_coding	37	38	38	39	1.015
10103	NM_005727,XM_01	**TSPAN1**	tetraspanin 1	protein_coding	0	0	1	1	1.000
10105	NM_005729,XM_00	**PPIF**	peptidylprolyl isomerase	protein_coding	300	283	301	284	0.942
10106	NM_005730,XM_00	**CTDSP2**	CTD small phosphatase	protein_coding	1	1	2	2	1.114
10107	NM_006778,NM_05	**TRIM10**	tripartite motif containing	protein_coding	0	0	1	1	1.000
10109	NM_005731,NM_15	**ARPC2**	actin related protein 2/3	protein_coding	1,118	1,139	1,119	1,140	1.019
10110	NM_001199264,NM	**SGK2**	serum/glucocorticoid reg	protein_coding	0	0	1	1	1.000
10111	NM_005732	**RAD50**	RAD50 double strand br	protein_coding	46	41	47	42	0.880
10112	NM_005733	**KIF20A**	kinesin family member 2	protein_coding	67	67	68	68	0.994
10113	NM_001330484,NM	**PREB**	prolactin regulatory elem	protein_coding	71	69	72	70	0.968
10114	NM_001048200,NM	**HIPK3**	homeodomain interacting	protein_coding	18	17	19	18	0.898
10116	NM_015322	**FEM1B**	fem-1 homolog B	protein_coding	37	31	38	32	0.842
10117	NM_001368133,NM	**ENAM**	enamelin	protein_coding	0	0	1	1	1.000
10120	NM_005735,XM_00	**ACTR1B**	actin related protein 1B	protein_coding	26	27	27	28	1.029
10121	NM_005736	**ACTR1A**	actin related protein 1A	protein_coding	91	87	92	88	0.955
10123	NM_001282431,NM	**ARL4C**	ADP ribosylation factor li	protein_coding	209	202	210	203	0.969

10124	NM_001037164,NM	**ARL4A**	ADP ribosylation factor li	protein_coding	0	0	1	1	1.000
10125	NM_001128602,NM	**RASGRP1**	RAS guanyl releasing pr	protein_coding	60	56	61	57	0.931
10126	NM_005740	**DNAL4**	dynein axonemal light ch	protein_coding	6	6	7	7	1.002
10127	NM_005741,XM_00	**ZNF263**	zinc finger protein 263	protein_coding	45	43	46	44	0.951
10128	NM_133259,XM_00	**LRPPRC**	leucine rich pentatricope	protein_coding	72	63	73	64	0.870
10129	NM_023037,XM_00	**FRY**	FRY microtubule binding	protein_coding	0	0	1	1	0.983
10130	NM_001282704,NM	**PDIA6**	protein disulfide isomera	protein_coding	278	276	279	277	0.994
10131	NM_001272049,NM	**TRAP1**	TNF receptor associated	protein_coding	281	280	282	281	0.994
10133	NM_001008211,NM	**OPTN**	optineurin	protein_coding	1	1	2	2	1.002
10134	NM_001139441,NM	**BCAP31**	B cell receptor associate	protein_coding	112	119	113	120	1.067
10135	NM_005746	**NAMPT**	nicotinamide phosphorib	protein_coding	143	129	144	130	0.905
10136	NM_005747	**CELA3A**	chymotrypsin like elastas	protein_coding	0	0	1	1	1.000
10137	NM_001198838,NM	**RBM12**	RNA binding motif protei	protein_coding	64	61	65	62	0.956
10138	NM_001190977,NM	**YAF2**	YY1 associated factor 2	protein_coding	9	7	10	8	0.785
10139	NM_001134758,NM	**ARFRP1**	ADP ribosylation factor re	protein_coding	51	59	52	60	1.137
10140	NM_001243877,NM	**TOB1**	transducer of ERBB2, 1	protein_coding	11	11	12	12	0.967
10141	NR_126517,NR_12	**LINC01587**	long intergenic non-prote	lncRNA	0	0	1	1	1.000
10142	NM_001379277,NM	**AKAP9**	A-kinase anchoring prote	protein_coding	14	11	15	12	0.780
10143	NM_005752	**CLEC3A**	C-type lectin domain fam	protein_coding	0	0	1	1	1.000
10144	NM_001015045,NM	**FAM13A**	family with sequence sim	protein_coding	0	0	1	1	1.000
10146	NM_005754,NM_19	**G3BP1**	G3BP stress granule ass	protein_coding	116	105	117	106	0.905
10147	NM_001017392,NM	**SUGP2**	SURP and G-patch dom	protein_coding	47	46	48	47	0.977
10148	NM_005755,XM_01	**EBI3**	Epstein-Barr virus induce	protein_coding	0	0	1	1	1.000
10149	NM_001079858,NM	**ADGRG2**	adhesion G protein-coup	protein_coding	0	0	1	1	1.000
10150	NM_001306070,NM	**MBNL2**	muscleblind like splicing	protein_coding	1	1	2	2	1.022
10151	NR_002726	**HNRNPA3P1**	heterogeneous nuclear r	transcribed_pseu	0	0	1	1	0.958
10152	NM_001282925,NM	**ABI2**	abl interactor 2	protein_coding	25	20	26	21	0.825
10153	NM_005760	**CEBPZ**	CCAAT enhancer binding	protein_coding	102	92	103	93	0.901
10154	NM_005761,NR_03	**PLXNC1**	plexin C1	protein_coding	11	11	12	12	0.964
10155	NM_005762,XM_02	**TRIM28**	tripartite motif containing	protein_coding	381	424	382	425	1.113
10156	NM_001079877,NM	**RASA4**	RAS p21 protein activato	protein_coding	9	10	10	11	1.135
10157	NM_005763,XM_01	**AASS**	aminoadipate-semialdeh	protein_coding	0	0	1	1	1.000
10158	NM_005764	**PDZK1IP1**	PDZK1 interacting protei	protein_coding	0	0	1	1	1.000
10159	NM_005765	**ATP6AP2**	ATPase H+ transporting	protein_coding	112	102	113	103	0.911
10160	NM_001001715,NM	**FARP1**	FERM, ARH/RhoGEF an	protein_coding	0	0	1	1	1.006
10161	NM_001162497,NM	**LPAR6**	lysophosphatidic acid re	protein_coding	0	0	1	1	0.899
10162	NM_005768,XM_02	**LPCAT3**	lysophosphatidylcholine	protein_coding	8	6	9	7	0.798
10163	NM_001201404,NM	**WASF2**	WASP family member 2	protein_coding	69	67	70	68	0.982
10164	NM_001166395,NM	**CHST4**	carbohydrate sulfotransf	protein_coding	0	0	1	1	1.000
10165	NM_001160210,NM	**SLC25A13**	solute carrier family 25 n	protein_coding	36	35	37	36	0.967
10166	NM_014252	**SLC25A15**	solute carrier family 25 n	protein_coding	9	8	10	9	0.933
10168	NM_001024855,NM	**ZNF197**	zinc finger protein 197	protein_coding	12	11	13	12	0.893
10169	NM_001018108,NM	**SERF2**	small EDRK-rich factor 2	protein_coding	181	214	182	215	1.176
10170	NM_001142270,NM	**DHRS9**	dehydrogenase/reductas	protein_coding	0	0	1	1	1.000
10171	NM_001286699,NM	**RCL1**	RNA terminal phosphate	protein_coding	46	38	47	39	0.831
10172	NM_001375403,NM	**ZNF256**	zinc finger protein 256	protein_coding	0	0	1	1	1.000
10174	NM_001018003,NM	**SORBS3**	sorbin and SH3 domain	protein_coding	0	0	1	1	0.857
10175	NM_005776	**CNIH1**	cornichon family AMPA r	protein_coding	47	41	48	42	0.883
10178	NM_001163278,NM	**TENM1**	teneurin transmembrane	protein_coding	0	0	1	1	1.000
10179	NM_001286045,NM	**RBM7**	RNA binding motif protei	protein_coding	30	26	31	27	0.871
10180	NM_001167582,NM	**RBM6**	RNA binding motif protei	protein_coding	36	34	37	35	0.952
10181	NM_005778,NR_03	**RBM5**	RNA binding motif protei	protein_coding	54	51	55	52	0.952
10184	NM_005779,XM_00	**LHFPL2**	LHFPL tetraspan subfam	protein_coding	0	0	1	1	1.013
10186	NM_005780,XM_01	**LHFPL6**	LHFPL tetraspan subfam	protein_coding	0	0	1	1	1.000
10187	gene-VDAC1P5	**VDAC1P5**	voltage dependent anion	pseudogene	0	0	1	1	1.000
10188	NM_001010938,NM	**TNK2**	tyrosine kinase non rece	protein_coding	17	18	18	19	1.066
10189	NM_005782,NR_15	**ALYREF**	Aly/REF export factor	protein_coding	318	340	319	341	1.071
10190	NM_005783,XM_01	**TXNDC9**	thioredoxin domain conta	protein_coding	51	46	52	47	0.896
10193	NM_001242826,NM	**RNF41**	ring finger protein 41	protein_coding	15	14	16	15	0.933
10194	NM_001308210,NM	**TSHZ1**	teashirt zinc finger home	protein_coding	19	19	20	20	1.023
10195	NM_001006941,NM	**ALG3**	ALG3 alpha-1,3- mannos	protein_coding	120	134	121	135	1.114
10196	NM_001145166,NM	**PRMT3**	protein arginine methyltr	protein_coding	60	56	61	57	0.927
10197	NM_001267045,NM	**PSME3**	proteasome activator sub	protein_coding	285	279	286	280	0.979
10198	NM_022782,NR_10	**MPHOSPH9**	M-phase phosphoprotein	protein_coding	24	19	25	20	0.784
10199	NM_005791	**MPHOSPH10**	M-phase phosphoprotein	protein_coding	96	87	97	88	0.904
10200	NM_005792,XM_01	**MPHOSPH6**	M-phase phosphoprotein	protein_coding	99	87	100	88	0.887
10201	NM_001308426,NM	**NME6**	NME/NM23 nucleoside d	protein_coding	18	17	19	18	0.914
10202	NM_001318835,NM	**DHRS2**	dehydrogenase/reductas	protein_coding	0	1	1	2	1.138
10203	NM_001271751,NM	**CALCRL**	calcitonin receptor like re	protein_coding	0	0	1	1	0.881
10204	NM_001322038,NM	**NUTF2**	nuclear transport factor 2	protein_coding	112	123	113	124	1.103
10205	NM_005797,NM_14	**MPZL2**	myelin protein zero like 2	protein_coding	0	0	1	1	1.000
10206	NM_001007278,NM	**TRIM13**	tripartite motif containing	protein_coding	7	6	8	7	0.905
10207	NM_001350145,NM	**PATJ**	PATJ crumbs cell polarit	protein_coding	0	0	1	1	1.005
10208	NM_001321532,NM	**USPL1**	ubiquitin specific peptida	protein_coding	24	20	25	21	0.863
10209	NM_005801	**EIF1**	eukaryotic translation init	protein_coding	408	401	409	402	0.982
10210	NM_001195622,NM	**TOPORS**	TOP1 binding arginine/se	protein_coding	16	15	17	16	0.952
10211	NM_001318875,NM	**FLOT1**	flotillin 1	protein_coding	374	338	375	339	0.905
10212	NM_005804,NR_04	**DDX39A**	DExD-box helicase 39A	protein_coding	390	425	391	426	1.090
10213	NM_005805	**PSMD14**	proteasome 26S subunit	protein_coding	243	220	244	221	0.906
10214	NM_021014,XM_01	**SSX3**	SSX family member 3	protein_coding	0	0	1	1	1.000
10215	NM_005806,XM_00	**OLIG2**	oligodendrocyte transcrip	protein_coding	0	0	1	1	1.000
10216	NM_001127708,NM	**PRG4**	proteoglycan 4	protein_coding	0	0	1	1	1.000
10217	NM_001008392,NM	**CTDSPL**	CTD small phosphatase	protein_coding	0	0	1	1	1.000
10218	NM_021146,XM_01	**ANGPTL7**	angiopoietin like 7	protein_coding	0	0	1	1	1.000
10219	NM_001329099,NM	**KLRG1**	killer cell lectin like recep	protein_coding	9	5	10	6	0.624
10220	NM_005811,XM_00	**GDF11**	growth differentiation fac	protein_coding	4	3	5	4	0.834
10221	NM_001282985,NM	**TRIB1**	tribbles pseudokinase 1	protein_coding	2	1	3	2	0.680
10223	NM_005814,XM_01	**GPA33**	glycoprotein A33	protein_coding	0	0	1	1	1.038
10224	NM_005815	**ZNF443**	zinc finger protein 443	protein_coding	0	0	1	1	1.004
10225	NM_001318889,NM	**CD96**	CD96 molecule	protein_coding	150	124	151	125	0.826
10226	NM_001164189,NM	**PLIN3**	perilipin 3	protein_coding	16	12	17	13	0.776
10227	NM_001120,NM_00	**MFSD10**	major facilitator superfan	protein_coding	29	30	30	31	1.045
10228	NM_001286210,NM	**STX6**	syntaxin 6	protein_coding	26	23	27	24	0.880
10229	NM_001190983,NM	**COQ7**	coenzyme Q7, hydroxyla	protein_coding	17	18	18	19	1.022
10230	NR_003108,NR_13	**NBR2**	neighbor of BRCA1 lncR	lncRNA	7	7	8	8	0.917
10231	NM_001251973,NM	**RCAN2**	regulator of calcineurin 2	protein_coding	3	4	4	5	1.063
10232	NM_001177355,NM	**MSLN**	mesothelin	protein_coding	0	0	1	1	1.000
10233	NM_001135217,NM	**LRRC23**	leucine rich repeat conta	protein_coding	2	2	3	3	1.228
10234	NM_001031692,NM	**LRRC17**	leucine rich repeat conta	protein_coding	0	0	1	1	1.000
10235	NM_001098670,NM	**RASGRP2**	RAS guanyl releasing pr	protein_coding	22	25	23	26	1.140
10236	NM_001102397,NM	**HNRNPR**	heterogeneous nuclear r	protein_coding	447	455	448	456	1.018
10237	NM_001278784,NM	**SLC35B1**	solute carrier family 35 n	protein_coding	94	97	95	98	1.029
10238	NM_005828,NR_07	**DCAF7**	DDB1 and CUL4 associa	protein_coding	86	81	87	82	0.941
10239	NM_005829,NR_02	**AP3S2**	adaptor related protein c	protein_coding	23	23	24	24	0.996
10240	NM_005830,XR_00	**MRPS31**	mitochondrial ribosomal	protein_coding	54	47	55	48	0.882
10241	NM_001261390,NM	**CALCOCO2**	calcium binding and coile	protein_coding	23	21	24	22	0.918
10242	NM_001278911,NM	**KCNMB2**	potassium calcium-activa	protein_coding	0	0	1	1	1.000
10243	NM_001024218,NM	**GPHN**	gephyrin	protein_coding	0	0	1	1	1.000
10244	NM_001174152,NM	**RABEPK**	Rab9 effector protein wit	protein_coding	59	55	60	56	0.937
10245	NM_001167947,NM	**TIMM17B**	translocase of inner mito	protein_coding	113	121	114	122	1.070
10246	NM_001286123,NM	**SLC17A2**	solute carrier family 17 n	protein_coding	0	0	1	1	1.000
10247	NM_005836	**RIDA**	reactive intermediate imi	protein_coding	65	65	66	66	0.990
10248	NM_005837	**POP7**	POP7 homolog, ribonucl	protein_coding	128	124	129	125	0.968
10249	NM_005838,NM_20	**GLYAT**	glycine-N-acyltransferase	protein_coding	0	0	1	1	1.000
10250	NM_001303448,NM	**SRRM1**	serine and arginine repe	protein_coding	119	100	120	101	0.842
10251	NM_001304990,NM	**SPRY3**	sprouty RTK signaling ar	protein_coding	0	0	1	1	1.009
10252	NM_001258038,NM	**SPRY1**	sprouty RTK signaling ar	protein_coding	17	14	18	15	0.843
10253	NM_001318536,NM	**SPRY2**	sprouty RTK signaling ar	protein_coding	0	0	1	1	1.000
10254	NM_005843,XR_00	**STAM2**	signal transducing adapt	protein_coding	13	10	14	11	0.776
10255	NR_028032	**HCG9**	HLA complex group 9	lncRNA	0	0	1	1	1.000
10256	NM_001297647,NM	**CNKSR1**	connector enhancer of ki	protein_coding	1	0	2	1	0.853
10257	NM_001105515,NM	**ABCC4**	ATP binding cassette sul	protein_coding	20	17	21	18	0.880
10260	NM_001144823,NM	**DENND4A**	DENN domain containing	protein_coding	14	12	15	13	0.900
10261	NM_005849	**IGSF6**	immunoglobulin superfar	protein_coding	0	0	1	1	0.708
10262	NM_005850	**SF3B4**	splicing factor 3b subuni	protein_coding	239	255	240	256	1.064
10263	NM_001271849,NM	**CDK2AP2**	cyclin dependent kinase	protein_coding	72	75	73	76	1.044

10265	NM_001252197,NM	**IRX5**	iroquois homeobox 5	protein_coding	0	0	1	1	1.000
10266	NM_005854	**RAMP2**	receptor activity modifyin	protein_coding	0	1	1	2	1.132
10267	NM_001308353,NM	**RAMP1**	receptor activity modifyin	protein_coding	0	0	1	1	1.000
10268	NM_005856,XM_00	**RAMP3**	receptor activity modifyin	protein_coding	0	0	1	1	1.000
10269	NM_005857,XR_00	**ZMPSTE24**	zinc metallopeptidase ST	protein_coding	133	118	134	119	0.889
10270	NM_005858	**AKAP8**	A-kinase anchoring prote	protein_coding	41	36	42	37	0.877
10272	NM_005860	**FSTL3**	follistatin like 3	protein_coding	4	5	5	6	1.071
10273	NM_001293197,NM	**STUB1**	STIP1 homology and U-b	protein_coding	108	114	109	115	1.059
10274	NM_005862,XM_01	**STAG1**	stromal antigen 1	protein_coding	40	37	41	38	0.936
10276	NM_001047160,NM	**NET1**	neuroepithelial cell trans	protein_coding	60	51	61	52	0.863
10277	NM_001105562,NM	**UBE4B**	ubiquitination factor E4B	protein_coding	26	25	27	26	0.965
10278	NM_001277174,NM	**EFS**	embryonal Fyn-associate	protein_coding	0	0	1	1	1.000
10279	NM_005865,XM_01	**PRSS16**	serine protease 16	protein_coding	0	0	1	1	1.000
10280	NM_001282205,NM	**SIGMAR1**	sigma non-opioid intrace	protein_coding	208	229	209	230	1.099
10281	NR_147130	**DSCR4**	Down syndrome critical r	lncRNA	0	0	1	1	0.866
10282	NM_001317739,NM	**BET1**	Bet1 golgi vesicular mem	protein_coding	49	46	50	47	0.950
10283	NM_001297644,NM	**CWC27**	CWC27 spliceosome ass	protein_coding	62	53	63	54	0.856
10284	NM_001366643,NM	**SAP18**	Sin3A associated protein	protein_coding	187	199	188	200	1.065
10285	NM_005871,XM_00	**SMNDC1**	survival motor neuron do	protein_coding	56	44	57	45	0.779
10286	NM_005872	**BCAS2**	BCAS2 pre-mRNA proce	protein_coding	169	172	170	173	1.023
10287	NM_001039467,NM	**RGS19**	regulator of G protein sig	protein_coding	27	27	28	28	0.982
10288	NM_001080978,NM	**LILRB2**	leukocyte immunoglobul	protein_coding	0	0	1	1	1.000
10289	NM_005875	**EIF1B**	eukaryotic translation init	protein_coding	74	61	75	62	0.822
10290	NM_001173476,NM	**SPEG**	striated muscle enriched	protein_coding	0	0	1	1	1.000
10291	NM_005877	**SF3A1**	splicing factor 3a subuni	protein_coding	103	104	104	105	1.010
10293	NM_005879,XM_01	**TRAIP**	TRAF interacting protein	protein_coding	25	26	26	27	1.048
10294	NM_005880	**DNAJA2**	DnaJ heat shock protein	protein_coding	103	99	104	100	0.954
10295	NM_001122957,NM	**BCKDK**	branched chain keto acid	protein_coding	39	37	40	38	0.940
10296	NM_001017405,NM	**MAEA**	macrophage erythroblast	protein_coding	80	91	81	92	1.129
10297	NM_001351273,NM	**APC2**	APC regulator of WNT si	protein_coding	0	0	1	1	1.075
10298	NM_001014831,NM	**PAK4**	p21 (RAC1) activated kin	protein_coding	20	20	21	21	1.015
10299	NM_001270660,NM	**MARCHF6**	membrane associated rin	protein_coding	29	28	30	29	0.984
10300	NM_005886	**KATNB1**	katanin regulatory subur	protein_coding	29	30	30	31	1.014
10301	NR_002605,NR_10	**DLEU1**	deleted in lymphocytic le	lncRNA	30	28	31	29	0.931
10302	NM_001329613,NM	**SNAPC5**	small nuclear RNA activa	protein_coding	49	51	50	52	1.022
10307	NM_006051,NM_13	**APBB3**	amyloid beta precursor p	protein_coding	4	5	5	6	1.268
10308	NM_001265588,NM	**ZNF267**	zinc finger protein 267	protein_coding	36	31	37	32	0.875
10309	NM_021147,NR_12	**CCNO**	cyclin O	protein_coding	0	0	1	1	1.000
10311	NM_001331018,NM	**VPS26C**	VPS26 endosomal protei	protein_coding	29	22	30	23	0.786
10312	NM_001351059,NM	**TCIRG1**	T cell immune regulator	protein_coding	19	22	20	23	1.139
10313	NM_001265589,NM	**RTN3**	reticulon 3	protein_coding	118	91	119	92	0.773
10314	NM_001136574,NM	**LANCL1**	LanC like 1	protein_coding	56	47	57	48	0.845
10316	NM_006056,XM_00	**NMUR1**	neuromedin U receptor 1	protein_coding	21	21	22	22	0.980
10317	NM_001278650,NM	**B3GALT5**	beta-1,3-galactosyltransf	protein_coding	0	0	1	1	0.993
10318	NM_001252385,NM	**TNIP1**	TNFAIP3 interacting prot	protein_coding	64	65	65	66	1.028
10319	NM_006059,XM_00	**LAMC3**	laminin subunit gamma 3	protein_coding	0	0	1	1	1.039
10320	NM_001220765,NM	**IKZF1**	IKAROS family zinc finge	protein_coding	23	24	24	25	1.055
10321	NM_001190986,NM	**CRISP3**	cysteine rich secretory pr	protein_coding	0	0	1	1	1.000
10322	NM_006062	**SMYD5**	SMYD family member 5	protein_coding	49	50	50	51	1.011
10324	NM_006063	**KLHL41**	kelch like family member	protein_coding	0	0	1	1	1.041
10325	NM_001354011,NM	**RRAGB**	Ras related GTP binding	protein_coding	4	3	5	4	0.867
10326	NM_001083910,NM	**SIRPB1**	signal regulatory protein	protein_coding	1	1	2	2	1.103
10327	NM_001202413,NM	**AKR1A1**	aldo-keto reductase fami	protein_coding	144	154	145	155	1.067
10328	NM_001142288,NM	**EMC8**	ER membrane protein cc	protein_coding	47	52	48	53	1.116
10329	NM_001278237,NM	**RXYLT1**	ribitol xylosyltransferase	protein_coding	38	38	39	39	1.000
10330	NM_001190991,NM	**CNPY2**	canopy FGF signaling re	protein_coding	131	126	132	127	0.962
10331	NM_014256,XM_01	**B3GNT3**	UDP-GlcNAc:betaGal be	protein_coding	0	0	1	1	1.000
10332	NM_001144904,NM	**CLEC4M**	C-type lectin domain fam	protein_coding	0	0	1	1	1.000
10333	NM_006068,XM_00	**TLR6**	toll like receptor 6	protein_coding	1	1	2	2	0.961
10335	NM_001098579,NM	**IRAG1**	inositol 1,4,5-triphosphat	protein_coding	0	0	1	1	1.000
10336	NM_001317836,NM	**PCGF3**	polycomb group ring fing	protein_coding	6	5	7	6	0.921
10342	NM_001007565,NM	**TFG**	trafficking from ER to gol	protein_coding	56	54	57	55	0.964
10343	NM_006071	**PKDREJ**	polycystin family recepto	protein_coding	0	0	1	1	1.000
10344	NM_001371936,NM	**CCL26**	C-C motif chemokine liga	protein_coding	0	0	1	1	1.000
10345	NM_001251987,NM	**TRDN**	triadin	protein_coding	2	2	3	3	0.881
10346	NM_001199573,NM	**TRIM22**	tripartite motif containing	protein_coding	13	11	14	12	0.889
10347	NM_019112,XM_00	**ABCA7**	ATP binding cassette sul	protein_coding	5	5	6	6	1.092
10349	NM_001377321,NM	**ABCA10**	ATP binding cassette sul	protein_coding	0	0	1	1	1.000
10350	NM_080283,XM_01	**ABCA9**	ATP binding cassette sul	protein_coding	0	0	1	1	1.000
10351	NM_001288985,NM	**ABCA8**	ATP binding cassette sul	protein_coding	0	0	1	1	1.000
10352	NM_001378226,NM	**WARS2**	tryptophanyl tRNA synth	protein_coding	10	8	11	9	0.864
10354	gene-HMGB1P5	**HMGB1P5**	high mobility group box 1	pseudogene	350	352	351	353	1.004
10355	gene-HMGB1P4	**HMGB1P4**	high mobility group box 1	pseudogene	0	0	1	1	1.006
10356	gene-HMGB1P3	**HMGB1P3**	high mobility group box 1	pseudogene	1	1	2	2	0.970
10357	gene-HMGB1P1	**HMGB1P1**	high mobility group box 1	pseudogene	5	4	6	5	0.799
10360	NM_006993	**NPM3**	nucleophosmin/nucleopl	protein_coding	102	95	103	96	0.933
10361	NM_001286680,NM	**NPM2**	nucleophosmin/nucleopl	protein_coding	0	0	1	1	1.000
10362	NM_006339,XM_01	**HMG20B**	high mobility group 20B	protein_coding	24	23	25	24	0.935
10363	NM_001304504,NM	**HMG20A**	high mobility group 20A	protein_coding	49	42	50	43	0.862
10365	NM_016270	**KLF2**	Kruppel like factor 2	protein_coding	13	16	14	17	1.168
10367	NM_001195518,NM	**MICU1**	mitochondrial calcium up	protein_coding	31	32	32	33	1.036
10368	NM_006539	**CACNG3**	calcium voltage-gated ch	protein_coding	0	0	1	1	1.000
10369	NM_001379051,NM	**CACNG2**	calcium voltage-gated ch	protein_coding	0	0	1	1	1.000
10370	NM_001168388,NM	**CITED2**	Cbp/p300 interacting trar	protein_coding	23	25	24	26	1.069
10371	NM_006080,XM_00	**SEMA3A**	semaphorin 3A	protein_coding	0	0	1	1	1.035
10376	NM_006082	**TUBA1B**	tubulin alpha 1b	protein_coding	3,807	3,978	3,808	3,979	1.045
10379	NM_006084	**IRF9**	interferon regulatory fact	protein_coding	47	43	48	44	0.920
10380	NM_001286149,NM	**BPNT1**	3'(2'), 5'-bisphosphate nu	protein_coding	44	38	45	39	0.866
10381	NM_001197181,NM	**TUBB3**	tubulin beta 3 class III	protein_coding	4	6	5	7	1.283
10382	NM_001289123,NM	**TUBB4A**	tubulin beta 4A class IVa	protein_coding	0	0	1	1	1.040
10383	NM_006088	**TUBB4B**	tubulin beta 4B class IVb	protein_coding	629	700	630	701	1.112
10384	NM_001242803,NM	**BTN3A3**	butyrophilin subfamily 3	protein_coding	20	18	21	19	0.874
10385	NM_001197237,NM	**BTN2A2**	butyrophilin subfamily 2	protein_coding	7	5	8	6	0.768
10388	NM_014258,XM_01	**SYCP2**	synaptonemal complex p	protein_coding	0	0	1	1	1.016
10389	NM_006089,NR_03	**SCML2**	Scm polycomb group pro	protein_coding	0	0	1	1	1.000
10390	NM_001007794,NM	**CEPT1**	choline/ethanolamine ph	protein_coding	41	37	42	38	0.913
10391	NM_001190456,NM	**CORO2B**	coronin 2B	protein_coding	0	0	1	1	1.000
10392	NM_001354849,NM	**NOD1**	nucleotide binding oligor	protein_coding	4	4	5	5	0.987
10393	NM_001256706,NM	**ANAPC10**	anaphase promoting con	protein_coding	29	25	30	26	0.866
10394	NM_006093	**PRG3**	proteoglycan 3, pro eosir	protein_coding	0	0	1	1	1.000
10395	NM_001164271,NM	**DLC1**	DLC1 Rho GTPase activa	protein_coding	0	0	1	1	1.000
10396	NM_001105529,NM	**ATP8A1**	ATPase phospholipid tra	protein_coding	7	6	8	7	0.878
10397	NM_001135242,NM	**NDRG1**	N-myc downstream regu	protein_coding	3	3	4	4	1.253
10398	NM_006097,NM_18	**MYL9**	myosin light chain 9	protein_coding	0	0	1	1	1.000
10399	NM_006098	**RACK1**	receptor for activated C k	protein_coding	2,175	2,281	2,176	2,282	1.049
10400	NM_001267551,NM	**PEMT**	phosphatidylethanolamir	protein_coding	10	14	11	15	1.390
10401	NM_006099	**PIAS3**	protein inhibitor of activa	protein_coding	13	13	14	14	1.036
10402	NM_001271142,NM	**ST3GAL6**	ST3 beta-galactoside alp	protein_coding	0	0	1	1	0.981
10403	NM_006101	**NDC80**	NDC80 kinetochore comp	protein_coding	68	58	69	59	0.857
10404	NM_016134	**CPQ**	carboxypeptidase Q	protein_coding	0	0	1	1	1.000
10406	NM_006103	**WFDC2**	WAP four-disulfide core	protein_coding	0	0	1	1	1.000
10407	NM_016512,NM_05	**SPAG11B**	sperm associated antige	protein_coding	0	0	1	1	1.000
10408	NR_110230,NR_16	**MYCNOS**	MYCN opposite strand	lncRNA	0	0	1	1	1.000
10409	NM_001271606,NM	**BASP1**	brain abundant membrar	protein_coding	0	0	1	1	1.000
10410	NM_021034,NR_04	**IFITM3**	interferon induced transr	protein_coding	11	17	12	18	1.572
10411	NM_001098531,NM	**RAPGEF3**	Rap guanine nucleotide	protein_coding	0	0	1	1	0.986
10412	NM_001271665,NM	**NSA2**	NSA2 ribosome biogenes	protein_coding	84	80	85	81	0.945
10413	NM_001130145,NM	**YAP1**	Yes1 associated transcri	protein_coding	0	0	1	1	1.000
10415	gene-SNAI1P1	**SNAI1P1**	snail family zinc finger 1	pseudogene	0	0	1	1	1.000
10417	NM_001128325,NM	**SPON2**	spondin 2	protein_coding	0	0	1	1	1.036
10418	NM_006108	**SPON1**	spondin 1	protein_coding	0	0	1	1	1.000
10419	NM_001039619,NM	**PRMT5**	protein arginine methyltr	protein_coding	129	135	130	136	1.050
10420	NM_001320800,NM	**TESK2**	testis associated actin re	protein_coding	1	1	2	2	0.872
10421	NM_001243646,NM	**CD2BP2**	CD2 cytoplasmic tail binc	protein_coding	107	108	108	109	1.016
10422	NM_016172	**UBAC1**	UBA domain containing 1	protein_coding	47	46	48	47	0.976
10423	NM_001286585,NM	**CDIPT**	CDP-diacylglycerol--inos	protein_coding	56	55	57	56	0.977

10424	NM_006320,XM_01	**PGRMC2**	progesterone receptor m	protein_coding	39	34	40	35	0.884
10425	NM_001317333,NM	**ARIH2**	ariadne RBR E3 ubiquitin	protein_coding	90	73	91	74	0.816
10426	NM_001286277,NM	**TUBGCP3**	tubulin gamma complex	protein_coding	57	58	58	59	1.031
10427	NM_001042734,NM	**SEC24B**	SEC24 homolog B, COP	protein_coding	18	14	19	15	0.787
10428	NM_006324,XM_01	**CFDP1**	craniofacial development	protein_coding	41	48	42	49	1.159
10430	NM_001242597,NM	**TMEM147**	transmembrane protein 1	protein_coding	102	104	103	105	1.013
10432	NM_001198836,NM	**RBM14**	RNA binding motif protei	protein_coding	52	56	53	57	1.087
10434	NM_001279356,NM	**LYPLA1**	lysophospholipase 1	protein_coding	159	139	160	140	0.878
10435	NM_006779	**CDC42EP2**	CDC42 effector protein 2	protein_coding	0	0	1	1	0.962
10436	NM_001320049,NM	**EMG1**	EMG1 N1-specific pseud	protein_coding	28	27	29	28	0.967
10437	NM_006332	**IFI30**	IFI30 lysosomal thiol red	protein_coding	230	247	231	248	1.073
10438	NM_001190263,NM	**C1D**	C1D nuclear receptor co	protein_coding	34	33	35	34	0.981
10439	NM_001282611,NM	**OLFM1**	olfactomedin 1	protein_coding	0	0	1	1	1.000
10440	NM_006335	**TIMM17A**	translocase of inner mito	protein_coding	183	183	184	184	0.999
10443	NM_001278432,NM	**N4BP2L2**	NEDD4 binding protein 2	protein_coding	23	21	24	22	0.894
10444	NM_001375954,NM	**ZER1**	zyg-11 related cell cycle	protein_coding	2	2	3	3	0.958
10445	NM_001012300,NM	**MCRS1**	microspherule protein 1	protein_coding	40	51	41	52	1.264
10446	NM_006338,NM_20	**LRRN2**	leucine rich repeat neuro	protein_coding	0	0	1	1	1.000
10447	NM_001040020,NM	**FAM3C**	FAM3 metabolism regula	protein_coding	499	482	500	483	0.966
10449	NM_006111	**ACAA2**	acetyl-CoA acyltransfera	protein_coding	200	182	201	183	0.912
10450	NM_001195007,NM	**PPIE**	peptidylprolyl isomerase	protein_coding	45	33	46	34	0.737
10451	NM_001079874,NM	**VAV3**	vav guanine nucleotide e	protein_coding	0	0	1	1	0.930
10452	NM_001128916,NM	**TOMM40**	translocase of outer mito	protein_coding	474	506	475	507	1.067
10454	NM_006116,NM_15	**TAB1**	TGF-beta activated kinas	protein_coding	26	27	27	28	1.040
10455	NM_001166010,NM	**ECI2**	enoyl-CoA delta isomera	protein_coding	42	37	43	38	0.879
10456	NM_001018837,NM	**HAX1**	HCLS1 associated protei	protein_coding	215	217	216	218	1.009
10457	NM_001005340,NM	**GPNMB**	glycoprotein nmb	protein_coding	1	1	2	2	0.953
10458	NM_001144888,NM	**BAIAP2**	BAR/IMD domain contain	protein_coding	0	0	1	1	1.000
10459	NM_001127325,NM	**MAD2L2**	mitotic arrest deficient 2	protein_coding	300	327	301	328	1.089
10460	NM_006342,XM_00	**TACC3**	transforming acidic coile	protein_coding	135	134	136	135	0.993
10461	NM_006343	**MERTK**	MER proto-oncogene, ty	protein_coding	1	1	2	2	1.141
10462	NM_001330070,NM	**CLEC10A**	C-type lectin domain con	protein_coding	0	0	1	1	1.000
10463	NM_006345,XM_01	**SLC30A9**	solute carrier family 30 m	protein_coding	55	45	56	46	0.819
10464	NM_001349655,NM	**PIBF1**	progesterone immunomo	protein_coding	17	14	18	15	0.869
10465	NM_001330510,NM	**PPIH**	peptidylprolyl isomerase	protein_coding	197	196	198	197	0.994
10466	NM_001161520,NM	**COG5**	component of oligomeric	protein_coding	21	18	22	19	0.866
10467	NM_006349,XM_01	**ZNHIT1**	zinc finger HIT-type cont	protein_coding	104	107	105	108	1.035
10468	NM_006350,NM_01	**FST**	follistatin	protein_coding	1	1	2	2	1.167
10469	NM_006351	**TIMM44**	translocase of inner mito	protein_coding	123	121	124	122	0.983
10471	NM_001185181,NM	**PFDN6**	prefoldin subunit 6	protein_coding	327	335	328	336	1.024
10472	NM_001278196,NM	**ZBTB18**	zinc finger and BTB dom	protein_coding	1	1	2	2	0.952
10473	NM_006353,XM_01	**HMGN4**	high mobility group nucle	protein_coding	120	105	121	106	0.874
10474	NM_001278270,NM	**TADA3**	transcriptional adaptor 3	protein_coding	110	117	111	118	1.056
10475	NM_006355,XM_00	**TRIM38**	tripartite motif containing	protein_coding	14	12	15	13	0.895
10476	NM_001003785,NM	**ATP5PD**	ATP synthase peripheral	protein_coding	442	445	443	446	1.006
10477	NM_001278554,NM	**UBE2E3**	ubiquitin conjugating enz	protein_coding	17	13	18	14	0.776
10478	NM_001282726,NM	**SLC25A17**	solute carrier family 25 m	protein_coding	44	43	45	44	0.967
10479	NM_001042537,NM	**SLC9A6**	solute carrier family 9 me	protein_coding	20	20	21	21	1.005
10480	NM_001307929,NM	**EIF3M**	eukaryotic translation init	protein_coding	127	127	128	128	1.001
10481	NM_006361	**HOXB13**	homeobox B13	protein_coding	1	1	2	2	0.887
10482	NM_001081491,NM	**NXF1**	nuclear RNA export facto	protein_coding	73	77	74	78	1.054
10483	NM_001172745,NM	**SEC23B**	SEC23 homolog B, COP	protein_coding	105	100	106	101	0.953
10484	NM_006364,XM_00	**SEC23A**	Sec23 homolog A, COPI	protein_coding	37	32	38	33	0.864
10485	NR_135260,NR_13	**MIR9-1HG**	MIR9-1 host gene	lncRNA	0	0	1	1	1.000
10486	NM_001363533,NM	**CAP2**	cyclase associated actin	protein_coding	0	0	1	1	1.000
10487	NM_001105530,NM	**CAP1**	cyclase associated actin	protein_coding	447	422	448	423	0.944
10488	NM_006368	**CREB3**	cAMP responsive elemen	protein_coding	29	31	30	32	1.092
10489	NM_006369,XR_00	**LRRC41**	leucine rich repeat conta	protein_coding	26	26	27	27	0.993
10490	NM_006370	**VTI1B**	vesicle transport through	protein_coding	35	33	36	34	0.950
10491	NM_006371	**CRTAP**	cartilage associated prot	protein_coding	3	3	4	4	0.944
10492	NM_001159673,NM	**SYNCRIP**	synaptotagmin binding c	protein_coding	291	256	292	257	0.881
10493	NM_006373	**VAT1**	vesicle amine transport 1	protein_coding	1	1	2	2	0.904
10494	NM_001271977,NM	**STK25**	serine/threonine kinase 2	protein_coding	48	52	49	53	1.080
10495	NM_001281736,NM	**ENOX2**	ecto-NOX disulfide-thiol	protein_coding	9	8	10	9	0.883
10497	NM_001330653,NM	**UNC13B**	unc-13 homolog B	protein_coding	0	0	1	1	1.000
10498	NM_001370088,NM	**CARM1**	coactivator associated ar	protein_coding	67	68	68	69	1.012
10499	NM_001321703,NM	**NCOA2**	nuclear receptor coactiva	protein_coding	23	18	24	19	0.823
10500	NM_001178061,NM	**SEMA6C**	semaphorin 6C	protein_coding	0	0	1	1	0.931
10501	NM_032108,XM_01	**SEMA6B**	semaphorin 6B	protein_coding	0	0	1	1	1.000
10505	NM_001271661,NM	**SEMA4F**	ssemaphorin 4F	protein_coding	1	1	2	2	1.042
10507	NM_001142287,NM	**SEMA4D**	semaphorin 4D	protein_coding	230	197	231	198	0.855
10509	NM_001324029,NM	**SEMA4B**	semaphorin 4B	protein_coding	2	2	3	3	0.910
10512	NM_001350120,NM	**SEMA3C**	semaphorin 3C	protein_coding	0	0	1	1	1.000
10513	NM_001282476,NM	**APPBP2**	amyloid beta precursor p	protein_coding	16	14	17	15	0.894
10514	NM_001105538,NM	**MYBBP1A**	MYB binding protein 1a	protein_coding	57	60	58	61	1.047
10516	NM_006329,XM_00	**FBLN5**	fibulin 5	protein_coding	0	0	1	1	0.957
10517	NM_001267585,NM	**FBXW10**	F-box and WD repeat do	protein_coding	0	0	1	1	1.000
10518	NM_001271888,NM	**CIB2**	calcium and integrin binc	protein_coding	0	1	1	2	1.270
10519	NM_001277764,NM	**CIB1**	calcium and integrin binc	protein_coding	79	86	80	87	1.095
10520	NM_001265597,NM	**ZNF211**	zinc finger protein 211	protein_coding	9	8	10	9	0.918
10521	NM_001098504,NM	**DDX17**	DEAD-box helicase 17	protein_coding	170	148	171	149	0.869
10522	NM_001293634,NM	**DEAF1**	DEAF1 transcription facto	protein_coding	21	24	22	25	1.156
10523	NM_006387	**CHERP**	calcium homeostasis enc	protein_coding	65	65	66	66	1.004
10524	NM_001206833,NM	**KAT5**	lysine acetyltransferase 5	protein_coding	31	40	32	41	1.277
10525	NM_001130991,NM	**HYOU1**	hypoxia up-regulated 1	protein_coding	195	199	196	200	1.024
10526	NM_001190995,NM	**IPO8**	importin 8	protein_coding	27	24	28	25	0.865
10527	NM_006391	**IPO7**	importin 7	protein_coding	182	154	183	155	0.847
10528	NM_006392,NR_02	**NOP56**	NOP56 ribonucleoprotein	protein_coding	423	418	424	419	0.986
10529	NM_001173484,NM	**NEBL**	nebulette	protein_coding	0	0	1	1	1.000
10531	NM_001242307,NM	**PITRM1**	pitrilysin metallopeptidas	protein_coding	32	33	33	34	1.027
10533	NM_001136031,NM	**ATG7**	autophagy related 7	protein_coding	7	7	8	8	0.965
10534	NM_001303024,NM	**ZNRD2**	zinc ribbon domain conta	protein_coding	110	116	111	117	1.060
10535	NM_006397	**RNASEH2A**	ribonuclease H2 subunit	protein_coding	111	113	112	114	1.015
10536	NM_014262	**P3H3**	prolyl 3-hydroxylase 3	protein_coding	0	0	1	1	1.000
10537	NM_006398	**UBD**	ubiquitin D	protein_coding	0	0	1	1	1.000
10538	NM_006399	**BATF**	basic leucine zipper ATF	protein_coding	95	112	96	113	1.174
10539	NM_001199868,NM	**GLRX3**	glutaredoxin 3	protein_coding	115	108	116	109	0.938
10540	NM_001261412,NM	**DCTN2**	dynactin subunit 2	protein_coding	84	82	85	83	0.976
10541	NM_006401	**ANP32B**	acidic nuclear phosphop	protein_coding	451	473	452	474	1.049
10542	NM_001382293,NM	**LAMTOR5**	late endosomal/lysosoma	protein_coding	201	211	202	212	1.052
10544	NM_006404,XM_01	**PROCR**	protein C receptor	protein_coding	0	0	1	1	1.000
10548	NM_001014842,NM	**TM9SF1**	transmembrane 9 superf	protein_coding	19	21	20	22	1.088
10549	NM_006406	**PRDX4**	peroxiredoxin 4	protein_coding	144	146	145	147	1.010
10550	NM_006407	**ARL6IP5**	ADP ribosylation factor li	protein_coding	126	124	127	125	0.982
10551	NM_006408,XM_00	**AGR2**	anterior gradient 2, prote	protein_coding	0	0	1	1	1.000
10552	NM_001190996,NM	**ARPC1A**	actin related protein 2/3 c	protein_coding	130	140	131	141	1.073
10553	NM_001098520,NM	**HTATIP2**	HIV-1 Tat interactive prot	protein_coding	28	30	29	31	1.058
10554	NM_001371437,NM	**AGPAT1**	1-acylglycerol-3-phospha	protein_coding	82	82	83	83	1.002
10555	NM_001012727,NM	**AGPAT2**	1-acylglycerol-3-phospha	protein_coding	2	2	3	3	1.056
10556	NM_001104546,NM	**RPP30**	ribonuclease P/MRP sub	protein_coding	24	21	25	22	0.904
10557	NM_001097590,NM	**RPP38**	ribonuclease P/MRP sub	protein_coding	19	20	20	21	1.052
10558	NM_001281303,NM	**SPTLC1**	serine palmitoyltransfera	protein_coding	75	66	76	67	0.890
10559	NM_001168398,NM	**SLC35A1**	solute carrier family 35 m	protein_coding	33	32	34	33	0.992
10560	NM_001319667,NM	**SLC19A2**	solute carrier family 19 m	protein_coding	6	5	7	6	0.801
10561	NM_006417,NR_13	**IFI44**	interferon induced protei	protein_coding	69	54	70	55	0.776
10562	NM_006418	**OLFM4**	olfactomedin 4	protein_coding	0	0	1	1	1.000
10563	NM_001371558,NM	**CXCL13**	C-X-C motif chemokine li	protein_coding	0	0	1	1	1.083
10564	NM_006420,XM_00	**ARFGEF2**	ADP ribosylation factor g	protein_coding	46	39	47	40	0.857
10565	NM_006421,XM_00	**ARFGEF1**	ADP ribosylation factor g	protein_coding	34	30	35	31	0.865
10566	NM_001278309,NM	**AKAP3**	A-kinase anchoring prote	protein_coding	0	0	1	1	1.080
10567	NM_006423	**RABAC1**	Rab acceptor 1	protein_coding	154	161	155	162	1.045
10568	NM_001177998,NM	**SLC34A2**	solute carrier family 34 m	protein_coding	0	0	1	1	1.000
10569	NM_001364517,NM	**SLU7**	SLU7 homolog, splicing	protein_coding	34	34	35	35	0.993
10570	NM_006426,XM_00	**DPYSL4**	dihydropyrimidinase like	protein_coding	0	0	1	1	1.000
10572	NM_006427,NM_02	**SIVA1**	SIVA1 apoptosis inducin	protein_coding	142	145	143	146	1.025
10573	NM_006428,XM_00	**MRPL28**	mitochondrial ribosomal	protein_coding	163	162	164	163	0.996

10574	NM_001009570,NM	**CCT7**	chaperonin containing T(	protein_coding	947	937	948	938	0.989
10575	NM_001256721,NM	**CCT4**	chaperonin containing T(	protein_coding	378	363	379	364	0.961
10576	NM_001198842,NM	**CCT2**	chaperonin containing T(	protein_coding	470	448	471	449	0.953
10577	NM_001363688,NM	**NPC2**	NPC intracellular cholest	protein_coding	46	44	47	45	0.965
10578	NM_001302758,NM	**GNLY**	granulysin	protein_coding	197	222	198	223	1.125
10579	NM_001291876,NM	**TACC2**	transforming acidic coile	protein_coding	0	0	1	1	1.000
10580	NM_001034954,NM	**SORBS1**	sorbin and SH3 domain (	protein_coding	0	0	1	1	0.966
10581	NM_006435	**IFITM2**	interferon induced transr	protein_coding	46	51	47	52	1.126
10584	NM_001324095,NM	**COLEC10**	collectin subfamily meml	protein_coding	0	0	1	1	1.000
10585	NM_001077365,NM	**POMT1**	protein O-mannosyltrans	protein_coding	7	7	8	8	0.993
10586	NM_006439	**MAB21L2**	mab-21 like 2	protein_coding	0	0	1	1	1.000
10587	NM_001282512,NM	**TXNRD2**	thioredoxin reductase 2	protein_coding	12	12	13	13	1.001
10588	NM_001199758,NM	**MTHFS**	methenyltetrahydrofolate	protein_coding	20	25	21	26	1.230
10589	NM_006442	**DRAP1**	DR1 associated protein 1	protein_coding	328	391	329	392	1.190
10590	NM_006998,XM_01	**SCGN**	secretagogin, EF-hand c	protein_coding	0	0	1	1	1.000
10591	NM_006443,NM_19	**DNPH1**	2'-deoxynucleoside 5'-ph	protein_coding	90	99	91	100	1.101
10592	NM_001042550,NM	**SMC2**	structural maintenance c	protein_coding	66	58	67	59	0.880
10594	NM_006445,XM_02	**PRPF8**	pre-mRNA processing fa	protein_coding	120	109	121	110	0.906
10595	NM_001308220,NM	**ERN2**	endoplasmic reticulum to	protein_coding	0	0	1	1	1.000
10597	NM_001355204	**TRAPPC2B**	trafficking protein particle	protein_coding	16	14	17	15	0.902
10598	NM_001321441,NM	**AHSA1**	activator of HSP90 ATPa	protein_coding	443	444	444	445	1.003
10599	NM_006446	**SLCO1B1**	solute carrier organic ani	protein_coding	0	0	1	1	1.000
10600	NM_001001992,NM	**USP16**	ubiquitin specific peptida	protein_coding	64	57	65	58	0.886
10602	NM_001270436,NM	**CDC42EP3**	CDC42 effector protein 3	protein_coding	17	14	18	15	0.824
10603	NM_001359228,NM	**SH2B2**	SH2B adaptor protein 2	protein_coding	0	0	1	1	0.970
10605	NM_006451,NM_18	**PAIP1**	poly(A) binding protein in	protein_coding	50	47	51	48	0.940
10606	NM_001079524,NM	**PAICS**	phosphoribosylaminoimi(	protein_coding	430	411	431	412	0.957
10607	NM_006453	**TBL3**	transducin beta like 3	protein_coding	29	29	30	30	1.023
10608	NM_006454	**MXD4**	MAX dimerization protein	protein_coding	4	5	5	6	1.043
10609	NM_006455,XM_00	**P3H4**	prolyl 3-hydroxylase fam	protein_coding	0	0	1	1	1.000
10610	NM_006456,XM_00	**ST6GALNAC2**	ST6 N-acetylgalactosami	protein_coding	0	0	1	1	1.000
10611	NM_001011513,NM	**PDLIM5**	PDZ and LIM domain 5	protein_coding	4	4	5	5	0.966
10612	NM_001248006,NM	**TRIM3**	tripartite motif containing	protein_coding	0	0	1	1	0.979
10613	NM_001100626,NM	**ERLIN1**	ER lipid raft associated 1	protein_coding	27	28	28	29	1.029
10614	NM_006460	**HEXIM1**	HEXIM P-TEFb complex	protein_coding	81	89	82	90	1.098
10615	NM_006461	**SPAG5**	sperm associated antige	protein_coding	107	95	108	96	0.895
10616	NM_001323956,NM	**RBCK1**	RANBP2-type and C3HC	protein_coding	109	106	110	107	0.976
10617	NM_001353967,NM	**STAMBP**	STAM binding protein	protein_coding	31	28	32	29	0.887
10618	NM_001206844,NM	**TGOLN2**	trans-golgi network prote	protein_coding	124	112	125	113	0.907
10620	NM_001307939,NM	**ARID3B**	AT-rich interaction domai	protein_coding	137	138	138	139	1.006
10621	NM_001282526,NM	**POLR3F**	RNA polymerase III subu	protein_coding	30	28	31	29	0.957
10622	NM_001370351,NM	**POLR3G**	RNA polymerase III subu	protein_coding	77	60	78	61	0.784
10623	NM_001303456,NM	**POLR3C**	RNA polymerase III subu	protein_coding	53	52	54	53	0.986
10625	NM_006469,XM_00	**IVNS1ABP**	influenza virus NS1A bin	protein_coding	139	131	140	132	0.947
10626	NM_001348119,NM	**TRIM16**	tripartite motif containing	protein_coding	4	3	5	4	0.881
10627	NM_001303047,NM	**MYL12A**	myosin light chain 12A	protein_coding	684	650	685	651	0.950
10628	NM_001313972,NM	**TXNIP**	thioredoxin interacting pr	protein_coding	112	127	113	128	1.136
10629	NM_006473,XM_00	**TAF6L**	TATA-box binding protein	protein_coding	15	14	16	15	0.959
10630	NM_001006624,NM	**PDPN**	podoplanin	protein_coding	0	0	1	1	1.000
10631	NM_001135934,NM	**POSTN**	periostin	protein_coding	0	0	1	1	1.000
10632	NM_006476,NR_03	**ATP5MG**	ATP synthase membrane	protein_coding	519	517	520	518	0.997
10633	NM_006477,XM_01	**RASL10A**	RAS like family 10 memb	protein_coding	1	1	2	2	0.782
10634	NM_001278730,NM	**GAS2L1**	growth arrest specific 2 l	protein_coding	0	0	1	1	1.000
10635	NM_001130862,NM	**RAD51AP1**	RAD51 associated protei	protein_coding	33	31	34	32	0.943
10636	NM_001366617,NM	**RGS14**	regulator of G protein sig	protein_coding	51	54	52	55	1.055
10637	NM_020997	**LEFTY1**	left-right determination fa	protein_coding	0	0	1	1	1.000
10640	NM_006544,XM_00	**EXOC5**	exocyst complex compor	protein_coding	23	20	24	21	0.886
10641	NM_006545,XM_00	**NPRL2**	NPR2 like, GATOR1 com	protein_coding	41	42	42	43	1.026
10642	NM_001160423,NM	**IGF2BP1**	insulin like growth factor	protein_coding	0	0	1	1	1.017
10643	NM_006547,XM_00	**IGF2BP3**	insulin like growth factor	protein_coding	0	0	1	1	0.953
10644	NM_001007225,NM	**IGF2BP2**	insulin like growth factor	protein_coding	0	0	1	1	1.000
10645	NM_001270485,NM	**CAMKK2**	calcium/calmodulin depe	protein_coding	28	27	29	28	0.962
10647	NM_006551	**SCGB1D2**	secretoglobin family 1D r	protein_coding	0	0	1	1	1.000
10648	NM_006552	**SCGB1D1**	secretoglobin family 1D r	protein_coding	0	0	1	1	1.000
10650	NM_001142405,NM	**PRELID3A**	PRELI domain containing	protein_coding	2	2	3	3	1.081
10651	NM_001006635,NM	**MTX2**	metaxin 2	protein_coding	150	146	151	147	0.974
10652	NM_001363678,NM	**YKT6**	YKT6 v-SNARE homolog	protein_coding	78	78	79	79	1.003
10653	NM_001166103,NM	**SPINT2**	serine peptidase inhibitor	protein_coding	0	0	1	1	1.000
10654	NM_001323011,NM	**PMVK**	phosphomevalonate kina	protein_coding	140	148	141	149	1.060
10655	NM_001130865,NM	**DMRT2**	doublesex and mab-3 rel	protein_coding	0	0	1	1	1.000
10656	NM_006558,XM_00	**KHDRBS3**	KH RNA binding domain	protein_coding	0	0	1	1	1.000
10657	NM_001271878,NM	**KHDRBS1**	KH RNA binding domain	protein_coding	258	257	259	258	0.994
10658	NM_001025596,NM	**CELF1**	CUGBP Elav-like family r	protein_coding	96	80	97	81	0.841
10659	NM_001025076,NM	**CELF2**	CUGBP Elav-like family r	protein_coding	47	44	48	45	0.952
10660	NM_006562	**LBX1**	ladybird homeobox 1	protein_coding	0	0	1	1	1.000
10661	NM_006563	**KLF1**	Kruppel like factor 1	protein_coding	0	0	1	1	1.000
10663	NM_006564,XM_00	**CXCR6**	C-X-C motif chemokine r	protein_coding	0	0	1	1	1.110
10664	NM_001191022,NM	**CTCF**	CCCTC-binding factor	protein_coding	72	71	73	72	0.998
10665	NM_001286474,NM	**TSBP1**	testis expressed basic pr	protein_coding	0	0	1	1	1.000
10666	NM_001303618,NM	**CD226**	CD226 molecule	protein_coding	0	0	1	1	0.986
10667	NM_001318872,NM	**FARS2**	phenylalanyl-tRNA synth	protein_coding	16	19	17	20	1.153
10668	NM_006568,XM_01	**CGRRF1**	cell growth regulator with	protein_coding	10	9	11	10	0.899
10669	NM_001166239,NM	**CGREF1**	cell growth regulator with	protein_coding	0	0	1	1	1.000
10670	NM_006570	**RRAGA**	Ras related GTP binding	protein_coding	32	32	33	33	1.000
10671	NM_006571	**DCTN6**	dynactin subunit 6	protein_coding	56	58	57	59	1.035
10672	NM_001282425,NM	**GNA13**	G protein subunit alpha	protein_coding	100	84	101	85	0.848
10673	NM_001145645,NM	**TNFSF13B**	TNF superfamily membe	protein_coding	1	1	2	2	0.933
10675	NM_001206942,NM	**CSPG5**	chondroitin sulfate prote(	protein_coding	0	0	1	1	1.014
10677	NM_006576,XM_01	**AVIL**	advillin	protein_coding	0	0	1	1	1.000
10678	NM_001319075,NM	**B3GNT2**	UDP-GlcNAc:betaGal be	protein_coding	43	40	44	41	0.920
10681	NM_001379343,NM	**GNB5**	G protein subunit beta 5	protein_coding	24	18	25	19	0.740
10682	NM_006579	**EBP**	EBP cholestenol delta-is	protein_coding	500	552	501	553	1.105
10683	NM_016941,NM_20	**DLL3**	delta like canonical Notc	protein_coding	0	0	1	1	1.000
10686	NM_001378492,NM	**CLDN16**	claudin 16	protein_coding	0	0	1	1	1.000
10687	NM_007257,XM_01	**PNMA2**	PNMA family member 2	protein_coding	0	0	1	1	1.000
10690	NM_006581,XM_01	**FUT9**	fucosyltransferase 9	protein_coding	0	0	1	1	1.000
10691	NM_001319674,NM	**GMEB1**	glucocorticoid modulator	protein_coding	7	7	8	8	0.963
10692	NM_006583	**RRH**	retinal pigment epitheliur	protein_coding	0	0	1	1	0.922
10693	NM_001193529,NM	**CCT6B**	chaperonin containing T(	protein_coding	1	1	2	2	0.926
10694	NM_001282907,NM	**CCT8**	chaperonin containing T(	protein_coding	666	627	667	628	0.942
10695	NM_001318842,NM	**CNPY3**	canopy FGF signaling re	protein_coding	96	114	97	115	1.181
10699	NM_001278585,NM	**CORIN**	corin, serine peptidase	protein_coding	0	0	1	1	0.979
10712	NM_001267608,NM	**FAM189B**	family with sequence sim	protein_coding	9	10	10	11	1.174
10713	NM_001256725,NM	**USP39**	ubiquitin specific peptida	protein_coding	154	143	155	144	0.931
10714	NM_001363597,NM	**POLD3**	DNA polymerase delta 3,	protein_coding	54	54	55	55	1.000
10715	NM_001290265,NM	**CERS1**	ceramide synthase 1	protein_coding	0	0	1	1	1.000
10716	NM_006593	**TBR1**	T-box brain transcription	protein_coding	0	0	1	1	1.123
10717	NM_001253852,NM	**AP4B1**	adaptor related protein c	protein_coding	48	46	49	47	0.952
10718	NM_001010848,NM	**NRG3**	neuregulin 3	protein_coding	0	0	1	1	1.000
10720	NM_001073,XM_01	**UGT2B11**	UDP glucuronosyltransfe	protein_coding	0	0	1	1	1.000
10721	NM_199420	**POLQ**	DNA polymerase theta	protein_coding	24	20	25	21	0.843
10723	NM_006598,XM_00	**SLC12A7**	solute carrier family 12 m	protein_coding	0	0	1	1	0.967
10724	NM_001142434,NM	**OGA**	O-GlcNAcase	protein_coding	43	42	44	43	0.971
10725	NM_001113178,NM	**NFAT5**	nuclear factor of activate	protein_coding	15	11	16	12	0.774
10726	NM_006600,XM_01	**NUDC**	nuclear distribution C, dy	protein_coding	489	525	490	526	1.074
10728	NM_001282601,NM	**PTGES3**	prostaglandin E synthas(	protein_coding	539	522	540	523	0.968
10730	NM_001253866,NM	**YME1L1**	YME1 like 1 ATPase	protein_coding	49	44	50	45	0.890
10732	NM_001301726,NM	**TCFL5**	transcription factor like 5	protein_coding	14	14	15	15	0.999
10733	NM_001190799,NM	**PLK4**	polo like kinase 4	protein_coding	36	31	37	32	0.856
10734	NM_001282716,NM	**STAG3**	stromal antigen 3	protein_coding	7	8	8	9	1.129
10735	NM_001042749,NM	**STAG2**	stromal antigen 2	protein_coding	140	124	141	125	0.887
10736	NM_016932,XM_00	**SIX2**	SIX homeobox 2	protein_coding	0	0	1	1	1.000
10737	NR_001450,NR_00	**RFPL3S**	RFPL3 antisense	lncRNA	1	0	2	1	0.481
10738	NM_001098535,NM	**RFPL3**	ret finger protein like 3	protein_coding	0	0	1	1	1.000
10739	NM_001098527,NM	**RFPL2**	ret finger protein like 2	protein_coding	0	0	1	1	1.000
10740	NR_002727	**RFPL1S**	RFPL1 antisense RNA 1	lncRNA	0	0	1	1	1.000

10741	NM_006606,XM_00!	**RBBP9**	RB binding protein 9, sei	protein_coding	16	16	17	17	0.989
10742	NM_001172732,NM	**RAI2**	retinoic acid induced 2	protein_coding	0	0	1	1	1.000
10743	NM_030665,XM_01	**RAI1**	retinoic acid induced 1	protein_coding	7	7	8	8	0.981
10744	NM_006607	**PTTG2**	pituitary tumor-transform	protein_coding	0	0	1	1	1.000
10745	NM_001323041,NM	**PHTF1**	putative homeodomain tr	protein_coding	27	24	28	25	0.896
10746	NM_001371910,NM	**MAP3K2**	mitogen-activated proteir	protein_coding	19	15	20	16	0.809
10747	NM_006610,NM_13	**MASP2**	mannan binding lectin se	protein_coding	0	0	1	1	0.714
10748	NR_028045	**KLRA1P**	killer cell lectin like recep	transcribed_pseu	2	1	3	2	0.923
10749	NM_006612,XM_00!	**KIF1C**	kinesin family member 1(	protein_coding	2	3	3	4	1.141
10750	NM_001330148,NM	**GRAP**	GRB2 related adaptor pr	protein_coding	0	0	1	1	1.000
10752	NM_001253387,NM	**CHL1**	cell adhesion molecule L	protein_coding	0	0	1	1	1.000
10753	NM_001319676,NM	**CAPN9**	calpain 9	protein_coding	0	0	1	1	1.000
10755	NM_005716,NM_20	**GIPC1**	GIPC PDZ domain conta	protein_coding	0	0	1	1	0.816
10758	NM_001164281,NM	**TRAF3IP2**	TRAF3 interacting protei	protein_coding	0	0	1	1	0.917
10761	NM_001316887,NM	**PLAC1**	placenta enriched 1	protein_coding	0	0	1	1	1.076
10762	NM_007172,NM_15	**NUP50**	nucleoporin 50	protein_coding	106	99	107	100	0.932
10763	NM_006617	**NES**	nestin	protein_coding	0	0	1	1	0.983
10765	NM_001314042,NM	**KDM5B**	lysine demethylase 5B	protein_coding	0	0	1	1	1.000
10766	NM_016272,XM_00!	**TOB2**	transducer of ERBB2, 2	protein_coding	28	26	29	27	0.933
10767	NM_001145158,NM	**HBS1L**	HBS1 like translational G	protein_coding	43	42	44	43	0.962
10768	NM_001242673,NM	**AHCYL1**	adenosylhomocysteinase	protein_coding	129	121	130	122	0.935
10769	NM_001252226,NM	**PLK2**	polo like kinase 2	protein_coding	0	0	1	1	1.000
10771	NM_001202464,NM	**ZMYND11**	zinc finger MYND-type c	protein_coding	11	12	12	13	1.076
10772	NM_001191005,NM	**SRSF10**	serine and arginine rich s	protein_coding	128	114	129	115	0.896
10773	NM_006626	**ZBTB6**	zinc finger and BTB dom	protein_coding	12	11	13	12	0.927
10775	NM_006627,NR_02	**POP4**	POP4 homolog, ribonucl	protein_coding	134	133	135	134	0.987
10776	NM_001306191,NM	**ARPP19**	cAMP regulated phosphc	protein_coding	169	145	170	146	0.859
10777	NM_001025068,NM	**ARPP21**	cAMP regulated phosphc	protein_coding	0	0	1	1	1.000
10778	NR_024565,NR_02	**ZNF271P**	zinc finger protein 271, p	transcribed_pseu	22	20	23	21	0.903
10780	NM_001144824,NM	**ZNF234**	zinc finger protein 234	protein_coding	0	0	1	1	1.022
10781	NM_001271314,NM	**ZNF266**	zinc finger protein 266	protein_coding	30	27	31	28	0.920
10782	NM_001278734,NM	**ZNF274**	zinc finger protein 274	protein_coding	10	11	11	12	1.073
10783	NM_001145001,NM	**NEK6**	NIMA related kinase 6	protein_coding	0	0	1	1	0.938
10785	NM_001260474,NM	**WDR4**	WD repeat domain 4	protein_coding	56	55	57	56	0.983
10786	NM_001098486,NM	**SLC17A3**	solute carrier family 17 n	protein_coding	0	0	1	1	1.000
10787	NM_013436,NM_20	**NCKAP1**	NCK associated protein 1	protein_coding	0	0	1	1	0.965
10788	NM_001285460,NM	**IQGAP2**	IQ motif containing GTPa	protein_coding	120	111	121	112	0.924
10789	gene-NUDCP2	**NUDCP2**	nuclear distribution C ps	pseudogene	0	0	1	1	1.231
10790	gene-NUDCP1	**NUDCP1**	nuclear distribution C ps	pseudogene	0	0	1	1	1.029
10791	NM_006634	**VAMP5**	vesicle associated memb	protein_coding	26	31	27	32	1.181
10793	NM_021148,NR_00	**ZNF273**	zinc finger protein 273	protein_coding	12	11	13	12	0.944
10794	NM_001330622,NM	**ZNF460**	zinc finger protein 460	protein_coding	5	4	6	5	0.951
10795	NM_001165881,NM	**ZNF268**	zinc finger protein 268	protein_coding	0	0	1	1	1.000
10797	NM_006636,NR_02	**MTHFD2**	methylenetetrahydrofolat	protein_coding	262	233	263	234	0.892
10798	NM_006637	**OR5I1**	olfactory receptor family	protein_coding	0	0	1	1	1.000
10799	NM_001286132,NM	**RPP40**	ribonuclease P/MRP sub	protein_coding	30	28	31	29	0.953
10800	NM_001282186,NM	**CYSLTR1**	cysteinyl leukotriene rece	protein_coding	0	0	1	1	1.000
10801	NM_001113491,NM	**SEPTIN9**	septin 9	protein_coding	124	110	125	111	0.888
10802	NM_001252231,NM	**SEC24A**	SEC24 homolog A, COP	protein_coding	29	24	30	25	0.826
10803	NM_001256369,NM	**CCR9**	C-C motif chemokine rec	protein_coding	0	0	1	1	1.000
10804	NM_001110219,NM	**GJB6**	gap junction protein beta	protein_coding	0	0	1	1	1.000
10806	NM_001350246,NM	**SDCCAG8**	SHH signaling and ciliog	protein_coding	7	5	8	6	0.796
10807	NM_001039707,NM	**ENTR1**	endosome associated tra	protein_coding	28	27	29	28	0.960
10808	NM_001286503,NM	**HSPH1**	heat shock protein family	protein_coding	256	235	257	236	0.918
10809	NM_006645	**STARD10**	StAR related lipid transfe	protein_coding	3	3	4	4	0.906
10810	NM_001291965,NM	**WASF3**	WASP family member 3	protein_coding	0	0	1	1	1.000
10811	NM_001256067,NM	**NOXA1**	NADPH oxidase activator	protein_coding	0	0	1	1	1.000
10813	NM_001166221,NM	**UTP14A**	UTP14A small subunit pi	protein_coding	64	60	65	61	0.941
10814	NM_001008220,NM	**CPLX2**	complexin 2	protein_coding	0	0	1	1	1.000
10815	NM_006651,XM_01	**CPLX1**	complexin 1	protein_coding	0	0	1	1	1.000
10816	NM_006652	**SPINT3**	serine peptidase inhibitor	protein_coding	0	0	1	1	1.000
10817	NM_006653,XM_01	**FRS3**	fibroblast growth factor r	protein_coding	5	6	6	7	1.266
10818	NM_001042555,NM	**FRS2**	fibroblast growth factor r	protein_coding	3	3	4	4	0.883
10819	NR_045002	**OR7E14P**	olfactory receptor family	transcribed_pseu	0	0	1	1	1.000
10821	NR_044999	**OR7E12P**	olfactory receptor family	transcribed_pseu	0	0	1	1	0.998
10824	NR_125391	**DIAPH2-AS1**	DIAPH2 antisense RNA 1	lncRNA	0	0	1	1	1.000
10825	NM_001367860,NM	**NEU3**	neuraminidase 3	protein_coding	51	46	52	47	0.904
10826	NM_032385,XM_00	**FAXDC2**	fatty acid hydroxylase dc	protein_coding	0	0	1	1	1.023
10827	NM_001317993,NM	**FAM114A2**	family with sequence sim	protein_coding	17	14	18	15	0.841
10838	NM_001080485,NM	**ZNF275**	zinc finger protein 275	protein_coding	10	10	11	11	0.977
10840	NM_001270364,NM	**ALDH1L1**	aldehyde dehydrogenase	protein_coding	18	18	19	19	0.990
10841	NM_001320412,NM	**FTCD**	formimidoyltransferase c	protein_coding	0	0	1	1	0.973
10842	NM_001145123,NM	**PPP1R17**	protein phosphatase 1 re	protein_coding	0	0	1	1	1.000
10844	NM_001256617,NM	**TUBGCP2**	tubulin gamma complex	protein_coding	29	32	30	33	1.096
10845	NM_006660,NR_13	**CLPX**	caseinolytic mitochondria	protein_coding	47	44	48	45	0.943
10846	NM_001130690,NM	**PDE10A**	phosphodiesterase 10A	protein_coding	0	0	1	1	1.000
10847	NM_006662	**SRCAP**	Snf2 related CREBBP ac	protein_coding	56	53	57	54	0.948
10848	NM_001142502,NM	**PPP1R13L**	protein phosphatase 1 re	protein_coding	2	2	3	3	0.895
10849	NM_001297590,NM	**POLR1G**	RNA polymerase I subur	protein_coding	74	66	75	67	0.889
10850	NM_006664	**CCL27**	C-C motif chemokine liga	protein_coding	0	0	1	1	1.000
10855	NM_001098540,NM	**HPSE**	heparanase	protein_coding	3	3	4	4	0.924
10856	NM_001321190,NM	**RUVBL2**	RuvB like AAA ATPase 2	protein_coding	241	300	242	301	1.241
10857	NM_001282621,NM	**PGRMC1**	progesterone receptor m	protein_coding	86	85	87	86	0.991
10858	NM_006668,XM_00!	**CYP46A1**	cytochrome P450 family	protein_coding	0	0	1	1	1.000
10859	NM_001081637,NM	**LILRB1**	leukocyte immunoglobul	protein_coding	19	19	20	20	0.973
10861	NM_022042,NM_13	**SLC26A1**	solute carrier family 26 n	protein_coding	0	0	1	1	1.000
10863	NM_001304351,NM	**ADAM28**	ADAM metallopeptidase	protein_coding	0	0	1	1	0.872
10864	NM_006672,NM_15	**SLC22A7**	solute carrier family 22 n	protein_coding	0	0	1	1	1.000
10865	NM_001319085,NM	**ARID5A**	AT-rich interaction doma	protein_coding	15	15	16	16	1.030
10866	NR_040662	**HCP5**	HLA complex P5	lncRNA	158	130	159	131	0.823
10867	NM_001168320,NM	**TSPAN9**	tetraspanin 9	protein_coding	0	0	1	1	0.979
10868	NM_001008563,NM	**USP20**	ubiquitin specific peptida	protein_coding	32	31	33	32	0.980
10869	NM_001199160,NM	**USP19**	ubiquitin specific peptida	protein_coding	40	40	41	41	1.011
10870	NM_001007469,NM	**HCST**	hematopoietic cell signal	protein_coding	300	323	301	324	1.075
10871	NM_006678,XM_01	**CD300C**	CD300c molecule	protein_coding	30	31	31	32	1.052
10873	NM_001014811,NM	**ME3**	malic enzyme 3	protein_coding	0	0	1	1	0.982
10874	NM_001292045,NM	**NMU**	neuromedin U	protein_coding	0	0	1	1	1.000
10875	NM_006682	**FGL2**	fibrinogen like 2	protein_coding	2	2	3	3	1.015
10876	NM_006683,XM_01	**EDDM3A**	epididymal protein 3A	protein_coding	0	0	1	1	1.000
10877	NM_001201550,NM	**CFHR4**	complement factor H rela	protein_coding	0	0	1	1	1.000
10878	NM_001166624,NM	**CFHR3**	complement factor H rela	protein_coding	0	0	1	1	0.822
10879	NM_006685	**SMR3B**	submaxillary gland andrc	protein_coding	0	0	1	1	1.000
10880	NM_006686	**ACTL7B**	actin like 7B	protein_coding	0	0	1	1	0.845
10881	NM_006687	**ACTL7A**	actin like 7A	protein_coding	0	0	1	1	1.000
10882	NM_006688	**C1QL1**	complement C1q like 1	protein_coding	0	0	1	1	1.000
10884	NM_016640	**MRPS30**	mitochondrial ribosomal	protein_coding	105	99	106	100	0.947
10885	NM_006784	**WDR3**	WD repeat domain 3	protein_coding	54	48	55	49	0.901
10886	NM_001144756,NM	**NPFFR2**	neuropeptide FF recepto	protein_coding	0	0	1	1	1.000
10887	NM_138964	**PROKR1**	prokineticin receptor 1	protein_coding	0	0	1	1	0.918
10888	NM_001330345,NM	**GPR83**	G protein-coupled recept	protein_coding	1	1	2	2	0.856
10890	NM_016131,XM_02	**RAB10**	RAB10, member RAS on	protein_coding	98	86	99	87	0.884
10891	NM_001330751,NM	**PPARGC1A**	PPARG coactivator 1 alp	protein_coding	0	0	1	1	1.000
10892	NM_006785,NM_17	**MALT1**	MALT1 paracaspase	protein_coding	9	9	10	10	0.954
10893	NM_006690,XM_01	**MMP24**	matrix metallopeptidase	protein_coding	0	0	1	1	1.000
10894	NM_006691	**LYVE1**	lymphatic vessel endothe	protein_coding	0	0	1	1	1.000
10895	NR_026769	**PPBPP2**	pro-platelet basic protein	transcribed_pseu	0	0	1	1	1.000
10897	NM_001300861,NM	**YIF1A**	Yip1 interacting factor hc	protein_coding	168	174	169	175	1.036
10898	NM_001081559,NM	**CPSF4**	cleavage and polyadenyl	protein_coding	112	106	113	107	0.949
10899	NM_006694	**JTB**	jumping translocation bre	protein_coding	297	304	298	305	1.023
10900	NM_001144825,NM	**RUNDC3A**	RUN domain containing	protein_coding	0	0	1	1	1.000
10901	NM_001282987,NM	**DHRS4**	dehydrogenase/reductas	protein_coding	13	17	14	18	1.284
10902	NM_001164326,NM	**BRD8**	bromodomain containing	protein_coding	50	54	51	55	1.080
10903	NM_001145862,NM	**MTMR11**	myotubularin related pro	protein_coding	0	0	1	1	1.058
10904	NM_001167820,NM	**BLCAP**	BLCAP apoptosis inducir	protein_coding	118	131	119	132	1.112
10905	NM_006699,XM_00	**MAN1A2**	mannosidase alpha class	protein_coding	18	14	19	15	0.802
10906	NM_001143906,NM	**TRAFD1**	TRAF-type zinc finger dc	protein_coding	56	58	57	59	1.033

10907	NM_001303471,NM	**TXNL4A**	thioredoxin like 4A	protein_coding	65	64	66	65	0.980
10908	NM_001166111,NM	**PNPLA6**	patatin like phospholipas	protein_coding	43	44	44	45	1.009
10910	NM_001130912,NM	**SUGT1**	SGT1 homolog, MIS12 k	protein_coding	15	14	16	15	0.930
10911	NM_006786,NM_02	**UTS2**	urotensin 2	protein_coding	0	0	1	1	1.000
10912	NM_006705,XM_01	**GADD45G**	growth arrest and DNA d	protein_coding	2	3	3	4	1.095
10913	NM_022336,XM_00	**EDAR**	ectodysplasin A receptor	protein_coding	0	0	1	1	1.000
10914	NM_001252006,NM	**PAPOLA**	poly(A) polymerase alph	protein_coding	159	138	160	139	0.872
10915	NM_001040006,NM	**TCERG1**	transcription elongation r	protein_coding	86	74	87	75	0.862
10916	NM_014599,NM_17	**MAGED2**	MAGE family member D2	protein_coding	31	35	32	36	1.149
10917	NM_197975	**BTNL3**	butyrophilin like 3	protein_coding	0	0	1	1	1.000
10919	NM_001289413,NM	**EHMT2**	euchromatic histone lysi	protein_coding	52	54	53	55	1.044
10920	NM_006710,NM_19	**COPS8**	COP9 signalosome subu	protein_coding	81	74	82	75	0.925
10921	NM_001286625,NM	**RNPS1**	RNA binding protein with	protein_coding	406	406	407	407	1.000
10922	NM_001258461,NM	**FASTK**	Fas activated serine/thre	protein_coding	36	39	37	40	1.082
10923	NM_006713,XM_01	**SUB1**	SUB1 regulator of transc	protein_coding	203	191	204	192	0.944
10924	NM_001286138,NM	**SMPDL3A**	sphingomyelin phosphod	protein_coding	0	0	1	1	1.000
10926	NM_001318060,NM	**DBF4**	DBF4 zinc finger	protein_coding	96	88	97	89	0.913
10927	NM_006717	**SPIN1**	spindlin 1	protein_coding	23	19	24	20	0.853
10928	NM_006788	**RALBP1**	ralA binding protein 1	protein_coding	0	0	1	1	1.000
10929	NM_032102,NR_10	**SRSF8**	serine and arginine rich s	protein_coding	25	27	26	28	1.099
10930	NM_006789	**APOBEC2**	apolipoprotein B mRNA e	protein_coding	0	0	1	1	1.000
10933	NM_001265603,NM	**MORF4L1**	mortality factor 4 like 1	protein_coding	372	346	373	347	0.930
10934	gene-MORF4	**MORF4**	mortality factor 4 (pseud	pseudogene	0	0	1	1	1.091
10935	NM_001302272,NM	**PRDX3**	peroxiredoxin 3	protein_coding	385	365	386	366	0.948
10936	NM_006794	**GPR75**	G protein-coupled recept	protein_coding	1	2	2	3	1.309
10938	NM_001282444,NM	**EHD1**	EH domain containing 1	protein_coding	64	66	65	67	1.038
10939	NM_006796,XM_01	**AFG3L2**	AFG3 like matrix AAA pe	protein_coding	68	69	69	70	1.005
10940	NM_001145860,NM	**POP1**	POP1 homolog, ribonucl	protein_coding	45	42	46	43	0.934
10941	NM_001252274,NM	**UGT2A1**	UDP glucuronosyltransfe	protein_coding	0	0	1	1	1.000
10942	NM_001270452,NM	**PRSS21**	serine protease 21	protein_coding	0	0	1	1	1.000
10943	NM_001193270,NM	**MSL3**	MSL complex subunit 3	protein_coding	15	13	16	14	0.870
10944	NM_014267,XM_01	**C11orf58**	chromosome 11 open rea	protein_coding	140	132	141	133	0.942
10945	NM_006801	**KDELR1**	KDEL endoplasmic reticu	protein_coding	110	110	111	111	0.998
10946	NM_001320830,NM	**SF3A3**	splicing factor 3a subuni	protein_coding	217	213	218	214	0.984
10947	NM_001134296,NM	**AP3M2**	adaptor related protein c	protein_coding	35	28	36	29	0.813
10948	NM_001165937,NM	**STARD3**	StAR related lipid transfe	protein_coding	45	43	46	44	0.958
10949	NM_006805	**HNRNPA0**	heterogeneous nuclear r	protein_coding	105	114	106	115	1.083
10950	NM_001130914,NM	**BTG3**	BTG anti-proliferation fac	protein_coding	57	56	58	57	0.984
10951	NM_001127228,NM	**CBX1**	chromobox 1	protein_coding	138	135	139	136	0.981
10952	NM_006808	**SEC61B**	SEC61 translocon subur	protein_coding	242	265	243	266	1.093
10953	NM_006809,XM_00	**TOMM34**	translocase of outer mito	protein_coding	94	100	95	101	1.064
10954	NM_006810,NR_02	**PDIA5**	protein disulfide isomera	protein_coding	11	11	12	12	1.005
10955	NM_006811,NM_19	**SERINC3**	serine incorporator 3	protein_coding	73	71	74	72	0.981
10956	NM_001017956,NM	**OS9**	OS9 endoplasmic reticul	protein_coding	88	92	89	93	1.048
10957	NM_006813,XR_00	**PNRC1**	proline rich nuclear recep	protein_coding	10	11	11	12	1.014
10959	NM_001321445,NM	**TMED2**	transmembrane p24 traff	protein_coding	163	152	164	153	0.934
10960	NM_006816	**LMAN2**	lectin, mannose binding	protein_coding	334	362	335	363	1.082
10961	NM_001034025,NM	**ERP29**	endoplasmic reticulum p	protein_coding	179	186	180	187	1.043
10962	NM_006818	**MLLT11**	MLLT11 transcription fac	protein_coding	1	2	2	3	1.144
10963	NM_001282652,NM	**STIP1**	stress induced phosphop	protein_coding	493	482	494	483	0.977
10964	NM_001375646,NM	**IFI44L**	interferon induced protei	protein_coding	21	15	22	16	0.730
10965	NM_001364177,NM	**ACOT2**	acyl-CoA thioesterase 2	protein_coding	12	13	13	14	1.014
10966	NM_006822,XM_00	**RAB40B**	RAB40B, member RAS o	protein_coding	0	0	1	1	1.000
10969	NM_001159936,NM	**EBNA1BP2**	EBNA1 binding protein 2	protein_coding	336	345	337	346	1.025
10970	NM_006825	**CKAP4**	cytoskeleton associated	protein_coding	0	0	1	1	1.000
10971	NM_006826	**YWHAQ**	tyrosine 3-monooxygena	protein_coding	366	354	367	355	0.967
10972	NM_006827	**TMED10**	transmembrane p24 traff	protein_coding	113	106	114	107	0.941
10973	NM_001284271,NM	**ASCC3**	activating signal cointegr	protein_coding	34	28	35	29	0.826
10974	NM_006829	**ADIRF**	adipogenesis regulatory	protein_coding	0	0	1	1	1.000
10975	NM_006830	**UQCR11**	ubiquinol-cytochrome c r	protein_coding	123	139	124	140	1.131
10978	NM_001142597,NM	**CLP1**	cleavage factor polyribor	protein_coding	37	38	38	39	1.014
10979	NM_001134999,NM	**FERMT2**	fermitin family member 2	protein_coding	0	0	1	1	1.059
10980	NM_006833	**COPS6**	COP9 signalosome subu	protein_coding	146	163	147	164	1.116
10981	NM_006834	**RAB32**	RAB32, member RAS on	protein_coding	0	0	1	1	1.000
10982	NM_001143826,NM	**MAPRE2**	microtubule associated p	protein_coding	32	29	33	30	0.930
10983	NM_001348132,NM	**CCNI**	cyclin I	protein_coding	119	114	120	115	0.954
10984	NR_002728	**KCNQ1OT1**	KCNQ1 opposite strand/	lncRNA	0	0	1	1	0.981
10985	NM_006836	**GCN1**	GCN1 activator of EIF2A	protein_coding	56	53	57	54	0.937
10987	NM_006837	**COPS5**	COP9 signalosome subu	protein_coding	175	177	176	178	1.016
10988	NM_001317182,NM	**METAP2**	methionyl aminopeptidas	protein_coding	136	124	137	125	0.914
10989	NM_001100169,NM	**IMMT**	inner membrane mitocho	protein_coding	111	103	112	104	0.930
10990	NM_001081442,NM	**LILRB5**	leukocyte immunoglobul	protein_coding	0	0	1	1	1.000
10991	NM_006841,XM_00	**SLC38A3**	solute carrier family 38 m	protein_coding	0	0	1	1	1.000
10992	NM_006842,XM_00	**SF3B2**	splicing factor 3b subuni	protein_coding	290	287	291	288	0.989
10993	NM_006843	**SDS**	serine dehydratase	protein_coding	0	0	1	1	1.000
10994	NM_006844,XM_00	**ILVBL**	ilvB acetolactate synthas	protein_coding	19	17	20	18	0.931
10998	NM_001321196,NM	**SLC27A5**	solute carrier family 27 m	protein_coding	10	12	11	13	1.154
10999	NM_005094,XM_01	**SLC27A4**	solute carrier family 27 m	protein_coding	22	22	23	23	1.012
11000	NM_001317929,NM	**SLC27A3**	solute carrier family 27 m	protein_coding	26	27	27	28	1.058
11001	NM_001159629,NM	**SLC27A2**	solute carrier family 27 m	protein_coding	88	78	89	79	0.892
11004	NM_001297655,NM	**KIF2C**	kinesin family member 2	protein_coding	152	150	153	151	0.986
11005	NM_001127698,NM	**SPINK5**	serine peptidase inhibito	protein_coding	0	1	1	2	1.202
11006	NM_001278426,NM	**LILRB4**	leukocyte immunoglobul	protein_coding	0	0	1	1	0.965
11007	NM_006848	**CCDC85B**	coiled-coil domain contai	protein_coding	103	131	104	132	1.275
11009	NM_001185156,NM	**IL24**	interleukin 24	protein_coding	0	0	1	1	1.000
11010	NM_006851,XM_01	**GLIPR1**	GLI pathogenesis relatec	protein_coding	37	31	38	32	0.855
11011	NM_001284333,NM	**TLK2**	tousled like kinase 2	protein_coding	38	33	39	34	0.882
11012	NM_001136032,NM	**KLK11**	kallikrein related peptida	protein_coding	0	0	1	1	1.000
11013	NM_021992	**TMSB15A**	thymosin beta 15a	protein_coding	0	0	1	1	1.000
11014	NM_001100603,NM	**KDELR2**	KDEL endoplasmic reticu	protein_coding	78	69	79	70	0.889
11015	NM_006855,NM_01	**KDELR3**	KDEL endoplasmic reticu	protein_coding	0	0	1	1	1.000
11016	NM_001130060,NM	**ATF7**	activating transcription fa	protein_coding	11	11	12	12	1.007
11017	NM_006857,NR_03	**SNRNP27**	small nuclear ribonucleo	protein_coding	114	108	115	109	0.948
11018	NM_006858,NR_10	**TMED1**	transmembrane p24 traff	protein_coding	23	28	24	29	1.198
11019	NM_001278590,NM	**LIAS**	lipoic acid synthetase	protein_coding	20	19	21	20	0.968
11020	NM_001177701,NM	**IFT27**	intraflagellar transport 27	protein_coding	9	9	10	10	0.971
11021	NM_001167606,NM	**RAB35**	RAB35, member RAS on	protein_coding	67	66	68	67	0.987
11022	NM_001083963,NM	**TDRKH**	tudor and KH domain co	protein_coding	15	14	16	15	0.908
11023	NM_001112704,NM	**VAX1**	ventral anterior homeob	protein_coding	0	0	1	1	1.000
11024	NM_001278318,NM	**LILRA1**	leukocyte immunoglobul	protein_coding	0	0	1	1	1.000
11025	NM_001081450,NM	**LILRB3**	leukocyte immunoglobul	protein_coding	0	0	1	1	0.862
11027	NM_001130917,NM	**LILRA2**	leukocyte immunoglobul	protein_coding	1	1	2	2	0.850
11030	NM_001008710,NM	**RBPMS**	RNA binding protein, mR	protein_coding	0	0	1	1	1.000
11031	NM_006868,XM_01	**RAB31**	RAB31, member RAS on	protein_coding	0	0	1	1	1.000
11033	NM_001284308,NM	**ADAP1**	ArfGAP with dual PH dor	protein_coding	59	59	60	60	0.997
11034	NM_001011546,NM	**DSTN**	destrin, actin depolymeri	protein_coding	17	16	18	17	0.972
11035	NM_006871	**RIPK3**	receptor interacting serin	protein_coding	0	0	1	1	1.000
11036	NM_001193487,NM	**GTF2A1L**	general transcription fact	protein_coding	0	0	1	1	1.000
11037	NM_001198595,NM	**STON1**	stonin 1	protein_coding	11	8	12	9	0.786
11039	NR_024054,NR_02	**GUSBP14**	GUSB pseudogene 14	transcribed_pseu	11	10	12	11	0.911
11040	NM_006875	**PIM2**	Pim-2 proto-oncogene, s	protein_coding	42	61	43	62	1.437
11041	NM_006876	**B4GAT1**	beta-1,4-glucuronyltrans	protein_coding	8	6	9	7	0.821
11042	NR_034021	**GUSBP15**	GUSB pseudogene 15	transcribed_pseu	6	6	7	7	0.987
11043	NM_001382751,NM	**MID2**	midline 2	protein_coding	0	0	1	1	1.000
11044	NM_001171805,NM	**TENT4A**	terminal nucleotidyltrans	protein_coding	24	21	25	22	0.871
11045	NM_001281443,NM	**UPK1A**	uroplakin 1A	protein_coding	0	0	1	1	1.000
11046	NM_001286990,NM	**SLC35D2**	solute carrier family 35 m	protein_coding	0	0	1	1	1.236
11047	NM_001281437,NM	**ADRM1**	adhesion regulating mole	protein_coding	363	376	364	377	1.036
11049	gene-NUS1P3	**NUS1P3**	NUS1 pseudogene 3	pseudogene	0	0	1	1	1.000
11051	NM_007006	**NUDT21**	nudix hydrolase 21	protein_coding	146	129	147	130	0.887
11052	NM_001300947,NM	**CPSF6**	cleavage and polyadeny	protein_coding	62	55	63	56	0.892
11054	NM_007346	**OGFR**	opioid growth factor rece	protein_coding	154	164	155	165	1.064
11055	NM_001159878,NM	**ZPBP**	zona pellucida binding p	protein_coding	0	0	1	1	1.000
11056	NM_001291476,NM	**DDX52**	DExD-box helicase 52	protein_coding	35	30	36	31	0.855
11057	NM_007011,NM_15	**ABHD2**	abhydrolase domain con	protein_coding	30	26	31	27	0.866
11059	NM_007013,XM_00	**WWP1**	WW domain containing E	protein_coding	19	18	20	19	0.930

11060	NM_001270453,NM	**WWP2**	WW domain containing E	protein_coding	18	18	19	19	1.016
11061	NM_001011705,NM	**CNMD**	chondromodulin	protein_coding	0	0	1	1	1.000
11062	NM_001270419,NM	**DUS4L**	dihydrouridine synthase	protein_coding	15	15	16	16	0.962
11063	NM_001308165,NM	**SOX30**	SRY-box transcription fa	protein_coding	0	0	1	1	1.031
11064	NM_001330762,NM	**CNTRL**	centriolin	protein_coding	54	46	55	47	0.847
11065	NM_001281741,NM	**UBE2C**	ubiquitin conjugating enz	protein_coding	630	607	631	608	0.964
11066	NM_022717,NM_18	**SNRNP35**	small nuclear ribonucleo	protein_coding	15	18	16	19	1.177
11067	NM_007021	**DEPP1**	DEPP1 autophagy regula	protein_coding	0	0	1	1	1.000
11068	NM_001291284,NM	**CYB561D2**	cytochrome b561 family	protein_coding	27	36	28	37	1.297
11069	NM_001100397,NM	**RAPGEF4**	Rap guanine nucleotide	protein_coding	0	0	1	1	1.095
11070	NM_007024	**TMEM115**	transmembrane protein 1	protein_coding	22	20	23	21	0.933
11072	NM_007026,XM_00	**DUSP14**	dual specificity phosphat	protein_coding	24	24	25	25	0.998
11073	NM_001363889,NM	**TOPBP1**	DNA topoisomerase II bin	protein_coding	154	133	155	134	0.865
11074	NM_007028,NR_13	**TRIM31**	tripartite motif containing	protein_coding	0	0	1	1	1.000
11075	NM_001199214,NM	**STMN2**	stathmin 2	protein_coding	0	0	1	1	1.000
11076	NM_007030,XM_00	**TPPP**	tubulin polymerization pr	protein_coding	0	0	1	1	1.000
11077	NM_007031,XM_01	**HSF2BP**	heat shock transcription	protein_coding	1	1	2	2	1.216
11078	NM_001039141,NM	**TRIOBP**	TRIO and F-actin binding	protein_coding	12	13	13	14	1.112
11079	NM_007033	**RER1**	retention in endoplasmic	protein_coding	103	110	104	111	1.066
11080	NM_001317099,NM	**DNAJB4**	DnaJ heat shock protein	protein_coding	7	6	8	7	0.948
11081	NM_007035	**KERA**	keratocan	protein_coding	0	0	1	1	1.000
11082	NM_001135604,NM	**ESM1**	endothelial cell specific r	protein_coding	1	2	2	3	1.324
11083	NM_001193369,NM	**DIDO1**	death inducer-obliterator	protein_coding	64	57	65	58	0.897
11085	NM_021794	**ADAM30**	ADAM metallopeptidase	protein_coding	0	0	1	1	1.000
11086	NM_001130703,NM	**ADAM29**	ADAM metallopeptidase	protein_coding	0	0	1	1	1.000
11091	NM_017588,NM_05	**WDR5**	WD repeat domain 5	protein_coding	70	65	71	66	0.930
11092	NM_001316897,NM	**SPACA9**	sperm acrosome associa	protein_coding	1	1	2	2	1.190
11093	NM_139025,NM_13	**ADAMTS13**	ADAM metallopeptidase	protein_coding	0	0	1	1	1.031
11094	NM_001135775,NM	**CACFD1**	calcium channel flower d	protein_coding	0	0	1	1	1.023
11095	NM_007037,XM_01	**ADAMTS8**	ADAM metallopeptidase	protein_coding	0	0	1	1	1.000
11096	NM_007038,XM_02	**ADAMTS5**	ADAM metallopeptidase	protein_coding	0	0	1	1	1.000
11097	NM_001370443,NM	**NUP42**	nucleoporin 42	protein_coding	27	28	28	29	1.041
11098	NM_001293179,NM	**PRSS23**	serine protease 23	protein_coding	0	0	1	1	1.000
11099	NM_007039,XM_00	**PTPN21**	protein tyrosine phospha	protein_coding	0	0	1	1	1.000
11100	NM_001301016,NM	**HNRNPUL1**	heterogeneous nuclear r	protein_coding	367	363	368	364	0.991
11101	NM_001001976,NM	**ATE1**	arginyltransferase 1	protein_coding	22	16	23	17	0.769
11102	NM_001098783,NM	**RPP14**	ribonuclease P/MRP sub	protein_coding	24	25	25	26	1.039
11103	NM_007043	**KRR1**	KRR1 small subunit proc	protein_coding	23	21	24	22	0.921
11104	NM_001204076,NM	**KATNA1**	katanin catalytic subunit	protein_coding	30	27	31	28	0.898
11105	NM_001098173,XM	**PRDM7**	PR/SET domain 7	protein_coding	0	0	1	1	1.000
11107	NM_001300823,NM	**PRDM5**	PR/SET domain 5	protein_coding	0	0	1	1	0.968
11108	NM_012406,XM_00	**PRDM4**	PR/SET domain 4	protein_coding	26	26	27	27	0.980
11112	NM_152740,XM_01	**HIBADH**	3-hydroxyisobutyrate deh	protein_coding	9	10	10	11	1.133
11113	NM_001206999,NM	**CIT**	citron rho-interacting ser	protein_coding	15	13	16	14	0.843
11116	NM_001278690,NM	**CEP43**	centrosomal protein 43	protein_coding	7	6	8	7	0.936
11117	NM_007046	**EMILIN1**	elastin microfibril interfac	protein_coding	0	0	1	1	0.935
11118	NM_001197246,NM	**BTN3A2**	butyrophilin subfamily 3	protein_coding	46	40	47	41	0.869
11119	NM_001145008,NM	**BTN3A1**	butyrophilin subfamily 3	protein_coding	22	18	23	19	0.844
11120	NM_001197233,NM	**BTN2A1**	butyrophilin subfamily 2	protein_coding	35	32	36	33	0.907
11122	NM_007050,NM_13	**PTPRT**	protein tyrosine phospha	protein_coding	0	0	1	1	1.000
11123	NM_001251977,NM	**RCAN3**	RCAN family member 3	protein_coding	31	28	32	29	0.903
11124	NM_007051,XM_01	**FAF1**	Fas associated factor 1	protein_coding	43	33	44	34	0.771
11126	NM_007053,NR_10	**CD160**	CD160 molecule	protein_coding	34	41	35	42	1.204
11127	NM_001300791,NM	**KIF3A**	kinesin family member 3	protein_coding	15	13	16	14	0.850
11128	NM_007055	**POLR3A**	RNA polymerase III subu	protein_coding	30	27	31	28	0.915
11129	NM_001278439,NM	**CLASRP**	CLK4 associating serine/	protein_coding	28	28	29	29	0.974
11130	NM_001005413,NM	**ZWINT**	ZW10 interacting kinetoc	protein_coding	171	158	172	159	0.928
11131	NM_007058,XM_00	**CAPN11**	calpain 11	protein_coding	0	0	1	1	1.000
11132	NM_023083,NM_02	**CAPN10**	calpain 10	protein_coding	30	26	31	27	0.870
11133	NM_001291296,NM	**KPTN**	kaptin, actin binding prot	protein_coding	9	11	10	12	1.180
11135	NM_152243	**CDC42EP1**	CDC42 effector protein 1	protein_coding	0	0	1	1	0.957
11136	NM_001126335,NM	**SLC7A9**	solute carrier family 7 me	protein_coding	0	0	1	1	1.000
11137	NM_001317962,NM	**PWP1**	PWP1 homolog, endonu	protein_coding	73	70	74	71	0.953
11138	NM_001102426,NM	**TBC1D8**	TBC1 domain family mer	protein_coding	0	0	1	1	0.983
11140	NM_007065,XM_01	**CDC37**	cell division cycle 37, HS	protein_coding	559	590	560	591	1.055
11141	NM_014271,XM_01	**IL1RAPL1**	interleukin 1 receptor acc	protein_coding	0	0	1	1	1.000
11142	NM_001281444,NM	**PKIG**	cAMP-dependent protein	protein_coding	0	0	1	1	0.977
11143	NM_001199155,NM	**KAT7**	lysine acetyltransferase	protein_coding	13	12	14	13	0.892
11144	NM_001278208,NM	**DMC1**	DNA meiotic recombinas	protein_coding	19	20	20	21	1.070
11145	NM_001128203,NM	**PLAAT3**	phospholipase A and acy	protein_coding	19	17	20	18	0.908
11146	NM_001319683,NM	**GLMN**	glomulin, FKBP associat	protein_coding	28	24	29	25	0.840
11147	NR_027404,NR_16	**HHLA3**	HERV-H LTR-associating	lncRNA	7	10	8	11	1.287
11148	NM_001282556,NM	**HHLA2**	HERV-H LTR-associating	protein_coding	69	56	70	57	0.809
11149	NM_001199563,NM	**BVES**	blood vessel epicardial s	protein_coding	1	1	2	2	0.967
11151	NM_001193333,NM	**CORO1A**	coronin 1A	protein_coding	2,491	2,470	2,492	2,471	0.992
11152	NM_001029896,NM	**WDR45**	WD repeat domain 45	protein_coding	9	10	10	11	1.078
11153	NM_007076	**FICD**	FIC domain protein aden	protein_coding	4	4	5	5	1.055
11154	NM_001128126,NM	**AP4S1**	adaptor related protein c	protein_coding	9	9	10	10	1.000
11155	NM_001080114,NM	**LDB3**	LIM domain binding 3	protein_coding	0	0	1	1	1.019
11156	NM_007079,NM_03	**PTP4A3**	protein tyrosine phospha	protein_coding	2	3	3	4	1.209
11157	NM_007080,XM_01	**LSM6**	LSM6 homolog, U6 smal	protein_coding	65	88	66	89	1.337
11158	NM_001003789,NM	**RABL2B**	RAB, member of RAS on	protein_coding	12	13	13	14	1.061
11159	NM_001306158,NM	**RABL2A**	RAB, member of RAS on	protein_coding	5	5	6	6	1.031
11160	NM_001003790,NM	**ERLIN2**	ER lipid raft associated 2	protein_coding	18	18	19	19	0.973
11161	NM_007176	**ERG28**	ergosterol biosynthesis 2	protein_coding	40	42	41	43	1.030
11162	NM_007083,NM_19	**NUDT6**	nudix hydrolase 6	protein_coding	14	12	15	13	0.886
11163	NM_001301022,NM	**NUDT4**	nudix hydrolase 4	protein_coding	21	18	22	19	0.895
11164	NM_001321647,NM	**NUDT5**	nudix hydrolase 5	protein_coding	30	26	31	27	0.879
11165	NM_006703	**NUDT3**	nudix hydrolase 3	protein_coding	29	28	30	29	0.957
11166	NM_007084	**SOX21**	SRY-box transcription fa	protein_coding	0	0	1	1	1.000
11167	NM_007085,XM_02	**FSTL1**	follistatin like 1	protein_coding	0	0	1	1	0.974
11168	NM_001128217,NM	**PSIP1**	PC4 and SFRS1 interact	protein_coding	85	72	86	73	0.852
11169	NM_001008396,NM	**WDHD1**	WD repeat and HMG-bo	protein_coding	37	31	38	32	0.837
11170	NM_001076778,NM	**FAM107A**	family with sequence sim	protein_coding	0	0	1	1	1.000
11171	NM_007178,XM_00	**STRAP**	serine/threonine kinase r	protein_coding	136	110	137	111	0.814
11172	NM_007179,XM_01	**INSL6**	insulin like 6	protein_coding	0	0	1	1	1.000
11173	NM_014272,XM_00	**ADAMTS7**	ADAM metallopeptidase	protein_coding	0	0	1	1	1.000
11174	NM_197941,NR_13	**ADAMTS6**	ADAM metallopeptidase	protein_coding	1	1	2	2	0.885
11176	NM_001300905,NM	**BAZ2A**	bromodomain adjacent to	protein_coding	44	35	45	36	0.785
11177	NM_013448,NM_18	**BAZ1A**	bromodomain adjacent to	protein_coding	77	64	78	65	0.838
11178	NM_001362884,NM	**LZTS1**	leucine zipper tumor sup	protein_coding	26	20	27	21	0.768
11179	NM_021994,XM_00	**ZNF277**	zinc finger protein 277	protein_coding	13	12	14	13	0.986
11180	NM_001320546,NM	**WDR6**	WD repeat domain 6	protein_coding	62	61	63	62	0.982
11181	NM_001301065,NM	**TREH**	trehalase	protein_coding	0	0	1	1	1.000
11182	NM_001145099,NM	**SLC2A6**	solute carrier family 2 me	protein_coding	2	3	3	4	1.192
11183	NM_006575,NM_19	**MAP4K5**	mitogen-activated protein	protein_coding	37	32	38	33	0.881
11184	NM_001042600,NM	**MAP4K1**	mitogen-activated protein	protein_coding	112	113	113	114	1.016
11185	NM_001199219,NM	**INMT**	indolethylamine N-methy	protein_coding	0	0	1	1	1.000
11186	NM_001206957,NM	**RASSF1**	Ras association domain	protein_coding	55	57	56	58	1.038
11187	NM_001303029,NM	**PKP3**	plakophilin 3	protein_coding	0	0	1	1	1.000
11188	NM_001276293,NM	**NISCH**	nischarin	protein_coding	29	28	30	29	0.974
11189	NM_001172648,NM	**CELF3**	CUGBP Elav-like family r	protein_coding	0	0	1	1	1.000
11190	NM_001318219,NM	**CEP250**	centrosomal protein 250	protein_coding	42	41	43	42	0.988
11191	NR_023917	**PTENP1**	phosphatase and tensin	transcribed_pseu	1	1	2	2	1.002
11193	NM_007187,XM_00	**WBP4**	WW domain binding prot	protein_coding	24	21	25	22	0.880
11194	NM_001282291,NM	**ABCB8**	ATP binding cassette sul	protein_coding	8	8	9	9	0.995
11196	NM_007190,NR_03	**SEC23IP**	SEC23 interacting protei	protein_coding	34	30	35	31	0.898
11197	NM_007191	**WIF1**	WNT inhibitory factor 1	protein_coding	0	0	1	1	1.000
11198	NM_007192,XM_01	**SUPT16H**	SPT16 homolog, facilitat	protein_coding	246	229	247	230	0.930
11199	NM_007193,XM_01	**ANXA10**	annexin A10	protein_coding	0	0	1	1	1.000
11200	NM_001005735,NM	**CHEK2**	checkpoint kinase 2	protein_coding	17	17	18	18	0.992
11201	NM_001351610,NM	**POLI**	DNA polymerase iota	protein_coding	2	2	3	3	1.011
11202	NM_001281431,NM	**KLK8**	kallikrein related peptida	protein_coding	0	0	1	1	1.000
11209	NR_027504	**MST1P2**	macrophage stimulating	transcribed_pseu	0	0	1	1	1.297
11211	NM_007197	**FZD10**	frizzled class receptor 10	protein_coding	0	0	1	1	1.000
11212	NM_001349346,NM	**PLPBP**	pyridoxal phosphate binc	protein_coding	167	160	168	161	0.959

11213	NM_001142523,NM	**IRAK3**	interleukin 1 receptor ass	protein_coding	0	0	1	1	0.973
11214	NM_001270546,NM	**AKAP13**	A-kinase anchoring prote	protein_coding	38	33	39	34	0.878
11215	NM_016248,XM_00	**AKAP11**	A-kinase anchoring prote	protein_coding	21	18	22	19	0.865
11216	NM_001330152,NM	**AKAP10**	A-kinase anchoring prote	protein_coding	12	12	13	13	1.014
11218	NM_007204	**DDX20**	DEAD-box helicase 20	protein_coding	24	22	25	23	0.943
11219	NM_080701	**TREX2**	three prime repair exonu	protein_coding	2	2	3	3	1.029
11221	NM_007207,NR_11	**DUSP10**	dual specificity phosphat	protein_coding	14	13	15	14	0.896
11222	NM_007208	**MRPL3**	mitochondrial ribosomal	protein_coding	333	309	334	310	0.926
11223	NM_001271733	**MST1L**	macrophage stimulating	protein_coding	0	0	1	1	1.084
11224	NM_007209	**RPL35**	ribosomal protein L35	protein_coding	2,648	2,707	2,649	2,708	1.022
11226	NM_007210,XM_00	**GALNT6**	polypeptide N-acetylgala	protein_coding	8	4	9	5	0.580
11227	NM_001329868,NM	**GALNT5**	polypeptide N-acetylgala	protein_coding	0	0	1	1	1.000
11228	NM_001164746,NM	**RASSF8**	Ras association domain	protein_coding	0	0	1	1	1.000
11230	NM_007213	**PRAF2**	PRA1 domain family mer	protein_coding	26	27	27	28	1.063
11231	NM_007214,XM_01	**SEC63**	SEC63 homolog, protein	protein_coding	54	45	55	46	0.834
11232	NM_007215	**POLG2**	DNA polymerase gamma	protein_coding	22	23	23	24	1.057
11234	NM_007216,NM_18	**HPS5**	HPS5 biogenesis of lyso	protein_coding	27	25	28	26	0.917
11235	NM_007217,NM_14	**PDCD10**	programmed cell death 1	protein_coding	100	94	101	95	0.945
11236	NM_007218	**RNF139**	ring finger protein 139	protein_coding	17	16	18	17	0.979
11237	NM_001134337,NM	**RNF24**	ring finger protein 24	protein_coding	4	3	5	4	0.844
11238	NM_007220	**CA5B**	carbonic anhydrase 5B	protein_coding	1	1	2	2	0.990
11240	NM_007365,XM_01	**PADI2**	peptidyl arginine deimina	protein_coding	0	0	1	1	1.039
11243	NM_001199653,NM	**PMF1**	polyamine modulated fac	protein_coding	134	125	135	126	0.931
11244	NM_001017926,NM	**ZHX1**	zinc fingers and homeob	protein_coding	47	43	48	44	0.922
11245	NM_001271854,NM	**GPR176**	G protein-coupled recep	protein_coding	0	0	1	1	1.000
11247	NM_007224,XM_01	**NXPH4**	neurexophilin 4	protein_coding	0	0	1	1	1.000
11248	NM_007225	**NXPH3**	neurexophilin 3	protein_coding	0	0	1	1	1.000
11249	NM_007226	**NXPH2**	neurexophilin 2	protein_coding	0	0	1	1	1.000
11250	NM_007227	**GPR45**	G protein-coupled recep	protein_coding	0	0	1	1	1.000
11251	NM_004778	**PTGDR2**	prostaglandin D2 recepto	protein_coding	0	0	1	1	1.000
11252	NM_001184970,NM	**PACSIN2**	protein kinase C and cas	protein_coding	121	110	122	111	0.916
11253	NM_016219,NR_04	**MAN1B1**	mannosidase alpha clas	protein_coding	25	25	26	26	0.996
11254	NM_007231	**SLC6A14**	solute carrier family 6 me	protein_coding	0	0	1	1	1.000
11255	NM_007232,XM_00	**HRH3**	histamine receptor H3	protein_coding	0	0	1	1	1.000
11257	NR_015381	**TP53TG1**	TP53 target 1	lncRNA	15	11	16	12	0.741
11258	NM_001281425,NM	**DCTN3**	dynactin subunit 3	protein_coding	91	99	92	100	1.080
11259	NM_001042459,NM	**FILIP1L**	filamin A interacting prot	protein_coding	0	0	1	1	1.000
11260	NM_007235,XM_01	**XPOT**	exportin for tRNA	protein_coding	61	52	62	53	0.858
11261	NM_007236,XM_01	**CHP1**	calcineurin like EF-hand	protein_coding	83	76	84	77	0.920
11262	NM_001005176,NM	**SP140**	SP140 nuclear body prot	protein_coding	64	54	65	55	0.847
11264	NM_007238,NM_18	**PXMP4**	peroxisomal membrane p	protein_coding	3	3	4	4	1.072
11266	NM_007240,XM_00	**DUSP12**	dual specificity phosphat	protein_coding	82	79	83	80	0.965
11267	NM_001317192,NM	**SNF8**	SNF8 subunit of ESCRT	protein_coding	132	143	133	144	1.086
11269	NM_001014449,NM	**DDX19B**	DEAD-box helicase 19B	protein_coding	52	49	53	50	0.946
11270	NM_001270707,NM	**NRM**	nurim	protein_coding	28	29	29	30	1.041
11272	NM_001098538,NM	**PRR4**	proline rich 4	protein_coding	0	0	1	1	1.000
11273	NM_001308230,NM	**ATXN2L**	ataxin 2 like	protein_coding	142	139	143	140	0.982
11274	NM_017414,XM_00	**USP18**	ubiquitin specific peptida	protein_coding	36	32	37	33	0.888
11275	NM_001161521,NM	**KLHL2**	kelch like family member	protein_coding	27	27	28	28	0.975
11276	NM_001163544,NM	**SYNRG**	synergin gamma	protein_coding	34	29	35	30	0.852
11277	NM_007248,NM_03	**TREX1**	three prime repair exonu	protein_coding	8	9	9	10	1.152
11278	NM_007249,XM_00	**KLF12**	Kruppel like factor 12	protein_coding	7	6	8	7	0.927
11279	NM_001159296,NM	**KLF8**	Kruppel like factor 8	protein_coding	0	0	1	1	1.000
11280	NM_001349253,NM	**SCN11A**	sodium voltage-gated ch	protein_coding	0	0	1	1	1.000
11281	NM_001166018,NM	**POU6F2**	POU class 6 homeobox 2	protein_coding	0	0	1	1	1.000
11282	NM_014275,NM_05	**MGAT4B**	alpha-1,3-mannosyl-glyc	protein_coding	34	38	35	39	1.113
11283	NM_007253,XM_02	**CYP4F8**	cytochrome P450 family	protein_coding	0	0	1	1	1.000
11284	NM_007254	**PNKP**	polynucleotide kinase 3'-	protein_coding	22	22	23	23	0.997
11285	NM_007255,XM_00	**B4GALT7**	beta-1,4-galactosyltransf	protein_coding	21	24	22	25	1.147
11309	NM_001145211,NM	**SLCO2B1**	solute carrier organic ani	protein_coding	0	0	1	1	0.946
11311	NM_001279353,NM	**VPS45**	vacuolar protein sorting 4	protein_coding	41	42	42	43	1.023
11313	NM_007260,XM_00	**LYPLA2**	lysophospholipase 2	protein_coding	107	128	108	129	1.194
11314	NM_001256841,NM	**CD300A**	CD300a molecule	protein_coding	0	0	1	1	1.000
11315	NM_001123377,NM	**PARK7**	Parkinsonism associated	protein_coding	491	506	492	507	1.032
11316	NM_001330469,NM	**COPE**	COPI coat complex subu	protein_coding	274	290	275	291	1.055
11317	NM_001281448,NM	**RBPJL**	recombination signal bin	protein_coding	0	0	1	1	1.000
11318	NM_007264,XM_01	**GPR182**	G protein-coupled recep	protein_coding	0	0	1	1	1.000
11319	NM_001135752,NM	**ECD**	ecdysoneless cell cycle r	protein_coding	46	43	47	44	0.934
11320	NM_001160154,NM	**MGAT4A**	alpha-1,3-mannosyl-glyc	protein_coding	0	0	1	1	1.000
11321	NM_001145047,NM	**GPN1**	GPN-loop GTPase 1	protein_coding	72	70	73	71	0.972
11322	NM_001127198,NM	**TMC6**	transmembrane channel	protein_coding	32	33	33	34	1.032
11325	NM_007372,NM_20	**DDX42**	DEAD-box helicase 42	protein_coding	135	125	136	126	0.923
11326	NM_001100431,NM	**VSIG4**	V-set and immunoglobul	protein_coding	0	0	1	1	1.000
11328	NM_001284341,NM	**FKBP9**	FKBP prolyl isomerase 9	protein_coding	0	0	1	1	1.000
11329	NM_001305102,NM	**STK38**	serine/threonine kinase 3	protein_coding	57	60	58	61	1.049
11330	NM_007272,XM_01	**CTRC**	chymotrypsin C	protein_coding	0	0	1	1	1.000
11331	NM_001144831,NM	**PHB2**	prohibitin 2	protein_coding	355	368	356	369	1.036
11332	NM_007274,NM_18	**ACOT7**	acyl-CoA thioesterase 7	protein_coding	178	200	179	201	1.122
11333	NM_014891	**PDAP1**	PDGFA associated prote	protein_coding	174	179	175	180	1.032
11334	NM_007275	**TUSC2**	tumor suppressor 2, mito	protein_coding	70	77	71	78	1.095
11335	NM_007276,NM_01	**CBX3**	chromobox 3	protein_coding	311	284	312	285	0.914
11336	NM_007277	**EXOC3**	exocyst complex compor	protein_coding	39	44	40	45	1.124
11337	NM_007278	**GABARAP**	GABA type A receptor-as	protein_coding	55	53	56	54	0.970
11338	NM_001012478,NM	**U2AF2**	U2 small nuclear RNA au	protein_coding	375	410	376	411	1.093
11339	NM_001317860,NM	**OIP5**	Opa interacting protein 5	protein_coding	64	73	65	74	1.140
11340	NM_181503,XM_00	**EXOSC8**	exosome component 8	protein_coding	126	119	127	120	0.944
11341	NM_001329597,NM	**SCRG1**	stimulator of chondroger	protein_coding	0	0	1	1	1.000
11342	NM_001378285,NM	**RNF13**	ring finger protein 13	protein_coding	34	26	35	27	0.779
11343	NM_001003794,NM	**MGLL**	monoglyceride lipase	protein_coding	0	0	1	1	1.000
11344	NM_007284	**TWF2**	twinfilin actin binding pro	protein_coding	85	91	86	92	1.075
11345	NM_007285	**GABARAPL2**	GABA type A receptor as	protein_coding	87	85	88	86	0.977
11346	NM_001109974,NM	**SYNPO**	synaptopodin	protein_coding	0	0	1	1	0.954
22794	NM_007359,XM_00	**CASC3**	CASC3 exon junction co	protein_coding	97	98	98	99	1.010
22795	NM_007361,XM_00	**NID2**	nidogen 2	protein_coding	0	0	1	1	0.988
22796	NM_001145036,NM	**COG2**	component of oligomeric	protein_coding	26	26	27	27	0.974
22797	NM_001018058,NM	**TFEC**	transcription factor EC	protein_coding	36	26	37	27	0.721
22798	NM_001318046,NM	**LAMB4**	laminin subunit beta 4	protein_coding	0	0	1	1	1.000
22800	NM_001102669,NM	**RRAS2**	RAS related 2	protein_coding	125	121	126	122	0.969
22801	NM_001004439,XM	**ITGA11**	integrin subunit alpha 11	protein_coding	0	0	1	1	1.000
22802	NM_012128,NR_02	**CLCA4**	chloride channel access	protein_coding	0	0	1	1	1.000
22803	NM_001317960,NM	**XRN2**	5'-3' exoribonuclease 2	protein_coding	120	107	121	108	0.897
22806	NM_001257408,NM	**IKZF3**	IKAROS family zinc finge	protein_coding	130	110	131	111	0.848
22807	NM_001079526,NM	**IKZF2**	IKAROS family zinc finge	protein_coding	68	54	69	55	0.803
22808	NM_001085049,NM	**MRAS**	muscle RAS oncogene h	protein_coding	0	0	1	1	1.000
22809	NM_001193646,NM	**ATF5**	activating transcription fa	protein_coding	33	40	34	41	1.186
22813	gene-TDGF1P6	**TDGF1P6**	teratocarcinoma-derived	pseudogene	0	0	1	1	1.000
22814	gene-TDGF1P5	**TDGF1P5**	teratocarcinoma-derived	pseudogene	0	0	1	1	1.000
22815	gene-TDGF1P4	**TDGF1P4**	teratocarcinoma-derived	pseudogene	0	0	1	1	1.000
22816	gene-TDGF1P2	**TDGF1P2**	teratocarcinoma-derived	pseudogene	0	0	1	1	1.000
22818	NM_001271734,NM	**COPZ1**	COPI coat complex subu	protein_coding	110	107	111	108	0.977
22820	NM_016128,XM_01	**COPG1**	COPI coat complex subu	protein_coding	93	92	94	93	0.984
22821	NM_001320821,NM	**RASA3**	RAS p21 protein activato	protein_coding	0	0	1	1	1.059
22822	NM_007350	**PHLDA1**	pleckstrin homology like	protein_coding	58	62	59	63	1.063
22823	NM_001164391,NM	**MTF2**	metal response element	protein_coding	71	56	72	57	0.789
22824	NM_001317381,NM	**HSPA4L**	heat shock protein family	protein_coding	0	0	1	1	0.967
22826	NM_014280,NR_15	**DNAJC8**	DnaJ heat shock protein	protein_coding	270	269	271	270	0.996
22827	NM_001136033,NM	**PUF60**	poly(U) binding splicing	protein_coding	106	120	107	121	1.132
22828	NM_001286188,NM	**SCAF8**	SR-related CTD associat	protein_coding	39	35	40	36	0.903
22829	NM_001164238,NM	**NLGN4Y**	neuroligin 4 Y-linked	protein_coding	0	0	1	1	1.013
22832	NM_001286206,NM	**CEP162**	centrosomal protein 162	protein_coding	3	3	4	4	0.984
22834	NM_001145365,NM	**ZNF652**	zinc finger protein 652	protein_coding	8	6	9	7	0.864
22835	NM_001320666,NM	**ZFP30**	ZFP30 zinc finger protein	protein_coding	0	0	1	1	1.000
22836	NM_014899,XM_01	**RHOBTB3**	Rho related BTB domain	protein_coding	0	0	1	1	1.000
22837	NM_001278458,NM	**COBLL1**	cordon-bleu WH2 repeat	protein_coding	0	0	1	1	1.000
22838	NM_014901,XM_00	**RNF44**	ring finger protein 44	protein_coding	39	40	40	41	1.010
22839	NM_001042486,NM	**DLGAP4**	DLG associated protein 4	protein_coding	21	19	22	20	0.900

22841	NM_001330167,NM	**RAB11FIP2**	RAB11 family interacting	protein_coding	8	6	9	7	0.792
22843	NM_014906,NR_04	**PPM1E**	protein phosphatase, Mg	protein_coding	0	0	1	1	1.000
22844	NM_001371223,NM	**FRMPD1**	FERM and PDZ domain	protein_coding	0	0	1	1	1.000
22845	NM_014908	**DOLK**	dolichol kinase	protein_coding	10	12	11	13	1.229
22846	NM_014909,XM_01	**VASH1**	vasohibin 1	protein_coding	0	0	1	1	1.090
22847	NM_001136156,NM	**ZNF507**	zinc finger protein 507	protein_coding	19	15	20	16	0.803
22848	NM_001371575,NM	**AAK1**	AP2 associated kinase 1	protein_coding	16	13	17	14	0.789
22849	NM_001178137,NM	**CPEB3**	cytoplasmic polyadenyla	protein_coding	1	1	2	2	1.001
22850	NM_014913,XM_00	**ADNP2**	ADNP homeobox 2	protein_coding	32	27	33	28	0.861
22852	NM_001256053,NM	**ANKRD26**	ankyrin repeat domain 26	protein_coding	2	2	3	3	1.122
22853	NM_014916,XM_01	**LMTK2**	lemur tyrosine kinase 2	protein_coding	16	15	17	16	0.932
22854	NM_001113226,NM	**NTNG1**	netrin G1	protein_coding	0	0	1	1	1.000
22856	NM_014918,XM_00	**CHSY1**	chondroitin sulfate synth	protein_coding	140	122	141	123	0.871
22858	NM_001375397,NM	**CILK1**	ciliogenesis associated k	protein_coding	2	1	3	2	0.836
22859	NM_001008701,NM	**ADGRL1**	adhesion G protein-coup	protein_coding	0	0	1	1	0.992
22861	NM_001033053,NM	**NLRP1**	NLR family pyrin domain	protein_coding	3	2	4	3	0.873
22862	NM_001079673,NM	**FNDC3A**	fibronectin type III domai	protein_coding	75	65	76	66	0.873
22863	NM_014924,XM_01	**ATG14**	autophagy related 14	protein_coding	4	3	5	4	0.832
22864	NM_001330121,NM	**R3HDM2**	R3H domain containing 2	protein_coding	12	13	13	14	1.051
22865	NM_001318810,NM	**SLITRK3**	SLIT and NTRK like fami	protein_coding	0	0	1	1	1.000
22866	NM_001168647,NM	**CNKSR2**	connector enhancer of ki	protein_coding	0	0	1	1	1.000
22868	NM_001136193,NM	**FASTKD2**	FAST kinase domains 2	protein_coding	21	21	22	22	1.002
22869	NM_001314059,NM	**ZNF510**	zinc finger protein 510	protein_coding	8	6	9	7	0.810
22870	NM_014931	**PPP6R1**	protein phosphatase 6 re	protein_coding	323	320	324	321	0.992
22871	NM_001365923,NM	**NLGN1**	neuroligin 1	protein_coding	1	1	2	2	1.089
22872	NM_001077206,NM	**SEC31A**	SEC31 homolog A, COP	protein_coding	42	42	43	43	1.000
22873	NM_014934,NM_19	**DZIP1**	DAZ interacting zinc fing	protein_coding	0	0	1	1	1.000
22874	NM_014935,XM_00	**PLEKHA6**	pleckstrin homology dom	protein_coding	0	0	1	1	0.992
22875	NM_014936	**ENPP4**	ectonucleotide pyrophos	protein_coding	32	25	33	26	0.801
22876	NM_001243194,NM	**INPP5F**	inositol polyphosphate-5	protein_coding	5	4	6	5	0.786
22877	NM_014938,XM_00	**MLXIP**	MLX interacting protein	protein_coding	10	10	11	11	0.963
22878	NM_014939,XM_00	**TRAPPC8**	trafficking protein particle	protein_coding	28	24	29	25	0.887
22879	NM_001286639,NM	**MON1B**	MON1 homolog B, secre	protein_coding	29	29	30	30	1.010
22880	NM_001303256,NM	**MORC2**	MORC family CW-type z	protein_coding	12	13	13	14	1.041
22881	NM_001242809,NM	**ANKRD6**	ankyrin repeat domain 6	protein_coding	0	0	1	1	1.024
22882	NM_001362797,NM	**ZHX2**	zinc fingers and homeob	protein_coding	0	0	1	1	1.000
22883	NM_001009566,NM	**CLSTN1**	calsyntenin 1	protein_coding	83	77	84	78	0.931
22884	NM_014023	**WDR37**	WD repeat domain 37	protein_coding	8	9	9	10	1.082
22885	NM_001301015,NM	**ABLIM3**	actin binding LIM protein	protein_coding	0	0	1	1	1.000
22887	NM_001198850,NM	**FOXJ3**	forkhead box J3	protein_coding	57	51	58	52	0.897
22888	NM_001267584,NM	**UBOX5**	U-box domain containing	protein_coding	6	7	7	8	1.097
22889	NM_014949	**KHDC4**	KH domain containing 4,	protein_coding	53	45	54	46	0.846
22890	NM_001123329,NM	**ZBTB1**	zinc finger and BTB dom	protein_coding	21	21	22	22	1.006
22891	NM_014951,NM_19	**ZNF365**	zinc finger protein 365	protein_coding	0	0	1	1	1.000
22893	NM_001301132,NM	**BAHD1**	bromo adjacent homolog	protein_coding	49	50	50	51	1.019
22894	NM_001128226,NM	**DIS3**	DIS3 homolog, exosome	protein_coding	30	27	31	28	0.901
22895	NM_001143854,NM	**RPH3A**	rabphilin 3A	protein_coding	0	0	1	1	1.000
22897	NM_001271933,NM	**CEP164**	centrosomal protein 164	protein_coding	21	19	22	20	0.902
22898	NM_001352890,NM	**DENND3**	DENN domain containing	protein_coding	29	23	30	24	0.782
22899	NM_025014,NM_17	**ARHGEF15**	Rho guanine nucleotide	protein_coding	0	0	1	1	1.000
22900	NM_001184900,NM	**CARD8**	caspase recruitment dom	protein_coding	47	41	48	42	0.879
22901	NM_001267727,NM	**ARSG**	arylsulfatase G	protein_coding	7	8	8	9	1.104
22902	NM_001037442,NM	**RUFY3**	RUN and FYVE domain c	protein_coding	2	2	3	3	1.086
22903	NM_001282550,NM	**BTBD3**	BTB domain containing 3	protein_coding	0	0	1	1	0.881
22904	NM_001100122,NM	**SBNO2**	strawberry notch homolo	protein_coding	36	37	37	38	1.034
22905	NM_001102664,NM	**EPN2**	epsin 2	protein_coding	0	0	1	1	0.977
22906	NM_001042646,NM	**TRAK1**	trafficking kinesin protein	protein_coding	9	7	10	8	0.805
22907	NM_001330990,NM	**DHX30**	DExH-box helicase 30	protein_coding	77	80	78	81	1.033
22908	NM_001319071,NM	**SACM1L**	SAC1 like phosphatidylin	protein_coding	84	75	85	76	0.899
22909	NM_001146094,NM	**FAN1**	FANCD2 and FANCI ass	protein_coding	1	1	2	2	0.995
22911	NM_001142550,NM	**WDR47**	WD repeat domain 47	protein_coding	12	12	13	13	0.974
22913	NM_007367,NM_01	**RALY**	RALY heterogeneous nu	protein_coding	247	251	248	252	1.015
22914	NM_007360	**KLRK1**	killer cell lectin like recep	protein_coding	17	16	18	17	0.907
22915	NM_001371403,NM	**MMRN1**	multimerin 1	protein_coding	0	0	1	1	0.980
22916	NM_001042540,NM	**NCBP2**	nuclear cap binding prot	protein_coding	150	137	151	138	0.910
22917	NM_207341,XM_01	**ZP1**	zona pellucida glycoprot	protein_coding	0	0	1	1	1.000
22918	NM_012072,XR_00	**CD93**	CD93 molecule	protein_coding	24	21	25	22	0.882
22919	NM_012325,XM_01	**MAPRE1**	microtubule associated p	protein_coding	454	418	455	419	0.922
22920	NM_001204514,NM	**KIFAP3**	kinesin associated protei	protein_coding	39	36	40	37	0.927
22921	NM_012228,XM_01	**MSRB2**	methionine sulfoxide red	protein_coding	0	0	1	1	1.000
22924	NM_001303050,NM	**MAPRE3**	microtubule associated p	protein_coding	0	0	1	1	1.000
22925	NM_001007267,NM	**PLA2R1**	phospholipase A2 recept	protein_coding	0	0	1	1	1.000
22926	NM_007348,XM_00	**ATF6**	activating transcription fa	protein_coding	28	26	29	27	0.931
22927	NM_014282,XM_00	**HABP4**	hyaluronan binding prote	protein_coding	7	8	8	9	1.030
22928	NM_012248	**SEPHS2**	selenophosphate synthe	protein_coding	178	194	179	195	1.089
22929	NM_001195602,NM	**SEPHS1**	selenophosphate synthe	protein_coding	70	66	71	67	0.934
22930	NM_001172435,NM	**RAB3GAP1**	RAB3 GTPase activating	protein_coding	28	21	29	22	0.781
22931	NM_001256410,NM	**RAB18**	RAB18, member RAS on	protein_coding	8	7	9	8	0.945
22932	NM_012230,NM_15	**POMZP3**	POM121 and ZP3 fusion	protein_coding	17	18	18	19	1.037
22933	NM_001193286,NM	**SIRT2**	sirtuin 2	protein_coding	88	90	89	91	1.021
22934	NM_144563	**RPIA**	ribose 5-phosphate isom	protein_coding	150	138	151	139	0.919
22936	NM_012081,XM_00	**ELL2**	elongation factor for RNA	protein_coding	97	94	98	95	0.972
22937	NM_001320044,NM	**SCAP**	SREBF chaperone	protein_coding	35	35	36	36	0.999
22938	NM_001318844,NM	**SNW1**	SNW domain containing	protein_coding	109	103	110	104	0.938
22941	NM_001379226,NM	**SHANK2**	SH3 and multiple ankyrin	protein_coding	0	0	1	1	0.979
22943	NM_012242	**DKK1**	dickkopf WNT signaling	protein_coding	0	0	1	1	1.000
22944	NM_012311,NR_04	**KIN**	Kin17 DNA and RNA bin	protein_coding	3	4	4	5	1.342
22945	gene-GPAA1P1	**GPAA1P1**	glycosylphosphatidylinos	pseudogene	0	0	1	1	1.000
22947	gene-DUX4L1	**DUX4L1**	double homeobox 4 like	pseudogene	0	0	1	1	1.000
22948	NM_001306153,NM	**CCT5**	chaperonin containing TC	protein_coding	588	572	589	573	0.972
22949	NM_001146108,NM	**PTGR1**	prostaglandin reductase	protein_coding	0	0	1	1	1.000
22950	NM_018158	**SLC4A1AP**	solute carrier family 4 me	protein_coding	33	33	34	34	0.991
22952	NR_040249	**CYP2G1P**	cytochrome P450 family	transcribed_pseu	0	0	1	1	1.000
22953	NM_001282164,NM	**P2RX2**	purinergic receptor P2X 2	protein_coding	0	0	1	1	1.000
22954	NM_001099679,NM	**TRIM32**	tripartite motif containing	protein_coding	10	9	11	10	0.934
22955	NM_001031694,NM	**SCMH1**	Scm polycomb group pro	protein_coding	8	7	9	8	0.975
22969	gene-RPL41P3	**RPL41P3**	ribosomal protein L41 ps	pseudogene	106	120	107	121	1.129
22970	gene-RPL41P2	**RPL41P2**	ribosomal protein L41 ps	pseudogene	3	1	4	2	0.668
22971	gene-RPL41P1	**RPL41P1**	ribosomal protein L41 ps	pseudogene	180	214	181	215	1.189
22973	NR_004405	**LAMB2P1**	laminin subunit beta 2 ps	transcribed_pseu	0	0	1	1	1.124
22974	NM_012112,XM_01	**TPX2**	TPX2 microtubule nuclea	protein_coding	311	291	312	292	0.935
22976	NM_007349,XM_00	**PAXIP1**	PAX interacting protein 1	protein_coding	27	25	28	26	0.925
22977	NM_012067,XM_01	**AKR7A3**	aldo-keto reductase fami	protein_coding	0	0	1	1	1.098
22978	NM_001134373,NM	**NT5C2**	5'-nucleotidase, cytosolic	protein_coding	54	47	55	48	0.880
22979	NM_001319099,NM	**EFR3B**	EFR3 homolog B	protein_coding	0	0	1	1	0.935
22980	NM_014972,XM_00	**TCF25**	transcription factor 25	protein_coding	67	77	68	78	1.142
22981	NM_001318226,NM	**NINL**	ninein like	protein_coding	0	0	1	1	1.016
22982	NM_014974,XM_00	**DIP2C**	disco interacting protein	protein_coding	0	0	1	1	1.000
22983	NM_014975	**MAST1**	microtubule associated s	protein_coding	0	0	1	1	1.000
22984	NM_014976,XM_00	**PDCD11**	programmed cell death 1	protein_coding	81	75	82	76	0.926
22985	NM_001164814,NM	**ACIN1**	apoptotic chromatin cond	protein_coding	141	136	142	137	0.960
22986	NM_014978,XM_01	**SORCS3**	sortilin related VPS10 do	protein_coding	0	0	1	1	1.000
22987	NM_001297716,NM	**SV2C**	synaptic vesicle glycopro	protein_coding	0	0	1	1	0.992
22989	NM_014981,XM_01	**MYH15**	myosin heavy chain 15	protein_coding	0	0	1	1	0.867
22990	NM_001308160,NM	**PCNX1**	pecanex 1	protein_coding	25	20	26	21	0.795
22992	NM_001256405,NM	**KDM2A**	lysine demethylase 2A	protein_coding	68	68	69	69	0.998
22993	NM_001366501,NM	**HMGXB3**	HMG-box containing 3	protein_coding	59	53	60	54	0.898
22994	NM_001009811,NM	**CEP131**	centrosomal protein 131	protein_coding	7	8	8	9	1.063
22995	NM_001194998,NM	**CEP152**	centrosomal protein 152	protein_coding	27	22	28	23	0.821
22996	NM_001080494,NM	**TTC39A**	tetratricopeptide repeat c	protein_coding	0	0	1	1	1.036
22997	NM_001277285	**IGSF9B**	immunoglobulin superfam	protein_coding	0	0	1	1	1.000
22998	NM_001112717,NM	**LIMCH1**	LIM and calponin homolo	protein_coding	0	0	1	1	1.000
22999	NM_001168407,NM	**RIMS1**	regulating synaptic mem	protein_coding	0	0	1	1	1.000
23001	NM_014991,XM_00	**WDFY3**	WD repeat and FYVE do	protein_coding	0	0	1	1	1.000
23002	NM_001270520,NM	**DAAM1**	dishevelled associated a	protein_coding	6	5	7	6	0.866
23005	NM_001128608,NM	**MAPKBP1**	mitogen-activated protein	protein_coding	16	14	17	15	0.889

23007	NM_001130960,NM	**PLCH1**	phospholipase C eta 1	protein_coding	0	0	1	1	1.000
23008	NM_014997,XM_00!	**KLHDC10**	kelch domain containing	protein_coding	13	12	14	13	0.938
23011	NM_014999	**RAB21**	RAB21, member RAS on	protein_coding	10	9	11	10	0.966
23012	NM_015000,XM_00!	**STK38L**	serine/threonine kinase 3	protein_coding	0	0	1	1	0.919
23013	NM_015001	**SPEN**	spen family transcription	protein_coding	36	30	37	31	0.853
23014	NM_015002,NM_03	**FBXO21**	F-box protein 21	protein_coding	19	17	20	18	0.904
23015	NM_001368071,NM	**GOLGA8A**	golgin A8 family member	protein_coding	25	17	26	18	0.696
23016	NM_015004,NR_02	**EXOSC7**	exosome component 7	protein_coding	113	125	114	126	1.107
23017	NM_012306,XM_00!	**FAIM2**	Fas apoptotic inhibitory r	protein_coding	0	0	1	1	1.000
23019	NM_001265612,NM	**CNOT1**	CCR4-NOT transcription	protein_coding	105	92	106	93	0.878
23020	NM_014014	**SNRNP200**	small nuclear ribonucleo	protein_coding	186	172	187	173	0.925
23022	NM_001166108,NM	**PALLD**	palladin, cytoskeletal ass	protein_coding	1	1	2	2	0.998
23023	NM_001017395,NM	**TMCC1**	transmembrane and coil	protein_coding	5	5	6	6	0.961
23024	NM_001303139,NM	**PDZRN3**	PDZ domain containing r	protein_coding	0	0	1	1	1.000
23025	NM_001080421,XM_	**UNC13A**	unc-13 homolog A	protein_coding	0	0	1	1	1.020
23026	NM_001198950,NM	**MYO16**	myosin XVI	protein_coding	1	0	2	1	0.935
23028	NM_001009999,NM	**KDM1A**	lysine demethylase 1A	protein_coding	107	103	108	104	0.959
23029	NM_001346738,NM	**RBM34**	RNA binding motif protei	protein_coding	74	66	75	67	0.890
23030	NM_001370093,NM	**KDM4B**	lysine demethylase 4B	protein_coding	17	17	18	18	1.020
23031	NM_015016,XM_00!	**MAST3**	microtubule associated s	protein_coding	13	13	14	14	0.955
23032	NM_001377430,NM	**USP33**	ubiquitin specific peptida	protein_coding	34	28	35	29	0.836
23033	NM_001199942,NM	**DOP1A**	DOP1 leucine zipper like	protein_coding	12	10	13	11	0.829
23034	NM_001161576,NM	**SAMD4A**	sterile alpha motif domai	protein_coding	0	0	1	1	1.014
23035	NM_001289003,NM	**PHLPP2**	PH domain and leucine r	protein_coding	5	4	6	5	0.889
23036	NM_001351444,NM	**ZNF292**	zinc finger protein 292	protein_coding	12	10	13	11	0.844
23037	NM_178140,XM_00!	**PDZD2**	PDZ domain containing 2	protein_coding	0	0	1	1	1.000
23038	NM_001276252,NM	**WDTC1**	WD and tetratricopeptide	protein_coding	10	10	11	11	0.970
23039	NM_001100161,NM	**XPO7**	exportin 7	protein_coding	47	40	48	41	0.857
23040	NM_001303052,NM	**MYT1L**	myelin transcription facto	protein_coding	0	0	1	1	1.000
23041	NM_001278469,NM	**MON2**	MON2 homolog, regulato	protein_coding	8	7	9	8	0.884
23042	NM_001285444,NM	**PDXDC1**	pyridoxal dependent dec	protein_coding	110	92	111	93	0.838
23043	NM_001161560,NM	**TNIK**	TRAF2 and NCK interact	protein_coding	25	23	26	24	0.903
23046	NM_001252100,NM	**KIF21B**	kinesin family member 2	protein_coding	112	101	113	102	0.900
23047	NM_015032,XM_00!	**PDS5B**	PDS5 cohesin associate	protein_coding	34	28	35	29	0.832
23048	NM_001363755,NM	**FNBP1**	formin binding protein 1	protein_coding	73	65	74	66	0.886
23049	NM_015092,XM_00!	**SMG1**	SMG1 nonsense mediate	protein_coding	74	57	75	58	0.779
23051	NM_015035,XM_00!	**ZHX3**	zinc fingers and homeob	protein_coding	6	5	7	6	0.938
23052	NM_015036	**ENDOD1**	endonuclease domain co	protein_coding	4	3	5	4	0.836
23053	NM_001242487,NM	**ZSWIM8**	zinc finger SWIM-type co	protein_coding	7	6	8	7	0.921
23054	NM_001242539,NM	**NCOA6**	nuclear receptor coactiva	protein_coding	38	32	39	33	0.851
23057	NM_015039,NM_17	**NMNAT2**	nicotinamide nucleotide a	protein_coding	0	0	1	1	1.018
23059	NM_001330454,NM	**CLUAP1**	clusterin associated prote	protein_coding	7	7	8	8	1.089
23060	NM_015042,XM_01	**ZNF609**	zinc finger protein 609	protein_coding	35	30	36	31	0.859
23061	NM_015043,NM_19	**TBC1D9B**	TBC1 domain family mer	protein_coding	23	25	24	26	1.090
23062	NM_015044,XM_01	**GGA2**	golgi associated, gamma	protein_coding	56	50	57	51	0.887
23063	NM_001318328,NM	**WAPL**	WAPL cohesin release fa	protein_coding	49	43	50	44	0.878
23064	NM_001351527,NM	**SETX**	senataxin	protein_coding	30	24	31	25	0.813
23065	NM_001271427,NM	**EMC1**	ER membrane protein co	protein_coding	46	44	47	45	0.955
23066	NM_001162499,NM	**CAND2**	cullin associated and ne	protein_coding	0	0	1	1	1.000
23067	NM_001353345,XM	**SETD1B**	SET domain containing 1	protein_coding	17	16	18	17	0.950
23070	NM_015050,XM_00!	**CMTR1**	cap methyltransferase 1	protein_coding	64	60	65	61	0.935
23071	NM_015051	**ERP44**	endoplasmic reticulum p	protein_coding	28	25	29	26	0.892
23072	NM_001287059,NM	**HECW1**	HECT, C2 and WW dom	protein_coding	0	0	1	1	1.000
23074	NM_001006947,NM	**UHRF1BP1L**	UHRF1 binding protein 1	protein_coding	22	18	23	19	0.839
23075	NM_001297714,NM	**SWAP70**	switching B cell complex	protein_coding	7	5	8	6	0.739
23076	NM_015056	**RRP1B**	ribosomal RNA processir	protein_coding	111	99	112	100	0.888
23077	NM_015057,XM_00!	**MYCBP2**	MYC binding protein 2	protein_coding	43	30	44	31	0.702
23078	NM_001009814,NM	**VWA8**	von Willebrand factor A c	protein_coding	11	11	12	12	0.952
23080	NM_015060,XM_00!	**AVL9**	AVL9 cell migration asso	protein_coding	10	9	11	10	0.901
23081	NM_001146695,NM	**KDM4C**	lysine demethylase 4C	protein_coding	9	9	10	10	1.018
23082	NM_001288727,NM	**PPRC1**	PPARG related coactivat	protein_coding	51	52	52	53	1.016
23085	NM_001301248,NM	**ERC1**	ELKS/RAB6-interacting/C	protein_coding	1	1	2	2	0.837
23086	NM_001144763,NM	**EXPH5**	exophilin 5	protein_coding	0	0	1	1	1.000
23087	NM_001304495,NM	**TRIM35**	tripartite motif containing	protein_coding	16	15	17	16	0.968
23089	NM_001040152,NM	**PEG10**	paternally expressed 10	protein_coding	0	0	1	1	1.015
23090	NM_001271620,NM	**ZNF423**	zinc finger protein 423	protein_coding	0	0	1	1	1.011
23091	NM_001076788,NM	**ZC3H13**	zinc finger CCCH-type co	protein_coding	63	52	64	53	0.828
23092	NM_001135608,NM	**ARHGAP26**	Rho GTPase activating p	protein_coding	21	18	22	19	0.879
23093	NM_015072	**TTLL5**	tubulin tyrosine ligase lik	protein_coding	7	6	8	7	0.891
23094	NM_015073,XM_00!	**SIPA1L3**	signal induced proliferati	protein_coding	1	1	2	2	0.890
23095	NM_001365951,NM	**KIF1B**	kinesin family member 1	protein_coding	6	4	7	5	0.743
23096	NM_001111125,NM	**IQSEC2**	IQ motif and Sec7 domai	protein_coding	0	0	1	1	0.939
23097	NM_001300960,NM	**CDK19**	cyclin dependent kinase	protein_coding	6	6	7	7	1.023
23098	NM_015077	**SARM1**	sterile alpha and TIR mo	protein_coding	1	1	2	2	0.976
23099	NM_001135776,NM	**ZBTB43**	zinc finger and BTB dom	protein_coding	13	12	14	13	0.950
23101	NM_015078,XM_01	**MCF2L2**	MCF.2 cell line derived tr	protein_coding	1	2	2	3	1.195
23102	NM_015079,NM_14	**TBC1D2B**	TBC1 domain family mer	protein_coding	103	83	104	84	0.808
23105	NM_015082,XM_01	**FSTL4**	follistatin like 4	protein_coding	0	0	1	1	1.000
23107	NM_001286748,NM	**MRPS27**	mitochondrial ribosomal	protein_coding	97	91	98	92	0.945
23108	NM_001100398,NM	**RAP1GAP2**	RAP1 GTPase activating	protein_coding	1	1	2	2	1.386
23109	NM_015086,XM_01	**DDN**	dendrin	protein_coding	16	15	17	16	0.983
23111	NM_001142294,NM	**SPART**	spartin	protein_coding	1	0	2	1	0.791
23112	NM_001024843,NM	**TNRC6B**	trinucleotide repeat conta	protein_coding	9	8	10	9	0.870
23113	NM_015089,XM_01	**CUL9**	cullin 9	protein_coding	8	8	9	9	0.896
23114	NM_001005388,NM	**NFASC**	neurofascin	protein_coding	0	0	1	1	1.000
23116	NM_001308120,NM	**TOGARAM1**	TOG array regulator of a	protein_coding	2	2	3	3	1.081
23117	NM_130464	**NPIPB3**	nuclear pore complex int	protein_coding	13	15	14	16	1.086
23118	NM_001292034,NM	**TAB2**	TGF-beta activated kinas	protein_coding	55	47	56	48	0.859
23119	NM_015094,XM_01	**HIC2**	HIC ZBTB transcriptional	protein_coding	6	6	7	7	1.000
23120	NM_001366652,NM	**ATP10B**	ATPase phospholipid tra	protein_coding	0	0	1	1	1.000
23122	NM_001207044,NM	**CLASP2**	cytoplasmic linker associ	protein_coding	28	25	29	26	0.894
23125	NM_001171166,NM	**CAMTA2**	calmodulin binding trans	protein_coding	14	13	15	14	0.990
23126	NM_001194937,NM	**POGZ**	pogo transposable eleme	protein_coding	32	27	33	28	0.824
23127	NM_001303420,NM	**COLGALT2**	collagen beta(1-O)galact	protein_coding	0	0	1	1	1.003
23129	NM_015103,XM_01	**PLXND1**	plexin D1	protein_coding	0	0	1	1	1.006
23130	NM_001367971,NM	**ATG2A**	autophagy related 2A	protein_coding	13	13	14	14	0.969
23131	NM_001002909,NM	**GPATCH8**	G-patch domain containi	protein_coding	39	36	40	37	0.926
23132	NM_001322253,NM	**RAD54L2**	RAD54 like 2	protein_coding	21	18	22	19	0.868
23133	NM_001184896,NM	**PHF8**	PHD finger protein 8	protein_coding	35	34	36	35	0.996
23135	NM_001080424,NM	**KDM6B**	lysine demethylase 6B	protein_coding	7	6	8	7	0.914
23136	NM_001281533,NM	**EPB41L3**	erythrocyte membrane p	protein_coding	0	0	1	1	1.000
23137	NM_015110,XM_00!	**SMC5**	structural maintenance c	protein_coding	36	28	37	29	0.796
23138	NM_015111,XM_00!	**N4BP3**	NEDD4 binding protein 3	protein_coding	0	0	1	1	1.000
23139	NM_001319245,NM	**MAST2**	microtubule associated s	protein_coding	38	35	39	36	0.933
23140	NM_015113,XM_00!	**ZZEF1**	zinc finger ZZ-type and E	protein_coding	15	13	16	14	0.893
23141	NM_015114,XM_00!	**ANKLE2**	ankyrin repeat and LEM	protein_coding	26	26	27	27	1.003
23142	NM_001040402,NM	**DCUN1D4**	defective in cullin neddyl	protein_coding	2	2	3	3	0.860
23143	NM_001164211,NM	**LRCH1**	leucine rich repeats and	protein_coding	32	27	33	28	0.859
23144	NM_015117,XM_00!	**ZC3H3**	zinc finger CCCH-type co	protein_coding	7	8	8	9	1.126
23145	NR_163594	**SSPOP**	SCO-spondin, pseudoge	transcribed_pseu	0	0	1	1	1.000
23148	NM_001146334,XM	**NACAD**	NAC alpha domain conta	protein_coding	0	0	1	1	1.000
23149	NM_001161357,NM	**FCHO1**	FCH and mu domain cor	protein_coding	23	18	24	19	0.810
23150	NM_015123,XM_00!	**FRMD4B**	FERM domain containing	protein_coding	26	17	27	18	0.652
23151	NM_001366660,NM	**GRAMD4**	GRAM domain containing	protein_coding	4	5	5	6	1.116
23152	NM_001304815,NM	**CIC**	capicua transcriptional re	protein_coding	28	26	29	27	0.935
23154	NM_001014839,NM	**NCDN**	neurochondrin	protein_coding	34	40	35	41	1.181
23155	NM_001048210,NM	**CLCC1**	chloride channel CLIC lik	protein_coding	17	13	18	14	0.803
23157	NM_015129,NM_14	**SEPTIN6**	septin 6	protein_coding	113	106	114	107	0.940
23158	NM_015130	**TBC1D9**	TBC1 domain family mer	protein_coding	0	0	1	1	1.000
23160	NM_015131	**WDR43**	WD repeat domain 43	protein_coding	111	101	112	102	0.910
23161	NM_001350862,NM	**SNX13**	sorting nexin 13	protein_coding	10	8	11	9	0.899
23162	NM_001040439,NM	**MAPK8IP3**	mitogen-activated protein	protein_coding	9	10	10	11	1.065
23163	NM_001172703,NM	**GGA3**	golgi associated, gamma	protein_coding	45	49	46	50	1.081
23164	NM_001364716,NM	**MPRIP**	myosin phosphatase Rho	protein_coding	20	18	21	19	0.908
23165	NM_001329434,NM	**NUP205**	nucleoporin 205	protein_coding	92	79	93	80	0.855
23166	NM_015136,XM_00!	**STAB1**	stabilin 1	protein_coding	0	0	1	1	1.000

23167	NM_001323553,NM	**EFR3A**	EFR3 homolog A	protein_coding	26	22	27	23	0.857
23168	NM_015138	**RTF1**	RTF1 homolog, Paf1/RN	protein_coding	67	55	68	56	0.825
23169	NM_015139,XM_00	**SLC35D1**	solute carrier family 35 m	protein_coding	22	21	23	22	0.950
23170	NM_015140	**TTLL12**	tubulin tyrosine ligase lik	protein_coding	70	70	71	71	1.001
23171	NM_015141,XM_00	**GPD1L**	glycerol-3-phosphate de	protein_coding	10	9	11	10	0.902
23172	NM_032182,XM_01	**ABRAXAS2**	abraxas 2, BRISC compl	protein_coding	31	27	32	28	0.875
23173	NM_015143,XM_01	**METAP1**	methionyl aminopeptidas	protein_coding	79	69	80	70	0.876
23174	NM_015144,XM_00	**ZCCHC14**	zinc finger CCHC-type co	protein_coding	0	0	1	1	1.000
23175	NM_001261427,NM	**LPIN1**	lipin 1	protein_coding	39	34	40	35	0.880
23176	NM_001098811,NM	**SEPTIN8**	septin 8	protein_coding	9	9	10	10	1.037
23177	NM_001319100,NM	**CEP68**	centrosomal protein 68	protein_coding	0	0	1	1	0.886
23178	NM_001252119,NM	**PASK**	PAS domain containing s	protein_coding	26	25	27	26	0.980
23179	NM_001297669,NM	**RGL1**	ral guanine nucleotide di	protein_coding	3	3	4	4	0.836
23180	NM_015150,XM_00	**RFTN1**	raftlin, lipid raft linker 1	protein_coding	106	91	107	92	0.861
23181	NM_001146115,NM	**DIP2A**	disco interacting protein	protein_coding	21	20	22	21	0.962
23184	NM_015154,NR_12	**MESD**	mesoderm development	protein_coding	59	56	60	57	0.953
23185	NM_001351277,NM	**LARP4B**	La ribonucleoprotein 4B	protein_coding	14	12	15	13	0.865
23186	NM_015156,XR_94	**RCOR1**	REST corepressor 1	protein_coding	15	13	16	14	0.897
23187	NM_001144758,NM	**PHLDB1**	pleckstrin homology like	protein_coding	0	0	1	1	0.989
23189	NM_001256876,NM	**KANK1**	KN motif and ankyrin rep	protein_coding	1	1	2	2	0.990
23190	NM_014607	**UBXN4**	UBX domain protein 4	protein_coding	75	69	76	70	0.919
23191	NM_001033028,NM	**CYFIP1**	cytoplasmic FMR1 intera	protein_coding	1	0	2	1	0.912
23192	NM_013325,NM_17	**ATG4B**	autophagy related 4B cy	protein_coding	82	84	83	85	1.024
23193	NM_001278192,NM	**GANAB**	glucosidase II alpha sub	protein_coding	249	248	250	249	0.995
23194	NM_001278317,NM	**FBXL7**	F-box and leucine rich re	protein_coding	0	0	1	1	1.000
23195	NM_014611,XM_00	**MDN1**	midasin AAA ATPase 1	protein_coding	32	25	33	26	0.785
23196	NM_001286722,NM	**FAM120A**	family with sequence sim	protein_coding	115	108	116	109	0.938
23197	NM_014613,XM_01	**FAF2**	Fas associated factor fan	protein_coding	64	59	65	60	0.923
23198	NM_014614,XM_00	**PSME4**	proteasome activator sub	protein_coding	37	30	38	31	0.825
23199	NM_001134473,NM	**GSE1**	Gse1 coiled-coil protein	protein_coding	7	8	8	9	1.033
23200	NM_014616,XM_00	**ATP11B**	ATPase phospholipid tra	protein_coding	80	67	81	68	0.836
23201	NM_001286050,NM	**FAM168A**	family with sequence sim	protein_coding	19	17	20	18	0.865
23203	NM_001282944,NM	**PMPCA**	peptidase, mitochondrial	protein_coding	96	100	97	101	1.042
23204	NM_001313858,NM	**ARL6IP1**	ADP ribosylation factor li	protein_coding	164	165	165	166	1.001
23205	NM_001199377,NM	**ACSBG1**	acyl-CoA synthetase bub	protein_coding	0	0	1	1	1.002
23207	NM_015164,XM_00	**PLEKHM2**	pleckstrin homology and	protein_coding	71	66	72	67	0.938
23208	NM_152280,XM_00	**SYT11**	synaptotagmin 11	protein_coding	68	64	69	65	0.953
23209	NM_001376472,NM	**MLC1**	modulator of VRAC curre	protein_coding	81	88	82	89	1.091
23210	NM_001081461,NM	**JMJD6**	jumonji domain containin	protein_coding	40	42	41	43	1.049
23211	NM_015168,XM_00	**ZC3H4**	zinc finger CCCH-type co	protein_coding	48	49	49	50	1.005
23212	NM_015169	**RRS1**	ribosome biogenesis reg	protein_coding	193	194	194	195	1.003
23213	NM_001128204,NM	**SULF1**	sulfatase 1	protein_coding	0	0	1	1	1.000
23214	NM_001270940,NM	**XPO6**	exportin 6	protein_coding	167	163	168	164	0.975
23215	NM_015172,XM_00	**PRRC2C**	proline rich coiled-coil 2C	protein_coding	130	104	131	105	0.802
23216	NM_001253912,NM	**TBC1D1**	TBC1 domain family mer	protein_coding	32	31	33	32	0.959
23217	NM_001145640,NM	**ZFR2**	zinc finger RNA binding p	protein_coding	0	0	1	1	1.161
23218	NM_001365116,NM	**NBEAL2**	neurobeachin like 2	protein_coding	28	24	29	25	0.869
23219	NM_001136115,NM	**FBXO28**	F-box protein 28	protein_coding	20	18	21	19	0.921
23220	NM_001300727,NM	**DTX4**	deltex E3 ubiquitin ligase	protein_coding	0	0	1	1	1.000
23221	NM_001160036,NM	**RHOBTB2**	Rho related BTB domain	protein_coding	10	10	11	11	1.003
23223	NM_001145114,NM	**RRP12**	ribosomal RNA processin	protein_coding	59	61	60	62	1.023
23224	NM_015180,NM_18	**SYNE2**	spectrin repeat containin	protein_coding	8	6	9	7	0.809
23225	NM_024923,XM_00	**NUP210**	nucleoporin 210	protein_coding	136	133	137	134	0.978
23228	NM_001144382,NM	**PLCL2**	phospholipase C like 2	protein_coding	34	32	35	33	0.924
23229	NM_001173479,NM	**ARHGEF9**	Cdc42 guanine nucleotic	protein_coding	11	11	12	12	1.035
23230	NM_001018037,NM	**VPS13A**	vacuolar protein sorting 1	protein_coding	15	11	16	12	0.761
23231	NM_001297592,NM	**SEL1L3**	SEL1L family member 3	protein_coding	84	78	85	79	0.933
23232	NM_015188,XM_00	**TBC1D12**	TBC1 domain family mer	protein_coding	0	0	1	1	1.052
23233	NM_001321729,NM	**EXOC6B**	exocyst complex compor	protein_coding	0	0	1	1	1.000
23234	NM_015190	**DNAJC9**	DnaJ heat shock protein	protein_coding	135	132	136	133	0.980
23235	NM_015191,XM_01	**SIK2**	salt inducible kinase 2	protein_coding	10	10	11	11	1.030
23236	NM_015192,NM_18	**PLCB1**	phospholipase C beta 1	protein_coding	0	0	1	1	0.981
23237	NM_015193	**ARC**	activity regulated cytoske	protein_coding	0	0	1	1	1.000
23239	NM_194449,XM_01	**PHLPP1**	PH domain and leucine r	protein_coding	0	0	1	1	1.010
23240	NM_001131007,NM	**TMEM131L**	transmembrane 131 like	protein_coding	33	31	34	32	0.939
23241	NM_001100913,NM	**PACS2**	phosphofurin acidic clust	protein_coding	10	10	11	11	1.037
23242	NM_001287436,NM	**COBL**	cordon-bleu WH2 repeat	protein_coding	0	0	1	1	1.018
23243	NM_001195098,NM	**ANKRD28**	ankyrin repeat domain 28	protein_coding	35	35	36	36	0.980
23244	NM_001100399,NM	**PDS5A**	PDS5 cohesin associated	protein_coding	118	102	119	103	0.870
23245	NM_001184734,NM	**ASTN2**	astrotactin 2	protein_coding	2	3	3	4	1.249
23246	NM_015201	**BOP1**	BOP1 ribosomal biogene	protein_coding	102	108	103	109	1.059
23247	NM_015202,XM_00	**KATNIP**	katanin interacting protei	protein_coding	4	4	5	5	0.908
23248	NM_001297673,NM	**RPRD2**	regulation of nuclear pre	protein_coding	28	27	29	28	0.968
23250	NM_015205,NM_03	**ATP11A**	ATPase phospholipid tra	protein_coding	39	33	40	34	0.854
23251	NM_015206,XM_01	**MINAR1**	membrane integral NOTC	protein_coding	1	1	2	2	0.866
23252	NM_015207,XM_00	**OTUD3**	OTU deubiquitinase 3	protein_coding	8	7	9	8	0.850
23253	NM_001083625,NM	**ANKRD12**	ankyrin repeat domain 12	protein_coding	8	6	9	7	0.779
23254	NM_001017999,NM	**KAZN**	kazrin, periplakin interac	protein_coding	0	0	1	1	1.036
23255	NM_001378205,NM	**MTCL1**	microtubule crosslinking	protein_coding	0	0	1	1	0.987
23256	NM_001257376,NM	**SCFD1**	sec1 family domain conta	protein_coding	40	42	41	43	1.049
23258	NM_001243254,NM	**DENND5A**	DENN domain containing	protein_coding	14	13	15	14	0.920
23259	NM_001164232,NM	**DDHD2**	DDHD domain containing	protein_coding	34	28	35	29	0.828
23261	NM_001195563,NM	**CAMTA1**	calmodulin binding trans	protein_coding	38	39	39	40	1.028
23262	NM_001276277,NM	**PPIP5K2**	diphosphoinositol pentak	protein_coding	22	20	23	21	0.874
23263	NM_001112732,NM	**MCF2L**	MCF.2 cell line derived t	protein_coding	0	0	1	1	1.000
23264	NM_017590,XM_01	**ZC3H7B**	zinc finger CCCH-type co	protein_coding	62	59	63	60	0.953
23265	NM_001013839,NM	**EXOC7**	exocyst complex compor	protein_coding	52	51	53	52	0.981
23266	NM_001297704,NM	**ADGRL2**	adhesion G protein-coup	protein_coding	66	59	67	60	0.900
23268	NM_001318326,NM	**DNMBP**	dynamin binding protein	protein_coding	2	2	3	3	0.968
23269	NM_001080541,NM	**MGA**	MAX dimerization protein	protein_coding	4	3	5	4	0.864
23270	NM_021648	**TSPYL4**	TSPY like 4	protein_coding	38	37	39	38	0.983
23271	NM_001297707,NM	**CAMSAP2**	calmodulin regulated spe	protein_coding	1	1	2	2	0.902
23272	NM_001112736,NM	**TASOR**	transcription activation su	protein_coding	51	44	52	45	0.854
23274	NM_001243403,NM	**CLEC16A**	C-type lectin domain con	protein_coding	8	7	9	8	0.924
23275	NM_015227,NM_13	**POFUT2**	protein O-fucosyltransfer	protein_coding	10	7	11	8	0.790
23276	NM_025010,XM_00	**KLHL18**	kelch like family member	protein_coding	33	30	34	31	0.919
23277	NM_001366661,NM	**CLUH**	clustered mitochondria h	protein_coding	69	66	70	67	0.964
23279	NM_001318399,NM	**NUP160**	nucleoporin 160	protein_coding	107	93	108	94	0.875
23281	NM_001033602,NM	**MTUS2**	microtubule associated s	protein_coding	0	0	1	1	1.000
23283	NM_015235	**CSTF2T**	cleavage stimulation fact	protein_coding	41	44	42	45	1.053
23284	NM_001322246,NM	**ADGRL3**	adhesion G protein-coup	protein_coding	0	0	1	1	1.000
23286	NM_001161661,NM	**WWC1**	WW and C2 domain con	protein_coding	0	0	1	1	1.000
23287	NM_001286715,NM	**AGTPBP1**	ATP/GTP binding protein	protein_coding	18	17	19	18	0.933
23288	NM_001287499,NM	**IQCE**	IQ motif containing E	protein_coding	4	4	5	5	0.991
23291	NM_001378974,NM	**FBXW11**	F-box and WD repeat do	protein_coding	69	60	70	61	0.879
23293	NM_001256827,NM	**SMG6**	SMG6 nonsense mediate	protein_coding	10	11	11	12	1.013
23294	NM_015245,XM_00	**ANKS1A**	ankyrin repeat and sterile	protein_coding	14	13	15	14	0.963
23295	NM_001142289,NM	**MGRN1**	mahogunin ring finger 1	protein_coding	45	44	46	45	0.982
23299	NM_001003800,NM	**BICD2**	BICD cargo adaptor 2	protein_coding	21	20	22	21	0.931
23300	NM_001300728,NM	**ATMIN**	ATM interactor	protein_coding	30	29	31	30	0.963
23301	NM_001142614,NM	**EHBP1**	EH domain binding prote	protein_coding	12	9	13	10	0.771
23302	NM_015253,XM_00	**WSCD1**	WSC domain containing	protein_coding	0	0	1	1	1.000
23303	NM_015254,XM_00	**KIF13B**	kinesin family member 13	protein_coding	5	4	6	5	0.957
23304	NM_001184801,NM	**UBR2**	ubiquitin protein ligase E	protein_coding	17	15	18	16	0.922
23305	NM_001009185,NM	**ACSL6**	acyl-CoA synthetase lon	protein_coding	0	0	1	1	1.029
23306	NM_001130963,NM	**NEMP1**	nuclear envelope integra	protein_coding	38	35	39	36	0.933
23307	NM_015258,XM_00	**FKBP15**	FKBP prolyl isomerase 1	protein_coding	18	15	19	16	0.821
23308	NM_001283050,NM	**ICOSLG**	inducible T cell costimula	protein_coding	0	0	1	1	1.000
23309	NM_001297595,NM	**SIN3B**	SIN3 transcription regula	protein_coding	22	20	23	21	0.949
23310	NM_001372065,NM	**NCAPD3**	non-SMC condensin II co	protein_coding	63	53	64	54	0.855
23312	NM_001174116,NM	**DMXL2**	Dmx like 2	protein_coding	0	0	1	1	1.000
23313	NM_001009880,NM	**KIAA0930**	KIAA0930	protein_coding	2	2	3	3	0.963
23314	NM_001172509,NM	**SATB2**	SATB homeobox 2	protein_coding	0	0	1	1	1.000
23315	NM_001260491,NM	**SLC9A8**	solute carrier family 9 me	protein_coding	12	12	13	13	0.939
23316	NM_001370598,NM	**CUX2**	cut like homeobox 2	protein_coding	0	0	1	1	1.000
23317	NM_001329126,NM	**DNAJC13**	DnaJ heat shock protein	protein_coding	22	18	23	19	0.823

23318	NM_001009881,NM	**TUT4**	terminal uridylyl transfera	protein_coding	39	31	40	32	0.804
23321	NM_001130067,NM	**TRIM2**	tripartite motif containing	protein_coding	0	0	1	1	1.000
23322	NM_001127897,NM	**RPGRIP1L**	RPGRIP1 like	protein_coding	7	7	8	8	0.955
23324	NM_001292038,NM	**MAN2B2**	mannosidase alpha class	protein_coding	15	15	16	16	0.957
23325	NM_001293640,NM	**WASHC4**	WASH complex subunit	protein_coding	24	20	25	21	0.811
23326	NM_015276,XM_00	**USP22**	ubiquitin specific peptida	protein_coding	66	57	67	58	0.860
23327	NM_001144964,NM	**NEDD4L**	NEDD4 like E3 ubiquitin	protein_coding	2	2	3	3	1.120
23328	NM_001346505,NM	**SASH1**	SAM and SH3 domain co	protein_coding	0	0	1	1	1.000
23329	NM_001330186,NM	**TBC1D30**	TBC1 domain family mer	protein_coding	0	0	1	1	0.959
23331	NM_001145418,XM	**TTC28**	tetratricopeptide repeat c	protein_coding	0	0	1	1	0.990
23332	NM_001142273,NM	**CLASP1**	cytoplasmic linker associ	protein_coding	35	30	36	31	0.874
23333	NM_001366673,NM	**DPY19L1**	dpy-19 like C-mannosylt	protein_coding	10	10	11	11	0.978
23334	NM_001365999,NM	**SZT2**	SZT2 subunit of KICSTC	protein_coding	9	8	10	9	0.896
23335	NM_001382485,NM	**WDR7**	WD repeat domain 7	protein_coding	14	12	15	13	0.901
23336	NM_015286,NM_14	**SYNM**	synemin	protein_coding	0	0	1	1	1.000
23338	NM_001289984,NM	**JADE2**	jade family PHD finger 2	protein_coding	58	59	59	60	1.014
23339	NM_001301138,NM	**VPS39**	VPS39 subunit of HOPS	protein_coding	36	34	37	35	0.967
23341	NM_001287811,NM	**DNAJC16**	DnaJ heat shock protein	protein_coding	11	9	12	10	0.897
23344	NM_001184796,NM	**ESYT1**	extended synaptotagmin	protein_coding	75	75	76	76	1.000
23345	NM_001347701,NM	**SYNE1**	spectrin repeat containin	protein_coding	4	4	5	5	1.121
23347	NM_015295,XM_01	**SMCHD1**	structural maintenance c	protein_coding	55	45	56	46	0.823
23348	NM_001130048,NM	**DOCK9**	dedicator of cytokinesis 9	protein_coding	28	22	29	23	0.786
23349	NM_001304333,NM	**PHF24**	PHD finger protein 24	protein_coding	0	0	1	1	1.000
23350	NM_001080415,NM	**U2SURP**	U2 snRNP associated SU	protein_coding	95	81	96	82	0.854
23351	NM_001290256,NM	**KHNYN**	KH and NYN domain con	protein_coding	23	20	24	21	0.889
23352	NM_020765,XM_01	**UBR4**	ubiquitin protein ligase E	protein_coding	41	32	42	33	0.791
23353	NM_001130965,NM	**SUN1**	Sad1 and UNC84 domai	protein_coding	27	22	28	23	0.813
23354	NM_015302,XM_01	**HAUS5**	HAUS augmin like compl	protein_coding	22	24	23	25	1.089
23355	NM_001009921,NM	**VPS8**	VPS8 subunit of CORVE	protein_coding	20	16	21	17	0.815
23357	NM_001370746,NM	**ANGEL1**	angel homolog 1	protein_coding	6	6	7	7	1.107
23358	NM_015306,XM_00	**USP24**	ubiquitin specific peptida	protein_coding	48	39	49	40	0.800
23359	NM_015307,XM_01	**FAM189A1**	family with sequence sim	protein_coding	0	0	1	1	1.000
23360	NM_001318339,NM	**FNBP4**	formin binding protein 4	protein_coding	46	42	47	43	0.925
23361	NM_001080417,NM	**ZNF629**	zinc finger protein 629	protein_coding	0	0	1	1	1.032
23362	NM_001362819,NM	**PSD3**	pleckstrin and Sec7 dom	protein_coding	0	0	1	1	0.984
23363	NM_001173408,NM	**OBSL1**	obscurin like cytoskeleta	protein_coding	0	0	1	1	1.000
23365	NM_001198665,NM	**ARHGEF12**	Rho guanine nucleotide	protein_coding	24	20	25	21	0.843
23366	NM_001100425,NM	**KIAA0895**	KIAA0895	protein_coding	0	0	1	1	1.022
23367	NM_001367713,NM	**LARP1**	La ribonucleoprotein 1, tr	protein_coding	247	240	248	241	0.971
23368	NM_015316,XM_00	**PPP1R13B**	protein phosphatase 1 re	protein_coding	9	9	10	10	1.030
23369	NM_001282752,NM	**PUM2**	pumilio RNA binding fam	protein_coding	72	60	73	61	0.842
23370	NM_001130955,NM	**ARHGEF18**	Rho/Rac guanine nucleo	protein_coding	25	24	26	25	0.962
23371	NM_015319,NM_17	**TNS2**	tensin 2	protein_coding	0	0	1	1	1.063
23373	NM_001098482,NM	**CRTC1**	CREB regulated transcri	protein_coding	4	4	5	5	1.206
23376	NM_015323	**UFL1**	UFM1 specific ligase 1	protein_coding	24	19	25	20	0.830
23378	NM_015324,XM_01	**RRP8**	ribosomal RNA processin	protein_coding	29	24	30	25	0.839
23379	NM_015325,XM_01	**ICE1**	interactor of little elongat	protein_coding	34	30	35	31	0.881
23380	NM_001170637,NM	**SRGAP2**	SLIT-ROBO Rho GTPase	protein_coding	17	16	18	17	0.946
23381	NM_001323614,NM	**SMG5**	SMG5 nonsense mediate	protein_coding	74	70	75	71	0.942
23382	NM_001130720,NM	**AHCYL2**	adenosylhomocysteinase	protein_coding	7	5	8	6	0.750
23383	NM_015329,XM_00	**MAU2**	MAU2 sister chromatid co	protein_coding	29	31	30	32	1.062
23384	NM_001145468,NM	**SPECC1L**	sperm antigen with calpo	protein_coding	24	23	25	24	0.963
23385	NM_001290184,NM	**NCSTN**	nicastrin	protein_coding	47	47	48	48	1.012
23386	NM_015332,XM_01	**NUDCD3**	NudC domain containing	protein_coding	15	14	16	15	0.930
23387	NM_001281748,NM	**SIK3**	SIK family kinase 3	protein_coding	21	20	22	21	0.930
23389	NM_015335,XM_01	**MED13L**	mediator complex subun	protein_coding	14	10	15	11	0.706
23390	NM_001359626,NM	**ZDHHC17**	zinc finger DHHC-type pa	protein_coding	11	10	12	11	0.964
23392	NM_001363756,NM	**ECPAS**	Ecm29 proteasome adap	protein_coding	38	33	39	34	0.857
23394	NM_001282531,NM	**ADNP**	activity dependent neuro	protein_coding	63	56	64	57	0.890
23395	NM_001368263,NM	**LARS2**	leucyl-tRNA synthetase 2	protein_coding	32	33	33	34	1.024
23396	NM_001195733,NM	**PIP5K1C**	phosphatidylinositol-4-ph	protein_coding	9	9	10	10	1.043
23397	NM_001281710,NM	**NCAPH**	non-SMC condensin I co	protein_coding	66	62	67	63	0.938
23398	NM_001278926,NM	**PPWD1**	peptidylprolyl isomerase	protein_coding	64	64	65	65	1.012
23399	NM_001143775,NM	**CTDNEP1**	CTD nuclear envelope ph	protein_coding	95	99	96	100	1.052
23400	NM_001141973,NM	**ATP13A2**	ATPase cation transporti	protein_coding	5	5	6	6	1.003
23401	NM_012083	**FRAT2**	FRAT regulator of WNT s	protein_coding	5	5	6	6	1.055
23403	NM_001080469,NM	**FBXO46**	F-box protein 46	protein_coding	21	21	22	22	1.007
23404	NM_001282708,NM	**EXOSC2**	exosome component 2	protein_coding	95	89	96	90	0.946
23405	NM_001195573,NM	**DICER1**	dicer 1, ribonuclease III	protein_coding	33	28	34	29	0.857
23406	NM_021149	**COTL1**	coactosin like F-actin bin	protein_coding	1,354	1,351	1,355	1,352	0.998
23408	NM_001193267,NM	**SIRT5**	sirtuin 5	protein_coding	7	7	8	8	1.069
23409	NM_012240,XM_00	**SIRT4**	sirtuin 4	protein_coding	0	0	1	1	1.229
23410	NM_001017524,NM	**SIRT3**	sirtuin 3	protein_coding	4	5	5	6	1.205
23411	NM_001142498,NM	**SIRT1**	sirtuin 1	protein_coding	25	22	26	23	0.895
23412	NM_012071	**COMMD3**	COMM domain containin	protein_coding	27	22	28	23	0.838
23413	NM_001128826,NM	**NCS1**	neuronal calcium sensor	protein_coding	0	0	1	1	0.982
23414	NM_001362836,NM	**ZFPM2**	zinc finger protein, FOG	protein_coding	0	0	1	1	1.000
23415	NM_012285,XM_01	**KCNH4**	potassium voltage-gated	protein_coding	0	0	1	1	1.000
23416	NM_001314030,NM	**KCNH3**	potassium voltage-gated	protein_coding	0	0	1	1	1.000
23417	NM_012213	**MLYCD**	malonyl-CoA decarboxyla	protein_coding	1	1	2	2	1.100
23418	NM_001193640,NM	**CRB1**	crumbs cell polarity com	protein_coding	0	0	1	1	1.031
23420	NM_014287	**NOMO1**	NODAL modulator 1	protein_coding	92	92	93	93	1.004
23421	NM_001206739,NM	**ITGB3BP**	integrin subunit beta 3 b	protein_coding	112	101	113	102	0.897
23423	NM_001301203,NM	**TMED3**	transmembrane p24 traff	protein_coding	139	133	140	134	0.953
23424	NM_001302884,NM	**TDRD7**	tudor domain containing	protein_coding	12	11	13	12	0.900
23426	NM_001178074,NM	**GRIP1**	glutamate receptor intera	protein_coding	0	0	1	1	0.982
23428	NM_001267036,NM	**SLC7A8**	solute carrier family 7 me	protein_coding	0	0	1	1	1.066
23429	NM_012234	**RYBP**	RING1 and YY1 binding	protein_coding	33	26	34	27	0.802
23430	NM_012217	**TPSD1**	tryptase delta 1	protein_coding	0	0	1	1	1.000
23431	NM_001252127,NM	**AP4E1**	adaptor related protein c	protein_coding	23	19	24	20	0.804
23432	NM_001267609,NM	**GPR161**	G protein-coupled recept	protein_coding	0	0	1	1	1.000
23433	NM_012249,XM_00	**RHOQ**	ras homolog family mem	protein_coding	13	10	14	11	0.805
23434	NR_125802	**LINC01565**	long intergenic non-prote	lncRNA	0	0	1	1	0.934
23435	NM_007375,XM_01	**TARDBP**	TAR DNA binding protei	protein_coding	214	185	215	186	0.868
23436	NM_007352	**CELA3B**	chymotrypsin like elastas	protein_coding	0	0	1	1	1.000
23437	rna-TRS-AGA2-3	**TRS-AGA2-3**	tRNA-Ser	tRNA	1	0	2	1	0.640
23438	NM_001278731,NM	**HARS2**	histidyl-tRNA synthetase	protein_coding	39	41	40	42	1.070
23439	NM_001142447,NM	**ATP1B4**	ATPase Na+/K+ transpor	protein_coding	0	0	1	1	1.000
23440	NM_032109	**OTP**	orthopedia homeobox	protein_coding	0	0	1	1	1.000
23443	NM_001271684,NM	**SLC35A3**	solute carrier family 35 m	protein_coding	15	11	16	12	0.746
23446	NM_001286730,NM	**SLC44A1**	solute carrier family 44 m	protein_coding	42	35	43	36	0.827
23450	NM_012426	**SF3B3**	splicing factor 3b subuni	protein_coding	80	76	81	77	0.947
23451	NM_001005526,NM	**SF3B1**	splicing factor 3b subuni	protein_coding	290	249	291	250	0.859
23452	NM_012098,XM_00	**ANGPTL2**	angiopoietin like 2	protein_coding	0	0	1	1	1.000
23456	NM_012089,XM_01	**ABCB10**	ATP binding cassette sul	protein_coding	20	18	21	19	0.930
23457	NM_001243013,NM	**ABCB9**	ATP binding cassette sul	protein_coding	0	0	1	1	1.058
23460	NM_080284,XM_01	**ABCA6**	ATP binding cassette sul	protein_coding	0	0	1	1	1.000
23461	NM_018672,NM_17	**ABCA5**	ATP binding cassette sul	protein_coding	0	0	1	1	0.955
23462	NM_001040708,NM	**HEY1**	hes related family bHLH	protein_coding	0	0	1	1	0.820
23463	NM_012405,XM_01	**ICMT**	isoprenylcysteine carbox	protein_coding	165	162	166	163	0.982
23464	NM_001171690,NM	**GCAT**	glycine C-acetyltransfera	protein_coding	17	18	18	19	1.094
23466	NM_001303494,NM	**CBX6**	chromobox 6	protein_coding	120	119	121	120	0.994
23467	NM_014293	**NPTXR**	neuronal pentraxin recep	protein_coding	0	0	1	1	1.000
23468	NM_001127321,NM	**CBX5**	chromobox 5	protein_coding	58	48	59	49	0.824
23469	NM_001290259,NM	**PHF3**	PHD finger protein 3	protein_coding	20	15	21	16	0.775
23471	NM_001317804,NM	**TRAM1**	translocation associated	protein_coding	126	106	127	107	0.848
23473	NM_001376086,NM	**CAPN7**	calpain 7	protein_coding	25	24	26	25	0.978
23474	NM_001320867,NM	**ETHE1**	ETHE1 persulfide dioxyg	protein_coding	13	13	14	14	0.969
23475	NM_001318249,NM	**QPRT**	quinolinate phosphoribos	protein_coding	13	11	14	12	0.829
23476	NM_001330384,NM	**BRD4**	bromodomain containing	protein_coding	54	53	55	54	0.975
23478	NM_001271918,NM	**SEC11A**	SEC11 homolog A, signa	protein_coding	227	219	228	220	0.963
23479	NM_001301140,NM	**ISCU**	iron-sulfur cluster assem	protein_coding	110	112	111	113	1.020
23480	NM_001012456,NM	**SEC61G**	SEC61 translocon subur	protein_coding	189	175	190	176	0.928
23481	NM_001243225,NM	**PES1**	pescadillo ribosomal bio	protein_coding	174	182	175	183	1.047
23483	NM_001304430,NM	**TGDS**	TDP-glucose 4,6-dehydr	protein_coding	49	49	50	50	0.997
23484	NM_001128208,NM	**LEPROTL1**	leptin receptor overlappi	protein_coding	22	21	23	22	0.948

23491	NM_001185176,NM	**CES3**	carboxylesterase 3	protein_coding	0	0	1	1	1.158
23492	NM_001346743,NM	**CBX7**	chromobox 7	protein_coding	1	1	2	2	1.100
23493	NM_012259,XM_01	**HEY2**	hes related family bHLH	protein_coding	0	0	1	1	1.000
23495	NM_012452	**TNFRSF13B**	TNF receptor superfamily	protein_coding	0	0	1	1	1.000
23498	NM_012205,XM_00	**HAAO**	3-hydroxyanthranilate 3,4	protein_coding	0	0	1	1	0.998
23499	NM_012090	**MACF1**	microtubule actin crosslin	protein_coding	57	42	58	43	0.740
23500	NM_001201427,NM	**DAAM2**	dishevelled associated a	protein_coding	0	0	1	1	1.000
23503	NM_015346,XM_01	**ZFYVE26**	zinc finger FYVE-type cc	protein_coding	9	8	10	9	0.921
23504	NM_001351226,NM	**RIMBP2**	RIMS binding protein 2	protein_coding	0	0	1	1	1.027
23505	NM_015348,XM_00	**TMEM131**	transmembrane protein 1	protein_coding	31	26	32	27	0.829
23506	NM_001318819,NM	**BICRAL**	BRD4 interacting chroma	protein_coding	15	12	16	13	0.803
23507	NM_001134476,NM	**LRRC8B**	leucine rich repeat conta	protein_coding	4	3	5	4	0.792
23508	NM_015351	**TTC9**	tetratricopeptide repeat c	protein_coding	0	0	1	1	1.000
23509	NM_015352,NM_17	**POFUT1**	protein O-fucosyltransfer	protein_coding	95	93	96	94	0.980
23510	NM_015353,NR_11	**KCTD2**	potassium channel tetrar	protein_coding	27	28	28	29	1.040
23511	NM_015354	**NUP188**	nucleoporin 188	protein_coding	69	66	70	67	0.957
23512	NM_001321207,NM	**SUZ12**	SUZ12 polycomb repres	protein_coding	168	145	169	146	0.864
23513	NM_015356,NM_18	**SCRIB**	scribble planar cell polar	protein_coding	17	17	18	18	0.969
23514	NM_001080394,NM	**SPIDR**	scaffold protein involved	protein_coding	29	28	30	29	0.942
23515	NM_001320445,NM	**MORC3**	MORC family CW-type z	protein_coding	25	19	26	20	0.774
23516	NM_001128431,NM	**SLC39A14**	solute carrier family 39 m	protein_coding	74	64	75	65	0.872
23517	NM_015360	**MTREX**	Mtr4 exosome RNA helic	protein_coding	101	87	102	88	0.860
23518	NM_001282798,NM	**R3HDM1**	R3H domain containing 1	protein_coding	73	61	74	62	0.835
23519	NM_012404	**ANP32D**	acidic nuclear phosphop	protein_coding	0	0	1	1	0.956
23520	NM_012403	**ANP32C**	acidic nuclear phosphop	protein_coding	0	0	1	1	0.937
23521	NM_001270491,NM	**RPL13A**	ribosomal protein L13a	protein_coding	3,018	3,062	3,019	3,063	1.015
23522	NM_001256468,NM	**KAT6B**	lysine acetyltransferase 6	protein_coding	16	14	17	15	0.904
23523	NM_001199281,NM	**CABIN1**	calcineurin binding prote	protein_coding	29	30	30	31	1.018
23524	NM_016333,XM_00	**SRRM2**	serine/arginine repetitive	protein_coding	157	150	158	151	0.959
23526	NM_001258328,NM	**ARHGAP45**	Rho GTPase activating p	protein_coding	185	180	186	181	0.972
23527	NM_012287,XM_00	**ACAP2**	ArfGAP with coiled-coil, a	protein_coding	38	33	39	34	0.872
23528	NM_001281293,NM	**ZNF281**	zinc finger protein 281	protein_coding	24	21	25	22	0.888
23529	NM_001166212,NM	**CLCF1**	cardiotrophin like cytokin	protein_coding	0	1	1	2	1.349
23530	NM_001331026,NM	**NNT**	nicotinamide nucleotide t	protein_coding	39	36	40	37	0.930
23531	NM_012329,XM_00	**MMD**	monocyte to macrophage	protein_coding	5	5	6	6	1.010
23532	NM_001291715,NM	**PRAME**	preferentially expressed	protein_coding	0	0	1	1	1.000
23533	NM_001142633,NM	**PIK3R5**	phosphoinositide-3-kinas	protein_coding	0	0	1	1	1.000
23534	NM_001191028,NM	**TNPO3**	transportin 3	protein_coding	93	83	94	84	0.897
23536	NM_001324444,NM	**ADAT1**	adenosine deaminase tR	protein_coding	19	17	20	18	0.909
23538	NM_012375	**OR52A1**	olfactory receptor family	protein_coding	0	0	1	1	1.000
23539	NM_013356,XM_01	**SLC16A8**	solute carrier family 16 m	protein_coding	0	0	1	1	1.000
23540	rna-TRS-TGA4-1	**TRS-TGA4-1**	tRNA-Ser	tRNA	0	0	1	1	1.000
23541	NM_001204204,NM	**SEC14L2**	SEC14 like lipid binding	protein_coding	0	0	1	1	0.978
23542	NM_012324,XM_01	**MAPK8IP2**	mitogen-activated protein	protein_coding	1	1	2	2	1.208
23543	NM_001031695,NM	**RBFOX2**	RNA binding fox-1 homo	protein_coding	1	1	2	2	0.904
23544	NM_001184773,NM	**SEZ6L**	seizure related 6 homolo	protein_coding	0	0	1	1	1.022
23545	NM_012463,XM_02	**ATP6V0A2**	ATPase H+ transporting	protein_coding	28	26	29	27	0.936
23546	NM_012451,XM_00	**SYNGR4**	synaptogyrin 4	protein_coding	0	0	1	1	1.231
23547	NM_012276	**LILRA4**	leukocyte immunoglobul	protein_coding	0	0	1	1	1.000
23548	NM_012382,XM_01	**TTC33**	tetratricopeptide repeat c	protein_coding	13	10	14	11	0.771
23549	NM_001319116,NM	**DNPEP**	aspartyl aminopeptidase	protein_coding	60	58	61	59	0.963
23550	NM_012455,XM_00	**PSD4**	pleckstrin and Sec7 dom	protein_coding	114	102	115	103	0.895
23551	NM_001366725,NM	**RASD2**	RASD family member 2	protein_coding	0	0	1	1	1.000
23552	NM_001039803,NM	**CDK20**	cyclin dependent kinase	protein_coding	1	1	2	2	1.176
23553	NM_012269,XM_01	**HYAL4**	hyaluronidase 4	protein_coding	0	0	1	1	1.000
23554	NM_012338,XM_00	**TSPAN12**	tetraspanin 12	protein_coding	0	0	1	1	1.000
23555	NM_001351263,NM	**TSPAN15**	tetraspanin 15	protein_coding	1	0	2	1	0.880
23556	NM_012327,NM_17	**PIGN**	phosphatidylinositol glyc	protein_coding	8	7	9	8	0.907
23557	NM_012437,NR_05	**SNAPIN**	SNAP associated protein	protein_coding	61	72	62	73	1.166
23558	NM_001330499,NM	**WBP2**	WW domain binding prot	protein_coding	56	64	57	65	1.144
23559	NM_012477	**WBP1**	WW domain binding prot	protein_coding	28	30	29	31	1.060
23560	NM_012341,XM_02	**GTPBP4**	GTP binding protein 4	protein_coding	79	76	80	77	0.967
23562	NM_001146077,NM	**CLDN14**	claudin 14	protein_coding	0	0	1	1	1.000
23563	NM_024533	**CHST5**	carbohydrate sulfotransfe	protein_coding	0	0	1	1	1.000
23564	NM_001303007,NM	**DDAH2**	dimethylarginine dimethy	protein_coding	24	22	25	23	0.919
23566	NM_012152,XM_02	**LPAR3**	lysophosphatidic acid rec	protein_coding	11	12	12	13	1.075
23567	NM_001308213,NM	**ZNF346**	zinc finger protein 346	protein_coding	21	22	22	23	1.022
23568	NM_012106	**ARL2BP**	ADP ribosylation factor li	protein_coding	67	67	68	68	1.003
23569	NM_012387,XM_01	**PADI4**	peptidyl arginine deimina	protein_coding	0	0	1	1	1.000
23574	NR_026881	**PRG1**	p53-responsive gene 1	lncRNA	0	0	1	1	1.000
23576	NM_001134445,NM	**DDAH1**	dimethylarginine dimethy	protein_coding	0	0	1	1	0.959
23580	NM_012121,XM_00	**CDC42EP4**	CDC42 effector protein 4	protein_coding	2	1	3	2	0.823
23581	NM_012114,XM_01	**CASP14**	caspase 14	protein_coding	0	0	1	1	1.000
23582	NM_012142,NR_02	**CCNDBP1**	cyclin D1 binding protein	protein_coding	39	39	40	40	0.987
23583	NM_001243787,NM	**SMUG1**	single-strand-selective m	protein_coding	9	10	10	11	1.067
23584	NM_001329920,NM	**VSIG2**	V-set and immunoglobul	protein_coding	0	0	1	1	1.000
23585	NM_014313,XM_00	**TMEM50A**	transmembrane protein 5	protein_coding	102	97	103	98	0.952
23586	NM_014314	**DDX58**	DExD/H-box helicase 58	protein_coding	15	12	16	13	0.802
23587	NM_015362,NM_20	**ELP5**	elongator acetyltransfera	protein_coding	63	63	64	64	0.998
23588	NM_014315,XM_00	**KLHDC2**	kelch domain containing	protein_coding	7	7	8	8	1.033
23589	NM_001042476,NM	**CARHSP1**	calcium regulated heat s	protein_coding	61	64	62	65	1.060
23590	NM_001321978,NM	**PDSS1**	decaprenyl diphosphate	protein_coding	11	13	12	14	1.142
23591	NR_026770	**FAM215A**	family with sequence sim	lncRNA	0	0	1	1	1.000
23592	NM_001167614,NM	**LEMD3**	LEM domain containing 3	protein_coding	25	22	26	23	0.888
23593	NM_001326380,NM	**HEBP2**	heme binding protein 2	protein_coding	1	1	2	2	1.034
23594	NM_014321,NR_03	**ORC6**	origin recognition comple	protein_coding	92	90	93	91	0.976
23595	NM_001197259,NM	**ORC3**	origin recognition comple	protein_coding	40	38	41	39	0.940
23596	NM_001381855,NM	**OPN3**	opsin 3	protein_coding	15	11	16	12	0.742
23597	NM_001033583,NM	**ACOT9**	acyl-CoA thioesterase 9	protein_coding	16	14	17	15	0.885
23598	NM_014323,NM_03	**PATZ1**	POZ/BTB and AT hook c	protein_coding	17	19	18	20	1.092
23600	NM_001167595,NM	**AMACR**	alpha-methylacyl-CoA ra	protein_coding	6	8	7	9	1.184
23601	NM_001301167,NM	**CLEC5A**	C-type lectin domain con	protein_coding	0	0	1	1	1.000
23603	NM_001105237,NM	**CORO1C**	coronin 1C	protein_coding	26	25	27	26	0.970
23604	NM_001363730,NM	**DAPK2**	death associated protein	protein_coding	11	7	12	8	0.667
23605	gene-HMGN2P9	**HMGN2P9**	high mobility group nucle	pseudogene	0	0	1	1	1.000
23606	gene-HMGN2P10	**HMGN2P10**	high mobility group nucle	pseudogene	0	0	1	1	1.000
23607	NM_012120,XM_00	**CD2AP**	CD2 associated protein	protein_coding	70	55	71	56	0.785
23608	NM_001145125,NM	**MKRN1**	makorin ring finger prote	protein_coding	41	45	42	46	1.084
23609	NM_001271707,NM	**MKRN2**	makorin ring finger prote	protein_coding	32	29	33	30	0.912
23612	NM_012396,NR_07	**PHLDA3**	pleckstrin homology like	protein_coding	0	0	1	1	1.000
23613	NM_001281769,NM	**ZMYND8**	zinc finger MYND-type co	protein_coding	12	13	13	14	1.056
23614	NR_002181	**PPY2P**	pancreatic polypeptide 2	transcribed_pseu	0	0	1	1	1.000
23615	NR_003064	**PYY2**	peptide YY 2 (pseudogen	transcribed_pseu	1	0	2	1	0.715
23616	NM_001350055,NM	**SH3BP1**	SH3 domain binding prot	protein_coding	141	143	142	144	1.013
23617	NM_053006	**TSSK2**	testis specific serine kina	protein_coding	0	0	1	1	1.000
23619	NM_001146326,NM	**ZIM2**	zinc finger imprinted 2	protein_coding	0	0	1	1	1.000
23620	NM_012344,XM_00	**NTSR2**	neurotensin receptor 2	protein_coding	0	0	1	1	1.000
23621	NM_001207048,NM	**BACE1**	beta-secretase 1	protein_coding	0	0	1	1	1.000
23623	NM_001105203,NM	**RUSC1**	RUN and SH3 domain co	protein_coding	9	8	10	9	0.925
23624	NM_001130852,NM	**CBLC**	Cbl proto-oncogene C	protein_coding	0	0	1	1	1.000
23625	NM_001098784,NM	**FAM89B**	family with sequence sim	protein_coding	70	79	71	80	1.139
23626	NM_012444,NM_19	**SPO11**	SPO11 initiator of meiotic	protein_coding	0	0	1	1	1.000
23627	NM_012409	**PRND**	prion like protein doppel	protein_coding	0	0	1	1	1.000
23629	NR_002730	**BRD7P3**	bromodomain containing	transcribed_pseu	0	0	1	1	1.000
23630	NM_012282	**KCNE5**	potassium voltage-gated	protein_coding	1	1	2	2	0.935
23632	NM_012113,XM_00	**CA14**	carbonic anhydrase 14	protein_coding	0	0	1	1	1.000
23633	NM_012316,XM_00	**KPNA6**	karyopherin subunit alph	protein_coding	47	42	48	43	0.904
23635	NM_001256732,NM	**SSBP2**	single stranded DNA bin	protein_coding	0	0	1	1	1.000
23636	NM_001193357,NM	**NUP62**	nucleoporin 62	protein_coding	185	190	186	191	1.025
23637	NM_012197,XM_01	**RABGAP1**	RAB GTPase activating p	protein_coding	25	21	26	22	0.828
23639	NM_001321961,NM	**LRRC6**	leucine rich repeat conta	protein_coding	0	0	1	1	1.001
23640	NM_001130106,NM	**HSPBP1**	HSPA (Hsp70) binding p	protein_coding	73	80	74	81	1.105
23641	NM_012317	**LDOC1**	LDOC1 regulator of NFK	protein_coding	0	0	1	1	1.000
23642	NR_003098,NR_15	**SNHG1**	small nucleolar RNA hos	lncRNA	179	182	180	183	1.021
23643	NM_001195797,NM	**LY96**	lymphocyte antigen 96	protein_coding	0	0	1	1	1.000
23644	NM_014329	**EDC4**	enhancer of mRNA deca	protein_coding	67	70	68	71	1.047

23645	NM_014330	**PPP1R15A**	protein phosphatase 1 re	protein_coding	21	18	22	19	0.891
23646	NM_001031696,NM	**PLD3**	phospholipase D family r	protein_coding	20	26	21	27	1.283
23647	NM_001242854,NM	**ARFIP2**	ADP ribosylation factor in	protein_coding	94	99	95	100	1.059
23648	NM_001009955,NM	**SSBP3**	single stranded DNA bin	protein_coding	119	123	120	124	1.031
23649	NM_002689,XM_01	**POLA2**	DNA polymerase alpha 2	protein_coding	75	73	76	74	0.975
23650	NM_001330382,NM	**TRIM29**	tripartite motif containing	protein_coding	0	0	1	1	1.000
23654	NM_001376864,NM	**PLXNB2**	plexin B2	protein_coding	0	0	1	1	1.000
23657	NM_014331,XM_01	**SLC7A11**	solute carrier family 7 me	protein_coding	7	5	8	6	0.679
23658	NM_001130710,NM	**LSM5**	LSM5 homolog, U6 smal	protein_coding	65	60	66	61	0.938
23659	NM_001363551,NM	**PLA2G15**	phospholipase A2 group	protein_coding	5	5	6	6	0.938
23660	NM_001318082,NM	**ZKSCAN5**	zinc finger with KRAB an	protein_coding	21	21	22	22	1.014
23666	NR_144546	**UBBP4**	ubiquitin B pseudogene	transcribed_pseu	1	1	2	2	1.023
23667	gene-UBBP3	**UBBP3**	ubiquitin B pseudogene	pseudogene	0	0	1	1	1.000
23668	gene-UBBP2	**UBBP2**	ubiquitin B pseudogene	pseudogene	0	0	1	1	1.000
23670	NM_001135820,NM	**CEMIP2**	cell migration inducing h	protein_coding	72	64	73	65	0.892
23671	NM_001305134,NM	**TMEFF2**	transmembrane protein v	protein_coding	0	0	1	1	1.000
23673	NM_177424	**STX12**	syntaxin 12	protein_coding	25	22	26	23	0.878
23676	NM_014332,NR_04	**SMPX**	small muscle protein X-li	protein_coding	0	0	1	1	1.000
23677	NM_001371302,NM	**SH3BP4**	SH3 domain binding prot	protein_coding	0	0	1	1	1.000
23678	NM_001033578,NM	**SGK3**	serum/glucocorticoid reg	protein_coding	2	2	3	3	0.855
23682	NM_022337,XM_01	**RAB38**	RAB38, member RAS on	protein_coding	3	4	4	5	1.096
23683	NM_005813,XM_00	**PRKD3**	protein kinase D3	protein_coding	51	41	52	42	0.803
23704	NM_080671	**KCNE4**	potassium voltage-gated	protein_coding	0	0	1	1	1.056
23705	NM_001098517,NM	**CADM1**	cell adhesion molecule 1	protein_coding	4	4	5	5	1.128
23708	NM_018094	**GSPT2**	G1 to S phase transition	protein_coding	0	0	1	1	0.997
23710	NM_001363598,NM	**GABARAPL1**	GABA type A receptor as	protein_coding	20	19	21	20	0.948
23729	NM_013276	**SHPK**	sedoheptulokinase	protein_coding	11	10	12	11	0.930
23731	NM_032012,XM_01	**TMEM245**	transmembrane protein 2	protein_coding	28	23	29	24	0.840
23732	NM_014334,XM_01	**FRRS1L**	ferric chelate reductase 1	protein_coding	0	0	1	1	1.000
23738	gene-OFD1P17	**OFD1P17**	OFD1 pseudogene 17	pseudogene	0	0	1	1	1.028
23741	NM_014335	**EID1**	EP300 interacting inhibit	protein_coding	362	336	363	337	0.930
23742	NM_018958	**NPAP1**	nuclear pore associated	protein_coding	0	0	1	1	1.000
23743	NM_001178005,NM	**BHMT2**	betaine--homocysteine S	protein_coding	0	0	1	1	1.000
23746	NM_001033054,NM	**AIPL1**	aryl hydrocarbon recepto	protein_coding	0	0	1	1	1.000
23752	gene-TSSK1A	**TSSK1A**	testis specific serine kina	pseudogene	0	0	1	1	0.914
23753	NM_022044	**SDF2L1**	stromal cell derived facto	protein_coding	119	136	120	137	1.142
23754	gene-RPL32P5	**RPL32P5**	ribosomal protein L32 ps	pseudogene	0	0	1	1	1.000
23755	gene-SIRPAP1	**SIRPAP1**	signal regulatory protein	pseudogene	0	0	1	1	1.000
23757	gene-ARSFP1	**ARSFP1**	arylsulfatase F pseudoge	pseudogene	0	0	1	1	1.000
23758	gene-SFPQP1	**SFPQP1**	splicing factor proline an	pseudogene	0	0	1	1	1.000
23759	NM_001317996,NM	**PPIL2**	peptidylprolyl isomerase	protein_coding	38	37	39	38	0.996
23760	NM_001284277,NM	**PITPNB**	phosphatidylinositol tran	protein_coding	109	94	110	95	0.868
23761	NM_001326411,NM	**PISD**	phosphatidylserine deca	protein_coding	30	31	31	32	1.040
23762	NM_001282738,NM	**OSBP2**	oxysterol binding protein	protein_coding	0	0	1	1	1.000
23764	NM_001161572,NM	**MAFF**	MAF bZIP transcription fa	protein_coding	1	1	2	2	0.894
23765	NM_001289905,NM	**IL17RA**	interleukin 17 receptor A	protein_coding	13	13	14	14	0.986
23766	NR_028287	**GABARAPL3**	GABA type A receptor as	transcribed_pseu	0	0	1	1	1.000
23767	NM_013281,NM_19	**FLRT3**	fibronectin leucine rich tr	protein_coding	0	0	1	1	0.927
23768	NM_001346143,NM	**FLRT2**	fibronectin leucine rich tr	protein_coding	0	0	1	1	1.000
23769	NM_013280,XM_00	**FLRT1**	fibronectin leucine rich tr	protein_coding	0	0	1	1	1.000
23770	NM_001308373,NM	**FKBP8**	FKBP prolyl isomerase 8	protein_coding	157	171	158	172	1.093
23774	NM_001304808,NM	**BRD1**	bromodomain containing	protein_coding	30	30	31	31	1.018
23779	NM_001017526,NM	**ARHGAP8**	Rho GTPase activating p	protein_coding	0	0	1	1	1.000
23780	NM_030882,NM_14	**APOL2**	apolipoprotein L2	protein_coding	76	78	77	79	1.023
23782	NR_040114	**AP1B1P1**	AP1B1 pseudogene 1	transcribed_pseu	0	0	1	1	1.000
23783	NR_040115	**ANKRD62P1-PARP4P3**	ANKRD62P1-PARP4P3 r	transcribed_pseu	1	1	2	2	0.749
23784	NM_001136213	**POTEH**	POTE ankyrin domain fa	protein_coding	0	0	1	1	1.000
23786	NM_001270726,NM	**BCL2L13**	BCL2 like 13	protein_coding	41	40	42	41	0.990
23787	NM_001271641,NM	**MTCH1**	mitochondrial carrier 1	protein_coding	139	134	140	135	0.966
23788	NM_001317231,NM	**MTCH2**	mitochondrial carrier 2	protein_coding	155	155	156	156	0.999
24137	NM_012310	**KIF4A**	kinesin family member 4A	protein_coding	55	49	56	50	0.899
24138	NM_012420	**IFIT5**	interferon induced protei	protein_coding	14	10	15	11	0.765
24139	NM_001193268,NM	**EML2**	EMAP like 2	protein_coding	50	49	51	50	0.989
24140	NM_001282157,NM	**FTSJ1**	FtsJ RNA 2'-O-methyltra	protein_coding	116	109	117	110	0.942
24141	NM_001199897,NM	**LAMP5**	lysosomal associated me	protein_coding	0	0	1	1	1.000
24142	NM_001200016,NM	**NAA80**	N-alpha-acetyltransferas	protein_coding	6	6	7	7	1.106
24144	NM_001008697,NM	**TFIP11**	tuftelin interacting protei	protein_coding	45	47	46	48	1.040
24145	NM_015368,XM_01	**PANX1**	pannexin 1	protein_coding	25	23	26	24	0.913
24146	NM_001185080,NM	**CLDN15**	claudin 15	protein_coding	12	14	13	15	1.149
24147	NM_014344	**FJX1**	four-jointed box kinase 1	protein_coding	0	0	1	1	1.000
24148	NM_012469,XM_00	**PRPF6**	pre-mRNA processing fa	protein_coding	124	126	125	127	1.013
24149	NM_014345	**ZNF318**	zinc finger protein 318	protein_coding	18	16	19	17	0.855
24150	NM_016212,NR_11	**TP53TG3**	TP53 target 3	protein_coding	0	0	1	1	1.000
25758	NM_012194,XM_00	**KIAA1549L**	KIAA1549 like	protein_coding	0	0	1	1	1.000
25759	NM_012435,XM_01	**SHC2**	SHC adaptor protein 2	protein_coding	0	0	1	1	1.000
25763	NM_012274	**H2AP**	H2A.P histone	protein_coding	0	0	1	1	1.000
25764	NM_001199885,NM	**HYPK**	huntingtin interacting pro	protein_coding	86	103	87	104	1.194
25766	NM_001031698,NM	**PRPF40B**	pre-mRNA processing fa	protein_coding	1	0	2	1	0.858
25769	NM_001193288,NM	**SLC24A2**	solute carrier family 24 m	protein_coding	0	0	1	1	1.019
25770	NM_015370,XM_00	**C22orf31**	chromosome 22 open rea	protein_coding	0	0	1	1	1.000
25771	NM_001284303,NM	**TBC1D22A**	TBC1 domain family mem	protein_coding	12	12	13	13	1.069
25774	NM_001358664,NR	**GSTT4**	glutathione S-transferase	protein_coding	0	0	1	1	1.000
25775	NM_001302819,NM	**C22orf24**	chromosome 22 open rea	protein_coding	3	2	4	3	0.896
25776	NM_001002880,NM	**CBY1**	chibby family member 1,	protein_coding	6	6	7	7	1.029
25777	NM_001199579,NM	**SUN2**	Sad1 and UNC84 domai	protein_coding	82	84	83	85	1.017
25778	NM_015375,NM_19	**DSTYK**	dual serine/threonine an	protein_coding	4	4	5	5	1.005
25780	NM_001139488,NM	**RASGRP3**	RAS guanyl releasing pr	protein_coding	11	10	12	11	0.913
25782	NM_012414	**RAB3GAP2**	RAB3 GTPase activating	protein_coding	21	18	22	19	0.886
25786	NR_024157	**DGCR11**	DiGeorge syndrome critic	lncRNA	1	1	2	2	1.182
25788	NM_001205262,NM	**RAD54B**	RAD54 homolog B	protein_coding	14	12	15	13	0.816
25789	NM_012109,XR_00	**TMEM59L**	transmembrane protein 5	protein_coding	0	0	1	1	1.000
25790	NM_012337	**CFAP45**	cilia and flagella associa	protein_coding	2	1	3	2	0.987
25791	NM_001114090,NM	**NGEF**	neuronal guanine nucleo	protein_coding	0	0	1	1	1.000
25792	NM_001131015,NM	**CIZ1**	CDKN1A interacting zinc	protein_coding	96	97	97	98	1.014
25793	NM_001033024,NM	**FBXO7**	F-box protein 7	protein_coding	117	118	118	119	1.010
25794	NM_001077182,NM	**FSCN2**	fascin actin-bundling pro	protein_coding	1	0	2	1	0.892
25796	NM_012088,XM_01	**PGLS**	6-phosphogluconolacton	protein_coding	52	62	53	63	1.187
25797	NM_012413	**QPCT**	glutaminyl-peptide cyclo	protein_coding	0	0	1	1	1.000
25798	NM_001159491,NM	**BRI3**	brain protein I3	protein_coding	25	28	26	29	1.112
25799	NM_014347,XM_00	**ZNF324**	zinc finger protein 324	protein_coding	10	11	11	12	1.128
25800	NM_001099406,NM	**SLC39A6**	solute carrier family 39 m	protein_coding	77	67	78	68	0.873
25801	NM_001330265,NM	**GCA**	grancalcin	protein_coding	6	6	7	7	1.001
25802	NM_012134	**LMOD1**	leiomodin 1	protein_coding	0	0	1	1	1.000
25803	NM_001252294,NM	**SPDEF**	SAM pointed domain con	protein_coding	0	0	1	1	1.029
25804	NM_001252129,NM	**LSM4**	LSM4 homolog, U6 smal	protein_coding	141	153	142	154	1.085
25805	NM_012342	**BAMBI**	BMP and activin membra	protein_coding	0	0	1	1	1.000
25806	NM_012476,XM_00	**VAX2**	ventral anterior homeobo	protein_coding	0	0	1	1	1.000
25807	NM_001329536,NM	**RHBDD3**	rhomboid domain contai	protein_coding	35	43	36	44	1.226
25809	NM_012263,NR_02	**TTLL1**	tubulin tyrosine ligase lik	protein_coding	1	1	2	2	0.822
25812	NR_024591	**POM121L1P**	POM121 transmembrane	transcribed_pseu	0	0	1	1	1.000
25813	NM_015380	**SAMM50**	SAMM50 sorting and ass	protein_coding	76	74	77	75	0.969
25814	NM_001167621,NM	**ATXN10**	ataxin 10	protein_coding	81	82	82	83	1.015
25816	NM_001077654,NM	**TNFAIP8**	TNF alpha induced prote	protein_coding	52	44	53	45	0.852
25817	NM_001082967,NM	**TAFA5**	TAFA chemokine like fam	protein_coding	0	0	1	1	1.000
25818	NM_001077491,NM	**KLK5**	kallikrein related peptida	protein_coding	0	0	1	1	1.000
25819	NM_012118	**NOCT**	nocturnin	protein_coding	9	10	10	11	1.114
25820	NM_005744	**ARIH1**	ariadne RBR E3 ubiquitin	protein_coding	13	13	14	14	1.008
25821	NM_001123226,NM	**MTO1**	mitochondrial tRNA trans	protein_coding	13	11	14	12	0.874
25822	NM_001135004,NM	**DNAJB5**	DnaJ heat shock protein	protein_coding	5	5	6	6	0.971
25823	NM_012467,XM_01	**TPSG1**	tryptase gamma 1	protein_coding	0	0	1	1	1.000
25824	NM_001358511,NM	**PRDX5**	peroxiredoxin 5	protein_coding	472	515	473	516	1.091
25825	NM_012105,NM_13	**BACE2**	beta-secretase 2	protein_coding	0	0	1	1	1.000
25826	NR_004398	**SNORD82**	small nucleolar RNA, C/I	snoRNA	1	3	2	4	1.562
25827	NM_001349316,NM	**FBXL2**	F-box and leucine rich re	protein_coding	0	0	1	1	1.000
25828	NM_012473,XM_00	**TXN2**	thioredoxin 2	protein_coding	138	147	139	148	1.059
25829	NM_001195071,NM	**TMEM184B**	transmembrane protein 1	protein_coding	21	19	22	20	0.926

25830	NM_014351,XM_01	**SULT4A1**	sulfotransferase family 4	protein_coding	0	0	1	1	1.000
25831	NM_015382,XM_00	**HECTD1**	HECT domain E3 ubiquit	protein_coding	30	26	31	27	0.871
25832	NM_015383	**NBPF14**	NBPF member 14	protein_coding	7	6	8	7	0.857
25833	NM_001244682,NM	**POU2F3**	POU class 2 homeobox 3	protein_coding	0	0	1	1	1.210
25834	NM_001351282,NM	**MGAT4C**	MGAT4 family member C	protein_coding	0	0	1	1	1.000
25836	NM_015384,NM_13	**NIPBL**	NIPBL cohesin loading fa	protein_coding	30	23	31	24	0.786
25837	NM_001308053,NM	**RAB26**	RAB26, member RAS on	protein_coding	0	0	1	1	1.000
25839	NM_001195139,NM	**COG4**	component of oligomeric	protein_coding	31	37	32	38	1.187
25840	NM_014033	**METTL7A**	methyltransferase like 7A	protein_coding	1	1	2	2	1.057
25841	NM_145804	**ABTB2**	ankyrin repeat and BTB (	protein_coding	2	3	3	4	1.327
25842	NM_014034	**ASF1A**	anti-silencing function 1A	protein_coding	36	36	37	37	0.981
25843	NM_001100819,NM	**MOB4**	MOB family member 4, p	protein_coding	71	66	72	67	0.921
25844	NM_015388	**YIPF3**	Yip1 domain family mem	protein_coding	70	70	71	71	1.005
25845	NR_024158	**PP7080**	uncharacterized LOC258	lncRNA	40	43	41	44	1.091
25847	NM_001242374,NM	**ANAPC13**	anaphase promoting con	protein_coding	36	37	37	38	1.022
25849	NM_015393,XM_01	**PARM1**	prostate androgen-regula	protein_coding	0	0	1	1	1.000
25850	NM_001242472,NM	**ZNF345**	zinc finger protein 345	protein_coding	0	0	1	1	1.000
25851	NM_015395,XM_00	**TECPR1**	tectonin beta-propeller re	protein_coding	5	5	6	6	0.984
25852	NM_001267041,NM	**ARMC8**	armadillo repeat containi	protein_coding	32	33	33	34	1.024
25853	NM_015397,XM_01	**DCAF12**	DDB1 and CUL4 associa	protein_coding	85	76	86	77	0.898
25854	NM_001006655,NM	**FAM149A**	family with sequence sim	protein_coding	0	0	1	1	1.037
25855	NM_001024957,NM	**BRMS1**	BRMS1 transcriptional re	protein_coding	155	165	156	166	1.066
25858	NM_001039496	**CATSPERZ**	catsper channel auxiliary	protein_coding	0	0	1	1	1.000
25859	NR_024617,NR_02	**PART1**	prostate androgen-regula	lncRNA	0	0	1	1	1.000
25861	NM_001083885,NM	**WHRN**	whirlin	protein_coding	0	0	1	1	1.000
25862	NM_001286554,NM	**USP49**	ubiquitin specific peptida	protein_coding	12	11	13	12	0.910
25864	NM_015407	**ABHD14A**	abhydrolase domain con	protein_coding	1	2	2	3	1.588
25865	NM_001079880,NM	**PRKD2**	protein kinase D2	protein_coding	62	59	63	60	0.957
25870	NM_001042468,NM	**SUMF2**	sulfatase modifying facto	protein_coding	54	56	55	57	1.029
25871	NM_001319109,NM	**NEPRO**	nucleolus and neural pro	protein_coding	61	55	62	56	0.897
25873	NM_015414,NM_03	**RPL36**	ribosomal protein L36	protein_coding	1,517	1,655	1,518	1,656	1.091
25874	NM_001143674,NM	**MPC2**	mitochondrial pyruvate c	protein_coding	53	57	54	58	1.082
25875	NM_001243689,NM	**LETMD1**	LETM1 domain containin	protein_coding	46	44	47	45	0.942
25876	NM_015417	**SPEF1**	sperm flagellar 1	protein_coding	0	0	1	1	1.000
25878	NM_015419	**MXRA5**	matrix remodeling associ	protein_coding	0	0	1	1	1.000
25879	NM_015420	**DCAF13**	DDB1 and CUL4 associa	protein_coding	195	181	196	182	0.932
25880	NM_015421	**TMEM186**	transmembrane protein 1	protein_coding	25	23	26	24	0.906
25884	NM_001278473,NM	**CHRDL2**	chordin like 2	protein_coding	0	0	1	1	1.000
25885	NM_015425	**POLR1A**	RNA polymerase I subur	protein_coding	54	51	55	52	0.941
25886	NM_001161580,NM	**POC1A**	POC1 centriolar protein /	protein_coding	29	28	30	29	0.951
25888	NM_001006656,NM	**ZNF473**	zinc finger protein 473	protein_coding	24	23	25	24	0.968
25890	NM_001349329,NM	**ABI3BP**	ABI family member 3 bin	protein_coding	0	0	1	1	0.942
25891	NM_001001991,NM	**PAMR1**	peptidase domain contai	protein_coding	0	0	1	1	1.000
25893	NM_015431	**TRIM58**	tripartite motif containing	protein_coding	0	0	1	1	1.000
25894	NM_001129727,NM	**PLEKHG4**	pleckstrin homology and	protein_coding	39	40	40	41	1.018
25895	NM_015433,NM_20	**EEF1AKMT3**	EEF1A lysine methyltran	protein_coding	4	4	5	5	1.010
25896	NM_001199809,NM	**INTS7**	integrator complex subur	protein_coding	31	27	32	28	0.876
25897	NM_001280539,NM	**RNF19A**	ring finger protein 19A, F	protein_coding	22	21	23	22	0.965
25898	NM_001009922,NM	**RCHY1**	ring finger and CHY zinc	protein_coding	31	31	32	32	0.978
25900	NM_001039670,NM	**IFFO1**	intermediate filament fan	protein_coding	6	6	7	7	0.999
25901	NM_001379071,NM	**CCDC28A**	coiled-coil domain contai	protein_coding	17	17	18	18	1.001
25902	NM_001242767,NM	**MTHFD1L**	methylenetetrahydrofolat	protein_coding	60	52	61	53	0.869
25903	NM_001297713,NM	**OLFML2B**	olfactomedin like 2B	protein_coding	0	0	1	1	1.000
25904	NM_001256741,NM	**CNOT10**	CCR4-NOT transcription	protein_coding	21	19	22	20	0.912
25906	NM_001278485,NM	**ANAPC15**	anaphase promoting con	protein_coding	80	87	81	88	1.081
25907	NM_015444	**TMEM158**	transmembrane protein 1	protein_coding	0	0	1	1	1.000
25909	NM_001323342,NM	**AHCTF1**	AT-hook containing trans	protein_coding	36	30	37	31	0.829
25911	NM_001329742,NM	**DPCD**	deleted in primary ciliary	protein_coding	12	11	13	12	0.885
25912	NM_001098616,NM	**C1orf43**	chromosome 1 open reac	protein_coding	257	268	258	269	1.040
25913	NM_001042594,NM	**POT1**	protection of telomeres 1	protein_coding	23	21	24	22	0.929
25914	NM_001318520,NM	**RTTN**	rotatin	protein_coding	8	7	9	8	0.867
25915	NM_199069,NM_19	**NDUFAF3**	NADH:ubiquinone oxidor	protein_coding	58	59	59	60	1.009
25917	NM_001114092,NM	**THUMPD3**	THUMP domain containi	protein_coding	33	32	34	33	0.954
25920	NM_015456	**NELFB**	negative elongation facto	protein_coding	55	52	56	53	0.952
25921	NM_015457,XM_01	**ZDHHC5**	zinc finger DHHC-type pa	protein_coding	93	86	94	87	0.924
25923	NM_001290048,NM	**ATL3**	atlastin GTPase 3	protein_coding	30	25	31	26	0.843
25924	NM_001284423,NM	**MYRIP**	myosin VIIA and Rab inte	protein_coding	0	0	1	1	1.000
25925	NM_001308225,NM	**ZNF521**	zinc finger protein 521	protein_coding	0	0	1	1	1.000
25926	NM_001303272,NM	**NOL11**	nucleolar protein 11	protein_coding	168	151	169	152	0.900
25927	NM_001111101,NM	**CNRIP1**	cannabinoid receptor inte	protein_coding	0	0	1	1	1.000
25928	NM_015464	**SOSTDC1**	sclerostin domain contai	protein_coding	0	0	1	1	1.000
25929	NM_001252156,NM	**GEMIN5**	gem nuclear organelle a	protein_coding	77	72	78	73	0.940
25930	NM_001304482,NM	**PTPN23**	protein tyrosine phospha	protein_coding	11	11	12	12	0.997
25932	NM_013943	**CLIC4**	chloride intracellular cha	protein_coding	0	0	1	1	1.000
25934	NM_001329570,NM	**NIPSNAP3A**	nipsnap homolog 3A	protein_coding	28	25	29	26	0.899
25936	NM_001042549,NM	**NSL1**	NSL1 component of MIS	protein_coding	8	8	9	9	1.000
25937	NM_001168278,NM	**WWTR1**	WW domain containing t	protein_coding	0	0	1	1	0.994
25938	NM_015473	**HEATR5A**	HEAT repeat containing	protein_coding	5	4	6	5	0.892
25939	NM_001363729,NM	**SAMHD1**	SAM and HD domain cor	protein_coding	189	184	190	185	0.974
25940	NM_001304538,NM	**FAM98A**	family with sequence sim	protein_coding	63	60	64	61	0.950
25941	NM_001271949,NM	**TPGS2**	tubulin polyglutamylase	protein_coding	69	68	70	69	0.992
25942	NM_001145357,NM	**SIN3A**	SIN3 transcription regula	protein_coding	46	44	47	45	0.969
25943	NM_001009984,XM	**C20orf194**	chromosome 20 open rea	protein_coding	0	0	1	1	1.000
25945	NM_001243286,NM	**NECTIN3**	nectin cell adhesion mole	protein_coding	10	7	11	8	0.704
25946	NM_001130967,NM	**ZNF385A**	zinc finger protein 385A	protein_coding	0	0	1	1	1.038
25948	NM_015483,XM_00	**KBTBD2**	kelch repeat and BTB do	protein_coding	18	14	19	15	0.797
25949	NM_015484,NM_20	**SYF2**	SYF2 pre-mRNA splicing	protein_coding	61	65	62	66	1.065
25950	NM_001128142,NM	**RWDD3**	RWD domain containing	protein_coding	18	16	19	17	0.878
25953	NM_001077399,NM	**PNKD**	PNKD metallo-beta-lacta	protein_coding	135	133	136	134	0.989
25956	NM_015490	**SEC31B**	SEC31 homolog B, COP	protein_coding	32	30	33	31	0.929
25957	NM_001322405,NM	**PNISR**	PNN interacting serine a	protein_coding	37	31	38	32	0.838
25959	NM_001136191,NM	**KANK2**	KN motif and ankyrin rep	protein_coding	0	0	1	1	1.000
25960	NM_032777,XM_01	**ADGRA2**	adhesion G protein-coup	protein_coding	0	0	1	1	1.004
25961	NM_001283014,NM	**NUDT13**	nudix hydrolase 13	protein_coding	0	0	1	1	1.000
25962	NM_015496,NM_18	**VIRMA**	vir like m6A methyltransf	protein_coding	58	51	59	52	0.874
25963	NM_001110503,NM	**TMEM87A**	transmembrane protein 8	protein_coding	84	79	85	80	0.949
25966	NM_015500,NM_19	**C2CD2**	C2 calcium dependent d	protein_coding	2	1	3	2	0.827
25970	NM_001145795,NM	**SH2B1**	SH2B adaptor protein 1	protein_coding	20	23	21	24	1.162
25972	NM_001330353,NM	**UNC50**	unc-50 inner nuclear me	protein_coding	66	66	67	67	0.997
25973	NM_152268	**PARS2**	prolyl-tRNA synthetase 2	protein_coding	9	9	10	10	0.990
25974	NM_001330540,NM	**MMACHC**	metabolism of cobalamin	protein_coding	17	18	18	19	1.010
25975	NM_001167890,NM	**EGFL6**	EGF like domain multiple	protein_coding	0	0	1	1	0.980
25976	NM_001184717,NM	**TIPARP**	TCDD inducible poly(ADI	protein_coding	11	10	12	11	0.944
25977	NM_015509,NR_02	**NECAP1**	NECAP endocytosis ass	protein_coding	15	15	16	16	1.025
25978	NM_001244644,NM	**CHMP2B**	charged multivesicular b	protein_coding	55	50	56	51	0.911
25979	NM_001330159,NM	**DHRS7B**	dehydrogenase/reductas	protein_coding	7	7	8	8	0.974
25980	NM_001271874,NM	**AAR2**	AAR2 splicing factor	protein_coding	78	78	79	79	1.007
25981	NM_015512,XM_01	**DNAH1**	dynein axonemal heavy c	protein_coding	0	0	1	1	0.961
25983	NM_001042635,NM	**NGDN**	neuroguidin	protein_coding	71	72	72	73	1.012
25984	NM_001282433,NM	**KRT23**	keratin 23	protein_coding	0	0	1	1	1.000
25987	NM_001258210,NM	**TSKU**	tsukushi, small leucine ri	protein_coding	0	0	1	1	1.000
25988	NM_001243259,NM	**HINFP**	histone H4 transcription f	protein_coding	15	18	16	19	1.168
25989	NM_001099436,NM	**ULK3**	unc-51 like kinase 3	protein_coding	31	30	32	31	0.982
25992	NM_001080437,XM	**SNED1**	sushi, nidogen and EGF	protein_coding	0	0	1	1	0.991
25994	NM_001099668,NM	**HIGD1A**	HIG1 hypoxia inducible c	protein_coding	192	191	193	192	0.998
25996	NM_015523	**REXO2**	RNA exonuclease 2	protein_coding	118	125	119	126	1.056
25998	NM_001300906,NM	**IBTK**	inhibitor of Bruton tyrosir	protein_coding	38	32	39	33	0.847
25999	NM_001199570,NM	**CLIP3**	CAP-Gly domain contain	protein_coding	0	0	1	1	0.996
26000	NM_015527,XM_01	**TBC1D10B**	TBC1 domain family mer	protein_coding	69	69	70	70	1.003
26001	NM_001320356,NM	**RNF167**	ring finger protein 167	protein_coding	61	63	62	64	1.041
26002	NM_015529,XM_01	**MOXD1**	monooxygenase DBH lik	protein_coding	0	0	1	1	1.000
26003	NM_001201428,NM	**GORASP2**	golgi reassembly stackin	protein_coding	167	167	168	168	0.997
26005	NM_001286577,NM	**C2CD3**	C2 domain containing 3 (	protein_coding	22	20	23	21	0.902
26007	NM_001351976,NM	**TKFC**	triokinase and FMN cycla	protein_coding	10	13	11	14	1.328
26009	NM_001308237,NM	**ZZZ3**	zinc finger ZZ-type conta	protein_coding	24	21	25	22	0.877
26010	NM_001100422,NM	**SPATS2L**	spermatogenesis associa	protein_coding	12	13	13	14	1.084

26011	NM_001098816,XM	**TENM4**	teneurin transmembrane	protein_coding	0	0	1	1	1.000
26012	NM_001130969,NM	**NSMF**	NMDA receptor synaptor	protein_coding	20	20	21	21	1.005
26013	NM_001377303,NM	**L3MBTL1**	L3MBTL histone methyl-l	protein_coding	1	1	2	2	0.832
26015	NM_015540,XM_00	**RPAP1**	RNA polymerase II assoc	protein_coding	30	31	31	32	1.014
26017	NM_014077,XM_01	**FAM32A**	family with sequence sin	protein_coding	157	158	158	159	1.006
26018	NM_001377344,NM	**LRIG1**	leucine rich repeats and	protein_coding	35	34	36	35	0.971
26019	NM_015542,NM_08	**UPF2**	UPF2 regulator of nonse	protein_coding	15	13	16	14	0.847
26020	NM_001329226,NM	**LRP10**	LDL receptor related pro	protein_coding	17	15	18	16	0.886
26022	NM_001033504,NM	**TMEM98**	transmembrane protein 9	protein_coding	11	9	12	10	0.810
26024	NM_015545	**PTCD1**	pentatricopeptide repeat	protein_coding	20	17	21	18	0.860
26025	NM_003735,NM_03	**PCDHGA12**	protocadherin gamma su	protein_coding	0	0	1	1	0.966
26027	NM_015547,NM_14	**ACOT11**	acyl-CoA thioesterase 11	protein_coding	0	0	1	1	1.000
26030	NM_001308147,XM	**PLEKHG3**	pleckstrin homology and	protein_coding	0	0	1	1	1.000
26031	NM_015550,NM_14	**OSBPL3**	oxysterol binding protein	protein_coding	35	28	36	29	0.826
26032	NM_015551,XM_00	**SUSD5**	sushi domain containing	protein_coding	0	0	1	1	1.000
26033	NM_001276282,NM	**ATRNL1**	attractin like 1	protein_coding	0	0	1	1	1.000
26034	NM_001130699,NM	**IPCEF1**	interaction protein for cyt	protein_coding	38	31	39	32	0.828
26035	NM_001324091,NM	**GLCE**	glucuronic acid epimeras	protein_coding	10	9	11	10	0.926
26036	NM_001031623,NM	**ZNF451**	zinc finger protein 451	protein_coding	22	20	23	21	0.890
26037	NM_001284245,NM	**SIPA1L1**	signal induced proliferati	protein_coding	14	12	15	13	0.894
26038	NM_015557	**CHD5**	chromodomain helicase	protein_coding	0	0	1	1	0.936
26039	NM_001301778,NM	**SS18L1**	SS18L1 subunit of BAF	protein_coding	18	16	19	17	0.893
26040	NM_001130110,NM	**SETBP1**	SET binding protein 1	protein_coding	5	5	6	6	0.944
26043	NM_015562,XM_01	**UBXN7**	UBX domain protein 7	protein_coding	15	13	16	14	0.858
26045	NM_015564	**LRRTM2**	leucine rich repeat trans	protein_coding	0	0	1	1	1.000
26046	NM_001320766,NM	**LTN1**	listerin E3 ubiquitin prote	protein_coding	27	21	28	22	0.794
26047	NM_014141,XM_01	**CNTNAP2**	contactin associated prot	protein_coding	0	0	1	1	1.000
26048	NM_001303450,NM	**ZNF500**	zinc finger protein 500	protein_coding	3	2	4	3	0.939
26049	NM_001376049,NM	**FAM169A**	family with sequence sin	protein_coding	6	5	7	6	0.797
26050	NM_015567,XM_00	**SLITRK5**	SLIT and NTRK like fami	protein_coding	0	0	1	1	1.000
26051	NM_001172735,NM	**PPP1R16B**	protein phosphatase 1 re	protein_coding	293	216	294	217	0.739
26052	NM_001136127,NM	**DNM3**	dynamin 3	protein_coding	0	0	1	1	0.925
26053	NM_001127231,NM	**AUTS2**	activator of transcription	protein_coding	1	1	2	2	0.936
26054	NM_001100409,NM	**SENP6**	SUMO specific peptidase	protein_coding	41	36	42	37	0.900
26056	NM_001371272,NM	**RAB11FIP5**	RAB11 family interacting	protein_coding	0	0	1	1	1.000
26057	NM_001286771,NM	**ANKRD17**	ankyrin repeat domain 1	protein_coding	54	47	55	48	0.875
26058	NM_001103146,NM	**GIGYF2**	GRB10 interacting GYF	protein_coding	53	47	54	48	0.895
26059	NM_015576,NR_13	**ERC2**	ELKS/RAB6-interacting/	protein_coding	0	0	1	1	1.000
26060	NM_012096,XM_01	**APPL1**	adaptor protein, phospho	protein_coding	30	26	31	27	0.853
26061	NM_001284413,NM	**HACL1**	2-hydroxyacyl-CoA lyase	protein_coding	17	16	18	17	0.955
26062	NR_002731	**HYAL6P**	hyaluronidase 6, pseudo	transcribed_pseu	0	0	1	1	1.000
26063	NM_020664	**DECR2**	2,4-dienoyl-CoA reducta	protein_coding	7	10	8	11	1.348
26064	NM_001145520,NM	**RAI14**	retinoic acid induced 14	protein_coding	0	0	1	1	1.000
26065	NM_001114093,NM	**LSM14A**	LSM14A mRNA processi	protein_coding	216	202	217	203	0.936
26070	NR_026882	**MYRF-AS1**	MYRF antisense RNA 1	lncRNA	1	1	2	2	1.058
26071	NM_001078172,NM	**RTL8A**	retrotransposon Gag like	protein_coding	25	26	26	27	1.035
26073	NM_001290145,NM	**POLDIP2**	DNA polymerase delta in	protein_coding	148	157	149	158	1.055
26074	NM_001167816,NM	**CFAP61**	cilia and flagella associa	protein_coding	0	0	1	1	0.955
26077	NR_026889	**DKFZP434H168**	uncharacterized LOC26	lncRNA	0	0	1	1	1.000
26080	NR_027278	**FAM230C**	family with sequence sin	lncRNA	0	0	1	1	1.000
26082	NR_026771	**LINC02249**	long intergenic non-prote	lncRNA	0	0	1	1	1.000
26083	XM_011524596	**TBC1D29P**	TBC1 domain family mer	protein_coding	0	0	1	1	1.000
26084	NM_001251962,NM	**ARHGEF26**	Rho guanine nucleotide	protein_coding	0	0	1	1	1.000
26085	NM_001348177,NM	**KLK13**	kallikrein related peptida	protein_coding	0	0	1	1	1.000
26086	NM_001145638,NM	**GPSM1**	G protein signaling modu	protein_coding	0	0	1	1	1.000
26088	NM_001001560,NM	**GGA1**	golgi associated, gamma	protein_coding	28	31	29	32	1.097
26090	NM_001042472,NM	**ABHD12**	abhydrolase domain con	protein_coding	36	41	37	42	1.138
26091	NM_001278185,NM	**HERC4**	HECT and RLD domain	protein_coding	24	19	25	20	0.803
26092	NM_001267578,NM	**TOR1AIP1**	torsin 1A interacting prot	protein_coding	78	69	79	70	0.880
26093	NM_015603,XM_01	**CCDC9**	coiled-coil domain contai	protein_coding	11	12	12	13	1.076
26094	NM_001163508,NM	**DCAF4**	DDB1 and CUL4 associa	protein_coding	24	22	25	23	0.948
26095	NM_001042357,NM	**PTPN20**	protein tyrosine phospha	protein_coding	18	18	19	19	0.969
26097	NM_001206612,NM	**CHTOP**	chromatin target of PRM	protein_coding	122	121	123	122	0.994
26098	NM_001202438,NM	**EDRF1**	erythroid differentiation r	protein_coding	19	17	20	18	0.893
26099	NM_001114600,NM	**SZRD1**	SUZ RNA binding domai	protein_coding	171	175	172	176	1.021
26100	NM_001033518,NM	**WIPI2**	WD repeat domain, phos	protein_coding	19	21	20	22	1.055
26102	NR_026964	**DKFZP434A062**	uncharacterized LOC261	lncRNA	0	0	1	1	1.000
26103	NM_015613,XM_01	**LRIT1**	leucine rich repeat, Ig-lik	protein_coding	0	0	1	1	1.000
26108	NM_001330326,NM	**PYGO1**	pygopus family PHD fing	protein_coding	0	0	1	1	1.000
26112	NM_015621	**CCDC69**	coiled-coil domain contai	protein_coding	62	55	63	56	0.898
26115	NM_025185,XM_00	**TANC2**	tetratricopeptide repeat,	protein_coding	0	0	1	1	1.000
26118	NM_001348350,NM	**WSB1**	WD repeat and SOCS b	protein_coding	38	35	39	36	0.939
26119	NM_015627,XM_00	**LDLRAP1**	low density lipoprotein re	protein_coding	0	0	1	1	1.023
26121	NM_015629,XM_00	**PRPF31**	pre-mRNA processing fa	protein_coding	141	153	142	154	1.082
26122	NM_015630,XM_01	**EPC2**	enhancer of polycomb h	protein_coding	21	19	22	20	0.930
26123	NM_001143973,NM	**TCTN3**	tectonic family member 3	protein_coding	36	37	37	38	1.023
26127	NM_001171887,NM	**FGFR1OP2**	FGFR1 oncogene partne	protein_coding	78	67	79	68	0.860
26128	NM_015634,XM_01	**KIFBP**	kinesin family binding pr	protein_coding	20	20	21	21	0.984
26130	NM_001282679,NM	**GAPVD1**	GTPase activating protei	protein_coding	23	20	24	21	0.863
26133	NM_015638,NM_19	**TRPC4AP**	transient receptor potent	protein_coding	69	65	70	66	0.948
26134	NR_104269	**RALGAPA1P1**	RALGAPA1 pseudogene	transcribed_pseu	1	1	2	2	0.916
26135	NM_001018067,NM	**SERBP1**	SERPINE1 mRNA bindin	protein_coding	417	382	418	383	0.915
26136	NM_015641,NM_15	**TES**	testin LIM domain protein	protein_coding	296	246	297	247	0.834
26137	NM_001164342,NM	**ZBTB20**	zinc finger and BTB dom	protein_coding	0	0	1	1	0.992
26138	NR_026772	**LINC00588**	long intergenic non-prote	lncRNA	0	0	1	1	0.807
26140	NM_001025930,NM	**TTLL3**	tubulin tyrosine ligase lik	protein_coding	9	10	10	11	1.076
26145	NM_015649	**IRF2BP1**	interferon regulatory fact	protein_coding	22	24	23	25	1.079
26146	NM_001139490,NM	**TRAF3IP1**	TRAF3 interacting protei	protein_coding	10	9	11	10	0.954
26147	NM_001009936,NM	**PHF19**	PHD finger protein 19	protein_coding	201	219	202	220	1.085
26149	NM_001317916,NM	**ZNF658**	zinc finger protein 658	protein_coding	0	0	1	1	1.000
26150	NM_015653,XM_00	**RIBC2**	RIB43A domain with coil	protein_coding	0	0	1	1	1.000
26151	NM_001305077,NM	**NAT9**	N-acetyltransferase 9 (pu	protein_coding	33	29	34	30	0.889
26152	NM_001290261,NM	**ZNF337**	zinc finger protein 337	protein_coding	6	7	7	8	1.049
26153	NM_015656,XM_01	**KIF26A**	kinesin family member 2	protein_coding	0	0	1	1	1.100
26154	NM_015657,NM_17	**ABCA12**	ATP binding cassette su	protein_coding	0	0	1	1	1.000
26155	NM_015658	**NOC2L**	NOC2 like nucleolar ass	protein_coding	272	281	273	282	1.032
26156	NM_015659	**RSL1D1**	ribosomal L1 domain cor	protein_coding	167	153	168	154	0.915
26157	NM_015660	**GIMAP2**	GTPase, IMAP family me	protein_coding	83	85	84	86	1.029
26160	NM_015662,XM_00	**IFT172**	intraflagellar transport 17	protein_coding	2	2	3	3	1.096
26164	NM_015666,XM_00	**MTG2**	mitochondrial ribosome a	protein_coding	45	44	46	45	0.984
26165	NM_015667	**SPATA31A7**	SPATA31 subfamily A m	protein_coding	0	0	1	1	1.000
26166	NM_001286692,NM	**RGS22**	regulator of G protein sig	protein_coding	0	0	1	1	1.000
26167	NM_015669	**PCDHB5**	protocadherin beta 5	protein_coding	0	0	1	1	1.000
26168	NM_015670	**SENP3**	SUMO specific peptidase	protein_coding	83	83	84	84	1.001
26173	NM_001080453,XM	**INTS1**	integrator complex subu	protein_coding	36	35	37	36	0.977
26175	NM_001098621,NM	**TMEM251**	transmembrane protein 2	protein_coding	14	14	15	15	0.996
26184	NR_002169	**OR1F2P**	olfactory receptor family	transcribed_pseu	0	0	1	1	1.000
26188	NM_012353	**OR1C1**	olfactory receptor family	protein_coding	0	0	1	1	1.000
26189	NM_012352	**OR1A2**	olfactory receptor family	protein_coding	0	0	1	1	1.000
26190	NM_001375888,NM	**FBXW2**	F-box and WD repeat do	protein_coding	40	40	41	41	1.015
26191	NM_001193431,NM	**PTPN22**	protein tyrosine phospha	protein_coding	120	103	121	104	0.860
26205	NM_012384,XM_00	**GMEB2**	glucocorticoid modulator	protein_coding	17	18	18	19	1.013
26206	NM_001039592,NM	**SPAG8**	sperm associated antige	protein_coding	0	0	1	1	1.198
26207	NM_012417,NM_18	**PITPNC1**	phosphatidylinositol tran	protein_coding	23	22	24	23	0.983
26211	NM_012369,XM_01	**OR2F1**	olfactory receptor family	protein_coding	0	0	1	1	1.000
26212	NM_012367	**OR2B6**	olfactory receptor family	protein_coding	1	1	2	2	0.968
26219	NM_001004452	**OR1J4**	olfactory receptor family	protein_coding	0	0	1	1	1.000
26220	NR_002733,NR_02	**DGCR5**	DiGeorge syndrome critic	lncRNA	0	0	1	1	1.000
26223	NR_152420,NR_15	**FBXL21P**	F-box and leucine rich re	transcribed_pseu	0	0	1	1	1.000
26224	NM_012158,XM_00	**FBXL3**	F-box and leucine rich re	protein_coding	29	24	30	25	0.831
26225	NM_001037174,NM	**ARL5A**	ADP ribosylation factor li	protein_coding	72	57	73	58	0.796
26226	NR_033408	**FBXW4P1**	F-box and WD repeat do	transcribed_pseu	0	0	1	1	1.000
26227	NM_006623,XM_01	**PHGDH**	phosphoglycerate dehyd	protein_coding	0	0	1	1	1.000
26228	NM_001317769,NM	**STAP1**	signal transducing adapt	protein_coding	19	14	20	15	0.745
26229	NM_001288721,NM	**B3GAT3**	beta-1,3-glucuronyltrans	protein_coding	85	84	86	85	0.977
26230	NM_001010927,NM	**TIAM2**	TIAM Rac1 associated G	protein_coding	0	0	1	1	1.000

26231	NM_001004055,NM	**LRRC29**	leucine rich repeat conta	protein_coding	0	1	1	2	1.259
26232	NM_012168	**FBXO2**	F-box protein 2	protein_coding	8	8	9	9	1.014
26233	NM_012162,NM_02	**FBXL6**	F-box and leucine rich re	protein_coding	7	10	8	11	1.295
26234	NM_001193534,NM	**FBXL5**	F-box and leucine rich re	protein_coding	31	30	32	31	0.974
26235	NM_001278716,NM	**FBXL4**	F-box and leucine rich re	protein_coding	2	1	3	2	0.966
26238	NR_026773	**LINC01558**	long intergenic non-prote	lncRNA	0	0	1	1	1.000
26239	NM_014357	**LCE2B**	late cornified envelope 2	protein_coding	0	0	1	1	1.000
26240	NM_012135,XM_01	**FAM50B**	family with sequence sim	protein_coding	0	0	1	1	1.000
26245	NM_017504	**OR2M4**	olfactory receptor family	protein_coding	0	0	1	1	1.000
26246	NM_001004686,XM	**OR2L2**	olfactory receptor family	protein_coding	0	0	1	1	1.000
26247	NR_002145	**OR2L1P**	olfactory receptor family	transcribed_pseu	0	0	1	1	1.000
26248	NM_205859	**OR2K2**	olfactory receptor family	protein_coding	0	0	1	1	1.000
26249	NM_001257194,NM	**KLHL3**	kelch like family member	protein_coding	1	1	2	2	0.953
26251	NM_012283,XM_01	**KCNG2**	potassium voltage-gated	protein_coding	0	0	1	1	1.000
26253	NM_014358,XM_01	**CLEC4E**	C-type lectin domain fam	protein_coding	0	0	1	1	1.000
26254	NM_014359,XM_01	**OPTC**	opticin	protein_coding	0	0	1	1	1.000
26255	NR_002734	**PTTG3P**	pituitary tumor-transform	transcribed_pseu	0	0	1	1	1.000
26256	NM_001308231,NM	**CABYR**	calcium binding tyrosine	protein_coding	1	1	2	2	0.850
26257	NM_014360	**NKX2-8**	NK2 homeobox 8	protein_coding	0	0	1	1	1.000
26258	NM_001311255,NM	**BLOC1S6**	biogenesis of lysosomal	protein_coding	74	65	75	66	0.880
26259	NM_012174,NM_15	**FBXW8**	F-box and WD repeat do	protein_coding	4	3	5	4	0.786
26260	NM_012173,NM_18	**FBXO25**	F-box protein 25	protein_coding	0	0	1	1	1.000
26261	NM_001163499,NM	**FBXO24**	F-box protein 24	protein_coding	0	0	1	1	1.018
26262	NM_001006616,NM	**TSPAN17**	tetraspanin 17	protein_coding	52	58	53	59	1.108
26263	NM_012170,NM_14	**FBXO22**	F-box protein 22	protein_coding	22	17	23	18	0.792
26266	NM_001318192,NM	**SLC13A4**	solute carrier family 13 m	protein_coding	0	0	1	1	1.000
26267	NM_012166,XM_00	**FBXO10**	F-box protein 10	protein_coding	9	10	10	11	1.032
26268	NM_012347,NM_03	**FBXO9**	F-box protein 9	protein_coding	38	36	39	37	0.944
26269	NM_012180,XM_01	**FBXO8**	F-box protein 8	protein_coding	10	10	11	11	0.918
26270	NM_018438,XM_00	**FBXO6**	F-box protein 6	protein_coding	130	121	131	122	0.935
26271	NM_001142522,NM	**FBXO5**	F-box protein 5	protein_coding	71	69	72	70	0.972
26272	NM_001297437,NM	**FBXO4**	F-box protein 4	protein_coding	25	23	26	24	0.946
26273	NM_012175,NM_03	**FBXO3**	F-box protein 3	protein_coding	7	7	8	8	1.085
26275	NM_014362,NM_19	**HIBCH**	3-hydroxyisobutyryl-CoA	protein_coding	26	27	27	28	1.042
26276	NM_001289148,NM	**VPS33B**	VPS33B late endosome	protein_coding	14	14	15	15	1.000
26277	NM_001099274,NM	**TINF2**	TERF1 interacting nuclea	protein_coding	46	50	47	51	1.094
26278	NM_001278055,NM	**SACS**	sacsin molecular chaper	protein_coding	38	28	39	29	0.759
26279	NM_001271814,NM	**PLA2G2D**	phospholipase A2 group	protein_coding	0	0	1	1	1.000
26280	NM_017416,XM_01	**IL1RAPL2**	interleukin 1 receptor ac	protein_coding	0	0	1	1	1.000
26281	NM_019851	**FGF20**	fibroblast growth factor 2	protein_coding	0	0	1	1	1.000
26284	NM_001317985,NM	**ERAL1**	Era like 12S mitochondri	protein_coding	99	109	100	110	1.094
26285	NM_012131	**CLDN17**	claudin 17	protein_coding	0	0	1	1	1.000
26286	NM_001142293,NM	**ARFGAP3**	ADP ribosylation factor G	protein_coding	23	22	24	23	0.975
26287	NM_001129981,NM	**ANKRD2**	ankyrin repeat domain 2	protein_coding	0	0	1	1	1.000
26289	NM_012093,NM_17	**AK5**	adenylate kinase 5	protein_coding	0	0	1	1	1.014
26290	NM_017417	**GALNT8**	polypeptide N-acetylgala	protein_coding	0	0	1	1	0.925
26291	NM_019113	**FGF21**	fibroblast growth factor 2	protein_coding	0	0	1	1	1.000
26292	NM_012333,NR_03	**MYCBP**	MYC binding protein	protein_coding	54	49	55	50	0.918
26297	NM_012139,NR_10	**SERGEF**	secretion regulating guai	protein_coding	15	12	16	13	0.781
26298	NM_001206615,NM	**EHF**	ETS homologous factor	protein_coding	0	0	1	1	1.000
26301	NM_001282629,NM	**GBGT1**	globoside alpha-1,3-N-ac	protein_coding	0	0	1	1	1.000
26330	NM_014364	**GAPDHS**	glyceraldehyde-3-phospl	protein_coding	0	0	1	1	1.059
26333	NM_030901	**OR7A17**	olfactory receptor family	protein_coding	0	0	1	1	1.000
26338	NM_001004739	**OR5L2**	olfactory receptor family	protein_coding	0	0	1	1	1.000
26339	NM_001004736	**OR5K1**	olfactory receptor family	protein_coding	0	0	1	1	1.000
26341	NM_001005338	**OR5H1**	olfactory receptor family	protein_coding	0	0	1	1	1.000
26343	NR_027711	**OR5E1P**	olfactory receptor family	transcribed_pseu	0	0	1	1	1.000
26353	NM_014365	**HSPB8**	heat shock protein family	protein_coding	0	0	1	1	1.000
26354	NM_014366,NM_20	**GNL3**	G protein nucleolar 3	protein_coding	197	176	198	177	0.891
26355	NM_014367	**FAM162A**	family with sequence sim	protein_coding	60	60	61	61	1.003
26468	NM_001242333,NM	**LHX6**	LIM homeobox 6	protein_coding	0	0	1	1	1.000
26469	NM_001142370,NM	**PTPN18**	protein tyrosine phospha	protein_coding	48	50	49	51	1.047
26470	NM_001114099,NM	**SEZ6L2**	seizure related 6 homolo	protein_coding	0	0	1	1	1.000
26471	NM_001042483,NM	**NUPR1**	nuclear protein 1, transc	protein_coding	0	0	1	1	1.000
26472	NM_138689	**PPP1R14B**	protein phosphatase 1 re	protein_coding	190	200	191	201	1.058
26476	NM_001363557,NM	**OR10J1**	olfactory receptor family	protein_coding	0	0	1	1	1.000
26492	NM_001291438	**OR8G2P**	olfactory receptor family	protein_coding	0	0	1	1	1.000
26493	NM_012378	**OR8B8**	olfactory receptor family	protein_coding	0	0	1	1	1.000
26494	NM_001002905	**OR8G1**	olfactory receptor family	protein_coding	0	0	1	1	1.000
26496	NM_001003745	**OR10A3**	olfactory receptor family	protein_coding	0	0	1	1	1.000
26497	NM_001355213	**OR10D3**	olfactory receptor family	protein_coding	0	0	1	1	1.000
26499	NM_016445	**PLEK2**	pleckstrin 2	protein_coding	0	0	1	1	1.000
26502	NM_001038618,NM	**NARF**	nuclear prelamin A recog	protein_coding	23	20	24	21	0.863
26503	NM_001382629,NM	**SLC17A5**	solute carrier family 17 m	protein_coding	2	1	3	2	0.764
26504	NM_020184,XM_00	**CNNM4**	cyclin and CBS domain c	protein_coding	6	6	7	7	1.018
26505	NM_017623,NM_19	**CNNM3**	cyclin and CBS domain c	protein_coding	14	14	15	15	1.034
26507	NM_001345887,NM	**CNNM1**	cyclin and CBS domain c	protein_coding	0	0	1	1	1.000
26508	NM_014571,XM_00	**HEYL**	hes related family bHLH	protein_coding	0	0	1	1	1.000
26509	NM_013451,NM_13	**MYOF**	myoferlin	protein_coding	0	0	1	1	1.000
26511	NM_012110,XM_00	**CHIC2**	cysteine rich hydrophobi	protein_coding	5	4	6	5	0.882
26512	NM_001039937,NM	**INTS6**	integrator complex subui	protein_coding	31	31	32	32	0.999
26513	gene-RPL34P2	**RPL34P2**	ribosomal protein L34 ps	pseudogene	0	0	1	1	1.149
26514	gene-RPL34P1	**RPL34P1**	ribosomal protein L34 ps	pseudogene	0	0	1	1	1.000
26515	NM_012192	**TIMM10B**	translocase of inner mito	protein_coding	55	51	56	52	0.929
26517	NM_012458	**TIMM13**	translocase of inner mito	protein_coding	138	139	139	140	1.008
26519	NM_012456,XM_02	**TIMM10**	translocase of inner mito	protein_coding	279	294	280	295	1.054
26520	NM_001304485,NM	**TIMM9**	translocase of inner mito	protein_coding	47	40	48	41	0.851
26521	NM_012459,NR_02	**TIMM8B**	translocase of inner mito	protein_coding	140	152	141	153	1.089
26523	NM_001317122,NM	**AGO1**	argonaute RISC compon	protein_coding	14	13	15	14	0.925
26524	NM_014572,XM_00	**LATS2**	large tumor suppressor k	protein_coding	0	0	1	1	1.000
26525	NM_012275,NM_17	**IL36RN**	interleukin 36 receptor ai	protein_coding	0	0	1	1	1.000
26526	NM_001282509,NM	**TSPAN16**	tetraspanin 16	protein_coding	0	0	1	1	1.000
26528	NM_001352033,NM	**DAZAP1**	DAZ associated protein 1	protein_coding	297	299	298	300	1.008
26529	NM_013936	**OR12D2**	olfactory receptor family	protein_coding	0	0	1	1	1.000
26530	NM_001348208,NR	**OR12D1**	olfactory receptor family	protein_coding	0	0	1	1	1.000
26531	NM_013937	**OR11A1**	olfactory receptor family	protein_coding	0	0	1	1	1.000
26532	NM_013938	**OR10H3**	olfactory receptor family	protein_coding	0	0	1	1	1.000
26533	NM_001005465	**OR10G3**	olfactory receptor family	protein_coding	0	0	1	1	1.000
26534	NM_001005466	**OR10G2**	olfactory receptor family	protein_coding	0	0	1	1	1.000
26538	NM_013939	**OR10H2**	olfactory receptor family	protein_coding	0	0	1	1	1.000
26539	NM_013940,XM_01	**OR10H1**	olfactory receptor family	protein_coding	0	0	1	1	1.000
26540	gene-OR10G1P	**OR10G1P**	olfactory receptor family	pseudogene	0	0	1	1	1.000
26548	NM_001303277,NM	**ITGB1BP2**	integrin subunit beta 1 b	protein_coding	1	1	2	2	0.958
26574	NM_012138,XM_01	**AATF**	apoptosis antagonizing t	protein_coding	205	203	206	204	0.990
26575	NM_012419,XM_01	**RGS17**	regulator of G protein sig	protein_coding	0	0	1	1	1.045
26576	NM_001170760,NM	**SRPK3**	SRSF protein kinase 3	protein_coding	0	0	1	1	1.000
26577	NM_013363	**PCOLCE2**	procollagen C-endopepti	protein_coding	0	0	1	1	1.000
26578	NM_012383,XM_00	**OSTF1**	osteoclast stimulating fa	protein_coding	143	139	144	140	0.971
26579	NM_001293291,NM	**MYEOV**	myeloma overexpressed	protein_coding	0	0	1	1	0.961
26580	NM_001122955,NM	**BSCL2**	BSCL2 lipid droplet biog	protein_coding	31	29	32	30	0.941
26583	gene-DUX4L8	**DUX4L8**	double homeobox 4 like	pseudogene	0	0	1	1	1.000
26585	NM_001191322,NM	**GREM1**	gremlin 1, DAN family Bl	protein_coding	0	0	1	1	1.000
26586	NM_001098525,NM	**CKAP2**	cytoskeleton associated	protein_coding	110	91	111	92	0.832
26589	NM_022163	**MRPL46**	mitochondrial ribosomal	protein_coding	90	100	91	101	1.110
26595	NM_001005468,XM	**OR8B2**	olfactory receptor family	protein_coding	0	0	1	1	1.000
26608	NM_001362660,NM	**TBL2**	transducin beta like 2	protein_coding	19	17	20	18	0.915
26609	NM_013452,XM_01	**VCX**	variable charge X-linked	protein_coding	0	0	1	1	1.000
26610	NM_001288725,NM	**ELP4**	elongator acetyltransfera	protein_coding	7	7	8	8	0.977
26628	NR_120438,NR_12	**OR7E47P**	olfactory receptor family	transcribed_pseu	0	0	1	1	1.000
26648	NM_001079935	**OR7E24**	olfactory receptor family	protein_coding	0	0	1	1	1.000
26658	NM_012377	**OR7C2**	olfactory receptor family	protein_coding	0	0	1	1	1.000
26659	NM_001370480,NM	**OR7A5**	olfactory receptor family	protein_coding	0	0	1	1	1.000
26664	NM_001370485,NM	**OR7C1**	olfactory receptor family	protein_coding	0	0	1	1	1.000
26682	NM_001004195	**OR4F4**	olfactory receptor family	protein_coding	0	0	1	1	1.000
26683	NM_001005224,XM	**OR4F3**	olfactory receptor family	protein_coding	0	0	1	1	1.009
26685	gene-OR4F1P	**OR4F1P**	olfactory receptor family	pseudogene	0	0	1	1	1.000

26686	NM_001001912	**OR4E2**	olfactory receptor family	protein_coding	0	0	1	1	1.000
26687	NM_001317107	**OR4E1**	olfactory receptor family	protein_coding	0	0	1	1	1.000
26689	NM_012374	**OR4D1**	olfactory receptor family	protein_coding	0	0	1	1	1.000
26692	NM_030903	**OR2W1**	olfactory receptor family	protein_coding	0	0	1	1	1.000
26693	NM_001258283	**OR2V1**	olfactory receptor family	protein_coding	0	0	1	1	1.000
26696	NM_030904	**OR2T1**	olfactory receptor family	protein_coding	0	0	1	1	1.000
26707	NM_030905	**OR2J2**	olfactory receptor family	protein_coding	0	0	1	1	1.000
26716	NM_001318014,NM	**OR2H1**	olfactory receptor family	protein_coding	0	0	1	1	1.000
26735	NM_001005234	**OR1L3**	olfactory receptor family	protein_coding	0	0	1	1	1.000
26737	NM_001005236	**OR1L1**	olfactory receptor family	protein_coding	0	0	1	1	1.000
26740	NM_054107,XM_02	**OR1J2**	olfactory receptor family	protein_coding	0	0	1	1	1.000
26747	NM_012345,XR_94	**NUFIP1**	nuclear FMR1 interacting	protein_coding	27	24	28	25	0.906
26750	NM_001136138,NM	**RPS6KC1**	ribosomal protein S6 kina	protein_coding	9	8	10	9	0.905
26751	NM_001159597,NM	**SH3YL1**	SH3 and SYLF domain c	protein_coding	7	6	8	7	0.904
26762	NM_001173393,NM	**HAVCR1**	hepatitis A virus cellular	protein_coding	0	0	1	1	1.114
26765	NR_002433	**SNORD12C**	small nucleolar RNA, C/I	snoRNA	1	1	2	2	1.078
26766	NR_004385	**RNU105C**	RNA, U105C small nucle	snoRNA	0	0	1	1	1.000
26767	NR_004386	**RNU105B**	RNA, U105B small nucle	snoRNA	0	0	1	1	1.000
26768	NR_004404	**SNORA73B**	small nucleolar RNA, H/	snoRNA	6	7	7	8	1.116
26769	NR_003938	**SNORD81**	small nucleolar RNA, C/I	snoRNA	58	49	59	50	0.854
26770	NR_003939	**SNORD79**	small nucleolar RNA, C/I	snoRNA	32	31	33	32	0.974
26771	NR_002574	**SNORD102**	small nucleolar RNA, C/I	snoRNA	10	9	11	10	0.883
26772	NR_000009	**SNORD4B**	small nucleolar RNA, C/I	snoRNA	1	0	2	1	0.527
26773	NR_000010	**SNORD4A**	small nucleolar RNA, C/I	snoRNA	12	12	13	13	1.034
26774	NR_003940	**SNORD80**	small nucleolar RNA, C/I	snoRNA	73	76	74	77	1.042
26775	NR_002581	**SNORA72**	small nucleolar RNA, H/	snoRNA	4	4	5	5	1.042
26776	NR_002910	**SNORA71B**	small nucleolar RNA, H/	snoRNA	7	7	8	8	1.019
26777	NR_002911	**SNORA71A**	small nucleolar RNA, H/	snoRNA	8	6	9	7	0.751
26778	NR_000011	**SNORA70**	small nucleolar RNA, H/	snoRNA	24	30	25	31	1.201
26779	NR_002584	**SNORA69**	small nucleolar RNA, H/	snoRNA	1	4	2	5	2.302
26780	NR_000012	**SNORA68**	small nucleolar RNA, H/	snoRNA	5	4	6	5	0.857
26781	NR_002912	**SNORA67**	small nucleolar RNA, H/	snoRNA	44	48	45	49	1.068
26782	NR_002444	**SNORA66**	small nucleolar RNA, H/	snoRNA	2	3	3	4	1.135
26783	NR_002449	**SNORA65**	small nucleolar RNA, H/	snoRNA	3	3	4	4	1.169
26784	NR_002326	**SNORA64**	small nucleolar RNA, H/	snoRNA	24	23	25	24	0.945
26785	NR_002913	**SNORD63**	small nucleolar RNA, C/I	snoRNA	4	5	5	6	1.055
26786	NR_002914	**SNORD62A**	small nucleolar RNA, C/I	snoRNA	2	4	3	5	1.622
26787	NR_002735	**SNORD61**	small nucleolar RNA, C/I	snoRNA	0	0	1	1	1.000
26788	NR_002736	**SNORD60**	small nucleolar RNA, C/I	snoRNA	6	8	7	9	1.233
26789	NR_002737	**SNORD59A**	small nucleolar RNA, C/I	snoRNA	1	1	2	2	1.110
26790	NR_002572	**SNORD58B**	small nucleolar RNA, C/I	snoRNA	0	0	1	1	1.003
26791	NR_002571	**SNORD58A**	small nucleolar RNA, C/I	snoRNA	16	16	17	17	0.990
26792	NR_002738	**SNORD57**	small nucleolar RNA, C/I	snoRNA	24	23	25	24	0.952
26793	NR_002739	**SNORD56**	small nucleolar RNA, C/I	snoRNA	12	18	13	19	1.450
26795	NR_002437	**SNORD54**	small nucleolar RNA, C/I	snoRNA	4	7	5	8	1.693
26796	NR_002741	**SNORD53**	small nucleolar RNA, C/I	snoRNA	2	2	3	3	1.186
26797	NR_002742	**SNORD52**	small nucleolar RNA, C/I	snoRNA	14	12	15	13	0.872
26798	NR_002589	**SNORD51**	small nucleolar RNA, C/I	snoRNA	5	4	6	5	0.770
26799	NR_002743	**SNORD50A**	small nucleolar RNA, C/I	snoRNA	4	3	5	4	0.717
26800	NR_002744	**SNORD49A**	small nucleolar RNA, C/I	snoRNA	10	9	11	10	0.902
26801	NR_002745	**SNORD48**	small nucleolar RNA, C/I	snoRNA	5	5	6	6	0.948
26802	NR_002746	**SNORD47**	small nucleolar RNA, C/I	snoRNA	81	86	82	87	1.058
26804	NR_002748	**SNORD45B**	small nucleolar RNA, C/I	snoRNA	7	4	8	5	0.615
26805	NR_002749	**SNORD45A**	small nucleolar RNA, C/I	snoRNA	6	4	7	5	0.759
26806	NR_002750	**SNORD44**	small nucleolar RNA, C/I	snoRNA	22	27	23	28	1.193
26807	NR_002439	**SNORD43**	small nucleolar RNA, C/I	snoRNA	5	2	6	3	0.599
26808	NR_000013	**SNORD42B**	small nucleolar RNA, C/I	snoRNA	2	2	3	3	0.862
26809	NR_000014	**SNORD42A**	small nucleolar RNA, C/I	snoRNA	1	2	2	3	1.313
26810	NR_002751	**SNORD41**	small nucleolar RNA, C/I	snoRNA	1	0	2	1	0.621
26811	NR_000015	**SNORD55**	small nucleolar RNA, C/I	snoRNA	14	17	15	18	1.188
26812	NR_002602	**SNORD37**	small nucleolar RNA, C/I	snoRNA	1	3	2	4	2.168
26813	NR_000016	**SNORD36C**	small nucleolar RNA, C/I	snoRNA	3	6	4	7	1.665
26814	NR_000017	**SNORD36B**	small nucleolar RNA, C/I	snoRNA	3	4	4	5	1.070
26815	NR_002448	**SNORD36A**	small nucleolar RNA, C/I	snoRNA	33	36	34	37	1.081
26816	NR_000018	**SNORD35A**	small nucleolar RNA, C/I	snoRNA	5	5	6	6	1.037
26817	NR_000019	**SNORD34**	small nucleolar RNA, C/I	snoRNA	9	8	10	9	0.891
26818	NR_000020	**SNORD33**	small nucleolar RNA, C/I	snoRNA	6	5	7	6	0.910
26819	NR_000021	**SNORD32A**	small nucleolar RNA, C/I	snoRNA	26	16	27	17	0.653
26820	NR_002447	**SNORD24**	small nucleolar RNA, C/I	snoRNA	4	4	5	5	0.942
26821	NR_002915	**SNORA74A**	small nucleolar RNA, H/	snoRNA	1	0	2	1	0.617
26822	NR_000022	**SNORD14A**	small nucleolar RNA, C/I	snoRNA	5	6	6	7	1.125
26823	gene-RNU12-2P	**RNU12-2P**	RNA, U12 small nuclear	pseudogene	0	1	1	2	1.368
26824	NR_004407	**RNU11**	RNA, U11 small nuclear	snRNA	0	0	1	1	1.020
26827	NR_004394	**RNU6-1**	RNA, U6 small nuclear 1	snRNA	10	11	11	12	1.143
26828	NR_002753	**RNU5F-1**	RNA, U5F small nuclear	snRNA	0	0	1	1	1.000
26829	NR_002754	**RNU5E-1**	RNA, U5E small nuclear	snRNA	1	1	2	2	0.951
26830	NR_002755	**RNU5D-1**	RNA, U5D small nuclear	snRNA	1	0	2	1	0.561
26831	NR_002756	**RNU5A-1**	RNA, U5A small nuclear	snRNA	8	5	9	6	0.735
26832	NR_002757	**RNU5B-1**	RNA, U5B small nuclear	snRNA	0	0	1	1	0.711
26834	NR_003137	**RNU4-2**	RNA, U4 small nuclear 2	snRNA	1	0	2	1	0.620
26835	NR_003925	**RNU4-1**	RNA, U4 small nuclear 1	snRNA	0	0	1	1	0.781
26843	NR_145983	**SNORD3P3**	small nucleolar RNA, C/I	snoRNA	0	0	1	1	1.000
26851	NR_003271	**SNORD3B-1**	small nucleolar RNA, C/I	snoRNA	0	1	1	2	1.273
26852	gene-RNU2-5P	**RNU2-5P**	RNA, U2 small nuclear 5	pseudogene	0	0	1	1	1.000
26853	gene-RNU2-4P	**RNU2-4P**	RNA, U2 small nuclear 4	pseudogene	0	0	1	1	1.095
26854	gene-RNU2-3P	**RNU2-3P**	RNA, U2 small nuclear 3	pseudogene	0	0	1	1	1.000
26863	NR_004400	**RNVU1-18**	RNA, variant U1 small nu	snRNA	0	0	1	1	1.054
26864	NR_004426	**RNVU1-7**	RNA, variant U1 small nu	snRNA	0	0	1	1	1.026
26869	NR_004408	**RNU1-3**	RNA, U1 small nuclear 3	snRNA	1	1	2	2	1.075
26870	NR_004427	**RNU1-2**	RNA, U1 small nuclear 2	snRNA	1	1	2	2	1.287
26871	NR_004430	**RNU1-1**	RNA, U1 small nuclear 1	snRNA	1	1	2	2	1.071
26872	NM_012449	**STEAP1**	STEAP family member 1	protein_coding	0	0	1	1	1.000
26873	NM_017570,XM_01	**OPLAH**	5-oxoprolinase, ATP-hyd	protein_coding	0	0	1	1	1.000
26952	NM_012390,XM_01	**SMR3A**	submaxillary gland andro	protein_coding	0	0	1	1	1.000
26953	NM_001243202,NM	**RANBP6**	RAN binding protein 6	protein_coding	33	30	34	31	0.902
26957	gene-ABCD1P4	**ABCD1P4**	ATP binding cassette sul	pseudogene	0	0	1	1	1.000
26958	NM_001290033,NM	**COPG2**	COPI coat complex subu	protein_coding	12	12	13	13	1.001
26959	NM_001244262,NM	**HBP1**	HMG-box transcription fa	protein_coding	5	6	6	7	1.138
26960	NM_001204197,NM	**NBEA**	neurobeachin	protein_coding	1	1	2	2	0.899
26973	NM_001144073,NM	**CHORDC1**	cysteine and histidine ric	protein_coding	146	124	147	125	0.844
26974	NM_001291488,NM	**ZNF285**	zinc finger protein 285	protein_coding	0	0	1	1	1.000
26978	gene-PABPC1P3	**PABPC1P3**	poly(A) binding protein c	pseudogene	0	0	1	1	1.000
26980	gene-PABPC1P1	**PABPC1P1**	poly(A) binding protein c	pseudogene	0	0	1	1	1.000
26982	gene-ABCD1P3	**ABCD1P3**	ATP binding cassette sul	pseudogene	0	0	1	1	1.000
26983	gene-ABCD1P2	**ABCD1P2**	ATP binding cassette sul	pseudogene	0	0	1	1	1.000
26984	NM_012430,XM_01	**SEC22A**	SEC22 homolog A, vesic	protein_coding	11	11	12	12	0.999
26985	NM_001320263,NM	**AP3M1**	adaptor related protein c	protein_coding	57	50	58	51	0.884
26986	NM_002568,XM_00	**PABPC1**	poly(A) binding protein c	protein_coding	1,363	1,317	1,364	1,318	0.967
26993	NM_001291478,NM	**AKAP8L**	A-kinase anchoring prote	protein_coding	66	68	67	69	1.035
26994	NM_014372	**RNF11**	ring finger protein 11	protein_coding	29	27	30	28	0.914
26995	NM_001329861,NM	**TRUB2**	TruB pseudouridine synt	protein_coding	21	22	22	23	1.011
26996	NM_014373,XM_00	**GPR160**	G protein-coupled recept	protein_coding	0	0	1	1	0.981
26998	NM_001308077,NM	**FETUB**	fetuin B	protein_coding	0	0	1	1	1.000
26999	NM_001037333,NM	**CYFIP2**	cytoplasmic FMR1 intera	protein_coding	163	159	164	160	0.975
27000	NM_001129887,NM	**DNAJC2**	DnaJ heat shock protein	protein_coding	106	90	107	91	0.845
27002	gene-PAFAH1B1P2	**PAFAH1B1P2**	platelet activating factor	pseudogene	0	0	1	1	1.000
27004	NR_028288,NR_15	**TCL6**	T cell leukemia/lymphom	lncRNA	0	0	1	1	1.000
27005	NM_001014443,NM	**USP21**	ubiquitin specific peptida	protein_coding	20	22	21	23	1.129
27006	NM_001300812,NM	**FGF22**	fibroblast growth factor 2	protein_coding	0	0	1	1	1.000
27010	NM_001042482,NM	**TPK1**	thiamin pyrophosphokina	protein_coding	2	1	3	2	0.851
27012	NM_014379	**KCNV1**	potassium voltage-gated	protein_coding	0	0	1	1	1.000
27013	NM_001321389,NM	**CNPPD1**	cyclin Pas1/PHO80 dom	protein_coding	74	81	75	82	1.090
27018	NM_001282674,NM	**BEX3**	brain expressed X-linked	protein_coding	0	0	1	1	1.000
27019	NM_001281428,NM	**DNAI1**	dynein axonemal interme	protein_coding	0	0	1	1	1.000
27020	NM_001161363,NM	**NPTN**	neuroplastin	protein_coding	104	94	105	95	0.908
27022	NM_012183	**FOXD3**	forkhead box D3	protein_coding	0	0	1	1	1.000

27023	NM_012182	**FOXB1**	forkhead box B1	protein_coding	0	0	1	1	1.000
27030	NM_001040108,NM	**MLH3**	mutL homolog 3	protein_coding	12	10	13	11	0.827
27031	NM_153240	**NPHP3**	nephrocystin 3	protein_coding	0	0	1	1	1.106
27032	NM_001001485,NM	**ATP2C1**	ATPase secretory pathwa	protein_coding	42	39	43	40	0.920
27033	NM_001316902,NM	**ZBTB32**	zinc finger and BTB dom	protein_coding	80	81	81	82	1.020
27034	NM_014384,XM_00	**ACAD8**	acyl-CoA dehydrogenase	protein_coding	35	38	36	39	1.087
27035	NM_001271815,NM	**NOX1**	NADPH oxidase 1	protein_coding	0	0	1	1	1.000
27036	NM_001277201,NM	**SIGLEC7**	sialic acid binding Ig like	protein_coding	0	0	1	1	1.000
27037	NM_001257994,NM	**TRMT2A**	tRNA methyltransferase	protein_coding	41	42	42	43	1.016
27039	NM_001258448,NM	**PKD2L2**	polycystin 2 like 2, transi	protein_coding	0	0	1	1	1.007
27040	NM_001014987,NM	**LAT**	linker for activation of T c	protein_coding	171	164	172	165	0.963
27042	NM_014388,XM_00	**UTP25**	UTP25 small subunit pro	protein_coding	12	11	13	12	0.916
27043	NM_001278241,NM	**PELP1**	proline, glutamate and le	protein_coding	31	32	32	33	1.020
27044	NM_014390,XM_01	**SND1**	staphylococcal nuclease	protein_coding	80	83	81	84	1.035
27063	NM_014391	**ANKRD1**	ankyrin repeat domain 1	protein_coding	0	0	1	1	1.000
27065	NM_001040101,NM	**NSG1**	neuronal vesicle traffickir	protein_coding	0	0	1	1	1.000
27067	NM_001164380,NM	**STAU2**	staufen double-stranded	protein_coding	41	36	42	37	0.868
27068	NM_006903,NM_17	**PPA2**	inorganic pyrophosphata	protein_coding	29	29	30	30	0.976
27069	NM_014394	**GHITM**	growth hormone inducibl	protein_coding	340	314	341	315	0.923
27071	NM_001306151,NM	**DAPP1**	dual adaptor of phospho	protein_coding	0	0	1	1	1.093
27072	NM_014396,NM_08	**VPS41**	VPS41 subunit of HOPS	protein_coding	9	9	10	10	0.992
27074	NM_014398,XM_00	**LAMP3**	lysosomal associated me	protein_coding	0	0	1	1	1.000
27075	NM_014399	**TSPAN13**	tetraspanin 13	protein_coding	0	0	1	1	1.170
27076	NM_014400	**LYPD3**	LY6/PLAUR domain cont	protein_coding	0	0	1	1	1.000
27077	NM_001243473,NM	**B9D1**	B9 domain containing 1	protein_coding	0	0	1	1	0.874
27079	NM_001286407,NM	**RPUSD2**	RNA pseudouridine synt	protein_coding	40	42	41	43	1.048
27085	NM_022045,XM_01	**MTBP**	MDM2 binding protein	protein_coding	24	23	25	24	0.974
27086	NM_001012505,NM	**FOXP1**	forkhead box P1	protein_coding	2	2	3	3	0.909
27087	NM_001367973,NM	**B3GAT1**	beta-1,3-glucuronyltrans	protein_coding	0	0	1	1	1.000
27089	NM_014402	**UQCRQ**	ubiquinol-cytochrome c r	protein_coding	120	135	121	136	1.131
27090	NM_175039,NM_17	**ST6GALNAC4**	ST6 N-acetylgalactosam	protein_coding	3	3	4	4	1.029
27091	NM_001371476,NM	**CACNG5**	calcium voltage-gated ch	protein_coding	0	0	1	1	1.000
27092	NM_014405	**CACNG4**	calcium voltage-gated ch	protein_coding	0	0	1	1	1.000
27093	gene-KCNMB3P1	**KCNMB3P1**	KCNMB3 pseudogene 1	pseudogene	0	0	1	1	0.981
27094	NM_001163677,NM	**KCNMB3**	potassium calcium-activa	protein_coding	0	0	1	1	0.956
27095	NM_001270894,NM	**TRAPPC3**	trafficking protein particle	protein_coding	122	119	123	120	0.978
27097	NM_001025247,NM	**TAF5L**	TATA-box binding protein	protein_coding	39	36	40	37	0.927
27098	NM_001289036,NM	**CLUL1**	clusterin like 1	protein_coding	0	0	1	1	0.978
27099	NR_027330	**SND1-IT1**	SND1 intronic transcript	lncRNA	0	0	1	1	1.094
27101	NM_001007214,NM	**CACYBP**	calcyclin binding protein	protein_coding	191	181	192	182	0.949
27102	NM_001134335,NM	**EIF2AK1**	eukaryotic translation init	protein_coding	71	78	72	79	1.087
27106	NM_001025604,NM	**ARRDC2**	arrestin domain containir	protein_coding	18	18	19	19	0.977
27107	NM_014415,XM_01	**ZBTB11**	zinc finger and BTB dom	protein_coding	22	20	23	21	0.905
27109	NM_001003803,NM	**DMAC2L**	distal membrane arm ass	protein_coding	14	13	15	14	0.914
27111	NM_001199784,NM	**SDCBP2**	syndecan binding proteir	protein_coding	1	1	2	2	1.339
27112	NM_015686,XM_01	**FAM155B**	family with sequence sim	protein_coding	0	0	1	1	1.000
27113	NM_001127240,NM	**BBC3**	BCL2 binding componen	protein_coding	1	1	2	2	0.998
27115	NM_018945	**PDE7B**	phosphodiesterase 7B	protein_coding	0	0	1	1	1.000
27120	NM_001197301,NM	**DKKL1**	dickkopf like acrosomal p	protein_coding	0	0	1	1	1.000
27121	NM_014420,XM_01	**DKK4**	dickkopf WNT signaling	protein_coding	0	0	1	1	1.000
27122	NM_001018057,NM	**DKK3**	dickkopf WNT signaling	protein_coding	0	0	1	1	1.000
27123	NM_014421	**DKK2**	dickkopf WNT signaling	protein_coding	0	0	1	1	1.000
27124	NM_001002837,NM	**INPP5J**	inositol polyphosphate-5	protein_coding	0	0	1	1	1.039
27125	NM_014423,XM_00	**AFF4**	AF4/FMR2 family membe	protein_coding	21	17	22	18	0.843
27127	NM_001291501,NM	**SMC1B**	structural maintenance c	protein_coding	0	0	1	1	1.000
27128	NM_001318024,NM	**CYTH4**	cytohesin 4	protein_coding	70	69	71	70	0.984
27129	NM_001349682,NM	**HSPB7**	heat shock protein family	protein_coding	0	0	1	1	1.000
27130	NM_001318381,NM	**INVS**	inversin	protein_coding	4	4	5	5	0.972
27131	NM_001282454,NM	**SNX5**	sorting nexin 5	protein_coding	115	109	116	110	0.947
27132	NM_014427,NM_15	**CPNE7**	copine 7	protein_coding	0	0	1	1	0.974
27133	NM_139318,NM_17	**KCNH5**	potassium voltage-gated	protein_coding	0	0	1	1	1.000
27134	NM_001267560,NM	**TJP3**	tight junction protein 3	protein_coding	0	0	1	1	1.000
27136	NM_014429,XM_00	**MORC1**	MORC family CW-type z	protein_coding	0	0	1	1	1.000
27141	NM_001318807,NM	**CIDEB**	cell death inducing DFF/	protein_coding	0	0	1	1	0.949
27143	NM_014431,XM_01	**PALD1**	phosphatase domain cor	protein_coding	0	0	1	1	0.986
27145	NM_001289987,NM	**FILIP1**	filamin A interacting prot	protein_coding	0	0	1	1	1.024
27146	NM_015688	**FAM184B**	family with sequence sim	protein_coding	0	0	1	1	0.951
27147	NM_001318052,NM	**DENND2A**	DENN domain containing	protein_coding	0	0	1	1	1.000
27148	NM_001243313,NM	**STK36**	serine/threonine kinase 3	protein_coding	13	13	14	14	1.003
27151	NM_015692,NR_14	**CPAMD8**	C3 and PZP like alpha-2	protein_coding	0	0	1	1	1.000
27152	NM_015693,XM_01	**INTU**	inturned planar cell pola	protein_coding	0	0	1	1	1.000
27153	NM_015694,XM_00	**ZNF777**	zinc finger protein 777	protein_coding	9	11	10	12	1.237
27154	NM_015695,XM_00	**BRPF3**	bromodomain and PHD f	protein_coding	56	51	57	52	0.925
27156	NM_014433,XM_01	**RSPH14**	radial spoke head 14 hor	protein_coding	0	0	1	1	1.013
27158	NM_001144026,NM	**NDOR1**	NADPH dependent diflav	protein_coding	22	23	23	24	1.025
27159	NM_001040623,NM	**CHIA**	chitinase acidic	protein_coding	0	0	1	1	1.000
27160	NR_002226	**INGX**	inhibitor of growth family	transcribed_pseu	0	0	1	1	1.000
27161	NM_001164623,NM	**AGO2**	argonaute RISC catalytic	protein_coding	26	20	27	21	0.787
27163	NM_001042402,NM	**NAAA**	N-acylethanolamine acid	protein_coding	18	18	19	19	0.999
27164	NM_171999	**SALL3**	spalt like transcription fa	protein_coding	0	0	1	1	1.000
27165	NM_001280796,NM	**GLS2**	glutaminase 2	protein_coding	0	0	1	1	1.000
27166	NM_001271828,NM	**PRELID1**	PRELI domain containing	protein_coding	448	456	449	457	1.018
27170	gene-COTL1P1	**COTL1P1**	COTL1 pseudogene 1	pseudogene	0	0	1	1	1.000
27173	NM_001271957,NM	**SLC39A1**	solute carrier family 39 m	protein_coding	149	142	150	143	0.950
27175	NM_001320509,NM	**TUBG2**	tubulin gamma 2	protein_coding	16	22	17	23	1.350
27177	NM_014438,NM_17	**IL36B**	interleukin 36 beta	protein_coding	0	0	1	1	1.000
27178	NM_014439,NM_17	**IL37**	interleukin 37	protein_coding	0	0	1	1	0.801
27179	NM_014440,XM_00	**IL36A**	interleukin 36 alpha	protein_coding	0	0	1	1	1.000
27180	NM_001198558,NM	**SIGLEC9**	sialic acid binding Ig like	protein_coding	0	0	1	1	1.000
27181	NM_001363548,NM	**SIGLEC8**	sialic acid binding Ig like	protein_coding	0	0	1	1	1.000
27183	NM_013245	**VPS4A**	vacuolar protein sorting 4	protein_coding	59	58	60	59	0.991
27184	NR_002227	**DISC2**	disrupted in schizophren	lncRNA	0	0	1	1	1.000
27185	NM_001012957,NM	**DISC1**	DISC1 scaffold protein	protein_coding	0	0	1	1	1.000
27187	gene-MOCS1P1	**MOCS1P1**	molybdenum cofactor sy	pseudogene	0	0	1	1	1.000
27189	NM_013278	**IL17C**	interleukin 17C	protein_coding	0	0	1	1	1.000
27190	NM_001317987,NM	**IL17B**	interleukin 17B	protein_coding	0	0	1	1	1.000
27196	gene-TRAPPC2P2	**TRAPPC2P2**	trafficking protein particle	pseudogene	0	0	1	1	1.000
27197	NM_080817	**GPR82**	G protein-coupled recept	protein_coding	0	0	1	1	1.000
27198	NM_032554	**HCAR1**	hydroxycarboxylic acid re	protein_coding	0	0	1	1	1.000
27199	NM_001346194,NM	**OXGR1**	oxoglutarate receptor 1	protein_coding	0	0	1	1	1.000
27201	NM_080819,NR_04	**GPR78**	G protein-coupled recept	protein_coding	0	0	1	1	1.000
27202	NM_001271749,NM	**C5AR2**	complement component	protein_coding	0	0	1	1	1.000
27229	NM_001286414,NM	**TUBGCP4**	tubulin gamma complex	protein_coding	25	20	26	21	0.817
27230	NM_014445	**SERP1**	stress associated endopl	protein_coding	146	146	147	147	0.996
27231	NM_001289117,NM	**NMRK2**	nicotinamide riboside kin	protein_coding	0	0	1	1	1.000
27232	NM_001318865,NM	**GNMT**	glycine N-methyltransfer	protein_coding	0	0	1	1	0.849
27233	NM_001321770,NM	**SULT1C4**	sulfotransferase family 1	protein_coding	0	0	1	1	1.000
27235	NM_001358921,NM	**COQ2**	coenzyme Q2, polypreny	protein_coding	11	10	12	11	0.987
27236	NM_001025593,NM	**ARFIP1**	ADP ribosylation factor ir	protein_coding	29	24	30	25	0.830
27237	NM_014448,XM_01	**ARHGEF16**	Rho guanine nucleotide	protein_coding	0	0	1	1	1.000
27238	NM_015698,XM_01	**GPKOW**	G-patch domain and KO	protein_coding	40	38	41	39	0.935
27239	NM_014449,NM_01	**GPR162**	G protein-coupled recept	protein_coding	0	0	1	1	1.000
27240	NM_014450	**SIT1**	signaling threshold regul	protein_coding	0	0	1	1	1.000
27241	NM_001033604,NM	**BBS9**	Bardet-Biedl syndrome 9	protein_coding	1	1	2	2	0.909
27242	NM_014452,XM_01	**TNFRSF21**	TNF receptor superfamily	protein_coding	0	0	1	1	1.000
27243	NM_014453,NM_19	**CHMP2A**	charged multivesicular b	protein_coding	104	127	105	128	1.218
27244	NM_001199933,NM	**SESN1**	sestrin 1	protein_coding	7	4	8	5	0.648
27245	NM_001029882,NM	**AHDC1**	AT-hook DNA binding mo	protein_coding	6	5	7	6	0.895
27246	NM_014455,XM_00	**RNF115**	ring finger protein 115	protein_coding	50	40	51	41	0.801
27247	NM_001002755,NM	**NFU1**	NFU1 iron-sulfur cluster	protein_coding	74	80	75	81	1.072
27248	NM_001127397,NM	**ERLEC1**	endoplasmic reticulum le	protein_coding	34	32	35	33	0.943
27249	NM_015702	**MMADHC**	metabolism of cobalamin	protein_coding	194	172	195	173	0.887
27250	NM_001199492,NM	**PDCD4**	programmed cell death 4	protein_coding	33	30	34	31	0.907
27252	NM_014458,XM_00	**KLHL20**	kelch like family member	protein_coding	16	14	17	15	0.871
27253	NM_001040429,XM	**PCDH17**	protocadherin 17	protein_coding	0	0	1	1	1.000
27254	NM_014460	**CSDC2**	cold shock domain conta	protein_coding	0	0	1	1	1.000

27255	NM_001289080,NM	**CNTN6**	contactin 6	protein_coding	0	0	1	1	1.000
27257	NM_014462,NR_04	**LSM1**	LSM1 homolog, mRNA d	protein_coding	196	191	197	192	0.971
27258	NM_014463	**LSM3**	LSM3 homolog, U6 smal	protein_coding	55	52	56	53	0.949
27283	NM_014464,XM_00	**TINAG**	tubulointerstitial nephritis	protein_coding	0	0	1	1	1.000
27284	NM_014465,XM_00	**SULT1B1**	sulfotransferase family 1	protein_coding	0	0	1	1	1.001
27285	NM_014466,XM_00	**TEKT2**	tektin 2	protein_coding	0	0	1	1	1.074
27286	NM_014467	**SRPX2**	sushi repeat containing p	protein_coding	0	0	1	1	1.000
27287	NM_014468,XM_01	**VENTX**	VENT homeobox	protein_coding	0	0	1	1	1.000
27288	NM_014469	**RBMXL2**	RBMX like 2	protein_coding	0	0	1	1	1.000
27289	NM_014470	**RND1**	Rho family GTPase 1	protein_coding	1	1	2	2	1.084
27290	NM_014471	**SPINK4**	serine peptidase inhibitor	protein_coding	0	0	1	1	1.000
27291	NM_001256619,NM	**R3HCC1L**	R3H domain and coiled-c	protein_coding	20	19	21	20	0.960
27292	NM_001348076,NM	**DIMT1**	DIMT1 rRNA methyltrans	protein_coding	51	47	52	48	0.930
27293	NM_001009568,NM	**SMPDL3B**	sphingomyelin phosphod	protein_coding	0	0	1	1	1.000
27294	NM_014475,XM_00	**DHDH**	dihydrodiol dehydrogena	protein_coding	0	0	1	1	1.000
27295	NM_001114107,NM	**PDLIM3**	PDZ and LIM domain 3	protein_coding	0	0	1	1	1.000
27296	NM_014477,XM_01	**TP53TG5**	TP53 target 5	protein_coding	0	0	1	1	1.000
27297	NM_001040647,NM	**CRCP**	CGRP receptor compone	protein_coding	61	52	62	53	0.862
27299	NM_001145271,NM	**ADAMDEC1**	ADAM like decysin 1	protein_coding	0	0	1	1	1.000
27300	NM_001320767,NM	**ZNF544**	zinc finger protein 544	protein_coding	18	17	19	18	0.953
27301	NM_001271748,NM	**APEX2**	apurinic/apyrimidinic end	protein_coding	41	45	42	46	1.106
27302	NM_014482	**BMP10**	bone morphogenetic pro	protein_coding	0	0	1	1	0.975
27303	NM_001003792,NM	**RBMS3**	RNA binding motif single	protein_coding	1	1	2	2	0.845
27304	NM_014484	**MOCS3**	molybdenum cofactor sy	protein_coding	11	11	12	12	0.997
27306	NM_014485,XM_00	**HPGDS**	hematopoietic prostaglar	protein_coding	0	0	1	1	1.000
27309	NM_001292002,NM	**ZNF330**	zinc finger protein 330	protein_coding	126	105	127	106	0.837
27314	NM_001286059,NM	**RAB30**	RAB30, member RAS on	protein_coding	1	1	2	2	0.816
27315	NM_001145438,NM	**PGAP2**	post-GPI attachment to p	protein_coding	25	30	26	31	1.202
27316	NM_001164803,NM	**RBMX**	RNA binding motif protei	protein_coding	417	418	418	419	1.001
27319	NM_152414	**BHLHE22**	basic helix-loop-helix fam	protein_coding	0	0	1	1	1.000
27321	NR_146582	**TNRC17**	trinucleotide repeat conta	lncRNA	0	0	1	1	1.000
27324	NM_001080430,NM	**TOX3**	TOX high mobility group	protein_coding	0	0	1	1	1.000
27327	NM_001330520,NM	**TNRC6A**	trinucleotide repeat conta	protein_coding	50	42	51	43	0.849
27328	NM_001168360,NM	**PCDH11X**	protocadherin 11 X-linke	protein_coding	0	0	1	1	1.000
27329	NM_014495	**ANGPTL3**	angiopoietin like 3	protein_coding	0	0	1	1	1.000
27330	NM_001330512,NM	**RPS6KA6**	ribosomal protein S6 kina	protein_coding	0	0	1	1	1.000
27332	NM_001014972,NM	**ZNF638**	zinc finger protein 638	protein_coding	35	32	36	33	0.919
27333	NM_001308155,NM	**GOLIM4**	golgi integral membrane	protein_coding	29	27	30	28	0.920
27334	NM_001324218,NM	**P2RY10**	P2Y receptor family mem	protein_coding	22	17	23	18	0.755
27335	NM_001300992,NM	**EIF3K**	eukaryotic translation init	protein_coding	723	769	724	770	1.063
27336	NM_001163280,NM	**HTATSF1**	HIV-1 Tat specific factor	protein_coding	109	102	110	103	0.939
27338	NM_014501,XM_01	**UBE2S**	ubiquitin conjugating enz	protein_coding	371	397	372	398	1.070
27339	NM_014502	**PRPF19**	pre-mRNA processing fa	protein_coding	356	362	357	363	1.017
27340	NM_014503	**UTP20**	UTP20 small subunit pro	protein_coding	36	31	37	32	0.865
27341	NM_015703	**RRP7A**	ribosomal RNA processin	protein_coding	54	57	55	58	1.050
27342	NM_001287060,NM	**RABGEF1**	RAB guanine nucleotide	protein_coding	29	28	30	29	0.957
27343	NM_001174084,NM	**POLL**	DNA polymerase lambda	protein_coding	14	14	15	15	1.045
27344	NM_013271	**PCSK1N**	proprotein convertase su	protein_coding	0	0	1	1	1.000
27345	NM_014505,XM_01	**KCNMB4**	potassium calcium-activa	protein_coding	0	0	1	1	1.000
27346	NM_014573,XM_00	**TMEM97**	transmembrane protein 9	protein_coding	111	109	112	110	0.983
27347	NM_013233,XM_00	**STK39**	serine/threonine kinase 3	protein_coding	93	88	94	89	0.946
27348	NM_001317893,NM	**TOR1B**	torsin family 1 member B	protein_coding	18	18	19	19	1.010
27349	NM_014507,NM_17	**MCAT**	malonyl-CoA-acyl carrier	protein_coding	14	15	15	16	1.062
27350	NM_014508,XM_02	**APOBEC3C**	apolipoprotein B mRNA e	protein_coding	192	195	193	196	1.013
27351	NM_015704,XM_00	**DESI1**	desumoylating isopeptida	protein_coding	69	71	70	72	1.027
27352	NM_001301849,NM	**SGSM3**	small G protein signaling	protein_coding	11	11	12	12	1.045
27429	NM_001321727,NM	**HTRA2**	HtrA serine peptidase 2	protein_coding	51	45	52	46	0.879
27430	NM_013283,NM_18	**MAT2B**	methionine adenosyltran	protein_coding	119	111	120	112	0.933
27433	NM_001085347,NM	**TOR2A**	torsin family 2 member A	protein_coding	25	27	26	28	1.108
27434	NM_001284330,NM	**POLM**	DNA polymerase mu	protein_coding	8	9	9	10	1.131
27436	NM_001145076,NM	**EML4**	EMAP like 4	protein_coding	84	71	85	72	0.848
27437	NR_003607	**HSFY1P1**	HSFY1 pseudogene 1	transcribed_pseu	0	0	1	1	1.000
27439	NM_001163079,NM	**TMEM121B**	transmembrane protein 1	protein_coding	1	1	2	2	1.045
27440	NM_017829,NM_03	**HDHD5**	haloacid dehalogenase l	protein_coding	81	85	82	86	1.048
27442	NR_038398	**CECR3**	cat eye syndrome chrom	lncRNA	0	0	1	1	1.000
27443	NM_001290046,NM	**CECR2**	CECR2 histone acetyl-ly	protein_coding	0	0	1	1	0.992
27445	NM_014510,NM_03	**PCLO**	piccolo presynaptic cytor	protein_coding	0	0	1	1	1.000
28227	NM_013239,NM_01	**PPP2R3B**	protein phosphatase 2 re	protein_coding	6	7	7	8	1.223
28231	NM_016354,XM_00	**SLCO4A1**	solute carrier organic ani	protein_coding	161	156	162	157	0.968
28232	NM_001145044,NM	**SLCO3A1**	solute carrier organic ani	protein_coding	1	1	2	2	0.769
28234	NM_001349920,NM	**SLCO1B3**	solute carrier organic ani	protein_coding	0	0	1	1	1.000
28299	id-IGKV1-5	**IGKV1-5**	immunoglobulin kappa v	V_segment	0	0	1	1	1.000
28316	NM_031891,XM_02	**CDH20**	cadherin 20	protein_coding	0	0	1	1	1.000
28337	id-IGHVIV-44-1	**IGHVIV-44-1**	immunoglobulin heavy va	V_segment_pseu	0	0	1	1	1.000
28338	id-IGHVIII-82	**IGHVIII-82**	immunoglobulin heavy va	V_segment_pseu	0	0	1	1	1.000
28339	id-IGHVIII-76-1	**IGHVIII-76-1**	immunoglobulin heavy va	V_segment_pseu	0	0	1	1	1.000
28340	id-IGHVIII-67-4	**IGHVIII-67-4**	immunoglobulin heavy va	V_segment_pseu	0	0	1	1	1.000
28341	id-IGHVIII-67-3	**IGHVIII-67-3**	immunoglobulin heavy va	V_segment_pseu	0	0	1	1	1.000
28342	id-IGHVIII-67-2	**IGHVIII-67-2**	immunoglobulin heavy va	V_segment_pseu	0	0	1	1	1.000
28343	id-IGHV8-51-1	**IGHV8-51-1**	immunoglobulin heavy va	V_segment_pseu	0	0	1	1	1.000
28344	id-IGHVIII-47-1	**IGHVIII-47-1**	immunoglobulin heavy va	V_segment_pseu	0	0	1	1	1.000
28345	id-IGHVIII-44	**IGHVIII-44**	immunoglobulin heavy va	V_segment_pseu	0	0	1	1	1.000
28346	id-IGHVIII-38-1	**IGHVIII-38-1**	immunoglobulin heavy va	V_segment_pseu	0	0	1	1	1.000
28347	id-IGHVIII-26-1	**IGHVIII-26-1**	immunoglobulin heavy va	V_segment_pseu	0	0	1	1	1.000
28348	id-IGHVIII-25-1	**IGHVIII-25-1**	immunoglobulin heavy va	V_segment_pseu	0	0	1	1	1.000
28349	id-IGHVIII-22-2	**IGHVIII-22-2**	immunoglobulin heavy va	V_segment_pseu	0	0	1	1	1.000
28350	id-IGHVIII-16-1	**IGHVIII-16-1**	immunoglobulin heavy va	V_segment_pseu	0	0	1	1	1.000
28351	id-IGHVIII-13-1	**IGHVIII-13-1**	immunoglobulin heavy va	V_segment_pseu	0	0	1	1	1.000
28352	id-IGHVIII-11-1	**IGHVIII-11-1**	immunoglobulin heavy va	V_segment_pseu	0	0	1	1	1.000
28353	id-IGHVIII-5-2	**IGHVIII-5-2**	immunoglobulin heavy va	V_segment_pseu	0	0	1	1	1.000
28354	id-IGHVIII-5-1	**IGHVIII-5-1**	immunoglobulin heavy va	V_segment_pseu	0	0	1	1	1.000
28355	id-IGHVIII-2-1	**IGHVIII-2-1**	immunoglobulin heavy va	V_segment_pseu	0	0	1	1	1.000
28356	id-IGHVII-78-1	**IGHVII-78-1**	immunoglobulin heavy va	V_segment_pseu	0	0	1	1	1.000
28357	id-IGHVII-74-1	**IGHVII-74-1**	immunoglobulin heavy va	V_segment_pseu	0	0	1	1	1.000
28358	id-IGHVII-67-1	**IGHVII-67-1**	immunoglobulin heavy va	V_segment_pseu	0	0	1	1	1.000
28359	id-IGHVII-65-1	**IGHVII-65-1**	immunoglobulin heavy va	V_segment_pseu	0	0	1	1	1.000
28360	id-IGHVII-62-1	**IGHVII-62-1**	immunoglobulin heavy va	V_segment_pseu	0	0	1	1	1.000
28361	id-IGHVII-60-1	**IGHVII-60-1**	immunoglobulin heavy va	V_segment_pseu	0	0	1	1	1.000
28362	id-IGHVII-53-1	**IGHVII-53-1**	immunoglobulin heavy va	V_segment_pseu	0	0	1	1	1.000
28363	id-IGHVII-51-2	**IGHVII-51-2**	immunoglobulin heavy va	V_segment_pseu	0	0	1	1	1.000
28364	id-IGHVII-49-1	**IGHVII-49-1**	immunoglobulin heavy va	V_segment_pseu	0	0	1	1	1.000
28365	id-IGHVII-46-1	**IGHVII-46-1**	immunoglobulin heavy va	V_segment_pseu	0	0	1	1	1.000
28366	id-IGHVII-44-2	**IGHVII-44-2**	immunoglobulin heavy va	V_segment_pseu	0	0	1	1	1.000
28367	id-IGHVII-43-1	**IGHVII-43-1**	immunoglobulin heavy va	V_segment_pseu	0	0	1	1	1.000
28368	id-IGHVII-40-1	**IGHVII-40-1**	immunoglobulin heavy va	V_segment_pseu	0	0	1	1	1.000
28369	id-IGHVII-33-1	**IGHVII-33-1**	immunoglobulin heavy va	V_segment_pseu	0	0	1	1	1.000
28371	id-IGHVII-30-1	**IGHVII-30-1**	immunoglobulin heavy va	V_segment_pseu	0	0	1	1	1.000
28372	id-IGHVII-28-1	**IGHVII-28-1**	immunoglobulin heavy va	V_segment_pseu	0	0	1	1	1.000
28373	id-IGHVII-26-2	**IGHVII-26-2**	immunoglobulin heavy va	V_segment_pseu	0	0	1	1	1.000
28374	id-IGHVII-22-1	**IGHVII-22-1**	immunoglobulin heavy va	V_segment_pseu	0	0	1	1	1.000
28375	id-IGHVII-20-1	**IGHVII-20-1**	immunoglobulin heavy va	V_segment_pseu	0	0	1	1	1.000
28376	id-IGHVII-15-1	**IGHVII-15-1**	immunoglobulin heavy va	V_segment_pseu	0	0	1	1	1.000
28377	id-IGHVII-1-1	**IGHVII-1-1**	immunoglobulin heavy va	V_segment_pseu	0	0	1	1	1.000
28378	id-IGHV7-81	**IGHV7-81**	immunoglobulin heavy va	V_segment	0	0	1	1	1.000
28380	id-IGHV7-56	**IGHV7-56**	immunoglobulin heavy va	V_segment_pseu	0	0	1	1	1.000
28381	id-IGHV7-40	**IGHV7-40**	immunoglobulin heavy va	V_segment_pseu	0	0	1	1	1.000
28382	id-IGHV7-34-1	**IGHV7-34-1**	immunoglobulin heavy va	V_segment_pseu	0	0	1	1	1.000
28383	id-IGHV7-27	**IGHV7-27**	immunoglobulin heavy va	V_segment_pseu	0	0	1	1	1.000
28385	id-IGHV6-1	**IGHV6-1**	immunoglobulin heavy va	V_segment	0	0	1	1	1.000
28386	id-IGHV5-10-1	**IGHV5-10-1**	immunoglobulin heavy va	V_segment	0	0	1	1	1.000
28387	id-IGHV5-78	**IGHV5-78**	immunoglobulin heavy va	V_segment_pseu	0	0	1	1	1.000
28388	id-IGHV5-51	**IGHV5-51**	immunoglobulin heavy va	V_segment	0	0	1	1	1.000
28390	id-IGHV4-80	**IGHV4-80**	immunoglobulin heavy va	V_segment_pseu	0	0	1	1	1.000
28391	id-IGHV4-61	**IGHV4-61**	immunoglobulin heavy va	V_segment	0	0	1	1	1.000
28392	id-IGHV4-59	**IGHV4-59**	immunoglobulin heavy va	V_segment	0	0	1	1	1.000
28393	id-IGHV4-55	**IGHV4-55**	immunoglobulin heavy va	V_segment_pseu	0	0	1	1	1.000

28394	id-IGHV4-39	IGHV4-39	immunoglobulin heavy va	V_segment	0	0	1	1	1.000
28395	id-IGHV4-34	IGHV4-34	immunoglobulin heavy va	V_segment	0	0	1	1	1.286
28398	id-IGHV4-30-2	IGHV4-30-2	immunoglobulin heavy va	V_segment	0	0	1	1	1.000
28400	id-IGHV4-28	IGHV4-28	immunoglobulin heavy va	V_segment	0	0	1	1	1.000
28401	id-IGHV4-4	IGHV4-4	immunoglobulin heavy va	V_segment	0	0	1	1	1.000
28402	id-IGHV3-69-1	IGHV3-69-1	immunoglobulin heavy va	V_segment_pseu	0	0	1	1	1.000
28405	id-IGHV3-79	IGHV3-79	immunoglobulin heavy va	V_segment_pseu	0	0	1	1	1.000
28406	id-IGHV3-76	IGHV3-76	immunoglobulin heavy va	V_segment_pseu	0	0	1	1	1.000
28407	id-IGHV3-75	IGHV3-75	immunoglobulin heavy va	V_segment_pseu	0	0	1	1	1.000
28408	id-IGHV3-74	IGHV3-74	immunoglobulin heavy va	V_segment	0	0	1	1	1.000
28409	id-IGHV3-73	IGHV3-73	immunoglobulin heavy va	V_segment	0	0	1	1	1.000
28410	id-IGHV3-72	IGHV3-72	immunoglobulin heavy va	V_segment	0	0	1	1	1.000
28411	id-IGHV3-71	IGHV3-71	immunoglobulin heavy va	V_segment_pseu	0	0	1	1	1.000
28412	id-IGHV3-66	IGHV3-66	immunoglobulin heavy va	V_segment	0	0	1	1	1.000
28413	id-IGHV3-65	IGHV3-65	immunoglobulin heavy va	V_segment_pseu	0	0	1	1	1.000
28414	id-IGHV3-64	IGHV3-64	immunoglobulin heavy va	V_segment	0	0	1	1	1.000
28415	id-IGHV3-63	IGHV3-63	immunoglobulin heavy va	V_segment_pseu	0	0	1	1	1.000
28416	id-IGHV3-62	IGHV3-62	immunoglobulin heavy va	V_segment_pseu	0	0	1	1	1.000
28417	id-IGHV3-60	IGHV3-60	immunoglobulin heavy va	V_segment_pseu	0	0	1	1	1.000
28418	id-IGHV3-57	IGHV3-57	immunoglobulin heavy va	V_segment_pseu	0	0	1	1	1.000
28419	id-IGHV3-54	IGHV3-54	immunoglobulin heavy va	V_segment_pseu	0	0	1	1	1.000
28420	id-IGHV3-53	IGHV3-53	immunoglobulin heavy va	V_segment	0	0	1	1	1.000
28421	id-IGHV3-52	IGHV3-52	immunoglobulin heavy va	V_segment_pseu	0	0	1	1	1.000
28422	id-IGHV3-50	IGHV3-50	immunoglobulin heavy va	V_segment_pseu	0	0	1	1	1.000
28423	id-IGHV3-49	IGHV3-49	immunoglobulin heavy va	V_segment	0	0	1	1	1.000
28424	id-IGHV3-48	IGHV3-48	immunoglobulin heavy va	V_segment	0	0	1	1	1.000
28425	id-IGHV3-47	IGHV3-47	immunoglobulin heavy va	V_segment_pseu	0	0	1	1	1.000
28426	id-IGHV3-43	IGHV3-43	immunoglobulin heavy va	V_segment	0	0	1	1	1.000
28427	id-IGHV3-42	IGHV3-42	immunoglobulin heavy va	V_segment_pseu	0	0	1	1	1.000
28428	id-IGHV3-41	IGHV3-41	immunoglobulin heavy va	V_segment_pseu	0	0	1	1	1.000
28429	id-IGHV3-38	IGHV3-38	immunoglobulin heavy va	V_segment	0	0	1	1	1.000
28430	id-IGHV3-37	IGHV3-37	immunoglobulin heavy va	V_segment_pseu	0	0	1	1	1.000
28431	id-IGHV3-36	IGHV3-36	immunoglobulin heavy va	V_segment_pseu	0	0	1	1	1.000
28432	id-IGHV3-35	IGHV3-35	immunoglobulin heavy va	V_segment	0	0	1	1	1.000
28433	id-IGHV3-33-2	IGHV3-33-2	immunoglobulin heavy va	V_segment_pseu	0	0	1	1	1.000
28434	id-IGHV3-33	IGHV3-33	immunoglobulin heavy va	V_segment	0	0	1	1	1.000
28435	id-IGHV3-32	IGHV3-32	immunoglobulin heavy va	V_segment_pseu	0	0	1	1	1.000
28438	id-IGHV3-30-2	IGHV3-30-2	immunoglobulin heavy va	V_segment_pseu	0	0	1	1	1.000
28439	id-IGHV3-30	IGHV3-30	immunoglobulin heavy va	V_segment	0	0	1	1	1.000
28440	id-IGHV3-29	IGHV3-29	immunoglobulin heavy va	V_segment_pseu	0	0	1	1	1.000
28441	id-IGHV3-25	IGHV3-25	immunoglobulin heavy va	V_segment_pseu	0	0	1	1	1.000
28442	id-IGHV3-23	IGHV3-23	immunoglobulin heavy va	V_segment	0	0	1	1	1.000
28443	id-IGHV3-22	IGHV3-22	immunoglobulin heavy va	V_segment_pseu	0	0	1	1	1.000
28444	id-IGHV3-21	IGHV3-21	immunoglobulin heavy va	V_segment	0	0	1	1	1.000
28445	id-IGHV3-20	IGHV3-20	immunoglobulin heavy va	V_segment	0	0	1	1	1.000
28446	id-IGHV3-19	IGHV3-19	immunoglobulin heavy va	V_segment_pseu	0	0	1	1	1.000
28447	id-IGHV3-16	IGHV3-16	immunoglobulin heavy va	V_segment	0	0	1	1	1.000
28448	id-IGHV3-15	IGHV3-15	immunoglobulin heavy va	V_segment	0	0	1	1	1.000
28449	id-IGHV3-13	IGHV3-13	immunoglobulin heavy va	V_segment	0	0	1	1	1.000
28450	id-IGHV3-11	IGHV3-11	immunoglobulin heavy va	V_segment	0	0	1	1	1.000
28452	id-IGHV3-7	IGHV3-7	immunoglobulin heavy va	V_segment	0	0	1	1	1.000
28453	id-IGHV3-6	IGHV3-6	immunoglobulin heavy va	V_segment_pseu	0	0	1	1	1.000
28454	id-IGHV2-70	IGHV2-70	immunoglobulin heavy va	V_segment	0	0	1	1	1.000
28455	id-IGHV2-26	IGHV2-26	immunoglobulin heavy va	V_segment	0	0	1	1	1.000
28457	id-IGHV2-5	IGHV2-5	immunoglobulin heavy va	V_segment	0	0	1	1	1.000
28458	id-IGHV1-69-2	IGHV1-69-2	immunoglobulin heavy va	V_segment	0	0	1	1	1.000
28461	id-IGHV1-69	IGHV1-69	immunoglobulin heavy va	V_segment	0	0	1	1	1.000
28462	id-IGHV1-68	IGHV1-68	immunoglobulin heavy va	V_segment_pseu	0	0	1	1	1.000
28463	id-IGHV1-67	IGHV1-67	immunoglobulin heavy va	V_segment_pseu	0	0	1	1	1.000
28464	id-IGHV1-58	IGHV1-58	immunoglobulin heavy va	V_segment	0	0	1	1	1.000
28465	id-IGHV1-46	IGHV1-46	immunoglobulin heavy va	V_segment	0	0	1	1	1.000
28466	id-IGHV1-45	IGHV1-45	immunoglobulin heavy va	V_segment	0	0	1	1	1.000
28467	id-IGHV1-24	IGHV1-24	immunoglobulin heavy va	V_segment	0	0	1	1	1.000
28468	id-IGHV1-18	IGHV1-18	immunoglobulin heavy va	V_segment	0	0	1	1	1.000
28469	id-IGHV1-17	IGHV1-17	immunoglobulin heavy va	V_segment_pseu	0	0	1	1	1.000
28470	id-IGHV1-14	IGHV1-14	immunoglobulin heavy va	V_segment_pseu	0	0	1	1	1.000
28471	id-IGHV1-12	IGHV1-12	immunoglobulin heavy va	V_segment_pseu	0	0	1	1	1.000
28473	id-IGHV1-3	IGHV1-3	immunoglobulin heavy va	V_segment	3	3	4	4	0.853
28474	id-IGHV1-2	IGHV1-2	immunoglobulin heavy va	V_segment	0	0	1	1	1.000
28475	id-IGHJ6	IGHJ6	immunoglobulin heavy jo	J_segment	0	1	1	2	1.814
28476	id-IGHJ5	IGHJ5	immunoglobulin heavy jo	J_segment	0	0	1	1	1.000
28477	id-IGHJ4	IGHJ4	immunoglobulin heavy jo	J_segment	0	0	1	1	1.000
28478	id-IGHJ3P	IGHJ3P	immunoglobulin heavy jo	J_segment_pseu	0	0	1	1	1.000
28479	id-IGHJ3	IGHJ3	immunoglobulin heavy jo	J_segment	0	0	1	1	1.000
28480	id-IGHJ2P	IGHJ2P	immunoglobulin heavy jo	J_segment_pseu	0	0	1	1	1.000
28481	id-IGHJ2	IGHJ2	immunoglobulin heavy jo	J_segment	0	0	1	1	1.000
28482	id-IGHJ1P	IGHJ1P	immunoglobulin heavy jo	J_segment_pseu	0	0	1	1	1.276
28483	id-IGHJ1	IGHJ1	immunoglobulin heavy jo	J_segment	1	1	2	2	1.392
28484	id-IGHD7-27	IGHD7-27	immunoglobulin heavy d	D_segment	0	0	1	1	1.000
28485	id-IGHD6-25	IGHD6-25	immunoglobulin heavy d	D_segment	0	0	1	1	1.000
28486	id-IGHD6-19	IGHD6-19	immunoglobulin heavy d	D_segment	0	0	1	1	1.000
28487	id-IGHD6-13	IGHD6-13	immunoglobulin heavy d	D_segment	0	0	1	1	1.000
28488	id-IGHD6-6	IGHD6-6	immunoglobulin heavy d	D_segment	0	0	1	1	1.000
28489	id-IGHD5-24	IGHD5-24	immunoglobulin heavy d	D_segment	0	0	1	1	1.000
28490	id-IGHD5-18	IGHD5-18	immunoglobulin heavy d	D_segment	0	0	1	1	1.000
28491	id-IGHD5-12	IGHD5-12	immunoglobulin heavy d	D_segment	0	0	1	1	1.000
28492	id-IGHD5-5	IGHD5-5	immunoglobulin heavy d	D_segment	0	0	1	1	1.000
28493	id-IGHD4-23	IGHD4-23	immunoglobulin heavy d	D_segment	0	0	1	1	1.000
28494	id-IGHD4-17	IGHD4-17	immunoglobulin heavy d	D_segment	0	0	1	1	1.000
28495	id-IGHD4-11	IGHD4-11	immunoglobulin heavy d	D_segment	0	0	1	1	1.000
28496	id-IGHD4-4	IGHD4-4	immunoglobulin heavy d	D_segment	0	0	1	1	1.000
28497	id-IGHD3-22	IGHD3-22	immunoglobulin heavy d	D_segment	0	0	1	1	1.000
28498	id-IGHD3-16	IGHD3-16	immunoglobulin heavy d	D_segment	0	0	1	1	1.000
28499	id-IGHD3-10	IGHD3-10	immunoglobulin heavy d	D_segment	0	0	1	1	1.000
28500	id-IGHD3-9	IGHD3-9	immunoglobulin heavy d	D_segment	0	0	1	1	1.000
28501	id-IGHD3-3	IGHD3-3	immunoglobulin heavy d	D_segment	0	0	1	1	1.000
28502	id-IGHD2-21	IGHD2-21	immunoglobulin heavy d	D_segment	0	0	1	1	1.000
28503	id-IGHD2-15	IGHD2-15	immunoglobulin heavy d	D_segment	0	0	1	1	1.000
28504	id-IGHD2-8	IGHD2-8	immunoglobulin heavy d	D_segment	0	0	1	1	1.000
28505	id-IGHD2-2	IGHD2-2	immunoglobulin heavy d	D_segment	0	0	1	1	1.000
28506	id-IGHD1-26	IGHD1-26	immunoglobulin heavy d	D_segment	0	0	1	1	1.000
28507	id-IGHD1-20	IGHD1-20	immunoglobulin heavy d	D_segment	0	0	1	1	1.000
28508	id-IGHD1-14	IGHD1-14	immunoglobulin heavy d	D_segment	0	0	1	1	1.000
28509	id-IGHD1-7	IGHD1-7	immunoglobulin heavy d	D_segment	0	0	1	1	1.000
28510	id-IGHD1-1	IGHD1-1	immunoglobulin heavy d	D_segment	0	0	1	1	1.000
28511	NM_001001349,NM	NKIRAS2	NFKB inhibitor interactin	protein_coding	59	63	60	64	1.051
28512	NM_001377351,NM	NKIRAS1	NFKB inhibitor interactin	protein_coding	6	5	7	6	0.882
28513	NM_001271028,NM	CDH19	cadherin 19	protein_coding	0	0	1	1	1.000
28514	NM_005618,XM_00	DLL1	delta like canonical Notcl	protein_coding	0	0	1	1	1.027
28516	id-TRDV3	TRDV3	T cell receptor delta varia	V_segment	0	0	1	1	1.000
28517	id-TRDV2	TRDV2	T cell receptor delta varia	V_segment	0	0	1	1	1.000
28518	id-TRDV1	TRDV1	T cell receptor delta varia	V_segment	0	0	1	1	1.000
28519	id-TRDJ4	TRDJ4	T cell receptor delta joini	J_segment	45	42	46	43	0.932
28520	id-TRDJ3	TRDJ3	T cell receptor delta joini	J_segment	28	33	29	34	1.178
28521	id-TRDJ2	TRDJ2	T cell receptor delta joini	J_segment	142	157	143	158	1.103
28522	id-TRDJ1	TRDJ1	T cell receptor delta joini	J_segment	192	204	193	205	1.064
28523	id-TRDD3	TRDD3	T cell receptor delta dive	D_segment	35	39	36	40	1.118
28526	id-TRDC	TRDC	T cell receptor delta cons	C_region	231	253	232	254	1.094
28552	id-TRBV23OR9-2	TRBV23OR9-2	T cell receptor beta varia	V_segment_pseu	0	0	1	1	1.000
28555	id-TRBVB	TRBVB	T cell receptor beta varia	V_segment_pseu	0	0	1	1	1.000
28556	id-TRBVA	TRBVA	T cell receptor beta varia	V_segment_pseu	0	0	1	1	1.000
28557	id-TRBV30	TRBV30	T cell receptor beta varia	V_segment	0	0	1	1	1.000
28558	id-TRBV29-1	TRBV29-1	T cell receptor beta varia	V_segment	0	0	1	1	1.000
28559	id-TRBV28	TRBV28	T cell receptor beta varia	V_segment	0	0	1	1	1.000
28560	id-TRBV27	TRBV27	T cell receptor beta varia	V_segment	0	0	1	1	1.000
28561	id-TRBV26	TRBV26	T cell receptor beta varia	V_segment_pseu	0	0	1	1	1.000

28562	id-TRBV25-1	**TRBV25-1**	T cell receptor beta varia	V_segment	0	0	1	1	1.000
28563	id-TRBV24-1	**TRBV24-1**	T cell receptor beta varia	V_segment	0	0	1	1	1.000
28564	id-TRBV23-1	**TRBV23-1**	T cell receptor beta varia	V_segment_pseu	0	0	1	1	1.000
28565	id-TRBV22-1	**TRBV22-1**	T cell receptor beta varia	V_segment_pseu	0	0	1	1	1.000
28566	id-TRBV21-1	**TRBV21-1**	T cell receptor beta varia	V_segment_pseu	0	0	1	1	1.000
28567	id-TRBV20-1	**TRBV20-1**	T cell receptor beta varia	V_segment	0	0	1	1	1.261
28568	id-TRBV19	**TRBV19**	T cell receptor beta varia	V_segment	0	0	1	1	1.000
28569	id-TRBV18	**TRBV18**	T cell receptor beta varia	V_segment	0	0	1	1	1.000
28570	id-TRBV17	**TRBV17**	T cell receptor beta varia	V_segment	0	0	1	1	1.000
28571	id-TRBV16	**TRBV16**	T cell receptor beta varia	V_segment_pseu	0	0	1	1	1.000
28572	id-TRBV15	**TRBV15**	T cell receptor beta varia	V_segment	0	0	1	1	1.000
28573	id-TRBV14	**TRBV14**	T cell receptor beta varia	V_segment	0	0	1	1	1.000
28574	id-TRBV13	**TRBV13**	T cell receptor beta varia	V_segment	0	0	1	1	0.742
28575	id-TRBV12-5	**TRBV12-5**	T cell receptor beta varia	V_segment	0	0	1	1	1.000
28576	id-TRBV12-4	**TRBV12-4**	T cell receptor beta varia	V_segment	0	0	1	1	1.000
28577	id-TRBV12-3	**TRBV12-3**	T cell receptor beta varia	V_segment	0	0	1	1	1.000
28578	id-TRBV12-2	**TRBV12-2**	T cell receptor beta varia	V_segment_pseu	0	0	1	1	1.000
28579	id-TRBV12-1	**TRBV12-1**	T cell receptor beta varia	V_segment_pseu	0	0	1	1	1.000
28580	id-TRBV11-3	**TRBV11-3**	T cell receptor beta varia	V_segment	0	0	1	1	1.000
28581	id-TRBV11-2	**TRBV11-2**	T cell receptor beta varia	V_segment	0	0	1	1	1.000
28582	id-TRBV11-1	**TRBV11-1**	T cell receptor beta varia	V_segment	0	0	1	1	1.000
28583	id-TRBV10-3	**TRBV10-3**	T cell receptor beta varia	V_segment	0	0	1	1	1.000
28584	id-TRBV10-2	**TRBV10-2**	T cell receptor beta varia	V_segment	0	0	1	1	1.000
28585	id-TRBV10-1	**TRBV10-1**	T cell receptor beta varia	V_segment	0	0	1	1	1.000
28586	id-TRBV9	**TRBV9**	T cell receptor beta varia	V_segment	0	0	1	1	1.000
28587	id-TRBV8-2	**TRBV8-2**	T cell receptor beta varia	V_segment_pseu	0	0	1	1	1.000
28588	id-TRBV8-1	**TRBV8-1**	T cell receptor beta varia	V_segment_pseu	0	0	1	1	1.000
28589	id-TRBV7-9	**TRBV7-9**	T cell receptor beta varia	V_segment	0	0	1	1	1.000
28591	id-TRBV7-7	**TRBV7-7**	T cell receptor beta varia	V_segment	0	0	1	1	1.000
28592	id-TRBV7-6	**TRBV7-6**	T cell receptor beta varia	V_segment	0	0	1	1	1.000
28593	id-TRBV7-5	**TRBV7-5**	T cell receptor beta varia	V_segment_pseu	0	0	1	1	1.000
28594	id-TRBV7-4	**TRBV7-4**	T cell receptor beta varia	V_segment	0	0	1	1	1.000
28595	id-TRBV7-3	**TRBV7-3**	T cell receptor beta varia	V_segment	0	0	1	1	1.000
28596	id-TRBV7-2	**TRBV7-2**	T cell receptor beta varia	V_segment	0	0	1	1	1.000
28597	id-TRBV7-1	**TRBV7-1**	T cell receptor beta varia	V_segment_pseu	0	0	1	1	1.000
28599	id-TRBV6-8	**TRBV6-8**	T cell receptor beta varia	V_segment	0	0	1	1	1.000
28600	id-TRBV6-7	**TRBV6-7**	T cell receptor beta varia	V_segment_pseu	0	0	1	1	1.000
28601	id-TRBV6-6	**TRBV6-6**	T cell receptor beta varia	V_segment	0	0	1	1	1.000
28602	id-TRBV6-5	**TRBV6-5**	T cell receptor beta varia	V_segment	0	0	1	1	1.000
28603	id-TRBV6-4	**TRBV6-4**	T cell receptor beta varia	V_segment	0	0	1	1	1.000
28604	id-TRBV6-3	**TRBV6-3**	T cell receptor beta varia	V_segment	0	0	1	1	1.000
28606	id-TRBV6-1	**TRBV6-1**	T cell receptor beta varia	V_segment	0	0	1	1	1.000
28608	id-TRBV5-7	**TRBV5-7**	T cell receptor beta varia	V_segment	0	0	1	1	1.000
28609	id-TRBV5-6	**TRBV5-6**	T cell receptor beta varia	V_segment	0	0	1	1	1.000
28610	id-TRBV5-5	**TRBV5-5**	T cell receptor beta varia	V_segment	0	0	1	1	1.000
28611	id-TRBV5-4	**TRBV5-4**	T cell receptor beta varia	V_segment	0	0	1	1	1.000
28612	id-TRBV5-3	**TRBV5-3**	T cell receptor beta varia	V_segment_pseu	0	0	1	1	1.000
28613	id-TRBV5-2	**TRBV5-2**	T cell receptor beta varia	V_segment_pseu	0	0	1	1	1.000
28614	id-TRBV5-1	**TRBV5-1**	T cell receptor beta varia	V_segment	0	0	1	1	1.000
28616	id-TRBV4-2	**TRBV4-2**	T cell receptor beta varia	V_segment	0	0	1	1	1.000
28617	id-TRBV4-1	**TRBV4-1**	T cell receptor beta varia	V_segment	0	0	1	1	1.000
28619	id-TRBV3-1	**TRBV3-1**	T cell receptor beta varia	V_segment	0	0	1	1	1.000
28620	id-TRBV2	**TRBV2**	T cell receptor beta varia	V_segment	0	0	1	1	1.000
28621	id-TRBV1	**TRBV1**	T cell receptor beta varia	V_segment_pseu	0	0	1	1	1.000
28622	id-TRBJ2-7	**TRBJ2-7**	T cell receptor beta joinir	J_segment	7	13	8	14	1.663
28623	id-TRBJ2-6	**TRBJ2-6**	T cell receptor beta joinir	J_segment	10	14	11	15	1.408
28624	id-TRBJ2-5	**TRBJ2-5**	T cell receptor beta joinir	J_segment	12	15	13	16	1.230
28625	id-TRBJ2-4	**TRBJ2-4**	T cell receptor beta joinir	J_segment	54	67	55	68	1.245
28626	id-TRBJ2-3	**TRBJ2-3**	T cell receptor beta joinir	J_segment	196	233	197	234	1.186
28627	id-TRBJ2-2P	**TRBJ2-2P**	T cell receptor beta joinir	J_segment_pseu	63	56	64	57	0.890
28628	id-TRBJ2-2	**TRBJ2-2**	T cell receptor beta joinir	J_segment	18	15	19	16	0.884
28629	id-TRBJ2-1	**TRBJ2-1**	T cell receptor beta joinir	J_segment	60	65	61	66	1.075
28630	id-TRBJ1-6	**TRBJ1-6**	T cell receptor beta joinir	J_segment	15	16	16	17	1.107
28631	id-TRBJ1-5	**TRBJ1-5**	T cell receptor beta joinir	J_segment	294	334	295	335	1.138
28632	id-TRBJ1-4	**TRBJ1-4**	T cell receptor beta joinir	J_segment	62	82	63	83	1.333
28633	id-TRBJ1-3	**TRBJ1-3**	T cell receptor beta joinir	J_segment	9	24	10	25	2.508
28634	id-TRBJ1-2	**TRBJ1-2**	T cell receptor beta joinir	J_segment	5	6	6	7	1.197
28635	id-TRBJ1-1	**TRBJ1-1**	T cell receptor beta joinir	J_segment	25	38	26	39	1.490
28636	id-TRBD2	**TRBD2**	T cell receptor beta diver	D_segment	0	0	1	1	1.000
28637	id-TRBD1	**TRBD1**	T cell receptor beta diver	D_segment	5	0	6	1	0.235
28638	id-TRBC2	**TRBC2**	T cell receptor beta cons	C_region	261	302	262	303	1.156
28639	id-TRBC1	**TRBC1**	T cell receptor beta cons	C_region	330	385	331	386	1.168
28640	id-TRAV41	**TRAV41**	T cell receptor alpha vari	V_segment	1	1	2	2	1.163
28641	id-TRAV40	**TRAV40**	T cell receptor alpha vari	V_segment	0	0	1	1	1.000
28642	id-TRAV39	**TRAV39**	T cell receptor alpha vari	V_segment	0	1	1	2	1.188
28643	id-TRAV38-2DV8	**TRAV38-2DV8**	T cell receptor alpha vari	V_segment	0	0	1	1	1.233
28644	id-TRAV38-1	**TRAV38-1**	T cell receptor alpha vari	V_segment	0	0	1	1	1.000
28645	id-TRAV37	**TRAV37**	T cell receptor alpha vari	V_segment_pseu	0	0	1	1	1.000
28646	id-TRAV36DV7	**TRAV36DV7**	T cell receptor alpha vari	V_segment	0	0	1	1	1.000
28647	id-TRAV35	**TRAV35**	T cell receptor alpha vari	V_segment	0	0	1	1	1.000
28648	id-TRAV34	**TRAV34**	T cell receptor alpha vari	V_segment	0	0	1	1	1.000
28649	id-TRAV33	**TRAV33**	T cell receptor alpha vari	V_segment_pseu	0	0	1	1	1.000
28650	id-TRAV32	**TRAV32**	T cell receptor alpha vari	V_segment_pseu	0	0	1	1	1.000
28651	id-TRAV31	**TRAV31**	T cell receptor alpha vari	V_segment_pseu	0	0	1	1	1.000
28652	id-TRAV30	**TRAV30**	T cell receptor alpha vari	V_segment	0	0	1	1	1.000
28653	id-TRAV29DV5	**TRAV29DV5**	T cell receptor alpha vari	V_segment	0	0	1	1	1.000
28654	id-TRAV28	**TRAV28**	T cell receptor alpha vari	V_segment_pseu	0	0	1	1	1.000
28655	id-TRAV27	**TRAV27**	T cell receptor alpha vari	V_segment	0	0	1	1	1.000
28656	id-TRAV26-2	**TRAV26-2**	T cell receptor alpha vari	V_segment	0	0	1	1	1.000
28657	id-TRAV26-1	**TRAV26-1**	T cell receptor alpha vari	V_segment	0	0	1	1	1.000
28658	id-TRAV25	**TRAV25**	T cell receptor alpha vari	V_segment	0	0	1	1	1.000
28659	id-TRAV24	**TRAV24**	T cell receptor alpha vari	V_segment	0	0	1	1	1.000
28660	id-TRAV23DV6	**TRAV23DV6**	T cell receptor alpha vari	V_segment	0	0	1	1	1.000
28661	id-TRAV22	**TRAV22**	T cell receptor alpha vari	V_segment	1	3	2	4	1.870
28662	id-TRAV21	**TRAV21**	T cell receptor alpha vari	V_segment	0	0	1	1	1.000
28663	id-TRAV20	**TRAV20**	T cell receptor alpha vari	V_segment	0	0	1	1	1.000
28664	id-TRAV19	**TRAV19**	T cell receptor alpha vari	V_segment	1	0	2	1	0.723
28665	id-TRAV18	**TRAV18**	T cell receptor alpha vari	V_segment	1	1	2	2	0.961
28666	id-TRAV17	**TRAV17**	T cell receptor alpha vari	V_segment	0	0	1	1	1.000
28667	id-TRAV16	**TRAV16**	T cell receptor alpha vari	V_segment	0	0	1	1	1.122
28668	id-TRAV15	**TRAV15**	T cell receptor alpha vari	V_segment_pseu	0	0	1	1	1.000
28669	id-TRAV14DV4	**TRAV14DV4**	T cell receptor alpha vari	V_segment	2	1	3	2	0.691
28670	id-TRAV13-2	**TRAV13-2**	T cell receptor alpha vari	V_segment	0	2	1	3	2.540
28671	id-TRAV13-1	**TRAV13-1**	T cell receptor alpha vari	V_segment	0	0	1	1	0.858
28672	id-TRAV12-3	**TRAV12-3**	T cell receptor alpha vari	V_segment	0	0	1	1	1.000
28673	id-TRAV12-2	**TRAV12-2**	T cell receptor alpha vari	V_segment	0	0	1	1	1.000
28674	id-TRAV12-1	**TRAV12-1**	T cell receptor alpha vari	V_segment	0	0	1	1	1.000
28675	id-TRAV11	**TRAV11**	T cell receptor alpha vari	V_segment_pseu	0	0	1	1	1.000
28676	id-TRAV10	**TRAV10**	T cell receptor alpha vari	V_segment	0	0	1	1	1.000
28677	id-TRAV9-2	**TRAV9-2**	T cell receptor alpha vari	V_segment	0	0	1	1	1.000
28678	id-TRAV9-1	**TRAV9-1**	T cell receptor alpha vari	V_segment	0	0	1	1	1.000
28679	id-TRAV8-7	**TRAV8-7**	T cell receptor alpha vari	V_segment	0	0	1	1	1.000
28680	id-TRAV8-6	**TRAV8-6**	T cell receptor alpha vari	V_segment	0	0	1	1	1.000
28681	id-TRAV8-5	**TRAV8-5**	T cell receptor alpha vari	V_segment_pseu	0	0	1	1	1.119
28682	id-TRAV8-4	**TRAV8-4**	T cell receptor alpha vari	V_segment	0	0	1	1	1.000
28683	id-TRAV8-3	**TRAV8-3**	T cell receptor alpha vari	V_segment	0	0	1	1	0.877
28684	id-TRAV8-2	**TRAV8-2**	T cell receptor alpha vari	V_segment	0	0	1	1	1.000
28685	id-TRAV8-1	**TRAV8-1**	T cell receptor alpha vari	V_segment	0	0	1	1	1.000
28686	id-TRAV7	**TRAV7**	T cell receptor alpha vari	V_segment	0	0	1	1	1.000
28688	id-TRAV5	**TRAV5**	T cell receptor alpha vari	V_segment	0	0	1	1	1.000
28689	id-TRAV4	**TRAV4**	T cell receptor alpha vari	V_segment	0	0	1	1	1.000
28690	id-TRAV3	**TRAV3**	T cell receptor alpha vari	V_segment	0	0	1	1	1.000
28691	id-TRAV2	**TRAV2**	T cell receptor alpha vari	V_segment	0	0	1	1	1.000
28692	id-TRAV1-2	**TRAV1-2**	T cell receptor alpha vari	V_segment	0	0	1	1	1.000
28693	id-TRAV1-1	**TRAV1-1**	T cell receptor alpha vari	V_segment	0	0	1	1	1.000
28694	id-TRAJ61	**TRAJ61**	T cell receptor alpha join	J_segment	0	0	1	1	1.000

28695	id-TRAJ60	**TRAJ60**	T cell receptor alpha join	J_segment_pseu	0	0	1	1	1.000
28696	id-TRAJ59	**TRAJ59**	T cell receptor alpha join	J_segment	0	0	1	1	1.000
28697	id-TRAJ58	**TRAJ58**	T cell receptor alpha join	J_segment	1	1	2	2	1.199
28698	id-TRAJ57	**TRAJ57**	T cell receptor alpha join	J_segment	0	0	1	1	1.000
28699	id-TRAJ56	**TRAJ56**	T cell receptor alpha join	J_segment	0	0	1	1	1.000
28700	id-TRAJ55	**TRAJ55**	T cell receptor alpha join	J_segment_pseu	0	0	1	1	1.000
28701	id-TRAJ54	**TRAJ54**	T cell receptor alpha join	J_segment	0	0	1	1	1.000
28702	id-TRAJ53	**TRAJ53**	T cell receptor alpha join	J_segment	2	2	3	3	0.822
28703	id-TRAJ52	**TRAJ52**	T cell receptor alpha join	J_segment	1	1	2	2	0.740
28704	id-TRAJ51	**TRAJ51**	T cell receptor alpha join	J_segment_pseu	0	0	1	1	1.000
28705	id-TRAJ50	**TRAJ50**	T cell receptor alpha join	J_segment	0	0	1	1	1.130
28706	id-TRAJ49	**TRAJ49**	T cell receptor alpha join	J_segment	14	8	15	9	0.593
28707	id-TRAJ48	**TRAJ48**	T cell receptor alpha join	J_segment	0	1	1	2	1.733
28708	id-TRAJ47	**TRAJ47**	T cell receptor alpha join	J_segment	10	8	11	9	0.815
28709	id-TRAJ46	**TRAJ46**	T cell receptor alpha join	J_segment	3	2	4	3	0.740
28710	id-TRAJ45	**TRAJ45**	T cell receptor alpha join	J_segment	21	21	22	22	1.029
28711	id-TRAJ44	**TRAJ44**	T cell receptor alpha join	J_segment	13	13	14	14	1.017
28712	id-TRAJ43	**TRAJ43**	T cell receptor alpha join	J_segment	0	2	1	3	2.282
28713	id-TRAJ42	**TRAJ42**	T cell receptor alpha join	J_segment	7	4	8	5	0.636
28714	id-TRAJ41	**TRAJ41**	T cell receptor alpha join	J_segment	0	0	1	1	0.803
28715	id-TRAJ40	**TRAJ40**	T cell receptor alpha join	J_segment	0	0	1	1	1.000
28716	id-TRAJ39	**TRAJ39**	T cell receptor alpha join	J_segment	0	0	1	1	0.886
28717	id-TRAJ38	**TRAJ38**	T cell receptor alpha join	J_segment	0	0	1	1	1.000
28718	id-TRAJ37	**TRAJ37**	T cell receptor alpha join	J_segment	1	0	2	1	0.603
28719	id-TRAJ36	**TRAJ36**	T cell receptor alpha join	J_segment	0	0	1	1	1.000
28720	id-TRAJ35	**TRAJ35**	T cell receptor alpha join	J_segment	0	0	1	1	1.000
28721	id-TRAJ34	**TRAJ34**	T cell receptor alpha join	J_segment	1	0	2	1	0.412
28722	id-TRAJ33	**TRAJ33**	T cell receptor alpha join	J_segment	0	0	1	1	1.496
28723	id-TRAJ32	**TRAJ32**	T cell receptor alpha join	J_segment	0	0	1	1	1.496
28724	id-TRAJ31	**TRAJ31**	T cell receptor alpha join	J_segment	0	0	1	1	1.000
28725	id-TRAJ30	**TRAJ30**	T cell receptor alpha join	J_segment	0	0	1	1	1.000
28726	id-TRAJ29	**TRAJ29**	T cell receptor alpha join	J_segment	0	0	1	1	0.857
28727	id-TRAJ28	**TRAJ28**	T cell receptor alpha join	J_segment	0	0	1	1	1.000
28728	id-TRAJ27	**TRAJ27**	T cell receptor alpha join	J_segment	0	1	1	2	1.985
28729	id-TRAJ26	**TRAJ26**	T cell receptor alpha join	J_segment	0	0	1	1	1.000
28730	id-TRAJ25	**TRAJ25**	T cell receptor alpha join	J_segment	0	0	1	1	1.000
28731	id-TRAJ24	**TRAJ24**	T cell receptor alpha join	J_segment	0	0	1	1	1.000
28732	id-TRAJ23	**TRAJ23**	T cell receptor alpha join	J_segment	0	0	1	1	1.000
28733	id-TRAJ22	**TRAJ22**	T cell receptor alpha join	J_segment	0	0	1	1	1.457
28734	id-TRAJ21	**TRAJ21**	T cell receptor alpha join	J_segment	0	0	1	1	1.000
28735	id-TRAJ20	**TRAJ20**	T cell receptor alpha join	J_segment	0	0	1	1	1.000
28736	id-TRAJ19	**TRAJ19**	T cell receptor alpha join	J_segment	0	0	1	1	1.000
28737	id-TRAJ18	**TRAJ18**	T cell receptor alpha join	J_segment	0	0	1	1	0.880
28738	id-TRAJ17	**TRAJ17**	T cell receptor alpha join	J_segment	0	0	1	1	1.000
28739	id-TRAJ16	**TRAJ16**	T cell receptor alpha join	J_segment	0	0	1	1	1.000
28740	id-TRAJ15	**TRAJ15**	T cell receptor alpha join	J_segment	0	0	1	1	1.000
28741	id-TRAJ14	**TRAJ14**	T cell receptor alpha join	J_segment	0	0	1	1	1.000
28742	id-TRAJ13	**TRAJ13**	T cell receptor alpha join	J_segment	0	0	1	1	1.000
28743	id-TRAJ12	**TRAJ12**	T cell receptor alpha join	J_segment	8	5	9	6	0.621
28744	id-TRAJ11	**TRAJ11**	T cell receptor alpha join	J_segment	0	0	1	1	1.000
28745	id-TRAJ10	**TRAJ10**	T cell receptor alpha join	J_segment	1	1	2	2	0.830
28746	id-TRAJ9	**TRAJ9**	T cell receptor alpha join	J_segment	0	0	1	1	0.732
28747	id-TRAJ8	**TRAJ8**	T cell receptor alpha join	J_segment	0	0	1	1	1.000
28748	id-TRAJ7	**TRAJ7**	T cell receptor alpha join	J_segment	0	0	1	1	1.000
28749	id-TRAJ6	**TRAJ6**	T cell receptor alpha join	J_segment	0	0	1	1	1.000
28750	id-TRAJ5	**TRAJ5**	T cell receptor alpha join	J_segment	0	2	1	3	2.778
28751	id-TRAJ4	**TRAJ4**	T cell receptor alpha join	J_segment	0	1	1	2	1.646
28752	id-TRAJ3	**TRAJ3**	T cell receptor alpha join	J_segment	0	0	1	1	1.000
28753	id-TRAJ2	**TRAJ2**	T cell receptor alpha join	J_segment	0	0	1	1	0.751
28754	id-TRAJ1	**TRAJ1**	T cell receptor alpha join	J_segment	0	0	1	1	1.000
28755	id-TRAC	**TRAC**	T cell receptor alpha con	C_region	75	69	76	70	0.914
28757	id-IGLVV-66	**IGLVV-66**	immunoglobulin lambda	V_segment_pseu	0	0	1	1	1.000
28758	id-IGLVV-58	**IGLVV-58**	immunoglobulin lambda	V_segment_pseu	0	0	1	1	1.000
28759	id-IGLVIV-65	**IGLVIV-65**	immunoglobulin lambda	V_segment_pseu	0	0	1	1	1.000
28760	id-IGLVIV-64	**IGLVIV-64**	immunoglobulin lambda	V_segment_pseu	0	0	1	1	1.000
28761	id-IGLVIV-59	**IGLVIV-59**	immunoglobulin lambda	V_segment_pseu	0	0	1	1	1.000
28762	id-IGLVIV-53	**IGLVIV-53**	immunoglobulin lambda	V_segment_pseu	0	0	1	1	1.000
28763	id-IGLVI-70	**IGLVI-70**	immunoglobulin lambda	V_segment_pseu	0	0	1	1	1.000
28764	id-IGLVI-68	**IGLVI-68**	immunoglobulin lambda	V_segment_pseu	0	0	1	1	1.000
28765	id-IGLVI-63	**IGLVI-63**	immunoglobulin lambda	V_segment_pseu	0	0	1	1	1.000
28766	id-IGLVI-56	**IGLVI-56**	immunoglobulin lambda	V_segment_pseu	0	0	1	1	1.000
28767	id-IGLVI-42	**IGLVI-42**	immunoglobulin lambda	V_segment_pseu	0	0	1	1	1.000
28768	id-IGLVI-38	**IGLVI-38**	immunoglobulin lambda	V_segment_pseu	0	0	1	1	1.000
28769	id-IGLVI-20	**IGLVI-20**	immunoglobulin lambda	V_segment_pseu	0	0	1	1	1.000
28770	id-IGLV11-55	**IGLV11-55**	immunoglobulin lambda	V_segment	0	0	1	1	1.000
28771	id-IGLV10-67	**IGLV10-67**	immunoglobulin lambda	V_segment_pseu	0	0	1	1	1.000
28772	id-IGLV10-54	**IGLV10-54**	immunoglobulin lambda	V_segment	0	0	1	1	1.000
28773	id-IGLV9-49	**IGLV9-49**	immunoglobulin lambda	V_segment	0	0	1	1	1.000
28774	id-IGLV8-61	**IGLV8-61**	immunoglobulin lambda	V_segment	0	0	1	1	1.000
28775	id-IGLV7-46	**IGLV7-46**	immunoglobulin lambda	V_segment	0	0	1	1	1.000
28776	id-IGLV7-43	**IGLV7-43**	immunoglobulin lambda	V_segment	0	0	1	1	1.000
28777	id-IGLV7-35	**IGLV7-35**	immunoglobulin lambda	V_segment_pseu	0	0	1	1	1.000
28778	id-IGLV6-57	**IGLV6-57**	immunoglobulin lambda	V_segment	0	0	1	1	1.000
28779	id-IGLV5-52	**IGLV5-52**	immunoglobulin lambda	V_segment	0	0	1	1	1.000
28780	id-IGLV5-48	**IGLV5-48**	immunoglobulin lambda	V_segment	0	0	1	1	1.000
28781	id-IGLV5-45	**IGLV5-45**	immunoglobulin lambda	V_segment	0	0	1	1	1.000
28783	id-IGLV5-37	**IGLV5-37**	immunoglobulin lambda	V_segment	0	0	1	1	1.000
28784	id-IGLV4-69	**IGLV4-69**	immunoglobulin lambda	V_segment	0	0	1	1	1.000
28785	id-IGLV4-60	**IGLV4-60**	immunoglobulin lambda	V_segment	0	0	1	1	1.000
28786	id-IGLV4-3	**IGLV4-3**	immunoglobulin lambda	V_segment	0	0	1	1	1.000
28787	id-IGLV3-32	**IGLV3-32**	immunoglobulin lambda	V_segment	0	0	1	1	1.000
28788	id-IGLV3-31	**IGLV3-31**	immunoglobulin lambda	V_segment_pseu	0	0	1	1	1.000
28789	id-IGLV3-30	**IGLV3-30**	immunoglobulin lambda	V_segment_pseu	0	0	1	1	1.000
28790	id-IGLV3-29	**IGLV3-29**	immunoglobulin lambda	V_segment_pseu	0	0	1	1	1.000
28791	id-IGLV3-27	**IGLV3-27**	immunoglobulin lambda	V_segment	0	0	1	1	1.000
28792	id-IGLV3-26	**IGLV3-26**	immunoglobulin lambda	V_segment_pseu	0	0	1	1	1.000
28793	id-IGLV3-25	**IGLV3-25**	immunoglobulin lambda	V_segment	0	0	1	1	1.000
28794	id-IGLV3-24	**IGLV3-24**	immunoglobulin lambda	V_segment_pseu	0	0	1	1	1.000
28795	id-IGLV3-22	**IGLV3-22**	immunoglobulin lambda	V_segment	0	0	1	1	1.000
28796	id-IGLV3-21	**IGLV3-21**	immunoglobulin lambda	V_segment	0	0	1	1	1.000
28797	id-IGLV3-19	**IGLV3-19**	immunoglobulin lambda	V_segment	0	0	1	1	1.000
28798	id-IGLV3-17	**IGLV3-17**	immunoglobulin lambda	V_segment_pseu	0	0	1	1	1.000
28799	id-IGLV3-16	**IGLV3-16**	immunoglobulin lambda	V_segment	0	0	1	1	1.000
28800	id-IGLV3-15	**IGLV3-15**	immunoglobulin lambda	V_segment_pseu	0	0	1	1	1.000
28801	id-IGLV3-13	**IGLV3-13**	immunoglobulin lambda	V_segment_pseu	0	0	1	1	1.000
28802	id-IGLV3-12	**IGLV3-12**	immunoglobulin lambda	V_segment	0	0	1	1	1.000
28803	id-IGLV3-10	**IGLV3-10**	immunoglobulin lambda	V_segment	0	0	1	1	1.000
28804	id-IGLV3-9	**IGLV3-9**	immunoglobulin lambda	V_segment	0	0	1	1	1.000
28805	id-IGLV3-7	**IGLV3-7**	immunoglobulin lambda	V_segment_pseu	0	0	1	1	1.000
28806	id-IGLV3-6	**IGLV3-6**	immunoglobulin lambda	V_segment_pseu	0	0	1	1	1.000
28807	id-IGLV3-4	**IGLV3-4**	immunoglobulin lambda	V_segment_pseu	0	0	1	1	1.000
28808	id-IGLV3-2	**IGLV3-2**	immunoglobulin lambda	V_segment_pseu	0	0	1	1	1.000
28809	id-IGLV3-1	**IGLV3-1**	immunoglobulin lambda	V_segment	0	0	1	1	1.000
28810	id-IGLV2-34	**IGLV2-34**	immunoglobulin lambda	V_segment_pseu	0	0	1	1	1.000
28811	id-IGLV2-33	**IGLV2-33**	immunoglobulin lambda	V_segment	0	0	1	1	1.000
28812	id-IGLV2-28	**IGLV2-28**	immunoglobulin lambda	V_segment_pseu	0	0	1	1	1.000
28813	id-IGLV2-23	**IGLV2-23**	immunoglobulin lambda	V_segment	0	0	1	1	1.000
28814	id-IGLV2-18	**IGLV2-18**	immunoglobulin lambda	V_segment	0	0	1	1	1.000
28815	id-IGLV2-14	**IGLV2-14**	immunoglobulin lambda	V_segment	0	0	1	1	1.000
28816	id-IGLV2-11	**IGLV2-11**	immunoglobulin lambda	V_segment	0	0	1	1	1.000
28817	id-IGLV2-8	**IGLV2-8**	immunoglobulin lambda	V_segment	0	0	1	1	1.000
28818	id-IGLV2-5	**IGLV2-5**	immunoglobulin lambda	V_segment_pseu	0	0	1	1	1.000
28819	id-IGLV1-62	**IGLV1-62**	immunoglobulin lambda	V_segment_pseu	0	0	1	1	1.000
28820	id-IGLV1-51	**IGLV1-51**	immunoglobulin lambda	V_segment	0	0	1	1	0.956
28821	id-IGLV1-50	**IGLV1-50**	immunoglobulin lambda	V_segment	0	0	1	1	1.000
28822	id-IGLV1-47	**IGLV1-47**	immunoglobulin lambda	V_segment	0	0	1	1	1.000

28823	id-IGLV1-44	**IGLV1-44**	immunoglobulin lambda	V_segment	0	0	1	1	1.000
28824	id-IGLV1-41	**IGLV1-41**	immunoglobulin lambda	V_segment_pseu	0	0	1	1	1.000
28825	id-IGLV1-40	**IGLV1-40**	immunoglobulin lambda	V_segment	0	0	1	1	1.000
28826	id-IGLV1-36	**IGLV1-36**	immunoglobulin lambda	V_segment	0	0	1	1	1.000
28827	id-IGLJ7	**IGLJ7**	immunoglobulin lambda	J_segment	0	0	1	1	1.000
28828	id-IGLJ6	**IGLJ6**	immunoglobulin lambda	J_segment	0	0	1	1	1.000
28829	id-IGLJ5	**IGLJ5**	immunoglobulin lambda	J_segment	0	0	1	1	1.000
28830	id-IGLJ4	**IGLJ4**	immunoglobulin lambda	J_segment	0	0	1	1	1.000
28831	id-IGLJ3	**IGLJ3**	immunoglobulin lambda	J_segment	0	0	1	1	1.000
28832	id-IGLJ2	**IGLJ2**	immunoglobulin lambda	J_segment	0	0	1	1	1.000
28833	id-IGLJ1	**IGLJ1**	immunoglobulin lambda	J_segment	0	0	1	1	1.000
28834	id-IGLC7	**IGLC7**	immunoglobulin lambda	C_region	0	0	1	1	1.000
28869	id-IGKV6D-41	**IGKV6D-41**	immunoglobulin kappa v	V_segment	0	0	1	1	1.000
28870	id-IGKV6D-21	**IGKV6D-21**	immunoglobulin kappa v	V_segment	0	0	1	1	1.000
28871	id-IGKV3D-34	**IGKV3D-34**	immunoglobulin kappa v	V_segment_pseu	0	0	1	1	1.000
28872	id-IGKV3D-31	**IGKV3D-31**	immunoglobulin kappa v	V_segment_pseu	0	0	1	1	1.000
28873	id-IGKV3D-25	**IGKV3D-25**	immunoglobulin kappa v	V_segment_pseu	0	0	1	1	1.000
28874	id-IGKV3D-20	**IGKV3D-20**	immunoglobulin kappa v	V_segment	0	0	1	1	1.000
28875	id-IGKV3D-15	**IGKV3D-15**	immunoglobulin kappa v	V_segment	0	0	1	1	1.000
28876	id-IGKV3D-11	**IGKV3D-11**	immunoglobulin kappa v	V_segment	0	0	1	1	1.000
28877	id-IGKV3D-7	**IGKV3D-7**	immunoglobulin kappa v	V_segment	0	0	1	1	1.000
28878	id-IGKV2D-40	**IGKV2D-40**	immunoglobulin kappa v	V_segment	0	0	1	1	1.000
28879	id-IGKV2D-38	**IGKV2D-38**	immunoglobulin kappa v	V_segment_pseu	0	0	1	1	1.000
28880	id-IGKV2D-36	**IGKV2D-36**	immunoglobulin kappa v	V_segment_pseu	0	0	1	1	1.000
28881	id-IGKV2D-30	**IGKV2D-30**	immunoglobulin kappa v	V_segment	0	0	1	1	1.000
28882	id-IGKV2D-29	**IGKV2D-29**	immunoglobulin kappa v	V_segment	0	0	1	1	1.000
28883	id-IGKV2D-28	**IGKV2D-28**	immunoglobulin kappa v	V_segment	0	0	1	1	1.000
28884	id-IGKV2D-26	**IGKV2D-26**	immunoglobulin kappa v	V_segment_pseu	0	0	1	1	1.000
28885	id-IGKV2D-24	**IGKV2D-24**	immunoglobulin kappa v	V_segment	0	0	1	1	1.000
28886	id-IGKV2D-23	**IGKV2D-23**	immunoglobulin kappa v	V_segment_pseu	0	0	1	1	1.000
28887	id-IGKV2D-19	**IGKV2D-19**	immunoglobulin kappa v	V_segment_pseu	0	0	1	1	1.000
28888	id-IGKV2D-18	**IGKV2D-18**	immunoglobulin kappa v	V_segment_pseu	0	0	1	1	1.000
28889	id-IGKV2D-14	**IGKV2D-14**	immunoglobulin kappa v	V_segment_pseu	0	0	1	1	1.000
28890	id-IGKV2D-10	**IGKV2D-10**	immunoglobulin kappa v	V_segment_pseu	0	0	1	1	1.000
28891	id-IGKV1D-43	**IGKV1D-43**	immunoglobulin kappa v	V_segment	0	0	1	1	1.000
28892	id-IGKV1D-42	**IGKV1D-42**	immunoglobulin kappa v	V_segment	0	0	1	1	1.000
28893	id-IGKV1D-39	**IGKV1D-39**	immunoglobulin kappa v	V_segment	0	0	1	1	1.000
28894	id-IGKV1D-37	**IGKV1D-37**	immunoglobulin kappa v	V_segment	0	0	1	1	1.000
28895	id-IGKV1D-35	**IGKV1D-35**	immunoglobulin kappa v	V_segment_pseu	0	0	1	1	1.000
28896	id-IGKV1D-33	**IGKV1D-33**	immunoglobulin kappa v	V_segment	0	0	1	1	1.000
28897	id-IGKV1D-32	**IGKV1D-32**	immunoglobulin kappa v	V_segment_pseu	0	0	1	1	1.000
28898	id-IGKV1D-27	**IGKV1D-27**	immunoglobulin kappa v	V_segment_pseu	0	0	1	1	1.000
28899	id-IGKV1D-22	**IGKV1D-22**	immunoglobulin kappa v	V_segment_pseu	0	0	1	1	1.000
28900	id-IGKV1D-17	**IGKV1D-17**	immunoglobulin kappa v	V_segment	0	0	1	1	1.000
28901	id-IGKV1D-16	**IGKV1D-16**	immunoglobulin kappa v	V_segment	0	0	1	1	1.000
28902	id-IGKV1D-13	**IGKV1D-13**	immunoglobulin kappa v	V_segment	0	0	1	1	1.000
28903	id-IGKV1D-12	**IGKV1D-12**	immunoglobulin kappa v	V_segment	0	0	1	1	1.000
28904	id-IGKV1D-8	**IGKV1D-8**	immunoglobulin kappa v	V_segment	0	0	1	1	1.000
28905	id-IGKV7-3	**IGKV7-3**	immunoglobulin kappa v	V_segment_pseu	0	0	1	1	1.000
28906	id-IGKV6-21	**IGKV6-21**	immunoglobulin kappa v	V_segment	0	0	1	1	1.000
28907	id-IGKV5-2	**IGKV5-2**	immunoglobulin kappa v	V_segment	0	0	1	1	1.000
28908	id-IGKV4-1	**IGKV4-1**	immunoglobulin kappa v	V_segment	0	0	1	1	1.000
28909	id-IGKV3-34	**IGKV3-34**	immunoglobulin kappa v	V_segment_pseu	0	0	1	1	1.000
28910	id-IGKV3-31	**IGKV3-31**	immunoglobulin kappa v	V_segment_pseu	0	0	1	1	1.000
28911	id-IGKV3-25	**IGKV3-25**	immunoglobulin kappa v	V_segment_pseu	0	0	1	1	1.000
28912	id-IGKV3-20	**IGKV3-20**	immunoglobulin kappa v	V_segment	0	0	1	1	1.000
28913	id-IGKV3-15	**IGKV3-15**	immunoglobulin kappa v	V_segment	0	0	1	1	1.000
28914	id-IGKV3-11	**IGKV3-11**	immunoglobulin kappa v	V_segment	0	0	1	1	1.000
28915	id-IGKV3-7	**IGKV3-7**	immunoglobulin kappa v	V_segment	0	0	1	1	1.000
28916	id-IGKV2-40	**IGKV2-40**	immunoglobulin kappa v	V_segment	0	0	1	1	1.000
28917	id-IGKV2-38	**IGKV2-38**	immunoglobulin kappa v	V_segment_pseu	0	0	1	1	1.000
28918	id-IGKV2-36	**IGKV2-36**	immunoglobulin kappa v	V_segment_pseu	0	0	1	1	1.000
28919	id-IGKV2-30	**IGKV2-30**	immunoglobulin kappa v	V_segment	0	0	1	1	1.000
28920	id-IGKV2-29	**IGKV2-29**	immunoglobulin kappa v	V_segment_pseu	0	0	1	1	1.000
28921	id-IGKV2-28	**IGKV2-28**	immunoglobulin kappa v	V_segment	0	0	1	1	1.000
28922	id-IGKV2-26	**IGKV2-26**	immunoglobulin kappa v	V_segment_pseu	0	0	1	1	1.000
28923	id-IGKV2-24	**IGKV2-24**	immunoglobulin kappa v	V_segment	0	0	1	1	1.000
28924	id-IGKV2-23	**IGKV2-23**	immunoglobulin kappa v	V_segment_pseu	0	0	1	1	1.000
28925	id-IGKV2-19	**IGKV2-19**	immunoglobulin kappa v	V_segment_pseu	0	0	1	1	1.000
28926	id-IGKV2-18	**IGKV2-18**	immunoglobulin kappa v	V_segment_pseu	0	0	1	1	1.000
28927	id-IGKV2-14	**IGKV2-14**	immunoglobulin kappa v	V_segment_pseu	0	0	1	1	1.000
28928	id-IGKV2-10	**IGKV2-10**	immunoglobulin kappa v	V_segment_pseu	0	0	1	1	1.000
28929	id-IGKV2-4	**IGKV2-4**	immunoglobulin kappa v	V_segment_pseu	0	0	1	1	1.000
28930	id-IGKV1-39	**IGKV1-39**	immunoglobulin kappa v	V_segment	0	0	1	1	1.000
28931	id-IGKV1-37	**IGKV1-37**	immunoglobulin kappa v	V_segment	0	0	1	1	1.000
28932	id-IGKV1-35	**IGKV1-35**	immunoglobulin kappa v	V_segment_pseu	0	0	1	1	1.000
28933	id-IGKV1-33	**IGKV1-33**	immunoglobulin kappa v	V_segment	0	0	1	1	1.000
28934	id-IGKV1-32	**IGKV1-32**	immunoglobulin kappa v	V_segment_pseu	0	0	1	1	1.000
28935	id-IGKV1-27	**IGKV1-27**	immunoglobulin kappa v	V_segment	0	0	1	1	1.000
28936	id-IGKV1-22	**IGKV1-22**	immunoglobulin kappa v	V_segment_pseu	0	0	1	1	1.000
28937	id-IGKV1-17	**IGKV1-17**	immunoglobulin kappa v	V_segment	0	0	1	1	1.000
28938	id-IGKV1-16	**IGKV1-16**	immunoglobulin kappa v	V_segment	0	0	1	1	1.000
28939	id-IGKV1-13	**IGKV1-13**	immunoglobulin kappa v	V_segment	0	0	1	1	1.000
28940	id-IGKV1-12	**IGKV1-12**	immunoglobulin kappa v	V_segment	0	0	1	1	1.000
28941	id-IGKV1-9	**IGKV1-9**	immunoglobulin kappa v	V_segment	0	0	1	1	1.000
28942	id-IGKV1-8	**IGKV1-8**	immunoglobulin kappa v	V_segment	0	0	1	1	1.000
28943	id-IGKV1-6	**IGKV1-6**	immunoglobulin kappa v	V_segment	0	0	1	1	1.000
28946	id-IGKJ5	**IGKJ5**	immunoglobulin kappa j	J_segment	0	0	1	1	1.000
28947	id-IGKJ4	**IGKJ4**	immunoglobulin kappa j	J_segment	0	0	1	1	1.000
28948	id-IGKJ3	**IGKJ3**	immunoglobulin kappa j	J_segment	0	0	1	1	1.000
28949	id-IGKJ2	**IGKJ2**	immunoglobulin kappa j	J_segment	0	0	1	1	1.000
28950	id-IGKJ1	**IGKJ1**	immunoglobulin kappa j	J_segment	0	0	1	1	1.000
28951	NM_021643,NR_02	**TRIB2**	tribbles pseudokinase 2	protein_coding	11	9	12	10	0.838
28952	NM_014008,XM_00	**CCDC22**	coiled-coil domain contai	protein_coding	14	16	15	17	1.126
28954	NM_014012,XM_00	**REM1**	RRAD and GEM like GT	protein_coding	0	0	1	1	1.000
28955	NM_014015	**DEXI**	Dexi homolog	protein_coding	3	4	4	5	1.451
28956	NM_001145264,NM	**LAMTOR2**	late endosomal/lysosom	protein_coding	117	124	118	125	1.058
28957	NM_014018	**MRPS28**	mitochondrial ribosomal	protein_coding	88	82	89	83	0.939
28958	NM_001040431	**COA3**	cytochrome c oxidase as	protein_coding	113	112	114	113	0.985
28959	NM_001101311,NM	**TMEM176B**	transmembrane protein	protein_coding	0	0	1	1	1.000
28960	NM_001350236,NM	**DCPS**	decapping enzyme, scav	protein_coding	30	31	31	32	1.034
28962	NM_014028,XR_94	**OSTM1**	osteoclastogenesis asso	protein_coding	29	25	30	26	0.857
28964	NM_001085454,NM	**GIT1**	GIT ArfGAP 1	protein_coding	67	56	68	57	0.837
28965	NM_001017372,NM	**SLC27A6**	solute carrier family 27 m	protein_coding	0	0	1	1	1.000
28966	NM_014035,NR_14	**SNX24**	sorting nexin 24	protein_coding	0	0	1	1	1.000
28968	NM_014037,XM_00	**SLC6A16**	solute carrier family 6 me	protein_coding	1	1	2	2	1.026
28969	NM_001159767,NM	**BZW2**	basic leucine zipper and	protein_coding	134	140	135	141	1.044
28970	NM_001286067,NM	**C11orf54**	chromosome 11 open rea	protein_coding	14	10	15	11	0.741
28971	NM_001316957,NM	**AAMDC**	adipogenesis associated	protein_coding	20	16	21	17	0.791
28972	NM_014041	**SPCS1**	signal peptidase comple	protein_coding	56	55	57	56	0.984
28973	NM_014046,XM_02	**MRPS18B**	mitochondrial ribosomal	protein_coding	137	129	138	130	0.942
28974	NM_014047	**C19orf53**	chromosome 19 open rea	protein_coding	165	192	166	193	1.162
28976	NM_014049,NR_03	**ACAD9**	acyl-CoA dehydrogenase	protein_coding	68	62	69	63	0.919
28977	NM_014050,NM_17	**MRPL42**	mitochondrial ribosomal	protein_coding	16	14	17	15	0.872
28978	NM_014051,XM_02	**TMEM14A**	transmembrane protein	protein_coding	65	63	66	64	0.965
28981	NM_001143779,NM	**IFT81**	intraflagellar transport 81	protein_coding	11	8	12	9	0.751
28982	NM_014053,XM_01	**FLVCR1**	FLVCR heme transporte	protein_coding	16	16	17	17	0.993
28983	NM_014058,XM_01	**TMPRSS11E**	transmembrane serine p	protein_coding	0	0	1	1	1.000
28984	NM_014059,XR_00	**RGCC**	regulator of cell cycle	protein_coding	0	0	1	1	1.000
28985	NM_001137554,NM	**MCTS1**	MCTS1 re-initiation and	protein_coding	17	14	18	15	0.855
28986	NM_014061	**MAGEH1**	MAGE family member H	protein_coding	2	3	3	4	1.096
28987	NM_014062,NR_07	**NOB1**	NIN1 (RPN12) binding p	protein_coding	129	145	130	146	1.118
28988	NM_001014436,NM	**DBNL**	drebrin like	protein_coding	26	32	27	33	1.244
28989	NM_001286796,NM	**NTMT1**	N-terminal Xaa-Pro-Lys	protein_coding	41	37	42	38	0.900
28990	NM_001288950,NM	**ASTE1**	asteroid homolog 1	protein_coding	11	11	12	12	0.994
28991	NM_001081003,NM	**COMMD5**	COMM domain containin	protein_coding	24	22	25	23	0.931

28992	NM_014067,XM_00!	**MACROD1**	mono-ADP ribosylhydrol	protein_coding	4	3	5	4	0.853
28996	NM_001113239,NM	**HIPK2**	homeodomain interacting	protein_coding	0	0	1	1	1.000
28998	NM_014078	**MRPL13**	mitochondrial ribosomal	protein_coding	163	150	164	151	0.922
28999	NM_014079,XM_00!	**KLF15**	Kruppel like factor 15	protein_coding	0	0	1	1	1.000
29015	NM_001278201,NM	**SLC43A3**	solute carrier family 43 m	protein_coding	460	465	461	466	1.012
29018	NR_024620	**FOXN3-AS2**	FOXN3 antisense RNA 2	lncRNA	0	0	1	1	1.000
29028	NM_014109,XM_01	**ATAD2**	ATPase family AAA doma	protein_coding	134	115	135	116	0.862
29034	NR_002763	**CPS1-IT1**	CPS1 intronic transcript	lncRNA	0	0	1	1	1.000
29035	NM_014117,XM_01	**C16orf72**	chromosome 16 open rea	protein_coding	56	56	57	57	0.992
29057	NM_001242489,NM	**FAM156A**	family with sequence sim	protein_coding	14	16	15	17	1.123
29058	NM_001009923,NM	**TMEM230**	transmembrane protein 2	protein_coding	179	166	180	167	0.925
29062	NM_001362736,NM	**WDR91**	WD repeat domain 91	protein_coding	3	3	4	4	1.110
29063	NM_001318148,NM	**ZCCHC4**	zinc finger CCHC-type co	protein_coding	17	16	18	17	0.918
29065	NR_002765	**ASAP1-IT1**	ASAP1 intronic transcript	lncRNA	0	0	1	1	1.000
29066	NM_014153,XM_00	**ZC3H7A**	zinc finger CCCH-type co	protein_coding	43	38	44	39	0.882
29068	NM_001301098,NM	**ZBTB44**	zinc finger and BTB dom	protein_coding	18	15	19	16	0.854
29070	NM_001142302,NM	**CCDC113**	coiled-coil domain contai	protein_coding	0	0	1	1	1.067
29071	NM_001011551,NM	**C1GALT1C1**	C1GALT1 specific chape	protein_coding	18	18	19	19	0.975
29072	NM_001349370,NM	**SETD2**	SET domain containing 2	protein_coding	39	31	40	32	0.810
29074	NM_001318817,NM	**MRPL18**	mitochondrial ribosomal	protein_coding	214	214	215	215	0.998
29075	NR_026883,NR_02	**LINC00652**	long intergenic non-prote	lncRNA	0	0	1	1	1.147
29078	NM_014165	**NDUFAF4**	NADH:ubiquinone oxidor	protein_coding	39	36	40	37	0.931
29079	NM_001270629,NM	**MED4**	mediator complex subun	protein_coding	81	80	82	81	0.984
29080	NM_014167,NR_03	**CCDC59**	coiled-coil domain contai	protein_coding	55	55	56	56	1.012
29081	NM_001293186,NM	**METTL5**	methyltransferase like 5	protein_coding	180	182	181	183	1.010
29082	NM_014169	**CHMP4A**	charged multivesicular b	protein_coding	90	97	91	98	1.078
29083	NM_014170,NM_13	**GTPBP8**	GTP binding protein 8 (p	protein_coding	99	87	100	88	0.885
29085	NM_001135861,NM	**PHPT1**	phosphohistidine phosph	protein_coding	93	90	94	91	0.968
29086	NM_001033549,NM	**BABAM1**	BRISC and BRCA1 A cor	protein_coding	159	164	160	165	1.029
29087	NM_001037304,NM	**THYN1**	thymocyte nuclear protei	protein_coding	153	155	154	156	1.012
29088	NM_014175,XM_01	**MRPL15**	mitochondrial ribosomal	protein_coding	166	165	167	166	0.989
29089	NM_001310326,NM	**UBE2T**	ubiquitin conjugating enz	protein_coding	164	166	165	167	1.009
29090	NM_014177,XM_00!	**TIMM21**	translocase of inner mito	protein_coding	48	47	49	48	0.970
29091	NM_001304476,NM	**STXBP6**	syntaxin binding protein	protein_coding	0	0	1	1	1.026
29092	NR_023918,NR_02	**LINC00339**	long intergenic non-prote	lncRNA	6	11	7	12	1.616
29093	NM_001014990,NM	**MRPL22**	mitochondrial ribosomal	protein_coding	36	38	37	39	1.056
29094	NM_014181	**LGALSL**	galectin like	protein_coding	1	1	2	2	0.889
29095	NM_014182	**ORMDL2**	ORMDL sphingolipid bio	protein_coding	24	24	25	25	0.979
29097	NM_001277197,NM	**CNIH4**	cornichon family AMPA r	protein_coding	15	14	16	15	0.941
29098	NM_001177801,NM	**RANGRF**	RAN guanine nucleotide	protein_coding	61	54	62	55	0.885
29099	NM_001101653,NM	**COMMD9**	COMM domain containin	protein_coding	25	26	26	27	1.017
29100	NM_001318217,NM	**TMEM208**	transmembrane protein 2	protein_coding	89	89	90	90	1.006
29101	NM_014188	**SSU72**	SSU72 homolog, RNA po	protein_coding	158	167	159	168	1.055
29102	NM_001100412,NM	**DROSHA**	drosha ribonuclease III	protein_coding	40	36	41	37	0.901
29103	NM_013238	**DNAJC15**	DnaJ heat shock protein	protein_coding	0	0	1	1	1.021
29104	NM_013240,NM_18	**N6AMT1**	N-6 adenine-specific DN	protein_coding	10	8	11	9	0.780
29105	NM_013242,NR_13	**CFAP20**	cilia and flagella associa	protein_coding	76	74	77	75	0.972
29106	NM_001165257,NM	**SCG3**	secretogranin III	protein_coding	0	0	1	1	1.000
29107	NM_013248	**NXT1**	nuclear transport factor 2	protein_coding	85	88	86	89	1.032
29108	NM_013258,NM_14	**PYCARD**	PYD and CARD domain	protein_coding	0	0	1	1	1.000
29109	NM_001318202,NM	**FHOD1**	formin homology 2 doma	protein_coding	28	28	29	29	0.992
29110	NM_013254,XM_00!	**TBK1**	TANK binding kinase 1	protein_coding	49	41	50	42	0.842
29113	NM_014070	**C6orf15**	chromosome 6 open rea	protein_coding	0	0	1	1	1.000
29114	NM_001008272,NM	**TAGLN3**	transgelin 3	protein_coding	0	0	1	1	1.000
29115	NM_001301839,NM	**SAP30BP**	SAP30 binding protein	protein_coding	62	64	63	65	1.042
29116	NM_013262,XM_00!	**MYLIP**	myosin regulatory light c	protein_coding	4	5	5	6	1.245
29117	NM_001173984,NM	**BRD7**	bromodomain containing	protein_coding	64	64	65	65	1.005
29118	NM_001330438,NM	**DDX25**	DEAD-box helicase 25	protein_coding	0	0	1	1	1.000
29119	NM_001127384,NM	**CTNNA3**	catenin alpha 3	protein_coding	0	0	1	1	1.000
29121	NM_001004419,NM	**CLEC2D**	C-type lectin domain fam	protein_coding	33	30	34	31	0.918
29122	NM_013270	**PRSS50**	serine protease 50	protein_coding	0	0	1	1	1.000
29123	NM_001256182,NM	**ANKRD11**	ankyrin repeat domain 1	protein_coding	27	24	28	25	0.889
29124	NM_013268,XM_01	**LGALS13**	galectin 13	protein_coding	0	0	1	1	1.000
29125	NM_001142946,NM	**C11orf21**	chromosome 11 open rea	protein_coding	2	3	3	4	1.203
29126	NM_001267706,NM	**CD274**	CD274 molecule	protein_coding	2	1	3	2	0.739
29127	NM_001126103,NM	**RACGAP1**	Rac GTPase activating p	protein_coding	124	117	125	118	0.946
29128	NM_001048201,NM	**UHRF1**	ubiquitin like with PHD a	protein_coding	91	96	92	97	1.057
29760	NM_001114094,NM	**BLNK**	B cell linker	protein_coding	0	0	1	1	0.971
29761	NM_001283041,NM	**USP25**	ubiquitin specific peptida	protein_coding	27	24	28	25	0.883
29763	NM_001184974,NM	**PACSIN3**	protein kinase C and cas	protein_coding	0	0	1	1	0.977
29765	NM_013353,XM_01	**TMOD4**	tropomodulin 4	protein_coding	1	1	2	2	1.135
29766	NM_014547,XM_01	**TMOD3**	tropomodulin 3	protein_coding	40	29	41	30	0.737
29767	NM_001142885,NM	**TMOD2**	tropomodulin 2	protein_coding	0	0	1	1	0.971
29768	gene-SDHDP6	**SDHDP6**	succinate dehydrogenas	pseudogene	0	0	1	1	0.886
29769	gene-SDHDP5	**SDHDP5**	succinate dehydrogenas	pseudogene	0	0	1	1	1.000
29770	gene-SDHDP4	**SDHDP4**	succinate dehydrogenas	pseudogene	0	0	1	1	1.000
29771	gene-SDHDP3	**SDHDP3**	succinate dehydrogenas	pseudogene	0	0	1	1	1.006
29774	NR_003714	**POM121L9P**	POM121 transmembrane	transcribed_pseu	0	0	1	1	1.000
29775	NM_014550	**CARD10**	caspase recruitment don	protein_coding	0	0	1	1	1.000
29777	NM_013375	**ABT1**	activator of basal transcr	protein_coding	85	91	86	92	1.063
29778	gene-MAP2K1P1	**MAP2K1P1**	mitogen-activated protein	pseudogene	0	0	1	1	1.000
29780	NM_001003828,NM	**PARVB**	parvin beta	protein_coding	12	14	13	15	1.152
29781	NM_001185011,NM	**NCAPH2**	non-SMC condensin II co	protein_coding	79	85	80	86	1.067
29785	NM_030622,XR_93	**CYP2S1**	cytochrome P450 family	protein_coding	0	0	1	1	1.000
29789	NM_001011708,NM	**OLA1**	Obg like ATPase 1	protein_coding	132	117	133	118	0.887
29796	NM_001003684,NM	**UQCR10**	ubiquinol-cytochrome c r	protein_coding	188	203	189	204	1.082
29797	NR_024583	**POM121L8P**	POM121 transmembrane	transcribed_pseu	0	0	1	1	1.000
29798	NR_149013,NR_14	**C2orf27A**	chromosome 2 open rea	lncRNA	8	6	9	7	0.777
29799	NM_013313,NR_13	**YPEL1**	yippee like 1	protein_coding	5	4	6	5	0.914
29800	NM_001323627,NM	**ZDHHC1**	zinc finger DHHC-type co	protein_coding	0	0	1	1	1.000
29801	NM_001185024,NM	**ZDHHC8**	zinc finger DHHC-type p	protein_coding	11	11	12	12	0.989
29802	NM_013378	**VPREB3**	V-set pre-B cell surrogat	protein_coding	0	0	1	1	1.000
29803	NM_001099695,NM	**REPIN1**	replication initiator 1	protein_coding	58	53	59	54	0.914
29841	NM_198182,XM_00	**GRHL1**	grainyhead like transcrip	protein_coding	0	0	1	1	1.000
29842	NM_014553,XM_01	**TFCP2L1**	transcription factor CP2 l	protein_coding	0	0	1	1	1.000
29843	NM_001267594,NM	**SENP1**	SUMO specific peptidase	protein_coding	43	39	44	40	0.911
29844	NM_001321792,NM	**TFPT**	TCF3 fusion partner	protein_coding	45	51	46	52	1.122
29850	NM_014555,XM_01	**TRPM5**	transient receptor potent	protein_coding	0	0	1	1	1.000
29851	NM_012092	**ICOS**	inducible T cell costimula	protein_coding	67	58	68	59	0.858
29855	NM_001079514,NM	**UBN1**	ubinuclein 1	protein_coding	51	45	52	46	0.886
29880	NM_001142364,NM	**ALG5**	ALG5 dolichyl-phosphate	protein_coding	95	83	96	84	0.875
29881	NM_001101648,NM	**NPC1L1**	NPC1 like intracellular cl	protein_coding	0	0	1	1	1.000
29882	NM_013366,XR_92	**ANAPC2**	anaphase promoting com	protein_coding	26	28	27	29	1.091
29883	NM_001322087,NM	**CNOT7**	CCR4-NOT transcription	protein_coding	38	33	39	34	0.872
29886	NM_013321,XM_01	**SNX8**	sorting nexin 8	protein_coding	13	11	14	12	0.873
29887	NM_001199835,NM	**SNX10**	sorting nexin 10	protein_coding	65	57	66	58	0.869
29888	NM_001039877,NM	**STRN4**	striatin 4	protein_coding	94	95	95	96	1.009
29889	NM_001323623,NM	**GNL2**	G protein nucleolar 2	protein_coding	145	133	146	134	0.921
29890	NM_013286	**RBM15B**	RNA binding motif protei	protein_coding	28	28	29	29	1.019
29893	NM_001256014,NM	**PSMC3IP**	PSMC3 interacting prote	protein_coding	51	57	52	58	1.106
29894	NM_013291,XM_00	**CPSF1**	cleavage and polyadenyl	protein_coding	27	29	28	30	1.056
29895	NM_001324458,NM	**MYLPF**	myosin light chain, phos	protein_coding	0	0	1	1	0.754
29896	NM_001282757,NM	**TRA2A**	transformer 2 alpha hom	protein_coding	109	102	110	103	0.935
29899	NM_001321038,NM	**GPSM2**	G protein signaling modu	protein_coding	14	11	15	12	0.790
29901	NM_001367485,NM	**SAC3D1**	SAC3 domain containing	protein_coding	23	23	24	24	1.007
29902	NM_013300,XM_00	**FAM216A**	family with sequence sim	protein_coding	79	84	80	85	1.056
29903	NM_001370467,NM	**CCDC106**	coiled-coil domain contai	protein_coding	1	1	2	2	1.254
29904	NM_013302,XM_01	**EEF2K**	eukaryotic elongation fac	protein_coding	11	10	12	11	0.895
29906	NM_001307986,NM	**ST8SIA5**	ST8 alpha-N-acetyl-neur	protein_coding	0	0	1	1	1.000
29907	NM_013306,NM_14	**SNX15**	sorting nexin 15	protein_coding	10	11	11	12	1.095
29909	NM_013308,XM_00	**GPR171**	G protein-coupled recept	protein_coding	2	3	3	4	1.299
29911	NM_001100176,NM	**HOOK2**	hook microtubule tetheri	protein_coding	2	2	3	3	1.028
29914	NM_001330349,NM	**UBIAD1**	UbiA prenyltransferase d	protein_coding	41	41	42	42	1.009
29915	NM_013320,XM_01	**HCFC2**	host cell factor C2	protein_coding	7	6	8	7	0.800
29916	NM_001330320,NM	**SNX11**	sorting nexin 11	protein_coding	23	24	24	25	1.043
29919	NM_001276342,NM	**RMC1**	regulator of MON1-CCZ1	protein_coding	42	40	43	41	0.952

29920	NM_001271681,NM	PYCR2	pyrroline-5-carboxylate re	protein_coding	100	108	101	109	1.080
29922	NM_013330,NM_19	NME7	NME/NM23 family memb	protein_coding	11	11	12	12	1.017
29923	NM_001098786,NM	HILPDA	hypoxia inducible lipid dr	protein_coding	15	14	16	15	0.917
29924	NM_001130071,NM	EPN1	epsin 1	protein_coding	70	72	71	73	1.038
29925	NM_013334,NM_02	GMPPB	GDP-mannose pyrophos	protein_coding	26	26	27	27	1.032
29926	NM_001374294,NM	GMPPA	GDP-mannose pyrophos	protein_coding	36	39	37	40	1.084
29927	NM_013336	SEC61A1	SEC61 translocon subur	protein_coding	232	232	233	233	1.001
29928	NM_013337	TIMM22	translocase of inner mito	protein_coding	11	12	12	13	1.053
29929	NM_013339	ALG6	ALG6 alpha-1,3-glucosyl	protein_coding	12	12	13	13	0.939
29930	NM_013340	PCDHB1	protocadherin beta 1	protein_coding	0	0	1	1	1.000
29931	NR_024065	LINC00312	long intergenic non-prote	lncRNA	0	0	1	1	1.000
29933	NM_001278694,NM	GPR132	G protein-coupled recept	protein_coding	23	21	24	22	0.912
29934	NM_001256185,NM	SNX12	sorting nexin 12	protein_coding	50	48	51	49	0.960
29935	NM_013347	RPA4	replication protein A4	protein_coding	1	1	2	2	1.271
29937	NM_013349,NR_02	NENF	neudesin neurotrophic fa	protein_coding	13	13	14	14	1.002
29940	NM_001080976,NM	DSE	dermatan sulfate epimera	protein_coding	0	0	1	1	0.936
29941	NM_001317926,NM	PKN3	protein kinase N3	protein_coding	0	0	1	1	1.000
29942	NM_001015508,NM	PURG	purine rich element bindi	protein_coding	0	0	1	1	0.986
29943	NM_013358,XM_01	PADI1	peptidyl arginine deimina	protein_coding	0	0	1	1	1.000
29944	NM_001282535,NM	PNMA3	PNMA family member 3	protein_coding	0	0	1	1	1.000
29945	NM_001286756,NM	ANAPC4	anaphase promoting con	protein_coding	29	26	30	27	0.899
29946	NM_013368,NM_20	SERTAD3	SERTA domain containir	protein_coding	6	7	7	8	1.152
29947	NM_013369,NM_17	DNMT3L	DNA methyltransferase 3	protein_coding	0	0	1	1	1.000
29948	NM_182981	OSGIN1	oxidative stress induced	protein_coding	0	0	1	1	1.000
29949	NM_001369605,NM	IL19	interleukin 19	protein_coding	3	3	4	4	1.018
29950	NM_013376	SERTAD1	SERTA domain containir	protein_coding	7	8	8	9	1.044
29951	NM_001164595,NM	PDZRN4	PDZ domain containing r	protein_coding	0	0	1	1	1.000
29952	NM_013379,XM_00	DPP7	dipeptidyl peptidase 7	protein_coding	48	50	49	51	1.030
29953	NM_013381,XM_00	TRHDE	thyrotropin releasing hor	protein_coding	0	0	1	1	1.000
29954	NM_013382,XM_01	POMT2	protein O-mannosyltrans	protein_coding	3	3	4	4	1.231
29956	NM_022075,NM_18	CERS2	ceramide synthase 2	protein_coding	202	191	203	192	0.944
29957	NM_013386,NM_21	SLC25A24	solute carrier family 25 m	protein_coding	31	27	32	28	0.877
29958	NM_013391,NR_10	DMGDH	dimethylglycine dehydro	protein_coding	0	0	1	1	1.013
29959	NM_001321357,NM	NRBP1	nuclear receptor binding	protein_coding	51	54	52	55	1.045
29960	NM_013393	MRM2	mitochondrial rRNA meth	protein_coding	66	71	67	72	1.075
29964	NM_013397	PRICKLE4	prickle planar cell polarit	protein_coding	2	2	3	3	1.012
29965	NM_001199054,NM	CDIP1	cell death inducing p53 t	protein_coding	54	60	55	61	1.110
29966	NM_001083893,NM	STRN3	striatin 3	protein_coding	16	18	17	19	1.078
29967	NM_001135703,NM	LRP12	LDL receptor related prot	protein_coding	0	0	1	1	0.956
29968	NM_021154,NM_05	PSAT1	phosphoserine aminotrar	protein_coding	63	51	64	52	0.811
29969	NM_001166345,NM	MDFIC	MyoD family inhibitor dor	protein_coding	48	46	49	47	0.959
29970	NM_001197107,NM	SCHIP1	schwannomin interacting	protein_coding	18	17	19	18	0.957
29974	NM_001198818,NM	A1CF	APOBEC1 complementa	protein_coding	0	0	1	1	1.000
29978	NM_013444	UBQLN2	ubiquilin 2	protein_coding	22	22	23	23	0.998
29979	NM_013438,NM_05	UBQLN1	ubiquilin 1	protein_coding	62	59	63	60	0.941
29980	NM_017613	DONSON	DNA replication fork stab	protein_coding	58	58	59	59	0.990
29982	NM_001282405,NM	NRBF2	nuclear receptor binding	protein_coding	38	31	39	32	0.834
29984	NM_001300886,NM	RHOD	ras homolog family mem	protein_coding	0	0	1	1	1.000
29985	NM_144564,NM_21	SLC39A3	solute carrier family 39 m	protein_coding	45	42	46	43	0.936
29986	NM_001256588,NM	SLC39A2	solute carrier family 39 m	protein_coding	0	0	1	1	1.023
29988	NM_001271711,NM	SLC2A8	solute carrier family 2 me	protein_coding	13	12	14	13	0.923
29989	NM_001288987,NM	OBP2B	odorant binding protein 2	protein_coding	0	0	1	1	1.000
29990	NM_001371931,NM	PILRB	paired immunoglobin like	protein_coding	11	8	12	9	0.791
29991	NM_001293189,NM	OBP2A	odorant binding protein 2	protein_coding	0	0	1	1	1.000
29992	NM_013439,NM_17	PILRA	paired immunoglobin like	protein_coding	2	4	3	5	1.388
29993	NM_001199583,NM	PACSIN1	protein kinase C and cas	protein_coding	0	0	1	1	0.959
29994	NM_001289975,NM	BAZ2B	bromodomain adjacent to	protein_coding	5	4	6	5	0.844
29995	NM_001278233,NM	LMCD1	LIM and cysteine rich do	protein_coding	0	0	1	1	1.000
29997	NM_015710	NOP53	NOP53 ribosome biogen	protein_coding	183	177	184	178	0.968
29998	NM_015711,XM_00	BICRA	BRD4 interacting chroma	protein_coding	7	7	8	8	1.030
29999	NM_020369	FSCN3	fascin actin-bundling pro	protein_coding	0	0	1	1	0.935
30000	NM_001136195,NM	TNPO2	transportin 2	protein_coding	61	56	62	57	0.932
30001	NM_001382464,NM	ERO1A	endoplasmic reticulum o	protein_coding	93	79	94	80	0.854
30008	NM_016938,NR_03	EFEMP2	EGF containing fibulin e	protein_coding	1	0	2	1	0.780
30009	NM_013351	TBX21	T-box transcription factor	protein_coding	299	341	300	342	1.141
30010	NM_152745	NXPH1	neurexophilin 1	protein_coding	0	0	1	1	1.000
30011	NM_001024666,NM	SH3KBP1	SH3 domain containing k	protein_coding	126	116	127	117	0.919
30012	NM_021025	TLX3	T cell leukemia homeobc	protein_coding	0	0	1	1	1.000
30014	NM_013453	SPANXA1	sperm protein associatec	protein_coding	0	0	1	1	1.000
30061	NM_014585,XM_00	SLC40A1	solute carrier family 40 m	protein_coding	0	0	1	1	1.000
30062	NM_013435	RAX	retina and anterior neura	protein_coding	0	0	1	1	1.000
30811	NM_014586,XM_01	HUNK	hormonally up-regulated	protein_coding	0	0	1	1	1.000
30812	NM_014587	SOX8	SRY-box transcription fa	protein_coding	0	0	1	1	1.000
30813	NM_001256271,NM	VSX1	visual system homeobox	protein_coding	0	0	1	1	1.029
30814	NM_014589	PLA2G2E	phospholipase A2 group	protein_coding	0	0	1	1	1.000
30815	NM_001286999,NM	ST6GALNAC6	ST6 N-acetylgalactosam	protein_coding	8	7	9	8	0.909
30816	NM_001130925,NM	ERVW-1	endogenous retrovirus g	protein_coding	0	0	1	1	1.000
30817	NM_001271052,NM	ADGRE2	adhesion G protein-coup	protein_coding	1	1	2	2	1.117
30818	NM_001034914,NM	KCNIP3	potassium voltage-gated	protein_coding	0	0	1	1	1.000
30819	NM_014591,NM_17	KCNIP2	potassium voltage-gated	protein_coding	0	1	1	2	1.098
30820	NM_001034837,NM	KCNIP1	potassium voltage-gated	protein_coding	0	0	1	1	1.000
30827	NM_001101654,NM	CXXC1	CXXC finger protein 1	protein_coding	60	59	61	60	0.985
30832	NM_014594,XM_01	ZNF354C	zinc finger protein 354C	protein_coding	0	0	1	1	1.000
30833	NM_001252377,NM	NT5C	5', 3'-nucleotidase, cytos	protein_coding	52	64	53	65	1.234
30834	NM_001278785,NM	POLR1H	RNA polymerase I subur	protein_coding	84	89	85	90	1.050
30835	NM_001144893,NM	CD209	CD209 molecule	protein_coding	0	0	1	1	1.000
30836	NM_014597	DNTTIP2	deoxynucleotidyltransfera	protein_coding	46	44	47	45	0.968
30837	NM_014598,XM_01	SOCS7	suppressor of cytokine si	protein_coding	13	11	14	12	0.862
30844	NM_139265	EHD4	EH domain containing 4	protein_coding	27	25	28	26	0.932
30845	NM_014600,XM_01	EHD3	EH domain containing 3	protein_coding	0	0	1	1	1.000
30846	NM_014601	EHD2	EH domain containing 2	protein_coding	0	0	1	1	1.000
30848	NM_020994,NM_17	CTAG2	cancer/testis antigen 2	protein_coding	0	0	1	1	1.000
30849	NM_014602	PIK3R4	phosphoinositide-3-kinas	protein_coding	28	24	29	25	0.870
30850	NM_014603,XM_00	CDR2L	cerebellar degeneration	protein_coding	0	0	1	1	1.001
30851	NM_001204698,NM	TAX1BP3	Tax1 binding protein 3	protein_coding	7	7	8	8	1.026
30968	NM_001287031,NM	STOML2	stomatin like 2	protein_coding	187	186	188	187	0.994
43847	NM_001311182,NM	KLK14	kallikrein related peptida	protein_coding	0	1	1	2	1.349
43849	NM_001370125,NM	KLK12	kallikrein related peptida	protein_coding	0	0	1	1	1.000
49854	NM_001098402,NM	ZBTB21	zinc finger and BTB dom	protein_coding	9	8	10	9	0.916
49855	NM_001145923,NM	SCAPER	S-phase cyclin A associa	protein_coding	14	12	15	13	0.872
49856	NM_017818,XM_00	WRAP73	WD repeat containing, ar	protein_coding	41	42	42	43	1.005
49860	NM_016190	CRNN	cornulin	protein_coding	0	0	1	1	1.017
49861	NM_001001346	CLDN20	claudin 20	protein_coding	0	0	1	1	1.000
50484	NM_001172477,NM	RRM2B	ribonucleotide reductase	protein_coding	19	16	20	17	0.848
50485	NM_001127207,NM	SMARCAL1	SWI/SNF related, matrix	protein_coding	25	23	26	24	0.918
50486	NM_015714	G0S2	G0/G1 switch 2	protein_coding	0	0	1	1	1.000
50487	NM_015715,XM_01	PLA2G3	phospholipase A2 group	protein_coding	0	0	1	1	1.000
50488	NM_001024937,NM	MINK1	misshapen like kinase 1	protein_coding	17	16	18	17	0.939
50489	NM_015717,XM_01	CD207	CD207 molecule	protein_coding	0	0	1	1	1.000
50506	NM_001363711,NM	DUOX2	dual oxidase 2	protein_coding	0	0	1	1	1.000
50507	NM_001143836,NM	NOX4	NADPH oxidase 4	protein_coding	0	0	1	1	1.000
50508	NM_015718	NOX3	NADPH oxidase 3	protein_coding	0	0	1	1	1.000
50509	NM_015719,XM_01	COL5A3	collagen type V alpha 3 c	protein_coding	0	0	1	1	1.000
50511	NM_001177948,NM	SYCP3	synaptonemal complex p	protein_coding	0	0	1	1	0.933
50512	NM_015720	PODXL2	podocalyxin like 2	protein_coding	0	0	1	1	1.000
50514	NR_163556	DELEC1	deleted in esophageal ca	lncRNA	0	0	1	1	1.000
50515	NM_001173982,NM	CHST11	carbohydrate sulfotransfe	protein_coding	67	65	68	66	0.971
50516	gene-PPP1R14BP1	PPP1R14BP1	protein phosphatase 1 re	pseudogene	0	0	1	1	1.099
50604	NM_018724,XM_00	IL20	interleukin 20	protein_coding	0	0	1	1	1.000
50613	NM_001347096,NM	UBQLN3	ubiquilin 3	protein_coding	0	0	1	1	1.000
50614	NM_001122636,NM	GALNT9	polypeptide N-acetylgala	protein_coding	0	0	1	1	1.000
50615	NM_021798,NM_18	IL21R	interleukin 21 receptor	protein_coding	36	35	37	36	0.966
50616	NM_020525	IL22	interleukin 22	protein_coding	0	0	1	1	1.000
50617	NM_020632,NM_13	ATP6V0A4	ATPase H+ transporting	protein_coding	0	0	1	1	1.000
50618	NM_001348181,NM	ITSN2	intersectin 2	protein_coding	43	33	44	34	0.771
50619	NM_022047	DEF6	DEF6 guanine nucleotide	protein_coding	107	107	108	108	1.002

50624	NM_022034,NR_03	**CUZD1**	CUB and zona pellucida	protein_coding	1	1	2	2	0.954
50626	NM_001129888,NM	**CYHR1**	cysteine and histidine ric	protein_coding	4	5	5	6	1.065
50628	NM_015721,XM_00	**GEMIN4**	gem nuclear organelle a	protein_coding	26	27	27	28	1.016
50631	gene-YBX1P1	**YBX1P1**	Y-box binding protein 1 p	pseudogene	11	13	12	14	1.173
50632	NM_001321617,NM	**CALY**	calcyon neuron specific v	protein_coding	0	0	1	1	1.000
50636	NM_001001666,NM	**ANO7**	anoctamin 7	protein_coding	0	0	1	1	1.000
50640	NM_001256007,NM	**PNPLA8**	patatin like phospholipas	protein_coding	10	9	11	10	0.935
50649	NM_001367493,NM	**ARHGEF4**	Rho guanine nucleotide	protein_coding	0	0	1	1	1.006
50650	NM_001128615,NM	**ARHGEF3**	Rho guanine nucleotide	protein_coding	58	47	59	48	0.820
50651	NM_001080397,NM	**SLC45A1**	solute carrier family 45 m	protein_coding	0	0	1	1	1.000
50652	NR_015342,NR_13	**PCA3**	prostate cancer associat	lncRNA	0	0	1	1	1.000
50674	NM_020999,XM_01	**NEUROG3**	neurogenin 3	protein_coding	0	0	1	1	1.000
50700	NM_015725	**RDH8**	retinol dehydrogenase 8	protein_coding	0	0	1	1	1.000
50717	NM_015726,NR_02	**DCAF8**	DDB1 and CUL4 associa	protein_coding	47	48	48	49	1.037
50801	NM_001317090,NM	**KCNK4**	potassium two pore dom	protein_coding	0	0	1	1	1.000
50804	NM_001301210,NM	**MYEF2**	myelin expression factor	protein_coding	0	0	1	1	1.000
50805	NM_001278632,NM	**IRX4**	iroquois homeobox 4	protein_coding	0	0	1	1	1.000
50807	NM_001247996,NM	**ASAP1**	ArfGAP with SH3 domair	protein_coding	14	12	15	13	0.899
50808	NM_001199852,NM	**AK3**	adenylate kinase 3	protein_coding	33	27	34	28	0.834
50809	NM_001372052,NM	**HP1BP3**	heterochromatin protein	protein_coding	86	71	87	72	0.827
50810	NM_016073,XM_00	**HDGFL3**	HDGF like 3	protein_coding	30	27	31	28	0.904
50813	NM_001164093,NM	**COPS7A**	COP9 signalosome subu	protein_coding	18	19	19	20	1.040
50814	NM_001129765,NM	**NSDHL**	NAD(P) dependent stero	protein_coding	46	46	47	47	1.003
50831	NM_016943	**TAS2R3**	taste 2 receptor member	protein_coding	0	0	1	1	0.766
50832	NM_016944	**TAS2R4**	taste 2 receptor member	protein_coding	0	0	1	1	1.045
50833	NM_016945	**TAS2R16**	taste 2 receptor member	protein_coding	0	0	1	1	1.000
50834	NM_019599	**TAS2R1**	taste 2 receptor member	protein_coding	0	0	1	1	1.000
50835	NM_023917	**TAS2R9**	taste 2 receptor member	protein_coding	0	0	1	1	1.000
50836	NM_023918	**TAS2R8**	taste 2 receptor member	protein_coding	0	0	1	1	1.000
50837	NM_023919	**TAS2R7**	taste 2 receptor member	protein_coding	0	0	1	1	1.000
50838	NM_023920	**TAS2R13**	taste 2 receptor member	protein_coding	0	0	1	1	0.957
50839	NM_023921	**TAS2R10**	taste 2 receptor member	protein_coding	0	0	1	1	0.997
50840	NM_023922	**TAS2R14**	taste 2 receptor member	protein_coding	0	0	1	1	0.942
50846	NM_021044,XM_01	**DHH**	desert hedgehog signali	protein_coding	0	0	1	1	1.000
50848	NM_001348091,NM	**F11R**	F11 receptor	protein_coding	35	32	36	33	0.927
50852	NM_001317747,NM	**TRAT1**	T cell receptor associate	protein_coding	14	12	15	13	0.897
50853	NM_001370264,NM	**VILL**	villin like	protein_coding	0	0	1	1	1.000
50854	NR_160945,NR_16	**SNHG32**	small nucleolar RNA hos	lncRNA	135	141	136	142	1.040
50855	NM_001037281,NM	**PARD6A**	par-6 family cell polarity	protein_coding	20	22	21	23	1.083
50856	NM_016184,NM_19	**CLEC4A**	C-type lectin domain fam	protein_coding	0	1	1	2	1.298
50858	NR_001538	**TTTY1**	testis-specific transcript,	lncRNA	0	0	1	1	1.000
50859	NM_001040159,NM	**SPOCK3**	SPARC (osteonectin), cw	protein_coding	0	0	1	1	1.000
50861	NM_001276310,NM	**STMN3**	stathmin 3	protein_coding	0	0	1	1	0.898
50862	NM_016422	**RNF141**	ring finger protein 141	protein_coding	12	12	13	13	0.995
50863	NM_001048209,NM	**NTM**	neurotrimin	protein_coding	3	3	4	4	0.869
50865	NM_015987	**HEBP1**	heme binding protein 1	protein_coding	0	0	1	1	1.000
50937	NM_001243597,NM	**CDON**	cell adhesion associated	protein_coding	0	0	1	1	1.053
50939	NM_016247	**IMPG2**	interphotoreceptor matri	protein_coding	0	0	1	1	1.016
50940	NM_001077196,NM	**PDE11A**	phosphodiesterase 11A	protein_coding	0	0	1	1	1.000
50943	NM_001114377,NM	**FOXP3**	forkhead box P3	protein_coding	0	0	1	1	1.037
50944	NM_016148,XM_01	**SHANK1**	SH3 and multiple ankyri	protein_coding	0	0	1	1	1.000
50945	NM_001109878,NM	**TBX22**	T-box transcription factor	protein_coding	0	0	1	1	1.000
50964	NM_025237	**SOST**	sclerostin	protein_coding	0	0	1	1	1.000
50999	NM_001167830,NM	**TMED5**	transmembrane p24 traff	protein_coding	60	53	61	54	0.886
51000	NM_001142540,NM	**SLC35B3**	solute carrier family 35 m	protein_coding	17	17	18	18	1.007
51001	NM_001286643,NM	**MTERF3**	mitochondrial transcriptic	protein_coding	56	58	57	59	1.025
51002	NM_001330386,NM	**TPRKB**	TP53RK binding protein	protein_coding	148	141	149	142	0.956
51003	NM_016060	**MED31**	mediator complex subun	protein_coding	4	3	5	4	0.946
51004	NM_182476,NM_18	**COQ6**	coenzyme Q6, monooxy	protein_coding	9	11	10	12	1.199
51005	NM_001145815,NM	**AMDHD2**	amidohydrolase domain	protein_coding	11	12	12	13	1.059
51006	NM_001281457,NM	**SLC35C2**	solute carrier family 35 m	protein_coding	33	39	34	40	1.182
51008	NM_001198798,NM	**ASCC1**	activating signal cointegr	protein_coding	25	21	26	22	0.853
51009	NM_001304777,NM	**DERL2**	derlin 2	protein_coding	14	14	15	15	0.958
51010	NM_001002269,NM	**EXOSC3**	exosome component 3	protein_coding	70	67	71	68	0.955
51011	NM_016044,XM_01	**FAHD2A**	fumarylacetoacetate hyd	protein_coding	15	15	16	16	0.998
51012	NM_001256403,NM	**PRELID3B**	PRELI domain containin	protein_coding	324	289	325	290	0.894
51013	NM_001318362,NM	**EXOSC1**	exosome component 1	protein_coding	63	73	64	74	1.156
51014	NM_181836	**TMED7**	transmembrane p24 traff	protein_coding	48	42	49	43	0.874
51015	NM_016048	**ISOC1**	isochorismatase domain	protein_coding	50	50	51	51	1.001
51016	NM_001346874,NM	**EMC9**	ER membrane protein co	protein_coding	22	27	23	28	1.213
51018	NM_016052,XM_01	**RRP15**	ribosomal RNA processin	protein_coding	18	17	19	18	0.935
51019	NM_001301107,NM	**WASHC3**	WASH complex subunit	protein_coding	43	47	44	48	1.087
51020	NM_016063,XM_02	**HDDC2**	HD domain containing 2	protein_coding	86	82	87	83	0.955
51021	NM_016065	**MRPS16**	mitochondrial ribosomal	protein_coding	100	93	101	94	0.929
51022	NM_001243399,NM	**GLRX2**	glutaredoxin 2	protein_coding	49	48	50	49	0.978
51023	NM_001297767,NM	**MRPS18C**	mitochondrial ribosomal	protein_coding	61	64	62	65	1.058
51024	NM_016068	**FIS1**	fission, mitochondrial 1	protein_coding	99	112	100	113	1.133
51025	NM_016069	**PAM16**	presequence translocase	protein_coding	86	79	87	80	0.919
51026	NM_016072,XM_02	**GOLT1B**	golgi transport 1B	protein_coding	30	27	31	28	0.888
51027	NM_001321025,NM	**BOLA1**	bolA family member 1	protein_coding	0	0	1	1	1.000
51028	NM_001282168,NM	**VPS36**	vacuolar protein sorting	protein_coding	29	28	30	29	0.959
51029	NM_001297746,NM	**DESI2**	desumoylating isopeptid	protein_coding	45	38	46	39	0.848
51030	NM_001316919,NM	**TVP23B**	trans-golgi network vesic	protein_coding	19	18	20	19	0.920
51031	NM_001366247,NM	**GLOD4**	glyoxalase domain conta	protein_coding	47	46	48	47	0.983
51032	NM_015849	**CELA2B**	chymotrypsin like elastas	protein_coding	0	0	1	1	1.000
51035	NM_001286077,NM	**UBXN1**	UBX domain protein 1	protein_coding	160	158	161	159	0.992
51042	NM_015871	**ZNF593**	zinc finger protein 593	protein_coding	140	142	141	143	1.012
51043	NM_001252406,NM	**ZBTB7B**	zinc finger and BTB dom	protein_coding	10	9	11	10	0.907
51046	NM_015879	**ST8SIA3**	ST8 alpha-N-acetyl-neur	protein_coding	0	0	1	1	1.000
51050	NM_001324403,NM	**PI15**	peptidase inhibitor 15	protein_coding	0	0	1	1	0.926
51052	NM_015893	**PRLH**	prolactin releasing horm	protein_coding	0	0	1	1	1.000
51053	NM_001251989,NM	**GMNN**	geminin DNA replication	protein_coding	121	123	122	124	1.018
51054	NR_037144	**PLEKHA8P1**	pleckstrin homology dom	transcribed_pseu	2	3	3	4	1.307
51056	NM_015907	**LAP3**	leucine aminopeptidase	protein_coding	314	289	315	290	0.920
51057	NM_001042692,NM	**WDPCP**	WD repeat containing pl	protein_coding	2	1	3	2	0.864
51058	NM_001242739,NM	**ZNF691**	zinc finger protein 691	protein_coding	9	11	10	12	1.214
51059	NM_001362965,NM	**FAM135B**	family with sequence sim	protein_coding	0	0	1	1	1.000
51060	NM_015913,NR_04	**TXNDC12**	thioredoxin domain conta	protein_coding	143	129	144	130	0.903
51061	NM_001303447,NM	**TXNDC11**	thioredoxin domain conta	protein_coding	23	24	24	25	1.031
51062	NM_001127713,NM	**ATL1**	atlastin GTPase 1	protein_coding	0	0	1	1	1.172
51063	NM_015916,NR_02	**CALHM2**	calcium homeostasis mo	protein_coding	4	3	5	4	0.762
51065	NM_015920	**RPS27L**	ribosomal protein S27 lik	protein_coding	17	17	18	18	1.007
51066	NM_001256748,NM	**SSUH2**	ssu-2 homolog	protein_coding	0	0	1	1	1.000
51067	NM_001040436,XR	**YARS2**	tyrosyl-tRNA synthetase	protein_coding	36	31	37	32	0.867
51068	NM_001320227,NM	**NMD3**	NMD3 ribosome export a	protein_coding	69	59	70	60	0.854
51069	NM_001300848,NM	**MRPL2**	mitochondrial ribosomal	protein_coding	98	105	99	106	1.074
51070	NM_001270960,NM	**NOSIP**	nitric oxide synthase inte	protein_coding	131	136	132	137	1.038
51071	NM_001300779,NM	**DERA**	deoxyribose-phosphate	protein_coding	12	15	13	16	1.244
51072	NM_001137602,NM	**MEMO1**	mediator of cell motility 1	protein_coding	14	12	15	13	0.882
51073	NM_015956,NM_14	**MRPL4**	mitochondrial ribosomal	protein_coding	244	250	245	251	1.027
51074	NM_015957,XM_01	**APIP**	APAF1 interacting protei	protein_coding	104	99	105	100	0.948
51075	NM_001144012,NM	**TMX2**	thioredoxin related trans	protein_coding	206	200	207	201	0.969
51076	NM_015960	**CUTC**	cutC copper transporter	protein_coding	50	51	51	52	1.019
51077	NM_001318508,NM	**FCF1**	FCF1 rRNA-processing	protein_coding	62	60	63	61	0.969
51078	NM_001164356,NM	**THAP4**	THAP domain containing	protein_coding	124	120	125	121	0.971
51079	NM_015965	**NDUFA13**	NADH:ubiquinone oxidor	protein_coding	397	439	398	440	1.105
51081	NM_015971	**MRPS7**	mitochondrial ribosomal	protein_coding	200	210	201	211	1.047
51082	NM_001206559,NM	**POLR1D**	RNA polymerase I and III	protein_coding	208	211	209	212	1.013
51083	NM_015973	**GAL**	galanin and GMAP prep	protein_coding	0	0	1	1	1.000
51084	NM_001363647,NM	**CRYL1**	crystallin lambda 1	protein_coding	0	1	1	2	1.427
51085	NM_032951,NM_03	**MLXIPL**	MLX interacting protein li	protein_coding	0	0	1	1	1.000
51086	NM_015978	**TNNI3K**	TNNI3 interacting kinase	protein_coding	0	0	1	1	1.000
51087	NM_015982,XM_01	**YBX2**	Y-box binding protein 2	protein_coding	0	0	1	1	1.000
51088	NM_001007075,NM	**KLHL5**	kelch like family member	protein_coding	9	7	10	8	0.820
51090	NM_015993	**PLLP**	plasmolipin	protein_coding	0	0	1	1	1.000
51091	NM_016955,XM_01	**SEPSECS**	Sep (O-phosphoserine) t	protein_coding	6	6	7	7	0.971

51092	NM_001040455	**SIDT2**	SID1 transmembrane far	protein_coding	3	3	4	4	1.006
51093	NM_001142560,NM	**RRNAD1**	ribosomal RNA adenine (	protein_coding	25	26	26	27	1.028
51094	NM_001290553,NM	**ADIPOR1**	adiponectin receptor 1	protein_coding	96	95	97	96	0.991
51095	NM_001302946,NM	**TRNT1**	tRNA nucleotidyl transfer	protein_coding	37	30	38	31	0.802
51096	NM_016001,XM_00(	**UTP18**	UTP18 small subunit pro	protein_coding	106	102	107	103	0.962
51097	NM_016002	**SCCPDH**	saccharopine dehydroge	protein_coding	19	19	20	20	1.010
51098	NM_001303458,NM	**IFT52**	intraflagellar transport 52	protein_coding	34	34	35	35	1.010
51099	NM_001355186,NM	**ABHD5**	abhydrolase domain con	protein_coding	11	10	12	11	0.986
51100	NM_001206651,NM	**SH3GLB1**	SH3 domain containing (	protein_coding	53	46	54	47	0.869
51101	NM_001362969,NM	**ZC2HC1A**	zinc finger C2HC-type cc	protein_coding	0	0	1	1	1.000
51102	NM_001024732,NM	**MECR**	mitochondrial trans-2-en(	protein_coding	26	29	27	30	1.093
51103	NM_016013,NR_04!	**NDUFAF1**	NADH:ubiquinone oxidor	protein_coding	40	43	41	44	1.071
51104	NM_001025780,NM	**ABHD17B**	abhydrolase domain con	protein_coding	19	16	20	17	0.843
51105	NM_001277196,NM	**PHF20L1**	PHD finger protein 20 lik	protein_coding	6	6	7	7	0.979
51106	NM_001350501,NM	**TFB1M**	transcription factor B1, m	protein_coding	10	10	11	11	1.066
51107	NM_001077628,NM	**APH1A**	aph-1 homolog A, gamm	protein_coding	289	304	290	305	1.054
51108	NM_001077180,NM	**METTL9**	methyltransferase like 9	protein_coding	75	71	76	72	0.943
51109	NM_001252650,NM	**RDH11**	retinol dehydrogenase 1:	protein_coding	55	51	56	52	0.930
51110	NM_016027	**LACTB2**	lactamase beta 2	protein_coding	31	26	32	27	0.848
51111	NM_001300907,NM	**KMT5B**	lysine methyltransferase	protein_coding	8	8	9	9	0.972
51112	NM_001321102,NM	**TRAPPC12**	trafficking protein particle	protein_coding	18	19	19	20	1.011
51114	NM_001008222,NM	**ZDHHC9**	zinc finger DHHC-type pa	protein_coding	4	4	5	5	0.962
51115	NM_001286707,NM	**RMDN1**	regulator of microtubule	protein_coding	50	47	51	48	0.949
51116	NM_001371401,NM	**MRPS2**	mitochondrial ribosomal	protein_coding	88	97	89	98	1.105
51117	NM_001305942,NM	**COQ4**	coenzyme Q4	protein_coding	42	46	43	47	1.104
51118	NM_016037,XM_01:	**UTP11**	UTP11 small subunit pro	protein_coding	153	141	154	142	0.919
51119	NM_016038	**SBDS**	SBDS ribosome maturati	protein_coding	117	113	118	114	0.967
51121	NM_001317980,NM	**RPL26L1**	ribosomal protein L26 lik(	protein_coding	115	132	116	133	1.152
51122	NM_016094,NR_13!	**COMMD2**	COMM domain containin	protein_coding	43	38	44	39	0.890
51123	NM_001042510,NM	**ZNF706**	zinc finger protein 706	protein_coding	38	38	39	39	1.011
51124	NM_016097	**IER3IP1**	immediate early respons	protein_coding	41	37	42	38	0.903
51125	NM_001002296,NM	**GOLGA7**	golgin A7	protein_coding	99	98	100	99	0.995
51126	NM_016100,NM_18	**NAA20**	N-alpha-acetyltransferas	protein_coding	167	160	168	161	0.955
51127	NM_001024940,NM	**TRIM17**	tripartite motif containing	protein_coding	0	0	1	1	1.000
51128	NM_001033503,NM	**SAR1B**	secretion associated Ras	protein_coding	30	28	31	29	0.932
51129	NM_001039667,NM	**ANGPTL4**	angiopoietin like 4	protein_coding	0	0	1	1	1.000
51130	NM_001201965,NM	**ASB3**	ankyrin repeat and SOC!	protein_coding	16	13	17	14	0.845
51131	NM_001040443,NM	**PHF11**	PHD finger protein 11	protein_coding	20	18	21	19	0.912
51132	NM_016120,NM_18	**RLIM**	ring finger protein, LIM d	protein_coding	26	23	27	24	0.900
51133	NM_001319294,NM	**KCTD3**	potassium channel tetrar	protein_coding	0	0	1	1	1.000
51134	NM_001042399,NM	**CEP83**	centrosomal protein 83	protein_coding	18	15	19	16	0.843
51135	NM_001114182,NM	**IRAK4**	interleukin 1 receptor ass	protein_coding	15	14	16	15	0.964
51136	NM_016125	**RNFT1**	ring finger protein, transr	protein_coding	10	9	11	10	0.947
51138	NM_001258006,NM	**COPS4**	COP9 signalosome subu	protein_coding	51	53	52	54	1.051
51141	NM_001321329,NM	**INSIG2**	insulin induced gene 2	protein_coding	14	12	15	13	0.880
51142	NM_001320327,NM	**CHCHD2**	coiled-coil-helix-coiled-cc	protein_coding	661	706	662	707	1.068
51143	NM_001329135,NM	**DYNC1LI1**	dynein cytoplasmic 1 ligh	protein_coding	63	56	64	57	0.897
51144	NM_016142,XM_01:	**HSD17B12**	hydroxysteroid 17-beta d	protein_coding	28	21	29	22	0.766
51145	NR_122032	**LOC51145**	uncharacterized LOC511	lncRNA	0	0	1	1	1.000
51146	NM_016161,XM_01	**A4GNT**	alpha-1,4-N-acetylglucos	protein_coding	0	0	1	1	1.000
51147	NM_001127582,NM	**ING4**	inhibitor of growth family	protein_coding	8	8	9	9	1.080
51148	NM_001286760,NM	**CERCAM**	cerebral endothelial cell	protein_coding	0	0	1	1	1.000
51149	NM_001017987,NM	**MRNIP**	MRN complex interacting	protein_coding	9	9	10	10	1.016
51150	NM_016176,NM_01(	**SDF4**	stromal cell derived facto	protein_coding	160	182	161	183	1.133
51151	NM_001012509,NM	**SLC45A2**	solute carrier family 45 m	protein_coding	0	0	1	1	1.000
51154	NM_016183,XM_00(	**MRTO4**	MRT4 homolog, ribosom(	protein_coding	265	257	266	258	0.970
51155	NM_001002032,NM	**JPT1**	Jupiter microtubule asso	protein_coding	305	327	306	328	1.072
51156	NM_001100607,NM	**SERPINA10**	serpin family A member :	protein_coding	0	0	1	1	1.000
51157	NM_001163423,NM	**ZNF580**	zinc finger protein 580	protein_coding	28	31	29	32	1.107
51160	NM_016208,NM_18	**VPS28**	VPS28 subunit of ESCR'	protein_coding	19	21	20	22	1.089
51161	NM_001171740,NM	**C3orf18**	chromosome 3 open rea(	protein_coding	7	9	8	10	1.266
51162	NM_016215,NM_20	**EGFL7**	EGF like domain multiple	protein_coding	0	0	1	1	1.000
51163	NM_016216	**DBR1**	debranching RNA lariats	protein_coding	45	40	46	41	0.884
51164	NM_001135643,NM	**DCTN4**	dynactin subunit 4	protein_coding	39	35	40	36	0.920
51166	NM_001286682,NM	**AADAT**	aminoadipate aminotrans	protein_coding	0	0	1	1	1.000
51167	NM_016230	**CYB5R4**	cytochrome b5 reductase	protein_coding	19	16	20	17	0.857
51168	NM_016239,XM_01:	**MYO15A**	myosin XVA	protein_coding	0	0	1	1	1.000
51170	NM_016245,XM_01:	**HSD17B11**	hydroxysteroid 17-beta d	protein_coding	5	3	6	4	0.678
51171	NM_016246,XM_00!	**HSD17B14**	hydroxysteroid 17-beta d	protein_coding	1	1	2	2	0.878
51172	NM_016256,XM_01:	**NAGPA**	N-acetylglucosamine-1-p	protein_coding	8	9	9	10	1.135
51174	NM_001193609,NM	**TUBD1**	tubulin delta 1	protein_coding	11	10	12	11	0.972
51175	NM_016262,XM_01:	**TUBE1**	tubulin epsilon 1	protein_coding	21	19	22	20	0.917
51176	NM_001130713,NM	**LEF1**	lymphoid enhancer bindi	protein_coding	0	0	1	1	1.000
51177	NM_001304722,NM	**PLEKHO1**	pleckstrin homology dom	protein_coding	136	134	137	135	0.979
51179	NM_001005783,NM	**HAO2**	hydroxyacid oxidase 2	protein_coding	0	0	1	1	1.000
51181	NM_001195218,NM	**DCXR**	dicarbonyl and L-xylulos(	protein_coding	95	98	96	99	1.026
51182	NM_016299	**HSPA14**	heat shock protein family	protein_coding	52	48	53	49	0.936
51184	NM_001164372,NM	**GPN3**	GPN-loop GTPase 3	protein_coding	56	59	57	60	1.053
51185	NM_001173482,NM	**CRBN**	cereblon	protein_coding	16	14	17	15	0.908
51186	NM_001006612,NM	**TCEAL9**	transcription elongation f	protein_coding	0	0	1	1	1.000
51187	NM_016304	**RSL24D1**	ribosomal L24 domain cc	protein_coding	207	180	208	181	0.870
51188	NM_001370300,NM	**SS18L2**	SS18 like 2	protein_coding	34	56	35	57	1.629
51191	NM_016323,XM_01:	**HERC5**	HECT and RLD domain (	protein_coding	14	12	15	13	0.843
51192	NM_001040138,NM	**CKLF**	chemokine like factor	protein_coding	228	227	229	228	0.996
51193	NM_001303425,NM	**ZNF639**	zinc finger protein 639	protein_coding	27	24	28	25	0.886
51194	NM_001134779,NM	**IPO11**	importin 11	protein_coding	36	32	37	33	0.889
51195	NM_001303533,NM	**RAPGEFL1**	Rap guanine nucleotide	protein_coding	3	2	4	3	0.811
51196	NM_001165979,NM	**PLCE1**	phospholipase C epsilon	protein_coding	0	0	1	1	1.000
51198	NR_024274	**CDKN2A-DT**	CDKN2A divergent trans	lncRNA	0	0	1	1	1.000
51199	NM_016350,NM_02(	**NIN**	ninein	protein_coding	3	3	4	4	1.022
51200	NM_001163446,NM	**CPA4**	carboxypeptidase A4	protein_coding	0	0	1	1	1.000
51201	NM_001362988,NM	**ZDHHC2**	zinc finger DHHC-type pa	protein_coding	0	0	1	1	1.000
51202	NM_016355,NM_20	**DDX47**	DEAD-box helicase 47	protein_coding	58	51	59	52	0.879
51203	NM_001243142,NM	**NUSAP1**	nucleolar and spindle as	protein_coding	236	215	237	216	0.912
51204	NM_016360	**TACO1**	translational activator of (	protein_coding	57	55	58	56	0.962
51205	NM_001323625,NM	**ACP6**	acid phosphatase 6, lysc	protein_coding	1	1	2	2	0.874
51206	NM_001083899,NM	**GP6**	glycoprotein VI platelet	protein_coding	0	0	1	1	0.880
51207	NM_001007271,NM	**DUSP13**	dual specificity phosphat	protein_coding	0	0	1	1	1.000
51208	NM_001002026,NM	**CLDN18**	claudin 18	protein_coding	0	0	1	1	1.000
51209	NM_016370,NR_14(	**RAB9B**	RAB9B, member RAS or	protein_coding	0	0	1	1	0.975
51213	NM_001318840,NM	**LUZP4**	leucine zipper protein 4	protein_coding	0	0	1	1	1.000
51214	NR_028043,NR_13:	**IGF2-AS**	IGF2 antisense RNA	lncRNA	0	0	1	1	1.000
51218	NM_016417	**GLRX5**	glutaredoxin 5	protein_coding	133	143	134	144	1.073
51222	NM_001101672,NM	**ZNF219**	zinc finger protein 219	protein_coding	1	1	2	2	0.865
51224	NM_016427	**ELOA2**	elongin A2	protein_coding	0	0	1	1	1.000
51225	NM_001135186,NM	**ABI3**	ABI family member 3	protein_coding	42	40	43	41	0.956
51226	NM_016429,XM_01:	**COPZ2**	COPI coat complex subu	protein_coding	2	2	3	3	1.019
51227	NM_001320480,NM	**PIGP**	phosphatidylinositol glyc	protein_coding	36	38	37	39	1.051
51228	NM_016433,XM_01:	**GLTP**	glycolipid transfer proteir	protein_coding	36	36	37	37	1.006
51230	NM_016436,XM_00!	**PHF20**	PHD finger protein 20	protein_coding	30	27	31	28	0.905
51231	NM_001025778,NM	**VRK3**	VRK serine/threonine kin	protein_coding	42	49	43	50	1.163
51232	NM_016441,XM_00!	**CRIM1**	cysteine rich transmemb	protein_coding	38	34	39	35	0.906
51233	NM_016449,XM_01:	**DRICH1**	aspartate rich 1	protein_coding	3	4	4	5	1.105
51234	NM_001286420,NM	**EMC4**	ER membrane protein cc	protein_coding	229	225	230	226	0.981
51236	NM_016458,XR_00:	**HGH1**	HGH1 homolog	protein_coding	25	25	26	26	1.022
51237	NM_016459	**MZB1**	marginal zone B and B1	protein_coding	181	193	182	194	1.066
51239	NM_016466	**ANKRD39**	ankyrin repeat domain 3!	protein_coding	26	27	27	28	1.046
51241	NM_001204090,NM	**COX16**	cytochrome c oxidase as	protein_coding	28	29	29	30	1.041
51244	NM_016474,NR_13!	**CCDC174**	coiled-coil domain contai	protein_coding	17	15	18	16	0.920
51246	NM_001272065,NM	**SHISA5**	shisa family member 5	protein_coding	209	216	210	217	1.032
51247	NM_001033112,NM	**PAIP2**	poly(A) binding protein ir	protein_coding	134	133	135	134	0.994
51248	NM_001370174,NM	**PDZD11**	PDZ domain containing :	protein_coding	64	63	65	64	0.994
51249	NM_016486,XM_02	**TMEM69**	transmembrane protein (	protein_coding	52	47	53	48	0.904
51250	NM_001142468,NM	**MTRES1**	mitochondrial transcriptic	protein_coding	16	16	17	17	0.970
51251	NM_001002009,NM	**NT5C3A**	5'-nucleotidase, cytosolic	protein_coding	41	35	42	36	0.852
51252	NM_001122646,NM	**FAM178B**	family with sequence sin	protein_coding	0	0	1	1	1.000

51253	NM_001330602,NM	**MRPL37**	mitochondrial ribosomal	protein_coding	310	317	311	318	1.023
51255	NM_016494,XM_00!	**RNF181**	ring finger protein 181	protein_coding	104	109	105	110	1.048
51256	NM_001143964,NM	**TBC1D7**	TBC1 domain family mer	protein_coding	23	22	24	23	0.966
51257	NM_001005415,NM	**MARCHF2**	membrane associated rir	protein_coding	5	5	6	6	0.966
51258	NM_016497	**MRPL51**	mitochondrial ribosomal	protein_coding	159	168	160	169	1.055
51259	NM_001173990,NM	**TMEM216**	transmembrane protein 2	protein_coding	34	38	35	39	1.143
51260	NM_001300888,NM	**PBDC1**	polysaccharide biosynthe	protein_coding	65	59	66	60	0.908
51263	NM_145212,NR_028	**MRPL30**	mitochondrial ribosomal	protein_coding	38	36	39	37	0.935
51264	NM_016504	**MRPL27**	mitochondrial ribosomal	protein_coding	120	127	121	128	1.055
51265	NM_001113575,NM	**CDKL3**	cyclin dependent kinase	protein_coding	2	2	3	3	0.939
51266	NM_001099431,NM	**CLEC1B**	C-type lectin domain fam	protein_coding	0	0	1	1	1.000
51267	NM_001297748,NM	**CLEC1A**	C-type lectin domain fam	protein_coding	0	0	1	1	1.000
51268	NM_016518,XM_01	**PIPOX**	pipecolic acid and sarcos	protein_coding	0	0	1	1	1.092
51270	NM_016521	**TFDP3**	transcription factor Dp fa	protein_coding	0	0	1	1	1.000
51271	NM_001171201,NM	**UBAP1**	ubiquitin associated prot	protein_coding	47	41	48	42	0.869
51272	NM_001098787,NM	**BET1L**	Bet1 golgi vesicular mem	protein_coding	64	71	65	72	1.103
51274	NM_016531,XM_01	**KLF3**	Kruppel like factor 3	protein_coding	0	0	1	1	0.984
51275	NR_015404,NR_152	**MAPKAPK5-AS1**	MAPKAPK5 antisense RI	lncRNA	21	18	22	19	0.872
51276	NM_001290314,NM	**ZNF571**	zinc finger protein 571	protein_coding	0	0	1	1	1.000
51277	NM_001198559,NM	**DNAJC27**	DnaJ heat shock protein	protein_coding	3	3	4	4	0.880
51278	NM_016545	**IER5**	immediate early respons	protein_coding	41	42	42	43	1.012
51279	NM_001297640,NM	**C1RL**	complement C1r subcom	protein_coding	5	5	6	6	1.087
51280	NM_016548,NM_17	**GOLM1**	golgi membrane protein	protein_coding	0	0	1	1	0.889
51281	NM_001282771,NM	**ANKMY1**	ankyrin repeat and MYNI	protein_coding	22	22	23	23	0.962
51282	NM_016558,NM_03	**SCAND1**	SCAN domain containing	protein_coding	73	84	74	85	1.143
51283	NM_001330500,NM	**BFAR**	bifunctional apoptosis re	protein_coding	71	68	72	69	0.952
51284	NM_016562	**TLR7**	toll like receptor 7	protein_coding	0	0	1	1	1.000
51285	NM_001307930,NM	**RASL12**	RAS like family 12	protein_coding	0	0	1	1	1.000
51286	NM_016564	**CEND1**	cell cycle exit and neuror	protein_coding	0	0	1	1	1.000
51287	NM_016565,XM_01	**COA4**	cytochrome c oxidase as	protein_coding	226	237	227	238	1.044
51289	NM_016568	**RXFP3**	relaxin family peptide rec	protein_coding	0	0	1	1	1.000
51290	NM_016570,XM_02	**ERGIC2**	ERGIC and golgi 2	protein_coding	19	16	20	17	0.837
51291	NM_001288998,NM	**GMIP**	GEM interacting protein	protein_coding	21	19	22	20	0.940
51292	NM_001002000,NM	**GMPR2**	guanosine monophospha	protein_coding	37	39	38	40	1.051
51293	NM_001165895,NM	**CD320**	CD320 molecule	protein_coding	178	200	179	201	1.119
51294	NM_016580,XM_02	**PCDH12**	protocadherin 12	protein_coding	0	0	1	1	1.000
51295	NM_001142464,NM	**ECSIT**	ECSIT signaling integrat	protein_coding	56	66	57	67	1.165
51296	NM_016582,NR_02	**SLC15A3**	solute carrier family 15 m	protein_coding	0	0	1	1	1.000
51297	NM_001243193,NM	**BPIFA1**	BPI fold containing famil	protein_coding	0	0	1	1	1.000
51298	NM_016585,NM_19	**THEG**	theg spermatid protein	protein_coding	0	0	1	1	0.957
51299	NM_001278710,NM	**NRN1**	neuritin 1	protein_coding	0	0	1	1	1.000
51300	NM_016589,XM_01	**TIMMDC1**	translocase of inner mito	protein_coding	77	62	78	63	0.805
51301	NM_001366737	**GCNT4**	glucosaminyl (N-acetyl) t	protein_coding	0	0	1	1	0.965
51302	NM_001278738,NM	**CYP39A1**	cytochrome P450 family	protein_coding	0	0	1	1	1.000
51303	NM_001143781,NM	**FKBP11**	FKBP prolyl isomerase 1	protein_coding	227	209	228	210	0.924
51304	NM_001135179,NM	**ZDHHC3**	zinc finger DHHC-type p	protein_coding	16	16	17	17	0.963
51305	NM_001282534,NR	**KCNK9**	potassium two pore dom	protein_coding	0	0	1	1	1.000
51306	NM_001101800,NM	**FAM13B**	family with sequence sim	protein_coding	50	47	51	48	0.956
51307	NM_001135647,NM	**FAM53C**	family with sequence sim	protein_coding	50	50	51	51	0.991
51308	NM_001271803,NM	**REEP2**	receptor accessory prote	protein_coding	0	0	1	1	0.907
51309	NM_016608	**ARMCX1**	armadillo repeat containi	protein_coding	0	0	1	1	1.000
51310	NM_001289050,NM	**SLC22A17**	solute carrier family 22 m	protein_coding	0	0	1	1	1.000
51311	NM_016610,NM_13	**TLR8**	toll like receptor 8	protein_coding	0	0	1	1	1.000
51312	NM_001317812,NM	**SLC25A37**	solute carrier family 25 m	protein_coding	19	18	20	19	0.980
51313	NM_001031700,NM	**GASK1B**	golgi associated kinase 1	protein_coding	0	0	1	1	1.000
51314	NM_016616	**NME8**	NME/NM23 family memb	protein_coding	0	0	1	1	1.028
51315	NM_001304526,NM	**KRCC1**	lysine rich coiled-coil 1	protein_coding	15	14	16	15	0.946
51316	NM_001130715,NM	**PLAC8**	placenta associated 8	protein_coding	0	0	1	1	1.000
51317	NM_001101802,NM	**PHF21A**	PHD finger protein 21A	protein_coding	12	10	13	11	0.862
51318	NM_001363782,NM	**MRPL35**	mitochondrial ribosomal	protein_coding	29	32	30	33	1.092
51319	NM_001271834,NM	**RSRC1**	arginine and serine rich c	protein_coding	31	31	32	32	0.992
51320	NM_016626	**MEX3C**	mex-3 RNA binding famil	protein_coding	90	77	91	78	0.855
51321	NM_001033569,NM	**AMZ2**	archaelysin family metall	protein_coding	93	95	94	96	1.020
51322	NM_016628,NM_10	**WAC**	WW domain containing a	protein_coding	34	28	35	29	0.820
51324	NM_001127889,NM	**SPG21**	SPG21 abhydrolase dom	protein_coding	93	83	94	84	0.895
51326	NM_001113738,NM	**ARL17A**	ADP ribosylation factor li	protein_coding	19	15	20	16	0.799
51327	NM_001318221,NM	**AHSP**	alpha hemoglobin stabili	protein_coding	0	0	1	1	1.000
51329	NM_001002251,NM	**ARL6IP4**	ADP ribosylation factor li	protein_coding	135	147	136	148	1.088
51330	NM_016639	**TNFRSF12A**	TNF receptor superfamily	protein_coding	5	6	6	7	1.247
51332	NM_016642,XM_01	**SPTBN5**	spectrin beta, non-erythr	protein_coding	0	0	1	1	1.024
51333	NM_001142305,NM	**ZNF771**	zinc finger protein 771	protein_coding	1	1	2	2	1.124
51334	NM_001300783,NM	**PRR16**	proline rich 16	protein_coding	0	0	1	1	1.000
51335	NM_001033088,NR	**NGRN**	neugrin, neurite outgrow	protein_coding	102	96	103	97	0.944
51337	NM_001363000,NM	**THEM6**	thioesterase superfamily	protein_coding	10	12	11	13	1.215
51338	NM_001243266,NM	**MS4A4A**	membrane spanning 4-d	protein_coding	0	0	1	1	1.000
51339	NM_001079520,NM	**DACT1**	dishevelled binding anta	protein_coding	0	0	1	1	0.954
51340	NM_001278625,NM	**CRNKL1**	crooked neck pre-mRNA	protein_coding	38	33	39	34	0.886
51341	NM_001317990,NM	**ZBTB7A**	zinc finger and BTB dom	protein_coding	24	24	25	25	0.998
51343	NM_001136197,NM	**FZR1**	fizzy and cell division cyc	protein_coding	31	32	32	33	1.036
51347	NM_001346487,NM	**TAOK3**	TAO kinase 3	protein_coding	56	46	57	47	0.827
51348	NM_001291822,NM	**KLRF1**	killer cell lectin like recep	protein_coding	0	0	1	1	0.928
51350	NM_015848	**KRT76**	keratin 76	protein_coding	0	0	1	1	1.000
51351	NM_015852	**ZNF117**	zinc finger protein 117	protein_coding	2	1	3	2	0.755
51352	NR_023920,NR_12	**WT1-AS**	WT1 antisense RNA	lncRNA	0	0	1	1	1.000
51360	NM_015884	**MBTPS2**	membrane bound transc	protein_coding	19	16	20	17	0.874
51361	NM_015888,XM_00	**HOOK1**	hook microtubule tetheri	protein_coding	0	0	1	1	1.000
51362	NM_015891	**CDC40**	cell division cycle 40	protein_coding	30	28	31	29	0.933
51363	NM_001270764,NM	**CHST15**	carbohydrate sulfotransf	protein_coding	0	0	1	1	1.000
51364	NM_001308379,NM	**ZMYND10**	zinc finger MYND-type c	protein_coding	4	5	5	6	1.231
51365	NM_001206960,NM	**PLA1A**	phospholipase A1 memb	protein_coding	0	0	1	1	1.000
51366	NM_001282873,NM	**UBR5**	ubiquitin protein ligase E	protein_coding	61	51	62	52	0.843
51367	NM_015918,NM_19	**POP5**	POP5 homolog, ribonucl	protein_coding	62	60	63	61	0.955
51368	NM_001129884,NM	**TEX264**	testis expressed 264, ER	protein_coding	44	53	45	54	1.216
51371	NM_015932	**POMP**	proteasome maturation p	protein_coding	247	236	248	237	0.956
51372	NM_001329417,NM	**TMA7**	translation machinery as	protein_coding	499	547	500	548	1.097
51373	NM_015969	**MRPS17**	mitochondrial ribosomal	protein_coding	25	28	26	29	1.105
51374	NM_001170795,NM	**ATRAID**	all-trans retinoic acid ind	protein_coding	96	100	97	101	1.046
51375	NM_001364903,NM	**SNX7**	sorting nexin 7	protein_coding	41	39	42	40	0.960
51377	NM_001199261,NM	**UCHL5**	ubiquitin C-terminal hydr	protein_coding	70	64	71	65	0.914
51378	NM_001322809,NM	**ANGPT4**	angiopoietin 4	protein_coding	0	0	1	1	1.010
51379	NM_015986,NR_07	**CRLF3**	cytokine receptor like fac	protein_coding	50	45	51	46	0.915
51380	NM_001244705,NM	**CSAD**	cysteine sulfinic acid dec	protein_coding	5	5	6	6	1.029
51382	NM_015994	**ATP6V1D**	ATPase H+ transporting	protein_coding	35	36	36	37	1.019
51384	NM_016087,NM_05	**WNT16**	Wnt family member 16	protein_coding	0	0	1	1	1.000
51385	NM_016089	**ZNF589**	zinc finger protein 589	protein_coding	17	15	18	16	0.861
51386	NM_001242923,NM	**EIF3L**	eukaryotic translation init	protein_coding	227	220	228	221	0.966
51388	NM_001199434,NM	**NIP7**	nucleolar pre-rRNA proc	protein_coding	155	149	156	150	0.962
51389	NM_001007464,NM	**RWDD1**	RWD domain containing	protein_coding	28	27	29	28	0.958
51390	NM_001286587,NM	**AIG1**	androgen induced 1	protein_coding	21	17	22	18	0.789
51393	NM_016113,XM_00!	**TRPV2**	transient receptor potent	protein_coding	35	34	36	35	0.974
51397	NM_001308080,NM	**COMMD10**	COMM domain containin	protein_coding	21	15	22	16	0.732
51398	NM_016145,XM_01	**WDR83OS**	WD repeat domain 83 op	protein_coding	130	141	131	142	1.083
51399	NM_001318486,NM	**TRAPPC4**	trafficking protein particle	protein_coding	72	76	73	77	1.055
51400	NM_001271593,NM	**PPME1**	protein phosphatase met	protein_coding	36	38	37	39	1.055
51406	NM_001317724,NM	**NOL7**	nucleolar protein 7	protein_coding	166	165	167	166	0.995
51409	NM_001317851,NM	**HEMK1**	HemK methyltransferase	protein_coding	19	14	20	15	0.733
51411	NM_001290007,NM	**BIN2**	bridging integrator 2	protein_coding	74	76	75	77	1.028
51412	NM_016188,NR_13	**ACTL6B**	actin like 6B	protein_coding	0	0	1	1	1.000
51421	NM_001278683,NM	**AMOTL2**	angiomotin like 2	protein_coding	0	0	1	1	1.000
51422	NM_001040633,NM	**PRKAG2**	protein kinase AMP-activ	protein_coding	0	0	1	1	1.000
51426	NM_001345921,NM	**POLK**	DNA polymerase kappa	protein_coding	11	10	12	11	0.905
51427	NM_001013746,NM	**ZNF107**	zinc finger protein 107	protein_coding	41	37	42	38	0.912
51428	NM_001321732,NM	**DDX41**	DEAD-box helicase 41	protein_coding	81	84	82	85	1.037
51429	NM_016224,XM_00!	**SNX9**	sorting nexin 9	protein_coding	0	0	1	1	1.000
51430	NM_001282750,NM	**SUCO**	SUN domain containing	protein_coding	22	19	23	20	0.878
51433	NM_001137559,NM	**ANAPC5**	anaphase promoting con	protein_coding	110	113	111	114	1.031

51434	NM_001137664,NM	ANAPC7	anaphase promoting con	protein_coding	80	82	81	83	1.025
51435	NM_016240,NM_18	SCARA3	scavenger receptor class	protein_coding	0	0	1	1	0.980
51438	NM_016249	MAGEC2	MAGE family member C2	protein_coding	0	0	1	1	1.000
51439	NM_016255	FAM8A1	family with sequence sim	protein_coding	14	14	15	15	1.050
51440	NM_001282396,NM	HPCAL4	hippocalcin like 4	protein_coding	0	0	1	1	1.021
51441	NM_001172828,NM	YTHDF2	YTH N6-methyladenosin	protein_coding	113	105	114	106	0.930
51442	NM_016267	VGLL1	vestigial like family meml	protein_coding	0	0	1	1	1.000
51444	NM_001191324,NM	RNF138	ring finger protein 138	protein_coding	124	110	125	111	0.891
51447	NM_001005909,NM	IP6K2	inositol hexakisphosphat	protein_coding	31	47	32	48	1.505
51449	NM_016297	PCYOX1	prenylcysteine oxidase 1	protein_coding	2	2	3	3	0.922
51450	NM_016307,XM_01	PRRX2	paired related homeobox	protein_coding	0	0	1	1	1.000
51451	NM_001032391,NM	LCMT1	leucine carboxyl methylt	protein_coding	40	47	41	48	1.173
51454	NM_001252668,NM	GULP1	GULP PTB domain conta	protein_coding	0	0	1	1	0.983
51455	NM_001037872,NM	REV1	REV1 DNA directed poly	protein_coding	21	18	22	19	0.874
51458	NM_001321041,NM	RHCG	Rh family C glycoprotein	protein_coding	0	0	1	1	1.000
51460	NM_016329,XM_00	SFMBT1	Scm like with four mbt do	protein_coding	11	14	12	15	1.221
51463	NM_001350180,NM	GPR89B	G protein-coupled recept	protein_coding	26	26	27	27	0.979
51465	NM_016021,XM_01	UBE2J1	ubiquitin conjugating enz	protein_coding	32	27	33	28	0.862
51466	NM_001330221,NM	EVL	Enah/Vasp-like	protein_coding	90	95	91	96	1.055
51471	NR_132338	NAT8B	N-acetyltransferase 8B (p	transcribed_pseu	0	0	1	1	1.000
51473	NM_001195610,NM	DCDC2	doublecortin domain con	protein_coding	0	0	1	1	1.035
51474	NM_001113546,NM	LIMA1	LIM domain and actin bir	protein_coding	21	15	22	16	0.735
51475	NM_001318496,NM	CABP2	calcium binding protein 2	protein_coding	0	0	1	1	1.000
51477	NM_001170938,NM	ISYNA1	inositol-3-phosphate syn	protein_coding	1	1	2	2	1.055
51478	NM_001304512,NM	HSD17B7	hydroxysteroid 17-beta d	protein_coding	27	27	28	28	0.978
51479	NM_001257999,NM	ANKFY1	ankyrin repeat and FYVE	protein_coding	12	10	13	11	0.890
51480	NM_016378	VCX2	variable charge X-linked	protein_coding	0	0	1	1	1.000
51481	NM_016379	VCX3A	variable charge X-linked	protein_coding	0	0	1	1	1.000
51490	NM_016390,XM_01	SPOUT1	SPOUT domain containi	protein_coding	36	37	37	38	1.037
51491	NM_001256539,NM	NOP16	NOP16 nucleolar protein	protein_coding	356	364	357	365	1.022
51493	NM_014306	RTCB	RNA 2',3'-cyclic phospha	protein_coding	119	122	120	123	1.018
51495	NM_016395,XM_01	HACD3	3-hydroxyacyl-CoA dehy	protein_coding	183	164	184	165	0.900
51496	NM_016396,XM_00	CTDSPL2	CTD small phosphatase	protein_coding	42	36	43	37	0.854
51497	NM_198976,XR_24	NELFCD	negative elongation facto	protein_coding	330	326	331	327	0.988
51499	NM_016399	TRIAP1	TP53 regulated inhibitor	protein_coding	76	78	77	79	1.024
51501	NM_001322404,NM	HIKESHI	heat shock protein nucle	protein_coding	91	98	92	99	1.069
51503	NM_001363371,NM	CWC15	CWC15 spliceosome ass	protein_coding	82	89	83	90	1.073
51504	NM_001286082,NM	TRMT112	tRNA methyltransferase	protein_coding	349	357	350	358	1.024
51506	NM_016406,XM_00	UFC1	ubiquitin-fold modifier co	protein_coding	202	209	203	210	1.034
51507	NM_001283035,NM	RTF2	replication termination fa	protein_coding	188	183	189	184	0.974
51510	NM_001195536,NM	CHMP5	charged multivesicular b	protein_coding	74	70	75	71	0.945
51512	NM_016426,XM_00	GTSE1	G2 and S-phase express	protein_coding	46	44	47	45	0.955
51513	NM_001207035,NM	ETV7	ETS variant transcription	protein_coding	7	7	8	8	0.960
51514	NM_001286229,NM	DTL	denticleless E3 ubiquitin	protein_coding	59	58	60	59	0.976
51517	NM_016453,NM_18	NCKIPSD	NCK interacting protein v	protein_coding	24	26	25	27	1.060
51520	NM_001317964,NM	LARS1	leucyl-tRNA synthetase 1	protein_coding	136	118	137	119	0.872
51522	NM_001165258,NM	TMEM14C	transmembrane protein 1	protein_coding	0	0	1	1	1.000
51523	NM_001317199,NM	CXXC5	CXXC finger protein 5	protein_coding	13	15	14	16	1.153
51524	NM_001330281,NM	TMEM138	transmembrane protein 1	protein_coding	53	59	54	60	1.118
51526	NM_016470,XM_01	OSER1	oxidative stress responsi	protein_coding	33	33	34	34	1.013
51527	NM_001271904,NM	GSKIP	GSK3B interacting protei	protein_coding	24	23	25	24	0.967
51528	NM_001098625,NM	JKAMP	JNK1/MAPK8 associated	protein_coding	29	25	30	26	0.840
51529	NM_001002244,NM	ANAPC11	anaphase promoting con	protein_coding	99	105	100	106	1.058
51530	NM_001282190,NM	ZC3HC1	zinc finger C3HC-type co	protein_coding	48	45	49	46	0.956
51531	NM_001330725,NM	TRMO	tRNA methyltransferase	protein_coding	10	9	11	10	0.894
51533	NM_001278221,NM	PHF7	PHD finger protein 7	protein_coding	6	6	7	7	1.053
51534	NM_001286371,NM	VTA1	vesicle trafficking 1	protein_coding	28	25	29	26	0.908
51535	NM_001143787,NM	PPHLN1	periphilin 1	protein_coding	49	50	50	51	1.024
51537	NM_001003704,NM	MTFP1	mitochondrial fission pro	protein_coding	98	97	99	98	0.991
51538	NM_001282566,NM	ZCCHC17	zinc finger CCHC-type co	protein_coding	100	91	101	92	0.915
51540	NM_016510	SCLY	selenocysteine lyase	protein_coding	62	68	63	69	1.091
51542	NM_001005739,NM	VPS54	VPS54 subunit of GARP	protein_coding	12	11	13	12	0.928
51545	NM_016535,XM_00	ZNF581	zinc finger protein 581	protein_coding	29	32	30	33	1.106
51547	NM_016538,XM_01	SIRT7	sirtuin 7	protein_coding	29	36	30	37	1.238
51548	NM_001193285,NM	SIRT6	sirtuin 6	protein_coding	52	55	53	56	1.053
51550	NM_001320046,NM	CINP	cyclin dependent kinase	protein_coding	59	61	60	62	1.030
51552	NM_016322	RAB14	RAB14, member RAS on	protein_coding	56	48	57	49	0.873
51554	NM_016557,NM_17	ACKR4	atypical chemokine recep	protein_coding	0	0	1	1	0.981
51555	NM_001256750,NM	PEX5L	peroxisomal biogenesis f	protein_coding	0	0	1	1	0.994
51557	NM_001143940,NM	LGSN	lengsin, lens protein with	protein_coding	0	0	1	1	0.971
51559	NM_001031701,XM	NT5DC3	5'-nucleotidase domain c	protein_coding	2	2	3	3	1.055
51560	NM_001363953,NM	RAB6B	RAB6B, member RAS on	protein_coding	0	0	1	1	1.000
51561	NM_016584,XM_01	IL23A	interleukin 23 subunit alp	protein_coding	1	1	2	2	1.192
51562	NM_001144891,NM	MBIP	MAP3K12 binding inhibit	protein_coding	20	20	21	21	0.976
51564	NM_001098416,NM	HDAC7	histone deacetylase 7	protein_coding	71	69	72	70	0.974
51566	NM_016607,NM_17	ARMCX3	armadillo repeat containi	protein_coding	17	16	18	17	0.939
51567	NM_016614	TDP2	tyrosyl-DNA phosphodies	protein_coding	129	122	130	123	0.951
51569	NM_001286703,NM	UFM1	ubiquitin fold modifier 1	protein_coding	98	89	99	90	0.911
51571	NM_001256763,NM	CYRIB	CYFIP related Rac1 inter	protein_coding	188	165	189	166	0.877
51573	NM_001324066,NM	GDE1	glycerophosphodiester p	protein_coding	71	71	72	72	1.002
51574	NM_001267039,NM	LARP7	La ribonucleoprotein 7, tr	protein_coding	60	55	61	56	0.925
51575	NM_001276380,NM	ESF1	ESF1 nucleolar pre-rRNA	protein_coding	55	45	56	46	0.831
51582	NM_001301668,NM	AZIN1	antizyme inhibitor 1	protein_coding	184	174	185	175	0.945
51585	NM_001346413,NM	PCF11	PCF11 cleavage and pol	protein_coding	31	35	32	36	1.115
51586	NM_001003891,NM	MED15	mediator complex subun	protein_coding	24	25	25	26	1.047
51588	NM_015897,XM_01	PIAS4	protein inhibitor of activa	protein_coding	35	37	36	38	1.050
51592	NM_015906,NM_03	TRIM33	tripartite motif containing	protein_coding	41	35	42	36	0.860
51593	NM_001128852,NM	SRRT	serrate, RNA effector mo	protein_coding	166	169	167	170	1.018
51594	NM_015909,NR_05	NBAS	NBAS subunit of NRZ te	protein_coding	8	7	9	8	0.880
51596	NM_001014433,NM	CUTA	cutA divalent cation toler	protein_coding	192	194	193	195	1.010
51599	NM_001260489,NM	LSR	lipolysis stimulated lipop	protein_coding	0	0	1	1	0.925
51601	NM_001204830,NM	LIPT1	lipoyltransferase 1	protein_coding	8	7	9	8	0.902
51602	NM_015934	NOP58	NOP58 ribonucleoprotein	protein_coding	284	261	285	262	0.919
51603	NM_001007239,NM	EEF1AKNMT	eEF1A lysine and N-term	protein_coding	70	70	71	71	0.988
51604	NM_001184728,NM	PIGT	phosphatidylinositol glyc	protein_coding	95	100	96	101	1.049
51605	NM_001281467,NM	TRMT6	tRNA methyltransferase	protein_coding	92	85	93	86	0.927
51606	NM_015941,NM_21	ATP6V1H	ATPase H+ transporting	protein_coding	29	30	30	31	1.024
51608	NM_015949	GET4	guided entry of tail-anch	protein_coding	50	54	51	55	1.068
51611	NM_001077394,NM	DPH5	diphthamide biosynthesi	protein_coding	31	43	32	44	1.355
51614	NM_015966,NM_19	ERGIC3	ERGIC and golgi 3	protein_coding	187	192	188	193	1.027
51616	NM_015975	TAF9B	TATA-box binding protei	protein_coding	42	40	43	41	0.973
51617	NM_015980	NSG2	neuronal vesicle traffickir	protein_coding	0	0	1	1	1.000
51619	NM_015983,XM_00	UBE2D4	ubiquitin conjugating enz	protein_coding	5	7	6	8	1.247
51621	NM_001302461,NM	KLF13	Kruppel like factor 13	protein_coding	85	81	86	82	0.957
51622	NM_015622	CCZ1	CCZ1 homolog, vacuolar	protein_coding	49	47	50	48	0.952
51626	NM_001193464,NM	DYNC2LI1	dynein cytoplasmic 2 ligh	protein_coding	11	9	12	10	0.765
51629	NM_001143780,NM	SLC25A39	solute carrier family 25 m	protein_coding	241	257	242	258	1.069
51631	NM_001244585,NM	LUC7L2	LUC7 like 2, pre-mRNA s	protein_coding	184	175	185	176	0.950
51633	NM_001286745,NM	OTUD6B	OTU deubiquitinase 6B	protein_coding	48	44	49	45	0.903
51634	NM_016024	RBMX2	RNA binding motif protei	protein_coding	43	39	44	40	0.912
51635	NM_001322280,NM	DHRS7	dehydrogenase/reductas	protein_coding	42	36	43	37	0.865
51637	NM_016039	RTRAF	RNA transcription, transl	protein_coding	46	46	47	47	0.996
51639	NM_016047	SF3B6	splicing factor 3b subuni	protein_coding	359	344	360	345	0.958
51642	NM_001318498,NM	MRPL48	mitochondrial ribosomal	protein_coding	46	49	47	50	1.059
51643	NM_001282606,NM	TMBIM4	transmembrane BAX inh	protein_coding	29	28	30	29	0.962
51645	NM_016059	PPIL1	peptidylprolyl isomerase	protein_coding	150	141	151	142	0.939
51646	NM_001127399,NM	YPEL5	yippee like 5	protein_coding	1	1	2	2	0.966
51647	NM_016062,NR_02	CIAO2B	cytosolic iron-sulfur asse	protein_coding	224	231	225	232	1.031
51649	NM_016070	MRPS23	mitochondrial ribosomal	protein_coding	43	43	44	44	1.004
51650	NM_016071,NM_05	MRPS33	mitochondrial ribosomal	protein_coding	19	20	20	21	1.052
51651	NM_001015509,NM	PTRH2	peptidyl-tRNA hydrolase	protein_coding	88	83	89	84	0.938
51652	NM_001005753,NM	CHMP3	charged multivesicular b	protein_coding	41	35	42	36	0.853
51654	NM_001278167,NM	CDK5RAP1	CDK5 regulatory subunit	protein_coding	81	78	82	79	0.963
51655	NM_001199989,NM	RASD1	ras related dexamethaso	protein_coding	0	0	1	1	1.000
51657	NM_001317785,NM	STYXL1	serine/threonine/tyrosine	protein_coding	27	29	28	30	1.073
51659	NM_016095	GINS2	GINS complex subunit 2	protein_coding	42	42	43	43	1.019

51660	NM_001270879,NM	**MPC1**	mitochondrial pyruvate c	protein_coding	68	56	69	57	0.826
51661	NM_001135212,NM	**FKBP7**	FKBP prolyl isomerase 7	protein_coding	17	15	18	16	0.888
51663	NM_016107,NR_14	**ZFR**	zinc finger RNA binding	protein_coding	93	85	94	86	0.908
51665	NM_001040445,NM	**ASB1**	ankyrin repeat and SOC	protein_coding	30	26	31	27	0.869
51666	NM_016116,NM_14	**ASB4**	ankyrin repeat and SOC	protein_coding	12	11	13	12	0.911
51667	NM_001243351,NM	**NUB1**	negative regulator of ubi	protein_coding	36	33	37	34	0.918
51668	NM_001316935,NM	**HSPB11**	heat shock protein family	protein_coding	241	240	242	241	0.998
51669	NM_001284239,NM	**SARAF**	store-operated calcium e	protein_coding	57	57	58	58	1.000
51673	NM_015964,NM_01	**TPPP3**	tubulin polymerization pr	protein_coding	0	0	1	1	1.000
51676	NM_001202429,NM	**ASB2**	ankyrin repeat and SOC	protein_coding	41	36	42	37	0.891
51678	NM_001303037,NM	**MPP6**	membrane palmitoylated	protein_coding	8	7	9	8	0.836
51684	NM_001178133,NM	**SUFU**	SUFU negative regulator	protein_coding	8	8	9	9	0.933
51686	NM_001134939,NM	**OAZ3**	ornithine decarboxylase	protein_coding	0	0	1	1	1.126
51690	NM_016199,XM_01	**LSM7**	LSM7 homolog, U6 smal	protein_coding	391	421	392	422	1.078
51691	NM_016200	**LSM8**	LSM8 homolog, U6 smal	protein_coding	18	16	19	17	0.908
51692	NM_001321833,NM	**CPSF3**	cleavage and polyadeny	protein_coding	82	78	83	79	0.959
51693	NM_001318524,NM	**TRAPPC2L**	trafficking protein particle	protein_coding	35	37	36	38	1.084
51696	NM_016217	**HECA**	hdc homolog, cell cycle	protein_coding	14	13	15	14	0.955
51699	NM_001282150,NM	**VPS29**	VPS29 retromer complex	protein_coding	99	93	100	94	0.937
51700	NM_001302826,NM	**CYB5R2**	cytochrome b5 reductase	protein_coding	0	0	1	1	1.000
51701	NM_016231,XM_00	**NLK**	nemo like kinase	protein_coding	23	20	24	21	0.906
51702	NM_016233,XM_01	**PADI3**	peptidyl arginine deimina	protein_coding	0	0	1	1	1.000
51703	NM_016234,NM_20	**ACSL5**	acyl-CoA synthetase lon	protein_coding	103	94	104	95	0.915
51704	NM_001304771,NM	**GPRC5B**	G protein-coupled recep	protein_coding	0	0	1	1	1.000
51705	NM_001159694,NM	**EMCN**	endomucin	protein_coding	0	0	1	1	1.000
51706	NM_016243	**CYB5R1**	cytochrome b5 reductase	protein_coding	15	13	16	14	0.909
51710	NM_001164276,NM	**ZNF44**	zinc finger protein 44	protein_coding	8	10	9	11	1.244
51714	NM_016275	**SELENOT**	selenoprotein T	protein_coding	103	99	104	100	0.961
51715	NM_001278666,NM	**RAB23**	RAB23, member RAS on	protein_coding	6	6	7	7	0.902
51716	NR_003276	**CES1P1**	carboxylesterase 1 pseu	transcribed_pseu	0	0	1	1	1.000
51719	NM_001130849,NM	**CAB39**	calcium binding protein 3	protein_coding	177	152	178	153	0.857
51720	NM_001199297,NM	**UIMC1**	ubiquitin interaction moti	protein_coding	56	53	57	54	0.956
51725	NM_016298	**FBXO40**	F-box protein 40	protein_coding	0	0	1	1	1.000
51726	NM_001378451,NM	**DNAJB11**	DnaJ heat shock protein	protein_coding	310	326	311	327	1.050
51727	NM_001136140,NM	**CMPK1**	cytidine/uridine monopho	protein_coding	178	156	179	157	0.874
51728	NM_016310	**POLR3K**	RNA polymerase III subu	protein_coding	129	127	130	128	0.988
51729	NM_016312	**WBP11**	WW domain binding prot	protein_coding	59	54	60	55	0.911
51733	NM_016327,XM_01	**UPB1**	beta-ureidopropionase 1	protein_coding	0	0	1	1	0.983
51734	NM_001382264,NM	**MSRB1**	methionine sulfoxide red	protein_coding	37	38	38	39	1.021
51735	NM_001164386,NM	**RAPGEF6**	Rap guanine nucleotide	protein_coding	26	21	27	22	0.828
51738	NM_001134941,NM	**GHRL**	ghrelin and obestatin pre	protein_coding	0	0	1	1	0.895
51741	NM_001291997,NM	**WWOX**	WW domain containing	protein_coding	16	16	17	17	1.045
51742	NM_001206794,NM	**ARID4B**	AT-rich interaction doma	protein_coding	23	20	24	21	0.871
51744	NM_001166663,NM	**CD244**	CD244 molecule	protein_coding	153	149	154	150	0.971
51747	NM_001330330,NM	**LUC7L3**	LUC7 like 3 pre-mRNA s	protein_coding	119	93	120	94	0.779
51750	NM_001283009,NM	**RTEL1**	regulator of telomere elo	protein_coding	16	12	17	13	0.767
51751	NM_001271880,NM	**HIGD1B**	HIG1 hypoxia inducible	protein_coding	0	0	1	1	0.873
51752	NM_001040458,NM	**ERAP1**	endoplasmic reticulum a	protein_coding	72	60	73	61	0.839
51754	NM_001042589,NM	**TMEM8B**	transmembrane protein 8	protein_coding	0	0	1	1	1.165
51755	NM_015083,NM_01	**CDK12**	cyclin dependent kinase	protein_coding	71	65	72	66	0.913
51759	NM_016520,XM_01	**C9orf78**	chromosome 9 open rea	protein_coding	253	249	254	250	0.983
51760	NM_001308157,NM	**SYT17**	synaptotagmin 17	protein_coding	0	0	1	1	1.000
51761	NM_001313741,NM	**ATP8A2**	ATPase phospholipid tra	protein_coding	1	0	2	1	0.948
51762	NM_016530,XM_01	**RAB8B**	RAB8B, member RAS or	protein_coding	84	70	85	71	0.833
51763	NM_001135642,NM	**INPP5K**	inositol polyphosphate-5	protein_coding	9	9	10	10	1.036
51764	NM_016541	**GNG13**	G protein subunit gamm	protein_coding	0	0	1	1	1.000
51765	NM_001042452,NM	**STK26**	serine/threonine kinase 2	protein_coding	96	83	97	84	0.859
51768	NM_016551,XM_00	**TM7SF3**	transmembrane 7 superf	protein_coding	55	52	56	53	0.948
51773	NM_016578,XM_00	**RSF1**	remodeling and spacing	protein_coding	22	18	23	19	0.858
51776	NM_016653,NM_13	**MAP3K20**	mitogen-activated protein	protein_coding	34	32	35	33	0.938
51778	NM_016599,XM_00	**MYOZ2**	myozenin 2	protein_coding	0	0	1	1	1.000
51780	NM_016604,XM_00	**KDM3B**	lysine demethylase 3B	protein_coding	39	37	40	38	0.937
51802	NM_017419,XM_01	**ASIC5**	acid sensing ion channel	protein_coding	0	0	1	1	1.000
51804	NM_017420,XM_00	**SIX4**	SIX homeobox 4	protein_coding	0	0	1	1	1.000
51805	NM_017421,XM_00	**COQ3**	coenzyme Q3, methyltra	protein_coding	11	10	12	11	0.961
51806	NM_017422	**CALML5**	calmodulin like 5	protein_coding	0	0	1	1	1.000
51807	NM_001193414,NM	**TUBA8**	tubulin alpha 8	protein_coding	1	1	2	2	1.102
51808	NM_032177	**PHAX**	phosphorylated adaptor	protein_coding	46	43	47	44	0.942
51809	NM_001375599,NM	**GALNT7**	polypeptide N-acetylgala	protein_coding	20	18	21	19	0.913
51816	NM_001282225,NM	**ADA2**	adenosine deaminase 2	protein_coding	10	9	11	10	0.909
53335	NM_001363864,NM	**BCL11A**	BAF chromatin remodelin	protein_coding	0	0	1	1	1.000
53336	NM_001184771,NM	**CPXCR1**	CPX chromosome region	protein_coding	0	0	1	1	1.000
53338	gene-CAPZA1P1	**CAPZA1P1**	CAPZA1 pseudogene 1	pseudogene	0	0	1	1	0.985
53339	NM_001011885,NM	**BTBD1**	BTB domain containing 1	protein_coding	129	115	130	116	0.893
53340	NM_017425,XM_01	**SPA17**	sperm autoantigenic prot	protein_coding	5	4	6	5	0.854
53342	NM_138284,XM_00	**IL17D**	interleukin 17D	protein_coding	3	3	4	4	1.095
53343	NM_001248011,NM	**NUDT9**	nudix hydrolase 9	protein_coding	5	5	6	6	0.998
53344	NM_001039840,NM	**CHIC1**	cysteine rich hydrophobi	protein_coding	5	5	6	6	0.938
53345	NM_001001524	**TM6SF2**	transmembrane 6 superf	protein_coding	0	0	1	1	1.000
53346	NM_001144903,NM	**TM6SF1**	transmembrane 6 superf	protein_coding	0	0	1	1	0.998
53347	NM_001001895,NM	**UBASH3A**	ubiquitin associated and	protein_coding	21	21	22	22	0.982
53349	NM_001281734,NM	**ZFYVE1**	zinc finger FYVE-type c	protein_coding	5	6	6	7	1.090
53353	NM_018557,XM_01	**LRP1B**	LDL receptor related pro	protein_coding	2	2	3	3	0.929
53354	NM_138316,NM_14	**PANK1**	pantothenate kinase 1	protein_coding	6	5	7	6	0.848
53358	NM_016848,XM_01	**SHC3**	SHC adaptor protein 3	protein_coding	0	0	1	1	1.000
53371	NM_001278603,NM	**NUP54**	nucleoporin 54	protein_coding	73	67	74	68	0.924
53373	NM_001143819,NM	**TPCN1**	two pore segment chann	protein_coding	2	1	3	2	0.862
53383	gene-MCUR1P1	**MCUR1P1**	MCUR1 pseudogene 1	pseudogene	0	0	1	1	1.076
53405	NM_001114086,NM	**CLIC5**	chloride intracellular cha	protein_coding	0	0	1	1	1.061
53407	NM_001346281,NM	**STX18**	syntaxin 18	protein_coding	38	40	39	41	1.060
53409	gene-ATP5MGP3	**ATP5MGP3**	ATP synthase membrane	pseudogene	0	0	1	1	1.000
53410	gene-ATP5MGP2	**ATP5MGP2**	ATP synthase membrane	pseudogene	0	0	1	1	1.000
53615	NM_001281453,NM	**MBD3**	methyl-CpG binding dom	protein_coding	45	49	46	50	1.084
53616	NM_001324417,NM	**ADAM22**	ADAM metallopeptidase	protein_coding	4	3	5	4	0.950
53630	NM_017429,XM_01	**BCO1**	beta-carotene oxygenase	protein_coding	0	0	1	1	1.000
53632	NM_017431,XM_01	**PRKAG3**	protein kinase AMP-activ	protein_coding	0	0	1	1	1.000
53635	NM_001305105,NM	**PTOV1**	PTOV1 extended AT-hoc	protein_coding	21	23	22	24	1.075
53637	NM_001166215,NM	**S1PR5**	sphingosine-1-phosphate	protein_coding	0	0	1	1	1.000
53820	NM_001317768,NM	**RIPPLY3**	ripply transcriptional rep	protein_coding	0	0	1	1	1.000
53822	NM_022006	**FXYD7**	FXYD domain containing	protein_coding	0	0	1	1	1.000
53826	NM_001164831,NM	**FXYD6**	FXYD domain containing	protein_coding	0	0	1	1	1.000
53827	NM_001164605,NM	**FXYD5**	FXYD domain containing	protein_coding	264	286	265	287	1.085
53828	NM_173160	**FXYD4**	FXYD domain containing	protein_coding	0	0	1	1	1.000
53829	NM_176894,XM_00	**P2RY13**	purinergic receptor P2Y1	protein_coding	0	0	1	1	1.000
53831	NM_020370,XM_01	**GPR84**	G protein-coupled recep	protein_coding	1	1	2	2	1.219
53832	NM_001278722,NM	**IL20RA**	interleukin 20 receptor su	protein_coding	0	0	1	1	1.000
53833	NM_144717,XM_00	**IL20RB**	interleukin 20 receptor su	protein_coding	1	1	2	2	1.104
53834	NM_001004356,NM	**FGFRL1**	fibroblast growth factor r	protein_coding	0	0	1	1	1.000
53836	NM_023915	**GPR87**	G protein-coupled recep	protein_coding	0	0	1	1	1.134
53838	NM_001300913,NM	**C11orf24**	chromosome 11 open rea	protein_coding	37	40	38	41	1.074
53840	NM_001003827,NM	**TRIM34**	tripartite motif containing	protein_coding	10	11	11	12	1.068
53841	NM_001171968,NM	**CDHR5**	cadherin related family m	protein_coding	0	0	1	1	1.000
53842	NM_001111319	**CLDN22**	claudin 22	protein_coding	0	0	1	1	1.000
53844	NR_024086	**COPG2IT1**	COPG2 imprinted transc	lncRNA	0	0	1	1	1.000
53904	NM_001368265,NM	**MYO3A**	myosin IIIA	protein_coding	0	0	1	1	1.000
53905	NM_017434,NM_17	**DUOX1**	dual oxidase 1	protein_coding	0	0	1	1	1.000
53916	NM_016154	**RAB4B**	RAB4B, member RAS or	protein_coding	13	15	14	16	1.169
53917	NM_001031677,NM	**RAB24**	RAB24, member RAS on	protein_coding	14	14	15	15	1.001
53918	NM_015946	**PELO**	pelota mRNA surveillanc	protein_coding	14	14	15	15	1.013
53919	NM_001145944,NM	**SLCO1C1**	solute carrier organic ani	protein_coding	0	0	1	1	1.012
53938	NM_032472,NM_13	**PPIL3**	peptidylprolyl isomerase	protein_coding	71	71	72	72	0.989
53940	NM_031894	**FTHL17**	ferritin heavy chain like 1	protein_coding	0	0	1	1	1.000
53942	NM_001243270,NM	**CNTN5**	contactin 5	protein_coding	0	0	1	1	1.000
53944	NM_001329605,NM	**CSNK1G1**	casein kinase 1 gamma 1	protein_coding	19	16	20	17	0.867
53947	NM_001318038,NM	**A4GALT**	alpha 1,4-galactosyltrans	protein_coding	0	0	1	1	1.000
53981	NM_001322270,NM	**CPSF2**	cleavage and polyadeny	protein_coding	31	27	32	28	0.871

54014	NM_001007246,NM	**BRWD1**	bromodomain and WD re	protein_coding	9	8	10	9	0.907
54019	gene-SLC6A6P1	**SLC6A6P1**	solute carrier family 6 me	pseudogene	0	0	1	1	1.000
54020	NM_001320537,NM	**SLC37A1**	solute carrier family 37 m	protein_coding	18	17	19	18	0.926
54021	gene-SRSF9P1	**SRSF9P1**	serine and arginine rich s	pseudogene	0	0	1	1	0.875
54027	gene-RPL31P1	**RPL31P1**	ribosomal protein L31 ps	pseudogene	0	0	1	1	1.000
54028	gene-RPL23P2	**RPL23P2**	ribosomal protein L23 ps	pseudogene	1	1	2	2	1.316
54029	gene-RPL23AP4	**RPL23AP4**	ribosomal protein L23a p	pseudogene	2	1	3	2	0.836
54031	gene-RIMKLBP1	**RIMKLBP1**	ribosomal modification pr	pseudogene	0	0	1	1	1.000
54033	NM_001320602,NM	**RBM11**	RNA binding motif protei	protein_coding	0	0	1	1	1.000
54035	gene-PSMD4P1	**PSMD4P1**	proteasome 26S subunit	pseudogene	0	0	1	1	1.000
54036	NR_144547	**PPP1R2P2**	protein phosphatase 1 re	transcribed_pseu	0	0	1	1	1.000
54038	gene-POLR2CP1	**POLR2CP1**	RNA polymerase II subur	pseudogene	0	0	1	1	1.000
54039	NM_001130141,NM	**PCBP3**	poly(rC) binding protein :	protein_coding	0	0	1	1	1.000
54043	gene-MYL6P1	**MYL6P1**	myosin light chain 6 pseu	pseudogene	0	0	1	1	0.793
54044	gene-KRT18P2	**KRT18P2**	keratin 18 pseudogene 2	pseudogene	0	0	1	1	1.000
54047	gene-HSPD1P7	**HSPD1P7**	heat shock protein family	pseudogene	0	0	1	1	1.000
54048	gene-HMGN1P2	**HMGN1P2**	high mobility group nucle	pseudogene	0	0	1	1	1.000
54049	gene-H2AZP1	**H2AZP1**	H2A.Z histone pseudoge	pseudogene	0	0	1	1	1.000
54050	gene-GAPDHP14	**GAPDHP14**	glyceraldehyde-3-phospl	pseudogene	0	0	1	1	1.000
54051	gene-FDPSP6	**FDPSP6**	farnesyl diphosphate syr	pseudogene	0	0	1	1	1.000
54052	gene-EIF4A1P1	**EIF4A1P1**	eukaryotic translation init	pseudogene	0	0	1	1	1.000
54053	gene-EIF3FP1	**EIF3FP1**	eukaryotic translation init	pseudogene	0	0	1	1	0.920
54054	gene-EEF1A1P1	**EEF1A1P1**	eukaryotic translation elo	pseudogene	0	0	1	1	0.996
54055	NR_026755	**CYP4F29P**	cytochrome P450 family	transcribed_pseu	0	0	1	1	1.000
54058	NM_001286462,NM	**C21orf58**	chromosome 21 open rea	protein_coding	14	12	15	13	0.879
54059	NM_001006114,NM	**YBEY**	ybeY metalloendoribonu	protein_coding	17	20	18	21	1.163
54064	NR_024351	**LINC00160**	long intergenic non-prote	lncRNA	0	0	1	1	1.000
54065	NM_001376899,NM	**SMIM11A**	small integral membrane	protein_coding	21	20	22	21	0.938
54067	NR_024622,NR_02	**C21orf62-AS1**	C21orf62 antisense RNA	lncRNA	1	1	2	2	1.002
54069	NM_018944,XM_01	**MIS18A**	MIS18 kinetochore protei	protein_coding	79	80	80	81	1.008
54072	NR_024027	**LINC00158**	long intergenic non-prote	lncRNA	0	0	1	1	1.000
54075	NR_024354	**CHODL-AS1**	CHODL antisense RNA 1	lncRNA	0	0	1	1	1.000
54082	NR_103707	**TSPEAR-AS1**	TSPEAR antisense RNA	lncRNA	0	0	1	1	1.000
54084	NM_001272037,NM	**TSPEAR**	thrombospondin type lan	protein_coding	0	0	1	1	1.000
54088	NR_024357	**LINC00113**	long intergenic non-prote	lncRNA	0	0	1	1	1.000
54089	NR_024028	**LINC00112**	long intergenic non-prote	lncRNA	0	0	1	1	1.000
54090	NR_024367	**LINC00111**	long intergenic non-prote	lncRNA	0	0	1	1	1.000
54093	NM_001007259,NM	**SETD4**	SET domain containing 4	protein_coding	8	8	9	9	1.016
54097	NM_058186,NM_20	**FAM3B**	FAM3 metabolism regula	protein_coding	0	0	1	1	1.000
54098	gene-C1QBPP1	**C1QBPP1**	complement C1q binding	pseudogene	0	0	1	1	1.000
54101	NM_020639	**RIPK4**	receptor interacting serin	protein_coding	0	0	1	1	1.000
54102	NM_001317009,NM	**CLIC6**	chloride intracellular cha	protein_coding	0	0	1	1	1.000
54103	NM_001350896,NM	**GSAP**	gamma-secretase activat	protein_coding	5	5	6	6	1.119
54106	NM_017442	**TLR9**	toll like receptor 9	protein_coding	0	0	1	1	0.935
54107	NM_001278255,NM	**POLE3**	DNA polymerase epsilon	protein_coding	230	241	231	242	1.048
54108	NM_017444,NR_02	**CHRAC1**	chromatin accessibility co	protein_coding	28	24	29	25	0.845
54112	NM_022049	**GPR88**	G protein-coupled recept	protein_coding	0	0	1	1	1.000
54143	NR_038400	**LINC00308**	long intergenic non-prote	lncRNA	0	0	1	1	1.000
54145	NM_017445	**H2BS1**	H2B.S histone 1	protein_coding	1	1	2	2	1.333
54147	NR_146594	**BACH1-IT3**	BACH1 intronic transcrip	lncRNA	0	0	1	1	1.000
54148	NM_017446,NM_08	**MRPL39**	mitochondrial ribosomal	protein_coding	88	78	89	79	0.888
54149	NM_001100420,NM	**C21orf91**	chromosome 21 open rea	protein_coding	53	47	54	48	0.893
54165	NM_001308101,NM	**DCUN1D1**	defective in cullin neddyl	protein_coding	25	19	26	20	0.781
54187	NM_018946,XM_01	**NANS**	N-acetylneuraminate syn	protein_coding	94	93	95	94	0.989
54205	NM_018947	**CYCS**	cytochrome c, somatic	protein_coding	183	172	184	173	0.938
54206	NM_018948,XM_00	**ERRFI1**	ERBB receptor feedback	protein_coding	0	0	1	1	1.000
54207	NM_021161,NM_13	**KCNK10**	potassium two pore dom	protein_coding	0	0	1	1	1.000
54209	NM_001271821,NM	**TREM2**	triggering receptor expre	protein_coding	0	0	1	1	1.000
54210	NM_001242589,NM	**TREM1**	triggering receptor expre	protein_coding	0	0	1	1	0.996
54212	NM_001287813,NM	**SNTG1**	syntrophin gamma 1	protein_coding	1	1	2	2	1.098
54221	NM_018968,XM_01	**SNTG2**	syntrophin gamma 2	protein_coding	0	0	1	1	1.000
54328	NM_018969,XM_01	**GPR173**	G protein-coupled recept	protein_coding	0	0	1	1	1.000
54329	NM_001146265,NM	**GPR85**	G protein-coupled recept	protein_coding	0	0	1	1	1.029
54331	NM_001243773,NM	**GNG2**	G protein subunit gamma	protein_coding	265	235	266	236	0.887
54332	NM_001040875,NM	**GDAP1**	ganglioside induced diffe	protein_coding	19	20	20	21	1.060
54344	NM_018973,NM_15	**DPM3**	dolichyl-phosphate mann	protein_coding	22	29	23	30	1.302
54345	NM_018419	**SOX18**	SRY-box transcription fa	protein_coding	0	0	1	1	1.000
54346	NM_001143947,NM	**UNC93A**	unc-93 homolog A	protein_coding	0	0	1	1	1.000
54360	NM_018659,XM_01	**CYTL1**	cytokine like 1	protein_coding	0	0	1	1	1.000
54361	NM_030761,XM_01	**WNT4**	Wnt family member 4	protein_coding	0	0	1	1	1.000
54363	NM_017545	**HAO1**	hydroxyacid oxidase 1	protein_coding	0	0	1	1	1.000
54386	NM_018975,NR_14	**TERF2IP**	TERF2 interacting protei	protein_coding	143	145	144	146	1.011
54407	NM_001307936,NM	**SLC38A2**	solute carrier family 38 m	protein_coding	39	26	40	27	0.671
54413	NM_001166660,NM	**NLGN3**	neuroligin 3	protein_coding	0	0	1	1	0.951
54414	NM_001199922,NM	**SIAE**	sialic acid acetylesterase	protein_coding	2	3	3	4	1.140
54429	NM_018980	**TAS2R5**	taste 2 receptor member	protein_coding	0	1	1	2	1.560
54431	NM_001271581,NM	**DNAJC10**	DnaJ heat shock protein	protein_coding	23	21	24	22	0.901
54432	NM_018982,NR_03	**YIPF1**	Yip1 domain family mem	protein_coding	23	22	24	23	0.955
54433	NM_018983,NM_03	**GAR1**	GAR1 ribonucleoprotein	protein_coding	148	145	149	146	0.982
54434	NM_001161330,NM	**SSH1**	slingshot protein phosph	protein_coding	29	28	30	29	0.957
54435	NR_002139	**HCG4**	HLA complex group 4	lncRNA	0	0	1	1	1.000
54436	NM_001318480,NM	**SH3TC1**	SH3 domain and tetratric	protein_coding	0	0	1	1	1.000
54437	NM_001031702,NM	**SEMA5B**	semaphorin 5B	protein_coding	0	0	1	1	1.000
54438	NM_001242628,NM	**GFOD1**	glucose-fructose oxidore	protein_coding	23	20	24	21	0.867
54439	NM_018989,XM_00	**RBM27**	RNA binding motif protei	protein_coding	53	46	54	47	0.878
54440	NM_018990,XM_00	**SASH3**	SAM and SH3 domain co	protein_coding	515	511	516	512	0.991
54441	NR_040583	**STAG3L1**	stromal antigen 3-like 1 (	transcribed_pseu	11	9	12	10	0.819
54442	NM_018992	**KCTD5**	potassium channel tetran	protein_coding	141	152	142	153	1.081
54443	NM_001284301,NM	**ANLN**	anillin actin binding prote	protein_coding	19	16	20	17	0.854
54453	NM_001242581,NM	**RIN2**	Ras and Rab interactor 2	protein_coding	0	0	1	1	1.000
54454	NM_001242338,NM	**ATAD2B**	ATPase family AAA doma	protein_coding	10	8	11	9	0.841
54455	NM_018994,XM_00	**FBXO42**	F-box protein 42	protein_coding	15	15	16	16	1.017
54456	NM_001164104,NM	**MOV10L1**	Mov10 like RISC comple:	protein_coding	0	0	1	1	1.000
54457	NM_001168474,NM	**TAF7L**	TATA-box binding protei	protein_coding	0	0	1	1	1.056
54458	NM_001005354,NM	**PRR13**	proline rich 13	protein_coding	332	344	333	345	1.039
54460	NM_018997,NM_03	**MRPS21**	mitochondrial ribosomal	protein_coding	93	106	94	107	1.140
54461	NM_018998,XM_00	**FBXW5**	F-box and WD repeat do	protein_coding	44	43	45	44	0.965
54462	NM_001284240,NM	**CCSER2**	coiled-coil serine rich pro	protein_coding	8	6	9	7	0.789
54463	NM_001034850,NM	**RETREG1**	reticulophagy regulator 1	protein_coding	0	0	1	1	1.000
54464	NM_001282857,NM	**XRN1**	5'-3' exoribonuclease 1	protein_coding	20	16	21	17	0.783
54465	NM_019002,XM_01	**ETAA1**	ETAA1 activator of ATR k	protein_coding	9	7	10	8	0.801
54466	NM_019003,XM_00	**SPIN2A**	spindlin family member 2	protein_coding	0	0	1	1	1.000
54467	NM_019004	**ANKIB1**	ankyrin repeat and IBR c	protein_coding	36	30	37	31	0.852
54468	NM_001370076,NM	**MIOS**	meiosis regulator for ooc	protein_coding	13	12	14	13	0.961
54469	NM_001242911,NM	**ZFAND6**	zinc finger AN1-type con	protein_coding	123	107	124	108	0.873
54470	NM_001009584,NM	**ARMCX6**	armadillo repeat containi	protein_coding	18	19	19	20	1.075
54471	NM_001304564,NM	**MIEF1**	mitochondrial elongation	protein_coding	56	52	57	53	0.927
54472	NM_001318512,NM	**TOLLIP**	toll interacting protein	protein_coding	26	26	27	27	1.022
54474	NM_019010	**KRT20**	keratin 20	protein_coding	0	0	1	1	1.000
54475	NM_001014445,NM	**NLE1**	notchless homolog 1	protein_coding	38	38	39	39	1.009
54476	NM_001377156,NM	**RNF216**	ring finger protein 216	protein_coding	19	18	20	19	0.939
54477	NM_001143821,NM	**PLEKHA5**	pleckstrin homology dom	protein_coding	0	0	1	1	1.000
54478	NM_001195228,NM	**PIMREG**	PICALM interacting mitot	protein_coding	0	0	1	1	1.000
54480	NM_001284295,NM	**CHPF2**	chondroitin polymerizing	protein_coding	13	13	14	14	0.965
54482	NM_019083,NR_13	**TRMT13**	tRNA methyltransferase	protein_coding	25	21	26	22	0.868
54487	NM_001190326,NM	**DGCR8**	DGCR8 microprocessor c	protein_coding	24	23	25	24	0.978
54490	NM_001207004,NM	**UGT2B28**	UDP glucuronosyltransfe	protein_coding	0	0	1	1	1.000
54491	NM_019018,XM_01	**OTULINL**	OTU deubiquitinase with	protein_coding	12	9	13	10	0.749
54492	NM_001142651,NM	**NEURL1B**	neuralized E3 ubiquitin p	protein_coding	0	0	1	1	1.000
54494	NM_001271562,NM	**C11orf71**	chromosome 11 open rea	protein_coding	10	12	11	13	1.191
54495	NM_001350512,NM	**TMX3**	thioredoxin related transi	protein_coding	56	47	57	48	0.851
54496	NM_001184824,NM	**PRMT7**	protein arginine methyltra	protein_coding	20	18	21	19	0.876
54497	NM_019024,XM_00	**HEATR5B**	HEAT repeat containing	protein_coding	11	10	12	11	0.925
54498	NM_001270691,NM	**SMOX**	spermine oxidase	protein_coding	0	0	1	1	1.000
54499	NM_001256164,NM	**TMCO1**	transmembrane and coile	protein_coding	135	134	136	135	0.992
54502	NM_001098634,NM	**RBM47**	RNA binding motif protei	protein_coding	0	0	1	1	0.945

54503	NM_001001483,NM	**ZDHHC13**	zinc finger DHHC-type pa	protein_coding	33	30	34	31	0.910
54504	NM_001348052,NM	**CPVL**	carboxypeptidase vitellog	protein_coding	0	0	1	1	1.000
54505	NM_001345964,NM	**DHX29**	DExH-box helicase 29	protein_coding	35	29	36	30	0.841
54507	NM_001288607,NM	**ADAMTSL4**	ADAMTS like 4	protein_coding	0	0	1	1	0.982
54508	NR_027706	**EPB41L4A-DT**	EPB41L4A divergent tran	lncRNA	0	0	1	1	1.000
54509	NM_019034	**RHOF**	ras homolog family mem	protein_coding	58	53	59	54	0.920
54510	NM_001300828,NM	**PCDH18**	protocadherin 18	protein_coding	0	0	1	1	1.000
54511	NM_001042406,NM	**HMGCLL1**	3-hydroxymethyl-3-methy	protein_coding	0	0	1	1	1.000
54512	NM_019037,XM_01	**EXOSC4**	exosome component 4	protein_coding	42	42	43	43	0.987
54514	NM_001142549,NM	**DDX4**	DEAD-box helicase 4	protein_coding	0	0	1	1	1.066
54516	NM_001114184,NM	**MTRF1L**	mitochondrial translation	protein_coding	21	19	22	20	0.891
54517	NM_001318163,NM	**PUS7**	pseudouridine synthase	protein_coding	46	38	47	39	0.827
54518	NM_019043,XM_00	**APBB1IP**	amyloid beta precursor p	protein_coding	5	6	6	7	1.151
54520	NM_019044,XM_00	**CCDC93**	coiled-coil domain contai	protein_coding	23	21	24	22	0.910
54521	NM_001184965,NM	**WDR44**	WD repeat domain 44	protein_coding	16	15	17	16	0.928
54522	NM_001009941,NM	**ANKRD16**	ankyrin repeat domain 16	protein_coding	4	4	5	5	0.899
54529	NM_001353497,NM	**ASNSD1**	asparagine synthetase d	protein_coding	94	81	95	82	0.864
54531	NM_001346105,NM	**MIER2**	MIER family member 2	protein_coding	0	0	1	1	1.000
54532	NM_001371395,NM	**USP53**	ubiquitin specific peptida	protein_coding	0	0	1	1	1.000
54534	NM_019051	**MRPL50**	mitochondrial ribosomal	protein_coding	35	32	36	33	0.933
54535	NM_001105563,NM	**CCHCR1**	coiled-coil alpha-helical r	protein_coding	28	30	29	31	1.097
54536	NM_001013848,NM	**EXOC6**	exocyst complex compor	protein_coding	13	12	14	13	0.886
54537	NM_001330112,NM	**SHLD2**	shieldin complex subunit	protein_coding	57	45	58	46	0.783
54538	NM_001301088,NM	**ROBO4**	roundabout guidance rec	protein_coding	0	0	1	1	1.000
54539	NM_001135998,NM	**NDUFB11**	NADH:ubiquinone oxidor	protein_coding	276	298	277	299	1.081
54540	NM_001190946,NM	**FAM193B**	family with sequence sim	protein_coding	14	15	15	16	1.071
54541	NM_019058	**DDIT4**	DNA damage inducible tr	protein_coding	337	329	338	330	0.975
54542	NM_001100588,NM	**RC3H2**	ring finger and CCCH-typ	protein_coding	28	24	29	25	0.854
54543	NM_019059,NR_16	**TOMM7**	translocase of outer mito	protein_coding	196	212	197	213	1.083
54544	NM_019060,XM_01	**CRCT1**	cysteine rich C-terminal 1	protein_coding	0	0	1	1	1.000
54545	NM_001040446,NM	**MTMR12**	myotubularin related pro	protein_coding	22	21	23	22	0.948
54546	NM_019062	**RNF186**	ring finger protein 186	protein_coding	0	0	1	1	1.000
54549	NM_001144952,XM	**SDK2**	sidekick cell adhesion m	protein_coding	0	0	1	1	1.068
54550	NM_001329748,NM	**NECAB2**	N-terminal EF-hand calc	protein_coding	0	0	1	1	1.000
54551	NM_019066	**MAGEL2**	MAGE family member L2	protein_coding	0	0	1	1	1.000
54552	NM_001184819,NM	**GNL3L**	G protein nucleolar 3 like	protein_coding	50	44	51	45	0.892
54553	NR_033797	**MIR4453HG**	MIR4453 host gene	lncRNA	7	6	8	7	0.966
54554	NM_019069	**WDR5B**	WD repeat domain 5B	protein_coding	6	6	7	7	0.978
54555	NM_019070,NR_03	**DDX49**	DEAD-box helicase 49	protein_coding	145	154	146	155	1.064
54556	NM_019071,NM_19	**ING3**	inhibitor of growth family	protein_coding	34	34	35	35	0.994
54557	NM_019072,XM_00	**SGTB**	small glutamine rich tetra	protein_coding	7	5	8	6	0.723
54558	NM_001286238,NM	**SPATA6**	spermatogenesis associa	protein_coding	0	0	1	1	1.000
54566	NM_018424,NM_01	**EPB41L4B**	erythrocyte membrane p	protein_coding	0	0	1	1	1.000
54567	NM_019074	**DLL4**	delta like canonical Notc	protein_coding	0	0	1	1	1.000
54568	gene-UGT2B29P	**UGT2B29P**	UDP glucuronosyltransfe	pseudogene	0	0	1	1	1.000
54569	gene-UGT2B27P	**UGT2B27P**	UDP glucuronosyltransfe	pseudogene	0	0	1	1	1.000
54570	gene-UGT2B26P	**UGT2B26P**	UDP glucuronosyltransfe	pseudogene	0	0	1	1	1.000
54571	gene-UGT2B25P	**UGT2B25P**	UDP glucuronosyltransfe	pseudogene	0	0	1	1	1.000
54572	gene-UGT2B24P	**UGT2B24P**	UDP glucuronosyltransfe	pseudogene	0	0	1	1	1.000
54575	NM_019075	**UGT1A10**	UDP glucuronosyltransfe	protein_coding	0	0	1	1	1.000
54576	NM_019076	**UGT1A8**	UDP glucuronosyltransfe	protein_coding	0	0	1	1	1.000
54577	NM_019077	**UGT1A7**	UDP glucuronosyltransfe	protein_coding	0	0	1	1	1.000
54578	NM_001072,NM_20	**UGT1A6**	UDP glucuronosyltransfe	protein_coding	0	0	1	1	1.000
54579	NM_019078	**UGT1A5**	UDP glucuronosyltransfe	protein_coding	0	0	1	1	1.000
54581	NR_003654,NR_00	**SCAND2P**	SCAN domain containing	transcribed_pseu	3	2	4	3	0.746
54583	NM_001377260,NM	**EGLN1**	egl-9 family hypoxia indu	protein_coding	16	15	17	16	0.954
54584	NM_053004	**GNB1L**	G protein subunit beta 1	protein_coding	4	5	5	6	1.109
54585	NM_001276378,NM	**LZTFL1**	leucine zipper transcripti	protein_coding	0	0	1	1	0.885
54586	NM_001161585,NM	**EQTN**	equatorin	protein_coding	0	0	1	1	1.000
54587	NM_001282582,NM	**MXRA8**	matrix remodeling associ	protein_coding	0	0	1	1	1.000
54596	NM_001164835,NM	**L1TD1**	LINE1 type transposase	protein_coding	0	0	1	1	1.000
54600	NM_021027	**UGT1A9**	UDP glucuronosyltransfe	protein_coding	0	0	1	1	1.000
54602	NM_001161407,NM	**NDFIP2**	Nedd4 family interacting	protein_coding	27	21	28	22	0.803
54606	NM_001257189,NM	**DDX56**	DEAD-box helicase 56	protein_coding	42	42	43	43	0.992
54617	NM_017553,NR_10	**INO80**	INO80 complex ATPase s	protein_coding	32	29	33	30	0.927
54619	NM_001134375,NM	**CCNJ**	cyclin J	protein_coding	9	9	10	10	0.967
54620	NM_001099784,NM	**FBXL19**	F-box and leucine rich re	protein_coding	27	27	28	28	0.988
54621	NM_019086,XM_00	**VSIG10**	V-set and immunoglobul	protein_coding	0	0	1	1	0.944
54622	NM_019087,XM_01	**ARL15**	ADP ribosylation factor li	protein_coding	6	5	7	6	0.883
54623	NM_001256826,NM	**PAF1**	PAF1 homolog, Paf1/RN	protein_coding	87	85	88	86	0.983
54625	NM_017554,XM_01	**PARP14**	poly(ADP-ribose) polyme	protein_coding	116	92	117	93	0.798
54626	NM_019089	**HES2**	hes family bHLH transcri	protein_coding	0	0	1	1	1.000
54627	NM_019090	**MAP10**	microtubule associated p	protein_coding	0	0	1	1	1.000
54629	NM_001040450,NM	**MINDY2**	MINDY lysine 48 deubiqu	protein_coding	5	5	6	6	0.913
54657	NM_007120	**UGT1A4**	UDP glucuronosyltransfe	protein_coding	0	0	1	1	1.000
54658	NM_000463	**UGT1A1**	UDP glucuronosyltransfe	protein_coding	0	0	1	1	1.000
54659	NM_019093	**UGT1A3**	UDP glucuronosyltransfe	protein_coding	0	0	1	1	1.000
54660	NR_001281	**PCDHB18P**	protocadherin beta 18 ps	transcribed_pseu	0	0	1	1	1.000
54661	NR_001280	**PCDHB17P**	protocadherin beta 17 ps	transcribed_pseu	0	0	1	1	1.001
54662	NM_001286772,NM	**TBC1D13**	TBC1 domain family mer	protein_coding	16	15	17	16	0.976
54663	NM_001307977,NM	**WDR74**	WD repeat domain 74	protein_coding	123	130	124	131	1.058
54664	NM_001134232,NM	**TMEM106B**	transmembrane protein 1	protein_coding	6	5	7	6	0.908
54665	NM_018364,NR_13	**RSBN1**	round spermatid basic pr	protein_coding	19	16	20	17	0.865
54674	NM_001099658,NM	**LRRN3**	leucine rich repeat neurc	protein_coding	4	3	5	4	0.801
54675	NM_001127458,NM	**CRLS1**	cardiolipin synthase 1	protein_coding	30	29	31	30	0.982
54676	NM_001286216,NM	**GTPBP2**	GTP binding protein 2	protein_coding	31	30	32	31	0.959
54677	NM_001143935,NM	**CROT**	carnitine O-octanoyltrans	protein_coding	14	12	15	13	0.843
54680	NM_001170670,NM	**ZNHIT6**	zinc finger HIT-type cont	protein_coding	47	45	48	46	0.957
54681	NM_177938,NM_17	**P4HTM**	prolyl 4-hydroxylase, tran	protein_coding	3	5	4	6	1.536
54682	NM_001363613,NM	**MANSC1**	MANSC domain containi	protein_coding	0	0	1	1	1.000
54700	NM_001301064,NM	**RRN3**	RRN3 homolog, RNA pol	protein_coding	117	101	118	102	0.860
54704	NM_001161779,NM	**PDP1**	pyruvate dehyrogenase p	protein_coding	13	13	14	14	0.955
54707	NM_018066	**GPN2**	GPN-loop GTPase 2	protein_coding	25	25	26	26	0.980
54708	NM_017824,XM_01	**MARCHF5**	membrane associated rir	protein_coding	22	18	23	19	0.815
54714	NM_019098,XM_01	**CNGB3**	cyclic nucleotide gated c	protein_coding	0	0	1	1	1.000
54715	NM_001142333,NM	**RBFOX1**	RNA binding fox-1 homo	protein_coding	0	0	1	1	1.000
54716	NM_020208,NM_02	**SLC6A20**	solute carrier family 6 me	protein_coding	0	0	1	1	1.000
54718	NR_027795	**BTN2A3P**	butyrophilin subfamily 2	transcribed_pseu	2	1	3	2	0.625
54726	NM_001102653,NM	**OTUD4**	OTU deubiquitinase 4	protein_coding	29	25	30	26	0.862
54729	NM_001290079	**NKX1-1**	NK1 homeobox 1	protein_coding	0	0	1	1	1.000
54732	NM_017510	**TMED9**	transmembrane p24 traff	protein_coding	169	179	170	180	1.061
54733	NM_017515	**SLC35F2**	solute carrier family 35 m	protein_coding	105	99	106	100	0.944
54734	NM_017516	**RAB39A**	RAB39A, member RAS o	protein_coding	0	0	1	1	1.000
54737	NM_017520,XM_01	**MPHOSPH8**	M-phase phosphoprotein	protein_coding	42	40	43	41	0.942
54738	NM_017521	**FEV**	FEV transcription factor,	protein_coding	0	0	1	1	1.000
54739	NM_001353134,NM	**XAF1**	XIAP associated factor 1	protein_coding	33	30	34	31	0.903
54741	NM_001198681,NM	**LEPROT**	leptin receptor overlappi	protein_coding	16	15	17	16	0.906
54742	NM_001160354,NM	**LY6K**	lymphocyte antigen 6 far	protein_coding	0	0	1	1	1.000
54749	NM_001242946,NM	**EPDR1**	ependymin related 1	protein_coding	0	0	1	1	1.000
54751	NM_001024215,NM	**FBLIM1**	filamin binding LIM prote	protein_coding	0	0	1	1	1.000
54752	NM_017559	**FNDC8**	fibronectin type III domai	protein_coding	0	0	1	1	1.072
54753	NM_001353546,NM	**ZNF853**	zinc finger protein 853	protein_coding	0	0	1	1	1.044
54754	NM_017561	**NUTM2F**	NUT family member 2F	protein_coding	0	0	1	1	1.000
54756	NM_001318864,NM	**IL17RD**	interleukin 17 receptor D	protein_coding	0	0	1	1	1.000
54757	NM_001243746,NM	**FAM20A**	FAM20A golgi associatec	protein_coding	0	0	1	1	0.991
54758	NM_001184854,NM	**KLHDC4**	kelch domain containing	protein_coding	57	52	58	53	0.906
54760	NM_017573,XM_00	**PCSK4**	proprotein convertase su	protein_coding	0	0	1	1	1.000
54762	NM_001172105,NM	**GRAMD1C**	GRAM domain containin	protein_coding	4	3	5	4	0.736
54763	NM_001317774,NM	**ROPN1**	rhophilin associated tail p	protein_coding	0	0	1	1	1.000
54764	NM_017580,XM_00	**ZRANB1**	zinc finger RANBP2-type	protein_coding	11	11	12	12	0.968
54765	NM_017583,XM_00	**TRIM44**	tripartite motif containing	protein_coding	28	26	29	27	0.922
54766	NM_001367974,NM	**BTG4**	BTG anti-proliferation fac	protein_coding	0	0	1	1	1.000
54768	NM_001198542,NM	**HYDIN**	HYDIN axonemal central	protein_coding	0	0	1	1	1.000
54769	NM_017594	**DIRAS2**	DIRAS family GTPase 2	protein_coding	0	0	1	1	1.000
54776	NM_001271618,NM	**PPP1R12C**	protein phosphatase 1 re	protein_coding	47	47	48	48	1.007
54777	NM_001200049	**CFAP46**	cilia and flagella associat	protein_coding	0	0	1	1	1.000

54778	NM_001270528,NM	**RNF111**	ring finger protein 111	protein_coding	17	19	18	20	1.058
54780	NM_001167865,NM	**NSMCE4A**	NSE4 homolog A, SMC5	protein_coding	64	57	65	58	0.901
54784	NM_017621,XM_00	**ALKBH4**	alkB homolog 4, lysine d	protein_coding	19	22	20	23	1.134
54785	NM_017622	**BORCS6**	BLOC-1 related complex	protein_coding	9	10	10	11	1.109
54788	NM_001002762,NM	**DNAJB12**	DnaJ heat shock protein	protein_coding	21	21	22	22	1.004
54790	NM_001127208,NM	**TET2**	tet methylcytosine dioxyg	protein_coding	0	0	1	1	0.943
54793	NM_017634,XR_00	**KCTD9**	potassium channel tetrar	protein_coding	36	27	37	28	0.750
54795	NM_001195227,NM	**TRPM4**	transient receptor potent	protein_coding	0	0	1	1	1.000
54796	NM_001317939,NM	**BNC2**	basonuclin 2	protein_coding	0	0	1	1	1.000
54797	NM_001127350,NM	**MED18**	mediator complex subun	protein_coding	23	24	24	25	1.037
54798	NM_001142552,NM	**DCHS2**	dachsous cadherin-relate	protein_coding	0	0	1	1	1.000
54799	NM_017643,XM_00	**MBTD1**	mbt domain containing 1	protein_coding	12	11	13	12	0.937
54800	NM_001349413,NM	**KLHL24**	kelch like family member	protein_coding	2	2	3	3	0.934
54801	NM_001270890,NM	**HAUS6**	HAUS augmin like compl	protein_coding	35	29	36	30	0.828
54802	NM_001312691,NM	**TRIT1**	tRNA isopentenyltransfer	protein_coding	20	19	21	20	0.957
54805	NM_017649,NM_19	**CNNM2**	cyclin and CBS domain c	protein_coding	2	2	3	3	0.957
54806	NM_001134830,NM	**AHI1**	Abelson helper integratio	protein_coding	7	5	8	6	0.761
54807	NM_001077426,NM	**ZNF586**	zinc finger protein 586	protein_coding	1	1	2	2	1.056
54808	NM_001353210,NM	**DYM**	dymeclin	protein_coding	11	8	12	9	0.739
54809	NM_001193307,NM	**SAMD9**	sterile alpha motif domai	protein_coding	65	47	66	48	0.724
54810	NM_001304725,NM	**GIPC2**	GIPC PDZ domain conta	protein_coding	0	0	1	1	0.896
54811	NM_001130031,NM	**ZNF562**	zinc finger protein 562	protein_coding	19	16	20	17	0.829
54812	NM_001002243,NM	**AFTPH**	aftiphilin	protein_coding	27	25	28	26	0.917
54813	NM_001308112,NM	**KLHL28**	kelch like family member	protein_coding	6	5	7	6	0.800
54814	NM_001163377,NM	**QPCTL**	glutaminyl-peptide cyclo	protein_coding	12	12	13	13	0.952
54815	NM_001300946,NM	**GATAD2A**	GATA zinc finger domain	protein_coding	174	168	175	169	0.966
54816	NM_001002843,NM	**ZNF280D**	zinc finger protein 280D	protein_coding	2	1	3	2	0.862
54819	NM_001300816,NM	**ZCCHC10**	zinc finger CCHC-type co	protein_coding	60	53	61	54	0.892
54820	NM_001143979,NM	**NDE1**	nudE neurodevelopment	protein_coding	39	34	40	35	0.880
54821	NM_001009954,NM	**ERCC6L**	ERCC excision repair 6 li	protein_coding	40	38	41	39	0.950
54822	NM_001301212,NM	**TRPM7**	transient receptor potent	protein_coding	78	62	79	63	0.793
54823	NM_001105518,NM	**SWT1**	SWT1 RNA endoribonuc	protein_coding	1	1	2	2	0.941
54825	NM_001171976,NM	**CDHR2**	cadherin related family m	protein_coding	0	0	1	1	1.000
54826	NM_001317954,NM	**GIN1**	gypsy retrotransposon in	protein_coding	6	6	7	7	1.067
54827	NM_001077639,NM	**NXPE4**	neurexophilin and PC-es	protein_coding	0	0	1	1	1.000
54828	NM_001099432,NM	**BCAS3**	BCAS3 microtubule asso	protein_coding	2	2	3	3	0.866
54829	NM_001193335,NM	**ASPN**	asporin	protein_coding	0	0	1	1	1.000
54830	NM_017681,NR_03	**NUP62CL**	nucleoporin 62 C-termina	protein_coding	0	0	1	1	1.000
54831	NM_017682	**BEST2**	bestrophin 2	protein_coding	0	0	1	1	1.000
54832	NM_001018088,NM	**VPS13C**	vacuolar protein sorting 1	protein_coding	28	22	29	23	0.802
54834	NM_001135589,NM	**GDAP2**	ganglioside induced diffe	protein_coding	7	6	8	7	0.864
54836	NM_001317943,NM	**BSPRY**	B-box and SPRY domain	protein_coding	0	0	1	1	1.000
54838	NM_001083913,NM	**WBP1L**	WW domain binding prot	protein_coding	35	38	36	39	1.075
54839	NM_001199017,NM	**LRRC49**	leucine rich repeat conta	protein_coding	0	0	1	1	1.000
54840	NM_001195248,NM	**APTX**	aprataxin	protein_coding	59	61	60	62	1.038
54841	NM_001159596,NM	**BIVM**	basic, immunoglobulin-lik	protein_coding	1	0	2	1	0.807
54842	NM_001375986,NM	**MFSD6**	major facilitator superfam	protein_coding	39	40	40	41	1.014
54843	NM_001162951,NM	**SYTL2**	synaptotagmin like 2	protein_coding	13	8	14	9	0.602
54845	NM_001034915,NM	**ESRP1**	epithelial splicing regulat	protein_coding	0	0	1	1	1.000
54847	NM_001308350,NM	**SIDT1**	SID1 transmembrane fan	protein_coding	19	20	20	21	1.028
54848	NM_001242729,NM	**ARHGEF38**	Rho guanine nucleotide	protein_coding	0	0	1	1	1.000
54849	NM_001242816,NM	**DEF8**	differentially expressed in	protein_coding	17	19	18	20	1.107
54850	NM_001316936,NM	**FBXL12**	F-box and leucine rich re	protein_coding	45	48	46	49	1.062
54851	NM_017704,XM_01	**ANKRD49**	ankyrin repeat domain 49	protein_coding	30	31	31	32	1.025
54852	NM_001104554,NM	**PAQR5**	progestin and adipoQ re	protein_coding	0	0	1	1	1.026
54853	NM_017706,XM_00	**WDR55**	WD repeat domain 55	protein_coding	49	48	50	49	0.980
54854	NM_017708,XM_00	**FAM83E**	family with sequence sim	protein_coding	0	0	1	1	0.903
54855	NM_017709	**TENT5C**	terminal nucleotidyltrans	protein_coding	34	35	35	36	1.031
54856	NM_001282856,NM	**GON4L**	gon-4 like	protein_coding	24	20	25	21	0.822
54857	NM_001171191,NM	**GDPD2**	glycerophosphodiester p	protein_coding	0	0	1	1	1.068
54858	NM_001300927,NM	**PGPEP1**	pyroglutamyl-peptidase I	protein_coding	22	20	23	21	0.910
54859	NM_001031703,NM	**ELP6**	elongator acetyltransfera	protein_coding	52	54	53	55	1.051
54860	NM_001164470,NM	**MS4A12**	membrane spanning 4-d	protein_coding	0	0	1	1	1.000
54861	NM_001100594,NM	**SNRK**	SNF related kinase	protein_coding	21	17	22	18	0.852
54862	NM_017721,XM_00	**CC2D1A**	coiled-coil and C2 domai	protein_coding	48	47	49	48	0.983
54863	NM_017723	**TOR4A**	torsin family 4 member A	protein_coding	0	1	1	2	1.370
54865	NM_015590,NM_18	**GPATCH4**	G-patch domain containi	protein_coding	251	264	252	265	1.050
54866	NM_001130143,NM	**PPP1R14D**	protein phosphatase 1 re	protein_coding	0	0	1	1	1.000
54867	NM_001083590,NM	**TMEM214**	transmembrane protein 2	protein_coding	44	46	45	47	1.033
54868	NM_001321264,NM	**TMEM104**	transmembrane protein 1	protein_coding	15	14	16	15	0.961
54869	NM_017729,NM_13	**EPS8L1**	EPS8 like 1	protein_coding	0	0	1	1	1.000
54870	NM_001320580,NM	**QRICH1**	glutamine rich 1	protein_coding	62	63	63	64	1.021
54872	NM_001127178,NM	**PIGG**	phosphatidylinositol glyc	protein_coding	23	24	24	25	1.035
54873	NM_017734	**PALMD**	palmdelphin	protein_coding	0	0	1	1	1.000
54874	NM_001024948,NM	**FNBP1L**	formin binding protein 1	protein_coding	0	0	1	1	1.080
54875	NM_001114395,NM	**CNTLN**	centlein	protein_coding	0	0	1	1	1.000
54876	NM_001345880,NM	**DCAF16**	DDB1 and CUL4 associa	protein_coding	34	30	35	31	0.894
54877	NM_017742,NR_12	**ZCCHC2**	zinc finger CCHC-type co	protein_coding	13	11	14	12	0.897
54878	NM_001320875,NM	**DPP8**	dipeptidyl peptidase 8	protein_coding	28	23	29	24	0.832
54879	NM_001308264,NM	**ST7L**	suppression of tumorigen	protein_coding	9	9	10	10	1.070
54880	NM_001123383,NM	**BCOR**	BCL6 corepressor	protein_coding	19	18	20	19	0.976
54881	NM_001161584,NM	**TEX10**	testis expressed 10	protein_coding	51	45	52	46	0.898
54882	NM_001197030,NM	**ANKHD1**	ankyrin repeat and KH d	protein_coding	48	42	49	43	0.871
54883	NM_017748,NR_07	**CWC25**	CWC25 spliceosome ass	protein_coding	34	35	35	36	1.051
54884	NM_017750	**RETSAT**	retinol saturase	protein_coding	16	15	17	16	0.955
54885	NM_017752,NM_19	**TBC1D8B**	TBC1 domain family mer	protein_coding	0	0	1	1	1.000
54886	NM_017753,NM_20	**PLPPR1**	phospholipid phosphatas	protein_coding	0	0	1	1	1.000
54887	NM_017754	**UHRF1BP1**	UHRF1 binding protein 1	protein_coding	11	10	12	11	0.934
54888	NM_001193455,NM	**NSUN2**	NOP2/Sun RNA methyltr	protein_coding	245	228	246	229	0.931
54890	NM_017758	**ALKBH5**	alkB homolog 5, RNA de	protein_coding	45	46	46	47	1.021
54891	NM_017759,XM_01	**INO80D**	INO80 complex subunit I	protein_coding	12	9	13	10	0.761
54892	NM_001281932,NM	**NCAPG2**	non-SMC condensin II co	protein_coding	80	63	81	64	0.793
54893	NM_017762,XM_00	**MTMR10**	myotubularin related pro	protein_coding	31	24	32	25	0.786
54894	NM_001305544,NM	**RNF43**	ring finger protein 43	protein_coding	2	3	3	4	1.106
54896	NM_001040125,NM	**SLC66A1**	solute carrier family 66 m	protein_coding	2	4	3	5	1.411
54897	NM_001079843,NM	**CASZ1**	castor zinc finger 1	protein_coding	3	3	4	4	0.946
54898	NM_017770,XM_01	**ELOVL2**	ELOVL fatty acid elongas	protein_coding	0	0	1	1	1.000
54899	NM_001289095,NM	**PXK**	PX domain containing se	protein_coding	57	46	58	47	0.811
54900	NM_001136190,NM	**LAX1**	lymphocyte transmembra	protein_coding	16	13	17	14	0.841
54901	NM_017774,XM_01	**CDKAL1**	CDK5 regulatory subunit	protein_coding	34	30	35	31	0.880
54902	NM_001271420,NM	**TTC19**	tetratricopeptide repeat c	protein_coding	23	22	24	23	0.976
54903	NM_001165927,NM	**MKS1**	MKS transition zone com	protein_coding	9	12	10	13	1.231
54904	NM_017778,NM_02	**NSD3**	nuclear receptor binding	protein_coding	49	40	50	41	0.820
54905	NM_017781,XM_01	**CYP2W1**	cytochrome P450 family	protein_coding	0	0	1	1	1.000
54906	NM_001321783,NM	**TASOR2**	transcription activation su	protein_coding	32	27	33	28	0.847
54908	NM_001329639,NM	**SPDL1**	spindle apparatus coiled	protein_coding	76	72	77	73	0.948
54910	NM_017789,XM_00	**SEMA4C**	semaphorin 4C	protein_coding	0	0	1	1	0.924
54913	NM_017793	**RPP25**	ribonuclease P and MRP	protein_coding	0	0	1	1	1.000
54914	NM_001375567,NM	**FOCAD**	focadhesin	protein_coding	11	10	12	11	0.962
54915	NM_017798,XM_02	**YTHDF1**	YTH N6-methyladenosin	protein_coding	99	102	100	103	1.026
54916	NM_017799,XM_00	**TMEM260**	transmembrane protein 2	protein_coding	7	7	8	8	1.041
54918	NM_017801	**CMTM6**	CKLF like MARVEL trans	protein_coding	125	114	126	115	0.914
54919	NM_017802,NR_07	**DNAAF5**	dynein axonemal assemb	protein_coding	28	27	29	28	0.963
54920	NM_001271762,NM	**DUS2**	dihydrouridine synthase	protein_coding	16	15	17	16	0.957
54921	NM_001039690,NM	**CHTF8**	chromosome transmissio	protein_coding	65	63	66	64	0.975
54922	NM_017805	**RASIP1**	Ras interacting protein 1	protein_coding	0	0	1	1	1.000
54923	NM_001305654,NM	**LIME1**	Lck interacting transmem	protein_coding	5	5	6	6	0.990
54925	NM_001284527,NM	**ZSCAN32**	zinc finger and SCAN do	protein_coding	29	26	30	27	0.912
54926	NM_017811,XM_00	**UBE2R2**	ubiquitin conjugating enz	protein_coding	29	28	30	29	0.973
54927	NM_001317177,NM	**CHCHD3**	coiled-coil-helix-coiled-co	protein_coding	249	245	250	246	0.983
54928	NM_017813,XR_92	**BPNT2**	3'(2'), 5'-bisphosphate nu	protein_coding	30	29	31	30	0.939
54929	NM_001256766,NM	**TMEM161A**	transmembrane protein 1	protein_coding	24	24	25	25	1.026
54930	NM_001166269,NM	**HAUS4**	HAUS augmin like compl	protein_coding	32	27	33	28	0.831
54931	NM_017819	**TRMT10C**	tRNA methyltransferase	protein_coding	54	46	55	47	0.845
54932	NM_001286823,NM	**EXD3**	exonuclease 3'-5' domair	protein_coding	1	1	2	2	0.924
54933	NM_001304746,NM	**RHBDL2**	rhomboid like 2	protein_coding	0	0	1	1	1.000

54934	NM_017822	**KANSL2**	KAT8 regulatory NSL cor	protein_coding	38	40	39	41	1.036
54935	NM_001319658,NM	**DUSP23**	dual specificity phosphat	protein_coding	8	11	9	12	1.291
54936	NM_017825,XM_01	**ADPRS**	ADP-ribosylserine hydrol	protein_coding	54	60	55	61	1.112
54937	NM_001282147,NM	**SOHLH2**	spermatogenesis and oo	protein_coding	0	0	1	1	1.000
54938	NM_001145901,NM	**SARS2**	seryl-tRNA synthetase 2,	protein_coding	26	30	27	31	1.124
54939	NM_001284377,NM	**COMMD4**	COMM domain containin	protein_coding	131	144	132	145	1.100
54940	NM_001079839,NM	**OCIAD1**	OCIA domain containing	protein_coding	142	132	143	133	0.932
54941	NM_017831,XM_01	**RNF125**	ring finger protein 125	protein_coding	10	9	11	10	0.962
54942	NM_017832,XM_01	**ABITRAM**	actin binding transcriptio	protein_coding	14	16	15	17	1.122
54943	NM_001040192,NM	**DNAJC28**	DnaJ heat shock protein	protein_coding	1	1	2	2	0.887
54944	NR_120386	**LINC01521**	long intergenic non-prote	lncRNA	3	3	4	4	0.926
54946	NM_001008485,NM	**SLC41A3**	solute carrier family 41 m	protein_coding	16	14	17	15	0.866
54947	NM_017839,XM_00	**LPCAT2**	lysophosphatidylcholine	protein_coding	0	0	1	1	1.000
54948	NM_017840	**MRPL16**	mitochondrial ribosomal	protein_coding	147	148	148	149	1.008
54949	NM_017841	**SDHAF2**	succinate dehydrogenas	protein_coding	96	97	97	98	1.006
54951	NM_001329668,NM	**COMMD8**	COMM domain containin	protein_coding	65	53	66	54	0.815
54952	NM_017846,NR_00	**TRNAU1AP**	tRNA selenocysteine 1 a	protein_coding	19	21	20	22	1.077
54953	NM_001164245,NM	**ODR4**	odr-4 GPCR localization	protein_coding	16	15	17	16	0.925
54954	NM_001300788,NM	**FAM120C**	family with sequence sim	protein_coding	3	3	4	4	0.965
54955	NM_001303030,NM	**C1orf109**	chromosome 1 open rea	protein_coding	61	55	62	56	0.910
54956	NM_001316943,NM	**PARP16**	poly(ADP-ribose) polyme	protein_coding	13	15	14	16	1.098
54957	NM_001142317,NM	**TXNL4B**	thioredoxin like 4B	protein_coding	35	33	36	34	0.934
54958	NM_017854	**TMEM160**	transmembrane protein 1	protein_coding	23	33	24	34	1.443
54959	NM_017855,XM_00	**ODAM**	odontogenic, ameloblast	protein_coding	0	0	1	1	1.000
54960	NM_001042479,NM	**GEMIN8**	gem nuclear organelle a	protein_coding	11	11	12	12	0.996
54961	NM_017857,XM_01	**SSH3**	slingshot protein phosph	protein_coding	3	3	4	4	1.014
54962	NM_001289986,NM	**TIPIN**	TIMELESS interacting pr	protein_coding	69	69	70	70	1.000
54963	NM_001193379,NM	**UCKL1**	uridine-cytidine kinase 1	protein_coding	40	37	41	38	0.936
54964	NM_017860	**C1orf56**	chromosome 1 open rea	protein_coding	0	0	1	1	1.169
54965	NM_001166304,NM	**PIGX**	phosphatidylinositol glyc	protein_coding	32	27	33	28	0.861
54967	NM_001031705,NM	**CT55**	cancer/testis antigen 55	protein_coding	0	0	1	1	1.000
54968	NM_001040613,NM	**TMEM70**	transmembrane protein 7	protein_coding	62	55	63	56	0.885
54969	NM_017867	**HPF1**	histone PARylation facto	protein_coding	22	19	23	20	0.851
54970	NM_001318533,NM	**TTC12**	tetratricopeptide repeat c	protein_coding	0	0	1	1	0.935
54971	NM_001173539,NM	**BANP**	BTG3 associated nuclea	protein_coding	16	16	17	17	0.972
54972	NM_017870,NM_17	**TMEM132A**	transmembrane protein 1	protein_coding	0	0	1	1	1.000
54973	NM_001256456,NM	**INTS11**	integrator complex subu	protein_coding	91	95	92	96	1.046
54974	NM_001317824,NM	**THG1L**	tRNA-histidine guanylylt	protein_coding	24	22	25	23	0.911
54976	NM_001039140,NM	**C20orf27**	chromosome 20 open rea	protein_coding	161	177	162	178	1.099
54977	NM_001354798,NM	**SLC25A38**	solute carrier family 25 m	protein_coding	40	43	41	44	1.067
54978	NM_017877	**SLC35F6**	solute carrier family 35 m	protein_coding	5	4	6	5	0.930
54979	NM_017878,XM_01	**PLAAT2**	phospholipase A and acy	protein_coding	6	6	7	7	0.986
54980	NM_001348758,NM	**C2orf42**	chromosome 2 open rea	protein_coding	10	9	11	10	0.938
54981	NM_001127603,NM	**NMRK1**	nicotinamide riboside kin	protein_coding	8	6	9	7	0.786
54982	NM_017882	**CLN6**	CLN6 transmembrane ER	protein_coding	107	113	108	114	1.057
54984	NM_001284356,NM	**PINX1**	PIN2 (TERF1) interacting	protein_coding	34	33	35	34	0.980
54985	NM_001002017,NM	**HCFC1R1**	host cell factor C1 regula	protein_coding	32	41	33	42	1.271
54986	NM_001322500,NM	**ULK4**	unc-51 like kinase 4	protein_coding	1	1	2	2	1.190
54987	NM_001304759,NM	**CZIB**	CXXC motif containing zi	protein_coding	45	54	46	55	1.189
54988	NM_001324371,NM	**ACSM5**	acyl-CoA synthetase me	protein_coding	0	0	1	1	1.000
54989	NM_014106,XM_01	**ZNF770**	zinc finger protein 770	protein_coding	62	53	63	54	0.845
54991	NM_001330306,NM	**C1orf159**	chromosome 1 open rea	protein_coding	13	12	14	13	0.927
54993	NM_001007072,NM	**ZSCAN2**	zinc finger and SCAN do	protein_coding	4	5	5	6	1.220
54994	NM_017896	**GID8**	GID complex subunit 8 h	protein_coding	75	72	76	73	0.963
54995	NM_001145391,NM	**OXSM**	3-oxoacyl-ACP synthase	protein_coding	13	13	14	14	1.037
54996	NM_001317338,NM	**MTARC2**	mitochondrial amidoxime	protein_coding	0	0	1	1	1.000
54997	NM_001168325,NM	**TESC**	tescalcin	protein_coding	171	186	172	187	1.085
54998	NM_001127229,NM	**AURKAIP1**	aurora kinase A interacti	protein_coding	238	282	239	283	1.183
55000	NR_002323,NR_11	**TUG1**	taurine up-regulated 1	lncRNA	51	43	52	44	0.852
55001	NM_001114108,NM	**TTC22**	tetratricopeptide repeat c	protein_coding	0	0	1	1	1.000
55002	NM_001349741,NM	**TMCO3**	transmembrane and coil	protein_coding	25	21	26	22	0.861
55003	NM_017906,XM_00	**PAK1IP1**	PAK1 interacting protein	protein_coding	77	63	78	64	0.824
55004	NM_017907	**LAMTOR1**	late endosomal/lysosom	protein_coding	143	165	144	166	1.155
55005	NM_001271937,NM	**RMND1**	required for meiotic nucl	protein_coding	29	28	30	29	0.961
55006	NM_017910,XM_00	**TRMT61B**	tRNA methyltransferase	protein_coding	16	14	17	15	0.915
55007	NM_001104595,NM	**FAM118A**	family with sequence sim	protein_coding	20	17	21	18	0.841
55008	NM_001165136,NM	**HERC6**	HECT and RLD domain c	protein_coding	4	4	5	5	0.991
55009	NM_017914	**FAM174C**	family with sequence sim	protein_coding	56	53	57	54	0.934
55010	NM_001319988,NM	**PARPBP**	PARP1 binding protein	protein_coding	23	19	24	20	0.843
55011	NM_017916,XM_02	**PIH1D1**	PIH1 domain containing	protein_coding	78	80	79	81	1.026
55012	NM_001305155,NM	**PPP2R3C**	protein phosphatase 2 re	protein_coding	27	23	28	24	0.869
55013	NM_017918,XM_00	**MCUB**	mitochondrial calcium ur	protein_coding	15	19	16	20	1.224
55014	NM_017919,XM_01	**STX17**	syntaxin 17	protein_coding	18	16	19	17	0.879
55015	NM_017922	**PRPF39**	pre-mRNA processing fa	protein_coding	28	28	29	29	0.984
55016	NM_001166373,NM	**MARCHF1**	membrane associated rir	protein_coding	0	0	1	1	1.000
55017	NM_017924,XM_01	**C14orf119**	chromosome 14 open rea	protein_coding	28	27	29	28	0.993
55018	NM_001358683,XM	**ANKRD40CL**	ANKRD40 C-terminal like	protein_coding	0	0	1	1	1.000
55020	NM_017931,XM_01	**TTC38**	tetratricopeptide repeat c	protein_coding	7	8	8	9	1.089
55022	NM_001100818,NM	**PID1**	phosphotyrosine interact	protein_coding	0	0	1	1	1.000
55023	NM_017934,XM_00	**PHIP**	pleckstrin homology dom	protein_coding	28	22	29	23	0.778
55024	NM_001083907,NM	**BANK1**	B cell scaffold protein wi	protein_coding	0	0	1	1	1.000
55025	NR_165282	**FLJ20712**	uncharacterized FLJ2071	lncRNA	0	0	1	1	1.000
55026	NM_001104544,NM	**TMEM255A**	transmembrane protein 2	protein_coding	0	0	1	1	0.964
55027	NM_001329729,NM	**HEATR3**	HEAT repeat containing	protein_coding	13	17	14	18	1.333
55028	NM_001100621,NM	**C17orf80**	chromosome 17 open rea	protein_coding	42	37	43	38	0.888
55030	NM_017943,NM_15	**FBXO34**	F-box protein 34	protein_coding	39	35	40	36	0.906
55031	NM_001282659,NM	**USP47**	ubiquitin specific peptida	protein_coding	23	18	24	19	0.824
55032	NM_001348905,NM	**SLC35A5**	solute carrier family 35 m	protein_coding	24	24	25	25	1.007
55033	NM_017946,NR_04	**FKBP14**	FKBP prolyl isomerase 1	protein_coding	0	0	1	1	1.040
55034	NM_017947	**MOCOS**	molybdenum cofactor su	protein_coding	0	0	1	1	1.033
55035	NM_001256394,NM	**NOL8**	nucleolar protein 8	protein_coding	48	43	49	44	0.913
55036	NM_001243342,NM	**CCDC40**	coiled-coil domain contai	protein_coding	0	0	1	1	1.000
55037	NM_017952	**PTCD3**	pentatricopeptide repeat	protein_coding	42	37	43	38	0.894
55038	NM_017955,NM_14	**CDCA4**	cell division cycle associ	protein_coding	78	73	79	74	0.938
55039	NM_017956	**TRMT12**	tRNA methyltransferase	protein_coding	6	6	7	7	0.994
55040	NM_017957	**EPN3**	epsin 3	protein_coding	0	0	1	1	1.000
55041	NM_001100623,NM	**PLEKHB2**	pleckstrin homology dom	protein_coding	143	134	144	135	0.937
55048	NM_017966,XM_00	**VPS37C**	VPS37C subunit of ESCI	protein_coding	2	2	3	3	0.919
55049	NM_001100418,NM	**REX1BD**	required for excision 1-B	protein_coding	52	62	53	63	1.186
55051	NM_017970,XM_01	**NRDE2**	NRDE-2, necessary for F	protein_coding	4	4	5	5	1.088
55052	NM_001318485,NM	**MRPL20**	mitochondrial ribosomal	protein_coding	292	304	293	305	1.039
55054	NM_001190266,NM	**ATG16L1**	autophagy related 16 like	protein_coding	36	34	37	35	0.929
55055	NM_001287821,NM	**ZWILCH**	zwilch kinetochore protei	protein_coding	81	72	82	73	0.887
55056	NR_026891	**GABPB1-IT1**	GABPB1 intronic transcri	lncRNA	3	4	4	5	1.128
55057	NM_001039775,XM	**CRYBG2**	crystallin beta-gamma do	protein_coding	0	0	1	1	1.001
55061	NM_001037175,NM	**SUSD4**	sushi domain containing	protein_coding	0	0	1	1	1.000
55062	NM_001320772,NM	**WIPI1**	WD repeat domain, phos	protein_coding	44	41	45	42	0.918
55063	NM_001258008,NM	**ZCWPW1**	zinc finger CW-type and	protein_coding	11	13	12	14	1.134
55064	NM_001039395,NM	**SPATA6L**	spermatogenesis associa	protein_coding	0	0	1	1	1.025
55065	NM_001104577,NM	**SLC52A1**	solute carrier family 52 m	protein_coding	0	0	1	1	1.000
55066	NM_001322117,NM	**PDPR**	pyruvate dehydrogenase	protein_coding	23	22	24	23	0.971
55068	NM_001127615,NM	**ENOX1**	ecto-NOX disulfide-thiol	protein_coding	0	0	1	1	1.000
55069	NM_017994,XM_00	**TMEM248**	transmembrane protein 2	protein_coding	77	70	78	71	0.913
55070	NM_001144074,NM	**DET1**	DET1 partner of COP1 E	protein_coding	2	2	3	3	1.015
55071	NM_017998	**C9orf40**	chromosome 9 open rea	protein_coding	21	25	22	26	1.162
55072	NM_001310332,NM	**RNF31**	ring finger protein 31	protein_coding	28	28	29	29	0.987
55073	NR_002940	**LRRC37A4P**	leucine rich repeat conta	transcribed_pseu	21	18	22	19	0.878
55074	NM_001198532,NM	**OXR1**	oxidation resistance 1	protein_coding	15	15	16	16	1.023
55075	NM_001008224,NM	**UACA**	uveal autoantigen with co	protein_coding	4	3	5	4	0.693
55076	NM_001363876,NM	**TMEM45A**	transmembrane protein 4	protein_coding	0	0	1	1	1.000
55079	NM_018008	**FEZF2**	FEZ family zinc finger 2	protein_coding	0	0	1	1	1.000
55080	NM_001351355,NM	**TAPBPL**	TAP binding protein like	protein_coding	21	21	22	22	1.036
55081	NM_018010	**IFT57**	intraflagellar transport 57	protein_coding	4	4	5	5	0.961
55082	NM_018011,XR_24	**ARGLU1**	arginine and glutamate r	protein_coding	106	97	107	98	0.919
55083	NM_018012	**KIF26B**	kinesin family member 2	protein_coding	0	0	1	1	1.000
55084	NM_018013,XM_00	**SOBP**	sine oculis binding prote	protein_coding	5	5	6	6	1.004

55086	NM_001184782,NM	**RADX**	RPA1 related single strai	protein_coding	13	11	14	12	0.894
55088	NM_001321829,NM	**CCDC186**	coiled-coil domain contai	protein_coding	15	13	16	14	0.864
55089	NM_001143824,NM	**SLC38A4**	solute carrier family 38 m	protein_coding	0	0	1	1	1.000
55090	NM_018019	**MED9**	mediator complex subun	protein_coding	14	16	15	17	1.117
55092	NM_001136216,NM	**TMEM51**	transmembrane protein 5	protein_coding	0	0	1	1	1.000
55093	NM_001283024,NM	**NTAQ1**	N-terminal glutamine am	protein_coding	25	23	26	24	0.941
55094	NM_018025,NR_13	**GPATCH1**	G-patch domain containi	protein_coding	15	15	16	16	1.017
55095	NM_001303614,NM	**SAMD4B**	sterile alpha motif domai	protein_coding	32	30	33	31	0.942
55096	NM_018029	**EBLN2**	endogenous Bornavirus	protein_coding	1	1	2	2	0.953
55100	NM_001345998,NM	**WDR70**	WD repeat domain 70	protein_coding	23	24	24	25	1.033
55101	NM_001167867,NM	**DMAC2**	distal membrane arm ass	protein_coding	91	93	92	94	1.025
55102	NM_018036	**ATG2B**	autophagy related 2B	protein_coding	6	5	7	6	0.865
55103	NM_001286247,NM	**RALGPS2**	Ral GEF with PH domair	protein_coding	1	0	2	1	0.880
55105	NM_001297754,NM	**GPATCH2**	G-patch domain containi	protein_coding	13	10	14	11	0.792
55106	NM_001289009,NM	**SLFN12**	schlafen family member	protein_coding	11	9	12	10	0.900
55107	NM_001378092,NM	**ANO1**	anoctamin 1	protein_coding	0	0	1	1	1.000
55108	NM_001143888,NM	**BSDC1**	BSD domain containing 1	protein_coding	28	31	29	32	1.081
55109	NM_018046	**AGGF1**	angiogenic factor with G	protein_coding	14	14	15	15	0.949
55110	NM_001300739,NM	**MAGOHB**	mago homolog B, exon ju	protein_coding	29	29	30	30	0.995
55111	NM_001300836,NM	**PLEKHJ1**	pleckstrin homology dom	protein_coding	144	147	145	148	1.017
55112	NM_001350914,NM	**DYNC2I1**	dynein 2 intermediate ch	protein_coding	6	7	7	8	1.067
55113	NM_018053,XM_01	**XKR8**	XK related 8	protein_coding	0	0	1	1	1.000
55114	NM_001006634,NM	**ARHGAP17**	Rho GTPase activating p	protein_coding	40	38	41	39	0.952
55116	NM_001319677,NM	**TMEM39B**	transmembrane protein 3	protein_coding	21	23	22	24	1.051
55117	NM_001146335,NM	**SLC6A15**	solute carrier family 6 me	protein_coding	0	0	1	1	0.957
55118	NM_001206528,NM	**CRTAC1**	cartilage acidic protein 1	protein_coding	0	0	1	1	1.000
55119	NM_001349757,NM	**PRPF38B**	pre-mRNA processing fa	protein_coding	66	59	67	60	0.894
55120	NM_001114636,NM	**FANCL**	FA complementation gro	protein_coding	26	23	27	24	0.895
55122	NM_018064	**AKIRIN2**	akirin 2	protein_coding	63	66	64	67	1.040
55124	NM_001135721,NM	**PIWIL2**	piwi like RNA-mediated g	protein_coding	0	0	1	1	1.000
55125	NM_032142,XM_00	**CEP192**	centrosomal protein 192	protein_coding	7	6	8	7	0.877
55127	NM_018072	**HEATR1**	HEAT repeat containing	protein_coding	45	35	46	36	0.787
55128	NM_001304496,NM	**TRIM68**	tripartite motif containing	protein_coding	13	13	14	14	1.009
55129	NM_001204831,NM	**ANO10**	anoctamin 10	protein_coding	1	1	2	2	0.807
55130	NM_001290020,NM	**ARMC4**	armadillo repeat containi	protein_coding	0	0	1	1	1.000
55131	NM_001166135,NM	**RBM28**	RNA binding motif protei	protein_coding	22	19	23	20	0.884
55132	NM_001278604,NM	**LARP1B**	La ribonucleoprotein 1B	protein_coding	9	9	10	10	0.946
55133	NM_018079,XM_01	**SRBD1**	S1 RNA binding domain	protein_coding	25	25	26	26	1.004
55135	NM_001143990,NM	**WRAP53**	WD repeat containing ar	protein_coding	26	23	27	24	0.887
55137	NM_001321825,NM	**FIGN**	fidgetin, microtubule sev	protein_coding	0	0	1	1	1.000
55138	NM_001319982,NM	**FAM90A1**	family with sequence sim	protein_coding	0	0	1	1	1.000
55139	NM_001042410,NM	**ANKZF1**	ankyrin repeat and zinc f	protein_coding	21	19	22	20	0.935
55140	NM_001284220,NM	**ELP3**	elongator acetyltransfera	protein_coding	24	22	25	23	0.916
55142	NM_001130447,NM	**HAUS2**	HAUS augmin like compl	protein_coding	50	45	51	46	0.896
55143	NM_001256875,NM	**CDCA8**	cell division cycle associ	protein_coding	220	224	221	225	1.019
55144	NM_001134479,NM	**LRRC8D**	leucine rich repeat conta	protein_coding	48	46	49	47	0.960
55145	NM_018105,NM_19	**THAP1**	THAP domain containing	protein_coding	19	18	20	19	0.948
55146	NM_001134387,NM	**ZDHHC4**	zinc finger DHHC-type p	protein_coding	34	35	35	36	1.047
55147	NM_001077351,NM	**RBM23**	RNA binding motif protei	protein_coding	48	47	49	48	0.981
55148	NM_175748,NR_03	**UBR7**	ubiquitin protein ligase E	protein_coding	71	68	72	69	0.956
55149	NM_018109	**MTPAP**	mitochondrial poly(A) pol	protein_coding	6	5	7	6	0.884
55150	NM_018111	**C19orf73**	chromosome 19 open rea	protein_coding	1	2	2	3	1.525
55151	NM_018112,XM_00	**TMEM38B**	transmembrane protein 3	protein_coding	24	22	25	23	0.907
55152	NM_001009996,NM	**DALRD3**	DALR anticodon binding	protein_coding	7	7	8	8	1.081
55153	NM_001288983,NM	**SDAD1**	SDA1 domain containing	protein_coding	71	64	72	65	0.910
55154	NM_001256532,NM	**MSTO1**	misato mitochondrial dist	protein_coding	34	40	35	41	1.171
55156	NM_001286702,NM	**ARMC1**	armadillo repeat containi	protein_coding	83	72	84	73	0.872
55157	NM_001365212,NM	**DARS2**	aspartyl-tRNA synthetase	protein_coding	27	24	28	25	0.916
55159	NM_001370534,NM	**RFWD3**	ring finger and WD repea	protein_coding	99	97	100	98	0.977
55160	NM_001011722,NM	**ARHGEF10L**	Rho guanine nucleotide	protein_coding	0	0	1	1	1.000
55161	NM_018126,XM_00	**TMEM33**	transmembrane protein 3	protein_coding	62	56	63	57	0.916
55163	NM_018129,XM_00	**PNPO**	pyridoxamine 5'-phospha	protein_coding	57	58	58	59	1.026
55164	NM_018130,XM_01	**SHQ1**	SHQ1, H/ACA ribonuclec	protein_coding	30	29	31	30	0.945
55165	NM_001127182,NM	**CEP55**	centrosomal protein 55	protein_coding	84	77	85	78	0.919
55166	NM_018132,XM_00	**CENPQ**	centromere protein Q	protein_coding	26	26	27	27	0.998
55167	NM_001145417,NM	**MSL2**	MSL complex subunit 2	protein_coding	35	32	36	33	0.918
55168	NM_001193343,NM	**MRPS18A**	mitochondrial ribosomal	protein_coding	96	104	97	105	1.082
55170	NM_018137	**PRMT6**	protein arginine methyltra	protein_coding	42	43	43	44	1.036
55171	NM_001134415,NM	**TBCCD1**	TBCC domain containing	protein_coding	26	24	27	25	0.928
55172	NM_001083908,NM	**DNAAF2**	dynein axonemal asseml	protein_coding	18	16	19	17	0.897
55173	NM_018141,XM_00	**MRPS10**	mitochondrial ribosomal	protein_coding	126	110	127	111	0.875
55174	NM_001353505,NM	**INTS10**	integrator complex subur	protein_coding	40	39	41	40	0.988
55175	NM_018143,XR_00	**KLHL11**	kelch like family member	protein_coding	11	10	12	11	0.901
55176	NM_001142627,NM	**SEC61A2**	SEC61 translocon subur	protein_coding	1	1	2	2	1.048
55177	NM_001304802,NM	**RMDN3**	regulator of microtubule	protein_coding	78	86	79	87	1.102
55178	NM_001317947,NM	**MRM3**	mitochondrial rRNA meth	protein_coding	26	30	27	31	1.149
55179	NM_001033030,NM	**FAIM**	Fas apoptotic inhibitory r	protein_coding	1	1	2	2	1.148
55180	NM_001040616,NM	**LINS1**	lines homolog 1	protein_coding	17	17	18	18	0.985
55181	NM_018149	**SMG8**	SMG8 nonsense mediate	protein_coding	30	29	31	30	0.969
55182	NM_001319956,NM	**RNF220**	ring finger protein 220	protein_coding	75	82	76	83	1.088
55183	NM_001177663,NM	**RIF1**	replication timing regulat	protein_coding	56	41	57	42	0.752
55184	NM_001099407,NM	**DZANK1**	double zinc ribbon and a	protein_coding	2	2	3	3	0.976
55186	NM_001104647,NM	**SLC25A36**	solute carrier family 25 m	protein_coding	46	39	47	40	0.846
55187	NM_015378,NM_01	**VPS13D**	vacuolar protein sorting 1	protein_coding	12	10	13	11	0.791
55188	NM_001330145,NM	**RIC8B**	RIC8 guanine nucleotide	protein_coding	0	0	1	1	1.000
55190	NM_018159	**NUDT11**	nudix hydrolase 11	protein_coding	0	0	1	1	1.000
55191	NM_018161	**NADSYN1**	NAD synthetase 1	protein_coding	30	29	31	30	0.954
55192	NM_018163,XM_01	**DNAJC17**	DnaJ heat shock protein	protein_coding	11	15	12	16	1.300
55193	NM_001350074,NM	**PBRM1**	polybromo 1	protein_coding	40	31	41	32	0.785
55194	NM_001304762,NM	**EVA1B**	eva-1 homolog B	protein_coding	0	0	1	1	1.000
55195	NM_001283056,NM	**CCDC198**	coiled-coil domain contai	protein_coding	0	0	1	1	1.000
55196	NM_018169,XM_00	**RESF1**	retroelement silencing fa	protein_coding	20	17	21	18	0.840
55197	NM_001303411,NM	**RPRD1A**	regulation of nuclear pre	protein_coding	41	35	42	36	0.858
55198	NM_001251904,NM	**APPL2**	adaptor protein, phosphc	protein_coding	7	7	8	8	1.046
55199	XR_004643250	**FAM86C1P**	family with sequence sim	misc_RNA	10	11	11	12	1.080
55200	NM_001144856,NM	**PLEKHG6**	pleckstrin homology and	protein_coding	0	0	1	1	1.000
55201	NM_001308363,NM	**MAP1S**	microtubule associated p	protein_coding	37	39	38	40	1.060
55203	NM_018176,XM_01	**LGI2**	leucine rich repeat LGI fa	protein_coding	0	0	1	1	0.985
55204	NM_018178,XM_00	**GOLPH3L**	golgi phosphoprotein 3 li	protein_coding	32	31	33	32	0.969
55205	NM_001318726,NM	**ZNF532**	zinc finger protein 532	protein_coding	0	0	1	1	1.000
55206	NM_001167856,NM	**SBNO1**	strawberry notch homolo	protein_coding	27	24	28	25	0.862
55207	NM_018184	**ARL8B**	ADP ribosylation factor li	protein_coding	63	60	64	61	0.962
55208	NM_001014283,XM	**DCUN1D2**	defective in cullin neddyl	protein_coding	9	8	10	9	0.854
55209	NM_001080517,NM	**SETD5**	SET domain containing 5	protein_coding	29	27	30	28	0.949
55210	NM_001170535,NM	**ATAD3A**	ATPase family AAA dom	protein_coding	175	181	176	182	1.036
55211	NM_001348928,NM	**DPPA4**	developmental pluripoter	protein_coding	0	0	1	1	1.000
55212	NM_018190,NM_17	**BBS7**	Bardet-Biedl syndrome 7	protein_coding	13	12	14	13	0.920
55213	NM_001352500,NM	**RCBTB1**	RCC1 and BTB domain c	protein_coding	26	24	27	25	0.900
55214	NM_001134418,NM	**P3H2**	prolyl 3-hydroxylase 2	protein_coding	0	0	1	1	1.000
55215	NM_001113378,NM	**FANCI**	FA complementation gro	protein_coding	153	130	154	131	0.848
55216	NM_001082969,NM	**NKAPD1**	NKAP domain containing	protein_coding	48	43	49	44	0.912
55217	NM_001184797,NM	**TMLHE**	trimethyllysine hydroxyla	protein_coding	0	0	1	1	1.000
55218	NM_001193360,NM	**EXD2**	exonuclease 3'-5' domair	protein_coding	0	0	1	1	1.000
55219	NM_001282564,NM	**MACO1**	macoilin 1	protein_coding	17	16	18	17	0.920
55220	NM_001271863,NM	**KLHDC8A**	kelch domain containing	protein_coding	0	0	1	1	1.000
55222	NM_001278211,NM	**LRRC20**	leucine rich repeat conta	protein_coding	11	11	12	12	1.028
55223	NM_001330483,NM	**TRIM62**	tripartite motif containing	protein_coding	9	10	10	11	1.021
55224	NM_001297760,NM	**ETNK2**	ethanolamine kinase 2	protein_coding	0	0	1	1	1.000
55225	NM_001366165,NM	**RAVER2**	ribonucleoprotein, PTB b	protein_coding	0	0	1	1	0.909
55226	NM_001144030,NM	**NAT10**	N-acetyltransferase 10	protein_coding	132	129	133	130	0.979
55227	NM_018214,XM_01	**LRRC1**	leucine rich repeat conta	protein_coding	6	5	7	6	0.939
55228	NM_001103149,NM	**PNMA8A**	PNMA family member 8A	protein_coding	0	0	1	1	1.000
55229	NM_018216,XM_01	**PANK4**	pantothenate kinase 4 (ir	protein_coding	20	21	21	22	1.078
55230	NM_001365479,NM	**USP40**	ubiquitin specific peptida	protein_coding	0	0	1	1	1.000
55231	NM_018219	**CCDC87**	coiled-coil domain contai	protein_coding	0	0	1	1	0.872
55233	NM_001317110,NM	**MOB1A**	MOB kinase activator 1A	protein_coding	149	132	150	133	0.883

55234	NM_018225,XM_00!	**SMU1**	SMU1 DNA replication re	protein_coding	33	32	34	33	0.964
55236	NM_018227,XM_01	**UBA6**	ubiquitin like modifier act	protein_coding	14	11	15	12	0.810
55237	NM_018228,XM_01	**VRTN**	vertebrae development a	protein_coding	0	0	1	1	1.000
55238	NM_001308384,NM	**SLC38A7**	solute carrier family 38 m	protein_coding	6	6	7	7	1.030
55239	NM_001324357,NM	**OGFOD1**	2-oxoglutarate and iron c	protein_coding	53	49	54	50	0.928
55240	NM_001008410,NM	**STEAP3**	STEAP3 metalloreductas	protein_coding	0	0	1	1	1.000
55243	NM_001286349,NM	**KIRREL1**	kirre like nephrin family a	protein_coding	0	0	1	1	1.000
55244	NM_018242	**SLC47A1**	solute carrier family 47 m	protein_coding	0	0	1	1	1.000
55245	NM_001184977,NM	**UQCC1**	ubiquinol-cytochrome c r	protein_coding	144	135	145	136	0.935
55246	NM_001304529,NM	**CCDC25**	coiled-coil domain contai	protein_coding	27	27	28	28	1.003
55247	NM_018248,XM_01	**NEIL3**	nei like DNA glycosylase	protein_coding	17	17	18	18	0.999
55248	NM_001198862,NM	**PACC1**	proton activated chloride	protein_coding	14	13	15	14	0.918
55249	NM_001198899,NM	**YY1AP1**	YY1 associated protein 1	protein_coding	72	72	73	73	1.000
55250	NM_001242875,NM	**ELP2**	elongator acetyltransfera	protein_coding	17	15	18	16	0.874
55251	NM_001104925,NM	**PCMTD2**	protein-L-isoaspartate (D	protein_coding	26	24	27	25	0.894
55252	NM_001369346,NM	**ASXL2**	ASXL transcriptional regu	protein_coding	11	9	12	10	0.831
55253	NM_018264,NR_13	**TYW1**	tRNA-yW synthesizing pi	protein_coding	31	35	32	36	1.108
55254	NM_018266,NR_07	**TMEM39A**	transmembrane protein 3	protein_coding	24	23	25	24	0.970
55255	NM_018268,XM_00!	**WDR41**	WD repeat domain 41	protein_coding	33	28	34	29	0.869
55256	NM_001306077,NM	**ADI1**	acireductone dioxygenas	protein_coding	26	29	27	30	1.100
55257	NM_018270,NR_13	**MRGBP**	MRG domain binding pro	protein_coding	55	55	56	56	1.004
55258	NM_001244676,NM	**THNSL2**	threonine synthase like 2	protein_coding	0	0	1	1	1.000
55259	NM_001082972,NM	**CFAP94**	cilia and flagella associa	protein_coding	0	0	1	1	1.101
55260	NM_001303538,NM	**TMEM143**	transmembrane protein 1	protein_coding	6	6	7	7	1.017
55262	NM_001303470,NM	**MAP11**	microtubule associated p	protein_coding	24	25	25	26	1.055
55266	NM_018279	**TMEM19**	transmembrane protein 1	protein_coding	12	9	13	10	0.757
55267	NR_165243	**PRR34**	PRR34 long non-coding	lncRNA	0	0	1	1	1.000
55268	NM_001198961,NM	**ECHDC2**	enoyl-CoA hydratase dor	protein_coding	0	0	1	1	1.000
55269	NM_001042414,NM	**PSPC1**	paraspeckle component	protein_coding	56	59	57	60	1.048
55270	NM_001304745,NM	**NUDT15**	nudix hydrolase 15	protein_coding	56	52	57	53	0.932
55272	NM_018285	**IMP3**	IMP U3 small nucleolar r	protein_coding	118	128	119	129	1.082
55273	NM_001099640,NM	**TMEM100**	transmembrane protein 1	protein_coding	0	0	1	1	1.000
55274	NM_018288,NM_13	**PHF10**	PHD finger protein 10	protein_coding	27	30	28	31	1.120
55275	NM_001128159,NM	**VPS53**	VPS53 subunit of GARP	protein_coding	5	5	6	6	1.013
55276	NM_018290,XM_01	**PGM2**	phosphoglucomutase 2	protein_coding	97	88	98	89	0.915
55277	NM_001113411,NM	**FGGY**	FGGY carbohydrate kina	protein_coding	2	2	3	3	0.976
55278	NM_018292,XM_01	**QRSL1**	glutaminyl-tRNA amidotr	protein_coding	23	22	24	23	0.962
55279	NM_001350134,NM	**ZNF654**	zinc finger protein 654	protein_coding	11	9	12	10	0.806
55280	NM_001303404,NM	**CWF19L1**	CWF19 like cell cycle co	protein_coding	34	33	35	34	0.986
55281	NM_018295	**TMEM140**	transmembrane protein 1	protein_coding	5	3	6	4	0.681
55282	NM_001161575,NM	**LRRC36**	leucine rich repeat conta	protein_coding	0	0	1	1	1.000
55283	NM_001253693,NM	**MCOLN3**	mucolipin 3	protein_coding	0	0	1	1	1.000
55284	NM_001001481,NM	**UBE2W**	ubiquitin conjugating enz	protein_coding	6	5	7	6	0.860
55285	NM_001171080,NM	**RBM41**	RNA binding motif protei	protein_coding	2	2	3	3	0.805
55286	NM_001104629,NM	**C4orf19**	chromosome 4 open rea	protein_coding	0	0	1	1	1.000
55287	NM_001284406,NM	**TMEM40**	transmembrane protein 4	protein_coding	0	0	1	1	1.000
55288	NM_001033566,NM	**RHOT1**	ras homolog family mem	protein_coding	39	34	40	35	0.865
55289	NM_001142807,NM	**ACOXL**	acyl-CoA oxidase like	protein_coding	8	6	9	7	0.737
55290	NM_018310	**BRF2**	BRF2 RNA polymerase I	protein_coding	24	25	25	26	1.033
55291	NM_001164160,NM	**PPP6R3**	protein phosphatase 6 re	protein_coding	83	71	84	72	0.855
55293	NM_001040697,NM	**UEVLD**	UEV and lactate/malate	protein_coding	20	16	21	17	0.787
55294	NM_001013415,NM	**FBXW7**	F-box and WD repeat do	protein_coding	14	13	15	14	0.883
55295	NM_001345981,NM	**KLHL26**	kelch like family member	protein_coding	8	9	9	10	1.148
55296	NM_001292054,NM	**TBC1D19**	TBC1 domain family mer	protein_coding	4	4	5	5	0.893
55297	NM_001330367,NM	**CCDC91**	coiled-coil domain contai	protein_coding	24	24	25	25	1.002
55298	NM_001300926,NM	**RNF121**	ring finger protein 121	protein_coding	33	36	34	37	1.100
55299	NM_018321	**BRIX1**	biogenesis of ribosomes	protein_coding	160	150	161	151	0.941
55300	NM_018323,NR_14	**PI4K2B**	phosphatidylinositol 4-kir	protein_coding	67	64	68	65	0.950
55301	NM_001039702,NM	**OLAH**	oleoyl-ACP hydrolase	protein_coding	0	0	1	1	0.913
55303	NM_001363532,NM	**GIMAP4**	GTPase, IMAP family me	protein_coding	226	227	227	228	1.003
55304	NM_001349945,NM	**SPTLC3**	serine palmitoyltransfera	protein_coding	0	0	1	1	1.000
55308	NM_001320522,NM	**DDX19A**	DEAD-box helicase 19A	protein_coding	60	62	61	63	1.027
55311	NM_001253792,NM	**ZNF444**	zinc finger protein 444	protein_coding	23	29	24	30	1.218
55312	NM_018339	**RFK**	riboflavin kinase	protein_coding	37	29	38	30	0.776
55313	NM_001099455,NM	**CPPED1**	calcineurin like phospho	protein_coding	0	0	1	1	1.000
55314	NM_018342,XM_00!	**TMEM144**	transmembrane protein 1	protein_coding	0	0	1	1	1.000
55315	NM_001174098,NM	**SLC29A3**	solute carrier family 29 m	protein_coding	5	5	6	6	1.122
55316	NM_018346,NR_13	**RSAD1**	radical S-adenosyl methi	protein_coding	39	37	40	38	0.944
55317	NM_001204446,NM	**AP5S1**	adaptor related protein c	protein_coding	9	9	10	10	1.026
55319	NM_018352	**TMA16**	translation machinery as	protein_coding	59	54	60	55	0.908
55320	NM_018353,XM_00!	**MIS18BP1**	MIS18 binding protein 1	protein_coding	11	9	12	10	0.815
55321	NM_001304748,NM	**TMEM74B**	transmembrane protein 7	protein_coding	0	0	1	1	1.000
55322	NM_018356,NR_13	**C5orf22**	chromosome 5 open rea	protein_coding	40	36	41	37	0.902
55323	NM_001286679,NM	**LARP6**	La ribonucleoprotein 6, t	protein_coding	0	0	1	1	1.000
55324	NM_001351298,NM	**ABCF3**	ATP binding cassette sul	protein_coding	47	49	48	50	1.045
55325	NM_018359,NR_02	**UFSP2**	UFM1 specific peptidase	protein_coding	19	17	20	18	0.897
55326	NM_018361,XM_01	**AGPAT5**	1-acylglycerol-3-phospha	protein_coding	29	27	30	28	0.934
55327	NM_018362	**LIN7C**	lin-7 homolog C, crumbs	protein_coding	26	23	27	24	0.891
55328	NM_001031709,NM	**RNLS**	renalase, FAD depender	protein_coding	5	5	6	6	0.943
55329	NM_018365	**MNS1**	meiosis specific nuclear	protein_coding	0	0	1	1	1.000
55330	NM_018366	**BLOC1S4**	biogenesis of lysosomal	protein_coding	21	21	22	22	0.995
55331	NM_001300953,NM	**ACER3**	alkaline ceramidase 3	protein_coding	14	12	15	13	0.886
55332	NM_018370,XM_00!	**DRAM1**	DNA damage regulated a	protein_coding	3	3	4	4	0.941
55333	NM_018373	**SYNJ2BP**	synaptojanin 2 binding p	protein_coding	7	7	8	8	0.984
55334	NM_001252148,NM	**SLC39A9**	solute carrier family 39 m	protein_coding	37	36	38	37	0.969
55335	NM_018376,NR_13	**NIPSNAP3B**	nipsnap homolog 3B	protein_coding	0	0	1	1	0.957
55336	NM_018378	**FBXL8**	F-box and leucine rich re	protein_coding	2	2	3	3	1.033
55337	NM_001308277,NM	**SHFL**	shiftless antiviral inhibito	protein_coding	99	104	100	105	1.046
55338	NR_130914	**LINC01949**	long intergenic non-prote	lncRNA	0	0	1	1	0.969
55339	NM_001006622,NM	**WDR33**	WD repeat domain 33	protein_coding	44	44	45	45	0.996
55340	NM_018384	**GIMAP5**	GTPase, IMAP family me	protein_coding	210	238	211	239	1.130
55341	NM_018385,XM_01	**LSG1**	large 60S subunit nuclea	protein_coding	77	77	78	78	1.001
55342	NM_001171137,NM	**STRBP**	spermatid perinuclear RI	protein_coding	35	23	36	24	0.674
55343	NM_001145265,NM	**SLC35C1**	solute carrier family 35 m	protein_coding	24	25	25	26	1.041
55344	NM_001370370,NM	**PLCXD1**	phosphatidylinositol spe	protein_coding	8	8	9	9	0.983
55345	NM_001350397,NM	**ZGRF1**	zinc finger GRF-type cor	protein_coding	11	9	12	10	0.841
55346	NM_001145541,NM	**TCP11L1**	t-complex 11 like 1	protein_coding	13	12	14	13	0.961
55347	NM_001272069,NM	**ABHD10**	abhydrolase domain con	protein_coding	46	48	47	49	1.046
55349	NM_018397,XM_00	**CHDH**	choline dehydrogenase	protein_coding	0	0	1	1	1.010
55350	NM_001291702,NM	**VNN3**	vanin 3	protein_coding	0	0	1	1	1.164
55351	NM_001306082,NM	**STK32B**	serine/threonine kinase 3	protein_coding	1	1	2	2	1.085
55352	NM_001330176,NM	**COPRS**	coordinator of PRMT5 an	protein_coding	40	42	41	43	1.033
55353	NM_018407	**LAPTM4B**	lysosomal protein transm	protein_coding	0	0	1	1	1.000
55355	NM_001282962,NM	**HJURP**	Holliday junction recogni	protein_coding	104	90	105	91	0.864
55356	NM_018420,XM_00!	**SLC22A15**	solute carrier family 22 m	protein_coding	0	0	1	1	1.000
55357	NM_001267571,NM	**TBC1D2**	TBC1 domain family mer	protein_coding	2	2	3	3	1.129
55359	NM_018423,XM_00!	**STYK1**	serine/threonine/tyrosine	protein_coding	4	3	5	4	0.780
55361	NM_018425	**PI4K2A**	phosphatidylinositol 4-kir	protein_coding	14	14	15	15	1.014
55362	NM_001318792,NM	**TMEM63B**	transmembrane protein 6	protein_coding	8	8	9	9	0.947
55363	NM_018437,NM_19	**HEMGN**	hemogen	protein_coding	1	2	2	3	1.346
55364	NM_018439,XM_01	**IMPACT**	impact RWD domain pro	protein_coding	26	23	27	24	0.884
55365	NM_018487,XM_01	**TMEM176A**	transmembrane protein 1	protein_coding	0	0	1	1	1.000
55366	NM_001346432,NM	**LGR4**	leucine rich repeat conta	protein_coding	0	0	1	1	1.000
55367	NM_145886,NM_14	**PIDD1**	p53-induced death doma	protein_coding	12	12	13	13	0.989
55370	NR_003505	**PPP4R1L**	protein phosphatase 4 re	transcribed_pseu	0	0	1	1	1.184
55374	NM_001300980,NM	**TMCO6**	transmembrane and coile	protein_coding	13	15	14	16	1.105
55379	NM_018509	**LRRC59**	leucine rich repeat conta	protein_coding	219	214	220	215	0.978
55380	gene-EPS15P1	**EPS15P1**	epidermal growth factor i	pseudogene	0	0	1	1	1.000
55384	NR_002766,NR_00	**MEG3**	maternally expressed 3	lncRNA	0	0	1	1	1.000
55388	NM_018518,NM_18	**MCM10**	minichromosome mainte	protein_coding	54	50	55	51	0.926
55421	NM_001114118,XM	**NCBP3**	nuclear cap binding subi	protein_coding	12	17	13	18	1.395
55422	NM_001079906,NM	**ZNF331**	zinc finger protein 331	protein_coding	0	0	1	1	1.012
55423	NM_001039508,NM	**SIRPG**	signal regulatory protein	protein_coding	155	160	156	161	1.032
55425	NM_001316951,NM	**GPALPP1**	GPALPP motifs containi	protein_coding	29	29	30	30	1.000
55432	NM_001276320,NM	**YOD1**	YOD1 deubiquitinase	protein_coding	22	17	23	18	0.766
55435	NM_001128426,NM	**AP1AR**	adaptor related protein c	protein_coding	14	13	15	14	0.965

55437	NM_001206864,NM	**STRADB**	STE20 related adaptor b	protein_coding	3	3	4	4	1.106
55449	NR_023921,NR_02	**DHRS4-AS1**	DHRS4 antisense RNA 1	lncRNA	14	15	15	16	1.050
55450	NM_018584	**CAMK2N1**	calcium/calmodulin depe	protein_coding	0	0	1	1	1.000
55451	NR_146595	**LINC00216**	long intergenic non-prote	lncRNA	0	0	1	1	1.000
55454	NM_001319654,NM	**CSGALNACT2**	chondroitin sulfate N-ace	protein_coding	48	40	49	41	0.821
55466	NM_001130182,NM	**DNAJA4**	DnaJ heat shock protein	protein_coding	0	0	1	1	1.000
55471	NM_001083946,NM	**NDUFAF7**	NADH:ubiquinone oxidor	protein_coding	23	16	24	17	0.725
55472	NR_027259	**RBM12B-AS1**	RBM12B antisense RNA	lncRNA	0	1	1	2	1.564
55486	NM_001037639,NM	**PARL**	presenilin associated rhc	protein_coding	82	84	83	85	1.019
55500	NM_001039481,NM	**ETNK1**	ethanolamine kinase 1	protein_coding	25	20	26	21	0.802
55501	NM_001243794,NM	**CHST12**	carbohydrate sulfotransfe	protein_coding	13	19	14	20	1.448
55502	NM_001142853,NM	**HES6**	hes family bHLH transcri	protein_coding	0	0	1	1	1.000
55503	NM_018646	**TRPV6**	transient receptor potent	protein_coding	0	0	1	1	1.000
55504	NM_001204458,NM	**TNFRSF19**	TNF receptor superfamily	protein_coding	0	0	1	1	1.000
55505	NM_018648	**NOP10**	NOP10 ribonucleoprotein	protein_coding	480	484	481	485	1.009
55506	NM_018649	**MACROH2A2**	macroH2A.2 histone	protein_coding	0	0	1	1	1.000
55507	NM_018654,XM_00!	**GPRC5D**	G protein-coupled recept	protein_coding	0	0	1	1	1.000
55508	NM_001354997,NM	**SLC35E3**	solute carrier family 35 m	protein_coding	2	2	3	3	1.017
55509	NM_018664,XM_01	**BATF3**	basic leucine zipper ATF	protein_coding	11	10	12	11	0.911
55510	NM_018665	**DDX43**	DEAD-box helicase 43	protein_coding	5	3	6	4	0.730
55511	NM_001381902,NM	**SAGE1**	sarcoma antigen 1	protein_coding	0	0	1	1	1.000
55512	NM_018667,XM_00!	**SMPD3**	sphingomyelin phosphod	protein_coding	0	0	1	1	1.000
55515	NM_018674,NM_18	**ASIC4**	acid sensing ion channel	protein_coding	0	0	1	1	1.000
55520	NM_018696	**ELAC1**	elaC ribonuclease Z 1	protein_coding	10	11	11	12	1.106
55521	NM_001017397,NM	**TRIM36**	tripartite motif containing	protein_coding	0	0	1	1	1.000
55526	NM_018706	**DHTKD1**	dehydrogenase E1 and t	protein_coding	1	1	2	2	0.902
55527	NM_018708	**FEM1A**	fem-1 homolog A	protein_coding	21	21	22	22	0.975
55529	NM_018710,XM_01	**PIP4P2**	phosphatidylinositol-4,5-	protein_coding	2	3	3	4	1.245
55530	NM_018711	**SVOP**	SV2 related protein	protein_coding	0	0	1	1	1.000
55531	NM_001130037,NM	**ELMOD1**	ELMO domain containing	protein_coding	0	0	1	1	1.000
55532	NM_001376929,NM	**SLC30A10**	solute carrier family 30 m	protein_coding	0	0	1	1	1.000
55534	NM_018717	**MAML3**	mastermind like transcrip	protein_coding	7	7	8	8	0.960
55536	NM_001127370,NM	**CDCA7L**	cell division cycle associ	protein_coding	1	1	2	2	1.025
55539	NR_024627	**KCNQ1DN**	KCNQ1 downstream neig	lncRNA	0	0	1	1	1.000
55540	NM_018725,XM_00!	**IL17RB**	interleukin 17 receptor B	protein_coding	0	0	1	1	1.000
55544	NM_001291780,NM	**RBM38**	RNA binding motif protei	protein_coding	359	339	360	340	0.944
55545	NR_002307	**MSX2P1**	msh homeobox 2 pseudo	transcribed_pseu	0	0	1	1	1.000
55552	NM_001080493,NM	**ZNF823**	zinc finger protein 823	protein_coding	12	14	13	15	1.145
55553	NM_001145811,NM	**SOX6**	SRY-box transcription fa	protein_coding	0	0	1	1	1.003
55554	NM_001277081,NM	**KLK15**	kallikrein related peptida	protein_coding	0	0	1	1	1.000
55556	NM_001318760,NM	**ENOSF1**	enolase superfamily mer	protein_coding	6	8	7	9	1.207
55558	NM_017514,XM_00!	**PLXNA3**	plexin A3	protein_coding	2	3	3	4	1.176
55559	NM_017518,NR_07	**HAUS7**	HAUS augmin like compl	protein_coding	49	50	50	51	1.031
55561	NM_017525,XM_01	**CDC42BPG**	CDC42 binding protein k	protein_coding	0	0	1	1	0.933
55565	NM_001201552,NM	**ZNF821**	zinc finger protein 821	protein_coding	1	1	2	2	0.870
55567	NM_001347886,NM	**DNAH3**	dynein axonemal heavy c	protein_coding	0	0	1	1	1.000
55568	NM_198321	**GALNT10**	polypeptide N-acetylgala	protein_coding	39	31	40	32	0.811
55571	NM_017546	**CNOT11**	CCR4-NOT transcription	protein_coding	63	64	64	65	1.013
55572	NM_017547,NR_03	**FOXRED1**	FAD dependent oxidored	protein_coding	70	75	71	76	1.072
55573	NM_001134422,NM	**CDV3**	CDV3 homolog	protein_coding	268	253	269	254	0.943
55576	NM_017564,XM_01	**STAB2**	stabilin 2	protein_coding	0	0	1	1	1.000
55577	NM_001330425,NM	**NAGK**	N-acetylglucosamine kin	protein_coding	28	32	29	33	1.170
55578	NM_001014286,NM	**SUPT20H**	SPT20 homolog, SAGA c	protein_coding	20	17	21	18	0.870
55582	NM_001271927,NM	**KIF27**	kinesin family member 27	protein_coding	0	0	1	1	1.041
55584	NM_017581	**CHRNA9**	cholinergic receptor nico	protein_coding	0	0	1	1	1.088
55585	NM_017582	**UBE2Q1**	ubiquitin conjugating enz	protein_coding	82	81	83	82	0.983
55586	NM_017584,XM_00!	**MIOX**	myo-inositol oxygenase	protein_coding	0	0	1	1	1.000
55588	NM_001317770,NM	**MED29**	mediator complex subun	protein_coding	51	52	52	53	1.011
55589	NM_017593,NM_19	**BMP2K**	BMP2 inducible kinase	protein_coding	7	6	8	7	0.884
55591	NM_001352088,NM	**VEZT**	vezatin, adherens junctic	protein_coding	21	21	22	22	1.022
55592	NR_024261,NR_03	**GOLGA2P5**	GOLGA2 pseudogene 5	transcribed_pseu	5	4	6	5	0.905
55593	NM_001136157,NM	**OTUD5**	OTU deubiquitinase 5	protein_coding	40	34	41	35	0.845
55596	NM_001350935,NM	**ZCCHC8**	zinc finger CCHC-type cc	protein_coding	17	16	18	17	0.925
55599	NM_017619	**RNPC3**	RNA binding region (RNI	protein_coding	10	8	11	9	0.746
55600	NM_017625	**ITLN1**	intelectin 1	protein_coding	0	0	1	1	0.833
55601	NM_017631,XM_01	**DDX60**	DExD/H-box helicase 60	protein_coding	19	15	20	16	0.793
55602	NM_001317343,NM	**CDKN2AIP**	CDKN2A interacting prot	protein_coding	30	30	31	31	1.012
55603	NM_017633	**TENT5A**	terminal nucleotidyltrans	protein_coding	0	0	1	1	1.000
55604	NM_001173977,NM	**CARMIL1**	capping protein regulato	protein_coding	0	0	1	1	1.018
55605	NM_001173463,NM	**KIF21A**	kinesin family member 2	protein_coding	21	18	22	19	0.847
55607	NM_001166160,NM	**PPP1R9A**	protein phosphatase 1 re	protein_coding	6	5	7	6	0.817
55608	NM_001286721,NM	**ANKRD10**	ankyrin repeat domain 1	protein_coding	60	53	61	54	0.884
55609	NM_017666,XM_00	**ZNF280C**	zinc finger protein 280C	protein_coding	14	12	15	13	0.854
55610	NM_001257998,NM	**VPS50**	VPS50 subunit of EARP/	protein_coding	14	13	15	14	0.903
55611	NM_017670,NR_00	**OTUB1**	OTU deubiquitinase, ubi	protein_coding	105	111	106	112	1.060
55612	NM_017671,XM_02	**FERMT1**	fermitin family member 1	protein_coding	0	0	1	1	1.000
55613	NM_017677	**MTMR8**	myotubularin related pro	protein_coding	0	1	1	2	1.120
55614	NM_001199865,NM	**KIF16B**	kinesin family member 1	protein_coding	0	0	1	1	1.000
55615	NM_001017528,NM	**PRR5**	proline rich 5	protein_coding	0	0	1	1	1.000
55616	NM_001143778,NM	**ASAP3**	ArfGAP with SH3 domair	protein_coding	0	0	1	1	1.000
55617	NM_001323602,NM	**TASP1**	taspase 1	protein_coding	5	6	6	7	1.091
55619	NM_001290263,NM	**DOCK10**	dedicator of cytokinesis 1	protein_coding	84	73	85	74	0.880
55620	NM_001013841,NM	**STAP2**	signal transducing adapt	protein_coding	0	0	1	1	1.064
55621	NM_001136035,NM	**TRMT1**	tRNA methyltransferase	protein_coding	66	74	67	75	1.116
55622	NM_001193509,NM	**TTC27**	tetratricopeptide repeat c	protein_coding	14	12	15	13	0.864
55623	NM_001304550,NM	**THUMPD1**	THUMP domain containi	protein_coding	50	42	51	43	0.855
55624	NM_001243766,NM	**POMGNT1**	protein O-linked mannos	protein_coding	42	44	43	45	1.053
55625	NM_001145548,NM	**ZDHHC7**	zinc finger DHHC-type p	protein_coding	42	34	43	35	0.827
55626	NM_001267782,NM	**AMBRA1**	autophagy and beclin 1 r	protein_coding	26	25	27	26	0.955
55627	NM_001171083,NM	**SMPD4**	sphingomyelin phosphod	protein_coding	106	109	107	110	1.027
55628	NM_001146189,NM	**ZNF407**	zinc finger protein 407	protein_coding	5	5	6	6	0.880
55629	NM_017761,XM_01	**PNRC2**	proline rich nuclear recep	protein_coding	291	254	292	255	0.873
55630	NM_001280557,NM	**SLC39A4**	solute carrier family 39 m	protein_coding	0	0	1	1	1.000
55631	NM_017768,XM_00!	**LRRC40**	leucine rich repeat conta	protein_coding	63	54	64	55	0.861
55632	NM_001308097,NM	**G2E3**	G2/M-phase specific E3	protein_coding	24	20	25	21	0.827
55633	NM_017772,NR_13	**TBC1D22B**	TBC1 domain family mer	protein_coding	20	18	21	19	0.938
55634	NM_001129898,NM	**KRBOX4**	KRAB box domain conta	protein_coding	4	4	5	5	1.103
55635	NM_001114120,NM	**DEPDC1**	DEP domain containing 1	protein_coding	0	0	1	1	1.000
55636	NM_001316690,NM	**CHD7**	chromodomain helicase	protein_coding	0	0	1	1	1.000
55638	NM_001099743,NM	**SYBU**	syntabulin	protein_coding	0	0	1	1	1.000
55640	NM_001195283,NM	**FLVCR2**	FLVCR heme transporter	protein_coding	0	1	1	2	1.215
55643	NM_017797,XM_01	**BTBD2**	BTB domain containing 2	protein_coding	1	1	2	2	1.172
55644	NM_017807	**OSGEP**	O-sialoglycoprotein endc	protein_coding	29	32	30	33	1.105
55646	NM_001145725,NM	**LYAR**	Ly1 antibody reactive	protein_coding	210	206	211	207	0.982
55647	NM_017817	**RAB20**	RAB20, member RAS on	protein_coding	0	0	1	1	1.000
55650	NM_001202554,NM	**PIGV**	phosphatidylinositol glyc	protein_coding	8	8	9	9	1.009
55651	NM_001034833,NM	**NHP2**	NHP2 ribonucleoprotein	protein_coding	481	503	482	504	1.045
55652	NM_017842,XM_00!	**SLC48A1**	solute carrier family 48 m	protein_coding	5	6	6	7	1.074
55653	NM_001010974,NM	**BCAS4**	breast carcinoma amplifi	protein_coding	16	16	17	17	0.950
55654	NM_001193304,NM	**TMEM127**	transmembrane protein 1	protein_coding	39	38	40	39	0.976
55655	NM_001174081,NM	**NLRP2**	NLR family pyrin domain	protein_coding	0	0	1	1	1.048
55656	NM_017864,NR_07	**INTS8**	integrator complex subur	protein_coding	27	25	28	26	0.919
55657	NM_001136036,NM	**ZNF692**	zinc finger protein 692	protein_coding	13	12	14	13	0.901
55658	NM_001366018,NM	**RNF126**	ring finger protein 126	protein_coding	80	83	81	84	1.034
55659	NM_001353405,NM	**ZNF416**	zinc finger protein 416	protein_coding	5	6	6	7	1.040
55660	NM_001354431,NM	**PRPF40A**	pre-mRNA processing fa	protein_coding	93	85	94	86	0.915
55661	NM_001348187,NM	**DDX27**	DEAD-box helicase 27	protein_coding	164	163	165	164	0.988
55662	NM_017902,XM_01	**HIF1AN**	hypoxia inducible factor 1	protein_coding	32	30	33	31	0.936
55663	NM_001304453,NM	**ZNF446**	zinc finger protein 446	protein_coding	3	3	4	4	0.959
55664	NM_017913	**CDC37L1**	cell division cycle 37 like	protein_coding	14	13	15	14	0.926
55665	NM_001077663,NM	**URGCP**	upregulator of cell prolife	protein_coding	13	14	14	15	1.075
55666	NM_001369698,NM	**NPLOC4**	NPL4 homolog, ubiquitin	protein_coding	88	85	89	86	0.963
55667	NM_001330640,NM	**DENND4C**	DENN domain containing	protein_coding	13	11	14	12	0.857
55668	NM_001322026,NM	**GPATCH2L**	G-patch domain containi	protein_coding	14	13	15	14	0.933
55669	NM_033540,XM_00!	**MFN1**	mitofusin 1	protein_coding	18	16	19	17	0.888
55670	NM_001127649,NM	**PEX26**	peroxisomal biogenesis f	protein_coding	7	6	8	7	0.904

55671	NM_001284280,NM	**PPP4R3A**	protein phosphatase 4 re	protein_coding	100	86	101	87	0.870
55672	NM_017940	**NBPF1**	NBPF member 1	protein_coding	0	0	1	1	0.991
55676	NM_001193513,NM	**SLC30A6**	solute carrier family 30 m	protein_coding	31	27	32	28	0.873
55677	NM_017969,XM_00!	**IWS1**	interacts with SUPT6H, C	protein_coding	79	75	80	76	0.942
55679	NM_001136037,NM	**LIMS2**	LIM zinc finger domain co	protein_coding	0	0	1	1	1.078
55680	NM_001042417,NM	**RUFY2**	RUN and FYVE domain c	protein_coding	3	2	4	3	0.863
55681	NM_001317784,NM	**SCYL2**	SCY1 like pseudokinase	protein_coding	26	22	27	23	0.836
55683	NM_001115016,NM	**KANSL3**	KAT8 regulatory NSL cor	protein_coding	45	41	46	42	0.928
55684	NM_001173988,NM	**RABL6**	RAB, member RAS onco	protein_coding	108	110	109	111	1.012
55686	NM_001372188,NM	**MREG**	melanoregulin	protein_coding	12	11	13	12	0.929
55687	NM_001282782,NM	**TRMU**	tRNA 5-methylaminometl	protein_coding	27	28	28	29	1.026
55689	NM_001351369,NM	**YEATS2**	YEATS domain containin	protein_coding	45	40	46	41	0.890
55690	NM_018026,XM_01	**PACS1**	phosphofurin acidic clust	protein_coding	5	5	6	6	0.953
55691	NM_001318336,NM	**FRMD4A**	FERM domain containing	protein_coding	0	0	1	1	1.000
55692	NM_001320226,NM	**LUC7L**	LUC7 like	protein_coding	46	52	47	53	1.133
55693	NM_018039	**KDM4D**	lysine demethylase 4D	protein_coding	3	3	4	4	0.906
55695	NM_001168347,NM	**NSUN5**	NOP2/Sun RNA methyltr	protein_coding	73	68	74	69	0.935
55696	NM_018047,XR_00	**RBM22**	RNA binding motif protei	protein_coding	113	108	114	109	0.955
55697	NM_001351157,NM	**VAC14**	VAC14 component of PIk	protein_coding	14	14	15	15	1.032
55698	NM_018059	**RADIL**	Rap associating with DIL	protein_coding	0	0	1	1	1.000
55699	NM_018060	**IARS2**	isoleucyl-tRNA synthetas	protein_coding	58	58	59	59	0.994
55700	NM_001286365,NM	**MAP7D1**	MAP7 domain containing	protein_coding	93	97	94	98	1.039
55701	NM_001278529,NM	**ARHGEF40**	Rho guanine nucleotide	protein_coding	0	0	1	1	1.016
55702	NM_018074	**YJU2**	YJU2 splicing factor hom	protein_coding	49	55	50	56	1.126
55703	NM_001160708,NM	**POLR3B**	RNA polymerase III subu	protein_coding	14	15	15	16	1.020
55704	NM_001135597,NM	**CCDC88A**	coiled-coil domain contai	protein_coding	5	4	6	5	0.768
55705	NM_018085	**IPO9**	importin 9	protein_coding	20	18	21	19	0.904
55706	NM_001168551,NM	**NDC1**	NDC1 transmembrane nu	protein_coding	89	76	90	77	0.855
55707	NM_001145277,NM	**NECAP2**	NECAP endocytosis asso	protein_coding	73	74	74	75	1.006
55709	NM_001318716,NM	**KBTBD4**	kelch repeat and BTB do	protein_coding	21	22	22	23	1.012
55711	NM_001271783,NM	**FAR2**	fatty acyl-CoA reductase	protein_coding	11	11	12	12	0.927
55713	NM_001270497,NM	**ZNF334**	zinc finger protein 334	protein_coding	0	0	1	1	1.000
55714	NM_001080477,XM	**TENM3**	teneurin transmembrane	protein_coding	0	0	1	1	0.983
55715	NM_001330556,NM	**DOK4**	docking protein 4	protein_coding	0	0	1	1	1.125
55716	NM_001300750,NM	**LMBR1L**	limb development memb	protein_coding	9	8	10	9	0.842
55717	NM_018117,XM_00!	**WDR11**	WD repeat domain 11	protein_coding	8	8	9	9	0.992
55718	NM_001258033,NM	**POLR3E**	RNA polymerase III subu	protein_coding	90	86	91	87	0.955
55719	NM_001136123,NM	**SLF2**	SMC5-SMC6 complex loc	protein_coding	33	27	34	28	0.845
55720	NM_018128	**TSR1**	TSR1 ribosome maturatio	protein_coding	67	62	68	63	0.931
55721	NM_001160042,NM	**IQCC**	IQ motif containing C	protein_coding	10	8	11	9	0.876
55722	NM_018140,NR_164	**CEP72**	centrosomal protein 72	protein_coding	9	7	10	8	0.828
55723	NM_018154	**ASF1B**	anti-silencing function 1E	protein_coding	147	158	148	159	1.074
55726	NM_018164,XM_00!	**INTS13**	integrator complex subur	protein_coding	90	79	91	80	0.878
55727	NM_001002860,NM	**BTBD7**	BTB domain containing 7	protein_coding	10	8	11	9	0.830
55728	NM_001318359,NM	**N4BP2**	NEDD4 binding protein 2	protein_coding	13	11	14	12	0.848
55729	NM_001286514,NM	**ATF7IP**	activating transcription fa	protein_coding	7	5	8	6	0.830
55731	NM_001077498,NM	**FAM222B**	family with sequence sim	protein_coding	17	17	18	18	1.037
55732	NM_001320047,NM	**C1orf112**	chromosome 1 open rea	protein_coding	17	17	18	18	0.972
55733	NM_001122834,NM	**HHAT**	hedgehog acyltransferas	protein_coding	0	0	1	1	1.000
55734	NM_001319146,NM	**ZFP64**	ZFP64 zinc finger protein	protein_coding	17	20	18	21	1.163
55735	NM_018198,XM_01	**DNAJC11**	DnaJ heat shock protein	protein_coding	91	89	92	90	0.977
55737	NM_018206,XM_00!	**VPS35**	VPS35 retromer complex	protein_coding	39	36	40	37	0.920
55738	NM_001281482,NM	**ARFGAP1**	ADP ribosylation factor G	protein_coding	54	58	55	59	1.060
55739	NM_001242881,NM	**NAXD**	NAD(P)HX dehydratase	protein_coding	29	29	30	30	1.015
55740	NM_001008493,NM	**ENAH**	ENAH actin regulator	protein_coding	0	0	1	1	1.000
55741	NM_001145025,NM	**EDEM2**	ER degradation enhancin	protein_coding	42	40	43	41	0.949
55742	NM_018222,XM_00!	**PARVA**	parvin alpha	protein_coding	0	0	1	1	1.000
55743	NM_001161344,NM	**CHFR**	checkpoint with forkhead	protein_coding	1	1	2	2	0.729
55744	NM_001321197,NM	**COA1**	cytochrome c oxidase as	protein_coding	23	21	24	22	0.895
55745	NM_018229,NR_02	**AP5M1**	adaptor related protein c	protein_coding	11	11	12	12	0.999
55746	NM_018230,XR_00	**NUP133**	nucleoporin 133	protein_coding	35	31	36	32	0.890
55748	NM_001168499,NM	**CNDP2**	carnosine dipeptidase 2	protein_coding	81	85	82	86	1.046
55749	NM_001282959,NM	**CCAR1**	cell division cycle and ap	protein_coding	93	82	94	83	0.883
55750	NM_001364948,NM	**AGK**	acylglycerol kinase	protein_coding	19	23	20	24	1.202
55751	NM_018241,XR_00	**TMEM184C**	transmembrane protein 1	protein_coding	27	22	28	23	0.836
55752	NM_001306147,NM	**SEPTIN11**	septin 11	protein_coding	30	27	31	28	0.919
55753	NM_001143996,NM	**OGDHL**	oxoglutarate dehydroger	protein_coding	0	0	1	1	1.000
55754	NM_001143958,NM	**TMEM30A**	transmembrane protein 3	protein_coding	70	58	71	59	0.829
55755	NM_001011649,NM	**CDK5RAP2**	CDK5 regulatory subunit	protein_coding	27	24	28	25	0.869
55756	NM_001145159,NM	**INTS9**	integrator complex subur	protein_coding	15	14	16	15	0.973
55757	NM_020121,XM_01	**UGGT2**	UDP-glucose glycoprotei	protein_coding	5	4	6	5	0.839
55758	NM_001136223,NM	**RCOR3**	REST corepressor 3	protein_coding	14	14	15	15	0.993
55759	NM_001371664,NM	**WDR12**	WD repeat domain 12	protein_coding	34	33	35	34	0.971
55760	NM_018180	**DHX32**	DEAH-box helicase 32 (p	protein_coding	0	0	1	1	1.000
55761	NM_001307943,NM	**TTC17**	tetratricopeptide repeat c	protein_coding	21	19	22	20	0.900
55762	NM_001172655,NM	**ZNF701**	zinc finger protein 701	protein_coding	0	0	1	1	1.058
55763	NM_001024924,NM	**EXOC1**	exocyst complex compor	protein_coding	17	17	18	18	1.001
55764	NM_001280541,NM	**IFT122**	intraflagellar transport 12	protein_coding	12	13	13	14	1.059
55765	NM_001142569,NM	**INAVA**	innate immunity activator	protein_coding	22	14	23	15	0.628
55766	NM_177925,NR_02	**H2AJ**	H2A.J histone	protein_coding	0	0	1	1	1.000
55768	NM_001145293,NM	**NGLY1**	N-glycanase 1	protein_coding	35	35	36	36	0.993
55769	NM_001105549,NM	**ZNF83**	zinc finger protein 83	protein_coding	9	7	10	8	0.836
55770	NM_018303,NR_07	**EXOC2**	exocyst complex compor	protein_coding	26	23	27	24	0.873
55771	NM_018304,XM_02	**PRR11**	proline rich 11	protein_coding	58	46	59	47	0.793
55773	NM_001199198,NM	**TBC1D23**	TBC1 domain family mer	protein_coding	25	22	26	23	0.899
55775	NM_001008744,NM	**TDP1**	tyrosyl-DNA phosphodies	protein_coding	27	25	28	26	0.937
55776	NM_001304793,NM	**SAYSD1**	SAYSVFN motif domain	protein_coding	10	8	11	9	0.871
55777	NM_001378120,NM	**MBD5**	methyl-CpG binding dom	protein_coding	2	2	3	3	0.940
55778	NM_001267827,NM	**ZNF839**	zinc finger protein 839	protein_coding	4	5	5	6	1.102
55779	NM_001164496,NM	**CFAP44**	cilia and flagella associa	protein_coding	3	2	4	3	0.824
55780	NM_001278531,NM	**ERMARD**	ER membrane associate	protein_coding	12	15	13	16	1.219
55781	NM_001159749,NM	**RIOK2**	RIO kinase 2	protein_coding	47	42	48	43	0.901
55783	NM_001099642,NM	**CMTR2**	cap methyltransferase 2	protein_coding	16	14	17	15	0.872
55784	NM_001159643,NM	**MCTP2**	multiple C2 and transme	protein_coding	33	27	34	28	0.825
55785	NM_018351	**FGD6**	FYVE, RhoGEF and PH	protein_coding	1	1	2	2	0.902
55786	NM_001136038,NM	**ZNF415**	zinc finger protein 415	protein_coding	0	0	1	1	1.000
55787	NM_001168683,NM	**TXLNG**	taxilin gamma	protein_coding	42	37	43	38	0.885
55788	NM_001363722,NM	**LMBRD1**	LMBR1 domain containir	protein_coding	15	13	16	14	0.906
55789	NM_001145208,NM	**DEPDC1B**	DEP domain containing 1	protein_coding	70	58	71	59	0.826
55790	NM_001130518,NM	**CSGALNACT1**	chondroitin sulfate N-ace	protein_coding	1	1	2	2	0.935
55791	NM_001006945,NM	**LRIF1**	ligand dependent nuclea	protein_coding	141	108	142	109	0.769
55793	NM_001040217,NM	**MINDY1**	MINDY lysine 48 deubiqu	protein_coding	0	0	1	1	1.000
55794	NM_018380	**DDX28**	DEAD-box helicase 28	protein_coding	20	20	21	21	0.977
55795	NM_001127202,NM	**PCID2**	PCI domain containing 2	protein_coding	71	61	72	62	0.855
55796	NM_001170701,NM	**MBNL3**	muscleblind like splicing	protein_coding	24	21	25	22	0.893
55798	NM_018396	**METTL2B**	methyltransferase like 2E	protein_coding	37	34	38	35	0.917
55799	NM_018398	**CACNA2D3**	calcium voltage-gated ch	protein_coding	0	0	1	1	1.000
55800	NM_001040151,NM	**SCN3B**	sodium voltage-gated ch	protein_coding	0	0	1	1	1.000
55801	NM_018402	**IL26**	interleukin 26	protein_coding	0	0	1	1	1.000
55802	NM_001290204,NM	**DCP1A**	decapping mRNA 1A	protein_coding	25	19	26	20	0.798
55803	NM_001346712,NM	**ADAP2**	ArfGAP with dual PH dor	protein_coding	0	0	1	1	0.935
55805	NM_001377440,NM	**LRP2BP**	LRP2 binding protein	protein_coding	0	0	1	1	1.170
55806	NM_005144,NM_01	**HR**	HR lysine demethylase a	protein_coding	0	0	1	1	1.000
55808	NM_001289107,NM	**ST6GALNAC1**	ST6 N-acetylgalactosami	protein_coding	0	0	1	1	1.000
55809	NM_001297573,NM	**TRERF1**	transcriptional regulating	protein_coding	20	20	21	21	0.971
55810	NM_018416,XM_01	**FOXJ2**	forkhead box J2	protein_coding	8	7	9	8	0.925
55811	NM_001167749,NM	**ADCY10**	adenylate cyclase 10	protein_coding	0	0	1	1	0.968
55812	NM_001040428,NM	**SPATA7**	spermatogenesis associa	protein_coding	2	2	3	3	0.900
55813	NM_018428,XM_00!	**UTP6**	UTP6 small subunit proc	protein_coding	102	101	103	102	0.984
55814	NM_018429,XM_01	**BDP1**	B double prime 1, subun	protein_coding	42	30	43	31	0.731
55815	NM_001288990,NM	**TSNAXIP1**	translin associated factor	protein_coding	0	0	1	1	1.208
55816	NM_001294161,NM	**DOK5**	docking protein 5	protein_coding	0	0	1	1	1.000
55818	NM_001146688,NM	**KDM3A**	lysine demethylase 3A	protein_coding	20	19	21	20	0.957
55819	NM_001280801,NM	**RNF130**	ring finger protein 130	protein_coding	8	8	9	9	1.114
55821	NM_018436,XM_01	**ALLC**	allantoicase	protein_coding	0	0	1	1	1.000
55823	NM_001290185,NM	**VPS11**	VPS11 core subunit of C	protein_coding	26	28	27	29	1.066

55824	NM_018440,XM_01	**PAG1**	phosphoprotein membra	protein_coding	12	12	13	13	0.978
55825	NM_018441,XM_02	**PECR**	peroxisomal trans-2-enoy	protein_coding	19	11	20	12	0.596
55827	NM_001017977,NM	**DCAF6**	DDB1 and CUL4 associa	protein_coding	32	30	33	31	0.961
55829	NM_018445,NM_20	**SELENOS**	selenoprotein S	protein_coding	96	93	97	94	0.962
55830	NM_001010983,NM	**GLT8D1**	glycosyltransferase 8 do	protein_coding	21	23	22	24	1.117
55831	NM_018447,NR_14	**EMC3**	ER membrane protein co	protein_coding	41	42	42	43	1.012
55832	NM_001329674,NM	**CAND1**	cullin associated and ne	protein_coding	48	41	49	42	0.857
55833	NM_001282530,NM	**UBAP2**	ubiquitin associated prot	protein_coding	48	45	49	46	0.948
55835	NM_018451,NR_04	**CENPJ**	centromere protein J	protein_coding	21	19	22	20	0.900
55837	NM_001318916,NM	**EAPP**	E2F associated phospho	protein_coding	26	30	27	31	1.130
55839	NM_001100624,NM	**CENPN**	centromere protein N	protein_coding	35	34	36	35	0.948
55840	NM_001320041,NM	**EAF2**	ELL associated factor 2	protein_coding	17	14	18	15	0.863
55841	NM_015691	**WWC3**	WWC family member 3	protein_coding	10	7	11	8	0.746
55843	NM_018460,XM_01	**ARHGAP15**	Rho GTPase activating p	protein_coding	73	66	74	67	0.906
55844	NM_001291310,NM	**PPP2R2D**	protein phosphatase 2 re	protein_coding	16	15	17	16	0.897
55845	NM_018462	**BRK1**	BRICK1 subunit of SCAR	protein_coding	227	220	228	221	0.971
55846	NM_018463,NR_13	**ITFG2**	integrin alpha FG-GAP re	protein_coding	16	15	17	16	0.920
55847	NM_018464	**CISD1**	CDGSH iron sulfur doma	protein_coding	31	29	32	30	0.945
55848	NM_018465,XM_00	**PLGRKT**	plasminogen receptor wi	protein_coding	34	30	35	31	0.900
55850	NM_018467,XM_01	**USE1**	unconventional SNARE i	protein_coding	23	32	24	33	1.347
55851	NM_001281532,NM	**PSENEN**	presenilin enhancer, gan	protein_coding	55	58	56	59	1.057
55852	NM_001288732,NM	**TEX2**	testis expressed 2	protein_coding	12	10	13	11	0.896
55853	NR_024628,NR_02	**IDI2-AS1**	IDI2 antisense RNA 1	lncRNA	1	1	2	2	0.907
55854	NM_018471,XM_01	**ZC3H15**	zinc finger CCCH-type co	protein_coding	298	263	299	264	0.882
55855	NR_027141	**DENND10P1**	DENND10 pseudogene 1	transcribed_pseu	7	6	8	7	0.831
55856	NM_001160094,NM	**ACOT13**	acyl-CoA thioesterase 13	protein_coding	17	16	18	17	0.937
55857	NM_001163022,NM	**KIZ**	kizuna centrosomal prote	protein_coding	7	7	8	8	0.932
55858	NM_018475,NR_07	**TMEM165**	transmembrane protein 1	protein_coding	79	81	80	82	1.019
55859	NM_018476	**BEX1**	brain expressed X-linked	protein_coding	0	0	1	1	1.000
55860	NM_018477,XM_01	**ACTR10**	actin related protein 10	protein_coding	56	53	57	54	0.949
55861	NM_001048221,NM	**DBNDD2**	dysbindin domain contai	protein_coding	0	0	1	1	0.893
55862	NM_001002030,NM	**ECHDC1**	ethylmalonyl-CoA decarb	protein_coding	83	70	84	71	0.844
55863	NM_001193537,NM	**TMEM126B**	transmembrane protein 1	protein_coding	124	116	125	117	0.930
55867	NM_001307985,NM	**SLC22A11**	solute carrier family 22 m	protein_coding	0	0	1	1	1.000
55869	NM_001166418,NM	**HDAC8**	histone deacetylase 8	protein_coding	25	24	26	25	0.946
55870	NM_001366177,NM	**ASH1L**	ASH1 like histone lysine	protein_coding	11	9	12	10	0.788
55871	NM_001145355,NM	**CBWD1**	COBW domain containin	protein_coding	65	62	66	63	0.951
55872	NM_001278945,NM	**PBK**	PDZ binding kinase	protein_coding	0	0	1	1	1.000
55876	NM_001042471,NM	**GSDMB**	gasdermin B	protein_coding	5	6	6	7	1.056
55879	NM_018558,XM_01	**GABRQ**	gamma-aminobutyric aci	protein_coding	0	0	1	1	1.000
55884	NM_001278557,NM	**WSB2**	WD repeat and SOCS bo	protein_coding	11	11	12	12	1.048
55885	NM_001001395,NM	**LMO3**	LIM domain only 3	protein_coding	0	0	1	1	1.000
55888	NM_001288590,NM	**ZKSCAN7**	zinc finger with KRAB an	protein_coding	0	0	1	1	1.000
55889	NM_018652	**GOLGA6B**	golgin A6 family member	protein_coding	0	0	1	1	1.000
55890	NM_001366261,NM	**GPRC5C**	G protein-coupled recept	protein_coding	0	0	1	1	1.000
55891	NM_018655	**LENEP**	lens epithelial protein	protein_coding	0	0	1	1	1.000
55892	NM_001185118,NM	**MYNN**	myoneurin	protein_coding	19	19	20	20	0.989
55893	NM_018660	**ZNF395**	zinc finger protein 395	protein_coding	0	0	1	1	1.000
55894	NM_018661	**DEFB103B**	defensin beta 103B	protein_coding	0	0	1	1	1.000
55897	NM_018670,XR_00	**MESP1**	mesoderm posterior bHL	protein_coding	0	1	1	2	1.302
55898	NM_001039675,NM	**UNC45A**	unc-45 myosin chaperon	protein_coding	43	44	44	45	1.012
55900	NM_001012320,NM	**ZNF302**	zinc finger protein 302	protein_coding	58	48	59	49	0.844
55901	NM_018676,NM_19	**THSD1**	thrombospondin type 1 d	protein_coding	1	1	2	2	0.782
55902	NM_001076552,NM	**ACSS2**	acyl-CoA synthetase sho	protein_coding	0	0	1	1	1.000
55904	NM_018682,NM_18	**KMT2E**	lysine methyltransferase	protein_coding	44	37	45	38	0.838
55905	NM_018683	**RNF114**	ring finger protein 114	protein_coding	123	120	124	121	0.979
55906	NM_001178032,NM	**ZC4H2**	zinc finger C4H2-type co	protein_coding	0	0	1	1	1.000
55907	NM_018686,NR_13	**CMAS**	cytidine monophosphate	protein_coding	50	49	51	50	0.977
55908	NM_018687	**ANGPTL8**	angiopoietin like 8	protein_coding	0	0	1	1	1.000
55909	NM_001363046,NM	**BIN3**	bridging integrator 3	protein_coding	18	15	19	16	0.867
55911	NM_018690	**APOBR**	apolipoprotein B recepto	protein_coding	50	53	51	54	1.063
55914	NM_001006600,NM	**ERBIN**	erbb2 interacting protein	protein_coding	39	33	40	34	0.866
55915	NM_018697	**LANCL2**	LanC like 2	protein_coding	23	24	24	25	1.011
55916	NM_001242617,NM	**NXT2**	nuclear transport factor 2	protein_coding	20	20	21	21	0.979
55917	NM_018704,XM_01	**CTTNBP2NL**	CTTNBP2 N-terminal like	protein_coding	3	3	4	4	0.960
55920	NM_001136204,NM	**RCC2**	regulator of chromosome	protein_coding	342	333	343	334	0.974
55922	NM_017544,NR_16	**NKRF**	NFKB repressing factor	protein_coding	27	25	28	26	0.951
55924	NM_019099,NM_19	**INKA2**	inka box actin regulator 2	protein_coding	0	0	1	1	0.951
55929	NM_001034023,NM	**DMAP1**	DNA methyltransferase 1	protein_coding	47	49	48	50	1.055
55930	NM_018728,XM_01	**MYO5C**	myosin VC	protein_coding	3	3	4	4	1.046
55937	NM_001256169,NM	**APOM**	apolipoprotein M	protein_coding	7	8	8	9	1.050
55954	NM_001003692,NM	**ZMAT5**	zinc finger matrin-type 5	protein_coding	29	31	30	32	1.066
55957	NM_001369780,NM	**LIN37**	lin-37 DREAM MuvB core	protein_coding	11	12	12	13	1.082
55958	NM_018847	**KLHL9**	kelch like family member	protein_coding	19	16	20	17	0.857
55959	NM_001161841,NM	**SULF2**	sulfatase 2	protein_coding	0	0	1	1	1.000
55964	NM_001363845,NM	**SEPTIN3**	septin 3	protein_coding	0	0	1	1	1.000
55966	NM_001042478,NM	**AJAP1**	adherens junctions asso	protein_coding	0	0	1	1	1.000
55967	NM_001258338,NM	**NDUFA12**	NADH:ubiquinone oxidor	protein_coding	203	208	204	209	1.023
55968	NM_001206736,NM	**NSFL1C**	NSFL1 cofactor	protein_coding	53	54	54	55	1.008
55969	NM_001199534,NM	**RAB5IF**	RAB5 interacting factor	protein_coding	245	261	246	262	1.064
55970	NM_018841,XM_01	**GNG12**	G protein subunit gamma	protein_coding	0	0	1	1	1.000
55971	NM_018842	**BAIAP2L1**	BAR/IMD domain contair	protein_coding	0	0	1	1	1.000
55972	NM_018843	**SLC25A40**	solute carrier family 25 m	protein_coding	22	18	23	19	0.836
55973	NM_001008405,NM	**BCAP29**	B cell receptor associate	protein_coding	26	19	27	20	0.740
55974	NM_001122837,NM	**SLC50A1**	solute carrier family 50 m	protein_coding	98	98	99	99	0.993
55975	NM_001031710,NM	**KLHL7**	kelch like family member	protein_coding	10	8	11	9	0.816
55997	NM_001270420,NM	**CFC1**	cripto, FRL-1, cryptic fam	protein_coding	0	0	1	1	1.000
55998	NM_032946,NR_02	**NXF5**	nuclear RNA export facto	protein_coding	0	0	1	1	1.000
55999	NR_002216	**NXF4**	nuclear RNA export facto	transcribed_pseu	0	0	1	1	1.000
56000	NM_022052	**NXF3**	nuclear RNA export facto	protein_coding	0	0	1	1	1.000
56001	NM_022053	**NXF2**	nuclear RNA export facto	protein_coding	0	0	1	1	1.000
56005	NM_019107,XM_01	**MYDGF**	myeloid derived growth f	protein_coding	208	205	209	206	0.988
56006	NM_019108,XM_00	**SMG9**	SMG9 nonsense mediate	protein_coding	26	32	27	33	1.219
56033	NM_021570	**BARX1**	BARX homeobox 1	protein_coding	0	0	1	1	1.000
56034	NM_016205,NR_03	**PDGFC**	platelet derived growth fa	protein_coding	0	0	1	1	1.000
56052	NM_001330504,NM	**ALG1**	ALG1 chitobiosyldiphosp	protein_coding	40	44	41	45	1.094
56061	NM_019116,XM_01	**UBFD1**	ubiquitin family domain c	protein_coding	63	59	64	60	0.937
56062	NM_019117,NM_05	**KLHL4**	kelch like family member	protein_coding	9	9	10	10	0.949
56063	NM_001366191,NM	**TMEM234**	transmembrane protein 2	protein_coding	2	3	3	4	1.080
56097	NM_018929,NM_03	**PCDHGC5**	protocadherin gamma su	protein_coding	0	0	1	1	1.000
56098	NM_018928,NM_03	**PCDHGC4**	protocadherin gamma su	protein_coding	0	0	1	1	1.000
56099	NM_018927,NM_03	**PCDHGB7**	protocadherin gamma su	protein_coding	0	0	1	1	1.056
56100	NM_018926,NM_03	**PCDHGB6**	protocadherin gamma su	protein_coding	0	0	1	1	1.030
56101	NM_018925,NM_03	**PCDHGB5**	protocadherin gamma su	protein_coding	0	0	1	1	0.990
56102	NM_018924,NM_03	**PCDHGB3**	protocadherin gamma su	protein_coding	1	1	2	2	0.975
56103	NM_018923,NM_03	**PCDHGB2**	protocadherin gamma su	protein_coding	2	2	3	3	0.973
56104	NM_018922,NM_03	**PCDHGB1**	protocadherin gamma su	protein_coding	0	0	1	1	1.076
56105	NM_018914,NM_03	**PCDHGA11**	protocadherin gamma su	protein_coding	0	0	1	1	0.962
56106	NM_018913,NM_03	**PCDHGA10**	protocadherin gamma su	protein_coding	0	0	1	1	1.000
56107	NM_018921,NM_03	**PCDHGA9**	protocadherin gamma su	protein_coding	0	0	1	1	0.937
56108	NM_018920,NM_03	**PCDHGA7**	protocadherin gamma su	protein_coding	0	0	1	1	1.093
56109	NM_018919,NM_03	**PCDHGA6**	protocadherin gamma su	protein_coding	1	1	2	2	0.825
56110	NM_018918,NM_03	**PCDHGA5**	protocadherin gamma su	protein_coding	0	1	1	2	1.441
56111	NM_018917,NM_03	**PCDHGA4**	protocadherin gamma su	protein_coding	0	0	1	1	1.017
56112	NM_018916,NM_03	**PCDHGA3**	protocadherin gamma su	protein_coding	0	0	1	1	0.981
56113	NM_018915,NM_03	**PCDHGA2**	protocadherin gamma su	protein_coding	0	0	1	1	0.965
56114	NM_018912,NM_03	**PCDHGA1**	protocadherin gamma su	protein_coding	0	0	1	1	0.941
56119	id-PCDHACT	**PCDHACT**	protocadherin alpha con	C_region	0	0	1	1	1.000
56120	NR_001297	**PCDHGB8P**	protocadherin gamma su	transcribed_pseu	0	0	1	1	1.039
56121	NM_018935	**PCDHB15**	protocadherin beta 15	protein_coding	0	0	1	1	0.984
56122	NM_018934	**PCDHB14**	protocadherin beta 14	protein_coding	0	0	1	1	0.926
56123	NM_018933	**PCDHB13**	protocadherin beta 13	protein_coding	0	0	1	1	0.985
56124	NM_018932	**PCDHB12**	protocadherin beta 12	protein_coding	0	0	1	1	1.023
56125	NM_018931	**PCDHB11**	protocadherin beta 11	protein_coding	0	0	1	1	1.000
56126	NM_018930	**PCDHB10**	protocadherin beta 10	protein_coding	0	0	1	1	1.081
56127	NM_019119	**PCDHB9**	protocadherin beta 9	protein_coding	1	0	2	1	0.967

56128	NM_019120	**PCDHB8**	protocadherin beta 8	protein_coding	0	0	1	1	1.000
56129	NM_018940	**PCDHB7**	protocadherin beta 7	protein_coding	0	0	1	1	1.021
56130	NM_001303145,NM	**PCDHB6**	protocadherin beta 6	protein_coding	0	0	1	1	1.010
56131	NM_018938	**PCDHB4**	protocadherin beta 4	protein_coding	0	0	1	1	0.994
56132	NM_018937	**PCDHB3**	protocadherin beta 3	protein_coding	0	0	1	1	1.000
56133	NM_018936	**PCDHB2**	protocadherin beta 2	protein_coding	0	0	1	1	1.005
56134	NM_018899,NM_03	**PCDHAC2**	protocadherin alpha sub	protein_coding	0	0	1	1	1.000
56135	NM_018898,NM_03	**PCDHAC1**	protocadherin alpha sub	protein_coding	0	0	1	1	1.000
56136	NM_018904,NM_03	**PCDHA13**	protocadherin alpha 13	protein_coding	0	0	1	1	1.000
56137	NM_018903,NM_03	**PCDHA12**	protocadherin alpha 12	protein_coding	0	0	1	1	1.000
56138	NM_018902,NM_03	**PCDHA11**	protocadherin alpha 11	protein_coding	0	0	1	1	1.000
56139	NM_018901,NM_03	**PCDHA10**	protocadherin alpha 10	protein_coding	0	0	1	1	1.000
56140	NM_018911,NM_03	**PCDHA8**	protocadherin alpha 8	protein_coding	0	0	1	1	0.949
56141	NM_018910,NM_03	**PCDHA7**	protocadherin alpha 7	protein_coding	0	0	1	1	1.000
56142	NM_018909,NM_03	**PCDHA6**	protocadherin alpha 6	protein_coding	0	0	1	1	1.000
56143	NM_018908,NM_03	**PCDHA5**	protocadherin alpha 5	protein_coding	0	0	1	1	1.000
56144	NM_018907,NM_03	**PCDHA4**	protocadherin alpha 4	protein_coding	0	0	1	1	1.000
56145	NM_018906,NM_03	**PCDHA3**	protocadherin alpha 3	protein_coding	0	0	1	1	1.000
56146	NM_018905,NM_03	**PCDHA2**	protocadherin alpha 2	protein_coding	0	0	1	1	1.000
56147	NM_018900,NM_03	**PCDHA1**	protocadherin alpha 1	protein_coding	0	0	1	1	1.000
56154	NM_001350162,NR	**TEX15**	testis expressed 15, meio	protein_coding	0	0	1	1	1.000
56155	NM_001201457,NM	**TEX14**	testis expressed 14, inter	protein_coding	0	0	1	1	1.000
56156	NM_031273	**TEX13B**	testis expressed 13B	protein_coding	0	0	1	1	1.000
56157	NM_001291277,NM	**TEX13A**	testis expressed 13A	protein_coding	0	0	1	1	1.000
56158	NM_031275	**TEX12**	testis expressed 12	protein_coding	1	1	2	2	0.896
56159	NM_001003811,NM	**TEX11**	testis expressed 11	protein_coding	1	1	2	2	1.039
56160	NM_138704	**NSMCE3**	NSE3 homolog, SMC5-S	protein_coding	19	20	20	21	1.036
56163	NM_001184993,NM	**RNF17**	ring finger protein 17	protein_coding	0	0	1	1	1.000
56164	NM_001260504,NM	**STK31**	serine/threonine kinase 3	protein_coding	0	0	1	1	1.000
56165	NM_001365891,NM	**TDRD1**	tudor domain containing	protein_coding	0	0	1	1	1.019
56169	NM_031415,XM_01	**GSDMC**	gasdermin C	protein_coding	0	0	1	1	0.803
56171	NM_018897,XM_01	**DNAH7**	dynein axonemal heavy c	protein_coding	0	0	1	1	1.000
56172	NM_054027,XM_01	**ANKH**	ANKH inorganic pyropho	protein_coding	5	4	6	5	0.847
56180	NM_001306188,NM	**MOSPD1**	motile sperm domain cor	protein_coding	15	13	16	14	0.891
56181	NM_001099625,NM	**MTFR1L**	mitochondrial fission reg	protein_coding	15	14	16	15	0.953
56203	NM_001304418,NM	**LMOD3**	leiomodin 3	protein_coding	0	0	1	1	1.000
56204	NM_001286495,NM	**FAM214A**	family with sequence sim	protein_coding	11	9	12	10	0.865
56241	NM_019601	**SUSD2**	sushi domain containing	protein_coding	0	0	1	1	0.987
56242	NM_001331133,NM	**ZNF253**	zinc finger protein 253	protein_coding	2	1	3	2	0.827
56243	NM_001098500,NM	**KIAA1217**	KIAA1217	protein_coding	2	2	3	3	0.921
56244	NM_001304561,XM	**BTNL2**	butyrophilin like 2	protein_coding	0	0	1	1	1.062
56245	NM_001162495,NM	**C21orf62**	chromosome 21 open rea	protein_coding	0	0	1	1	1.000
56246	NM_001285394,NM	**MRAP**	melanocortin 2 receptor a	protein_coding	0	0	1	1	0.969
56252	NM_019589,XM_00	**YLPM1**	YLP motif containing 1	protein_coding	28	23	29	24	0.825
56253	NM_001304782,NM	**CRTAM**	cytotoxic and regulatory	protein_coding	0	0	1	1	0.926
56254	NM_019592,XM_01	**RNF20**	ring finger protein 20	protein_coding	39	39	40	40	0.991
56255	NM_021156	**TMX4**	thioredoxin related transı	protein_coding	26	27	27	28	1.057
56256	NM_001354173,NM	**SERTAD4**	SERTA domain containir	protein_coding	0	0	1	1	1.000
56257	NM_001194990,NM	**MEPCE**	methylphosphate cappin	protein_coding	87	100	88	101	1.142
56259	NM_001281495,NM	**CTNNBL1**	catenin beta like 1	protein_coding	150	156	151	157	1.039
56260	NR_161216,NR_16	**C8orf44**	chromosome 8 open rea	lncRNA	10	7	11	8	0.748
56261	NM_019593,XM_00	**GPCPD1**	glycerophosphocholine p	protein_coding	15	16	16	17	1.111
56262	NM_001127244,NM	**LRRC8A**	leucine rich repeat conta	protein_coding	16	19	17	20	1.130
56265	NM_001184699,NM	**CPXM1**	carboxypeptidase X, M14	protein_coding	0	0	1	1	1.000
56267	NM_001008661,NM	**KYAT3**	kynurenine aminotransfe	protein_coding	34	27	35	28	0.808
56269	NM_019612,XM_00	**IRGC**	immunity related GTPase	protein_coding	0	0	1	1	1.000
56270	NM_019613,XM_00	**WDR45B**	WD repeat domain 45B	protein_coding	72	71	73	72	0.974
56271	NM_001080425,NM	**BEX4**	brain expressed X-linked	protein_coding	0	0	1	1	1.000
56286	gene-DAD1P1	**DAD1P1**	defender against cell dea	pseudogene	0	0	1	1	0.975
56287	NM_019617	**GKN1**	gastrokine 1	protein_coding	0	0	1	1	1.000
56288	NM_001184785,NM	**PARD3**	par-3 family cell polarity	protein_coding	0	0	1	1	1.000
56300	NM_001278568,NM	**IL36G**	interleukin 36 gamma	protein_coding	0	0	1	1	0.870
56301	NM_019849,XM_00	**SLC7A10**	solute carrier family 7 me	protein_coding	0	0	1	1	1.000
56302	NM_019841	**TRPV5**	transient receptor potent	protein_coding	0	0	1	1	1.000
56311	NM_019644	**ANKRD7**	ankyrin repeat domain 7	protein_coding	0	0	1	1	1.000
56339	NM_019852,XM_00	**METTL3**	methyltransferase like 3	protein_coding	38	36	39	37	0.952
56341	NM_001256536,NM	**PRMT8**	protein arginine methyltr	protein_coding	0	0	1	1	1.000
56342	NM_001346139,NM	**PPAN**	peter pan homolog	protein_coding	101	100	102	101	0.994
56344	NM_019855,XM_01	**CABP5**	calcium binding protein 5	protein_coding	0	0	1	1	1.000
56413	NM_001164692,NM	**LTB4R2**	leukotriene B4 receptor 2	protein_coding	5	4	6	5	0.866
56474	NM_001144002,NM	**CTPS2**	CTP synthase 2	protein_coding	4	4	5	5	1.014
56475	NM_019845	**RPRM**	reprimo, TP53 dependen	protein_coding	0	0	1	1	1.000
56476	gene-ABCB10P1	**ABCB10P1**	ABCB10 pseudogene 1	pseudogene	0	0	1	1	1.000
56477	NM_001301873,NM	**CCL28**	C-C motif chemokine liga	protein_coding	0	0	1	1	1.000
56478	NM_001164501,NM	**EIF4ENIF1**	eukaryotic translation init	protein_coding	12	13	13	14	1.047
56479	NM_001160130,NM	**KCNQ5**	potassium voltage-gated	protein_coding	23	20	24	21	0.866
56521	NM_021800,NM_20	**DNAJC12**	DnaJ heat shock protein	protein_coding	3	3	4	4	0.873
56547	NM_021801,XM_01	**MMP26**	matrix metallopeptidase 2	protein_coding	0	0	1	1	1.000
56548	NM_019886	**CHST7**	carbohydrate sulfotransfe	protein_coding	0	1	1	2	1.052
56603	NM_001277742,NM	**CYP26B1**	cytochrome P450 family	protein_coding	0	0	1	1	1.000
56604	gene-TUBB7P	**TUBB7P**	tubulin beta 7 pseudoge	pseudogene	0	0	1	1	1.000
56605	NM_019891,XM_01	**ERO1B**	endoplasmic reticulum o	protein_coding	0	0	1	1	1.022
56606	NM_001001290,NM	**SLC2A9**	solute carrier family 2 me	protein_coding	2	2	3	3	0.973
56616	NM_001278302,NM	**DIABLO**	diablo IAP-binding mitocl	protein_coding	81	87	82	88	1.072
56623	NM_001318502,NM	**INPP5E**	inositol polyphosphate-5	protein_coding	6	7	7	8	1.120
56624	NM_001143974,NM	**ASAH2**	N-acylsphingosine amido	protein_coding	1	1	2	2	1.002
56647	NM_016567,NM_07	**BCCIP**	BRCA2 and CDKN1A inte	protein_coding	206	197	207	198	0.957
56648	NM_020390	**EIF5A2**	eukaryotic translation init	protein_coding	0	0	1	1	1.000
56649	NM_001083947,NM	**TMPRSS4**	transmembrane serine p	protein_coding	0	0	1	1	1.000
56650	NM_001040181,NM	**CLDND1**	claudin domain containir	protein_coding	260	231	261	232	0.889
56651	NR_023925,NR_02	**LINC00470**	long intergenic non-prote	lncRNA	0	0	1	1	1.000
56652	NM_001163812,NM	**TWNK**	twinkle mtDNA helicase	protein_coding	39	41	40	42	1.071
56654	NM_015392	**NPDC1**	neural proliferation, differ	protein_coding	12	15	13	16	1.267
56655	NM_019896	**POLE4**	DNA polymerase epsilon	protein_coding	46	49	47	50	1.062
56656	NM_019897	**OR2S2**	olfactory receptor family	protein_coding	0	0	1	1	1.000
56658	NM_001369521,NM	**TRIM39**	tripartite motif containing	protein_coding	14	13	15	14	0.941
56659	NM_022054	**KCNK13**	potassium two pore dom	protein_coding	0	0	1	1	1.000
56660	NM_022055	**KCNK12**	potassium two pore dom	protein_coding	0	0	1	1	1.000
56662	NR_026705	**VTRNA1-3**	vault RNA 1-3	vault_RNA	0	0	1	1	1.000
56663	NR_026704	**VTRNA1-2**	vault RNA 1-2	vault_RNA	0	0	1	1	1.000
56664	NR_026703	**VTRNA1-1**	vault RNA 1-1	vault_RNA	0	0	1	1	0.980
56666	NM_001160300,NM	**PANX2**	pannexin 2	protein_coding	0	0	1	1	1.000
56667	NM_033049	**MUC13**	mucin 13, cell surface as	protein_coding	0	0	1	1	1.000
56670	NM_033050	**SUCNR1**	succinate receptor 1	protein_coding	0	0	1	1	0.979
56672	NM_001206646,NM	**AKIP1**	A-kinase interacting prote	protein_coding	18	17	19	18	0.968
56673	NM_020643,XM_01	**C11orf16**	chromosome 11 open rea	protein_coding	0	0	1	1	1.000
56674	NM_001286094,NM	**TMEM9B**	TMEM9 domain family m	protein_coding	57	55	58	56	0.970
56675	NM_020645,XM_02	**NRIP3**	nuclear receptor interact	protein_coding	0	0	1	1	0.940
56676	NM_020646	**ASCL3**	achaete-scute family bHl	protein_coding	0	0	1	1	1.000
56677	gene-FABP3P2	**FABP3P2**	fatty acid binding protein	pseudogene	0	0	1	1	1.000
56681	NM_001142648,NM	**SAR1A**	secretion associated Ras	protein_coding	50	46	51	47	0.926
56683	NM_001350334,NM	**CFAP298**	cilia and flagella associat	protein_coding	58	50	59	51	0.858
56704	NM_001317830,NM	**JPH1**	junctophilin 1	protein_coding	0	0	1	1	1.000
56729	NM_001193374,NM	**RETN**	resistin	protein_coding	0	0	1	1	1.000
56731	NM_020062	**SLC2A4RG**	SLC2A4 regulator	protein_coding	1	2	2	3	1.048
56751	NM_020064	**BARHL1**	BarH like homeobox 1	protein_coding	0	0	1	1	1.000
56776	NM_001305424,NM	**FMN2**	formin 2	protein_coding	18	14	19	15	0.751
56829	NM_001363491,NM	**ZC3HAV1**	zinc finger CCCH-type co	protein_coding	71	65	72	66	0.925
56832	NM_020124	**IFNK**	interferon kappa	protein_coding	0	0	1	1	1.000
56833	NM_001330741,NM	**SLAMF8**	SLAM family member 8	protein_coding	4	5	5	6	1.190
56834	NM_001170880,NM	**GPR137**	G protein-coupled recept	protein_coding	25	26	26	27	1.023
56848	NM_001204158,NM	**SPHK2**	sphingosine kinase 2	protein_coding	34	40	35	41	1.184
56849	NM_001348258,NM	**TCEAL7**	transcription elongation f	protein_coding	0	0	1	1	1.000
56850	NM_020137	**GRIPAP1**	GRIP1 associated protei	protein_coding	29	34	30	35	1.163
56851	NM_020154	**EMC7**	ER membrane protein co	protein_coding	165	168	166	169	1.017
56852	NM_020165,XM_01	**RAD18**	RAD18 E3 ubiquitin prote	protein_coding	20	18	21	19	0.923

56853	NM_001025087,NM	**CELF4**	CUGBP Elav-like family	protein_coding	0	0	1	1	1.000
56882	NM_001038707,NM	**CDC42SE1**	CDC42 small effector 1	protein_coding	141	140	142	141	0.996
56884	NM_001128427,NM	**FSTL5**	follistatin like 5	protein_coding	0	0	1	1	1.000
56886	NM_020120,NR_02	**UGGT1**	UDP-glucose glycoprote	protein_coding	43	38	44	39	0.904
56888	NM_020122,XM_00	**KCMF1**	potassium channel modu	protein_coding	27	25	28	26	0.926
56889	NM_020123,XM_01	**TM9SF3**	transmembrane 9 superf	protein_coding	96	78	97	79	0.811
56890	NM_001205028,NM	**MDM1**	Mdm1 nuclear protein	protein_coding	17	13	18	14	0.780
56891	NM_020129,NM_20	**LGALS14**	galectin 14	protein_coding	0	0	1	1	1.000
56892	NM_020130	**TCIM**	transcriptional and immu	protein_coding	0	0	1	1	1.000
56893	NM_001304342,NM	**UBQLN4**	ubiquilin 4	protein_coding	55	53	56	54	0.950
56894	NM_001037553,NM	**AGPAT3**	1-acylglycerol-3-phospha	protein_coding	42	38	43	39	0.912
56895	NM_020133,XM_00	**AGPAT4**	1-acylglycerol-3-phospha	protein_coding	1	1	2	2	0.936
56896	NM_001253723,NM	**DPYSL5**	dihydropyrimidinase like	protein_coding	0	0	1	1	1.000
56897	NM_020135,NM_13	**WRNIP1**	WRN helicase interacting	protein_coding	56	58	57	59	1.032
56898	NM_020139,XM_00	**BDH2**	3-hydroxybutyrate dehyd	protein_coding	7	9	8	10	1.190
56899	NM_001204065,NM	**ANKS1B**	ankyrin repeat and sterile	protein_coding	0	0	1	1	1.017
56900	NM_001322248,NM	**TMEM167B**	transmembrane protein 1	protein_coding	48	45	49	46	0.933
56901	NM_020142	**NDUFA4L2**	NDUFA4 mitochondrial c	protein_coding	0	0	1	1	1.000
56902	NM_001329916,NM	**PNO1**	partner of NOB1 homolo	protein_coding	97	102	98	103	1.048
56903	NM_020144	**PAPOLB**	poly(A) polymerase beta	protein_coding	0	0	1	1	1.000
56904	NM_001287045,NM	**SH3GLB2**	SH3 domain containing (	protein_coding	25	30	26	31	1.176
56905	NM_015492	**C15orf39**	chromosome 15 open rea	protein_coding	94	100	95	101	1.064
56906	NM_020147	**THAP10**	THAP domain containing	protein_coding	0	0	1	1	1.000
56907	NM_001128626,NM	**SPIRE1**	spire type actin nucleatic	protein_coding	1	1	2	2	0.871
56910	NM_020151,XM_01	**STARD7**	StAR related lipid transfe	protein_coding	286	270	287	271	0.944
56911	NM_001286617,NM	**MAP3K7CL**	MAP3K7 C-terminal like	protein_coding	3	3	4	4	0.791
56912	NM_001168618,NM	**IFT46**	intraflagellar transport 46	protein_coding	31	36	32	37	1.155
56913	NM_020156,XM_01	**C1GALT1**	core 1 synthase, glycopr	protein_coding	20	17	21	18	0.848
56914	NM_020157,XM_01	**OTOR**	otoraplin	protein_coding	0	0	1	1	1.000
56915	NM_020158	**EXOSC5**	exosome component 5	protein_coding	184	194	185	195	1.053
56916	NM_001128429,NM	**SMARCAD1**	SWI/SNF-related, matrix-	protein_coding	12	9	13	10	0.800
56917	NM_001009813,NM	**MEIS3**	Meis homeobox 3	protein_coding	0	0	1	1	1.000
56918	NM_001162483,NM	**C2orf83**	chromosome 2 open rea	protein_coding	0	0	1	1	1.000
56919	NM_001199699,NM	**DHX33**	DEAH-box helicase 33	protein_coding	38	36	39	37	0.952
56920	NM_020163,XM_02	**SEMA3G**	semaphorin 3G	protein_coding	0	0	1	1	1.040
56922	NM_001293273,NM	**MCCC1**	methylcrotonoyl-CoA car	protein_coding	32	32	33	33	0.988
56923	NM_020167,XM_01	**NMUR2**	neuromedin U receptor 2	protein_coding	0	0	1	1	1.000
56924	NM_001276717,NM	**PAK6**	p21 (RAC1) activated kin	protein_coding	0	0	1	1	1.000
56925	NM_020169,XM_01	**LXN**	latexin	protein_coding	0	0	1	1	1.000
56926	NM_001321463,NM	**NCLN**	nicalin	protein_coding	81	80	82	81	0.989
56927	NM_001080452,NM	**GPR108**	G protein-coupled recept	protein_coding	21	23	22	24	1.076
56928	NM_001077238,NM	**SPPL2B**	signal peptide peptidase	protein_coding	16	16	17	17	1.003
56929	NM_020177,XM_00	**FEM1C**	fem-1 homolog C	protein_coding	11	10	12	11	0.913
56931	NM_001161619,NM	**DUS3L**	dihydrouridine synthase	protein_coding	53	59	54	60	1.112
56934	NM_001082533,NM	**CA10**	carbonic anhydrase 10	protein_coding	0	0	1	1	1.000
56935	NM_020179,XM_01	**SMCO4**	single-pass membrane p	protein_coding	0	0	1	1	1.000
56936	NM_001271507	**CCDC177**	coiled-coil domain contai	protein_coding	0	0	1	1	1.000
56937	NM_001255976,NM	**PMEPA1**	prostate transmembrane	protein_coding	0	0	1	1	1.000
56938	NM_001248002,NM	**ARNTL2**	aryl hydrocarbon recepto	protein_coding	40	33	41	34	0.839
56940	NM_001286555,NM	**DUSP22**	dual specificity phosphat	protein_coding	23	20	24	21	0.891
56941	NM_001006109,NM	**HMCES**	5-hydroxymethylcytosine	protein_coding	29	27	30	28	0.939
56942	NM_001351967,NM	**CMC2**	C-X9-C motif containing	protein_coding	25	14	26	15	0.593
56943	NM_001193557,NM	**ENY2**	ENY2 transcription and e	protein_coding	65	66	66	67	1.012
56944	NM_001286352,NM	**OLFML3**	olfactomedin like 3	protein_coding	0	1	1	2	1.075
56945	NM_001363857,NM	**MRPS22**	mitochondrial ribosomal	protein_coding	119	105	120	106	0.887
56946	NM_001300942,NM	**EMSY**	EMSY transcriptional rep	protein_coding	18	15	19	16	0.850
56947	NM_001277061,NM	**MFF**	mitochondrial fission fact	protein_coding	58	55	59	56	0.946
56948	NM_001290292,NM	**SDR39U1**	short chain dehydrogena	protein_coding	17	18	18	19	1.041
56949	NM_020196	**XAB2**	XPA binding protein 2	protein_coding	75	77	76	78	1.034
56950	NM_020197,XR_42	**SMYD2**	SET and MYND domain	protein_coding	45	39	46	40	0.880
56951	NM_020199	**C5orf15**	chromosome 5 open rea	protein_coding	88	79	89	80	0.900
56952	NM_001282786,NM	**PRTFDC1**	phosphoribosyl transfera	protein_coding	0	0	1	1	1.000
56953	NM_020201,XM_00	**NT5M**	5',3'-nucleotidase, mitoc	protein_coding	0	0	1	1	0.907
56954	NM_020202	**NIT2**	nitrilase family member 2	protein_coding	10	10	11	11	1.002
56955	NM_001184694,NM	**MEPE**	matrix extracellular phos	protein_coding	0	0	1	1	1.000
56956	NM_001014434,NM	**LHX9**	LIM homeobox 9	protein_coding	0	0	1	1	1.000
56957	NM_020205,XM_01	**OTUD7B**	OTU deubiquitinase 7B	protein_coding	13	11	14	12	0.848
56961	NM_001372011,NM	**SHD**	Src homology 2 domain	protein_coding	0	0	1	1	1.000
56963	NM_001166283,NM	**RGMA**	repulsive guidance mole	protein_coding	0	0	1	1	1.000
56964	NM_001284395,NM	**WDR93**	WD repeat domain 93	protein_coding	0	0	1	1	1.000
56965	NM_001323515,NM	**PARP6**	poly(ADP-ribose) polyme	protein_coding	59	58	60	59	0.980
56967	NM_001252507,NM	**C14orf132**	chromosome 14 open rea	protein_coding	0	0	1	1	1.000
56969	NR_002229	**RPL23AP32**	ribosomal protein L23a p	transcribed_pseu	0	0	1	1	1.000
56970	NM_001382308,NM	**ATXN7L3**	ataxin 7 like 3	protein_coding	71	67	72	68	0.950
56971	NM_001127893,NM	**CEACAM19**	CEA cell adhesion molec	protein_coding	0	0	1	1	1.000
56975	NM_020223,XM_01	**FAM20C**	FAM20C golgi associated	protein_coding	0	0	1	1	1.000
56977	NM_020225,NR_13	**STOX2**	storkhead box 2	protein_coding	0	0	1	1	0.980
56978	NM_001099403,NM	**PRDM8**	PR/SET domain 8	protein_coding	47	48	48	49	1.023
56979	NM_001376900,NM	**PRDM9**	PR/SET domain 9	protein_coding	0	0	1	1	1.000
56980	NM_001367890,NM	**PRDM10**	PR/SET domain 10	protein_coding	10	7	11	8	0.766
56981	NM_001256695,NM	**PRDM11**	PR/SET domain 11	protein_coding	2	1	3	2	0.793
56983	NM_152305,NR_02	**POGLUT1**	protein O-glucosyltransfe	protein_coding	24	24	25	25	1.015
56984	NM_020232,NM_14	**PSMG2**	proteasome assembly ch	protein_coding	71	76	72	77	1.057
56985	NM_020233	**ADPRM**	ADP-ribose/CDP-alcohol	protein_coding	2	2	3	3	0.992
56986	NM_001144955,NM	**DTWD1**	DTW domain containing	protein_coding	9	11	10	12	1.151
56987	NM_001142568,NM	**BBX**	BBX high mobility group	protein_coding	20	16	21	17	0.787
56990	NM_001038702,NM	**CDC42SE2**	CDC42 small effector 2	protein_coding	79	70	80	71	0.897
56992	NM_020242,XM_00	**KIF15**	kinesin family member 1	protein_coding	34	28	35	29	0.837
56993	NM_020243	**TOMM22**	translocase of outer mito	protein_coding	168	181	169	182	1.076
56994	NM_020244,XM_01	**CHPT1**	choline phosphotransfera	protein_coding	1	1	2	2	1.002
56995	NM_001007466,NM	**TULP4**	TUB like protein 4	protein_coding	4	3	5	4	0.935
56996	NM_001267812,NM	**SLC12A9**	solute carrier family 12 m	protein_coding	18	18	19	19	0.979
56997	NM_020247,XM_00	**COQ8A**	coenzyme Q8A	protein_coding	21	22	22	23	1.036
56998	NM_001012329,NM	**CTNNBIP1**	catenin beta interacting	protein_coding	5	7	6	8	1.240
56999	NM_001318781,NM	**ADAMTS9**	ADAM metallopeptidase	protein_coding	0	0	1	1	1.000
57000	NR_103560	**GSN-AS1**	GSN antisense RNA 1	lncRNA	0	0	1	1	1.000
57001	NM_020186	**SDHAF3**	succinate dehydrogenas	protein_coding	81	77	82	78	0.945
57002	NM_001282446,NM	**YAE1**	YAE1 maturation factor c	protein_coding	11	12	12	13	1.087
57003	NM_020198,XM_00	**CCDC47**	coiled-coil domain contai	protein_coding	138	122	139	123	0.882
57005	gene-UNGP2	**UNGP2**	uracil-DNA glycosylase p	pseudogene	0	0	1	1	1.000
57007	NM_020311,XM_00	**ACKR3**	atypical chemokine recep	protein_coding	0	0	1	1	0.998
57008	gene-RLFP1	**RLFP1**	RLF pseudogene 1	pseudogene	0	0	1	1	1.000
57010	NM_001300895,NM	**CABP4**	calcium binding protein 4	protein_coding	0	0	1	1	0.986
57016	NM_020299,XM_01	**AKR1B10**	aldo-keto reductase fami	protein_coding	0	0	1	1	1.000
57017	NM_020312	**COQ9**	coenzyme Q9	protein_coding	50	54	51	55	1.085
57018	NM_001308185,NM	**CCNL1**	cyclin L1	protein_coding	49	48	50	49	0.967
57019	NM_001308347,NM	**CIAPIN1**	cytokine induced apopto	protein_coding	67	70	68	71	1.047
57020	NM_001300743,NM	**VPS35L**	VPS35 endosomal protei	protein_coding	10	11	11	12	1.100
57026	NM_020315	**PDXP**	pyridoxal phosphatase	protein_coding	82	87	83	88	1.061
57030	NM_020309	**SLC17A7**	solute carrier family 17 m	protein_coding	0	0	1	1	1.061
57035	NM_001321772,NM	**RSRP1**	arginine and serine rich p	protein_coding	81	85	82	86	1.042
57037	NM_020319,XM_01	**ANKMY2**	ankyrin repeat and MYN	protein_coding	8	6	9	7	0.834
57038	NM_001318785,NM	**RARS2**	arginyl-tRNA synthetase	protein_coding	19	18	20	19	0.958
57045	NM_020648	**TWSG1**	twisted gastrulation BMP	protein_coding	0	0	1	1	1.000
57047	NM_001199978,NM	**PLSCR2**	phospholipid scramblase	protein_coding	0	0	1	1	1.000
57048	NM_001201576,NM	**PLSCR3**	phospholipid scramblase	protein_coding	2	3	3	4	1.484
57050	NM_020368	**UTP3**	UTP3 small subunit proc	protein_coding	91	86	92	87	0.949
57053	NM_001303034,NM	**CHRNA10**	cholinergic receptor nico	protein_coding	0	1	1	2	1.150
57054	NM_020364	**DAZ3**	deleted in azoospermia 3	protein_coding	0	0	1	1	1.044
57055	NM_001005785,NM	**DAZ2**	deleted in azoospermia 2	protein_coding	0	0	1	1	1.017
57057	NM_001077653,NM	**TBX20**	T-box transcription factor	protein_coding	0	0	1	1	1.000
57060	NM_001174100,NM	**PCBP4**	poly(rC) binding protein	protein_coding	0	0	1	1	1.000
57061	NR_002768	**HYMAI**	hydatidiform mole associ	lncRNA	0	0	1	1	1.000
57062	NM_020414	**DDX24**	DEAD-box helicase 24	protein_coding	52	47	53	48	0.905
57082	NM_144508,NM_17	**KNL1**	kinetochore scaffold 1	protein_coding	48	40	49	41	0.839
57084	NM_020346	**SLC17A6**	solute carrier family 17 m	protein_coding	0	0	1	1	1.000

57085	NM_001040194,NM	**AGTRAP**	angiotensin II receptor as	protein_coding	5	5	6	6	0.957
57088	NM_001128304,NM	**PLSCR4**	phospholipid scramblase	protein_coding	0	0	1	1	1.000
57089	NM_001349962,NM	**ENTPD7**	ectonucleoside triphosph	protein_coding	7	6	8	7	0.914
57091	NM_001164114,NM	**CASS4**	Cas scaffold protein fami	protein_coding	0	0	1	1	1.048
57092	NM_001320395,NM	**PCNP**	PEST proteolytic signal c	protein_coding	210	188	211	189	0.894
57093	NM_020358,XM_01	**TRIM49**	tripartite motif containing	protein_coding	0	0	1	1	1.000
57094	NM_020361,XM_01	**CPA6**	carboxypeptidase A6	protein_coding	0	0	1	1	1.000
57095	NM_020362,XM_01	**PITHD1**	PITH domain containing	protein_coding	131	132	132	133	1.008
57096	NM_001377523,NM	**RPGRIP1**	RPGR interacting protein	protein_coding	0	0	1	1	1.000
57097	NM_001286521,NM	**PARP11**	poly(ADP-ribose) polyme	protein_coding	6	6	7	7	0.999
57099	NM_020371,XM_01	**AVEN**	apoptosis and caspase a	protein_coding	37	29	38	30	0.784
57101	NM_001278596,NM	**ANO2**	anoctamin 2	protein_coding	0	0	1	1	1.000
57102	NM_001304811,NM	**C12orf4**	chromosome 12 open rea	protein_coding	14	14	15	15	1.019
57103	NM_020375	**TIGAR**	TP53 induced glycolysis	protein_coding	6	5	7	6	0.866
57104	NM_020376,XM_00	**PNPLA2**	patatin like phospholipas	protein_coding	39	43	40	44	1.103
57105	NM_001308465,NM	**CYSLTR2**	cysteinyl leukotriene rece	protein_coding	2	1	3	2	0.787
57106	NM_020378	**NAT14**	N-acetyltransferase 14 (p	protein_coding	15	18	16	19	1.244
57107	NM_020381,XM_01	**PDSS2**	decaprenyl diphosphate	protein_coding	15	13	16	14	0.884
57108	gene-KMT5AP1	**KMT5AP1**	KMT5A pseudogene 1	pseudogene	1	1	2	2	1.197
57109	NM_001279349,NM	**REXO4**	REX4 homolog, 3'-5' exo	protein_coding	71	71	72	72	1.005
57110	NM_001366112,NM	**PLAAT1**	phospholipase A and acy	protein_coding	0	0	1	1	0.974
57111	NM_020387,NR_13	**RAB25**	RAB25, member RAS on	protein_coding	0	0	1	1	1.091
57113	NM_001167576,NM	**TRPC7**	transient receptor potent	protein_coding	0	0	1	1	1.000
57115	NM_020393,XM_01	**PGLYRP4**	peptidoglycan recognitio	protein_coding	0	0	1	1	1.000
57116	NM_001204221,NM	**ZNF695**	zinc finger protein 695	protein_coding	0	0	1	1	1.000
57117	NM_001142471,NM	**INTS12**	integrator complex subu	protein_coding	25	24	26	25	0.941
57118	NM_001351032,NM	**CAMK1D**	calcium/calmodulin depe	protein_coding	0	0	1	1	1.005
57119	NM_001302861,NM	**EPPIN**	epididymal peptidase inh	protein_coding	0	0	1	1	1.000
57120	NM_001017408,NM	**GOPC**	golgi associated PDZ an	protein_coding	40	37	41	38	0.925
57121	NM_001142961,NM	**LPAR5**	lysophosphatidic acid rec	protein_coding	0	0	1	1	0.979
57122	NM_001330192,NM	**NUP107**	nucleoporin 107	protein_coding	56	49	57	50	0.872
57124	NM_020404	**CD248**	CD248 molecule	protein_coding	0	0	1	1	1.000
57125	NM_020405	**PLXDC1**	plexin domain containing	protein_coding	0	0	1	1	1.121
57126	NM_020406,XM_01	**CD177**	CD177 molecule	protein_coding	0	0	1	1	1.000
57127	NM_001256395,NM	**RHBG**	Rh family B glycoprotein	protein_coding	0	0	1	1	1.000
57128	NM_001164840,NM	**LYRM4**	LYR motif containing 4	protein_coding	60	59	61	60	0.982
57129	NM_020409,NM_17	**MRPL47**	mitochondrial ribosomal	protein_coding	135	127	136	128	0.943
57130	NM_020410	**ATP13A1**	ATPase 13A1	protein_coding	32	34	33	35	1.038
57132	NM_020412	**CHMP1B**	charged multivesicular b	protein_coding	15	14	16	15	0.966
57134	NM_001289010,NM	**MAN1C1**	mannosidase alpha class	protein_coding	0	0	1	1	1.000
57135	NM_001005375,NM	**DAZ4**	deleted in azoospermia 4	protein_coding	0	0	1	1	1.015
57136	NM_020531,XM_00	**APMAP**	adipocyte plasma memb	protein_coding	185	183	186	184	0.988
57139	NM_001035223,NM	**RGL3**	ral guanine nucleotide di	protein_coding	0	0	1	1	1.000
57140	NM_018226,XM_00	**RNPEPL1**	arginyl aminopeptidase l	protein_coding	38	39	39	40	1.031
57142	NM_001321859,NM	**RTN4**	reticulon 4	protein_coding	122	106	123	107	0.867
57143	NM_001142545,NM	**ADCK1**	aarF domain containing k	protein_coding	3	3	4	4	1.075
57144	NM_020341,NM_17	**PAK5**	p21 (RAC1) activated kin	protein_coding	0	0	1	1	1.000
57146	NM_001301769,NM	**TMEM159**	transmembrane protein 1	protein_coding	0	0	1	1	1.000
57147	NM_020423,NM_18	**SCYL3**	SCY1 like pseudokinase	protein_coding	7	6	8	7	0.900
57148	NM_001282917,NM	**RALGAPB**	Ral GTPase activating pr	protein_coding	37	31	38	32	0.839
57149	NM_001128301,NM	**LYRM1**	LYR motif containing 1	protein_coding	40	46	41	47	1.156
57150	NM_001042493,NM	**SMIM8**	small integral membrane	protein_coding	13	12	14	13	0.936
57151	NM_001199951,NM	**LYZL6**	lysozyme like 6	protein_coding	0	0	1	1	1.000
57152	NM_020427	**SLURP1**	secreted LY6/PLAUR dom	protein_coding	0	0	1	1	1.000
57153	NM_001145056,NM	**SLC44A2**	solute carrier family 44 m	protein_coding	24	25	25	26	1.009
57154	NM_001199847,NM	**SMURF1**	SMAD specific E3 ubiqui	protein_coding	16	12	17	13	0.772
57156	NM_020431	**TMEM63C**	transmembrane protein 6	protein_coding	0	0	1	1	1.044
57157	NM_001127357,NM	**PHTF2**	putative homeodomain tr	protein_coding	37	28	38	29	0.772
57158	NM_020433,NM_17	**JPH2**	junctophilin 2	protein_coding	1	0	2	1	0.699
57159	NM_032546,NM_18	**TRIM54**	tripartite motif containing	protein_coding	0	0	1	1	1.000
57161	NM_021255,XM_00	**PELI2**	pellino E3 ubiquitin prote	protein_coding	0	0	1	1	1.000
57162	NM_020651,XM_00	**PELI1**	pellino E3 ubiquitin prote	protein_coding	11	10	12	11	0.928
57165	NM_020435	**GJC2**	gap junction protein gam	protein_coding	0	0	1	1	1.240
57167	NM_001318031,NM	**SALL4**	spalt like transcription fa	protein_coding	0	0	1	1	1.000
57168	NM_020437	**ASPHD2**	aspartate beta-hydroxyla	protein_coding	5	4	6	5	0.853
57169	NM_021035	**ZNFX1**	zinc finger NFX1-type co	protein_coding	43	38	44	39	0.895
57171	NM_001135917,NM	**DOLPP1**	dolichyldiphosphatase 1	protein_coding	31	28	32	29	0.898
57172	NM_020439,XM_01	**CAMK1G**	calcium/calmodulin depe	protein_coding	0	0	1	1	1.000
57175	NM_001018070,NM	**CORO1B**	coronin 1B	protein_coding	8	12	9	13	1.380
57176	NM_001167733,NM	**VARS2**	valyl-tRNA synthetase 2,	protein_coding	19	20	20	21	1.062
57178	NM_020338,XM_00	**ZMIZ1**	zinc finger MIZ-type cont	protein_coding	83	73	84	74	0.880
57179	NM_001079684,NM	**KIAA1191**	KIAA1191	protein_coding	47	41	48	42	0.876
57180	NM_001040135,NM	**ACTR3B**	actin related protein 3B	protein_coding	4	4	5	5	0.915
57181	NM_001127257,NM	**SLC39A10**	solute carrier family 39 m	protein_coding	13	9	14	10	0.725
57182	NM_001167882,NM	**ANKRD50**	ankyrin repeat domain 50	protein_coding	0	0	1	1	1.000
57184	NM_001321920,NM	**FAM219B**	family with sequence sim	protein_coding	14	11	15	12	0.859
57185	NM_001322854,NM	**NIPAL3**	NIPA like domain contain	protein_coding	28	27	29	28	0.962
57186	NM_020343,XM_00	**RALGAPA2**	Ral GTPase activating pr	protein_coding	18	13	19	14	0.744
57187	NM_001081550,XM	**THOC2**	THO complex 2	protein_coding	60	47	61	48	0.793
57188	NM_001301110,NM	**ADAMTSL3**	ADAMTS like 3	protein_coding	0	0	1	1	1.000
57189	NM_001080392,NM	**DENND11**	DENN domain containing	protein_coding	1	1	2	2	1.109
57190	NM_020451,NM_20	**SELENON**	selenoprotein N	protein_coding	0	0	1	1	1.000
57191	NM_020633	**VN1R1**	vomeronasal 1 receptor 1	protein_coding	1	0	2	1	0.708
57192	NM_020533	**MCOLN1**	mucolipin 1	protein_coding	12	15	13	16	1.248
57194	NM_024490,XM_00	**ATP10A**	ATPase phospholipid tra	protein_coding	0	0	1	1	1.016
57198	NM_001005855,NM	**ATP8B2**	ATPase phospholipid tra	protein_coding	37	37	38	38	0.995
57205	NM_020453,XM_00	**ATP10D**	ATPase phospholipid tra	protein_coding	12	9	13	10	0.736
57209	NM_001267597,NM	**ZNF248**	zinc finger protein 248	protein_coding	3	2	4	3	0.791
57210	NM_001080431,NM	**SLC45A4**	solute carrier family 45 m	protein_coding	2	2	3	3	0.950
57211	NM_001032394,NM	**ADGRG6**	adhesion G protein-coup	protein_coding	0	0	1	1	1.014
57212	NR_033708,NR_03	**TP73-AS1**	TP73 antisense RNA 1	lncRNA	0	0	1	1	1.000
57213	NM_001127482,NM	**SPRYD7**	SPRY domain containing	protein_coding	27	25	28	26	0.936
57214	NM_001293298,NM	**CEMIP**	cell migration inducing h	protein_coding	0	0	1	1	0.958
57215	NM_020457	**THAP11**	THAP domain containing	protein_coding	77	78	78	79	1.023
57216	NM_020335,XM_00	**VANGL2**	VANGL planar cell polari	protein_coding	0	0	1	1	1.000
57217	NM_001288951,NM	**TTC7A**	tetratricopeptide repeat c	protein_coding	20	20	21	21	1.007
57221	NM_020340,XR_00	**ARFGEF3**	ARFGEF family member	protein_coding	0	0	1	1	1.000
57222	NM_001031711,XM	**ERGIC1**	endoplasmic reticulum-g	protein_coding	49	44	50	45	0.904
57223	NM_001122964,NM	**PPP4R3B**	protein phosphatase 4 re	protein_coding	66	56	67	57	0.860
57224	NM_001144060,NM	**NHSL1**	NHS like 1	protein_coding	0	0	1	1	1.000
57226	NM_020466,NR_02	**LYRM2**	LYR motif containing 2	protein_coding	23	20	24	21	0.884
57228	NM_001031628,NM	**SMAGP**	small cell adhesion glyco	protein_coding	0	0	1	1	1.000
57231	NM_001297614,NM	**SNX14**	sorting nexin 14	protein_coding	32	31	33	32	0.954
57232	NM_001037735,NM	**ZNF630**	zinc finger protein 630	protein_coding	0	0	1	1	1.000
57234	NR_111950,NR_11	**LINC00869**	long intergenic non-prote	lncRNA	19	18	20	19	0.962
57282	NM_001178015,NM	**SLC4A10**	solute carrier family 4 me	protein_coding	96	94	97	95	0.981
57289	id-IGHV7-4-1	**IGHV7-4-1**	immunoglobulin heavy va	V_segment	0	0	1	1	1.000
57291	NR_024031,NR_14	**DANCR**	differentiation antagonizi	lncRNA	159	165	160	166	1.040
57325	NM_020536,NR_02	**KAT14**	lysine acetyltransferase 1	protein_coding	21	22	22	23	1.007
57326	NM_001317734,NM	**PBXIP1**	PBX homeobox interactir	protein_coding	5	5	6	6	0.984
57332	NM_020649	**CBX8**	chromobox 8	protein_coding	9	9	10	10	1.076
57333	NM_020650,XM_02	**RCN3**	reticulocalbin 3	protein_coding	0	0	1	1	1.000
57335	NM_001130842,NM	**ZNF286A**	zinc finger protein 286A	protein_coding	12	9	13	10	0.826
57336	NM_001346167,NM	**ZNF287**	zinc finger protein 287	protein_coding	1	1	2	2	0.834
57337	NM_001077203,NM	**SENP7**	SUMO specific peptidase	protein_coding	11	8	12	9	0.766
57338	NM_001271604,NM	**JPH3**	junctophilin 3	protein_coding	0	0	1	1	1.000
57343	NM_001290318,NM	**ZNF304**	zinc finger protein 304	protein_coding	6	7	7	8	1.184
57348	NM_001005367,NM	**TTYH1**	tweety family member 1	protein_coding	0	0	1	1	1.000
57369	NM_020660,XM_01	**GJD2**	gap junction protein delt	protein_coding	0	0	1	1	1.000
57379	NM_001330343,NM	**AICDA**	activation induced cytidir	protein_coding	0	0	1	1	1.000
57380	NM_001286264,NM	**MRS2**	magnesium transporter N	protein_coding	19	15	20	16	0.820
57381	NM_020663,XM_00	**RHOJ**	ras homolog family mem	protein_coding	0	0	1	1	1.000
57393	NM_020665,XM_01	**CLTRN**	collectrin, amino acid tra	protein_coding	0	0	1	1	1.000
57396	NM_020666	**CLK4**	CDC like kinase 4	protein_coding	8	9	9	10	1.118
57402	NM_020672,XM_00	**S100A14**	S100 calcium binding pro	protein_coding	0	0	1	1	1.000
57403	NM_020673	**RAB22A**	RAB22A, member RAS o	protein_coding	16	14	17	15	0.913

57404	NM_001371695,NM	**CYP20A1**	cytochrome P450 family	protein_coding	6	5	7	6	0.927
57405	NM_020675,XM_01	**SPC25**	SPC25 component of NE	protein_coding	87	81	88	82	0.927
57406	NM_001320126,NM	**ABHD6**	abhydrolase domain con	protein_coding	7	5	8	6	0.861
57407	NM_001305141,NM	**NMRAL1**	NmrA like redox sensor 1	protein_coding	64	72	65	73	1.134
57408	NM_001304389,NM	**LRTM1**	leucine rich repeats and	protein_coding	0	0	1	1	1.000
57409	NM_001242498,NM	**MIF4GD**	MIF4G domain containin	protein_coding	39	44	40	45	1.146
57410	NM_001048218,NM	**SCYL1**	SCY1 like pseudokinase	protein_coding	67	67	68	68	0.999
57412	NM_020682	**AS3MT**	arsenite methyltransfera	protein_coding	0	0	1	1	1.000
57413	NM_001081976,NM	**TMIGD3**	transmembrane and imm	protein_coding	0	0	1	1	1.382
57414	NM_001040456,NM	**RHBDD2**	rhomboid domain contai	protein_coding	63	72	64	73	1.126
57415	NM_001291941,NM	**C3orf14**	chromosome 3 open rea	protein_coding	0	0	1	1	1.000
57418	NM_001372085,NM	**WDR18**	WD repeat domain 18	protein_coding	125	131	126	132	1.050
57419	NM_020689	**SLC24A3**	solute carrier family 24 m	protein_coding	0	0	1	1	1.083
57446	NM_022477,NM_03	**NDRG3**	NDRG family member 3	protein_coding	39	36	40	37	0.921
57447	NM_001282211,NM	**NDRG2**	NDRG family member 2	protein_coding	0	0	1	1	1.000
[truncated: 3,559,280 more chars]
